# The influenza A virus NS genome segment displays lineage-specific patterns in predicted RNA secondary structure

**DOI:** 10.1186/s13104-016-2083-6

**Published:** 2016-05-20

**Authors:** A. V. Vasin, A. V. Petrova, V. V. Egorov, M. A. Plotnikova, S. A. Klotchenko, M. N. Karpenko, O. I. Kiselev

**Affiliations:** Research Institute of Influenza, 197376 St-Petersburg, Russia; Peter the Great St-Petersburg Polytechnic University, 195251 St-Petersburg, Russia

**Keywords:** Influenza A virus, RNA secondary structure, RNA hairpin, Pathogenicity, NS gene, Evolution

## Abstract

**Background:**

Influenza A virus (IAV) is a segmented negative-sense RNA virus that causes seasonal epidemics and periodic pandemics in humans. Two regions (nucleotide positions 82–148 and 497–564) in the positive-sense RNA of the NS segment fold into a multi-branch loop or hairpin structures.

**Results:**

We studied 25,384 NS segment positive-sense RNA unique sequences of human and non-human IAVs in order to predict secondary RNA structures of the 82–148 and 497–564 regions using RNAfold software, and determined their host- and lineage-specific distributions. Hairpins prevailed in avian and avian-origin human IAVs, including H1N1_pdm1918_ and H5N1. In human and swine IAV hairpins distribution varied between evolutionary lineages.

**Conclusions:**

These results suggest a possible functional role for these RNA secondary structures and the need for experimental evaluation of these structures in the influenza life cycle.

**Electronic supplementary material:**

The online version of this article (doi:10.1186/s13104-016-2083-6) contains supplementary material, which is available to authorized users.

## Background

Influenza A virus (IAV) is an important pathogen responsible for annual seasonal epidemics and periodic pandemics in humans. The IAV genome consists of eight negative-sense RNA segments, encoding up to 18 proteins [1]. Pathogenicity, host adaptation, and transmissibility of IAVs are complex multifactorial processes involving interactions between virus and host and are likely to be under independent selective pressures [2]. Many IAV proteins, particularly HA, PB2, PB1-F2, PA-X, NA and NS1, contribute to viral virulence [3]; however the role of viral RNAs, and especially their secondary structures, cannot be excluded.

The NS segment positive-sense RNA, encoding NS1 and NEP proteins, has been the most extensively studied with regard to secondary structure [4–8]. There are at least two regions with conserved secondary structures. Both are located near the 5′ and 3′ splice sites of the NS gene, at nucleotide positions 82–148 and 497–564 nucleotide positions [7–9]. The 82–148 region is located within the NS1 open reading frame, while the 497–564 region is located within the NS1 and NEP open reading frames. Secondary structures have been recognized to differ between IAV strains. For example, the 497–564 region is in equilibrium between a hairpin and a pseudoknot structure, and the hairpin is stabilized in H5N1 viruses isolated after 2001 as a result of a silent G → C mutation [9]. The experimentally-derived explorations of NS genomic segment secondary structure showed also the presence of stem loop structures in both regions with a high probability (more than 95 % for 82–148) [10], but most part of base pairs in these stems were evaluated as non-canonical. Generally, however, these structural patterns and their roles in influenza pathogenesis are still to be determined. Since evolution of the NS gene is strongly associated with host adaptation [11, 12], it is intriguing to compare the prevalence of the predicted secondary structures among IAVs of a different origin. Using computer methods, we predicted the secondary RNA structures of the 82–148 and 497–564 regions in the positive-sense RNA of segment 8 in human and non-human IAVs and identified host- and subtype-specific patterns (Additional files 1, 2).

## Methods

Nucleotide sequences of the non-human NS gene were downloaded from the NCBI Influenza Virus Resource [13] NCBI and sequences of the human NS gene from Influenza Research Database-IRD [14] in different periods of time. All duplicated sequences were removed and all data was aligned using MAFFT software [15]. The phylogenetic analysis was made with the help of online service “Generate Phylogenetic Tree” on FLUDB.org using RAxML algorithm and bootstrap analyses with number of replicates = 500. The secondary RNA structures of the 82–148 and 497–564 regions were predicted using RNAfold [16]. RNAfold does not include pseudoknot prediction; therefore, only “stem-loops” (hairpin) and “non loops” structures were evaluated and could be easy visually divided into two groups, according to review Svoboda et al. [17]. Two types of RNA secondary structures were observed. A hairpin structure was defined by the following parameters: stem length, >16 base pairs (bp); loop length, >6 nucleotides (nt); number of branches, 0, ∆G < −19 kcal/mol. A multi-branch stem-loop structure was defined by the following parameters: stem length, >14 bp; number of branches, <2; branch length, <5 bp. Otherwise, the structure with very short stem that divides into two or more smaller stem-loops was referred to as “melt” or “non-looped structure”. Statistical data mining was performed using non-parametric statistical methods in Statistika 6.0 software.

## Results and discussion

In our analysis of the NS segment positive-sense RNA secondary structure in 25,384 isolates including 12,192 human, 2794 swine and 10,398 other non-human isolates, we observed host-specific patterns (Table 1). In human and swine IAVs, stem-loop structures were predicted in the 82–148 region of 3/5 (60.1 %) of the isolates, and in the 497–564 region of 1/2 (52 %) of the isolates. In avian and other host IAVs, secondary structure was predicted for the majority of strains in both regions. Equine viruses were distinct, with a hairpin predicted in the 497–564 region for only 8 % of strains. Ignoring host dividing hairpin structures were predicted for 35 % of isolates for the first region and 66 % of isolates for the second.

**Table 1:** Host distribution of the predicted hairpin RNA secondary structures in the NS gene positive-sense RNA of influenza A viruses

Influenza A virus (IAV)	Number of strains	Predicted secondary structure %		
		Nt 82–148		Nt 497–564
		Hairpin (%)	Multi-branch stem-loop (%)	Hairpin (%)
Human IAVs	12,192	18	41	52
Human H1N1 IAVs	1483	88	3	87*
Human H2N2 IAVs	128	90	1	5*
Human H3N2 IAVs	5342	8*	4	1*
Human H1N1_pdm2009_ IAVs	4913	2*	97*	98*
Human and swine IAVs of avian origin (e.g., H5, H7, and H9)	279	91	6	69*
Swine IAV	2794	10	58	50
Swine triple reassortant IAVs	1467	1	69*	14*
“Classical” swine IAVs lineage	349	4	46*	88*
“Eurasian” swine IAVs lineage	382	55*	0*	98*
Avian IAVs	9674	60	35	90
Canine IAVs	234	95	0	85
Equine IAVs	153	91	2	8
Environmental IAVs	337	73	13	66
IAVs from other species	94	70	10	79

*Nt* nucleotide positions

* Differences are statistically significant (p < 0.5)

To study the distribution of hairpin structures within human IAVs, it was necessary to cluster IAV strains according to the evolutionary origin of the NS segment. Swine IAVs were also included to the analysis, since they are closely related to human IAVs. The phylogenetic tree of the NS gene of human and swine IAVs was reconstructed using RAxML [18]. The “Spanish flu” A/H1N1_pdm1918_ virus was used as an outgroup, since it originated from a pool of avian IAVs and is the ancestor of the majority of contemporary human IAVs [19]. The topology of the phylogenetic tree (Additional file 3) agreed with the accepted model of NS gene evolution [2, 20]. The NS gene sequences of human and swine IAVs formed two distinct groups. The first group corresponded to human and swine IAVs originating from the A/H1N1_pdm1918_ virus. It consisted of (a) human H1N1 (clade NS_H1N1_), H2N2 (clade NS_H2N2_), and H3N2 (clade NS_H3N2_) IAVs, and (b) classical (clade NS_sw_clas_) and triple reassortant (clade NS_sw_tri_) swine IAV lineages and human H1N1_pdm09_ (clade NS_H1N1pdm09_) viruses. The second group corresponded to avian-origin human and swine IAVs, including the Eurasian swine lineage (clade NS_sw_eur_). In these clades some exceptions were observed. For example, several strains of human H1N1 virus, isolated in 1976, were located in the NS_sw_clas_ clade, which can be explained by their swine origin. Sporadic cases of this swine H1N1 virus transmission took place in Fort Dix, New Jersey, USA, in 1976 [21].

Prediction of the secondary structure in the 82–148 and 497–564 regions was determined for viruses in each clade. Data, presented in Table 1, showed significant differences in the secondary structures in human IAVs of different subtypes and in swine IAVs of different origins. Hairpins were predicted in the 82–148 and 497–564 regions for the majority of isolates in the NS_H1N1_ clade, while they were not predicted for NS_H3N2_ clade IAVs. In the NS_H2N2_ clade, hairpins were predicted only in the 82–148 region. In the NS_sw_clas_ and NS_H1N1pdm09_ clades, hairpins were predicted only in the 497–564 region for the majority of isolates. However, there were multi-branch stem-loop structures in the 82–148 region in the majority of NS_H1N1pdm09_ and half of the NS_sw_clas_ viruses. For the NS_sw_tri_ clade, multi-branch stem-loop structures were predicted in the 82–148 region for 70 % of strains; however, in the 497–564 region, hairpin structures were predicted in a minority of isolates.

In avian-origin human and swine IAVs, such as H5N1, H7N7 and H7N9, the secondary structure distribution matches that of avian IAVs. For human H5N1 IAVs, hairpins were predicted in the 82–148 region in a majority of isolates, while in the 497–56 region, hairpins were predicted for viruses isolated after 2001, but not those isolated in 1997–1998. In human H7N9 IAVs, hairpins were predicted in the 82–148 but not 497–564 region. In the NS_sw_eur_ clade, hairpins were predicted in the 497–564 region in the majority of IAV isolates, and in half of IAV isolates for the 82–148 region.

**Fig. 1:**
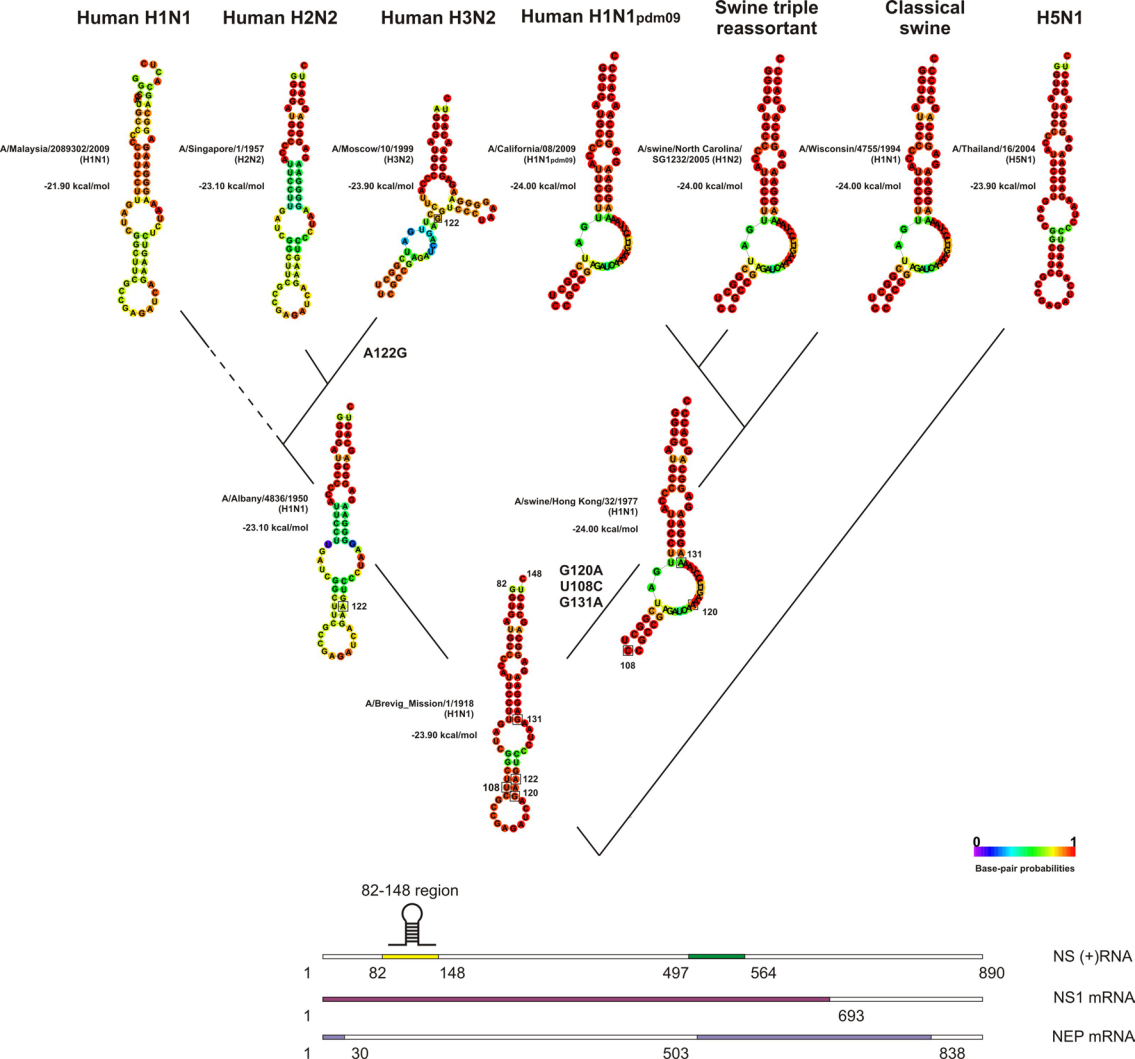
RNA secondary structures in the 82–148 nucleotide region of positive-sense NS segments of human and swine influenza A viruses. The structures that result from the sequences of representative viruses A/Brevig_Mission/1/1918 (H1N1) (ac.no. AF333238), A/Albany/4836/1950 (H1N1) (ac.no. CY021705), A/swine/Hong Kong/32/1977 (H1N1) (ac.no. CY084549), A/Malaysia/2089302/2009 (H1N1) (ac.no. CY074575), A/Singapore/1/1957 (H2N2) (ac.no. CY125898), A/Moscow/10/1999 (H3N2) (ac.no. CY112913), A/California/08/2009 (H1N1pdm09) (ac.no. FJ969533), A/swine/North Carolina/SG1232/2005 (H1N2) (ac.no. CY157835), A/Wisconsin/4755/1994 (H1N1) (ac.no. U53171), A/Thailand/16/2004 (H5N1) (ac.no. CY111602) from evolutionarily distinct clades are shown. Key nucleotide substitutions, those that influence RNA secondary structure, are *boxed*

**Fig. 2:**
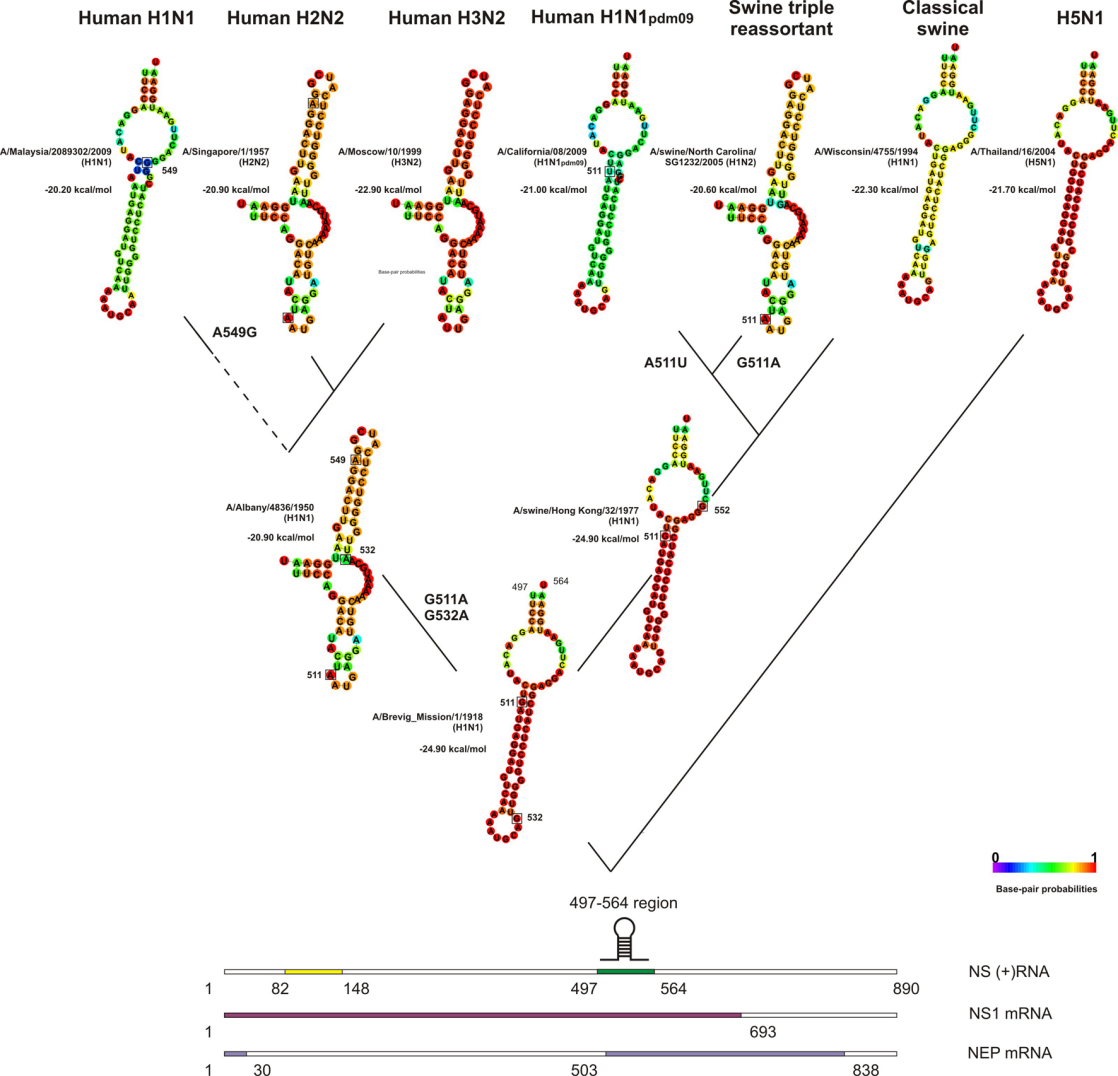
RNA secondary structures in the 497–564 nucleotide region of positive-sense NS segments of human and swine influenza A viruses. The structures that result from the sequences of representative viruses A/Brevig_Mission/1/1918 (H1N1) (ac.no. AF333238), A/Albany/4836/1950 (H1N1) (ac.no. CY021705), A/swine/Hong Kong/32/1977 (H1N1) (ac.no. CY084549), A/Malaysia/2089302/2009 (H1N1) (ac.no. CY074575), A/Singapore/1/1957 (H2N2) (ac.no. CY125898), A/Moscow/10/1999 (H3N2) (ac.no. CY112913), A/California/08/2009 (H1N1_pdm09_) (ac.no. FJ969533), A/swine/North Carolina/SG1232/2005 (H1N2) (ac.no. CY157835), A/Wisconsin/4755/1994 (H1N1) (ac.no. U53171), A/Thailand/16/2004 (H5N1) (ac.no. CY111602) from evolutionarily distinct clades are shown. Key nucleotide substitutions, those that influence RNA secondary structure, are *boxed*

We evaluated the distribution of the observed hairpin secondary structures in different time periods (Figs. 1, 2), and found the key nt substitutions influenced these structures. Pandemic H1N1_1918_ virus was of avian origin and its NS segment positive-sense RNA contained stable hairpins in both the 82–148 and 497–564 regions. By about 1920, seasonally endemic IAVs began to circulate and drift antigenically for nearly 40 years, until 1957. In this period, the hairpin structure prevailed in the 82–148 region; however, sporadic mutations led to the formation of multi-brunch stem-loop structures or elimination of the hairpin. In contrast with the ancestral A/H1N1_1918_ virus, NS_H1N1_ IAVs isolated after 1940 had a single major nt substitution, A132G, which had no effect on the hairpin structure, and two main nonsynonymous substitutions, G511A (N → D in NS1, no effect in NEP) and G532A (V → I in NS1, T → I in NEP), which led to the elimination of the hairpin structure in the 497–564 region.

In 1957, H1N1 IAVs (clade NS_H1N1_) were replaced in circulation by H2N2 viruses (clade NS_H2N2_) as a result of reassortment; however, the NS gene was not changed. There was only one significant mutation (A512U) in the NS_H2N2_ clade IAVs: it was in the same codon as the G511A mutation and led to amino acid substitutions of D to I in NS1 and M to L in NEP. However, it had no influence on the RNA secondary structure of the 497–564 region. In 1968, H3N2 viruses (NS_H3N2_ clade) replaced H2N2 viruses. In 1974, the nonsynonymous mutation A122G (K → R) appeared in isolates from the NS_H3N2_ clade. This mutation was finally fixed in the IAV population in 1977, leading to the loss of the hairpin in the 82–148 region. Interestingly, this was the year that H1N1 viruses began circulation again in the human population. All nucleotide substitutions after 1977 had no influence on the secondary structures of viruses in the NS_H3N2_ clade.

The secondary structure of the 82–148 region of H1N1 viruses remained unchanged since 1977, when the virus returned to circulation in the human population. In 1983, however, two substitutions, C96U and G131A (R → K), appeared, leading to the loss of the hairpin secondary structure. However, within 2 years, the reverse U96C substitution occurred, restoring the hairpin. Another synonymous C126U mutation occurred in 1996, but it did not influence RNA secondary structure. In 1999, a U84C mutation appeared, once again destabilizing the secondary structure; however, it was compensated by A93C so that the hairpin was preserved. At all later times, this hairpin structure has generally been conserved. All of these mutations in H1N1 viruses, with the exception of G131A, were synonymous. In the 497–564 region of H1N1 viruses, circulating after 1977, an A549G mutation (synonymous in NS1, E → G in NEP) appeared in 1983, which led to the re-emergence of the hairpin.

Thus, hairpins from the ancestral H1N1_1918_ were conserved in human H1N1 and H2N2 viruses in the 82–148 region of the NS segment. The hairpin was passed to H3N2 viruses through reassortment of the NS gene, but it was eliminated there. However, hairpins remained in H1N1 viruses re-emerged in 1977. The hairpin was absent in the 497–564 region in human H2N2 and H3N2 viruses. It was also absent in human H1N1 viruses between 1940 and 1982, but it reemerged in 1982.

In order to trace the history of secondary structure and the significance of mutations in the NS_H1N1pdm_ clade of human IAV strains, swine IAVs also needed to be analyzed. In comparison with the H1N1_1918_ ancestral strain, swine isolates from the NS_sw_classic_ clade had three synonymous mutations U108C, G120A, U147C and one nonsynonymous mutation, G137A (R → K), which appeared in 1971. All together, they led to the formation of a multi-branch stem-loop structure in the 82–148 region. However, some substitutions led to stem-loop structure elimination in the NS_sw_classic_ clade after 2000. In the swine NS_sw_tri_ clade IAVs, one major mutation, G143A (S → D). Compared to swine isolates, viruses from the NS_H1N1pdm_ clade had a single synonymous mutations, C127U, which was at the first position of the codon. Both G143A and C127U mutations did not influence a multi-branch stem-loop structure in the 82–148 region.

The NS_sw_clas_ clade viruses had one mutation A552G (synonymous in NS1, D → G in NEP) in the 497–564 region, which did not disturb hairpin structure. NS_sw_tri_ viruses had a G511A substitution, which eliminated the hairpin. Interestingly, in the first years of their isolation (1998–2000), swine triple reassortant IAVs had sequences in the 497–564 region that were identical to the A/H1N1_1918_ ancestral strain sequence. Since 2009, the U513C substitution (synonymous in NS1, S → P in NEP) appeared periodically, but had no influence on secondary structure. At the same time, H1N1_pdm2009_ strains had only one substitution, A511U (N → Y in NS1, no effect for NEP), in comparison with viruses in the NS_sw_tri_ clade, which led to hairpin secondary structure formation.

Compared to the H1N1_1918_ strain, swine isolates in the NS_sw_eur_ clade had three synonymous substitutions C90U, C96U and U99C and one nonsynonymous G139A (G → S). Initially, mutations C96U and U99C dominated, with C90U and G139A mutations appearing periodically. From 2010, all of these mutations were fixed, resulting in the conservation of the hairpin structure in the 82–148 region. Three major substitutions, G532A (V → I, synonymous for NEP) and a pair of G547A/G548A mutations (G → K in NS1; P → T in NEP), were found in the majority of NS_sw_eur_; they had no effect on hairpin structure in the 497–564 region.

Other avian-origin IAVs had two main substitutions: synonymous U102C and G143A (S → D) in the 82–148 region. This last one appeared and was fixed only in 2004. The majority of H5N1 human isolates have a hairpin structure in this region. The same is true of H7N9 isolates, although they have other synonymous substitutions (C96U, U99A, U102C, A129G) and a nonsynonymous substitutions G139A (G → S) that do not appear in the A/H1N1_1918_ strain. These substitutions were also found in H9N2 isolates, but the secondary structure in this region was eliminated as a result of other random substitutions. In human H5N1 viruses, isolated in 1997–1998, there were two substitutions, U513A (N → Q in NS1, L → Q in NEP) and G532A (V → I in NS1, synonymous for NEP), which led to hairpin structure loss in the 497–564 region. From 2001–2003, only one mutation, G532A, appeared, and since 2003, two more substitutions, A512G (V → G in NS1; M → V in NEP) and G537C (synonymous for NS1; G → A in NEP), appeared and were fixed, leading to the re-emergence and maintenance of the hairpin structure. H7N9 viruses have no hairpin secondary structure and have five substitutions in their sequences: A513U (synonymous for NS; S → P in NEP), G514A (E → K in NS1; S → P in NEP), G532A (I → V in NS1, synonymous in NEP), G536A (G → Q in NS1 and G → R in NEP), G538A (V → I in NS1; synonymous for NEP), and C553U (L → F in NS1; synonymous for NEP).

## Conclusions

The role of the observed hairpins is still to be determined. They could be associated with the downregulation of NS1 protein, since a mutation in the hairpin structure was shown to inhibit NS1 protein expression [6]. RNA secondary structure in the 5′ and 3′ splice site region may play an important role in the splicing of IAV segment 8 [22, 23]. Interestingly, segment 7 of IAVs display a similar pattern of predicted secondary structure surrounding the splice sites. Our findings on host-specific patterns of RNA secondary structure are in accordance with species-specific global ordered RNA structure [24] and free energy distributions in the NS segment. The global RNA structure of the NS segment may have evolved for host specificity, since avian, swine, and human viruses replicate at distinct temperatures and pH values that are expected to influence RNA base pairing [25]. RNA secondary structure can also be associated with viral pathogenicity. Hairpin structures in the IAV genome form dsRNA that can activate PKR pathways. Cytokine imbalance is recognized as a main molecular mechanism for complications in IAV infections [26, 27]. These observed differences in the RNA structures at the 5′ and 3′ splice sites in the NS genome segment suggest the need for experiments to test their functional role in the life cycle of IAVs.

## Additional files

10.1186/s13104-016-2083-6 List of sequences and RNA second structure for Nt 82–148.
10.1186/s13104-016-2083-6 List of sequences and RNA second structure for ​Nt 497–564.
10.1186/s13104-016-2083-6 Scheme of human and swine NS gene phylogenetic tree.

## Abbreviations

IAV: influenza A virus
NS1: non-structural protein
NEP: nuclear export protein
