# Supplementary material for: The influenza A virus NS genome segment displays lineage-specific patterns in predicted RNA secondary structure
Source: BMC Res Notes. 2016 May 20;9:279. doi: 10.1186/s13104-016-2083-6 (PMC4875733; doi:10.1186/s13104-016-2083-6)
Supplement: Supplementary file 1 — 10.1186/s13104-016-2083-6 List of sequences and RNA second structure for Nt 82–148. [file 13104_2016_2083_MOESM1_ESM.pdf]

ID	Clade	Host	Subtype	pdm	Year	Country	Strain	Seq 82-148	hairpin loop 2d structure	multi-branch loop 2d structure
AF333238	A/BM/Human1918	Human	H1N1		1918	USA	A/Brevig_Mission/1/1918	ggtgatgccccattccttgatcggcttcgccgagatcag aagtccctaagaggaagaggcagcactc	+	+
CY090849	Human H1N1 IAVs	Human	H1N1		1933	United_Kingdom	A/United_Kingdom/1_MA/1933	ggtgatgccccattccttgatcggcttcgccgagatcag aagtccctaagaggaagaggcagcactc	+	+
CY045760	Human H1N1 IAVs	Human	H1N1		1933	United_Kingdom	A/United_Kingdom/1/1933	ggtgatgccccattccttgatcggcttcgccgagatcag aagtccctaagaggaagaggcagcactc	+	+
CY009608	Human H1N1 IAVs	Human	H1N1		1933	United_Kingdom	A/Wilson_Smith/1933	ggtgatgccccattccttgatcggcttcgccgagatcag aagtccctaagaggaagaggcagcactc	+	+
U13683	Human H1N1 IAVs	Human	H1N1		1933	United_Kingdom	A/WS/1933	ggtgatgccccattccttgatcggcttcgccgagatcag aagtccctaagaggaagaggcagcactc	+	+
DQ508909	Human H1N1 IAVs	Human	H1N1		1933	United_Kingdom	A/Wilson_Smith/1933	ggtgatgccccattccttgatcggcttcgccgagatcag aagtccctaagaggaagaggcagcactc	+	+
M12597	Human H1N1 IAVs	Human	H1N1		1933	United_Kingdom	A/WSN/1933	ggtgatgccccattccttgatcggcttcgccgagatcag aagtccctaagaggaagaggcagcactc	+	+
CY010792	Human H1N1 IAVs	Human	H1N1		1933	United_Kingdom	A/WSN/1933_TS61	ggtgatgccccattccttgatcggcttcgccgagatcag aagtccctaagaggaagaggcagcactc	+	+
CY034136	Human H1N1 IAVs	Human	H1N1		1933	United_Kingdom	A/WSN/1933	ggtgatgccccattccttgatcggcttcgccgagatcag aagtccctaagaggaagaggcagcactc	+	+
Z21498	Human H1N1 IAVs	Human	H1N1		1933	United_Kingdom	A/WS/1933	ggtgattccccattccttgatcggcttcgccgagatcag aagtccctaagaggaagaggcagcactc	+	+
L25720	Human H1N1 IAVs	Human	H1N1		1933	United_Kingdom	A/NWS/1933	ggtgattccccattccttgatcggcttcgccgagatcag aagtccctaagaggaagaggcagcactc	+	+
CY146885	Human H1N1 IAVs	Human	H1N1		1934	Puerto_Rico	A/Puerto_Rico/8_CV10/1934	ggcgatgccccattccttgatcggcttcgccgagatca gaaatccctaagaggaaggggcagtactc	-	*
CY146893	Human H1N1 IAVs	Human	H1N1		1934	Puerto_Rico	A/Puerto_Rico/8_KV20/1934	ggcgatgccccattccttgatcggcttcgccgagatca gaaatccctaagaggaaggggcagtactc	-	*
CY146829	Human H1N1 IAVs	Human	H1N1		1934	Puerto_Rico	A/Puerto_Rico/8_LVD1/1934	ggcgatgccccattccttgatcggcttcgccgagatca gaaatccctaagaggaaggggcagtactc	-	*
CY147466	Human H1N1 IAVs	Human	H1N1		1934	Puerto_Rico	A/Puerto_Rico/8_LVD4/1934	ggcgatgccccattccttgatcggcttcgccgagatca gaaatccctaagaggaaggggcagtactc	-	*
CY147474	Human H1N1 IAVs	Human	H1N1		1934	Puerto_Rico	A/Puerto_Rico/8_SV1/1934	ggcgatgccccattccttgatcggcttcgccgagatca gaaatccctaagaggaaggggcagtactc	-	*
CY147514	Human H1N1 IAVs	Human	H1N1		1934	Puerto_Rico	A/Puerto_Rico/8_SV10/1934	ggcgatgccccattccttgatcggcttcgccgagatca gaaatccctaagaggaaggggcagtactc	-	*
CY147522	Human H1N1 IAVs	Human	H1N1		1934	Puerto_Rico	A/Puerto_Rico/8_SV11/1934	ggcgatgccccattccttgatcggcttcgccgagatca gaaatccctaagaggaaggggcagtactc	-	*
CY146877	Human H1N1 IAVs	Human	H1N1		1934	Puerto_Rico	A/Puerto_Rico/8_SV120/1934	ggcgatgccccattccttgatcggcttcgccgagatca gaaatccctaagaggaaggggcagtactc	-	*
CY147530	Human H1N1 IAVs	Human	H1N1		1934	Puerto_Rico	A/Puerto_Rico/8_SV13/1934	ggcgatgccccattccttgatcggcttcgccgagatca gaaatccctaagaggaaggggcagtactc	-	*
CY147538	Human H1N1 IAVs	Human	H1N1		1934	Puerto_Rico	A/Puerto_Rico/8_SV14/1934	ggcgatgccccattccttgatcggcttcgccgagatca gaaatccctaagaggaaggggcagtactc	-	*
CY040174	Human H1N1 IAVs	Human	H1N1		1934	Puerto_Rico	A/Puerto_Rico/8_SV14/1934	ggcgatgccccattccttgatcggcttcgccgagatca gaaatccctaagaggaaggggcagtactc	-	*
CY146845	Human H1N1 IAVs	Human	H1N1		1934	Puerto_Rico	A/Puerto_Rico/8_SV20/1934	ggcgatgccccattccttgatcggcttcgccgagatca gaaatccctaagaggaaggggcagtactc	-	*

CY146853	Human H1N1 IAVs	Human	H1N1		1934	Puerto_Rico	A/Puerto_Rico/8_SV30/1934	ggcgatgccccattccttgatcggcttcgccgagatca<br>gaaatccctaagaggaaggggcagtactc	-	*
CY146861	Human H1N1 IAVs	Human	H1N1		1934	Puerto_Rico	A/Puerto_Rico/8_SV40/1934	ggcgatgccccattccttgatcggcttcgccgagatca<br>gaaatccctaagaggaaggggcagtactc	-	*
CY146869	Human H1N1 IAVs	Human	H1N1		1934	Puerto_Rico	A/Puerto_Rico/8_SV60/1934	ggcgatgccccattccttgatcggcttcgccgagatca<br>gaaatccctaagaggaaggggcagtactc	-	*
CY147490	Human H1N1 IAVs	Human	H1N1		1934	Puerto_Rico	A/Puerto_Rico/8_SV7/1934	ggcgatgccccattccttgatcggcttcgccgagatca<br>gaaatccctaagaggaaggggcagtactc	-	*
CY147498	Human H1N1 IAVs	Human	H1N1		1934	Puerto_Rico	A/Puerto_Rico/8_SV8/1934	ggcgatgccccattccttgatcggcttcgccgagatca<br>gaaatccctaagaggaaggggcagtactc	-	*
CY147506	Human H1N1 IAVs	Human	H1N1		1934	Puerto_Rico	A/Puerto_Rico/8_SV9/1934	ggcgatgccccattccttgatcggcttcgccgagatca<br>gaaatccctaagaggaaggggcagtactc	-	*
CY084010	Human H1N1 IAVs	Human	H1N1		1934	Puerto_Rico	A/Puerto_Rico/8_WG/1934	ggcgatgccccattccttgatcggcttcgccgagatca<br>gaaatccctaagaggaaggggcagtactc	-	*
JX120148	Human H1N1 IAVs	Human	H1N1		1934	Puerto_Rico	A/Puerto_Rico/8/1934	ggcgatgccccattccttgatcggcttcgccgagatca<br>gaaatccctaagaggaaggggcagtactc	-	*
CY147482	Human H1N1 IAVs	Human	H1N1		1934	Puerto_Rico	A/Puerto_Rico/8_SV5/1934	ggcgatgccccattccttgatcggcttcgccgagatca<br>gaaatccctaagaggaaggggcagtactc	-	*
CY121113	Human H1N1 IAVs	Human	H1N1		1934	Puerto_Rico	A/Puerto_Rico/8/1934	ggtgatgccccattccttgatcggcttcgccgagatcag<br>aaatccctaagaggaaggggcagcaccc	-	*
CY148247	Human H1N1 IAVs	Human	H1N1		1934	Puerto_Rico	A/Puerto_Rico/8/1934	ggtgatgccccattccttgatcggcttcgccgagatcag<br>aaatccctaagaggaaggggcagcaccc	-	*
AF389122	Human H1N1 IAVs	Human	H1N1		1934	Puerto_Rico	A/Puerto_Rico/8/34/Mount_Sinai	ggtgatgccccattccttgatcggcttcgccgagatcag<br>aaatccctaagaggaaggggcagcaccc	-	*
V01104	Human H1N1 IAVs	Human	H1N1		1934	Puerto_Rico	A/Puerto_Rico/8/1934	ggtgatgccccattccttgatcggcttcgccgagatcag<br>aaatccctaagaggaaggggcagcaccc	-	*
CY045768	Human H1N1 IAVs	Human	H1N1		1934	Puerto_Rico	A/Puerto_Rico/8_1/1934	ggtgatgccccattccttgatcggcttcgccgagatcag<br>aaatccctaagaggaaggggcagcactc	-	*
J02150	Human H1N1 IAVs	Human	H1N1		1934	Puerto_Rico	A/Puerto_Rico/8/1934	ggtgatgccccattccttgatcggcttcgccgagatcag<br>aaatccctaagaggaaggggcagcactc	-	*
CY147458	Human H1N1 IAVs	Human	H1N1		1934	Puerto_Rico	A/Puerto_Rico/8_LVD2/1934	ggtgatgccccattccttgatcggcttcgccgagatcag<br>aaatccctaagaggaaggggcagcactc	-	*
CY146837	Human H1N1 IAVs	Human	H1N1		1934	Puerto_Rico	A/Puerto_Rico/8_LVD3/1934	ggtgatgccccattccttgatcggcttcgccgagatcag<br>aaatccctaagaggaaggggcagcactc	-	*
CY033581	Human H1N1 IAVs	Human	H1N1		1934	Puerto_Rico	A/Puerto_Rico/8/34	ggtgatgccccattccttgatcggcttcgccgagatcag<br>aaatccctaagaggaaggggcagtaccc	-	*
KC866599	Human H1N1 IAVs	Human	H1N1		1934	Puerto_Rico	A/Puerto_Rico/8/1934	ggtgatgccccattccttgatcggcttcgccgagatcag<br>aaatccctaagaggaaggggcagtactc	-	*
CY009448	Human H1N1 IAVs	Human	H1N1		1934	Puerto_Rico	A/Puerto_Rico/8/1934	ggtgatgccccattccttgatcggcttcgccgagatcag<br>aaatccctaagaggaaggggcagtactc	-	*
EF467817	Human H1N1 IAVs	Human	H1N1		1934	Puerto_Rico	A/Puerto_Rico/8/34	ggtgatgccccattccttgatcggcttcgccgagatcag<br>aaatccctaagaggaaggggcagtactc	-	*
CY019959	Human H1N1 IAVs	Human	H1N1		1935	USA	A/Alaska/1935	ggtgatgccccattccttgatcggcttcgccgagatcag<br>aaatccctaagaggaaggggcagtactc	-	*
CY009328	Human H1N1 IAVs	Human	H1N1		1935	Australia	A/Melbourne/1935	ggtgatgccccattccttgatcggcttcgccgagatcag<br>aagtccctaagaggaagaggcagcactc	+	+
CY147402	Human H1N1 IAVs	Human	H1N1		1935	Australia	A/Melbourne/JY2/1935	ggtgatgccccattccttgatcggcttcgccgagatcag<br>aagtccctaagaggaagaggcagcactc	+	+
CY096815	Human H1N1 IAVs	Human	H1N1		1935	NA	A/bh/1935	ggtgatgccccattccttgatcggcttcgccgagatcag<br>aagtccctaagaggaagaggcagcactc	+	+

CY147330	Human H1N1 IAVs	Human	H1N1		1935	NA	A/BH/JY2/1935	ggtgatgccccattccttgatcggcttcgccgagatcag aagtccctaagaggaagaggcagcactc	+	+
CY020449	Human H1N1 IAVs	Human	H1N1		1936	NA	A/Henry/1936	ggtgatgccccattccttgatcggcttcgccgagatcag aaatccctaagaggaaggggcagcactc	-	*
CY013275	Human H1N1 IAVs	Human	H1N1		1940	USA	A/Hickox/1940	ggtgatgccccattccttgatcggcttcgccgagatcag aagtccctaaagggaagaggcagcactc	+	+
CY146781	Human H1N1 IAVs	Human	H1N1		1940	NA	A/Hickox/JY2/1940	ggtgatgccccattccttgatcggcttcgccgagatcag aagtccctaaagggaagaggcagcactc	+	+
U13682	Human H1N1 IAVs	Human	H1N1		1940	United_Kingdom	A/WSN/1940	ggtgatgccccattccttgatcggcttcgccgagatcag aagtccctaagaggaagaggcagcactc	+	+
CY009280	Human H1N1 IAVs	Human	H1N1		1942	USA	A/Bel/1942	ggtgatgccccattccttgatcggcttcgccgagatcag aagtccctaagaggaagaggcagcactc	+	+
CY146773	Human H1N1 IAVs	Human	H1N1		1942	NA	A/Bellamy/JY2/1942	ggtgatgccccattccttgatcggcttcgccgagatcag aagtccctaagaggaagaggcagcactc	+	+
CY020289	Human H1N1 IAVs	Human	H1N1		1943	USA	A/AA/Marton/1943	ggtgatgccccattccttgatcggcttcgccgagatcag aagtccctaagaggaagaggcagcactc	+	+
CY021713	Human H1N1 IAVs	Human	H1N1		1945	USA	A/AA/Huston/1945	ggtgatgccccattccttgatcggcttcgccgagatcag aagtccctaagaggaagaggcagcactc	+	+
CY009600	Human H1N1 IAVs	Human	H1N1		1946	Unknown	A/Cam/46	ggtgatgccccattccttgatcggcttcgcagagatca gaagtccctaaagggaagaggcagcactc	+	+
CY147354	Human H1N1 IAVs	Human	H1N1		1946	NA	A/Cameron/JY2/1946	ggtgatgccccattccttgatcggcttcgccgagatcag aagtccctaaagggaagaggcagcactc	+	+
CY045776	Human H1N1 IAVs	Human	H1N1		1946	Australia	A/Melbourne/1/1946	ggtgatgccccattccttgatcggcttcgccgagatcag aagtccctaagaggaagaggcagcactc	+	+
CY147346	Human H1N1 IAVs	Human	H1N1		1947	USA	A/Fort_Monmouth/1_JY2/1947	ggtgatgccccattccttgatcggcttcgccgagatcag aaatccctaaagggaagaggcagcactc	-	*
CY087788	Human H1N1 IAVs	Human	H1N1		1947	USA	A/Fort_Monmouth/1_MA/1947	ggtgatgccccattccttgatcggcttcgccgagatcag aaatccctaaagggaagaggcagcactc	-	*
U02087	Human H1N1 IAVs	Human	H1N1		1947	USA	A/Fort_Monmouth/1/1947	ggtgatgccccattccttgatcggcttcgccgagatcag aaatccctaaagggaagaggcagcactc	-	*
CY045784	Human H1N1 IAVs	Human	H1N1		1947	USA	A/Fort_Monmouth/1/1947	ggtgatgccccattccttgatcggcttcgccgagatcag aaatccctaaagggaagaggcagcactc	-	*
K00577	Human H1N1 IAVs	Human	H1N1		1947	USA	A/Fort_Monmouth/1/1947	ggtgatgccccattccttgatcggcttcgccgagatcag aaatccctaaagggaagaggcagcactc	-	*
CY009616	Human H1N1 IAVs	Human	H1N1		1947	USA	A/Fort_Monmouth/1/1947	ggtgatgccccattccttgatcggcttcgccgagatcag aaatccctaaagggaagaggcagcactc	-	*
CY077760	Human H1N1 IAVs	Human	H1N1		1948	Netherlands	A/Netherlands/001S1/1948	ggtgatgccccattccttgatcggcttcgccgagatcag aagtccctaagaggaagaggcagcactc	+	+
CY077885	Human H1N1 IAVs	Human	H1N1		1948	USA	A/Hemsbury/1948	ggtgatgccccattccttgatcggcttcgccgagatcag aagtccctaagagggagaggcagcactc	+	+
CY019951	Human H1N1 IAVs	Human	H1N1		1948	USA	A/Albany/4835/1948	ggtgatgccccattccttgatcggcttcgccgagatcag aagtccctaaggggaagaggcagcactc	+	+
CY077767	Human H1N1 IAVs	Human	H1N1		1949	Netherlands	A/Netherlands/002K1/1949	ggtgatgccccattccttgatcggcttcgccgagatcag aaatccctaaagggaagaggcagcactc	-	*
CY019975	Human H1N1 IAVs	Human	H1N1		1949	Italy	A/Roma/1949	ggtgatgccccattccttgatcggcttcgccgagatcag aagtccctaaggggaagaggcagcactc	+	+
CY147362	Human H1N1 IAVs	Human	H1N1		1949	NA	A/Roma/JY2/1949	ggtgatgccccattccttgatcggcttcgccgagatcag aagtccctaaggggaagaggcagcactc	+	+
CY077721	Human H1N1 IAVs	Human	H1N1		1950	Netherlands	A/Netherlands/001G1/1950	ggtgatgccccattccttgatcggcttcgccgagatcag aaatccctaaagggaagaggcagcactc	-	*

CY021705	Human H1N1 IAVs	Human	H1N1		1950	USA	A/Albany/4836/1950	ggtgatgccccattccttgatcggcttcgccgagatcag<br>aagtccctaaggggaagaggcagcactc	+	+
K00576	Human H1N1 IAVs	Human	H1N1		1950	USA	A/Fort_Warren/1/1950	ggtgatgccccattccttgatcggcttcgccgagatcag<br>aagtccctaaggggaagaggcagcactc	+	+
CY009336	Human H1N1 IAVs	Human	H1N1		1950	USA	A/Fort_Warren/1/1950	ggtgatgccccattccttgatcggcttcgccgagatcag<br>aagtccctaaggggaagaggcagcactc	+	+
CY147338	Human H1N1 IAVs	Human	H1N1		1950	USA	A/Fort_Warren/50_JY2/1950	ggtgatgccccattccttgatcggcttcgccgagatcag<br>aagtccctaaggggaagaggcagcactc	+	+
CY021825	Human H1N1 IAVs	Human	H1N1		1951	USA	A/Albany/12/1951	ggtgatgccccattccttgatcggcttcgccgagatcag<br>aagtccctaaggggaagaggcagcactc	+	+
CY077893	Human H1N1 IAVs	Human	H1N1		1951	United_Kingdom	A/Liverpool/1951	ggtgatgccccattccttgatcggcttcgccgagatcag<br>aagtccctaaggggaagaggcagcactc	+	+
CY021905	Human H1N1 IAVs	Human	H1N1		1951	USA	A/Albany/1618/1951	ggtgatgccccattccttgatcggcttcgccgagatcag<br>aagtccctaaggggaagaggcagcactc	+	+
CY022025	Human H1N1 IAVs	Human	H1N1		1951	USA	A/Albany/14/1951	ggtgatgccccattccttgatcggcttcgccgagatcag<br>aagtccctaaggggaagaggcagcactc	+	+
CY022097	Human H1N1 IAVs	Human	H1N1		1951	USA	A/Albany/13/1951	ggtgatgccccattccttgatcggcttcgccgagatcag<br>aagtccctaaggggaagaggcagcactc	+	+
CY147378	Human H1N1 IAVs	Human	H1N1		1951	NA	A/FLW/1951	ggtgatgccccattccttgatcggcttcgccgagatcag<br>aagtccctaaggggaagaggcagcactc	+	+
CY077775	Human H1N1 IAVs	Human	H1N1		1953	Netherlands	A/Netherlands/002W1/1953	ggtgatgccccattccttgatcggcttcgccgagatcag<br>aagtccctaaggggaagaggcagcactc	+	+
CY077752	Human H1N1 IAVs	Human	H1N1		1953	Netherlands	A/Netherlands/001R1/1953	ggtgatgccccattccttgatcggcttcgccgagatcag<br>aagtccctaaggggaagaggcagcactc	+	+
X52146	Human H1N1 IAVs	Human	H1N1		1954	Russia	A/Leningrad/1954/1	ggtgatgccccattccttgatcggcttcgccgagatcag<br>aaatccctaagaggaaggggcagtactc	-	*
CY077729	Human H1N1 IAVs	Human	H1N1		1954	Netherlands	A/Netherlands/001H1/1954	ggtgatgccccattccttgatcggcttcgccgagatcag<br>aagtccctaaggggaagaggcagcactc	+	+
CY021057	Human H1N1 IAVs	Human	H1N1		1954	Malaysia	A/Malaya/302/1954	ggtgatgccccattccttgatcggcttcgccgagatcag<br>aagtccctaaggggaagaggcagcactc	+	+
CY009344	Human H1N1 IAVs	Human	H1N1		1954	Malaysia	A/Malaysia/54	ggtgatgccccattccttgatcggcttcgccgagatcag<br>aagtccctaaggggaagaggcagcactc	+	+
CY146789	Human H1N1 IAVs	Human	H1N1		1954	Malaysia	A/Malaysia/JY2/1954	ggtgatgccccattccttgatcggcttcgccgagatcag<br>aagtccctaaggggaagaggcagcactc	+	+
CY077715	Human H1N1 IAVs	Human	H1N1		1956	Netherlands	A/Netherlands/001B1/1956	ggtgatgccccattccttgatcggcttcgccgagatcag<br>aagtccctaaggggaagaggcagcactc	+	+
CY077782	Human H1N1 IAVs	Human	H1N1		1956	Netherlands	A/Netherlands/002Z1/1956	ggtgatgccccattccttgatcggcttcgccgagatcag<br>aagtccctaaggggaagaggcagcactc	+	+
CY125866	Human H1N1 IAVs	Human	H1N1		1957	China	A/Kw/1/1957	ggtgatgccccattccttgatcggcttcgccgagatcag<br>aagtccctaaggggaagaggcagcactc	+	+
CY008992	Human H1N1 IAVs	Human	H1N1		1957	USA	A/Denver/1/1957	ggtgatgccccattccttgatcggcttcgccgagatcag<br>aagtccctaaggggaagaggcagcactc	+	+
CY146797	Human H1N1 IAVs	Human	H1N1		1957	USA	A/Denver/JY2/1957	ggtgatgccccattccttgatcggcttcgccgagatcag<br>aagtccctaaggggaagaggcagcactc	+	+
CY014980	Human H1N1 IAVs	Human	H2N2		1957	Japan	A/Japan/305/1957	ggtgatgccccattccttgatcggcttcgccgagatcag<br>aagtccctaaggggaagaggcagtactc	+	+
CY147442	Human H1N1 IAVs	Human	H3N2		1968	Hong_Kong	A/Hong_Kong/JY2/1968	ggtgatgccccattccttgatcggcttcgccgagatcag<br>aaatccctaagaggaaggggcagtactc	-	*
CY021961	Human H1N1 IAVs	Human	H1N1		1976	USA	A/New_Jersey/1976	ggtgatgccccattccttgatcggcttcgccgagatcag<br>aaatccctaagaggaaggggcagtactc	-	*

CY147426	Human H1N1 IAVs	Human	H1N1		1976	USA	A/New_Jersey/Wistar/1976	ggtgatgccccattccttgatcggcttcgccgagatcag<br>aaatccctaagaggaaggggcagtactc	-	*
CY121882	Human H1N1 IAVs	Human	H1N1		1977	Russia	A/USSR/90/1977	ggtgatgccccattccttgatcggcttcgccgagatcag<br>aagtccctaaggggaagaggcagcactc	+	+
K00578	Human H1N1 IAVs	Human	H1N1		1977	Russia	A/USSR/90/1977	ggtgatgccccattccttgatcggcttcgccgagatcag<br>aagtccctaaggggaagaggcagcactc	+	+
DQ508901	Human H1N1 IAVs	Human	H1N1		1977	Russia	A/USSR/90/1977	ggtgatgccccattccttgatcggcttcgccgagatcag<br>aagtccctaaggggaagaggcagcactc	+	+
CY010376	Human H1N1 IAVs	Human	H1N1		1977	Russia	A/USSR/90/1977	ggtgatgccccattccttgatcggcttcgccgagatcag<br>aagtccctaaggggaagaggcagcactc	+	+
CY009296	Human H1N1 IAVs	Human	H1N1		1977	Hong_Kong	A/Hong_Kong/117/1977	ggtgatgccccattccttgatcggcttcgccgagatcag<br>aagtccctaaggggaagaggcagcactc	+	+
CY009288	Human H1N1 IAVs	Human	H1N1		1977	Russia	A/USSR/92/1977	ggtgatgccccattccttgatcggcttcgccgagatcag<br>aagtccctaaggggaagaggcagcactc	+	+
CY021801	Human H1N1 IAVs	Human	H1N1		1978	USA	A/Albany/20/1978	ggtgatgccccattccttgatcggcttcgccgagatcag<br>aagtccctaaggggaagaggcagcactc	+	+
CY020297	Human H1N1 IAVs	Human	H1N1		1978	Brazil	A/Brazil/11/1978	ggtgatgccccattccttgatcggcttcgccgagatcag<br>aagtccctaaggggaagaggcagcactc	+	+
CY010880	Human H1N1 IAVs	Human	H1N1		1978	USA	A/Memphis/11/1978	ggtgatgccccattccttgatcggcttcgccgagatcag<br>aagtccctaaggggaagaggcagcactc	+	+
CY010888	Human H1N1 IAVs	Human	H1N1		1978	USA	A/Memphis/13/1978	ggtgatgccccattccttgatcggcttcgccgagatcag<br>aagtccctaaggggaagaggcagcactc	+	+
CY019967	Human H1N1 IAVs	Human	H1N1		1978	USA	A/Arizona/14/1978	ggtgatgccccattccttgatcggcttcgccgagatcag<br>aagtccctaaggggaagaggcagcactc	+	+
CY020169	Human H1N1 IAVs	Human	H1N1		1978	USA	A/Lackland/3/1978	ggtgatgccccattccttgatcggcttcgccgagatcag<br>aagtccctaaggggaagaggcagcactc	+	+
CY020177	Human H1N1 IAVs	Human	H1N1		1978	USA	A/Lackland/7/1978	ggtgatgccccattccttgatcggcttcgccgagatcag<br>aagtccctaaggggaagaggcagcactc	+	+
CY011300	Human H1N1 IAVs	Human	H1N1		1978	USA	A/Memphis/1/1978	ggtgatgccccattccttgatcggcttcgccgagatcag<br>aagtccctaaggggaagaggcagcactc	+	+
CY010904	Human H1N1 IAVs	Human	H1N1		1978	USA	A/Memphis/17/1978	ggtgatgccccattccttgatcggcttcgccgagatcag<br>aagtccctaaggggaagaggcagcactc	+	+
CY017367	Human H1N1 IAVs	Human	H1N1		1978	USA	A/Memphis/20/1978	ggtgatgccccattccttgatcggcttcgccgagatcag<br>aagtccctaaggggaagaggcagcactc	+	+
CY010896	Human H1N1 IAVs	Human	H1N1		1978	USA	A/Memphis/15/1978	ggtgatgccccattccttgatcggcttcgccgagatcag<br>aagtccctaaggggaagaggcagcactc	+	+
CY010872	Human H1N1 IAVs	Human	H1N1		1978	USA	A/Memphis/10/1978	ggtgatgccccattccttgatcggcttcgccgagatcag<br>aagtccctaaggggaagaggcagcactc	+	+
CY021721	Human H1N1 IAVs	Human	H1N1		1978	USA	A/California/10/1978	ggtgatgccccattccttgatcggcttcgccgagatcag<br>aagtccctaaggggaagaggcagcactc	+	+
CY026415	Human H1N1 IAVs	Human	H1N1		1979	USA	A/Albany/8/1979	ggtgatgccccattccttgatcggcttcgccgagatcag<br>aagtccctaaggggaagaggcagcactc	+	+
CY019743	Human H1N1 IAVs	Human	H1N1		1979	USA	A/Memphis/1/1979	ggtgatgccccattccttgatcggcttcgccgagatcag<br>aagtccctaaggggaagaggcagcactc	+	+
CY021913	Human H1N1 IAVs	Human	H1N1		1979	Russia	A/USSR/46/1979	ggtgatgccccattccttgatcggcttcgccgagatcag<br>aagtccctaaggggaagaggcagcactc	+	+
CY010912	Human H1N1 IAVs	Human	H1N1		1980	USA	A/Memphis/7/1980	ggtgatgccccattccttgatcggcttcgccgagatcag<br>aagtccctaaaaggaagaggcagcactc	+	+
CY020185	Human H1N1 IAVs	Human	H1N1		1980	USA	A/Maryland/2/1980	ggtgatgccccattccttgatcggcttcgccgagatcag<br>aagtccctaaggggaagaggcagcactc	+	+

CY021033	Human H1N1 IAVs	Human	H1N1		1981	USA	A/Baylor/4052/1981	ggtgatgccccattccttgatcggcttcgccgagatcag<br>aagtccctaaggggaagaggcagcactc	+	+
CY010368	Human H1N1 IAVs	Human	H1N1		1982	USA	A/Baylor/11515/1982	ggcgatgccccattccttgatcggcttcgccgagatca<br>gaagtccctaaggggaagaggcagcactc	-	*
CY009624	Human H1N1 IAVs	Human	H1N1		1982	USA	A/Baylor/11735/1982	ggcgatgccccattccttgatcggcttcgccgagatca<br>gaagtccctaaggggaagaggcagcactc	-	*
CY021041	Human H1N1 IAVs	Human	H1N1		1982	United_Kingdom	A/Christ's_Hospital/157/1982	ggtgatgccccatttcttgatcggcttcgccgagatcag<br>aagtccctaaagggaagaggcagcactc	-	-
CY015528	Human H1N1 IAVs	Human	H1N1		1983	USA	A/Memphis/6/1983	ggcgatgccccattccttgatcggcttcgccgagatca<br>gaagtccctaaggggaagaggcagcactc	-	*
CY019759	Human H1N1 IAVs	Human	H1N1		1983	USA	A/Memphis/49/1983	ggtgatgccccattccttgatcggcttcgccgagatcag<br>aagtccctaaggggaagaggcagcactc	+	+
CY012884	Human H1N1 IAVs	Human	H1N1		1983	USA	A/Memphis/1/1983	ggtgatgccccattccttgatcggcttcgccgagatcag<br>aagtccctaaggggaagaggcagcactc	+	+
CY012892	Human H1N1 IAVs	Human	H1N1		1983	USA	A/Memphis/2/1983	ggtgatgccccattccttgatcggcttcgccgagatcag<br>aagtccctaaggggaagaggcagcactc	+	+
CY010928	Human H1N1 IAVs	Human	H1N1		1983	USA	A/Memphis/4/1983	ggtgatgccccatttcttgatcggcttcgccgagatcag<br>aagtccctaaagagaagaggcagcactc	-	*
CY121265	Human H1N1 IAVs	Human	H1N1		1983	Chile	A/Chile/1/1983	ggtgatgccccatttcttgatcggcttcgccgagatcag<br>aagtccctaaagggaagaggcagcactc	-	-
CY020441	Human H1N1 IAVs	Human	H1N1		1983	Chile	A/Chile/1/1983	ggtgatgccccatttcttgatcggcttcgccgagatcag<br>aagtccctaaagggaagaggcagcactc	-	-
X15282	Human H1N1 IAVs	Human	H1N1		1983	Chile	A/Chile/1/1983	ggtgatgccccatttcttgatcggcttcgccgagatcag<br>aagtccctaaagggaagaggcagcactc	-	-
CY013883	Human H1N1 IAVs	Human	H1N1		1983	USA	A/Memphis/10/1983	ggtgatgccccatttcttgatcggcttcgccgagatcag<br>aagtccctaaagggaagaggcagcactc	-	-
CY011308	Human H1N1 IAVs	Human	H1N1		1983	USA	A/Memphis/11/1983	ggtgatgccccatttcttgatcggcttcgccgagatcag<br>aagtccctaaagggaagaggcagcactc	-	-
CY010952	Human H1N1 IAVs	Human	H1N1		1983	USA	A/Memphis/12/1983	ggtgatgccccatttcttgatcggcttcgccgagatcag<br>aagtccctaaagggaagaggcagcactc	-	-
CY011316	Human H1N1 IAVs	Human	H1N1		1983	USA	A/Memphis/13/1983	ggtgatgccccatttcttgatcggcttcgccgagatcag<br>aagtccctaaagggaagaggcagcactc	-	-
CY010984	Human H1N1 IAVs	Human	H1N1		1983	USA	A/Memphis/18/1983	ggtgatgccccatttcttgatcggcttcgccgagatcag<br>aagtccctaaagggaagaggcagcactc	-	-
CY012444	Human H1N1 IAVs	Human	H1N1		1983	USA	A/Memphis/19/1983	ggtgatgccccatttcttgatcggcttcgccgagatcag<br>aagtccctaaagggaagaggcagcactc	-	-
CY017199	Human H1N1 IAVs	Human	H1N1		1983	USA	A/Memphis/20/1983	ggtgatgccccatttcttgatcggcttcgccgagatcag<br>aagtccctaaagggaagaggcagcactc	-	-
CY017207	Human H1N1 IAVs	Human	H1N1		1983	USA	A/Memphis/23/1983	ggtgatgccccatttcttgatcggcttcgccgagatcag<br>aagtccctaaagggaagaggcagcactc	-	-
CY017215	Human H1N1 IAVs	Human	H1N1		1983	USA	A/Memphis/24/1983	ggtgatgccccatttcttgatcggcttcgccgagatcag<br>aagtccctaaagggaagaggcagcactc	-	-
CY017873	Human H1N1 IAVs	Human	H1N1		1983	USA	A/Memphis/25/1983	ggtgatgccccatttcttgatcggcttcgccgagatcag<br>aagtccctaaagggaagaggcagcactc	-	-
CY017881	Human H1N1 IAVs	Human	H1N1		1983	USA	A/Memphis/35/1983	ggtgatgccccatttcttgatcggcttcgccgagatcag<br>aagtccctaaagggaagaggcagcactc	-	-
CY019097	Human H1N1 IAVs	Human	H1N1		1983	USA	A/Memphis/51/1983	ggtgatgccccatttcttgatcggcttcgccgagatcag<br>aagtccctaaagggaagaggcagcactc	-	-
CY010936	Human H1N1 IAVs	Human	H1N1		1983	USA	A/Memphis/7/1983	ggtgatgccccatttcttgatcggcttcgccgagatcag<br>aagtccctaaagggaagaggcagcactc	-	-

CY010944	Human H1N1 IAVs	Human	H1N1		1983	USA	A/Memphis/8/1983	ggtgatgccccatttcttgatcggcttcgccgagatcag<br>aagtccctaaagggaagaggcagcactc	-	-
CY019241	Human H1N1 IAVs	Human	H1N1		1983	USA	A/Memphis/53/1983	ggtgatgccccatttcttgatcggcttcgccgagatcag<br>aagtccctaaagggaagaggcagcactc	-	-
CY017423	Human H1N1 IAVs	Human	H1N1		1983	USA	A/Memphis/21/1983	ggtgatgccccatttcttgatcggcttcgccgagatcag<br>aagtccctaaagggaagaggcagcactc	-	-
CY019049	Human H1N1 IAVs	Human	H1N1		1983	USA	A/Memphis/40/1983	ggtgatgccccatttcttgatcggcttcgccgagatcag<br>aagtccctaaagggaagaggcagcactc	-	-
CY019233	Human H1N1 IAVs	Human	H1N1		1983	USA	A/Memphis/47/1983	ggtgatgccccatttcttgatcggcttcgccgagatcag<br>aagtccctaaagggaagaggcagcactc	-	-
CY019089	Human H1N1 IAVs	Human	H1N1		1983	USA	A/Memphis/50/1983	ggtgatgccccatttcttgatcggcttcgccgagatcag<br>aagtccctaaagggaagaggcagcactc	-	-
CY013299	Human H1N1 IAVs	Human	H1N1		1983	USA	A/Memphis/14/1983	ggtgatgccccatttcttgatcggcttcgccgagatcag<br>aagtccctaaagggaagaggcagcactc	-	-
CY010968	Human H1N1 IAVs	Human	H1N1		1983	USA	A/Memphis/16/1983	ggtgatgccccatttcttgatcggcttcgccgagatcag<br>aagtccctaaagggaagaggcagcactc	-	-
CY010976	Human H1N1 IAVs	Human	H1N1		1983	USA	A/Memphis/17/1983	ggtgatgccccatttcttgatcggcttcgccgagatcag<br>aagtccctaaagggaagaggcagcactc	-	-
CY017223	Human H1N1 IAVs	Human	H1N1		1983	USA	A/Memphis/27/1983	ggtgatgccccatttcttgatcggcttcgccgagatcag<br>aagtccctaaagggaagaggcagcactc	-	-
CY017231	Human H1N1 IAVs	Human	H1N1		1983	USA	A/Memphis/28/1983	ggtgatgccccatttcttgatcggcttcgccgagatcag<br>aagtccctaaagggaagaggcagcactc	-	-
CY017239	Human H1N1 IAVs	Human	H1N1		1983	USA	A/Memphis/29/1983	ggtgatgccccatttcttgatcggcttcgccgagatcag<br>aagtccctaaagggaagaggcagcactc	-	-
CY010920	Human H1N1 IAVs	Human	H1N1		1983	USA	A/Memphis/3/1983	ggtgatgccccatttcttgatcggcttcgccgagatcag<br>aagtccctaaagggaagaggcagcactc	-	-
CY020241	Human H1N1 IAVs	Human	H1N1		1983	USA	A/Memphis/39/1983	ggtgatgccccatttcttgatcggcttcgccgagatcag<br>aagtccctaaagggaagaggcagcactc	-	-
CY017431	Human H1N1 IAVs	Human	H1N1		1983	USA	A/Memphis/22/1983	ggtgatgccccatttcttgatcggcttcgccgagatcag<br>aagtccctaaagggaagaggcagcactc	-	-
CY017439	Human H1N1 IAVs	Human	H1N1		1983	USA	A/Memphis/26/1983	ggtgatgccccatttcttgatcggcttcgccgagatcag<br>aagtccctaaagggaagaggcagcactc	-	-
CY019057	Human H1N1 IAVs	Human	H1N1		1983	USA	A/Memphis/41/1983	ggtgatgccccatttcttgatcggcttcgccgagatcag<br>aagtccctaaagggaagaggcagcactc	-	-
CY019081	Human H1N1 IAVs	Human	H1N1		1983	USA	A/Memphis/48/1983	ggtgatgccccatttcttgatcggcttcgccgagatcag<br>aagtccctaaagggaagaggcagcactc	-	-
CY019041	Human H1N1 IAVs	Human	H1N1		1983	USA	A/Memphis/38/1983	ggtgatgccccatttcttgatcggcttcgccgagatcag<br>aagtccctaaagggaagaggcagcactc	-	-
CY010960	Human H1N1 IAVs	Human	H1N1		1983	USA	A/Memphis/15/1983	ggtgatgccccatttcttgatcggcttcgccgagatcag<br>aagtccctaaagggaagaggcagcactc	-	-
CY019225	Human H1N1 IAVs	Human	H1N1		1983	USA	A/Memphis/31/1983	ggtgatgccccatttcttgatcggcttcgccgagatcag<br>aagtccctaaagggaagaggcagcactc	-	-
CY017247	Human H1N1 IAVs	Human	H1N1		1983	USA	A/Memphis/30/1983	ggtgatgccccatttcttgatcggcttcgccgagatcag<br>aagtccctaaagggaagaggcagcactc	-	-
CY019065	Human H1N1 IAVs	Human	H1N1		1983	USA	A/Memphis/42/1983	ggtgatgccccatttcttgatcggcttcgccgagatcag<br>aagtccctaaagggaagaggcagcactc	-	-
CY017255	Human H1N1 IAVs	Human	H1N1		1983	USA	A/Memphis/32/1983	ggtgatgccccatttcttgatcggcttcgccgagatcag<br>aagtccctaaagggaagaggcagcactc	-	-
CY020193	Human H1N1 IAVs	Human	H1N1		1983	New_Zealand	A/New_Zealand/7/1983	ggtgatgccccatttcttgatcggcttcgccgagatcag<br>aagtccctaaagggaagaggcagcactc	-	-

AJ298950	Human H1N1 IAVs	Human	H1N1		1983	Fiji	A/Fiji/15899/83	ggtgatgccccatttcttgatcggcttcgccgagatcag aagtccctaaagggaagaggcagcactc	-	-
CY027535	Human H1N1 IAVs	Human	H1N1		1983	USA	A/Memphis/44/1983	tgtgatgccccatttcttgatcggcttcgccgagatcag aagtccctaaagggaagaggcagcactc	-	-
CY019767	Human H1N1 IAVs	Human	H1N1		1983	USA	A/Memphis/54/1983	tgtgatgccccatttcttgatcggcttcgccgagatcag aagtccctaaagggaagaggcagcactc	-	-
CY021729	Human H1N1 IAVs	Human	H1N1		1984	USA	A/Memphis/1/1984	ggtgatgccccatttcttgatcggcttcgccgagatcag aagtccctaaagggaagaggcagcactc	-	-
CY187388	Human H1N1 IAVs	Human	H3N2		1984	France	A/Caen/1/1984	ggtgatgccccatttcttgatcggcttcgccgagatcag aagtccctaaagggaagaggcagcactc	-	-
CY019105	Human H1N1 IAVs	Human	H1N1		1986	USA	A/Memphis/12/1986	ggtgatgccccattccttgatcggcttcgccgagatcag aagtccctaaagggaagaggcagcactc	+	+
CY021737	Human H1N1 IAVs	Human	H1N1		1986	USA	A/New_York/2924_1/1986	ggtgatgccccattccttgatcggcttcgccgagatcag aagtccctaaagggaagaggcagcactc	+	+
DQ508877	Human H1N1 IAVs	Human	H1N1		1986	Taiwan	A/Taiwan/01/1986	ggtgatgccccattccttgatcggcttcgccgagatcag aagtccctaaagggaagaggcagcactc	+	+
CY020569	Human H1N1 IAVs	Human	H1N1		1986	USA	A/Texas/2922_3/1986	ggtgatgccccattccttgatcggcttcgccgagatcag aagtccctaaagggaagaggcagcactc	+	+
CY019775	Human H1N1 IAVs	Human	H1N1		1987	USA	A/Memphis/3/1987	ggtgatgccccattccttgatcggcttcgccgagatcag aagtccctaaagggaagaggcagcactc	+	+
CY045856	Human H1N1 IAVs	Human	H1N1		1987	Italy	A/Siena/4/1987	ggtgatgccccattccttgatcggcttcgccgagatcag aagtccctaaagggaagaggcagcactc	+	+
CY021977	Human H1N1 IAVs	Human	H1N1		1987	USA	A/Memphis/1/1987	ggtgatgccccattccttgatcggcttcgccgagatcag aagtccctaaagggaagaggtagcactc	+	+
CY019783	Human H1N1 IAVs	Human	H1N1		1987	USA	A/Memphis/4/1987	ggtgatgccccattccttgatcggcttcgccgagatcag aagtccctaaagggaagaggtagcactc	+	+
CY113481	Human H1N1 IAVs	Human	H3N2		1989	Australia	A/Victoria/1/1989	ggcgatgccccattccttgatcggcttcgccgagatca gaaatccctaagaggaaggggcagtactc	-	-
CY036827	Human H1N1 IAVs	Human	H1N1		1989	Italy	A/Siena/10/1989	ggtgatgccccattccttgatcggcttcgccgagatcaa aagtccctaaagggaagaggcagcactc	-	-
CY036947	Human H1N1 IAVs	Human	H1N1		1989	Italy	A/Siena/9/1989	ggtgatgccccattccttgatcggcttcgccgagatcag aagtccctaaagggaagaggcagcactc	+	+
CY147386	Human H1N1 IAVs	Human	H1N1		1991	USA	A/Texas/36_JY2/1991	ggtgatgccccattccttgatcggcttcgccgagatcag aagtccctaaagggaagaggcagcactc	+	+
DQ508893	Human H1N1 IAVs	Human	H1N1		1991	USA	A/Texas/36/1991	ggtgatgccccattccttgatcggcttcgccgagatcag aagtccctaaagggaagaggcagcactc	+	+
CY033602	Human H1N1 IAVs	Human	H1N1		1991	USA	A/Texas/36/1991	ggtgatgccccattccttgatcggcttcgccgagatcag aagtccctaaagggaagaggcagcactc	+	+
CY009320	Human H1N1 IAVs	Human	H1N1		1991	USA	A/Texas/36/91	ggtgatgccccattccttgatcggcttcgccgagatcag aagtccctaaagggaagaggcagcactc	+	+
CY125040	Human H1N1 IAVs	Human	H1N1		1992	New_Zealand	A/Wellington/47/1992	ggtgatgccccattccttgatcggcttcgccgagatcag aagtccctaaagggaagaggcagcactc	+	+
AF055422	Human H1N1 IAVs	Human	H1N1		1992	Taiwan	A/Taiwan/2243/1992	ggtgatgccccattccttgatcggcttcgccgagatcag aagtccctaaagggaagaggcagcactc	+	+
CY125048	Human H1N1 IAVs	Human	H1N1		1993	USA	A/Florida/2/1993	ggtgatgccccattccttgatcggcttcgccgagatcag aagtccctaaagggaagaggcagcactc	+	+
CY013307	Human H1N1 IAVs	Human	H1N1		1995	USA	A/New_York/656/1995	ggtgacgccccattccttgatcggcttcgccgagatca gaagtccctaaagggaagaagcagcactc	+	+
CY013283	Human H1N1 IAVs	Human	H1N1		1995	USA	A/New_York/643/1995	ggtgatgccccattccttgatcggctccgccgagatca gaaatccctaaagggaagaggcagcactc	-	*

CY010496	Human H1N1 IAVs	Human	H1N1		1995	USA	A/New_York/605/1995	ggtgatgccccattccttgatcggcttcgccgagatcag aaatccctaaagggaagaggcagcactc	-	*
CY010536	Human H1N1 IAVs	Human	H1N1		1995	USA	A/New_York/621/1995	ggtgatgccccattccttgatcggcttcgccgagatcag aaatccctaaagggaagaggcagcactc	-	*
CY010512	Human H1N1 IAVs	Human	H1N1		1995	USA	A/New_York/615/1995	ggtgatgccccattccttgatcggcttcgccgagatcag aaatccctaaagggaagaggcagcactc	-	*
AF398877	Human H1N1 IAVs	Human	H1N1		1995	USA	A/Charlottesville/31/95	ggtgatgccccattccttgatcggcttcgccgagatcag aagtccctaaagggaagaggcagcactc	+	+
CY011804	Human H1N1 IAVs	Human	H1N1		1995	USA	A/New_York/629/1995	ggtgatgccccattccttgatcggcttcgccgagatcag aagtccctaaagggaagaggcagcactc	+	+
CY117695	Human H1N1 IAVs	Human	H1N1		1995	Malaysia	A/Malaysia/06768/1995	ggtgatgccccattccttgatcggcttcgccgagatcag aagtccctaaagggaagaggcagcactc	+	+
CY010528	Human H1N1 IAVs	Human	H1N1		1995	USA	A/New_York/620/1995	ggtgatgccccattccttgatcggcttcgccgagatcag aagtccctaaagggaagaggcagcactc	+	+
CY010504	Human H1N1 IAVs	Human	H1N1		1995	USA	A/New_York/607/1995	ggtgatgccccattccttgatcggcttcgccgagatcag aagtccctaaagggaagaggcagcactc	+	+
CY013875	Human H1N1 IAVs	Human	H1N1		1995	USA	A/New_York/616/1995	ggtgatgccccattccttgatcggcttcgccgagatcag aagtccctaaagggaagaggcagcactc	+	+
CY012860	Human H1N1 IAVs	Human	H1N1		1995	USA	A/New_York/630/1995	ggtgatgccccattccttgatcggcttcgccgagatcag aagtccctaaagggaagaggcagcactc	+	+
CY012868	Human H1N1 IAVs	Human	H1N1		1995	USA	A/New_York/642/1995	ggtgatgccccattccttgatcggcttcgccgagatcag aagtccctaaagggaagaggcagcactc	+	+
CY011276	Human H1N1 IAVs	Human	H1N1		1995	USA	A/New_York/645/1995	ggtgatgccccattccttgatcggcttcgccgagatcag aagtccctaaagggaagaggcagcactc	+	+
CY010832	Human H1N1 IAVs	Human	H1N1		1995	USA	A/New_York/651/1995	ggtgatgccccattccttgatcggcttcgccgagatcag aagtccctaaagggaagaggcagcactc	+	+
CY015536	Human H1N1 IAVs	Human	H1N1		1995	USA	A/New_York/694/1995	ggtgatgccccattccttgatcggcttcgccgagatcag aagtccctaaagggaagaggcagcactc	+	+
CY125056	Human H1N1 IAVs	Human	H1N1		1995	China	A/Shengzhen/227/1995	ggtgatgccccattccttgatcggcttcgccgagatcag aagtccctaaagggaagaggcagcactc	+	+
CY010488	Human H1N1 IAVs	Human	H1N1		1995	USA	A/New_York/604/1995	ggtgatgccccattccttgatcggcttcgccgagatcag aagtccctaaagggaagaggcagcactc	+	+
CY010808	Human H1N1 IAVs	Human	H1N1		1995	USA	A/New_York/614/1995	ggtgatgccccattccttgatcggcttcgccgagatcag aagtccctaaagggaagaggcagcactc	+	+
CY010544	Human H1N1 IAVs	Human	H1N1		1995	USA	A/New_York/627/1995	ggtgatgccccattccttgatcggcttcgccgagatcag aagtccctaaagggaagaggcagcactc	+	+
CY010824	Human H1N1 IAVs	Human	H1N1		1995	USA	A/New_York/638/1995	ggtgatgccccattccttgatcggcttcgccgagatcag aagtccctaaagggaagaggcagcactc	+	+
CY016967	Human H1N1 IAVs	Human	H1N1		1995	USA	A/New_York/633/1995	ggtgatgccccattccttgatcggcttcgccgagatcag aagtccctaaagggaagaggcagcactc	+	+
CY010744	Human H1N1 IAVs	Human	H1N1		1995	USA	A/New_York/644/1995	ggtgatgccccattccttgatcggcttcgccgagatcag aagtccctaaagggaagaggcagcactc	+	+
CY036843	Human H1N1 IAVs	Human	H1N1		1995	Italy	A/Siena/14/1995	ggtgatgccccattccttgatcggcttcgccgagatcag aagtccctaaagggaagaggcagcactc	+	+
CY011284	Human H1N1 IAVs	Human	H1N1		1995	USA	A/New_York/649/1995	ggtgatgccccattccttgatcggcttcgccgagatcag aagtccctaaagggaagaggcagcactc	+	+
CY012876	Human H1N1 IAVs	Human	H1N1		1995	USA	A/New_York/650/1995	ggtgatgccccattccttgatcggcttcgccgagatcag aagtccctaaagggaagaggcagcactc	+	+
CY033618	Human H1N1 IAVs	Human	H1N1		1995	China	A/Beijing/262/1995	ggtgatgccccattccttgatcggcttcgccgagatcag aagtctctaaagggaagaggcagcactc	+	+

DQ415360	Human H1N1 IAVs	Human	H1N1		1996	Taiwan	A/TW/130/96	ggtgatgccccattccttgatcgacttcgccgagatcag aagtccctaaagggaagaggcagcactc	+	+
CY019113	Human H1N1 IAVs	Human	H1N1		1996	USA	A/Memphis/2/1996	ggtgatgccccattccttgatcggcttcgccgagatcag aagtccctaaagggaagaggcagcactc	+	+
CY019121	Human H1N1 IAVs	Human	H1N1		1996	USA	A/Memphis/3/1996	ggtgatgccccattccttgatcggcttcgccgagatcag aagtccctaaagggaagaggcagcactc	+	+
CY019791	Human H1N1 IAVs	Human	H1N1		1996	USA	A/Memphis/6/1996	ggtgatgccccattccttgatcggcttcgccgagatcag aagtccctaaagggaagaggcagcactc	+	+
CY125064	Human H1N1 IAVs	Human	H1N1		1996	China	A/Shanghai/8/1996	ggtgatgccccattccttgatcggcttcgccgagatcag aagtccctaaagggaagaggcagcactc	+	+
CY019137	Human H1N1 IAVs	Human	H1N1		1996	USA	A/Memphis/7/1996	ggtgatgccccattccttgatcggcttcgccgagatcag aagtccctaaagggaagaggcagcactc	+	+
CY010840	Human H1N1 IAVs	Human	H1N1		1996	USA	A/New_York/653/1996	ggtgatgccccattccttgatcggcttcgccgagatcag aagtccctaaagggaagaggcagcactc	+	+
CY011796	Human H1N1 IAVs	Human	H1N1		1996	USA	A/New_York/626/1996	ggtgatgccccattccttgatcggcttcgccgagatcag aagtccctaaagggaagaggcagcactc	+	+
CY019799	Human H1N1 IAVs	Human	H1N1		1996	USA	A/Memphis/10/1996	ggtgatgccccattccttgatcggcttcgccgagatcag aagtccctaaagggaagaggcagcactc	+	+
CY019807	Human H1N1 IAVs	Human	H1N1		1996	USA	A/Memphis/11/1996	ggtgatgccccattccttgatcggcttcgccgagatcag aagtccctaaagggaagaggcagcactc	+	+
CY019871	Human H1N1 IAVs	Human	H1N1		1996	USA	A/Memphis/13/1996	ggtgatgccccattccttgatcggcttcgccgagatcag aagtccctaaagggaagaggcagcactc	+	+
CY010848	Human H1N1 IAVs	Human	H1N1		1996	USA	A/New_York/640/1996	ggtgatgccccattccttgatcggcttcgccgagatcag aagtccctaaagggaagaggcagcactc	+	+
CY013291	Human H1N1 IAVs	Human	H1N1		1996	USA	A/New_York/646/1996	ggtgatgccccattccttgatcggcttcgccgagatcag aagtccctaaagggaagaggcagcactc	+	+
CY020257	Human H1N1 IAVs	Human	H1N1		1996	USA	A/Memphis/14/1996	ggtgatgccccattccttgatcggcttcgccgagatcag aagtccctaaagggaagaggcagcactc	+	+
CY019129	Human H1N1 IAVs	Human	H1N1		1996	USA	A/Memphis/5/1996	ggtgatgccccattccttgatcggcttcgccgagatcag aagtccctaaagggaagaggcagcactc	+	+
CY021009	Human H1N1 IAVs	Human	H1N1		1996	USA	A/Memphis/15/1996	ggtgatgccccattccttgatcggcttcgccgagatcag aagtccctaaagggaagaggcagcactc	+	+
AF055425	Human H1N1 IAVs	Human	H1N1		1996	Taiwan	A/Taiwan/118/1996	ggtgatgccccattccttgatcggcttcgccgagatcag aagtccctaaagggaagaggcagcactc	+	+
AF055423	Human H1N1 IAVs	Human	H1N1		1996	Taiwan	A/Taiwan/112/1996	ggtgatgccccattccttgatcggcttcgccgagatcag aagtccctaaagggaagaggcagcactc	+	+
AF055424	Human H1N1 IAVs	Human	H1N1		1996	Taiwan	A/Taiwan/117/1996	ggtgatgccccattccttgatcggcttcgccgagatcag aagtccctaaatggaagaggcagcactc	+	+
CY016232	Human H1N1 IAVs	Human	H1N1		1996	China	A/Nanchang/8/1996	ggtgatgccccattccttgatcggcttcgccgagatcag aagtctctaaagggaagaggcagcactc	+	+
CY016240	Human H1N1 IAVs	Human	H1N1		1996	China	A/Nanchang/11/1996	ggtgatgccccattccttgatcggcttcgccgggatcag aagtctctaaagggaagaggcagcactc	+	+
CY013817	Human H1N1 IAVs	Human	H1N1		1996	China	A/Nanchang/13/1996	ggtgatgccccattccttgatcggcttcgccgggatcag aagtctctaaagggaagaggcagcactc	+	+
CY017015	Human H1N1 IAVs	Human	H1N1		1996	China	A/Nanchang/14/1996	ggtgatgccccattccttgatcggcttcgccgggatcag aagtctctaaagggaagaggcagcactc	+	+
CY013833	Human H1N1 IAVs	Human	H1N1		1996	China	A/Nanchang/16/1996	ggtgatgccccattccttgatcggcttcgccgggatcag aagtctctaaagggaagaggcagcactc	+	+
CY013841	Human H1N1 IAVs	Human	H1N1		1996	China	A/Nanchang/17/1996	ggtgatgccccattccttgatcggcttcgccgggatcag aagtctctaaagggaagaggcagcactc	+	+

CY013849	Human H1N1 IAVs	Human	H1N1		1996	China	A/Nanchang/19/1996	ggtgatgccccattccttgatcggcttcgccgggatcag<br>aagtctctaaagggaagaggcagcactc	+	+
CY016056	Human H1N1 IAVs	Human	H1N1		1996	China	A/Nanchang/21/1996	ggtgatgccccattccttgatcggcttcgccgggatcag<br>aagtctctaaagggaagaggcagcactc	+	+
CY016248	Human H1N1 IAVs	Human	H1N1		1996	China	A/Nanchang/23/1996	ggtgatgccccattccttgatcggcttcgccgggatcag<br>aagtctctaaagggaagaggcagcactc	+	+
CY016256	Human H1N1 IAVs	Human	H1N1		1996	China	A/Nanchang/25/1996	ggtgatgccccattccttgatcggcttcgccgggatcag<br>aagtctctaaagggaagaggcagcactc	+	+
CY013857	Human H1N1 IAVs	Human	H1N1		1996	China	A/Nanchang/26/1996	ggtgatgccccattccttgatcggcttcgccgggatcag<br>aagtctctaaagggaagaggcagcactc	+	+
CY017007	Human H1N1 IAVs	Human	H1N1		1996	China	A/Nanchang/9/1996	ggtgatgccccattccttgatcggcttcgccgggatcag<br>aagtctctaaagggaagaggcagcactc	+	+
CY013825	Human H1N1 IAVs	Human	H1N1		1996	China	A/Nanchang/15/1996	ggtgatgccccattccttgatcggcttcgccgggatcag<br>aagtctctaaagggaagaggcagcactc	+	+
CY117812	Human H1N1 IAVs	Human	mixed		1997	Malaysia	A/Malaysia/13990/1997_mixed_	ggcgatgccccattccttgatcggcttcgccgagatca<br>gaagtctctaaagggaagaggcagcactc	-	-
CY117748	Human H1N1 IAVs	Human	H1N1		1997	Malaysia	A/Malaysia/12530/1997	ggtgatgccccattccttgatcggcttcgccgagatcag<br>aagtccctaaagggaagaggcagcactc	+	+
CY125072	Human H1N1 IAVs	Human	H1N1		1997	South_Africa	A/Johannesburg/159/1997	ggtgatgccccattccttgatcggcttcgccgagatcag<br>aagtctctaaagggaagaggcagcaatc	+	+
CY119070	Human H1N1 IAVs	Human	H1N1		1997	Malaysia	A/Malaysia/11641/1997	ggtgatgccccattccttgatcggcttcgccgagatcag<br>aagtctctaaagggaagaggcagcactc	+	+
CY119078	Human H1N1 IAVs	Human	H1N1		1997	Malaysia	A/Malaysia/11642/1997	ggtgatgccccattccttgatcggcttcgccgagatcag<br>aagtctctaaagggaagaggcagcactc	+	+
CY119118	Human H1N1 IAVs	Human	H1N1		1997	Malaysia	A/Malaysia/14817/1997	ggtgatgccccattccttgatcggcttcgccgagatcag<br>aagtctctaaagggaagaggcagcactc	+	+
CY125080	Human H1N1 IAVs	Human	H1N1		1997	China	A/Shanghai/2/1997	ggtgatgccccattccttgatcggcttcgccgagatcag<br>aagtctctaaagggaagaggcagcactc	+	+
DQ415361	Human H1N1 IAVs	Human	H1N1		1997	Taiwan	A/TW/3355/97	ggtgatgccccattccttgatcggcttcgccgagatcag<br>aagtctctaaagggaagaggcagcactc	+	+
CY125088	Human H1N1 IAVs	Human	H1N1		1998	Russia	A/Moscow/13/1998	ggtgatgccccattccttgatcggcttcgccgagatcag<br>aagtccctaaagggaagaggcagcactc	+	+
CY125096	Human H1N1 IAVs	Human	H1N1		1998	Czech_Republic	A/Ostrava/801/1998	ggtgatgccccattccttgatcggcttcgccgagatcag<br>aagtctctaaagggaagaggcagcactc	+	+
CY119126	Human H1N1 IAVs	Human	H1N1		1998	Malaysia	A/Malaysia/15042/1998	ggtgatgccccattccttgatcggcttcgccgagatcag<br>aagtctctaaagggaagaggcagcactc	+	+
CY147322	Human H1N1 IAVs	Human	H1N1		1999	New_Caledonia	A/New_Caledonia/20_JY2/1999	ggcgatgccccattccttgatcggcttcgccgagatca<br>gaagtctctaaagggaagaggcagcactc	-	-
DQ508861	Human H1N1 IAVs	Human	H1N1		1999	New_Caledonia	A/New_Caledonia/20/1999	ggcgatgccccattccttgatcggcttcgccgagatca<br>gaagtctctaaagggaagaggcagcactc	-	-
CY033626	Human H1N1 IAVs	Human	H1N1		1999	New_Caledonia	A/New_Caledonia/20/1999	ggcgatgccccattccttgatcggcttcgccgagatca<br>gaagtctctaaagggaagaggcagcactc	-	-
CY016647	Human H1N1 IAVs	Human	H1N1		1999	Australia	A/New_South_Wales/18/1999	ggcgatgccccattccttgatcggcttcgccgagatca<br>gaagtctctaaagggaagaggcagcactc	-	-
CY017127	Human H1N1 IAVs	Human	H1N1		1999	Australia	A/New_South_Wales/24/1999	ggcgatgccccattccttgatcggcttcgccgagatca<br>gaagtctctaaagggaagaggcagcactc	-	-
CY040142	Human H1N1 IAVs	Human	H1N1		1999	Taiwan	A/Taiwan/5072/1999	ggcgatgccccattccttgatcggcttcgtcgagatcag<br>aagtctctaaagggaagaggcagcactc	+	+
DQ415362	Human H1N1 IAVs	Human	H1N1		1999	Taiwan	A/TW/4845/99	ggcgatgccccattccttgatcggcttcgtcgagatcag<br>aagtctctaaagggaagaggcagcactc	+	+

CY125016	Human H1N1 IAVs	Human	H1N1		1999	China	A/Nanchang/16A/1999	ggtgatgccccattccttgatcggcttcgccgagatcag<br>aagtctctaaagggaagaggcagcactc	+	+
CY021697	Human H1N1 IAVs	Human	H1N1		2000	USA	A/Memphis/15/2000	ggcgatgccccattccttgatcgacttcgccgagatca<br>gaagtctctaaagggaagaggcagcactc	+	+
CY119102	Human H1N1 IAVs	Human	H1N1		2000	Malaysia	A/Malaysia/14075/2000	ggcgatgccccattccttgatcggcttcgccgagatca<br>gaagtctctaaagggaagaggcaacactc	-	-
CY119094	Human H1N1 IAVs	Human	H1N1		2000	Malaysia	A/Malaysia/14458/2000	ggcgatgccccattccttgatcggcttcgccgagatca<br>gaagtctctaaagggaagaggcaacactc	-	-
CY022161	Human H1N1 IAVs	Human	H1N1		2000	New_Zealand	A/Auckland/580/2000	ggcgatgccccattccttgatcggcttcgccgagatca<br>gaagtctctaaagggaagaggcagcactc	-	-
CY125112	Human H1N1 IAVs	Human	H1N1		2000	Thailand	A/Bangkok/163/2000	ggcgatgccccattccttgatcggcttcgccgagatca<br>gaagtctctaaagggaagaggcagcactc	-	-
CY125120	Human H1N1 IAVs	Human	H1N1		2000	Brazil	A/Brazil/74200/2000	ggcgatgccccattccttgatcggcttcgccgagatca<br>gaagtctctaaagggaagaggcagcactc	-	-
CY010392	Human H1N1 IAVs	Human	H1N1		2000	New_Zealand	A/Canterbury/63/2000	ggcgatgccccattccttgatcggcttcgccgagatca<br>gaagtctctaaagggaagaggcagcactc	-	-
CY016567	Human H1N1 IAVs	Human	H1N1		2000	Australia	A/New_South_Wales/30/2000	ggcgatgccccattccttgatcggcttcgccgagatca<br>gaagtctctaaagggaagaggcagcactc	-	-
CY010104	Human H1N1 IAVs	Human	H1N1		2000	New_Zealand	A/Canterbury/27/2000	ggcgatgccccattccttgatcggcttcgccgagatca<br>gaagtctctaaagggaagaggcagcactc	-	-
CY016264	Human H1N1 IAVs	Human	H1N1		2000	Australia	A/New_South_Wales/26/2000	ggcgatgccccattccttgatcggcttcgccgagatca<br>gaagtctctaaagggaagaggcagcactc	-	-
CY119110	Human H1N1 IAVs	Human	H1N1		2000	Malaysia	A/Malaysia/14210/2000	ggcgatgccccattccttgatcggcttcgccgagatca<br>gaagtctctaaagggaagaggcagcactc	-	-
CY022153	Human H1N1 IAVs	Human	H1N1		2000	New_Zealand	A/Auckland/579/2000	ggcgatgcccccttccttgatcggcttcgccgagatca<br>gaagtctctaaagggaagaggcagcactc	+	+
CY022497	Human H1N1 IAVs	Human	H1N1		2000	New_Zealand	A/Auckland/587/2000	ggcgatgcccccttccttgatcggcttcgccgagatca<br>gaagtctctaaagggaagaggcagcactc	+	+
CY022513	Human H1N1 IAVs	Human	H1N1		2000	New_Zealand	A/Auckland/590/2000	ggcgatgcccccttccttgatcggcttcgccgagatca<br>gaagtctctaaagggaagaggcagcactc	+	+
CY023014	Human H1N1 IAVs	Human	H1N1		2000	New_Zealand	A/Auckland/591/2000	ggcgatgcccccttccttgatcggcttcgccgagatca<br>gaagtctctaaagggaagaggcagcactc	+	+
CY009816	Human H1N1 IAVs	Human	H1N1		2000	New_Zealand	A/Canterbury/78/2000	ggcgatgcccccttccttgatcggcttcgccgagatca<br>gaagtctctaaagggaagaggcagcactc	+	+
CY016200	Human H1N1 IAVs	Human	H1N1		2000	New_Zealand	A/Wellington/14/2000	ggcgatgcccccttccttgatcggcttcgccgagatca<br>gaagtctctaaagggaagaggcagcactc	+	+
CY012604	Human H1N1 IAVs	Human	H1N1		2000	New_Zealand	A/Wellington/11/2000	ggcgatgcccccttccttgatcggcttcgccgagatca<br>gaagtctctaaagggaagaggcagcactc	+	+
CY022169	Human H1N1 IAVs	Human	H1N1		2000	New_Zealand	A/Auckland/581/2000	ggcgatgcccccttccttgatcggcttcgccgagatca<br>gaagtctctaaagggaagaggcagcactc	+	+
CY025030	Human H1N1 IAVs	Human	H1N1		2000	New_Zealand	A/Auckland/584/2000	ggcgatgcccccttccttgatcggcttcgccgagatca<br>gaagtctctaaagggaagaggcagcactc	+	+
CY026159	Human H1N1 IAVs	Human	H1N1		2000	New_Zealand	A/Auckland/585/2000	ggcgatgcccccttccttgatcggcttcgccgagatca<br>gaagtctctaaagggaagaggcagcactc	+	+
CY022489	Human H1N1 IAVs	Human	H1N1		2000	New_Zealand	A/Auckland/586/2000	ggcgatgcccccttccttgatcggcttcgccgagatca<br>gaagtctctaaagggaagaggcagcactc	+	+
CY025038	Human H1N1 IAVs	Human	H1N1		2000	New_Zealand	A/Auckland/597/2000	ggcgatgcccccttccttgatcggcttcgccgagatca<br>gaagtctctaaagggaagaggcagcactc	+	+
CY010136	Human H1N1 IAVs	Human	H1N1		2000	New_Zealand	A/Canterbury/100/2000	ggcgatgcccccttccttgatcggcttcgccgagatca<br>gaagtctctaaagggaagaggcagcactc	+	+

CY009760	Human H1N1 IAVs	Human	H1N1		2000	New_Zealand	A/Canterbury/23/2000	ggcgatgcccccttccttgatcggcttcgccgagatca<br>gaagtctctaaagggaagaggcagcactc	+	+
CY009192	Human H1N1 IAVs	Human	H1N1		2000	New_Zealand	A/Canterbury/28/2000	ggcgatgcccccttccttgatcggcttcgccgagatca<br>gaagtctctaaagggaagaggcagcactc	+	+
CY009184	Human H1N1 IAVs	Human	H1N1		2000	New_Zealand	A/Canterbury/30/2000	ggcgatgcccccttccttgatcggcttcgccgagatca<br>gaagtctctaaagggaagaggcagcactc	+	+
CY009224	Human H1N1 IAVs	Human	H1N1		2000	New_Zealand	A/Canterbury/32/2000	ggcgatgcccccttccttgatcggcttcgccgagatca<br>gaagtctctaaagggaagaggcagcactc	+	+
CY009216	Human H1N1 IAVs	Human	H1N1		2000	New_Zealand	A/Canterbury/33/2000	ggcgatgcccccttccttgatcggcttcgccgagatca<br>gaagtctctaaagggaagaggcagcactc	+	+
CY009232	Human H1N1 IAVs	Human	H1N1		2000	New_Zealand	A/Canterbury/34/2000	ggcgatgcccccttccttgatcggcttcgccgagatca<br>gaagtctctaaagggaagaggcagcactc	+	+
CY009536	Human H1N1 IAVs	Human	H1N1		2000	New_Zealand	A/Canterbury/37/2000	ggcgatgcccccttccttgatcggcttcgccgagatca<br>gaagtctctaaagggaagaggcagcactc	+	+
CY011780	Human H1N1 IAVs	Human	H1N1		2000	New_Zealand	A/Canterbury/48/2000	ggcgatgcccccttccttgatcggcttcgccgagatca<br>gaagtctctaaagggaagaggcagcactc	+	+
CY009544	Human H1N1 IAVs	Human	H1N1		2000	New_Zealand	A/Canterbury/5/2000	ggcgatgcccccttccttgatcggcttcgccgagatca<br>gaagtctctaaagggaagaggcagcactc	+	+
CY010112	Human H1N1 IAVs	Human	H1N1		2000	New_Zealand	A/Canterbury/51/2000	ggcgatgcccccttccttgatcggcttcgccgagatca<br>gaagtctctaaagggaagaggcagcactc	+	+
CY010128	Human H1N1 IAVs	Human	H1N1		2000	New_Zealand	A/Canterbury/54/2000	ggcgatgcccccttccttgatcggcttcgccgagatca<br>gaagtctctaaagggaagaggcagcactc	+	+
CY010120	Human H1N1 IAVs	Human	H1N1		2000	New_Zealand	A/Canterbury/58/2000	ggcgatgcccccttccttgatcggcttcgccgagatca<br>gaagtctctaaagggaagaggcagcactc	+	+
CY009824	Human H1N1 IAVs	Human	H1N1		2000	New_Zealand	A/Canterbury/60/2000	ggcgatgcccccttccttgatcggcttcgccgagatca<br>gaagtctctaaagggaagaggcagcactc	+	+
CY009768	Human H1N1 IAVs	Human	H1N1		2000	New_Zealand	A/Canterbury/7/2000	ggcgatgcccccttccttgatcggcttcgccgagatca<br>gaagtctctaaagggaagaggcagcactc	+	+
CY009792	Human H1N1 IAVs	Human	H1N1		2000	New_Zealand	A/Canterbury/76/2000	ggcgatgcccccttccttgatcggcttcgccgagatca<br>gaagtctctaaagggaagaggcagcactc	+	+
CY009800	Human H1N1 IAVs	Human	H1N1		2000	New_Zealand	A/Canterbury/79/2000	ggcgatgcccccttccttgatcggcttcgccgagatca<br>gaagtctctaaagggaagaggcagcactc	+	+
CY009808	Human H1N1 IAVs	Human	H1N1		2000	New_Zealand	A/Canterbury/87/2000	ggcgatgcccccttccttgatcggcttcgccgagatca<br>gaagtctctaaagggaagaggcagcactc	+	+
CY013036	Human H1N1 IAVs	Human	H1N1		2000	New_Zealand	A/Wellington/18/2000	ggcgatgcccccttccttgatcggcttcgccgagatca<br>gaagtctctaaagggaagaggcagcactc	+	+
CY016975	Human H1N1 IAVs	Human	H1N1		2000	New_Zealand	A/Wellington/24/2000	ggcgatgcccccttccttgatcggcttcgccgagatca<br>gaagtctctaaagggaagaggcagcactc	+	+
CY012300	Human H1N1 IAVs	Human	H1N1		2000	New_Zealand	A/Wellington/7/2000	ggcgatgcccccttccttgatcggcttcgccgagatca<br>gaagtctctaaagggaagaggcagcactc	+	+
CY009832	Human H1N1 IAVs	Human	H1N1		2000	New_Zealand	A/Canterbury/41/2000	ggcgatgcccccttccttgatcggcttcgccgagatca<br>gaagtctctaaagggaagaggcagcactc	+	+
CY011788	Human H1N1 IAVs	Human	H1N1		2000	New_Zealand	A/Canterbury/55/2000	ggcgatgcccccttccttgatcggcttcgccgagatca<br>gaagtctctaaagggaagaggcagcactc	+	+
CY009552	Human H1N1 IAVs	Human	H1N1		2000	New_Zealand	A/Canterbury/65/2000	ggcgatgcccccttccttgatcggcttcgccgagatca<br>gaagtctctaaagggaagaggcagcactc	+	+
CY022177	Human H1N1 IAVs	Human	H1N1		2000	New_Zealand	A/Auckland/582/2000	ggcgatgcccccttccttgatcggcttcgccgagatca<br>gaagtctctaaagggaagaggcagcactc	+	+
CY023022	Human H1N1 IAVs	Human	H1N1		2000	New_Zealand	A/Auckland/595/2000	ggcgatgcccccttccttgatcggcttcgccgagatca<br>gaagtctctaaagggaagaggcagcactc	+	+

CY010144	Human H1N1 IAVs	Human	H1N1		2000	New_Zealand	A/Canterbury/95/2000	ggcgatgcccccttccttgatcggcttcgccgagatca<br>gaagtctctaaagggaagaggcagcactc	+	+
CY013044	Human H1N1 IAVs	Human	H1N1		2000	New_Zealand	A/Waikato/18/2000	ggcgatgcccccttccttgatcggcttcgccgagatca<br>gaagtctctaaagggaagaggcagcactc	+	+
CY011956	Human H1N1 IAVs	Human	H1N1		2000	New_Zealand	A/Waikato/19/2000	ggcgatgcccccttccttgatcggcttcgccgagatca<br>gaagtctctaaagggaagaggcagcactc	+	+
CY009200	Human H1N1 IAVs	Human	H1N1		2000	New_Zealand	A/Canterbury/36/2000	ggcgatgcccccttccttgatcggcttcgccgagatca<br>gaagtctctaaagggaagaggcagcactc	+	+
CY125128	Human H1N1 IAVs	Human	H1N1		2000	China	A/Fujian/156/2000	ggcgatgcccccttccttgatcggcttcgccgagatca<br>gaagtctctaaagggaagaggcagcactc	+	+
CY009776	Human H1N1 IAVs	Human	H1N1		2000	New_Zealand	A/South_Canterbury/31/2000	ggcgatgcccccttccttgatcggcttcgccgagatca<br>gaagtctctaaagggaagaggcagcactc	+	+
CY009784	Human H1N1 IAVs	Human	H1N1		2000	New_Zealand	A/South_Canterbury/40/2000	ggcgatgcccccttccttgatcggcttcgccgagatca<br>gaagtctctaaagggaagaggcagcactc	+	+
CY009176	Human H1N1 IAVs	Human	H1N1		2000	New_Zealand	A/South_Canterbury/50/2000	ggcgatgcccccttccttgatcggcttcgccgagatca<br>gaagtctctaaagggaagaggcagcactc	+	+
CY009840	Human H1N1 IAVs	Human	H1N1		2000	New_Zealand	A/South_Canterbury/59/2000	ggcgatgcccccttccttgatcggcttcgccgagatca<br>gaagtctctaaagggaagaggcagcactc	+	+
CY011000	Human H1N1 IAVs	Human	H1N1		2000	New_Zealand	A/Wellington/2/2000	ggcgatgcccccttccttgatcggcttcgccgagatca<br>gaagtctctaaagggaagaggcagcactc	+	+
CY011008	Human H1N1 IAVs	Human	H1N1		2000	New_Zealand	A/Wellington/3/2000	ggcgatgcccccttccttgatcggcttcgccgagatca<br>gaagtctctaaagggaagaggcagcactc	+	+
CY021633	Human H1N1 IAVs	Human	H1N1		2000	New_Zealand	A/Wellington/4/2000	ggcgatgcccccttccttgatcggcttcgccgagatca<br>gaagtctctaaagggaagaggcagcactc	+	+
CY011016	Human H1N1 IAVs	Human	H1N1		2000	New_Zealand	A/Wellington/5/2000	ggcgatgcccccttccttgatcggcttcgccgagatca<br>gaagtctctaaagggaagaggcagcactc	+	+
CY020513	Human H1N1 IAVs	Human	H1N1		2000	New_Zealand	A/Wellington/6/2000	ggcgatgcccccttccttgatcggcttcgccgagatca<br>gaagtctctaaagggaagaggcagcactc	+	+
CY009208	Human H1N1 IAVs	Human	H1N1		2000	New_Zealand	A/South_Canterbury/35/2000	ggcgatgcccccttccttgatcggcttcgccgagatca<br>gaagtctctaaagggaagaggcagcactc	+	+
CY010096	Human H1N1 IAVs	Human	H1N1		2000	New_Zealand	A/Canterbury/43/2000	ggcgatgcccccttccttgatcggcttcgccgagatca<br>gaagtctctaaagggaagaggcagcactc	+	+
CY009848	Human H1N1 IAVs	Human	H1N1		2000	New_Zealand	A/Canterbury/57/2000	ggcgatgcccccttccttgatcggcttcgccgagatca<br>gaagtctctaaagggaagaggcagcactc	+	+
CY010384	Human H1N1 IAVs	Human	H1N1		2000	New_Zealand	A/Canterbury/8/2000	ggcgatgcccccttccttgatcggcttcgccgagatca<br>gaagtctctaaagggaagaggcagcactc	+	+
CY009944	Human H1N1 IAVs	Human	H1N1		2000	New_Zealand	A/Canterbury/9/2000	ggcgatgcccccttccttgatcggcttcgccgagatca<br>gaagtctctaaagggaagaggcagcactc	+	+
CY012612	Human H1N1 IAVs	Human	H1N1		2000	New_Zealand	A/Wellington/16/2000	ggcgatgcccccttccttgatcggcttcgccgagatca<br>gaagtctctaaagggaagaggcagcactc	+	+
CY011588	Human H1N1 IAVs	Human	H1N1		2000	New_Zealand	A/Dunedin/2/2000	ggcgatgcccccttccttgatcggcttcgccgagatca<br>gaagtctctaaagggaagaggcagcactc	+	+
CY002652	Human H1N1 IAVs	Human	H1N1		2000	USA	A/New_York/234/2000	ggcgatgcccccttccttgatcggcttcgccgagatca<br>gaagtctctaaagggaagaggcagcactc	+	+
CY017143	Human H1N1 IAVs	Human	H1N1		2000	Australia	A/South_Australia/25/2000	ggtgatgccccattccttgatcggcttcgccgagatcag<br>aaatccctaaagggaagaggcagcactc	-	*
CY125136	Human H1N1 IAVs	Human	H1N1		2000	Chile	A/Chile/4795/2000	ggtgatgccccattccttgatcggcttcgccgagatcag<br>aagtccctaaagggaagaggcagcactc	+	+
CY125670	Human H1N1 IAVs	Human	H1N1		2000	United_Kingdom	A/England/192/2000	ggtgatgccccattccttgatcggcttcgccgagatcag<br>aagtccctaaagggaagaggcagcactc	+	+

CY000453	Human H1N1 IAVs	Human	H1N1		2000	USA	A/New_York/146/2000	ggtgatgccccattccttgatcggcttcgccgagatcag aagtccctaaagggaagaggcagcactc	+	+
CY017383	Human H1N1 IAVs	Human	H1N1		2000	Australia	A/South_Australia/26/2000	ggtgatgccccattccttgatcggcttcgccgagatcag aagtccctaaagggaagaggcagcactc	+	+
CY017023	Human H1N1 IAVs	Human	H1N1		2000	Australia	A/South_Australia/29/2000	ggtgatgccccattccttgatcggcttcgccgagatcag aagtccctaaagggaagaggcagcactc	+	+
CY017817	Human H1N1 IAVs	Human	H1N1		2000	Australia	A/South_Australia/30/2000	ggtgatgccccattccttgatcggcttcgccgagatcag aagtccctaaagggaagaggcagcactc	+	+
CY017151	Human H1N1 IAVs	Human	H1N1		2000	Australia	A/South_Australia/36/2000	ggtgatgccccattccttgatcggcttcgccgagatcag aagtccctaaagggaagaggcagcactc	+	+
CY016671	Human H1N1 IAVs	Human	H1N1		2000	Australia	A/South_Australia/45/2000	ggtgatgccccattccttgatcggcttcgccgagatcag aagtccctaaagggaagaggcagcactc	+	+
CY016735	Human H1N1 IAVs	Human	H1N1		2000	Australia	A/South_Australia/64/2000	ggtgatgccccattccttgatcggcttcgccgagatcag aagtccctaaagggaagaggcagcactc	+	+
CY017159	Human H1N1 IAVs	Human	H1N1		2000	Australia	A/South_Australia/46/2000	ggtgatgccccattccttgatcggcttcgccgagatcag aagtccctaaagggaagaggcagcactc	+	+
CY021753	Human H1N1 IAVs	Human	H1N1		2000	Australia	A/South_Australia/44/2000	ggtgatgccccattccttgatcggcttcgccgagatcag aagtccctaaagggaagaggcagcactc	+	+
CY016727	Human H1N1 IAVs	Human	H1N1		2000	Australia	A/South_Australia/63/2000	ggtgatgccccattccttgatcggcttcgccgagatcag aagtccctaaagggaagaggcagcactc	+	+
CY018937	Human H1N1 IAVs	Human	H1N1		2000	Australia	A/South_Australia/40/2000	ggtgatgccccattccttgatcggcttcgccgagatcag aagtccctaaagggaagaggcagcactc	+	+
CY017833	Human H1N1 IAVs	Human	H1N1		2000	Australia	A/South_Australia/39/2000	ggtgatgccccgttccttgatcggcttcgccgagatcag aagtccctaaagggaagaggcagcactc	-	-
CY002676	Human H1N1 IAVs	Human	H1N1		2001	USA	A/New_York/310/2001	ggcgatgccccattccttgatcgacttcgccgagatca gaagtctctaaagggaagaggcagcactc	+	+
CY009240	Human H1N1 IAVs	Human	H1N1		2001	USA	A/New_York/441/2001	ggcgatgccccattccttgatcgacttcgccgagatca gaagtctctaaagggaagaggcagcactc	+	+
CY020265	Human H1N1 IAVs	Human	H1N1		2001	USA	A/Memphis/1/2001	ggcgatgccccattccttgatcggattcgccgagatca gaagtctctaaagggaagaggcagcactc	+	+
CY020145	Human H1N1 IAVs	Human	H1N1		2001	USA	A/Memphis/6/2001	ggcgatgccccattccttgatcggcttcgccgagatca gaaatctctaaagggaagaggcagcactc	-	-
CY001956	Human H1N1 IAVs	Human	H1N1		2001	USA	A/New_York/205/2001	ggcgatgccccattccttgatcggcttcgccgagatca gaagtctctaaagggaagaggcagcactc	-	-
CY006423	Human H1N1 IAVs	Human	H1N1		2001	USA	A/New_York/212/2001	ggcgatgccccattccttgatcggcttcgccgagatca gaagtctctaaagggaagaggcagcactc	-	-
CY003316	Human H1N1 IAVs	Human	H1N1		2001	USA	A/New_York/302/2001	ggcgatgccccattccttgatcggcttcgccgagatca gaagtctctaaagggaagaggcagcactc	-	-
CY003396	Human H1N1 IAVs	Human	H1N1		2001	USA	A/New_York/305/2001	ggcgatgccccattccttgatcggcttcgccgagatca gaagtctctaaagggaagaggcagcactc	-	-
CY002396	Human H1N1 IAVs	Human	H1N1		2001	USA	A/New_York/343/2001	ggcgatgccccattccttgatcggcttcgccgagatca gaagtctctaaagggaagaggcagcactc	-	-
CY003468	Human H1N1 IAVs	Human	H1N1		2001	USA	A/New_York/442/2001	ggcgatgccccattccttgatcggcttcgccgagatca gaagtctctaaagggaagaggcagcactc	-	-
CY003292	Human H1N1 IAVs	Human	H1N1		2001	USA	A/New_York/444/2001	ggcgatgccccattccttgatcggcttcgccgagatca gaagtctctaaagggaagaggcagcactc	-	-
CY002620	Human H1N1 IAVs	Human	H1N1		2001	USA	A/New_York/208/2001	ggcgatgccccattccttgatcggcttcgccgagatca gaagtctctaaagggaagaggcagcactc	-	-
CY019879	Human H1N1 IAVs	Human	H1N1		2001	USA	A/Memphis/8/2001	ggcgatgccccattccttgatcggcttcgccgagatca gaagtctctaaagggaagaggcagcactc	-	-

CY003404	Human H1N1 IAVs	Human	H1N1		2001	USA	A/New_York/306/2001	ggcgatgccccattccttgatcggcttcgccgagatca gaagtctctaaagggaagaggcagcactc	-	-
CY002700	Human H1N1 IAVs	Human	H1N1		2001	USA	A/New_York/312/2001	ggcgatgccccattccttgatcggcttcgccgagatca gaagtctctaaagggaagaggcagcactc	-	-
CY006367	Human H1N1 IAVs	Human	H1N1		2001	USA	A/New_York/303/2001	ggcgatgccccattccttgatcggcttcgccgagatca gaagtctctaaagggaagaggcagcactc	-	-
CY020153	Human H1N1 IAVs	Human	H1N1		2001	USA	A/Memphis/7/2001	ggcgatgccccattccttgatcggcttcgccgagatca gaagtctctaaagggaagaggcagcactc	-	-
CY020273	Human H1N1 IAVs	Human	H1N1		2001	Australia	A/Western_Australia/18/2001	ggcgatgcccccttccttgatcggcttcgccgagatca gaagtctctaaagggaagaggcagcactc	+	+
CY010400	Human H1N1 IAVs	Human	H1N1		2001	New_Zealand	A/Canterbury/01/2001	ggcgatgcccccttccttgatcggcttcgccgagatca gaagtctctaaagggaagaggcagcactc	+	+
CY010272	Human H1N1 IAVs	Human	H1N1		2001	New_Zealand	A/Canterbury/53/2001	ggcgatgcccccttccttgatcggcttcgccgagatca gaagtctctaaagggaagaggcagcactc	+	+
CY103984	Human H1N1 IAVs	Human	H1N1		2001	Viet_Nam	A/HaNoi/2253/2001	ggcgatgcccccttccttgatcggcttcgccgagatca gaagtctctaaagggaagaggcagcactc	+	+
CY003476	Human H1N1 IAVs	Human	H1N1		2001	USA	A/New_York/443/2001	ggcgatgcccccttccttgatcggcttcgccgagatca gaagtctctaaagggaagaggcagcactc	+	+
CY011156	Human H1N1 IAVs	Human	H1N1		2001	New_Zealand	A/Wellington/1/2001	ggcgatgcccccttccttgatcggcttcgccgagatca gaagtctctaaagggaagaggcagcactc	+	+
CY103976	Human H1N1 IAVs	Human	H1N1		2001	Viet_Nam	A/HaNoi/2017/2001	ggcgatgcccccttccttgatcggcttcgccgagatca gaagtctctaaagggaagaggcagcactc	+	+
CY104698	Human H1N1 IAVs	Human	H1N1		2001	Viet_Nam	A/HaNoi/2480/2001	ggcgatgcccccttccttgatcggcttcgccgagatca gaagtctctaaagggaagaggcagcactc	+	+
CY104000	Human H1N1 IAVs	Human	H1N1		2001	Viet_Nam	A/HaNoi/2532/2001	ggcgatgcccccttccttgatcggcttcgccgagatca gaagtctctaaagggaagaggcagcactc	+	+
CY103992	Human H1N1 IAVs	Human	H1N1		2001	Viet_Nam	A/HaNoi/2476/2001	ggcgatgcccccttccttgatcggcttcgccgagatca gaagtctctaaagggaagaggcagcactc	+	+
CY022537	Human H1N1 IAVs	Human	H1N1		2001	New_Zealand	A/Auckland/605/2001	ggcgatgcccccttccttgatcggcttcgccgagatca gaagtctctaaagggaagaggcagcactc	+	+
CY022545	Human H1N1 IAVs	Human	H1N1		2001	New_Zealand	A/Auckland/606/2001	ggcgatgcccccttccttgatcggcttcgccgagatca gaagtctctaaagggaagaggcagcactc	+	+
CY019209	Human H1N1 IAVs	Human	H1N1		2001	New_Zealand	A/Canterbury/05/2001	ggcgatgcccccttccttgatcggcttcgccgagatca gaagtctctaaagggaagaggcagcactc	+	+
CY011092	Human H1N1 IAVs	Human	H1N1		2001	New_Zealand	A/Canterbury/152/2001	ggcgatgcccccttccttgatcggcttcgccgagatca gaagtctctaaagggaagaggcagcactc	+	+
CY010160	Human H1N1 IAVs	Human	H1N1		2001	New_Zealand	A/Canterbury/19/2001	ggcgatgcccccttccttgatcggcttcgccgagatca gaagtctctaaagggaagaggcagcactc	+	+
CY010184	Human H1N1 IAVs	Human	H1N1		2001	New_Zealand	A/Canterbury/24/2001	ggcgatgcccccttccttgatcggcttcgccgagatca gaagtctctaaagggaagaggcagcactc	+	+
CY010248	Human H1N1 IAVs	Human	H1N1		2001	New_Zealand	A/Canterbury/45/2001	ggcgatgcccccttccttgatcggcttcgccgagatca gaagtctctaaagggaagaggcagcactc	+	+
CY010264	Human H1N1 IAVs	Human	H1N1		2001	New_Zealand	A/Canterbury/48/2001	ggcgatgcccccttccttgatcggcttcgccgagatca gaagtctctaaagggaagaggcagcactc	+	+
CY010432	Human H1N1 IAVs	Human	H1N1		2001	New_Zealand	A/Canterbury/65/2001	ggcgatgcccccttccttgatcggcttcgccgagatca gaagtctctaaagggaagaggcagcactc	+	+
CY010456	Human H1N1 IAVs	Human	H1N1		2001	New_Zealand	A/Canterbury/70/2001	ggcgatgcccccttccttgatcggcttcgccgagatca gaagtctctaaagggaagaggcagcactc	+	+
CY010464	Human H1N1 IAVs	Human	H1N1		2001	New_Zealand	A/Canterbury/71/2001	ggcgatgcccccttccttgatcggcttcgccgagatca gaagtctctaaagggaagaggcagcactc	+	+

CY010472	Human H1N1 IAVs	Human	H1N1		2001	New_Zealand	A/Canterbury/79/2001	ggcgatgcccccttccttgatcggcttcgccgagatca gaagtctctaaagggaagaggcagcactc	+	+
CY010360	Human H1N1 IAVs	Human	H1N1		2001	New_Zealand	A/South_Canterbury/159/2001	ggcgatgcccccttccttgatcggcttcgccgagatca gaagtctctaaagggaagaggcagcactc	+	+
CY010152	Human H1N1 IAVs	Human	H1N1		2001	New_Zealand	A/Canterbury/17/2001	ggcgatgcccccttccttgatcggcttcgccgagatca gaagtctctaaagggaagaggcagcactc	+	+
CY016439	Human H1N1 IAVs	Human	H1N1		2001	New_Zealand	A/Waikato/92/2001	ggcgatgcccccttccttgatcggcttcgccgagatca gaagtctctaaagggaagaggcagcactc	+	+
CY009880	Human H1N1 IAVs	Human	H1N1		2001	New_Zealand	A/Canterbury/16/2001	ggcgatgcccccttccttgatcggcttcgccgagatca gaagtctctaaagggaagaggcagcactc	+	+
CY010776	Human H1N1 IAVs	Human	H1N1		2001	New_Zealand	A/Canterbury/22/2001	ggcgatgcccccttccttgatcggcttcgccgagatca gaagtctctaaagggaagaggcagcactc	+	+
CY011412	Human H1N1 IAVs	Human	H1N1		2001	New_Zealand	A/Canterbury/26/2001	ggcgatgcccccttccttgatcggcttcgccgagatca gaagtctctaaagggaagaggcagcactc	+	+
CY010216	Human H1N1 IAVs	Human	H1N1		2001	New_Zealand	A/Canterbury/30/2001	ggcgatgcccccttccttgatcggcttcgccgagatca gaagtctctaaagggaagaggcagcactc	+	+
CY010224	Human H1N1 IAVs	Human	H1N1		2001	New_Zealand	A/Canterbury/34/2001	ggcgatgcccccttccttgatcggcttcgccgagatca gaagtctctaaagggaagaggcagcactc	+	+
CY010256	Human H1N1 IAVs	Human	H1N1		2001	New_Zealand	A/Canterbury/47/2001	ggcgatgcccccttccttgatcggcttcgccgagatca gaagtctctaaagggaagaggcagcactc	+	+
CY010280	Human H1N1 IAVs	Human	H1N1		2001	New_Zealand	A/Canterbury/58/2001	ggcgatgcccccttccttgatcggcttcgccgagatca gaagtctctaaagggaagaggcagcactc	+	+
CY011084	Human H1N1 IAVs	Human	H1N1		2001	New_Zealand	A/Canterbury/60/2001	ggcgatgcccccttccttgatcggcttcgccgagatca gaagtctctaaagggaagaggcagcactc	+	+
CY009872	Human H1N1 IAVs	Human	H1N1		2001	New_Zealand	A/South_Canterbury/15/2001	ggcgatgcccccttccttgatcggcttcgccgagatca gaagtctctaaagggaagaggcagcactc	+	+
CY011196	Human H1N1 IAVs	Human	H1N1		2001	New_Zealand	A/Waikato/16/2001	ggcgatgcccccttccttgatcggcttcgccgagatca gaagtctctaaagggaagaggcagcactc	+	+
CY011204	Human H1N1 IAVs	Human	H1N1		2001	New_Zealand	A/Waikato/20/2001	ggcgatgcccccttccttgatcggcttcgccgagatca gaagtctctaaagggaagaggcagcactc	+	+
CY011612	Human H1N1 IAVs	Human	H1N1		2001	New_Zealand	A/Wellington/32/2001	ggcgatgcccccttccttgatcggcttcgccgagatca gaagtctctaaagggaagaggcagcactc	+	+
CY011076	Human H1N1 IAVs	Human	H1N1		2001	New_Zealand	A/West_Coast/31/2001	ggcgatgcccccttccttgatcggcttcgccgagatca gaagtctctaaagggaagaggcagcactc	+	+
CY020281	Human H1N1 IAVs	Human	H1N1		2001	Australia	A/Western_Australia/19/2001	ggcgatgcccccttccttgatcggcttcgccgagatca gaagtctctaaagggaagaggcagcactc	+	+
CY011604	Human H1N1 IAVs	Human	H1N1		2001	New_Zealand	A/Waikato/3/2001	ggcgatgcccccttccttgatcggcttcgccgagatca gaagtctctaaagggaagaggcagcactc	+	+
CY011180	Human H1N1 IAVs	Human	H1N1		2001	New_Zealand	A/Wellington/3/2001	ggcgatgcccccttccttgatcggcttcgccgagatca gaagtctctaaagggaagaggcagcactc	+	+
CY011228	Human H1N1 IAVs	Human	H1N1		2001	New_Zealand	A/Waikato/51/2001	ggcgatgcccccttccttgatcggcttcgccgagatca gaagtctctaaagggaagaggcagcactc	+	+
CY012308	Human H1N1 IAVs	Human	H1N1		2001	New_Zealand	A/Wellington/17/2001	ggcgatgcccccttccttgatcggcttcgccgagatca gaagtctctaaagggaagaggcagcactc	+	+
CY011220	Human H1N1 IAVs	Human	H1N1		2001	New_Zealand	A/Wellington/9/2001	ggcgatgcccccttccttgatcggcttcgccgagatca gaagtctctaaagggaagaggcagcactc	+	+
CY010784	Human H1N1 IAVs	Human	H1N1		2001	New_Zealand	A/Canterbury/42/2001	ggcgatgcccccttccttgatcggcttcgccgagatca gaagtctctaaagggaagaggcagcactc	+	+
CY010336	Human H1N1 IAVs	Human	H1N1		2001	New_Zealand	A/Canterbury/139/2001	ggcgatgcccccttccttgatcggcttcgccgagatca gaagtctctaaagggaagaggcagcactc	+	+

CY010296	Human H1N1 IAVs	Human	H1N1		2001	New_Zealand	A/Canterbury/72/2001	ggcgatgcccccttccttgatcggcttcgccgagatca<br>gaagtctctaaagggaagaggcagcactc	+	+
CY010408	Human H1N1 IAVs	Human	H1N1		2001	New_Zealand	A/West_Coast/33/2001	ggcgatgcccccttccttgatcggcttcgccgagatca<br>gaagtctctaaagggaagaggcagcactc	+	+
CY010480	Human H1N1 IAVs	Human	H1N1		2001	New_Zealand	A/Canterbury/106/2001	ggcgatgcccccttccttgatcggcttcgccgagatca<br>gaagtctctaaagggaagaggcagcactc	+	+
CY010344	Human H1N1 IAVs	Human	H1N1		2001	New_Zealand	A/Canterbury/144/2001	ggcgatgcccccttccttgatcggcttcgccgagatca<br>gaagtctctaaagggaagaggcagcactc	+	+
CY010200	Human H1N1 IAVs	Human	H1N1		2001	New_Zealand	A/Canterbury/27/2001	ggcgatgcccccttccttgatcggcttcgccgagatca<br>gaagtctctaaagggaagaggcagcactc	+	+
CY010208	Human H1N1 IAVs	Human	H1N1		2001	New_Zealand	A/Canterbury/29/2001	ggcgatgcccccttccttgatcggcttcgccgagatca<br>gaagtctctaaagggaagaggcagcactc	+	+
CY010240	Human H1N1 IAVs	Human	H1N1		2001	New_Zealand	A/Canterbury/40/2001	ggcgatgcccccttccttgatcggcttcgccgagatca<br>gaagtctctaaagggaagaggcagcactc	+	+
CY010288	Human H1N1 IAVs	Human	H1N1		2001	New_Zealand	A/Canterbury/64/2001	ggcgatgcccccttccttgatcggcttcgccgagatca<br>gaagtctctaaagggaagaggcagcactc	+	+
CY009960	Human H1N1 IAVs	Human	H1N1		2001	New_Zealand	A/Canterbury/66/2001	ggcgatgcccccttccttgatcggcttcgccgagatca<br>gaagtctctaaagggaagaggcagcactc	+	+
CY010440	Human H1N1 IAVs	Human	H1N1		2001	New_Zealand	A/Canterbury/68/2001	ggcgatgcccccttccttgatcggcttcgccgagatca<br>gaagtctctaaagggaagaggcagcactc	+	+
CY010304	Human H1N1 IAVs	Human	H1N1		2001	New_Zealand	A/Canterbury/73/2001	ggcgatgcccccttccttgatcggcttcgccgagatca<br>gaagtctctaaagggaagaggcagcactc	+	+
CY125160	Human H1N1 IAVs	Human	H1N1		2001	New_Zealand	A/Christchurch/4/2001	ggcgatgcccccttccttgatcggcttcgccgagatca<br>gaagtctctaaagggaagaggcagcactc	+	+
CY020433	Human H1N1 IAVs	Human	H1N1		2001	Australia	A/Western_Australia/22/2001	ggcgatgcccccttccttgatcggcttcgccgagatca<br>gaagtctctaaagggaagaggcagcactc	+	+
CY011164	Human H1N1 IAVs	Human	H1N1		2001	New_Zealand	A/Waikato/4/2001	ggcgatgcccccttccttgatcggcttcgccgagatca<br>gaagtctctaaagggaagaggcagcactc	+	+
CY011188	Human H1N1 IAVs	Human	H1N1		2001	New_Zealand	A/Waikato/10/2001	ggcgatgcccccttccttgatcggcttcgccgagatca<br>gaagtctctaaagggaagaggcagcactc	+	+
CY011212	Human H1N1 IAVs	Human	H1N1		2001	New_Zealand	A/Waikato/18/2001	ggcgatgcccccttccttgatcggcttcgccgagatca<br>gaagtctctaaagggaagaggcagcactc	+	+
CY011236	Human H1N1 IAVs	Human	H1N1		2001	New_Zealand	A/Waikato/42/2001	ggcgatgcccccttccttgatcggcttcgccgagatca<br>gaagtctctaaagggaagaggcagcactc	+	+
CY011172	Human H1N1 IAVs	Human	H1N1		2001	New_Zealand	A/Waikato/7/2001	ggcgatgcccccttccttgatcggcttcgccgagatca<br>gaagtctctaaagggaagaggcagcactc	+	+
CY020425	Human H1N1 IAVs	Human	H1N1		2001	Australia	A/Western_Australia/21/2001	ggcgatgcccccttccttgatcggcttcgccgagatca<br>gaagtctctaaagggaagaggcagcactc	+	+
CY011396	Human H1N1 IAVs	Human	H1N1		2001	New_Zealand	A/Waikato/80/2001	ggcgatgcccccttccttgatcggcttcgccgagatca<br>gaagtctctaaagggaagaggcagcactc	+	+
CY019927	Human H1N1 IAVs	Human	H1N1		2001	Australia	A/Western_Australia/20/2001	ggcgatgcccccttccttgatcggcttcgccgagatca<br>gaagtctctaaagggaagaggcagcactc	+	+
CY010320	Human H1N1 IAVs	Human	H1N1		2001	New_Zealand	A/Canterbury/125/2001	ggcgatgcccccttccttgatcggcttcgccgagatca<br>gaagtctctaaagggaagaggcagcactc	+	+
CY009984	Human H1N1 IAVs	Human	H1N1		2001	New_Zealand	A/Canterbury/153/2001	ggcgatgcccccttccttgatcggcttcgccgagatca<br>gaagtctctaaagggaagaggcagcactc	+	+
CY010768	Human H1N1 IAVs	Human	H1N1		2001	New_Zealand	A/Canterbury/20/2001	ggcgatgcccccttccttgatcggcttcgccgagatca<br>gaagtctctaaagggaagaggcagcactc	+	+
CY010168	Human H1N1 IAVs	Human	H1N1		2001	New_Zealand	A/Canterbury/21/2001	ggcgatgcccccttccttgatcggcttcgccgagatca<br>gaagtctctaaagggaagaggcagcactc	+	+

CY010176	Human H1N1 IAVs	Human	H1N1		2001	New_Zealand	A/Canterbury/23/2001	ggcgatgcccccttccttgatcggcttcgccgagatca gaagtctctaaagggaagaggcagcactc	+	+
CY010192	Human H1N1 IAVs	Human	H1N1		2001	New_Zealand	A/Canterbury/25/2001	ggcgatgcccccttccttgatcggcttcgccgagatca gaagtctctaaagggaagaggcagcactc	+	+
CY009968	Human H1N1 IAVs	Human	H1N1		2001	New_Zealand	A/Canterbury/74/2001	ggcgatgcccccttccttgatcggcttcgccgagatca gaagtctctaaagggaagaggcagcactc	+	+
CY009976	Human H1N1 IAVs	Human	H1N1		2001	New_Zealand	A/Canterbury/76/2001	ggcgatgcccccttccttgatcggcttcgccgagatca gaagtctctaaagggaagaggcagcactc	+	+
CY010232	Human H1N1 IAVs	Human	H1N1		2001	New_Zealand	A/Canterbury/35/2001	ggcgatgcccccttccttgatcggcttcgccgagatca gaagtctctaaagggaagaggcagcactc	+	+
CY010448	Human H1N1 IAVs	Human	H1N1		2001	New_Zealand	A/Canterbury/69/2001	ggcgatgcccccttccttgatcggcttcgccgagatca gaagtctctaaagggaagaggcagcactc	+	+
CY009888	Human H1N1 IAVs	Human	H1N1		2001	New_Zealand	A/Canterbury/41/2001	ggcgatgcccccttccttgatcggcttcgccgagatca gaagtctctaaagggaagaggcagcactc	+	+
CY010424	Human H1N1 IAVs	Human	H1N1		2001	New_Zealand	A/Canterbury/63/2001	ggcgatgcccccttccttgatcggcttcgccgagatca gaagtctctaaagggaagaggcagcactc	+	+
CY011244	Human H1N1 IAVs	Human	H1N1		2001	New_Zealand	A/Wellington/28/2001	ggcgatgcccccttccttgatcggcttcgccgagatca gaagtctctaaagggaagaggcagcactc	+	+
CY010352	Human H1N1 IAVs	Human	H1N1		2001	New_Zealand	A/Canterbury/155/2001	ggcgatgcccccttccttgatcggcttcgccgagatca gaagtctctaaagggaagaggcagcactc	+	+
CY010416	Human H1N1 IAVs	Human	H1N1		2001	New_Zealand	A/Canterbury/51/2001	ggcgatgcccccttccttgatcggcttcgccgagatca gaagtctctaaagggaagaggcagcactc	+	+
CY009864	Human H1N1 IAVs	Human	H1N1		2001	New_Zealand	A/Canterbury/08/2001	ggcgatgcccccttccttgatcggcttcgccgagatca gaagtctctaaagggaagaggcagcactc	+	+
CY010328	Human H1N1 IAVs	Human	H1N1		2001	New_Zealand	A/Canterbury/126/2001	ggcgatgcccccttccttgatcggcttcgccgagatca gaagtctctaaagggaagaggcagcactc	+	+
CY003012	Human H1N1 IAVs	Human	H1N1		2001	USA	A/New_York/241/2001	ggcgatgcccccttccttgatcggcttcgccgagatca gaagtctctaaagggaagaggcagcactc	+	+
CY006359	Human H1N1 IAVs	Human	H1N1		2001	USA	A/New_York/242/2001	ggcgatgcccccttccttgatcggcttcgccgagatca gaagtctctaaagggaagaggcagcactc	+	+
CY002804	Human H1N1 IAVs	Human	H1N1		2001	USA	A/New_York/308/2001	ggcgatgcccccttccttgatcggcttcgccgagatca gaagtctctaaagggaagaggcagcactc	+	+
CY003028	Human H1N1 IAVs	Human	H1N1		2001	USA	A/New_York/341/2001	ggcgatgcccccttccttgatcggcttcgccgagatca gaagtctctaaagggaagaggcagcactc	+	+
CY006879	Human H1N1 IAVs	Human	H1N1		2001	USA	A/New_York/309/2001	ggcgatgcccccttccttgatcggcttcgccgagatca gaagtctctaaagggaagaggcagcactc	+	+
CY006783	Human H1N1 IAVs	Human	H1N1		2001	USA	A/New_York/344/2001	ggcgatgcccccttccttgatcggcttcgccgagatca gaagtctctaaagggaagaggcagcactc	+	+
CY003837	Human H1N1 IAVs	Human	H1N1		2001	USA	A/New_York/445/2001	ggcgatgcccccttccttgatcggcttcgccgagatca gaagtctctaaagggaagaggcagcactc	+	+
CY002572	Human H1N1 IAVs	Human	H1N1		2001	USA	A/New_York/281/2001	ggcgatgcccccttccttgatcggcttcgccgagatca gaagtctctaaagggaagaggcagcactc	+	+
CY003484	Human H1N1 IAVs	Human	H1N1		2001	USA	A/New_York/446/2001	ggcgatgcccccttccttgatcggcttcgccgagatca gaagtctctaaagggaagaggcagcactc	+	+
CY006175	Human H1N1 IAVs	Human	H1N1		2001	USA	A/New_York/447/2001	ggcgatgcccccttccttgatcggcttcgccgagatca gaagtctctaaagggaagaggcagcactc	+	+
CY010856	Human H1N1 IAVs	Human	H1N1		2001	USA	A/New_York/235/2001	ggcgatgcccccttccttgatcggcttcgccgagatca gaagtctctaaagggaagaggcagcactc	+	+
CY125144	Human H1N1 IAVs	Human	H1N1		2001	Chile	A/Chile/8885/2001	ggcgatgcccccttccttgatcggcttcgccgagatca gaagtctctaaagggaagaggcagcactc	+	+

CY104690	Human H1N1 IAVs	Human	H1N1		2001	Viet_Nam	A/HaNoi/2143/2001	ggcgatgccccccttccttgatcggcttcgccgagatca gaagtctctaaagggaagaggcagcactc	+	+
CY125152	Human H1N1 IAVs	Human	H1N1		2001	Singapore	A/Singapore/14/2001	ggcgatgccccccttccttgatcggcttcgccgagatca gaagtctctaaagggaagaggcagcactc	+	+
CY010312	Human H1N1 IAVs	Human	H1N1		2001	New_Zealand	A/Canterbury/119/2001	ggcgatgctcccttccttgatcggcttcgccgagatcag aagtctctaaagggaagaggcagcactc	-	-
CY083002	Human H1N1 IAVs	Swine	H1N1		2001	South_Korea	A/swine/KU/8/2001	ggtgatgccccattccttgatcggcttcgccgagatcag aaatccctaagaggaaggggcagcactc	-	*
CY083003	Human H1N1 IAVs	Swine	H1N1		2001	South_Korea	A/swine/KU/9/2001	ggtgatgccccattccttgatcggcttcgccgagatcag aaatccctaagaggaaggggcagcactc	-	*
CY008152	Human H1N1 IAVs	Human	H1N1		2001	USA	A/New_York/307/2001	ggtgatgccccattccttgatcggcttcgccgagatcag aagtccctaaagggaagaggcagcactc	+	+
CY104016	Human H1N1 IAVs	Human	H1N1		2002	Viet_Nam	A/HaNoi/2704/2002	ggcgatgccccccttccttgatcggcttcgccgagatca gaaatctctaaagggaagaggcagcactc	-	*
CY104008	Human H1N1 IAVs	Human	H1N1		2002	Viet_Nam	A/HaNoi/2546/2002	ggcgatgccccccttccttgatcggcttcgccgagatca gaagtctctaaagggaagaggcagcactc	+	+
CY125184	Human H1N1 IAVs	Human	H1N1		2002	Mongolia	A/Neimenggu/52/2002	ggcgatgccccccttccttgatcggcttcgccgagatca gaagtctctaaagggaagaggcagcactc	+	+
CY002532	Human H1N1 IAVs	Human	H1N1		2002	USA	A/New_York/220/2002	ggcgatgccccccttccttgatcggcttcgccgagatca gaagtctctaaagggaagaggcagcactc	+	+
CY040078	Human H1N1 IAVs	Human	H1N1		2002	Taiwan	A/Taiwan/567/2002	ggcgatgccccccttccttgatcggcttcgccgagatca gaagtctctaaagggaagaggcagcactc	+	+
CY040150	Human H1N1 IAVs	Human	H1N1		2002	Taiwan	A/Taiwan/52/2002	ggcgatgccccccttccttgatcggcttcgccgagatca gaagtctctaaagggaagaggcagcactc	+	+
DQ249270	Human H1N1 IAVs	Human	H1N1		2002	Taiwan	A/Taiwan/2985/2002	ggcgatgccccccttccttgatcggcttcgccgagatca gaagtctctaaagggaagaggcagcactc	+	+
CY125168	Human H1N1 IAVs	Human	H1N1		2002	Russia	A/Russia/2187/2002	ggcgatgccccccttccttgatcggcttcgccgagatca gaagtctctaaagggaagaggcagcactc	+	+
CY117836	Human H1N1 IAVs	Human	Mixed		2002	Malaysia	A/Malaysia/21870/2002	ggcgatgccccccttccttgatcggcttcgccgagatca gaagtctctaaagggaagaggcagcactc	+	+
CY006679	Human H1N1 IAVs	Human	H1N1		2002	USA	A/New_York/494/2002	ggcgatgccccccttccttgatcggcttcgccgagatca gaagtctctaaagggaagaggcagcactc	+	+
DQ249269	Human H1N1 IAVs	Human	H1N1		2002	Taiwan	A/Taiwan/30027/2002	ggcgatgcccccctttcttgatcggcttcgccgagatcaa aagtctctaaagggaaaaggcagcactc	-	*
AB434115	Human H1N1 IAVs	Human	H1N1		2002	Japan	A/Morioka/17/2002	ggcgatgcccccctttcttgatcggcttcgccgagatcag aagtctctaaagggaagaggcagcactc	-	-
AB434119	Human H1N1 IAVs	Human	H1N1		2002	Japan	A/Morioka/27/2002	ggcgatgcccccctttcttgatcggcttcgccgagatcag aagtctctaaagggaagaggcagcactc	-	-
CY040158	Human H1N1 IAVs	Human	H1N1		2002	Taiwan	A/Taiwan/123/2002	ggcgatgcccccctttcttgatcggcttcgccgagatcag aagtctctaaagggaagaggcagcactc	-	-
CY084235	Human H1N1 IAVs	Human	H1N1		2002	Taiwan	A/Taiwan/141/2002	ggcgatgcccccctttcttgatcggcttcgccgagatcag aagtctctaaagggaagaggcagcactc	-	-
CY006199	Human H1N1 IAVs	Human	H1N1		2003	USA	A/New_York/497/2003	ggcgatgccccccttccttgatcggcttcgccgagatca gaagtctctaaagagaagaggcagcactc	+	+
CY003380	Human H1N1 IAVs	Human	H1N1		2003	USA	A/New_York/292/2003	ggcgatgccccccttccttgatcggcttcgccgagatca gaagtctctaaagggaagaggcagaactc	+	+
CY002708	Human H1N1 IAVs	Human	H1N1		2003	USA	A/New_York/348/2003	ggcgatgccccccttccttgatcggcttcgccgagatca gaagtctctaaagggaagaggcagaactc	+	+
CY105202	Human H1N1 IAVs	Human	H1N1		2003	Viet_Nam	A/HaNoi/ARI172/2003	ggcgatgccccccttccttgatcggcttcgccgagatca gaagtctctaaagggaagaggcagcactc	+	+

CY104706	Human H1N1 IAVs	Human	H1N1		2003	Viet_Nam	A/HaNoi/ARI36/2003	ggcgatgccccсttccttgatcggcttcgccgagatca gaagtctctaaagggaagaggcagcactc	+	+
CY105226	Human H1N1 IAVs	Human	H1N1		2003	Viet_Nam	A/HaNoi/BM857/2003	ggcgatgcccccttccttgatcggcttcgccgagatca gaagtctctaaagggaagaggcagcactc	+	+
CY105234	Human H1N1 IAVs	Human	H1N1		2003	Viet_Nam	A/HaNoi/BM862/2003	ggcgatgcccccttccttgatcggcttcgccgagatca gaagtctctaaagggaagaggcagcactc	+	+
CY104714	Human H1N1 IAVs	Human	H1N1		2003	Viet_Nam	A/HaNoi/BM870/2003	ggcgatgcccccttccttgatcggcttcgccgagatca gaagtctctaaagggaagaggcagcactc	+	+
CY104722	Human H1N1 IAVs	Human	H1N1		2003	Viet_Nam	A/HaNoi/BM893/2003	ggcgatgcccccttccttgatcggcttcgccgagatca gaagtctctaaagggaagaggcagcactc	+	+
CY104024	Human H1N1 IAVs	Human	H1N1		2003	Viet_Nam	A/HaNoi/BM898/2003	ggcgatgcccccttccttgatcggcttcgccgagatca gaagtctctaaagggaagaggcagcactc	+	+
CY104730	Human H1N1 IAVs	Human	H1N1		2003	Viet_Nam	A/HaNoi/BM902/2003	ggcgatgcccccttccttgatcggcttcgccgagatca gaagtctctaaagggaagaggcagcactc	+	+
CY104738	Human H1N1 IAVs	Human	H1N1		2003	Viet_Nam	A/HaNoi/BM910/2003	ggcgatgcccccttccttgatcggcttcgccgagatca gaagtctctaaagggaagaggcagcactc	+	+
CY104746	Human H1N1 IAVs	Human	H1N1		2003	Viet_Nam	A/HaNoi/BM945/2003	ggcgatgcccccttccttgatcggcttcgccgagatca gaagtctctaaagggaagaggcagcactc	+	+
CY104032	Human H1N1 IAVs	Human	H1N1		2003	Viet_Nam	A/HaNoi/BM949/2003	ggcgatgcccccttccttgatcggcttcgccgagatca gaagtctctaaagggaagaggcagcactc	+	+
CY104754	Human H1N1 IAVs	Human	H1N1		2003	Viet_Nam	A/HaNoi/BM959/2003	ggcgatgcccccttccttgatcggcttcgccgagatca gaagtctctaaagggaagaggcagcactc	+	+
CY104040	Human H1N1 IAVs	Human	H1N1		2003	Viet_Nam	A/HaNoi/HN1004/2003	ggcgatgcccccttccttgatcggcttcgccgagatca gaagtctctaaagggaagaggcagcactc	+	+
CY104762	Human H1N1 IAVs	Human	H1N1		2003	Viet_Nam	A/HaNoi/HN1017/2003	ggcgatgcccccttccttgatcggcttcgccgagatca gaagtctctaaagggaagaggcagcactc	+	+
CY104048	Human H1N1 IAVs	Human	H1N1		2003	Viet_Nam	A/HaNoi/HN1024/2003	ggcgatgcccccttccttgatcggcttcgccgagatca gaagtctctaaagggaagaggcagcactc	+	+
CY104770	Human H1N1 IAVs	Human	H1N1		2003	Viet_Nam	A/HaNoi/HN337/2003	ggcgatgcccccttccttgatcggcttcgccgagatca gaagtctctaaagggaagaggcagcactc	+	+
CY104778	Human H1N1 IAVs	Human	H1N1		2003	Viet_Nam	A/HaNoi/HN603/2003	ggcgatgcccccttccttgatcggcttcgccgagatca gaagtctctaaagggaagaggcagcactc	+	+
CY104056	Human H1N1 IAVs	Human	H1N1		2003	Viet_Nam	A/HaNoi/HN719/2003	ggcgatgcccccttccttgatcggcttcgccgagatca gaagtctctaaagggaagaggcagcactc	+	+
CY104786	Human H1N1 IAVs	Human	H1N1		2003	Viet_Nam	A/HaNoi/HN756/2003	ggcgatgcccccttccttgatcggcttcgccgagatca gaagtctctaaagggaagaggcagcactc	+	+
CY104794	Human H1N1 IAVs	Human	H1N1		2003	Viet_Nam	A/HaNoi/HN777/2003	ggcgatgcccccttccttgatcggcttcgccgagatca gaagtctctaaagggaagaggcagcactc	+	+
CY104802	Human H1N1 IAVs	Human	H1N1		2003	Viet_Nam	A/HaNoi/HN859/2003	ggcgatgcccccttccttgatcggcttcgccgagatca gaagtctctaaagggaagaggcagcactc	+	+
CY104064	Human H1N1 IAVs	Human	H1N1		2003	Viet_Nam	A/HaNoi/HN867/2003	ggcgatgcccccttccttgatcggcttcgccgagatca gaagtctctaaagggaagaggcagcactc	+	+
CY104810	Human H1N1 IAVs	Human	H1N1		2003	Viet_Nam	A/HaNoi/HN979/2003	ggcgatgcccccttccttgatcggcttcgccgagatca gaagtctctaaagggaagaggcagcactc	+	+
CY104818	Human H1N1 IAVs	Human	H1N1		2003	Viet_Nam	A/HaNoi/HN981/2003	ggcgatgcccccttccttgatcggcttcgccgagatca gaagtctctaaagggaagaggcagcactc	+	+
CY104072	Human H1N1 IAVs	Human	H1N1		2003	Viet_Nam	A/TayNguyen/TN101/2003	ggcgatgcccccttccttgatcggcttcgccgagatca gaagtctctaaagggaagaggcagcactc	+	+
CY125192	Human H1N1 IAVs	Human	H1N1		2003	New_Zealand	A/Christchurch/1/2003	ggcgatgcccccttccttgatcggcttcgccgagatca gaagtctctaaagggaagaggcagcactc	+	+

CY019345	Human H1N1 IAVs	Human	H1N1		2003	USA	A/Memphis/6/2003	ggcgatgcccccttccttgatcggcttcgccgagatca<br>gaagtctctaaagggaagaggcagcactc	+	+
CY002692	Human H1N1 IAVs	Human	H1N1		2003	USA	A/New_York/223/2003	ggcgatgcccccttccttgatcggcttcgccgagatca<br>gaagtctctaaagggaagaggcagcactc	+	+
CY003692	Human H1N1 IAVs	Human	H1N1		2003	USA	A/New_York/486/2003	ggcgatgcccccttccttgatcggcttcgccgagatca<br>gaagtctctaaagggaagaggcagcactc	+	+
CY002812	Human H1N1 IAVs	Human	H1N1		2003	USA	A/New_York/399/2003	ggcgatgcccccttccttgatcggcttcgccgagatca<br>gaagtctctaaagggaagaggcagcactc	+	+
CY006919	Human H1N1 IAVs	Human	H1N1		2003	USA	A/New_York/488/2003	ggcgatgcccccttccttgatcggcttcgccgagatca<br>gaagtctctaaagggaagaggcagcactc	+	+
CY006671	Human H1N1 IAVs	Human	H1N1		2003	USA	A/New_York/493/2003	ggcgatgcccccttccttgatcggcttcgccgagatca<br>gaagtctctaaagggaagaggcagcactc	+	+
CY003708	Human H1N1 IAVs	Human	H1N1		2003	USA	A/New_York/496/2003	ggcgatgcccccttccttgatcggcttcgccgagatca<br>gaagtctctaaagggaagaggcagcactc	+	+
CY002540	Human H1N1 IAVs	Human	H1N1		2003	USA	A/New_York/227/2003	ggcgatgcccccttccttgatcggcttcgccgagatca<br>gaagtctctaaagggaagaggcagcactc	+	+
CY002628	Human H1N1 IAVs	Human	H1N1		2003	USA	A/New_York/230/2003	ggcgatgcccccttccttgatcggcttcgccgagatca<br>gaagtctctaaagggaagaggcagcactc	+	+
CY008528	Human H1N1 IAVs	Human	H1N1		2003	USA	A/New_York/483/2003	ggcgatgcccccttccttgatcggcttcgccgagatca<br>gaagtctctaaagggaagaggcagcactc	+	+
CY009000	Human H1N1 IAVs	Human	H1N1		2003	USA	A/New_York/484/2003	ggcgatgcccccttccttgatcggcttcgccgagatca<br>gaagtctctaaagggaagaggcagcactc	+	+
CY002684	Human H1N1 IAVs	Human	H1N1		2003	USA	A/New_York/222/2003	ggcgatgcccccttccttgatcggcttcgccgagatca<br>gaagtctctaaagggaagaggcagcactc	+	+
CY003388	Human H1N1 IAVs	Human	H1N1		2003	USA	A/New_York/293/2003	ggcgatgcccccttccttgatcggcttcgccgagatca<br>gaagtctctaaagggaagaggcagcactc	+	+
CY019887	Human H1N1 IAVs	Human	H1N1		2003	USA	A/Memphis/5/2003	ggcgatgcccccttccttgatcggcttcgccgagatca<br>gaagtctctaaagggaagaggcagcactc	+	+
CY002988	Human H1N1 IAVs	Human	H1N1		2003	USA	A/New_York/221/2003	ggcgatgcccccttccttgatcggcttcgccgagatca<br>gaagtctctaaagggaagaggcagcactc	+	+
CY003300	Human H1N1 IAVs	Human	H1N1		2003	USA	A/New_York/228/2003	ggcgatgcccccttccttgatcggcttcgccgagatca<br>gaagtctctaaagggaagaggcagcactc	+	+
CY119150	Human H1N1 IAVs	Human	H1N1		2003	Malaysia	A/Malaysia/25862/2003	ggcgatgcccccttccttgatcggcttcgccgagatca<br>gaagtctctaaagggaagaggcagcactc	+	+
CY119134	Human H1N1 IAVs	Human	H1N1		2003	Malaysia	A/Malaysia/25531/2003	ggcgatgcccccttccttgatcggcttcgccgagatca<br>gaagtctctaaagggaagaggcagcactc	+	+
CY119142	Human H1N1 IAVs	Human	H1N1		2003	Malaysia	A/Malaysia/25750/2003	ggcgatgcccccttccttgatcggcttcgccgagatca<br>gaagtctctaaagggaagaggcagcactc	+	+
CY006431	Human H1N1 IAVs	Human	H1N1		2003	USA	A/New_York/350/2003	ggcgatgcccccttccttgatcggcttcgccgagatca<br>gaagtctctaaagggaagaggcagcactc	+	+
CY117956	Human H1N1 IAVs	Human	mixed		2003	Malaysia	A/Malaysia/25405/2003	ggcgatgcccccttccttgatcggcttcgccgagatca<br>gaagtctctaaagggaagaggcagcactc	+	+
CY125200	Human H1N1 IAVs	Human	H1N1		2004	Canada	A/Canada/591/2004	ggcgatgcccccttccttgatcggcttcgccgagatca<br>gaagtctctaaagggaagaggcagcactc	+	+
CY125208	Human H1N1 IAVs	Human	H1N1		2004	Hong_Kong	A/Hong_Kong/2637/2004	ggcgatgcccccttccttgatcggcttcgccgagatca<br>gaagtctctaaagggaagaggcagcactc	+	+
CY007471	Human H1N1 IAVs	Human	H1N1		2004	New_Zealand	A/Canterbury/106/2004	ggcgatgcccccttccttgatcggcttcgccgagatca<br>gaagtctctaaagggaagaggcagcactc	+	+
CY119158	Human H1N1 IAVs	Human	H1N1		2004	Malaysia	A/Malaysia/30025/2004	ggcgatgcccccttccttgatcggcttcgccgagatca<br>gaagtctctaaagggaagaggcagcactc	+	+

EU004441	Human H1N1 IAVs	Swine	H1N1		2004	China	A/swine/Tianjin/01/2004	ggtgatgccccattccttgatcggcttcgccgagatcag aagtccctaaagggaagaggcagcactc	+	+
CY016463	Human H1N1 IAVs	Human	H1N1		2005	New_Zealand	A/Waikato/11/2005	ggagatgctcccttccttgatcggcttcgccgagatcag aagtctctaaagggaagaggcagcactc	+	+
CY013585	Human H1N1 IAVs	Human	H1N1		2005	New_Zealand	A/Waikato/13/2005	ggagatgctcccttccttgatcggcttcgccgagatcag aagtctctaaagggaagaggcagcactc	+	+
CY017319	Human H1N1 IAVs	Human	H1N1		2005	New_Zealand	A/Waikato/4/2005	ggagatgctcccttccttgatcggcttcgccgagatcag aagtctctaaagggaagaggcagcactc	+	+
CY020001	Human H1N1 IAVs	Human	H1N1		2005	New_Zealand	A/Waikato/17/2005	ggagatgctcccttccttgatcggcttcgccgagatcag aagtctctaaagggaagaggcagcactc	+	+
CY022585	Human H1N1 IAVs	Human	H1N1		2005	New_Zealand	A/Auckland/619/2005	ggcgatgccccccttccttgatcggcttcgccgagatca gaagtctctaaagggaagaggcagcactc	+	+
CY013577	Human H1N1 IAVs	Human	H1N1		2005	New_Zealand	A/Otago/5/2005	ggcgatgccccccttccttgatcggcttcgccgagatca gaagtctctaaagggaagaggcagcactc	+	+
CY021761	Human H1N1 IAVs	Human	H1N1		2005	Australia	A/South_Australia/51/2005	ggcgatgccccccttccttgatcggcttcgccgagatca gaagtctctaaagggaagaggcagcactc	+	+
CY016687	Human H1N1 IAVs	Human	H1N1		2005	Australia	A/South_Australia/56/2005	ggcgatgccccccttccttgatcggcttcgccgagatca gaagtctctaaagggaagaggcagcactc	+	+
CY016695	Human H1N1 IAVs	Human	H1N1		2005	Australia	A/South_Australia/57/2005	ggcgatgccccccttccttgatcggcttcgccgagatca gaagtctctaaagggaagaggcagcactc	+	+
CY013601	Human H1N1 IAVs	Human	H1N1		2005	New_Zealand	A/Waikato/14/2005	ggcgatgccccccttccttgatcggcttcgccgagatca gaagtctctaaagggaagaggcagcactc	+	+
CY020161	Human H1N1 IAVs	Human	H1N1		2005	Australia	A/Western_Australia/77/2005	ggcgatgccccccttccttgatcggcttcgccgagatca gaagtctctaaagggaagaggcagcactc	+	+
CY016679	Human H1N1 IAVs	Human	H1N1		2005	Australia	A/South_Australia/55/2005	ggcgatgccccccttccttgatcggcttcgccgagatca gaagtctctaaagggaagaggcagcactc	+	+
CY013561	Human H1N1 IAVs	Human	H1N1		2005	New_Zealand	A/Wellington/10/2005	ggcgatgccccccttccttgatcggcttcgccgagatca gaagtctctaaagggaagaggcagcactc	+	+
CY013593	Human H1N1 IAVs	Human	H1N1		2005	New_Zealand	A/Wellington/13/2005	ggcgatgccccccttccttgatcggcttcgccgagatca gaagtctctaaagggaagaggcagcactc	+	+
CY014011	Human H1N1 IAVs	Human	H1N1		2005	New_Zealand	A/Wellington/11/2005	ggcgatgccccccttccttgatcggcttcgccgagatca gaagtctctaaagggaagaggcagcactc	+	+
CY013569	Human H1N1 IAVs	Human	H1N1		2005	New_Zealand	A/Wellington/12/2005	ggcgatgccccccttccttgatcggcttcgccgagatca gaagtctctaaagggaagaggcagcactc	+	+
CY015584	Human H1N1 IAVs	Human	H1N1		2005	New_Zealand	A/Wellington/14/2005	ggcgatgccccccttccttgatcggcttcgccgagatca gaagtctctaaagggaagaggcagcactc	+	+
CY104826	Human H1N1 IAVs	Human	H1N1		2005	Viet_Nam	A/Hanoi/ISBM31/2005	ggcgatgccccccttccttgatcggcttcgccgagatca gaagtctctaaagggaagaggcagcactc	+	+
CY104248	Human H1N1 IAVs	Human	H1N1		2005	Viet_Nam	A/TayNguyen/TN371/2005	ggcgatgccccccttccttgatcggcttcgccgagatca gaagtctctaaagggaagaggcagcactc	+	+
CY105354	Human H1N1 IAVs	Human	H1N1		2005	Viet_Nam	A/TayNguyen/TN334/2005	ggcgatgccccccttccttgatcggcttcgccgagatca gaagtctctaaagggaagaggcagcactc	+	+
AB434116	Human H1N1 IAVs	Human	H1N1		2005	Japan	A/Morioka/3/2005	ggcgatgccccccttccttgatcggcttcgccgagatca gaagtctctaaagggaagaggcagcactc	+	+
AB434117	Human H1N1 IAVs	Human	H1N1		2005	Japan	A/Morioka/4/2005	ggcgatgccccccttccttgatcggcttcgccgagatca gaagtctctaaagggaagaggcagcactc	+	+
AB434120	Human H1N1 IAVs	Human	H1N1		2005	Japan	A/Morioka/5/2005	ggcgatgccccccttccttgatcggcttcgccgagatca gaagtctctaaagggaagaggcagcactc	+	+
AB434121	Human H1N1 IAVs	Human	H1N1		2005	Japan	A/Morioka/7/2005	ggcgatgccccccttccttgatcggcttcgccgagatca gaagtctctaaagggaagaggcagcactc	+	+

CY016703	Human H1N1 IAVs	Human	H1N1		2005	Australia	A/South_Australia/58/2005	ggcgatgcccccttccttgatcggcttcgccgagatca<br>gaagtctctaaagggaagaggcagcactc	+	+
HQ291864	Human H1N1 IAVs	Human	H1N1		2005	Taiwan	A/Taiwan/5515/2005	ggcgatgcccccttccttgatcggcttcgccgagatca<br>gaagtctctaaagggaagaggcagcactc	+	+
HQ291863	Human H1N1 IAVs	Human	H1N1		2005	Taiwan	A/Taiwan/5524/2005	ggcgatgcccccttccttgatcggcttcgccgagatca<br>gaagtctctaaagggaagaggcagcactc	+	+
AB434122	Human H1N1 IAVs	Human	H1N1		2005	Japan	A/Morioka/1/2005	ggcgatgcccccttccttgatcggcttcgccgagatca<br>gaagtctctaaagggaagaggcagcactc	+	+
AB434118	Human H1N1 IAVs	Human	H1N1		2005	Japan	A/Morioka/21/2005	ggcgatgcccccttccttgatcggcttcgccgagatca<br>gaagtctctaaagggaagaggcagcactc	+	+
CY119166	Human H1N1 IAVs	Human	H1N1		2005	Malaysia	A/Malaysia/32110/2005	ggcgatgcccccttccttgatcggcttcgccgagatca<br>gaagtctctaaagggaagaggcagcactc	+	+
HQ291845	Human H1N1 IAVs	Human	H1N1		2005	Taiwan	A/Taiwan/10393/2005	ggcgatgcccccttccttgatcggcttcgccgagatca<br>gaagtctctaaagggaagaggcagcactc	+	+
CY119174	Human H1N1 IAVs	Human	H1N1		2005	Malaysia	A/Malaysia/32217/2005	ggcgatgcccccttccttgatcggcttcgccgagatca<br>gaagtctctaaagggaagaggcagcactc	+	+
CY118000	Human H1N1 IAVs	Human	H1N1		2005	Malaysia	A/Malaysia/32151/2005	ggcgatgcccccttccttgatcggcttcgccgagatca<br>gaagtctctaaagggaagaggcagcactc	+	+
CY119182	Human H1N1 IAVs	Human	H1N1		2005	Malaysia	A/Malaysia/33132/2005	ggcgatgcccccttccttgatcggcttcgccgagatca<br>gaagtctctaaagggaagaggcagcactc	+	+
CY119190	Human H1N1 IAVs	Human	H1N1		2005	Malaysia	A/Malaysia/33166/2005	ggcgatgcccccttccttgatcggcttcgccgagatca<br>gaagtctctaaagggaagaggcagcactc	+	+
CY172563	Human H1N1 IAVs	Human	H1N1		2006	USA	A/New_York/1052/2006	ggcgatgcccccttcctagatcggcttcgccgagatca<br>gaagtctctaaagggaagaggcagcactc	+	+
CY172571	Human H1N1 IAVs	Human	H1N1		2006	USA	A/New_York/1053/2006	ggcgatgcccccttcctagatcggcttcgccgagatca<br>gaagtctctaaagggaagaggcagcactc	+	+
CY027151	Human H1N1 IAVs	Human	H1N1		2006	USA	A/North_Carolina/UR06_0011/2006	ggcgatgcccccttcctagatcggcttcgccgagatca<br>gaagtctctaaagggaagaggcagcactc	+	+
CY025225	Human H1N1 IAVs	Human	H1N1		2006	USA	A/Michigan/UR06_0015/2006	ggcgatgcccccttcctagatcggcttcgccgagatca<br>gaagtctctaaagggaagaggcagcactc	+	+
FJ912918	Human H1N1 IAVs	Human	H1N1		2006	Thailand	A/Thailand/CU44/2006	ggcgatgcccccttccttgatcggcttcgcagagatca<br>gaagtctctaaagggaagaggcagcactc	+	+
FJ912936	Human H1N1 IAVs	Human	H1N1		2006	Thailand	A/Thailand/CU68/2006	ggcgatgcccccttccttgatcggcttcgcagagatca<br>gaagtctctaaagggaagaggcagcactc	+	+
FJ445061	Human H1N1 IAVs	Human	H1N1		2006	United_Kingdom	A/England/494/2006	ggcgatgcccccttccttgatcggcttcgccgagatca<br>gaagtctctaaagggaagaggcagcactc	+	+
FJ445049	Human H1N1 IAVs	Human	H1N1		2006	United_Kingdom	A/England/593/2006	ggcgatgcccccttccttgatcggcttcgccgagatca<br>gaagtctctaaagggaagaggcagcactc	+	+
FJ445086	Human H1N1 IAVs	Human	H1N1		2006	United_Kingdom	A/England/594/2006	ggcgatgcccccttccttgatcggcttcgccgagatca<br>gaagtctctaaagggaagaggcagcactc	+	+
CY025217	Human H1N1 IAVs	Human	H1N1		2006	USA	A/Texas/UR06_0012/2006	ggcgatgcccccttccttgatcggcttcgccgagatca<br>gaagtctctaaagggaagaggcagcactc	+	+
EU247846	Human H1N1 IAVs	Human	H1N1		2006	France	A/Lyon/712/2006	ggcgatgcccccttccttgatcggcttcgccgagatca<br>gaagtctctaaagggaagaggcagcactc	+	+
CY172539	Human H1N1 IAVs	Human	H1N1		2006	USA	A/New_York/1049/2006	ggcgatgcccccttccttgatcggcttcgccgagatca<br>gaagtctctaaagggaagaggcagcactc	+	+
CY017375	Human H1N1 IAVs	Human	H1N1		2006	USA	A/New_York/8/2006	ggcgatgcccccttccttgatcggcttcgccgagatca<br>gaagtctctaaagggaagaggcagcactc	+	+
HQ291866	Human H1N1 IAVs	Human	H1N1		2006	Taiwan	A/Taiwan/6566/2006	ggcgatgcccccttccttgatcggcttcgccgagatca<br>gaagtctctaaagggaagaggcagcactc	+	+

CY035130	Human H1N1 IAVs	Human	H1N1		2006	Russia	A/St._Petersburg/8/2006	ggcgatgcccccttccttgatcggcttcgccgagatca<br>gaagtctctaaagggaagaggcagcactc	+	+
CY104842	Human H1N1 IAVs	Human	H1N1		2006	Viet_Nam	A/HaNoi/Q421/2006	ggcgatgcccccttccttgatcggcttcgccgagatca<br>gaagtctctaaagggaagaggcagcactc	+	+
CY104850	Human H1N1 IAVs	Human	H1N1		2006	Viet_Nam	A/HaNoi/Q555/2006	ggcgatgcccccttccttgatcggcttcgccgagatca<br>gaagtctctaaagggaagaggcagcactc	+	+
CY105178	Human H1N1 IAVs	Human	H1N1		2006	Viet_Nam	A/HoChiMinh/HCM639/2006	ggcgatgcccccttccttgatcggcttcgccgagatca<br>gaagtctctaaagggaagaggcagcactc	+	+
CY104674	Human H1N1 IAVs	Human	H1N1		2006	Viet_Nam	A/LongAn/HCM665/2006	ggcgatgcccccttccttgatcggcttcgccgagatca<br>gaagtctctaaagggaagaggcagcactc	+	+
CY104312	Human H1N1 IAVs	Human	H1N1		2006	Viet_Nam	A/TayNguyen/TN161/2006	ggcgatgcccccttccttgatcggcttcgccgagatca<br>gaagtctctaaagggaagaggcagcactc	+	+
CY105170	Human H1N1 IAVs	Human	H1N1		2006	Viet_Nam	A/HoChiMinh/HCM526/2006	ggcgatgcccccttccttgatcggcttcgccgagatca<br>gaagtctctaaagggaagaggcagcactc	+	+
CY104666	Human H1N1 IAVs	Human	H1N1		2006	Viet_Nam	A/HoChiMinh/HCM554/2006	ggcgatgcccccttccttgatcggcttcgccgagatca<br>gaagtctctaaagggaagaggcagcactc	+	+
CY104658	Human H1N1 IAVs	Human	H1N1		2006	Viet_Nam	A/LongAn/HCM504/2006	ggcgatgcccccttccttgatcggcttcgccgagatca<br>gaagtctctaaagggaagaggcagcactc	+	+
CY104858	Human H1N1 IAVs	Human	H1N1		2006	Viet_Nam	A/TayNguyen/TN178/2006	ggcgatgcccccttccttgatcggcttcgccgagatca<br>gaagtctctaaagggaagaggcagcactc	+	+
CY104866	Human H1N1 IAVs	Human	H1N1		2006	Viet_Nam	A/TayNguyen/TN182/2006	ggcgatgcccccttccttgatcggcttcgccgagatca<br>gaagtctctaaagggaagaggcagcactc	+	+
CY104288	Human H1N1 IAVs	Human	H1N1		2006	Viet_Nam	A/HaNoi/Q580/2006	ggcgatgcccccttccttgatcggcttcgccgagatca<br>gaagtctctaaagggaagaggcagcactc	+	+
CY104296	Human H1N1 IAVs	Human	H1N1		2006	Viet_Nam	A/HaNoi/Q591/2006	ggcgatgcccccttccttgatcggcttcgccgagatca<br>gaagtctctaaagggaagaggcagcactc	+	+
CY118024	Human H1N1 IAVs	Human	H1N1		2006	Malaysia	A/Malaysia/1652509/2006	ggcgatgcccccttccttgatcggcttcgccgagatca<br>gaagtctctaaagggaagaggcagcactc	+	+
CY119214	Human H1N1 IAVs	Human	H1N1		2006	Malaysia	A/Malaysia/35405/2006	ggcgatgcccccttccttgatcggcttcgccgagatca<br>gaagtctctaaagggaagaggcagcactc	+	+
HQ291848	Human H1N1 IAVs	Human	H1N1		2006	Taiwan	A/Taiwan/0586/2006	ggcgatgcccccttccttgatcggcttcgccgagatca<br>gaagtctctaaagggaagaggcagcactc	+	+
HQ291869	Human H1N1 IAVs	Human	H1N1		2006	Taiwan	A/Taiwan/192/2006	ggcgatgcccccttccttgatcggcttcgccgagatca<br>gaagtctctaaagggaagaggcagcactc	+	+
HQ291860	Human H1N1 IAVs	Human	H1N1		2006	Taiwan	A/Taiwan/2900/2006	ggcgatgcccccttccttgatcggcttcgccgagatca<br>gaagtctctaaagggaagaggcagcactc	+	+
CY038883	Human H1N1 IAVs	Human	H1N1		2006	Taiwan	A/Taiwan/2645/2006	ggcgatgcccccttccttgatcggcttcgccgagatca<br>gaagtctctaaagggaagaggcagcactc	+	+
FJ912942	Human H1N1 IAVs	Human	H1N1		2006	Thailand	A/Thailand/CU88/2006	ggcgatgcccccttccttgatcggcttcgccgagatca<br>gaagtctctaaagggaagaggcagcactc	+	+
FJ912930	Human H1N1 IAVs	Human	H1N1		2006	Thailand	A/Thailand/CU51/2006	ggcgatgcccccttccttgatcggcttcgccgagatca<br>gaagtctctaaagggaagaggcagcactc	+	+
HQ291861	Human H1N1 IAVs	Human	H1N1		2006	Taiwan	A/Taiwan/5505/2006	ggcgatgcccccttccttgatcggcttcgccgagatca<br>gaagtctctaaagggaagaggcagcactc	+	+
CY118016	Human H1N1 IAVs	Human	H1N1		2006	Malaysia	A/Malaysia/34291/2006	ggcgatgcccccttccttgatcggcttcgccgagatca<br>gaagtctctaaagggaagaggcagcactc	+	+
CY119198	Human H1N1 IAVs	Human	H1N1		2006	Malaysia	A/Malaysia/34450/2006	ggcgatgcccccttccttgatcggcttcgccgagatca<br>gaagtctctaaagggaagaggcagcactc	+	+
HQ291849	Human H1N1 IAVs	Human	H1N1		2006	Taiwan	A/Taiwan/0045/2006	ggcgatgcccccttccttgatcggcttcgccgagatca<br>gaagtctctaaagggaagaggcagcactc	+	+

CY119206	Human H1N1 IAVs	Human	H1N1		2006	Malaysia	A/Malaysia/35164/2006	ggcgatgccccttccttgatcggcttcgccgagatca gaagtctctaaagggaagaggcagcactc	+	+
CY119222	Human H1N1 IAVs	Human	H1N1		2006	Malaysia	A/Malaysia/1686034/2006	ggcgatgccccttccttgatcggcttcgccgagatca gaagtctctaaagggaagaggcagcactc	+	+
FJ445039	Human H1N1 IAVs	Human	H1N1		2006	United_Kingdom	A/England/493/2006	ggcgatgccccttccttgatcggcttcgccgagatca gaagtctctaaagggaagaggcagcactc	+	+
CY104280	Human H1N1 IAVs	Human	H1N1		2006	Viet_Nam	A/Hanoi/Q137/2006	ggcgatgccccttccttgatcggcttcgccgagatca gaagtctctaaagggaagaggcagcactc	+	+
CY104834	Human H1N1 IAVs	Human	H1N1		2006	Viet_Nam	A/Hanoi/Q177/2006	ggcgatgccccttccttgatcggcttcgccgagatca gaagtctctaaagggaagaggcagcactc	+	+
FJ912912	Human H1N1 IAVs	Human	H1N1		2006	Thailand	A/Thailand/CU32/2006	ggcgatgccccttccttgatcggcttcgccgagatca gaagtctctaaagggaagaggcagcactc	+	+
CY105162	Human H1N1 IAVs	Human	H1N1		2006	Viet_Nam	A/HoChiMinh/HCM395/2006	ggcgatgccccttccttgatcggcttcgccgagatca gaagtctctaaagggaagaggcagcactc	+	+
EU247847	Human H1N1 IAVs	Human	H1N1		2006	France	A/Lyon/813/2006	ggcgatgccccttccttgatcggcttcgccgagatca gaagtctctaaagggacgaggcagcactc	+	+
CY025233	Human H1N1 IAVs	Human	H1N1		2006	USA	A/Kentucky/UR06_0010/2006	ggcgatgccccttccttgatcggcttcgccgggatca gaagtctctaaagggaagaggcagcactc	+	+
CY028199	Human H1N1 IAVs	Human	H1N1		2006	USA	A/Kentucky/UR06_0007/2006	ggcgatgccccttccttgatcggcttcgccgggatca gaagtctctaaagggaagaggcagcactc	+	+
CY172547	Human H1N1 IAVs	Human	H1N1		2006	USA	A/New_York/1050/2006	ggcgatgccccttccttgatcggcttcgccgggatca gaagtctctaaagggaagaggcagcactc	+	+
CY172555	Human H1N1 IAVs	Human	H1N1		2006	USA	A/New_York/1051/2006	ggcgatgccccttccttgatcggcttcgccgggatca gaagtctctaaagggaagaggcagcactc	+	+
CY027879	Human H1N1 IAVs	Human	H1N1		2006	USA	A/Mississippi/UR06_0014/2006	ggcgatgccccttccttgatcggcttcgccgggatca gaagtctctaaagggaagaggcagcactc	+	+
CY104304	Human H1N1 IAVs	Human	H1N1		2006	Viet_Nam	A/TayNguyen/TN158/2006	ggcgatgccccttccttgatcgkcttcgccgagatca gaagtctctaaagggaagaggcagcactc	+	+
CY034128	Human H1N1 IAVs	Human	H1N1		2006	NA	A/Nymc_X_163_A/Nymc_X_157_A /St.Petersburg/8/2006__RX	ggtgatgccccattccttgatcggcttcgccgagatcag aaatccctaagaggaaggggcagcaccc	-	*
EU004448	Human H1N1 IAVs	Swine	H1N1		2006	China	A/swine/Henan/01/2006	ggtgatgccccattccttgatcggcttcgccgagatcag aagtccctaaagggaagaggcagcactc	+	+
HQ853498	Human H1N1 IAVs	Human	H1N1		2007	India	A/KOL/565/2007	ggcgataccccttccttgatcggcttcgccgagatca gaagtctctaaagggaagaggcagcactc	+	+
CY027767	Human H1N1 IAVs	Human	H1N1		2007	USA	A/Ohio/UR06_0112/2007	ggcgatgccccttcctagatcggcttcgccgagatca gaagtctataaagggaagaggcagcactc	+	+
CY027847	Human H1N1 IAVs	Human	H1N1		2007	USA	A/California/UR06_0125/2007	ggcgatgccccttcctagatcggcttcgccgagatca gaagtctctaaagggaagaggcagcactc	+	+
CY172579	Human H1N1 IAVs	Human	H1N1		2007	USA	A/New_York/1054/2007	ggcgatgccccttcctagatcggcttcgccgagatca gaagtctctaaagggaagaggcagcactc	+	+
CY028776	Human H1N1 IAVs	Human	H1N1		2007	USA	A/Virginia/UR06_0361/2007	ggcgatgccccttcctagatcggcttcgccgagatca gaagtctctaaagggaagaggcagcactc	+	+
CY025377	Human H1N1 IAVs	Human	H1N1		2007	USA	A/California/UR06_0435/2007	ggcgatgccccttcctagatcggcttcgccgagatca gaagtctctaaagggaagaggcagcactc	+	+
CY026223	Human H1N1 IAVs	Human	H1N1		2007	USA	A/Kentucky/UR06_0028/2007	ggcgatgccccttcctagatcggcttcgccgagatca gaagtctctaaagggaagaggcagcactc	+	+
CY026647	Human H1N1 IAVs	Human	H1N1		2007	USA	A/New_York/UR06_0253/2007	ggcgatgccccttcctagatcggcttcgccgagatca gaagtctctaaagggaagaggcagcactc	+	+
CY025289	Human H1N1 IAVs	Human	H1N1		2007	USA	A/New_York/UR06_0386/2007	ggcgatgccccttcctagatcggcttcgccgagatca gaagtctctaaagggaagaggcagcactc	+	+

CY027423	Human H1N1 IAVs	Human	H1N1		2007	USA	A/Tennessee/UR06_0262/2007	ggcgatgcccccttcctagatcggcttcgccgagatca gaagtctctaaagggaagaggcagcactc	+	+
CY027943	Human H1N1 IAVs	Human	H1N1		2007	USA	A/Virginia/UR06_0295/2007	ggcgatgcccccttcctagatcggcttcgccgagatca gaagtctctaaagggaagaggcagcactc	+	+
CY027359	Human H1N1 IAVs	Human	H1N1		2007	USA	A/California/UR06_0564/2007	ggcgatgcccccttcctagatcggcttcgccgagatca gaagtctctaaagggaagaggcagcactc	+	+
CY027231	Human H1N1 IAVs	Human	H1N1		2007	USA	A/Colorado/UR06_0499/2007	ggcgatgcccccttcctagatcggcttcgccgagatca gaagtctctaaagggaagaggcagcactc	+	+
CY026983	Human H1N1 IAVs	Human	H1N1		2007	USA	A/Kentucky/UR06_0161/2007	ggcgatgcccccttcctagatcggcttcgccgagatca gaagtctctaaagggaagaggcagcactc	+	+
CY028151	Human H1N1 IAVs	Human	H1N1		2007	USA	A/Kentucky/UR06_0182/2007	ggcgatgcccccttcctagatcggcttcgccgagatca gaagtctctaaagggaagaggcagcactc	+	+
CY027375	Human H1N1 IAVs	Human	H1N1		2007	USA	A/Kentucky/UR06_0424/2007	ggcgatgcccccttcctagatcggcttcgccgagatca gaagtctctaaagggaagaggcagcactc	+	+
CY027679	Human H1N1 IAVs	Human	H1N1		2007	USA	A/Kentucky/UR06_0449/2007	ggcgatgcccccttcctagatcggcttcgccgagatca gaagtctctaaagggaagaggcagcactc	+	+
CY025943	Human H1N1 IAVs	Human	H1N1		2007	USA	A/Kentucky/UR06_0539/2007	ggcgatgcccccttcctagatcggcttcgccgagatca gaagtctctaaagggaagaggcagcactc	+	+
HQ166048	Human H1N1 IAVs	Human	H1N1		2007	Netherlands	A/Netherlands/26/2007	ggcgatgcccccttcctagatcggcttcgccgagatca gaagtctctaaagggaagaggcagcactc	+	+
CY172595	Human H1N1 IAVs	Human	H1N1		2007	USA	A/New_York/1056/2007	ggcgatgcccccttcctagatcggcttcgccgagatca gaagtctctaaagggaagaggcagcactc	+	+
CY172627	Human H1N1 IAVs	Human	H1N1		2007	USA	A/New_York/1061/2007	ggcgatgcccccttcctagatcggcttcgccgagatca gaagtctctaaagggaagaggcagcactc	+	+
CY027143	Human H1N1 IAVs	Human	H1N1		2007	USA	A/Ohio/UR06_0177/2007	ggcgatgcccccttcctagatcggcttcgccgagatca gaagtctctaaagggaagaggcagcactc	+	+
CY025671	Human H1N1 IAVs	Human	H1N1		2007	USA	A/Kentucky/UR06_0553/2007	ggcgatgcccccttcctagatcggcttcgccgagatca gaagtctctaaagggaagaggcagcactc	+	+
CY027247	Human H1N1 IAVs	Human	H1N1		2007	USA	A/Tennessee/UR06_0124/2007	ggcgatgcccccttcctagatcggcttcgccgagatca gaagtctctaaagggaagaggcagcactc	+	+
CY172707	Human H1N1 IAVs	Human	H1N1		2007	USA	A/New_York/1073/2007	ggcgatgcccccttcctagatcggcttcgccgagatca gaagtctctaaagggaagaggcagcactc	+	+
CY025497	Human H1N1 IAVs	Human	H1N1		2007	USA	A/Kentucky/UR06_0042/2007	ggcgatgcccccttcctagatcggcttcgccgagatca gaagtctctaaagggaagaggcagcactc	+	+
CY026535	Human H1N1 IAVs	Human	H1N1		2007	USA	A/California/UR06_0302/2007	ggcgatgcccccttcctagatcggcttcgccgagatca gaagtctctaaagggaagaggcagcactc	+	+
CY026231	Human H1N1 IAVs	Human	H1N1		2007	USA	A/Kentucky/UR06_0538/2007	ggcgatgcccccttcctagatcggcttcgccgagatca gaagtctctaaagggaagaggcagcactc	+	+
CY027215	Human H1N1 IAVs	Human	H1N1		2007	USA	A/California/UR06_0585/2007	ggcgatgcccccttcctagatcggcttcgccgagatca gaagtctctaaagggaagaggcagcactc	+	+
CY025441	Human H1N1 IAVs	Human	H1N1		2007	USA	A/Illinois/UR06_0333/2007	ggcgatgcccccttcctagatcggcttcgccgagatca gaagtctctaaagggaagaggcagcactc	+	+
CY025599	Human H1N1 IAVs	Human	H1N1		2007	USA	A/Illinois/UR06_0415/2007	ggcgatgcccccttcctagatcggcttcgccgagatca gaagtctctaaagggaagaggcagcactc	+	+
CY026799	Human H1N1 IAVs	Human	H1N1		2007	USA	A/Illinois/UR06_0491/2007	ggcgatgcccccttcctagatcggcttcgccgagatca gaagtctctaaagggaagaggcagcactc	+	+
CY027239	Human H1N1 IAVs	Human	H1N1		2007	USA	A/Virginia/UR06_0117/2007	ggcgatgcccccttcctagatcggcttcgccgagatca gaagtctctaaagggaagaggcagcactc	+	+
CY027711	Human H1N1 IAVs	Human	H1N1		2007	USA	A/Virginia/UR06_0549/2007	ggcgatgcccccttcctagatcggcttcgccgagatca gaagtctctaaagggaagaggcagcactc	+	+

CY026863	Human H1N1 IAVs	Human	H1N1		2007	USA	A/North_Carolina/UR06_0099/2007	ggcgatgccccttcctagatcggcttcgccgagatca gaagtctctaaagggaagaggcagcactc	+	+
CY028407	Human H1N1 IAVs	Human	H1N1		2007	USA	A/Ohio/UR06_0429/2007	ggcgatgccccttcctagatcggcttcgccgagatca gaagtctctaaagggaagaggcagcactc	+	+
CY026911	Human H1N1 IAVs	Human	H1N1		2007	USA	A/Virginia/UR06_0254/2007	ggcgatgccccttcctagatcggcttcgccgagatca gaagtctctaaagggaagaggcagcactc	+	+
CY026359	Human H1N1 IAVs	Human	H1N1		2007	USA	A/California/UR06_0232/2007	ggcgatgccccttcctagatcggcttcgccgagatca gaagtctctaaagggaagaggcagcactc	+	+
CY027823	Human H1N1 IAVs	Human	H1N1		2007	USA	A/Illinois/UR06_0074/2007	ggcgatgccccttcctagatcggcttcgccgagatca gaagtctctaaagggaagaggcagcactc	+	+
CY027439	Human H1N1 IAVs	Human	H1N1		2007	USA	A/Illinois/UR06_0094/2007	ggcgatgccccttcctagatcggcttcgccgagatca gaagtctctaaagggaagaggcagcactc	+	+
CY028071	Human H1N1 IAVs	Human	H1N1		2007	USA	A/Illinois/UR06_0093/2007	ggcgatgccccttcctagatcggcttcgccgagatca gaagtctctaaagggaagaggcagcactc	+	+
CY026975	Human H1N1 IAVs	Human	H1N1		2007	USA	A/Illinois/UR06_0095/2007	ggcgatgccccttcctagatcggcttcgccgagatca gaagtctctaaagggaagaggcagcactc	+	+
CY027703	Human H1N1 IAVs	Human	H1N1		2007	USA	A/Illinois/UR06_0131/2007	ggcgatgccccttcctagatcggcttcgccgagatca gaagtctctaaagggaagaggcagcactc	+	+
CY027095	Human H1N1 IAVs	Human	H1N1		2007	USA	A/Illinois/UR06_0137/2007	ggcgatgccccttcctagatcggcttcgccgagatca gaagtctctaaagggaagaggcagcactc	+	+
CY027487	Human H1N1 IAVs	Human	H1N1		2007	USA	A/Illinois/UR06_0115/2007	ggcgatgccccttcctagatcggcttcgccgagatca gaagtctctaaagggaagaggcagcactc	+	+
CY027399	Human H1N1 IAVs	Human	H1N1		2007	USA	A/Illinois/UR06_0136/2007	ggcgatgccccttcctagatcggcttcgccgagatca gaagtctctaaagggaagaggcagcactc	+	+
CY028423	Human H1N1 IAVs	Human	H1N1		2007	USA	A/Illinois/UR06_0215/2007	ggcgatgccccttcctagatcggcttcgccgagatca gaagtctctaaagggaagaggcagcactc	+	+
CY027279	Human H1N1 IAVs	Human	H1N1		2007	USA	A/Illinois/UR06_0116/2007	ggcgatgccccttcctagatcggcttcgccgagatca gaagtctctaaagggaagaggcagcactc	+	+
CY025591	Human H1N1 IAVs	Human	H1N1		2007	USA	A/Texas/UR06_0526/2007	ggcgatgccccttcctagatcggcttcgccgagatca gaagtctctaaagggaagaggcagcactc	+	+
CY041454	Human H1N1 IAVs	Human	H1N1		2007	USA	A/California/UR06_0439/2007	ggcgatgccccttcctagatcggcttcgccgagatca gaagtctctaaagggaagaggcagcactc	+	+
CY027471	Human H1N1 IAVs	Human	H1N1		2007	USA	A/Ohio/UR06_0394/2007	ggcgatgccccttcctagatcggcttcgccgagatca gaagtctctaaagggaagaggcagcactc	+	+
CY028143	Human H1N1 IAVs	Human	H1N1		2007	USA	A/Kansas/UR06_0191/2007	ggcgatgccccttcctagatcggcttcgccgagatca gaagtctctaaagggaagaggcagcactc	+	+
CY028463	Human H1N1 IAVs	Human	H1N1		2007	USA	A/California/UR06_0442/2007	ggcgatgccccttcctagatcggcttcgccgagatca gaagtctctaaagggaagaggcagcactc	+	+
CY172603	Human H1N1 IAVs	Human	H1N1		2007	USA	A/New_York/1058/2007	ggcgatgccccttcctagatcggcttcgccgagatca gaagtctctaaagggaagaggcagcactc	+	+
CY172619	Human H1N1 IAVs	Human	H1N1		2007	USA	A/New_York/1060/2007	ggcgatgccccttcctagatcggcttcgccgagatca gaagtctctaaagggaagaggcagcactc	+	+
CY026591	Human H1N1 IAVs	Human	H1N1		2007	USA	A/Kentucky/UR06_0476/2007	ggcgatgccccttccttgatcggcttcgccgagacca gaagtctctaaagggaagaggcagcactc	+	+
KF836393	Human H1N1 IAVs	Human	H1N1		2007	Singapore	A/Singapore/23J/2007	ggcgatgccccttccttgatcggcttcgccgagacca gaagtctctaaagggaagaggcagcactc	+	+
CY027775	Human H1N1 IAVs	Human	H1N1		2007	USA	A/Oklahoma/UR06_0063/2007	ggcgatgccccttccttgatcggcttcgccgagatca gaagtctctaaagagaagaggcagcactc	+	+
KF836397	Human H1N1 IAVs	Human	H1N1		2007	Singapore	A/Singapore/49O/2007	ggcgatgccccttccttgatcggcttcgccgagatca gaagtctctaaagggaagaggcaacactc	+	+

CY025679	Human H1N1 IAVs	Human	H1N1		2007	USA	A/Kentucky/UR06_0240/2007	ggcgatgcccccttccttgatcggcttcgccgagatca<br>gaagtctctaaagggaagaggcagcactc	+	+
CY172659	Human H1N1 IAVs	Human	H1N1		2007	USA	A/New_York/1066/2007	ggcgatgcccccttccttgatcggcttcgccgagatca<br>gaagtctctaaagggaagaggcagcactc	+	+
CY026687	Human H1N1 IAVs	Human	H1N1		2007	USA	A/Vermont/UR06_0035/2007	ggcgatgcccccttccttgatcggcttcgccgagatca<br>gaagtctctaaagggaagaggcagcactc	+	+
CY096855	Human H1N1 IAVs	Human	H1N1		2007	USA	A/Colorado/UR06_0495/2007	ggcgatgcccccttccttgatcggcttcgccgagatca<br>gaagtctctaaagggaagaggcagcactc	+	+
CY026519	Human H1N1 IAVs	Human	H1N1		2007	USA	A/Kentucky/UR06_0057/2007	ggcgatgcccccttccttgatcggcttcgccgagatca<br>gaagtctctaaagggaagaggcagcactc	+	+
CY025265	Human H1N1 IAVs	Human	H1N1		2007	USA	A/Kentucky/UR06_0059/2007	ggcgatgcccccttccttgatcggcttcgccgagatca<br>gaagtctctaaagggaagaggcagcactc	+	+
CY027663	Human H1N1 IAVs	Human	H1N1		2007	USA	A/Texas/UR06_0133/2007	ggcgatgcccccttccttgatcggcttcgccgagatca<br>gaagtctctaaagggaagaggcagcactc	+	+
CY025959	Human H1N1 IAVs	Human	H1N1		2007	USA	A/Texas/UR06_0174/2007	ggcgatgcccccttccttgatcggcttcgccgagatca<br>gaagtctctaaagggaagaggcagcactc	+	+
CY026215	Human H1N1 IAVs	Human	H1N1		2007	USA	A/Texas/UR06_0195/2007	ggcgatgcccccttccttgatcggcttcgccgagatca<br>gaagtctctaaagggaagaggcagcactc	+	+
CY026407	Human H1N1 IAVs	Human	H1N1		2007	USA	A/Texas/UR06_0270/2007	ggcgatgcccccttccttgatcggcttcgccgagatca<br>gaagtctctaaagggaagaggcagcactc	+	+
CY027327	Human H1N1 IAVs	Human	H1N1		2007	USA	A/Texas/UR06_0342/2007	ggcgatgcccccttccttgatcggcttcgccgagatca<br>gaagtctctaaagggaagaggcagcactc	+	+
CY025393	Human H1N1 IAVs	Human	H1N1		2007	USA	A/Texas/UR06_0398/2007	ggcgatgcccccttccttgatcggcttcgccgagatca<br>gaagtctctaaagggaagaggcagcactc	+	+
CY026271	Human H1N1 IAVs	Human	H1N1		2007	USA	A/Texas/UR06_0306/2007	ggcgatgcccccttccttgatcggcttcgccgagatca<br>gaagtctctaaagggaagaggcagcactc	+	+
CY025513	Human H1N1 IAVs	Human	H1N1		2007	USA	A/Kentucky/UR06_0097/2007	ggcgatgcccccttccttgatcggcttcgccgagatca<br>gaagtctctaaagggaagaggcagcactc	+	+
CY025361	Human H1N1 IAVs	Human	H1N1		2007	USA	A/Kentucky/UR06_0363/2007	ggcgatgcccccttccttgatcggcttcgccgagatca<br>gaagtctctaaagggaagaggcagcactc	+	+
CY026191	Human H1N1 IAVs	Human	H1N1		2007	USA	A/Texas/UR06_0303/2007	ggcgatgcccccttccttgatcggcttcgccgagatca<br>gaagtctctaaagggaagaggcagcactc	+	+
CY163780	Human H1N1 IAVs	Human	H1N1		2007	Australia	A/Brisbane/59/2007	ggcgatgcccccttccttgatcggcttcgccgagatca<br>gaagtctctaaagggaagaggcagcactc	+	+
CY163860	Human H1N1 IAVs	Human	H1N1		2007	Australia	A/Brisbane/59/2007	ggcgatgcccccttccttgatcggcttcgccgagatca<br>gaagtctctaaagggaagaggcagcactc	+	+
CY163764	Human H1N1 IAVs	Human	H1N1		2007	Australia	A/Brisbane/59/2007	ggcgatgcccccttccttgatcggcttcgccgagatca<br>gaagtctctaaagggaagaggcagcactc	+	+
CY163796	Human H1N1 IAVs	Human	H1N1		2007	Australia	A/Brisbane/59/2007	ggcgatgcccccttccttgatcggcttcgccgagatca<br>gaagtctctaaagggaagaggcagcactc	+	+
CY163868	Human H1N1 IAVs	Human	H1N1		2007	Australia	A/Brisbane/59/2007	ggcgatgcccccttccttgatcggcttcgccgagatca<br>gaagtctctaaagggaagaggcagcactc	+	+
CY163548	Human H1N1 IAVs	Human	H1N1		2007	Australia	A/Brisbane/59/2007	ggcgatgcccccttccttgatcggcttcgccgagatca<br>gaagtctctaaagggaagaggcagcactc	+	+
CY163636	Human H1N1 IAVs	Human	H1N1		2007	Australia	A/Brisbane/59/2007	ggcgatgcccccttccttgatcggcttcgccgagatca<br>gaagtctctaaagggaagaggcagcactc	+	+
CY163820	Human H1N1 IAVs	Human	H1N1		2007	Australia	A/Brisbane/59/2007	ggcgatgcccccttccttgatcggcttcgccgagatca<br>gaagtctctaaagggaagaggcagcactc	+	+
CY163516	Human H1N1 IAVs	Human	H1N1		2007	Australia	A/Brisbane/59/2007	ggcgatgcccccttccttgatcggcttcgccgagatca<br>gaagtctctaaagggaagaggcagcactc	+	+

CY163540	Human H1N1 IAVs	Human	H1N1		2007	Australia	A/Brisbane/59/2007	ggcgatgcccccttccttgatcggcttcgccgagatca gaagtctctaaagggaagaggcagcactc	+	+
CY163572	Human H1N1 IAVs	Human	H1N1		2007	Australia	A/Brisbane/59/2007	ggcgatgcccccttccttgatcggcttcgccgagatca gaagtctctaaagggaagaggcagcactc	+	+
CY163564	Human H1N1 IAVs	Human	H1N1		2007	Australia	A/Brisbane/59/2007	ggcgatgcccccttccttgatcggcttcgccgagatca gaagtctctaaagggaagaggcagcactc	+	+
CY163772	Human H1N1 IAVs	Human	H1N1		2007	Australia	A/Brisbane/59/2007	ggcgatgcccccttccttgatcggcttcgccgagatca gaagtctctaaagggaagaggcagcactc	+	+
CY163532	Human H1N1 IAVs	Human	H1N1		2007	Australia	A/Brisbane/59/2007	ggcgatgcccccttccttgatcggcttcgccgagatca gaagtctctaaagggaagaggcagcactc	+	+
CY163852	Human H1N1 IAVs	Human	H1N1		2007	Australia	A/Brisbane/59/2007	ggcgatgcccccttccttgatcggcttcgccgagatca gaagtctctaaagggaagaggcagcactc	+	+
CY163556	Human H1N1 IAVs	Human	H1N1		2007	Australia	A/Brisbane/59/2007	ggcgatgcccccttccttgatcggcttcgccgagatca gaagtctctaaagggaagaggcagcactc	+	+
CY163596	Human H1N1 IAVs	Human	H1N1		2007	Australia	A/Brisbane/59/2007	ggcgatgcccccttccttgatcggcttcgccgagatca gaagtctctaaagggaagaggcagcactc	+	+
CY163604	Human H1N1 IAVs	Human	H1N1		2007	Australia	A/Brisbane/59/2007	ggcgatgcccccttccttgatcggcttcgccgagatca gaagtctctaaagggaagaggcagcactc	+	+
CY163756	Human H1N1 IAVs	Human	H1N1		2007	Australia	A/Brisbane/59/2007	ggcgatgcccccttccttgatcggcttcgccgagatca gaagtctctaaagggaagaggcagcactc	+	+
CY163836	Human H1N1 IAVs	Human	H1N1		2007	Australia	A/Brisbane/59/2007	ggcgatgcccccttccttgatcggcttcgccgagatca gaagtctctaaagggaagaggcagcactc	+	+
CY163612	Human H1N1 IAVs	Human	H1N1		2007	Australia	A/Brisbane/59/2007	ggcgatgcccccttccttgatcggcttcgccgagatca gaagtctctaaagggaagaggcagcactc	+	+
CY163828	Human H1N1 IAVs	Human	H1N1		2007	Australia	A/Brisbane/59/2007	ggcgatgcccccttccttgatcggcttcgccgagatca gaagtctctaaagggaagaggcagcactc	+	+
CY163644	Human H1N1 IAVs	Human	H1N1		2007	Australia	A/Brisbane/59/2007	ggcgatgcccccttccttgatcggcttcgccgagatca gaagtctctaaagggaagaggcagcactc	+	+
CY163676	Human H1N1 IAVs	Human	H1N1		2007	Australia	A/Brisbane/59/2007	ggcgatgcccccttccttgatcggcttcgccgagatca gaagtctctaaagggaagaggcagcactc	+	+
CY163588	Human H1N1 IAVs	Human	H1N1		2007	Australia	A/Brisbane/59/2007	ggcgatgcccccttccttgatcggcttcgccgagatca gaagtctctaaagggaagaggcagcactc	+	+
CY163628	Human H1N1 IAVs	Human	H1N1		2007	Australia	A/Brisbane/59/2007	ggcgatgcccccttccttgatcggcttcgccgagatca gaagtctctaaagggaagaggcagcactc	+	+
CY163812	Human H1N1 IAVs	Human	H1N1		2007	Australia	A/Brisbane/59/2007	ggcgatgcccccttccttgatcggcttcgccgagatca gaagtctctaaagggaagaggcagcactc	+	+
CY163844	Human H1N1 IAVs	Human	H1N1		2007	Australia	A/Brisbane/59/2007	ggcgatgcccccttccttgatcggcttcgccgagatca gaagtctctaaagggaagaggcagcactc	+	+
CY163804	Human H1N1 IAVs	Human	H1N1		2007	Australia	A/Brisbane/59/2007	ggcgatgcccccttccttgatcggcttcgccgagatca gaagtctctaaagggaagaggcagcactc	+	+
CY163788	Human H1N1 IAVs	Human	H1N1		2007	Australia	A/Brisbane/59/2007	ggcgatgcccccttccttgatcggcttcgccgagatca gaagtctctaaagggaagaggcagcactc	+	+
CY163620	Human H1N1 IAVs	Human	H1N1		2007	Australia	A/Brisbane/59/2007	ggcgatgcccccttccttgatcggcttcgccgagatca gaagtctctaaagggaagaggcagcactc	+	+
CY163580	Human H1N1 IAVs	Human	H1N1		2007	Australia	A/Brisbane/59/2007	ggcgatgcccccttccttgatcggcttcgccgagatca gaagtctctaaagggaagaggcagcactc	+	+
CY163524	Human H1N1 IAVs	Human	H1N1		2007	Australia	A/Brisbane/59/2007	ggcgatgcccccttccttgatcggcttcgccgagatca gaagtctctaaagggaagaggcagcactc	+	+
CY058491	Human H1N1 IAVs	Human	H1N1		2007	Australia	A/Brisbane/59/2007	ggcgatgcccccttccttgatcggcttcgccgagatca gaagtctctaaagggaagaggcagcactc	+	+

CY026543	Human H1N1 IAVs	Human	H1N1		2007	USA	A/California/UR06_0393/2007	ggcgatgcccccttccttgatcggcttcgccgagatca gaagtctctaaagggaagaggcagcactc	+	+
CY028311	Human H1N1 IAVs	Human	H1N1		2007	USA	A/California/UR06_0479/2007	ggcgatgcccccttccttgatcggcttcgccgagatca gaagtctctaaagggaagaggcagcactc	+	+
CY026375	Human H1N1 IAVs	Human	H1N1		2007	USA	A/Mississippi/UR06_0378/2007	ggcgatgcccccttccttgatcggcttcgccgagatca gaagtctctaaagggaagaggcagcactc	+	+
CY026703	Human H1N1 IAVs	Human	H1N1		2007	USA	A/Tennessee/UR06_0045/2007	ggcgatgcccccttccttgatcggcttcgccgagatca gaagtctctaaagggaagaggcagcactc	+	+
CY028207	Human H1N1 IAVs	Human	H1N1		2007	USA	A/Tennessee/UR06_0055/2007	ggcgatgcccccttccttgatcggcttcgccgagatca gaagtctctaaagggaagaggcagcactc	+	+
CY028319	Human H1N1 IAVs	Human	H1N1		2007	USA	A/Texas/UR06_0217/2007	ggcgatgcccccttccttgatcggcttcgccgagatca gaagtctctaaagggaagaggcagcactc	+	+
HQ853501	Human H1N1 IAVs	Human	H1N1		2007	India	A/KOL/611/2007	ggcgatgcccccttccttgatcggcttcgccgagatca gaagtctctaaagggaagaggcagcactc	+	+
CY040062	Human H1N1 IAVs	Human	H1N1		2007	Taiwan	A/Taiwan/71720/2007	ggcgatgcccccttccttgatcggcttcgccgagatca gaagtctctaaagggaagaggcagcactc	+	+
HQ853495	Human H1N1 IAVs	Human	H1N1		2007	India	A/KOL/507/2007	ggcgatgcccccttccttgatcggcttcgccgagatca gaagtctctaaagggaagaggcagcactc	+	+
CY058499	Human H1N1 IAVs	Human	H1N1		2007	USA	A/New_Jersey/15/2007	ggcgatgcccccttccttgatcggcttcgccgagatca gaagtctctaaagggaagaggcagcactc	+	+
FJ445057	Human H1N1 IAVs	Human	H1N1		2007	United_Kingdom	A/England/557/2007	ggcgatgcccccttccttgatcggcttcgccgagatca gaagtctctaaagggaagaggcagcactc	+	+
CY121596	Human H1N1 IAVs	Human	H1N1		2007	USA	A/South_Dakota/06/2007	ggcgatgcccccttccttgatcggcttcgccgagatca gaagtctctaaagggaagaggcagcactc	+	+
HQ853511	Human H1N1 IAVs	Human	H1N1		2007	India	A/KOL/964/2007	ggcgatgcccccttccttgatcggcttcgccgagatca gaagtctctaaagggaagaggcagcactc	+	+
HQ853504	Human H1N1 IAVs	Human	H1N1		2007	India	A/KOL/929/2007	ggcgatgcccccttccttgatcggcttcgccgagatca gaagtctctaaagggaagaggcagcactc	+	+
HQ853507	Human H1N1 IAVs	Human	H1N1		2007	India	A/KOL/943/2007	ggcgatgcccccttccttgatcggcttcgccgagatca gaagtctctaaagggaagaggcagcactc	+	+
CY119278	Human H1N1 IAVs	Human	H1N1		2007	Malaysia	A/Malaysia/1842338/2007	ggcgatgcccccttccttgatcggcttcgccgagatca gaagtctctaaagggaagaggcagcactc	+	+
CY119286	Human H1N1 IAVs	Human	H1N1		2007	Malaysia	A/Malaysia/1882831/2007	ggcgatgcccccttccttgatcggcttcgccgagatca gaagtctctaaagggaagaggcagcactc	+	+
HQ853505	Human H1N1 IAVs	Human	H1N1		2007	India	A/KOL/936/2007	ggcgatgcccccttccttgatcggcttcgccgagatca gaagtctctaaagggaagaggcagcactc	+	+
FJ798789	Human H1N1 IAVs	Human	H1N1		2007	Hungary	A/Nyiregyhaza/01/2007	ggcgatgcccccttccttgatcggcttcgccgagatca gaagtctctaaagggaagaggcagcactc	+	+
HQ853510	Human H1N1 IAVs	Human	H1N1		2007	India	A/KOL/958/2007	ggcgatgcccccttccttgatcggcttcgccgagatca gaagtctctaaagggaagaggcagcactc	+	+
HQ291851	Human H1N1 IAVs	Human	H1N1		2007	Taiwan	A/Taiwan/10092/2007	ggcgatgcccccttccttgatcggcttcgccgagatca gaagtctctaaagggaagaggcagcactc	+	+
GU811752	Human H1N1 IAVs	Human	H1N1		2007	Mexico	A/Mexico/UASLP_002/2007	ggcgatgcccccttccttgatcggcttcgccgagatca gaagtctctaaagggaagaggcagcactc	+	+
CY043429	Human H1N1 IAVs	Human	H1N1		2007	Japan	A/Hokkaido/07H007/2007	ggcgatgcccccttccttgatcggcttcgccgagatca gaagtctctaaagggaagaggcagcactc	+	+
CY043437	Human H1N1 IAVs	Human	H1N1		2007	Japan	A/Hyogo/07K030/2007	ggcgatgcccccttccttgatcggcttcgccgagatca gaagtctctaaagggaagaggcagcactc	+	+
HQ853506	Human H1N1 IAVs	Human	H1N1		2007	India	A/KOL/937/2007	ggcgatgcccccttccttgatcggcttcgccgagatca gaagtctctaaagggaagaggcagcactc	+	+

HQ853509	Human H1N1 IAVs	Human	H1N1		2007	India	A/KOL/956/2007	ggcgatgcccccttccttgatcggcttcgccgagatca<br>gaagtctctaaagggaagaggcagcactc	+	+
HQ853514	Human H1N1 IAVs	Human	H1N1		2007	India	A/KOL/985/2007	ggcgatgcccccttccttgatcggcttcgccgagatca<br>gaagtctctaaagggaagaggcagcactc	+	+
HQ853508	Human H1N1 IAVs	Human	H1N1		2007	India	A/KOL/951/2007	ggcgatgcccccttccttgatcggcttcgccgagatca<br>gaagtctctaaagggaagaggcagcactc	+	+
HQ853513	Human H1N1 IAVs	Human	H1N1		2007	India	A/KOL/975/2007	ggcgatgcccccttccttgatcggcttcgccgagatca<br>gaagtctctaaagggaagaggcagcactc	+	+
FJ445064	Human H1N1 IAVs	Human	H1N1		2007	United_Kingdom	A/England/545/2007	ggcgatgcccccttccttgatcggcttcgccgagatca<br>gaagtctctaaagggaagaggcagcactc	+	+
FJ445030	Human H1N1 IAVs	Human	H1N1		2007	United_Kingdom	A/England/654/2007	ggcgatgcccccttccttgatcggcttcgccgagatca<br>gaagtctctaaagggaagaggcagcactc	+	+
HQ853515	Human H1N1 IAVs	Human	H1N1		2007	India	A/KOL/990/2007	ggcgatgcccccttccttgatcggcttcgccgagatca<br>gaagtctctaaagggaagaggcagcactc	+	+
CY070068	Human H1N1 IAVs	Human	H1N1		2007	Peru	A/Peru/WRAIR1296P/2007	ggcgatgcccccttccttgatcggcttcgccgagatca<br>gaagtctctaaagggaagaggcagcactc	+	+
CY027319	Human H1N1 IAVs	Human	H1N1		2007	USA	A/Florida/UR06_0208/2007	ggcgatgcccccttccttgatcggcttcgccgagatca<br>gaagtctctaaagggaagaggcagcactc	+	+
CY028343	Human H1N1 IAVs	Human	H1N1		2007	USA	A/Florida/UR06_0209/2007	ggcgatgcccccttccttgatcggcttcgccgagatca<br>gaagtctctaaagggaagaggcagcactc	+	+
CY025775	Human H1N1 IAVs	Human	H1N1		2007	USA	A/Florida/UR06_0280/2007	ggcgatgcccccttccttgatcggcttcgccgagatca<br>gaagtctctaaagggaagaggcagcactc	+	+
CY027695	Human H1N1 IAVs	Human	H1N1		2007	USA	A/Oregon/UR06_0179/2007	ggcgatgcccccttccttgatcggcttcgccgagatca<br>gaagtctctaaagggaagaggcagcactc	+	+
CY027927	Human H1N1 IAVs	Human	H1N1		2007	USA	A/Oregon/UR06_0186/2007	ggcgatgcccccttccttgatcggcttcgccgagatca<br>gaagtctctaaagggaagaggcagcactc	+	+
CY027727	Human H1N1 IAVs	Human	H1N1		2007	USA	A/Tennessee/UR06_0113/2007	ggcgatgcccccttccttgatcggcttcgccgagatca<br>gaagtctctaaagggaagaggcagcactc	+	+
CY027135	Human H1N1 IAVs	Human	H1N1		2007	USA	A/Tennessee/UR06_0120/2007	ggcgatgcccccttccttgatcggcttcgccgagatca<br>gaagtctctaaagggaagaggcagcactc	+	+
CY027735	Human H1N1 IAVs	Human	H1N1		2007	USA	A/Tennessee/UR06_0508/2007	ggcgatgcccccttccttgatcggcttcgccgagatca<br>gaagtctctaaagggaagaggcagcactc	+	+
CY028383	Human H1N1 IAVs	Human	H1N1		2007	USA	A/Virginia/UR06_0332/2007	ggcgatgcccccttccttgatcggcttcgccgagatca<br>gaagtctctaaagggaagaggcagcactc	+	+
CY027839	Human H1N1 IAVs	Human	H1N1		2007	USA	A/Kansas/UR06_0085/2007	ggcgatgcccccttccttgatcggcttcgccgagatca<br>gaagtctctaaagggaagaggcagcactc	+	+
CY027087	Human H1N1 IAVs	Human	H1N1		2007	USA	A/Oregon/UR06_0230/2007	ggcgatgcccccttccttgatcggcttcgccgagatca<br>gaagtctctaaagggaagaggcagcactc	+	+
CY026903	Human H1N1 IAVs	Human	H1N1		2007	USA	A/Oregon/UR06_0231/2007	ggcgatgcccccttccttgatcggcttcgccgagatca<br>gaagtctctaaagggaagaggcagcactc	+	+
CY028215	Human H1N1 IAVs	Human	H1N1		2007	USA	A/Oregon/UR06_0219/2007	ggcgatgcccccttccttgatcggcttcgccgagatca<br>gaagtctctaaagggaagaggcagcactc	+	+
CY037787	Human H1N1 IAVs	Human	H1N1		2007	USA	A/Tennessee/UR06_0106/2007	ggcgatgcccccttccttgatcggcttcgccgagatca<br>gaagtctctaaagggaagaggcagcactc	+	+
CY027751	Human H1N1 IAVs	Human	H1N1		2007	USA	A/Tennessee/UR06_0459/2007	ggcgatgcccccttccttgatcggcttcgccgagatca<br>gaagtctctaaagggaagaggcagcactc	+	+
CY027383	Human H1N1 IAVs	Human	H1N1		2007	USA	A/Tennessee/UR06_0119/2007	ggcgatgcccccttccttgatcggcttcgccgagatca<br>gaagtctctaaagggaagaggcagcactc	+	+
CY172683	Human H1N1 IAVs	Human	H1N1		2007	USA	A/New_York/1070/2007	ggcgatgcccccttccttgatcggcttcgccgagatca<br>gaagtctctaaagggaagaggcagcactc	+	+

CY027207	Human H1N1 IAVs	Human	H1N1		2007	USA	A/Mississippi/UR06_0047/2007	ggcgatgcccccttccttgatcggcttcgccgagatca<br>gaagtctctaaagggaagaggcagcactc	+	+
CY025799	Human H1N1 IAVs	Human	H1N1		2007	USA	A/Vermont/UR06_0089/2007	ggcgatgcccccttccttgatcggcttcgccgagatca<br>gaagtctctaaagggaagaggcagcactc	+	+
CY025663	Human H1N1 IAVs	Human	H1N1		2007	USA	A/Vermont/UR06_0301/2007	ggcgatgcccccttccttgatcggcttcgccgagatca<br>gaagtctctaaagggaagaggcagcactc	+	+
CY028039	Human H1N1 IAVs	Human	H1N1		2007	USA	A/Kentucky/UR06_0046/2007	ggcgatgcccccttccttgatcggcttcgccgagatca<br>gaagtctctaaagggaagaggcagcactc	+	+
CY027023	Human H1N1 IAVs	Human	H1N1		2007	USA	A/Kentucky/UR06_0183/2007	ggcgatgcccccttccttgatcggcttcgccgagatca<br>gaagtctctaaagggaagaggcagcactc	+	+
CY028055	Human H1N1 IAVs	Human	H1N1		2007	USA	A/Kentucky/UR06_0425/2007	ggcgatgcccccttccttgatcggcttcgccgagatca<br>gaagtctctaaagggaagaggcagcactc	+	+
CY028087	Human H1N1 IAVs	Human	H1N1		2007	USA	A/Kentucky/UR06_0391/2007	ggcgatgcccccttccttgatcggcttcgccgagatca<br>gaagtctctaaagggaagaggcagcactc	+	+
CY027959	Human H1N1 IAVs	Human	H1N1		2007	USA	A/Oregon/UR06_0185/2007	ggcgatgcccccttccttgatcggcttcgccgagatca<br>gaagtctctaaagggaagaggcagcactc	+	+
CY026967	Human H1N1 IAVs	Human	H1N1		2007	USA	A/Texas/UR06_0157/2007	ggcgatgcccccttccttgatcggcttcgccgagatca<br>gaagtctctaaagggaagaggcagcactc	+	+
CY027167	Human H1N1 IAVs	Human	H1N1		2007	USA	A/Texas/UR06_0305/2007	ggcgatgcccccttccttgatcggcttcgccgagatca<br>gaagtctctaaagggaagaggcagcactc	+	+
CY028760	Human H1N1 IAVs	Human	H1N1		2007	USA	A/Virginia/UR06_0180/2007	ggcgatgcccccttccttgatcggcttcgccgagatca<br>gaagtctctaaagggaagaggcagcactc	+	+
CY026895	Human H1N1 IAVs	Human	H1N1		2007	USA	A/Tennessee/UR06_0078/2007	ggcgatgcccccttccttgatcggcttcgccgagatca<br>gaagtctctaaagggaagaggcagcactc	+	+
CY028095	Human H1N1 IAVs	Human	H1N1		2007	Lebanon	A/Tennessee/UR06_0523/2007	ggcgatgcccccttccttgatcggcttcgccgagatca<br>gaagtctctaaagggaagaggcagcactc	+	+
CY172731	Human H1N1 IAVs	Human	H1N1		2007	USA	A/New_York/1076/2007	ggcgatgcccccttccttgatcggcttcgccgagatca<br>gaagtctctaaagggaagaggcagcactc	+	+
CY037779	Human H1N1 IAVs	Human	H1N1		2007	USA	A/Tennessee/UR06_0379/2007	ggcgatgcccccttccttgatcggcttcgccgagatca<br>gaagtctctaaagggaagaggcagcactc	+	+
CY172715	Human H1N1 IAVs	Human	H1N1		2007	USA	A/New_York/1074/2007	ggcgatgcccccttccttgatcggcttcgccgagatca<br>gaagtctctaaagggaagaggcagcactc	+	+
CY172699	Human H1N1 IAVs	Human	H1N1		2007	USA	A/New_York/1072/2007	ggcgatgcccccttccttgatcggcttcgccgagatca<br>gaagtctctaaagggaagaggcagcactc	+	+
CY027455	Human H1N1 IAVs	Human	H1N1		2007	USA	A/Tennessee/UR06_0414/2007	ggcgatgcccccttccttgatcggcttcgccgagatca<br>gaagtctctaaagggaagaggcagcactc	+	+
CY026663	Human H1N1 IAVs	Human	H1N1		2007	USA	A/Florida/UR06_0501/2007	ggcgatgcccccttccttgatcggcttcgccgagatca<br>gaagtctctaaagggaagaggcagcactc	+	+
CY028079	Human H1N1 IAVs	Human	H1N1		2007	USA	A/Tennessee/UR06_0080/2007	ggcgatgcccccttccttgatcggcttcgccgagatca<br>gaagtctctaaagggaagaggcagcactc	+	+
CY028399	Human H1N1 IAVs	Human	H1N1		2007	USA	A/Tennessee/UR06_0277/2007	ggcgatgcccccttccttgatcggcttcgccgagatca<br>gaagtctctaaagggaagaggcagcactc	+	+
CY028007	Human H1N1 IAVs	Human	H1N1		2007	USA	A/Tennessee/UR06_0312/2007	ggcgatgcccccttccttgatcggcttcgccgagatca<br>gaagtctctaaagggaagaggcagcactc	+	+
HQ853496	Human H1N1 IAVs	Human	H1N1		2007	India	A/KOL/526/2007	ggcgatgcccccttccttgatcggcttcgccgagatca<br>gaagtctctaaagggaagaggcagcactc	+	+
HQ853499	Human H1N1 IAVs	Human	H1N1		2007	India	A/KOL/578/2007	ggcgatgcccccttccttgatcggcttcgccgagatca<br>gaagtctctaaagggaagaggcagcactc	+	+
CY026527	Human H1N1 IAVs	Human	H1N1		2007	USA	A/Colorado/UR06_0053/2007	ggcgatgcccccttccttgatcggcttcgccgagatca<br>gaagtctctaaagggaagaggcagcactc	+	+

CY119238	Human H1N1 IAVs	Human	H1N1		2007	Malaysia	A/Malaysia/1715991/2007	ggcgatgccccttccttgatcggcttcgccgagatca gaagtctctaaagggaagaggcagcactc	+	+
CY119246	Human H1N1 IAVs	Human	H1N1		2007	Malaysia	A/Malaysia/1718249/2007	ggcgatgccccttccttgatcggcttcgccgagatca gaagtctctaaagggaagaggcagcactc	+	+
CY119254	Human H1N1 IAVs	Human	H1N1		2007	Malaysia	A/Malaysia/1718958/2007	ggcgatgccccttccttgatcggcttcgccgagatca gaagtctctaaagggaagaggcagcactc	+	+
CY119310	Human H1N1 IAVs	Human	H1N1		2007	Malaysia	A/Malaysia/1823766/2007	ggcgatgccccttccttgatcggcttcgccgagatca gaagtctctaaagggaagaggcagcactc	+	+
CY118103	Human H1N1 IAVs	Human	H1N1		2007	Malaysia	A/Malaysia/1828663/2007	ggcgatgccccttccttgatcggcttcgccgagatca gaagtctctaaagggaagaggcagcactc	+	+
CY118087	Human H1N1 IAVs	Human	H1N1		2007	Malaysia	A/Malaysia/1788397/2007	ggcgatgccccttccttgatcggcttcgccgagatca gaagtctctaaagggaagaggcagcactc	+	+
CY119270	Human H1N1 IAVs	Human	H1N1		2007	Malaysia	A/Malaysia/1811552/2007	ggcgatgccccttccttgatcggcttcgccgagatca gaagtctctaaagggaagaggcagcactc	+	+
HQ291850	Human H1N1 IAVs	Human	H1N1		2007	Taiwan	A/Taiwan/3700/2007	ggcgatgccccttccttgatcggcttcgccgagatca gaagtctctaaagggaagaggcagcactc	+	+
CY044353	Human H1N1 IAVs	Human	H1N1		2007	South_Korea	A/South_Korea/AF10/2008	ggcgatgccccttccttgatcggcttcgccgagatca gaagtctctaaagggaagaggcagcactc	+	+
KF836391	Human H1N1 IAVs	Human	H1N1		2007	Singapore	A/Singapore/20J/2007	ggcgatgccccttccttgatcggcttcgccgagatca gaagtctctaaagggaagaggcagcactc	+	+
KF836392	Human H1N1 IAVs	Human	H1N1		2007	Singapore	A/Singapore/22A/2007	ggcgatgccccttccttgatcggcttcgccgagatca gaagtctctaaagggaagaggcagcactc	+	+
CY118048	Human H1N1 IAVs	Human	H1N1		2007	Malaysia	A/Malaysia/1758994/2007	ggcgatgccccttccttgatcggcttcgccgagatca gaagtctctaaagggaagaggcagcactc	+	+
CY118111	Human H1N1 IAVs	Human	H1N1		2007	Malaysia	A/Malaysia/1887206/2007	ggcgatgccccttccttgatcggcttcgccgagatca gaagtctctaaagggaagaggcagcactc	+	+
CY118095	Human H1N1 IAVs	Human	H1N1		2007	Malaysia	A/Malaysia/1794173/2007	ggcgatgccccttccttgatcggcttcgccgagatca gaagtctctaaagggaagaggcagcactc	+	+
CY119262	Human H1N1 IAVs	Human	H1N1		2007	Malaysia	A/Malaysia/1798564/2007	ggcgatgccccttccttgatcggcttcgccgagatca gaagtctctaaagggaagaggcagcactc	+	+
CY026631	Human H1N1 IAVs	Human	H1N1		2007	USA	A/New_York/UR06_0199/2007	ggcgatgccccttccttgatcggcttcgccgagatca gaagtctctaaagggaagaggcagcactc	+	+
CY027607	Human H1N1 IAVs	Human	H1N1		2007	USA	A/New_York/UR06_0326/2007	ggcgatgccccttccttgatcggcttcgccgagatca gaagtctctaaagggaagaggcagcactc	+	+
CY119230	Human H1N1 IAVs	Human	H1N1		2007	Malaysia	A/Malaysia/1706215/2007	ggcgatgccccttccttgatcggcttcgccgagatca gaagtctctaaagggaagaggcagcactc	+	+
CY172635	Human H1N1 IAVs	Human	H1N1		2007	USA	A/New_York/1062/2007	ggcgatgccccttccttgatcggcttcgccgagatca gaagtctctaaagggaagaggcagcactc	+	+
KF836395	Human H1N1 IAVs	Human	H1N1		2007	Singapore	A/Singapore/30L/2007	ggcgatgccccttccttgatcggcttcgccgagatca gaagtctctaaagggaagaggcagtactc	+	+
HQ853502	Human H1N1 IAVs	Human	H1N1		2007	India	A/KOL/918/2007	ggcgatgccccttccttgatcggcttcgccgagatca gaagtctctaaagggaagaggcatcactc	+	+
HQ853503	Human H1N1 IAVs	Human	H1N1		2007	India	A/KOL/919/2007	ggcgatgccccttccttgatcggcttcgccgagatca gaagtctctaaagggaagaggcatcactc	+	+
CY031144	Human H1N1 IAVs	Human	H1N1		2007	USA	A/Tennessee/UR06_0236/2007	ggcgatgccccttccttgatcggcttcgccgagatca gaagtctctaaagggacgaggcagcactc	+	+
CY028327	Human H1N1 IAVs	Human	H1N1		2007	USA	A/Texas/UR06_0026/2007	ggcgatgccccttccttgatcggcttcgccgagatca gaagtctctaaagggacgaggcagcactc	+	+
CY028223	Human H1N1 IAVs	Human	H1N1		2007	USA	A/Texas/UR06_0420/2007	ggcgatgccccttccttgatcggcttcgccgagatca gaagtctctaaagggacgaggcagcactc	+	+

CY027887	Human H1N1 IAVs	Human	H1N1		2007	USA	A/Texas/UR06_0503/2007	ggcgatgcccccttccttgatcggcttcgccgagatca<br>gaagtctctaaagggacgaggcagcactc	+	+
CY025449	Human H1N1 IAVs	Human	H1N1		2007	USA	A/Texas/UR06_0467/2007	ggcgatgcccccttccttgatcggcttcgccgagatca<br>gaagtctctaaagggacgaggcagcactc	+	+
CY026503	Human H1N1 IAVs	Human	H1N1		2007	USA	A/Texas/UR06_0468/2007	ggcgatgcccccttccttgatcggcttcgccgagatca<br>gaagtctctaaagggacgaggcagcactc	+	+
CY026343	Human H1N1 IAVs	Human	H1N1		2007	USA	A/Texas/UR06_0542/2007	ggcgatgcccccttccttgatcggcttcgccgagatca<br>gaagtctctaaagggacgaggcagcactc	+	+
CY027047	Human H1N1 IAVs	Human	H1N1		2007	USA	A/Illinois/UR06_0146/2007	ggcgatgcccccttccttgatcggcttcgccgagatca<br>gaagtctctaaggggaaggggcagcactc	-	-
CY026735	Human H1N1 IAVs	Human	H1N1		2007	USA	A/New_York/UR06_0134/2007	ggcgatgcccccttccttgatcggcttcgccgggatca<br>gaagtctctaaaaggaagaggcagcactc	-	-
CY025385	Human H1N1 IAVs	Human	H1N1		2007	USA	A/Alabama/UR06_0455/2007	ggcgatgcccccttccttgatcggcttcgccgggatca<br>gaagtctctaaagggaagaggcagcactc	+	+
CY026679	Human H1N1 IAVs	Human	H1N1		2007	USA	A/California/UR06_0375/2007	ggcgatgcccccttccttgatcggcttcgccgggatca<br>gaagtctctaaagggaagaggcagcactc	+	+
CY026583	Human H1N1 IAVs	Human	H1N1		2007	USA	A/Colorado/UR06_0110/2007	ggcgatgcccccttccttgatcggcttcgccgggatca<br>gaagtctctaaagggaagaggcagcactc	+	+
CY028768	Human H1N1 IAVs	Human	H1N1		2007	USA	A/Kansas/UR06_0299/2007	ggcgatgcccccttccttgatcggcttcgccgggatca<br>gaagtctctaaagggaagaggcagcactc	+	+
CY026655	Human H1N1 IAVs	Human	H1N1		2007	USA	A/Kentucky/UR06_0027/2007	ggcgatgcccccttccttgatcggcttcgccgggatca<br>gaagtctctaaagggaagaggcagcactc	+	+
CY027223	Human H1N1 IAVs	Human	H1N1		2007	USA	A/Kentucky/UR06_0029/2007	ggcgatgcccccttccttgatcggcttcgccgggatca<br>gaagtctctaaagggaagaggcagcactc	+	+
CY026807	Human H1N1 IAVs	Human	H1N1		2007	USA	A/Kentucky/UR06_0058/2007	ggcgatgcccccttccttgatcggcttcgccgggatca<br>gaagtctctaaagggaagaggcagcactc	+	+
CY025457	Human H1N1 IAVs	Human	H1N1		2007	USA	A/Kentucky/UR06_0062/2007	ggcgatgcccccttccttgatcggcttcgccgggatca<br>gaagtctctaaagggaagaggcagcactc	+	+
CY025919	Human H1N1 IAVs	Human	H1N1		2007	USA	A/Kentucky/UR06_0069/2007	ggcgatgcccccttccttgatcggcttcgccgggatca<br>gaagtctctaaagggaagaggcagcactc	+	+
CY026815	Human H1N1 IAVs	Human	H1N1		2007	USA	A/Kentucky/UR06_0071/2007	ggcgatgcccccttccttgatcggcttcgccgggatca<br>gaagtctctaaagggaagaggcagcactc	+	+
CY027175	Human H1N1 IAVs	Human	H1N1		2007	USA	A/Kentucky/UR06_0081/2007	ggcgatgcccccttccttgatcggcttcgccgggatca<br>gaagtctctaaagggaagaggcagcactc	+	+
CY025639	Human H1N1 IAVs	Human	H1N1		2007	USA	A/Kentucky/UR06_0188/2007	ggcgatgcccccttccttgatcggcttcgccgggatca<br>gaagtctctaaagggaagaggcagcactc	+	+
CY025895	Human H1N1 IAVs	Human	H1N1		2007	USA	A/Mississippi/UR06_0537/2007	ggcgatgcccccttccttgatcggcttcgccgggatca<br>gaagtctctaaagggaagaggcagcactc	+	+
CY026383	Human H1N1 IAVs	Human	H1N1		2007	USA	A/New_York/UR06_0056/2007	ggcgatgcccccttccttgatcggcttcgccgggatca<br>gaagtctctaaagggaagaggcagcactc	+	+
CY027447	Human H1N1 IAVs	Human	H1N1		2007	USA	A/Ohio/UR06_0121/2007	ggcgatgcccccttccttgatcggcttcgccgggatca<br>gaagtctctaaagggaagaggcagcactc	+	+
CY028127	Human H1N1 IAVs	Human	H1N1		2007	USA	A/Ohio/UR06_0353/2007	ggcgatgcccccttccttgatcggcttcgccgggatca<br>gaagtctctaaagggaagaggcagcactc	+	+
CY031575	Human H1N1 IAVs	Human	H1N1		2007	USA	A/Ohio/UR06_0591/2007	ggcgatgcccccttccttgatcggcttcgccgggatca<br>gaagtctctaaagggaagaggcagcactc	+	+
CY027967	Human H1N1 IAVs	Human	H1N1		2007	USA	A/Oklahoma/UR06_0241/2007	ggcgatgcccccttccttgatcggcttcgccgggatca<br>gaagtctctaaagggaagaggcagcactc	+	+
CY027639	Human H1N1 IAVs	Human	H1N1		2007	USA	A/Tennessee/UR06_0388/2007	ggcgatgcccccttccttgatcggcttcgccgggatca<br>gaagtctctaaagggaagaggcagcactc	+	+

CY025369	Human H1N1 IAVs	Human	H1N1		2007	USA	A/Texas/UR06_0025/2007	ggcgatgcccccttccttgatcggcttcgccgggatca gaagtctctaaagggaagaggcagcactc	+	+
CY026367	Human H1N1 IAVs	Human	H1N1		2007	USA	A/Texas/UR06_0175/2007	ggcgatgcccccttccttgatcggcttcgccgggatca gaagtctctaaagggaagaggcagcactc	+	+
CY027559	Human H1N1 IAVs	Human	H1N1		2007	USA	A/Texas/UR06_0204/2007	ggcgatgcccccttccttgatcggcttcgccgggatca gaagtctctaaagggaagaggcagcactc	+	+
CY028335	Human H1N1 IAVs	Human	H1N1		2007	USA	A/Texas/UR06_0250/2007	ggcgatgcccccttccttgatcggcttcgccgggatca gaagtctctaaagggaagaggcagcactc	+	+
CY026351	Human H1N1 IAVs	Human	H1N1		2007	USA	A/Texas/UR06_0271/2007	ggcgatgcccccttccttgatcggcttcgccgggatca gaagtctctaaagggaagaggcagcactc	+	+
CY026639	Human H1N1 IAVs	Human	H1N1		2007	USA	A/Texas/UR06_0308/2007	ggcgatgcccccttccttgatcggcttcgccgggatca gaagtctctaaagggaagaggcagcactc	+	+
CY025297	Human H1N1 IAVs	Human	H1N1		2007	USA	A/Texas/UR06_0380/2007	ggcgatgcccccttccttgatcggcttcgccgggatca gaagtctctaaagggaagaggcagcactc	+	+
CY027951	Human H1N1 IAVs	Human	H1N1		2007	USA	A/Virginia/UR06_0594/2007	ggcgatgcccccttccttgatcggcttcgccgggatca gaagtctctaaagggaagaggcagcactc	+	+
CY026015	Human H1N1 IAVs	Human	H1N1		2007	USA	A/Ohio/UR06_0122/2007	ggcgatgcccccttccttgatcggcttcgccgggatca gaagtctctaaagggaagaggcagcactc	+	+
CY027119	Human H1N1 IAVs	Human	H1N1		2007	USA	A/Tennessee/UR06_0152/2007	ggcgatgcccccttccttgatcggcttcgccgggatca gaagtctctaaagggaagaggcagcactc	+	+
CY027255	Human H1N1 IAVs	Human	H1N1		2007	USA	A/Tennessee/UR06_0294/2007	ggcgatgcccccttccttgatcggcttcgccgggatca gaagtctctaaagggaagaggcagcactc	+	+
CY027895	Human H1N1 IAVs	Human	H1N1		2007	USA	A/California/UR06_0440/2007	ggcgatgcccccttccttgatcggcttcgccgggatca gaagtctctaaagggaagaggcagcactc	+	+
CY027903	Human H1N1 IAVs	Human	H1N1		2007	USA	A/Colorado/UR06_0496/2007	ggcgatgcccccttccttgatcggcttcgccgggatca gaagtctctaaagggaagaggcagcactc	+	+
CY027807	Human H1N1 IAVs	Human	H1N1		2007	USA	A/Illinois/UR06_0032/2007	ggcgatgcccccttccttgatcggcttcgccgggatca gaagtctctaaagggaagaggcagcactc	+	+
CY025465	Human H1N1 IAVs	Human	H1N1		2007	USA	A/Kentucky/UR06_0129/2007	ggcgatgcccccttccttgatcggcttcgccgggatca gaagtctctaaagggaagaggcagcactc	+	+
CY025807	Human H1N1 IAVs	Human	H1N1		2007	USA	A/Mississippi/UR06_0048/2007	ggcgatgcccccttccttgatcggcttcgccgggatca gaagtctctaaagggaagaggcagcactc	+	+
CY030065	Human H1N1 IAVs	Human	H1N1		2007	USA	A/Ohio/UR06_0296/2007	ggcgatgcccccttccttgatcggcttcgccgggatca gaagtctctaaagggaagaggcagcactc	+	+
CY027007	Human H1N1 IAVs	Human	H1N1		2007	USA	A/Ohio/UR06_0465/2007	ggcgatgcccccttccttgatcggcttcgccgggatca gaagtctctaaagggaagaggcagcactc	+	+
CY025401	Human H1N1 IAVs	Human	H1N1		2007	USA	A/Texas/UR06_0203/2007	ggcgatgcccccttccttgatcggcttcgccgggatca gaagtctctaaagggaagaggcagcactc	+	+
CY025273	Human H1N1 IAVs	Human	H1N1		2007	USA	A/Texas/UR06_0502/2007	ggcgatgcccccttccttgatcggcttcgccgggatca gaagtctctaaagggaagaggcagcactc	+	+
CY030731	Human H1N1 IAVs	Human	H1N1		2007	USA	A/Kentucky/UR06_0070/2007	ggcgatgcccccttccttgatcggcttcgccgggatca gaagtctctaaagggaagaggcagcactc	+	+
CY025313	Human H1N1 IAVs	Human	H1N1		2007	USA	A/Kentucky/UR06_0082/2007	ggcgatgcccccttccttgatcggcttcgccgggatca gaagtctctaaagggaagaggcagcactc	+	+
CY027263	Human H1N1 IAVs	Human	H1N1		2007	USA	A/Ohio/UR06_0521/2007	ggcgatgcccccttccttgatcggcttcgccgggatca gaagtctctaaagggaagaggcagcactc	+	+
CY028471	Human H1N1 IAVs	Human	H1N1		2007	USA	A/California/UR06_0552/2007	ggcgatgcccccttccttgatcggcttcgccgggatca gaagtctctaaagggaagaggcagcactc	+	+
CY026951	Human H1N1 IAVs	Human	H1N1		2007	USA	A/Ohio/UR06_0518/2007	ggcgatgcccccttccttgatcggcttcgccgggatca gaagtctctaaagggaagaggcagcactc	+	+

CY026719	Human H1N1 IAVs	Human	H1N1		2007	USA	A/Texas/UR06_0582/2007	ggcgatgccccccttccttgatcggcttcgccgggatca gaagtctctaaagggaagaggcagcactc	+	+
CY025927	Human H1N1 IAVs	Human	H1N1		2007	USA	A/Kentucky/UR06_0034/2007	ggcgatgccccccttccttgatcggcttcgccgggatca gaagtctctaaagggaagaggcagcactc	+	+
CY025703	Human H1N1 IAVs	Human	H1N1		2007	USA	A/Kentucky/UR06_0043/2007	ggcgatgccccccttccttgatcggcttcgccgggatca gaagtctctaaagggaagaggcagcactc	+	+
CY027031	Human H1N1 IAVs	Human	H1N1		2007	USA	A/Virginia/UR06_0244/2007	ggcgatgccccccttccttgatcggcttcgccgggatca gaagtctctaaagggaagaggcagcactc	+	+
CY025991	Human H1N1 IAVs	Human	H1N1		2007	USA	A/Virginia/UR06_0245/2007	ggcgatgccccccttccttgatcggcttcgccgggatca gaagtctctaaagggaagaggcagcactc	+	+
CY028415	Human H1N1 IAVs	Human	H1N1		2007	USA	A/Virginia/UR06_0346/2007	ggcgatgccccccttccttgatcggcttcgccgggatca gaagtctctaaagggaagaggcagcactc	+	+
CY028031	Human H1N1 IAVs	Human	H1N1		2007	USA	A/Ohio/UR06_0522/2007	ggcgatgccccccttccttgatcggcttcgccgggatca gaagtctctaaagggaagaggcagcactc	+	+
CY025321	Human H1N1 IAVs	Human	H1N1		2007	USA	A/Mississippi/UR06_0242/2007	ggcgatgccccccttccttgatcggcttcgccgggatca gaagtctctaaagggaagaggcagcactc	+	+
CY026855	Human H1N1 IAVs	Human	H1N1		2007	USA	A/Ohio/UR06_0341/2007	ggcgatgccccccttccttgatcggcttcgccgggatca gaagtctctaaagggaagaggcagcactc	+	+
CY026743	Human H1N1 IAVs	Human	H1N1		2007	USA	A/Kentucky/UR06_0061/2007	ggcgatgccccccttccttgatcggcttcgccgggatca gaagtctctaaagggaagaggcagcactc	+	+
CY027407	Human H1N1 IAVs	Human	H1N1		2007	USA	A/Ohio/UR06_0166/2007	ggcgatgccccccttccttgatcggcttcgccgggatca gaagtctctaaagggaagaggcagcactc	+	+
CY025791	Human H1N1 IAVs	Human	H1N1		2007	USA	A/Kentucky/UR06_0033/2007	ggcgatgccccccttccttgatcggcttcgccgggatca gaagtctctaaagggaagaggcagcactc	+	+
CY027495	Human H1N1 IAVs	Human	H1N1		2007	USA	A/Tennessee/UR06_0473/2007	ggcgatgccccccttccttgatcggcttcgccgggatca gaagtctctaaagggaagaggcagcactc	+	+
CY028119	Human H1N1 IAVs	Human	H1N1		2007	USA	A/Tennessee/UR06_0509/2007	ggcgatgccccccttccttgatcggcttcgccgggatca gaagtctctaaagggaagaggcagcactc	+	+
CY026839	Human H1N1 IAVs	Human	H1N1		2007	USA	A/Virginia/UR06_0139/2007	ggcgatgccccccttccttgatcggcttcgccgggatca gaagtctctaaagggaagaggcagcactc	+	+
CY028103	Human H1N1 IAVs	Human	H1N1		2007	USA	A/Virginia/UR06_0387/2007	ggcgatgccccccttccttgatcggcttcgccgggatca gaagtctctaaagggaagaggcagcactc	+	+
CY037475	Human H1N1 IAVs	Human	H1N1		2007	USA	A/Virginia/UR06_0065/2007	ggcgatgccccccttccttgatcggcttcgccgggatca gaagtctctaaagggaagaggcagcactc	+	+
CY027831	Human H1N1 IAVs	Human	H1N1		2007	USA	A/Virginia/UR06_0075/2007	ggcgatgccccccttccttgatcggcttcgccgggatca gaagtctctaaagggaagaggcagcactc	+	+
CY026943	Human H1N1 IAVs	Human	H1N1		2007	USA	A/Virginia/UR06_0164/2007	ggcgatgccccccttccttgatcggcttcgccgggatca gaagtctctaaagggaagaggcagcactc	+	+
CY027935	Human H1N1 IAVs	Human	H1N1		2007	USA	A/Oregon/UR06_0291/2007	ggcgatgccccccttccttgatcggcttcgccgggatca gaagtctctaaagggaagaggcagcactc	+	+
CY027479	Human H1N1 IAVs	Human	H1N1		2007	USA	A/Tennessee/UR06_0234/2007	ggcgatgccccccttccttgatcggcttcgccgggatca gaagtctctaaagggaagaggcagcactc	+	+
CY026007	Human H1N1 IAVs	Human	H1N1		2007	USA	A/Ohio/UR06_0100/2007	ggcgatgccccccttccttgatcggcttcgccgggatca gaagtctctaaagggaagaggcagcactc	+	+
CY025975	Human H1N1 IAVs	Human	H1N1		2007	USA	A/Ohio/UR06_0325/2007	ggcgatgccccccttccttgatcggcttcgccgggatca gaagtctctaaagggaagaggcagcactc	+	+
CY025551	Human H1N1 IAVs	Human	H1N1		2007	USA	A/Texas/UR06_0563/2007	ggcgatgccccccttccttgatcggcttcgccgggatca gaagtctctaaagggaagaggcagcactc	+	+
CY027183	Human H1N1 IAVs	Human	H1N1		2007	USA	A/Texas/UR06_0176/2007	ggcgatgccccccttccttgatcggcttcgccgggatca gaagtctctaaagggaagaggcagcactc	+	+

CY027335	Human H1N1 IAVs	Human	H1N1		2007	USA	A/Kentucky/UR06_0220/2007	ggcgatgccccccttccttgatcggcttcgccgggatca gaagtctctaaagggaagaggcagcactc	+	+
CY025567	Human H1N1 IAVs	Human	H1N1		2007	USA	A/Texas/UR06_0461/2007	ggcgatgccccccttccttgatcggcttcgccgggatca gaagtctctaaagggaagaggcagcactc	+	+
CY026207	Human H1N1 IAVs	Human	H1N1		2007	USA	A/Kentucky/UR06_0187/2007	ggcgatgccccccttccttgatcggcttcgccgggatca gaagtctctaaagggaagaggcagcactc	+	+
CY027391	Human H1N1 IAVs	Human	H1N1		2007	USA	A/Ohio/UR06_0233/2007	ggcgatgccccccttccttgatcggcttcgccgggatca gaagtctctaaagggaagaggcagcactc	+	+
CY027071	Human H1N1 IAVs	Human	H1N1		2007	USA	A/Ohio/UR06_0443/2007	ggcgatgccccccttccttgatcggcttcgccgggatca gaagtctctaaagggaagaggcagcactc	+	+
CY172675	Human H1N1 IAVs	Human	H1N1		2007	USA	A/New_York/1069/2007	ggcgatgccccccttccttgatcggcttcgccgggatca gaagtctctaaagggaagaggcagcactc	+	+
CY025999	Human H1N1 IAVs	Human	H1N1		2007	USA	A/Virginia/UR06_0109/2007	ggcgatgccccccttccttgatcggcttcgccgggatca gaagtctctaaagggaagaggcagcactc	+	+
CY037659	Human H1N1 IAVs	Human	H1N1		2007	USA	A/Ohio/UR06_0493/2007	ggcgatgccccccttccttgatcggcttcgccgggatca gaagtctctaaagggaagaggcagcactc	+	+
CY026959	Human H1N1 IAVs	Human	H1N1		2007	USA	A/Ohio/UR06_0091/2007	ggcgatgccccccttccttgatcggcttcgccgggatca gaagtctctaaagggaagaggcagcactc	+	+
CY027415	Human H1N1 IAVs	Human	H1N1		2007	USA	A/Alabama/UR06_0536/2007	ggcgatgccccccttccttgatcggcttcgccgggatca gaagtctctaaagggaagaggcagcactc	+	+
CY025631	Human H1N1 IAVs	Human	H1N1		2007	USA	A/Mississippi/UR06_0086/2007	ggcgatgccccccttccttgatcggcttcgccgggatca gaagtctctaaagggaagaggcagcactc	+	+
CY025559	Human H1N1 IAVs	Human	H1N1		2007	USA	A/Mississippi/UR06_0130/2007	ggcgatgccccccttccttgatcggcttcgccgggatca gaagtctctaaagggaagaggcagcactc	+	+
CY025623	Human H1N1 IAVs	Human	H1N1		2007	USA	A/Mississippi/UR06_0142/2007	ggcgatgccccccttccttgatcggcttcgccgggatca gaagtctctaaagggaagaggcagcactc	+	+
CY026767	Human H1N1 IAVs	Human	H1N1		2007	USA	A/California/UR06_0321/2007	ggcgatgccccccttccttgatcggcttcgccgggatca gaagtctctaaagggaagaggcagcactc	+	+
CY025831	Human H1N1 IAVs	Human	H1N1		2007	USA	A/Colorado/UR06_0111/2007	ggcgatgccccccttccttgatcggcttcgccgggatca gaagtctctaaagggaagaggcagcactc	+	+
CY025337	Human H1N1 IAVs	Human	H1N1		2007	USA	A/Colorado/UR06_0255/2007	ggcgatgccccccttccttgatcggcttcgccgggatca gaagtctctaaagggaagaggcagcactc	+	+
CY026783	Human H1N1 IAVs	Human	H1N1		2007	USA	A/Colorado/UR06_0287/2007	ggcgatgccccccttccttgatcggcttcgccgggatca gaagtctctaaagggaagaggcagcactc	+	+
CY026823	Human H1N1 IAVs	Human	H1N1		2007	USA	A/Colorado/UR06_0336/2007	ggcgatgccccccttccttgatcggcttcgccgggatca gaagtctctaaagggaagaggcagcactc	+	+
CY027103	Human H1N1 IAVs	Human	H1N1		2007	USA	A/Florida/UR06_0049/2007	ggcgatgccccccttccttgatcggcttcgccgggatca gaagtctctaaagggaagaggcagcactc	+	+
CY037451	Human H1N1 IAVs	Human	H1N1		2007	USA	A/Florida/UR06_0265/2007	ggcgatgccccccttccttgatcggcttcgccgggatca gaagtctctaaagggaagaggcagcactc	+	+
CY026327	Human H1N1 IAVs	Human	H1N1		2007	USA	A/Florida/UR06_0354/2007	ggcgatgccccccttccttgatcggcttcgccgggatca gaagtctctaaagggaagaggcagcactc	+	+
CY025783	Human H1N1 IAVs	Human	H1N1		2007	USA	A/Florida/UR06_0383/2007	ggcgatgccccccttccttgatcggcttcgccgggatca gaagtctctaaagggaagaggcagcactc	+	+
CY026727	Human H1N1 IAVs	Human	H1N1		2007	USA	A/Florida/UR06_0412/2007	ggcgatgccccccttccttgatcggcttcgccgggatca gaagtctctaaagggaagaggcagcactc	+	+
CY027615	Human H1N1 IAVs	Human	H1N1		2007	USA	A/Florida/UR06_0447/2007	ggcgatgccccccttccttgatcggcttcgccgggatca gaagtctctaaagggaagaggcagcactc	+	+
CY026551	Human H1N1 IAVs	Human	H1N1		2007	USA	A/Florida/UR06_0577/2007	ggcgatgccccccttccttgatcggcttcgccgggatca gaagtctctaaagggaagaggcagcactc	+	+

CY027631	Human H1N1 IAVs	Human	H1N1		2007	USA	A/Florida/UR06_0578/2007	ggcgatgccccccttccttgatcggcttcgccgggatca gaagtctctaaagggaagaggcagcactc	+	+
CY027855	Human H1N1 IAVs	Human	H1N1		2007	USA	A/Kansas/UR06_0143/2007	ggcgatgccccccttccttgatcggcttcgccgggatca gaagtctctaaagggaagaggcagcactc	+	+
CY027287	Human H1N1 IAVs	Human	H1N1		2007	USA	A/Kentucky/UR06_0371/2007	ggcgatgccccccttccttgatcggcttcgccgggatca gaagtctctaaagggaagaggcagcactc	+	+
CY026399	Human H1N1 IAVs	Human	H1N1		2007	USA	A/Mississippi/UR06_0595/2007	ggcgatgccccccttccttgatcggcttcgccgggatca gaagtctctaaagggaagaggcagcactc	+	+
CY172587	Human H1N1 IAVs	Human	H1N1		2007	USA	A/New_York/1055/2007	ggcgatgccccccttccttgatcggcttcgccgggatca gaagtctctaaagggaagaggcagcactc	+	+
CY172611	Human H1N1 IAVs	Human	H1N1		2007	USA	A/New_York/1059/2007	ggcgatgccccccttccttgatcggcttcgccgggatca gaagtctctaaagggaagaggcagcactc	+	+
CY172667	Human H1N1 IAVs	Human	H1N1		2007	USA	A/New_York/1068/2007	ggcgatgccccccttccttgatcggcttcgccgggatca gaagtctctaaagggaagaggcagcactc	+	+
CY172691	Human H1N1 IAVs	Human	H1N1		2007	USA	A/New_York/1071/2007	ggcgatgccccccttccttgatcggcttcgccgggatca gaagtctctaaagggaagaggcagcactc	+	+
CY028391	Human H1N1 IAVs	Human	H1N1		2007	USA	A/North_Carolina/UR06_0365/2007	ggcgatgccccccttccttgatcggcttcgccgggatca gaagtctctaaagggaagaggcagcactc	+	+
CY026511	Human H1N1 IAVs	Human	H1N1		2007	USA	A/Tennessee/UR06_0087/2007	ggcgatgccccccttccttgatcggcttcgccgggatca gaagtctctaaagggaagaggcagcactc	+	+
CY041446	Human H1N1 IAVs	Human	H1N1		2007	USA	A/Texas/UR06_0323/2007	ggcgatgccccccttccttgatcggcttcgccgggatca gaagtctctaaagggaagaggcagcactc	+	+
CY027343	Human H1N1 IAVs	Human	H1N1		2007	USA	A/Texas/UR06_0357/2007	ggcgatgccccccttccttgatcggcttcgccgggatca gaagtctctaaagggaagaggcagcactc	+	+
CY025695	Human H1N1 IAVs	Human	H1N1		2007	USA	A/Texas/UR06_0359/2007	ggcgatgccccccttccttgatcggcttcgccgggatca gaagtctctaaagggaagaggcagcactc	+	+
CY025655	Human H1N1 IAVs	Human	H1N1		2007	USA	A/Vermont/UR06_0090/2007	ggcgatgccccccttccttgatcggcttcgccgggatca gaagtctctaaagggaagaggcagcactc	+	+
CY037459	Human H1N1 IAVs	Human	H1N1		2007	USA	A/Vermont/UR06_0330/2007	ggcgatgccccccttccttgatcggcttcgccgggatca gaagtctctaaagggaagaggcagcactc	+	+
CY026623	Human H1N1 IAVs	Human	H1N1		2007	USA	A/Vermont/UR06_0511/2007	ggcgatgccccccttccttgatcggcttcgccgggatca gaagtctctaaagggaagaggcagcactc	+	+
CY037467	Human H1N1 IAVs	Human	H1N1		2007	USA	A/Vermont/UR06_0513/2007	ggcgatgccccccttccttgatcggcttcgccgggatca gaagtctctaaagggaagaggcagcactc	+	+
CY026759	Human H1N1 IAVs	Human	H1N1		2007	USA	A/Vermont/UR06_0556/2007	ggcgatgccccccttccttgatcggcttcgccgggatca gaagtctctaaagggaagaggcagcactc	+	+
CY025823	Human H1N1 IAVs	Human	H1N1		2007	USA	A/Vermont/UR06_0574/2007	ggcgatgccccccttccttgatcggcttcgccgggatca gaagtctctaaagggaagaggcagcactc	+	+
CY026175	Human H1N1 IAVs	Human	H1N1		2007	USA	A/Vermont/UR06_0575/2007	ggcgatgccccccttccttgatcggcttcgccgggatca gaagtctctaaagggaagaggcagcactc	+	+
CY025473	Human H1N1 IAVs	Human	H1N1		2007	USA	A/Vermont/UR06_0576/2007	ggcgatgccccccttccttgatcggcttcgccgggatca gaagtctctaaagggaagaggcagcactc	+	+
CY027975	Human H1N1 IAVs	Human	H1N1		2007	USA	A/Virginia/UR06_0266/2007	ggcgatgccccccttccttgatcggcttcgccgggatca gaagtctctaaagggaagaggcagcactc	+	+
CY026935	Human H1N1 IAVs	Human	H1N1		2007	USA	A/Virginia/UR06_0267/2007	ggcgatgccccccttccttgatcggcttcgccgggatca gaagtctctaaagggaagaggcagcactc	+	+
CY026919	Human H1N1 IAVs	Human	H1N1		2007	USA	A/Virginia/UR06_0384/2007	ggcgatgccccccttccttgatcggcttcgccgggatca gaagtctctaaagggaagaggcagcactc	+	+
CY025537	Human H1N1 IAVs	Human	H1N1		2007	USA	A/Florida/UR06_0355/2007	ggcgatgccccccttccttgatcggcttcgccgggatca gaagtctctaaagggaagaggcagcactc	+	+

CY026999	Human H1N1 IAVs	Human	H1N1		2007	USA	A/North_Carolina/UR06_0364/2007	ggcgatgccccccttccttgatcggcttcgccgggatca<br>gaagtctctaaagggaagaggcagcactc	+	+
CY028063	Human H1N1 IAVs	Human	H1N1		2007	USA	A/Tennessee/UR06_0238/2007	ggcgatgccccccttccttgatcggcttcgccgggatca<br>gaagtctctaaagggaagaggcagcactc	+	+
CY025305	Human H1N1 IAVs	Human	H1N1		2007	USA	A/Vermont/UR06_0050/2007	ggcgatgccccccttccttgatcggcttcgccgggatca<br>gaagtctctaaagggaagaggcagcactc	+	+
CY025815	Human H1N1 IAVs	Human	H1N1		2007	USA	A/Vermont/UR06_0051/2007	ggcgatgccccccttccttgatcggcttcgccgggatca<br>gaagtctctaaagggaagaggcagcactc	+	+
CY027999	Human H1N1 IAVs	Human	H1N1		2007	USA	A/Virginia/UR06_0092/2007	ggcgatgccccccttccttgatcggcttcgccgggatca<br>gaagtctctaaagggaagaggcagcactc	+	+
CY031136	Human H1N1 IAVs	Human	H1N1		2007	USA	A/Virginia/UR06_0228/2007	ggcgatgccccccttccttgatcggcttcgccgggatca<br>gaagtctctaaagggaagaggcagcactc	+	+
CY026567	Human H1N1 IAVs	Human	H1N1		2007	USA	A/Texas/UR06_0397/2007	ggcgatgccccccttccttgatcggcttcgccgggatca<br>gaagtctctaaagggaagaggcagcactc	+	+
CY025767	Human H1N1 IAVs	Human	H1N1		2007	USA	A/Vermont/UR06_0485/2007	ggcgatgccccccttccttgatcggcttcgccgggatca<br>gaagtctctaaagggaagaggcagcactc	+	+
CY026575	Human H1N1 IAVs	Human	H1N1		2007	USA	A/Vermont/UR06_0573/2007	ggcgatgccccccttccttgatcggcttcgccgggatca<br>gaagtctctaaagggaagaggcagcactc	+	+
CY026319	Human H1N1 IAVs	Human	H1N1		2007	USA	A/California/UR06_0374/2007	ggcgatgccccccttccttgatcggcttcgccgggatca<br>gaagtctctaaagggaagaggcagcactc	+	+
CY172643	Human H1N1 IAVs	Human	H1N1		2007	USA	A/New_York/1063/2007	ggcgatgccccccttccttgatcggcttcgccgggatca<br>gaagtctctaaagggaagaggcagcactc	+	+
CY027599	Human H1N1 IAVs	Human	H1N1		2007	USA	A/Texas/UR06_0445/2007	ggcgatgccccccttccttgatcggcttcgccgggatca<br>gaagtctctaaagggaagaggcagcactc	+	+
CY025583	Human H1N1 IAVs	Human	H1N1		2007	USA	A/Mississippi/UR06_0145/2007	ggcgatgccccccttccttgatcggcttcgccgggatca<br>gaagtctctaaagggaagaggcagcactc	+	+
CY027191	Human H1N1 IAVs	Human	H1N1		2007	USA	A/Vermont/UR06_0472/2007	ggcgatgccccccttccttgatcggcttcgccgggatca<br>gaagtctctaaagggaagaggcagcactc	+	+
CY028159	Human H1N1 IAVs	Human	H1N1		2007	USA	A/Virginia/UR06_0351/2007	ggcgatgccccccttccttgatcggcttcgccgggatca<br>gaagtctctaaagggaagaggcagcactc	+	+
CY027863	Human H1N1 IAVs	Human	H1N1		2007	USA	A/Virginia/UR06_0360/2007	ggcgatgccccccttccttgatcggcttcgccgggatca<br>gaagtctctaaagggaagaggcagcactc	+	+
CY037443	Human H1N1 IAVs	Human	H1N1		2007	USA	A/Texas/UR06_0422/2007	ggcgatgccccccttccttgatcggcttcgccgggatca<br>gaagtctctaaagggaagaggcagcactc	+	+
CY172723	Human H1N1 IAVs	Human	H1N1		2007	USA	A/New_York/1075/2007	ggcgatgccccccttccttgatcggcttcgccgggatca<br>gaagtctctaaagggaagaggcagcactc	+	+
CY027271	Human H1N1 IAVs	Human	H1N1		2007	USA	A/Tennessee/UR06_0239/2007	ggcgatgccccccttccttgatcggcttcgccgggatca<br>gaagtctctaaagggaagaggcagcactc	+	+
CY027647	Human H1N1 IAVs	Human	H1N1		2007	USA	A/Virginia/UR06_0297/2007	ggcgatgccccccttccttgatcggcttcgccgggatca<br>gaagtctctaaagggaagaggcagcactc	+	+
CY028736	Human H1N1 IAVs	Human	H1N1		2007	USA	A/California/UR06_0462/2007	ggcgatgccccccttccttgatcggcttcgccgggatca<br>gaagtctctaaagggaagaggcagcactc	+	+
CY026239	Human H1N1 IAVs	Human	H1N1		2007	USA	A/Colorado/UR06_0498/2007	ggcgatgccccccttccttgatcggcttcgccgggatca<br>gaagtctctaaagggaagaggcagcactc	+	+
CY172651	Human H1N1 IAVs	Human	H1N1		2007	USA	A/New_York/1064/2007	ggcgatgccccccttccttgatcggcttcgccgggatca<br>gaagtctctaaagggaagaggcagcactc	+	+
CY027671	Human H1N1 IAVs	Human	H1N1		2007	USA	A/Illinois/UR06_0088/2007	ggcgatgccccccttccttgatcggcttcgccgggatca<br>gaagtctctaaagggaagaggcagcactc	+	+
CY028351	Human H1N1 IAVs	Human	H1N1		2007	USA	A/Illinois/UR06_0096/2007	ggcgatgccccccttccttgatcggcttcgccgggatca<br>gaagtctctaaagggaagaggcagcactc	+	+

CY027367	Human H1N1 IAVs	Human	H1N1		2007	USA	A/Illinois/UR06_0098/2007	ggcgatgcccccttccttgatcggcttcgccgggatca gaagtctctaaagggaagaggcagcactc	+	+
CY026183	Human H1N1 IAVs	Human	H1N1		2007	USA	A/Illinois/UR06_0223/2007	ggcgatgcccccttccttgatcggcttcgccgggatca gaagtctctaaagggaagaggcagcactc	+	+
CY025521	Human H1N1 IAVs	Human	H1N1		2007	USA	A/Illinois/UR06_0456/2007	ggcgatgcccccttccttgatcggcttcgccgggatca gaagtctctaaagggaagaggcagcactc	+	+
CY028015	Human H1N1 IAVs	Human	H1N1		2007	USA	A/Kansas/UR06_0084/2007	ggcgatgcccccttccttgatcggcttcgccgggatca gaagtctctaaagggaagaggcagcactc	+	+
CY027039	Human H1N1 IAVs	Human	H1N1		2007	USA	A/Kansas/UR06_0104/2007	ggcgatgcccccttccttgatcggcttcgccgggatca gaagtctctaaagggaagaggcagcactc	+	+
CY025967	Human H1N1 IAVs	Human	H1N1		2007	USA	A/Kansas/UR06_0140/2007	ggcgatgcccccttccttgatcggcttcgccgggatca gaagtctctaaagggaagaggcagcactc	+	+
CY028023	Human H1N1 IAVs	Human	H1N1		2007	USA	A/Kentucky/UR06_0162/2007	ggcgatgcccccttccttgatcggcttcgccgggatca gaagtctctaaagggaagaggcagcactc	+	+
CY025951	Human H1N1 IAVs	Human	H1N1		2007	USA	A/Kentucky/UR06_0257/2007	ggcgatgcccccttccttgatcggcttcgccgggatca gaagtctctaaagggaagaggcagcactc	+	+
CY036859	Human H1N1 IAVs	Human	H1N1		2007	USA	A/Texas/UR06_0156/2007	ggcgatgcccccttccttgatcggcttcgccgggatca gaagtctctaaagggaagaggcagcactc	+	+
CY027623	Human H1N1 IAVs	Human	H1N1		2007	USA	A/Texas/UR06_0216/2007	ggcgatgcccccttccttgatcggcttcgccgggatca gaagtctctaaagggaagaggcagcactc	+	+
CY026599	Human H1N1 IAVs	Human	H1N1		2007	USA	A/Texas/UR06_0309/2007	ggcgatgcccccttccttgatcggcttcgccgggatca gaagtctctaaagggaagaggcagcactc	+	+
CY026391	Human H1N1 IAVs	Human	H1N1		2007	USA	A/Texas/UR06_0444/2007	ggcgatgcccccttccttgatcggcttcgccgggatca gaagtctctaaagggaagaggcagcactc	+	+
CY027791	Human H1N1 IAVs	Human	H1N1		2007	USA	A/Illinois/UR06_0227/2007	ggcgatgcccccttccttgatcggcttcgccgggatca gaagtctctaaagggaagaggcagcactc	+	+
CY027351	Human H1N1 IAVs	Human	H1N1		2007	USA	A/Illinois/UR06_0249/2007	ggcgatgcccccttccttgatcggcttcgccgggatca gaagtctctaaagggaagaggcagcactc	+	+
CY025329	Human H1N1 IAVs	Human	H1N1		2007	USA	A/Illinois/UR06_0377/2007	ggcgatgcccccttccttgatcggcttcgccgggatca gaagtctctaaagggaagaggcagcactc	+	+
CY027431	Human H1N1 IAVs	Human	H1N1		2007	USA	A/Kansas/UR06_0283/2007	ggcgatgcccccttccttgatcggcttcgccgggatca gaagtctctaaagggaagaggcagcactc	+	+
CY027983	Human H1N1 IAVs	Human	H1N1		2007	USA	A/Kansas/UR06_0284/2007	ggcgatgcccccttccttgatcggcttcgccgggatca gaagtctctaaagggaagaggcagcactc	+	+
CY025983	Human H1N1 IAVs	Human	H1N1		2007	USA	A/Kentucky/UR06_0128/2007	ggcgatgcccccttccttgatcggcttcgccgggatca gaagtctctaaagggaagaggcagcactc	+	+
CY027463	Human H1N1 IAVs	Human	H1N1		2007	USA	A/Kentucky/UR06_0184/2007	ggcgatgcccccttccttgatcggcttcgccgggatca gaagtctctaaagggaagaggcagcactc	+	+
CY027015	Human H1N1 IAVs	Human	H1N1		2007	USA	A/Kentucky/UR06_0327/2007	ggcgatgcccccttccttgatcggcttcgccgggatca gaagtctctaaagggaagaggcagcactc	+	+
CY026991	Human H1N1 IAVs	Human	H1N1		2007	USA	A/Kentucky/UR06_0328/2007	ggcgatgcccccttccttgatcggcttcgccgggatca gaagtctctaaagggaagaggcagcactc	+	+
CY028752	Human H1N1 IAVs	Human	H1N1		2007	USA	A/Kentucky/UR06_0372/2007	ggcgatgcccccttccttgatcggcttcgccgggatca gaagtctctaaagggaagaggcagcactc	+	+
CY025687	Human H1N1 IAVs	Human	H1N1		2007	USA	A/Texas/UR06_0193/2007	ggcgatgcccccttccttgatcggcttcgccgggatca gaagtctctaaagggaagaggcagcactc	+	+
CY025529	Human H1N1 IAVs	Human	H1N1		2007	USA	A/Texas/UR06_0196/2007	ggcgatgcccccttccttgatcggcttcgccgggatca gaagtctctaaagggaagaggcagcactc	+	+
CY028455	Human H1N1 IAVs	Human	H1N1		2007	USA	A/Tennessee/UR06_0073/2007	ggcgatgcccccttccttgatcggcttcgccgggatca gaagtctctaaagggaagaggcagcactc	+	+

CY026615	Human H1N1 IAVs	Human	H1N1		2007	USA	A/Illinois/UR06_0376/2007	ggcgatgcccccttccttgatcggcttcgccgggatca<br>gaagtctctaaagggaagaggcagcactc	+	+
CY025249	Human H1N1 IAVs	Human	H1N1		2007	USA	A/Illinois/UR006_018/2007	ggcgatgcccccttccttgatcggcttcgccgggatca<br>gaagtctctaaagggaagaggcagcactc	+	+
CY025575	Human H1N1 IAVs	Human	H1N1		2007	USA	A/Illinois/UR06_0248/2007	ggcgatgcccccttccttgatcggcttcgccgggatca<br>gaagtctctaaagggaagaggcagcactc	+	+
CY027655	Human H1N1 IAVs	Human	H1N1		2007	USA	A/Virginia/UR06_0562/2007	ggcgatgcccccttccttgatcggcttcgccgggatca<br>gaagtctctaaagggaagaggcagcactc	+	+
CY031534	Human H1N1 IAVs	Human	H1N1		2007	USA	A/Kansas/UR06_0102/2007	ggcgatgcccccttccttgatcggcttcgccgggatca<br>gaagtctctaaagggaagaggcagcactc	+	+
CY025241	Human H1N1 IAVs	Human	H1N1		2007	USA	A/Illinois/UR06_0019/2007	ggcgatgcccccttccttgatcggcttcgccgggatca<br>gaagtctctaaagggaagaggcagcactc	+	+
CY025433	Human H1N1 IAVs	Human	H1N1		2007	USA	A/Kentucky/UR06_0072/2007	ggcgatgcccccttccttgatcggcttcgccgggatca<br>gaagtctctaaagggaagaggcagcactc	+	+
CY026695	Human H1N1 IAVs	Human	H1N1		2007	USA	A/Illinois/UR06_0224/2007	ggcgatgcccccttccttgatcggcttcgccgggatca<br>gaagtctctaaagggaagaggcagcactc	+	+
CY027743	Human H1N1 IAVs	Human	H1N1		2007	USA	A/Kansas/UR06_0068/2007	ggcgatgcccccttccttgatcggcttcgccgggatca<br>gaagtctctaaagggaagaggcagcactc	+	+
CY028367	Human H1N1 IAVs	Human	H1N1		2007	USA	A/Kansas/UR06_0192/2007	ggcgatgcccccttccttgatcggcttcgccgggatca<br>gaagtctctaaagggaagaggcagcactc	+	+
CY030073	Human H1N1 IAVs	Human	H1N1		2007	USA	A/Kansas/UR06_0103/2007	ggcgatgcccccttccttgatcggcttcgccgggatca<br>gaagtctctaaagggaagaggcagcactc	+	+
CY027783	Human H1N1 IAVs	Human	H1N1		2007	USA	A/Kentucky/UR06_0339/2007	ggcgatgcccccttccttgatcggcttcgccgggatca<br>gaagtctctaaagggaagaggcagcactc	+	+
CY026335	Human H1N1 IAVs	Human	H1N1		2007	USA	A/Texas/UR06_0540/2007	ggcgatgcccccttccttgatcggcttcgccgggatca<br>gaagtctctaaagggaagaggcagcactc	+	+
CY025409	Human H1N1 IAVs	Human	H1N1		2007	USA	A/Colorado/UR06_0207/2007	ggcgatgcccccttccttgatcggcttcgccgggatca<br>gaagtctctaaagggaagaggcagcactc	+	+
CY026879	Human H1N1 IAVs	Human	H1N1		2007	USA	A/Kentucky/UR06_0123/2007	ggcgatgcccccttccttgatcggcttcgccgggatca<br>gaagtctctaaagggaagaggcagcactc	+	+
CY026871	Human H1N1 IAVs	Human	H1N1		2007	USA	A/Kentucky/UR06_0127/2007	ggcgatgcccccttccttgatcggcttcgccgggatca<br>gaagtctctaaagggaagaggcagcactc	+	+
CY027055	Human H1N1 IAVs	Human	H1N1		2007	USA	A/Kentucky/UR06_0154/2007	ggcgatgcccccttccttgatcggcttcgccgggatca<br>gaagtctctaaagggaagaggcagcactc	+	+
CY028167	Human H1N1 IAVs	Human	H1N1		2007	USA	A/Kentucky/UR06_0258/2007	ggcgatgcccccttccttgatcggcttcgccgggatca<br>gaagtctctaaagggaagaggcagcactc	+	+
CY027991	Human H1N1 IAVs	Human	H1N1		2007	USA	A/Ohio/UR06_0411/2007	ggcgatgcccccttccttgatcggcttcgccgggatca<br>gaagtctctaaagggaagaggcagcactc	+	+
CY028047	Human H1N1 IAVs	Human	H1N1		2007	USA	A/Kentucky/UR06_0401/2007	ggcgatgcccccttccttgatcggcttcgccgggatca<br>gaagtctctaaagggaagaggcagcactc	+	+
CY028295	Human H1N1 IAVs	Human	H1N1		2007	USA	A/Texas/UR06_0038/2007	ggcgatgcccccttccttgatcggcttcgccgggatca<br>gaagtctctaaagggaagaggcagcactc	+	+
CY027815	Human H1N1 IAVs	Human	H1N1		2007	USA	A/Texas/UR06_0039/2007	ggcgatgcccccttccttgatcggcttcgccgggatca<br>gaagtctctaaagggaagaggcagcactc	+	+
CY031533	Human H1N1 IAVs	Human	H1N1		2007	USA	A/Virginia/UR06_0107/2007	ggcgatgcccccttccttgatcggcttcgccgggatca<br>gaagtctctaaagggaagaggcagcactc	+	+
CY028111	Human H1N1 IAVs	Human	H1N1		2007	USA	A/Tennessee/UR06_0076/2007	ggcgatgcccccttccttgatcggcttcgccgggatca<br>gaagtctctaaagggaagaggcagcactc	+	+
CY033469	Human H1N1 IAVs	Human	H1N1		2007	USA	A/Oregon/UR06_0609/2007	ggcgatgcccccttccttgatcggcttcgccgggatca<br>gaagtctctaaagggaagaggcagcactc	+	+

CY028135	Human H1N1 IAVs	Human	H1N1		2007	USA	A/Oklahoma/UR06_0519/2007	ggcgatgccccttccttgatcggcttcgccgggatca gaagtctctaaagggaagaggcagcactc	+	+
CY027911	Human H1N1 IAVs	Human	H1N1		2007	USA	A/Illinois/UR06_0475/2007	ggcgatgccccttccttgatcggcttcgccgggatca gaagtctctaaagggaagaggcagcactc	+	+
CY027759	Human H1N1 IAVs	Human	H1N1		2007	USA	A/Virginia/UR06_0114/2007	ggcgatgccccttccttgatcggcttcgccgggatca gaagtctctaaagggaagaggcagcactc	+	+
CY028359	Human H1N1 IAVs	Human	H1N1		2007	USA	A/Kentucky/UR06_0181/2007	ggcgatgccccttccttgatcggcttcgccgggatca gaagtctctaaagggacgaggcagcactc	+	+
CY027919	Human H1N1 IAVs	Human	H1N1		2007	USA	A/Kentucky/UR06_0259/2007	ggcgatgccccttccttgatcggcttcgccgggatca gaagtctctaaagggacgaggcagcactc	+	+
CY044488	Human H1N1 IAVs	Human	H1N1		2007	USA	A/Boston/12/2007	ggcgatgcccccttttcttgatcggcttcgccgagatcag aagtctctaaagggaagaggcagcactc	-	-
CY027063	Human H1N1 IAVs	Human	H1N1		2007	USA	A/Tennessee/UR06_0151/2007	ggcgatgctcccttccttgatcggcttcgccgggatcag aagtctctaaagggaagaggcagcactc	+	+
HQ853497	Human H1N1 IAVs	Human	H1N1		2007	India	A/KOL/536/2007	ggtgatgccccattccttgatcggcttcgccgagatcag aaatccctaagaggaaggggcagcactc	-	*
HQ853500	Human H1N1 IAVs	Human	H1N1		2007	India	A/KOL/596/2007	ggtgatgccccattccttgatcggcttcgccgagatcag aaatccctaagaggaaggggcagcactc	-	*
HQ853512	Human H1N1 IAVs	Human	H1N1		2007	India	A/KOL/968/2007	ggtgatgccccattccttgatcggcttcgccgagatcag aaatccctaagaggaaggggcagcactc	-	*
HQ291856	Human H1N1 IAVs	Human	H1N1		2008	Taiwan	A/Taiwan/8885/2008	ggcgacgccccttccttgatcggcttcgccgagatca gaagtctctaaagggaagaggcagcactc	+	+
CY173059	Human H1N1 IAVs	Human	H1N1		2008	USA	A/New_York/1123/2008	ggcgatgccccttccttgaccggcttcgccgagatca gaagtctctaaagggaagaggcagcactc	-	-
GU646020	Human H1N1 IAVs	Swine	H1N1		2008	China	A/swine/Fujian/0325/2008	ggcgatgccccttccttgatcggcctcgccgagatca gaagtctctaaagggaagaggcagcactc	-	-
CY040254	Human H1N1 IAVs	Human	H1N1		2008	Nicaragua	A/Managua/3153.01/2008	ggcgatgccccttccttgatcggctccgccgagatca gaagtctctaaagggaagaggcagcactc	-	*
CY037699	Human H1N1 IAVs	Human	H1N1		2008	USA	A/Kentucky/UR07_0061/2008	ggcgatgccccttccttgatcggcttcgccgagatca gaagtccctaaagggaagaggcagcactc	+	+
CY039147	Human H1N1 IAVs	Human	H1N1		2008	Nicaragua	A/Managua/254.01/2008	ggcgatgccccttccttgatcggcttcgccgagatca gaagtccctaaagggaagaggcagcactc	+	+
CY039139	Human H1N1 IAVs	Human	H1N1		2008	Nicaragua	A/Managua/1409.01/2008	ggcgatgccccttccttgatcggcttcgccgagatca gaagtctctaaagggaagaggcagcactc	+	+
CY172963	Human H1N1 IAVs	Human	H1N1		2008	USA	A/New_York/1111/2008	ggcgatgccccttccttgatcggcttcgccgagatca gaagtctctaaagggaagaggcagcactc	+	+
CY173083	Human H1N1 IAVs	Human	H1N1		2008	USA	A/New_York/1126/2008	ggcgatgccccttccttgatcggcttcgccgagatca gaagtctctaaagggaagaggcagcactc	+	+
CY045864	Human H1N1 IAVs	Human	H1N1		2008	Nicaragua	A/Managua/1985.01/2008	ggcgatgccccttccttgatcggcttcgccgagatca gaagtctctaaagggaagaggcagcactc	+	+
CY040278	Human H1N1 IAVs	Human	H1N1		2008	Nicaragua	A/Managua/4924.01/2008	ggcgatgccccttccttgatcggcttcgccgagatca gaagtctctaaagggaagaggcagcactc	+	+
CY043804	Human H1N1 IAVs	Human	H1N1		2008	Nicaragua	A/Managua/5007.01/2008	ggcgatgccccttccttgatcggcttcgccgagatca gaagtctctaaagggaagaggcagcactc	+	+
CY044417	Human H1N1 IAVs	Human	H1N1		2008	Nicaragua	A/Managua/496.01/2008	ggcgatgccccttccttgatcggcttcgccgagatca gaagtctctaaagggaagaggcagcactc	+	+
CY040094	Human H1N1 IAVs	Human	H1N1		2008	Taiwan	A/Taiwan/70013/2008	ggcgatgccccttccttgatcggcttcgccgagatca gaagtctctaaagggaagaggcagcactc	+	+
CY037339	Human H1N1 IAVs	Human	H1N1		2008	Japan	A/Japan/AF07/2008	ggcgatgccccttccttgatcggcttcgccgagatca gaagtctctaaagggaagaggcagcactc	+	+

CY043389	Human H1N1 IAVs	Human	H1N1		2008	Japan	A/Niigata/07F102/2008	ggcgatgcccccttccttgatcggcttcgccgagatca<br>gaagtctctaaagggaagaggcagcactc	+	+
CY172971	Human H1N1 IAVs	Human	H1N1		2008	USA	A/New_York/1112/2008	ggcgatgcccccttccttgatcggcttcgccgagatca<br>gaagtctctaaagggaagaggcagcactc	+	+
CY172995	Human H1N1 IAVs	Human	H1N1		2008	USA	A/New_York/1115/2008	ggcgatgcccccttccttgatcggcttcgccgagatca<br>gaagtctctaaagggaagaggcagcactc	+	+
CY173027	Human H1N1 IAVs	Human	H1N1		2008	USA	A/New_York/1119/2008	ggcgatgcccccttccttgatcggcttcgccgagatca<br>gaagtctctaaagggaagaggcagcactc	+	+
CY173035	Human H1N1 IAVs	Human	H1N1		2008	USA	A/New_York/1120/2008	ggcgatgcccccttccttgatcggcttcgccgagatca<br>gaagtctctaaagggaagaggcagcactc	+	+
CY044688	Human H1N1 IAVs	Human	H1N1		2008	USA	A/Boston/52/2008	ggcgatgcccccttccttgatcggcttcgccgagatca<br>gaagtctctaaagggaagaggcagcactc	+	+
CY100880	Human H1N1 IAVs	Human	H1N1		2008	Colombia	A/Medellin/WRAIR1297P/2008	ggcgatgcccccttccttgatcggcttcgccgagatca<br>gaagtctctaaagggaagaggcagcactc	+	+
FJ265019	Human H1N1 IAVs	Human	H1N1		2008	Denmark	A/Denmark/21/2008	ggcgatgcccccttccttgatcggcttcgccgagatca<br>gaagtctctaaagggaagaggcagcactc	+	+
CY104874	Human H1N1 IAVs	Human	H1N1		2008	Viet_Nam	A/HaNoi/BT241/2008	ggcgatgcccccttccttgatcggcttcgccgagatca<br>gaagtctctaaagggaagaggcagcactc	+	+
CY105122	Human H1N1 IAVs	Human	H1N1		2008	Viet_Nam	A/HaNoi/N229/2008	ggcgatgcccccttccttgatcggcttcgccgagatca<br>gaagtctctaaagggaagaggcagcactc	+	+
CY104970	Human H1N1 IAVs	Human	H1N1		2008	Viet_Nam	A/Hue/H274/2008	ggcgatgcccccttccttgatcggcttcgccgagatca<br>gaagtctctaaagggaagaggcagcactc	+	+
CY105114	Human H1N1 IAVs	Human	H1N1		2008	Viet_Nam	A/KhanhHoa/KH57/2008	ggcgatgcccccttccttgatcggcttcgccgagatca<br>gaagtctctaaagggaagaggcagcactc	+	+
CY173067	Human H1N1 IAVs	Human	H1N1		2008	USA	A/New_York/1124/2008	ggcgatgcccccttccttgatcggcttcgccgagatca<br>gaagtctctaaagggaagaggcagcactc	+	+
CY043565	Human H1N1 IAVs	Human	H1N1		2008	Japan	A/Yokohama/78/2008	ggcgatgcccccttccttgatcggcttcgccgagatca<br>gaagtctctaaagggaagaggcagcactc	+	+
JN582059	Human H1N1 IAVs	Human	H1N1		2008	USA	A/New_York/08_1326/2008	ggcgatgcccccttccttgatcggcttcgccgagatca<br>gaagtctctaaagggaagaggcagcactc	+	+
CY173019	Human H1N1 IAVs	Human	H1N1		2008	USA	A/New_York/1118/2008	ggcgatgcccccttccttgatcggcttcgccgagatca<br>gaagtctctaaagggaagaggcagcactc	+	+
CY105154	Human H1N1 IAVs	Human	H1N1		2008	Viet_Nam	A/HaNoi/TX233/2008	ggcgatgcccccttccttgatcggcttcgccgagatca<br>gaagtctctaaagggaagaggcagcactc	+	+
JN582051	Human H1N1 IAVs	Human	H1N1		2008	USA	A/New_York/08_1253/2008	ggcgatgcccccttccttgatcggcttcgccgagatca<br>gaagtctctaaagggaagaggcagcactc	+	+
CY104978	Human H1N1 IAVs	Human	H1N1		2008	Viet_Nam	A/Hue/H339/2008	ggcgatgcccccttccttgatcggcttcgccgagatca<br>gaagtctctaaagggaagaggcagcactc	+	+
CY105090	Human H1N1 IAVs	Human	H1N1		2008	Viet_Nam	A/KhanhHoa/KH161/2008	ggcgatgcccccttccttgatcggcttcgccgagatca<br>gaagtctctaaagggaagaggcagcactc	+	+
CY105186	Human H1N1 IAVs	Human	H1N1		2008	Viet_Nam	A/TayNinh/HCM1329/2008	ggcgatgcccccttccttgatcggcttcgccgagatca<br>gaagtctctaaagggaagaggcagcactc	+	+
CY104954	Human H1N1 IAVs	Human	H1N1		2008	Viet_Nam	A/Hue/H257/2008	ggcgatgcccccttccttgatcggcttcgccgagatca<br>gaagtctctaaagggaagaggcagcactc	+	+
CY104986	Human H1N1 IAVs	Human	H1N1		2008	Viet_Nam	A/Hue/H386/2008	ggcgatgcccccttccttgatcggcttcgccgagatca<br>gaagtctctaaagggaagaggcagcactc	+	+
CY104583	Human H1N1 IAVs	Human	H1N1		2008	Viet_Nam	A/Hue/H433/2008	ggcgatgcccccttccttgatcggcttcgccgagatca<br>gaagtctctaaagggaagaggcagcactc	+	+
CY104994	Human H1N1 IAVs	Human	H1N1		2008	Viet_Nam	A/Hue/H396/2008	ggcgatgcccccttccttgatcggcttcgccgagatca<br>gaagtctctaaagggaagaggcagcactc	+	+

CY105026	Human H1N1 IAVs	Human	H1N1		2008	Viet_Nam	A/Hue/H413/2008	ggcgatgccccccttccttgatcggcttcgccgagatca gaagtctctaaagggaagaggcagcactc	+	+
CY105042	Human H1N1 IAVs	Human	H1N1		2008	Viet_Nam	A/Hue/H418/2008	ggcgatgccccccttccttgatcggcttcgccgagatca gaagtctctaaagggaagaggcagcactc	+	+
CY105050	Human H1N1 IAVs	Human	H1N1		2008	Viet_Nam	A/Hue/H423/2008	ggcgatgccccccttccttgatcggcttcgccgagatca gaagtctctaaagggaagaggcagcactc	+	+
CY105058	Human H1N1 IAVs	Human	H1N1		2008	Viet_Nam	A/Hue/H432/2008	ggcgatgccccccttccttgatcggcttcgccgagatca gaagtctctaaagggaagaggcagcactc	+	+
CY105066	Human H1N1 IAVs	Human	H1N1		2008	Viet_Nam	A/Hue/H435/2008	ggcgatgccccccttccttgatcggcttcgccgagatca gaagtctctaaagggaagaggcagcactc	+	+
CY105074	Human H1N1 IAVs	Human	H1N1		2008	Viet_Nam	A/Hue/H436/2008	ggcgatgccccccttccttgatcggcttcgccgagatca gaagtctctaaagggaagaggcagcactc	+	+
CY105018	Human H1N1 IAVs	Human	H1N1		2008	Viet_Nam	A/Hue/H441/2008	ggcgatgccccccttccttgatcggcttcgccgagatca gaagtctctaaagggaagaggcagcactc	+	+
CY105082	Human H1N1 IAVs	Human	H1N1		2008	Viet_Nam	A/Hue/H442/2008	ggcgatgccccccttccttgatcggcttcgccgagatca gaagtctctaaagggaagaggcagcactc	+	+
CY105010	Human H1N1 IAVs	Human	H1N1		2008	Viet_Nam	A/Hue/H409/2008	ggcgatgccccccttccttgatcggcttcgccgagatca gaagtctctaaagggaagaggcagcactc	+	+
CY104600	Human H1N1 IAVs	Human	H1N1		2008	Viet_Nam	A/Hue/H485/2008	ggcgatgccccccttccttgatcggcttcgccgagatca gaagtctctaaagggaagaggcagcactc	+	+
CY173003	Human H1N1 IAVs	Human	H1N1		2008	USA	A/New_York/1116/2008	ggcgatgccccccttccttgatcggcttcgccgagatca gaagtctctaaagggaagaggcagcactc	+	+
CY037683	Human H1N1 IAVs	Human	H1N1		2008	USA	A/Florida/UR07_0022/2008	ggcgatgccccccttccttgatcggcttcgccgagatca gaagtctctaaagggaagaggcagcactc	+	+
CY040246	Human H1N1 IAVs	Human	H1N1		2008	Nicaragua	A/Managua/3307.01/2008	ggcgatgccccccttccttgatcggcttcgccgagatca gaagtctctaaagggaagaggcagcactc	+	+
FJ445062	Human H1N1 IAVs	Human	H1N1		2008	United_Kingdom	A/England/26/2008	ggcgatgccccccttccttgatcggcttcgccgagatca gaagtctctaaagggaagaggcagcactc	+	+
CY044345	Human H1N1 IAVs	Human	H1N1		2008	USA	A/New_Jersey/AF09/2008	ggcgatgccccccttccttgatcggcttcgccgagatca gaagtctctaaagggaagaggcagcactc	+	+
CY105106	Human H1N1 IAVs	Human	H1N1		2008	Viet_Nam	A/KhanhHoa/KH38/2008	ggcgatgccccccttccttgatcggcttcgccgagatca gaagtctctaaagggaagaggcagcactc	+	+
CY040118	Human H1N1 IAVs	Human	H1N1		2008	Taiwan	A/Taiwan/70167/2008	ggcgatgccccccttccttgatcggcttcgccgagatca gaagtctctaaagggaagaggcagcactc	+	+
CY118145	Human H1N1 IAVs	Human	mixed		2008	Malaysia	A/Malaysia/1978169/2008	ggcgatgccccccttccttgatcggcttcgccgagatca gaagtctctaaagggaagaggcagcactc	+	+
CY040110	Human H1N1 IAVs	Human	H1N1		2008	Taiwan	A/Taiwan/70132/2008	ggcgatgccccccttccttgatcggcttcgccgagatca gaagtctctaaagggaagaggcagcactc	+	+
CY040238	Human H1N1 IAVs	Human	H1N1		2008	Nicaragua	A/Managua/2860.01/2008	ggcgatgccccccttccttgatcggcttcgccgagatca gaagtctctaaagggaagaggcagcactc	+	+
CY040270	Human H1N1 IAVs	Human	H1N1		2008	Nicaragua	A/Managua/309.01/2008	ggcgatgccccccttccttgatcggcttcgccgagatca gaagtctctaaagggaagaggcagcactc	+	+
CY043740	Human H1N1 IAVs	Human	H1N1		2008	Nicaragua	A/Managua/3418.01/2008	ggcgatgccccccttccttgatcggcttcgccgagatca gaagtctctaaagggaagaggcagcactc	+	+
CY040262	Human H1N1 IAVs	Human	H1N1		2008	Nicaragua	A/Managua/3759.02/2008	ggcgatgccccccttccttgatcggcttcgccgagatca gaagtctctaaagggaagaggcagcactc	+	+
CY039155	Human H1N1 IAVs	Human	H1N1		2008	Nicaragua	A/Managua/5380.01/2008	ggcgatgccccccttccttgatcggcttcgccgagatca gaagtctctaaagggaagaggcagcactc	+	+
CY043780	Human H1N1 IAVs	Human	H1N1		2008	Nicaragua	A/Managua/5751.01/2008	ggcgatgccccccttccttgatcggcttcgccgagatca gaagtctctaaagggaagaggcagcactc	+	+

CY040286	Human H1N1 IAVs	Human	H1N1		2008	Nicaragua	A/Managua/4412.01/2008	ggcgatgccccccttccttgatcggcttcgccgagatca gaagtctctaaagggaagaggcagcactc	+	+
CY043796	Human H1N1 IAVs	Human	H1N1		2008	Nicaragua	A/Managua/4444.01/2008	ggcgatgccccccttccttgatcggcttcgccgagatca gaagtctctaaagggaagaggcagcactc	+	+
CY038931	Human H1N1 IAVs	Human	H1N1		2008	Nicaragua	A/Managua/4537.03/2008	ggcgatgccccccttccttgatcggcttcgccgagatca gaagtctctaaagggaagaggcagcactc	+	+
CY039451	Human H1N1 IAVs	Human	H1N1		2008	Nicaragua	A/Managua/4315.04/2008	ggcgatgccccccttccttgatcggcttcgccgagatca gaagtctctaaagggaagaggcagcactc	+	+
GU811749	Human H1N1 IAVs	Human	H1N1		2008	Mexico	A/Mexico/UASLP_003/2008	ggcgatgccccccttccttgatcggcttcgccgagatca gaagtctctaaagggaagaggcagcactc	+	+
CY073931	Human H1N1 IAVs	Human	H1N1		2008	Mexico	A/Mexico/UASLP_005/2008	ggcgatgccccccttccttgatcggcttcgccgagatca gaagtctctaaagggaagaggcagcactc	+	+
CY073939	Human H1N1 IAVs	Human	H1N1		2008	Mexico	A/Mexico/UASLP_006/2008	ggcgatgccccccttccttgatcggcttcgccgagatca gaagtctctaaagggaagaggcagcactc	+	+
CY073956	Human H1N1 IAVs	Human	H1N1		2008	Mexico	A/Mexico/UASLP_008/2008	ggcgatgccccccttccttgatcggcttcgccgagatca gaagtctctaaagggaagaggcagcactc	+	+
CY073964	Human H1N1 IAVs	Human	H1N1		2008	Mexico	A/Mexico/UASLP_009/2008	ggcgatgccccccttccttgatcggcttcgccgagatca gaagtctctaaagggaagaggcagcactc	+	+
CY173043	Human H1N1 IAVs	Human	H1N1		2008	USA	A/New_York/1121/2008	ggcgatgccccccttccttgatcggcttcgccgagatca gaagtctctaaagggaagaggcagcactc	+	+
CY173075	Human H1N1 IAVs	Human	H1N1		2008	USA	A/New_York/1125/2008	ggcgatgccccccttccttgatcggcttcgccgagatca gaagtctctaaagggaagaggcagcactc	+	+
CY173051	Human H1N1 IAVs	Human	H1N1		2008	USA	A/New_York/1122/2008	ggcgatgccccccttccttgatcggcttcgccgagatca gaagtctctaaagggaagaggcagcactc	+	+
CY043485	Human H1N1 IAVs	Human	H1N1		2008	Japan	A/Nagasaki/07N020/2008	ggcgatgccccccttccttgatcggcttcgccgagatca gaagtctctaaagggaagaggcagcactc	+	+
HQ291847	Human H1N1 IAVs	Human	H1N1		2008	Taiwan	A/Taiwan/8949/2008	ggcgatgccccccttccttgatcggcttcgccgagatca gaagtctctaaagggaagaggcagcactc	+	+
HQ291867	Human H1N1 IAVs	Human	H1N1		2008	Taiwan	A/Taiwan/9042/2008	ggcgatgccccccttccttgatcggcttcgccgagatca gaagtctctaaagggaagaggcagcactc	+	+
CY043557	Human H1N1 IAVs	Human	H1N1		2008	Japan	A/Tottori/08T010/2008	ggcgatgccccccttccttgatcggcttcgccgagatca gaagtctctaaagggaagaggcagcactc	+	+
FJ265018	Human H1N1 IAVs	Human	H1N1		2008	Denmark	A/Denmark/151/2008	ggcgatgccccccttccttgatcggcttcgccgagatca gaagtctctaaagggaagaggcagcactc	+	+
FJ265015	Human H1N1 IAVs	Human	H1N1		2008	Denmark	A/Denmark/52/2008	ggcgatgccccccttccttgatcggcttcgccgagatca gaagtctctaaagggaagaggcagcactc	+	+
CY118171	Human H1N1 IAVs	Human	H1N1		2008	Malaysia	A/Malaysia/1999752/2008	ggcgatgccccccttccttgatcggcttcgccgagatca gaagtctctaaagggaagaggcagcactc	+	+
HQ291857	Human H1N1 IAVs	Human	H1N1		2008	Taiwan	A/Taiwan/10103/2008	ggcgatgccccccttccttgatcggcttcgccgagatca gaagtctctaaagggaagaggcagcactc	+	+
HQ291858	Human H1N1 IAVs	Human	H1N1		2008	Taiwan	A/Taiwan/11526/2008	ggcgatgccccccttccttgatcggcttcgccgagatca gaagtctctaaagggaagaggcagcactc	+	+
HQ291854	Human H1N1 IAVs	Human	H1N1		2008	Taiwan	A/Taiwan/2885/2008	ggcgatgccccccttccttgatcggcttcgccgagatca gaagtctctaaagggaagaggcagcactc	+	+
CY040190	Human H1N1 IAVs	Human	H1N1		2008	Nicaragua	A/Managua/1038.01/2008	ggcgatgccccccttccttgatcggcttcgccgagatca gaagtctctaaagggaagaggcagcactc	+	+
CY104938	Human H1N1 IAVs	Human	H1N1		2008	Viet_Nam	A/DaNang/DN467/2008	ggcgatgccccccttccttgatcggcttcgccgagatca gaagtctctaaagggaagaggcagcactc	+	+
CY104591	Human H1N1 IAVs	Human	H1N1		2008	Viet_Nam	A/Hue/H445/2008	ggcgatgccccccttccttgatcggcttcgccgagatca gaagtctctaaagggaagaggcagcactc	+	+

CY119318	Human H1N1 IAVs	Human	H1N1		2008	Malaysia	A/Malaysia/2104192/2008	ggcgatgccccccttccttgatcggcttcgccgagatca gaagtctctaaagggaagaggcagcactc	+	+
CY119294	Human H1N1 IAVs	Human	H1N1		2008	Malaysia	A/Malaysia/2132379/2008	ggcgatgccccccttccttgatcggcttcgccgagatca gaagtctctaaagggaagaggcagcactc	+	+
FJ265017	Human H1N1 IAVs	Human	H1N1		2008	Denmark	A/Denmark/122/2008	ggcgatgccccccttccttgatcggcttcgccgagatca gaagtctctaaagggaagaggcagcactc	+	+
CY172923	Human H1N1 IAVs	Human	H1N1		2008	USA	A/New_York/1105/2008	ggcgatgccccccttccttgatcggcttcgccgagatca gaagtctctaaagggaagaggcagcactc	+	+
CY173307	Human H1N1 IAVs	Human	H1N1		2008	USA	A/New_York/1158/2008	ggcgatgccccccttccttgatcggcttcgccgagatca gaagtctctaaagggaagaggcagcactc	+	+
FJ265016	Human H1N1 IAVs	Human	H1N1		2008	Denmark	A/Denmark/22/2008	ggcgatgccccccttccttgatcggcttcgccgagatca gaagtctctaaagggaagaggcagcactc	+	+
CY041222	Human H1N1 IAVs	Human	H1N1		2008	Nicaragua	A/Managua/1225.02/2008	ggcgatgccccccttccttgatcggcttcgccgagatca gaagtctctaaagggaagaggcagcactc	+	+
CY039123	Human H1N1 IAVs	Human	H1N1		2008	Nicaragua	A/Managua/3029.01/2008	ggcgatgccccccttccttgatcggcttcgccgagatca gaagtctctaaagggaagaggcagcactc	+	+
CY044393	Human H1N1 IAVs	Human	H1N1		2008	Nicaragua	A/Managua/4359.01/2008	ggcgatgccccccttccttgatcggcttcgccgagatca gaagtctctaaagggaagaggcagcactc	+	+
CY039131	Human H1N1 IAVs	Human	H1N1		2008	Nicaragua	A/Managua/521.01/2008	ggcgatgccccccttccttgatcggcttcgccgagatca gaagtctctaaagggaagaggcagcactc	+	+
CY172939	Human H1N1 IAVs	Human	H1N1		2008	USA	A/New_York/1108/2008	ggcgatgccccccttccttgatcggcttcgccgagatca gaagtctctaaagggaagaggcagcactc	+	+
CY039467	Human H1N1 IAVs	Human	H1N1		2008	Nicaragua	A/Managua/156.01/2008	ggcgatgccccccttccttgatcggcttcgccgagatca gaagtctctaaagggaagaggcagcactc	+	+
CY039459	Human H1N1 IAVs	Human	H1N1		2008	Nicaragua	A/Managua/5214.01/2008	ggcgatgccccccttccttgatcggcttcgccgagatca gaagtctctaaagggaagaggcagcactc	+	+
CY040294	Human H1N1 IAVs	Human	H1N1		2008	Nicaragua	A/Managua/3570.01/2008	ggcgatgccccccttccttgatcggcttcgccgagatca gaagtctctaaagggaagaggcagcactc	+	+
CY037331	Human H1N1 IAVs	Human	H1N1		2008	USA	A/Washington/AF06/2007	ggcgatgccccccttccttgatcggcttcgccgagatca gaagtctctaaagggaagaggcagcactc	+	+
CY172915	Human H1N1 IAVs	Human	H1N1		2008	USA	A/New_York/1104/2008	ggcgatgccccccttccttgatcggcttcgccgagatca gaagtctctaaagggaagaggcagcactc	+	+
CY172931	Human H1N1 IAVs	Human	H1N1		2008	USA	A/New_York/1107/2008	ggcgatgccccccttccttgatcggcttcgccgagatca gaagtctctaaagggaagaggcagcactc	+	+
CY172947	Human H1N1 IAVs	Human	H1N1		2008	USA	A/New_York/1109/2008	ggcgatgccccccttccttgatcggcttcgccgagatca gaagtctctaaagggaagaggcagcactc	+	+
CY038811	Human H1N1 IAVs	Human	H1N1		2008	USA	A/Florida/UR07_0026/2008	ggcgatgccccccttccttgatcggcttcgccgagatca gaagtctctaaagggaagaggcagcactc	+	+
CY173011	Human H1N1 IAVs	Human	H1N1		2008	USA	A/New_York/1117/2008	ggcgatgccccccttccttgatcggcttcgccgagatca gaagtctctaaagggaagaggcagcactc	+	+
CY038923	Human H1N1 IAVs	Human	H1N1		2008	Nicaragua	A/Managua/107.01/2008	ggcgatgccccccttccttgatcggcttcgccgagatca gaagtctctaaagggaagaggcagcactc	+	+
CY043788	Human H1N1 IAVs	Human	H1N1		2008	Nicaragua	A/Managua/954.02/2008	ggcgatgccccccttccttgatcggcttcgccgagatca gaagtctctaaagggaagaggcagcactc	+	+
CY105002	Human H1N1 IAVs	Human	H1N1		2008	Viet_Nam	A/Hue/H400/2008	ggcgatgccccccttccttgatcggcttcgccgagatca gaagtctctaaagggaagaggcagcactc	+	+
HQ291852	Human H1N1 IAVs	Human	H1N1		2008	Taiwan	A/Taiwan/0258/2008	ggcgatgccccccttccttgatcggcttcgccgagatca gaagtctctaaagggaagaggcagcactc	+	+
CY038539	Human H1N1 IAVs	Human	H1N1		2008	Nicaragua	A/Managua/4032.01/2008	ggcgatgccccccttccttgatcggcttcgccgagatca gaagtctctaaagggaagaggcagcactc	+	+

CY044409	Human H1N1 IAVs	Human	H1N1		2008	Nicaragua	A/Managua/4070.01/2008	ggcgatgcccccttccttgatcggcttcgccgagatcagaagtctctaaagggaagaggcagcactc	+	+
CY040230	Human H1N1 IAVs	Human	H1N1		2008	Nicaragua	A/Managua/2055.01/2008	ggcgatgcccccttccttgatcggcttcgccgagatcagaagtctctaaagggaagaggcagcactc	+	+
CY039475	Human H1N1 IAVs	Human	H1N1		2008	Nicaragua	A/Managua/3027.01/2008	ggcgatgcccccttccttgatcggcttcgccgagatcagaagtctctaaagggaagaggcagcactc	+	+
CY040166	Human H1N1 IAVs	Human	H1N1		2008	Nicaragua	A/Managua/3180.01/2008	ggcgatgcccccttccttgatcggcttcgccgagatcagaagtctctaaagggaagaggcagcactc	+	+
CY040182	Human H1N1 IAVs	Human	H1N1		2008	Nicaragua	A/Managua/4341.01/2008	ggcgatgcccccttccttgatcggcttcgccgagatcagaagtctctaaagggaagaggcagcactc	+	+
CY044425	Human H1N1 IAVs	Human	H1N1		2008	Nicaragua	A/Managua/4017.02/2008	ggcgatgcccccttccttgatcggcttcgccgagatcagaagtctctaaagggaagaggcagcactc	+	+
CY039483	Human H1N1 IAVs	Human	H1N1		2008	Nicaragua	A/Managua/4086.02/2008	ggcgatgcccccttccttgatcggcttcgccgagatcagaagtctctaaagggaagaggcagcactc	+	+
HQ291865	Human H1N1 IAVs	Human	H1N1		2008	Taiwan	A/Taiwan/5857/2008	ggcgatgcccccttccttgatcggcttcgccgagatcagaagtctctaaagggaagaggcagcactc	+	+
HQ291846	Human H1N1 IAVs	Human	H1N1		2008	Taiwan	A/Taiwan/5858/2008	ggcgatgcccccttccttgatcggcttcgccgagatcagaagtctctaaagggaagaggcagcactc	+	+
CY044664	Human H1N1 IAVs	Human	H1N1		2008	USA	A/Boston/49/2008	ggcgatgcccccttccttgatcggcttcgccgagatcagaagtctctaaagggaagaggcagcactc	+	+
CY105138	Human H1N1 IAVs	Human	H1N1		2008	Viet_Nam	A/HaNoi/TX200/2008	ggcgatgcccccttccttgatcggcttcgccgagatcagaagtctctaaagggaagaggcagcactc	+	+
CY105130	Human H1N1 IAVs	Human	H1N1		2008	Viet_Nam	A/ThaiBinh/TB289/2008	ggcgatgcccccttccttgatcggcttcgccgagatcagaagtctctaaagggaagaggcagcactc	+	+
CY043493	Human H1N1 IAVs	Human	H1N1		2008	Japan	A/Nagasaki/07N035/2008	ggcgatgcccccttccttgatcggcttcgccgagatcagaagtctctaaagggaagaggcagcactc	+	+
GQ396602	Human H1N1 IAVs	Human	H1N1		2008	Myanmar	A/Yangon/M195/2008	ggcgatgcccccttccttgatcggcttcgccgagatcagaagtctctaaagggaagaggcagcactc	+	+
CY044560	Human H1N1 IAVs	Human	H1N1		2008	USA	A/Boston/34/2008	ggcgatgcccccttccttgatcggcttcgccgagatcagaagtctctaaagggaagaggcagcactc	+	+
HQ291855	Human H1N1 IAVs	Human	H1N1		2008	Taiwan	A/Taiwan/3293/2008	ggcgatgcccccttccttgatcggcttcgccgagatcagaagtctctaaagggaagaggcagcactc	+	+
CY041214	Human H1N1 IAVs	Human	H1N1		2008	Nicaragua	A/Managua/2512.01/2008	ggcgatgcccccttccttgatcggcttcgccgagatcagaagtctctaaagggaagaggcagcactc	+	+
CY043453	Human H1N1 IAVs	Human	H1N1		2008	Japan	A/Kyoto/07K316/2008	ggcgatgcccccttccttgatcggcttcgccgagatcagaagtctctaaagggaagaggcagcactc	+	+
CY104882	Human H1N1 IAVs	Human	H1N1		2008	Viet_Nam	A/DaNang/DN238/2008	ggcgatgcccccttccttgatcggcttcgccgagatcagaagtctctaaagggaagaggcagcactc	+	+
CY104890	Human H1N1 IAVs	Human	H1N1		2008	Viet_Nam	A/DaNang/DN329/2008	ggcgatgcccccttccttgatcggcttcgccgagatcagaagtctctaaagggaagaggcagcactc	+	+
CY104962	Human H1N1 IAVs	Human	H1N1		2008	Viet_Nam	A/Hue/H259/2008	ggcgatgcccccttccttgatcggcttcgccgagatcagaagtctctaaagggaagaggcagcactc	+	+
CY105098	Human H1N1 IAVs	Human	H1N1		2008	Viet_Nam	A/KhanhHoa/KH321/2008	ggcgatgcccccttccttgatcggcttcgccgagatcagaagtctctaaagggaagaggcagcactc	+	+
CY118187	Human H1N1 IAVs	Human	H1N1		2008	Malaysia	A/Malaysia/2095797/2008	ggcgatgcccccttccttgatcggcttcgccgagatcagaagtctctaaagggaagaggcagcactc	+	+
CY043405	Human H1N1 IAVs	Human	H1N1		2008	Japan	A/Niigata/07F191/2008	ggcgatgcccccttccttgatcggcttcgccgagatcagaagtctctaaagggaagaggcagcactc	+	+
CY104946	Human H1N1 IAVs	Human	H1N1		2008	Viet_Nam	A/DaNang/DN479/2008	ggcgatgcccccttccttgatcggcttcgccgagatcagaagtctctaaagggaagaggcagcactc	+	+

CY105034	Human H1N1 IAVs	Human	H1N1		2008	Viet_Nam	A/Hue/H414/2008	ggcgatgccccccttccttgatcggcttcgccgagatca gaagtctctaaagggaagaggcagcactc	+	+
CY104575	Human H1N1 IAVs	Human	H1N1		2008	Viet_Nam	A/Hue/H354/2008	ggcgatgccccccttccttgatcggcttcgccgagatca gaagtctctaaagggaagaggcagcactc	+	+
CY104906	Human H1N1 IAVs	Human	H1N1		2008	Viet_Nam	A/DaNang/DN364/2008	ggcgatgccccccttccttgatcggcttcgccgagatca gaagtctctaaagggaagaggcagcactc	+	+
CY104914	Human H1N1 IAVs	Human	H1N1		2008	Viet_Nam	A/DaNang/DN365/2008	ggcgatgccccccttccttgatcggcttcgccgagatca gaagtctctaaagggaagaggcagcactc	+	+
CY105146	Human H1N1 IAVs	Human	H1N1		2008	Viet_Nam	A/HaNoi/TX222/2008	ggcgatgccccccttccttgatcggcttcgccgagatca gaagtctctaaagggaagaggcagcactc	+	+
CY043445	Human H1N1 IAVs	Human	H1N1		2008	Japan	A/Kyoto/07K303/2008	ggcgatgccccccttccttgatcggcttcgccgagatca gaagtctctaaagggaagaggcagcactc	+	+
CY121652	Human H1N1 IAVs	Human	H1N1		2008	Hong_Kong	A/Hong_Kong/1870/2008	ggcgatgccccccttccttgatcggcttcgccgagatca gaagtctctaaagggaagaggcagcactc	+	+
HQ291868	Human H1N1 IAVs	Human	H1N1		2008	Taiwan	A/Taiwan/6723/2008	ggcgatgccccccttccttgatcggcttcgccgagatca gaagtctctaaagggaagaggcagcactc	+	+
HQ291862	Human H1N1 IAVs	Human	H1N1		2008	Taiwan	A/Taiwan/2832/2008	ggcgatgccccccttccttgatcggcttcgccgagatca gaagtctctaaagggaagaggcagcactc	+	+
GQ396634	Human H1N1 IAVs	Human	H1N1		2008	Myanmar	A/Naypyitaw/M783/2008	ggcgatgccccccttccttgatcggcttcgccgagatca gaagtctctaaagggaagaggcagcactc	+	+
GQ396610	Human H1N1 IAVs	Human	H1N1		2008	Myanmar	A/Yangon/M285/2008	ggcgatgccccccttccttgatcggcttcgccgagatca gaagtctctaaagggaagaggcagcactc	+	+
CY043413	Human H1N1 IAVs	Human	H1N1		2008	Japan	A/Gunma/07G002/2008	ggcgatgccccccttccttgatcggcttcgccgagatca gaagtctctaaagggaagaggcagcactc	+	+
CY043469	Human H1N1 IAVs	Human	H1N1		2008	Japan	A/Nagasaki/07N005/2008	ggcgatgccccccttccttgatcggcttcgccgagatca gaagtctctaaagggaagaggcagcactc	+	+
CY043397	Human H1N1 IAVs	Human	H1N1		2008	Japan	A/Niigata/07F125/2008	ggcgatgccccccttccttgatcggcttcgccgagatca gaagtctctaaagggaagaggcagcactc	+	+
CY104930	Human H1N1 IAVs	Human	H1N1		2008	Viet_Nam	A/DaNang/DN432/2008	ggcgatgccccccttccttgatcggcttcgccgagatca gaagtctctaaagggaagaggcagcactc	+	+
HQ291853	Human H1N1 IAVs	Human	H1N1		2008	Taiwan	A/Taiwan/2823/2008	ggcgatgccccccttccttgatcggcttcgccgagatca gaagtctctaaagggaagaggcagcactc	+	+
CY104560	Human H1N1 IAVs	Human	H1N1		2008	Viet_Nam	A/DaNang/DN302/2008	ggcgatgccccccttccttgatcggcttcgccgggatca gaagtctctaaagggaagaggcagcactc	+	+
CY104898	Human H1N1 IAVs	Human	H1N1		2008	Viet_Nam	A/DaNang/DN345/2008	ggcgatgccccccttccttgatcggcttcgccgggatca gaagtctctaaagggaagaggcagcactc	+	+
CY044377	Human H1N1 IAVs	Human	H1N1		2008	USA	A/Boston/26/2008	ggcgatgcccccctttcttgatcggcttcgcagagatcag aagtctctaaagggaagaggcagcactc	+	+
CY044536	Human H1N1 IAVs	Human	H1N1		2008	USA	A/Boston/27/2008	ggcgatgcccccctttcttgatcggcttcgccgagatcag aagtctctaaagggaagaggcagcactc	-	-
CY044568	Human H1N1 IAVs	Human	H1N1		2008	USA	A/Boston/35/2008	ggcgatgcccccctttcttgatcggcttcgccgagatcag aagtctctaaagggaagaggcagcactc	-	-
CY043421	Human H1N1 IAVs	Human	H1N1		2008	Japan	A/Gunma/07G006/2008	ggcgatgcccccctttcttgatcggcttcgccgagatcag aagtctctaaagggaagaggcagcactc	-	-
CY043477	Human H1N1 IAVs	Human	H1N1		2008	Japan	A/Nagasaki/07N011/2008	ggcgatgcccccctttcttgatcggcttcgccgagatcag aagtctctaaagggaagaggcagcactc	-	-
CY172979	Human H1N1 IAVs	Human	H1N1		2008	USA	A/New_York/1113/2008	ggcgatgcccccctttcttgatcggcttcgccgagatcag aagtctctaaagggaagaggcagcactc	-	-
CY172987	Human H1N1 IAVs	Human	H1N1		2008	USA	A/New_York/1114/2008	ggcgatgcccccctttcttgatcggcttcgccgagatcag aagtctctaaagggaagaggcagcactc	-	-

CY172955	Human H1N1 IAVs	Human	H1N1		2008	USA	A/New_York/1110/2008	ggcgatgccccctttcttgatcggcttcgccgagatcag aagtctctaaagggaagaggcagcactc	-	-
CY043461	Human H1N1 IAVs	Human	H1N1		2008	Japan	A/Kyoto/07K454/2008	ggcgatgccccctttcttgatcggcttcgccgagatcag aagtctctaaagggaagaggcagcactc	-	-
CY038766	Human H1N1 IAVs	Human	H1N1		2008	USA	A/District_of_Columbia/WRAMC_ 1154047/2008	ggcgatgccccctttcttgatcggcttcgccgagatcag aagtctctaaagggaagaggcagcactc	-	-
GQ396618	Human H1N1 IAVs	Human	H1N1		2008	Myanmar	A/Naypyitaw/M499/2008	ggcgatgcccctttccttgatcggcttcgccgagatcag aagtctctaaagggaagaggcagcactc	+	+
GQ396626	Human H1N1 IAVs	Human	H1N1		2008	Myanmar	A/Naypyitaw/M721/2008	ggcgatgcccctttccttgatcggcttcgccgagatcag aagtctctaaagggaagaggcagcactc	+	+
CY104922	Human H1N1 IAVs	Human	H1N1		2008	Viet_Nam	A/DaNang/DN431/2008	ggcgattccccttccttgatcggcttcgccgagatcag aagtctctaaagggaagaggcagcactc	+	+
HQ853523	Human H1N1 IAVs	Human	H1N1		2009	India	A/KOL/2449/2009	ggcgatgccccttccttgatcgacttcgccgagatca gaagtctctaaagggaagaggcagcactc	+	+
CY050552	Human H1N1 IAVs	Human	H1N1		2009	USA	A/New_York/3095/2009	ggcgatgccccttccttgatcgacttcgccgagatca gaagtctctaaagggaagaggcagcactc	+	+
HQ853524	Human H1N1 IAVs	Human	H1N1		2009	India	A/KOL/2465/2009	ggcgatgccccttccttgatcgacttcgccgagatca gaagtctctaaagggaagaggcagcactc	+	+
CY074615	Human H1N1 IAVs	Human	H1N1		2009	USA	A/California/VRDL366/2009	ggcgatgccccttccttgatcggctccgccgagatca gaagtctctaaagggaagaggcagcactc	-	*
CY080821	Human H1N1 IAVs	Human	H1N1		2009	USA	A/Boston/45/2009	ggcgatgccccttccttgatcggctccgccgagatca gaagtctctaaagggaagaggcagcactc	-	*
GU183803	Human H1N1 IAVs	Human	H1N1		2009	Thailand	A/Thailand/CU_B589/2009	ggcgatgccccttccttgatcggcttcgccgacatcag aagtctctaaagggaagaggcagcactc	+	+
CY173315	Human H1N1 IAVs	Human	H1N1		2009	USA	A/New_York/1159/2009	ggcgatgccccttccttgatcggcttcgccgagatca gaagtccctaaagggaagaggcagcactc	+	+
CY173339	Human H1N1 IAVs	Human	H1N1		2009	USA	A/New_York/1162/2009	ggcgatgccccttccttgatcggcttcgccgagatca gaagtccctaaagggaagaggcagcactc	+	+
CY100848	Human H1N1 IAVs	Human	H1N1		2009	USA	A/Alabama/WRAIR1235P/2009	ggcgatgccccttccttgatcggcttcgccgagatca gaagtctctaaagagaagaggcagcactc	+	+
CY074295	Human H1N1 IAVs	Human	H1N1		2009	USA	A/California/VRDL236/2009	ggcgatgccccttccttgatcggcttcgccgagatca gaagtctctaaagggaaaaggcaacactc	+	+
CY074167	Human H1N1 IAVs	Human	H1N1		2009	USA	A/California/VRDL213/2009	ggcgatgccccttccttgatcggcttcgccgagatca gaagtctctaaagggaagaggcaacactc	+	+
GQ850599	Human H1N1 IAVs	Human	H1N1		2009	China	A/Shanghai/LWS1/2009	ggcgatgccccttccttgatcggcttcgccgagatca gaagtctctaaagggaagaggcaacactc	+	+
CY069545	Human H1N1 IAVs	Human	H1N1		2009	USA	A/Arkansas/WRAIR1249P/2009	ggcgatgccccttccttgatcggcttcgccgagatca gaagtctctaaagggaagaggcagcactc	+	+
CY073841	Human H1N1 IAVs	Human	H1N1		2009	USA	A/California/VRDL163/2009	ggcgatgccccttccttgatcggcttcgccgagatca gaagtctctaaagggaagaggcagcactc	+	+
CY074639	Human H1N1 IAVs	Human	H1N1		2009	USA	A/California/VRDL377/2009	ggcgatgccccttccttgatcggcttcgccgagatca gaagtctctaaagggaagaggcagcactc	+	+
CY074647	Human H1N1 IAVs	Human	H1N1		2009	USA	A/California/VRDL378/2009	ggcgatgccccttccttgatcggcttcgccgagatca gaagtctctaaagggaagaggcagcactc	+	+
KJ130176	Human H1N1 IAVs	Human	H1N1		2009	New_Zealand	A/New_Zealand/1212a/2009	ggcgatgccccttccttgatcggcttcgccgagatca gaagtctctaaagggaagaggcagcactc	+	+
KJ130168	Human H1N1 IAVs	Human	H1N1		2009	New_Zealand	A/New_Zealand/1212b/2009	ggcgatgccccttccttgatcggcttcgccgagatca gaagtctctaaagggaagaggcagcactc	+	+
CY069553	Human H1N1 IAVs	Human	H1N1		2009	USA	A/Texas/WRAIR1254P/2009	ggcgatgccccttccttgatcggcttcgccgagatca gaagtctctaaagggaagaggcagcactc	+	+

CY147815	Human H1N1 IAVs	Human	H1N1		2009	Mexico	A/Mexico/24042/2009	ggcgatgcccccttccttgatcggcttcgccgagatca gaagtctctaaagggaagaggcagcactc	+	+
CY147599	Human H1N1 IAVs	Human	H1N1		2009	Mexico	A/Mexico/24005/2009	ggcgatgcccccttccttgatcggcttcgccgagatca gaagtctctaaagggaagaggcagcactc	+	+
CY147687	Human H1N1 IAVs	Human	H1N1		2009	Mexico	A/Mexico/24019/2009	ggcgatgcccccttccttgatcggcttcgccgagatca gaagtctctaaagggaagaggcagcactc	+	+
CY074487	Human H1N1 IAVs	Human	H1N1		2009	USA	A/California/VRDL292/2009	ggcgatgcccccttccttgatcggcttcgccgagatca gaagtctctaaagggaagaggcagcactc	+	+
CY070899	Human H1N1 IAVs	Human	H1N1		2009	USA	A/California/VRDL149/2009	ggcgatgcccccttccttgatcggcttcgccgagatca gaagtctctaaagggaagaggcagcactc	+	+
CY074191	Human H1N1 IAVs	Human	H1N1		2009	USA	A/California/VRDL218/2009	ggcgatgcccccttccttgatcggcttcgccgagatca gaagtctctaaagggaagaggcagcactc	+	+
CY074199	Human H1N1 IAVs	Human	H1N1		2009	USA	A/California/VRDL219/2009	ggcgatgcccccttccttgatcggcttcgccgagatca gaagtctctaaagggaagaggcagcactc	+	+
CY074495	Human H1N1 IAVs	Human	H1N1		2009	USA	A/California/VRDL295/2009	ggcgatgcccccttccttgatcggcttcgccgagatca gaagtctctaaagggaagaggcagcactc	+	+
CY064835	Human H1N1 IAVs	Human	H1N1		2009	USA	A/California/VRDL140/2009	ggcgatgcccccttccttgatcggcttcgccgagatca gaagtctctaaagggaagaggcagcactc	+	+
CY173323	Human H1N1 IAVs	Human	H1N1		2009	USA	A/New_York/1160/2009	ggcgatgcccccttccttgatcggcttcgccgagatca gaagtctctaaagggaagaggcagcactc	+	+
CY147695	Human H1N1 IAVs	Human	H1N1		2009	Mexico	A/Mexico/24020/2009	ggcgatgcccccttccttgatcggcttcgccgagatca gaagtctctaaagggaagaggcagcactc	+	+
CY089713	Human H1N1 IAVs	Human	H1N1		2009	USA	A/Boston/19/2009	ggcgatgcccccttccttgatcggcttcgccgagatca gaagtctctaaagggaagaggcagcactc	+	+
CY080949	Human H1N1 IAVs	Human	H1N1		2009	USA	A/Boston/76/2009	ggcgatgcccccttccttgatcggcttcgccgagatca gaagtctctaaagggaagaggcagcactc	+	+
CY074175	Human H1N1 IAVs	Human	H1N1		2009	USA	A/California/VRDL215/2009	ggcgatgcccccttccttgatcggcttcgccgagatca gaagtctctaaagggaagaggcagcactc	+	+
CY074383	Human H1N1 IAVs	Human	H1N1		2009	USA	A/California/VRDL258/2009	ggcgatgcccccttccttgatcggcttcgccgagatca gaagtctctaaagggaagaggcagcactc	+	+
CY074655	Human H1N1 IAVs	Human	H1N1		2009	USA	A/California/VRDL379/2009	ggcgatgcccccttccttgatcggcttcgccgagatca gaagtctctaaagggaagaggcagcactc	+	+
CY173395	Human H1N1 IAVs	Human	H1N1		2009	USA	A/New_York/1171/2009	ggcgatgcccccttccttgatcggcttcgccgagatca gaagtctctaaagggaagaggcagcactc	+	+
CY173411	Human H1N1 IAVs	Human	H1N1		2009	USA	A/New_York/1173/2009	ggcgatgcccccttccttgatcggcttcgccgagatca gaagtctctaaagggaagaggcagcactc	+	+
CY173427	Human H1N1 IAVs	Human	H1N1		2009	USA	A/New_York/1175/2009	ggcgatgcccccttccttgatcggcttcgccgagatca gaagtctctaaagggaagaggcagcactc	+	+
CY173451	Human H1N1 IAVs	Human	H1N1		2009	USA	A/New_York/1179/2009	ggcgatgcccccttccttgatcggcttcgccgagatca gaagtctctaaagggaagaggcagcactc	+	+
CY173459	Human H1N1 IAVs	Human	H1N1		2009	USA	A/New_York/1180/2009	ggcgatgcccccttccttgatcggcttcgccgagatca gaagtctctaaagggaagaggcagcactc	+	+
CY173467	Human H1N1 IAVs	Human	H1N1		2009	USA	A/New_York/1181/2009	ggcgatgcccccttccttgatcggcttcgccgagatca gaagtctctaaagggaagaggcagcactc	+	+
CY080765	Human H1N1 IAVs	Human	H1N1		2009	USA	A/Boston/36/2009	ggcgatgcccccttccttgatcggcttcgccgagatca gaagtctctaaagggaagaggcagcactc	+	+
CY080853	Human H1N1 IAVs	Human	H1N1		2009	USA	A/Boston/52/2009	ggcgatgcccccttccttgatcggcttcgccgagatca gaagtctctaaagggaagaggcagcactc	+	+
CY074287	Human H1N1 IAVs	Human	H1N1		2009	USA	A/California/VRDL235/2009	ggcgatgcccccttccttgatcggcttcgccgagatca gaagtctctaaagggaagaggcagcactc	+	+

CY043525	Human H1N1 IAVs	Human	H1N1		2009	Japan	A/Gunma/08G006/2009	ggcgatgccccccttccttgatcggcttcgccgagatca gaagtctctaaagggaagaggcagcactc	+	+
CY043549	Human H1N1 IAVs	Human	H1N1		2009	Japan	A/Nagasaki/08N006/2009	ggcgatgccccccttccttgatcggcttcgccgagatca gaagtctctaaagggaagaggcagcactc	+	+
CY080630	Human H1N1 IAVs	Human	H1N1		2009	USA	A/Boston/1/2009	ggcgatgccccccttccttgatcggcttcgccgagatca gaagtctctaaagggaagaggcagcactc	+	+
CY080622	Human H1N1 IAVs	Human	H1N1		2009	USA	A/Boston/100/2009	ggcgatgccccccttccttgatcggcttcgccgagatca gaagtctctaaagggaagaggcagcactc	+	+
CY088581	Human H1N1 IAVs	Human	H1N1		2009	USA	A/Boston/2/2009	ggcgatgccccccttccttgatcggcttcgccgagatca gaagtctctaaagggaagaggcagcactc	+	+
CY080693	Human H1N1 IAVs	Human	H1N1		2009	USA	A/Boston/20/2009	ggcgatgccccccttccttgatcggcttcgccgagatca gaagtctctaaagggaagaggcagcactc	+	+
CY089055	Human H1N1 IAVs	Human	H1N1		2009	USA	A/Boston/22/2009	ggcgatgccccccttccttgatcggcttcgccgagatca gaagtctctaaagggaagaggcagcactc	+	+
CY089079	Human H1N1 IAVs	Human	H1N1		2009	USA	A/Boston/44/2009	ggcgatgccccccttccttgatcggcttcgccgagatca gaagtctctaaagggaagaggcagcactc	+	+
CY089103	Human H1N1 IAVs	Human	H1N1		2009	USA	A/Boston/54/2009	ggcgatgccccccttccttgatcggcttcgccgagatca gaagtctctaaagggaagaggcagcactc	+	+
CY080869	Human H1N1 IAVs	Human	H1N1		2009	USA	A/Boston/60/2009	ggcgatgccccccttccttgatcggcttcgccgagatca gaagtctctaaagggaagaggcagcactc	+	+
CY089119	Human H1N1 IAVs	Human	H1N1		2009	USA	A/Boston/61/2009	ggcgatgccccccttccttgatcggcttcgccgagatca gaagtctctaaagggaagaggcagcactc	+	+
CY080925	Human H1N1 IAVs	Human	H1N1		2009	USA	A/Boston/68/2009	ggcgatgccccccttccttgatcggcttcgccgagatca gaagtctctaaagggaagaggcagcactc	+	+
CY089834	Human H1N1 IAVs	Human	H1N1		2009	USA	A/Boston/79/2009	ggcgatgccccccttccttgatcggcttcgccgagatca gaagtctctaaagggaagaggcagcactc	+	+
CY080965	Human H1N1 IAVs	Human	H1N1		2009	USA	A/Boston/84/2009	ggcgatgccccccttccttgatcggcttcgccgagatca gaagtctctaaagggaagaggcagcactc	+	+
CY080997	Human H1N1 IAVs	Human	H1N1		2009	USA	A/Boston/89/2009	ggcgatgccccccttccttgatcggcttcgccgagatca gaagtctctaaagggaagaggcagcactc	+	+
CY089175	Human H1N1 IAVs	Human	H1N1		2009	USA	A/Boston/94/2009	ggcgatgccccccttccttgatcggcttcgccgagatca gaagtctctaaagggaagaggcagcactc	+	+
CY080614	Human H1N1 IAVs	Human	H1N1		2009	USA	A/Boston/95/2009	ggcgatgccccccttccttgatcggcttcgccgagatca gaagtctctaaagggaagaggcagcactc	+	+
CY074135	Human H1N1 IAVs	Human	H1N1		2009	USA	A/California/VRDL200/2009	ggcgatgccccccttccttgatcggcttcgccgagatca gaagtctctaaagggaagaggcagcactc	+	+
CY173347	Human H1N1 IAVs	Human	H1N1		2009	USA	A/New_York/1163/2009	ggcgatgccccccttccttgatcggcttcgccgagatca gaagtctctaaagggaagaggcagcactc	+	+
CY050616	Human H1N1 IAVs	Human	Mixed		2009	USA	A/New_York/3052/2009_mixed_	ggcgatgccccccttccttgatcggcttcgccgagatca gaagtctctaaagggaagaggcagcactc	+	+
CY050760	Human H1N1 IAVs	Human	H1N1		2009	USA	A/New_York/3467/2009	ggcgatgccccccttccttgatcggcttcgccgagatca gaagtctctaaagggaagaggcagcactc	+	+
CY080725	Human H1N1 IAVs	Human	H1N1		2009	USA	A/Boston/27/2009	ggcgatgccccccttccttgatcggcttcgccgagatca gaagtctctaaagggaagaggcagcactc	+	+
CY080909	Human H1N1 IAVs	Human	H1N1		2009	USA	A/Boston/66/2009	ggcgatgccccccttccttgatcggcttcgccgagatca gaagtctctaaagggaagaggcagcactc	+	+
CY080957	Human H1N1 IAVs	Human	H1N1		2009	USA	A/Boston/83/2009	ggcgatgccccccttccttgatcggcttcgccgagatca gaagtctctaaagggaagaggcagcactc	+	+
CY080646	Human H1N1 IAVs	Human	H1N1		2009	USA	A/Boston/11/2009	ggcgatgccccccttccttgatcggcttcgccgagatca gaagtctctaaagggaagaggcagcactc	+	+

CY080773	Human H1N1 IAVs	Human	H1N1		2009	USA	A/Boston/37/2009	ggcgatgccccccttccttgatcggcttcgccgagatca gaagtctctaaagggaagaggcagcactc	+	+
CY080781	Human H1N1 IAVs	Human	H1N1		2009	USA	A/Boston/38/2009	ggcgatgccccccttccttgatcggcttcgccgagatca gaagtctctaaagggaagaggcagcactc	+	+
CY080797	Human H1N1 IAVs	Human	H1N1		2009	USA	A/Boston/41/2009	ggcgatgccccccttccttgatcggcttcgccgagatca gaagtctctaaagggaagaggcagcactc	+	+
CY080829	Human H1N1 IAVs	Human	H1N1		2009	USA	A/Boston/48/2009	ggcgatgccccccttccttgatcggcttcgccgagatca gaagtctctaaagggaagaggcagcactc	+	+
CY080861	Human H1N1 IAVs	Human	H1N1		2009	USA	A/Boston/58/2009	ggcgatgccccccttccttgatcggcttcgccgagatca gaagtctctaaagggaagaggcagcactc	+	+
CY080877	Human H1N1 IAVs	Human	H1N1		2009	USA	A/Boston/62/2009	ggcgatgccccccttccttgatcggcttcgccgagatca gaagtctctaaagggaagaggcagcactc	+	+
CY080893	Human H1N1 IAVs	Human	H1N1		2009	USA	A/Boston/64/2009	ggcgatgccccccttccttgatcggcttcgccgagatca gaagtctctaaagggaagaggcagcactc	+	+
CY080901	Human H1N1 IAVs	Human	H1N1		2009	USA	A/Boston/65/2009	ggcgatgccccccttccttgatcggcttcgccgagatca gaagtctctaaagggaagaggcagcactc	+	+
CY080981	Human H1N1 IAVs	Human	H1N1		2009	USA	A/Boston/86/2009	ggcgatgccccccttccttgatcggcttcgccgagatca gaagtctctaaagggaagaggcagcactc	+	+
CY081005	Human H1N1 IAVs	Human	H1N1		2009	USA	A/Boston/91/2009	ggcgatgccccccttccttgatcggcttcgccgagatca gaagtctctaaagggaagaggcagcactc	+	+
CY073865	Human H1N1 IAVs	Human	H1N1		2009	USA	A/California/VRDL229/2009	ggcgatgccccccttccttgatcggcttcgccgagatca gaagtctctaaagggaagaggcagcactc	+	+
CY074319	Human H1N1 IAVs	Human	H1N1		2009	USA	A/California/VRDL239/2009	ggcgatgccccccttccttgatcggcttcgccgagatca gaagtctctaaagggaagaggcagcactc	+	+
CY089135	Human H1N1 IAVs	Human	H1N1		2009	USA	A/Boston/74/2009	ggcgatgccccccttccttgatcggcttcgccgagatca gaagtctctaaagggaagaggcagcactc	+	+
CY080709	Human H1N1 IAVs	Human	H1N1		2009	USA	A/Boston/24/2009	ggcgatgccccccttccttgatcggcttcgccgagatca gaagtctctaaagggaagaggcagcactc	+	+
CY080606	Human H1N1 IAVs	Human	H1N1		2009	USA	A/Boston/80/2009	ggcgatgccccccttccttgatcggcttcgccgagatca gaagtctctaaagggaagaggcagcactc	+	+
CY089785	Human H1N1 IAVs	Human	H1N1		2009	USA	A/Boston/32/2009	ggcgatgccccccttccttgatcggcttcgccgagatca gaagtctctaaagggaagaggcagcactc	+	+
CY080717	Human H1N1 IAVs	Human	H1N1		2009	USA	A/Boston/26/2009	ggcgatgccccccttccttgatcggcttcgccgagatca gaagtctctaaagggaagaggcagcactc	+	+
CY080733	Human H1N1 IAVs	Human	H1N1		2009	USA	A/Boston/28/2009	ggcgatgccccccttccttgatcggcttcgccgagatca gaagtctctaaagggaagaggcagcactc	+	+
CY089111	Human H1N1 IAVs	Human	H1N1		2009	USA	A/Boston/59/2009	ggcgatgccccccttccttgatcggcttcgccgagatca gaagtctctaaagggaagaggcagcactc	+	+
CY081013	Human H1N1 IAVs	Human	H1N1		2009	USA	A/Boston/92/2009	ggcgatgccccccttccttgatcggcttcgccgagatca gaagtctctaaagggaagaggcagcactc	+	+
CY173483	Human H1N1 IAVs	Human	H1N1		2009	USA	A/New_York/1183/2009	ggcgatgccccccttccttgatcggcttcgccgagatca gaagtctctaaagggaagaggcagcactc	+	+
CY089127	Human H1N1 IAVs	Human	H1N1		2009	USA	A/Boston/70/2009	ggcgatgccccccttccttgatcggcttcgccgagatca gaagtctctaaagggaagaggcagcactc	+	+
CY080749	Human H1N1 IAVs	Human	H1N1		2009	USA	A/Boston/33/2009	ggcgatgccccccttccttgatcggcttcgccgagatca gaagtctctaaagggaagaggcagcactc	+	+
CY080685	Human H1N1 IAVs	Human	H1N1		2009	USA	A/Boston/18/2009	ggcgatgccccccttccttgatcggcttcgccgagatca gaagtctctaaagggaagaggcagcactc	+	+
CY080805	Human H1N1 IAVs	Human	H1N1		2009	USA	A/Boston/42/2009	ggcgatgccccccttccttgatcggcttcgccgagatca gaagtctctaaagggaagaggcagcactc	+	+

CY080941	Human H1N1 IAVs	Human	H1N1		2009	USA	A/Boston/72/2009	ggcgatgcccccttccttgatcggcttcgccgagatca gaagtctctaaagggaagaggcagcactc	+	+
CY080885	Human H1N1 IAVs	Human	H1N1		2009	USA	A/Boston/63/2009	ggcgatgcccccttccttgatcggcttcgccgagatca gaagtctctaaagggaagaggcagcactc	+	+
CY173331	Human H1N1 IAVs	Human	H1N1		2009	USA	A/New_York/1161/2009	ggcgatgcccccttccttgatcggcttcgccgagatca gaagtctctaaagggaagaggcagcactc	+	+
CY173363	Human H1N1 IAVs	Human	H1N1		2009	USA	A/New_York/1165/2009	ggcgatgcccccttccttgatcggcttcgccgagatca gaagtctctaaagggaagaggcagcactc	+	+
CY173387	Human H1N1 IAVs	Human	H1N1		2009	USA	A/New_York/1170/2009	ggcgatgcccccttccttgatcggcttcgccgagatca gaagtctctaaagggaagaggcagcactc	+	+
CY173403	Human H1N1 IAVs	Human	H1N1		2009	USA	A/New_York/1172/2009	ggcgatgcccccttccttgatcggcttcgccgagatca gaagtctctaaagggaagaggcagcactc	+	+
CY173435	Human H1N1 IAVs	Human	H1N1		2009	USA	A/New_York/1176/2009	ggcgatgcccccttccttgatcggcttcgccgagatca gaagtctctaaagggaagaggcagcactc	+	+
KJ130160	Human H1N1 IAVs	Human	H1N1		2009	New_Zealand	A/New_Zealand/3958/2009	ggcgatgcccccttccttgatcggcttcgccgagatca gaagtctctaaagggaagaggcagcactc	+	+
CY093129	Human H1N1 IAVs	Human	H1N1		2009	USA	A/Boston/47/2009	ggcgatgcccccttccttgatcggcttcgccgagatca gaagtctctaaagggaagaggcagcactc	+	+
CY050664	Human H1N1 IAVs	Human	H1N1		2009	USA	A/New_York/3150/2009	ggcgatgcccccttccttgatcggcttcgccgagatca gaagtctctaaagggaagaggcagcactc	+	+
CY074343	Human H1N1 IAVs	Human	H1N1		2009	USA	A/California/VRDL249/2009	ggcgatgcccccttccttgatcggcttcgccgagatca gaagtctctaaagggaagaggcagcactc	+	+
CY147591	Human H1N1 IAVs	Human	H1N1		2009	Mexico	A/Mexico/24004/2009	ggcgatgcccccttccttgatcggcttcgccgagatca gaagtctctaaagggaagaggcagcactc	+	+
CY074479	Human H1N1 IAVs	Human	H1N1		2009	USA	A/California/VRDL290/2009	ggcgatgcccccttccttgatcggcttcgccgagatca gaagtctctaaagggaagaggcagcactc	+	+
CY074535	Human H1N1 IAVs	Human	H1N1		2009	USA	A/California/VRDL301/2009	ggcgatgcccccttccttgatcggcttcgccgagatca gaagtctctaaagggaagaggcagcactc	+	+
CY147575	Human H1N1 IAVs	Human	H1N1		2009	Mexico	A/Mexico/24001/2009	ggcgatgcccccttccttgatcggcttcgccgagatca gaagtctctaaagggaagaggcagcactc	+	+
CY147546	Human H1N1 IAVs	Human	H1N1		2009	Mexico	A/Mexico/24018/2009	ggcgatgcccccttccttgatcggcttcgccgagatca gaagtctctaaagggaagaggcagcactc	+	+
CY147959	Human H1N1 IAVs	Human	H1N1		2009	Mexico	A/Mexico/24062/2009	ggcgatgcccccttccttgatcggcttcgccgagatca gaagtctctaaagggaagaggcagcactc	+	+
CY074311	Human H1N1 IAVs	Human	H1N1		2009	USA	A/California/VRDL238/2009	ggcgatgcccccttccttgatcggcttcgccgagatca gaagtctctaaagggaagaggcagcactc	+	+
CY074359	Human H1N1 IAVs	Human	H1N1		2009	USA	A/California/VRDL252/2009	ggcgatgcccccttccttgatcggcttcgccgagatca gaagtctctaaagggaagaggcagcactc	+	+
CY050480	Human H1N1 IAVs	Human	H1N1		2009	USA	A/New_York/1692/2009	ggcgatgcccccttccttgatcggcttcgccgagatca gaagtctctaaagggaagaggcagcactc	+	+
HQ853516	Human H1N1 IAVs	Human	H1N1		2009	India	A/KOL/46/2009	ggcgatgcccccttccttgatcggcttcgccgagatca gaagtctctaaagggaagaggcagcactc	+	+
HQ853518	Human H1N1 IAVs	Human	H1N1		2009	India	A/KOL/781/2009	ggcgatgcccccttccttgatcggcttcgccgagatca gaagtctctaaagggaagaggcagcactc	+	+
HQ853519	Human H1N1 IAVs	Human	H1N1		2009	India	A/KOL/927/2009	ggcgatgcccccttccttgatcggcttcgccgagatca gaagtctctaaagggaagaggcagcactc	+	+
HQ853520	Human H1N1 IAVs	Human	H1N1		2009	India	A/KOL/2172/2009	ggcgatgcccccttccttgatcggcttcgccgagatca gaagtctctaaagggaagaggcagcactc	+	+
CY089039	Human H1N1 IAVs	Human	H1N1		2009	USA	A/Boston/6/2009	ggcgatgcccccttccttgatcggcttcgccgagatca gaagtctctaaagggaagaggcagcactc	+	+

CY043541	Human H1N1 IAVs	Human	H1N1		2009	Japan	A/Kyoto/08K056/2009	ggcgatgcccccttccttgatcggcttcgccgagatca gaagtctctaaagggaagaggcagcactc	+	+
CY100928	Human H1N1 IAVs	Human	H1N1		2009	Peru	A/Pucallpa/WRAIR1704P/2009	ggcgatgcccccttccttgatcggcttcgccgagatca gaagtctctaaagggaagaggcagcactc	+	+
HQ853525	Human H1N1 IAVs	Human	H1N1		2009	India	A/KOL/2482/2009	ggcgatgcccccttccttgatcggcttcgccgagatca gaagtctctaaagggaagaggcagcactc	+	+
CY064803	Human H1N1 IAVs	Human	H1N1		2009	USA	A/California/VRDL134/2009	ggcgatgcccccttccttgatcggcttcgccgagatca gaagtctctaaagggaagaggcagcactc	+	+
CY073849	Human H1N1 IAVs	Human	H1N1		2009	USA	A/California/VRDL167/2009	ggcgatgcccccttccttgatcggcttcgccgagatca gaagtctctaaagggaagaggcagcactc	+	+
CY074215	Human H1N1 IAVs	Human	H1N1		2009	USA	A/California/VRDL221/2009	ggcgatgcccccttccttgatcggcttcgccgagatca gaagtctctaaagggaagaggcagcactc	+	+
CY074303	Human H1N1 IAVs	Human	H1N1		2009	USA	A/California/VRDL237/2009	ggcgatgcccccttccttgatcggcttcgccgagatca gaagtctctaaagggaagaggcagcactc	+	+
CY074431	Human H1N1 IAVs	Human	H1N1		2009	USA	A/California/VRDL276/2009	ggcgatgcccccttccttgatcggcttcgccgagatca gaagtctctaaagggaagaggcagcactc	+	+
CY070907	Human H1N1 IAVs	Human	H1N1		2009	USA	A/California/VRDL150/2009	ggcgatgcccccttccttgatcggcttcgccgagatca gaagtctctaaagggaagaggcagcactc	+	+
CY074631	Human H1N1 IAVs	Human	H1N1		2009	USA	A/California/VRDL371/2009	ggcgatgcccccttccttgatcggcttcgccgagatca gaagtctctaaagggaagaggcagcactc	+	+
CY074095	Human H1N1 IAVs	Human	H1N1		2009	USA	A/California/VRDL186/2009	ggcgatgcccccttccttgatcggcttcgccgagatca gaagtctctaaagggaagaggcagcactc	+	+
CY074247	Human H1N1 IAVs	Human	H1N1		2009	USA	A/California/VRDL225/2009	ggcgatgcccccttccttgatcggcttcgccgagatca gaagtctctaaagggaagaggcagcactc	+	+
CY074255	Human H1N1 IAVs	Human	H1N1		2009	USA	A/California/VRDL226/2009	ggcgatgcccccttccttgatcggcttcgccgagatca gaagtctctaaagggaagaggcagcactc	+	+
CY074263	Human H1N1 IAVs	Human	H1N1		2009	USA	A/California/VRDL227/2009	ggcgatgcccccttccttgatcggcttcgccgagatca gaagtctctaaagggaagaggcagcactc	+	+
CY074271	Human H1N1 IAVs	Human	H1N1		2009	USA	A/California/VRDL228/2009	ggcgatgcccccttccttgatcggcttcgccgagatca gaagtctctaaagggaagaggcagcactc	+	+
CY092097	Human H1N1 IAVs	Human	H1N1		2009	USA	A/California/VRDL372/2009	ggcgatgcccccttccttgatcggcttcgccgagatca gaagtctctaaagggaagaggcagcactc	+	+
CY074607	Human H1N1 IAVs	Human	H1N1		2009	USA	A/California/VRDL365/2009	ggcgatgcccccttccttgatcggcttcgccgagatca gaagtctctaaagggaagaggcagcactc	+	+
HQ291859	Human H1N1 IAVs	Human	H1N1		2009	Taiwan	A/Taiwan/1026/2009	ggcgatgcccccttccttgatcggcttcgccgagatca gaagtctctaaagggaagaggcagcactc	+	+
CY074599	Human H1N1 IAVs	Human	H1N1		2009	USA	A/California/VRDL358/2009	ggcgatgcccccttccttgatcggcttcgccgagatca gaagtctctaaagggaagaggcagcactc	+	+
GU271954	Human H1N1 IAVs	Human	H1N1		2009	Thailand	A/Thailand/CU_B97/2009	ggcgatgcccccttccttgatcggcttcgccgagatca gaagtctctaaagggaagaggcagcactc	+	+
HQ853521	Human H1N1 IAVs	Human	H1N1		2009	India	A/KOL/2218/2009	ggcgatgcccccttccttgatcggcttcgccgagatca gaagtctctaaagggaagaggcagcactc	+	+
CY092806	Human H1N1 IAVs	Human	mixed		2009	USA	A/California/VRDL395/2009	ggcgatgcccccttccttgatcggcttcgccgagatca gaagtctctaaagggaagaggcagcactc	+	+
CY080917	Human H1N1 IAVs	Human	H1N1		2009	USA	A/Boston/67/2009	ggcgatgcccccttccttgatcggcttcgccgagatca gaagtctctaaagggaagaggcagcactc	+	+
CY118195	Human H1N1 IAVs	Human	H1N1		2009	Malaysia	A/Malaysia/2155235/2009	ggcgatgcccccttccttgatcggcttcgccgagatca gaagtctctaaagggaagaggcagcactc	+	+
CY073809	Human H1N1 IAVs	Human	H1N1		2009	USA	A/California/VRDL152/2009	ggcgatgcccccttccttgatcggcttcgccgagatca gaagtctctaaagggaagaggcagcactc	+	+

CY073833	Human H1N1 IAVs	Human	H1N1		2009	USA	A/California/VRDL155/2009	ggcgatgcccccttccttgatcggcttcgccgagatca gaagtctctaaagggaagaggcagcactc	+	+
CY074087	Human H1N1 IAVs	Human	H1N1		2009	USA	A/California/VRDL180/2009	ggcgatgcccccttccttgatcggcttcgccgagatca gaagtctctaaagggaagaggcagcactc	+	+
CY074351	Human H1N1 IAVs	Human	H1N1		2009	USA	A/California/VRDL251/2009	ggcgatgcccccttccttgatcggcttcgccgagatca gaagtctctaaagggaagaggcagcactc	+	+
CY074503	Human H1N1 IAVs	Human	H1N1		2009	USA	A/California/VRDL297/2009	ggcgatgcccccttccttgatcggcttcgccgagatca gaagtctctaaagggaagaggcagcactc	+	+
CY074559	Human H1N1 IAVs	Human	H1N1		2009	USA	A/California/VRDL324/2009	ggcgatgcccccttccttgatcggcttcgccgagatca gaagtctctaaagggaagaggcagcactc	+	+
CY073817	Human H1N1 IAVs	Human	H1N1		2009	USA	A/California/VRDL153/2009	ggcgatgcccccttccttgatcggcttcgccgagatca gaagtctctaaagggaagaggcagcactc	+	+
CY073825	Human H1N1 IAVs	Human	H1N1		2009	USA	A/California/VRDL154/2009	ggcgatgcccccttccttgatcggcttcgccgagatca gaagtctctaaagggaagaggcagcactc	+	+
CY074367	Human H1N1 IAVs	Human	H1N1		2009	USA	A/California/VRDL254/2009	ggcgatgcccccttccttgatcggcttcgccgagatca gaagtctctaaagggaagaggcagcactc	+	+
CY074447	Human H1N1 IAVs	Human	H1N1		2009	USA	A/California/VRDL281/2009	ggcgatgcccccttccttgatcggcttcgccgagatca gaagtctctaaagggaagaggcagcactc	+	+
CY074511	Human H1N1 IAVs	Human	H1N1		2009	USA	A/California/VRDL298/2009	ggcgatgcccccttccttgatcggcttcgccgagatca gaagtctctaaagggaagaggcagcactc	+	+
CY074527	Human H1N1 IAVs	Human	H1N1		2009	USA	A/California/VRDL300/2009	ggcgatgcccccttccttgatcggcttcgccgagatca gaagtctctaaagggaagaggcagcactc	+	+
CY074143	Human H1N1 IAVs	Human	H1N1		2009	USA	A/California/VRDL202/2009	ggcgatgcccccttccttgatcggcttcgccgagatca gaagtctctaaagggaagaggcagcactc	+	+
CY074223	Human H1N1 IAVs	Human	H1N1		2009	USA	A/California/VRDL222/2009	ggcgatgcccccttccttgatcggcttcgccgagatca gaagtctctaaagggaagaggcagcactc	+	+
CY074471	Human H1N1 IAVs	Human	H1N1		2009	USA	A/California/VRDL289/2009	ggcgatgcccccttccttgatcggcttcgccgagatca gaagtctctaaagggaagaggcagcactc	+	+
CY074207	Human H1N1 IAVs	Human	H1N1		2009	USA	A/California/VRDL220/2009	ggcgatgcccccttccttgatcggcttcgccgagatca gaagtctctaaagggaagaggcagcactc	+	+
CY043533	Human H1N1 IAVs	Human	H1N1		2009	Japan	A/Hokkaido/08H024/2009	ggcgatgcccccttccttgatcggcttcgccgagatca gaagtctctaaagggaagaggcagcactc	+	+
CY043509	Human H1N1 IAVs	Human	H1N1		2009	Japan	A/Niigata/08F093/2009	ggcgatgcccccttccttgatcggcttcgccgagatca gaagtctctaaagggaagaggcagcactc	+	+
GQ902837	Human H1N1 IAVs	Human	H1N1		2009	Thailand	A/Thailand/CU_H17/2009	ggcgatgcccccttccttgatcggcttcgccgagatca gaagtctctaaagggaagaggcagcactc	+	+
GU271962	Human H1N1 IAVs	Human	H1N1		2009	Thailand	A/Thailand/CU_B267/2009	ggcgatgcccccttccttgatcggcttcgccgagatca gaagtctctaaagggaagaggcagcactc	+	+
GQ902805	Human H1N1 IAVs	Human	H1N1		2009	Thailand	A/Thailand/CU_B42/2009	ggcgatgcccccttccttgatcggcttcgccgagatca gaagtctctaaagggaagaggcagcactc	+	+
CY118262	Human H1N1 IAVs	Human	H1N1		2009	Malaysia	A/Malaysia/2089302/2009	ggcgatgcccccttccttgatcggcttcgccgagatca gaagtctctaaagggaagaggcagcactc	+	+
CY050768	Human H1N1 IAVs	Human	H1N1		2009	USA	A/New_York/3315/2009	ggcgatgcccccttccttgatcggcttcgccgagatca gaagtctctaaagggaagaggcagcactc	+	+
HQ533873	Human H1N1 IAVs	Human	H1N1		2009	China	A/Tianjin/15/2009	ggcgatgcccccttccttgatcggcttcgccgagatca gaagtctctaaagggaagaggcagcactc	+	+
CY119302	Human H1N1 IAVs	Human	H1N1		2009	Malaysia	A/Malaysia/2143035/2009	ggcgatgcccccttccttgatcggcttcgccgagatca gaagtctctaaagggaagaggcagcactc	+	+
CY119326	Human H1N1 IAVs	Human	H1N1		2009	Malaysia	A/Malaysia/2156486/2009	ggcgatgcccccttccttgatcggcttcgccgagatca gaagtctctaaagggaagaggcagcactc	+	+

CY074391	Human H1N1 IAVs	Human	H1N1		2009	USA	A/California/VRDL264/2009	ggcgatgcccccttccttgatcggcttcgccgagatca<br>gaagtctctaaagggaagaggcagcactc	+	+
CY173443	Human H1N1 IAVs	Human	H1N1		2009	USA	A/New_York/1177/2009	ggcgatgcccccttccttgatcggcttcgccgagatca<br>gaagtctctaaagggaagaggcagcactc	+	+
CY173491	Human H1N1 IAVs	Human	H1N1		2009	USA	A/New_York/1184/2009	ggcgatgcccccttccttgatcggcttcgccgagatca<br>gaagtctctaaagggaagaggcagcactc	+	+
CY173507	Human H1N1 IAVs	Human	H1N1		2009	USA	A/New_York/1186/2009	ggcgatgcccccttccttgatcggcttcgccgagatca<br>gaagtctctaaagggaagaggcagcactc	+	+
CY173499	Human H1N1 IAVs	Human	H1N1		2009	USA	A/New_York/1185/2009	ggcgatgcccccttccttgatcggcttcgccgagatca<br>gaagtctctaaagggaagaggcagcactc	+	+
GU183811	Human H1N1 IAVs	Human	H1N1		2009	Thailand	A/Thailand/CU_B685/2009	ggcgatgcccccttccttgatcggcttcgccgagatca<br>gaagtctctaaagggaagaggcagcactc	+	+
CY074671	Human H1N1 IAVs	Human	H1N1		2009	USA	A/California/VRDL382/2009	ggcgatgcccccttccttgatcggcttcgccgagatca<br>gaagtctctaaagggaagaggcagcactc	+	+
CY070915	Human H1N1 IAVs	Human	H1N1		2009	USA	A/California/VRDL151/2009	ggcgatgcccccttccttgatcggcttcgccgagatca<br>gaagtctctaaagggaagaggcagcactc	+	+
CY074231	Human H1N1 IAVs	Human	H1N1		2009	USA	A/California/VRDL223/2009	ggcgatgcccccttccttgatcggcttcgccgagatca<br>gaagtctctaaagggaagaggcagcactc	+	+
CY074415	Human H1N1 IAVs	Human	H1N1		2009	USA	A/California/VRDL273/2009	ggcgatgcccccttccttgatcggcttcgccgagatca<br>gaagtctctaaagggaagaggcagcactc	+	+
CY074519	Human H1N1 IAVs	Human	H1N1		2009	USA	A/California/VRDL299/2009	ggcgatgcccccttccttgatcggcttcgccgagatca<br>gaagtctctaaagggaagaggcagcactc	+	+
CY074575	Human H1N1 IAVs	Human	H1N1		2009	USA	A/California/VRDL341/2009	ggcgatgcccccttccttgatcggcttcgccgagatca<br>gaagtctctaaagggaagaggcagcactc	+	+
CY074103	Human H1N1 IAVs	Human	H1N1		2009	USA	A/California/VRDL189/2009	ggcgatgcccccttccttgatcggcttcgccgagatca<br>gaagtctctaaagggaagaggcagcactc	+	+
CY074399	Human H1N1 IAVs	Human	H1N1		2009	USA	A/California/VRDL267/2009	ggcgatgcccccttccttgatcggcttcgccgagatca<br>gaagtctctaaagggaagaggcagcactc	+	+
CY074463	Human H1N1 IAVs	Human	H1N1		2009	USA	A/California/VRDL284/2009	ggcgatgcccccttccttgatcggcttcgccgagatca<br>gaagtctctaaagggaagaggcagcactc	+	+
CY074567	Human H1N1 IAVs	Human	H1N1		2009	USA	A/California/VRDL326/2009	ggcgatgcccccttccttgatcggcttcgccgagatca<br>gaagtctctaaagggaagaggcagcactc	+	+
CY074583	Human H1N1 IAVs	Human	H1N1		2009	USA	A/California/VRDL346/2009	ggcgatgcccccttccttgatcggcttcgccgagatca<br>gaagtctctaaagggaagaggcagcactc	+	+
CY074591	Human H1N1 IAVs	Human	H1N1		2009	USA	A/California/VRDL348/2009	ggcgatgcccccttccttgatcggcttcgccgagatca<br>gaagtctctaaagggaagaggcagcactc	+	+
CY089087	Human H1N1 IAVs	Human	H1N1		2009	USA	A/Boston/46/2009	ggcgatgcccccttccttgatcggcttcgccgagatca<br>gaagtctctaaagggaagaggcagcactc	+	+
CY080837	Human H1N1 IAVs	Human	H1N1		2009	USA	A/Boston/49/2009	ggcgatgcccccttccttgatcggcttcgccgagatca<br>gaagtctctaaagggaagaggcagcactc	+	+
CY089761	Human H1N1 IAVs	Human	H1N1		2009	USA	A/Boston/87/2009	ggcgatgcccccttccttgatcggcttcgccgagatca<br>gaagtctctaaagggaagaggcagcactc	+	+
CY080989	Human H1N1 IAVs	Human	H1N1		2009	USA	A/Boston/88/2009	ggcgatgcccccttccttgatcggcttcgccgagatca<br>gaagtctctaaagggaagaggcagcactc	+	+
CY074335	Human H1N1 IAVs	Human	H1N1		2009	USA	A/California/VRDL245/2009	ggcgatgcccccttccttgatcggcttcgccgagatca<br>gaagtctctaaagggaagaggcagcactc	+	+
CY173419	Human H1N1 IAVs	Human	H1N1		2009	USA	A/New_York/1174/2009	ggcgatgcccccttccttgatcggcttcgccgagatca<br>gaagtctctaaagggaagaggcagcactc	+	+
CY173379	Human H1N1 IAVs	Human	H1N1		2009	USA	A/New_York/1169/2009	ggcgatgcccccttccttgatcggcttcgccgagatca<br>gaagtctctaaagggaagaggcagcactc	+	+

CY173371	Human H1N1 IAVs	Human	H1N1		2009	USA	A/New_York/1168/2009	ggcgatgcccccttccttgatcggcttcgccgagatca gaagtctctaaagggaagaggcagcactc	+	+
CY173355	Human H1N1 IAVs	Human	H1N1		2009	USA	A/New_York/1164/2009	ggcgatgcccccttccttgatcggcttcgccgagatca gaagtctctaaagggaagaggcagcactc	+	+
CY064843	Human H1N1 IAVs	Human	H1N1		2009	USA	A/California/VRDL141/2009	ggcgatgcccccttccttgatcggcttcgccgagatca gaagtctctaaagggaagaggcagcactc	+	+
CY092341	Human H1N1 IAVs	Human	H1N1		2009	USA	A/California/VRDL190/2009	ggcgatgcccccttccttgatcggcttcgccgagatca gaagtctctaaagggaagaggcagcactc	+	+
CY074159	Human H1N1 IAVs	Human	H1N1		2009	USA	A/California/VRDL209/2009	ggcgatgcccccttccttgatcggcttcgccgagatca gaagtctctaaagggaagaggcagcactc	+	+
CY100792	Human H1N1 IAVs	Human	H1N1		2009	USA	A/Oklahoma/WRAIR1110P/2009	ggcgatgcccccttccttgatcggcttcgccgagatca gaagtctctaaagggaagaggcagcactc	+	+
CY173475	Human H1N1 IAVs	Human	H1N1		2009	USA	A/New_York/1182/2009	ggcgatgcccccttccttgatcggcttcgccgagatca gaagtctctaaagggaagaggcagcactc	+	+
CY074119	Human H1N1 IAVs	Human	H1N1		2009	USA	A/California/VRDL193/2009	ggcgatgcccccttccttgatcggcttcgccgagatca gaagtctctaaagggaagaggcagcactc	+	+
GU183819	Human H1N1 IAVs	Human	H1N1		2009	Thailand	A/Thailand/CU_H223/2009	ggcgatgcccccttccttgatcggcttcgccgagatca gaagtctctaaagggaagaggcagcactc	+	+
GU271970	Human H1N1 IAVs	Human	H1N1		2009	Thailand	A/Thailand/CU_H565/2009	ggcgatgcccccttccttgatcggcttcgccgagatca gaagtctctaaagggaagaggcagcactc	+	+
KJ690475	Human H1N1 IAVs	Human	H1N1		2009	Uganda	A/Uganda/MUWRP_094/2009	ggcgatgcccccttccttgatcggcttcgccgagatca gaagtctctaaagggaagaggcagcactc	+	+
CY081021	Human H1N1 IAVs	Human	H1N1		2009	USA	A/Boston/93/2009	ggcgatgcccccttccttgatcggcttcgccgagatca gaagtctctaaagggaagaggcagcactc	+	+
CY074279	Human H1N1 IAVs	Human	H1N1		2009	USA	A/California/VRDL232/2009	ggcgatgcccccttccttgatcggcttcgccgagatca gaagtctctaaagggaagaggcagcactc	+	+
CY147607	Human H1N1 IAVs	Human	H1N1		2009	Mexico	A/Mexico/24006/2009	ggcgatgcccccttccttgatcggcttcgccgagatca gaagtctctaaagggaagaggcagcactc	+	+
CY092349	Human H1N1 IAVs	Human	H1N1		2009	USA	A/California/VRDL191/2009	ggcgatgcccccttccttgatcggcttcgccgagatca gaagtctctaaagggaagaggcagcactc	+	+
CY074151	Human H1N1 IAVs	Human	H1N1		2009	USA	A/California/VRDL206/2009	ggcgatgcccccttccttgatcggcttcgccgagatca gaagtctctaaagggaagaggcagcactc	+	+
CY074439	Human H1N1 IAVs	Human	H1N1		2009	USA	A/California/VRDL280/2009	ggcgatgcccccttccttgatcggcttcgccgagatca gaagtctctaaagggaagaggcagcactc	+	+
CY074127	Human H1N1 IAVs	Human	H1N1		2009	USA	A/California/VRDL194/2009	ggcgatgcccccttccttgatcggcttcgccgagatca gaagtctctaaagggaagaggcagcactc	+	+
CY074327	Human H1N1 IAVs	Human	H1N1		2009	USA	A/California/VRDL244/2009	ggcgatgcccccttccttgatcggcttcgccgagatca gaagtctctaaagggaagaggcagcactc	+	+
CY074663	Human H1N1 IAVs	Human	H1N1		2009	USA	A/California/VRDL380/2009	ggcgatgcccccttccttgatcggcttcgccgagatca gaagtctctaaagggaagaggcagcactc	+	+
CY089047	Human H1N1 IAVs	Human	H1N1		2009	USA	A/Boston/10/2009	ggcgatgcccccttccttgatcggcttcgccgagatca gaagtctctaaagggaagaggcagcactc	+	+
CY080654	Human H1N1 IAVs	Human	H1N1		2009	USA	A/Boston/13/2009	ggcgatgcccccttccttgatcggcttcgccgagatca gaagtctctaaagggaagaggcagcactc	+	+
CY080662	Human H1N1 IAVs	Human	H1N1		2009	USA	A/Boston/14/2009	ggcgatgcccccttccttgatcggcttcgccgagatca gaagtctctaaagggaagaggcagcactc	+	+
CY080677	Human H1N1 IAVs	Human	H1N1		2009	USA	A/Boston/17/2009	ggcgatgcccccttccttgatcggcttcgccgagatca gaagtctctaaagggaagaggcagcactc	+	+
CY080789	Human H1N1 IAVs	Human	H1N1		2009	USA	A/Boston/39/2009	ggcgatgcccccttccttgatcggcttcgccgagatca gaagtctctaaagggaagaggcagcactc	+	+

CY089143	Human H1N1 IAVs	Human	H1N1		2009	USA	A/Boston/77/2009	ggcgatgcccccttccttgatcggcttcgccgagatca gaagtctctaaagggaagaggcagcactc	+	+
CY089159	Human H1N1 IAVs	Human	H1N1		2009	USA	A/Boston/81/2009	ggcgatgcccccttccttgatcggcttcgccgagatca gaagtctctaaagggaagaggcagcactc	+	+
CY080701	Human H1N1 IAVs	Human	H1N1		2009	USA	A/Boston/21/2009	ggcgatgcccccttccttgatcggcttcgccgagatca gaagtctctaaagggaagaggcagcactc	+	+
CY089071	Human H1N1 IAVs	Human	H1N1		2009	USA	A/Boston/25/2009	ggcgatgcccccttccttgatcggcttcgccgagatca gaagtctctaaagggaagaggcagcactc	+	+
CY080598	Human H1N1 IAVs	Human	H1N1		2009	USA	A/Boston/3/2009	ggcgatgcccccttccttgatcggcttcgccgagatca gaagtctctaaagggaagaggcagcactc	+	+
CY089095	Human H1N1 IAVs	Human	H1N1		2009	USA	A/Boston/50/2009	ggcgatgcccccttccttgatcggcttcgccgagatca gaagtctctaaagggaagaggcagcactc	+	+
CY089151	Human H1N1 IAVs	Human	H1N1		2009	USA	A/Boston/78/2009	ggcgatgcccccttccttgatcggcttcgccgagatca gaagtctctaaagggaagaggcagcactc	+	+
CY080973	Human H1N1 IAVs	Human	H1N1		2009	USA	A/Boston/85/2009	ggcgatgcccccttccttgatcggcttcgccgagatca gaagtctctaaagggaagaggcagcactc	+	+
CY080741	Human H1N1 IAVs	Human	H1N1		2009	USA	A/Boston/30/2009	ggcgatgcccccttccttgatcggcttcgccgagatca gaagtctctaaagggaagaggcagcactc	+	+
CY080757	Human H1N1 IAVs	Human	H1N1		2009	USA	A/Boston/35/2009	ggcgatgcccccttccttgatcggcttcgccgagatca gaagtctctaaagggaagaggcagcactc	+	+
CY080813	Human H1N1 IAVs	Human	H1N1		2009	USA	A/Boston/43/2009	ggcgatgcccccttccttgatcggcttcgccgagatca gaagtctctaaagggaagaggcagcactc	+	+
CY080933	Human H1N1 IAVs	Human	H1N1		2009	USA	A/Boston/71/2009	ggcgatgcccccttccttgatcggcttcgccgagatca gaagtctctaaagggaagaggcagcactc	+	+
CY089167	Human H1N1 IAVs	Human	H1N1		2009	USA	A/Boston/82/2009	ggcgatgcccccttccttgatcggcttcgccgagatca gaagtctctaaagggaagaggcagcactc	+	+
CY089063	Human H1N1 IAVs	Human	H1N1		2009	USA	A/Boston/23/2009	ggcgatgcccccttccttgatcggcttcgccgagatca gaagtctctaaagggaagaggcagtactc	+	+
CY080845	Human H1N1 IAVs	Human	H1N1		2009	USA	A/Boston/51/2009	ggcgatgcccccttccttgatcggcttcgccgagatca gaagtctctaaagggaagaggcagtactc	+	+
CY064875	Human H1N1 IAVs	Human	H1N1		2009	USA	A/California/VRDL146/2009	ggcgatgcccccttccttgatcggcttcgccgagatca gaagtctctaaagggaagaggcagtactc	+	+
CY100864	Human H1N1 IAVs	Human	H1N1		2009	South_Korea	A/South_Korea/WRAIR1238P/2009	ggcgatgcccccttccttgatcggcttcgccgagatca gaagtctctaaagggaagaggcagtactc	+	+
CY074423	Human H1N1 IAVs	Human	H1N1		2009	USA	A/California/VRDL275/2009	ggcgatgcccccttccttgatcggcttcgccgagatca gaagtctctaaagggaagaggcagtactc	+	+
CY064811	Human H1N1 IAVs	Human	H1N1		2009	USA	A/California/VRDL135/2009	ggcgatgcccccttccttgatcggcttcgccgagatca gaagtctctaaagggaagaggcagtactc	+	+
CY074111	Human H1N1 IAVs	Human	H1N1		2009	USA	A/California/VRDL192/2009	ggcgatgcccccttccttgatcggcttcgccgagatca gaagtctctaaagggaagaggcagtactc	+	+
CY074239	Human H1N1 IAVs	Human	H1N1		2009	USA	A/California/VRDL224/2009	ggcgatgcccccttccttgatcggcttcgccgagatca gaagtctctaaagggaagaggcagtactc	+	+
CY074543	Human H1N1 IAVs	Human	H1N1		2009	USA	A/California/VRDL311/2009	ggcgatgcccccttccttgatcggcttcgccgagatca gaagtctctaaagggaagaggcagtactc	+	+
CY074551	Human H1N1 IAVs	Human	H1N1		2009	USA	A/California/VRDL320/2009	ggcgatgcccccttccttgatcggcttcgccgagatca gaagtctctaaagggaagaggcagtactc	+	+
CY074623	Human H1N1 IAVs	Human	H1N1		2009	USA	A/California/VRDL369/2009	ggcgatgcccccttccttgatcggcttcgccgagatca gaagtctctaaagggaagaggcagtactc	+	+
CY043517	Human H1N1 IAVs	Human	H1N1		2009	Japan	A/Niigata/08F188/2009	ggcgatgcccccttccttgatcggcttcgccgagatca gaagtctctaaagggaagaggcagtactc	+	+

CY092763	Human H1N1 IAVs	Human	mixed		2009	USA	A/California/VRDL179/2009	ggcgatgccccccttccttgatcggcttcgccgagatca gaagtctctaaagggaagaggcagtactc	+	+
CY074455	Human H1N1 IAVs	Human	H1N1		2009	USA	A/California/VRDL283/2009	ggcgatgccccccttccttgatcggcttcgccgagatca gaagtctctaaagggaagaggcagtactc	+	+
CY043501	Human H1N1 IAVs	Human	H1N1		2009	Japan	A/Niigata/08F031/2009	ggcgatgccccccttccttgatcggcttcgccgagatca gaagtctctaaagggaagaggcagtactc	+	+
CY074183	Human H1N1 IAVs	Human	H1N1		2009	USA	A/California/VRDL217/2009	ggcgatgccccccttccttgatcggcttcgccgagatca gaagtctctaaagggaaggggcagcactc	+	+
CY080638	Human H1N1 IAVs	Human	H1N1		2009	USA	A/Boston/4/2009	ggcgatgcccccctttcttgatcggcttcgccgagatcag aagtctctaaagggaagaggcagcactc	-	-
CY074375	Human H1N1 IAVs	Human	H1N1		2009	USA	A/California/VRDL256/2009	ggcgatgcccccctttcttgatcggcttcgccgagatcag aagtctctaaagggaagaggcagcactc	-	-
CY073857	Human H1N1 IAVs	Human	H1N1		2009	USA	A/California/VRDL175/2009	ggcgatgctcccttccttgatcggcttcgccgagatcag aagtctctaaagggaagaggcagcactc	-	-
CY074407	Human H1N1 IAVs	Human	H1N1		2009	USA	A/California/VRDL270/2009	ggcgatgctcccttccttgatcggcttcgccgagatcag aagtctctaaagggaagaggcagcactc	-	-
HQ853522	Human H1N1 IAVs	Human	H1N1		2009	India	A/KOL/2086/2009	ggtgatgccccattccttgatcggcttcgccgagatcag aaatccctaagaggaaggggcagcactc	-	*
HQ853517	Human H1N1 IAVs	Human	H1N1		2009	India	A/KOL/230/2009	ggtgatgccccattccttgatcggcttcgccgagatcag aaatccctaagaggaaggggcagcactc	-	*
CY146805	Human H1N1 IAVs	Human	H3N2		NA	USA	A/Texas/JY2/unknown	ggcgatgccccattccttgatcggcttcgccgagatca gaaatccctaagaggaaggggcagtactc	-	-
DQ508845	Human H2N2 IAVs	Human	H2N2		1957	Japan	A/Japan/305/57	ggtgatgccccattccttgatcggcttcgccgagatcag aagtccctaaggagaagaggcagtactc	+	+
CY125874	Human H2N2 IAVs	Human	H2N2		1957	Japan	A/ITS/1/1957	ggtgatgccccattccttgatcggcttcgccgagatcag aagtccctaaggggaagaggcagcactc	+	+
CY032273	Human H2N2 IAVs	Human	H2N2		1957	China	A/Guiyang/1/1957	ggtgatgccccattccttgatcggcttcgccgagatcag aagtccctaaggggaagaggcagcactc	+	+
CY034064	Human H2N2 IAVs	Human	H2N2		1957	Singapore	A/Singapore/1_MA12A/1957	ggtgatgccccattccttgatcggcttcgccgagatcag aagtccctaaggggaagaggcagcactc	+	+
CY087796	Human H2N2 IAVs	Human	H2N2		1957	Singapore	A/Singapore/1_MA12E/1957	ggtgatgccccattccttgatcggcttcgccgagatcag aagtccctaaggggaagaggcagcactc	+	+
CY034048	Human H2N2 IAVs	Human	H2N2		1957	Singapore	A/Singapore/1/1957	ggtgatgccccattccttgatcggcttcgccgagatcag aagtccctaaggggaagaggcagcactc	+	+
CY034072	Human H2N2 IAVs	Human	H2N2		1957	Singapore	A/Singapore/1_MA12B/1957	ggtgatgccccattccttgatcggcttcgccgagatcag aagtccctaaggggaagaggcagcactc	+	+
CY034056	Human H2N2 IAVs	Human	H2N2		1957	Singapore	A/Singapore/1_MA12/1957	ggtgatgccccattccttgatcggcttcgccgagatcag aagtccctaaggggaagaggcagcactc	+	+
CY034080	Human H2N2 IAVs	Human	H2N2		1957	Singapore	A/Singapore/1_MA12D/1957	ggtgatgccccattccttgatcggcttcgccgagatcag aagtccctaaggggaagaggcagcactc	+	+
AY210151	Human H2N2 IAVs	Human	H2N2		1957	Singapore	A/Singapore/1/57	ggtgatgccccattccttgatcggcttcgccgagatcag aagtccctaaggggaagaggcagcactc	+	+
CY125898	Human H2N2 IAVs	Human	H2N2		1957	Singapore	A/Singapore/1/1957	ggtgatgccccattccttgatcggcttcgccgagatcag aagtccctaaggggaagaggcagcactc	+	+
AX399731	Human H2N2 IAVs	Human	H2N2		1957	Singapore	A/Singapore/1/57	ggtgatgccccattccttgatcggcttcgccgagatcag aagtccctaaggggaagaggcagcactc	+	+
AY210153	Human H2N2 IAVs	Human	H2N2		1957	USA	A/Davis/1/57	ggtgatgccccattccttgatcggcttcgccgagatcag aagtccctaaggggaagaggcagtaccc	+	+
AY210152	Human H2N2 IAVs	Human	H2N2		1957	USA	A/Albany/7/57	ggtgatgccccattccttgatcggcttcgccgagatcag aagtccctaaggggaagaggcagtaccc	+	+

AY210154	Human H2N2 IAVs	Human	H2N2		1957	Chile	A/Chile/13/57	ggtgatgccccattccttgatcggcttcgccgagatcag<br>aagtccctaaggggaagaggcagtaccc	+	+
CY022017	Human H2N2 IAVs	Human	H2N2		1957	USA	A/Albany/20/1957	ggtgatgccccattccttgatcggcttcgccgagatcag<br>aagtccctaaggggaagaggcagtactc	+	+
CY096790	Human H2N2 IAVs	Human	mixed		1957	USA	A/Albany/21/1957_mixed_	ggtgatgccccattccttgatcggcttcgccgagatcag<br>aagtccctaaggggaagaggcagtactc	+	+
CY021809	Human H2N2 IAVs	Human	H2N2		1957	USA	A/Albany/22/1957	ggtgatgccccattccttgatcggcttcgccgagatcag<br>aagtccctaaggggaagaggcagtactc	+	+
CY020385	Human H2N2 IAVs	Human	H2N2		1957	USA	A/Albany/26/1957	ggtgatgccccattccttgatcggcttcgccgagatcag<br>aagtccctaaggggaagaggcagtactc	+	+
CY125834	Human H2N2 IAVs	Human	H2N2		1957	USA	A/Rockville_Illinois/5/1957	ggtgatgccccattccttgatcggcttcgccgagatcag<br>aagtccctaaggggaagaggcagtactc	+	+
CY077901	Human H2N2 IAVs	Human	H2N2		1957	Netherlands	A/Rotterdam/1957	ggtgatgccccattccttgatcggcttcgccgagatcag<br>aagtccctaaggggaagaggcagtactc	+	+
CY125914	Human H2N2 IAVs	Human	H2N2		1957	USA	A/Ann_Arbor/23/1957	ggtgatgccccattccttgatcggcttcgccgagatcag<br>aagtccctaaggggaagaggcagtactc	+	+
CY031583	Human H2N2 IAVs	Human	H2N2		1957	USA	A/Ann_Arbor/23/1957	ggtgatgccccattccttgatcggcttcgccgagatcag<br>aagtccctaaggggaagaggcagtactc	+	+
CY087804	Human H2N2 IAVs	Human	H2N2		1957	Japan	A/Japan/305_MA12/1957	ggtgatgccccattccttgatcggcttcgccgagatcag<br>aagtccctaaggggaagaggcagtactc	+	+
CY045808	Human H2N2 IAVs	Human	H2N2		1957	Japan	A/Japan/305/1957	ggtgatgccccattccttgatcggcttcgccgagatcag<br>aagtccctaaggggaagaggcagtactc	+	+
CY044329	Human H2N2 IAVs	Human	H2N2		1957	Japan	A/Japan/305/1957	ggtgatgccccattccttgatcggcttcgccgagatcag<br>aagtccctaaggggaagaggcagtactc	+	+
CY125882	Human H2N2 IAVs	Human	H2N2		1957	USA	A/Rockville_Illinois/5_CA/1957	ggtgatgccccattccttgatcggcttcgccgagatcag<br>aagtccctaaggggaagaggcagtactc	+	+
M81578	Human H2N2 IAVs	Human	H2N2		1957	Russia	A/Leningrad/134/17/1957	ggtgatgccccattccttgatcggcttcgccgagatcag<br>aagtccctaaggggaagaggcagtactc	+	+
M81584	Human H2N2 IAVs	Human	H2N2		1957	Russia	A/Leningrad/134/47/1957	ggtgatgccccattccttgatcggcttcgccgagatcag<br>aagtccctaaggggaagaggcagtactc	+	+
M81572	Human H2N2 IAVs	Human	H2N2		1957	Russia	A/Leningrad/134/1957	ggtgatgccccattccttgatcggcttcgccgagatcag<br>aagtccctaaggggaagaggcagtactc	+	+
HM204778	Human H2N2 IAVs	Human	H2N2		1957	El_Salvador	A/El_Salvador/2_Q226L/1957	ggtgatgccccattccttgatcggcttcgccgagatcag<br>aagtccctaaggggaagaggcagtactc	+	+
AY210155	Human H2N2 IAVs	Human	H2N2		1957	El_Salvador	A/ElSalvador/2/57	ggtgatgccccattccttgatcggcttcgccgagatcag<br>aagtccctaaggggaagaggcagtactc	+	+
HM204771	Human H2N2 IAVs	Human	H2N2		1957	El_Salvador	A/El_Salvador/2/1957	ggtgatgccccattccttgatcggcttcgccgagatcag<br>aagtccctaaggggaagaggcagtactc	+	+
CY021817	Human H2N2 IAVs	Human	H2N2		1958	USA	A/Albany/1/1958	ggtgatgccccattccttgatcggcttcgccgagatcag<br>aagtccctaaggggaagaggcagtactc	+	+
CY020321	Human H2N2 IAVs	Human	H2N2		1958	USA	A/Albany/2/1958	ggtgatgccccattccttgatcggcttcgccgagatcag<br>aagtccctaaggggaagaggcagtactc	+	+
CY020377	Human H2N2 IAVs	Human	H2N2		1958	USA	A/Albany/4/1958	ggtgatgccccattccttgatcggcttcgccgagatcag<br>aagtccctaaggggaagaggcagtactc	+	+
CY021017	Human H2N2 IAVs	Human	H2N2		1958	USA	A/Albany/5/1958	ggtgatgccccattccttgatcggcttcgccgagatcag<br>aagtccctaaggggaagaggcagtactc	+	+
AY210157	Human H2N2 IAVs	Human	H2N2		1958	USA	A/Albany/6/58	ggtgatgccccattccttgatcggcttcgccgagatcag<br>aagtccctaaggggaagaggcagtactc	+	+
CY021073	Human H2N2 IAVs	Human	H2N2		1958	USA	A/Albany/3/1958	ggtgatgccccattccttgatcggcttcgccgagatcag<br>aagtccctaaggggaagaggcagtactc	+	+

CY021793	Human H2N2 IAVs	Human	H2N2		1958	USA	A/Albany/24/1958	ggtgatgccccattccttgatcggcttcgccgagatcag<br>aagtccctaaggggaagaggcagtactc	+	+
CY077744	Human H2N2 IAVs	Human	H2N2		1958	Netherlands	A/Netherlands/001M/1958	ggtgatgccccattccttgatcggcttcgccgagatcag<br>aagtccctaaggggaagaggcagtactc	+	+
AY210156	Human H2N2 IAVs	Human	H2N2		1958	Malaysia	A/Malaya/16/58	ggtgatgccccattccttgatcggcttcgccgagatcag<br>aagtccctaaggggaagaggcagtactc	+	+
CY032257	Human H2N2 IAVs	Human	H2N2		1959	Russia	A/Krasnodar/101/1959	ggtgatgccccattcctcgatcggcttcgccgagatca<br>gaagtccctaaggggaagaggcagcactc	+	+
JN596877	Human H2N2 IAVs	Human	H2N2		1959	Russia	A/Krasnodar/101/1959	ggtgatgccccattccttgatcggcttcgccgagatcag<br>aagtccctaaggggaagaggcagcactc	+	+
CY020545	Human H2N2 IAVs	Human	H2N2		1959	USA	A/Albany/1/1959	ggtgatgccccattccttgatcggcttcgccgagatcag<br>aagtccctaaggggaagaggcagtactc	+	+
AY210158	Human H2N2 IAVs	Human	H2N2		1959	Australia	A/Victoria/15681/59	ggtgatgccccattccttgatcggcttcgccgagatcag<br>aagtccctaaggggaagaggcagtactc	+	+
CY077908	Human H2N2 IAVs	Human	H2N2		1959	Netherlands	A/Nijmegen/1959	ggtgatgccccattccttgatcggcttcgccgagatcag<br>aagtccctaaggggaagaggcagtactc	+	+
AY210159	Human H2N2 IAVs	Human	H2N2		1959	Brazil	A/SaoPaolo/3/59	ggtgatgccccattccttgatcggcttcgccgagatcag<br>aagtccctaaggggaagaggcagtactc	+	+
CY021937	Human H2N2 IAVs	Human	H2N2		1960	USA	A/Albany/1/1960	ggtgatgccccattccttgatcggcttcgccgagatcag<br>aagtccctaaggggaagaggcagtactc	+	+
M23968	Human H2N2 IAVs	Human	H2N2		1960	USA	A/Ann_Arbor/6/1960	ggtgatgccccattccttgatcggcttcgccgagatcag<br>aagtccctaaggggaagaggcagtactc	+	+
CY125906	Human H2N2 IAVs	Human	H2N2		1960	USA	A/Ann_Arbor/6/1960	ggtgatgccccattccttgatcggcttcgccgagatcag<br>aagtccctaaggggaagaggcagtactc	+	+
AY210161	Human H2N2 IAVs	Human	H2N2		1960	USA	A/Ann_Arbor/6/60	ggtgatgccccattccttgatcggcttcgccgagatcag<br>aagtccctaaggggaagaggcagtactc	+	+
AY210160	Human H2N2 IAVs	Human	H2N2		1960	Philippines	A/Philippines/2/60	ggtgatgccccattccttgatcggcttcgccgagatcag<br>aagtccctaaggggaagaggcagtactc	+	+
CY077790	Human H2N2 IAVs	Human	H2N2		1960	Netherlands	A/Netherlands/056H1/1960	ggtgatgccccattccttgatcggcttcgccgagatcag<br>aagtccctaaggggaagaggcagtactc	+	+
CY125858	Human H2N2 IAVs	Human	H2N2		1961	United_Kingdom	A/England/1/1961	ggtgatgccccattccttgatcggcttcgccgagatcag<br>aaatccctaaggggaagaggcagcactc	-	-
AY210163	Human H2N2 IAVs	Human	H2N2		1961	United_Kingdom	A/England/1/61	ggtgatgccccattccttgatcggcttcgccgagatcag<br>aagtccctaaggggaagaggcagtactc	+	+
AY210162	Human H2N2 IAVs	Human	H2N2		1961	Panama	A/Panama/1/61	ggtgatgccccattccttgatcggcttcgccgagatcag<br>aagtccctaaggggaagaggcagtactc	+	+
AY210166	Human H2N2 IAVs	Human	H2N2		1962	Taiwan	A/Taiwan/1/62	ggtgatgccccattccttgatcggctccgccgagatca<br>gaagtccctaaggggaagaggcagtactc	-	-
AY210167	Human H2N2 IAVs	Human	H2N2		1962	Japan	A/Yokosuka/3/62	ggtgatgccccattccttgatcggctccgccgagatca<br>gaagtccctaaggggaagaggcagtactc	-	-
AY210164	Human H2N2 IAVs	Human	H2N2		1962	Japan	A/Japan/170/62	ggtgatgccccattccttgatcggcttcgccgagatcag<br>aagtccctaaggggaagaggcagtactc	+	+
AY210165	Human H2N2 IAVs	Human	H2N2		1962	Netherlands	A/Netherlands/60/62	ggtgatgccccattccttgatcggcttcgccgagatcag<br>aagtccctaaggggaagaggcagtactc	+	+
AY210169	Human H2N2 IAVs	Human	H2N2		1963	USA	A/Albany/1/63	ggtgatgccccattccttgatcggcttcgccgagatcag<br>aagtccctaaggggaagaggcagcactc	+	+
AY210170	Human H2N2 IAVs	Human	H2N2		1963	USA	A/Georgia/1/63	ggtgatgccccattccttgatcggcttcgccgagatcag<br>aagtccctaaggggaagaggcagcactc	+	+
CY045792	Human H2N2 IAVs	Human	H2N2		1963	USA	A/North_Carolina/1/1963	ggtgatgccccattccttgatcggcttcgccgagatcag<br>aagtccctaaggggaagaggcagcactc	+	+

CY125890	Human H2N2 IAVs	Human	H2N2		1963	Netherlands	A/Ned/65/1963	ggtgatgccccattccttgatcggcttcgccgagatcag aagtccctaaggggaagaggcagcactc	+	+
CY077737	Human H2N2 IAVs	Human	H2N2		1963	Netherlands	A/Netherlands/001K1/1963	ggtgatgccccattccttgatcggcttcgccgagatcag aagtccctaaggggaagaggcagcactc	+	+
CY035082	Human H2N2 IAVs	Human	H2N2		1963	Netherlands	A/Netherlands/56/1963	ggtgatgccccattccttgatcggcttcgccgagatcag aagtccctaaggggaagaggcagcactc	+	+
AY210168	Human H2N2 IAVs	Human	H2N2		1963	Netherlands	A/Netherlands/65/63	ggtgatgccccattccttgatcggcttcgccgagatcag aagtccctaaggggaagaggcagcactc	+	+
CY032241	Human H2N2 IAVs	Human	H2N2		1964	Germany	A/Berlin/3/1964	ggtgatgccccattccttgatcggcttcgccgagatcag aagtccctaaggggaagaggcagcactc	+	+
AY210171	Human H2N2 IAVs	Human	H2N2		1964	Japan	A/Murakami/4/64	ggtgatgccccattccttgatcggcttcgccgagatcag aagtccctaaggggaagaggcagcactc	+	+
CY032265	Human H2N2 IAVs	Human	H2N2		1964	Germany	A/Cottbus/1/1964	ggtgatgccccattccttgatcggcttcgccgagatcag aagtccctaaggggaagaggcagcactc	+	+
AY210173	Human H2N2 IAVs	Human	H2N2		1964	United_Kingdom	A/England/12/64	ggtgatgccccattccttgatcggcttcgccgagatcag aagtccctaaggggaagaggcagcactc	+	+
AY210172	Human H2N2 IAVs	Human	H2N2		1964	Taiwan	A/Taiwan/1/64	ggtgatgccccattccttgatcggcttcgccgagatcag aagtccctaaggggaataggcagtactc	+	+
DQ508885	Human H2N2 IAVs	Human	H2N2		1964	Taiwan	A/Taiwan/1964	ggtgatgccccattccttgatcggcttcgccgagatcag aagtccctaatgggaagaggcagcactc	-	-
AY210176	Human H2N2 IAVs	Human	H2N2		1965	USA	A/Albany/1/65	ggtgatgccccattccttgatcggcttcgccgagatcag aagtccctaaggggaagaggcagcactc	+	+
AY210174	Human H2N2 IAVs	Human	H2N2		1965	USA	A/NewJersey/3/65	ggtgatgccccattccttgatcggcttcgccgagatcag aagtccctaaggggaagaggcagcactc	+	+
CY032249	Human H2N2 IAVs	Human	H2N2		1965	Germany	A/Potsdam/2/1965	ggtgatgccccattccttgatcggcttcgccgagatcag aagtccctaaggggaagaggcagcactc	+	+
AY210175	Human H2N2 IAVs	Human	H2N2		1965	USA	A/Pittsburgh/2/65	ggtgatgccccattccttgatcggcttcgccgagatcag aagtccctaaggggaagaggcagcactc	+	+
AY210177	Human H2N2 IAVs	Human	H2N2		1965	Japan	A/Kumamoto/1/65	ggtgatgccccattccttgatcggcttcgccgagatcag aagtccctaaggggaagaggcagcactc	+	+
CY031607	Human H2N2 IAVs	Human	H2N2		1965	Russia	A/Moscow/1019/1965	ggtgatgccccattccttgatcggcttcgccgagatcag aagtccctaaggggaagaggcagtgctc	-	*
AY210180	Human H2N2 IAVs	Human	H2N2		1966	USA	A/Berkeley/1/66	ggtgatgccccattccttgatcggcttcgccgagatcag aagtccctaaggggaagaggcagcactc	+	+
AY210179	Human H2N2 IAVs	Human	H2N2		1966	USA	A/California/1/66	ggtgatgccccattccttgatcggcttcgccgagatcag aagtccctaaggggaagaggcagcactc	+	+
AY210181	Human H2N2 IAVs	Human	H2N2		1966	Canada	A/Canada/1/66	ggtgatgccccattccttgatcggcttcgccgagatcag aagtccctaaggggaagaggcagcactc	+	+
CY034930	Human H2N2 IAVs	Human	H2N2		1966	Czech_Republic	A/Czech_Republic/1/1966	ggtgatgccccattccttgatcggcttcgccgagatcag aagtccctaaggggaagaggcagcactc	+	+
AY210178	Human H2N2 IAVs	Human	H2N2		1966	Panama	A/Panama/1/66	ggtgatgccccattccttgatcggcttcgccgagatcag aagtccctaaggggaagaggcagcactc	+	+
CY033984	Human H2N2 IAVs	Human	H2N2		1967	USA	A/Georgia/1/1967	ggtgatgccccattccttgatcggcttcgccgagacca gaagtccctaaggggaagaggcagcactc	+	+
AY210188	Human H2N2 IAVs	Human	H2N2		1967	USA	A/Georgia/1/67	ggtgatgccccattccttgatcggcttcgccgagacca gaagtccctaaggggaagaggcagcactc	+	+
AY210189	Human H2N2 IAVs	Human	H2N2		1967	United_Kingdom	A/England/10/67	ggtgatgccccattccttgatcggcttcgccgagatcag aaatccctaaggggaagaggcagcactc	-	-
CY125826	Human H2N2 IAVs	Human	H2N2		1967	Uruguay	A/Montevideo/2208/1967	ggtgatgccccattccttgatcggcttcgccgagatcag aagtccctaaagggaagaggcagcactc	+	+

AY210185	Human H2N2 IAVs	Human	H2N2		1967	Uruguay	A/Montevideo/2208/67	ggtgatgccccattccttgatcggcttcgccgagatcag<br>aagtccctaaagggaagaggcagcactc	+	+
AY210190	Human H2N2 IAVs	Human	H2N2		1967	Taiwan	A/Taiwan/1/67	ggtgatgccccattccttgatcggcttcgccgagatcag<br>aagtccctaaggggaagaggcagcactc	+	+
CY032289	Human H2N2 IAVs	Human	H2N2		1967	South_Africa	A/Johannesburg/617/1967	ggtgatgccccattccttgatcggcttcgccgagatcag<br>aagtccctaaggggaagaggcagcactc	+	+
AY210184	Human H2N2 IAVs	Human	H2N2		1967	Poland	A/Poland/5/67	ggtgatgccccattccttgatcggcttcgccgagatcag<br>aagtccctaaggggaagaggcagcactc	+	+
CY032281	Human H2N2 IAVs	Human	H2N2		1967	Uzbekistan	A/Tashkent/1046/1967	ggtgatgccccattccttgatcggcttcgccgagatcag<br>aagtccctaaggggaagaggcagcactc	+	+
CY020409	Human H2N2 IAVs	Human	H2N2		1967	USA	A/Albany/3/1967	ggtgatgccccattccttgatcggcttcgccgagatcag<br>aagtccctaaggggaagaggcagcactc	+	+
CY022001	Human H2N2 IAVs	Human	H2N2		1967	USA	A/Albany/4/1967	ggtgatgccccattccttgatcggcttcgccgagatcag<br>aagtccctaaggggaagaggcagcactc	+	+
CY020393	Human H2N2 IAVs	Human	H2N2		1967	USA	A/Albany/6/1967	ggtgatgccccattccttgatcggcttcgccgagatcag<br>aagtccctaaggggaagaggcagcactc	+	+
CY020401	Human H2N2 IAVs	Human	H2N2		1967	USA	A/Albany/7/1967	ggtgatgccccattccttgatcggcttcgccgagatcag<br>aagtccctaaggggaagaggcagcactc	+	+
CY096799	Human H2N2 IAVs	Human	mixed		1967	USA	A/Albany/5/1967_mixed_	ggtgatgccccattccttgatcggcttcgccgagatcag<br>aagtccctaaggggaagaggcagcactc	+	+
CY020521	Human H2N2 IAVs	Human	H2N2		1967	USA	A/Albany/9/1967	ggtgatgccccattccttgatcggcttcgccgagatcag<br>aagtccctaaggggaagaggcagcactc	+	+
AY210186	Human H2N2 IAVs	Human	H2N2		1967	USA	A/AnnArbor/7/67	ggtgatgccccattccttgatcggcttcgccgagatcag<br>aagtccctaaggggaagaggcagcactc	+	+
CY125842	Human H2N2 IAVs	Human	H2N2		1967	USA	A/Ann_Arbor/7/1967	ggtgatgccccattccttgatcggcttcgccgagatcag<br>aagtccctaaggggaagaggcagcactc	+	+
AY210183	Human H2N2 IAVs	Human	H2N2		1967	Japan	A/Tokyo/3/67	ggtgatgccccattccttgatcggcttcgccgagatcag<br>aagtccctaaggggaagaggcagcactc	+	+
AY210187	Human H2N2 IAVs	Human	H2N2		1967	Argentina	A/Cordoba/522/67	ggtgatgccccattccttgatcggcttcgccgagatcag<br>aagtccctaaggggaagaggcagcactc	+	+
CY020553	Human H2N2 IAVs	Human	H2N2		1967	USA	A/Albany/8/1967	ggtgatgccccatttcttgatcggcttcgccgagatcag<br>aagtccctaaggggaagaggcagcactc	-	-
CY125922	Human H2N2 IAVs	Human	H2N2		1967	USA	A/Cornell/1001/1967	ggtgatgccccatttcttgatcggcttcgccgagatcag<br>aagtccctaaggggaagaggcagcactc	-	-
AY210182	Human H2N2 IAVs	Human	H2N2		1967	Panama	A/Panama/1/67	ggtgatgccccatttcttgatcggcttcgccgagatcag<br>aagtccctaaggggaagaggcagcactc	-	-
CY125850	Human H2N2 IAVs	Human	H2N2		1968	South_Korea	A/Korea/426/1968	ggtgatgccccattccttgatcggcttcgccgagatcag<br>aagtccctaaggggaagaggcagcactc	+	+
CY031599	Human H2N2 IAVs	Human	H2N2		1968	South_Korea	A/Korea/426/1968	ggtgatgccccattccttgatcggcttcgccgagatcag<br>aagtccctaaggggaagaggcagcactc	+	+
CY036819	Human H2N2 IAVs	Human	H2N2		1968	South_Korea	A/Korea/426/1968	ggtgatgccccattccttgatcggcttcgccgagatcag<br>aagtccctaaggggaagaggcagcactc	+	+
AY210191	Human H2N2 IAVs	Human	H2N2		1968	South_Korea	A/Korea/426/68	ggtgatgccccattccttgatcggcttcgccgagatcag<br>aagtccctaaggggaagaggcagcactc	+	+
CY031591	Human H2N2 IAVs	Human	H2N2		1968	USA	A/North_Carolina/1/1968	ggtgatgccccattccttgatcggcttcgccgagatcag<br>aagtccctaaggggaagaggcagcactc	+	+
CY021025	Human H2N2 IAVs	Human	H2N2		1968	USA	A/Albany/1/1968	ggtgatgccccatttcttgatcggcttcgccgagatcag<br>aagtccctaaggggaagaggcagcactc	-	-
CY020417	Human H2N2 IAVs	Human	H2N2		1968	USA	A/Albany/2/1968	ggtgatgccccatttcttgatcggcttcgccgagatcag<br>aagtccctaaggggaagaggcagcactc	-	-

CY033477	Human H2N2 IAVs	Human	H2N2		1968	USA	A/berkeley/1/1968	ggtgatgccccatttcttgatcggcttcgccgagatcag<br>aagtccctaaggggaagaggcagcactc	-	-
AY210192	Human H2N2 IAVs	Human	H2N2		1968	USA	A/Berkeley/1/68	ggtgatgccccatttcttgatcggcttcgccgagatcag<br>aagtccctaaggggaagaggcagcactc	-	-
AY210300	Human H2N2 IAVs	Human	H3N2		1969	United_Kingdom	A/England/878/69	ggtgatgccccattccttgatcggcttcgccgagatcag<br>aagtccctaaggggaagaggcagcactc	+	+
CY006303	Human H2N2 IAVs	Human	H3N2		1969	Hong_Kong	A/Hong_Kong/3/1969	ggtgatgccccattccttgatcggcttcgccgagatcag<br>aagtccctaaggggaagaggcagcactc	+	+
CY022942	Human H2N2 IAVs	Human	H3N2		1970	USA	A/Albany/1/1970	ggtgatgccccattccttgatcggcttcgccgagatcag<br>aagtccctaaggggaagaggcagcactc	+	+
CY022950	Human H2N2 IAVs	Human	H3N2		1970	USA	A/Albany/3/1970	ggtgatgccccattccttgatcggcttcgccgagatcag<br>aagtccctaaggggaagaggcagcactc	+	+
CY021121	Human H2N2 IAVs	Human	H3N2		1970	USA	A/Albany/6/1970	ggtgatgccccattccttgatcggcttcgccgagatcag<br>aagtccctaaggggaagaggcagcactc	+	+
CY112277	Human H2N2 IAVs	Human	H3N2		1970	Netherlands	A/Bilthoven/2668/1970	ggtgatgccccattccttgatcggcttcgccgagatcag<br>aagtccctaaggggaagaggcagcactc	+	+
CY077844	Human H2N2 IAVs	Human	H3N2		1970	Netherlands	A/Bilthoven/2668/1970	ggtgatgccccattccttgatcggcttcgccgagatcag<br>aagtccctaaggggaagaggcagcactc	+	+
AY210304	Human H2N2 IAVs	Human	H3N2		1970	Canada	A/Canada/2/70	ggtgatgccccattccttgatcggcttcgccgagatcag<br>aagtccctaaggggaagaggcagcactc	+	+
AY210308	Human H2N2 IAVs	Human	H3N2		1970	Trinidad_and_Tobago	A/Trinidad/697/70	ggtgatgccccattccttgatcggcttcgccgagatcag<br>aagtccctaaggggaagaggcagcactc	+	+
CY021089	Human H2N2 IAVs	Human	H3N2		1970	USA	A/Albany/2/1970	ggtgatgccccattccttgatcggcttcgccgagatcag<br>aagtccctaaggggaagaggcagcactc	+	+
CY112269	Human H2N2 IAVs	Human	H3N2		1970	Netherlands	A/Bilthoven/93/1970	ggtgatgccccattccttgatcggcttcgccgagatcag<br>aagtccctaaggggaagaggcagcactc	+	+
DQ009924	Human H2N2 IAVs	Human	H2N2		2005	Canada	A/Canada/720/05	ggtgatgccccattccttgatcggcttcgccgagatcag<br>aagtccctaaggggaagaggcagtactc	+	+
CY021113	Human H3N2 IAVs	Human	H3N2		1968	USA	A/Albany/17/1968	ggagatgccccattccttgatcggcttcgccgagatca<br>gaagtccctaaggggaagaggcagcactc	+	+
AY210288	Human H3N2 IAVs	Human	H3N2		1968	Iran	A/Tehran/101/68	ggtgatgccccattccttgatcggcttcgccgagatcag<br>aagtccctaaggggaagaggcaacactc	+	+
CY121121	Human H3N2 IAVs	Human	H3N2		1968	Japan	A/Aichi/2/1968	ggtgatgccccattccttgatcggcttcgccgagatcag<br>aagtccctaaggggaagaggcagcactc	+	+
D10571	Human H3N2 IAVs	Human	H3N2		1968	Japan	A/Aichi/2/68_	ggtgatgccccattccttgatcggcttcgccgagatcag<br>aagtccctaaggggaagaggcagcactc	+	+
M34829	Human H3N2 IAVs	Human	H3N2		1968	Japan	A/Aichi/2/68	ggtgatgccccattccttgatcggcttcgccgagatcag<br>aagtccctaaggggaagaggcagcactc	+	+
CY019911	Human H3N2 IAVs	Human	H3N2		1968	USA	A/Albany/19/1968	ggtgatgccccattccttgatcggcttcgccgagatcag<br>aagtccctaaggggaagaggcagcactc	+	+
CY008160	Human H3N2 IAVs	Human	H3N2		1968	China	A/Beijing/1/1968	ggtgatgccccattccttgatcggcttcgccgagatcag<br>aagtccctaaggggaagaggcagcactc	+	+
AY210298	Human H3N2 IAVs	Human	H3N2		1968	USA	A/Hawaii/2/68	ggtgatgccccattccttgatcggcttcgccgagatcag<br>aagtccctaaggggaagaggcagcactc	+	+
CY044289	Human H3N2 IAVs	Human	H3N2		1968	LAB	A/Hong_Kong/1_6_MA21_1/1968	ggtgatgccccattccttgatcggcttcgccgagatcag<br>aagtccctaaggggaagaggcagcactc	+	+
AY210285	Human H3N2 IAVs	Human	H3N2		1968	Hong_Kong	A/HongKong/16/68	ggtgatgccccattccttgatcggcttcgccgagatcag<br>aagtccctaaggggaagaggcagcactc	+	+
AY210286	Human H3N2 IAVs	Human	H3N2		1968	Hong_Kong	A/HongKong/19/68	ggtgatgccccattccttgatcggcttcgccgagatcag<br>aagtccctaaggggaagaggcagcactc	+	+

CY006215	Human H3N2 IAVs	Human	H3N2		1968	USA	A/Memphis/1/1968	ggtgatgccccattccttgatcggcttcgccgagatcag<br>aagtccctaaggggaagaggcagcactc	+	+
AY210293	Human H3N2 IAVs	Human	H3N2		1968	Philippines	A/Philippines/104/68	ggtgatgccccattccttgatcggcttcgccgagatcag<br>aagtccctaaggggaagaggcagcactc	+	+
AY210291	Human H3N2 IAVs	Human	H3N2		1968	Puerto_Rico	A/PuertoRico/1/68	ggtgatgccccattccttgatcggcttcgccgagatcag<br>aagtccctaaggggaagaggcagcactc	+	+
AY210294	Human H3N2 IAVs	Human	H3N2		1968	Singapore	A/Singapore/1/68	ggtgatgccccattccttgatcggcttcgccgagatcag<br>aagtccctaaggggaagaggcagcactc	+	+
CY033509	Human H3N2 IAVs	Human	H3N2		1968	Hong_Kong	A/Hong_Kong/1_1_MA_12/1968	ggtgatgccccattccttgatcggcttcgccgagatcag<br>aagtccctaaggggaagaggcagcactc	+	+
CY033005	Human H3N2 IAVs	Human	H3N2		1968	Hong_Kong	A/Hong_Kong/1_1/1968	ggtgatgccccattccttgatcggcttcgccgagatcag<br>aagtccctaaggggaagaggcagcactc	+	+
CY033557	Human H3N2 IAVs	Human	H3N2		1968	Hong_Kong	A/Hong_Kong/1_11_MA21_3/196<br>8	ggtgatgccccattccttgatcggcttcgccgagatcag<br>aagtccctaaggggaagaggcagcactc	+	+
CY034008	Human H3N2 IAVs	Human	H3N2		1968	Hong_Kong	A/Hong_Kong/1_11/1968	ggtgatgccccattccttgatcggcttcgccgagatcag<br>aagtccctaaggggaagaggcagcactc	+	+
CY033565	Human H3N2 IAVs	Human	H3N2		1968	Hong_Kong	A/Hong_Kong/1_12_MA21_1/196<br>8	ggtgatgccccattccttgatcggcttcgccgagatcag<br>aagtccctaaggggaagaggcagcactc	+	+
CY033573	Human H3N2 IAVs	Human	H3N2		1968	Hong_Kong	A/Hong_Kong/1_12_MA21_2/196<br>8	ggtgatgccccattccttgatcggcttcgccgagatcag<br>aagtccctaaggggaagaggcagcactc	+	+
CY034040	Human H3N2 IAVs	Human	H3N2		1968	Hong_Kong	A/Hong_Kong/1_12_MA21_3/196<br>8	ggtgatgccccattccttgatcggcttcgccgagatcag<br>aagtccctaaggggaagaggcagcactc	+	+
CY033013	Human H3N2 IAVs	Human	H3N2		1968	Hong_Kong	A/Hong_Kong/1_2/1968	ggtgatgccccattccttgatcggcttcgccgagatcag<br>aagtccctaaggggaagaggcagcactc	+	+
CY033021	Human H3N2 IAVs	Human	H3N2		1968	Hong_Kong	A/Hong_Kong/1_4/1968	ggtgatgccccattccttgatcggcttcgccgagatcag<br>aagtccctaaggggaagaggcagcactc	+	+
CY033029	Human H3N2 IAVs	Human	H3N2		1968	Hong_Kong	A/Hong_Kong/1_5/1968	ggtgatgccccattccttgatcggcttcgccgagatcag<br>aagtccctaaggggaagaggcagcactc	+	+
CY033037	Human H3N2 IAVs	Human	H3N2		1968	Hong_Kong	A/Hong_Kong/1_6/1968	ggtgatgccccattccttgatcggcttcgccgagatcag<br>aagtccctaaggggaagaggcagcactc	+	+
CY034024	Human H3N2 IAVs	Human	H3N2		1968	Hong_Kong	A/Hong_Kong/1_8_MA21_1/1968	ggtgatgccccattccttgatcggcttcgccgagatcag<br>aagtccctaaggggaagaggcagcactc	+	+
CY033517	Human H3N2 IAVs	Human	H3N2		1968	Hong_Kong	A/Hong_Kong/1_9_MA21_1/1968	ggtgatgccccattccttgatcggcttcgccgagatcag<br>aagtccctaaggggaagaggcagcactc	+	+
CY033525	Human H3N2 IAVs	Human	H3N2		1968	Hong_Kong	A/Hong_Kong/1_9_MA21_2/1968	ggtgatgccccattccttgatcggcttcgccgagatcag<br>aagtccctaaggggaagaggcagcactc	+	+
CY033053	Human H3N2 IAVs	Human	H3N2		1968	Hong_Kong	A/Hong_Kong/1_9/1968	ggtgatgccccattccttgatcggcttcgccgagatcag<br>aagtccctaaggggaagaggcagcactc	+	+
CY112253	Human H3N2 IAVs	Human	H3N2		1968	Hong_Kong	A/Hong_Kong/1/1968	ggtgatgccccattccttgatcggcttcgccgagatcag<br>aagtccctaaggggaagaggcagcactc	+	+
CY033061	Human H3N2 IAVs	Human	H3N2		1968	Hong_Kong	A/Hong_Kong/1_10/1968	ggtgatgccccattccttgatcggcttcgccgagatcag<br>aagtccctaaggggaagaggcagcactc	+	+
CY034032	Human H3N2 IAVs	Human	H3N2		1968	Hong_Kong	A/Hong_Kong/1_8_MA21_3/1968	ggtgatgccccattccttgatcggcttcgccgagatcag<br>aagtccctaaggggaagaggcagcactc	+	+
CY034000	Human H3N2 IAVs	Human	H3N2		1968	Hong_Kong	A/Hong_Kong/1_8/1968	ggtgatgccccattccttgatcggcttcgccgagatcag<br>aagtccctaaggggaagaggcagcactc	+	+
CY044265	Human H3N2 IAVs	Human	H3N2		1968	Hong_Kong	A/Hong_Kong/1/1968	ggtgatgccccattccttgatcggcttcgccgagatcag<br>aagtccctaaggggaagaggcagcactc	+	+
CY044297	Human H3N2 IAVs	Human	H3N2		1968	LAB	A/Hong_Kong/1_10_MA21_3/196<br>8	ggtgatgccccattccttgatcggcttcgccgagatcag<br>aagtccctaaggggaagaggcagcactc	+	+

CY033045	Human H3N2 IAVs	Human	H3N2		1968	Hong_Kong	A/Hong_Kong/1_7/1968	ggtgatgccccattccttgatcggcttcgccgagatcagaagtccctaaggggaagaggcagcactc	+	+
AY210287	Human H3N2 IAVs	Human	H3N2		1968	Philippines	A/Philippines/3/68	ggtgatgccccattccttgatcggcttcgccgagatcagaagtccctaaggggaagaggcagcactc	+	+
KC296479	Human H3N2 IAVs	Human	H3N2		1968	Netherlands	A/Bilthoven/16190/1968	ggtgatgccccattccttgatcggcttcgccgagatcagaagtccctaaggggaagaggcagcactc	+	+
CY112245	Human H3N2 IAVs	Human	H3N2		1968	Netherlands	A/Bilthoven/16398/1968	ggtgatgccccattccttgatcggcttcgccgagatcagaagtccctaaggggaagaggcagcactc	+	+
AF348198	Human H3N2 IAVs	Human	H3N2		1968	Hong_Kong	A/Hong_Kong/1/68	ggtgatgccccattccttgatcggcttcgccgagatcagaagtccctaaggggaagaggcagcactc	+	+
CY021849	Human H3N2 IAVs	Human	H3N2		1968	USA	A/Albany/10/1968	ggtgatgccccattccttgatcggcttcgccgagatcagaagtccctaaggggaagaggcagcactc	+	+
CY019895	Human H3N2 IAVs	Human	H3N2		1968	USA	A/Albany/11/1968	ggtgatgccccattccttgatcggcttcgccgagatcagaagtccctaaggggaagaggcagcactc	+	+
CY022089	Human H3N2 IAVs	Human	H3N2		1968	USA	A/Albany/6/1968	ggtgatgccccattccttgatcggcttcgccgagatcagaagtccctaaggggaagaggcagcactc	+	+
CY112237	Human H3N2 IAVs	Human	H3N2		1968	Netherlands	A/Bilthoven/15793/1968	ggtgatgccccattccttgatcggcttcgccgagatcagaagtccctaaggggaagaggcagcactc	+	+
AY210296	Human H3N2 IAVs	Human	H3N2		1968	Canada	A/Canada/228/68	ggtgatgccccattccttgatcggcttcgccgagatcagaagtccctaaggggaagaggcagcactc	+	+
AY210295	Human H3N2 IAVs	Human	H3N2		1968	Russia	A/USSR/039/68	ggtgatgccccattccttgatcggcttcgccgagatcagaagtccctaaggggaagaggcagcactc	+	+
CY020529	Human H3N2 IAVs	Human	H3N2		1968	USA	A/Albany/18/1968	ggtgatgccccattccttgatcggcttcgccgagatcagaagtccctaaggggaagaggcagcactc	+	+
AY210297	Human H3N2 IAVs	Human	H3N2		1968	USA	A/Georgia/122/68	ggtgatgccccattccttgatcggcttcgccgagatcagaagtccctaaggggaagaggcagcactc	+	+
AY210289	Human H3N2 IAVs	Human	H3N2		1968	Malaysia	A/Malaysia/1/68	ggtgatgccccattccttgatcggcttcgccgagatcagaagtccctaaggggaagaggcagcactc	+	+
AY210290	Human H3N2 IAVs	Human	H3N2		1968	Malaysia	A/Malaysia/221/68	ggtgatgccccattccttgatcggcttcgccgagatcagaagtccctaaggggaagaggcagcactc	+	+
AY210292	Human H3N2 IAVs	Human	H3N2		1968	Panama	A/Panama/1/68	ggtgatgccccattccttgatcggcttcgccgagatcagaagtccctaaggggaagaggcagcactc	+	+
CY033992	Human H3N2 IAVs	Human	H3N2		1968	Hong_Kong	A/Hong_Kong/1_1_MA_12D/1968	ggtgatgccccattccttgatcggcttcgccgagatcagaagtccctaaggggaagaggcagcactc	+	+
CY044273	Human H3N2 IAVs	Human	H3N2		1968	Hong_Kong	A/Hong_Kong/1_1_MA_20/1968	ggtgatgccccattccttgatcggcttcgccgagatcagaagtccctaaggggaagaggcagcactc	+	+
CY033541	Human H3N2 IAVs	Human	H3N2		1968	Hong_Kong	A/Hong_Kong/1_11_MA21_1/1968	ggtgatgccccattccttgatcggcttcgccgagatcagaagtccctaaggggaagaggcagcactc	+	+
CY034016	Human H3N2 IAVs	Human	H3N2		1968	LAB	A/Hong_Kong/1_6_MA21_2/1968	ggtgatgccccattccttgatcggcttcgccgagatcagaagtccctaaggggaagaggcagcactc	+	+
CY147450	Human H3N2 IAVs	Human	H3N2		1968	Australia	A/Northern_Territories/60_JY2/1968	ggtgatgccccattccttgatcggcttcgccgagatcagaagtccctaaggggaagaggcagcactc	+	+
CY011124	Human H3N2 IAVs	Human	H3N2		1968	Australia	A/Northern_Territory/60/1968	ggtgatgccccattccttgatcggcttcgccgagatcagaagtccctaaggggaagaggcagcactc	+	+
CY033549	Human H3N2 IAVs	Human	H3N2		1968	Hong_Kong	A/Hong_Kong/1_11_MA21_2/1968	ggtgatgccccattccttgatcggcttcgccgagatcagaagtccctaaggggaagaggcagcactc	+	+
CY044281	Human H3N2 IAVs	Human	H3N2		1968	LAB	A/Hong_Kong/1_5_MA21_3/1968	ggtgatgccccattccttgatcggcttcgccgagatcagaagtccctaaggggaagaggcagcactc	+	+
EU403423	Human H3N2 IAVs	Human	H3N2		1968	Hong_Kong	A/Hong_Kong/1/68	ggtgatgccccattccttgatcggcttcgccgagatcagaagtccctaaggggaagaggcagcactc	+	+

EU403422	Human H3N2 IAVs	Human	H3N2		1968	Hong_Kong	A/Hong_Kong/1/68	ggtgatgccccattccttgatcggcttcgccgagatcag aagtccctaaggggaagaggcagcactc	+	+
EU403421	Human H3N2 IAVs	Human	H3N2		1968	Hong_Kong	A/Hong_Kong/1/68	ggtgatgccccattccttgatcggcttcgccgagatcag aagtccctaaggggaagaggcagcactc	+	+
CY033533	Human H3N2 IAVs	Human	H3N2		1968	Hong_Kong	A/Hong_Kong/1_9_MA21_3/1968	ggtgatgccccattccttgatcggcttcgccgagatcag aagtccctaaggggaagaggcagcactc	+	+
CY146813	Human H3N2 IAVs	Human	H3N2		1968	Australia	A/Victoria/JY2/1968	ggtgatgccccattccttgatcggcttcgccgagatcag aagtccctaaggggaagaggcagcactc	+	+
AY210299	Human H3N2 IAVs	Human	H3N2		1969	USA	A/Alaska/1/69	ggtgatgccccattccttgatcggcttcgccgagatcag aagtccctaaggggaagaggcagcactc	+	+
AY210301	Human H3N2 IAVs	Human	H3N2		1969	Brazil	A/Rio/6/69	ggtgatgccccattccttgatcggcttcgccgagatcag aagtccctaaggggaagaggcagcactc	+	+
CY019903	Human H3N2 IAVs	Human	H3N2		1969	USA	A/Albany/1/1969	ggtgatgccccattccttgatcggcttcgccgagatcag aagtccctaaggggaagaggcagcactc	+	+
CY019919	Human H3N2 IAVs	Human	H3N2		1969	USA	A/Albany/3/1969	ggtgatgccccattccttgatcggcttcgccgagatcag aagtccctaaggggaagaggcagcactc	+	+
CY113057	Human H3N2 IAVs	Human	H3N2		1969	Netherlands	A/Bilthoven/17938/1969	ggtgatgccccattccttgatcggcttcgccgagatcag aagtccctaaggggaagaggcagcactc	+	+
AY210303	Human H3N2 IAVs	Human	H3N2		1969	Russia	A/USSR/0270/69	ggtgatgccccattccttgatcggcttcgccgagatcag aagtccctaaggggaagaggcagcactc	+	+
CY112261	Human H3N2 IAVs	Human	H3N2		1969	Netherlands	A/Bilthoven/808/1969	ggtgatgccccattccttgatcggcttcgccgagatcag aagtccctaaggggaagaggcagcactc	+	+
AY210302	Human H3N2 IAVs	Human	H3N2		1969	Taiwan	A/Taiwan/1/69	ggtgatgccccattccttgatcggcttcgccgagatcag aagtccctaaggggaagaggcagcactc	+	+
CY021841	Human H3N2 IAVs	Human	H3N2		1969	USA	A/Albany/4/1969	ggtgatgccccattccttgatcggcttcgccgagatcag aagtccctaaggggaagaggcagcactc	+	+
CY113049	Human H3N2 IAVs	Human	H3N2		1969	Netherlands	A/Bilthoven/908/1969	ggtgatgccccattccttgatcggcttcgccgagatcag aagtccctaaggggaagaggcagcactc	+	+
AY210306	Human H3N2 IAVs	Human	H3N2		1970	Taiwan	A/Taiwan/2/70	ggtgatgccccattccttgatcggcttcgccgagatcag aagtccctaaggggaagaggcagcactc	+	+
AY210305	Human H3N2 IAVs	Human	H3N2		1970	Australia	A/Queensland/7/70	ggtgatgccccattccttgatcggcttcgccgagatcag aagtccctaaggggaagaggcagcactc	+	+
CY112293	Human H3N2 IAVs	Human	H3N2		1971	Hong_Kong	A/Hong_Kong/107/1971	ggtgatgccccattccttgatcggcttcgccgagatcag aagtccctaaggggaagaggcagcactc	+	+
CY006687	Human H3N2 IAVs	Human	H3N2		1971	Hong_Kong	A/Hong_Kong/46/1971	ggtgatgccccattccttgatcggcttcgccgagatcag aagtccctaaggggaagaggcagcactc	+	+
AY210307	Human H3N2 IAVs	Human	H3N2		1971	Taiwan	A/Taiwan/3/71	ggtgatgccccattccttgatcggcttcgccgagatcag aagtccctaaggggaagaggcagcactc	+	+
CY113065	Human H3N2 IAVs	Human	H3N2		1971	Netherlands	A/Bilthoven/21438/1971	ggtgatgccccattccttgatcggcttcgccgagatcag aagtccctaaggggaagaggcagcactc	+	+
CY077816	Human H3N2 IAVs	Human	H3N2		1971	Netherlands	A/Bilthoven/21438/1971	ggtgatgccccattccttgatcggcttcgccgagatcag aagtccctaaggggaagaggcagcactc	+	+
CY021601	Human H3N2 IAVs	Human	H3N2		1971	USA	A/Memphis/3/1971	ggtgatgccccattccttgatcggcttcgccgagatcag aagtccctaaggggaagaggcagcactc	+	+
AY210311	Human H3N2 IAVs	Human	H3N2		1971	Hungary	A/Hungary/2/71	ggtgatgccccattccttgatcggcttcgccgagatcag aagtccctaaggggaagaggcagcactc	+	+
CY112285	Human H3N2 IAVs	Human	H3N2		1971	Netherlands	A/Bilthoven/6449/1971	ggtgatgccccattccttgatcggcttcgccgagatcag aagtccctaaggggaagaggcagcactc	+	+
CY077864	Human H3N2 IAVs	Human	H3N2		1971	Netherlands	A/Bilthoven/6449/1971	ggtgatgccccattccttgatcggcttcgccgagatcag aagtccctaaggggaagaggcagcactc	+	+

AY210310	Human H3N2 IAVs	Human	H3N2		1971	Japan	A/Chiba/5/71	ggtgatgccccattccttgatcggcttcgccgagatcag<br>aagtccctaaggggaagaggcagcactc	+	+
CY002500	Human H3N2 IAVs	Human	H3N2		1971	USA	A/Memphis/1/1971	ggtgatgccccattccttgatcggcttcgccgagatcag<br>aagtccctaaggggaagaggcagcactc	+	+
AY210309	Human H3N2 IAVs	Human	H3N2		1971	Venezuela	A/Caracas/1/71	ggtgatgccccattccttgatcggcttcgccgagatcag<br>aagtccctaaggggaagaggcagcactc	+	+
CY113073	Human H3N2 IAVs	Human	H3N2		1971	Netherlands	A/Bilthoven/21801/1971	ggtgatgccccattccttgatcggcttcgccgagatcag<br>aagtccctaagggggagaggcagcactc	+	+
CY008464	Human H3N2 IAVs	Human	H3N2		1972	USA	A/Memphis/103/1972	ggtgatgccccattccttgatcggcttcgccgagacca<br>gaagtccctaaggggaagaggcagcactc	+	+
CY008688	Human H3N2 IAVs	Human	H3N2		1972	USA	A/Memphis/105/1972	ggtgatgccccattccttgatcggcttcgccgagacca<br>gaagtccctaaggggaagaggcagcactc	+	+
CY006311	Human H3N2 IAVs	Human	H3N2		1972	Hong_Kong	A/Hong_Kong/50/1972	ggtgatgccccattccttgatcggcttcgccgagatcag<br>aagtccctaaggggaagaggcagcactc	+	+
CY003556	Human H3N2 IAVs	Human	H3N2		1972	Hong_Kong	A/Hong_Kong/6/1972	ggtgatgccccattccttgatcggcttcgccgagatcag<br>aagtccctaaggggaagaggcagcactc	+	+
CY112309	Human H3N2 IAVs	Human	H3N2		1972	Netherlands	A/Bilthoven/21793/1972	ggtgatgccccattccttgatcggcttcgccgagatcag<br>aagtccctaaggggaagaggcagcactc	+	+
CY077823	Human H3N2 IAVs	Human	H3N2		1972	Netherlands	A/Bilthoven/21793/1972	ggtgatgccccattccttgatcggcttcgccgagatcag<br>aagtccctaaggggaagaggcagcactc	+	+
CY112317	Human H3N2 IAVs	Human	H3N2		1972	Netherlands	A/Bilthoven/23337/1972	ggtgatgccccattccttgatcggcttcgccgagatcag<br>aagtccctaaggggaagaggcagcactc	+	+
CY077830	Human H3N2 IAVs	Human	H3N2		1972	Netherlands	A/Bilthoven/23337/1972	ggtgatgccccattccttgatcggcttcgccgagatcag<br>aagtccctaaggggaagaggcagcactc	+	+
CY002748	Human H3N2 IAVs	Human	H3N2		1972	USA	A/Memphis/109/1972	ggtgatgccccattccttgatcggcttcgccgagatcag<br>aagtccctaaggggaagaggcagcactc	+	+
CY007975	Human H3N2 IAVs	Human	H3N2		1972	China	A/Guandong/243/1972	ggtgatgccccattccttgatcggcttcgccgagatcag<br>aagtccctaaggggaagaggcagcactc	+	+
CY008680	Human H3N2 IAVs	Human	H3N2		1972	USA	A/Memphis/101/1972	ggtgatgccccattccttgatcggcttcgccgagatcag<br>aagtccctaaggggaagaggcagcactc	+	+
CY002100	Human H3N2 IAVs	Human	H3N2		1972	USA	A/Memphis/102/1972	ggtgatgccccattccttgatcggcttcgccgagatcag<br>aagtccctaaggggaagaggcagcactc	+	+
CY009640	Human H3N2 IAVs	Human	H3N2		1972	Russia	A/udorn/1972	ggtgatgccccattccttgatcggcttcgccgagatcag<br>aagtccctaaggggaagaggcagcactc	+	+
AX350200	Human H3N2 IAVs	Human	H3N2		1972	Russia	A/Udorn/307/1972	ggtgatgccccattccttgatcggcttcgccgagatcag<br>aagtccctaaggggaagaggcagcactc	+	+
V01102	Human H3N2 IAVs	Human	H3N2		1972	Russia	A/Udorn/8/1972	ggtgatgccccattccttgatcggcttcgccgagatcag<br>aagtccctaaggggaagaggcagcactc	+	+
DQ508933	Human H3N2 IAVs	Human	H3N2		1972	Russia	A/Udorn/307/1972	ggtgatgccccattccttgatcggcttcgccgagatcag<br>aagtccctaaggggaagaggcagcactc	+	+
AY210315	Human H3N2 IAVs	Human	H3N2		1972	Japan	A/Tokyo/31/72	ggtgatgccccattccttgatcggcttcgccgagatcag<br>aagtccctaaggggaagaggcagcactc	+	+
AY210316	Human H3N2 IAVs	Human	H3N2		1972	Australia	A/Victoria/4/72	ggtgatgccccattccttgatcggcttcgccgagatcag<br>aagtccctaaggggaagaggcagcactc	+	+
CY113081	Human H3N2 IAVs	Human	H3N2		1972	Netherlands	A/Bilthoven/23290/1972	ggtgatgccccattccttgatcggcttcgccgagatcag<br>aagtccctaaggggaagaggcagcactc	+	+
AY210314	Human H3N2 IAVs	Human	H3N2		1972	Taiwan	A/Taiwan/1/72	ggtgatgccccattccttgatcggcttcgccgagatcag<br>aagtccctaaggggaagaggcagcactc	+	+
AY210313	Human H3N2 IAVs	Human	H3N2		1972	Puerto_Rico	A/PuertoRico/3/72	ggtgatgccccattccttgatcggcttcgccgagatcag<br>aagtccctaaggggaagaggcagcactc	+	+

CY112301	Human H3N2 IAVs	Human	H3N2		1972	Netherlands	A/Bilthoven/6022/1972	ggtgatgccccattccttgatcggcttcgccgagatcag aagtccctaaggggaagaggcagcactc	+	+
CY113089	Human H3N2 IAVs	Human	H3N2		1972	United_Kingdom	A/England/42/1972	ggtgatgccccattccttgatcggcttcgccgagatcag aagtccctaaggggaagaggcagcactc	+	+
CY121161	Human H3N2 IAVs	Human	H3N2		1972	United_Kingdom	A/England/42/1972	ggtgatgccccattccttgatcggcttcgccgagatcag aagtccctaaggggaagaggcagcactc	+	+
AY210312	Human H3N2 IAVs	Human	H3N2		1972	United_Kingdom	A/England/42/72	ggtgatgccccattccttgatcggcttcgccgagatcag aagtccctaaggggaagaggcagcactc	+	+
CY009360	Human H3N2 IAVs	Human	H3N2		1972	United_Kingdom	A/England/72	ggtgatgccccattccttgatcggcttcgccgagatcag aagtccctaaggggaagaggcagcactc	+	+
CY113113	Human H3N2 IAVs	Human	H3N2		1973	LAB	A/Port_chalmers/1/1973	ggtgatgccccattccttgatcggcttcgccgagacca gaagtccctaaggggaagaggcagcactc	+	+
CY113097	Human H3N2 IAVs	Human	H3N2		1973	Netherlands	A/Bilthoven/552/1973	ggtgatgccccattccttgatcggcttcgccgagatcag aagtccctaaggggaagaggcagcactc	+	+
CY006815	Human H3N2 IAVs	Human	H3N2		1973	USA	A/Memphis/3/1973	ggtgatgccccattccttgatcggcttcgccgagatcag aagtccctaaggggaagaggcagcactc	+	+
CY113105	Human H3N2 IAVs	Human	H3N2		1973	Netherlands	A/Bilthoven/3517/1973	ggtgatgccccattccttgatcggcttcgccgagatcag aagtccctaaggggaagaggcagcactc	+	+
CY112325	Human H3N2 IAVs	Human	H3N2		1973	Netherlands	A/Bilthoven/748/1973	ggtgatgccccattccttgatcggcttcgccgagatcag aagtccctaaggggaagaggcagcactc	+	+
CY009008	Human H3N2 IAVs	Human	H3N2		1973	Hong_Kong	A/Hong_Kong/33/1973	ggtgatgccccattccttgatcggcttcgccgagatcag aagtccctaaggggaagaggcagcactc	+	+
CY009352	Human H3N2 IAVs	Human	H3N2		1973	LAB	A/Port_chalmers/1/1973	ggtgatgccccattccttgatcggcttcgccgagatcag aagtccctaaggggaagaggcagcactc	+	+
CY121185	Human H3N2 IAVs	Human	H3N2		1973	LAB	A/Port_chalmers/1/1973	ggtgatgccccattccttgatcggcttcgccgagatcag aagtccctaaggggaagaggcagcactc	+	+
CY147434	Human H3N2 IAVs	Human	H3N2		1973	New_Zealand	A/Port_Chalmers/JY2/1973	ggtgatgccccattccttgatcggcttcgccgagatcag aagtccctaaggggaagaggcagcactc	+	+
CY003532	Human H3N2 IAVs	Human	H3N2		1973	Hong_Kong	A/Hong_Kong/11/1973	ggtgatgccccattccttgatcggcttcgccgagatcag aagtccctaaggggaagaggcagcactc	+	+
CY113121	Human H3N2 IAVs	Human	H3N2		1974	Netherlands	A/Bilthoven/5930/1974	ggtgatgccccattccttgatcgacttcgccgagatcag aagtccctaaggggaagaggcagcactc	+	+
CY113129	Human H3N2 IAVs	Human	H3N2		1974	Netherlands	A/Bilthoven/5931/1974	ggtgatgccccattccttgatcgacttcgccgagatcag aagtccctaaggggaagaggcagcactc	+	+
CY113145	Human H3N2 IAVs	Human	H3N2		1974	Netherlands	A/Bilthoven/9459/1974	ggtgatgccccattccttgatcgacttcgccgagatcag aagtccctaaggggaagaggcagcactc	+	+
CY113137	Human H3N2 IAVs	Human	H3N2		1974	Netherlands	A/Bilthoven/7398/1974	ggtgatgccccattccttgatcgacttcgccgagatcag aagtccctaaggggaagaggcagcactc	+	+
CY077871	Human H3N2 IAVs	Human	H3N2		1974	Netherlands	A/Bilthoven/7398/1974	ggtgatgccccattccttgatcgacttcgccgagatcag aagtccctaaggggaagaggcagcactc	+	+
CY003500	Human H3N2 IAVs	Human	H3N2		1974	Hong_Kong	A/Hong_Kong/14/1974	ggtgatgccccattccttgatcgacttcgccgagatcag aagtccctaaggggaagaggcagcactc	+	+
CY021097	Human H3N2 IAVs	Human	H3N2		1974	USA	A/Albany/20/1974	ggtgatgccccattccttgatcggcttcgccgagatcag aagtccctaaggggaagaggcagcactc	+	+
CY006911	Human H3N2 IAVs	Human	H3N2		1974	Hong_Kong	A/Hong_Kong/49/1974	ggtgatgccccattccttgatcggcttcgccgagatcag aagtccctaaggggaagaggcagcactc	+	+
CY112333	Human H3N2 IAVs	Human	H3N2		1974	Netherlands	A/Bilthoven/5146/1974	ggtgatgccccattccttgatcggcttcgccgagatcag aagtccctaaggggaagaggcagcactc	+	+
CY006719	Human H3N2 IAVs	Human	H3N2		1974	USA	A/Memphis/101/1974	ggtgatgccccattccttgatcggcttcgccgagatcag aggtccctaaggggaagaggcagcactc	-	-

CY006831	Human H3N2 IAVs	Human	H3N2		1974	USA	A/Memphis/103/1974	ggtgatgccccattccttgatcggcttcgccgagatcag<br>aggtccctaaggggaagaggcagcactc	-	-
CY006823	Human H3N2 IAVs	Human	H3N2		1974	USA	A/Memphis/102/1974	ggtgatgccccattccttgatcggcttcgccgagatcag<br>aggtccctaaggggaagaggcagcactc	-	-
CY113169	Human H3N2 IAVs	Human	H3N2		1975	Netherlands	A/Bilthoven/2600/1975	ggtgatgccccattccttgatcggcttcgccgagatcag<br>aagtccctaaggggaagaagcagcactc	+	+
CY113177	Human H3N2 IAVs	Human	H3N2		1975	Netherlands	A/Bilthoven/2813/1975	ggtgatgccccattccttgatcggcttcgccgagatcag<br>aagtccctaaggggaagaagcagcactc	+	+
CY112341	Human H3N2 IAVs	Human	H3N2		1975	Netherlands	A/Bilthoven/4273/1975	ggtgatgccccattccttgatcggcttcgccgagatcag<br>aagtccctaaggggaagaagcagcactc	+	+
CY006048	Human H3N2 IAVs	Human	H3N2		1975	China	A/Beijing/39/1975	ggtgatgccccattccttgatcggcttcgccgagatcag<br>aagtccctaaggggaagaggcagcactc	+	+
CY003732	Human H3N2 IAVs	Human	H3N2		1975	Hong_Kong	A/Hong_Kong/43/1975	ggtgatgccccattccttgatcggcttcgccgagatcag<br>aagtccctaaggggaagaggcagcactc	+	+
CY121201	Human H3N2 IAVs	Human	H3N2		1975	Australia	A/Victoria/3/1975	ggtgatgccccattccttgatcggcttcgccgagatcag<br>aagtccctaaggggaagaggcagcactc	+	+
CY113185	Human H3N2 IAVs	Human	H3N2		1975	Australia	A/Victoria/3/1975	ggtgatgccccattccttgatcggcttcgccgagatcag<br>aagtccctaaggggaagaggcagcactc	+	+
CY021081	Human H3N2 IAVs	Human	H3N2		1975	USA	A/Albany/42/1975	ggtgatgccccattccttgatcggcttcgccgagatcag<br>aggtccctaaggggaagaggcagcactc	-	-
CY113161	Human H3N2 IAVs	Human	H3N2		1975	Netherlands	A/Bilthoven/1843/1975	ggtgatgccccattccttgatcggcttcgccgagatcag<br>aggtccctaaggggaagaggcagcactc	-	-
CY113153	Human H3N2 IAVs	Human	H3N2		1975	Netherlands	A/Bilthoven/334/1975	ggtgatgccccattccttgatcggcttcgccgagatcag<br>aggtccctaaggggaagaggcagcactc	-	-
CY113193	Human H3N2 IAVs	Human	H3N2		1976	Netherlands	A/Bilthoven/628/1976	ggtgatgccccattccttgataggcttcgccgagatca<br>gaagtccctaaggggaagaagcagcactc	+	+
CY021945	Human H3N2 IAVs	Human	H3N2		1976	USA	A/Albany/1/1976	ggtgatgccccattccttgatcggcttcgccgagatcag<br>aagtccctaaggggaagaggcagcactc	+	+
CY021833	Human H3N2 IAVs	Human	H3N2		1976	USA	A/Albany/15/1976	ggtgatgccccattccttgatcggcttcgccgagatcag<br>aagtccctaaggggaagaggcagcactc	+	+
CY113201	Human H3N2 IAVs	Human	H3N2		1976	Netherlands	A/Bilthoven/1761/1976	ggtgatgccccattccttgatcggcttcgccgagatcag<br>aagtccctaaggggaagaggcagcactc	+	+
CY077796	Human H3N2 IAVs	Human	H3N2		1976	Netherlands	A/Bilthoven/1761/1976	ggtgatgccccattccttgatcggcttcgccgagatcag<br>aagtccctaaggggaagaggcagcactc	+	+
CY113209	Human H3N2 IAVs	Human	H3N2		1976	Netherlands	A/Bilthoven/5029/1976	ggtgatgccccattccttgatcggcttcgccgagatcag<br>aagtccctaaggggaagaggcagcactc	+	+
CY112349	Human H3N2 IAVs	Human	H3N2		1976	Netherlands	A/Bilthoven/5168/1976	ggtgatgccccattccttgatcggcttcgccgagatcag<br>aagtccctaaggggaagaggcagcactc	+	+
CY113217	Human H3N2 IAVs	Human	H3N2		1976	Netherlands	A/Bilthoven/5657/1976	ggtgatgccccattccttgatcggcttcgccgagatcag<br>aagtccctaaggggaagaggcagcactc	+	+
CY009064	Human H3N2 IAVs	Human	H3N2		1976	USA	A/Memphis/105/1976	ggtgatgccccattccttgatcggcttcgccgagatcag<br>aagtccctaaggggaagaggcagcactc	+	+
CY006839	Human H3N2 IAVs	Human	H3N2		1976	USA	A/Memphis/110/1976	ggtgatgccccattccttgatcggcttcgccgagatcag<br>aagtccctaaggggaagaggcagcactc	+	+
CY006727	Human H3N2 IAVs	Human	H3N2		1976	USA	A/Memphis/137/1976	ggtgatgccccattccttgatcggcttcgccgagatcag<br>aagtccctaaggggaagaggcagcactc	+	+
CY008704	Human H3N2 IAVs	Human	H3N2		1976	USA	A/Memphis/108/1976	ggtgatgccccattccttgatcggcttcgccgagatcag<br>aagtccctaaggggaagaggcagcactc	+	+
CY006887	Human H3N2 IAVs	Human	H3N2		1976	USA	A/Memphis/103/1976	ggtgatgccccattccttgatcggcttcgccgagatcag<br>aagtccctaaggggaagaggcagcactc	+	+

CY022313	Human H3N2 IAVs	Human	H3N2		1976	USA	A/Memphis/104/1976	ggtgatgccccattccttgatcggcttcgccgagatcag aagtccctaaggggaagaggcagcactc	+	+
CY113225	Human H3N2 IAVs	Human	H3N2		1976	Netherlands	A/Bilthoven/6545/1976	ggtgatgccccattccttgatcggcttcgccgagatcag aagtccctaaggggaagaggcagcactc	+	+
CY008696	Human H3N2 IAVs	Human	H3N2		1976	USA	A/Memphis/106/1976	ggtgatgccccattccttgatcggcttcgccgagatcag aagtccctaaggggaagaggcatcactc	-	-
KC296466	Human H3N2 IAVs	Human	H3N2		1976	Netherlands	A/Bilthoven/2271/1976	ggtgatgccccattccttgatcggcttcgccgagatcag aggtccctaaggggaagaggcagcactc	-	-
CY077916	Human H3N2 IAVs	Swine	H3N2		1977	Italy	A/swine/Italy/1850/1977	ggtgatgccccattccttgatcggcttcgcagagatca gaagtccttaaggggaagaggcagcactc	-	-
M80962	Human H3N2 IAVs	Swine	H3N2		1977	Italy	A/swine/Italy/1850/1977	ggtgatgccccattccttgatcggcttcgcagagatca gaagtccttaaggggaagaggcagcactc	-	-
CY021105	Human H3N2 IAVs	Human	H3N2		1977	USA	A/Albany/4/1977	ggtgatgccccattccttgatcggcttcgccgagatcag aagtccctaaggggaagaggcaacactc	-	-
CY113249	Human H3N2 IAVs	Human	H3N2		1977	Netherlands	A/Rotterdam/5828/1977	ggtgatgccccattccttgatcggcttcgccgagatcag aagtccctaaggggaagaggcaacactc	-	-
CY113241	Human H3N2 IAVs	Human	H3N2		1977	Netherlands	A/Bilthoven/3895/1977	ggtgatgccccattccttgatcggcttcgccgagatcag aagtccctaaggggaagaggcaacactc	-	-
CY113257	Human H3N2 IAVs	Human	H3N2		1977	Netherlands	A/Rotterdam/8179/1977	ggtgatgccccattccttgatcggcttcgccgagatcag aagtccctaaggggaagaggcaacactc	-	-
CY113233	Human H3N2 IAVs	Human	H3N2		1977	Netherlands	A/Amsterdam/1609/1977	ggtgatgccccattccttgatcggcttcgccgagatcag aagtccctaaggggaagaggcaacactc	-	-
CY006767	Human H3N2 IAVs	Human	H3N2		1977	China	A/Nanjing/49/1977	ggtgatgccccattccttgatcggcttcgccgagatcag aagtccctaaggggaagaggcagcactc	+	+
CY009304	Human H3N2 IAVs	Swine	H3N2		1977	USA	A/swine/Colorado/1/1977	ggtgatgccccattccttgatcggcttcgccgagatcag aagtccctaaggggaagaggcagcactc	+	+
M80960	Human H3N2 IAVs	Swine	H3N2		1977	USA	A/swine/Colorado/1/1977	ggtgatgccccattccttgatcggcttcgccgagatcag aagtccctaaggggaagaggcagcactc	+	+
K01332	Human H3N2 IAVs	Human	H3N2		1977	USA	A/Alaska/6/1977	ggtgatgccccattccttgatcggcttcgccgagatcag aggtccctaaggggaagaggcagcactc	-	-
CY113265	Human H3N2 IAVs	Human	H3N2		1977	USA	A/Texas/1/1977	ggtgatgccccattccttgatcggcttcgccgagatcag aggtccctaaggggaagaggcagcactc	-	-
CY120996	Human H3N2 IAVs	Human	H3N2		1977	USA	A/Texas/1/1977	ggtgatgccccattccttgatcggcttcgccgagatcag aggtccctaaggggaagaggcagcactc	-	-
CY006735	Human H3N2 IAVs	Human	H3N2		1977	USA	A/Memphis/1/1977	ggtgatgccccattccttgatcggcttcgccgagatcag aggtccctaaggggaagaggcagcactc	-	-
CY008119	Human H3N2 IAVs	Human	H3N2		1977	USA	A/Memphis/2/1977	ggtgatgccccattccttgatcggcttcgccgagatcag aggtccctaaggggaagaggcagcactc	-	-
CY006743	Human H3N2 IAVs	Human	H3N2		1977	USA	A/Memphis/3/1977	ggtgatgccccattccttgatcggcttcgccgagatcag aggtccctaaggggaagaggcagcactc	-	-
CY008127	Human H3N2 IAVs	Human	H3N2		1977	USA	A/Memphis/4/1977	ggtgatgccccattccttgatcggcttcgccgagatcag aggtccctaaggggaagaggcagcactc	-	-
CY006847	Human H3N2 IAVs	Human	H3N2		1977	USA	A/Memphis/5/1977	ggtgatgccccattccttgatcggcttcgccgagatcag aggtccctaaggggaagaggcagcactc	-	-
CY020329	Human H3N2 IAVs	Human	H3N2		1978	USA	A/Memphis/16/1978	ggtgatgccccattccttgatcggcttcgccgagatcag aggtccctaaggggaagaggcagcactc	-	-
CY006711	Human H3N2 IAVs	Human	H3N2		1978	USA	A/Memphis/18/1978	ggtgatgccccattccttgatcggcttcgccgagatcag aggtccctaaggggaagaggcagcactc	-	-
CY006695	Human H3N2 IAVs	Human	H3N2		1978	USA	A/Memphis/2/1978	ggtgatgccccattccttgatcggcttcgccgagatcag aggtccctaaggggaagaggcagcactc	-	-

CY006703	Human H3N2 IAVs	Human	H3N2		1978	USA	A/Memphis/12/1978	ggtgatgccccattccttgatcggcttcgccgagatcag aggtccctaaggggaagaggcagcactc	-	-
CY020225	Human H3N2 IAVs	Human	H3N2		1978	USA	A/Albany/14/1978	ggtgatgccccattccttgatcggcttcgccgagatcag aggtccctaaggggaagaggcagcactc	-	-
CY007615	Human H3N2 IAVs	Human	H3N2		1978	USA	A/Memphis/19/1978	ggtgatgccccattccttgatcggcttcgccgagatcag aggtccctaaggggaagaggcagcactc	-	-
DQ508829	Human H3N2 IAVs	Human	H3N2		1979	Thailand	A/Bangkok/01/1979	ggtgatgccccattccttgatcggcttcgccgagatcag aggtccctaaggggaagaggcagcactc	-	-
CY114433	Human H3N2 IAVs	Human	H3N2		1979	Thailand	A/Bangkok/1/1979	ggtgatgccccattccttgatcggcttcgccgagatcag aggtccctaaggggaagaggcagcactc	-	-
CY121004	Human H3N2 IAVs	Human	H3N2		1979	Thailand	A/Bangkok/1/1979	ggtgatgccccattccttgatcggcttcgccgagatcag aggtccctaaggggaagaggcagcactc	-	-
CY006207	Human H3N2 IAVs	Human	H3N2		1980	China	A/Nanjing/13/1980	ggtgatgccccattccttgatcggcttcgccgagatcag aggtccctaagaggaagaggcagcactc	+	+
CY008664	Human H3N2 IAVs	Human	H3N2		1980	USA	A/Memphis/1/1980	ggtgatgccccattccttgatcggcttcgccgagatcag aggtccctaaggggaagaggcagcactc	-	-
CY008472	Human H3N2 IAVs	Human	H3N2		1980	USA	A/Memphis/3/1980	ggtgatgccccattccttgatcggcttcgccgagatcag aggtccctaaggggaagaggcagcactc	-	-
CY006895	Human H3N2 IAVs	Human	H3N2		1980	USA	A/Memphis/9/1980	ggtgatgccccattccttgatcggcttcgccgagatcag aggtccctaaggggaagaggcagcactc	-	-
CY007623	Human H3N2 IAVs	Human	H3N2		1980	USA	A/Memphis/4/1980	ggtgatgccccattccttgatcggcttcgccgagatcag aggtccctaaggggaagaggcagcactc	-	-
CY112357	Human H3N2 IAVs	Human	H3N2		1980	Netherlands	A/Rotterdam/577/1980	ggtgatgccccattccttgatcggcttcgccgagatcag aggtccctaaggggaagaggcagcactc	-	-
CY113273	Human H3N2 IAVs	Human	H3N2		1980	Netherlands	A/Netherlands/209/1980	ggtgatgccccattccttgatcggcttcgccgagatcag aggtccctaaggggaagaggcagcactc	-	-
CY009312	Human H3N2 IAVs	Swine	H3N2		1980	USA	A/swine/Wisconsin/194/1980	ggtgatgccccattccttgatcggcttcgccgagatcag aggtccctaaggggaagaggcagcactc	-	-
CY006103	Human H3N2 IAVs	Human	H3N2		1980	Hong_Kong	A/Hong_Kong/45/1980	ggtgatgccccatttcttgatcggcttcgccgagatcag aggtccctaagaggaagaggcagcactc	+	+
CY003492	Human H3N2 IAVs	Human	H3N2		1980	Hong_Kong	A/Hong_Kong/46/1980	ggtgatgccccatttcttgatcggcttcgccgagatcag aggtccctaagaggaagaggcagcactc	+	+
CY113281	Human H3N2 IAVs	Human	H3N2		1981	France	A/Lyon/2380/1981	ggtgatgccccattccttgatcggcttcgccgagacca gaggtccctaaggggaagaggcagcactc	-	-
CY077931	Human H3N2 IAVs	Swine	H3N2		1981	Italy	A/swine/Italy/6/1981	ggtgatgccccattccttgatcggcttcgccgagatcag aagtccctaaggggaagaggcagcactc	+	+
CY007631	Human H3N2 IAVs	Human	H3N2		1981	USA	A/Memphis/1/1981	ggtgatgccccattccttgatcggcttcgccgagatcag aggtccctaaggggaagaggcagcactc	-	-
CY112365	Human H3N2 IAVs	Human	H3N2		1981	Netherlands	A/Bilthoven/4791/1981	ggtgatgccccattccttgatcggcttcgccgagatcag aggtccctaaggggaagaggcagcactc	-	-
CY121249	Human H3N2 IAVs	Human	H3N2		1982	Philippines	A/Philippines/2/1982	ggtgatgccccattccttgatcggcttcgccgagacca gaggtccctaaggggaagaggcagcactc	-	-
CY113305	Human H3N2 IAVs	Human	H3N2		1982	Philippines	A/Philippines/2/1982	ggtgatgccccattccttgatcggcttcgccgagacca gaggtccctaaggggaagaggcagcactc	-	-
CY065980	Human H3N2 IAVs	Human	H3N2		1982	Philippines	A/Philippines/2_MA/1982	ggtgatgccccattccttgatcggcttcgccgagacca gaggtccctaaggggaagaggcagcactc	-	-
CY065972	Human H3N2 IAVs	Human	H3N2		1982	Philippines	A/Philippines/2/1982	ggtgatgccccattccttgatcggcttcgccgagacca gaggtccctaaggggaagaggcagcactc	-	-
CY077837	Human H3N2 IAVs	Human	H3N2		1982	Netherlands	A/Netherlands/233/1982	ggtgatgccccattccttgatcggcttcgccgagatcag aagtccctaaggggaagaggcagcactc	+	+

CY113297	Human H3N2 IAVs	Human	H3N2		1982	Netherlands	A/Netherlands/241/1982	ggtgatgccccattccttgatcggcttcgccgagatcag<br>aggtccctaaggggaagaggcaacactc	-	-
CY112375	Human H3N2 IAVs	Human	Mixed		1982	New_Zealand	A/Auckland/4382/1982	ggtgatgccccattccttgatcggcttcgccgagatcag<br>aggtccctaaggggaagaggcagcactc	-	-
CY113289	Human H3N2 IAVs	Human	H3N2		1982	Netherlands	A/Bilthoven/10684/1982	ggtgatgccccattccttgatcggcttcgccgagatcag<br>aggtccctaaggggaagaggcagcactc	-	-
CY114441	Human H3N2 IAVs	Human	H3N2		1982	Netherlands	A/Netherlands/233/1982	ggtgatgccccattccttgatcggcttcgccgagatcag<br>aggtccctaaggggaagaggcagcactc	-	-
CY006056	Human H3N2 IAVs	Human	H3N2		1982	Hong_Kong	A/Hong_Kong/1/1982	ggtgatgccccatttcttgatcggcttcgccgagatcag<br>aagtccctaagaggaagaggcagcactc	+	+
CY006759	Human H3N2 IAVs	Human	H3N2		1982	China	A/Nanjing/2/1982	ggtgatgccccatttcttgatcggcttcgccgagatcag<br>aggtccctaagaggaagaggcagcactc	+	+
CY006319	Human H3N2 IAVs	Human	H3N2		1983	Hong_Kong	A/Hong_Kong/14/1983	agtgatgccccattccttgatcggcttcgccgagatcag<br>aggtccctaaggggaagaggcagcactc	-	-
CY003724	Human H3N2 IAVs	Human	H3N2		1983	Hong_Kong	A/Hong_Kong/26/1983	agtgatgccccattccttgatcggcttcgccgagatcag<br>aggtccctaaggggaagaggcagcactc	-	-
CY009056	Human H3N2 IAVs	Human	H3N2		1983	USA	A/Memphis/33/1983	agtgatgccccattccttgatcggcttcgccgagatcag<br>aggtccctaaggggaagaggcagcactc	-	-
CY003740	Human H3N2 IAVs	Human	H3N2		1983	Hong_Kong	A/Hong_Kong/5/1983	agtgatgccccattccttgatcggcttcgccgagatcag<br>aggtccctaaggggaagaggcagcactc	-	-
CY113313	Human H3N2 IAVs	Human	H3N2		1983	Norway	A/Oslo/13676/1983	ggtgatgccccattccttgatcggcttcgccgagatcag<br>aggtccctaaggggaagaggcagcactc	-	-
CY006855	Human H3N2 IAVs	Human	H3N2		1983	China	A/Nanjing/36/1983	ggtgatgccccatttcttgatcggcttcgccgagatcag<br>aagtccctaagaggaagaggcagcactc	+	+
CY003748	Human H3N2 IAVs	Human	H3N2		1984	Hong_Kong	A/Hong_Kong/7/1984	agtgatgccccattccttgatcggattcgccgagatca<br>gaggtccctaaggggaagaggcaacactc	-	-
CY008176	Human H3N2 IAVs	Human	H3N2		1984	China	A/Nanjing/28/1984	agtgatgccccattccttgatcggcttcgccgagatcag<br>aggtccctaaggggaagaggcaacactc	-	-
CY006327	Human H3N2 IAVs	Human	H3N2		1984	Hong_Kong	A/Hong_Kong/4/1984	agtgatgccccattccttgatcggcttcgccgagatcag<br>aggtccctaaggggaagaggcagcactc	-	-
CY009072	Human H3N2 IAVs	Human	H3N2		1985	USA	A/Memphis/2/1985	agtgatgccccattccttgatagacttcgccgagatca<br>gaggtccctaaggggaagaggcagcactc	-	-
CY113337	Human H3N2 IAVs	Human	H3N2		1985	Netherlands	A/Netherlands/333/1985	agtgatgccccattccttgatagacttcgccgagatca<br>gaggtccctaaggggaagaggcagcactc	-	-
CY113321	Human H3N2 IAVs	Human	H3N2		1985	United_Kingdom	A/Guildford/V728/1985	agtgatgccccattccttgatcgacttcgccgagatca<br>gaggtccctaaggggaagaggcagcactc	+	+
CY113353	Human H3N2 IAVs	Human	H3N2		1985	New_Zealand	A/Wellington/4/1985	agtgatgccccattccttgatcgacttcgccgagatca<br>gaggtccctaaggggaagaggcagcactc	+	+
CY003524	Human H3N2 IAVs	Human	H3N2		1985	Hong_Kong	A/Hong_Kong/24/1985	agtgatgccccattccttgatcgacttcgccgagatca<br>gaggtccctaaggggaagaggcagcactc	+	+
CY113345	Human H3N2 IAVs	Human	H3N2		1985	Sweden	A/Stockholm/10/1985	agtgatgccccattccttgatcgacttcgccgagatca<br>gaggtccctaaggggaagaggcagcactc	+	+
CY008456	Human H3N2 IAVs	Human	H3N2		1985	USA	A/Memphis/5/1985	agtgatgccccattccttgatcgacttcgccgagatca<br>gaggtccctaaggggaagaggcagcactc	+	+
CY008712	Human H3N2 IAVs	Human	H3N2		1985	USA	A/Memphis/7/1985	agtgatgccccattccttgatcggcttcaccgagatca<br>gaggtccctaaggggaagaggcaacactc	-	-
CY003540	Human H3N2 IAVs	Human	H3N2		1985	Hong_Kong	A/Hong_Kong/7/1985	agtgatgccccattccttgatcggcttcgccgagatcag<br>aggtccctaaggggaagaggcaacactc	-	-
CY003508	Human H3N2 IAVs	Human	H3N2		1985	Hong_Kong	A/Hong_Kong/6/1985	agtgatgccccattccttgatcggcttcgccgagatcag<br>aggtccctaaggggaagaggcaacactc	-	-

CY011476	Human H3N2 IAVs	Human	H3N2		1985	USA	A/Memphis/12/1985	agtgatgccccattccttgatcggcttcgccgagatcag<br>aggtccctaaggggaagaggcagcaccc	-	-
CY008672	Human H3N2 IAVs	Human	H3N2		1985	USA	A/Memphis/25/1985	agtgatgccccattccttgatcggcttcgccgagatcag<br>aggtccctaaggggaagaggcagcaccc	-	-
CY113329	Human H3N2 IAVs	Human	H3N2		1985	Netherlands	A/Netherlands/330/1985	agtgatgccccattccttgatcggcttcgccgagatcag<br>aggtccctaaggggaagaggcagcaccc	-	-
CY121281	Human H3N2 IAVs	Human	H3N2		1986	Russia	A/Leningrad/360/1986	agcgatgccccattccttgatcgacttcgccgagatca<br>gaggtccctaaggggaagaggcagcactc	+	+
DQ508853	Human H3N2 IAVs	Human	H3N2		1986	Russia	A/Leningrad/360/1986	agcgatgccccattccttgatcgacttcgccgagatca<br>gaggtccctaaggggaagaggcagcactc	+	+
CY113361	Human H3N2 IAVs	Human	H3N2		1986	USA	A/Colorado/2/1986	agtgatgccccattccttgatcgacttcgcagagatca<br>gagatccctaaggggaagaggcagcactc	-	-
CY008720	Human H3N2 IAVs	Human	H3N2		1986	USA	A/Memphis/11/1986	agtgatgccccattccttgatcgacttcgccgagatca<br>gaagtccctaaggggaagaggcagcactc	+	+
CY002092	Human H3N2 IAVs	Human	H3N2		1986	USA	A/Memphis/66/1986	agtgatgccccattccttgatcgacttcgccgagatca<br>gaagtccctaaggggaagaggcagcactc	+	+
CY020337	Human H3N2 IAVs	Human	H3N2		1986	USA	A/Memphis/3/1986	agtgatgccccattccttgatcggcttcgccgagatcag<br>aggtccctaaggggaagaggcagcaccc	-	-
CY011292	Human H3N2 IAVs	Human	H3N2		1986	USA	A/Memphis/5/1986	agtgatgccccattccttgatcggcttcgccgagatcag<br>aggtccctaaggggaagaggcagcaccc	-	-
CY016064	Human H3N2 IAVs	Human	H3N2		1986	USA	A/Memphis/2/1986	agtgatgccccattccttgatcggcttcgccgagatcag<br>aggtccctaaggggaagaggcagcaccc	-	-
CY003548	Human H3N2 IAVs	Human	H3N2		1987	Hong_Kong	A/Hong_Kong/7/1987	agtgatgccccattccttgatcggcttcgccgagatcag<br>aggtccctaaggggaagaggcaacactc	-	-
M80975	Human H3N2 IAVs	Human	H3N2		1987	USA	A/Los_Angeles/2/1987	agtgatgccccattccttgatcggcttcgccgagatcag<br>aggtccctaaggggaagaggcaacactc	-	-
CY113369	Human H3N2 IAVs	Human	H3N2		1987	China	A/Shanghai/11/1987	agtgatgccccattccttgatcggcttcgccgagatcag<br>aggtccctaaggggaagaggcaacactc	-	-
CY121313	Human H3N2 IAVs	Human	H3N2		1987	China	A/Shanghai/11/1987	agtgatgccccattccttgatcggcttcgccgagatcag<br>aggtccctaaggggaagaggcaacactc	-	-
CY112400	Human H3N2 IAVs	Human	H3N2		1987	China	A/Sichuan/2/1987	agtgatgccccattccttgatcggcttcgccgagatcag<br>aggtccctaaggggaagaggcaacactc	-	-
CY121297	Human H3N2 IAVs	Human	H3N2		1987	China	A/Sichuan/2/1987	agtgatgccccattccttgatcggctttgccgagatcag<br>aggtccctaaggggaagaggcaacactc	-	-
CY116000	Human H3N2 IAVs	Swine	H3N2		1987	United_Kingdom	A/swine/England/163266/1987	ggtgacgccccattcctcgatcggcttcgccgagatca<br>gaagtcactaagggggagaagcagcactc	-	-
CY113393	Human H3N2 IAVs	Human	H3N2		1988	Netherlands	A/Netherlands/450/1988	agtgatgccccattccttgatcgacttcgccgagatca<br>gaggtccctaagaggaagaggcagcactc	+	+
CY096863	Human H3N2 IAVs	Human	H3N2		1988	USA	A/Memphis/3/1988	agtgatgccccattccttgatcggcttcgccgagacca<br>gaggtccctaaggggaagaggcaacactc	-	-
CY114449	Human H3N2 IAVs	Human	H3N2		1988	Australia	A/Victoria/1/1988	agtgatgccccattccttgatcggcttcgccgagatcag<br>aggtccctaaggagaagaggcaacactc	-	*
CY113377	Human H3N2 IAVs	Human	H3N2		1988	USA	A/Atlanta/3572/1988	agtgatgccccattccttgatcggcttcgccgagatcag<br>aggtccctaaggggaagaggcaacactc	-	-
CY114457	Human H3N2 IAVs	Human	H3N2		1988	USA	A/Oklahoma/5/1988	agtgatgccccattccttgatcggcttcgccgagatcag<br>aggtccctaaggggaagaggcaacactc	-	-
CY008728	Human H3N2 IAVs	Human	H3N2		1988	USA	A/Memphis/5/1988	agtgatgccccattccttgatcggcttcgccgagatcag<br>aggtccctaaggggaagaggcaacactc	-	-
CY113401	Human H3N2 IAVs	Human	H3N2		1988	Sweden	A/Stockholm/12/1988	agtgatgccccattccttgatcggcttcgccgagatcag<br>aggtccctaaggggaagaggcaacactc	-	-

CY003516	Human H3N2 IAVs	Human	H3N2		1988	Hong_Kong	A/Hong_Kong/2/1988	agtgatgccccattccttgatcggcttcgccgagatcag<br>aggtccctaaggggaagaggcaacactc	-	-
CY035202	Human H3N2 IAVs	Human	H3N2		1988	Italy	A/Siena/3/1988	agtgatgccccattccttgatcggcttcgccgagatcag<br>aggtccctaaggggaagaggcaacactc	-	-
CY113385	Human H3N2 IAVs	Human	H3N2		1988	United_Kingdom	A/England/427/1988	agtgatgccccattccttgatcggcttcgccgagatcag<br>aggtccctaaggggaagaggcaacactc	-	-
CY008736	Human H3N2 IAVs	Human	H3N2		1988	USA	A/Memphis/15/1988	ggtgatgccccattccttgatcggcttcgccgagatcag<br>aggtccctaaggggaagaggaaacactc	+	+
CY003356	Human H3N2 IAVs	Human	H3N2		1988	USA	A/Memphis/13/1988	ggtgatgccccattccttgatcggcttcgccgagatcag<br>aggtccctaaggggaagaggcaacactc	-	-
CY010760	Human H3N2 IAVs	Human	H3N2		1988	USA	A/Memphis/8/1988	ggtgatgccccattccttgatcggcttcgccgagatcag<br>aggtccctaaggggaagaggcaacactc	-	-
CY113417	Human H3N2 IAVs	Human	H3N2		1989	United_Kingdom	A/England/138/1989	aatgatgccccattccttgatcggcttcgccgagatca<br>gaggtccctaaggggaagaggcaacactc	-	-
CY112416	Human H3N2 IAVs	Human	H3N2		1989	Netherlands	A/Eindhoven/3447/1989	agtgatgccccattccttgatcgacttcgccgagatca<br>gaggtccctaagaggaagaggcagcactc	+	+
CY112408	Human H3N2 IAVs	Human	H3N2		1989	China	A/Beijing/352/1989	agtgatgccccattccttgatcggcttcgccgagatcag<br>agatccctaaggggaagaggcaacactc	-	*
CY121028	Human H3N2 IAVs	Human	H3N2		1989	China	A/Beijing/353/1989	agtgatgccccattccttgatcggcttcgccgagatcag<br>agatccctaaggggaagaggcaacactc	-	*
CY114465	Human H3N2 IAVs	Human	H3N2		1989	China	A/Beijing/353/1989	agtgatgccccattccttgatcggcttcgccgagatcag<br>agatccctaaggggaagaggcaacactc	-	*
DQ508837	Human H3N2 IAVs	Human	H3N2		1989	China	A/Beijing/353/1989	agtgatgccccattccttgatcggcttcgccgagatcag<br>agatccctaaggggaagaggcaacactc	-	*
CY113409	Human H3N2 IAVs	Human	H3N2		1989	USA	A/Atlanta/211/1989	agtgatgccccattccttgatcggcttcgccgagatcag<br>aggtccctaaggggaagaggcaacactc	-	-
CY113433	Human H3N2 IAVs	Human	H3N2		1989	Netherlands	A/Netherlands/650/1989	agtgatgccccattccttgatcggcttcgccgagatcag<br>aggtccctaaggggaagaggcaacactc	-	-
CY113441	Human H3N2 IAVs	Human	H3N2		1989	Netherlands	A/Netherlands/738/1989	agtgatgccccattccttgatcggcttcgccgagatcag<br>aggtccctaaggggaagaggcaacactc	-	-
CY112424	Human H3N2 IAVs	Human	H3N2		1989	Switzerland	A/Geneva/5007/1989	agtgatgccccattccttgatcggcttcgccgagatcag<br>aggtccctaaggggaagaggcaacactc	-	-
CY113457	Human H3N2 IAVs	Human	H3N2		1989	Singapore	A/Singapore/34/1989	agtgatgccccattccttgatcggcttcgccgagatcag<br>aggtccctaaggggaagaggcaacactc	-	-
CY113465	Human H3N2 IAVs	Human	H3N2		1989	Singapore	A/Singapore/35/1989	agtgatgccccattccttgatcggcttcgccgagatcag<br>aggtccctaaggggaagaggcaacactc	-	-
CY114129	Human H3N2 IAVs	Human	H3N2		1989	Singapore	A/Singapore/36/1989	agtgatgccccattccttgatcggcttcgccgagatcag<br>aggtccctaaggggaagaggcaacactc	-	-
CY113473	Human H3N2 IAVs	Human	H3N2		1989	Singapore	A/Singapore/40/1989	agtgatgccccattccttgatcggcttcgccgagatcag<br>aggtccctaaggggaagaggcaacactc	-	-
CY113497	Human H3N2 IAVs	Human	H3N2		1989	New_Zealand	A/Waikato/1/1989	agtgatgccccattccttgatcggcttcgccgagatcag<br>aggtccctaaggggaagaggcaacactc	-	-
CY113489	Human H3N2 IAVs	Human	H3N2		1989	New_Zealand	A/Wellington/5/1989	agtgatgccccattccttgatcggcttcgccgagatcag<br>aggtccctaaggggaagaggcaacactc	-	-
CY112440	Human H3N2 IAVs	Human	H3N2		1989	Singapore	A/Singapore/53/1989	agtgatgccccattccttgatcggcttcgccgagatcag<br>aggtccctaaggggaagaggcaacactc	-	-
CY112432	Human H3N2 IAVs	Human	H3N2		1989	Hong_Kong	A/Hong_Kong/1/1989	agtgatgccccattccttgatcggcttcgccgagatcag<br>aggtccctaaggggaagaggcaacactc	-	-
CY077858	Human H3N2 IAVs	Human	H3N2		1989	Netherlands	A/Netherlands/620/1989	agtgatgccccattccttgatcggcttcgccgagatcag<br>aggtccttaaggggaagaggcaacactc	+	+

CY113425	Human H3N2 IAVs	Human	H3N2		1989	Netherlands	A/Netherlands/620/1989	agtgatgccccattccttgatcggcttcgccgagatcag<br>aggtccttaaggggaagaggcaacactc	+	+
CY113537	Human H3N2 IAVs	Human	H3N2		1990	Australia	A/Victoria/2/1990	agtgatgccccattccttgatcggcttcgccgagatcag<br>agatccctaaggggaagaggcaacactc	-	*
CY113545	Human H3N2 IAVs	Human	H3N2		1990	New_Zealand	A/Wellington/3/1990	agtgatgccccattccttgatcggcttcgccgagatcag<br>agatccctaaggggaagaggcaacactc	-	*
CY008744	Human H3N2 IAVs	Human	H3N2		1990	USA	A/Memphis/7/1990	agtgatgccccattccttgatcggcttcgccgagatcag<br>aggtccctaaggggaagaggcaacactc	-	-
CY113529	Human H3N2 IAVs	Human	H3N2		1990	Japan	A/Suita/1/1990	agtgatgccccattccttgatcggcttcgccgagatcag<br>aggtccctaaggggaagaggcaacactc	-	-
CY035210	Human H3N2 IAVs	Human	H3N2		1990	Italy	A/Siena/4/1990	agtgatgccccattccttgatcggcttcgccgagatcag<br>aggtccctaaggggaagaggcaacactc	-	-
CY035218	Human H3N2 IAVs	Human	H3N2		1990	Italy	A/Siena/10/1990	agtgatgccccattccttgatcggcttcgccgagatcag<br>aggtccctaaggggaagaggcaacactc	-	-
CY113505	Human H3N2 IAVs	Human	H3N2		1990	USA	A/Memphis/2/1990	agtgatgccccattccttgatcggcttcgccgagatcag<br>aggtccctaaggggaagaggcaacactc	-	-
CY113513	Human H3N2 IAVs	Human	H3N2		1990	USA	A/Memphis/5/1990	agtgatgccccattccttgatcggcttcgccgagatcag<br>aggtccctaaggggaagaggcaacactc	-	-
CY011484	Human H3N2 IAVs	Human	H3N2		1990	USA	A/Memphis/2/1990	agtgatgccccattccttgatcggcttcgccgagatcag<br>aggtccctaaggggaagaggcaacactc	-	-
CY113521	Human H3N2 IAVs	Human	H3N2		1990	China	A/Shanghai/24/1990	agtgatgccccattccttgatcggcttcgccgagatcag<br>aggtccctaaggggaagaggcaacactc	-	-
CY116319	Human H3N2 IAVs	Swine	H3N2		1990	United_Kingdom	A/swine/England/87842/1990	ggtgacgccccattcctcgatcggcttcgccgagatca<br>gaagtcactaagggggagaagcagcactc	-	-
CY113793	Human H3N2 IAVs	Human	H3N2		1991	France	A/Paris/325/1991	agtgatgccccattcctcgatcggcttcgccgagatca<br>gagatccctaaggggaagaggcaacactc	-	-
CY113801	Human H3N2 IAVs	Human	H3N2		1991	France	A/Paris/407/1991	agtgatgccccattcctcgatcggcttcgccgagatca<br>gagatccctaaggggaagaggcaacactc	-	-
CY113825	Human H3N2 IAVs	Human	H3N2		1991	France	A/Paris/457/1991	agtgatgccccattcctcgatcggcttcgccgagatca<br>gagatccctaaggggaagaggcaacactc	-	-
CY113857	Human H3N2 IAVs	Human	H3N2		1991	France	A/Paris/564/1991	agtgatgccccattcctcgatcggcttcgccgagatca<br>gagatccctaaggggaagaggcaacactc	-	-
CY113873	Human H3N2 IAVs	Human	H3N2		1991	France	A/Paris/597/1991	agtgatgccccattcctcgatcggcttcgccgagatca<br>gagatccctaaggggaagaggcaacactc	-	-
CY113817	Human H3N2 IAVs	Human	H3N2		1991	France	A/Paris/424/1991	agtgatgccccattcctcgatcggcttcgccgagatca<br>gagatccctaaggggaagaggcaacactc	-	-
CY113841	Human H3N2 IAVs	Human	H3N2		1991	France	A/Paris/512/1991	agtgatgccccattcctcgatcggcttcgccgagatca<br>gagatccctaaggggaagaggcaacactc	-	-
CY113785	Human H3N2 IAVs	Human	H3N2		1991	France	A/Paris/320/1991	agtgatgccccattcctcgatcggcttcgccgagatca<br>gagatccctaaggggaagaggcaacactc	-	-
CY036835	Human H3N2 IAVs	Human	H3N2		1991	Italy	A/Siena/1/1991	agtgatgccccattccttgaccggcttcgccgagatca<br>gagatccctaaggggaagaggcaacactc	-	-
CY035234	Human H3N2 IAVs	Human	H3N2		1991	Italy	A/Siena/6/1991	agtgatgccccattccttgaccggcttcgccgagatca<br>gagatccctaaggggaagaggcaacactc	-	-
CY035226	Human H3N2 IAVs	Human	H3N2		1991	Italy	A/Siena/5/1991	agtgatgccccattccttgaccggcttcgccgagatca<br>gagatccctaaggggaagaggcaacactc	-	-
CY113569	Human H3N2 IAVs	Human	H3N2		1991	Switzerland	A/Geneva/5366/1991	agtgatgccccattccttgatcggcttcgccgagacca<br>gagatccctaaggggaagaggcaacactc	-	-
CY113657	Human H3N2 IAVs	Human	H3N2		1991	Spain	A/Madrid/G12/1991	agtgatgccccattccttgatcggcttcgccgagatcag<br>aaatccctaaggggaagaggcaacactc	-	-

CY113777	Human H3N2 IAVs	Human	H3N2		1991	Spain	A/Oviedo/31/1991	agtgatgccccattccttgatcggcttcgccgagatcag aaatccctaaggggaagaggcaacactc	-	-
CY113665	Human H3N2 IAVs	Human	H3N2		1991	Netherlands	A/Netherlands/816/1991	agtgatgccccattccttgatcggcttcgccgagatcag aaatccctaaggggaagaggcaacactc	-	-
CY113673	Human H3N2 IAVs	Human	H3N2		1991	Netherlands	A/Netherlands/891/1991	agtgatgccccattccttgatcggcttcgccgagatcag aaatccctaaggggaagaggcaacactc	-	-
CY113929	Human H3N2 IAVs	Human	H3N2		1991	Netherlands	A/Tilburg/5957/1991	agtgatgccccattccttgatcggcttcgccgagatcag aaatccctaaggggaagaggcaacactc	-	-
CY113561	Human H3N2 IAVs	Human	H3N2		1991	United_Kingdom	A/England/260/1991	agtgatgccccattccttgatcggcttcgccgagatcag agatccctaaggggaagaggcaacactc	-	*
CY114473	Human H3N2 IAVs	Human	H3N2		1991	United_Kingdom	A/United_Kingdom/261/1991	agtgatgccccattccttgatcggcttcgccgagatcag agatccctaaggggaagaggcaacactc	-	*
CY113833	Human H3N2 IAVs	Human	H3N2		1991	France	A/Paris/467/1991	agtgatgccccattccttgatcggcttcgccgagatcag agatccctaaggggaagaggcaacactc	-	*
CY112448	Human H3N2 IAVs	Human	H3N2		1991	Switzerland	A/Geneva/6447/1991	agtgatgccccattccttgatcggcttcgccgagatcag agatccctaaggggaagaggcaacactc	-	*
CY113585	Human H3N2 IAVs	Human	H3N2		1991	France	A/Lyon/1182/1991	agtgatgccccattccttgatcggcttcgccgagatcag agatccctaaggggaagaggcaacactc	-	*
CY113601	Human H3N2 IAVs	Human	H3N2		1991	France	A/Lyon/1276/1991	agtgatgccccattccttgatcggcttcgccgagatcag agatccctaaggggaagaggcaacactc	-	*
CY113609	Human H3N2 IAVs	Human	H3N2		1991	France	A/Lyon/1337/1991	agtgatgccccattccttgatcggcttcgccgagatcag agatccctaaggggaagaggcaacactc	-	*
CY113625	Human H3N2 IAVs	Human	H3N2		1991	France	A/Lyon/1594/1991	agtgatgccccattccttgatcggcttcgccgagatcag agatccctaaggggaagaggcaacactc	-	*
CY113633	Human H3N2 IAVs	Human	H3N2		1991	France	A/Lyon/23672/1991	agtgatgccccattccttgatcggcttcgccgagatcag agatccctaaggggaagaggcaacactc	-	*
CY113641	Human H3N2 IAVs	Human	H3N2		1991	France	A/Lyon/24103/1991	agtgatgccccattccttgatcggcttcgccgagatcag agatccctaaggggaagaggcaacactc	-	*
CY113809	Human H3N2 IAVs	Human	H3N2		1991	France	A/Paris/417/1991	agtgatgccccattccttgatcggcttcgccgagatcag agatccctaaggggaagaggcaacactc	-	*
CY113849	Human H3N2 IAVs	Human	H3N2		1991	France	A/Paris/548/1991	agtgatgccccattccttgatcggcttcgccgagatcag agatccctaaggggaagaggcaacactc	-	*
CY113593	Human H3N2 IAVs	Human	H3N2		1991	France	A/Lyon/1189/1991	agtgatgccccattccttgatcggcttcgccgagatcag agatccctaaggggaagaggcaacactc	-	*
CY113617	Human H3N2 IAVs	Human	H3N2		1991	France	A/Lyon/1373/1991	agtgatgccccattccttgatcggcttcgccgagatcag agatccctaaggggaagaggcaacactc	-	*
CY112504	Human H3N2 IAVs	Human	H3N2		1991	France	A/Paris/490/1991	agtgatgccccattccttgatcggcttcgccgagatcag agatccctaaggggaagaggcaacactc	-	*
CY113577	Human H3N2 IAVs	Human	H3N2		1991	France	A/Lyon/1149/1991	agtgatgccccattccttgatcggcttcgccgagatcag agatccctaaggggaagaggcaacactc	-	*
CY113649	Human H3N2 IAVs	Human	H3N2		1991	France	A/Lyon/24222/1991	agtgatgccccattccttgatcggcttcgccgagatcag agatccctaaggggaagaggcaacactc	-	*
CY043732	Human H3N2 IAVs	Human	H3N2		1991	Italy	A/Siena/3/1991	agtgatgccccattccttgatcggcttcgccgagatcag agatccctaaggggaagaggcaacactc	-	*
CY112456	Human H3N2 IAVs	Human	H3N2		1991	Sweden	A/Stockholm/20/1991	agtgatgccccattccttgatcggcttcgccgagatcag agatccctaaggggaagaggcaacactc	-	*
CY113553	Human H3N2 IAVs	Human	H3N2		1991	Australia	A/Canberra/1/1991	agtgatgccccattccttgatcggcttcgccgagatcag aggtccctaaggggaagaggcaacactc	-	-
CY113753	Human H3N2 IAVs	Human	H3N2		1991	Netherlands	A/Netherlands/938/1991	agtgatgccccattccttgatcggcttcgccgagatcag aggtccctaaggggaagaggcaacactc	-	-

CY113713	Human H3N2 IAVs	Human	H3N2		1992	USA	A/Houston/56941/1992	agtgatgccccattcctcgatcggcttcgccgagatca<br>gagatccctaaggggaagaggcaacactc	-	-
CY112464	Human H3N2 IAVs	Human	H3N2		1992	Netherlands	A/Amsterdam/4112/1992	agtgatgccccattccttgatcggcttcgccgagatcag<br>aaatccctaaggggaagaggcaacactc	-	-
CY113721	Human H3N2 IAVs	Human	H3N2		1992	Spain	A/Madrid/G58/1992	agtgatgccccattccttgatcggcttcgccgagatcag<br>aaatccctaaggggaagaggcaacactc	-	-
CY113769	Human H3N2 IAVs	Human	H3N2		1992	Netherlands	A/Nijmegen/3129/1992	agtgatgccccattccttgatcggcttcgccgagatcag<br>aaatccctaaggggaagaggcaacactc	-	-
CY113729	Human H3N2 IAVs	Human	H3N2		1992	Netherlands	A/Netherlands/819/1992	agtgatgccccattccttgatcggcttcgccgagatcag<br>agatccctaaggggaagaggcaacactc	-	*
CY113881	Human H3N2 IAVs	Human	H3N2		1992	France	A/Paris/614/1992	agtgatgccccattccttgatcggcttcgccgagatcag<br>agatccctaaggggaagaggcaacactc	-	*
CY113865	Human H3N2 IAVs	Human	H3N2		1992	France	A/Paris/583/1992	agtgatgccccattccttgatcggcttcgccgagatcag<br>agatccctaaggggaagaggcaacactc	-	*
CY113905	Human H3N2 IAVs	Human	H3N2		1992	Sweden	A/Stockholm/7/1992	agtgatgccccattccttgatcggcttcgccgagatcag<br>agatccctaaggggaagaggcaacactc	-	*
CY113937	Human H3N2 IAVs	Human	H3N2		1992	Sweden	A/Umea/1982/1992	agtgatgccccattccttgatcggcttcgccgagatcag<br>agatccctaaggggaagaggcaacactc	-	*
CY113945	Human H3N2 IAVs	Human	H3N2		1992	Sweden	A/Umea/2000/1992	agtgatgccccattccttgatcggcttcgccgagatcag<br>agatccctaaggggaagaggcaacactc	-	*
CY112472	Human H3N2 IAVs	Human	H3N2		1992	Netherlands	A/Enschede/1285/1992	agtgatgccccattccttgatcggcttcgccgagatcag<br>agatccctaaggggaagaggcaacactc	-	*
CY113745	Human H3N2 IAVs	Human	H3N2		1992	Netherlands	A/Netherlands/935/1992	agtgatgccccattccttgatcggcttcgccgagatcag<br>agatccctaaggggaagaggcaacactc	-	*
CY113921	Human H3N2 IAVs	Human	H3N2		1992	Sweden	A/Stockholm/13/1992	agtgatgccccattccttgatcggcttcgccgagatcag<br>agatccctaaggggaagaggcaacactc	-	*
CY113953	Human H3N2 IAVs	Human	H3N2		1992	Australia	A/Victoria/68/1992	agtgatgccccattccttgatcggcttcgccgagatcag<br>agatccctaaggggaagaggcaacactc	-	*
CY003716	Human H3N2 IAVs	Human	H3N2		1992	Hong_Kong	A/Hong_Kong/14/1992	agtgatgccccattccttgatcggcttcgccgagatcag<br>agatccctaaggggaagaggcaacactc	-	*
CY112544	Human H3N2 IAVs	Human	H3N2		1992	Australia	A/Victoria/33/1992	agtgatgccccattccttgatcggcttcgccgagatcag<br>agatccctaaggggaagaggcaacactc	-	*
CY112480	Human H3N2 IAVs	Human	H3N2		1992	Finland	A/Finland/218/1992	agtgatgccccattccttgatcggcttcgccgagatcag<br>agatccctaaggggaagaggcaacactc	-	*
CY113689	Human H3N2 IAVs	Human	H3N2		1992	Switzerland	A/Geneva/5113/1992	agtgatgccccattccttgatcggcttcgccgagatcag<br>agatccctaaggggaagaggcaacactc	-	*
CY113697	Human H3N2 IAVs	Human	H3N2		1992	USA	A/Houston/56798/1992	agtgatgccccattccttgatcggcttcgccgagatcag<br>agatccctaaggggaagaggcaacactc	-	*
CY113737	Human H3N2 IAVs	Human	H3N2		1992	Netherlands	A/Netherlands/823/1992	agtgatgccccattccttgatcggcttcgccgagatcag<br>agatccctaaggggaagaggcaacactc	-	*
CY112512	Human H3N2 IAVs	Human	H3N2		1992	Netherlands	A/Rotterdam/100540/1992	agtgatgccccattccttgatcggcttcgccgagatcag<br>agatccctaaggggaagaggcaacactc	-	*
CY113889	Human H3N2 IAVs	Human	H3N2		1992	Australia	A/South_Austalia/23/1992	agtgatgccccattccttgatcggcttcgccgagatcag<br>agatccctaaggggaagaggcaacactc	-	*
CY113897	Human H3N2 IAVs	Human	H3N2		1992	Australia	A/South_Austalia/27/1992	agtgatgccccattccttgatcggcttcgccgagatcag<br>agatccctaaggggaagaggcaacactc	-	*
CY112528	Human H3N2 IAVs	Human	H3N2		1992	Australia	A/South_Austalia/8/1992	agtgatgccccattccttgatcggcttcgccgagatcag<br>agatccctaaggggaagaggcaacactc	-	*
CY113705	Human H3N2 IAVs	Human	H3N2		1992	USA	A/Houston/56829/1992	agtgatgccccattccttgatcggcttcgccgagatcag<br>agatccctaaggggaagaggcaacactc	-	*

CY077877	Human H3N2 IAVs	Human	H3N2		1992	Netherlands	A/Netherlands/823/1992	agtgatgccccattccttgatcggcttcgccgagatcag<br>agatccctaaggggaagaggcaacactc	-	*
CY113761	Human H3N2 IAVs	Human	H3N2		1992	Netherlands	A/Nijmegen/3126/1992	agtgatgccccattccttgatcggcttcgccgagatcag<br>agatccctaaggggaagaggcaacactc	-	*
CY112488	Human H3N2 IAVs	Human	H3N2		1992	Finland	A/Finland/220/1992	agtgatgccccattccttgatcggcttcgccgagatcag<br>agatccctaaggggaagaggcaacactc	-	*
CY112536	Human H3N2 IAVs	Human	H3N2		1992	Sweden	A/Stockholm/8/1992	agtgatgccccattccttgatcggcttcgccgagatcag<br>agatccctaaggggaagaggcaacactc	-	*
CY121329	Human H3N2 IAVs	Human	H3N2		1992	China	A/Harbin/15/1992	agtgatgccccattccttgatcggcttcgccgagatcag<br>aggtccctaaggggaagaggcaacactc	-	-
CY113681	Human H3N2 IAVs	Human	H3N2		1992	China	A/Beijing/32/1992	agtgatgccccattccttgatcggcttcgccgagatcag<br>aggtccctaaggggaagaggcaacactc	-	-
CY033610	Human H3N2 IAVs	Human	H3N2		1992	China	A/Beijing/32/1992	agtgatgccccattccttgatcggcttcgccgagatcag<br>aggtccctaaggggaagaggcaacactc	-	-
CY112496	Human H3N2 IAVs	Human	H3N2		1992	Finland	A/Finland/247/1992	agtgatgccccattccttgatcggcttcgccgagatcag<br>aggtccctaaggggaagaggcaacactc	-	-
CY112520	Human H3N2 IAVs	Human	H3N2		1992	Japan	A/Sendai/C273/1992	agtgatgccccattccttgatcggcttcgccgagatcag<br>aggtccctaaggggaagaggcaacactc	-	-
CY113913	Human H3N2 IAVs	Human	H3N2		1992	Sweden	A/Stockholm/12/1992	agtgatgccccattccttgatcggcttcgccgagatcag<br>aggtccctaaggggaagaggcaacactc	-	-
CY112585	Human H3N2 IAVs	Human	H3N2		1992	Netherlands	A/Netherlands/3/1992	agtgatgccccattccttgatcggcttcgccgagatcag<br>aggtccctaaggggaagaggcgacactc	-	-
CY114041	Human H3N2 IAVs	Human	H3N2		1993	Spain	A/Madrid/G252/1993	aatgatgccccattccttgatcggcttcgccgagatca<br>gaggtccctaaggggaagaggcaacactc	-	-
CY112552	Human H3N2 IAVs	Human	H3N2		1993	France	A/Lyon/1803/1993	agtgatgccccattccttgatcggcttcgccgagatcag<br>agatcactaaggggaagaggcaacactc	-	*
CY114065	Human H3N2 IAVs	Human	H3N2		1993	Netherlands	A/Netherlands/165/1993	agtgatgccccattccttgatcggcttcgccgagatcag<br>agatccctaaggggaagaggcaacactc	-	*
CY114073	Human H3N2 IAVs	Human	H3N2		1993	Netherlands	A/Netherlands/357/1993	agtgatgccccattccttgatcggcttcgccgagatcag<br>agatccctaaggggaagaggcaacactc	-	*
CY114105	Human H3N2 IAVs	Human	H3N2		1993	United_Kingdom	A/Scotland/142/1993	agtgatgccccattccttgatcggcttcgccgagatcag<br>agatccctaaggggaagaggcaacactc	-	*
CY112633	Human H3N2 IAVs	Human	H3N2		1993	Netherlands	A/Netherlands/399/1993	agtgatgccccattccttgatcggcttcgccgagatcag<br>agatccctaaggggaagaggcaacactc	-	*
CY112681	Human H3N2 IAVs	Human	H3N2		1993	Australia	A/Victoria/104/1993	agtgatgccccattccttgatcggcttcgccgagatcag<br>agatccctaaggggaagaggcaacactc	-	*
CY112881	Human H3N2 IAVs	Human	H3N2		1993	Norway	A/Oslo/2352/1993	agtgatgccccattccttgatcggcttcgccgagatcag<br>agatccctaaggggaagaggcaacactc	-	*
CY114017	Human H3N2 IAVs	Human	H3N2		1993	Spain	A/Madrid/G101/1993	agtgatgccccattccttgatcggcttcgccgagatcag<br>agatccctaaggggaagaggcaacactc	-	*
CY114033	Human H3N2 IAVs	Human	H3N2		1993	Spain	A/Madrid/G116/1993	agtgatgccccattccttgatcggcttcgccgagatcag<br>agatccctaaggggaagaggcaacactc	-	*
CY114097	Human H3N2 IAVs	Human	H3N2		1993	France	A/Paris/287/1993	agtgatgccccattccttgatcggcttcgccgagatcag<br>agatccctaaggggaagaggcaacactc	-	*
CY113969	Human H3N2 IAVs	Human	H3N2		1993	Netherlands	A/Enschede/5458/1993	agtgatgccccattccttgatcggcttcgccgagatcag<br>agatccctaaggggaagaggcaacactc	-	*
CY013673	Human H3N2 IAVs	Human	H3N2		1993	USA	A/New_York/780/1993	agtgatgccccattccttgatcggcttcgccgagatcag<br>aggtccctaaggggaagaggcaacaccc	+	+
CY113961	Human H3N2 IAVs	Human	H3N2		1993	Japan	A/Akita/4/1993	agtgatgccccattccttgatcggcttcgccgagatcag<br>aggtccctaaggggaagaggcaacactc	-	-

CY113985	Human H3N2 IAVs	Human	H3N2		1993	France	A/Lyon/672/1993	agtgatgccccattccttgatcggcttcgccgagatcag<br>aggtccctaaggggaagaggcaacactc	-	-
CY013196	Human H3N2 IAVs	Human	H3N2		1993	USA	A/New_York/782/1993	agtgatgccccattccttgatcggcttcgccgagatcag<br>aggtccctaaggggaagaggcaacactc	-	-
CY114057	Human H3N2 IAVs	Human	H3N2		1993	Netherlands	A/Netherlands/126/1993	agtgatgccccattccttgatcggcttcgccgagatcag<br>aggtccctaaggggaagaggcaacactc	-	-
CY013172	Human H3N2 IAVs	Human	H3N2		1993	USA	A/New_York/754/1993	agtgatgccccattccttgatcggcttcgccgagatcag<br>aggtccctaaggggaagaggcaacactc	-	-
CY012740	Human H3N2 IAVs	Human	H3N2		1993	USA	A/New_York/770/1993	agtgatgccccattccttgatcggcttcgccgagatcag<br>aggtccctaaggggaagaggcaacactc	-	-
CY112601	Human H3N2 IAVs	Human	H3N2		1993	Netherlands	A/Netherlands/115/1993	agtgatgccccattccttgatcggcttcgccgagatcag<br>aggtccctaaggggaagaggcaacactc	-	-
CY112569	Human H3N2 IAVs	Human	H3N2		1993	Spain	A/Madrid/G102/1993	agtgatgccccattccttgatcggcttcgccgagatcag<br>aggtccctaaggggaagaggcaacactc	-	-
CY114121	Human H3N2 IAVs	Human	H3N2		1993	China	A/Shangdong/9/1993	agtgatgccccattccttgatcggcttcgccgagatcag<br>aggtccctaaggggaagaggcaacactc	-	-
CY036907	Human H3N2 IAVs	Human	H3N2		1993	China	A/Shangdong/9/1993	agtgatgccccattccttgatcggcttcgccgagatcag<br>aggtccctaaggggaagaggcaacactc	-	-
CY113449	Human H3N2 IAVs	Human	H3N2		1993	Singapore	A/Singapore/3/1993	agtgatgccccattccttgatcggcttcgccgagatcag<br>aggtccctaaggggaagaggcaacactc	-	-
CY114049	Human H3N2 IAVs	Human	H3N2		1993	Netherlands	A/Netherlands/101/1993	agtgatgccccattccttgatcggcttcgccgagatcag<br>aggtccctaaggggaagaggcaacactc	-	-
CY012148	Human H3N2 IAVs	Human	H3N2		1993	USA	A/New_York/760/1993	agtgatgccccattccttgatcggcttcgccgagatcag<br>aggtccctaaggggaagaggcaacactc	-	-
CY013737	Human H3N2 IAVs	Human	H3N2		1993	USA	A/New_York/794/1993	agtgatgccccattccttgatcggcttcgccgagatcag<br>aggtccctaaggggaagaggcaacactc	-	-
CY011948	Human H3N2 IAVs	Human	H3N2		1993	USA	A/New_York/752/1993	agtgatgccccattccttgatcggcttcgccgagatcag<br>aggtccctaaggggaagaggcaacactc	-	-
CY113977	Human H3N2 IAVs	Human	H3N2		1993	China	A/Guangdong/25/1993	agtgatgccccattccttgatcggcttcgccgagatcag<br>aggtccctaaggggaagaggcaacactc	-	-
CY112560	Human H3N2 IAVs	Human	H3N2		1993	France	A/Lyon/22686/1993	agtgatgccccattccttgatcggcttcgccgagatcag<br>aggtccctaaggggaagaggcaacactc	-	-
CY114009	Human H3N2 IAVs	Human	H3N2		1993	Spain	A/Madrid/G122/1993	agtgatgccccattccttgatcggcttcgccgagatcag<br>aggtccctaaggggaagaggcaacactc	-	-
CY077803	Human H3N2 IAVs	Human	H3N2		1993	Netherlands	A/Netherlands/179/1993	agtgatgccccattccttgatcggcttcgccgagatcag<br>aggtccctaaggggaagaggcaacactc	-	-
CY112617	Human H3N2 IAVs	Human	H3N2		1993	Netherlands	A/Netherlands/241/1993	agtgatgccccattccttgatcggcttcgccgagatcag<br>aggtccctaaggggaagaggcaacactc	-	-
CY012124	Human H3N2 IAVs	Human	H3N2		1993	USA	A/New_York/756/1993	agtgatgccccattccttgatcggcttcgccgagatcag<br>aggtccctaaggggaagaggcaacactc	-	-
CY012140	Human H3N2 IAVs	Human	H3N2		1993	USA	A/New_York/759/1993	agtgatgccccattccttgatcggcttcgccgagatcag<br>aggtccctaaggggaagaggcaacactc	-	-
CY013665	Human H3N2 IAVs	Human	H3N2		1993	USA	A/New_York/776/1993	agtgatgccccattccttgatcggcttcgccgagatcag<br>aggtccctaaggggaagaggcaacactc	-	-
CY012772	Human H3N2 IAVs	Human	H3N2		1993	USA	A/New_York/778/1993	agtgatgccccattccttgatcggcttcgccgagatcag<br>aggtccctaaggggaagaggcaacactc	-	-
CY012780	Human H3N2 IAVs	Human	H3N2		1993	USA	A/New_York/779/1993	agtgatgccccattccttgatcggcttcgccgagatcag<br>aggtccctaaggggaagaggcaacactc	-	-
CY013212	Human H3N2 IAVs	Human	H3N2		1993	USA	A/New_York/785/1993	agtgatgccccattccttgatcggcttcgccgagatcag<br>aggtccctaaggggaagaggcaacactc	-	-

CY013689	Human H3N2 IAVs	Human	H3N2		1993	USA	A/New_York/787/1993	agtgatgccccattccttgatcggcttcgccgagatcag aggtccctaaggggaagaggcaacactc	-	-
CY013729	Human H3N2 IAVs	Human	H3N2		1993	USA	A/New_York/792/1993	agtgatgccccattccttgatcggcttcgccgagatcag aggtccctaaggggaagaggcaacactc	-	-
CY014147	Human H3N2 IAVs	Human	H3N2		1993	USA	A/New_York/793/1993	agtgatgccccattccttgatcggcttcgccgagatcag aggtccctaaggggaagaggcaacactc	-	-
CY013745	Human H3N2 IAVs	Human	H3N2		1993	USA	A/New_York/795/1993	agtgatgccccattccttgatcggcttcgccgagatcag aggtccctaaggggaagaggcaacactc	-	-
CY014155	Human H3N2 IAVs	Human	H3N2		1993	USA	A/New_York/796/1993	agtgatgccccattccttgatcggcttcgccgagatcag aggtccctaaggggaagaggcaacactc	-	-
CY013753	Human H3N2 IAVs	Human	H3N2		1993	USA	A/New_York/797/1993	agtgatgccccattccttgatcggcttcgccgagatcag aggtccctaaggggaagaggcaacactc	-	-
CY013761	Human H3N2 IAVs	Human	H3N2		1993	USA	A/New_York/798/1993	agtgatgccccattccttgatcggcttcgccgagatcag aggtccctaaggggaagaggcaacactc	-	-
CY013769	Human H3N2 IAVs	Human	H3N2		1993	USA	A/New_York/799/1993	agtgatgccccattccttgatcggcttcgccgagatcag aggtccctaaggggaagaggcaacactc	-	-
CY016487	Human H3N2 IAVs	Human	H3N2		1993	USA	A/New_York/800/1993	agtgatgccccattccttgatcggcttcgccgagatcag aggtccctaaggggaagaggcaacactc	-	-
CY013777	Human H3N2 IAVs	Human	H3N2		1993	USA	A/New_York/801/1993	agtgatgccccattccttgatcggcttcgccgagatcag aggtccctaaggggaagaggcaacactc	-	-
CY013785	Human H3N2 IAVs	Human	H3N2		1993	USA	A/New_York/802/1993	agtgatgccccattccttgatcggcttcgccgagatcag aggtccctaaggggaagaggcaacactc	-	-
CY013793	Human H3N2 IAVs	Human	H3N2		1993	USA	A/New_York/803/1993	agtgatgccccattccttgatcggcttcgccgagatcag aggtccctaaggggaagaggcaacactc	-	-
CY114113	Human H3N2 IAVs	Human	H3N2		1993	United_Kingdom	A/Scotland/160/1993	agtgatgccccattccttgatcggcttcgccgagatcag aggtccctaaggggaagaggcaacactc	-	-
CY013633	Human H3N2 IAVs	Human	H3N2		1993	USA	A/New_York/769/1993	agtgatgccccattccttgatcggcttcgccgagatcag aggtccctaaggggaagaggcaacactc	-	-
CY011876	Human H3N2 IAVs	Human	H3N2		1993	USA	A/New_York/712/1993	agtgatgccccattccttgatcggcttcgccgagatcag aggtccctaaggggaagaggcaacactc	-	-
CY011580	Human H3N2 IAVs	Human	H3N2		1993	USA	A/New_York/751/1993	agtgatgccccattccttgatcggcttcgccgagatcag aggtccctaaggggaagaggcaacactc	-	-
CY012172	Human H3N2 IAVs	Human	H3N2		1993	USA	A/New_York/763/1993	agtgatgccccattccttgatcggcttcgccgagatcag aggtccctaaggggaagaggcaacactc	-	-
CY012180	Human H3N2 IAVs	Human	H3N2		1993	USA	A/New_York/764/1993	agtgatgccccattccttgatcggcttcgccgagatcag aggtccctaaggggaagaggcaacactc	-	-
CY012188	Human H3N2 IAVs	Human	H3N2		1993	USA	A/New_York/765/1993	agtgatgccccattccttgatcggcttcgccgagatcag aggtccctaaggggaagaggcaacactc	-	-
CY013641	Human H3N2 IAVs	Human	H3N2		1993	USA	A/New_York/772/1993	agtgatgccccattccttgatcggcttcgccgagatcag aggtccctaaggggaagaggcaacactc	-	-
CY012764	Human H3N2 IAVs	Human	H3N2		1993	USA	A/New_York/777/1993	agtgatgccccattccttgatcggcttcgccgagatcag aggtccctaaggggaagaggcaacactc	-	-
CY013188	Human H3N2 IAVs	Human	H3N2		1993	USA	A/New_York/781/1993	agtgatgccccattccttgatcggcttcgccgagatcag aggtccctaaggggaagaggcaacactc	-	-
CY112609	Human H3N2 IAVs	Human	H3N2		1993	Netherlands	A/Netherlands/179/1993	agtgatgccccattccttgatcggcttcgccgagatcag aggtccctaaggggaagaggcaacactc	-	-
CY112673	Human H3N2 IAVs	Human	H3N2		1993	Sweden	A/Stockholm/20/1993	agtgatgccccattccttgatcggcttcgccgagatcag aggtccctaaggggaagaggcaacactc	-	-
CY113993	Human H3N2 IAVs	Human	H3N2		1993	France	A/Lyon/1815/1993	agtgatgccccattccttgatcggcttcgccgagatcag aggtccctaaggggaagaggcaacactc	-	-

CY114089	Human H3N2 IAVs	Human	H3N2		1993	Netherlands	A/Netherlands/398/1993	agtgatgccccattccttgatcggcttcgccgagatcag<br>aggtccctaaggggaagaggcaacactc	-	-
CY013625	Human H3N2 IAVs	Human	H3N2		1993	USA	A/New_York/768/1993	agtgatgccccattccttgatcggcttcgccgagatcag<br>aggtccctaaggggaagaggcaacactc	-	-
CY013657	Human H3N2 IAVs	Human	H3N2		1993	USA	A/New_York/774/1993	agtgatgccccattccttgatcggcttcgccgagatcag<br>aggtccctaaggggaagaggcaacactc	-	-
CY012748	Human H3N2 IAVs	Human	H3N2		1993	USA	A/New_York/771/1993	agtgatgccccattccttgatcggcttcgccgagatcag<br>aggtccctaaggggaagaggcaacactc	-	-
CY013681	Human H3N2 IAVs	Human	H3N2		1993	USA	A/New_York/784/1993	agtgatgccccattccttgatcggcttcgccgagatcag<br>aggtccctaaggggaagaggcaacactc	-	-
CY013180	Human H3N2 IAVs	Human	H3N2		1993	USA	A/New_York/755/1993	agtgatgccccattccttgatcggcttcgccgagatcag<br>aggtccctaaggggaagaggcaacactc	-	-
CY012756	Human H3N2 IAVs	Human	H3N2		1993	USA	A/New_York/775/1993	agtgatgccccattccttgatcggcttcgccgagatcag<br>aggtccctaaggggaagaggcaacactc	-	-
CY012788	Human H3N2 IAVs	Human	H3N2		1993	USA	A/New_York/786/1993	agtgatgccccattccttgatcggcttcgccgagatcag<br>aggtccctaaggggaagaggcaacactc	-	-
CY012732	Human H3N2 IAVs	Human	H3N2		1993	USA	A/New_York/767/1993	agtgatgccccattccttgatcggcttcgccgagatcag<br>aggtccctaaggggaagaggcaacactc	-	-
CY013649	Human H3N2 IAVs	Human	H3N2		1993	USA	A/New_York/773/1993	agtgatgccccattccttgatcggcttcgccgagatcag<br>aggtccctaaggggaagaggcaacactc	-	-
CY013705	Human H3N2 IAVs	Human	H3N2		1993	USA	A/New_York/789/1993	agtgatgccccattccttgatcggcttcgccgagatcag<br>aggtccctaaggggaagaggcaacactc	-	-
CY016431	Human H3N2 IAVs	Human	H3N2		1993	USA	A/New_York/706/1993	agtgatgccccattccttgatcggcttcgccgagatcag<br>aggtccctaaggggaagaggcaacactc	-	-
CY013609	Human H3N2 IAVs	Human	H3N2		1993	USA	A/New_York/757/1993	agtgatgccccattccttgatcggcttcgccgagatcag<br>aggtccctaaggggaagaggcaacactc	-	-
CY013713	Human H3N2 IAVs	Human	H3N2		1993	USA	A/New_York/790/1993	agtgatgccccattccttgatcggcttcgccgagatcag<br>aggtccctaaggggaagaggcaacactc	-	-
CY114001	Human H3N2 IAVs	Human	H3N2		1993	France	A/Lyon/23602/1993	agtgatgccccattccttgatcggcttcgccgagatcag<br>aggtccctaaggggaagaggcaacactc	-	-
CY112625	Human H3N2 IAVs	Human	H3N2		1993	Netherlands	A/Netherlands/371/1993	agtgatgccccattccttgatcggcttcgccgagatcag<br>aggtccctaaggggaagaggcaacactc	-	-
CY114081	Human H3N2 IAVs	Human	H3N2		1993	Netherlands	A/Netherlands/372/1993	agtgatgccccattccttgatcggcttcgccgagatcag<br>aggtccctaaggggaagaggcaacactc	-	-
CY012164	Human H3N2 IAVs	Human	H3N2		1993	USA	A/New_York/762/1993	agtgatgccccattccttgatcggcttcgccgagatcag<br>aggtccctaaggggaagaggcaacactc	-	-
CY013617	Human H3N2 IAVs	Human	H3N2		1993	USA	A/New_York/766/1993	agtgatgccccattccttgatcggcttcgccgagatcag<br>aggtccctaaggggaagaggcaacactc	-	-
CY013721	Human H3N2 IAVs	Human	H3N2		1993	USA	A/New_York/791/1993	agtgatgccccattccttgatcggcttcgccgagatcag<br>aggtccctaaggggaagaggcaacactc	-	-
CY114025	Human H3N2 IAVs	Human	H3N2		1993	Spain	A/Madrid/G109/1993	agtgatgccccattccttgatcggcttcgccgagatcag<br>aggtccctaaggggaagaggcaacactc	-	-
CY112641	Human H3N2 IAVs	Human	H3N2		1993	Norway	A/Oslo/2219/1993	agtgatgccccattccttgatcggcttcgccgagatcag<br>aggtccctaaggggaagaggcaacactc	-	-
CY112689	Human H3N2 IAVs	Human	H3N2		1993	New_Zealand	A/Wellington/59/1993	agtgatgccccattccttgatcggcttcgccgagatcag<br>aggtccctaaggggaagaggcaacactc	-	-
CY012156	Human H3N2 IAVs	Human	H3N2		1993	USA	A/New_York/761/1993	agtgatgccccattccttgatcggcttcgccgagatcag<br>aggtccctaaggggaagaggcaacactc	-	-
CY013204	Human H3N2 IAVs	Human	H3N2		1993	USA	A/New_York/783/1993	agtgatgccccattccttgatcggcttcgccgagatcag<br>aggtccctaaggggaagaggcaacactc	-	-

CY013697	Human H3N2 IAVs	Human	H3N2		1993	USA	A/New_York/788/1993	agtgatgccccattccttgatcggcttcgccgagatcag<br>aggtccctaaggggaagaggcaacactc	-	-
CY112665	Human H3N2 IAVs	Human	H3N2		1993	Singapore	A/Singapore/19/1993	agtgatgccccattccttgatcggcttcgccgagatcag<br>aggtccctaaggggaagaggcaacactc	-	-
CY009016	Human H3N2 IAVs	Human	H3N2		1993	China	A/Nanchang/12/1993	agtgatgccccattccttgatcggcttcgccgagatcag<br>aggtccctaaggggaagaggcaacactc	-	-
CY006351	Human H3N2 IAVs	Human	H3N2		1993	China	A/Nanchang/58/1993	agtgatgccccattccttgatcggcttcgccgagatcag<br>aggtccctaaggggaagaggcaacactc	-	-
CY114137	Human H3N2 IAVs	Human	H3N2		1993	Japan	A/Yamagata/56/1993	agtgatgccccattccttgatcggcttcgccgagatcag<br>aggtccctaaggggaagaggcaacactc	-	-
CY114145	Human H3N2 IAVs	Human	H3N2		1993	Japan	A/Yamagata/61/1993	agtgatgccccattccttgatcggcttcgccgagatcag<br>aggtccctaaggggaagaggcaacactc	-	-
CY112697	Human H3N2 IAVs	Human	H3N2		1993	Japan	A/Yamagata/62/1993	agtgatgccccattccttgatcggcttcgccgagatcag<br>aggtccctaaggggaagaggcaacactc	-	-
CY112657	Human H3N2 IAVs	Human	H3N2		1993	Japan	A/Shiga/6/1993	agtgatgccccattccttgatcggcttcgccgagatcag<br>aggtccctaaggggaagaggcaacactc	-	-
CY012132	Human H3N2 IAVs	Human	H3N2		1993	USA	A/New_York/758/1993	agtgatgccccattccttgatcggcttcgccgagatcag<br>aggtccctaaggggaagaggcaacactc	-	-
CY112593	Human H3N2 IAVs	Human	H3N2		1993	Netherlands	A/Netherlands/17/1993	agtgatgccccattccttgatcggcttcgccgagatcag<br>aggtccctaaggggaagaggcgacactc	-	-
CY112577	Human H3N2 IAVs	Human	H3N2		1993	Spain	A/Madrid/G130/1993	agtgatgccccattccttgatcggmttcgccgagatca<br>gaggtccctaaggggaagaggcaacactc	-	-
CY011932	Human H3N2 IAVs	Human	H3N2		1994	USA	A/New_York/727/1994	agtgatgccccattcctcgatcggcttcgccgagatca<br>gaggtccctaaggggaagaggcaacactc	-	-
CY012452	Human H3N2 IAVs	Human	H3N2		1994	USA	A/New_York/655/1994	agtgatgccccattccttgatcggcttcgcagagatca<br>gaggtccctaaggggaagaggcaacactc	-	-
CY012532	Human H3N2 IAVs	Human	H3N2		1994	USA	A/New_York/705/1994	agtgatgccccattccttgatcggcttcgccgagatcag<br>agatccctaaggggaagaggcaacactc	-	*
CY112761	Human H3N2 IAVs	Human	H3N2		1994	Australia	A/South_Austalia/25/1994	agtgatgccccattccttgatcggcttcgccgagatcag<br>aggtcactaagaggaagaggcaacactc	+	+
CY112753	Human H3N2 IAVs	Human	H3N2		1994	Australia	A/South_Austalia/15/1994	agtgatgccccattccttgatcggcttcgccgagatcag<br>aggtcactaaggggaagaggcaacactc	+	+
CY013891	Human H3N2 IAVs	Human	H3N2		1994	USA	A/New_York/717/1994	agtgatgccccattccttgatcggcttcgccgagatcag<br>aggtccctaagaggaagaggcaacactc	-	-
CY012996	Human H3N2 IAVs	Human	H3N2		1994	USA	A/New_York/739/1994	agtgatgccccattccttgatcggcttcgccgagatcag<br>aggtccctaagaggaagaggcaacactc	-	-
U65670	Human H3N2 IAVs	Human	H3N2		1994	Japan	A/Akita/1/94	agtgatgccccattccttgatcggcttcgccgagatcag<br>aggtccctaaggggaagaggcaacactc	-	-
CY112705	Human H3N2 IAVs	Human	H3N2		1994	United_Kingdom	A/England/7/1994	agtgatgccccattccttgatcggcttcgccgagatcag<br>aggtccctaaggggaagaggcaacactc	-	-
CY011916	Human H3N2 IAVs	Human	H3N2		1994	USA	A/New_York/721/1994	agtgatgccccattccttgatcggcttcgccgagatcag<br>aggtccctaaggggaagaggcaacactc	-	-
CY011388	Human H3N2 IAVs	Human	H3N2		1994	USA	A/New_York/732/1994	agtgatgccccattccttgatcggcttcgccgagatcag<br>aggtccctaaggggaagaggcaacactc	-	-
CY017311	Human H3N2 IAVs	Human	H3N2		1994	USA	A/New_York/704/1994	agtgatgccccattccttgatcggcttcgccgagatcag<br>aggtccctaaggggaagaggcaacactc	-	-
CY012292	Human H3N2 IAVs	Human	H3N2		1994	USA	A/New_York/734/1994	agtgatgccccattccttgatcggcttcgccgagatcag<br>aggtccctaaggggaagaggcaacactc	-	-
CY013347	Human H3N2 IAVs	Human	H3N2		1994	USA	A/New_York/742/1994	agtgatgccccattccttgatcggcttcgccgagatcag<br>aggtccctaaggggaagaggcaacactc	-	-

CY013355	Human H3N2 IAVs	Human	H3N2		1994	USA	A/New_York/743/1994	agtgatgccccattccttgatcggcttcgccgagatcag aggtccctaaggggaagaggcaacactc	-	-
CY012284	Human H3N2 IAVs	Human	H3N2		1994	USA	A/New_York/730/1994	agtgatgccccattccttgatcggcttcgccgagatcag aggtccctaaggggaagaggcaacactc	-	-
CY112737	Human H3N2 IAVs	Human	H3N2		1994	South_Africa	A/Johannesburg/33/1994	agtgatgccccattccttgatcggcttcgccgagatcag aggtccctaaggggaagaggcaacactc	-	-
CY112745	Human H3N2 IAVs	Human	H3N2		1994	South_Africa	A/Johannesburg/47/1994	agtgatgccccattccttgatcggcttcgccgagatcag aggtccctaaggggaagaggcaacactc	-	-
CY077809	Human H3N2 IAVs	Human	H3N2		1994	Netherlands	A/Netherlands/18/1994	agtgatgccccattccttgatcggcttcgccgagatcag aggtccctaaggggaagaggcaacactc	-	-
CY114161	Human H3N2 IAVs	Human	H3N2		1994	Netherlands	A/Netherlands/18/1994	agtgatgccccattccttgatcggcttcgccgagatcag aggtccctaaggggaagaggcaacactc	-	-
CY012540	Human H3N2 IAVs	Human	H3N2		1994	USA	A/New_York/707/1994	agtgatgccccattccttgatcggcttcgccgagatcag aggtccctaaggggaagaggcaacactc	-	-
CY012548	Human H3N2 IAVs	Human	H3N2		1994	USA	A/New_York/710/1994	agtgatgccccattccttgatcggcttcgccgagatcag aggtccctaaggggaagaggcaacactc	-	-
CY012948	Human H3N2 IAVs	Human	H3N2		1994	USA	A/New_York/723/1994	agtgatgccccattccttgatcggcttcgccgagatcag aggtccctaaggggaagaggcaacactc	-	-
CY013012	Human H3N2 IAVs	Human	H3N2		1994	USA	A/New_York/741/1994	agtgatgccccattccttgatcggcttcgccgagatcag aggtccctaaggggaagaggcaacactc	-	-
CY121345	Human H3N2 IAVs	Human	H3N2		1994	South_Africa	A/Johannesburg/33/1994	agtgatgccccattccttgatcggcttcgccgagatcag aggtccctaaggggaagaggcaacactc	-	-
CY011380	Human H3N2 IAVs	Human	H3N2		1994	USA	A/New_York/708/1994	agtgatgccccattccttgatcggcttcgccgagatcag aggtccctaaggggaagaggcaacactc	-	-
CY011868	Human H3N2 IAVs	Human	H3N2		1994	USA	A/New_York/711/1994	agtgatgccccattccttgatcggcttcgccgagatcag aggtccctaaggggaagaggcaacactc	-	-
CY012556	Human H3N2 IAVs	Human	H3N2		1994	USA	A/New_York/713/1994	agtgatgccccattccttgatcggcttcgccgagatcag aggtccctaaggggaagaggcaacactc	-	-
CY012268	Human H3N2 IAVs	Human	H3N2		1994	USA	A/New_York/722/1994	agtgatgccccattccttgatcggcttcgccgagatcag aggtccctaaggggaagaggcaacactc	-	-
CY012964	Human H3N2 IAVs	Human	H3N2		1994	USA	A/New_York/725/1994	agtgatgccccattccttgatcggcttcgccgagatcag aggtccctaaggggaagaggcaacactc	-	-
CY013339	Human H3N2 IAVs	Human	H3N2		1994	USA	A/New_York/731/1994	agtgatgccccattccttgatcggcttcgccgagatcag aggtccctaaggggaagaggcaacactc	-	-
CY010992	Human H3N2 IAVs	Human	H3N2		1994	USA	A/New_York/733/1994	agtgatgccccattccttgatcggcttcgccgagatcag aggtccctaaggggaagaggcaacactc	-	-
CY015544	Human H3N2 IAVs	Human	H3N2		1994	USA	A/New_York/737/1994	agtgatgccccattccttgatcggcttcgccgagatcag aggtccctaaggggaagaggcaacactc	-	-
CY012988	Human H3N2 IAVs	Human	H3N2		1994	USA	A/New_York/738/1994	agtgatgccccattccttgatcggcttcgccgagatcag aggtccctaaggggaagaggcaacactc	-	-
CY012572	Human H3N2 IAVs	Human	H3N2		1994	USA	A/New_York/746/1994	agtgatgccccattccttgatcggcttcgccgagatcag aggtccctaaggggaagaggcaacactc	-	-
CY012588	Human H3N2 IAVs	Human	H3N2		1994	USA	A/New_York/748/1994	agtgatgccccattccttgatcggcttcgccgagatcag aggtccctaaggggaagaggcaacactc	-	-
CY112721	Human H3N2 IAVs	Human	H3N2		1994	Hong_Kong	A/Hong_Kong/2/1994	agtgatgccccattccttgatcggcttcgccgagatcag aggtccctaaggggaagaggcaacactc	-	-
CY012228	Human H3N2 IAVs	Human	H3N2		1994	USA	A/New_York/657/1994	agtgatgccccattccttgatcggcttcgccgagatcag aggtccctaaggggaagaggcaacactc	-	-
CY012596	Human H3N2 IAVs	Human	H3N2		1994	USA	A/New_York/749/1994	agtgatgccccattccttgatcggcttcgccgagatcag aggtccctaaggggaagaggcaacactc	-	-

CY013020	Human H3N2 IAVs	Human	H3N2		1994	USA	A/New_York/750/1994	agtgatgccccattccttgatcggcttcgccgagatcag aggtccctaaggggaagaggcaacactc	-	-
CY012940	Human H3N2 IAVs	Human	H3N2		1994	USA	A/New_York/718/1994	agtgatgccccattccttgatcggcttcgccgagatcag aggtccctaaggggaagaggcaacactc	-	-
CY011900	Human H3N2 IAVs	Human	H3N2		1994	USA	A/New_York/719/1994	agtgatgccccattccttgatcggcttcgccgagatcag aggtccctaaggggaagaggcaacactc	-	-
CY011908	Human H3N2 IAVs	Human	H3N2		1994	USA	A/New_York/720/1994	agtgatgccccattccttgatcggcttcgccgagatcag aggtccctaaggggaagaggcaacactc	-	-
CY013004	Human H3N2 IAVs	Human	H3N2		1994	USA	A/New_York/740/1994	agtgatgccccattccttgatcggcttcgccgagatcag aggtccctaaggggaagaggcaacactc	-	-
CY012956	Human H3N2 IAVs	Human	H3N2		1994	USA	A/New_York/724/1994	agtgatgccccattccttgatcggcttcgccgagatcag aggtccctaaggggaagaggcaacactc	-	-
CY012580	Human H3N2 IAVs	Human	H3N2		1994	USA	A/New_York/747/1994	agtgatgccccattccttgatcggcttcgccgagatcag aggtccctaaggggaagaggcaacactc	-	-
CY011940	Human H3N2 IAVs	Human	H3N2		1994	USA	A/New_York/728/1994	agtgatgccccattccttgatcggcttcgccgagatcag aggtccctaaggggaagaggcaacactc	-	-
CY012276	Human H3N2 IAVs	Human	H3N2		1994	USA	A/New_York/729/1994	agtgatgccccattccttgatcggcttcgccgagatcag aggtccctaaggggaagaggcaacactc	-	-
CY012980	Human H3N2 IAVs	Human	H3N2		1994	USA	A/New_York/736/1994	agtgatgccccattccttgatcggcttcgccgagatcag aggtccctaaggggaagaggcaacactc	-	-
CY011924	Human H3N2 IAVs	Human	H3N2		1994	USA	A/New_York/726/1994	agtgatgccccattccttgatcggcttcgccgagatcag aggtccctaaggggaagaggcaacactc	-	-
CY013028	Human H3N2 IAVs	Human	H3N2		1994	USA	A/New_York/753/1994	agtgatgccccattccttgatcggcttcgccgagatcag aggtccctaaggggaagaggcaacactc	-	-
CY013371	Human H3N2 IAVs	Human	H3N2		1994	USA	A/New_York/745/1994	agtgatgccccattccttgatcggcttcgccgagatcag aggtccctaaggggaagaggcaacactc	-	-
CY013363	Human H3N2 IAVs	Human	H3N2		1994	USA	A/New_York/744/1994	agtgatgccccattccttgatcggcttcgccgagatcag aggtccctaaggggaagaggcaacactc	-	-
CY112713	Human H3N2 IAVs	Human	H3N2		1994	Hong_Kong	A/Hong_Kong/1/1994	agtgatgccccattccttgatcggcttcgccgagatcag aggtccctaaggggaagaggcaacactc	-	-
CY112729	Human H3N2 IAVs	Human	H3N2		1994	Hong_Kong	A/Hong_Kong/55/1994	agtgatgccccattccttgatcggcttcgccgagatcag aggtccctaaggggaagaggcaacactc	-	-
CY011892	Human H3N2 IAVs	Human	H3N2		1994	USA	A/New_York/716/1994	agtgatgccccattccttgatcggcttcgccgagatcag aggtccctaaggggaagaggcaacactc	-	-
CY012972	Human H3N2 IAVs	Human	H3N2		1994	USA	A/New_York/735/1994	agtgatgccccattccttgatcggcttcgccgagatcag aggtccctaaggggaagaggcaacactc	-	-
CY011340	Human H3N2 IAVs	Human	H3N2		1994	USA	A/New_York/696/1994	agtgatgccccattccttgatcggcttcgccgagatcag aggtccctaaggggaagaggcaacactc	-	-
CY011508	Human H3N2 IAVs	Human	H3N2		1994	USA	A/New_York/659/1994	agtgatgccccattccttgatcggcttcgccgagatcag aggtccctaaggggaagaggcaacactc	-	-
CY012460	Human H3N2 IAVs	Human	H3N2		1994	USA	A/New_York/661/1994	agtgatgccccattccttgatcggcttcgccgagatcag aggtccctaaggggaagaggcaacactc	-	-
CY011524	Human H3N2 IAVs	Human	H3N2		1994	USA	A/New_York/665/1994	agtgatgccccattccttgatcggcttcgccgagatcag aggtccctaaggggaagaggcaacactc	-	-
CY013331	Human H3N2 IAVs	Human	H3N2		1994	USA	A/New_York/688/1994	agtgatgccccattccttgatcggcttcgccgagatcag aggtccctaaggggaagaggcaacactc	-	-
CY012468	Human H3N2 IAVs	Human	H3N2		1994	USA	A/New_York/663/1994	agtgatgccccattccttgatcggcttcgccgagatcag aggtccctaaggggaagaggcaacactc	-	-
CY114153	Human H3N2 IAVs	Human	H3N2		1994	Hong_Kong	A/Hong_Kong/56/1994	agtgatgccccattccttgatcggcttcgccgagatcag aggtccctaaggggaagaggcaacactc	-	-

CY011884	Human H3N2 IAVs	Human	H3N2		1994	USA	A/New_York/715/1994	agtgatgccccattccttgatcggcttcgccgagatcag<br>aggtccctaaggggaagaggcaacactc	-	-
CY011860	Human H3N2 IAVs	Human	H3N2		1994	USA	A/New_York/709/1994	agtgatgccccattccttgatcggcttcgccgagatcag<br>aggtccctaaggggaagaggcaacactc	-	-
CY012564	Human H3N2 IAVs	Human	H3N2		1994	USA	A/New_York/714/1994	agtgatgccccattccttgatcggcttcgccgagatcag<br>aggtccctaaggggaagaggcaacactc	-	-
CY006343	Human H3N2 IAVs	Human	H3N2		1994	China	A/Nanchang/0058/1994	agtgatgccccattccttgatcggcttcgccgagatcag<br>aggtccctaaggggaagaggcaacactc	-	-
CY003756	Human H3N2 IAVs	Human	H3N2		1994	China	A/Nanchang/0074/1994	agtgatgccccattccttgatcggcttcgccgagatcag<br>aggtccctaaggggaagaggcaacactc	-	-
CY006335	Human H3N2 IAVs	Human	H3N2		1994	China	A/Nanchang/A2/1994	agtgatgccccattccttgatcggcttcgccgagatcag<br>aggtccctaaggggaagaggcaacactc	-	-
CY007839	Human H3N2 IAVs	Human	H3N2		1994	China	A/Nanchang/A1/1994	agtgatgccccattccttgatcggcttcgccgagatcag<br>aggtccctaaggggaagaggcaacactc	-	-
CY011492	Human H3N2 IAVs	Human	H3N2		1994	USA	A/New_York/654/1994	agtgatgccccattccttgatcggcttcgccgagatcag<br>aggtccctaaggggaagaggcaacactc	-	-
CY011364	Human H3N2 IAVs	Human	H3N2		1995	USA	A/New_York/702/1995	agtgacgccccattccttgatcggcttcgccgagatca<br>gaggtccctaaggggaagaggcaacactc	+	+
U65671	Human H3N2 IAVs	Human	H3N2		1995	Japan	A/Akita/1/95	agtgatgccccattcattgatcggcttcgccgagatca<br>gaggtccctaaggggaagaggcaacactc	-	-
CY012220	Human H3N2 IAVs	Human	H3N2		1995	USA	A/New_York/639/1995	agtgatgccccattccttgatcggcttcgccgagatcag<br>agatccctaaggggaagaggcaacactc	-	*
CY010728	Human H3N2 IAVs	Human	H3N2		1995	USA	A/New_York/634/1995	agtgatgccccattccttgatcggcttcgccgagatcag<br>agatccctaaggggaagaggcaacactc	-	*
CY114169	Human H3N2 IAVs	Human	H3N2		1995	Finland	A/Finland/338/1995	agtgatgccccattccttgatcggcttcgccgagatcag<br>aggtccctaaggggaagaggcaacactc	-	-
CY112809	Human H3N2 IAVs	Human	H3N2		1995	Sweden	A/Stockholm/5/1995	agtgatgccccattccttgatcggcttcgccgagatcag<br>aggtccctaaggggaagaggcaacactc	-	-
CY114193	Human H3N2 IAVs	Human	H3N2		1995	Switzerland	A/Geneva/A9509/1995	agtgatgccccattccttgatcggcttcgccgagatcag<br>aggtccctaaggggaagaggcaacactc	-	-
CY002276	Human H3N2 IAVs	Human	H3N2		1995	USA	A/Memphis/24/1995	agtgatgccccattccttgatcggcttcgccgagatcag<br>aggtccctaaggggaagaggcaacactc	-	-
CY011324	Human H3N2 IAVs	Human	H3N2		1995	USA	A/New_York/692/1995	agtgatgccccattccttgatcggcttcgccgagatcag<br>aggtccctaaggggaagaggcaacactc	-	-
CY013323	Human H3N2 IAVs	Human	H3N2		1995	USA	A/New_York/676/1995	agtgatgccccattccttgatcggcttcgccgagatcag<br>aggtccctaaggggaagaggcaacactc	-	-
CY011516	Human H3N2 IAVs	Human	H3N2		1995	USA	A/New_York/662/1995	agtgatgccccattccttgatcggcttcgccgagatcag<br>aggtccctaaggggaagaggcaacactc	-	-
CY012500	Human H3N2 IAVs	Human	H3N2		1995	USA	A/New_York/668/1995	agtgatgccccattccttgatcggcttcgccgagatcag<br>aggtccctaaggggaagaggcaacactc	-	-
CY011556	Human H3N2 IAVs	Human	H3N2		1995	USA	A/New_York/674/1995	agtgatgccccattccttgatcggcttcgccgagatcag<br>aggtccctaaggggaagaggcaacactc	-	-
CY012508	Human H3N2 IAVs	Human	H3N2		1995	USA	A/New_York/678/1995	agtgatgccccattccttgatcggcttcgccgagatcag<br>aggtccctaaggggaagaggcaacactc	-	-
CY011844	Human H3N2 IAVs	Human	H3N2		1995	USA	A/New_York/687/1995	agtgatgccccattccttgatcggcttcgccgagatcag<br>aggtccctaaggggaagaggcaacactc	-	-
CY012932	Human H3N2 IAVs	Human	H3N2		1995	USA	A/New_York/699/1995	agtgatgccccattccttgatcggcttcgccgagatcag<br>aggtccctaaggggaagaggcaacactc	-	-
CY011372	Human H3N2 IAVs	Human	H3N2		1995	USA	A/New_York/703/1995	agtgatgccccattccttgatcggcttcgccgagatcag<br>aggtccctaaggggaagaggcaacactc	-	-

CY012524	Human H3N2 IAVs	Human	H3N2		1995	USA	A/New_York/700/1995	agtgatgccccattccttgatcggcttcgccgagatcag aggtccctaaggggaagaggcaacactc	-	-
U65672	Human H3N2 IAVs	Human	H3N2		1995	China	A/Hebei/19/95	agtgatgccccattccttgatcggcttcgccgagatcag aggtccctaaggggaagaggcaacactc	-	-
CY017303	Human H3N2 IAVs	Human	H3N2		1995	USA	A/New_York/701/1995	agtgatgccccattccttgatcggcttcgccgagatcag aggtccctaaggggaagaggcaacactc	-	-
AF038275	Human H3N2 IAVs	Human	H3N2		1995	Japan	A/Tochigi/44/95	agtgatgccccattccttgatcggcttcgccgagatcag aggtccctaaggggaagaggcaacactc	-	-
CY013315	Human H3N2 IAVs	Human	H3N2		1995	USA	A/New_York/660/1995	agtgatgccccattccttgatcggcttcgccgagatcag aggtccctaaggggaagaggcaacactc	-	-
CY011540	Human H3N2 IAVs	Human	H3N2		1995	USA	A/New_York/671/1995	agtgatgccccattccttgatcggcttcgccgagatcag aggtccctaaggggaagaggcaacactc	-	-
CY118422	Human H3N2 IAVs	Human	H3N2		1995	Malaysia	A/Malaysia/07145/1995	agtgatgccccattccttgatcggcttcgccgagatcag aggtccctaaggggaagaggcaacactc	-	-
CY112777	Human H3N2 IAVs	Human	H3N2		1995	Hong_Kong	A/Hong_Kong/38/1995	agtgatgccccattccttgatcggcttcgccgagatcag aggtccctaaggggaagaggcaacactc	-	-
CY114201	Human H3N2 IAVs	Human	H3N2		1995	Hong_Kong	A/Hong_Kong/3/1995	agtgatgccccattccttgatcggcttcgccgagatcag aggtccctaaggggaagaggcaacactc	-	-
CY114217	Human H3N2 IAVs	Human	H3N2		1995	France	A/Lyon/2279/1995	agtgatgccccattccttgatcggcttcgccgagatcag aggtccctaaggggaagaggcaacactc	-	-
CY011500	Human H3N2 IAVs	Human	H3N2		1995	USA	A/New_York/658/1995	agtgatgccccattccttgatcggcttcgccgagatcag aggtccctaaggggaagaggcaacactc	-	-
CY010624	Human H3N2 IAVs	Human	H3N2		1995	USA	A/New_York/606/1995	agtgatgccccattccttgatcggcttcgccgagatcag aggtccctaaggggaagaggcaacactc	-	-
CY010648	Human H3N2 IAVs	Human	H3N2		1995	USA	A/New_York/610/1995	agtgatgccccattccttgatcggcttcgccgagatcag aggtccctaaggggaagaggcaacactc	-	-
CY011812	Human H3N2 IAVs	Human	H3N2		1995	USA	A/New_York/647/1995	agtgatgccccattccttgatcggcttcgccgagatcag aggtccctaaggggaagaggcaacactc	-	-
CY010752	Human H3N2 IAVs	Human	H3N2		1995	USA	A/New_York/648/1995	agtgatgccccattccttgatcggcttcgccgagatcag aggtccctaaggggaagaggcaacactc	-	-
CY010640	Human H3N2 IAVs	Human	H3N2		1995	USA	A/New_York/609/1995	agtgatgccccattccttgatcggcttcgccgagatcag aggtccctaaggggaagaggcaacactc	-	-
CY112793	Human H3N2 IAVs	Human	H3N2		1995	China	A/Nanchang/933/1995	agtgatgccccattccttgatcggcttcgccgagatcag aggtccctaaggggaagaggcaacactc	-	-
CY112825	Human H3N2 IAVs	Human	H3N2		1995	China	A/WUHAN/359/1995	agtgatgccccattccttgatcggcttcgccgagatcag aggtccctaaggggaagaggcaacactc	-	-
CY114225	Human H3N2 IAVs	Human	H3N2		1995	Netherlands	A/Netherlands/1/1995	agtgatgccccattccttgatcggcttcgccgagatcag aggtccctaaggggaagaggcaacactc	-	-
CY011548	Human H3N2 IAVs	Human	H3N2		1995	USA	A/New_York/672/1995	agtgatgccccattccttgatcggcttcgccgagatcag aggtccctaaggggaagaggcaacactc	-	-
CY012900	Human H3N2 IAVs	Human	H3N2		1995	USA	A/New_York/673/1995	agtgatgccccattccttgatcggcttcgccgagatcag aggtccctaaggggaagaggcaacactc	-	-
CY017295	Human H3N2 IAVs	Human	H3N2		1995	USA	A/New_York/681/1995	agtgatgccccattccttgatcggcttcgccgagatcag aggtccctaaggggaagaggcaacactc	-	-
CY012260	Human H3N2 IAVs	Human	H3N2		1995	USA	A/New_York/682/1995	agtgatgccccattccttgatcggcttcgccgagatcag aggtccctaaggggaagaggcaacactc	-	-
CY012908	Human H3N2 IAVs	Human	H3N2		1995	USA	A/New_York/683/1995	agtgatgccccattccttgatcggcttcgccgagatcag aggtccctaaggggaagaggcaacactc	-	-
CY011348	Human H3N2 IAVs	Human	H3N2		1995	USA	A/New_York/697/1995	agtgatgccccattccttgatcggcttcgccgagatcag aggtccctaaggggaagaggcaacactc	-	-

CY012484	Human H3N2 IAVs	Human	H3N2		1995	USA	A/New_York/666/1995	agtgatgccccattccttgatcggcttcgccgagatcag aggtccctaaggggaagaggcaacactc	-	-
CY011532	Human H3N2 IAVs	Human	H3N2		1995	USA	A/New_York/669/1995	agtgatgccccattccttgatcggcttcgccgagatcag aggtccctaaggggaagaggcaacactc	-	-
CY012236	Human H3N2 IAVs	Human	H3N2		1995	USA	A/New_York/670/1995	agtgatgccccattccttgatcggcttcgccgagatcag aggtccctaaggggaagaggcaacactc	-	-
CY012252	Human H3N2 IAVs	Human	H3N2		1995	USA	A/New_York/677/1995	agtgatgccccattccttgatcggcttcgccgagatcag aggtccctaaggggaagaggcaacactc	-	-
CY011564	Human H3N2 IAVs	Human	H3N2		1995	USA	A/New_York/679/1995	agtgatgccccattccttgatcggcttcgccgagatcag aggtccctaaggggaagaggcaacactc	-	-
CY012916	Human H3N2 IAVs	Human	H3N2		1995	USA	A/New_York/686/1995	agtgatgccccattccttgatcggcttcgccgagatcag aggtccctaaggggaagaggcaacactc	-	-
CY012516	Human H3N2 IAVs	Human	H3N2		1995	USA	A/New_York/691/1995	agtgatgccccattccttgatcggcttcgccgagatcag aggtccctaaggggaagaggcaacactc	-	-
CY011332	Human H3N2 IAVs	Human	H3N2		1995	USA	A/New_York/695/1995	agtgatgccccattccttgatcggcttcgccgagatcag aggtccctaaggggaagaggcaacactc	-	-
CY012492	Human H3N2 IAVs	Human	H3N2		1995	USA	A/New_York/667/1995	agtgatgccccattccttgatcggcttcgccgagatcag aggtccctaaggggaagaggcaacactc	-	-
CY011836	Human H3N2 IAVs	Human	H3N2		1995	USA	A/New_York/685/1995	agtgatgccccattccttgatcggcttcgccgagatcag aggtccctaaggggaagaggcaacactc	-	-
CY011572	Human H3N2 IAVs	Human	H3N2		1995	USA	A/New_York/693/1995	agtgatgccccattccttgatcggcttcgccgagatcag aggtccctaaggggaagaggcaacactc	-	-
CY012244	Human H3N2 IAVs	Human	H3N2		1995	USA	A/New_York/675/1995	agtgatgccccattccttgatcggcttcgccgagatcag aggtccctaaggggaagaggcaacactc	-	-
CY011356	Human H3N2 IAVs	Human	H3N2		1995	USA	A/New_York/698/1995	agtgatgccccattccttgatcggcttcgccgagatcag aggtccctaaggggaagaggcaacactc	-	-
CY017287	Human H3N2 IAVs	Human	H3N2		1995	USA	A/New_York/680/1995	agtgatgccccattccttgatcggcttcgccgagatcag aggtccctaaggggaagaggcaacactc	-	-
CY011828	Human H3N2 IAVs	Human	H3N2		1995	USA	A/New_York/684/1995	agtgatgccccattccttgatcggcttcgccgagatcag aggtccctaaggggaagaggcaacactc	-	-
CY012476	Human H3N2 IAVs	Human	H3N2		1995	USA	A/New_York/664/1995	agtgatgccccattccttgatcggcttcgccgagatcag aggtccctaaggggaagaggcaacactc	-	-
CY011852	Human H3N2 IAVs	Human	H3N2		1995	USA	A/New_York/689/1995	agtgatgccccattccttgatcggcttcgccgagatcag aggtccctaaggggaagaggcaacactc	-	-
CY012924	Human H3N2 IAVs	Human	H3N2		1995	USA	A/New_York/690/1995	agtgatgccccattccttgatcggcttcgccgagatcag aggtccctaaggggaagaggcaacactc	-	-
CY114177	Human H3N2 IAVs	Human	H3N2		1995	Finland	A/Finland/339/1995	agtgatgccccattccttgatcggcttcgccgagatcag aggtccctaaggggaagaggcaacactc	-	-
U65673	Human H3N2 IAVs	Human	H3N2		1995	Japan	A/Shiga/20/95	agtgatgccccattccttgatcggcttcgccgagatcag aggtccctaaggggaagaggcaacactc	-	-
CY112817	Human H3N2 IAVs	Human	H3N2		1995	Australia	A/Victoria/75/1995	agtgatgccccattccttgatcggcttcgccgagatcag aggtccctaaggggaagaggcaacactc	-	-
CY038507	Human H3N2 IAVs	Human	H3N2		1995	Italy	A/Siena/3/1995	agtgatgccccattccttgatcggcttcgccgagatcag aggtccctaaggggaagaggcaacactc	-	-
CY114185	Human H3N2 IAVs	Human	H3N2		1995	Finland	A/Finland/381/1995	agtgatgccccattccttgatcggcttcgccgagatcag aggtccctaaggggaagaggcaacactc	-	-
CY112801	Human H3N2 IAVs	Human	H3N2		1995	Netherlands	A/Netherlands/271/1995	agtgatgccccattccttgatcggcttcgccgagatcag aggtccctaaggggaagaggcaacactc	-	-
CY112769	Human H3N2 IAVs	Human	H3N2		1995	Hong_Kong	A/Hong_Kong/32/1995	agtgatgccccattccttgatcggcttcgccgagatcag aggtccctaaggggaagaggcaacactc	-	-

CY114209	Human H3N2 IAVs	Human	H3N2		1995	Hong_Kong	A/Hong_Kong/49/1995	agtgatgccccattccttgatcggcttcgccgagatcag aggtccctaaggggaagaggcaacactc	-	-
U65674	Human H3N2 IAVs	Human	H3N2		1995	Japan	A/Miyagi/69/95	agtgatgccccattccttgatcggcttcgccgagatcag aggtccctaaggggaagaggcaacactc	-	-
KC296473	Human H3N2 IAVs	Human	H3N2		1995	Netherlands	A/Netherlands/178/1995	agtgatgccccattccttgatcggcttcgccgagatcag aggtccctaaggggaagaggcaacactc	-	-
CY112785	Human H3N2 IAVs	Human	H3N2		1995	Hong_Kong	A/Hong_Kong/55/1995	agtgatgccccattccttgatcggcttcgccgagatcag aggtccctaaggggaagaggcaacactc	-	-
CY118430	Human H3N2 IAVs	Human	H3N2		1995	Malaysia	A/Malaysia/07831/1995	agtgatgccccattccttgatcggcttcgccgagatcag aggtccctaaggggaagaggcaacactc	-	-
CY010656	Human H3N2 IAVs	Human	H3N2		1995	USA	A/New_York/611/1995	agtgatgctccattccttgatcggcttcgccgagatcag agatccctaaggggaagaggcaacactc	-	-
CY010664	Human H3N2 IAVs	Human	H3N2		1995	USA	A/New_York/612/1995	agtgatgctccattccttgatcggcttcgccgagatcag agatccctaaggggaagaggcaacactc	-	-
CY010680	Human H3N2 IAVs	Human	H3N2		1995	USA	A/New_York/618/1995	agtgatgctccattccttgatcggcttcgccgagatcag agatccctaaggggaagaggcaacactc	-	-
CY010816	Human H3N2 IAVs	Human	H3N2		1995	USA	A/New_York/623/1995	agtgatgctccattccttgatcggcttcgccgagatcag agatccctaaggggaagaggcaacactc	-	-
CY010712	Human H3N2 IAVs	Human	H3N2		1995	USA	A/New_York/628/1995	agtgatgctccattccttgatcggcttcgccgagatcag agatccctaaggggaagaggcaacactc	-	-
CY114265	Human H3N2 IAVs	Human	H3N2		1996	Hong_Kong	A/Hong_Kong/358/1996	agcgatgccccattccttgatcggcttcgccgagatca gaggtccctaaggggaagaggcaacactc	-	-
CY114281	Human H3N2 IAVs	Human	H3N2		1996	France	A/Lyon/1781/1996	agtgatgccccattccttgaccggcttcgccgagatca gaggtccctaaggggaagaggcaacactc	-	-
CY112865	Human H3N2 IAVs	Human	H3N2		1996	France	A/Nice/491/1996	agtgatgccccattccttgaccggcttcgccgagatca gaggtccctaaggggaagaggcaatactc	-	-
CY114289	Human H3N2 IAVs	Human	H3N2		1996	Netherlands	A/Netherlands/91/1996	agtgatgccccattccttgatcggcttcgccgagatcag agatccctaaggggaagaggcaacactc	-	*
CY009688	Human H3N2 IAVs	Human	H3N2		1996	USA	A/New_York/580/1996	agtgatgccccattccttgatcggcttcgccgagatcag aggtccctaagggaaagaggcaacactc	+	+
CY114481	Human H3N2 IAVs	Human	H3N2		1996	Australia	A/Brisbane/8/1996	agtgatgccccattccttgatcggcttcgccgagatcag aggtccctaaggggaagaagcaacactc	-	-
CY114233	Human H3N2 IAVs	Human	H3N2		1996	Switzerland	A/Geneva/3958/1996	agtgatgccccattccttgatcggcttcgccgagatcag aggtccctaaggggaagaggcaacactc	-	-
CY114273	Human H3N2 IAVs	Human	H3N2		1996	Hong_Kong	A/Hong_Kong/434/1996	agtgatgccccattccttgatcggcttcgccgagatcag aggtccctaaggggaagaggcaacactc	-	-
CY010520	Human H3N2 IAVs	Human	H3N2		1996	USA	A/New_York/617/1996	agtgatgccccattccttgatcggcttcgccgagatcag aggtccctaaggggaagaggcaacactc	-	-
CY010632	Human H3N2 IAVs	Human	H3N2		1996	USA	A/New_York/608/1996	agtgatgccccattccttgatcggcttcgccgagatcag aggtccctaaggggaagaggcaacactc	-	-
CY112833	Human H3N2 IAVs	Human	H3N2		1996	Singapore	A/Singapore/1/1996	agtgatgccccattccttgatcggcttcgccgagatcag aggtccctaaggggaagaggcaacactc	-	-
CY010720	Human H3N2 IAVs	Human	H3N2		1996	USA	A/New_York/631/1996	agtgatgccccattccttgatcggcttcgccgagatcag aggtccctaaggggaagaggcaacactc	-	-
CY010688	Human H3N2 IAVs	Human	H3N2		1996	USA	A/New_York/619/1996	agtgatgccccattccttgatcggcttcgccgagatcag aggtccctaaggggaagaggcaacactc	-	-
CY010696	Human H3N2 IAVs	Human	H3N2		1996	USA	A/New_York/622/1996	agtgatgccccattccttgatcggcttcgccgagatcag aggtccctaaggggaagaggcaacactc	-	-
CY010736	Human H3N2 IAVs	Human	H3N2		1996	USA	A/New_York/637/1996	agtgatgccccattccttgatcggcttcgccgagatcag aggtccctaaggggaagaggcaacactc	-	-

CY117702	Human H3N2 IAVs	Human	H3N2		1996	Malaysia	A/Malaysia/10081/1996	agtgatgccccattccttgatcggcttcgccgagatcag aggtccctaaggggaagaggcaacactc	-	-
CY118446	Human H3N2 IAVs	Human	H3N2		1996	Malaysia	A/Malaysia/10111/1996	agtgatgccccattccttgatcggcttcgccgagatcag aggtccctaaggggaagaggcaacactc	-	-
CY117710	Human H3N2 IAVs	Human	H3N2		1996	Malaysia	A/Malaysia/10135/1996	agtgatgccccattccttgatcggcttcgccgagatcag aggtccctaaggggaagaggcaacactc	-	-
CY010000	Human H3N2 IAVs	Human	H3N2		1996	USA	A/New_York/562/1996	agtgatgccccattccttgatcggcttcgccgagatcag aggtccctaaggggaagaggcaacactc	-	-
CY009912	Human H3N2 IAVs	Human	H3N2		1996	USA	A/New_York/593/1996	agtgatgccccattccttgatcggcttcgccgagatcag aggtccctaaggggaagaggcaacactc	-	-
CY009904	Human H3N2 IAVs	Human	H3N2		1996	USA	A/New_York/584/1996	agtgatgccccattccttgatcggcttcgccgagatcag aggtccctaaggggaagaggcaacactc	-	-
CY011420	Human H3N2 IAVs	Human	H3N2		1996	USA	A/New_York/571/1996	agtgatgccccattccttgatcggcttcgccgagatcag aggtccctaaggggaagaggcaacactc	-	-
CY010024	Human H3N2 IAVs	Human	H3N2		1996	USA	A/New_York/582/1996	agtgatgccccattccttgatcggcttcgccgagatcag aggtccctaaggggaagaggcaacactc	-	-
CY011268	Human H3N2 IAVs	Human	H3N2		1996	USA	A/New_York/559/1996	agtgatgccccattccttgatcggcttcgccgagatcag aggtccctaaggggaagaggcaacactc	-	-
CY010608	Human H3N2 IAVs	Human	H3N2		1996	USA	A/New_York/561/1996	agtgatgccccattccttgatcggcttcgccgagatcag aggtccctaaggggaagaggcaacactc	-	-
CY009664	Human H3N2 IAVs	Human	H3N2		1996	USA	A/New_York/568/1996	agtgatgccccattccttgatcggcttcgccgagatcag aggtccctaaggggaagaggcaacactc	-	-
CY009496	Human H3N2 IAVs	Human	H3N2		1996	USA	A/New_York/570/1996	agtgatgccccattccttgatcggcttcgccgagatcag aggtccctaaggggaagaggcaacactc	-	-
CY010056	Human H3N2 IAVs	Human	H3N2		1996	USA	A/New_York/595/1996	agtgatgccccattccttgatcggcttcgccgagatcag aggtccctaaggggaagaggcaacactc	-	-
CY010064	Human H3N2 IAVs	Human	H3N2		1996	USA	A/New_York/596/1996	agtgatgccccattccttgatcggcttcgccgagatcag aggtccctaaggggaagaggcaacactc	-	-
CY010800	Human H3N2 IAVs	Human	H3N2		1996	USA	A/New_York/555/1996	agtgatgccccattccttgatcggcttcgccgagatcag aggtccctaaggggaagaggcaacactc	-	-
CY121361	Human H3N2 IAVs	Human	H3N2		1996	South_Africa	A/South_Africa/1147/1996	agtgatgccccattccttgatcggcttcgccgagatcag aggtccctaaggggaagaggcaacactc	-	-
CY009504	Human H3N2 IAVs	Human	H3N2		1996	USA	A/New_York/573/1996	agtgatgccccattccttgatcggcttcgccgagatcag aggtccctaaggggaagaggcaacactc	-	-
CY114257	Human H3N2 IAVs	Human	H3N2		1996	Hong_Kong	A/Hong_Kong/357/1996	agtgatgccccattccttgatcggcttcgccgagatcag aggtccctaaggggaagaggcaacactc	-	-
CY012852	Human H3N2 IAVs	Human	H3N2		1996	USA	A/New_York/554/1996	agtgatgccccattccttgatcggcttcgccgagatcag aggtccctaaggggaagaggcaacactc	-	-
CY010600	Human H3N2 IAVs	Human	H3N2		1996	USA	A/New_York/578/1996	agtgatgccccattccttgatcggcttcgccgagatcag aggtccctaaggggaagaggcaacactc	-	-
CY011260	Human H3N2 IAVs	Human	H3N2		1996	USA	A/New_York/557/1996	agtgatgccccattccttgatcggcttcgccgagatcag aggtccctaaggggaagaggcaacactc	-	-
CY011428	Human H3N2 IAVs	Human	H3N2		1996	USA	A/New_York/577/1996	agtgatgccccattccttgatcggcttcgccgagatcag aggtccctaaggggaagaggcaacactc	-	-
CY009712	Human H3N2 IAVs	Human	H3N2		1996	USA	A/New_York/586/1996	agtgatgccccattccttgatcggcttcgccgagatcag aggtccctaaggggaagaggcaacactc	-	-
CY009728	Human H3N2 IAVs	Human	H3N2		1996	USA	A/New_York/588/1996	agtgatgccccattccttgatcggcttcgccgagatcag aggtccctaaggggaagaggcaacactc	-	-
CY009736	Human H3N2 IAVs	Human	H3N2		1996	USA	A/New_York/589/1996	agtgatgccccattccttgatcggcttcgccgagatcag aggtccctaaggggaagaggcaacactc	-	-

CY010032	Human H3N2 IAVs	Human	H3N2		1996	USA	A/New_York/591/1996	agtgatgccccattccttgatcggcttcgccgagatcag<br>aggtccctaaggggaagaggcaacactc	-	-
CY012212	Human H3N2 IAVs	Human	H3N2		1996	USA	A/New_York/598/1996	agtgatgccccattccttgatcggcttcgccgagatcag<br>aggtccctaaggggaagaggcaacactc	-	-
CY011140	Human H3N2 IAVs	Human	H3N2		1996	USA	A/New_York/602/1996	agtgatgccccattccttgatcggcttcgccgagatcag<br>aggtccctaaggggaagaggcaacactc	-	-
AF038276	Human H3N2 IAVs	Human	H3N2		1996	Japan	A/Niigata/137/96	agtgatgccccattccttgatcggcttcgccgagatcag<br>aggtccctaaggggaagaggcaacactc	-	-
CY010616	Human H3N2 IAVs	Human	H3N2		1996	USA	A/New_York/603/1996	agtgatgccccattccttgatcggcttcgccgagatcag<br>aggtccctaaggggaagaggcaacactc	-	-
CY010016	Human H3N2 IAVs	Human	H3N2		1996	USA	A/New_York/567/1996	agtgatgccccattccttgatcggcttcgccgagatcag<br>aggtccctaaggggaagaggcaacactc	-	-
CY118454	Human H3N2 IAVs	Human	H3N2		1996	Malaysia	A/Malaysia/10360/1996	agtgatgccccattccttgatcggcttcgccgagatcag<br>aggtccctaaggggaagaggcaacactc	-	-
CY118462	Human H3N2 IAVs	Human	H3N2		1996	Malaysia	A/Malaysia/10370/1996	agtgatgccccattccttgatcggcttcgccgagatcag<br>aggtccctaaggggaagaggcaacactc	-	-
CY114241	Human H3N2 IAVs	Human	H3N2		1996	Hong_Kong	A/Hong_Kong/20/1996	agtgatgccccattccttgatcggcttcgccgagatcag<br>aggtccctaaggggaagaggcaacactc	-	-
AF038278	Human H3N2 IAVs	Human	H3N2		1996	Japan	A/Fukushima/140/96	agtgatgccccattccttgatcggcttcgccgagatcag<br>aggtccctaaggggaagaggcaacactc	-	-
AF038277	Human H3N2 IAVs	Human	H3N2		1996	Japan	A/Fukushima/114/96	agtgatgccccattccttgatcggcttcgccgagatcag<br>aggtccctaaggggaagaggcaacactc	-	-
CY010704	Human H3N2 IAVs	Human	H3N2		1996	USA	A/New_York/625/1996	agtgatgccccattccttgatcggcttcgccgagatcag<br>aggtccctaaggggaagaggcaacactc	-	-
CY011444	Human H3N2 IAVs	Human	H3N2		1996	USA	A/New_York/632/1996	agtgatgccccattccttgatcggcttcgccgagatcag<br>aggtccctaaggggaagaggcaacactc	-	-
CY011820	Human H3N2 IAVs	Human	H3N2		1996	USA	A/New_York/652/1996	agtgatgccccattccttgatcggcttcgccgagatcag<br>aggtccctaaggggaagaggcaacactc	-	-
CY010040	Human H3N2 IAVs	Human	H3N2		1996	USA	A/New_York/592/1996	agtgatgccccattccttgatcggcttcgccgagatcag<br>aggtccctaaggggaagaggcaacactc	-	-
CY118510	Human H3N2 IAVs	Human	H3N2		1996	Malaysia	A/Malaysia/11473/1996	agtgatgccccattccttgatcggcttcgccgagatcag<br>aggtccctaaggggaagaggcaacactc	-	-
CY009512	Human H3N2 IAVs	Human	H3N2		1996	USA	A/New_York/575/1996	agtgatgccccattccttgatcggcttcgccgagatcag<br>aggtccctaaggggaagaggcaacactc	-	-
CY009720	Human H3N2 IAVs	Human	H3N2		1996	USA	A/New_York/587/1996	agtgatgccccattccttgatcggcttcgccgagatcag<br>aggtccctaaggggaagaggcaacactc	-	-
CY010048	Human H3N2 IAVs	Human	H3N2		1996	USA	A/New_York/594/1996	agtgatgccccattccttgatcggcttcgccgagatcag<br>aggtccctaaggggaagaggcaacactc	-	-
CY011132	Human H3N2 IAVs	Human	H3N2		1996	USA	A/New_York/599/1996	agtgatgccccattccttgatcggcttcgccgagatcag<br>aggtccctaaggggaagaggcaacactc	-	-
CY010072	Human H3N2 IAVs	Human	H3N2		1996	USA	A/New_York/600/1996	agtgatgccccattccttgatcggcttcgccgagatcag<br>aggtccctaaggggaagaggcaacactc	-	-
CY009672	Human H3N2 IAVs	Human	H3N2		1996	USA	A/New_York/574/1996	agtgatgccccattccttgatcggcttcgccgagatcag<br>aggtccctaaggggaagaggcaacactc	-	-
CY009480	Human H3N2 IAVs	Human	H3N2		1996	USA	A/New_York/565/1996	agtgatgccccattccttgatcggcttcgccgagatcag<br>aggtccctaaggggaagaggcaacactc	-	-
CY010592	Human H3N2 IAVs	Human	H3N2		1996	USA	A/New_York/572/1996	agtgatgccccattccttgatcggcttcgccgagatcag<br>aggtccctaaggggaagaggcaacactc	-	-
CY009744	Human H3N2 IAVs	Human	H3N2		1996	USA	A/New_York/590/1996	agtgatgccccattccttgatcggcttcgccgagatcag<br>aggtccctaaggggaagaggcaacactc	-	-

CY114249	Human H3N2 IAVs	Human	H3N2		1996	Hong_Kong	A/Hong_Kong/42/1996	agtgatgccccattccttgatcggcttcgccgagatcag aggtccctaaggggaagaggcaacactc	-	-
CY012204	Human H3N2 IAVs	Human	H3N2		1996	USA	A/New_York/556/1996	agtgatgccccattccttgatcggcttcgccgagatcag aggtccctaaggggaagaggcaacactc	-	-
CY015520	Human H3N2 IAVs	Human	H3N2		1996	USA	A/New_York/601/1996	agtgatgccccattccttgatcggcttcgccgagatcag aggtccctaaggggaagaggcaacactc	-	-
CY010008	Human H3N2 IAVs	Human	H3N2		1996	USA	A/New_York/563/1996	agtgatgccccattccttgatcggcttcgccgagatcag aggtccctaaggggaagaggcaatactc	-	-
CY010672	Human H3N2 IAVs	Human	H3N2		1996	USA	A/New_York/613/1996	agtgatgctccattccttgatcggcttcgccgagatcag agatccctaaggggaagaggcaacactc	-	-
CY011452	Human H3N2 IAVs	Human	H3N2		1996	USA	A/New_York/635/1996	agtgatgctccattccttgatcggcttcgccgagatcag agatccctaaggggaagaggcaacactc	-	-
CY011468	Human H3N2 IAVs	Human	H3N2		1996	USA	A/New_York/641/1996	agtgatgctccattccttgatcggcttcgccgagatcag agatccctaaggggaagaggcaacactc	-	-
CY011436	Human H3N2 IAVs	Human	H3N2		1996	USA	A/New_York/624/1996	agtgatgctccattccttgatcggcttcgccgagatcag agatccctaaggggaagaggcaacactc	-	-
CY011460	Human H3N2 IAVs	Human	H3N2		1996	USA	A/New_York/636/1996	agtgatgctccatttcttgatcggcttcgccgagatcag agatccctaaggggaagaggcaacactc	+	+
AF251400	Human H3N2 IAVs	Swine	H3N2		1997	Canada	A/swine/Ontario/00130/97	agtgatgccccattccttgaccggcttcgccgagatca gaggtccctaaggggaagaggcaacactc	-	-
CY009656	Human H3N2 IAVs	Human	H3N2		1997	USA	A/New_York/566/1997	agtgatgccccattccttgatcggcttcgccgagacca gaggtccctaaggggaagaggcaacactc	-	-
CY009648	Human H3N2 IAVs	Human	H3N2		1997	USA	A/New_York/558/1997	agtgatgccccattccttgatcggcttcgccgagatcag aggtccctaaggggaagaggcaacactc	-	-
CY009704	Human H3N2 IAVs	Human	H3N2		1997	USA	A/New_York/585/1997	agtgatgccccattccttgatcggcttcgccgagatcag aggtccctaaggggaagaggcaacactc	-	-
CY118534	Human H3N2 IAVs	Human	H3N2		1997	Malaysia	A/Malaysia/12726/1997	agtgatgccccattccttgatcggcttcgccgagatcag aggtccctaaggggaagaggcaacactc	-	-
CY036851	Human H3N2 IAVs	Human	H3N2		1997	Italy	A/Siena/1/1997	agtgatgccccattccttgatcggcttcgccgagatcag aggtccctaaggggaagaggcaacactc	-	-
CY117756	Human H3N2 IAVs	Human	H3N2		1997	Malaysia	A/Malaysia/13241/1997	agtgatgccccattccttgatcggcttcgccgagatcag aggtccctaaggggaagaggcaacactc	-	-
CY009488	Human H3N2 IAVs	Human	H3N2		1997	USA	A/New_York/569/1997	agtgatgccccattccttgatcggcttcgccgagatcag aggtccctaaggggaagaggcaacactc	-	-
CY117788	Human H3N2 IAVs	Human	H3N2		1997	Malaysia	A/Malaysia/14712/1997	agtgatgccccattccttgatcggcttcgccgagatcag aggtccctaaggggaagaggcaacactc	-	-
CY114297	Human H3N2 IAVs	Human	H3N2		1997	New_Zealand	A/Auckland/10/1997	agtgatgccccattccttgatcggcttcgccgagatcag aggtccctaaggggaagaggcaacactc	-	-
CY006231	Human H3N2 IAVs	Human	H3N2		1997	USA	A/New_York/503/1997	agtgatgccccattccttgatcggcttcgccgagatcag aggtccctaaggggaagaggcaacactc	-	-
CY006247	Human H3N2 IAVs	Human	H3N2		1997	USA	A/New_York/508/1997	agtgatgccccattccttgatcggcttcgccgagatcag aggtccctaaggggaagaggcaacactc	-	-
CY006263	Human H3N2 IAVs	Human	H3N2		1997	USA	A/New_York/511/1997	agtgatgccccattccttgatcggcttcgccgagatcag aggtccctaaggggaagaggcaacactc	-	-
CY006471	Human H3N2 IAVs	Human	H3N2		1997	USA	A/New_York/516/1997	agtgatgccccattccttgatcggcttcgccgagatcag aggtccctaaggggaagaggcaacactc	-	-
CY006527	Human H3N2 IAVs	Human	H3N2		1997	USA	A/New_York/524/1997	agtgatgccccattccttgatcggcttcgccgagatcag aggtccctaaggggaagaggcaacactc	-	-
CY006615	Human H3N2 IAVs	Human	H3N2		1997	USA	A/New_York/545/1997	agtgatgccccattccttgatcggcttcgccgagatcag aggtccctaaggggaagaggcaacactc	-	-

CY006631	Human H3N2 IAVs	Human	H3N2		1997	USA	A/New_York/547/1997	agtgatgccccattccttgatcggcttcgccgagatcag aggtccctaaggggaagaggcaacactc	-	-
CY009752	Human H3N2 IAVs	Human	H3N2		1997	USA	A/New_York/597/1997	agtgatgccccattccttgatcggcttcgccgagatcag aggtccctaaggggaagaggcaacactc	-	-
CY112889	Human H3N2 IAVs	Human	H3N2		1997	Australia	A/Sydney/5/1997	agtgatgccccattccttgatcggcttcgccgagatcag aggtccctaaggggaagaggcaacactc	-	-
CY039083	Human H3N2 IAVs	Human	H3N2		1997	Australia	A/Sydney/5/1997	agtgatgccccattccttgatcggcttcgccgagatcag aggtccctaaggggaagaggcaacactc	-	-
CY006455	Human H3N2 IAVs	Human	H3N2		1997	USA	A/New_York/509/1997	agtgatgccccattccttgatcggcttcgccgagatcag aggtccctaaggggaagaggcaacactc	-	-
CY114305	Human H3N2 IAVs	Human	H3N2		1997	Netherlands	A/Netherlands/300/1997	agtgatgccccattccttgatcggcttcgccgagatcag aggtccctaaggggaagaggcaacactc	-	-
CY006279	Human H3N2 IAVs	Human	H3N2		1997	USA	A/New_York/515/1997	agtgatgccccattccttgatcggcttcgccgagatcag aggtccctaaggggaagaggcaacactc	-	-
CY006447	Human H3N2 IAVs	Human	H3N2		1997	USA	A/New_York/501/1997	agtgatgccccattccttgatcggcttcgccgagatcag aggtccctaaggggaagaggcaacactc	-	-
CY006511	Human H3N2 IAVs	Human	H3N2		1997	USA	A/New_York/522/1997	agtgatgccccattccttgatcggcttcgccgagatcag aggtccctaaggggaagaggcaacactc	-	-
CY007983	Human H3N2 IAVs	Human	H3N2		1997	USA	A/New_York/526/1997	agtgatgccccattccttgatcggcttcgccgagatcag aggtccctaaggggaagaggcaacactc	-	-
CY006239	Human H3N2 IAVs	Human	H3N2		1997	USA	A/New_York/505/1997	agtgatgccccattccttgatcggcttcgccgagatcag aggtccctaaggggaagaggcaacactc	-	-
CY008936	Human H3N2 IAVs	Human	H3N2		1997	USA	A/New_York/507/1997	agtgatgccccattccttgatcggcttcgccgagatcag aggtccctaaggggaagaggcaacactc	-	-
CY006271	Human H3N2 IAVs	Human	H3N2		1997	USA	A/New_York/513/1997	agtgatgccccattccttgatcggcttcgccgagatcag aggtccctaaggggaagaggcaacactc	-	-
AF225531	Human H3N2 IAVs	Swine	H3N2		1997	Japan	A/sw/Shizuoka/115/97	agtgatgccccattccttgatcggcttcgccgagatcag aggtccctaaggggaagaggcaacactc	-	-
AF225530	Human H3N2 IAVs	Swine	H3N2		1997	Japan	A/sw/Shizuoka/110/97	agtgatgccccattccttgatcggcttcgccgagatcag aggtccctaaggggaagaggcaacactc	-	-
AF225532	Human H3N2 IAVs	Swine	H3N2		1997	Japan	A/sw/Shizuoka/119/97	agtgatgccccattccttgatcggcttcgccgagatcag aggtccctaaggggaagaggcaacactc	-	-
AF225533	Human H3N2 IAVs	Swine	H3N2		1997	Japan	A/swine/Shizuoka/120/97	agtgatgccccattccttgatcggcttcgccgagatcag aggtccctaaggggaagaggcaacactc	-	-
CY112841	Human H3N2 IAVs	Human	H3N2		1997	Hong_Kong	A/Hong_Kong/1/1997	agtgatgccccattccttgatcggcttcgccgagatcag aggtccctaaggggaagaggcaacactc	-	-
CY112873	Human H3N2 IAVs	Human	H3N2		1997	Norway	A/Oslo/21/1997	agtgatgccccattccttgatcggcttcgccgagatcag aggtccctaaggggaagaggcaacactc	-	-
CY112649	Human H3N2 IAVs	Human	H3N2		1997	Norway	A/Oslo/244/1997	agtgatgccccattccttgatcggcttcgccgagatcag aggtccctaaggggaagaggcaacactc	-	-
CY009472	Human H3N2 IAVs	Human	H3N2		1997	USA	A/New_York/564/1997	agtgatgccccattccttgatcggcttcgccgagatcag aggtccctaaggggaagaggcaacactc	-	-
CY009680	Human H3N2 IAVs	Human	H3N2		1997	USA	A/New_York/576/1997	agtgatgccccattccttgatcggcttcgccgagatcag aggtccctaaggggaagaggcaacactc	-	-
CY009520	Human H3N2 IAVs	Human	H3N2		1997	USA	A/New_York/579/1997	agtgatgccccattccttgatcggcttcgccgagatcag aggtccctaaggggaagaggcaacactc	-	-
CY009696	Human H3N2 IAVs	Human	H3N2		1997	USA	A/New_York/581/1997	agtgatgccccattccttgatcggcttcgccgagatcag aggtccctaaggggaagaggcaacactc	-	-
CY009528	Human H3N2 IAVs	Human	H3N2		1997	USA	A/New_York/583/1997	agtgatgccccattccttgatcggcttcgccgagatcag aggtccctaaggggaagaggcaacactc	-	-

CY009464	Human H3N2 IAVs	Human	H3N2		1997	USA	A/New_York/560/1997	agtgatgccccattccttgatcggcttcgccgagatcag<br>aggtccctaaggggaagaggcaacactc	-	-
CY112857	Human H3N2 IAVs	Human	H3N2		1997	South_Africa	A/Johannesburg/10/1997	agtgatgccccattccttgatcggcttcgccgagatcag<br>aggtccctaaggggaagaggcaacactc	-	-
CY112849	Human H3N2 IAVs	Human	H3N2		1997	Hong_Kong	A/Hong_Kong/280/1997	agtgatgccccattccttgatcggcttcgccgagatcag<br>aggtccctaaggggaagaggcaacactc	-	-
AF038279	Human H3N2 IAVs	Human	H3N2		1997	Japan	A/Shiga/25/97	agtgatgccccattccttgatcggcttcgccgagatcag<br>aggtccctaaggggaagaggcaacactc	-	-
AF256182	Human H3N2 IAVs	Human	H3N2		1997	Hong_Kong	A/Hong_Kong/497/97	agtgatgccccattccttgatcggcttcgccgagatcag<br>aggtccctaaggggaagaggcaacactc	-	-
AF256183	Human H3N2 IAVs	Human	H3N2		1997	Hong_Kong	A/Hong_Kong/498/97	agtgatgccccattccttgatcggcttcgccgagatcag<br>aggtccctaaggggaagaggcagaactc	-	-
CY008960	Human H3N2 IAVs	Human	H3N2		1998	USA	A/New_York/530/1998	aatgatgccccattccttgatcggcttcgccgagatca<br>gaggtccctaaggggaagaggcaacactc	-	-
CY006559	Human H3N2 IAVs	Human	H3N2		1998	USA	A/New_York/532/1998	agtgatgccccattccttgataggcttcgccgagatca<br>gaggtccctaaggggaagaggcaacactc	+	+
AF400775	Human H3N2 IAVs	Swine	H3N2		1998	Hong_Kong	A/Swine/Hong_Kong/2429/98	agtgatgccccattccttgatcggcttcgccgagatcag<br>agatccctaaggggaagaggcaacactc	-	*
CY001497	Human H3N2 IAVs	Human	H3N2		1998	USA	A/New_York/250/1998	agtgatgccccattccttgatcggcttcgccgagatcag<br>agatccctaaggggaagaggcaacactc	-	*
CY001676	Human H3N2 IAVs	Human	H3N2		1998	USA	A/New_York/287/1998	agtgatgccccattccttgatcggcttcgccgagatcag<br>agatccctaaggggaagaggcaacactc	-	*
CY006799	Human H3N2 IAVs	Human	H3N2		1998	USA	A/New_York/527/1998	agtgatgccccattccttgatcggcttcgccgagatcag<br>aggtccctaaggggaagaggcaacactc	-	-
CY006255	Human H3N2 IAVs	Human	H3N2		1998	USA	A/New_York/510/1998	agtgatgccccattccttgatcggcttcgccgagatcag<br>aggtccctaaggggaagaggcaacactc	-	-
CY006463	Human H3N2 IAVs	Human	H3N2		1998	USA	A/New_York/512/1998	agtgatgccccattccttgatcggcttcgccgagatcag<br>aggtccctaaggggaagaggcaacactc	-	-
CY006487	Human H3N2 IAVs	Human	H3N2		1998	USA	A/New_York/519/1998	agtgatgccccattccttgatcggcttcgccgagatcag<br>aggtccctaaggggaagaggcaacactc	-	-
CY006543	Human H3N2 IAVs	Human	H3N2		1998	USA	A/New_York/528/1998	agtgatgccccattccttgatcggcttcgccgagatcag<br>aggtccctaaggggaagaggcaacactc	-	-
CY008952	Human H3N2 IAVs	Human	H3N2		1998	USA	A/New_York/529/1998	agtgatgccccattccttgatcggcttcgccgagatcag<br>aggtccctaaggggaagaggcaacactc	-	-
CY006551	Human H3N2 IAVs	Human	H3N2		1998	USA	A/New_York/531/1998	agtgatgccccattccttgatcggcttcgccgagatcag<br>aggtccctaaggggaagaggcaacactc	-	-
CY006575	Human H3N2 IAVs	Human	H3N2		1998	USA	A/New_York/534/1998	agtgatgccccattccttgatcggcttcgccgagatcag<br>aggtccctaaggggaagaggcaacactc	-	-
CY009992	Human H3N2 IAVs	Human	H3N2		1998	USA	A/New_York/536/1998	agtgatgccccattccttgatcggcttcgccgagatcag<br>aggtccctaaggggaagaggcaacactc	-	-
CY008968	Human H3N2 IAVs	Human	H3N2		1998	USA	A/New_York/540/1998	agtgatgccccattccttgatcggcttcgccgagatcag<br>aggtccctaaggggaagaggcaacactc	-	-
CY006599	Human H3N2 IAVs	Human	H3N2		1998	USA	A/New_York/542/1998	agtgatgccccattccttgatcggcttcgccgagatcag<br>aggtccctaaggggaagaggcaacactc	-	-
CY006607	Human H3N2 IAVs	Human	H3N2		1998	USA	A/New_York/543/1998	agtgatgccccattccttgatcggcttcgccgagatcag<br>aggtccctaaggggaagaggcaacactc	-	-
CY006639	Human H3N2 IAVs	Human	H3N2		1998	USA	A/New_York/548/1998	agtgatgccccattccttgatcggcttcgccgagatcag<br>aggtccctaaggggaagaggcaacactc	-	-
CY008976	Human H3N2 IAVs	Human	H3N2		1998	USA	A/New_York/549/1998	agtgatgccccattccttgatcggcttcgccgagatcag<br>aggtccctaaggggaagaggcaacactc	-	-

CY008984	Human H3N2 IAVs	Human	H3N2		1998	USA	A/New_York/550/1998	agtgatgccccattccttgatcggcttcgccgagatcag aggtccctaaggggaagaggcaacactc	-	-
CY006495	Human H3N2 IAVs	Human	H3N2		1998	USA	A/New_York/520/1998	agtgatgccccattccttgatcggcttcgccgagatcag aggtccctaaggggaagaggcaacactc	-	-
CY006567	Human H3N2 IAVs	Human	H3N2		1998	USA	A/New_York/533/1998	agtgatgccccattccttgatcggcttcgccgagatcag aggtccctaaggggaagaggcaacactc	-	-
CY008944	Human H3N2 IAVs	Human	H3N2		1998	USA	A/New_York/514/1998	agtgatgccccattccttgatcggcttcgccgagatcag aggtccctaaggggaagaggcaacactc	-	-
CY008536	Human H3N2 IAVs	Human	H3N2		1998	USA	A/New_York/544/1998	agtgatgccccattccttgatcggcttcgccgagatcag aggtccctaaggggaagaggcaacactc	-	-
CY006775	Human H3N2 IAVs	Human	H3N2		1998	USA	A/New_York/535/1998	agtgatgccccattccttgatcggcttcgccgagatcag aggtccctaaggggaagaggcaacactc	-	-
CY006623	Human H3N2 IAVs	Human	H3N2		1998	USA	A/New_York/546/1998	agtgatgccccattccttgatcggcttcgccgagatcag aggtccctaaggggaagaggcaacactc	-	-
CY006519	Human H3N2 IAVs	Human	H3N2		1998	USA	A/New_York/523/1998	agtgatgccccattccttgatcggcttcgccgagatcag aggtccctaaggggaagaggcaacactc	-	-
CY006791	Human H3N2 IAVs	Human	H3N2		1998	USA	A/New_York/506/1998	agtgatgccccattccttgatcggcttcgccgagatcag aggtccctaaggggaagaggcaacactc	-	-
CY008928	Human H3N2 IAVs	Human	H3N2		1998	USA	A/New_York/504/1998	agtgatgccccattccttgatcggcttcgccgagatcag aggtccctaaggggaagaggcaacactc	-	-
CY006535	Human H3N2 IAVs	Human	H3N2		1998	USA	A/New_York/525/1998	agtgatgccccattccttgatcggcttcgccgagatcag aggtccctaaggggaagaggcaacactc	-	-
CY114489	Human H3N2 IAVs	Human	H3N2		1998	Netherlands	A/Netherlands/5/1998	agtgatgccccattccttgatcggcttcgccgagatcag aggtccctaaggggaagaggcaacactc	-	-
CY006479	Human H3N2 IAVs	Human	H3N2		1998	USA	A/New_York/518/1998	agtgatgccccattccttgatcggcttcgccgagatcag aggtccctaaggggaagaggcaacactc	-	-
CY006503	Human H3N2 IAVs	Human	H3N2		1998	USA	A/New_York/521/1998	agtgatgccccattccttgatcggcttcgccgagatcag aggtccctaaggggaagaggcaacactc	-	-
CY006591	Human H3N2 IAVs	Human	H3N2		1998	USA	A/New_York/541/1998	agtgatgccccattccttgatcggcttcgccgagatcag aggtccctaaggggaagaggcaacactc	-	-
CY006807	Human H3N2 IAVs	Human	H3N2		1998	USA	A/New_York/538/1998	agtgatgccccattccttgatcggcttcgccgagatcag aggtccctaaggggaagaggcaacactc	-	-
CY008184	Human H3N2 IAVs	Human	H3N2		1998	USA	A/New_York/502/1998	agtgatgccccattccttgatcggcttcgccgagatcag aggtccctaaggggaagaggcaacactc	-	-
AF400773	Human H3N2 IAVs	Swine	H3N2		1998	Hong_Kong	A/Swine/Hong_Kong/2405/98	agtgatgccccattccttgatcggcttcgccgagatcag aggtccctaaggggaagaggcaacactc	-	-
CY006287	Human H3N2 IAVs	Human	H3N2		1998	USA	A/New_York/517/1998	agtgatgccccattccttgatcggcttcgccgagatcag aggtccctaaggggaagaggcaacactc	-	-
CY006583	Human H3N2 IAVs	Human	H3N2		1998	USA	A/New_York/539/1998	agtgatgccccattccttgatcggcttcgccgagatcag aggtccctaaggggaagaggcaacactc	-	-
CY117718	Human H3N2 IAVs	Human	H3N2		1998	Malaysia	A/Malaysia/10675/1998	agtgatgccccattccttgatcggcttcgccgagatcag aggtccctaaggggaagaggcaacactc	-	-
CY118606	Human H3N2 IAVs	Human	H3N2		1998	Malaysia	A/Malaysia/17230/1998	agtgatgccccattccttgatcggcttcgccgagatcag aggtccctaaggggaagaggcaacactc	-	-
CY112905	Human H3N2 IAVs	Human	H3N2		1998	Netherlands	A/Netherlands/427/1998	agtgatgccccattccttgatcggcttcgccgagatcag aggtccctaaggggaagaggcaacactc	-	-
CY001572	Human H3N2 IAVs	Human	H3N2		1998	USA	A/New_York/224/1998	agtgatgccccattccttgatcggcttcgccgagatcag aggtccctaaggggaagaggcaacactc	-	-
CY002124	Human H3N2 IAVs	Human	H3N2		1998	USA	A/New_York/254/1998	agtgatgccccattccttgatcggcttcgccgagatcag aggtccctaaggggaagaggcaacactc	-	-

CY001804	Human H3N2 IAVs	Human	H3N2		1998	USA	A/New_York/304/1998	agtgatgccccattccttgatcggcttcgccgagatcag aggtccctaaggggaagaggcaacactc	-	-
CY001820	Human H3N2 IAVs	Human	H3N2		1998	USA	A/New_York/313/1998	agtgatgccccattccttgatcggcttcgccgagatcag aggtccctaaggggaagaggcaacactc	-	-
CY002548	Human H3N2 IAVs	Human	H3N2		1998	USA	A/New_York/251/1998	agtgatgccccattccttgatcggcttcgccgagatcag aggtccctaaggggaagaggcaacactc	-	-
CY001481	Human H3N2 IAVs	Human	H3N2		1998	USA	A/New_York/240/1998	agtgatgccccattccttgatcggcttcgccgagatcag aggtccctaaggggaagaggcaacactc	-	-
CY002380	Human H3N2 IAVs	Human	H3N2		1998	USA	A/New_York/328/1998	agtgatgccccattccttgatcggcttcgccgagatcag aggtccctaaggggaagaggcaacactc	-	-
CY117796	Human H3N2 IAVs	Human	H3N2		1998	Malaysia	A/Malaysia/17276/1998	agtgatgccccattccttgatcggcttcgccgagatcag aggtccctaaggggaagaggcaacactc	-	-
CY001489	Human H3N2 IAVs	Human	H3N2		1998	USA	A/New_York/249/1998	agtgatgccccattccttgatcggcttcgccgagatcag aggtccctaaggggaagaggcaacactc	-	-
CY001588	Human H3N2 IAVs	Human	H3N2		1998	USA	A/New_York/256/1998	agtgatgccccattccttgatcggcttcgccgagatcag aggtccctaaggggaagaggcaacactc	-	-
CY002388	Human H3N2 IAVs	Human	H3N2		1998	USA	A/New_York/330/1998	agtgatgccccattccttgatcggcttcgccgagatcag aggtccctaaggggaagaggcaacactc	-	-
CY001508	Human H3N2 IAVs	Human	H3N2		1998	USA	A/New_York/247/1998	agtgatgccccattccttgatcggcttcgccgagatcag aggtccctaaggggaagaggcaacactc	-	-
CY118622	Human H3N2 IAVs	Human	H3N2		1998	Malaysia	A/Malaysia/17392/1998	agtgatgccccattccttgatcggcttcgccgagatcag aggtccctaaggggaagaggcaacactc	-	-
CY118614	Human H3N2 IAVs	Human	H3N2		1998	Malaysia	A/Malaysia/17332/1998	agtgatgccccattccttgatcggcttcgccgagatcag aggtccctaaggggaagaggcaacactc	-	-
CY001788	Human H3N2 IAVs	Human	H3N2		1998	USA	A/New_York/289/1998	agtgatgccccattccttgatcggcttcgccgagatcag aggtccctaaggggaagaggcaacactc	-	-
CY003564	Human H3N2 IAVs	Human	H3N2		1998	USA	A/New_York/448/1998	agtgatgccccattccttgatcggcttcgccgagatcag aggtccctaaggggaagaggcaacactc	-	-
CY114313	Human H3N2 IAVs	Human	H3N2		1998	Netherlands	A/Netherlands/462/1998	agtgatgccccattccttgatcggcttcgccgagatcag aggtccctaaggggaagaggcaacactc	-	-
AF400774	Human H3N2 IAVs	Swine	H3N2		1998	Hong_Kong	A/Swine/Hong_Kong/2422/98	agtgatgccccattccttgatcggcttcgccgagatcag aggtccctaaggggaagaggcaacactc	-	-
CY112897	Human H3N2 IAVs	Human	H3N2		1998	Netherlands	A/Netherlands/414/1998	agtgatgccccattccttgatcggcttcgccgagatcag aggtccctaaggggaagaggcaacactc	-	-
FJ830859	Human H3N2 IAVs	Swine	H3N2		1998	China	A/swine/Guangdong/01/1998	ggtgatgccccattccttgatcggcttcgccgagatcag aagtccctaaggggaagaggcagcactc	+	+
CY001417	Human H3N2 IAVs	Human	H3N2		1999	USA	A/New_York/263/1999	agtgatgccccattccttgatcggcttcgccgagatcag agatccctaaggggaagaggcaacactc	-	*
CY002164	Human H3N2 IAVs	Human	H3N2		1999	USA	A/New_York/316/1999	agtgatgccccattccttgatcggcttcgccgagatcag agatccctaaggggaagaggcaacactc	-	*
CY003612	Human H3N2 IAVs	Human	H3N2		1999	USA	A/New_York/456/1999	agtgatgccccattccttgatcggcttcgccgagatcag agatccctaaggggaagaggcaacactc	-	*
CY003628	Human H3N2 IAVs	Human	H3N2		1999	USA	A/New_York/458/1999	agtgatgccccattccttgatcggcttcgccgagatcag agatccctaaggggaagaggcaacactc	-	*
CY001972	Human H3N2 IAVs	Human	H3N2		1999	USA	A/New_York/278/1999	agtgatgccccattccttgatcggcttcgccgagatcag agatccctaaggggaagaggcaacactc	-	*
CY001908	Human H3N2 IAVs	Human	H3N2		1999	USA	A/New_York/335/1999	agtgatgccccattccttgatcggcttcgccgagatcag agatccctaaggggaagaggcaacactc	-	*
CY118486	Human H3N2 IAVs	Human	H3N2		1999	Malaysia	A/Malaysia/10877/1999	agtgatgccccattccttgatcggcttcgccgagatcag agatccctaaggggaagaggcaacactc	-	*

CY001836	Human H3N2 IAVs	Human	H3N2		1999	USA	A/New_York/315/1999	agtgatgccccattccttgatcggcttcgccgagatcag<br>agatccctaaggggaagaggcaacactc	-	*
CY002116	Human H3N2 IAVs	Human	H3N2		1999	USA	A/Memphis/59/1999	agtgatgccccattccttgatcggcttcgccgagatcag<br>agatccctaaggggaagaggcaacactc	-	*
CY000725	Human H3N2 IAVs	Human	H3N2		1999	USA	A/New_York/177/1999	agtgatgccccattccttgatcggcttcgccgagatcag<br>agatccctaaggggaagaggcaacactc	-	*
CY001980	Human H3N2 IAVs	Human	H3N2		1999	USA	A/New_York/324/1999	agtgatgccccattccttgatcggcttcgccgagatcag<br>agatccctaaggggaagaggcaacactc	-	*
CY001620	Human H3N2 IAVs	Human	H3N2		1999	USA	A/New_York/265/1999	agtgatgccccattccttgatcggcttcgccgagatcag<br>agatccctaaggggaagaggcaacactc	-	*
CY117725	Human H3N2 IAVs	Human	H3N2		1999	Malaysia	A/Malaysia/10816/1999	agtgatgccccattccttgatcggcttcgccgagatcag<br>aggtccctaaggggaagaggcaacactc	-	-
CY119086	Human H3N2 IAVs	Human	H3N2		1999	Malaysia	A/Malaysia/12106/1999	agtgatgccccattccttgatcggcttcgccgagatcag<br>aggtccctaaggggaagaggcaacactc	-	-
CY118518	Human H3N2 IAVs	Human	H3N2		1999	Malaysia	A/Malaysia/12132/1999	agtgatgccccattccttgatcggcttcgccgagatcag<br>aggtccctaaggggaagaggcaacactc	-	-
CY118566	Human H3N2 IAVs	Human	H3N2		1999	Malaysia	A/Malaysia/12974/1999	agtgatgccccattccttgatcggcttcgccgagatcag<br>aggtccctaaggggaagaggcaacactc	-	-
CY118598	Human H3N2 IAVs	Human	H3N2		1999	Malaysia	A/Malaysia/13246/1999	agtgatgccccattccttgatcggcttcgccgagatcag<br>aggtccctaaggggaagaggcaacactc	-	-
CY112913	Human H3N2 IAVs	Human	H3N2		1999	Russia	A/Moscow/10/1999	agtgatgccccattccttgatcggcttcgccgagatcag<br>aggtccctaaggggaagaggcaacactc	-	-
DQ487332	Human H3N2 IAVs	Human	H3N2		1999	Russia	A/Moscow/10/99	agtgatgccccattccttgatcggcttcgccgagatcag<br>aggtccctaaggggaagaggcaacactc	-	-
CY016527	Human H3N2 IAVs	Human	H3N2		1999	Australia	A/New_South_Wales/6/1999	agtgatgccccattccttgatcggcttcgccgagatcag<br>aggtccctaaggggaagaggcaacactc	-	-
CY016535	Human H3N2 IAVs	Human	H3N2		1999	Australia	A/New_South_Wales/8/1999	agtgatgccccattccttgatcggcttcgccgagatcag<br>aggtccctaaggggaagaggcaacactc	-	-
CY121377	Human H3N2 IAVs	Human	H3N2		1999	Russia	A/Moscow/10/1999	agtgatgccccattccttgatcggcttcgccgagatcag<br>aggtccctaaggggaagaggcaacactc	-	-
CY020209	Human H3N2 IAVs	Human	H3N2		1999	Australia	A/New_South_Wales/19/1999	agtgatgccccattccttgatcggcttcgccgagatcag<br>aggtccctaaggggaagaggcaacactc	-	-
CY021065	Human H3N2 IAVs	Human	H3N2		1999	Australia	A/New_South_Wales/3/1999	agtgatgccccattccttgatcggcttcgccgagatcag<br>aggtccctaaggggaagaggcaacactc	-	-
CY020201	Human H3N2 IAVs	Human	H3N2		1999	Australia	A/New_South_Wales/7/1999	agtgatgccccattccttgatcggcttcgccgagatcag<br>aggtccctaaggggaagaggcaacactc	-	-
CY118558	Human H3N2 IAVs	Human	H3N2		1999	Malaysia	A/Malaysia/12942/1999	agtgatgccccattccttgatcggcttcgccgagatcag<br>aggtccctaaggggaagaggcaacactc	-	-
CY118574	Human H3N2 IAVs	Human	H3N2		1999	Malaysia	A/Malaysia/12990/1999	agtgatgccccattccttgatcggcttcgccgagatcag<br>aggtccctaaggggaagaggcaacactc	-	-
CY118582	Human H3N2 IAVs	Human	H3N2		1999	Malaysia	A/Malaysia/13002/1999	agtgatgccccattccttgatcggcttcgccgagatcag<br>aggtccctaaggggaagaggcaacactc	-	-
CY118526	Human H3N2 IAVs	Human	H3N2		1999	Malaysia	A/Malaysia/12550/1999	agtgatgccccattccttgatcggcttcgccgagatcag<br>aggtccctaaggggaagaggcaacactc	-	-
CY016655	Human H3N2 IAVs	Human	H3N2		1999	Australia	A/New_South_Wales/22/1999	agtgatgccccattccttgatcggcttcgccgagatcag<br>aggtccctaaggggaagaggcaacactc	-	-
AY363581	Human H3N2 IAVs	Swine	H3N2		1999	Hong_Kong	A/Swine/Hong_Kong/4361/99	agtgatgccccattccttgatcggcttcgccgagatcag<br>aggtccctaaggggaagaggcaacactc	-	-
CY118590	Human H3N2 IAVs	Human	H3N2		1999	Malaysia	A/Malaysia/13094/1999	agtgatgccccattccttgatcggcttcgccgagatcag<br>aggtccctaaggggaagaggcaacactc	-	-

CY118502	Human H3N2 IAVs	Human	H3N2		1999	Malaysia	A/Malaysia/10997/1999	agtgatgccccattccttgatcggcttcgccgagatcag aggtccctaaggggaagaggcaacactc	-	-
CY000805	Human H3N2 IAVs	Human	H3N2		1999	USA	A/New_York/141/1999	agtgatgccccattccttgatcggcttcgccgagatcag aggtccctaaggggaagaggcaacactc	-	-
CY016631	Human H3N2 IAVs	Human	H3N2		1999	Australia	A/New_South_Wales/4/1999	agtgatgccccattccttgatcggcttcgccgagatcag aggtccctaaggggaagaggcaacactc	-	-
CY118542	Human H3N2 IAVs	Human	H3N2		1999	Malaysia	A/Malaysia/12766/1999	agtgatgccccattccttgatcggcttcgccgagatcag aggtccctaaggggaagaggcaacactc	-	-
CY118550	Human H3N2 IAVs	Human	H3N2		1999	Malaysia	A/Malaysia/12904/1999	agtgatgccccattccttgatcggcttcgccgagatcag aggtccctaaggggaagaggcaacactc	-	-
CY117764	Human H3N2 IAVs	Human	H3N2		1999	Malaysia	A/Malaysia/13328/1999	agtgatgccccattccttgatcggcttcgccgagatcag aggtccctaaggggaagaggcaacactc	-	-
CY117804	Human H3N2 IAVs	Human	H3N2		1999	Malaysia	A/Malaysia/13381/1999	agtgatgccccattccttgatcggcttcgccgagatcag aggtccctaaggggaagaggcaacactc	-	-
CY117772	Human H3N2 IAVs	Human	H3N2		1999	Malaysia	A/Malaysia/13329/1999	agtgatgccccattccttgatcggcttcgccgagatcag aggtccctaaggggaagaggcaacactc	-	-
CY006072	Human H3N2 IAVs	Human	H3N2		1999	USA	A/New_York/455/1999	agtgatgccccattccttgatcggcttcgccgagatcag aggtccctaaggggaagaggcaacactc	-	-
CY002516	Human H3N2 IAVs	Human	H3N2		1999	USA	A/New_York/140/1999	agtgatgccccattccttgatcggcttcgccgagatcag aggtccctaaggggaagaggcaacactc	-	-
CY000461	Human H3N2 IAVs	Human	H3N2		1999	USA	A/New_York/149/1999	agtgatgccccattccttgatcggcttcgccgagatcag aggtccctaaggggaagaggcaacactc	-	-
CY000621	Human H3N2 IAVs	Human	H3N2		1999	USA	A/New_York/161/1999	agtgatgccccattccttgatcggcttcgccgagatcag aggtccctaaggggaagaggcaacactc	-	-
CY001457	Human H3N2 IAVs	Human	H3N2		1999	USA	A/New_York/184/1999	agtgatgccccattccttgatcggcttcgccgagatcag aggtccctaaggggaagaggcaacactc	-	-
CY000861	Human H3N2 IAVs	Human	H3N2		1999	USA	A/New_York/186/1999	agtgatgccccattccttgatcggcttcgccgagatcag aggtccctaaggggaagaggcaacactc	-	-
CY003436	Human H3N2 IAVs	Human	H3N2		1999	USA	A/New_York/421/1999	agtgatgccccattccttgatcggcttcgccgagatcag aggtccctaaggggaagaggcaacactc	-	-
CY003444	Human H3N2 IAVs	Human	H3N2		1999	USA	A/New_York/425/1999	agtgatgccccattccttgatcggcttcgccgagatcag aggtccctaaggggaagaggcaacactc	-	-
CY090905	Human H3N2 IAVs	Human	H3N2		1999	USA	A/South_Carolina/NHRC0001/1999	agtgatgccccattccttgatcggcttcgccgagatcag aggtccctaaggggaagaggcaacactc	-	-
CY121428	Human H3N2 IAVs	Human	H3N2		1999	USA	A/California/32/1999	agtgatgccccattccttgatcggcttcgccgagatcag aggtccctaaggggaagaggcaacactc	-	-
CY000653	Human H3N2 IAVs	Human	H3N2		1999	USA	A/New_York/167/1999	agtgatgccccattccttgatcggcttcgccgagatcag aggtccctaaggggaagaggcaacactc	-	-
CY000685	Human H3N2 IAVs	Human	H3N2		1999	USA	A/New_York/172/1999	agtgatgccccattccttgatcggcttcgccgagatcag aggtccctaaggggaagaggcaacactc	-	-
CY090889	Human H3N2 IAVs	Human	H3N2		1999	USA	A/Georgia/NHRC0001/1999	agtgatgccccattccttgatcggcttcgccgagatcag aggtccctaaggggaagaggcaacactc	-	-
CY117732	Human H3N2 IAVs	Human	H3N2		1999	Malaysia	A/Malaysia/10879/1999	agtgatgccccattccttgatcggcttcgccgagatcag aggtccctaaggggaagaggcaacactc	-	-
CY118478	Human H3N2 IAVs	Human	H3N2		1999	Malaysia	A/Malaysia/10717/1999	agtgatgccccattccttgatcggcttcgccgagatcag aggtccctaaggggaagaggcaacactc	-	-
CY118470	Human H3N2 IAVs	Human	H3N2		1999	Malaysia	A/Malaysia/10807/1999	agtgatgccccattccttgatcggcttcgccgagatcag aggtccctaaggggaagaggcaacactc	-	-
CY118494	Human H3N2 IAVs	Human	H3N2		1999	Malaysia	A/Malaysia/10969/1999	agtgatgccccattccttgatcggcttcgccgagatcag aggtccctaaggggaagaggcaacactc	-	-

CY077851	Human H3N2 IAVs	Human	H3N2		1999	Netherlands	A/Netherlands/301/1999	agtgatgccccattccttgatcggcttcgccgagatcag aggtccctaaggggaagaggcaacactc	-	-
CY016519	Human H3N2 IAVs	Human	H3N2		1999	Australia	A/New_South_Wales/5/1999	agtgatgccccattccttgatcggcttcgccgagatcag aggtccctaaggggaagaggcaacactc	-	-
CY001708	Human H3N2 IAVs	Human	H3N2		1999	USA	A/New_York/257/1999	agtgatgccccattccttgatcggcttcgccgagatcag aggtccctaaggggaagaggcaacactc	-	-
CY002148	Human H3N2 IAVs	Human	H3N2		1999	USA	A/New_York/285/1999	agtgatgccccattccttgatcggcttcgccgagatcag aggtccctaaggggaagaggcaacactc	-	-
CY114497	Human H3N2 IAVs	Human	H3N2		1999	Netherlands	A/Netherlands/301/1999	agtgatgccccattccttgatcggcttcgccgagatcag aggtccctaaggggaagaggcaacactc	-	-
CY001385	Human H3N2 IAVs	Human	H3N2		1999	USA	A/New_York/145/1999	agtgatgccccattccttgatcggcttcgccgagatcag aggtccctaaggggaagaggcaacactc	-	-
CY001692	Human H3N2 IAVs	Human	H3N2		1999	USA	A/New_York/248/1999	agtgatgccccattccttgatcggcttcgccgagatcag aggtccctaaggggaagaggcaacactc	-	-
CY001580	Human H3N2 IAVs	Human	H3N2		1999	USA	A/New_York/253/1999	agtgatgccccattccttgatcggcttcgccgagatcag aggtccctaaggggaagaggcaacactc	-	-
CY001756	Human H3N2 IAVs	Human	H3N2		1999	USA	A/New_York/279/1999	agtgatgccccattccttgatcggcttcgccgagatcag aggtccctaaggggaagaggcaacactc	-	-
CY001764	Human H3N2 IAVs	Human	H3N2		1999	USA	A/New_York/280/1999	agtgatgccccattccttgatcggcttcgccgagatcag aggtccctaaggggaagaggcaacactc	-	-
CY001772	Human H3N2 IAVs	Human	H3N2		1999	USA	A/New_York/282/1999	agtgatgccccattccttgatcggcttcgccgagatcag aggtccctaaggggaagaggcaacactc	-	-
CY001780	Human H3N2 IAVs	Human	H3N2		1999	USA	A/New_York/288/1999	agtgatgccccattccttgatcggcttcgccgagatcag aggtccctaaggggaagaggcaacactc	-	-
CY001796	Human H3N2 IAVs	Human	H3N2		1999	USA	A/New_York/290/1999	agtgatgccccattccttgatcggcttcgccgagatcag aggtccctaaggggaagaggcaacactc	-	-
CY001812	Human H3N2 IAVs	Human	H3N2		1999	USA	A/New_York/311/1999	agtgatgccccattccttgatcggcttcgccgagatcag aggtccctaaggggaagaggcaacactc	-	-
CY001844	Human H3N2 IAVs	Human	H3N2		1999	USA	A/New_York/317/1999	agtgatgccccattccttgatcggcttcgccgagatcag aggtccctaaggggaagaggcaacactc	-	-
CY001852	Human H3N2 IAVs	Human	H3N2		1999	USA	A/New_York/318/1999	agtgatgccccattccttgatcggcttcgccgagatcag aggtccctaaggggaagaggcaacactc	-	-
CY001860	Human H3N2 IAVs	Human	H3N2		1999	USA	A/New_York/320/1999	agtgatgccccattccttgatcggcttcgccgagatcag aggtccctaaggggaagaggcaacactc	-	-
CY001868	Human H3N2 IAVs	Human	H3N2		1999	USA	A/New_York/321/1999	agtgatgccccattccttgatcggcttcgccgagatcag aggtccctaaggggaagaggcaacactc	-	-
CY001876	Human H3N2 IAVs	Human	H3N2		1999	USA	A/New_York/323/1999	agtgatgccccattccttgatcggcttcgccgagatcag aggtccctaaggggaagaggcaacactc	-	-
CY001892	Human H3N2 IAVs	Human	H3N2		1999	USA	A/New_York/329/1999	agtgatgccccattccttgatcggcttcgccgagatcag aggtccctaaggggaagaggcaacactc	-	-
CY001988	Human H3N2 IAVs	Human	H3N2		1999	USA	A/New_York/331/1999	agtgatgccccattccttgatcggcttcgccgagatcag aggtccctaaggggaagaggcaacactc	-	-
CY001900	Human H3N2 IAVs	Human	H3N2		1999	USA	A/New_York/333/1999	agtgatgccccattccttgatcggcttcgccgagatcag aggtccctaaggggaagaggcaacactc	-	-
CY006167	Human H3N2 IAVs	Human	H3N2		1999	USA	A/New_York/397/1999	agtgatgccccattccttgatcggcttcgccgagatcag aggtccctaaggggaagaggcaacactc	-	-
CY003797	Human H3N2 IAVs	Human	H3N2		1999	USA	A/New_York/423/1999	agtgatgccccattccttgatcggcttcgccgagatcag aggtccctaaggggaagaggcaacactc	-	-
CY003588	Human H3N2 IAVs	Human	H3N2		1999	USA	A/New_York/451/1999	agtgatgccccattccttgatcggcttcgccgagatcag aggtccctaaggggaagaggcaacactc	-	-

CY003596	Human H3N2 IAVs	Human	H3N2		1999	USA	A/New_York/453/1999	agtgatgccccattccttgatcggcttcgccgagatcag<br>aggtccctaaggggaagaggcaacactc	-	-
CY003604	Human H3N2 IAVs	Human	H3N2		1999	USA	A/New_York/454/1999	agtgatgccccattccttgatcggcttcgccgagatcag<br>aggtccctaaggggaagaggcaacactc	-	-
CY003636	Human H3N2 IAVs	Human	H3N2		1999	USA	A/New_York/459/1999	agtgatgccccattccttgatcggcttcgccgagatcag<br>aggtccctaaggggaagaggcaacactc	-	-
CY001932	Human H3N2 IAVs	Human	H3N2		1999	USA	A/New_York/338/1999	agtgatgccccattccttgatcggcttcgccgagatcag<br>aggtccctaaggggaagaggcaacactc	-	-
CY003620	Human H3N2 IAVs	Human	H3N2		1999	USA	A/New_York/457/1999	agtgatgccccattccttgatcggcttcgccgagatcag<br>aggtccctaaggggaagaggcaacactc	-	-
CY001700	Human H3N2 IAVs	Human	H3N2		1999	USA	A/New_York/255/1999	agtgatgccccattccttgatcggcttcgccgagatcag<br>aggtccctaaggggaagaggcaacactc	-	-
CY002372	Human H3N2 IAVs	Human	H3N2		1999	USA	A/New_York/325/1999	agtgatgccccattccttgatcggcttcgccgagatcag<br>aggtccctaaggggaagaggcaacactc	-	-
CY002308	Human H3N2 IAVs	Human	H3N2		1999	USA	A/New_York/398/1999	agtgatgccccattccttgatcggcttcgccgagatcag<br>aggtccctaaggggaagaggcaacactc	-	-
CY003580	Human H3N2 IAVs	Human	H3N2		1999	USA	A/New_York/450/1999	agtgatgccccattccttgatcggcttcgccgagatcag<br>aggtccctaaggggaagaggcaacactc	-	-
CY003572	Human H3N2 IAVs	Human	H3N2		1999	USA	A/New_York/449/1999	agtgatgccccattccttgatcggcttcgccgagatcag<br>aggtccctaaggggaagaggcaacactc	-	-
CY002556	Human H3N2 IAVs	Human	H3N2		1999	USA	A/New_York/252/1999	agtgatgccccattccttgatcggcttcgccgagatcag<br>aggtccctaaggggaagaggcaacactc	-	-
CY001596	Human H3N2 IAVs	Human	H3N2		1999	USA	A/New_York/259/1999	agtgatgccccattccttgatcggcttcgccgagatcag<br>aggtccctaaggggaagaggcaacactc	-	-
CY002172	Human H3N2 IAVs	Human	H3N2		1999	USA	A/New_York/322/1999	agtgatgccccattccttgatcggcttcgccgagatcag<br>aggtccctaaggggaagaggcaacactc	-	-
CY001884	Human H3N2 IAVs	Human	H3N2		1999	USA	A/New_York/327/1999	agtgatgccccattccttgatcggcttcgccgagatcag<br>aggtccctaaggggaagaggcaacactc	-	-
CY001916	Human H3N2 IAVs	Human	H3N2		1999	USA	A/New_York/336/1999	agtgatgccccattccttgatcggcttcgccgagatcag<br>aggtccctaaggggaagaggcaacactc	-	-
CY006903	Human H3N2 IAVs	Human	H3N2		1999	USA	A/New_York/460/1999	agtgatgccccattccttgatcggcttcgccgagatcag<br>aggtccctaaggggaagaggcaacactc	-	-
CY001604	Human H3N2 IAVs	Human	H3N2		1999	USA	A/New_York/262/1999	agtgatgccccattccttgatcggcttcgccgagatcag<br>aggtccctaaggggaagaggcaacactc	-	-
CY001828	Human H3N2 IAVs	Human	H3N2		1999	USA	A/New_York/314/1999	agtgatgccccattccttgatcggcttcgccgagatcag<br>aggtccctaaggggaagaggcaacactc	-	-
CY001748	Human H3N2 IAVs	Human	H3N2		1999	USA	A/New_York/277/1999	agtgatgccccattccttgatcggcttcgccgagatcag<br>aggtccctaaggggaagaggcaacactc	-	-
CY002140	Human H3N2 IAVs	Human	H3N2		1999	USA	A/New_York/283/1999	agtgatgccccattccttgatcggcttcgccgagatcag<br>aggtccctaaggggaagaggcaacactc	-	-
CY006064	Human H3N2 IAVs	Human	H3N2		1999	USA	A/New_York/452/1999	agtgatgccccattccttgatcggcttcgccgagatcag<br>aggtccctaaggggaagaggcaacactc	-	-
CY001940	Human H3N2 IAVs	Human	H3N2		1999	USA	A/New_York/340/1999	agtgatgccccattccttgatcggcttcgccgagatcag<br>aggtccctaaggggaagaggcaacactc	-	-
CY001964	Human H3N2 IAVs	Human	H3N2		1999	USA	A/New_York/260/1999	agtgatgccccattccttgatcggcttcgccgagatcag<br>aggtccctaaggggaagaggcaacactc	-	-
CY002300	Human H3N2 IAVs	Human	H3N2		1999	USA	A/New_York/347/1999	agtgatgccccattccttgatcggcttcgccgagatcag<br>aggtccctaaggggaagaggcaacactc	-	-
CY001668	Human H3N2 IAVs	Human	H3N2		1999	USA	A/New_York/286/1999	agtgatgccccattccttgatcggcttcgccgagatcag<br>aggtccctaaggggaagaggcaacactc	-	-

CY001996	Human H3N2 IAVs	Human	H3N2		1999	USA	A/New_York/332/1999	agtgatgccccattccttgatcggcttcgccgagatcag aggtccctaaggggaagaggcaacactc	-	-
CY016072	Human H3N2 IAVs	Human	H3N2		1999	Australia	A/New_South_Wales/13/1999	agtgatgccccattccttgatcggcttcgccgagatcag aggtccctaaggggaagaggcaacactc	-	-
CY016551	Human H3N2 IAVs	Human	H3N2		1999	Australia	A/New_South_Wales/16/1999	agtgatgccccattccttgatcggcttcgccgagatcag aggtccctaaggggaagaggcaacactc	-	-
CY016511	Human H3N2 IAVs	Human	H3N2		1999	Australia	A/New_South_Wales/2/1999	agtgatgccccattccttgatcggcttcgccgagatcag aggtccctaaggggaagaggcaacactc	-	-
CY007639	Human H3N2 IAVs	Human	H3N2		1999	USA	A/New_York/326/1999	agtgatgccccattccttgatcggcttcgccgagatcag aggtccctaaggggaagaggcaacactc	-	-
CY118638	Human H3N2 IAVs	Human	H3N2		1999	Malaysia	A/Malaysia/10759/1999	agtgatgccccattccttgatcggcttcgccgagatcag aggtccctaaggggaagaggcaacactc	-	-
CY000993	Human H3N2 IAVs	Human	H3N2		1999	USA	A/New_York/138/1999	agtgatgccccattccttgatcggcttcgccgagatcag aggtccctaaggggaagaggcaacactc	-	-
CY001660	Human H3N2 IAVs	Human	H3N2		1999	USA	A/New_York/284/1999	agtgatgccccattccttgatcggcttcgccgagatcag aggtccctaaggggaagaggcaacactc	-	-
CY001924	Human H3N2 IAVs	Human	H3N2		1999	USA	A/New_York/337/1999	agtgatgccccattccttgatcggcttcgccgagatcag aggtccctaaggggaagaggcaacactc	-	-
CY002564	Human H3N2 IAVs	Human	H3N2		1999	USA	A/New_York/261/1999	agtgatgccccattccttgatcggcttcgccgagatcag aggtccctaaggggaagaggcaacactc	-	-
CY117740	Human H3N2 IAVs	Human	H3N2		1999	Malaysia	A/Malaysia/10970/1999	agtgatgccccattccttgatcggcttcgccgagatcag aggtccctaaggggaagaggcaacactc	-	-
CY002340	Human H3N2 IAVs	Human	H3N2		1999	USA	A/New_York/266/1999	agtgatgccccattccttgatcggcttcgccgagatcag aggtccctaaggggaagaggcaacactc	-	-
CY001124	Human H3N2 IAVs	Human	H3N2		1999	USA	A/New_York/137/1999	agtgatgccccattccttgatcggcttcgccgagatcag aggtccctaaggggaagaggcaacactc	-	-
CY112921	Human H3N2 IAVs	Human	H3N2		1999	Panama	A/Panama/2007/1999	agtgatgccccattccttgatcggcttcgccgagatcag aggtccctaaggggaagaggcaacactc	-	-
DQ508869	Human H3N2 IAVs	Human	H3N2		1999	Panama	A/Panama/2007/1999	agtgatgccccattccttgatcggcttcgccgagatcag aggtccctaaggggaagaggcaacactc	-	-
AM502801	Human H3N2 IAVs	Human	H3N2		1999	Panama	A/Panama/2007/1999	agtgatgccccattccttgatcggcttcgccgagatcag aggtccctaaggggaagaggcaacactc	-	-
DQ487336	Human H3N2 IAVs	Human	H3N2		1999	Panama	A/Panama/2007/1999	agtgatgccccattccttgatcggcttcgccgagatcag aggtccctaaggggaagaggcaacactc	-	-
CY034104	Human H3N2 IAVs	Human	H3N2		1999	Panama	A/Panama/2007/1999	agtgatgccccattccttgatcggcttcgccgagatcag aggtccctaaggggaagaggcaacactc	-	-
CY001612	Human H3N2 IAVs	Human	H3N2		1999	USA	A/New_York/264/1999	agtgatgccccattccttgatcggcttcgccgagatcag aggtccctaaggggaagaggcaacactc	-	-
CY002580	Human H3N2 IAVs	Human	H3N2		1999	USA	A/New_York/339/1999	agtgatgccccattccttgatcggcttcgccgagatcag aggtccctaaggggaagaggcaacactc	-	-
CY016080	Human H3N2 IAVs	Human	H3N2		1999	Australia	A/New_South_Wales/20/1999	agtgatgccccattccttgatcggcttcgccgagatcag aggtccctaaggggaaggggcaacactc	-	-
CY016559	Human H3N2 IAVs	Human	H3N2		1999	Australia	A/New_South_Wales/21/1999	agtgatgccccattccttgatcggcttcgccgagatcag aggtccctaaggggaaggggcaacactc	-	-
CY009112	Human H3N2 IAVs	Human	H3N2		1999	New_Zealand	A/Canterbury/179/1999	agtgatgcccccttccttgatcggcttcgccgagatcag aggtccctaaggggaagaggcaacactc	-	-
CY000597	Human H3N2 IAVs	Human	H3N2		1999	USA	A/New_York/147/1999	agtgatgcccccttccttgatcggcttcgccgagatcag aggtccctaaggggaagaggcaacactc	-	-
CY000749	Human H3N2 IAVs	Human	H3N2		1999	USA	A/New_York/185/1999	agtgatgcccccttccttgatcggcttcgccgagatcag aggtccctaaggggaagaggcaacactc	-	-

CY001009	Human H3N2 IAVs	Human	H3N2		1999	USA	A/New_York/189/1999	agtgatgcccccttccttgatcggcttcgccgagatcag aggtccctaaggggaagaggcaacactc	-	-
CY001353	Human H3N2 IAVs	Human	H3N2		1999	USA	A/New_York/139/1999	agtgatgcccccttccttgatcggcttcgccgagatcag aggtccctaaggggaagaggcaacactc	-	-
CY000637	Human H3N2 IAVs	Human	H3N2		1999	USA	A/New_York/143/1999	agtgatgcccccttccttgatcggcttcgccgagatcag aggtccctaaggggaagaggcaacactc	-	-
CY000605	Human H3N2 IAVs	Human	H3N2		1999	USA	A/New_York/151/1999	agtgatgcccccttccttgatcggcttcgccgagatcag aggtccctaaggggaagaggcaacactc	-	-
CY002316	Human H3N2 IAVs	Human	H3N2		1999	USA	A/New_York/153/1999	agtgatgcccccttccttgatcggcttcgccgagatcag aggtccctaaggggaagaggcaacactc	-	-
CY001449	Human H3N2 IAVs	Human	H3N2		1999	USA	A/New_York/155/1999	agtgatgcccccttccttgatcggcttcgccgagatcag aggtccctaaggggaagaggcaacactc	-	-
CY000829	Human H3N2 IAVs	Human	H3N2		1999	USA	A/New_York/157/1999	agtgatgcccccttccttgatcggcttcgccgagatcag aggtccctaaggggaagaggcaacactc	-	-
CY000645	Human H3N2 IAVs	Human	H3N2		1999	USA	A/New_York/163/1999	agtgatgcccccttccttgatcggcttcgccgagatcag aggtccctaaggggaagaggcaacactc	-	-
CY001273	Human H3N2 IAVs	Human	H3N2		1999	USA	A/New_York/164/1999	agtgatgcccccttccttgatcggcttcgccgagatcag aggtccctaaggggaagaggcaacactc	-	-
CY000813	Human H3N2 IAVs	Human	H3N2		1999	USA	A/New_York/166/1999	agtgatgcccccttccttgatcggcttcgccgagatcag aggtccctaaggggaagaggcaacactc	-	-
CY000733	Human H3N2 IAVs	Human	H3N2		1999	USA	A/New_York/179/1999	agtgatgcccccttccttgatcggcttcgccgagatcag aggtccctaaggggaagaggcaacactc	-	-
CY001180	Human H3N2 IAVs	Human	H3N2		1999	USA	A/New_York/181/1999	agtgatgcccccttccttgatcggcttcgccgagatcag aggtccctaaggggaagaggcaacactc	-	-
CY001361	Human H3N2 IAVs	Human	H3N2		1999	USA	A/New_York/183/1999	agtgatgcccccttccttgatcggcttcgccgagatcag aggtccctaaggggaagaggcaacactc	-	-
CY001532	Human H3N2 IAVs	Human	H3N2		1999	USA	A/New_York/188/1999	agtgatgcccccttccttgatcggcttcgccgagatcag aggtccctaaggggaagaggcaacactc	-	-
CY003789	Human H3N2 IAVs	Human	H3N2		1999	USA	A/New_York/422/1999	agtgatgcccccttccttgatcggcttcgccgagatcag aggtccctaaggggaagaggcaacactc	-	-
CY003228	Human H3N2 IAVs	Human	H3N2		1999	USA	A/New_York/426/1999	agtgatgcccccttccttgatcggcttcgccgagatcag aggtccctaaggggaagaggcaacactc	-	-
AY363590	Human H3N2 IAVs	Swine	H3N2		1999	Hong_Kong	A/Swine/Hong_Kong/q066/99	agtgatgcccccttccttgatcggcttcgccgagatcag aggtccctaaggggaagaggcaacactc	-	-
CY090897	Human H3N2 IAVs	Human	H3N2		1999	USA	A/Illinois/NHRC0001/1999	agtgatgcccccttccttgatcggcttcgccgagatcag aggtccctaaggggaagaggcaacactc	-	-
CY000677	Human H3N2 IAVs	Human	H3N2		1999	USA	A/New_York/171/1999	agtgatgcccccttccttgatcggcttcgccgagatcag aggtccctaaggggaagaggcaacactc	-	-
CY003236	Human H3N2 IAVs	Human	H3N2		1999	USA	A/New_York/427/1999	agtgatgcccccttccttgatcggcttcgccgagatcag aggtccctaaggggaagaggcaacactc	-	-
CY003220	Human H3N2 IAVs	Human	H3N2		1999	USA	A/New_York/424/1999	agtgatgcccccttccttgatcggcttcgccgagatcag aggtccctaaggggaagaggcaacactc	-	-
CY016543	Human H3N2 IAVs	Human	H3N2		1999	Australia	A/New_South_Wales/15/1999	agtgatgctccattccttgatcggcttcgccgagatcag aggtccctaaggggaagaggcaacactc	-	-
CY016639	Human H3N2 IAVs	Human	H3N2		1999	Australia	A/New_South_Wales/17/1999	agtgatgctccattccttgatcggcttcgccgagatcag aggtccctaaggggaagaggcaacactc	-	-
CY114321	Human H3N2 IAVs	Human	H3N2		2000	Netherlands	A/Netherlands/3/2000	agtgatgccccattccttgaccggcttcgccgagatca gaggtccctaaggggaagaggcaacactc	-	-
CY016711	Human H3N2 IAVs	Human	H3N2		2000	Australia	A/South_Australia/59/2000	agtgatgccccattccttgatcggcttcgccgagatcag agatccctaaagggaagaggcaacactc	-	-

CY022505	Human H3N2 IAVs	Human	H3N2		2000	New_Zealand	A/Auckland/588/2000	agtgatgccccattccttgatcggcttcgccgagatcag<br>agatccctaaggggaagaggcaacactc	-	*
CY023006	Human H3N2 IAVs	Human	H3N2		2000	New_Zealand	A/Auckland/589/2000	agtgatgccccattccttgatcggcttcgccgagatcag<br>agatccctaaggggaagaggcaacactc	-	*
CY022521	Human H3N2 IAVs	Human	H3N2		2000	New_Zealand	A/Auckland/593/2000	agtgatgccccattccttgatcggcttcgccgagatcag<br>agatccctaaggggaagaggcaacactc	-	*
CY023038	Human H3N2 IAVs	Human	H3N2		2000	New_Zealand	A/Auckland/599/2000	agtgatgccccattccttgatcggcttcgccgagatcag<br>agatccctaaggggaagaggcaacactc	-	*
CY023054	Human H3N2 IAVs	Human	H3N2		2000	New_Zealand	A/Auckland/601/2000	agtgatgccccattccttgatcggcttcgccgagatcag<br>agatccctaaggggaagaggcaacactc	-	*
CY008840	Human H3N2 IAVs	Human	H3N2		2000	New_Zealand	A/Canterbury/103/2000	agtgatgccccattccttgatcggcttcgccgagatcag<br>agatccctaaggggaagaggcaacactc	-	*
CY008135	Human H3N2 IAVs	Human	H3N2		2000	New_Zealand	A/Canterbury/42/2000	agtgatgccccattccttgatcggcttcgccgagatcag<br>agatccctaaggggaagaggcaacactc	-	*
CY008752	Human H3N2 IAVs	Human	H3N2		2000	New_Zealand	A/Canterbury/56/2000	agtgatgccccattccttgatcggcttcgccgagatcag<br>agatccctaaggggaagaggcaacactc	-	*
CY008760	Human H3N2 IAVs	Human	H3N2		2000	New_Zealand	A/Canterbury/68/2000	agtgatgccccattccttgatcggcttcgccgagatcag<br>agatccctaaggggaagaggcaacactc	-	*
CY008776	Human H3N2 IAVs	Human	H3N2		2000	New_Zealand	A/Canterbury/71/2000	agtgatgccccattccttgatcggcttcgccgagatcag<br>agatccctaaggggaagaggcaacactc	-	*
CY016096	Human H3N2 IAVs	Human	H3N2		2000	Australia	A/New_South_Wales/27/2000	agtgatgccccattccttgatcggcttcgccgagatcag<br>agatccctaaggggaagaggcaacactc	-	*
CY016575	Human H3N2 IAVs	Human	H3N2		2000	Australia	A/New_South_Wales/32/2000	agtgatgccccattccttgatcggcttcgccgagatcag<br>agatccctaaggggaagaggcaacactc	-	*
CY016272	Human H3N2 IAVs	Human	H3N2		2000	Australia	A/New_South_Wales/33/2000	agtgatgccccattccttgatcggcttcgccgagatcag<br>agatccctaaggggaagaggcaacactc	-	*
CY016583	Human H3N2 IAVs	Human	H3N2		2000	Australia	A/New_South_Wales/34/2000	agtgatgccccattccttgatcggcttcgccgagatcag<br>agatccctaaggggaagaggcaacactc	-	*
CY016591	Human H3N2 IAVs	Human	H3N2		2000	Australia	A/New_South_Wales/35/2000	agtgatgccccattccttgatcggcttcgccgagatcag<br>agatccctaaggggaagaggcaacactc	-	*
CY021745	Human H3N2 IAVs	Human	H3N2		2000	Australia	A/South_Australia/11/2000	agtgatgccccattccttgatcggcttcgccgagatcag<br>agatccctaaggggaagaggcaacactc	-	*
CY021921	Human H3N2 IAVs	Human	H3N2		2000	Australia	A/South_Australia/16/2000	agtgatgccccattccttgatcggcttcgccgagatcag<br>agatccctaaggggaagaggcaacactc	-	*
CY021929	Human H3N2 IAVs	Human	H3N2		2000	Australia	A/South_Australia/47/2000	agtgatgccccattccttgatcggcttcgccgagatcag<br>agatccctaaggggaagaggcaacactc	-	*
CY020017	Human H3N2 IAVs	Human	H3N2		2000	Australia	A/South_Australia/49/2000	agtgatgccccattccttgatcggcttcgccgagatcag<br>agatccctaaggggaagaggcaacactc	-	*
CY017167	Human H3N2 IAVs	Human	H3N2		2000	Australia	A/South_Australia/52/2000	agtgatgccccattccttgatcggcttcgccgagatcag<br>agatccctaaggggaagaggcaacactc	-	*
CY016743	Human H3N2 IAVs	Human	H3N2		2000	Australia	A/South_Australia/66/2000	agtgatgccccattccttgatcggcttcgccgagatcag<br>agatccctaaggggaagaggcaacactc	-	*
CY016751	Human H3N2 IAVs	Human	H3N2		2000	Australia	A/South_Australia/67/2000	agtgatgccccattccttgatcggcttcgccgagatcag<br>agatccctaaggggaagaggcaacactc	-	*
CY017391	Human H3N2 IAVs	Human	H3N2		2000	Australia	A/South_Australia/75/2000	agtgatgccccattccttgatcggcttcgccgagatcag<br>agatccctaaggggaagaggcaacactc	-	*
CY020025	Human H3N2 IAVs	Human	H3N2		2000	Australia	A/South_Australia/80/2000	agtgatgccccattccttgatcggcttcgccgagatcag<br>agatccctaaggggaagaggcaacactc	-	*
CY013387	Human H3N2 IAVs	Human	H3N2		2000	New_Zealand	A/Waikato/15/2000	agtgatgccccattccttgatcggcttcgccgagatcag<br>agatccctaaggggaagaggcaacactc	-	*

CY013401	Human H3N2 IAVs	Human	H3N2		2000	New_Zealand	A/Waikato/17/2000	agtgatgccccattccttgatcggcttcgccgagatcag agatccctaaggggaagaggcaacactc	-	*
CY013068	Human H3N2 IAVs	Human	H3N2		2000	New_Zealand	A/Waikato/20/2000	agtgatgccccattccttgatcggcttcgccgagatcag agatccctaaggggaagaggcaacactc	-	*
CY015672	Human H3N2 IAVs	Human	H3N2		2000	Australia	A/Western_Australia/11/2000	agtgatgccccattccttgatcggcttcgccgagatcag agatccctaaggggaagaggcaacactc	-	*
CY016503	Human H3N2 IAVs	Human	H3N2		2000	Australia	A/Western_Australia/12/2000	agtgatgccccattccttgatcggcttcgccgagatcag agatccctaaggggaagaggcaacactc	-	*
CY015656	Human H3N2 IAVs	Human	H3N2		2000	Australia	A/Western_Australia/3/2000	agtgatgccccattccttgatcggcttcgccgagatcag agatccctaaggggaagaggcaacactc	-	*
CY017335	Human H3N2 IAVs	Human	H3N2		2000	Australia	A/Western_Australia/5/2000	agtgatgccccattccttgatcggcttcgccgagatcag agatccctaaggggaagaggcaacactc	-	*
CY111526	Human H3N2 IAVs	Human	H3N2		2000	Australia	A/Western_Australia/6/2000	agtgatgccccattccttgatcggcttcgccgagatcag agatccctaaggggaagaggcaacactc	-	*
CY017351	Human H3N2 IAVs	Human	H3N2		2000	Australia	A/Western_Australia/9/2000	agtgatgccccattccttgatcggcttcgccgagatcag agatccctaaggggaagaggcaacactc	-	*
CY016112	Human H3N2 IAVs	Human	H3N2		2000	Australia	A/New_South_Wales/29/2000	agtgatgccccattccttgatcggcttcgccgagatcag agatccctaaggggaagaggcaacactc	-	*
CY016120	Human H3N2 IAVs	Human	H3N2		2000	Australia	A/New_South_Wales/36/2000	agtgatgccccattccttgatcggcttcgccgagatcag agatccctaaggggaagaggcaacactc	-	*
CY020217	Human H3N2 IAVs	Human	H3N2		2000	Australia	A/South_Australia/15/2000	agtgatgccccattccttgatcggcttcgccgagatcag agatccctaaggggaagaggcaacactc	-	*
CY016759	Human H3N2 IAVs	Human	H3N2		2000	Australia	A/South_Australia/68/2000	agtgatgccccattccttgatcggcttcgccgagatcag agatccctaaggggaagaggcaacactc	-	*
CY021953	Human H3N2 IAVs	Human	H3N2		2000	Australia	A/South_Australia/73/2000	agtgatgccccattccttgatcggcttcgccgagatcag agatccctaaggggaagaggcaacactc	-	*
CY016775	Human H3N2 IAVs	Human	H3N2		2000	Australia	A/South_Australia/74/2000	agtgatgccccattccttgatcggcttcgccgagatcag agatccctaaggggaagaggcaacactc	-	*
CY017399	Human H3N2 IAVs	Human	H3N2		2000	Australia	A/South_Australia/77/2000	agtgatgccccattccttgatcggcttcgccgagatcag agatccctaaggggaagaggcaacactc	-	*
CY021785	Human H3N2 IAVs	Human	H3N2		2000	Australia	A/South_Australia/81/2000	agtgatgccccattccttgatcggcttcgccgagatcag agatccctaaggggaagaggcaacactc	-	*
CY022257	Human H3N2 IAVs	Human	H3N2		2000	Australia	A/Western_Australia/4/2000	agtgatgccccattccttgatcggcttcgccgagatcag agatccctaaggggaagaggcaacactc	-	*
CY008480	Human H3N2 IAVs	Human	H3N2		2000	New_Zealand	A/Canterbury/17/2000	agtgatgccccattccttgatcggcttcgccgagatcag agatccctaaggggaagaggcaacactc	-	*
CY008488	Human H3N2 IAVs	Human	H3N2		2000	New_Zealand	A/Canterbury/38/2000	agtgatgccccattccttgatcggcttcgccgagatcag agatccctaaggggaagaggcaacactc	-	*
CY008143	Human H3N2 IAVs	Human	H3N2		2000	New_Zealand	A/Canterbury/39/2000	agtgatgccccattccttgatcggcttcgccgagatcag agatccctaaggggaagaggcaacactc	-	*
CY009144	Human H3N2 IAVs	Human	H3N2		2000	New_Zealand	A/Canterbury/64/2000	agtgatgccccattccttgatcggcttcgccgagatcag agatccctaaggggaagaggcaacactc	-	*
CY008768	Human H3N2 IAVs	Human	H3N2		2000	New_Zealand	A/Canterbury/66/2000	agtgatgccccattccttgatcggcttcgccgagatcag agatccctaaggggaagaggcaacactc	-	*
CY009160	Human H3N2 IAVs	Human	H3N2		2000	New_Zealand	A/Canterbury/67/2000	agtgatgccccattccttgatcggcttcgccgagatcag agatccctaaggggaagaggcaacactc	-	*
CY008496	Human H3N2 IAVs	Human	H3N2		2000	New_Zealand	A/Canterbury/73/2000	agtgatgccccattccttgatcggcttcgccgagatcag agatccctaaggggaagaggcaacactc	-	*
CY008808	Human H3N2 IAVs	Human	H3N2		2000	New_Zealand	A/Canterbury/85/2000	agtgatgccccattccttgatcggcttcgccgagatcag agatccctaaggggaagaggcaacactc	-	*

CY009080	Human H3N2 IAVs	Human	H3N2		2000	New_Zealand	A/Canterbury/87/2000	agtgatgccccattccttgatcggcttcgccgagatcag agatccctaaggggaagaggcaacactc	-	*
CY008504	Human H3N2 IAVs	Human	H3N2		2000	New_Zealand	A/Canterbury/88/2000	agtgatgccccattccttgatcggcttcgccgagatcag agatccctaaggggaagaggcaacactc	-	*
CY008824	Human H3N2 IAVs	Human	H3N2		2000	New_Zealand	A/Canterbury/89/2000	agtgatgccccattccttgatcggcttcgccgagatcag agatccctaaggggaagaggcaacactc	-	*
CY008512	Human H3N2 IAVs	Human	H3N2		2000	New_Zealand	A/Canterbury/99/2000	agtgatgccccattccttgatcggcttcgccgagatcag agatccctaaggggaagaggcaacactc	-	*
CY011964	Human H3N2 IAVs	Human	H3N2		2000	New_Zealand	A/Waikato/5/2000	agtgatgccccattccttgatcggcttcgccgagatcag agatccctaaggggaagaggcaacactc	-	*
CY011024	Human H3N2 IAVs	Human	H3N2		2000	New_Zealand	A/Wellington/9/2000	agtgatgccccattccttgatcggcttcgccgagatcag agatccctaaggggaagaggcaacactc	-	*
CY016088	Human H3N2 IAVs	Human	H3N2		2000	Australia	A/New_South_Wales/25/2000	agtgatgccccattccttgatcggcttcgccgagatcag agatccctaaggggaagaggcaacactc	-	*
CY016104	Human H3N2 IAVs	Human	H3N2		2000	Australia	A/New_South_Wales/28/2000	agtgatgccccattccttgatcggcttcgccgagatcag agatccctaaggggaagaggcaacactc	-	*
CY016495	Human H3N2 IAVs	Human	H3N2		2000	Australia	A/Western_Australia/10/2000	agtgatgccccattccttgatcggcttcgccgagatcag agatccctaaggggaagaggcaacactc	-	*
CY025046	Human H3N2 IAVs	Human	H3N2		2000	New_Zealand	A/Auckland/598/2000	agtgatgccccattccttgatcggcttcgccgagatcag agatccctaaggggaagaggcaacactc	-	*
CY017471	Human H3N2 IAVs	Human	H3N2		2000	Australia	A/Queensland/8/2000	agtgatgccccattccttgatcggcttcgccgagatcag agatccctaaggggaagaggcaacactc	-	*
CY021777	Human H3N2 IAVs	Human	H3N2		2000	Australia	A/South_Australia/72/2000	agtgatgccccattccttgatcggcttcgccgagatcag agatccctaaggggaagaggcaacactc	-	*
CY013907	Human H3N2 IAVs	Human	H3N2		2000	New_Zealand	A/Wellington/30/2000	agtgatgccccattccttgatcggcttcgccgagatcag agatccctaaggggaagaggcaacactc	-	*
CY015664	Human H3N2 IAVs	Human	H3N2		2000	Australia	A/Western_Australia/8/2000	agtgatgccccattccttgatcggcttcgccgagatcag agatccctaaggggaagaggcaacactc	-	*
CY008848	Human H3N2 IAVs	Human	H3N2		2000	New_Zealand	A/Canterbury/101/2000	agtgatgccccattccttgatcggcttcgccgagatcag agatccctaaggggaagaggcaacactc	-	*
CY015648	Human H3N2 IAVs	Human	H3N2		2000	Australia	A/Western_Australia/2/2000	agtgatgccccattccttgatcggcttcgccgagatcag agatccctaaggggaagaggcaacactc	-	*
CY013899	Human H3N2 IAVs	Human	H3N2		2000	New_Zealand	A/Wellington/26/2000	agtgatgccccattccttgatcggcttcgccgagatcag agatccctaaggggaagaggcaacactc	-	*
CY017327	Human H3N2 IAVs	Human	H3N2		2000	Australia	A/Western_Australia/1/2000	agtgatgccccattccttgatcggcttcgccgagatcag agatccctaaggggaagaggcaacactc	-	*
CY016407	Human H3N2 IAVs	Human	H3N2		2000	New_Zealand	A/Canterbury/75/2000	agtgatgccccattccttgatcggcttcgccgagatcag agatccctaaggggaagaggcaacactc	-	*
CY017263	Human H3N2 IAVs	Human	H3N2		2000	Australia	A/Queensland/1/2000	agtgatgccccattccttgatcggcttcgccgagatcag agatccctaaggggaagaggcaacactc	-	*
CY017175	Human H3N2 IAVs	Human	H3N2		2000	Australia	A/South_Australia/76/2000	agtgatgccccattccttgatcggcttcgccgagatcag agatccctaaggggaagaggcaacactc	-	*
CY017809	Human H3N2 IAVs	Human	H3N2		2000	Australia	A/South_Australia/2/2000	agtgatgccccattccttgatcggcttcgccgagatcag agatccctaaggggaagaggcaacactc	-	*
CY012628	Human H3N2 IAVs	Human	H3N2		2000	New_Zealand	A/Waikato/4/2000	agtgatgccccattccttgatcggcttcgccgagatcag agatccctaaggggaagaggcaacactc	-	*
CY017479	Human H3N2 IAVs	Human	H3N2		2000	Australia	A/Queensland/9/2000	agtgatgccccattccttgatcggcttcgccgagatcag agatccctaaggggaagaggcaacactc	-	*
CY017343	Human H3N2 IAVs	Human	H3N2		2000	Australia	A/Western_Australia/7/2000	agtgatgccccattccttgatcggcttcgccgagatcag agatccctaaggggaagaggcaacactc	-	*

CY006663	Human H3N2 IAVs	Human	H3N2		2000	USA	A/New_York/435/2000	agtgatgccccattccttgatcggcttcgccgagatcag agatccctaaggggaagaggcaacactc	-	*
CY009096	Human H3N2 IAVs	Human	H3N2		2000	New_Zealand	A/Canterbury/92/2000	agtgatgccccattccttgatcggcttcgccgagatcag agatccctaaggggaagaggcagcactc	-	-
CY012436	Human H3N2 IAVs	Human	H3N2		2000	New_Zealand	A/Canterbury/97/2000	agtgatgccccattccttgatcggcttcgccgagatcag agatccctaaggggaagaggcagcactc	-	-
CY117780	Human H3N2 IAVs	Human	H3N2		2000	Malaysia	A/Malaysia/14590/2000	agtgatgccccattccttgatcggcttcgccgagatcag aggtccctaaggggaagaggcaacactc	-	-
AY363583	Human H3N2 IAVs	Swine	H3N2		2000	Hong_Kong	A/Swine/Hong_Kong/312/00	agtgatgccccattccttgatcggcttcgccgagatcag aggtccctaaggggaagaggcaacactc	-	-
CY019993	Human H3N2 IAVs	Human	H3N2		2000	New_Zealand	A/Wellington/21/2000	agtgatgccccattccttgatcggcttcgccgagatcag aggtccctaaggggaagaggcaacactc	-	-
AY363582	Human H3N2 IAVs	Swine	H3N2		2000	Hong_Kong	A/Swine/Hong_Kong/7220/00	agtgatgccccattccttgatcggcttcgccgagatcag aggtccctaaggggaagaggcaacactc	-	-
CY009152	Human H3N2 IAVs	Human	H3N2		2000	New_Zealand	A/Canterbury/58/2000	agtgatgccccattccttgatcggcttcgccgagatcag aggtccctaaggggaagaggcaacactc	-	-
CY009168	Human H3N2 IAVs	Human	H3N2		2000	New_Zealand	A/Canterbury/61/2000	agtgatgccccattccttgatcggcttcgccgagatcag aggtccctaaggggaagaggcaacactc	-	-
CY011068	Human H3N2 IAVs	Human	H3N2		2000	New_Zealand	A/Canterbury/62/2000	agtgatgccccattccttgatcggcttcgccgagatcag aggtccctaaggggaagaggcaacactc	-	-
CY008816	Human H3N2 IAVs	Human	H3N2		2000	New_Zealand	A/Hutt/82/2000	agtgatgccccattccttgatcggcttcgccgagatcag aggtccctaaggggaagaggcaacactc	-	-
CY013393	Human H3N2 IAVs	Human	H3N2		2000	New_Zealand	A/Waikato/16/2000	agtgatgccccattccttgatcggcttcgccgagatcag aggtccctaaggggaagaggcaacactc	-	-
CY013060	Human H3N2 IAVs	Human	H3N2		2000	New_Zealand	A/Waikato/9/2000	agtgatgccccattccttgatcggcttcgccgagatcag aggtccctaaggggaagaggcaacactc	-	-
CY025022	Human H3N2 IAVs	Human	H3N2		2000	New_Zealand	A/Auckland/583/2000	agtgatgccccattccttgatcggcttcgccgagatcag aggtccctaaggggaagaggcaacactc	-	-
CY019985	Human H3N2 IAVs	Human	H3N2		2000	New_Zealand	A/Canterbury/50/2000	agtgatgccccattccttgatcggcttcgccgagatcag aggtccctaaggggaagaggcaacactc	-	-
CY000985	Human H3N2 IAVs	Human	H3N2		2000	USA	A/New_York/160/2000	agtgatgccccattccttgatcggcttcgccgagatcag aggtccctaaggggaagaggcaacactc	-	-
CY003244	Human H3N2 IAVs	Human	H3N2		2000	USA	A/New_York/431/2000	agtgatgccccattccttgatcggcttcgccgagatcag aggtccctaaggggaagaggcaacactc	-	-
CY003829	Human H3N2 IAVs	Human	H3N2		2000	USA	A/New_York/440/2000	agtgatgccccattccttgatcggcttcgccgagatcag aggtccctaaggggaagaggcaacactc	-	-
CY016719	Human H3N2 IAVs	Human	H3N2		2000	Australia	A/South_Australia/62/2000	agtgatgccccattccttgatcggcttcgccgagatcag aggtccctaaggggaagaggcaacactc	-	-
CY012636	Human H3N2 IAVs	Human	H3N2		2000	New_Zealand	A/Waikato/7/2000	agtgatgccccattccttgatcggcttcgccgagatcag aggtccctaaggggaagaggcaacactc	-	-
CY016767	Human H3N2 IAVs	Human	H3N2		2000	Australia	A/South_Australia/71/2000	agtgatgccccattccttgatcggcttcgccgagatcag aggtccctaaggggaagaggcaacactc	-	-
CY020497	Human H3N2 IAVs	Human	H3N2		2000	Australia	A/Queensland/6/2000	agtgatgccccattccttgatcggcttcgccgagatcag aggtccctaaggggaagaggcaacactc	-	-
CY016208	Human H3N2 IAVs	Human	H3N2		2000	New_Zealand	A/Waikato/8/2000	agtgatgccccattccttgatcggcttcgccgagatcag aggtccctaaggggaagaggcaacactc	-	-
CY000701	Human H3N2 IAVs	Human	H3N2		2000	USA	A/New_York/174/2000	agtgatgccccattccttgatcggcttcgccgagatcag aggtccctaaggggaagaggcaacactc	-	-
CY000853	Human H3N2 IAVs	Human	H3N2		2000	USA	A/New_York/178/2000	agtgatgccccattccttgatcggcttcgccgagatcag aggtccctaaggggaagaggcaacactc	-	-

CY017447	Human H3N2 IAVs	Human	H3N2		2000	Australia	A/Queensland/2/2000	agtgatgccccattccttgatcggcttcgccgagatcag<br>aggtccctaaggggaagaggcaacactc	-	-
CY001401	Human H3N2 IAVs	Human	H3N2		2000	USA	A/New_York/156/2000	agtgatgccccattccttgatcggcttcgccgagatcag<br>aggtccctaaggggaagaggcaacactc	-	-
CY000837	Human H3N2 IAVs	Human	H3N2		2000	USA	A/New_York/158/2000	agtgatgccccattccttgatcggcttcgccgagatcag<br>aggtccctaaggggaagaggcaacactc	-	-
CY000845	Human H3N2 IAVs	Human	H3N2		2000	USA	A/New_York/168/2000	agtgatgccccattccttgatcggcttcgccgagatcag<br>aggtccctaaggggaagaggcaacactc	-	-
CY000661	Human H3N2 IAVs	Human	H3N2		2000	USA	A/New_York/169/2000	agtgatgccccattccttgatcggcttcgccgagatcag<br>aggtccctaaggggaagaggcaacactc	-	-
CY008832	Human H3N2 IAVs	Human	H3N2		2000	New_Zealand	A/Canterbury/93/2000	agtgatgccccattccttgatcggcttcgccgagatcag<br>aggtccctaaggggaagaggcaacactc	-	-
CY009392	Human H3N2 IAVs	Human	H3N2		2000	New_Zealand	A/Canterbury/94/2000	agtgatgccccattccttgatcggcttcgccgagatcag<br>aggtccctaaggggaagaggcaacactc	-	-
CY008856	Human H3N2 IAVs	Human	H3N2		2000	New_Zealand	A/Canterbury/98/2000	agtgatgccccattccttgatcggcttcgccgagatcag<br>aggtccctaaggggaagaggcaacactc	-	-
CY003268	Human H3N2 IAVs	Human	H3N2		2000	USA	A/New_York/436/2000	agtgatgccccattccttgatcggcttcgccgagatcag<br>aggtccctaaggggaagaggcaacactc	-	-
CY000717	Human H3N2 IAVs	Human	H3N2		2000	USA	A/New_York/176/2000	agtgatgccccattccttgatcggcttcgccgagatcag<br>aggtccctaaggggaagaggcaacactc	-	-
CY009128	Human H3N2 IAVs	Human	H3N2		2000	New_Zealand	A/Nelson_Marlborough/1/2000	agtgatgccccattccttgatcggcttcgccgagatcag<br>aggtccctaaggggaagaggcaacactc	-	-
CY011596	Human H3N2 IAVs	Human	H3N2		2000	New_Zealand	A/Waikato/1/2000	agtgatgccccattccttgatcggcttcgccgagatcag<br>aggtccctaaggggaagaggcaacgctc	-	-
CY023030	Human H3N2 IAVs	Human	H3N2		2000	New_Zealand	A/Auckland/596/2000	agtgatgccccattcctttattggcttcgccgagatcag<br>agatccctaaggggaagaggcaacactc	+	+
CY001140	Human H3N2 IAVs	Human	H3N2		2000	USA	A/New_York/152/2000	agtgatgcccccttccttgatcggctccgccgagatca<br>gaggtccctaaggggaagaggcaacactc	-	-
CY008864	Human H3N2 IAVs	Human	H3N2		2000	New_Zealand	A/Canterbury/90/2000	agtgatgcccccttccttgatcggcttcgccgagatcag<br>agatccctaaggggaagaggcaacactc	-	-
CY003284	Human H3N2 IAVs	Human	H3N2		2000	USA	A/New_York/438/2000	agtgatgcccccttccttgatcggcttcgccgagatcag<br>aggtccctaaggggaagaggcaacacta	-	-
CY023046	Human H3N2 IAVs	Human	H3N2		2000	New_Zealand	A/Auckland/600/2000	agtgatgcccccttccttgatcggcttcgccgagatcag<br>aggtccctaaggggaagaggcaacactc	-	-
CY013379	Human H3N2 IAVs	Human	H3N2		2000	New_Zealand	A/Dunedin/3/2000	agtgatgcccccttccttgatcggcttcgccgagatcag<br>aggtccctaaggggaagaggcaacactc	-	-
CY003452	Human H3N2 IAVs	Human	H3N2		2000	USA	A/New_York/430/2000	agtgatgcccccttccttgatcggcttcgccgagatcag<br>aggtccctaaggggaagaggcaacactc	-	-
CY003276	Human H3N2 IAVs	Human	H3N2		2000	USA	A/New_York/437/2000	agtgatgcccccttccttgatcggcttcgccgagatcag<br>aggtccctaaggggaagaggcaacactc	-	-
CY012620	Human H3N2 IAVs	Human	H3N2		2000	New_Zealand	A/Dunedin/1/2000	agtgatgcccccttccttgatcggcttcgccgagatcag<br>aggtccctaaggggaagaggcaacactc	-	-
CY009120	Human H3N2 IAVs	Human	H3N2		2000	New_Zealand	A/Canterbury/2/2000	agtgatgcccccttccttgatcggcttcgccgagatcag<br>aggtccctaaggggaagaggcaacactc	-	-
CY001524	Human H3N2 IAVs	Human	H3N2		2000	USA	A/New_York/142/2000	agtgatgcccccttccttgatcggcttcgccgagatcag<br>aggtccctaaggggaagaggcaacactc	-	-
CY000821	Human H3N2 IAVs	Human	H3N2		2000	USA	A/New_York/148/2000	agtgatgcccccttccttgatcggcttcgccgagatcag<br>aggtccctaaggggaagaggcaacactc	-	-
CY000613	Human H3N2 IAVs	Human	H3N2		2000	USA	A/New_York/154/2000	agtgatgcccccttccttgatcggcttcgccgagatcag<br>aggtccctaaggggaagaggcaacactc	-	-

CY001249	Human H3N2 IAVs	Human	H3N2		2000	USA	A/New_York/162/2000	agtgatgcccccttccttgatcggcttcgccgagatcag aggtccctaaggggaagaggcaacactc	-	-
CY000693	Human H3N2 IAVs	Human	H3N2		2000	USA	A/New_York/173/2000	agtgatgcccccttccttgatcggcttcgccgagatcag aggtccctaaggggaagaggcaacactc	-	-
CY000709	Human H3N2 IAVs	Human	H3N2		2000	USA	A/New_York/175/2000	agtgatgcccccttccttgatcggcttcgccgagatcag aggtccctaaggggaagaggcaacactc	-	-
CY000741	Human H3N2 IAVs	Human	H3N2		2000	USA	A/New_York/180/2000	agtgatgcccccttccttgatcggcttcgccgagatcag aggtccctaaggggaagaggcaacactc	-	-
CY013052	Human H3N2 IAVs	Human	H3N2		2000	New_Zealand	A/Waikato/6/2000	agtgatgcccccttccttgatcggcttcgccgagatcag aggtccctaaggggaagaggcaacactc	-	-
CY090913	Human H3N2 IAVs	Human	H3N2		2000	USA	A/Missouri/NHRC0001/2000	agtgatgcccccttccttgatcggcttcgccgagatcag aggtccctaaggggaagaggcaacactc	-	-
CY001001	Human H3N2 IAVs	Human	H3N2		2000	USA	A/New_York/165/2000	agtgatgcccccttccttgatcggcttcgccgagatcag aggtccctaaggggaagaggcaacactc	-	-
JF710773	Human H3N2 IAVs	Human	H3N2		2000	United_Kingdom	A/England/24/2000	agtgatgcccccttccttgatcggcttcgccgagatcag aggtccctaaggggaagaggcaacactc	-	-
CY000469	Human H3N2 IAVs	Human	H3N2		2000	USA	A/New_York/150/2000	agtgatgcccccttccttgatcggcttcgccgagatcag aggtccctaaggggaagaggcaacactc	-	-
CY003260	Human H3N2 IAVs	Human	H3N2		2000	USA	A/New_York/434/2000	agtgatgcccccttccttgatcggcttcgccgagatcag aggtccctaaggggaagaggcaacactc	-	-
CY020313	Human H3N2 IAVs	Human	H3N2		2000	Australia	A/Queensland/10/2000	agtgatgcccccttccttgatcggcttcgccgagatcag aggtccctaaggggaagaggcaacactc	-	-
CY017455	Human H3N2 IAVs	Human	H3N2		2000	Australia	A/Queensland/3/2000	agtgatgcccccttccttgatcggcttcgccgagatcag aggtccctaaggggaagaggcaacactc	-	-
CY017271	Human H3N2 IAVs	Human	H3N2		2000	Australia	A/Queensland/4/2000	agtgatgcccccttccttgatcggcttcgccgagatcag aggtccctaaggggaagaggcaacactc	-	-
CY017889	Human H3N2 IAVs	Human	H3N2		2000	Australia	A/Queensland/7/2000	agtgatgcccccttccttgatcggcttcgccgagatcag aggtccctaaggggaagaggcaacactc	-	-
CY003821	Human H3N2 IAVs	Human	H3N2		2000	USA	A/New_York/432/2000	agtgatgcccccttccttgatcggcttcgccgagatcag aggtccctaaggggaagaggcaacactc	-	-
CY013409	Human H3N2 IAVs	Human	H3N2		2000	New_Zealand	A/Wellington/28/2000	agtgatgcccccttccttgatcggcttcgccgagatcag aggtccctaaggggaagaggcaacactc	-	-
CY009104	Human H3N2 IAVs	Human	H3N2		2000	New_Zealand	A/Canterbury/3/2000	agtgatgcccccttccttgatcggcttcgccgagatcag aggtccctaaggggaagaggcaacactc	-	-
CY009136	Human H3N2 IAVs	Human	H3N2		2000	New_Zealand	A/Canterbury/55/2000	agtgatgcccccttccttgatcggcttcgccgagatcag aggtccctaaggggaagaggcaacactc	-	-
CY008784	Human H3N2 IAVs	Human	H3N2		2000	New_Zealand	A/Canterbury/80/2000	agtgatgcccccttccttgatcggcttcgccgagatcag aggtccctaaggggaagaggcaacactc	-	-
CY008792	Human H3N2 IAVs	Human	H3N2		2000	New_Zealand	A/Canterbury/81/2000	agtgatgcccccttccttgatcggcttcgccgagatcag aggtccctaaggggaagaggcaacactc	-	-
CY008800	Human H3N2 IAVs	Human	H3N2		2000	New_Zealand	A/Canterbury/84/2000	agtgatgcccccttccttgatcggcttcgccgagatcag aggtccctaaggggaagaggcaacactc	-	-
CY003252	Human H3N2 IAVs	Human	H3N2		2000	USA	A/New_York/433/2000	agtgatgcccccttccttgatcggcttcgccgagatcag aggtccctaaggggaagaggcaacactc	-	-
CY003460	Human H3N2 IAVs	Human	H3N2		2000	USA	A/New_York/439/2000	agtgatgcccccttccttgatcggcttcgccgagatcag aggtccctaaggggaagaggcaacactc	-	-
CY017463	Human H3N2 IAVs	Human	H3N2		2000	Australia	A/Queensland/5/2000	agtgatgcccccttccttgatcggcttcgccgagatcag aggtccctaaggggaagaggcaacactc	-	-
CY001369	Human H3N2 IAVs	Human	H3N2		2000	USA	A/New_York/187/2000	agtgatgcccccttccttgatcggcttcgccgagatcag aggtccctaaggggaagaggcaacactc	-	-

CY000669	Human H3N2 IAVs	Human	H3N2		2000	USA	A/New_York/170/2000	agtgatgccccctttcttgatcggcttcgccgagatcag<br>aggtccctaaggggaagaggcaacactc	-	-
CY121412	Human H3N2 IAVs	Human	H3N2		2000	Russia	A/Ulan_Ude/01/2000	ggtgatgccccttccttgatcggcttcgccgagatcag<br>aggtccctaaggggaagaggcaacactc	-	-
CY013076	Human H3N2 IAVs	Human	H3N2		2001	New_Zealand	A/Waikato/5/2001	agtgatgccccattccttgatcggcttagccgagatca<br>gaggtccctaaggggaagaggcaacactc	+	+
CY011972	Human H3N2 IAVs	Human	H3N2		2001	New_Zealand	A/Waikato/1/2001	agtgatgccccattccttgatcggcttcgccgagatcag<br>agatccctaaggggaagaggcaacactc	-	*
CY009432	Human H3N2 IAVs	Human	H3N2		2001	New_Zealand	A/West_Coast/55/2001	agtgatgccccattccttgatcggcttcgccgagatcag<br>agatccctaaggggaagaggcaacactc	-	*
CY009400	Human H3N2 IAVs	Human	H3N2		2001	New_Zealand	A/Canterbury/10/2001	agtgatgccccattccttgatcggcttcgccgagatcag<br>agatccctaaggggaagaggcaacactc	-	*
CY009416	Human H3N2 IAVs	Human	H3N2		2001	New_Zealand	A/Canterbury/37/2001	agtgatgccccattccttgatcggcttcgccgagatcag<br>agatccctaaggggaagaggcaacactc	-	*
CY009576	Human H3N2 IAVs	Human	H3N2		2001	New_Zealand	A/Canterbury/43/2001	agtgatgccccattccttgatcggcttcgccgagatcag<br>agatccctaaggggaagaggcaacactc	-	*
CY009568	Human H3N2 IAVs	Human	H3N2		2001	New_Zealand	A/West_Coast/28/2001	agtgatgccccattccttgatcggcttcgccgagatcag<br>agatccctaaggggaagaggcaacactc	-	*
CY009408	Human H3N2 IAVs	Human	H3N2		2001	New_Zealand	A/West_Coast/32/2001	agtgatgccccattccttgatcggcttcgccgagatcag<br>agatccctaaggggaagaggcaacactc	-	*
CY009424	Human H3N2 IAVs	Human	H3N2		2001	New_Zealand	A/Canterbury/44/2001	agtgatgccccattccttgatcggcttcgccgagatcag<br>agatccctaaggggaagaggcaacactc	-	*
CY114337	Human H3N2 IAVs	Human	H3N2		2001	Netherlands	A/Netherlands/126/2001	agtgatgccccattccttgatcggcttcgccgagatcag<br>agatccctaaggggaagaggcaacactc	-	*
CY009952	Human H3N2 IAVs	Human	H3N2		2001	New_Zealand	A/Canterbury/06/2001	agtgatgccccattccttgatcggcttcgccgagatcag<br>aggtccctaaggggaagaggcaacactc	-	-
CY009560	Human H3N2 IAVs	Human	H3N2		2001	New_Zealand	A/Canterbury/07/2001	agtgatgccccattccttgatcggcttcgccgagatcag<br>aggtccctaaggggaagaggcaacactc	-	-
CY010552	Human H3N2 IAVs	Human	H3N2		2001	New_Zealand	A/Canterbury/36/2001	agtgatgccccattccttgatcggcttcgccgagatcag<br>aggtccctaaggggaagaggcaacactc	-	-
CY009584	Human H3N2 IAVs	Human	H3N2		2001	New_Zealand	A/Canterbury/50/2001	agtgatgccccattccttgatcggcttcgccgagatcag<br>aggtccctaaggggaagaggcaacactc	-	-
CY011620	Human H3N2 IAVs	Human	H3N2		2001	New_Zealand	A/Wellington/22/2001	agtgatgccccattccttgatcggcttcgccgagatcag<br>aggtccctaaggggaagaggcaacactc	-	-
CY009592	Human H3N2 IAVs	Human	H3N2		2001	New_Zealand	A/Canterbury/140/2001	agtgatgccccattccttgatcggcttcgccgagatcag<br>aggtccctaaggggaagaggcaacactc	-	-
CY009856	Human H3N2 IAVs	Human	H3N2		2001	New_Zealand	A/Canterbury/146/2001	agtgatgccccattccttgatcggcttcgccgagatcag<br>aggtccctaaggggaagaggcaacactc	-	-
CY009440	Human H3N2 IAVs	Human	H3N2		2001	New_Zealand	A/Canterbury/149/2001	agtgatgccccattccttgatcggcttcgccgagatcag<br>aggtccctaaggggaagaggcaacactc	-	-
CY118630	Human H3N2 IAVs	Human	H3N2		2001	Malaysia	A/Malaysia/17998/2001	agtgatgccccattccttgatcggcttcgccgagatcag<br>aggtccctaaggggaagaggcaacactc	-	-
CY118646	Human H3N2 IAVs	Human	H3N2		2001	Malaysia	A/Malaysia/18121/2001	agtgatgccccattccttgatcggcttcgccgagatcag<br>aggtccctaaggggaagaggcaacactc	-	-
CY118654	Human H3N2 IAVs	Human	H3N2		2001	Malaysia	A/Malaysia/18128/2001	agtgatgccccattccttgatcggcttcgccgagatcag<br>aggtccctaaggggaagaggcaacactc	-	-
CY114329	Human H3N2 IAVs	Human	H3N2		2001	Netherlands	A/Netherlands/124/2001	agtgatgccccattccttgatcggcttcgccgagatcag<br>aggtccctaaggggaagaggcaacactc	-	-
AY363584	Human H3N2 IAVs	Swine	H3N2		2001	Hong_Kong	A/Swine/Hong_Kong/9285/01	agtgatgccccattccttgatcggcttcgccgagatcag<br>aggtccctaaggggaagaggcaacactc	-	-

AY363585	Human H3N2 IAVs	Swine	H3N2		2001	Hong_Kong	A/Swine/Hong_Kong/1311/01	agtgatgccccattccttgatcggcttcgccgagatcag aggtccctaaggggaagaggcaacactc	-	-
AY363586	Human H3N2 IAVs	Swine	H3N2		2001	Hong_Kong	A/Swine/Hong_Kong/9840/01	agtgatgccccattccttgatcggcttcgccgagatcag aggtccctaaggggaagaggcaacactc	-	-
CY105194	Human H3N2 IAVs	Human	H3N2		2001	Viet_Nam	A/HaNoi/1766/2001	agtgatgccccattccttgatcggcttcgccgagatcag aggtccctaaggggaagaggcaacactc	-	-
CY022529	Human H3N2 IAVs	Human	H3N2		2001	New_Zealand	A/Auckland/602/2001	agtgatgccccattccttgatcggcttcgccgagatcag aggtccctaaggggaagaggcaacactc	-	-
CY022185	Human H3N2 IAVs	Human	H3N2		2001	New_Zealand	A/Auckland/607/2001	agtgatgccccattccttgatcggcttcgccgagatcag aggtccctaaggggaagaggcaacactc	-	-
CY023062	Human H3N2 IAVs	Human	H3N2		2001	New_Zealand	A/Auckland/603/2001	agtgatgccccattccttgatcggcttcgccgagatcag aggtccctaaggggaagaggcaacactc	-	-
CY112929	Human H3N2 IAVs	Human	H3N2		2001	Netherlands	A/Netherlands/118/2001	agtgatgccccattccttgatcggcttcgccgagatcag aggtccctaaggggaagaggcaacactc	-	-
CY002820	Human H3N2 IAVs	Human	H3N2		2001	USA	A/New_York/301/2001	agtgatgccccattccttgatcggcttcgccgagatcag aggtccctaaggggaagaggcaacactc	-	-
CY017511	Human H3N2 IAVs	Human	H3N2		2001	Australia	A/Queensland/16/2001	agtgatgccccattccttgatcggcttcgccgagatcag aggtccctaaggggaagaggcaacactc	-	-
CY015688	Human H3N2 IAVs	Human	H3N2		2001	Australia	A/Western_Australia/14/2001	agtgatgccccattccttgatcggcttcgccgagatcag aggtccctaaggggaagaggcaacactc	-	-
CY015696	Human H3N2 IAVs	Human	H3N2		2001	Australia	A/Western_Australia/16/2001	agtgatgccccattccttgatcggcttcgccgagatcag aggtccctaaggggaagaggcaacactc	-	-
CY012324	Human H3N2 IAVs	Human	H3N2		2001	New_Zealand	A/Waikato/105/2001	agtgatgccccattccttgatcggcttcgccgagatcag aggtccctaaggggaagaggcaacactc	-	-
CY015704	Human H3N2 IAVs	Human	H3N2		2001	Australia	A/Western_Australia/17/2001	agtgatgccccattccttgatcggcttcgccgagatcag aggtccctaaggggaagaggcaacactc	-	-
CY013220	Human H3N2 IAVs	Human	H3N2		2001	Australia	A/Western_Australia/15/2001	agtgatgccccattccttgatcggcttcgccgagatcag aggtccctaaggggaagaggcaacactc	-	-
CY023070	Human H3N2 IAVs	Human	H3N2		2001	New_Zealand	A/Auckland/604/2001	agtgatgccccattccttgatcggcttcgccgagatcag aggtccctaaggggaagaggcaacactc	-	-
CY000389	Human H3N2 IAVs	Human	H3N2		2001	USA	A/New_York/85/2001	agtgatgccccattccttgatcggcttcgccgagatcag aggtccctaaggggaagaggcaacactc	-	-
CY012316	Human H3N2 IAVs	Human	H3N2		2001	New_Zealand	A/Wellington/34/2001	agtgatgccccattccttgatcggcttcgccgagatcag aggtccctaaggggaagaggcaacactc	-	-
CY003092	Human H3N2 IAVs	Human	H3N2		2001	USA	A/New_York/402/2001	agtgatgccccattccttgatcggcttcgccgagatcag aggtccctaaggggaagaggcaacactc	-	-
CY017487	Human H3N2 IAVs	Human	H3N2		2001	Australia	A/Queensland/11/2001	agtgatgccccattccttgatcggcttcgccgagatcag aggtccctaaggggaagaggcaacactc	-	-
CY018961	Human H3N2 IAVs	Human	H3N2		2001	Australia	A/Queensland/14/2001	agtgatgccccattccttgatcggcttcgccgagatcag aggtccctaaggggaagaggcaacactc	-	-
CY017503	Human H3N2 IAVs	Human	H3N2		2001	Australia	A/Queensland/15/2001	agtgatgccccattccttgatcggcttcgccgagatcag aggtccctaaggggaagaggcaacactc	-	-
CY017631	Human H3N2 IAVs	Human	H3N2		2001	Australia	A/Queensland/17/2001	agtgatgccccattccttgatcggcttcgccgagatcag aggtccctaaggggaagaggcaacactc	-	-
CY017519	Human H3N2 IAVs	Human	H3N2		2001	Australia	A/Queensland/18/2001	agtgatgccccattccttgatcggcttcgccgagatcag aggtccctaaggggaagaggcaacactc	-	-
CY017527	Human H3N2 IAVs	Human	H3N2		2001	Australia	A/Queensland/20/2001	agtgatgccccattccttgatcggcttcgccgagatcag aggtccctaaggggaagaggcaacactc	-	-
CY017897	Human H3N2 IAVs	Human	H3N2		2001	Australia	A/Queensland/19/2001	agtgatgccccattccttgatcggcttcgccgagatcag aggtccctaaggggaagaggcaacactc	-	-

CY017495	Human H3N2 IAVs	Human	H3N2		2001	Australia	A/Queensland/12/2001	agtgatgccccattccttgatcggcttcgccgagatcag<br>aggtccctaaggggaagaggcaacactc	-	-
CY000325	Human H3N2 IAVs	Human	H3N2		2001	USA	A/New_York/131/2001	agtgatgccccattccttgatcggcttcgccgagatcag<br>aggtccctaaggggaagaggcaacactc	-	-
CY001724	Human H3N2 IAVs	Human	H3N2		2001	USA	A/New_York/273/2001	agtgatgccccattccttgatcggcttcgccgagatcag<br>aggtccctaaggggaagaggcaacactc	-	-
CY000485	Human H3N2 IAVs	Human	H3N2		2001	USA	A/New_York/71/2001	agtgatgccccattccttgatcggcttcgccgagatcag<br>aggtccctaaggggaagaggcaacactc	-	-
CY000189	Human H3N2 IAVs	Human	H3N2		2001	USA	A/New_York/83/2001	agtgatgccccattccttgatcggcttcgccgagatcag<br>aggtccctaaggggaagaggcaacactc	-	-
CY001172	Human H3N2 IAVs	Human	H3N2		2001	USA	A/New_York/94/2001	agtgatgccccattccttgatcggcttcgccgagatcag<br>aggtccctaaggggaagaggcaacactc	-	-
CY000580	Human H3N2 IAVs	Human	H3N2		2001	USA	A/New_York/127/2001	agtgatgccccattccttgatcggcttcgccgagatcag<br>aggtccctaaggggaagaggcaacactc	-	-
CY002332	Human H3N2 IAVs	Human	H3N2		2001	USA	A/New_York/77/2001	agtgatgccccattccttgatcggcttcgccgagatcag<br>aggtccctaaggggaagaggcaacactc	-	-
CY000277	Human H3N2 IAVs	Human	H3N2		2001	USA	A/New_York/82/2001	agtgatgccccattccttgatcggcttcgccgagatcag<br>aggtccctaaggggaagaggcaacactc	-	-
CY003084	Human H3N2 IAVs	Human	H3N2		2001	USA	A/New_York/401/2001	agtgatgccccattccttgatcggcttcgccgagatcag<br>aggtccctaaggggaagaggcaacactc	-	-
CY000573	Human H3N2 IAVs	Human	H3N2		2001	USA	A/New_York/80/2001	agtgatgccccattccttgatcggcttcgccgagatcag<br>aggtccctaaggggaagaggcaacactc	-	-
CY000205	Human H3N2 IAVs	Human	H3N2		2001	USA	A/New_York/84/2001	agtgatgccccattccttgatcggcttcgccgagatcag<br>aggtccctaaggggaagaggcaacactc	-	-
CY015680	Human H3N2 IAVs	Human	H3N2		2001	Australia	A/Western_Australia/13/2001	agtgatgccccattccttgatcggcttcgccgagatcag<br>aggtccctaaggggaagaggcaacactc	-	-
CY011652	Human H3N2 IAVs	Human	H3N2		2002	New_Zealand	A/Waikato/21/2002	aatgatgccccattccttgatcggcttcgccgagatca<br>gaggtccctaaggggaagaggcaacactc	-	-
CY001321	Human H3N2 IAVs	Human	H3N2		2002	USA	A/New_York/102/2002	aatgatgccccattccttgatcggcttcgccgagatca<br>gaggtccctaaggggaagaggcaacactc	-	-
CY002132	Human H3N2 IAVs	Human	H3N2		2002	USA	A/New_York/275/2002	aatgatgccccattccttgatcggcttcgccgagatca<br>gaggtccctaaggggaagaggcaacactc	-	-
CY013809	Human H3N2 IAVs	Human	H3N2		2002	USA	A/New_York/C4/2002	aatgatgccccattccttgatcggcttcgccgagatca<br>gaggtccctaaggggaagaggcaacactc	-	-
AY363587	Human H3N2 IAVs	Swine	H3N2		2002	Hong_Kong	A/Swine/Hong_Kong/74/02	agcgatgccccattccttgatcggcttcgccgagatca<br>gaggtccctaaggggaagaggcaacactc	-	-
JF710729	Human H3N2 IAVs	Human	H3N2		2002	United_Kingdom	A/England/427/2002	agcgatgctccattccttgatcggcttcgccgagatcag<br>aggtccctaaggggaagaggcaacactc	-	-
CY017095	Human H3N2 IAVs	Human	H3N2		2002	Australia	A/Western_Australia/32/2002	agtgatgccccattccttgatcggctccgccgagatca<br>gaggtccctaaggggaagaggcaacactc	-	-
CY016224	Human H3N2 IAVs	Human	H3N2		2002	Australia	A/Western_Australia/33/2002	agtgatgccccattccttgatcggctccgccgagatca<br>gaggtccctaaggggaagaggcaacactc	-	-
CY011988	Human H3N2 IAVs	Human	H3N2		2002	New_Zealand	A/Wellington/6/2002	agtgatgccccattccttgatcggcttcgccgagatca<br>aaggtccctaaggggaagaggcaacactc	+	+
CY012332	Human H3N2 IAVs	Human	H3N2		2002	New_Zealand	A/Wellington/7/2002	agtgatgccccattccttgatcggcttcgccgagatca<br>aaggtccctaaggggaagaggcaacactc	+	+
CY000341	Human H3N2 IAVs	Human	H3N2		2002	USA	A/New_York/135/2002	agtgatgccccattccttgatcggcttcgccgagatca<br>aaggtccctaaggggaagaggcaacactc	+	+
AY363588	Human H3N2 IAVs	Swine	H3N2		2002	Hong_Kong	A/Swine/Hong_Kong/411/02	agtgatgccccattccttgatcggcttcgccgagatcag<br>aagtccctaaggggaagaggcaacactc	+	+

CY000333	Human H3N2 IAVs	Human	H3N2		2002	USA	A/New_York/133/2002	agtgatgccccattccttgatcggcttcgccgagatcag<br>agatccctaaggggaagaggcaacactc	-	*
CY000213	Human H3N2 IAVs	Human	H3N2		2002	USA	A/New_York/91/2002	agtgatgccccattccttgatcggcttcgccgagatcag<br>agatccctaaggggaagaggcaacactc	-	*
CY011668	Human H3N2 IAVs	Human	H3N2		2002	New_Zealand	A/Waikato/58/2002	agtgatgccccattccttgatcggcttcgccgagatcag<br>aggtccctaagaggaagaggcaacactc	-	-
JF710771	Human H3N2 IAVs	Human	H3N2		2002	United_Kingdom	A/England/125/2002	agtgatgccccattccttgatcggcttcgccgagatcag<br>aggtccctaaggggaagaggcaacactc	-	-
CY125176	Human H3N2 IAVs	Human	mixed		2002	USA	A/New_York/26/2002	agtgatgccccattccttgatcggcttcgccgagatcag<br>aggtccctaaggggaagaggcaacactc	-	-
CY001684	Human H3N2 IAVs	Human	H1N2		2002	USA	A/New_York/78/2002	agtgatgccccattccttgatcggcttcgccgagatcag<br>aggtccctaaggggaagaggcaacactc	-	-
CY003773	Human H3N2 IAVs	Human	H1N2		2002	USA	A/New_York/417/2002	agtgatgccccattccttgatcggcttcgccgagatcag<br>aggtccctaaggggaagaggcaacactc	-	-
AB126628	Human H3N2 IAVs	Human	H1N2		2002	Japan	A/Yokohama/22/2002	agtgatgccccattccttgatcggcttcgccgagatcag<br>aggtccctaaggggaagaggcaacactc	-	-
CY003372	Human H3N2 IAVs	Human	H1N2		2002	USA	A/New_York/217/2002	agtgatgccccattccttgatcggcttcgccgagatcag<br>aggtccctaaggggaagaggcaacactc	-	-
JF710772	Human H3N2 IAVs	Human	H3N2		2002	United_Kingdom	A/England/198/2002	agtgatgccccattccttgatcggcttcgccgagatcag<br>aggtccctaaggggaagaggcaacactc	-	-
CY025054	Human H3N2 IAVs	Human	H3N2		2002	New_Zealand	A/Auckland/610/2002	agtgatgccccattccttgatcggcttcgccgagatcag<br>aggtccctaaggggaagaggcaacactc	-	-
CY022209	Human H3N2 IAVs	Human	H3N2		2002	New_Zealand	A/Auckland/614/2002	agtgatgccccattccttgatcggcttcgccgagatcag<br>aggtccctaaggggaagaggcaacactc	-	-
CY023078	Human H3N2 IAVs	Human	H3N2		2002	New_Zealand	A/Auckland/615/2002	agtgatgccccattccttgatcggcttcgccgagatcag<br>aggtccctaaggggaagaggcaacactc	-	-
CY091113	Human H3N2 IAVs	Human	H3N2		2002	USA	A/Georgia/NHRC0001/2002	agtgatgccccattccttgatcggcttcgccgagatcag<br>aggtccctaaggggaagaggcaacactc	-	-
CY090921	Human H3N2 IAVs	Human	H3N2		2002	USA	A/Illinois/NHRC0001/2002	agtgatgccccattccttgatcggcttcgccgagatcag<br>aggtccctaaggggaagaggcaacactc	-	-
CY091097	Human H3N2 IAVs	Human	H3N2		2002	USA	A/Missouri/NHRC0001/2002	agtgatgccccattccttgatcggcttcgccgagatcag<br>aggtccctaaggggaagaggcaacactc	-	-
CY000229	Human H3N2 IAVs	Human	H3N2		2002	USA	A/New_York/100/2002	agtgatgccccattccttgatcggcttcgccgagatcag<br>aggtccctaaggggaagaggcaacactc	-	-
CY001108	Human H3N2 IAVs	Human	H3N2		2002	USA	A/New_York/101/2002	agtgatgccccattccttgatcggcttcgccgagatcag<br>aggtccctaaggggaagaggcaacactc	-	-
CY003180	Human H3N2 IAVs	Human	H3N2		2002	USA	A/New_York/414/2002	agtgatgccccattccttgatcggcttcgccgagatcag<br>aggtccctaaggggaagaggcaacactc	-	-
CY091105	Human H3N2 IAVs	Human	H3N2		2002	USA	A/Texas/NHRC0001/2002	agtgatgccccattccttgatcggcttcgccgagatcag<br>aggtccctaaggggaagaggcaacactc	-	-
CY017087	Human H3N2 IAVs	Human	H3N2		2002	Australia	A/Western_Australia/27/2002	agtgatgccccattccttgatcggcttcgccgagatcag<br>aggtccctaaggggaagaggcaacactc	-	-
CY015760	Human H3N2 IAVs	Human	H3N2		2002	Australia	A/Western_Australia/35/2002	agtgatgccccattccttgatcggcttcgccgagatcag<br>aggtccctaaggggaagaggcaacactc	-	-
CY003125	Human H3N2 IAVs	Human	H3N2		2002	USA	A/New_York/406/2002	agtgatgccccattccttgatcggcttcgccgagatcag<br>aggtccctaaggggaagaggcaacactc	-	-
CY011628	Human H3N2 IAVs	Human	H3N2		2002	New_Zealand	A/Waikato/5/2002	agtgatgccccattccttgatcggcttcgccgagatcag<br>aggtccctaaggggaagaggcaacactc	-	-
CY011996	Human H3N2 IAVs	Human	H3N2		2002	New_Zealand	A/Wellington/70/2002	agtgatgccccattccttgatcggcttcgccgagatcag<br>aggtccctaaggggaagaggcaacactc	-	-

CY000285	Human H3N2 IAVs	Human	H3N2		2002	USA	A/New_York/86/2002	agtgatgccccattccttgatcggcttcgccgagatcagaggtccctaaggggaagaggcaacactc	-	-
CY015728	Human H3N2 IAVs	Human	H3N2		2002	Australia	A/Western_Australia/28/2002	agtgatgccccattccttgatcggcttcgccgagatcagaggtccctaaggggaagaggcaacactc	-	-
CY015736	Human H3N2 IAVs	Human	H3N2		2002	Australia	A/Western_Australia/29/2002	agtgatgccccattccttgatcggcttcgccgagatcagaggtccctaaggggaagaggcaacactc	-	-
CY011692	Human H3N2 IAVs	Human	H3N2		2002	New_Zealand	A/Wellington/86/2002	agtgatgccccattccttgatcggcttcgccgagatcagaggtccctaaggggaagaggcaacactc	-	-
CY019935	Human H3N2 IAVs	Human	H3N2		2002	Australia	A/Western_Australia/34/2002	agtgatgccccattccttgatcggcttcgccgagatcagaggtccctaaggggaagaggcaacactc	-	-
CY001132	Human H3N2 IAVs	Human	H3N2		2002	USA	A/New_York/105/2002	agtgatgccccattccttgatcggcttcgccgagatcagaggtccctaaggggaagaggcaacactc	-	-
CY000541	Human H3N2 IAVs	Human	H3N2		2002	USA	A/New_York/114/2002	agtgatgccccattccttgatcggcttcgccgagatcagaggtccctaaggggaagaggcaacactc	-	-
CY000557	Human H3N2 IAVs	Human	H3N2		2002	USA	A/New_York/116/2002	agtgatgccccattccttgatcggcttcgccgagatcagaggtccctaaggggaagaggcaacactc	-	-
CY000797	Human H3N2 IAVs	Human	H3N2		2002	USA	A/New_York/117/2002	agtgatgccccattccttgatcggcttcgccgagatcagaggtccctaaggggaagaggcaacactc	-	-
CY001732	Human H3N2 IAVs	Human	H3N2		2002	USA	A/New_York/274/2002	agtgatgccccattccttgatcggcttcgccgagatcagaggtccctaaggggaagaggcaacactc	-	-
CY000221	Human H3N2 IAVs	Human	H3N2		2002	USA	A/New_York/95/2002	agtgatgccccattccttgatcggcttcgccgagatcagaggtccctaaggggaagaggcaacactc	-	-
CY001433	Human H3N2 IAVs	Human	H3N2		2002	USA	A/New_York/76/2002	agtgatgccccattccttgatcggcttcgccgagatcagaggtccctaaggggaagaggcaacactc	-	-
CY000293	Human H3N2 IAVs	Human	H3N2		2002	USA	A/New_York/92/2002	agtgatgccccattccttgatcggcttcgccgagatcagaggtccctaaggggaagaggcaacactc	-	-
CY112945	Human H3N2 IAVs	Human	H3N2		2002	Netherlands	A/Netherlands/1/2002	agtgatgccccattccttgatcggcttcgccgagatcagaggtccctaaggggaagaggcaacactc	-	-
CY117825	Human H3N2 IAVs	Human	mixed		2002	Malaysia	A/Malaysia/22387/2002	agtgatgccccattccttgatcggcttcgccgagatcagaggtccctaaggggaagaggcaacactc	-	-
CY017905	Human H3N2 IAVs	Human	H3N2		2002	Australia	A/Queensland/22/2002	agtgatgccccattccttgatcggcttcgccgagatcagaggtccctaaggggaagaggcaacactc	-	-
CY013417	Human H3N2 IAVs	Human	H3N2		2002	New_Zealand	A/Waikato/31/2002	agtgatgccccattccttgatcggcttcgccgagatcagaggtccctaaggggaagaggcaacactc	-	-
CY013084	Human H3N2 IAVs	Human	H3N2		2002	New_Zealand	A/Wellington/9/2002	agtgatgccccattccttgatcggcttcgccgagatcagaggtccctaaggggaagaggcaacactc	-	-
CY000421	Human H3N2 IAVs	Human	H3N2		2002	USA	A/New_York/103/2002	agtgatgccccattccttgatcggcttcgccgagatcagaggtccctaaggggaagaggcaacactc	-	-
CY001948	Human H3N2 IAVs	Human	H3N2		2002	USA	A/New_York/126/2002	agtgatgccccattccttgatcggcttcgccgagatcagaggtccctaaggggaagaggcaacactc	-	-
CY003108	Human H3N2 IAVs	Human	H3N2		2002	USA	A/New_York/404/2002	agtgatgccccattccttgatcggcttcgccgagatcagaggtccctaaggggaagaggcaacactc	-	-
CY000405	Human H3N2 IAVs	Human	H3N2		2002	USA	A/New_York/89/2002	agtgatgccccattccttgatcggcttcgccgagatcagaggtccctaaggggaagaggcaacactc	-	-
CY000929	Human H3N2 IAVs	Human	H3N2		2002	USA	A/New_York/93/2002	agtgatgccccattccttgatcggcttcgccgagatcagaggtccctaaggggaagaggcaacactc	-	-
CY001337	Human H3N2 IAVs	Human	H3N2		2002	USA	A/New_York/118/2002	agtgatgccccattccttgatcggcttcgccgagatcagaggtccctaaggggaagaggcaacactc	-	-
CY003164	Human H3N2 IAVs	Human	H3N2		2002	USA	A/New_York/412/2002	agtgatgccccattccttgatcggcttcgccgagatcagaggtccctaaggggaagaggcaacactc	-	-

CY000117	Human H3N2 IAVs	Human	H3N2		2002	USA	A/New_York/110/2002	agtgatgccccattccttgatcggcttcgccgagatcag<br>aggtccctaaggggaagaggcaacactc	-	-
CY001201	Human H3N2 IAVs	Human	H3N2		2002	USA	A/New_York/122/2002	agtgatgccccattccttgatcggcttcgccgagatcag<br>aggtccctaaggggaagaggcaacactc	-	-
CY000429	Human H3N2 IAVs	Human	H3N2		2002	USA	A/New_York/128/2002	agtgatgccccattccttgatcggcttcgccgagatcag<br>aggtccctaaggggaagaggcaacactc	-	-
CY003140	Human H3N2 IAVs	Human	H3N2		2002	USA	A/New_York/409/2002	agtgatgccccattccttgatcggcttcgccgagatcag<br>aggtccctaaggggaagaggcaacactc	-	-
CY001305	Human H3N2 IAVs	Human	H3N2		2002	USA	A/New_York/75/2002	agtgatgccccattccttgatcggcttcgccgagatcag<br>aggtccctaaggggaagaggcaacactc	-	-
JF710774	Human H3N2 IAVs	Human	H3N2		2002	United_Kingdom	A/England/304/2002	agtgatgccccattccttgatcggcttcgccgagatcag<br>aggtccctaaggggaagaggcaacactc	-	-
CY022553	Human H3N2 IAVs	Human	H3N2		2002	New_Zealand	A/Auckland/608/2002	agtgatgccccattccttgatcggcttcgccgagatcag<br>aggtccctaaggggaagaggcaacactc	-	-
CY001188	Human H3N2 IAVs	Human	H3N2		2002	USA	A/New_York/104/2002	agtgatgccccattccttgatcggcttcgccgagatcag<br>aggtccctaaggggaagaggcaacactc	-	-
CY000549	Human H3N2 IAVs	Human	H3N2		2002	USA	A/New_York/115/2002	agtgatgccccattccttgatcggcttcgccgagatcag<br>aggtccctaaggggaagaggcaacactc	-	-
CY000245	Human H3N2 IAVs	Human	H3N2		2002	USA	A/New_York/121/2002	agtgatgccccattccttgatcggcttcgccgagatcag<br>aggtccctaaggggaagaggcaacactc	-	-
CY001740	Human H3N2 IAVs	Human	H3N2		2002	USA	A/New_York/276/2002	agtgatgccccattccttgatcggcttcgccgagatcag<br>aggtccctaaggggaagaggcaacactc	-	-
CY003100	Human H3N2 IAVs	Human	H3N2		2002	USA	A/New_York/403/2002	agtgatgccccattccttgatcggcttcgccgagatcag<br>aggtccctaaggggaagaggcaacactc	-	-
CY003116	Human H3N2 IAVs	Human	H3N2		2002	USA	A/New_York/405/2002	agtgatgccccattccttgatcggcttcgccgagatcag<br>aggtccctaaggggaagaggcaacactc	-	-
CY003428	Human H3N2 IAVs	Human	H3N2		2002	USA	A/New_York/407/2002	agtgatgccccattccttgatcggcttcgccgagatcag<br>aggtccctaaggggaagaggcaacactc	-	-
CY003156	Human H3N2 IAVs	Human	H3N2		2002	USA	A/New_York/411/2002	agtgatgccccattccttgatcggcttcgccgagatcag<br>aggtccctaaggggaagaggcaacactc	-	-
CY003188	Human H3N2 IAVs	Human	H3N2		2002	USA	A/New_York/415/2002	agtgatgccccattccttgatcggcttcgccgagatcag<br>aggtccctaaggggaagaggcaacactc	-	-
CY003212	Human H3N2 IAVs	Human	H3N2		2002	USA	A/New_York/419/2002	agtgatgccccattccttgatcggcttcgccgagatcag<br>aggtccctaaggggaagaggcaacactc	-	-
CY001265	Human H3N2 IAVs	Human	H3N2		2002	USA	A/New_York/81/2002	agtgatgccccattccttgatcggcttcgccgagatcag<br>aggtccctaaggggaagaggcaacactc	-	-
CY000397	Human H3N2 IAVs	Human	H3N2		2002	USA	A/New_York/87/2002	agtgatgccccattccttgatcggcttcgccgagatcag<br>aggtccctaaggggaagaggcaacactc	-	-
CY013100	Human H3N2 IAVs	Human	H3N2		2002	New_Zealand	A/Waikato/36/2002	agtgatgccccattccttgatcggcttcgccgagatcag<br>aggtccctaaggggaagaggcaacactc	-	-
CY003148	Human H3N2 IAVs	Human	H3N2		2002	USA	A/New_York/410/2002	agtgatgccccattccttgatcggcttcgccgagatcag<br>aggtccctaaggggaagaggcaacactc	-	-
CY001441	Human H3N2 IAVs	Human	H3N2		2002	USA	A/New_York/125/2002	agtgatgccccattccttgatcggcttcgccgagatcag<br>aggtccctaaggggaagaggcaacactc	-	-
DQ415365	Human H3N2 IAVs	Human	H3N2		2002	Taiwan	A/TW/3565/02	agtgatgccccattccttgatcggcttcgccgagatcag<br>aggtccctaaggggaagaggcaacactc	-	-
DQ415364	Human H3N2 IAVs	Human	H3N2		2002	Taiwan	A/TW/3446/02	agtgatgccccattccttgatcggcttcgccgagatcag<br>aggtccctaaggggaagaggcaacactc	-	-
CY003131	Human H3N2 IAVs	Human	H3N2		2002	USA	A/New_York/408/2002	agtgatgccccattccttgatcggcttcgccgagatcag<br>aggtccctaaggggaagaggcaacactc	-	-

CY000937	Human H3N2 IAVs	Human	H3N2		2002	USA	A/New_York/111/2002	agtgatgccccattccttgatcggcttcgccgagatcag<br>aggtccctaaggggaagaggcaacactc	-	-
CY001329	Human H3N2 IAVs	Human	H3N2		2002	USA	A/New_York/112/2002	agtgatgccccattccttgatcggcttcgccgagatcag<br>aggtccctaaggggaagaggcaacactc	-	-
CY015712	Human H3N2 IAVs	Human	H3N2		2002	Australia	A/Western_Australia/25/2002	agtgatgccccattccttgatcggcttcgccgagatcag<br>aggtccctaaggggaagaggcaacactc	-	-
CY000501	Human H3N2 IAVs	Human	H3N2		2002	USA	A/New_York/108/2002	agtgatgccccattccttgatcggcttcgccgagatcag<br>aggtccctaaggggaagaggcaacactc	-	-
CY000533	Human H3N2 IAVs	Human	H3N2		2002	USA	A/New_York/109/2002	agtgatgccccattccttgatcggcttcgccgagatcag<br>aggtccctaaggggaagaggcaacactc	-	-
CY000945	Human H3N2 IAVs	Human	H3N2		2002	USA	A/New_York/119/2002	agtgatgccccattccttgatcggcttcgccgagatcag<br>aggtccctaaggggaagaggcaacactc	-	-
CY000589	Human H3N2 IAVs	Human	H3N2		2002	USA	A/New_York/130/2002	agtgatgccccattccttgatcggcttcgccgagatcag<br>aggtccctaaggggaagaggcaacactc	-	-
CY000445	Human H3N2 IAVs	Human	H3N2		2002	USA	A/New_York/134/2002	agtgatgccccattccttgatcggcttcgccgagatcag<br>aggtccctaaggggaagaggcaacactc	-	-
CY001076	Human H3N2 IAVs	Human	H3N2		2002	USA	A/New_York/88/2002	agtgatgccccattccttgatcggcttcgccgagatcag<br>aggtccctaaggggaagaggcaacactc	-	-
CY001241	Human H3N2 IAVs	Human	H3N2		2002	USA	A/New_York/136/2002	agtgatgccccattccttgatcggcttcgccgagatcag<br>aggtccctaaggggaagaggcaacactc	-	-
CY000629	Human H3N2 IAVs	Human	H3N2		2002	USA	A/New_York/120/2002	agtgatgccccattccttgatcggcttcgccgagatcag<br>aggtccctaaggggaagaggcaacactc	-	-
CY003204	Human H3N2 IAVs	Human	H3N2		2002	USA	A/New_York/418/2002	agtgatgccccattccttgatcggcttcgccgagatcag<br>aggtccctaaggggaagaggcaacactc	-	-
CY015552	Human H3N2 IAVs	Human	H3N2		2002	New_Zealand	A/Dunedin/1/2002	agtgatgccccattccttgatcggcttcgccgagatcag<br>aggtccctaaggggaagaggcaacactc	-	-
CY001313	Human H3N2 IAVs	Human	H3N2		2002	USA	A/New_York/97/2002	agtgatgccccattccttgatcggcttcgccgagatcag<br>aggtccctaaggggaagaggcaacactc	-	-
CY015744	Human H3N2 IAVs	Human	H3N2		2002	Australia	A/Western_Australia/30/2002	agtgatgccccattccttgatcggcttcgccgagatcag<br>aggtccctaaggggaagaggcaacactc	-	-
CY000413	Human H3N2 IAVs	Human	H3N2		2002	USA	A/New_York/90/2002	agtgatgccccattccttgatcggcttcgccgagatcag<br>aggtccctaaggggaagaggcaacactc	-	-
CY017913	Human H3N2 IAVs	Human	H3N2		2002	Australia	A/Queensland/27/2002	agtgatgccccattccttgatcggcttcgccgagatcag<br>aggtccctaaggggaagaggcaacactc	-	-
CY003196	Human H3N2 IAVs	Human	H3N2		2002	USA	A/New_York/416/2002	agtgatgccccattccttgatcggcttcgccgagatcag<br>aggtccctaaggggaagaggcaacactc	-	-
CY117877	Human H3N2 IAVs	Human	mixed		2002	Malaysia	A/Malaysia/23582/2002	agtgatgccccattccttgatcggcttcgccgagatcag<br>aggtccctaaggggaagaggcaacactc	-	-
DQ415363	Human H3N2 IAVs	Human	H3N2		2002	Taiwan	A/TW/872/02	agtgatgccccattccttgatcggcttcgccgagatcag<br>aggtccctaaggggaagaggcaacactc	-	-
CY118662	Human H3N2 IAVs	Human	H3N2		2002	Malaysia	A/Malaysia/23606/2002	agtgatgccccattccttgatcggcttcgccgagatcag<br>aggtccctaaggggaagaggcaatactc	-	-
AY363589	Human H3N2 IAVs	Swine	H3N2		2002	Hong_Kong	A/Swine/Hong_Kong/1212/02	agtgatgccccattccttgatcggcttcgccgagatcag<br>aggtccctaaggggaagaggcaatactc	-	-
CY117848	Human H3N2 IAVs	Human	mixed		2002	Malaysia	A/Malaysia/23329/2002	agtgatgccccattccttgatcggcttcgccgagatcag<br>aggtccctaaggggaagaggcaatactc	-	-
CY117863	Human H3N2 IAVs	Human	mixed		2002	Malaysia	A/Malaysia/23378/2002	agtgatgccccattccttgatcggcttcgccgagatcag<br>aggtccctaaggggaagaggcaatactc	-	-
CY117906	Human H3N2 IAVs	Human	mixed		2002	Malaysia	A/Malaysia/23721/2002	agtgatgccccattccttgatcggcttcgccgagatcag<br>aggtccctaaggggaagaggcaatactc	-	-

CY003781	Human H3N2 IAVs	Human	H3N2		2002	USA	A/New_York/420/2002	agtgatgccccattccttgatcggcttcgccgagatcag aggtctctaaggggaagaggcaacactc	+	+
CY013108	Human H3N2 IAVs	Human	H3N2		2002	New_Zealand	A/Dunedin/18/2002	agtgatgccccattccttgatcggcttcgccgggatcag aggtccctaagaggaagaggcaacactc	+	+
CY022561	Human H3N2 IAVs	Human	H3N2		2002	New_Zealand	A/Auckland/609/2002	agtgatgccccattccttgatcggcttcgccgggatcag aggtccctaaggggaagaggcaacactc	-	-
JF710777	Human H3N2 IAVs	Human	H3N2		2002	United_Kingdom	A/England/95/2002	agtgatgccccattccttgatcggcttcgccgggatcag aggtccctaaggggaagaggcaacactc	-	-
CY007943	Human H3N2 IAVs	Human	H3N2		2002	New_Zealand	A/Canterbury/72/2002	agtgatgccccattccttgatcggcttcgccgggatcag aggtccctaaggggaagaggcaacactc	-	-
JF710728	Human H3N2 IAVs	Human	H3N2		2002	United_Kingdom	A/England/370/2002	agtgatgccccattccttgatcggcttcgccgggatcag aggtccctaaggggaagaggcaacactc	-	-
JF710776	Human H3N2 IAVs	Human	H3N2		2002	United_Kingdom	A/England/54/2002	agtgatgccccattccttgatcggcttcgccgggatcag aggtccctaaggggaagaggcaacactc	-	-
CY007751	Human H3N2 IAVs	Human	H3N2		2002	New_Zealand	A/Canterbury/68/2002	agtgatgccccattccttgatcggcttcgccgggatcag aggtccctaaggggaagaggcaacactc	-	-
JF710775	Human H3N2 IAVs	Human	H3N2		2002	United_Kingdom	A/England/407/2002	agtgatgccccattccttgatcggcttcgccgggatcag aggtccctaaggggaagaggcaacactc	-	-
CY011980	Human H3N2 IAVs	Human	H3N2		2002	New_Zealand	A/Waikato/2/2002	agtgatgccccattccttgatcggcttcgccgggatcag aggtccctaaggggaagaggcaacactc	-	-
CY037347	Human H3N2 IAVs	Human	H3N2		2002	Italy	A/Genoa/1/2002	agtgatgccccattccttgatcggcttcgccgggatcag aggtccctaaggggaagaggcaacactc	-	-
CY012020	Human H3N2 IAVs	Human	H3N2		2002	New_Zealand	A/Wellington/71/2002	agtgatgccccattccttgatcggcttcgccgggatcag aggtccctaaggggaagaggcaacactc	-	-
CY012044	Human H3N2 IAVs	Human	H3N2		2002	New_Zealand	A/Wellington/79/2002	agtgatgccccattccttgatcggcttcgccgggatcag aggtccctaaggggaagaggcaacactc	-	-
AB434123	Human H3N2 IAVs	Human	H3N2		2002	Japan	A/Morioka/34/2002	agtgatgccccattccttgatcggcttcgccgggatcag aggtccctaaggggaagaggcaacactc	-	-
AB434124	Human H3N2 IAVs	Human	H3N2		2002	Japan	A/Morioka/52/2002	agtgatgccccattccttgatcggcttcgccgggatcag aggtccctaaggggaagaggcaacactc	-	-
CY000301	Human H3N2 IAVs	Human	H3N2		2002	USA	A/New_York/96/2002	agtgatgccccatttcttgatcggcttcgccgagatcag aggtccctaaggggaagaggcaacactc	-	-
CY117892	Human H3N2 IAVs	Human	mixed		2002	Malaysia	A/Malaysia/23637/2002	agtgatgccccatttcttgatcggcttcgccgagatcag aggtccctaaggggaagaggcaacactc	-	-
CY007895	Human H3N2 IAVs	Human	H3N2		2002	New_Zealand	A/Canterbury/33/2002	agtgatgctccattccttgatcggcttcgccgagatcag agggccctaaggggaagaggcaacactc	-	-
CY017543	Human H3N2 IAVs	Human	H3N2		2002	Australia	A/Queensland/23/2002	agtgatgctccattccttgatcggcttcgccgagatcag aggtccctaagaggaagaggcaacactc	-	*
CY017929	Human H3N2 IAVs	Human	H3N2		2002	Australia	A/Queensland/29/2002	agtgatgctccattccttgatcggcttcgccgagatcag aggtccctaagaggaagaggcaacactc	-	*
CY017559	Human H3N2 IAVs	Human	H3N2		2002	Australia	A/Queensland/26/2002	agtgatgctccattccttgatcggcttcgccgagatcag aggtccctaagaggaagaggcaacactc	-	*
CY018969	Human H3N2 IAVs	Human	H3N2		2002	Australia	A/Queensland/30/2002	agtgatgctccattccttgatcggcttcgccgagatcag aggtccctaagaggaagaggcaacactc	-	*
CY013228	Human H3N2 IAVs	Human	H3N2		2002	Australia	A/Western_Australia/23/2002	agtgatgctccattccttgatcggcttcgccgagatcag aggtccctaagaggaagaggcaacactc	-	*
CY017359	Human H3N2 IAVs	Human	H3N2		2002	Australia	A/Western_Australia/24/2002	agtgatgctccattccttgatcggcttcgccgagatcag aggtccctaagaggaagaggcaacactc	-	*
CY015720	Human H3N2 IAVs	Human	H3N2		2002	Australia	A/Western_Australia/26/2002	agtgatgctccattccttgatcggcttcgccgagatcag aggtccctaagaggaagaggcaacactc	-	*

CY015752	Human H3N2 IAVs	Human	H3N2		2002	Australia	A/Western_Australia/31/2002	agtgatgctccattccttgatcggcttcgccgagatcag aggtccctaagaggaagaggcaacactc	-	*
CY000493	Human H3N2 IAVs	Human	H3N2		2002	USA	A/New_York/106/2002	agtgatgctccattccttgatcggcttcgccgagatcag aggtccctaaggggaagaggcaacactc	-	-
CY000437	Human H3N2 IAVs	Human	H3N2		2002	USA	A/New_York/132/2002	agtgatgctccattccttgatcggcttcgccgagatcag aggtccctaaggggaagaggcaacactc	-	-
CY001156	Human H3N2 IAVs	Human	H3N2		2002	USA	A/New_York/74/2002	agtgatgctccattccttgatcggcttcgccgagatcag aggtccctaaggggaagaggcaacactc	-	-
CY000237	Human H3N2 IAVs	Human	H3N2		2002	USA	A/New_York/113/2002	agtgatgctccattccttgatcggcttcgccgagatcag aggtccctaaggggaagaggcaacactc	-	-
CY001084	Human H3N2 IAVs	Human	H3N2		2002	USA	A/New_York/99/2002	agtgatgctccattccttgatcggcttcgccgagatcag aggtccctaaggggaagaggcaacactc	-	-
CY000317	Human H3N2 IAVs	Human	H3N2		2002	USA	A/New_York/129/2002	agtgatgctccattccttgatcggcttcgccgagatcag aggtccctaaggggaagaggcaacactc	-	-
CY114345	Human H3N2 IAVs	Human	H3N2		2002	Netherlands	A/Netherlands/120/2002	agtgatgctccattccttgatcggcttcgccgagatcag aggtccctaaggggaagaggcaacactc	-	-
CY022193	Human H3N2 IAVs	Human	H3N2		2002	New_Zealand	A/Auckland/611/2002	agtgatgctccattccttgatcggcttcgccgagatcag aggtccctaaggggaagaggcaacactc	-	-
CY022569	Human H3N2 IAVs	Human	H3N2		2002	New_Zealand	A/Auckland/612/2002	agtgatgctccattccttgatcggcttcgccgagatcag aggtccctaaggggaagaggcaacactc	-	-
CY022201	Human H3N2 IAVs	Human	H3N2		2002	New_Zealand	A/Auckland/613/2002	agtgatgctccattccttgatcggcttcgccgagatcag aggtccctaaggggaagaggcaacactc	-	-
CY007591	Human H3N2 IAVs	Human	H3N2		2002	New_Zealand	A/Canterbury/01/2002	agtgatgctccattccttgatcggcttcgccgagatcag aggtccctaaggggaagaggcaacactc	-	-
CY008007	Human H3N2 IAVs	Human	H3N2		2002	New_Zealand	A/Canterbury/04/2002	agtgatgctccattccttgatcggcttcgccgagatcag aggtccctaaggggaagaggcaacactc	-	-
CY008272	Human H3N2 IAVs	Human	H3N2		2002	New_Zealand	A/Canterbury/09/2002	agtgatgctccattccttgatcggcttcgccgagatcag aggtccctaaggggaagaggcaacactc	-	-
CY007887	Human H3N2 IAVs	Human	H3N2		2002	New_Zealand	A/Canterbury/31/2002	agtgatgctccattccttgatcggcttcgccgagatcag aggtccctaaggggaagaggcaacactc	-	-
CY007719	Human H3N2 IAVs	Human	H3N2		2002	New_Zealand	A/Canterbury/50/2002	agtgatgctccattccttgatcggcttcgccgagatcag aggtccctaaggggaagaggcaacactc	-	-
CY007743	Human H3N2 IAVs	Human	H3N2		2002	New_Zealand	A/Canterbury/59/2002	agtgatgctccattccttgatcggcttcgccgagatcag aggtccctaaggggaagaggcaacactc	-	-
CY012028	Human H3N2 IAVs	Human	H3N2		2002	New_Zealand	A/Waikato/29/2002	agtgatgctccattccttgatcggcttcgccgagatcag aggtccctaaggggaagaggcaacactc	-	-
CY012052	Human H3N2 IAVs	Human	H3N2		2002	New_Zealand	A/Waikato/52/2002	agtgatgctccattccttgatcggcttcgccgagatcag aggtccctaaggggaagaggcaacactc	-	-
CY015560	Human H3N2 IAVs	Human	H3N2		2002	New_Zealand	A/Waikato/23/2002	agtgatgctccattccttgatcggcttcgccgagatcag aggtccctaaggggaagaggcaacactc	-	-
CY007599	Human H3N2 IAVs	Human	H3N2		2002	New_Zealand	A/Canterbury/02/2002	agtgatgctccattccttgatcggcttcgccgagatcag aggtccctaaggggaagaggcaacactc	-	-
CY007607	Human H3N2 IAVs	Human	H3N2		2002	New_Zealand	A/Canterbury/05/2002	agtgatgctccattccttgatcggcttcgccgagatcag aggtccctaaggggaagaggcaacactc	-	-
CY008264	Human H3N2 IAVs	Human	H3N2		2002	New_Zealand	A/Canterbury/06/2002	agtgatgctccattccttgatcggcttcgccgagatcag aggtccctaaggggaagaggcaacactc	-	-
CY007791	Human H3N2 IAVs	Human	H3N2		2002	New_Zealand	A/Canterbury/102/2002	agtgatgctccattccttgatcggcttcgccgagatcag aggtccctaaggggaagaggcaacactc	-	-
CY008015	Human H3N2 IAVs	Human	H3N2		2002	New_Zealand	A/Canterbury/13/2002	agtgatgctccattccttgatcggcttcgccgagatcag aggtccctaaggggaagaggcaacactc	-	-

CY007847	Human H3N2 IAVs	Human	H3N2		2002	New_Zealand	A/Canterbury/14/2002	agtgatgctccattccttgatcggcttcgccgagatcag<br>aggtccctaaggggaagaggcaacactc	-	-
CY008288	Human H3N2 IAVs	Human	H3N2		2002	New_Zealand	A/Canterbury/15/2002	agtgatgctccattccttgatcggcttcgccgagatcag<br>aggtccctaaggggaagaggcaacactc	-	-
CY007655	Human H3N2 IAVs	Human	H3N2		2002	New_Zealand	A/Canterbury/16/2002	agtgatgctccattccttgatcggcttcgccgagatcag<br>aggtccctaaggggaagaggcaacactc	-	-
CY007663	Human H3N2 IAVs	Human	H3N2		2002	New_Zealand	A/Canterbury/18/2002	agtgatgctccattccttgatcggcttcgccgagatcag<br>aggtccctaaggggaagaggcaacactc	-	-
CY007855	Human H3N2 IAVs	Human	H3N2		2002	New_Zealand	A/Canterbury/19/2002	agtgatgctccattccttgatcggcttcgccgagatcag<br>aggtccctaaggggaagaggcaacactc	-	-
CY007863	Human H3N2 IAVs	Human	H3N2		2002	New_Zealand	A/Canterbury/20/2002	agtgatgctccattccttgatcggcttcgccgagatcag<br>aggtccctaaggggaagaggcaacactc	-	-
CY008023	Human H3N2 IAVs	Human	H3N2		2002	New_Zealand	A/Canterbury/21/2002	agtgatgctccattccttgatcggcttcgccgagatcag<br>aggtccctaaggggaagaggcaacactc	-	-
CY007671	Human H3N2 IAVs	Human	H3N2		2002	New_Zealand	A/Canterbury/22/2002	agtgatgctccattccttgatcggcttcgccgagatcag<br>aggtccctaaggggaagaggcaacactc	-	-
CY007879	Human H3N2 IAVs	Human	H3N2		2002	New_Zealand	A/Canterbury/29/2002	agtgatgctccattccttgatcggcttcgccgagatcag<br>aggtccctaaggggaagaggcaacactc	-	-
CY007903	Human H3N2 IAVs	Human	H3N2		2002	New_Zealand	A/Canterbury/34/2002	agtgatgctccattccttgatcggcttcgccgagatcag<br>aggtccctaaggggaagaggcaacactc	-	-
CY007687	Human H3N2 IAVs	Human	H3N2		2002	New_Zealand	A/Canterbury/41/2002	agtgatgctccattccttgatcggcttcgccgagatcag<br>aggtccctaaggggaagaggcaacactc	-	-
CY007919	Human H3N2 IAVs	Human	H3N2		2002	New_Zealand	A/Canterbury/44/2002	agtgatgctccattccttgatcggcttcgccgagatcag<br>aggtccctaaggggaagaggcaacactc	-	-
CY008031	Human H3N2 IAVs	Human	H3N2		2002	New_Zealand	A/Canterbury/46/2002	agtgatgctccattccttgatcggcttcgccgagatcag<br>aggtccctaaggggaagaggcaacactc	-	-
CY007695	Human H3N2 IAVs	Human	H3N2		2002	New_Zealand	A/Canterbury/47/2002	agtgatgctccattccttgatcggcttcgccgagatcag<br>aggtccctaaggggaagaggcaacactc	-	-
CY007703	Human H3N2 IAVs	Human	H3N2		2002	New_Zealand	A/Canterbury/48/2002	agtgatgctccattccttgatcggcttcgccgagatcag<br>aggtccctaaggggaagaggcaacactc	-	-
CY007927	Human H3N2 IAVs	Human	H3N2		2002	New_Zealand	A/Canterbury/58/2002	agtgatgctccattccttgatcggcttcgccgagatcag<br>aggtccctaaggggaagaggcaacactc	-	-
CY008296	Human H3N2 IAVs	Human	H3N2		2002	New_Zealand	A/Canterbury/60/2002	agtgatgctccattccttgatcggcttcgccgagatcag<br>aggtccctaaggggaagaggcaacactc	-	-
CY008304	Human H3N2 IAVs	Human	H3N2		2002	New_Zealand	A/Canterbury/61/2002	agtgatgctccattccttgatcggcttcgccgagatcag<br>aggtccctaaggggaagaggcaacactc	-	-
CY007759	Human H3N2 IAVs	Human	H3N2		2002	New_Zealand	A/Canterbury/69/2002	agtgatgctccattccttgatcggcttcgccgagatcag<br>aggtccctaaggggaagaggcaacactc	-	-
CY008336	Human H3N2 IAVs	Human	H3N2		2002	New_Zealand	A/Canterbury/76/2002	agtgatgctccattccttgatcggcttcgccgagatcag<br>aggtccctaaggggaagaggcaacactc	-	-
CY007951	Human H3N2 IAVs	Human	H3N2		2002	New_Zealand	A/Canterbury/79/2002	agtgatgctccattccttgatcggcttcgccgagatcag<br>aggtccctaaggggaagaggcaacactc	-	-
CY011660	Human H3N2 IAVs	Human	H3N2		2002	New_Zealand	A/Dunedin/14/2002	agtgatgctccattccttgatcggcttcgccgagatcag<br>aggtccctaaggggaagaggcaacactc	-	-
CY012004	Human H3N2 IAVs	Human	H3N2		2002	New_Zealand	A/Dunedin/9/2002	agtgatgctccattccttgatcggcttcgccgagatcag<br>aggtccctaaggggaagaggcaacactc	-	-
CY012060	Human H3N2 IAVs	Human	H3N2		2002	New_Zealand	A/Waikato/51/2002	agtgatgctccattccttgatcggcttcgccgagatcag<br>aggtccctaaggggaagaggcaacactc	-	-
CY011676	Human H3N2 IAVs	Human	H3N2		2002	New_Zealand	A/Wellington/83/2002	agtgatgctccattccttgatcggcttcgccgagatcag<br>aggtccctaaggggaagaggcaacactc	-	-

CY011684	Human H3N2 IAVs	Human	H3N2		2002	New_Zealand	A/Wellington/84/2002	agtgatgctccattccttgatcggcttcgccgagatcag aggtccctaaggggaagaggcaacactc	-	-
CY007727	Human H3N2 IAVs	Human	H3N2		2002	New_Zealand	A/Canterbury/53/2002	agtgatgctccattccttgatcggcttcgccgagatcag aggtccctaaggggaagaggcaacactc	-	-
CY008312	Human H3N2 IAVs	Human	H3N2		2002	New_Zealand	A/Canterbury/62/2002	agtgatgctccattccttgatcggcttcgccgagatcag aggtccctaaggggaagaggcaacactc	-	-
CY011636	Human H3N2 IAVs	Human	H3N2		2002	New_Zealand	A/Wellington/38/2002	agtgatgctccattccttgatcggcttcgccgagatcag aggtccctaaggggaagaggcaacactc	-	-
CY012036	Human H3N2 IAVs	Human	H3N2		2002	New_Zealand	A/Wellington/80/2002	agtgatgctccattccttgatcggcttcgccgagatcag aggtccctaaggggaagaggcaacactc	-	-
CY015768	Human H3N2 IAVs	Human	H3N2		2002	Australia	A/Western_Australia/36/2002	agtgatgctccattccttgatcggcttcgccgagatcag aggtccctaaggggaagaggcaacactc	-	-
CY007711	Human H3N2 IAVs	Human	H3N2		2002	New_Zealand	A/Canterbury/49/2002	agtgatgctccattccttgatcggcttcgccgagatcag aggtccctaaggggaagaggcaacactc	-	-
CY008039	Human H3N2 IAVs	Human	H3N2		2002	New_Zealand	A/Canterbury/57/2002	agtgatgctccattccttgatcggcttcgccgagatcag aggtccctaaggggaagaggcaacactc	-	-
CY008328	Human H3N2 IAVs	Human	H3N2		2002	New_Zealand	A/Canterbury/70/2002	agtgatgctccattccttgatcggcttcgccgagatcag aggtccctaaggggaagaggcaacactc	-	-
CY007783	Human H3N2 IAVs	Human	H3N2		2002	New_Zealand	A/Canterbury/81/2002	agtgatgctccattccttgatcggcttcgccgagatcag aggtccctaaggggaagaggcaacactc	-	-
CY008280	Human H3N2 IAVs	Human	H3N2		2002	New_Zealand	A/Canterbury/10/2002	agtgatgctccattccttgatcggcttcgccgagatcag aggtccctaaggggaagaggcaacactc	-	-
CY007871	Human H3N2 IAVs	Human	H3N2		2002	New_Zealand	A/Canterbury/27/2002	agtgatgctccattccttgatcggcttcgccgagatcag aggtccctaaggggaagaggcaacactc	-	-
CY008320	Human H3N2 IAVs	Human	H3N2		2002	New_Zealand	A/Canterbury/64/2002	agtgatgctccattccttgatcggcttcgccgagatcag aggtccctaaggggaagaggcaacactc	-	-
CY007767	Human H3N2 IAVs	Human	H3N2		2002	New_Zealand	A/Canterbury/75/2002	agtgatgctccattccttgatcggcttcgccgagatcag aggtccctaaggggaagaggcaacactc	-	-
CY011404	Human H3N2 IAVs	Human	H3N2		2002	New_Zealand	A/Dunedin/10/2002	agtgatgctccattccttgatcggcttcgccgagatcag aggtccctaaggggaagaggcaacactc	-	-
CY007935	Human H3N2 IAVs	Human	H3N2		2002	New_Zealand	A/Canterbury/66/2002	agtgatgctccattccttgatcggcttcgccgagatcag aggtccctaaggggaagaggcaacactc	-	-
CY007775	Human H3N2 IAVs	Human	H3N2		2002	New_Zealand	A/Canterbury/80/2002	agtgatgctccattccttgatcggcttcgccgagatcag aggtccctaaggggaagaggcaacactc	-	-
CY012012	Human H3N2 IAVs	Human	H3N2		2002	New_Zealand	A/Dunedin/8/2002	agtgatgctccattccttgatcggcttcgccgagatcag aggtccctaaggggaagaggcaacactc	-	-
CY017551	Human H3N2 IAVs	Human	H3N2		2002	Australia	A/Queensland/24/2002	agtgatgctccattccttgatcggcttcgccgagatcag aggtccctaaggggaagaggcaacactc	-	-
CY007679	Human H3N2 IAVs	Human	H3N2		2002	New_Zealand	A/Canterbury/35/2002	agtgatgctccattccttgatcggcttcgccgagatcag aggtccctaaggggaagaggcaacactc	-	-
CY017535	Human H3N2 IAVs	Human	H3N2		2002	Australia	A/Queensland/21/2002	agtgatgctccattccttgatcggcttcgccgagatcag aggtccctaaggggaagaggcaacactc	-	-
CY001148	Human H3N2 IAVs	Human	H3N2		2002	USA	A/New_York/107/2002	agtgatgctccattccttgatcggcttcgccgagatcag aggtccctaaggggaagaggcaacactc	-	-
CY007735	Human H3N2 IAVs	Human	H3N2		2002	New_Zealand	A/Canterbury/56/2002	agtgatgctccattccttgatcggcttcgccgagatcag aggtccctaaggggaagaggcaacactc	-	-
CY013092	Human H3N2 IAVs	Human	H3N2		2002	New_Zealand	A/Waikato/25/2002	agtgatgctccattccttgatcggcttcgccgagatcag aggtccctaaggggaagaggcaacactc	-	-
CY013425	Human H3N2 IAVs	Human	H3N2		2002	New_Zealand	A/Dunedin/12/2002	agtgatgctccattccttgatcggcttcgccgagatcag aggtccctaaggggaagaggcaacactc	-	-

CY012340	Human H3N2 IAVs	Human	H3N2		2002	New_Zealand	A/Wellington/63/2002	agtgatgctccattccttgatcggcttcgccgagatcag aggtccctaaggggaagaggcaacactc	-	-
CY011644	Human H3N2 IAVs	Human	H3N2		2002	New_Zealand	A/Wellington/66/2002	agtgatgctccattccttgatcggcttcgccgagatcag aggtccctaaggggaagaggcaacactc	-	-
CY012644	Human H3N2 IAVs	Human	H3N2		2002	New_Zealand	A/Dunedin/3/2002	agtgatgctccattccttgatcggcttcgccgagatcag aggtccctaaggggaagaggcaacactc	-	-
CY007911	Human H3N2 IAVs	Human	H3N2		2002	New_Zealand	A/South_Canterbury/37/2002	agtgatgctccattccttgatcggcttcgccgaratcag aggtccctaaggggaagaggcaacactc	-	-
CY017921	Human H3N2 IAVs	Human	H3N2		2002	Australia	A/Queensland/28/2002	agtgatgctccattctttgatcggcttcgccgagatcag aggtccctaaggggaagaggcaacactc	+	+
CY003172	Human H3N2 IAVs	Human	H3N2		2002	USA	A/New_York/413/2002	agtgatgtcccattccttgatcggcttcgccgagatcag aggtccctaaggggaagaggcaacactc	-	-
GQ422408	Human H3N2 IAVs	Swine	H3N2		2002	China	A/swine/Guangdong/102/2002	ggtgatgccccattccttgatcggcttcgccgagatcag aagtccctaaggggaagaggcagcactc	+	+
GQ422409	Human H3N2 IAVs	Swine	H3N2		2002	China	A/swine/Guangdong/106/2002	ggtgatgccccattccttgatcggcttcgccgagatcag aagtccctaaggggaagaggcagcactc	+	+
GQ422410	Human H3N2 IAVs	Swine	H3N2		2002	China	A/swine/Guangdong/107/2002	ggtgatgccccattccttgatcggcttcgccgagatcag aagtccctaaggggaagaggcagcactc	+	+
GQ422411	Human H3N2 IAVs	Swine	H3N2		2002	China	A/swine/Guangdong/110/2002	ggtgatgccccattccttgatcggcttcgccgagatcag aagtccctaaggggaagaggcagcactc	+	+
GQ422412	Human H3N2 IAVs	Swine	H3N2		2002	China	A/swine/Guangdong/111/2002	ggtgatgccccattccttgatcggcttcgccgagatcag aagtccctaaggggaagaggcagcactc	+	+
GQ422413	Human H3N2 IAVs	Swine	H3N2		2002	China	A/swine/Guangdong/113/2002	ggtgatgccccattccttgatcggcttcgccgagatcag aagtccctaaggggaagaggcagcactc	+	+
CY112937	Human H3N2 IAVs	Human	H3N2		2002	China	A/Fujian/411/2002	ggtgatgccccattccttgatcggcttcgccgagatcag aggtccctaaggggaagaggcaatactc	-	-
CY088487	Human H3N2 IAVs	Human	H3N2		2002	China	A/Fujian/411/2002	ggtgatgccccattccttgatcggcttcgccgagatcag aggtccctaaggggaagaggcaatactc	-	-
AM502797	Human H3N2 IAVs	Human	H3N2		2002	China	A/Fujian/411/2002	ggtgatgccccattccttgatcggcttcgccgagatcag aggtccctaaggggaagaggcaatactc	-	-
CY008552	Human H3N2 IAVs	Human	H3N2		2003	New_Zealand	A/Canterbury/426/2003	agtgatgccccattccttgaccggcttcgccgagatca gaggtccctaaggggaagaggcaacactc	-	-
CY011756	Human H3N2 IAVs	Human	H3N2		2003	New_Zealand	A/Waikato/61/2003	agtgatgccccattccttgatcgacttcgccgagatca gaggtccctaaggggaagaggcaacactc	-	-
CY007231	Human H3N2 IAVs	Human	H3N2		2003	New_Zealand	A/Canterbury/439/2003	agtgatgccccattccttgatcggcttcgccgagataa gaggtccctaaggggaagaggcaacactc	-	-
CY015832	Human H3N2 IAVs	Human	H3N2		2003	Australia	A/Western_Australia/44/2003	agtgatgccccattccttgatcggcttcgccgagatcag aagtccctaaggggaagaggcaacactc	+	+
CY090953	Human H3N2 IAVs	Human	H3N2		2003	USA	A/South_Carolina/NHRC0002/200 3	agtgatgccccattccttgatcggcttcgccgagatcag aggtccataaggggaagaggcaacactc	+	+
CY003765	Human H3N2 IAVs	Human	H1N2		2003	USA	A/New_York/400/2003	agtgatgccccattccttgatcggcttcgccgagatcag aggtccctaaggggaagaggcaacacta	-	-
CY002356	Human H3N2 IAVs	Human	H1N2		2003	USA	A/New_York/209/2003	agtgatgccccattccttgatcggcttcgccgagatcag aggtccctaaggggaagaggcaacactc	-	-
CY006871	Human H3N2 IAVs	Human	H1N2		2003	USA	A/New_York/211/2003	agtgatgccccattccttgatcggcttcgccgagatcag aggtccctaaggggaagaggcaacactc	-	-
CY010080	Human H3N2 IAVs	Human	H1N2		2003	USA	A/New_York/229/2003	agtgatgccccattccttgatcggcttcgccgagatcag aggtccctaaggggaagaggcaacactc	-	-
CY002364	Human H3N2 IAVs	Human	H1N2		2003	USA	A/New_York/300/2003	agtgatgccccattccttgatcggcttcgccgagatcag aggtccctaaggggaagaggcaacactc	-	-

CY017119	Human H3N2 IAVs	Human	H1N2		2003	USA	A/New_York/C1/2003	agtgatgccccattccttgatcggcttcgccgagatcag aggtccctaaggggaagaggcaacactc	-	-
CY002636	Human H3N2 IAVs	Human	H1N2		2003	USA	A/New_York/231/2003	agtgatgccccattccttgatcggcttcgccgagatcag aggtccctaaggggaagaggcaacactc	-	-
CY002156	Human H3N2 IAVs	Human	H1N2		2003	USA	A/New_York/296/2003	agtgatgccccattccttgatcggcttcgccgagatcag aggtccctaaggggaagaggcaacactc	-	-
CY002660	Human H3N2 IAVs	Human	H1N2		2003	USA	A/New_York/294/2003	agtgatgccccattccttgatcggcttcgccgagatcag aggtccctaaggggaagaggcaacactc	-	-
CY006415	Human H3N2 IAVs	Human	H1N2		2003	USA	A/New_York/492/2003	agtgatgccccattccttgatcggcttcgccgagatcag aggtccctaaggggaagaggcaacactc	-	-
CY006111	Human H3N2 IAVs	Human	H1N2		2003	USA	A/New_York/219/2003	agtgatgccccattccttgatcggcttcgccgagatcag aggtccctaaggggaagaggcaacactc	-	-
CY002996	Human H3N2 IAVs	Human	H1N2		2003	USA	A/New_York/226/2003	agtgatgccccattccttgatcggcttcgccgagatcag aggtccctaaggggaagaggcaacactc	-	-
CY006751	Human H3N2 IAVs	Human	H1N2		2003	USA	A/New_York/225/2003	agtgatgccccattccttgatcggcttcgccgagatcag aggtccctaaggggaagaggcaacactc	-	-
CY006191	Human H3N2 IAVs	Human	H1N2		2003	USA	A/New_York/491/2003	agtgatgccccattccttgatcggcttcgccgagatcag aggtccctaaggggaagaggcaacactc	-	-
CY003676	Human H3N2 IAVs	Human	H1N2		2003	USA	A/New_York/482/2003	agtgatgccccattccttgatcggcttcgccgagatcag aggtccctaaggggaagaggcaacactc	-	-
CY006407	Human H3N2 IAVs	Human	H1N2		2003	USA	A/New_York/490/2003	agtgatgccccattccttgatcggcttcgccgagatcag aggtccctaaggggaagaggcaacactc	-	-
DQ280224	Human H3N2 IAVs	Swine	H1N2		2003	Canada	A/swine/Ontario/52156/03	agtgatgccccattccttgatcggcttcgccgagatcag aggtccctaaggggaagaggcaacactc	-	-
CY006391	Human H3N2 IAVs	Human	H1N2		2003	USA	A/New_York/481/2003	agtgatgccccattccttgatcggcttcgccgagatcag aggtccctaaggggaagaggcaacactc	-	-
CY006399	Human H3N2 IAVs	Human	H1N2		2003	USA	A/New_York/487/2003	agtgatgccccattccttgatcggcttcgccgagatcag aggtccctaaggggaagaggcaacactc	-	-
CY003700	Human H3N2 IAVs	Human	H1N2		2003	USA	A/New_York/489/2003	agtgatgccccattccttgatcggcttcgccgagatcag aggtccctaaggggaagaggcaacactc	-	-
FJ970923	Human H3N2 IAVs	Swine	H3N2		2003	China	A/swine/Guangdong/Z5/2003	agtgatgccccattccttgatcggcttcgccgagatcag aggtccctaaggggaagaggcaacactc	-	-
CY112969	Human H3N2 IAVs	Human	H3N2		2003	Netherlands	A/Netherlands/88/2003	agtgatgccccattccttgatcggcttcgccgagatcag aggtccctaaggggaagaggcaacactc	-	-
CY088018	Human H3N2 IAVs	Human	H3N2		2003	United_Kingdom	A/England/419/2003	agtgatgccccattccttgatcggcttcgccgagatcag aggtccctaaggggaagaggcaacactc	-	-
CY088042	Human H3N2 IAVs	Human	H3N2		2003	United_Kingdom	A/England/438/2003	agtgatgccccattccttgatcggcttcgccgagatcag aggtccctaaggggaagaggcaacactc	-	-
CY088090	Human H3N2 IAVs	Human	H3N2		2003	United_Kingdom	A/England/460/2003	agtgatgccccattccttgatcggcttcgccgagatcag aggtccctaaggggaagaggcaacactc	-	-
CY107134	Human H3N2 IAVs	Human	H3N2		2003	United_Kingdom	A/England/484/2003	agtgatgccccattccttgatcggcttcgccgagatcag aggtccctaaggggaagaggcaacactc	-	-
CY088266	Human H3N2 IAVs	Human	H3N2		2003	United_Kingdom	A/England/484/2003	agtgatgccccattccttgatcggcttcgccgagatcag aggtccctaaggggaagaggcaacactc	-	-
AM502784	Human H3N2 IAVs	Human	H3N2		2003	United_Kingdom	A/England/484/2003	agtgatgccccattccttgatcggcttcgccgagatcag aggtccctaaggggaagaggcaacactc	-	-
CY107118	Human H3N2 IAVs	Human	H3N2		2003	United_Kingdom	A/England/516/2003	agtgatgccccattccttgatcggcttcgccgagatcag aggtccctaaggggaagaggcaacactc	-	-
CY088202	Human H3N2 IAVs	Human	H3N2		2003	United_Kingdom	A/England/516/2003	agtgatgccccattccttgatcggcttcgccgagatcag aggtccctaaggggaagaggcaacactc	-	-

AM502790	Human H3N2 IAVs	Human	H3N2		2003	United_Kingdom	A/England/516/2003	agtgatgccccattccttgatcggcttcgccgagatcag aggtccctaaggggaagaggcaacactc	-	-
AM502796	Human H3N2 IAVs	Human	H3N2		2003	United_Kingdom	A/England/539/2003	agtgatgccccattccttgatcggcttcgccgagatcag aggtccctaaggggaagaggcaacactc	-	-
CY088410	Human H3N2 IAVs	Human	H3N2		2003	United_Kingdom	A/England/547/2003	agtgatgccccattccttgatcggcttcgccgagatcag aggtccctaaggggaagaggcaacactc	-	-
JF710755	Human H3N2 IAVs	Human	H3N2		2003	United_Kingdom	A/England/612/2003	agtgatgccccattccttgatcggcttcgccgagatcag aggtccctaaggggaagaggcaacactc	-	-
CY107086	Human H3N2 IAVs	Human	H3N2		2003	United_Kingdom	A/England/754/2003	agtgatgccccattccttgatcggcttcgccgagatcag aggtccctaaggggaagaggcaacactc	-	-
CY088082	Human H3N2 IAVs	Human	H3N2		2003	United_Kingdom	A/England/754/2003	agtgatgccccattccttgatcggcttcgccgagatcag aggtccctaaggggaagaggcaacactc	-	-
AM502806	Human H3N2 IAVs	Human	H3N2		2003	United_Kingdom	A/England/754/2003	agtgatgccccattccttgatcggcttcgccgagatcag aggtccctaaggggaagaggcaacactc	-	-
CY107110	Human H3N2 IAVs	Human	H3N2		2003	United_Kingdom	A/England/786/2003	agtgatgccccattccttgatcggcttcgccgagatcag aggtccctaaggggaagaggcaacactc	-	-
CY088170	Human H3N2 IAVs	Human	H3N2		2003	United_Kingdom	A/England/786/2003	agtgatgccccattccttgatcggcttcgccgagatcag aggtccctaaggggaagaggcaacactc	-	-
CY088362	Human H3N2 IAVs	Human	H3N2		2003	United_Kingdom	A/England/805/2003	agtgatgccccattccttgatcggcttcgccgagatcag aggtccctaaggggaagaggcaacactc	-	-
AM502811	Human H3N2 IAVs	Human	H3N2		2003	United_Kingdom	A/England/805/2003	agtgatgccccattccttgatcggcttcgccgagatcag aggtccctaaggggaagaggcaacactc	-	-
CY088402	Human H3N2 IAVs	Human	H3N2		2003	United_Kingdom	A/England/909/2003	agtgatgccccattccttgatcggcttcgccgagatcag aggtccctaaggggaagaggcaacactc	-	-
CY114369	Human H3N2 IAVs	Human	H3N2		2003	Netherlands	A/Netherlands/312/2003	agtgatgccccattccttgatcggcttcgccgagatcag aggtccctaaggggaagaggcaacactc	-	-
CY107094	Human H3N2 IAVs	Human	H3N2		2003	United_Kingdom	A/Scotland/79/2003	agtgatgccccattccttgatcggcttcgccgagatcag aggtccctaaggggaagaggcaacactc	-	-
CY088146	Human H3N2 IAVs	Human	H3N2		2003	United_Kingdom	A/Scotland/79/2003	agtgatgccccattccttgatcggcttcgccgagatcag aggtccctaaggggaagaggcaacactc	-	-
AM502817	Human H3N2 IAVs	Human	H3N2		2003	United_Kingdom	A/Scotland/79/2003	agtgatgccccattccttgatcggcttcgccgagatcag aggtccctaaggggaagaggcaacactc	-	-
CY087986	Human H3N2 IAVs	Human	H3N2		2003	United_Kingdom	A/England/431/2003	agtgatgccccattccttgatcggcttcgccgagatcag aggtccctaaggggaagaggcaacactc	-	-
AM502780	Human H3N2 IAVs	Human	H3N2		2003	United_Kingdom	A/England/431/2003	agtgatgccccattccttgatcggcttcgccgagatcag aggtccctaaggggaagaggcaacactc	-	-
CY112953	Human H3N2 IAVs	Human	H3N2		2003	Netherlands	A/NETHERLANDS/20/2003	agtgatgccccattccttgatcggcttcgccgagatcag aggtccctaaggggaagaggcaacactc	-	-
CY088479	Human H3N2 IAVs	Human	H3N2		2003	United_Kingdom	A/England/4024313/2003	agtgatgccccattccttgatcggcttcgccgagatcag aggtccctaaggggaagaggcaacactc	-	-
AM502807	Human H3N2 IAVs	Human	H3N2		2003	United_Kingdom	A/England/786/2003	agtgatgccccattccttgatcggcttcgccgagatcag aggtccctaaggggaagaggcaacactc	-	-
CY000961	Human H3N2 IAVs	Human	H3N2		2003	USA	A/New_York/59/2003	agtgatgccccattccttgatcggcttcgccgagatcag aggtccctaaggggaagaggcaacactc	-	-
CY091185	Human H3N2 IAVs	Human	H3N2		2003	Australia	A/Australia/NHRC0001/2003	agtgatgccccattccttgatcggcttcgccgagatcag aggtccctaaggggaagaggcaacactc	-	-
CY091305	Human H3N2 IAVs	Human	H3N2		2003	Australia	A/Australia/NHRC0002/2003	agtgatgccccattccttgatcggcttcgccgagatcag aggtccctaaggggaagaggcaacactc	-	-
CY091297	Human H3N2 IAVs	Human	H3N2		2003	Australia	A/Australia/NHRC0003/2003	agtgatgccccattccttgatcggcttcgccgagatcag aggtccctaaggggaagaggcaacactc	-	-

CY091193	Human H3N2 IAVs	Human	H3N2		2003	Australia	A/Australia/NHRC0004/2003	agtgatgccccattccttgatcggcttcgccgagatcag aggtccctaaggggaagaggcaacactc	-	-
CY092229	Human H3N2 IAVs	Human	H3N2		2003	Australia	A/Australia/NHRC0005/2003	agtgatgccccattccttgatcggcttcgccgagatcag aggtccctaaggggaagaggcaacactc	-	-
CY091289	Human H3N2 IAVs	Human	H3N2		2003	Australia	A/Australia/NHRC0006/2003	agtgatgccccattccttgatcggcttcgccgagatcag aggtccctaaggggaagaggcaacactc	-	-
CY091201	Human H3N2 IAVs	Human	H3N2		2003	Australia	A/Australia/NHRC0007/2003	agtgatgccccattccttgatcggcttcgccgagatcag aggtccctaaggggaagaggcaacactc	-	-
CY091281	Human H3N2 IAVs	Human	H3N2		2003	Australia	A/Australia/NHRC0008/2003	agtgatgccccattccttgatcggcttcgccgagatcag aggtccctaaggggaagaggcaacactc	-	-
CY091273	Human H3N2 IAVs	Human	H3N2		2003	Australia	A/Australia/NHRC0009/2003	agtgatgccccattccttgatcggcttcgccgagatcag aggtccctaaggggaagaggcaacactc	-	-
CY091209	Human H3N2 IAVs	Human	H3N2		2003	Australia	A/Australia/NHRC0010/2003	agtgatgccccattccttgatcggcttcgccgagatcag aggtccctaaggggaagaggcaacactc	-	-
CY090945	Human H3N2 IAVs	Human	H3N2		2003	USA	A/California/NHRC0001/2003	agtgatgccccattccttgatcggcttcgccgagatcag aggtccctaaggggaagaggcaacactc	-	-
CY006967	Human H3N2 IAVs	Human	H3N2		2003	New_Zealand	A/Canterbury/391/2003	agtgatgccccattccttgatcggcttcgccgagatcag aggtccctaaggggaagaggcaacactc	-	-
CY007023	Human H3N2 IAVs	Human	H3N2		2003	New_Zealand	A/Canterbury/399/2003	agtgatgccccattccttgatcggcttcgccgagatcag aggtccctaaggggaagaggcaacactc	-	-
CY009024	Human H3N2 IAVs	Human	H3N2		2003	New_Zealand	A/Canterbury/400/2003	agtgatgccccattccttgatcggcttcgccgagatcag aggtccctaaggggaagaggcaacactc	-	-
CY007031	Human H3N2 IAVs	Human	H3N2		2003	New_Zealand	A/Canterbury/401/2003	agtgatgccccattccttgatcggcttcgccgagatcag aggtccctaaggggaagaggcaacactc	-	-
CY007055	Human H3N2 IAVs	Human	H3N2		2003	New_Zealand	A/Canterbury/405/2003	agtgatgccccattccttgatcggcttcgccgagatcag aggtccctaaggggaagaggcaacactc	-	-
CY007151	Human H3N2 IAVs	Human	H3N2		2003	New_Zealand	A/Canterbury/427/2003	agtgatgccccattccttgatcggcttcgccgagatcag aggtccctaaggggaagaggcaacactc	-	-
CY007831	Human H3N2 IAVs	Human	H3N2		2003	New_Zealand	A/Canterbury/429/2003	agtgatgccccattccttgatcggcttcgccgagatcag aggtccctaaggggaagaggcaacactc	-	-
CY007167	Human H3N2 IAVs	Human	H3N2		2003	New_Zealand	A/Canterbury/430/2003	agtgatgccccattccttgatcggcttcgccgagatcag aggtccctaaggggaagaggcaacactc	-	-
CY008200	Human H3N2 IAVs	Human	H3N2		2003	New_Zealand	A/Canterbury/432/2003	agtgatgccccattccttgatcggcttcgccgagatcag aggtccctaaggggaagaggcaacactc	-	-
CY088026	Human H3N2 IAVs	Human	H3N2		2003	United_Kingdom	A/England/442/2003	agtgatgccccattccttgatcggcttcgccgagatcag aggtccctaaggggaagaggcaacactc	-	-
CY088058	Human H3N2 IAVs	Human	H3N2		2003	United_Kingdom	A/England/445/2003	agtgatgccccattccttgatcggcttcgccgagatcag aggtccctaaggggaagaggcaacactc	-	-
CY088002	Human H3N2 IAVs	Human	H3N2		2003	United_Kingdom	A/England/446/2003	agtgatgccccattccttgatcggcttcgccgagatcag aggtccctaaggggaagaggcaacactc	-	-
CY088162	Human H3N2 IAVs	Human	H3N2		2003	United_Kingdom	A/England/470/2003	agtgatgccccattccttgatcggcttcgccgagatcag aggtccctaaggggaagaggcaacactc	-	-
CY107102	Human H3N2 IAVs	Human	H3N2		2003	United_Kingdom	A/England/470/2003	agtgatgccccattccttgatcggcttcgccgagatcag aggtccctaaggggaagaggcaacactc	-	-
AM502781	Human H3N2 IAVs	Human	H3N2		2003	United_Kingdom	A/England/470/2003	agtgatgccccattccttgatcggcttcgccgagatcag aggtccctaaggggaagaggcaacactc	-	-
CY088186	Human H3N2 IAVs	Human	H3N2		2003	United_Kingdom	A/England/479/2003	agtgatgccccattccttgatcggcttcgccgagatcag aggtccctaaggggaagaggcaacactc	-	-
AM502783	Human H3N2 IAVs	Human	H3N2		2003	United_Kingdom	A/England/479/2003	agtgatgccccattccttgatcggcttcgccgagatcag aggtccctaaggggaagaggcaacactc	-	-

CY088306	Human H3N2 IAVs	Human	H3N2		2003	United_Kingdom	A/England/493/2003	agtgatgccccattccttgatcggcttcgccgagatcag aggtccctaaggggaagaggcaacactc	-	-
CY107158	Human H3N2 IAVs	Human	H3N2		2003	United_Kingdom	A/England/493/2003	agtgatgccccattccttgatcggcttcgccgagatcag aggtccctaaggggaagaggcaacactc	-	-
AM502788	Human H3N2 IAVs	Human	H3N2		2003	United_Kingdom	A/England/493/2003	agtgatgccccattccttgatcggcttcgccgagatcag aggtccctaaggggaagaggcaacactc	-	-
AM502791	Human H3N2 IAVs	Human	H3N2		2003	United_Kingdom	A/England/520/2003	agtgatgccccattccttgatcggcttcgccgagatcag aggtccctaaggggaagaggcaacactc	-	-
CY088226	Human H3N2 IAVs	Human	H3N2		2003	United_Kingdom	A/England/520/2003	agtgatgccccattccttgatcggcttcgccgagatcag aggtccctaaggggaagaggcaacactc	-	-
CY088394	Human H3N2 IAVs	Human	H3N2		2003	United_Kingdom	A/England/522/2003	agtgatgccccattccttgatcggcttcgccgagatcag aggtccctaaggggaagaggcaacactc	-	-
CY088258	Human H3N2 IAVs	Human	H3N2		2003	United_Kingdom	A/England/525/2003	agtgatgccccattccttgatcggcttcgccgagatcag aggtccctaaggggaagaggcaacactc	-	-
AM502794	Human H3N2 IAVs	Human	H3N2		2003	United_Kingdom	A/England/532/2003	agtgatgccccattccttgatcggcttcgccgagatcag aggtccctaaggggaagaggcaacactc	-	-
AM502795	Human H3N2 IAVs	Human	H3N2		2003	United_Kingdom	A/England/534/2003	agtgatgccccattccttgatcggcttcgccgagatcag aggtccctaaggggaagaggcaacactc	-	-
CY088418	Human H3N2 IAVs	Human	H3N2		2003	United_Kingdom	A/England/552/2003	agtgatgccccattccttgatcggcttcgccgagatcag aggtccctaaggggaagaggcaacactc	-	-
CY088074	Human H3N2 IAVs	Human	H3N2		2003	United_Kingdom	A/England/642/2003	agtgatgccccattccttgatcggcttcgccgagatcag aggtccctaaggggaagaggcaacactc	-	-
CY088066	Human H3N2 IAVs	Human	H3N2		2003	United_Kingdom	A/England/740/2003	agtgatgccccattccttgatcggcttcgccgagatcag aggtccctaaggggaagaggcaacactc	-	-
AM502802	Human H3N2 IAVs	Human	H3N2		2003	United_Kingdom	A/England/740/2003	agtgatgccccattccttgatcggcttcgccgagatcag aggtccctaaggggaagaggcaacactc	-	-
AM502803	Human H3N2 IAVs	Human	H3N2		2003	United_Kingdom	A/England/741/2003	agtgatgccccattccttgatcggcttcgccgagatcag aggtccctaaggggaagaggcaacactc	-	-
AM502804	Human H3N2 IAVs	Human	H3N2		2003	United_Kingdom	A/England/752/2003	agtgatgccccattccttgatcggcttcgccgagatcag aggtccctaaggggaagaggcaacactc	-	-
AM502805	Human H3N2 IAVs	Human	H3N2		2003	United_Kingdom	A/England/753/2003	agtgatgccccattccttgatcggcttcgccgagatcag aggtccctaaggggaagaggcaacactc	-	-
CY088242	Human H3N2 IAVs	Human	H3N2		2003	United_Kingdom	A/England/788/2003	agtgatgccccattccttgatcggcttcgccgagatcag aggtccctaaggggaagaggcaacactc	-	-
CY006927	Human H3N2 IAVs	Human	H3N2		2003	New_Zealand	A/Canterbury/382/2003	agtgatgccccattccttgatcggcttcgccgagatcag aggtccctaaggggaagaggcaacactc	-	-
CY006935	Human H3N2 IAVs	Human	H3N2		2003	New_Zealand	A/Canterbury/384/2003	agtgatgccccattccttgatcggcttcgccgagatcag aggtccctaaggggaagaggcaacactc	-	-
CY006943	Human H3N2 IAVs	Human	H3N2		2003	New_Zealand	A/Canterbury/386/2003	agtgatgccccattccttgatcggcttcgccgagatcag aggtccctaaggggaagaggcaacactc	-	-
CY006951	Human H3N2 IAVs	Human	H3N2		2003	New_Zealand	A/Canterbury/387/2003	agtgatgccccattccttgatcggcttcgccgagatcag aggtccctaaggggaagaggcaacactc	-	-
CY006959	Human H3N2 IAVs	Human	H3N2		2003	New_Zealand	A/Canterbury/390/2003	agtgatgccccattccttgatcggcttcgccgagatcag aggtccctaaggggaagaggcaacactc	-	-
CY006983	Human H3N2 IAVs	Human	H3N2		2003	New_Zealand	A/Canterbury/393/2003	agtgatgccccattccttgatcggcttcgccgagatcag aggtccctaaggggaagaggcaacactc	-	-
CY006991	Human H3N2 IAVs	Human	H3N2		2003	New_Zealand	A/Canterbury/394/2003	agtgatgccccattccttgatcggcttcgccgagatcag aggtccctaaggggaagaggcaacactc	-	-
CY006999	Human H3N2 IAVs	Human	H3N2		2003	New_Zealand	A/Canterbury/395/2003	agtgatgccccattccttgatcggcttcgccgagatcag aggtccctaaggggaagaggcaacactc	-	-

CY007007	Human H3N2 IAVs	Human	H3N2		2003	New_Zealand	A/Canterbury/397/2003	agtgatgccccattccttgatcggcttcgccgagatcag<br>aggtccctaaggggaagaggcaacactc	-	-
CY007047	Human H3N2 IAVs	Human	H3N2		2003	New_Zealand	A/Canterbury/404/2003	agtgatgccccattccttgatcggcttcgccgagatcag<br>aggtccctaaggggaagaggcaacactc	-	-
CY007063	Human H3N2 IAVs	Human	H3N2		2003	New_Zealand	A/Canterbury/406/2003	agtgatgccccattccttgatcggcttcgccgagatcag<br>aggtccctaaggggaagaggcaacactc	-	-
CY007071	Human H3N2 IAVs	Human	H3N2		2003	New_Zealand	A/Canterbury/408/2003	agtgatgccccattccttgatcggcttcgccgagatcag<br>aggtccctaaggggaagaggcaacactc	-	-
CY007823	Human H3N2 IAVs	Human	H3N2		2003	New_Zealand	A/Canterbury/409/2003	agtgatgccccattccttgatcggcttcgccgagatcag<br>aggtccctaaggggaagaggcaacactc	-	-
CY007079	Human H3N2 IAVs	Human	H3N2		2003	New_Zealand	A/Canterbury/410/2003	agtgatgccccattccttgatcggcttcgccgagatcag<br>aggtccctaaggggaagaggcaacactc	-	-
CY007087	Human H3N2 IAVs	Human	H3N2		2003	New_Zealand	A/Canterbury/411/2003	agtgatgccccattccttgatcggcttcgccgagatcag<br>aggtccctaaggggaagaggcaacactc	-	-
CY007103	Human H3N2 IAVs	Human	H3N2		2003	New_Zealand	A/Canterbury/416/2003	agtgatgccccattccttgatcggcttcgccgagatcag<br>aggtccctaaggggaagaggcaacactc	-	-
CY007111	Human H3N2 IAVs	Human	H3N2		2003	New_Zealand	A/Canterbury/417/2003	agtgatgccccattccttgatcggcttcgccgagatcag<br>aggtccctaaggggaagaggcaacactc	-	-
CY007119	Human H3N2 IAVs	Human	H3N2		2003	New_Zealand	A/Canterbury/418/2003	agtgatgccccattccttgatcggcttcgccgagatcag<br>aggtccctaaggggaagaggcaacactc	-	-
CY007127	Human H3N2 IAVs	Human	H3N2		2003	New_Zealand	A/Canterbury/420/2003	agtgatgccccattccttgatcggcttcgccgagatcag<br>aggtccctaaggggaagaggcaacactc	-	-
CY007135	Human H3N2 IAVs	Human	H3N2		2003	New_Zealand	A/Canterbury/423/2003	agtgatgccccattccttgatcggcttcgccgagatcag<br>aggtccctaaggggaagaggcaacactc	-	-
CY007159	Human H3N2 IAVs	Human	H3N2		2003	New_Zealand	A/Canterbury/428/2003	agtgatgccccattccttgatcggcttcgccgagatcag<br>aggtccctaaggggaagaggcaacactc	-	-
CY007175	Human H3N2 IAVs	Human	H3N2		2003	New_Zealand	A/Canterbury/431/2003	agtgatgccccattccttgatcggcttcgccgagatcag<br>aggtccctaaggggaagaggcaacactc	-	-
CY007191	Human H3N2 IAVs	Human	H3N2		2003	New_Zealand	A/Canterbury/434/2003	agtgatgccccattccttgatcggcttcgccgagatcag<br>aggtccctaaggggaagaggcaacactc	-	-
CY007207	Human H3N2 IAVs	Human	H3N2		2003	New_Zealand	A/Canterbury/436/2003	agtgatgccccattccttgatcggcttcgccgagatcag<br>aggtccctaaggggaagaggcaacactc	-	-
CY007215	Human H3N2 IAVs	Human	H3N2		2003	New_Zealand	A/Canterbury/437/2003	agtgatgccccattccttgatcggcttcgccgagatcag<br>aggtccctaaggggaagaggcaacactc	-	-
CY007223	Human H3N2 IAVs	Human	H3N2		2003	New_Zealand	A/Canterbury/438/2003	agtgatgccccattccttgatcggcttcgccgagatcag<br>aggtccctaaggggaagaggcaacactc	-	-
CY007247	Human H3N2 IAVs	Human	H3N2		2003	New_Zealand	A/Canterbury/441/2003	agtgatgccccattccttgatcggcttcgccgagatcag<br>aggtccctaaggggaagaggcaacactc	-	-
CY007255	Human H3N2 IAVs	Human	H3N2		2003	New_Zealand	A/Canterbury/442/2003	agtgatgccccattccttgatcggcttcgccgagatcag<br>aggtccctaaggggaagaggcaacactc	-	-
CY007263	Human H3N2 IAVs	Human	H3N2		2003	New_Zealand	A/Canterbury/443/2003	agtgatgccccattccttgatcggcttcgccgagatcag<br>aggtccctaaggggaagaggcaacactc	-	-
CY007271	Human H3N2 IAVs	Human	H3N2		2003	New_Zealand	A/Canterbury/444/2003	agtgatgccccattccttgatcggcttcgccgagatcag<br>aggtccctaaggggaagaggcaacactc	-	-
CY013449	Human H3N2 IAVs	Human	H3N2		2003	New_Zealand	A/Dunedin/38/2003	agtgatgccccattccttgatcggcttcgccgagatcag<br>aggtccctaaggggaagaggcaacactc	-	-
CY107126	Human H3N2 IAVs	Human	H3N2		2003	United_Kingdom	A/England/788/2003	agtgatgccccattccttgatcggcttcgccgagatcag<br>aggtccctaaggggaagaggcaacactc	-	-
AM502809	Human H3N2 IAVs	Human	H3N2		2003	United_Kingdom	A/England/788/2003	agtgatgccccattccttgatcggcttcgccgagatcag<br>aggtccctaaggggaagaggcaacactc	-	-

CY000905	Human H3N2 IAVs	Human	H3N2		2003	USA	A/New_York/13/2003	agtgatgccccattccttgatcggcttcgccgagatcag<br>aggtccctaaggggaagaggcaacactc	-	-
CY001193	Human H3N2 IAVs	Human	H3N2		2003	USA	A/New_York/18/2003	agtgatgccccattccttgatcggcttcgccgagatcag<br>aggtccctaaggggaagaggcaacactc	-	-
CY000253	Human H3N2 IAVs	Human	H3N2		2003	USA	A/New_York/19/2003	agtgatgccccattccttgatcggcttcgccgagatcag<br>aggtccctaaggggaagaggcaacactc	-	-
CY000477	Human H3N2 IAVs	Human	H3N2		2003	USA	A/New_York/2/2003	agtgatgccccattccttgatcggcttcgccgagatcag<br>aggtccctaaggggaagaggcaacactc	-	-
CY000197	Human H3N2 IAVs	Human	H3N2		2003	USA	A/New_York/23/2003	agtgatgccccattccttgatcggcttcgccgagatcag<br>aggtccctaaggggaagaggcaacactc	-	-
CY001652	Human H3N2 IAVs	Human	H3N2		2003	USA	A/New_York/270/2003	agtgatgccccattccttgatcggcttcgccgagatcag<br>aggtccctaaggggaagaggcaacactc	-	-
CY001716	Human H3N2 IAVs	Human	H3N2		2003	USA	A/New_York/271/2003	agtgatgccccattccttgatcggcttcgccgagatcag<br>aggtccctaaggggaagaggcaacactc	-	-
CY002348	Human H3N2 IAVs	Human	H3N2		2003	USA	A/New_York/272/2003	agtgatgccccattccttgatcggcttcgccgagatcag<br>aggtccctaaggggaagaggcaacactc	-	-
CY000021	Human H3N2 IAVs	Human	H3N2		2003	USA	A/New_York/30/2003	agtgatgccccattccttgatcggcttcgccgagatcag<br>aggtccctaaggggaagaggcaacactc	-	-
CY000053	Human H3N2 IAVs	Human	H3N2		2003	USA	A/New_York/35/2003	agtgatgccccattccttgatcggcttcgccgagatcag<br>aggtccctaaggggaagaggcaacactc	-	-
CY000061	Human H3N2 IAVs	Human	H3N2		2003	USA	A/New_York/36/2003	agtgatgccccattccttgatcggcttcgccgagatcag<br>aggtccctaaggggaagaggcaacactc	-	-
CY001297	Human H3N2 IAVs	Human	H3N2		2003	USA	A/New_York/37/2003	agtgatgccccattccttgatcggcttcgccgagatcag<br>aggtccctaaggggaagaggcaacactc	-	-
CY000165	Human H3N2 IAVs	Human	H3N2		2003	USA	A/New_York/42/2003	agtgatgccccattccttgatcggcttcgccgagatcag<br>aggtccctaaggggaagaggcaacactc	-	-
CY008872	Human H3N2 IAVs	Human	H3N2		2003	USA	A/New_York/473/2003	agtgatgccccattccttgatcggcttcgccgagatcag<br>aggtccctaaggggaagaggcaacactc	-	-
CY008888	Human H3N2 IAVs	Human	H3N2		2003	USA	A/New_York/475/2003	agtgatgccccattccttgatcggcttcgccgagatcag<br>aggtccctaaggggaagaggcaacactc	-	-
CY008920	Human H3N2 IAVs	Human	H3N2		2003	USA	A/New_York/479/2003	agtgatgccccattccttgatcggcttcgccgagatcag<br>aggtccctaaggggaagaggcaacactc	-	-
CY000093	Human H3N2 IAVs	Human	H3N2		2003	USA	A/New_York/50/2003	agtgatgccccattccttgatcggcttcgccgagatcag<br>aggtccctaaggggaagaggcaacactc	-	-
CY000101	Human H3N2 IAVs	Human	H3N2		2003	USA	A/New_York/54/2003	agtgatgccccattccttgatcggcttcgccgagatcag<br>aggtccctaaggggaagaggcaacactc	-	-
CY000109	Human H3N2 IAVs	Human	H3N2		2003	USA	A/New_York/60A/2003	agtgatgccccattccttgatcggcttcgccgagatcag<br>aggtccctaaggggaagaggcaacactc	-	-
CY000921	Human H3N2 IAVs	Human	H3N2		2003	USA	A/New_York/62A/2003	agtgatgccccattccttgatcggcttcgccgagatcag<br>aggtccctaaggggaagaggcaacactc	-	-
CY000969	Human H3N2 IAVs	Human	H3N2		2003	USA	A/New_York/64/2003	agtgatgccccattccttgatcggcttcgccgagatcag<br>aggtccctaaggggaagaggcaacactc	-	-
CY001041	Human H3N2 IAVs	Human	H3N2		2003	USA	A/New_York/7/2003	agtgatgccccattccttgatcggcttcgccgagatcag<br>aggtccctaaggggaagaggcaacactc	-	-
CY088010	Human H3N2 IAVs	Human	H3N2		2003	United_Kingdom	A/Scotland/50/2003	agtgatgccccattccttgatcggcttcgccgagatcag<br>aggtccctaaggggaagaggcaacactc	-	-
AM502814	Human H3N2 IAVs	Human	H3N2		2003	United_Kingdom	A/Scotland/50/2003	agtgatgccccattccttgatcggcttcgccgagatcag<br>aggtccctaaggggaagaggcaacactc	-	-
CY088098	Human H3N2 IAVs	Human	H3N2		2003	United_Kingdom	A/Scotland/71/2003	agtgatgccccattccttgatcggcttcgccgagatcag<br>aggtccctaaggggaagaggcaacactc	-	-

CY007183	Human H3N2 IAVs	Human	H3N2		2003	New_Zealand	A/South_Canterbury/433/2003	agtgatgccccattccttgatcggcttcgccgagatcag<br>aggtccctaaggggaagaggcaacactc	-	-
CY013441	Human H3N2 IAVs	Human	H3N2		2003	New_Zealand	A/Waikato/108/2003	agtgatgccccattccttgatcggcttcgccgagatcag<br>aggtccctaaggggaagaggcaacactc	-	-
CY012372	Human H3N2 IAVs	Human	H3N2		2003	New_Zealand	A/Waikato/115/2003	agtgatgccccattccttgatcggcttcgccgagatcag<br>aggtccctaaggggaagaggcaacactc	-	-
CY011772	Human H3N2 IAVs	Human	H3N2		2003	New_Zealand	A/Waikato/120/2003	agtgatgccccattccttgatcggcttcgccgagatcag<br>aggtccctaaggggaagaggcaacactc	-	-
CY012388	Human H3N2 IAVs	Human	H3N2		2003	New_Zealand	A/Waikato/122/2003	agtgatgccccattccttgatcggcttcgccgagatcag<br>aggtccctaaggggaagaggcaacactc	-	-
CY012396	Human H3N2 IAVs	Human	H3N2		2003	New_Zealand	A/Waikato/129/2003	agtgatgccccattccttgatcggcttcgccgagatcag<br>aggtccctaaggggaagaggcaacactc	-	-
CY012404	Human H3N2 IAVs	Human	H3N2		2003	New_Zealand	A/Waikato/133/2003	agtgatgccccattccttgatcggcttcgccgagatcag<br>aggtccctaaggggaagaggcaacactc	-	-
CY012412	Human H3N2 IAVs	Human	H3N2		2003	New_Zealand	A/Waikato/139/2003	agtgatgccccattccttgatcggcttcgccgagatcag<br>aggtccctaaggggaagaggcaacactc	-	-
CY012420	Human H3N2 IAVs	Human	H3N2		2003	New_Zealand	A/Waikato/147/2003	agtgatgccccattccttgatcggcttcgccgagatcag<br>aggtccctaaggggaagaggcaacactc	-	-
CY011732	Human H3N2 IAVs	Human	H3N2		2003	New_Zealand	A/Waikato/3/2003	agtgatgccccattccttgatcggcttcgccgagatcag<br>aggtccctaaggggaagaggcaacactc	-	-
CY013140	Human H3N2 IAVs	Human	H3N2		2003	New_Zealand	A/Waikato/54/2003	agtgatgccccattccttgatcggcttcgccgagatcag<br>aggtccctaaggggaagaggcaacactc	-	-
CY012084	Human H3N2 IAVs	Human	H3N2		2003	New_Zealand	A/Waikato/91/2003	agtgatgccccattccttgatcggcttcgccgagatcag<br>aggtccctaaggggaagaggcaacactc	-	-
CY011724	Human H3N2 IAVs	Human	H3N2		2003	New_Zealand	A/Wellington/10/2003	agtgatgccccattccttgatcggcttcgccgagatcag<br>aggtccctaaggggaagaggcaacactc	-	-
CY011700	Human H3N2 IAVs	Human	H3N2		2003	New_Zealand	A/Wellington/03/2003	agtgatgccccattccttgatcggcttcgccgagatcag<br>aggtccctaaggggaagaggcaacactc	-	-
CY011708	Human H3N2 IAVs	Human	H3N2		2003	New_Zealand	A/Wellington/4/2003	agtgatgccccattccttgatcggcttcgccgagatcag<br>aggtccctaaggggaagaggcaacactc	-	-
CY012652	Human H3N2 IAVs	Human	H3N2		2003	New_Zealand	A/Wellington/9/2003	agtgatgccccattccttgatcggcttcgccgagatcag<br>aggtccctaaggggaagaggcaacactc	-	-
CY092213	Human H3N2 IAVs	Human	H3N2		2003	USA	A/Georgia/NHRC0001/2003	agtgatgccccattccttgatcggcttcgccgagatcag<br>aggtccctaaggggaagaggcaacactc	-	-
CY092205	Human H3N2 IAVs	Human	H3N2		2003	USA	A/Illinois/NHRC0001/2003	agtgatgccccattccttgatcggcttcgccgagatcag<br>aggtccctaaggggaagaggcaacactc	-	-
CY090937	Human H3N2 IAVs	Human	H3N2		2003	USA	A/Missouri/NHRC0001/2003	agtgatgccccattccttgatcggcttcgccgagatcag<br>aggtccctaaggggaagaggcaacactc	-	-
CY018977	Human H3N2 IAVs	Human	H3N2		2003	Australia	A/Queensland/31/2003	agtgatgccccattccttgatcggcttcgccgagatcag<br>aggtccctaaggggaagaggcaacactc	-	-
CY018985	Human H3N2 IAVs	Human	H3N2		2003	Australia	A/Queensland/32/2003	agtgatgccccattccttgatcggcttcgccgagatcag<br>aggtccctaaggggaagaggcaacactc	-	-
CY017567	Human H3N2 IAVs	Human	H3N2		2003	Australia	A/Queensland/33/2003	agtgatgccccattccttgatcggcttcgccgagatcag<br>aggtccctaaggggaagaggcaacactc	-	-
CY019001	Human H3N2 IAVs	Human	H3N2		2003	Australia	A/Queensland/35/2003	agtgatgccccattccttgatcggcttcgccgagatcag<br>aggtccctaaggggaagaggcaacactc	-	-
CY017575	Human H3N2 IAVs	Human	H3N2		2003	Australia	A/Queensland/36/2003	agtgatgccccattccttgatcggcttcgccgagatcag<br>aggtccctaaggggaagaggcaacactc	-	-
CY017953	Human H3N2 IAVs	Human	H3N2		2003	Australia	A/Queensland/40/2003	agtgatgccccattccttgatcggcttcgccgagatcag<br>aggtccctaaggggaagaggcaacactc	-	-

CY013433	Human H3N2 IAVs	Human	H3N2		2003	New_Zealand	A/Waikato/15/2003	agtgatgccccattccttgatcggcttcgccgagatcag aggtccctaaggggaagaggcaacactc	-	-
CY012100	Human H3N2 IAVs	Human	H3N2		2003	New_Zealand	A/Waikato/150/2003	agtgatgccccattccttgatcggcttcgccgagatcag aggtccctaaggggaagaggcaacactc	-	-
CY012348	Human H3N2 IAVs	Human	H3N2		2003	New_Zealand	A/Waikato/155/2003	agtgatgccccattccttgatcggcttcgccgagatcag aggtccctaaggggaagaggcaacactc	-	-
CY012356	Human H3N2 IAVs	Human	H3N2		2003	New_Zealand	A/Waikato/156/2003	agtgatgccccattccttgatcggcttcgccgagatcag aggtccctaaggggaagaggcaacactc	-	-
CY013116	Human H3N2 IAVs	Human	H3N2		2003	New_Zealand	A/Waikato/21/2003	agtgatgccccattccttgatcggcttcgccgagatcag aggtccctaaggggaagaggcaacactc	-	-
CY012364	Human H3N2 IAVs	Human	H3N2		2003	New_Zealand	A/Waikato/29/2003	agtgatgccccattccttgatcggcttcgccgagatcag aggtccctaaggggaagaggcaacactc	-	-
CY013124	Human H3N2 IAVs	Human	H3N2		2003	New_Zealand	A/Waikato/46/2003	agtgatgccccattccttgatcggcttcgccgagatcag aggtccctaaggggaagaggcaacactc	-	-
CY013148	Human H3N2 IAVs	Human	H3N2		2003	New_Zealand	A/Waikato/94/2003	agtgatgccccattccttgatcggcttcgccgagatcag aggtccctaaggggaagaggcaacactc	-	-
CY012660	Human H3N2 IAVs	Human	H3N2		2003	New_Zealand	A/Wellington/47/2003	agtgatgccccattccttgatcggcttcgccgagatcag aggtccctaaggggaagaggcaacactc	-	-
CY015784	Human H3N2 IAVs	Human	H3N2		2003	Australia	A/Western_Australia/38/2003	agtgatgccccattccttgatcggcttcgccgagatcag aggtccctaaggggaagaggcaacactc	-	-
CY015792	Human H3N2 IAVs	Human	H3N2		2003	Australia	A/Western_Australia/39/2003	agtgatgccccattccttgatcggcttcgccgagatcag aggtccctaaggggaagaggcaacactc	-	-
CY015808	Human H3N2 IAVs	Human	H3N2		2003	Australia	A/Western_Australia/41/2003	agtgatgccccattccttgatcggcttcgccgagatcag aggtccctaaggggaagaggcaacactc	-	-
CY015816	Human H3N2 IAVs	Human	H3N2		2003	Australia	A/Western_Australia/42/2003	agtgatgccccattccttgatcggcttcgccgagatcag aggtccctaaggggaagaggcaacactc	-	-
CY015824	Human H3N2 IAVs	Human	H3N2		2003	Australia	A/Western_Australia/43/2003	agtgatgccccattccttgatcggcttcgccgagatcag aggtccctaaggggaagaggcaacactc	-	-
CY015864	Human H3N2 IAVs	Human	H3N2		2003	Australia	A/Western_Australia/48/2003	agtgatgccccattccttgatcggcttcgccgagatcag aggtccctaaggggaagaggcaacactc	-	-
CY017801	Human H3N2 IAVs	Human	H3N2		2003	Australia	A/Western_Australia/49/2003	agtgatgccccattccttgatcggcttcgccgagatcag aggtccctaaggggaagaggcaacactc	-	-
CY012076	Human H3N2 IAVs	Human	H3N2		2003	New_Zealand	A/Waikato/154/2003	agtgatgccccattccttgatcggcttcgccgagatcag aggtccctaaggggaagaggcaacactc	-	-
CY011764	Human H3N2 IAVs	Human	H3N2		2003	New_Zealand	A/Waikato/75/2003	agtgatgccccattccttgatcggcttcgccgagatcag aggtccctaaggggaagaggcaacactc	-	-
CY001289	Human H3N2 IAVs	Human	H3N2		2003	USA	A/New_York/9/2003	agtgatgccccattccttgatcggcttcgccgagatcag aggtccctaaggggaagaggcaacactc	-	-
CY000977	Human H3N2 IAVs	Human	H3N2		2003	USA	A/New_York/67/2003	agtgatgccccattccttgatcggcttcgccgagatcag aggtccctaaggggaagaggcaacactc	-	-
CY088453	Human H3N2 IAVs	Human	H3N2		2003	United_Kingdom	A/England/592/2003	agtgatgccccattccttgatcggcttcgccgagatcag aggtccctaaggggaagaggcaacactc	-	-
CY000173	Human H3N2 IAVs	Human	H3N2		2003	USA	A/New_York/43/2003	agtgatgccccattccttgatcggcttcgccgagatcag aggtccctaaggggaagaggcaacactc	-	-
CY001217	Human H3N2 IAVs	Human	H3N2		2003	USA	A/New_York/63/2003	agtgatgccccattccttgatcggcttcgccgagatcag aggtccctaaggggaagaggcaacactc	-	-
CY088106	Human H3N2 IAVs	Human	H3N2		2003	United_Kingdom	A/Scotland/72/2003	agtgatgccccattccttgatcggcttcgccgagatcag aggtccctaaggggaagaggcaacactc	-	-
CY088354	Human H3N2 IAVs	Human	H3N2		2003	United_Kingdom	A/England/567/2003	agtgatgccccattccttgatcggcttcgccgagatcag aggtccctaaggggaagaggcaacactc	-	-

CY088050	Human H3N2 IAVs	Human	H3N2		2003	United_Kingdom	A/Scotland/61/2003	agtgatgccccattccttgatcggcttcgccgagatcag aggtccctaaggggaagaggcaacactc	-	-
CY012428	Human H3N2 IAVs	Human	H3N2		2003	New_Zealand	A/Waikato/148/2003	agtgatgccccattccttgatcggcttcgccgagatcag aggtccctaaggggaagaggcaacactc	-	-
CY001068	Human H3N2 IAVs	Human	H3N2		2003	USA	A/New_York/51/2003	agtgatgccccattccttgatcggcttcgccgagatcag aggtccctaaggggaagaggcaacactc	-	-
AM502782	Human H3N2 IAVs	Human	H3N2		2003	United_Kingdom	A/England/476/2003	agtgatgccccattccttgatcggcttcgccgagatcag aggtccctaaggggaagaggcaacactc	-	-
CY107142	Human H3N2 IAVs	Human	H3N2		2003	United_Kingdom	A/England/492/2003	agtgatgccccattccttgatcggcttcgccgagatcag aggtccctaaggggaagaggcaacactc	-	-
CY088290	Human H3N2 IAVs	Human	H3N2		2003	United_Kingdom	A/England/492/2003	agtgatgccccattccttgatcggcttcgccgagatcag aggtccctaaggggaagaggcaacactc	-	-
AM502787	Human H3N2 IAVs	Human	H3N2		2003	United_Kingdom	A/England/492/2003	agtgatgccccattccttgatcggcttcgccgagatcag aggtccctaaggggaagaggcaacactc	-	-
CY088442	Human H3N2 IAVs	Human	H3N2		2003	United_Kingdom	A/England/584/2003	agtgatgccccattccttgatcggcttcgccgagatcag aggtccctaaggggaagaggcaacactc	-	-
CY107182	Human H3N2 IAVs	Human	H3N2		2003	United_Kingdom	A/England/584/2003	agtgatgccccattccttgatcggcttcgccgagatcag aggtccctaaggggaagaggcaacactc	-	-
AM502799	Human H3N2 IAVs	Human	H3N2		2003	United_Kingdom	A/England/584/2003	agtgatgccccattccttgatcggcttcgccgagatcag aggtccctaaggggaagaggcaacactc	-	-
CY088274	Human H3N2 IAVs	Human	H3N2		2003	United_Kingdom	A/England/585/2003	agtgatgccccattccttgatcggcttcgccgagatcag aggtccctaaggggaagaggcaacactc	-	-
AM502800	Human H3N2 IAVs	Human	H3N2		2003	United_Kingdom	A/England/585/2003	agtgatgccccattccttgatcggcttcgccgagatcag aggtccctaaggggaagaggcaacactc	-	-
CY001057	Human H3N2 IAVs	Human	H3N2		2003	USA	A/New_York/17/2003	agtgatgccccattccttgatcggcttcgccgagatcag aggtccctaaggggaagaggcaacactc	-	-
CY000069	Human H3N2 IAVs	Human	H3N2		2003	USA	A/New_York/45/2003	agtgatgccccattccttgatcggcttcgccgagatcag aggtccctaaggggaagaggcaacactc	-	-
CY000005	Human H3N2 IAVs	Human	H3N2		2003	USA	A/New_York/61A/2003	agtgatgccccattccttgatcggcttcgccgagatcag aggtccctaaggggaagaggcaacactc	-	-
CY018993	Human H3N2 IAVs	Human	H3N2		2003	Australia	A/Queensland/34/2003	agtgatgccccattccttgatcggcttcgccgagatcag aggtccctaaggggaagaggcaacactc	-	-
CY001425	Human H3N2 IAVs	Human	H3N2		2003	USA	A/New_York/20/2003	agtgatgccccattccttgatcggcttcgccgagatcag aggtccctaaggggaagaggcaacactc	-	-
CY000516	Human H3N2 IAVs	Human	H3N2		2003	USA	A/New_York/11/2003	agtgatgccccattccttgatcggcttcgccgagatcag aggtccctaaggggaagaggcaacactc	-	-
CY001345	Human H3N2 IAVs	Human	H3N2		2003	USA	A/New_York/66/2003	agtgatgccccattccttgatcggcttcgccgagatcag aggtccctaaggggaagaggcaacactc	-	-
CY007095	Human H3N2 IAVs	Human	H3N2		2003	New_Zealand	A/Canterbury/412/2003	agtgatgccccattccttgatcggcttcgccgagatcag aggtccctaaggggaagaggcaacactc	-	-
CY000013	Human H3N2 IAVs	Human	H3N2		2003	USA	A/New_York/28/2003	agtgatgccccattccttgatcggcttcgccgagatcag aggtccctaaggggaagaggcaacactc	-	-
CY000885	Human H3N2 IAVs	Human	H3N2		2003	USA	A/New_York/3/2003	agtgatgccccattccttgatcggcttcgccgagatcag aggtccctaaggggaagaggcaacactc	-	-
CY008904	Human H3N2 IAVs	Human	H3N2		2003	USA	A/New_York/478/2003	agtgatgccccattccttgatcggcttcgccgagatcag aggtccctaaggggaagaggcaacactc	-	-
CY008880	Human H3N2 IAVs	Human	H3N2		2003	USA	A/New_York/474/2003	agtgatgccccattccttgatcggcttcgccgagatcag aggtccctaaggggaagaggcaacactc	-	-
CY007039	Human H3N2 IAVs	Human	H3N2		2003	New_Zealand	A/Canterbury/403/2003	agtgatgccccattccttgatcggcttcgccgagatcag aggtccctaaggggaagaggcaacactc	-	-

CY015856	Human H3N2 IAVs	Human	H3N2		2003	Australia	A/Western_Australia/47/2003	agtgatgccccattccttgatcggcttcgccgagatcag aggtccctaaggggaagaggcaacactc	-	-
CY015872	Human H3N2 IAVs	Human	H3N2		2003	Australia	A/Western_Australia/50/2003	agtgatgccccattccttgatcggcttcgccgagatcag aggtccctaaggggaagaggcaacactc	-	-
CY090969	Human H3N2 IAVs	Human	H3N2		2003	USA	A/New_Jersey/NHRC0001/2003	agtgatgccccattccttgatcggcttcgccgagatcag aggtccctaaggggaagaggcaacactc	-	-
CY090961	Human H3N2 IAVs	Human	H3N2		2003	USA	A/Texas/NHRC0001/2003	agtgatgccccattccttgatcggcttcgccgagatcag aggtccctaaggggaagaggcaacactc	-	-
CY011748	Human H3N2 IAVs	Human	H3N2		2003	New_Zealand	A/Wellington/28/2003	agtgatgccccattccttgatcggcttcgccgagatcag aggtccctaaggggaagaggcaacactc	-	-
CY015776	Human H3N2 IAVs	Human	H3N2		2003	Australia	A/Western_Australia/37/2003	agtgatgccccattccttgatcggcttcgccgagatcag aggtccctaaggggaagaggcaacactc	-	-
CY015840	Human H3N2 IAVs	Human	H3N2		2003	Australia	A/Western_Australia/45/2003	agtgatgccccattccttgatcggcttcgccgagatcag aggtccctaaggggaagaggcaacactc	-	-
CY007143	Human H3N2 IAVs	Human	H3N2		2003	New_Zealand	A/Canterbury/424/2003	agtgatgccccattccttgatcggcttcgccgagatcag aggtccctaaggggaagaggcaacactc	-	-
CY088378	Human H3N2 IAVs	Human	H3N2		2003	United_Kingdom	A/England/534/2003	agtgatgccccattccttgatcggcttcgccgagatcag aggtccctaaggggaagaggcaacactc	-	-
CY008896	Human H3N2 IAVs	Human	H3N2		2003	USA	A/New_York/476/2003	agtgatgccccattccttgatcggcttcgccgagatcag aggtccctaaggggaagaggcaacactc	-	-
CY088322	Human H3N2 IAVs	Human	H3N2		2003	United_Kingdom	A/England/485/2003	agtgatgccccattccttgatcggcttcgccgagatcag aggtccctaaggggaagaggcaacactc	-	-
AM502785	Human H3N2 IAVs	Human	H3N2		2003	United_Kingdom	A/England/485/2003	agtgatgccccattccttgatcggcttcgccgagatcag aggtccctaaggggaagaggcaacactc	-	-
CY002108	Human H3N2 IAVs	Human	H3N2		2003	USA	A/Memphis/31/03	agtgatgccccattccttgatcggcttcgccgagatcag aggtccctaaggggaagaggcaacactc	-	-
CY017583	Human H3N2 IAVs	Human	H3N2		2003	Australia	A/Queensland/39/2003	agtgatgccccattccttgatcggcttcgccgagatcag aggtccctaaggggaagaggcaacactc	-	-
CY001025	Human H3N2 IAVs	Human	H3N2		2003	USA	A/New_York/1/2003	agtgatgccccattccttgatcggcttcgccgagatcag aggtccctaaggggaagaggcaacactc	-	-
CY008544	Human H3N2 IAVs	Human	H3N2		2003	New_Zealand	A/Canterbury/425/2003	agtgatgccccattccttgatcggcttcgccgagatcag aggtccctaaggggaagaggcaacactc	-	-
CY088250	Human H3N2 IAVs	Human	H3N2		2003	United_Kingdom	A/England/789/2003	agtgatgccccattccttgatcggcttcgccgagatcag aggtccctaaggggaagaggcaacactc	-	-
AM502810	Human H3N2 IAVs	Human	H3N2		2003	United_Kingdom	A/England/789/2003	agtgatgccccattccttgatcggcttcgccgagatcag aggtccctaaggggaagaggcaacactc	-	-
CY000157	Human H3N2 IAVs	Human	H3N2		2003	USA	A/New_York/41/2003	agtgatgccccattccttgatcggcttcgccgagatcag aggtccctaaggggaagaggcaacactc	-	-
CY088138	Human H3N2 IAVs	Human	H3N2		2003	United_Kingdom	A/Scotland/78/2003	agtgatgccccattccttgatcggcttcgccgagatcag aggtccctaaggggaagaggcaacactc	-	-
CY088122	Human H3N2 IAVs	Human	H3N2		2003	United_Kingdom	A/England/467/2003	agtgatgccccattccttgatcggcttcgccgagatcag aggtccctaaggggaagaggcaacactc	-	-
CY088114	Human H3N2 IAVs	Human	H3N2		2003	United_Kingdom	A/England/475/2003	agtgatgccccattccttgatcggcttcgccgagatcag aggtccctaaggggaagaggcaacactc	-	-
CY088469	Human H3N2 IAVs	Human	H3N2		2003	United_Kingdom	A/England/602/2003	agtgatgccccattccttgatcggcttcgccgagatcag aggtccctaaggggaagaggcaacactc	-	-
CY087994	Human H3N2 IAVs	Human	H3N2		2003	United_Kingdom	A/Scotland/52/2003	agtgatgccccattccttgatcggcttcgccgagatcag aggtccctaaggggaagaggcaacactc	-	-
AM502816	Human H3N2 IAVs	Human	H3N2		2003	United_Kingdom	A/Scotland/76/2003	agtgatgccccattccttgatcggcttcgccgagatcag aggtccctaaggggaagaggcaacactc	-	-

CY088130	Human H3N2 IAVs	Human	H3N2		2003	United_Kingdom	A/Scotland/76/2003	agtgatgccccattccttgatcggcttcgccgagatcag<br>aggtccctaaggggaagaggcaacactc	-	-
CY088154	Human H3N2 IAVs	Human	H3N2		2003	United_Kingdom	A/Scotland/81/2003	agtgatgccccattccttgatcggcttcgccgagatcag<br>aggtccctaaggggaagaggcaacactc	-	-
CY000085	Human H3N2 IAVs	Human	H3N2		2003	USA	A/New_York/48/2003	agtgatgccccattccttgatcggcttcgccgagatcag<br>aggtccctaaggggaagaggcaacactc	-	-
CY013132	Human H3N2 IAVs	Human	H3N2		2003	New_Zealand	A/Waikato/53/2003	agtgatgccccattccttgatcggcttcgccgagatcag<br>aggtccctaaggggaagaggcaacactc	-	-
CY007239	Human H3N2 IAVs	Human	H3N2		2003	New_Zealand	A/Canterbury/440/2003	agtgatgccccattccttgatcggcttcgccgagatcag<br>aggtccctaaggggaagaggcaacactc	-	-
AM502812	Human H3N2 IAVs	Human	H3N2		2003	United_Kingdom	A/England/3494349/2003	agtgatgccccattccttgatcggcttcgccgagatcag<br>aggtccctaaggggaagaggcaacactc	-	-
CY107078	Human H3N2 IAVs	Human	H3N2		2003	United_Kingdom	A/England/425/2003	agtgatgccccattccttgatcggcttcgccgagatcag<br>aggtccctaaggggaagaggcaacactc	-	-
CY088034	Human H3N2 IAVs	Human	H3N2		2003	United_Kingdom	A/England/425/2003	agtgatgccccattccttgatcggcttcgccgagatcag<br>aggtccctaaggggaagaggcaacactc	-	-
CY087978	Human H3N2 IAVs	Human	H3N2		2003	United_Kingdom	A/England/430/2003	agtgatgccccattccttgatcggcttcgccgagatcag<br>aggtccctaaggggaagaggcaacactc	-	-
AM502779	Human H3N2 IAVs	Human	H3N2		2003	United_Kingdom	A/England/430/2003	agtgatgccccattccttgatcggcttcgccgagatcag<br>aggtccctaaggggaagaggcaacactc	-	-
CY107174	Human H3N2 IAVs	Human	H3N2		2003	United_Kingdom	A/England/532/2003	agtgatgccccattccttgatcggcttcgccgagatcag<br>aggtccctaaggggaagaggcaacactc	-	-
CY088386	Human H3N2 IAVs	Human	H3N2		2003	United_Kingdom	A/England/532/2003	agtgatgccccattccttgatcggcttcgccgagatcag<br>aggtccctaaggggaagaggcaacactc	-	-
CY088426	Human H3N2 IAVs	Human	H3N2		2003	United_Kingdom	A/England/587/2003	agtgatgccccattccttgatcggcttcgccgagatcag<br>aggtccctaaggggaagaggcaacactc	-	-
CY088461	Human H3N2 IAVs	Human	H3N2		2003	United_Kingdom	A/England/593/2003	agtgatgccccattccttgatcggcttcgccgagatcag<br>aggtccctaaggggaagaggcaacactc	-	-
CY088194	Human H3N2 IAVs	Human	H3N2		2003	United_Kingdom	A/England/944/2003	agtgatgccccattccttgatcggcttcgccgagatcag<br>aggtccctaaggggaagaggcaacactc	-	-
CY088346	Human H3N2 IAVs	Human	H3N2		2003	United_Kingdom	A/England/957/2003	agtgatgccccattccttgatcggcttcgccgagatcag<br>aggtccctaaggggaagaggcaacactc	-	-
CY088434	Human H3N2 IAVs	Human	H3N2		2003	United_Kingdom	A/England/917/2003	agtgatgccccattccttgatcggcttcgccgagatcag<br>aggtccctaaggggaagaggcaacactc	-	-
CY107150	Human H3N2 IAVs	Human	H3N2		2003	United_Kingdom	A/England/491/2003	agtgatgccccattccttgatcggcttcgccgagatcag<br>aggtccctaaggggaagaggcaacactc	-	-
CY088298	Human H3N2 IAVs	Human	H3N2		2003	United_Kingdom	A/England/491/2003	agtgatgccccattccttgatcggcttcgccgagatcag<br>aggtccctaaggggaagaggcaacactc	-	-
AM502786	Human H3N2 IAVs	Human	H3N2		2003	United_Kingdom	A/England/491/2003	agtgatgccccattccttgatcggcttcgccgagatcag<br>aggtccctaaggggaagaggcaacactc	-	-
CY088234	Human H3N2 IAVs	Human	H3N2		2003	United_Kingdom	A/England/531/2003	agtgatgccccattccttgatcggcttcgccgagatcag<br>aggtccctaaggggaagaggcaacactc	-	-
AM502793	Human H3N2 IAVs	Human	H3N2		2003	United_Kingdom	A/England/531/2003	agtgatgccccattccttgatcggcttcgccgagatcag<br>aggtccctaaggggaagaggcaacactc	-	-
CY088210	Human H3N2 IAVs	Human	H3N2		2003	United_Kingdom	A/England/518/2003	agtgatgccccattccttgatcggcttcgccgagatcag<br>aggtccctaaggggaagaggcaacactc	-	-
CY012092	Human H3N2 IAVs	Human	H3N2		2003	New_Zealand	A/Waikato/102/2003	agtgatgccccattccttgatcggcttcgccgagatcag<br>aggtccctaaggggaagaggcaacactc	-	-
CY011740	Human H3N2 IAVs	Human	H3N2		2003	New_Zealand	A/Wellington/25/2003	agtgatgccccattccttgatcggcttcgccgagatcag<br>aggtccctaaggggaagaggcaacactc	-	-

CY012068	Human H3N2 IAVs	Human	H3N2		2003	New_Zealand	A/Wellington/34/2003	agtgatgccccattccttgatcggcttcgccgagatcag aggtccctaaggggaagaggcaacactc	-	-
CY012668	Human H3N2 IAVs	Human	H3N2		2003	New_Zealand	A/Wellington/49/2003	agtgatgccccattccttgatcggcttcgccgagatcag aggtccctaaggggaagaggcaacactc	-	-
CY012380	Human H3N2 IAVs	Human	H3N2		2003	New_Zealand	A/Wellington/53/2003	agtgatgccccattccttgatcggcttcgccgagatcag aggtccctaaggggaagaggcaacactc	-	-
CY088218	Human H3N2 IAVs	Human	H3N2		2003	United_Kingdom	A/England/787/2003	agtgatgccccattccttgatcggcttcgccgagatcag aggtccctaaggggaagaggcaacactc	-	-
AM502808	Human H3N2 IAVs	Human	H3N2		2003	United_Kingdom	A/England/787/2003	agtgatgccccattccttgatcggcttcgccgagatcag aggtccctaaggggaagaggcaacactc	-	-
CY118678	Human H3N2 IAVs	Human	H3N2		2003	Malaysia	A/Malaysia/25920/2003	agtgatgccccattccttgatcggcttcgccgagatcag aggtccctaaggggaagaggcaacactc	-	-
CY090929	Human H3N2 IAVs	Human	H3N2		2003	USA	A/South_Carolina/NHRC0001/2003	agtgatgccccattccttgatcggcttcgccgagatcag aggtccctaaggggaagaggcaacactc	-	-
CY015800	Human H3N2 IAVs	Human	H3N2		2003	Australia	A/Western_Australia/40/2003	agtgatgccccattccttgatcggcttcgccgagatcag aggtccctaaggggaagaggcaacactc	-	-
CY015848	Human H3N2 IAVs	Human	H3N2		2003	Australia	A/Western_Australia/46/2003	agtgatgccccattccttgatcggcttcgccgagatcag aggtccctaaggggaagaggcaacactc	-	-
CY118670	Human H3N2 IAVs	Human	H3N2		2003	Malaysia	A/Malaysia/25849/2003	agtgatgccccattccttgatcggcttcgccgagatcag aggtccctaaggggaagaggcaacactc	-	-
CY017937	Human H3N2 IAVs	Human	H3N2		2003	Australia	A/Queensland/37/2003	agtgatgccccattccttgatcggcttcgccgagatcag aggtccctaaggggaagaggcaacactc	-	-
CY088330	Human H3N2 IAVs	Human	H3N2		2003	United_Kingdom	A/England/508/2003	agtgatgccccattccttgatcggcttcgccgagatcag aggtccctaaggggaagaggcaacactc	-	-
AM502789	Human H3N2 IAVs	Human	H3N2		2003	United_Kingdom	A/England/508/2003	agtgatgccccattccttgatcggcttcgccgagatcag aggtccctaaggggaagaggcaacactc	-	-
CY000141	Human H3N2 IAVs	Human	H3N2		2003	USA	A/New_York/26/2003	agtgatgccccattccttgatcggcttcgccgagatcag aggtccctaaggggaagaggcaacactc	-	-
CY117965	Human H3N2 IAVs	Human	mixed		2003	Malaysia	A/Malaysia/25804/2003	agtgatgccccattccttgatcggcttcgccgagatcag aggtccctaaggggaagaggcaacactc	-	-
CY118686	Human H3N2 IAVs	Human	H3N2		2003	Malaysia	A/Malaysia/25957/2003	agtgatgccccattccttgatcggcttcgccgagatcag aggtccctaaggggaagaggcaacactc	-	-
CY118694	Human H3N2 IAVs	Human	H3N2		2003	Malaysia	A/Malaysia/26003/2003	agtgatgccccattccttgatcggcttcgccgagatcag aggtccctaaggggaagaggcaacactc	-	-
CY117975	Human H3N2 IAVs	Human	Mixed		2003	Malaysia	A/Malaysia/25890/2003	agtgatgccccattccttgatcggcttcgccgagatcag aggtccctaaggggaagaggcaacactc	-	-
CY001628	Human H3N2 IAVs	Human	H3N2		2003	USA	A/New_York/267/2003	agtgatgccccattccttgatcggcttcgccgagatcag aggtccctaaggggaagaggcaacactc	-	-
CY001209	Human H3N2 IAVs	Human	H3N2		2003	USA	A/New_York/56/2003	agtgatgccccattccttgatcggcttcgccgagatcag aggtccctaaggggaagaggcaacactc	-	-
CY007015	Human H3N2 IAVs	Human	H3N2		2003	New_Zealand	A/Canterbury/398/2003	agtgatgccccattccttgatcggcttcgccgagatcag aggtccctaaggggaagaggcaacactc	-	-
CY000953	Human H3N2 IAVs	Human	H3N2		2003	USA	A/New_York/55/2003	agtgatgccccattccttgatcggcttcgccgagatcag aggtccctaaggggaagaggcaacactc	-	-
CY011716	Human H3N2 IAVs	Human	H3N2		2003	New_Zealand	A/Waikato/1/2003	agtgatgccccattccttgatcggcttcgccgagatcag aggtccctaaggggaagaggcaacactc	-	-
CY017945	Human H3N2 IAVs	Human	H3N2		2003	Australia	A/Queensland/38/2003	agtgatgccccattccttgatcggcttcgccgagatcag aggtccctaaggggaagaggcaacactc	-	-
CY000509	Human H3N2 IAVs	Human	H3N2		2003	USA	A/New_York/4/2003	agtgatgccccattccttgatcggcttcgccgagatcag aggtccctaaggggaagaggcaacactc	-	-

CY007199	Human H3N2 IAVs	Human	H3N2		2003	New_Zealand	A/Canterbury/435/2003	agtgatgccccattccttgatcggcttcgccgagatcag<br>aggtccctaaggggaagaggcaacactc	-	-
CY006975	Human H3N2 IAVs	Human	H3N2		2003	New_Zealand	A/Canterbury/392/2003	agtgatgccccattccttgatcggcttcgccgagatcag<br>aggtccctaaggggaagaggcaacactc	-	-
CY088282	Human H3N2 IAVs	Human	H3N2		2003	United_Kingdom	A/England/487/2003	agtgatgccccattccttgatcggcttcgccgagatcag<br>aggtccctaaggggaagaggcaacactc	-	-
CY088338	Human H3N2 IAVs	Human	H3N2		2003	United_Kingdom	A/England/568/2003	agtgatgccccattccttgatcggcttcgccgagatcag<br>aggtccctaaggggaagaggcaacactc	-	-
AM502798	Human H3N2 IAVs	Human	H3N2		2003	United_Kingdom	A/England/568/2003	agtgatgccccattccttgatcggcttcgccgagatcag<br>aggtccctaaggggaagaggcaacactc	-	-
CY091465	Human H3N2 IAVs	Human	H3N2		2003	USA	A/California/NHRC0006/2003	agtgatgccccattccttgatcggcttcgccgagatcag<br>aggtccctaaggggaagaggcaacactc	-	-
CY000913	Human H3N2 IAVs	Human	H3N2		2003	USA	A/New_York/14/2003	agtgatgccccattccttgatcggcttcgccgagatcag<br>aggtccctaaggggaagaggcaacactc	-	-
CY000349	Human H3N2 IAVs	Human	H3N2		2003	USA	A/New_York/15/2003	agtgatgccccattccttgatcggcttcgccgagatcag<br>aggtccctaaggggaagaggcaacactc	-	-
CY000133	Human H3N2 IAVs	Human	H3N2		2003	USA	A/New_York/16/2003	agtgatgccccattccttgatcggcttcgccgagatcag<br>aggtccctaaggggaagaggcaacactc	-	-
CY000357	Human H3N2 IAVs	Human	H3N2		2003	USA	A/New_York/21/2003	agtgatgccccattccttgatcggcttcgccgagatcag<br>aggtccctaaggggaagaggcaacactc	-	-
CY000365	Human H3N2 IAVs	Human	H3N2		2003	USA	A/New_York/22/2003	agtgatgccccattccttgatcggcttcgccgagatcag<br>aggtccctaaggggaagaggcaacactc	-	-
CY001116	Human H3N2 IAVs	Human	H3N2		2003	USA	A/New_York/27/2003	agtgatgccccattccttgatcggcttcgccgagatcag<br>aggtccctaaggggaagaggcaacactc	-	-
CY000045	Human H3N2 IAVs	Human	H3N2		2003	USA	A/New_York/34/2003	agtgatgccccattccttgatcggcttcgccgagatcag<br>aggtccctaaggggaagaggcaacactc	-	-
CY000781	Human H3N2 IAVs	Human	H3N2		2003	USA	A/New_York/38/2003	agtgatgccccattccttgatcggcttcgccgagatcag<br>aggtccctaaggggaagaggcaacactc	-	-
CY001100	Human H3N2 IAVs	Human	H3N2		2003	USA	A/New_York/39/2003	agtgatgccccattccttgatcggcttcgccgagatcag<br>aggtccctaaggggaagaggcaacactc	-	-
CY000181	Human H3N2 IAVs	Human	H3N2		2003	USA	A/New_York/44/2003	agtgatgccccattccttgatcggcttcgccgagatcag<br>aggtccctaaggggaagaggcaacactc	-	-
CY000789	Human H3N2 IAVs	Human	H3N2		2003	USA	A/New_York/46/2003	agtgatgccccattccttgatcggcttcgccgagatcag<br>aggtccctaaggggaagaggcaacactc	-	-
CY000381	Human H3N2 IAVs	Human	H3N2		2003	USA	A/New_York/49/2003	agtgatgccccattccttgatcggcttcgccgagatcag<br>aggtccctaaggggaagaggcaacactc	-	-
CY000269	Human H3N2 IAVs	Human	H3N2		2003	USA	A/New_York/53/2003	agtgatgccccattccttgatcggcttcgccgagatcag<br>aggtccctaaggggaagaggcaacactc	-	-
CY001516	Human H3N2 IAVs	Human	H3N2		2003	USA	A/New_York/58/2003	agtgatgccccattccttgatcggcttcgccgagatcag<br>aggtccctaaggggaagaggcaacactc	-	-
KJ855455	Human H3N2 IAVs	Human	H3N2		2003	Mexico	A/Mexico/DIF2662/2003	agtgatgccccattccttgatcggcttcgccgagatcag<br>aggtccctaaggggaagaggcaacactc	-	-
CY001092	Human H3N2 IAVs	Human	H3N2		2003	USA	A/New_York/24/2003	agtgatgccccattccttgatcggcttcgccgagatcag<br>aggtccctaaggggaagaggcaacactc	-	-
CY000077	Human H3N2 IAVs	Human	H3N2		2003	USA	A/New_York/47/2003	agtgatgccccattccttgatcggcttcgccgagatcag<br>aggtccctaaggggaagaggcaacactc	-	-
CY000525	Human H3N2 IAVs	Human	H3N2		2003	USA	A/New_York/29/2003	agtgatgccccattccttgatcggcttcgccgagatcag<br>aggtccctaaggggaagaggcaacactc	-	-
CY000773	Human H3N2 IAVs	Human	H3N2		2003	USA	A/New_York/25/2003	agtgatgccccattccttgatcggcttcgccgagatcag<br>aggtccctaaggggaagaggcaacactc	-	-

CY100598	Human H3N2 IAVs	Human	H3N2		2003	Mexico	A/Mexico/InDRE2662/2003	agtgatgccccattccttgatcggcttcgccgagatcag<br>aggtccctaaggggaagaggcaacactc	-	-
CY001049	Human H3N2 IAVs	Human	H3N2		2003	USA	A/New_York/8/2003	agtgatgccccattccttgatcggcttcgccgagatcag<br>aggtccctaaggggaagaggcaacactc	-	-
CY001636	Human H3N2 IAVs	Human	H3N2		2003	USA	A/New_York/268/2003	agtgatgccccattccttgatcggcttcgccgagatcag<br>aggtccctaaggggaagaggcaacactc	-	-
CY107166	Human H3N2 IAVs	Human	H3N2		2003	United_Kingdom	A/England/528/2003	agtgatgccccattccttgatcggcttcgccgagatcag<br>aggtccctaaggggaagaggcaacactc	-	-
CY088314	Human H3N2 IAVs	Human	H3N2		2003	United_Kingdom	A/England/528/2003	agtgatgccccattccttgatcggcttcgccgagatcag<br>aggtccctaaggggaagaggcaacactc	-	-
AM502792	Human H3N2 IAVs	Human	H3N2		2003	United_Kingdom	A/England/528/2003	agtgatgccccattccttgatcggcttcgccgagatcag<br>aggtccctaaggggaagaggcaacactc	-	-
CY009248	Human H3N2 IAVs	Human	H3N2		2003	USA	A/New_York/477/2003	agtgatgccccattccttgatcggcttcgccgagatcag<br>aggtccctaaggggaagaggcaacactc	-	-
AM502815	Human H3N2 IAVs	Human	H3N2		2003	United_Kingdom	A/Scotland/71/2003	agtgatgccccattccttgatcggcttcgccgagatcag<br>aggtccctaaggggaagaggcaacactc	-	-
CY001409	Human H3N2 IAVs	Human	H3N2		2003	USA	A/New_York/213/2003	agtgatgccccattccttgatcggcttcgccgagatcag<br>aggtccctaaggggaagaggcaacactc	-	-
CY001556	Human H3N2 IAVs	Human	H3N2		2003	USA	A/New_York/215/2003	agtgatgccccattccttgatcggcttcgccgagatcag<br>aggtccctaaggggaagaggcaacactc	-	-
CY001564	Human H3N2 IAVs	Human	H3N2		2003	USA	A/New_York/216/2003	agtgatgccccattccttgatcggcttcgccgagatcag<br>aggtccctaaggggaagaggcaacactc	-	-
CY000877	Human H3N2 IAVs	Human	H3N2		2003	USA	A/New_York/194/2003	agtgatgccccattccttgatcggcttcgccgagatcag<br>aggtccctaaggggaagaggcaacactc	-	-
CY001465	Human H3N2 IAVs	Human	H3N2		2003	USA	A/New_York/195/2003	agtgatgccccattccttgatcggcttcgccgagatcag<br>aggtccctaaggggaagaggcaacactc	-	-
CY104080	Human H3N2 IAVs	Human	H3N2		2003	Viet_Nam	A/HaNoi/ARI189/2003	agtgatgccccattccttgatcggcttcgccgagatcag<br>aggtccctaaggggaagaggcaatactc	-	-
CY118702	Human H3N2 IAVs	Human	H3N2		2003	Malaysia	A/Malaysia/26430/2003	agtgatgccccattccttgatcggcttcgccgagatcag<br>aggtccctaaggggaagaggcaatactc	-	-
KJ855383	Human H3N2 IAVs	Human	H3N2		2003	Mexico	A/Mexico/DIF835/2003	agtgatgccccattccttgatcggcttcgccgagatcag<br>aggtccctaaggggaagaggcaatactc	-	-
CY100622	Human H3N2 IAVs	Human	H3N2		2003	Mexico	A/Mexico/InDRE835/2003	agtgatgccccattccttgatcggcttcgccgagatcag<br>aggtccctaaggggaagaggcaatactc	-	-
CY001540	Human H3N2 IAVs	Human	H3N2		2003	USA	A/New_York/196/2003	agtgatgccccattccttgatcggcttcgccgagatcag<br>aggtccctaaggggaagaggcaatactc	-	-
CY001257	Human H3N2 IAVs	Human	H3N2		2003	USA	A/New_York/199/2003	agtgatgccccattccttgatcggcttcgccgagatcag<br>aggtccctaaggggaagaggcaatactc	-	-
KC197839	Human H3N2 IAVs	Human	H3N2		2003	Sri_Lanka	A/Ragama/166/2003	agtgatgccccattccttgatcggcttcgccgagatcag<br>aggtccctaaggggaagaggcaatactc	-	-
CY091153	Human H3N2 IAVs	Human	H3N2		2003	Singapore	A/Singapore/NHRC0001/2003	agtgatgccccattccttgatcggcttcgccgagatcag<br>aggtccctaaggggaagaggcaatactc	-	-
CY091497	Human H3N2 IAVs	Human	H3N2		2003	Singapore	A/Singapore/NHRC0002/2003	agtgatgccccattccttgatcggcttcgccgagatcag<br>aggtccctaaggggaagaggcaatactc	-	-
CY091489	Human H3N2 IAVs	Human	H3N2		2003	Singapore	A/Singapore/NHRC0003/2003	agtgatgccccattccttgatcggcttcgccgagatcag<br>aggtccctaaggggaagaggcaatactc	-	-
CY091481	Human H3N2 IAVs	Human	H3N2		2003	Singapore	A/Singapore/NHRC0004/2003	agtgatgccccattccttgatcggcttcgccgagatcag<br>aggtccctaaggggaagaggcaatactc	-	-
CY091161	Human H3N2 IAVs	Human	H3N2		2003	Singapore	A/Singapore/NHRC0005/2003	agtgatgccccattccttgatcggcttcgccgagatcag<br>aggtccctaaggggaagaggcaatactc	-	-

CY091473	Human H3N2 IAVs	Human	H3N2		2003	Singapore	A/Singapore/NHRC0006/2003	agtgatgccccattccttgatcggcttcgccgagatcag<br>aggtccctaaggggaagaggcaatactc	-	-
CY091265	Human H3N2 IAVs	Human	H3N2		2003	Singapore	A/Singapore/NHRC0007/2003	agtgatgccccattccttgatcggcttcgccgagatcag<br>aggtccctaaggggaagaggcaatactc	-	-
CY091257	Human H3N2 IAVs	Human	H3N2		2003	Singapore	A/Singapore/NHRC0008/2003	agtgatgccccattccttgatcggcttcgccgagatcag<br>aggtccctaaggggaagaggcaatactc	-	-
CY091241	Human H3N2 IAVs	Human	H3N2		2003	Singapore	A/Singapore/NHRC0010/2003	agtgatgccccattccttgatcggcttcgccgagatcag<br>aggtccctaaggggaagaggcaatactc	-	-
CY091169	Human H3N2 IAVs	Human	H3N2		2003	Singapore	A/Singapore/NHRC0011/2003	agtgatgccccattccttgatcggcttcgccgagatcag<br>aggtccctaaggggaagaggcaatactc	-	-
CY091233	Human H3N2 IAVs	Human	H3N2		2003	Singapore	A/Singapore/NHRC0012/2003	agtgatgccccattccttgatcggcttcgccgagatcag<br>aggtccctaaggggaagaggcaatactc	-	-
CY104088	Human H3N2 IAVs	Human	H3N2		2003	Viet_Nam	A/TayNguyen/TN152/2003	agtgatgccccattccttgatcggcttcgccgagatcag<br>aggtccctaaggggaagaggcaatactc	-	-
CY105242	Human H3N2 IAVs	Human	H3N2		2003	Viet_Nam	A/TayNguyen/TN160/2003	agtgatgccccattccttgatcggcttcgccgagatcag<br>aggtccctaaggggaagaggcaatactc	-	-
CY104096	Human H3N2 IAVs	Human	H3N2		2003	Viet_Nam	A/TayNguyen/TN170/2003	agtgatgccccattccttgatcggcttcgccgagatcag<br>aggtccctaaggggaagaggcaatactc	-	-
CY104104	Human H3N2 IAVs	Human	H3N2		2003	Viet_Nam	A/TayNguyen/TN171/2003	agtgatgccccattccttgatcggcttcgccgagatcag<br>aggtccctaaggggaagaggcaatactc	-	-
CY105250	Human H3N2 IAVs	Human	H3N2		2003	Viet_Nam	A/TayNguyen/TN186/2003	agtgatgccccattccttgatcggcttcgccgagatcag<br>aggtccctaaggggaagaggcaatactc	-	-
CY104112	Human H3N2 IAVs	Human	H3N2		2003	Viet_Nam	A/TayNguyen/TN83/2003	agtgatgccccattccttgatcggcttcgccgagatcag<br>aggtccctaaggggaagaggcaatactc	-	-
CY104120	Human H3N2 IAVs	Human	H3N2		2003	Viet_Nam	A/TayNguyen/TN85/2003	agtgatgccccattccttgatcggcttcgccgagatcag<br>aggtccctaaggggaagaggcaatactc	-	-
CY105266	Human H3N2 IAVs	Human	H3N2		2003	Viet_Nam	A/TayNguyen/TN905/2003	agtgatgccccattccttgatcggcttcgccgagatcag<br>aggtccctaaggggaagaggcaatactc	-	-
CY104128	Human H3N2 IAVs	Human	H3N2		2003	Viet_Nam	A/TayNguyen/TN92/2003	agtgatgccccattccttgatcggcttcgccgagatcag<br>aggtccctaaggggaagaggcaatactc	-	-
CY034112	Human H3N2 IAVs	Human	H3N2		2003	USA	A/Wyoming/03/2003	agtgatgccccattccttgatcggcttcgccgagatcag<br>aggtccctaaggggaagaggcaatactc	-	-
CY006863	Human H3N2 IAVs	Human	H3N2		2003	USA	A/New_York/214/2003	agtgatgccccattccttgatcggcttcgccgagatcag<br>aggtccctaaggggaagaggcaatactc	-	-
CY105218	Human H3N2 IAVs	Human	H3N2		2003	Viet_Nam	A/Hanoi/BM767/2003	agtgatgccccattccttgatcggcttcgccgagatcag<br>aggtccctaaggggaagaggcaatactc	-	-
CY091225	Human H3N2 IAVs	Human	H3N2		2003	USA	A/California/NHRC0002/2003	agtgatgccccattccttgatcggcttcgccgagatcag<br>aggtccctaaggggaagaggcaatactc	-	-
CY091177	Human H3N2 IAVs	Human	H3N2		2003	USA	A/California/NHRC0003/2003	agtgatgccccattccttgatcggcttcgccgagatcag<br>aggtccctaaggggaagaggcaatactc	-	-
CY091217	Human H3N2 IAVs	Human	H3N2		2003	USA	A/California/NHRC0004/2003	agtgatgccccattccttgatcggcttcgccgagatcag<br>aggtccctaaggggaagaggcaatactc	-	-
CY112977	Human H3N2 IAVs	Human	H3N2		2003	Netherlands	A/Netherlands/109/2003	agtgatgccccattccttgatcggcttcgccgagatcag<br>aggtccctaaggggaagaggcaatactc	-	-
CY112985	Human H3N2 IAVs	Human	H3N2		2003	Netherlands	A/Netherlands/217/2003	agtgatgccccattccttgatcggcttcgccgagatcag<br>aggtccctaaggggaagaggcaatactc	-	-
CY114361	Human H3N2 IAVs	Human	H3N2		2003	Netherlands	A/Netherlands/222/2003	agtgatgccccattccttgatcggcttcgccgagatcag<br>aggtccctaaggggaagaggcaatactc	-	-
HQ166056	Human H3N2 IAVs	Human	H3N2		2003	Netherlands	A/Netherlands/213/2003	agtgatgccccattccttgatcggcttcgccgagatcag<br>aggtccctaaggggaagaggcaatactc	-	-

CY001644	Human H3N2 IAVs	Human	H3N2		2003	USA	A/New_York/269/2003	agtgatgccccattccttgatcggcttcgccgagatcag aggtccctaaggggaagaggcaatactc	-	-
CY000029	Human H3N2 IAVs	Human	H3N2		2003	USA	A/New_York/32/2003	agtgatgccccattccttgatcggcttcgccgagatcag aggtccctaaggggaagaggcaatactc	-	-
KJ855375	Human H3N2 IAVs	Human	H3N2		2003	Mexico	A/Mexico/DIF756/2003	agtgatgccccattccttgatcggcttcgccgagatcag aggtccctaaggggaagaggcaatactc	-	-
CY100614	Human H3N2 IAVs	Human	H3N2		2003	Mexico	A/Mexico/InDRE756/2003	agtgatgccccattccttgatcggcttcgccgagatcag aggtccctaaggggaagaggcaatactc	-	-
CY003684	Human H3N2 IAVs	Human	H3N2		2003	USA	A/New_York/485/2003	agtgatgccccattccttgatcggcttcgccgagatcag aggtccctaaggggaagaggcaatactc	-	-
CY091249	Human H3N2 IAVs	Human	H3N2		2003	Singapore	A/Singapore/NHRC0009/2003	agtgatgccccattccttgatcggcttcgccgagatcag aggtccctaaggggaagaggcaatactc	-	-
KJ855391	Human H3N2 IAVs	Human	H3N2		2003	Mexico	A/Mexico/DIF940/2003	agtgatgccccattccttgatcggcttcgccgagatcag aggtccctaaggggaagaggcaatactc	-	-
CY100630	Human H3N2 IAVs	Human	H3N2		2003	Mexico	A/Mexico/InDRE940/2003	agtgatgccccattccttgatcggcttcgccgagatcag aggtccctaaggggaagaggcaatactc	-	-
CY118710	Human H3N2 IAVs	Human	H3N2		2003	Malaysia	A/Malaysia/26468/2003	agtgatgccccattccttgatcggcttcgccgagatcag aggtccctaaggggaagaggcaatactc	-	-
CY100590	Human H3N2 IAVs	Human	H3N2		2003	Mexico	A/Mexico/InDRE2601/2003	agtgatgccccattccttgatcggcttcgccgagatcag aggtccctaaggggaagaggcaatactc	-	-
DQ098263	Human H3N2 IAVs	Human	H3N2		2003	Russia	A/Moscow/343/2003	agtgatgccccattccttgatcggcttcgccgagatcag aggtccctaaggggaagaggcaatactc	-	-
DQ098262	Human H3N2 IAVs	Human	H3N2		2003	Russia	A/Moscow/343/2003	agtgatgccccattccttgatcggcttcgccgagatcag aggtccctaaggggaagaggcaatactc	-	-
DQ098265	Human H3N2 IAVs	Human	H3N2		2003	Russia	A/Moscow/346/2003	agtgatgccccattccttgatcggcttcgccgagatcag aggtccctaaggggaagaggcaatactc	-	-
DQ098264	Human H3N2 IAVs	Human	H3N2		2003	Russia	A/Moscow/346/2003	agtgatgccccattccttgatcggcttcgccgagatcag aggtccctaaggggaagaggcaatactc	-	-
CY117918	Human H3N2 IAVs	Human	mixed		2003	Malaysia	A/Malaysia/23941/2003	agtgatgccccattccttgatcggcttcgccgagatcag aggtccctaaggggaagaggcaatactc	-	-
CY117933	Human H3N2 IAVs	Human	mixed		2003	Malaysia	A/Malaysia/23995/2003	agtgatgccccattccttgatcggcttcgccgagatcag aggtccctaaggggaagaggcaatactc	-	-
KC197840	Human H3N2 IAVs	Human	H3N2		2003	Sri_Lanka	A/Ragama/190/2003	agtgatgccccattccttgatcggcttcgccgagatcag aggtccctaaggggaagaggcaatactc	-	-
CY088370	Human H3N2 IAVs	Human	H3N2		2003	United_Kingdom	A/England/558/2003	agtgatgccccattccttgatcggcttcgccgagatcag aggtccctaaggggaagaggcaatactc	-	-
CY032965	Human H3N2 IAVs	Human	H3N2		2003	Mexico	A/Yucatan/844/2003	agtgatgccccattccttgatcggcttcgccgagatcag aggtccctaaggggaagaggcaatactc	-	-
DQ415366	Human H3N2 IAVs	Human	H3N2		2003	Taiwan	A/TW/229/03	agtgatgccccattccttgatcggcttcgccgagatcag aggtccctaaggggaagaggcaatactc	-	-
CY117984	Human H3N2 IAVs	Human	H3N2		2003	Malaysia	A/Malaysia/26227/2003	agtgatgccccattccttgatcggcttcgccgagatcag aggtccctaaggggaagaggcaatactc	-	-
CY118718	Human H3N2 IAVs	Human	H3N2		2003	Malaysia	A/Malaysia/26571/2003	agtgatgccccattccttgatcggcttcgccgagatcag aggtccctaaggggaagaggcaatactc	-	-
CY121452	Human H3N2 IAVs	Human	H3N2		2003	China	A/Fujian/445/2003	agtgatgccccattccttgatcggcttcgccgagatcag aggtccctaaggggaagaggcaatactc	-	-
CY002524	Human H3N2 IAVs	Human	H3N2		2003	USA	A/New_York/204/2003	agtgatgccccattccttgatcggcttcgccgagatcag aggtccctaaggggaagaggcaatactc	-	-
CY001377	Human H3N2 IAVs	Human	H3N2		2003	USA	A/New_York/201/2003	agtgatgccccattccttgatcggcttcgccgagatcag aggtccctaaggggaagaggcaatactc	-	-

CY001017	Human H3N2 IAVs	Human	H3N2		2003	USA	A/New_York/198/2003	agtgatgccccattccttgatcggcttcgccgagatcag<br>aggtccctaaggggaagaggcaatactc	-	-
CY001473	Human H3N2 IAVs	Human	H3N2		2003	USA	A/New_York/203/2003	agtgatgccccattccttgatcggcttcgccgagatcag<br>aggtccctaaggggaagaggcaatactc	-	-
KC197837	Human H3N2 IAVs	Human	H3N2		2003	Sri_Lanka	A/Ragama/143/2003	agtgatgccccattccttgatcggcttcgccgagatcag<br>aggtccctaaggggaagaggcaatactc	-	-
CY105210	Human H3N2 IAVs	Human	H3N2		2003	Viet_Nam	A/Hanoi/BM766/2003	agtgatgccccattccttgatcggcttcgccgagatcag<br>aggtccctaaggggaagaggcaatactc	-	-
EU268231	Human H3N2 IAVs	Human	H3N2		2003	USA	A/Wyoming/03/2003	agtgatgccccattccttgatcggcttcgccgagatcag<br>aggtccctaaggggaagaggcaatactc	-	-
CY117946	Human H3N2 IAVs	Human	mixed		2003	Malaysia	A/Malaysia/24083/2003	agtgatgccccattccttgatcggcttcgccgagatcag<br>aggtccctaaggggaagaggcaatactc	-	-
CY105258	Human H3N2 IAVs	Human	H3N2		2003	Viet_Nam	A/HaNoi/HN695/2003	agtgatgccccattccttgatcggcttcgccgagatcag<br>aggtccctaaggggaagaggcaatactc	-	-
DQ096580	Human H3N2 IAVs	Human	H3N2		2003	Russia	A/Moscow/328/2003	agtgatgccccattccttgatcggcttcgccgagatcag<br>aggtccctaaggggaagaggcaatactc	-	-
DQ098261	Human H3N2 IAVs	Human	H3N2		2003	Russia	A/Moscow/328/2003	agtgatgccccattccttgatcggcttcgccgagatcag<br>aggtccctaaggggaagaggcaatactc	-	-
CY112961	Human H3N2 IAVs	Human	H3N2		2003	Netherlands	A/Netherlands/22/2003	agtgatgccccattccttgatcggcttcgccgagatcag<br>aggtccctaaggggaagaggcaatactc	-	-
DQ415367	Human H3N2 IAVs	Human	H3N2		2003	Taiwan	A/TW/3286/03	agtgatgccccattccttgatcggcttcgccgagatcag<br>aggtccctaaggggaagaggcaatactc	-	-
CY001225	Human H3N2 IAVs	Human	H3N2		2003	USA	A/New_York/65/2003	agtgatgccccattccttgatcggcttcgccgagatcag<br>aggtccctaaggggaagaggcagcactc	-	-
CY000869	Human H3N2 IAVs	Human	H3N2		2003	USA	A/New_York/193/2003	agtgatgccccattccttgatcggcttcgccgagatcag<br>aggtccctaaggggggagaggcaacactc	-	-
CY001548	Human H3N2 IAVs	Human	H3N2		2003	USA	A/New_York/197/2003	agtgatgccccattccttgatcggcttcgccgagatcag<br>aggtccctaaggggggagaggcaacactc	-	-
CY088178	Human H3N2 IAVs	Human	H3N2		2003	United_Kingdom	A/England/539/2003	agtgatgccccattccttgatcggcttcgccgagatcag<br>aggtccttaaggggaagaggcaacactc	+	+
CY037355	Human H3N2 IAVs	Human	H3N2		2003	Italy	A/Genoa/14/2003	agtgatgccccattccttgatcggcttcgccgggatcag<br>aggtccctaaggggaagaggcaacactc	-	-
CY000757	Human H3N2 IAVs	Human	H3N2		2003	USA	A/New_York/192/2003	agtgatgccccattccttgatcggcttcgccgggatcag<br>aggtccctaaggggaagaggcaacactc	-	-
JF710768	Human H3N2 IAVs	Human	H3N2		2003	United_Kingdom	A/England/367/2003	agtgatgctccattccttgatcggcttcgccgagatcag<br>aggtccctaaggggaagaggcaacactc	-	-
JF710769	Human H3N2 IAVs	Human	H3N2		2003	United_Kingdom	A/England/368/2003	agtgatgctccattccttgatcggcttcgccgagatcag<br>aggtccctaaggggaagaggcaacactc	-	-
CY001164	Human H3N2 IAVs	Human	H3N2		2003	USA	A/New_York/202/2003	agtgatgctccattccttgatcggcttcgccgagatcag<br>aggtccctaaggggaagaggcaacactc	-	-
JF710770	Human H3N2 IAVs	Human	H3N2		2003	United_Kingdom	A/England/375/2003	agtgatgctccattccttgatcggcttcgccgagatcag<br>aggtccctaaggggaagaggcaacactc	-	-
CY114353	Human H3N2 IAVs	Human	H3N2		2003	Finland	A/Finland/170/2003	agtgatgctccattccttgatcggcttcgccgagatcag<br>aggtccctaaggggaagaggcaacactc	-	-
JF710767	Human H3N2 IAVs	Human	H3N2		2003	United_Kingdom	A/England/364/2003	agtgatgctccattccttgatcggcttcgccgagatcag<br>aggtccctaaggggaagaggcaacactc	-	-
CY032973	Human H3N2 IAVs	Human	H3N2		2003	Mexico	A/Yucatan/ME6057/2003	agtgatgtcccattccttgatcggcttcgccgagatcag<br>aggtccctaaggggaagaggcaatactc	+	+
CY002444	Human H3N2 IAVs	Human	H3N2		2004	USA	A/New_York/365/2004	agtgatgccccattccttgaccggcttcgccgagatca<br>gaggtccctaagaggaagaggcaatactc	+	+

FJ157993	Human H3N2 IAVs	Swine	H3N2		2004	China	A/swine/Guangxi/1/2004	agtgatgccccattccttgatcgacttcgccgagatca<br>gaggtccctaaggggaagaggcaacactc	-	-
CY104168	Human H3N2 IAVs	Human	H3N2		2004	Viet_Nam	A/HaNoi/HN30136/2004	agtgatgccccattccttgatcggattcgccgagatca<br>gaggtccctaaggggaagaggcaatactc	-	-
CY105330	Human H3N2 IAVs	Human	H3N2		2004	Viet_Nam	A/Hanoi/HN3069/2004	agtgatgccccattccttgatcggcttcgccgagatcag<br>aagtccctaaggggaagaggcaatactc	+	+
CY002220	Human H3N2 IAVs	Human	H3N2		2004	USA	A/New_York/371/2004	agtgatgccccattccttgatcggcttcgccgagatcag<br>agatccctaagaggaagaggcaatactc	-	*
CY003668	Human H3N2 IAVs	Human	H3N2		2004	USA	A/New_York/471/2004	agtgatgccccattccttgatcggcttcgccgagatcag<br>aggtccctaaaaggaagaggcaatactc	+	+
CY002958	Human H3N2 IAVs	Human	H3N2		2004	New_Zealand	A/Ashburton/280/2004	agtgatgccccattccttgatcggcttcgccgagatcag<br>aggtccctaagaggaagaggcaatactc	+	+
CY007575	Human H3N2 IAVs	Human	H3N2		2004	New_Zealand	A/Canterbury/313/2004	agtgatgccccattccttgatcggcttcgccgagatcag<br>aggtccctaagaggaagaggcaatactc	+	+
CY002973	Human H3N2 IAVs	Human	H3N2		2004	New_Zealand	A/Christchurch/10/2004	agtgatgccccattccttgatcggcttcgccgagatcag<br>aggtccctaagaggaagaggcaatactc	+	+
CY013481	Human H3N2 IAVs	Human	H3N2		2004	New_Zealand	A/Wellington/27/2004	agtgatgccccattccttgatcggcttcgccgagatcag<br>aggtccctaagaggaagaggcaatactc	+	+
CY013915	Human H3N2 IAVs	Human	H3N2		2004	New_Zealand	A/Wellington/4/2004	agtgatgccccattccttgatcggcttcgccgagatcag<br>aggtccctaagaggaagaggcaatactc	+	+
CY012684	Human H3N2 IAVs	Human	H3N2		2004	New_Zealand	A/Wellington/8/2004	agtgatgccccattccttgatcggcttcgccgagatcag<br>aggtccctaagaggaagaggcaatactc	+	+
CY002910	Human H3N2 IAVs	Human	H3N2		2004	New_Zealand	A/Christchurch/13/2004	agtgatgccccattccttgatcggcttcgccgagatcag<br>aggtccctaagaggaagaggcaatactc	+	+
CY006383	Human H3N2 IAVs	Human	H3N2		2004	USA	A/New_York/467/2004	agtgatgccccattccttgatcggcttcgccgagatcag<br>aggtccctaagaggaagaggcaatactc	+	+
CY003660	Human H3N2 IAVs	Human	H3N2		2004	USA	A/New_York/468/2004	agtgatgccccattccttgatcggcttcgccgagatcag<br>aggtccctaagaggaagaggcaatactc	+	+
CY006183	Human H3N2 IAVs	Human	H3N2		2004	USA	A/New_York/469/2004	agtgatgccccattccttgatcggcttcgccgagatcag<br>aggtccctaagaggaagaggcaatactc	+	+
CY033642	Human H3N2 IAVs	Human	H3N2		2004	USA	A/New_York/55/2004	agtgatgccccattccttgatcggcttcgccgagatcag<br>aggtccctaagaggaagaggcaatactc	+	+
CY121129	Human H3N2 IAVs	Human	H3N2		2004	USA	A/New_York/55/2004	agtgatgccccattccttgatcggcttcgccgagatcag<br>aggtccctaagaggaagaggcaatactc	+	+
CY002284	Human H3N2 IAVs	Human	H3N2		2004	USA	A/New_York/98/2004	agtgatgccccattccttgatcggcttcgccgagatcag<br>aggtccctaagaggaagaggcaatactc	+	+
CY002716	Human H3N2 IAVs	Human	H3N2		2004	USA	A/New_York/319/2004	agtgatgccccattccttgatcggcttcgccgagatcag<br>aggtccctaagaggaagaggcaatactc	+	+
CY002476	Human H3N2 IAVs	Human	H3N2		2004	USA	A/New_York/385/2004	agtgatgccccattccttgatcggcttcgccgagatcag<br>aggtccctaagaggaagaggcaatactc	+	+
CY016216	Human H3N2 IAVs	Human	H3N2		2004	New_Zealand	A/Waikato/1/2004	agtgatgccccattccttgatcggcttcgccgagatcag<br>aggtccctaagaggaagaggcaatactc	+	+
CY003076	Human H3N2 IAVs	Human	H3N2		2004	USA	A/New_York/123/2004	agtgatgccccattccttgatcggcttcgccgagatcag<br>aggtccctaagaggaagaggcaatactc	+	+
CY002788	Human H3N2 IAVs	Human	H3N2		2004	USA	A/New_York/232/2004	agtgatgccccattccttgatcggcttcgccgagatcag<br>aggtccctaagaggaagaggcaatactc	+	+
CY002212	Human H3N2 IAVs	Human	H3N2		2004	USA	A/New_York/370/2004	agtgatgccccattccttgatcggcttcgccgagatcag<br>aggtccctaagaggaagaggcaatactc	+	+
CY002596	Human H3N2 IAVs	Human	H3N2		2004	USA	A/New_York/377/2004	agtgatgccccattccttgatcggcttcgccgagatcag<br>aggtccctaagaggaagaggcaatactc	+	+

CY002732	Human H3N2 IAVs	Human	H3N2		2004	USA	A/New_York/363/2004	agtgatgccccattccttgatcggcttcgccgagatcag<br>aggtccctaagaggaagaggcaatactc	+	+
CY003420	Human H3N2 IAVs	Human	H3N2		2004	USA	A/New_York/237/2004	agtgatgccccattccttgatcggcttcgccgagatcag<br>aggtccctaagaggaagaggcaatactc	+	+
CY002180	Human H3N2 IAVs	Human	H3N2		2004	USA	A/New_York/356/2004	agtgatgccccattccttgatcggcttcgccgagatcag<br>aggtccctaagaggaagaggcaatactc	+	+
CY002436	Human H3N2 IAVs	Human	H3N2		2004	USA	A/New_York/362/2004	agtgatgccccattccttgatcggcttcgccgagatcag<br>aggtccctaagaggaagaggcaatactc	+	+
CY002236	Human H3N2 IAVs	Human	H3N2		2004	USA	A/New_York/374/2004	agtgatgccccattccttgatcggcttcgccgagatcag<br>aggtccctaagaggaagaggcaatactc	+	+
CY019185	Human H3N2 IAVs	Human	H3N2		2004	USA	A/New_York/905/2004	agtgatgccccattccttgatcggcttcgccgagatcag<br>aggtccctaagaggaagaggcaatactc	+	+
CY019177	Human H3N2 IAVs	Human	H3N2		2004	USA	A/New_York/905/2004	agtgatgccccattccttgatcggcttcgccgagatcag<br>aggtccctaagaggaagaggcaatactc	+	+
CY021993	Human H3N2 IAVs	Human	H3N2		2004	USA	A/New_York/908/2004	agtgatgccccattccttgatcggcttcgccgagatcag<br>aggtccctaagaggaagaggcaatactc	+	+
CY019249	Human H3N2 IAVs	Human	H3N2		2004	USA	A/New_York/908/2004	agtgatgccccattccttgatcggcttcgccgagatcag<br>aggtccctaagaggaagaggcaatactc	+	+
CY019265	Human H3N2 IAVs	Human	H3N2		2004	USA	A/New_York/908/2004	agtgatgccccattccttgatcggcttcgccgagatcag<br>aggtccctaagaggaagaggcaatactc	+	+
CY019823	Human H3N2 IAVs	Human	H3N2		2004	USA	A/New_York/908/2004	agtgatgccccattccttgatcggcttcgccgagatcag<br>aggtccctaagaggaagaggcaatactc	+	+
CY019257	Human H3N2 IAVs	Human	H3N2		2004	USA	A/New_York/908/2004	agtgatgccccattccttgatcggcttcgccgagatcag<br>aggtccctaagaggaagaggcaatactc	+	+
CY002252	Human H3N2 IAVs	Human	H3N2		2004	USA	A/New_York/381/2004	agtgatgccccattccttgatcggcttcgccgagatcag<br>aggtccctaagaggaagaggcaatactc	+	+
CY003412	Human H3N2 IAVs	Human	H3N2		2004	USA	A/New_York/236/2004	agtgatgccccattccttgatcggcttcgccgagatcag<br>aggtccctaagaggaagaggcaatactc	+	+
CY006096	Human H3N2 IAVs	Human	H3N2		2004	USA	A/New_York/470/2004	agtgatgccccattccttgatcggcttcgccgagatcag<br>aggtccctaagaggaagaggcaatactc	+	+
CY002612	Human H3N2 IAVs	Human	H3N2		2004	USA	A/New_York/190/2004	agtgatgccccattccttgatcggcttcgccgagatcag<br>aggtccctaagaggaagaggcaatactc	+	+
CY007647	Human H3N2 IAVs	Human	H3N2		2004	USA	A/New_York/245/2004	agtgatgccccattccttgatcggcttcgccgagatcag<br>aggtccctaagaggaagaggcaatactc	+	+
CY002420	Human H3N2 IAVs	Human	H3N2		2004	USA	A/New_York/353/2004	agtgatgccccattccttgatcggcttcgccgagatcag<br>aggtccctaagaggaagaggcaatactc	+	+
CY002044	Human H3N2 IAVs	Human	H3N2		2004	USA	A/New_York/386/2004	agtgatgccccattccttgatcggcttcgccgagatcag<br>aggtccctaagaggaagaggcaatactc	+	+
CY002772	Human H3N2 IAVs	Human	H3N2		2004	USA	A/New_York/200/2004	agtgatgccccattccttgatcggcttcgccgagatcag<br>aggtccctaagaggaagaggcaatactc	+	+
CY002428	Human H3N2 IAVs	Human	H3N2		2004	USA	A/New_York/360/2004	agtgatgccccattccttgatcggcttcgccgagatcag<br>aggtccctaagaggaagaggcaatactc	+	+
CY006439	Human H3N2 IAVs	Human	H3N2		2004	USA	A/New_York/368/2004	agtgatgccccattccttgatcggcttcgccgagatcag<br>aggtccctaagaggaagaggcaatactc	+	+
CY002228	Human H3N2 IAVs	Human	H3N2		2004	USA	A/New_York/372/2004	agtgatgccccattccttgatcggcttcgccgagatcag<br>aggtccctaagaggaagaggcaatactc	+	+
CY019145	Human H3N2 IAVs	Human	H3N2		2004	USA	A/New_York/900/2004	agtgatgccccattccttgatcggcttcgccgagatcag<br>aggtccctaagaggaagaggcaatactc	+	+
CY019153	Human H3N2 IAVs	Human	H3N2		2004	USA	A/New_York/900/2004	agtgatgccccattccttgatcggcttcgccgagatcag<br>aggtccctaagaggaagaggcaatactc	+	+

CY019815	Human H3N2 IAVs	Human	H3N2		2004	USA	A/New_York/900/2004	agtgatgccccattccttgatcggcttcgccgagatcag<br>aggtccctaagaggaagaggcaatactc	+	+
CY019161	Human H3N2 IAVs	Human	H3N2		2004	USA	A/New_York/900/2004	agtgatgccccattccttgatcggcttcgccgagatcag<br>aggtccctaagaggaagaggcaatactc	+	+
CY019169	Human H3N2 IAVs	Human	H3N2		2004	USA	A/New_York/900/2004	agtgatgccccattccttgatcggcttcgccgagatcag<br>aggtccctaagaggaagaggcaatactc	+	+
CY006119	Human H3N2 IAVs	Human	H3N2		2004	USA	A/New_York/355/2004	agtgatgccccattccttgatcggcttcgccgagatcag<br>aggtccctaagaggaagaggcaatactc	+	+
CY008168	Human H3N2 IAVs	Human	H3N2		2004	USA	A/New_York/472/2004	agtgatgccccattccttgatcggcttcgccgagatcag<br>aggtccctaagaggaagaggcaatactc	+	+
CY003340	Human H3N2 IAVs	Human	H3N2		2004	USA	A/New_York/354/2004	agtgatgccccattccttgatcggcttcgccgagatcag<br>aggtccctaagaggaagaggcaatactc	+	+
CY002196	Human H3N2 IAVs	Human	H3N2		2004	USA	A/New_York/366/2004	agtgatgccccattccttgatcggcttcgccgagatcag<br>aggtccctaagaggaagaggcaatactc	+	+
CY002604	Human H3N2 IAVs	Human	H3N2		2004	USA	A/New_York/380/2004	agtgatgccccattccttgatcggcttcgccgagatcag<br>aggtccctaagaggaagaggcaatactc	+	+
CY003036	Human H3N2 IAVs	Human	H3N2		2004	USA	A/New_York/351/2004	agtgatgccccattccttgatcggcttcgccgagatcag<br>aggtccctaagaggaagaggcaatactc	+	+
CY002764	Human H3N2 IAVs	Human	H3N2		2004	USA	A/New_York/68/2004	agtgatgccccattccttgatcggcttcgccgagatcag<br>aggtccctaagaggaagaggcaatactc	+	+
CY006375	Human H3N2 IAVs	Human	H3N2		2004	USA	A/New_York/466/2004	agtgatgccccattccttgatcggcttcgccgagatcag<br>aggtccctaagaggaagaggcaatactc	+	+
CY002508	Human H3N2 IAVs	Human	H3N2		2004	USA	A/New_York/218/2004	agtgatgccccattccttgatcggcttcgccgagatcag<br>aggtccctaagaggaagaggcaatactc	+	+
CY009272	Human H3N2 IAVs	Human	H3N2		2004	USA	A/New_York/500/2004	agtgatgccccattccttgatcggcttcgccgagatcag<br>aggtccctaagaggaagaggcaatactc	+	+
CY000261	Human H3N2 IAVs	Human	H3N2		2004	USA	A/New_York/52/2004	agtgatgccccattccttgatcggcttcgccgagatcag<br>aggtccctaaggggaagaggcaacactc	-	-
AM502813	Human H3N2 IAVs	Human	H3N2		2004	United_Kingdom	A/England/4024313/2004	agtgatgccccattccttgatcggcttcgccgagatcag<br>aggtccctaaggggaagaggcaacactc	-	-
CY000765	Human H3N2 IAVs	Human	H3N2		2004	USA	A/New_York/10/2004	agtgatgccccattccttgatcggcttcgccgagatcag<br>aggtccctaaggggaagaggcaacactc	-	-
CY009264	Human H3N2 IAVs	Human	H3N2		2004	USA	A/New_York/499/2004	agtgatgccccattccttgatcggcttcgccgagatcag<br>aggtccctaaggggaagaggcaacactc	-	-
CY000893	Human H3N2 IAVs	Human	H3N2		2004	USA	A/New_York/5/2004	agtgatgccccattccttgatcggcttcgccgagatcag<br>aggtccctaaggggaagaggcaacactc	-	-
CY001033	Human H3N2 IAVs	Human	H3N2		2004	USA	A/New_York/6/2004	agtgatgccccattccttgatcggcttcgccgagatcag<br>aggtccctaaggggaagaggcaacactc	-	-
CY000565	Human H3N2 IAVs	Human	H3N2		2004	USA	A/New_York/69/2004	agtgatgccccattccttgatcggcttcgccgagatcag<br>aggtccctaaggggaagaggcaacactc	-	-
CY090977	Human H3N2 IAVs	Human	H3N2		2004	USA	A/Georgia/NHRC0001/2004	agtgatgccccattccttgatcggcttcgccgagatcag<br>aggtccctaaggggaagaggcaacactc	-	-
CY104144	Human H3N2 IAVs	Human	H3N2		2004	Viet_Nam	A/HaNoi/HN30110/2004	agtgatgccccattccttgatcggcttcgccgagatcag<br>aggtccctaaggggaagaggcaacactc	-	-
CY000037	Human H3N2 IAVs	Human	H3N2		2004	USA	A/New_York/33/2004	agtgatgccccattccttgatcggcttcgccgagatcag<br>aggtccctaaggggaagaggcaacactc	-	-
CY001233	Human H3N2 IAVs	Human	H3N2		2004	USA	A/New_York/70/2004	agtgatgccccattccttgatcggcttcgccgagatcag<br>aggtccctaaggggaagaggcaacactc	-	-
CY009256	Human H3N2 IAVs	Human	H3N2		2004	USA	A/New_York/480/2004	agtgatgccccattccttgatcggcttcgccgagatcag<br>aggtccctaaggggaagaggcaacactc	-	-

CY038571	Human H3N2 IAVs	Human	H3N2		2004	Hong_Kong	A/Hong_Kong/HKU2/2004	agtgatgccccattccttgatcggcttcgccgagatcag<br>aggtccctaaggggaagaggcaacactc	-	-
CY038979	Human H3N2 IAVs	Human	H3N2		2004	Hong_Kong	A/Hong_Kong/HKU10/2004	agtgatgccccattccttgatcggcttcgccgagatcag<br>aggtccctaaggggaagaggcaacactc	-	-
CY038939	Human H3N2 IAVs	Human	H3N2		2004	Hong_Kong	A/Hong_Kong/HKU3/2004	agtgatgccccattccttgatcggcttcgccgagatcag<br>aggtccctaaggggaagaggcaacactc	-	-
CY038579	Human H3N2 IAVs	Human	H3N2		2004	Hong_Kong	A/Hong_Kong/HKU8/2004	agtgatgccccattccttgatcggcttcgccgagatcag<br>aggtccctaaggggaagaggcaacactc	-	-
CY039163	Human H3N2 IAVs	Human	H3N2		2004	Hong_Kong	A/Hong_Kong/HKU5/2004	agtgatgccccattccttgatcggcttcgccgagatcag<br>aggtccctaaggggaagaggcaacactc	-	-
CY038595	Human H3N2 IAVs	Human	H3N2		2004	Hong_Kong	A/Hong_Kong/HKU12/2004	agtgatgccccattccttgatcggcttcgccgagatcag<br>aggtccctaaggggaagaggcaacactc	-	-
CY038963	Human H3N2 IAVs	Human	H3N2		2004	Hong_Kong	A/Hong_Kong/HKU7/2004	agtgatgccccattccttgatcggcttcgccgagatcag<br>aggtccctaaggggaagaggcaacactc	-	-
CY117992	Human H3N2 IAVs	Human	H3N2		2004	Malaysia	A/Malaysia/29997/2004	agtgatgccccattccttgatcggcttcgccgagatcag<br>aggtccctaaggggaagaggcaacactc	-	-
CY019009	Human H3N2 IAVs	Human	H3N2		2004	Australia	A/Queensland/41/2004	agtgatgccccattccttgatcggcttcgccgagatcag<br>aggtccctaaggggaagaggcaacactc	-	-
CY020033	Human H3N2 IAVs	Human	H3N2		2004	Australia	A/Queensland/44/2004	agtgatgccccattccttgatcggcttcgccgagatcag<br>aggtccctaaggggaagaggcaacactc	-	-
CY017599	Human H3N2 IAVs	Human	H3N2		2004	Australia	A/Queensland/48/2004	agtgatgccccattccttgatcggcttcgccgagatcag<br>aggtccctaaggggaagaggcaacactc	-	-
CY015912	Human H3N2 IAVs	Human	H3N2		2004	Australia	A/Western_Australia/55/2004	agtgatgccccattccttgatcggcttcgccgagatcag<br>aggtccctaaggggaagaggcaacactc	-	-
CY015936	Human H3N2 IAVs	Human	H3N2		2004	Australia	A/Western_Australia/58/2004	agtgatgccccattccttgatcggcttcgccgagatcag<br>aggtccctaaggggaagaggcaacactc	-	-
CY017961	Human H3N2 IAVs	Human	H3N2		2004	Australia	A/Queensland/42/2004	agtgatgccccattccttgatcggcttcgccgagatcag<br>aggtccctaaggggaagaggcaacactc	-	-
CY017591	Human H3N2 IAVs	Human	H3N2		2004	Australia	A/Queensland/47/2004	agtgatgccccattccttgatcggcttcgccgagatcag<br>aggtccctaaggggaagaggcaacactc	-	-
CY000373	Human H3N2 IAVs	Human	H3N2		2004	USA	A/New_York/31/2004	agtgatgccccattccttgatcggcttcgccgagatcag<br>aggtccctaaggggaagaggcaacactc	-	-
CY091121	Human H3N2 IAVs	Human	H3N2		2004	USA	A/Missouri/NHRC0002/2004	agtgatgccccattccttgatcggcttcgccgagatcag<br>aggtccctaaggggaagaggcaacactc	-	-
CY105346	Human H3N2 IAVs	Human	H3N2		2004	Viet_Nam	A/HaNoi/BM544/2004	agtgatgccccattccttgatcggcttcgccgagatcag<br>aggtccctaaggggaagaggcaacactc	-	-
CY104136	Human H3N2 IAVs	Human	H3N2		2004	Viet_Nam	A/BacGiang/BG003/2004	agtgatgccccattccttgatcggcttcgccgagatcag<br>aggtccctaaggggaagaggcaatactc	-	-
EU247848	Human H3N2 IAVs	Human	H3N2		2004	USA	A/California/07/2004	agtgatgccccattccttgatcggcttcgccgagatcag<br>aggtccctaaggggaagaggcaatactc	-	-
CY114377	Human H3N2 IAVs	Human	H3N2		2004	USA	A/California/7/2004	agtgatgccccattccttgatcggcttcgccgagatcag<br>aggtccctaaggggaagaggcaatactc	-	-
CY007455	Human H3N2 IAVs	Human	H3N2		2004	New_Zealand	A/Canterbury/104/2004	agtgatgccccattccttgatcggcttcgccgagatcag<br>aggtccctaaggggaagaggcaatactc	-	-
CY007519	Human H3N2 IAVs	Human	H3N2		2004	New_Zealand	A/Canterbury/207/2004	agtgatgccccattccttgatcggcttcgccgagatcag<br>aggtccctaaggggaagaggcaatactc	-	-
CY008256	Human H3N2 IAVs	Human	H3N2		2004	New_Zealand	A/Canterbury/312/2004	agtgatgccccattccttgatcggcttcgccgagatcag<br>aggtccctaaggggaagaggcaatactc	-	-
CY105274	Human H3N2 IAVs	Human	H3N2		2004	Viet_Nam	A/HaNoi/662/2004	agtgatgccccattccttgatcggcttcgccgagatcag<br>aggtccctaaggggaagaggcaatactc	-	-

CY105282	Human H3N2 IAVs	Human	H3N2		2004	Viet_Nam	A/HaNoi/BM769/2004	agtgatgccccattccttgatcggcttcgccgagatcag aggtccctaaggggaagaggcaatactc	-	-
CY104176	Human H3N2 IAVs	Human	H3N2		2004	Viet_Nam	A/HaNoi/HN30108/2004	agtgatgccccattccttgatcggcttcgccgagatcag aggtccctaaggggaagaggcaatactc	-	-
CY104184	Human H3N2 IAVs	Human	H3N2		2004	Viet_Nam	A/Hanoi/HN30109/2004	agtgatgccccattccttgatcggcttcgccgagatcag aggtccctaaggggaagaggcaatactc	-	-
CY104160	Human H3N2 IAVs	Human	H3N2		2004	Viet_Nam	A/HaNoi/HN30127/2004	agtgatgccccattccttgatcggcttcgccgagatcag aggtccctaaggggaagaggcaatactc	-	-
CY105290	Human H3N2 IAVs	Human	H3N2		2004	Viet_Nam	A/HaNoi/HN30130/2004	agtgatgccccattccttgatcggcttcgccgagatcag aggtccctaaggggaagaggcaatactc	-	-
CY105298	Human H3N2 IAVs	Human	H3N2		2004	Viet_Nam	A/Hanoi/HN30135/2004	agtgatgccccattccttgatcggcttcgccgagatcag aggtccctaaggggaagaggcaatactc	-	-
CY105314	Human H3N2 IAVs	Human	H3N2		2004	Viet_Nam	A/HaNoi/HN30147/2004	agtgatgccccattccttgatcggcttcgccgagatcag aggtccctaaggggaagaggcaatactc	-	-
CY105338	Human H3N2 IAVs	Human	H3N2		2004	Viet_Nam	A/Hanoi/HN3094/2004	agtgatgccccattccttgatcggcttcgccgagatcag aggtccctaaggggaagaggcaatactc	-	-
CY038667	Human H3N2 IAVs	Human	H3N2		2004	Hong_Kong	A/Hong_Kong/HKU37/2004	agtgatgccccattccttgatcggcttcgccgagatcag aggtccctaaggggaagaggcaatactc	-	-
CY038955	Human H3N2 IAVs	Human	H3N2		2004	Hong_Kong	A/Hong_Kong/HKU6/2004	agtgatgccccattccttgatcggcttcgccgagatcag aggtccctaaggggaagaggcaatactc	-	-
CY104192	Human H3N2 IAVs	Human	H3N2		2004	Viet_Nam	A/HungYen/HY028/2004	agtgatgccccattccttgatcggcttcgccgagatcag aggtccctaaggggaagaggcaatactc	-	-
CY118726	Human H3N2 IAVs	Human	H3N2		2004	Malaysia	A/Malaysia/26682/2004	agtgatgccccattccttgatcggcttcgccgagatcag aggtccctaaggggaagaggcaatactc	-	-
CY118750	Human H3N2 IAVs	Human	H3N2		2004	Malaysia	A/Malaysia/28114/2004	agtgatgccccattccttgatcggcttcgccgagatcag aggtccctaaggggaagaggcaatactc	-	-
CY118758	Human H3N2 IAVs	Human	H3N2		2004	Malaysia	A/Malaysia/28991/2004	agtgatgccccattccttgatcggcttcgccgagatcag aggtccctaaggggaagaggcaatactc	-	-
CY118774	Human H3N2 IAVs	Human	H3N2		2004	Malaysia	A/Malaysia/29242/2004	agtgatgccccattccttgatcggcttcgccgagatcag aggtccctaaggggaagaggcaatactc	-	-
CY118782	Human H3N2 IAVs	Human	H3N2		2004	Malaysia	A/Malaysia/29255/2004	agtgatgccccattccttgatcggcttcgccgagatcag aggtccctaaggggaagaggcaatactc	-	-
CY002950	Human H3N2 IAVs	Human	H3N2		2004	New_Zealand	A/Christchurch/184/2004	agtgatgccccattccttgatcggcttcgccgagatcag aggtccctaaggggaagaggcaatactc	-	-
CY002966	Human H3N2 IAVs	Human	H3N2		2004	New_Zealand	A/Christchurch/297/2004	agtgatgccccattccttgatcggcttcgccgagatcag aggtccctaaggggaagaggcaatactc	-	-
CY002980	Human H3N2 IAVs	Human	H3N2		2004	New_Zealand	A/Christchurch/339/2004	agtgatgccccattccttgatcggcttcgccgagatcag aggtccctaaggggaagaggcaatactc	-	-
CY039011	Human H3N2 IAVs	Human	H3N2		2004	Hong_Kong	A/Hong_Kong/HKU21/2004	agtgatgccccattccttgatcggcttcgccgagatcag aggtccctaaggggaagaggcaatactc	-	-
CY039043	Human H3N2 IAVs	Human	H3N2		2004	Hong_Kong	A/Hong_Kong/HKU33/2004	agtgatgccccattccttgatcggcttcgccgagatcag aggtccctaaggggaagaggcaatactc	-	-
CY038659	Human H3N2 IAVs	Human	H3N2		2004	Hong_Kong	A/Hong_Kong/HKU36/2004	agtgatgccccattccttgatcggcttcgccgagatcag aggtccctaaggggaagaggcaatactc	-	-
CY091457	Human H3N2 IAVs	Human	H3N2		2004	Malaysia	A/Malaysia/NHRC0001/2004	agtgatgccccattccttgatcggcttcgccgagatcag aggtccctaaggggaagaggcaatactc	-	-
CY091441	Human H3N2 IAVs	Human	H3N2		2004	Malaysia	A/Malaysia/NHRC0003/2004	agtgatgccccattccttgatcggcttcgccgagatcag aggtccctaaggggaagaggcaatactc	-	-
CY038619	Human H3N2 IAVs	Human	H3N2		2004	Hong_Kong	A/Hong_Kong/HKU20/2004	agtgatgccccattccttgatcggcttcgccgagatcag aggtccctaaggggaagaggcaatactc	-	-

CY039499	Human H3N2 IAVs	Human	H3N2		2004	Hong_Kong	A/Hong_Kong/HKU32/2004	agtgatgccccattccttgatcggcttcgccgagatcag<br>aggtccctaaggggaagaggcaatactc	-	-
CY104152	Human H3N2 IAVs	Human	H3N2		2004	Viet_Nam	A/NamDinh/HN30119/2004	agtgatgccccattccttgatcggcttcgccgagatcag<br>aggtccctaaggggaagaggcaatactc	-	-
CY002084	Human H3N2 IAVs	Human	H3N2		2004	USA	A/New_York/244/2004	agtgatgccccattccttgatcggcttcgccgagatcag<br>aggtccctaaggggaagaggcaatactc	-	-
CY002412	Human H3N2 IAVs	Human	H3N2		2004	USA	A/New_York/334/2004	agtgatgccccattccttgatcggcttcgccgagatcag<br>aggtccctaaggggaagaggcaatactc	-	-
CY003044	Human H3N2 IAVs	Human	H3N2		2004	USA	A/New_York/364/2004	agtgatgccccattccttgatcggcttcgccgagatcag<br>aggtccctaaggggaagaggcaatactc	-	-
CY002260	Human H3N2 IAVs	Human	H3N2		2004	USA	A/New_York/383/2004	agtgatgccccattccttgatcggcttcgccgagatcag<br>aggtccctaaggggaagaggcaatactc	-	-
CY002068	Human H3N2 IAVs	Human	H3N2		2004	USA	A/New_York/392/2004	agtgatgccccattccttgatcggcttcgccgagatcag<br>aggtccctaaggggaagaggcaatactc	-	-
CY038995	Human H3N2 IAVs	Human	H3N2		2004	Hong_Kong	A/Hong_Kong/HKU15/2004	agtgatgccccattccttgatcggcttcgccgagatcag<br>aggtccctaaggggaagaggcaatactc	-	-
CY039027	Human H3N2 IAVs	Human	H3N2		2004	Hong_Kong	A/Hong_Kong/HKU25/2004	agtgatgccccattccttgatcggcttcgccgagatcag<br>aggtccctaaggggaagaggcaatactc	-	-
CY040310	Human H3N2 IAVs	Human	H3N2		2004	Hong_Kong	A/Hong_Kong/HKU35/2004	agtgatgccccattccttgatcggcttcgccgagatcag<br>aggtccctaaggggaagaggcaatactc	-	-
KC197836	Human H3N2 IAVs	Swine	H3N2		2004	Sri_Lanka	A/swine/Colombo/48/2004	agtgatgccccattccttgatcggcttcgccgagatcag<br>aggtccctaaggggaagaggcaatactc	-	-
CY104200	Human H3N2 IAVs	Human	H3N2		2004	Viet_Nam	A/TayNguyen/TN46/2004	agtgatgccccattccttgatcggcttcgccgagatcag<br>aggtccctaaggggaagaggcaatactc	-	-
CY104208	Human H3N2 IAVs	Human	H3N2		2004	Viet_Nam	A/TayNguyen/TN49/2004	agtgatgccccattccttgatcggcttcgccgagatcag<br>aggtccctaaggggaagaggcaatactc	-	-
CY013955	Human H3N2 IAVs	Human	H3N2		2004	New_Zealand	A/Waikato/64/2004	agtgatgccccattccttgatcggcttcgccgagatcag<br>aggtccctaaggggaagaggcaatactc	-	-
CY013473	Human H3N2 IAVs	Human	H3N2		2004	New_Zealand	A/Wellington/18/2004	agtgatgccccattccttgatcggcttcgccgagatcag<br>aggtccctaaggggaagaggcaatactc	-	-
CY015576	Human H3N2 IAVs	Human	H3N2		2004	New_Zealand	A/Wellington/34/2004	agtgatgccccattccttgatcggcttcgccgagatcag<br>aggtccctaaggggaagaggcaatactc	-	-
CY015984	Human H3N2 IAVs	Human	H3N2		2004	Australia	A/Western_Australia/64/2004	agtgatgccccattccttgatcggcttcgccgagatcag<br>aggtccctaaggggaagaggcaatactc	-	-
CY013923	Human H3N2 IAVs	Human	H3N2		2004	New_Zealand	A/Waikato/3/2004	agtgatgccccattccttgatcggcttcgccgagatcag<br>aggtccctaaggggaagaggcaatactc	-	-
CY013529	Human H3N2 IAVs	Human	H3N2		2004	New_Zealand	A/Waikato/35/2004	agtgatgccccattccttgatcggcttcgccgagatcag<br>aggtccctaaggggaagaggcaatactc	-	-
CY013947	Human H3N2 IAVs	Human	H3N2		2004	New_Zealand	A/Waikato/56/2004	agtgatgccccattccttgatcggcttcgccgagatcag<br>aggtccctaaggggaagaggcaatactc	-	-
CY012676	Human H3N2 IAVs	Human	H3N2		2004	New_Zealand	A/Wellington/14/2004	agtgatgccccattccttgatcggcttcgccgagatcag<br>aggtccctaaggggaagaggcaatactc	-	-
CY012692	Human H3N2 IAVs	Human	H3N2		2004	New_Zealand	A/Wellington/22/2004	agtgatgccccattccttgatcggcttcgccgagatcag<br>aggtccctaaggggaagaggcaatactc	-	-
CY012700	Human H3N2 IAVs	Human	H3N2		2004	New_Zealand	A/Wellington/31/2004	agtgatgccccattccttgatcggcttcgccgagatcag<br>aggtccctaaggggaagaggcaatactc	-	-
CY013164	Human H3N2 IAVs	Human	H3N2		2004	New_Zealand	A/Wellington/45/2004	agtgatgccccattccttgatcggcttcgccgagatcag<br>aggtccctaaggggaagaggcaatactc	-	-
CY013521	Human H3N2 IAVs	Human	H3N2		2004	New_Zealand	A/Wellington/58/2004	agtgatgccccattccttgatcggcttcgccgagatcag<br>aggtccctaaggggaagaggcaatactc	-	-

CY017969	Human H3N2 IAVs	Human	H3N2		2004	Australia	A/Queensland/43/2004	agtgatgccccattccttgatcggcttcgccgagatcag aggtccctaaggggaagaggcaatactc	-	-
CY020041	Human H3N2 IAVs	Human	H3N2		2004	Australia	A/Queensland/45/2004	agtgatgccccattccttgatcggcttcgccgagatcag aggtccctaaggggaagaggcaatactc	-	-
CY020049	Human H3N2 IAVs	Human	H3N2		2004	Australia	A/Queensland/50/2004	agtgatgccccattccttgatcggcttcgccgagatcag aggtccctaaggggaagaggcaatactc	-	-
CY013971	Human H3N2 IAVs	Human	H3N2		2004	New_Zealand	A/Waikato/69/2004	agtgatgccccattccttgatcggcttcgccgagatcag aggtccctaaggggaagaggcaatactc	-	-
CY013995	Human H3N2 IAVs	Human	H3N2		2004	New_Zealand	A/Waikato/72/2004	agtgatgccccattccttgatcggcttcgccgagatcag aggtccctaaggggaagaggcaatactc	-	-
CY014003	Human H3N2 IAVs	Human	H3N2		2004	New_Zealand	A/Waikato/73/2004	agtgatgccccattccttgatcggcttcgccgagatcag aggtccctaaggggaagaggcaatactc	-	-
CY121484	Human H3N2 IAVs	Human	H3N2		2004	New_Zealand	A/Wellington/01/2004	agtgatgccccattccttgatcggcttcgccgagatcag aggtccctaaggggaagaggcaatactc	-	-
CY013987	Human H3N2 IAVs	Human	H3N2		2004	New_Zealand	A/Wellington/64/2004	agtgatgccccattccttgatcggcttcgccgagatcag aggtccctaaggggaagaggcaatactc	-	-
CY015880	Human H3N2 IAVs	Human	H3N2		2004	Australia	A/Western_Australia/51/2004	agtgatgccccattccttgatcggcttcgccgagatcag aggtccctaaggggaagaggcaatactc	-	-
CY015888	Human H3N2 IAVs	Human	H3N2		2004	Australia	A/Western_Australia/52/2004	agtgatgccccattccttgatcggcttcgccgagatcag aggtccctaaggggaagaggcaatactc	-	-
CY015896	Human H3N2 IAVs	Human	H3N2		2004	Australia	A/Western_Australia/53/2004	agtgatgccccattccttgatcggcttcgccgagatcag aggtccctaaggggaagaggcaatactc	-	-
CY015904	Human H3N2 IAVs	Human	H3N2		2004	Australia	A/Western_Australia/54/2004	agtgatgccccattccttgatcggcttcgccgagatcag aggtccctaaggggaagaggcaatactc	-	-
CY015944	Human H3N2 IAVs	Human	H3N2		2004	Australia	A/Western_Australia/59/2004	agtgatgccccattccttgatcggcttcgccgagatcag aggtccctaaggggaagaggcaatactc	-	-
CY015952	Human H3N2 IAVs	Human	H3N2		2004	Australia	A/Western_Australia/60/2004	agtgatgccccattccttgatcggcttcgccgagatcag aggtccctaaggggaagaggcaatactc	-	-
CY015960	Human H3N2 IAVs	Human	H3N2		2004	Australia	A/Western_Australia/61/2004	agtgatgccccattccttgatcggcttcgccgagatcag aggtccctaaggggaagaggcaatactc	-	-
CY040086	Human H3N2 IAVs	Human	H3N2		2004	Taiwan	A/Taiwan/9/2004	agtgatgccccattccttgatcggcttcgccgagatcag aggtccctaaggggaagaggcaatactc	-	-
CY007295	Human H3N2 IAVs	Human	H3N2		2004	New_Zealand	A/Bay_of_Plenty/279/2004	agtgatgccccattccttgatcggcttcgccgagatcag aggtccctaaggggaagaggcaatactc	-	-
CY007351	Human H3N2 IAVs	Human	H3N2		2004	New_Zealand	A/Canterbury/12/2004	agtgatgccccattccttgatcggcttcgccgagatcag aggtccctaaggggaagaggcaatactc	-	-
CY007527	Human H3N2 IAVs	Human	H3N2		2004	New_Zealand	A/Canterbury/208/2004	agtgatgccccattccttgatcggcttcgccgagatcag aggtccctaaggggaagaggcaatactc	-	-
CY007535	Human H3N2 IAVs	Human	H3N2		2004	New_Zealand	A/Canterbury/209/2004	agtgatgccccattccttgatcggcttcgccgagatcag aggtccctaaggggaagaggcaatactc	-	-
CY007543	Human H3N2 IAVs	Human	H3N2		2004	New_Zealand	A/Canterbury/210/2004	agtgatgccccattccttgatcggcttcgccgagatcag aggtccctaaggggaagaggcaatactc	-	-
CY008232	Human H3N2 IAVs	Human	H3N2		2004	New_Zealand	A/Canterbury/308/2004	agtgatgccccattccttgatcggcttcgccgagatcag aggtccctaaggggaagaggcaatactc	-	-
CY007583	Human H3N2 IAVs	Human	H3N2		2004	New_Zealand	A/Canterbury/315/2004	agtgatgccccattccttgatcggcttcgccgagatcag aggtccctaaggggaagaggcaatactc	-	-
CY002588	Human H3N2 IAVs	Human	H3N2		2004	USA	A/New_York/358/2004	agtgatgccccattccttgatcggcttcgccgagatcag aggtccctaaggggaagaggcaatactc	-	-
CY008520	Human H3N2 IAVs	Human	H3N2		2004	USA	A/New_York/73/2004	agtgatgccccattccttgatcggcttcgccgagatcag aggtccctaaggggaagaggcaatactc	-	-

CY008216	Human H3N2 IAVs	Human	H3N2		2004	New_Zealand	A/Tairawhiti/223/2004	agtgatgccccattccttgatcggcttcgccgagatcag aggtccctaaggggaagaggcaatactc	-	-
CY007311	Human H3N2 IAVs	Human	H3N2		2004	New_Zealand	A/Tairawhiti/369/2004	agtgatgccccattccttgatcggcttcgccgagatcag aggtccctaaggggaagaggcaatactc	-	-
CY013513	Human H3N2 IAVs	Human	H3N2		2004	New_Zealand	A/Waikato/26/2004	agtgatgccccattccttgatcggcttcgccgagatcag aggtccctaaggggaagaggcaatactc	-	-
CY020897	Human H3N2 IAVs	Human	H3N2		2004	New_Zealand	A/Waikato/31/2004	agtgatgccccattccttgatcggcttcgccgagatcag aggtccctaaggggaagaggcaatactc	-	-
CY013537	Human H3N2 IAVs	Human	H3N2		2004	New_Zealand	A/Waikato/43/2004	agtgatgccccattccttgatcggcttcgccgagatcag aggtccctaaggggaagaggcaatactc	-	-
CY013963	Human H3N2 IAVs	Human	H3N2		2004	New_Zealand	A/Waikato/68/2004	agtgatgccccattccttgatcggcttcgccgagatcag aggtccctaaggggaagaggcaatactc	-	-
CY012108	Human H3N2 IAVs	Human	H3N2		2004	New_Zealand	A/Wellington/01/2004	agtgatgccccattccttgatcggcttcgccgagatcag aggtccctaaggggaagaggcaatactc	-	-
CY013156	Human H3N2 IAVs	Human	H3N2		2004	New_Zealand	A/Wellington/35/2004	agtgatgccccattccttgatcggcttcgccgagatcag aggtccctaaggggaagaggcaatactc	-	-
CY013489	Human H3N2 IAVs	Human	H3N2		2004	New_Zealand	A/Wellington/44/2004	agtgatgccccattccttgatcggcttcgccgagatcag aggtccctaaggggaagaggcaatactc	-	-
CY016447	Human H3N2 IAVs	Human	H3N2		2004	New_Zealand	A/Wellington/61/2004	agtgatgccccattccttgatcggcttcgccgagatcag aggtccctaaggggaagaggcaatactc	-	-
CY013553	Human H3N2 IAVs	Human	H3N2		2004	New_Zealand	A/Wellington/62/2004	agtgatgccccattccttgatcggcttcgccgagatcag aggtccctaaggggaagaggcaatactc	-	-
CY002292	Human H3N2 IAVs	Human	H3N2		2004	USA	A/New_York/207/2004	agtgatgccccattccttgatcggcttcgccgagatcag aggtccctaaggggaagaggcaatactc	-	-
CY038971	Human H3N2 IAVs	Human	H3N2		2004	Hong_Kong	A/Hong_Kong/HKU9/2004	agtgatgccccattccttgatcggcttcgccgagatcag aggtccctaaggggaagaggcaatactc	-	-
CY012708	Human H3N2 IAVs	Human	H3N2		2004	New_Zealand	A/Waikato/21/2004	agtgatgccccattccttgatcggcttcgccgagatcag aggtccctaaggggaagaggcaatactc	-	-
CY015920	Human H3N2 IAVs	Human	H3N2		2004	Australia	A/Western_Australia/56/2004	agtgatgccccattccttgatcggcttcgccgagatcag aggtccctaaggggaagaggcaatactc	-	-
CY105322	Human H3N2 IAVs	Human	H3N2		2004	Viet_Nam	A/Hanoi/HN30240/2004	agtgatgccccattccttgatcggcttcgccgagatcag aggtccctaaggggaagaggcaatactc	-	-
CY015568	Human H3N2 IAVs	Human	H3N2		2004	New_Zealand	A/Waikato/16/2004	agtgatgccccattccttgatcggcttcgccgagatcag aggtccctaaggggaagaggcaatactc	-	-
CY091449	Human H3N2 IAVs	Human	H3N2		2004	Malaysia	A/Malaysia/NHRC0002/2004	agtgatgccccattccttgatcggcttcgccgagatcag aggtccctaaggggaagaggcaatactc	-	-
CY015968	Human H3N2 IAVs	Human	H3N2		2004	Australia	A/Western_Australia/62/2004	agtgatgccccattccttgatcggcttcgccgagatcag aggtccctaaggggaagaggcaatactc	-	-
CY118806	Human H3N2 IAVs	Human	H3N2		2004	Malaysia	A/Malaysia/29711/2004	agtgatgccccattccttgatcggcttcgccgagatcag aggtccctaaggggaagaggcaatactc	-	-
CY038587	Human H3N2 IAVs	Human	H3N2		2004	Hong_Kong	A/Hong_Kong/HKU11/2004	agtgatgccccattccttgatcggcttcgccgagatcag aggtccctaaggggaagaggcaatactc	-	-
CY118798	Human H3N2 IAVs	Human	H3N2		2004	Malaysia	A/Malaysia/29361/2004	agtgatgccccattccttgatcggcttcgccgagatcag aggtccctaaggggaagaggcaatactc	-	-
CY007303	Human H3N2 IAVs	Human	H3N2		2004	New_Zealand	A/Bay_of_Plenty/332/2004	agtgatgccccattccttgatcggcttcgccgagatcag aggtccctaaggggaagaggcaatactc	-	-
CY007431	Human H3N2 IAVs	Human	H3N2		2004	New_Zealand	A/Canterbury/101/2004	agtgatgccccattccttgatcggcttcgccgagatcag aggtccctaaggggaagaggcaatactc	-	-
CY013939	Human H3N2 IAVs	Human	H3N2		2004	New_Zealand	A/Waikato/51/2004	agtgatgccccattccttgatcggcttcgccgagatcag aggtccctaaggggaagaggcaatactc	-	-

CY013931	Human H3N2 IAVs	Human	H3N2		2004	New_Zealand	A/Wellington/59/2004	agtgatgccccattccttgatcggcttcgccgagatcag aggtccctaaggggaagaggcaatactc	-	-
CY008208	Human H3N2 IAVs	Human	H3N2		2004	New_Zealand	A/Whanganui/69/2004	agtgatgccccattccttgatcggcttcgccgagatcag aggtccctaaggggaagaggcaatactc	-	-
CY105306	Human H3N2 IAVs	Human	H3N2		2004	Viet_Nam	A/HaNoi/HN30137/2004	agtgatgccccattccttgatcggcttcgccgagatcag aggtccctaaggggaagaggcaatactc	-	-
CY020345	Human H3N2 IAVs	Human	H3N2		2004	New_Zealand	A/Wellington/55/2004	agtgatgccccattccttgatcggcttcgccgagatcag aggtccctaaggggaagaggcaatactc	-	-
CY039491	Human H3N2 IAVs	Human	H3N2		2004	Hong_Kong	A/Hong_Kong/HKU1/2004	agtgatgccccattccttgatcggcttcgccgagatcag aggtccctaaggggaagaggcaatactc	-	-
CY039171	Human H3N2 IAVs	Human	H3N2		2004	Hong_Kong	A/Hong_Kong/HKU24/2004	agtgatgccccattccttgatcggcttcgccgagatcag aggtccctaaggggaagaggcaatactc	-	-
CY015928	Human H3N2 IAVs	Human	H3N2		2004	Australia	A/Western_Australia/57/2004	agtgatgccccattccttgatcggcttcgccgagatcag aggtccctaaggggaagaggcaatactc	-	-
CY118814	Human H3N2 IAVs	Human	H3N2		2004	Malaysia	A/Malaysia/29890/2004	agtgatgccccattccttgatcggcttcgccgagatcag aggtccctaaggggaagaggcaatactc	-	-
CY038627	Human H3N2 IAVs	Human	H3N2		2004	Hong_Kong	A/Hong_Kong/HKU22/2004	agtgatgccccattccttgatcggcttcgccgagatcag aggtccctaaggggaagaggcaatactc	-	-
CY118742	Human H3N2 IAVs	Human	H3N2		2004	Malaysia	A/Malaysia/26989/2004	agtgatgccccattccttgatcggcttcgccgagatcag aggtccctaaggggaagaggcaatactc	-	-
CY040302	Human H3N2 IAVs	Human	H3N2		2004	Hong_Kong	A/Hong_Kong/HKU18/2004	agtgatgccccattccttgatcggcttcgccgagatcag aggtccctaaggggaagaggcaatactc	-	-
CY017977	Human H3N2 IAVs	Human	H3N2		2004	Australia	A/Queensland/46/2004	agtgatgccccattccttgatcggcttcgccgagatcag aggtccctaaggggaagaggcaatactc	-	-
CY017607	Human H3N2 IAVs	Human	H3N2		2004	Australia	A/Queensland/49/2004	agtgatgccccattccttgatcggcttcgccgagatcag aggtccctaaggggaagaggcaatactc	-	-
CY118822	Human H3N2 IAVs	Human	H3N2		2004	Malaysia	A/Malaysia/29930/2004	agtgatgccccattccttgatcggcttcgccgagatcag aggtccctaaggggaagaggcaatactc	-	-
AB761210	Human H3N2 IAVs	Human	H3N2		2004	Japan	A/Hyogo/36/2004	agtgatgccccattccttgatcggcttcgccgagatcag aggtccctaaggggaagaggcaatactc	-	-
CY013497	Human H3N2 IAVs	Human	H3N2		2004	New_Zealand	A/Wellington/38/2004	agtgatgccccattccttgatcggcttcgccgagatcag aggtccctaaggggaagaggcaatactc	-	-
CY013979	Human H3N2 IAVs	Human	H3N2		2004	New_Zealand	A/Waikato/71/2004	agtgatgccccattccttgatcggcttcgccgagatcag aggtccctaaggggaagaggcaatactc	-	-
CY007319	Human H3N2 IAVs	Human	H3N2		2004	New_Zealand	A/Bay_of_Plenty/383/2004	agtgatgccccattccttgatcggcttcgccgagatcag aggtccctaaggggaagaggcaatactc	-	-
DQ415369	Human H3N2 IAVs	Human	H3N2		2004	Taiwan	A/TW/220/04	agtgatgccccattccttgatcggcttcgccgagatcag aggtccctaaggggaagaggcaatactc	-	-
CY013457	Human H3N2 IAVs	Human	H3N2		2004	New_Zealand	A/Wellington/23/2004	agtgatgccccattccttgatcggcttcgccgagatcag aggtccctaaggggaagaggcaatactc	-	-
CY038611	Human H3N2 IAVs	Human	H3N2		2004	Hong_Kong	A/Hong_Kong/HKU19/2004	agtgatgccccattccttgatcggcttcgccgagatcag aggtccctaaggggaagaggcaatactc	-	-
CY038643	Human H3N2 IAVs	Human	H3N2		2004	Hong_Kong	A/Hong_Kong/HKU31/2004	agtgatgccccattccttgatcggcttcgccgagatcag aggtccctaaggggaagaggcaatactc	-	-
CY038675	Human H3N2 IAVs	Human	H3N2		2004	Hong_Kong	A/Hong_Kong/HKU39/2004	agtgatgccccattccttgatcggcttcgccgagatcag aggtccctaaggggaagaggcaatactc	-	-
CY039019	Human H3N2 IAVs	Human	H3N2		2004	Hong_Kong	A/Hong_Kong/HKU23/2004	agtgatgccccattccttgatcggcttcgccgagatcag aggtccctaaggggaagaggcaatactc	-	-
CY012716	Human H3N2 IAVs	Human	H3N2		2004	New_Zealand	A/Wellington/52/2004	agtgatgccccattccttgatcggcttcgccgagatcag aggtccctaaggggaagaggcaatactc	-	-

CY008240	Human H3N2 IAVs	Human	H3N2		2004	New_Zealand	A/Canterbury/309/2004	agtgatgccccattccttgatcggcttcgccgagatcag<br>aggtccctaaggggaagaggcaatactc	-	-
CY013545	Human H3N2 IAVs	Human	H3N2		2004	New_Zealand	A/Waikato/45/2004	agtgatgccccattccttgatcggcttcgccgagatcag<br>aggtccctaaggggaagaggcaatactc	-	-
CY007423	Human H3N2 IAVs	Human	H3N2		2004	New_Zealand	A/Canterbury/100/2004	agtgatgccccattccttgatcggcttcgccgagatcag<br>aggtccctaaggggaagaggcaatactc	-	-
CY007439	Human H3N2 IAVs	Human	H3N2		2004	New_Zealand	A/Canterbury/102/2004	agtgatgccccattccttgatcggcttcgccgagatcag<br>aggtccctaaggggaagaggcaatactc	-	-
CY007447	Human H3N2 IAVs	Human	H3N2		2004	New_Zealand	A/Canterbury/103/2004	agtgatgccccattccttgatcggcttcgccgagatcag<br>aggtccctaaggggaagaggcaatactc	-	-
CY007463	Human H3N2 IAVs	Human	H3N2		2004	New_Zealand	A/Canterbury/105/2004	agtgatgccccattccttgatcggcttcgccgagatcag<br>aggtccctaaggggaagaggcaatactc	-	-
CY007479	Human H3N2 IAVs	Human	H3N2		2004	New_Zealand	A/Canterbury/107/2004	agtgatgccccattccttgatcggcttcgccgagatcag<br>aggtccctaaggggaagaggcaatactc	-	-
CY007991	Human H3N2 IAVs	Human	H3N2		2004	New_Zealand	A/Canterbury/108/2004	agtgatgccccattccttgatcggcttcgccgagatcag<br>aggtccctaaggggaagaggcaatactc	-	-
CY007999	Human H3N2 IAVs	Human	H3N2		2004	New_Zealand	A/Canterbury/109/2004	agtgatgccccattccttgatcggcttcgccgagatcag<br>aggtccctaaggggaagaggcaatactc	-	-
CY007503	Human H3N2 IAVs	Human	H3N2		2004	New_Zealand	A/Canterbury/205/2004	agtgatgccccattccttgatcggcttcgccgagatcag<br>aggtccctaaggggaagaggcaatactc	-	-
CY007407	Human H3N2 IAVs	Human	H3N2		2004	New_Zealand	A/Canterbury/23/2004	agtgatgccccattccttgatcggcttcgccgagatcag<br>aggtccctaaggggaagaggcaatactc	-	-
CY007415	Human H3N2 IAVs	Human	H3N2		2004	New_Zealand	A/Canterbury/24/2004	agtgatgccccattccttgatcggcttcgccgagatcag<br>aggtccctaaggggaagaggcaatactc	-	-
CY016455	Human H3N2 IAVs	Human	H3N2		2004	New_Zealand	A/Waikato/70/2004	agtgatgccccattccttgatcggcttcgccgagatcag<br>aggtccctaaggggaagaggcaatactc	-	-
CY015976	Human H3N2 IAVs	Human	H3N2		2004	Australia	A/Western_Australia/63/2004	agtgatgccccattccttgatcggcttcgccgagatcag<br>aggtccctaaggggaagaggcaatactc	-	-
CY007343	Human H3N2 IAVs	Human	H3N2		2004	New_Zealand	A/Canterbury/11/2004	agtgatgccccattccttgatcggcttcgccgagatcag<br>aggtccctaaggggaagaggcaatactc	-	-
CY007359	Human H3N2 IAVs	Human	H3N2		2004	New_Zealand	A/Canterbury/16/2004	agtgatgccccattccttgatcggcttcgccgagatcag<br>aggtccctaaggggaagaggcaatactc	-	-
CY007367	Human H3N2 IAVs	Human	H3N2		2004	New_Zealand	A/Canterbury/17/2004	agtgatgccccattccttgatcggcttcgccgagatcag<br>aggtccctaaggggaagaggcaatactc	-	-
CY007375	Human H3N2 IAVs	Human	H3N2		2004	New_Zealand	A/Canterbury/18/2004	agtgatgccccattccttgatcggcttcgccgagatcag<br>aggtccctaaggggaagaggcaatactc	-	-
CY007383	Human H3N2 IAVs	Human	H3N2		2004	New_Zealand	A/Canterbury/19/2004	agtgatgccccattccttgatcggcttcgccgagatcag<br>aggtccctaaggggaagaggcaatactc	-	-
CY007391	Human H3N2 IAVs	Human	H3N2		2004	New_Zealand	A/Canterbury/20/2004	agtgatgccccattccttgatcggcttcgccgagatcag<br>aggtccctaaggggaagaggcaatactc	-	-
CY096847	Human H3N2 IAVs	Human	H3N2		2004	New_Zealand	A/Canterbury/200/2004	agtgatgccccattccttgatcggcttcgccgagatcag<br>aggtccctaaggggaagaggcaatactc	-	-
CY007487	Human H3N2 IAVs	Human	H3N2		2004	New_Zealand	A/Canterbury/201/2004	agtgatgccccattccttgatcggcttcgccgagatcag<br>aggtccctaaggggaagaggcaatactc	-	-
CY007511	Human H3N2 IAVs	Human	H3N2		2004	New_Zealand	A/Canterbury/206/2004	agtgatgccccattccttgatcggcttcgccgagatcag<br>aggtccctaaggggaagaggcaatactc	-	-
CY007399	Human H3N2 IAVs	Human	H3N2		2004	New_Zealand	A/Canterbury/21/2004	agtgatgccccattccttgatcggcttcgccgagatcag<br>aggtccctaaggggaagaggcaatactc	-	-
CY007551	Human H3N2 IAVs	Human	H3N2		2004	New_Zealand	A/Canterbury/303/2004	agtgatgccccattccttgatcggcttcgccgagatcag<br>aggtccctaaggggaagaggcaatactc	-	-

CY002926	Human H3N2 IAVs	Human	H3N2		2004	New_Zealand	A/Christchurch/14/2004	agtgatgccccattccttgatcggcttcgccgagatcagaggtccctaaggggaagaggcaatactc	-	-
CY002934	Human H3N2 IAVs	Human	H3N2		2004	New_Zealand	A/Christchurch/89/2004	agtgatgccccattccttgatcggcttcgccgagatcagaggtccctaaggggaagaggcaatactc	-	-
CY009928	Human H3N2 IAVs	Human	H3N2		2004	New_Zealand	A/Whanganui/127/2004	agtgatgccccattccttgatcggcttcgccgagatcagaggtccctaaggggaagaggcaatactc	-	-
CY007279	Human H3N2 IAVs	Human	H3N2		2004	New_Zealand	A/Whanganui/128/2004	agtgatgccccattccttgatcggcttcgccgagatcagaggtccctaaggggaagaggcaatactc	-	-
CY007287	Human H3N2 IAVs	Human	H3N2		2004	New_Zealand	A/Whanganui/129/2004	agtgatgccccattccttgatcggcttcgccgagatcagaggtccctaaggggaagaggcaatactc	-	-
CY007335	Human H3N2 IAVs	Human	H3N2		2004	New_Zealand	A/Whanganui/417/2004	agtgatgccccattccttgatcggcttcgccgagatcagaggtccctaaggggaagaggcaatactc	-	-
CY002942	Human H3N2 IAVs	Human	H3N2		2004	New_Zealand	A/Christchurch/90/2004	agtgatgccccattccttgatcggcttcgccgagatcagaggtccctaaggggaagaggcaatactc	-	-
CY007327	Human H3N2 IAVs	Human	H3N2		2004	New_Zealand	A/Whanganui/386/2004	agtgatgccccattccttgatcggcttcgccgagatcagaggtccctaaggggaagaggcaatactc	-	-
CY007559	Human H3N2 IAVs	Human	H3N2		2004	New_Zealand	A/Canterbury/304/2004	agtgatgccccattccttgatcggcttcgccgagatcagaggtccctaaggggaagaggcaatactc	-	-
CY007495	Human H3N2 IAVs	Human	H3N2		2004	New_Zealand	A/Canterbury/202/2004	agtgatgccccattccttgatcggcttcgccgagatcagaggtccctaaggggaagaggcaatactc	-	-
CY008248	Human H3N2 IAVs	Human	H3N2		2004	New_Zealand	A/Canterbury/310/2004	agtgatgccccattccttgatcggcttcgccgagatcagaggtccctaaggggaagaggcaatactc	-	-
CY039984	Human H3N2 IAVs	Human	H3N2		2004	New_Zealand	A/Canterbury/200/2004	agtgatgccccattccttgatcggcttcgccgagatcagaggtccctaaggggaagaggcaatactc	-	-
CY007567	Human H3N2 IAVs	Human	H3N2		2004	New_Zealand	A/Canterbury/311/2004	agtgatgccccattccttgatcggcttcgccgagatcagaggtccctaaggggaagaggcaatactc	-	-
CY039035	Human H3N2 IAVs	Human	H3N2		2004	Hong_Kong	A/Hong_Kong/HKU30/2004	agtgatgccccattccttgatcggcttcgccgagatcagaggtccctaaggggaagaggcaatactc	-	-
CY013465	Human H3N2 IAVs	Human	H3N2		2004	New_Zealand	A/Waikato/12/2004	agtgatgccccattccttgatcggcttcgccgagatcagaggtccctaaggggaagaggcaatactc	-	-
CY013505	Human H3N2 IAVs	Human	H3N2		2004	New_Zealand	A/Waikato/22/2004	agtgatgccccattccttgatcggcttcgccgagatcagaggtccctaaggggaagaggcaatactc	-	-
CY012724	Human H3N2 IAVs	Human	H3N2		2004	New_Zealand	A/Waikato/40/2004	agtgatgccccattccttgatcggcttcgccgagatcagaggtccctaaggggaagaggcaatactc	-	-
CY118790	Human H3N2 IAVs	Human	H3N2		2004	Malaysia	A/Malaysia/29350/2004	agtgatgccccattccttgatcggcttcgccgagatcagaggtccctaaggggaagaggcaatactc	-	-
CY002052	Human H3N2 IAVs	Human	H3N2		2004	USA	A/New_York/387/2004	agtgatgccccattccttgatcggcttcgccgagatcagaggtccctaaggggaagaggcaatactc	-	-
CY043748	Human H3N2 IAVs	Human	H3N2		2004	Hong_Kong	A/Hong_Kong/HKU28/2004	agtgatgccccattccttgatcggcttcgccgagatcagaggtccctaaggggaagaggcaatactc	-	-
CY038635	Human H3N2 IAVs	Human	H3N2		2004	Hong_Kong	A/Hong_Kong/HKU29/2004	agtgatgccccattccttgatcggcttcgccgagatcagaggtccctaaggggaagaggcaatactc	-	-
CY038987	Human H3N2 IAVs	Human	H3N2		2004	Hong_Kong	A/Hong_Kong/HKU14/2004	agtgatgccccattccttgatcggcttcgccgagatcagaggtccctaaggggaagaggcaatactc	-	-
CY038651	Human H3N2 IAVs	Human	H3N2		2004	Hong_Kong	A/Hong_Kong/HKU34/2004	agtgatgccccattccttgatcggcttcgccgagatcagaggtccctaaggggaagaggcaatactc	-	-
DQ415370	Human H3N2 IAVs	Human	H3N2		2004	Taiwan	A/TW/875/04	agtgatgccccattccttgatcggcttcgccgagatcagaggtccctaaggggaagaggcaatactc	-	-
CY039051	Human H3N2 IAVs	Human	H3N2		2004	Hong_Kong	A/Hong_Kong/HKU40/2004	agtgatgccccattccttgatcggcttcgccgagatcagaggtccctaaggggaagaggcaatactc	-	-

CY112993	Human H3N2 IAVs	Human	H3N2		2004	Netherlands	A/Netherlands/132/2004	agtgatgccccattccttgatcggcttcgccgagatcag<br>aggtccctaaggggaagaggcaatactc	-	-
CY040318	Human H3N2 IAVs	Human	H3N2		2004	Hong_Kong	A/Hong_Kong/HKU38/2004	agtgatgccccattccttgatcggcttcgccgagatcag<br>aggtccctaaggggaagaggcaatactc	-	-
CY118734	Human H3N2 IAVs	Human	H3N2		2004	Malaysia	A/Malaysia/26822/2004	agtgatgccccattccttgatcggcttcgccgagatcag<br>aggtccctaaggggaagaggcaatactc	-	-
DQ415368	Human H3N2 IAVs	Human	H3N2		2004	Taiwan	A/TW/36/04	agtgatgccccattccttgatcggcttcgccgagatcag<br>aggtccctaaggggaagaggcaatactc	-	-
CY002918	Human H3N2 IAVs	Human	H3N2		2004	New_Zealand	A/Christchurch/15/2004	agtgatgccccattccttgatcggcttcgccgagatcag<br>aggtccctaaggggaagaggcaatactc	-	-
CY012116	Human H3N2 IAVs	Human	H3N2		2004	New_Zealand	A/Wellington/6/2004	agtgatgccccattccttgatcggcttcgccgagatcag<br>aggtccctaaggggaagaggcaatactc	-	-
CY118766	Human H3N2 IAVs	Human	H3N2		2004	Malaysia	A/Malaysia/29197/2004	agtgatgccccattccttgatcggcttcgccgagatcag<br>aggtccctaaggggaagaggcaatactc	-	-
CY038603	Human H3N2 IAVs	Human	H3N2		2004	Hong_Kong	A/Hong_Kong/HKU16/2004	agtgatgccccattccttgatcggcttcgccgagatcag<br>aggtccctaaggggaagaggcaatactc	-	-
CY038947	Human H3N2 IAVs	Human	H3N2		2004	Hong_Kong	A/Hong_Kong/HKU4/2004	agtgatgccccattccttgatcggcttcgccgagatcag<br>aggtccctaaggggaagaggcaatactc	-	-
CY039003	Human H3N2 IAVs	Human	H3N2		2004	Hong_Kong	A/Hong_Kong/HKU17/2004	agtgatgccccattccttgatcggcttcgccgagatcag<br>aggtccctaaggggaagaggcaatactc	-	-
CY008912	Human H3N2 IAVs	Human	H3N2		2004	USA	A/New_York/498/2004	agtgatgccccattccttgatcggcttcgccgggatcag<br>aggtccctaaggggaagaggcaacactc	-	-
CY002028	Human H3N2 IAVs	Human	H3N2		2004	USA	A/New_York/379/2004	agtgattccccattccttgatcggcttcgccgagatcag<br>aggtccctaaggggaagaggcaatactc	-	-
CY091137	Human H3N2 IAVs	Human	H3N2		2005	USA	A/Texas/NHRC0001/2005	agcgatgccccattccttgatcggcttcgccgagatca<br>gaggtccctaagaggaagaggcaatgctc	+	+
FJ769881	Human H3N2 IAVs	Human	H3N2		2005	Iran	A/Tabriz/12/2005	agtgatgccccattccttgatcggctccgccgagatca<br>gaggtccctaagaggaagaggcaatactc	-	*
KJ855431	Human H3N2 IAVs	Human	H3N2		2005	Mexico	A/Mexico/QUE2270/2005	agtgatgccccattccttgatcggcttcgcagagatca<br>gaggtccctaaggggaagaggcaatactc	+	+
CY014019	Human H3N2 IAVs	Human	H3N2		2005	New_Zealand	A/Waikato/1/2005	agtgatgccccattccttgatcggcttcgcagagatca<br>gaggtccctaaggggaaggggcaatactc	-	-
CY015592	Human H3N2 IAVs	Human	H3N2		2005	New_Zealand	A/Waikato/2/2005	agtgatgccccattccttgatcggcttcgcagagatca<br>gaggtccctaaggggaaggggcaatactc	-	-
CY105890	Human H3N2 IAVs	Human	H3N2		2005	Viet_Nam	A/HaNoi/311/2005	agtgatgccccattccttgatcggcttcgccgagatca<br>aaggtccctaaggggaagaggcaatactc	-	-
CY104216	Human H3N2 IAVs	Human	H3N2		2005	Viet_Nam	A/HaNoi/ISBM16/2005	agtgatgccccattccttgatcggcttcgccgagatca<br>aaggtccctaaggggaagaggcaatactc	-	-
CY003052	Human H3N2 IAVs	Human	H3N2		2005	USA	A/New_York/376/2005	agtgatgccccattccttgatcggcttcgccgagatcag<br>aggtccctaagaggaagaggcaatactc	+	+
CY091001	Human H3N2 IAVs	Human	H3N2		2005	USA	A/South_Carolina/NHRC0002/200<br>5	agtgatgccccattccttgatcggcttcgccgagatcag<br>aggtccctaagaggaagaggcaatactc	+	+
CY002020	Human H3N2 IAVs	Human	H3N2		2005	USA	A/New_York/378/2005	agtgatgccccattccttgatcggcttcgccgagatcag<br>aggtccctaagaggaagaggcaatactc	+	+
CY003644	Human H3N2 IAVs	Human	H3N2		2005	USA	A/New_York/463/2005	agtgatgccccattccttgatcggcttcgccgagatcag<br>aggtccctaagaggaagaggcaatactc	+	+
CY003652	Human H3N2 IAVs	Human	H3N2		2005	USA	A/New_York/464/2005	agtgatgccccattccttgatcggcttcgccgagatcag<br>aggtccctaagaggaagaggcaatactc	+	+
CY003348	Human H3N2 IAVs	Human	H3N2		2005	USA	A/New_York/389/2005	agtgatgccccattccttgatcggcttcgccgagatcag<br>aggtccctaagaggaagaggcaatactc	+	+

CY006088	Human H3N2 IAVs	Human	H3N2		2005	USA	A/New_York/465/2005	agtgatgccccattccttgatcggcttcgccgagatcag aggtccctaagaggaagaggcaatactc	+	+
CY019289	Human H3N2 IAVs	Human	H3N2		2005	USA	A/New_York/913/2005	agtgatgccccattccttgatcggcttcgccgagatcag aggtccctaagaggaagaggcaatactc	+	+
CY019273	Human H3N2 IAVs	Human	H3N2		2005	USA	A/New_York/913/2005	agtgatgccccattccttgatcggcttcgccgagatcag aggtccctaagaggaagaggcaatactc	+	+
CY020057	Human H3N2 IAVs	Human	H3N2		2005	USA	A/New_York/918/2005	agtgatgccccattccttgatcggcttcgccgagatcag aggtccctaagaggaagaggcaatactc	+	+
CY019305	Human H3N2 IAVs	Human	H3N2		2005	USA	A/New_York/918/2005	agtgatgccccattccttgatcggcttcgccgagatcag aggtccctaagaggaagaggcaatactc	+	+
CY019321	Human H3N2 IAVs	Human	H3N2		2005	USA	A/New_York/918/2005	agtgatgccccattccttgatcggcttcgccgagatcag aggtccctaagaggaagaggcaatactc	+	+
CY020537	Human H3N2 IAVs	Human	H3N2		2005	USA	A/New_York/913/2005	agtgatgccccattccttgatcggcttcgccgagatcag aggtccctaagaggaagaggcaatactc	+	+
CY019281	Human H3N2 IAVs	Human	H3N2		2005	USA	A/New_York/913/2005	agtgatgccccattccttgatcggcttcgccgagatcag aggtccctaagaggaagaggcaatactc	+	+
CY019297	Human H3N2 IAVs	Human	H3N2		2005	USA	A/New_York/913/2005	agtgatgccccattccttgatcggcttcgccgagatcag aggtccctaagaggaagaggcaatactc	+	+
CY019313	Human H3N2 IAVs	Human	H3N2		2005	USA	A/New_York/918/2005	agtgatgccccattccttgatcggcttcgccgagatcag aggtccctaagaggaagaggcaatactc	+	+
CY019193	Human H3N2 IAVs	Human	H3N2		2005	USA	A/New_York/918/2005	agtgatgccccattccttgatcggcttcgccgagatcag aggtccctaagaggaagaggcaatactc	+	+
CY002484	Human H3N2 IAVs	Human	H3N2		2005	USA	A/New_York/390/2005	agtgatgccccattccttgatcggcttcgccgagatcag aggtccctaagaggaagaggcaatactc	+	+
CY002012	Human H3N2 IAVs	Human	H3N2		2005	USA	A/New_York/361/2005	agtgatgccccattccttgatcggcttcgccgagatcag aggtccctaagaggaagaggcaatactc	+	+
CY003060	Human H3N2 IAVs	Human	H3N2		2005	USA	A/New_York/395/2005	agtgatgccccattccttgatcggcttcgccgagatcag aggtccctaagaggaagaggcaatactc	+	+
CY002244	Human H3N2 IAVs	Human	H3N2		2005	USA	A/New_York/375/2005	agtgatgccccattccttgatcggcttcgccgagatcag aggtccctaagaggaagaggcaatactc	+	+
CY002268	Human H3N2 IAVs	Human	H3N2		2005	USA	A/New_York/388/2005	agtgatgccccattccttgatcggcttcgccgagatcag aggtccctaagaggaagaggcaatactc	+	+
CY002492	Human H3N2 IAVs	Human	H3N2		2005	USA	A/New_York/393/2005	agtgatgccccattccttgatcggcttcgccgagatcag aggtccctaagaggaagaggcaatactc	+	+
CY006159	Human H3N2 IAVs	Human	H3N2		2005	USA	A/New_York/258/2005	agtgatgccccattccttgatcggcttcgccgagatcag aggtccctaagaggaagaggcaatactc	+	+
CY002724	Human H3N2 IAVs	Human	H3N2		2005	USA	A/New_York/352/2005	agtgatgccccattccttgatcggcttcgccgagatcag aggtccctaagaggaagaggcaatactc	+	+
CY006127	Human H3N2 IAVs	Human	H3N2		2005	USA	A/New_York/191/2005	agtgatgccccattccttgatcggcttcgccgagatcag aggtccctaagaggaagaggcaatactc	+	+
CY002204	Human H3N2 IAVs	Human	H3N2		2005	USA	A/New_York/369/2005	agtgatgccccattccttgatcggcttcgccgagatcag aggtccctaagaggaagaggcaatactc	+	+
CY002452	Human H3N2 IAVs	Human	H3N2		2005	USA	A/New_York/367/2005	agtgatgccccattccttgatcggcttcgccgagatcag aggtccctaagaggaagaggcaatactc	+	+
CY006143	Human H3N2 IAVs	Human	H3N2		2005	USA	A/New_York/238/2005	agtgatgccccattccttgatcggcttcgccgagatcag aggtccctaagaggaagaggcaatactc	+	+
CY014035	Human H3N2 IAVs	Human	H3N2		2005	New_Zealand	A/Wellington/2/2005	agtgatgccccattccttgatcggcttcgccgagatcag aggtccctaagaggaagaggcaatactc	+	+
CY014051	Human H3N2 IAVs	Human	H3N2		2005	New_Zealand	A/Wellington/3/2005	agtgatgccccattccttgatcggcttcgccgagatcag aggtccctaagaggaagaggcaatactc	+	+

FJ769882	Human H3N2 IAVs	Human	H3N2		2005	Iran	A/Babol/36/2005	agtgatgccccattccttgatcggcttcgccgagatcag aggtccctaagaggaagaggcaatactc	+	+
CY002076	Human H3N2 IAVs	Human	H3N2		2005	USA	A/New_York/396/2005	agtgatgccccattccttgatcggcttcgccgagatcag aggtccctaagaggaagaggcaatactc	+	+
CY002188	Human H3N2 IAVs	Human	H3N2		2005	USA	A/New_York/357/2005	agtgatgccccattccttgatcggcttcgccgagatcag aggtccctaagaggaagaggcaatactc	+	+
CY006295	Human H3N2 IAVs	Human	H3N2		2005	USA	A/New_York/462/2005	agtgatgccccattccttgatcggcttcgccgagatcag aggtccctaagaggaagaggcaatactc	+	+
CY002780	Human H3N2 IAVs	Human	H3N2		2005	USA	A/New_York/210/2005	agtgatgccccattccttgatcggcttcgccgagatcag aggtccctaagaggaagaggcaatactc	+	+
CY002060	Human H3N2 IAVs	Human	H3N2		2005	USA	A/New_York/391/2005	agtgatgccccattccttgatcggcttcgccgagatcag aggtccctaagaggaagaggcaatactc	+	+
CY008360	Human H3N2 IAVs	Human	H3N2		2005	New_Zealand	A/Canterbury/104/2005	agtgatgccccattccttgatcggcttcgccgagatcag aggtccctaagaggaagaggcaatactc	+	+
CY008560	Human H3N2 IAVs	Human	H3N2		2005	New_Zealand	A/Canterbury/105/2005	agtgatgccccattccttgatcggcttcgccgagatcag aggtccctaagaggaagaggcaatactc	+	+
EU620746	Human H3N2 IAVs	Swine	H3N2		2005	China	A/swine/Guangdong/04/2005	agtgatgccccattccttgatcggcttcgccgagatcag aggtccctaaggggaagaggcaacactc	-	-
KJ855447	Human H3N2 IAVs	Human	H3N2		2005	Mexico	A/Mexico/MEX2640/2005	agtgatgccccattccttgatcggcttcgccgagatcag aggtccctaaggggaagaggcaatactc	-	-
CY022601	Human H3N2 IAVs	Human	H3N2		2005	New_Zealand	A/Auckland/621/2005	agtgatgccccattccttgatcggcttcgccgagatcag aggtccctaaggggaagaggcaatactc	-	-
CY091553	Human H3N2 IAVs	Human	H3N2		2005	USA	A/California/NHRC0009/2005	agtgatgccccattccttgatcggcttcgccgagatcag aggtccctaaggggaagaggcaatactc	-	-
CY091129	Human H3N2 IAVs	Human	H3N2		2005	USA	A/Georgia/NHRC0001/2005	agtgatgccccattccttgatcggcttcgccgagatcag aggtccctaaggggaagaggcaatactc	-	-
CY104264	Human H3N2 IAVs	Human	H3N2		2005	Viet_Nam	A/HaNoi/Q372/2005	agtgatgccccattccttgatcggcttcgccgagatcag aggtccctaaggggaagaggcaatactc	-	-
CY039507	Human H3N2 IAVs	Human	H3N2		2005	Hong_Kong	A/Hong_Kong/HKU50/2005	agtgatgccccattccttgatcggcttcgccgagatcag aggtccctaaggggaagaggcaatactc	-	-
CY038691	Human H3N2 IAVs	Human	H3N2		2005	Hong_Kong	A/Hong_Kong/HKU51/2005	agtgatgccccattccttgatcggcttcgccgagatcag aggtccctaaggggaagaggcaatactc	-	-
CY039195	Human H3N2 IAVs	Human	H3N2		2005	Hong_Kong	A/Hong_Kong/HKU55/2005	agtgatgccccattccttgatcggcttcgccgagatcag aggtccctaaggggaagaggcaatactc	-	-
CY039515	Human H3N2 IAVs	Human	H3N2		2005	Hong_Kong	A/Hong_Kong/HKU56/2005	agtgatgccccattccttgatcggcttcgccgagatcag aggtccctaaggggaagaggcaatactc	-	-
CY039251	Human H3N2 IAVs	Human	H3N2		2005	Hong_Kong	A/Hong_Kong/HKU66/2005	agtgatgccccattccttgatcggcttcgccgagatcag aggtccctaaggggaagaggcaatactc	-	-
CY118838	Human H3N2 IAVs	Human	H3N2		2005	Malaysia	A/Malaysia/31998/2005	agtgatgccccattccttgatcggcttcgccgagatcag aggtccctaaggggaagaggcaatactc	-	-
CY092261	Human H3N2 IAVs	Human	H3N2		2005	USA	A/California/NHRC0010/2005	agtgatgccccattccttgatcggcttcgccgagatcag aggtccctaaggggaagaggcaatactc	-	-
CY039067	Human H3N2 IAVs	Human	H3N2		2005	Hong_Kong	A/Hong_Kong/HKU47/2005	agtgatgccccattccttgatcggcttcgccgagatcag aggtccctaaggggaagaggcaatactc	-	-
CY043756	Human H3N2 IAVs	Human	H3N2		2005	Hong_Kong	A/Hong_Kong/HKU49/2005	agtgatgccccattccttgatcggcttcgccgagatcag aggtccctaaggggaagaggcaatactc	-	-
CY040326	Human H3N2 IAVs	Human	H3N2		2005	Hong_Kong	A/Hong_Kong/HKU52/2005	agtgatgccccattccttgatcggcttcgccgagatcag aggtccctaaggggaagaggcaatactc	-	-
CY039227	Human H3N2 IAVs	Human	H3N2		2005	Hong_Kong	A/Hong_Kong/HKU61/2005	agtgatgccccattccttgatcggcttcgccgagatcag aggtccctaaggggaagaggcaatactc	-	-

CY043764	Human H3N2 IAVs	Human	H3N2		2005	Hong_Kong	A/Hong_Kong/HKU72/2005	agtgatgccccattccttgatcggcttcgccgagatcag aggtccctaaggggaagaggcaatactc	-	-
CY118854	Human H3N2 IAVs	Human	H3N2		2005	Malaysia	A/Malaysia/32133/2005	agtgatgccccattccttgatcggcttcgccgagatcag aggtccctaaggggaagaggcaatactc	-	-
AB434125	Human H3N2 IAVs	Human	H3N2		2005	Japan	A/Morioka/35/2005	agtgatgccccattccttgatcggcttcgccgagatcag aggtccctaaggggaagaggcaatactc	-	-
AB434126	Human H3N2 IAVs	Human	H3N2		2005	Japan	A/Morioka/38/2005	agtgatgccccattccttgatcggcttcgccgagatcag aggtccctaaggggaagaggcaatactc	-	-
CY038699	Human H3N2 IAVs	Human	H3N2		2005	Hong_Kong	A/Hong_Kong/HKU62/2005	agtgatgccccattccttgatcggcttcgccgagatcag aggtccctaaggggaagaggcaatactc	-	-
CY038747	Human H3N2 IAVs	Human	H3N2		2005	Hong_Kong	A/Hong_Kong/HKU71/2005	agtgatgccccattccttgatcggcttcgccgagatcag aggtccctaaggggaagaggcaatactc	-	-
CY002004	Human H3N2 IAVs	Human	H3N2		2005	USA	A/New_York/359/2005	agtgatgccccattccttgatcggcttcgccgagatcag aggtccctaaggggaagaggcaatactc	-	-
CY002468	Human H3N2 IAVs	Human	H3N2		2005	USA	A/New_York/384/2005	agtgatgccccattccttgatcggcttcgccgagatcag aggtccctaaggggaagaggcaatactc	-	-
CY006080	Human H3N2 IAVs	Human	H3N2		2005	USA	A/New_York/461/2005	agtgatgccccattccttgatcggcttcgccgagatcag aggtccctaaggggaagaggcaatactc	-	-
CY039211	Human H3N2 IAVs	Human	H3N2		2005	Hong_Kong	A/Hong_Kong/HKU58/2005	agtgatgccccattccttgatcggcttcgccgagatcag aggtccctaaggggaagaggcaatactc	-	-
CY039219	Human H3N2 IAVs	Human	H3N2		2005	Hong_Kong	A/Hong_Kong/HKU59/2005	agtgatgccccattccttgatcggcttcgccgagatcag aggtccctaaggggaagaggcaatactc	-	-
CY038707	Human H3N2 IAVs	Human	H3N2		2005	Hong_Kong	A/Hong_Kong/HKU65/2005	agtgatgccccattccttgatcggcttcgccgagatcag aggtccctaaggggaagaggcaatactc	-	-
CY038715	Human H3N2 IAVs	Human	H3N2		2005	Hong_Kong	A/Hong_Kong/HKU67/2005	agtgatgccccattccttgatcggcttcgccgagatcag aggtccctaaggggaagaggcaatactc	-	-
CY038723	Human H3N2 IAVs	Human	H3N2		2005	Hong_Kong	A/Hong_Kong/HKU68/2005	agtgatgccccattccttgatcggcttcgccgagatcag aggtccctaaggggaagaggcaatactc	-	-
CY105370	Human H3N2 IAVs	Human	H3N2		2005	Viet_Nam	A/TayNguyen/TN343/2005	agtgatgccccattccttgatcggcttcgccgagatcag aggtccctaaggggaagaggcaatactc	-	-
CY104232	Human H3N2 IAVs	Human	H3N2		2005	Viet_Nam	A/TayNguyen/TN346/2005	agtgatgccccattccttgatcggcttcgccgagatcag aggtccctaaggggaagaggcaatactc	-	-
CY104240	Human H3N2 IAVs	Human	H3N2		2005	Viet_Nam	A/TayNguyen/TN360/2005	agtgatgccccattccttgatcggcttcgccgagatcag aggtccctaaggggaagaggcaatactc	-	-
CY105386	Human H3N2 IAVs	Human	H3N2		2005	Viet_Nam	A/TayNguyen/TN362/2005	agtgatgccccattccttgatcggcttcgccgagatcag aggtccctaaggggaagaggcaatactc	-	-
CY105394	Human H3N2 IAVs	Human	H3N2		2005	Viet_Nam	A/TayNguyen/TN363/2005	agtgatgccccattccttgatcggcttcgccgagatcag aggtccctaaggggaagaggcaatactc	-	-
CY105402	Human H3N2 IAVs	Human	H3N2		2005	Viet_Nam	A/TayNguyen/TN367/2005	agtgatgccccattccttgatcggcttcgccgagatcag aggtccctaaggggaagaggcaatactc	-	-
CY105410	Human H3N2 IAVs	Human	H3N2		2005	Viet_Nam	A/TayNguyen/TN368/2005	agtgatgccccattccttgatcggcttcgccgagatcag aggtccctaaggggaagaggcaatactc	-	-
CY105466	Human H3N2 IAVs	Human	H3N2		2005	Viet_Nam	A/TayNguyen/TN410/2005	agtgatgccccattccttgatcggcttcgccgagatcag aggtccctaaggggaagaggcaatactc	-	-
CY114385	Human H3N2 IAVs	Human	H3N2		2005	USA	A/Wisconsin/67/2005	agtgatgccccattccttgatcggcttcgccgagatcag aggtccctaaggggaagaggcaatactc	-	-
CY163908	Human H3N2 IAVs	Human	H3N2		2005	USA	A/Wisconsin/67/2005	agtgatgccccattccttgatcggcttcgccgagatcag aggtccctaaggggaagaggcaatactc	-	-
CY163972	Human H3N2 IAVs	Human	H3N2		2005	USA	A/Wisconsin/67/2005	agtgatgccccattccttgatcggcttcgccgagatcag aggtccctaaggggaagaggcaatactc	-	-

CY006151	Human H3N2 IAVs	Human	H3N2		2005	USA	A/New_York/243/2005	agtgatgccccattccttgatcggcttcgccgagatcag<br>aggtccctaaggggaagaggcaatactc	-	-
CY014043	Human H3N2 IAVs	Human	H3N2		2005	New_Zealand	A/Otago/1/2005	agtgatgccccattccttgatcggcttcgccgagatcag<br>aggtccctaaggggaagaggcaatactc	-	-
CY015992	Human H3N2 IAVs	Human	H3N2		2005	Australia	A/Western_Australia/65/2005	agtgatgccccattccttgatcggcttcgccgagatcag<br>aggtccctaaggggaagaggcaatactc	-	-
CY020009	Human H3N2 IAVs	Human	H3N2		2005	Australia	A/Western_Australia/71/2005	agtgatgccccattccttgatcggcttcgccgagatcag<br>aggtccctaaggggaagaggcaatactc	-	-
CY017103	Human H3N2 IAVs	Human	H3N2		2005	Australia	A/Western_Australia/76/2005	agtgatgccccattccttgatcggcttcgccgagatcag<br>aggtccctaaggggaagaggcaatactc	-	-
CY163692	Human H3N2 IAVs	Human	H3N2		2005	USA	A/Wisconsin/67/2005	agtgatgccccattccttgatcggcttcgccgagatcag<br>aggtccctaaggggaagaggcaatactc	-	-
CY163668	Human H3N2 IAVs	Human	H3N2		2005	USA	A/Wisconsin/67/2005	agtgatgccccattccttgatcggcttcgccgagatcag<br>aggtccctaaggggaagaggcaatactc	-	-
CY164052	Human H3N2 IAVs	Human	H3N2		2005	USA	A/Wisconsin/67/2005	agtgatgccccattccttgatcggcttcgccgagatcag<br>aggtccctaaggggaagaggcaatactc	-	-
CY163964	Human H3N2 IAVs	Human	H3N2		2005	USA	A/Wisconsin/67/2005	agtgatgccccattccttgatcggcttcgccgagatcag<br>aggtccctaaggggaagaggcaatactc	-	-
CY164076	Human H3N2 IAVs	Human	H3N2		2005	USA	A/Wisconsin/67/2005	agtgatgccccattccttgatcggcttcgccgagatcag<br>aggtccctaaggggaagaggcaatactc	-	-
CY163932	Human H3N2 IAVs	Human	H3N2		2005	USA	A/Wisconsin/67/2005	agtgatgccccattccttgatcggcttcgccgagatcag<br>aggtccctaaggggaagaggcaatactc	-	-
CY164012	Human H3N2 IAVs	Human	H3N2		2005	USA	A/Wisconsin/67/2005	agtgatgccccattccttgatcggcttcgccgagatcag<br>aggtccctaaggggaagaggcaatactc	-	-
CY163988	Human H3N2 IAVs	Human	H3N2		2005	USA	A/Wisconsin/67/2005	agtgatgccccattccttgatcggcttcgccgagatcag<br>aggtccctaaggggaagaggcaatactc	-	-
CY163724	Human H3N2 IAVs	Human	H3N2		2005	USA	A/Wisconsin/67/2005	agtgatgccccattccttgatcggcttcgccgagatcag<br>aggtccctaaggggaagaggcaatactc	-	-
CY163732	Human H3N2 IAVs	Human	H3N2		2005	USA	A/Wisconsin/67/2005	agtgatgccccattccttgatcggcttcgccgagatcag<br>aggtccctaaggggaagaggcaatactc	-	-
CY163956	Human H3N2 IAVs	Human	H3N2		2005	USA	A/Wisconsin/67/2005	agtgatgccccattccttgatcggcttcgccgagatcag<br>aggtccctaaggggaagaggcaatactc	-	-
CY164060	Human H3N2 IAVs	Human	H3N2		2005	USA	A/Wisconsin/67/2005	agtgatgccccattccttgatcggcttcgccgagatcag<br>aggtccctaaggggaagaggcaatactc	-	-
CY164068	Human H3N2 IAVs	Human	H3N2		2005	USA	A/Wisconsin/67/2005	agtgatgccccattccttgatcggcttcgccgagatcag<br>aggtccctaaggggaagaggcaatactc	-	-
CY163740	Human H3N2 IAVs	Human	H3N2		2005	USA	A/Wisconsin/67/2005	agtgatgccccattccttgatcggcttcgccgagatcag<br>aggtccctaaggggaagaggcaatactc	-	-
CY163700	Human H3N2 IAVs	Human	H3N2		2005	USA	A/Wisconsin/67/2005	agtgatgccccattccttgatcggcttcgccgagatcag<br>aggtccctaaggggaagaggcaatactc	-	-
CY163916	Human H3N2 IAVs	Human	H3N2		2005	USA	A/Wisconsin/67/2005	agtgatgccccattccttgatcggcttcgccgagatcag<br>aggtccctaaggggaagaggcaatactc	-	-
CY164108	Human H3N2 IAVs	Human	H3N2		2005	USA	A/Wisconsin/67/2005	agtgatgccccattccttgatcggcttcgccgagatcag<br>aggtccctaaggggaagaggcaatactc	-	-
CY163900	Human H3N2 IAVs	Human	H3N2		2005	USA	A/Wisconsin/67/2005	agtgatgccccattccttgatcggcttcgccgagatcag<br>aggtccctaaggggaagaggcaatactc	-	-
CY163660	Human H3N2 IAVs	Human	H3N2		2005	USA	A/Wisconsin/67/2005	agtgatgccccattccttgatcggcttcgccgagatcag<br>aggtccctaaggggaagaggcaatactc	-	-
CY164092	Human H3N2 IAVs	Human	H3N2		2005	USA	A/Wisconsin/67/2005	agtgatgccccattccttgatcggcttcgccgagatcag<br>aggtccctaaggggaagaggcaatactc	-	-

CY163876	Human H3N2 IAVs	Human	H3N2		2005	USA	A/Wisconsin/67/2005	agtgatgccccattccttgatcggcttcgccgagatcag aggtccctaaggggaagaggcaatactc	-	-
CY164028	Human H3N2 IAVs	Human	H3N2		2005	USA	A/Wisconsin/67/2005	agtgatgccccattccttgatcggcttcgccgagatcag aggtccctaaggggaagaggcaatactc	-	-
CY163652	Human H3N2 IAVs	Human	H3N2		2005	USA	A/Wisconsin/67/2005	agtgatgccccattccttgatcggcttcgccgagatcag aggtccctaaggggaagaggcaatactc	-	-
CY164004	Human H3N2 IAVs	Human	H3N2		2005	USA	A/Wisconsin/67/2005	agtgatgccccattccttgatcggcttcgccgagatcag aggtccctaaggggaagaggcaatactc	-	-
CY164084	Human H3N2 IAVs	Human	H3N2		2005	USA	A/Wisconsin/67/2005	agtgatgccccattccttgatcggcttcgccgagatcag aggtccctaaggggaagaggcaatactc	-	-
CY163748	Human H3N2 IAVs	Human	H3N2		2005	USA	A/Wisconsin/67/2005	agtgatgccccattccttgatcggcttcgccgagatcag aggtccctaaggggaagaggcaatactc	-	-
CY163684	Human H3N2 IAVs	Human	H3N2		2005	USA	A/Wisconsin/67/2005	agtgatgccccattccttgatcggcttcgccgagatcag aggtccctaaggggaagaggcaatactc	-	-
CY164036	Human H3N2 IAVs	Human	H3N2		2005	USA	A/Wisconsin/67/2005	agtgatgccccattccttgatcggcttcgccgagatcag aggtccctaaggggaagaggcaatactc	-	-
CY163716	Human H3N2 IAVs	Human	H3N2		2005	USA	A/Wisconsin/67/2005	agtgatgccccattccttgatcggcttcgccgagatcag aggtccctaaggggaagaggcaatactc	-	-
CY163892	Human H3N2 IAVs	Human	H3N2		2005	USA	A/Wisconsin/67/2005	agtgatgccccattccttgatcggcttcgccgagatcag aggtccctaaggggaagaggcaatactc	-	-
CY163708	Human H3N2 IAVs	Human	H3N2		2005	USA	A/Wisconsin/67/2005	agtgatgccccattccttgatcggcttcgccgagatcag aggtccctaaggggaagaggcaatactc	-	-
CY164100	Human H3N2 IAVs	Human	H3N2		2005	USA	A/Wisconsin/67/2005	agtgatgccccattccttgatcggcttcgccgagatcag aggtccctaaggggaagaggcaatactc	-	-
CY163924	Human H3N2 IAVs	Human	H3N2		2005	USA	A/Wisconsin/67/2005	agtgatgccccattccttgatcggcttcgccgagatcag aggtccctaaggggaagaggcaatactc	-	-
CY163940	Human H3N2 IAVs	Human	H3N2		2005	USA	A/Wisconsin/67/2005	agtgatgccccattccttgatcggcttcgccgagatcag aggtccctaaggggaagaggcaatactc	-	-
CY164044	Human H3N2 IAVs	Human	H3N2		2005	USA	A/Wisconsin/67/2005	agtgatgccccattccttgatcggcttcgccgagatcag aggtccctaaggggaagaggcaatactc	-	-
CY163980	Human H3N2 IAVs	Human	H3N2		2005	USA	A/Wisconsin/67/2005	agtgatgccccattccttgatcggcttcgccgagatcag aggtccctaaggggaagaggcaatactc	-	-
CY163996	Human H3N2 IAVs	Human	H3N2		2005	USA	A/Wisconsin/67/2005	agtgatgccccattccttgatcggcttcgccgagatcag aggtccctaaggggaagaggcaatactc	-	-
CY163884	Human H3N2 IAVs	Human	H3N2		2005	USA	A/Wisconsin/67/2005	agtgatgccccattccttgatcggcttcgccgagatcag aggtccctaaggggaagaggcaatactc	-	-
CY163948	Human H3N2 IAVs	Human	H3N2		2005	USA	A/Wisconsin/67/2005	agtgatgccccattccttgatcggcttcgccgagatcag aggtccctaaggggaagaggcaatactc	-	-
CY008087	Human H3N2 IAVs	Human	H3N2		2005	New_Zealand	A/Canterbury/166/2005	agtgatgccccattccttgatcggcttcgccgagatcag aggtccctaaggggaagaggcaatactc	-	-
CY008568	Human H3N2 IAVs	Human	H3N2		2005	New_Zealand	A/Canterbury/205/2005	agtgatgccccattccttgatcggcttcgccgagatcag aggtccctaaggggaagaggcaatactc	-	-
CY008095	Human H3N2 IAVs	Human	H3N2		2005	New_Zealand	A/Canterbury/206/2005	agtgatgccccattccttgatcggcttcgccgagatcag aggtccctaaggggaagaggcaatactc	-	-
CY008384	Human H3N2 IAVs	Human	H3N2		2005	New_Zealand	A/Canterbury/220/2005	agtgatgccccattccttgatcggcttcgccgagatcag aggtccctaaggggaagaggcaatactc	-	-
CY008584	Human H3N2 IAVs	Human	H3N2		2005	New_Zealand	A/Canterbury/233/2005	agtgatgccccattccttgatcggcttcgccgagatcag aggtccctaaggggaagaggcaatactc	-	-
CY008392	Human H3N2 IAVs	Human	H3N2		2005	New_Zealand	A/Canterbury/234/2005	agtgatgccccattccttgatcggcttcgccgagatcag aggtccctaaggggaagaggcaatactc	-	-

CY008408	Human H3N2 IAVs	Human	H3N2		2005	New_Zealand	A/Canterbury/236/2005	agtgatgccccattccttgatcggcttcgccgagatcag<br>aggtccctaagggaagaggcaatactc	-	-
CY008416	Human H3N2 IAVs	Human	H3N2		2005	New_Zealand	A/Canterbury/237/2005	agtgatgccccattccttgatcggcttcgccgagatcag<br>aggtccctaagggaagaggcaatactc	-	-
CY008424	Human H3N2 IAVs	Human	H3N2		2005	New_Zealand	A/Canterbury/238/2005	agtgatgccccattccttgatcggcttcgccgagatcag<br>aggtccctaagggaagaggcaatactc	-	-
CY008432	Human H3N2 IAVs	Human	H3N2		2005	New_Zealand	A/Canterbury/242/2005	agtgatgccccattccttgatcggcttcgccgagatcag<br>aggtccctaagggaagaggcaatactc	-	-
CY008111	Human H3N2 IAVs	Human	H3N2		2005	New_Zealand	A/Canterbury/259/2005	agtgatgccccattccttgatcggcttcgccgagatcag<br>aggtccctaagggaagaggcaatactc	-	-
CY008055	Human H3N2 IAVs	Human	H3N2		2005	New_Zealand	A/Canterbury/29/2005	agtgatgccccattccttgatcggcttcgccgagatcag<br>aggtccctaagggaagaggcaatactc	-	-
CY007967	Human H3N2 IAVs	Human	H3N2		2005	New_Zealand	A/Canterbury/33/2005	agtgatgccccattccttgatcggcttcgccgagatcag<br>aggtccctaagggaagaggcaatactc	-	-
CY164020	Human H3N2 IAVs	Human	H3N2		2005	USA	A/Wisconsin/67/2005	agtgatgccccattccttgatcggcttcgccgagatcag<br>aggtccctaagggaagaggcaatactc	-	-
CY002460	Human H3N2 IAVs	Human	H3N2		2005	USA	A/New_York/373/2005	agtgatgccccattccttgatcggcttcgccgagatcag<br>aggtccctaagggaagaggcaatactc	-	-
FJ769883	Human H3N2 IAVs	Human	H3N2		2005	Iran	A/Babol/37/2005	agtgatgccccattccttgatcggcttcgccgagatcag<br>aggtccctaagggaagaggcaatactc	-	-
CY040334	Human H3N2 IAVs	Human	H3N2		2005	Hong_Kong	A/Hong_Kong/HKU53/2005	agtgatgccccattccttgatcggcttcgccgagatcag<br>aggtccctaagggaagaggcaatactc	-	-
CY039075	Human H3N2 IAVs	Human	H3N2		2005	Hong_Kong	A/Hong_Kong/HKU54/2005	agtgatgccccattccttgatcggcttcgccgagatcag<br>aggtccctaagggaagaggcaatactc	-	-
CY118830	Human H3N2 IAVs	Human	H3N2		2005	Malaysia	A/Malaysia/31944/2005	agtgatgccccattccttgatcggcttcgccgagatcag<br>aggtccctaagggaagaggcaatactc	-	-
CY044401	Human H3N2 IAVs	Human	H3N2		2005	Hong_Kong	A/Hong_Kong/HKU73/2005	agtgatgccccattccttgatcggcttcgccgagatcag<br>aggtccctaagggaagaggcaatactc	-	-
CY017135	Human H3N2 IAVs	Human	H3N2		2005	Australia	A/South_Australia/22/2005	agtgatgccccattccttgatcggcttcgccgagatcag<br>aggtccctaagggaagaggcaatactc	-	-
CY105426	Human H3N2 IAVs	Human	H3N2		2005	Viet_Nam	A/TayNguyen/TN388/2005	agtgatgccccattccttgatcggcttcgccgagatcag<br>aggtccctaagggaagaggcaatactc	-	-
CY118870	Human H3N2 IAVs	Human	H3N2		2005	Malaysia	A/Malaysia/33386/2005	agtgatgccccattccttgatcggcttcgccgagatcag<br>aggtccctaagggaagaggcaatactc	-	-
CY105450	Human H3N2 IAVs	Human	H3N2		2005	Viet_Nam	A/TayNguyen/TN405/2005	agtgatgccccattccttgatcggcttcgccgagatcag<br>aggtccctaagggaagaggcaatactc	-	-
CY008632	Human H3N2 IAVs	Human	H3N2		2005	New_Zealand	A/Canterbury/257/2005	agtgatgccccattccttgatcggcttcgccgagatcag<br>aggtccctaagggaagaggcaatactc	-	-
CY014075	Human H3N2 IAVs	Human	H3N2		2005	New_Zealand	A/Waikato/7/2005	agtgatgccccattccttgatcggcttcgccgagatcag<br>aggtccctaagggaagaggcaatactc	-	-
CY014067	Human H3N2 IAVs	Human	H3N2		2005	New_Zealand	A/Waikato/8/2005	agtgatgccccattccttgatcggcttcgccgagatcag<br>aggtccctaagggaagaggcaatactc	-	-
CY091505	Human H3N2 IAVs	Human	H3N2		2005	USA	A/California/NHRC0003/2005	agtgatgccccattccttgatcggcttcgccgagatcag<br>aggtccctaagggaagaggcaatactc	-	-
CY091521	Human H3N2 IAVs	Human	H3N2		2005	USA	A/California/NHRC0005/2005	agtgatgccccattccttgatcggcttcgccgagatcag<br>aggtccctaagggaagaggcaatactc	-	-
KJ855423	Human H3N2 IAVs	Human	H3N2		2005	Mexico	A/Mexico/DIF2246/2005	agtgatgccccattccttgatcggcttcgccgagatcag<br>aggtccctaagggaagaggcaatactc	-	-
CY100582	Human H3N2 IAVs	Human	H3N2		2005	Mexico	A/Mexico/InDRE2246/2005	agtgatgccccattccttgatcggcttcgccgagatcag<br>aggtccctaagggaagaggcaatactc	-	-

CY105458	Human H3N2 IAVs	Human	H3N2		2005	Viet_Nam	A/TayNguyen/TN406/2005	agtgatgccccattccttgatcggcttcgccgagatcag aggtccctaaggggaagaggcaatactc	-	-
KJ855407	Human H3N2 IAVs	Human	H3N2		2005	Mexico	A/Mexico/DIF2112/2005	agtgatgccccattccttgatcggcttcgccgagatcag aggtccctaaggggaagaggcaatactc	-	-
CY100550	Human H3N2 IAVs	Human	H3N2		2005	Mexico	A/Mexico/InDRE2112/2005	agtgatgccccattccttgatcggcttcgccgagatcag aggtccctaaggggaagaggcaatactc	-	-
CY100574	Human H3N2 IAVs	Human	H3N2		2005	Mexico	A/Mexico/InDRE2227/2005	agtgatgccccattccttgatcggcttcgccgagatcag aggtccctaaggggaagaggcaatactc	-	-
KJ855479	Human H3N2 IAVs	Human	H3N2		2005	Mexico	A/Mexico/TLA2227/2005	agtgatgccccattccttgatcggcttcgccgagatcag aggtccctaaggggaagaggcaatactc	-	-
CY113001	Human H3N2 IAVs	Human	H3N2		2005	Netherlands	A/Netherlands/548/2005	agtgatgccccattccttgatcggcttcgccgagatcag aggtccctaaggggaagaggcaatactc	-	-
CY091513	Human H3N2 IAVs	Human	H3N2		2005	USA	A/California/NHRC0004/2005	agtgatgccccattccttgatcggcttcgccgagatcag aggtccctaaggggaagaggcaatactc	-	-
CY090985	Human H3N2 IAVs	Human	H3N2		2005	USA	A/Missouri/NHRC0001/2005	agtgatgccccattccttgatcggcttcgccgagatcag aggtccctaaggggaagaggcaatactc	-	-
CY091025	Human H3N2 IAVs	Human	H3N2		2005	USA	A/Texas/NHRC0002/2005	agtgatgccccattccttgatcggcttcgccgagatcag aggtccctaaggggaagaggcaatactc	-	-
CY017993	Human H3N2 IAVs	Human	H3N2		2005	Australia	A/Queensland/52/2005	agtgatgccccattccttgatcggcttcgccgagatcag aggtccctaaggggaagaggcaatactc	-	-
CY017985	Human H3N2 IAVs	Human	H3N2		2005	Australia	A/Queensland/51/2005	agtgatgccccattccttgatcggcttcgccgagatcag aggtccctaaggggaagaggcaatactc	-	-
CY020305	Human H3N2 IAVs	Human	H3N2		2005	Australia	A/South_Australia/61/2005	agtgatgccccattccttgatcggcttcgccgagatcag aggtccctaaggggaagaggcaatactc	-	-
KJ855439	Human H3N2 IAVs	Human	H3N2		2005	Mexico	A/Mexico/DIF2601/2005	agtgatgccccattccttgatcggcttcgccgagatcag aggtccctaaggggaagaggcaatactc	-	-
CY105474	Human H3N2 IAVs	Human	H3N2		2005	Viet_Nam	A/TayNguyen/TN332/2005	agtgatgccccattccttgatcggcttcgccgagatcag aggtccctaaggggaagaggcaatactc	-	-
CY091313	Human H3N2 IAVs	Human	H3N2		2005	Hong_Kong	A/Hong_Kong/NHRC0001/2005	agtgatgccccattccttgatcggcttcgccgagatcag aggtccctaaggggaagaggcaatactc	-	-
CY105362	Human H3N2 IAVs	Human	H3N2		2005	Viet_Nam	A/TayNguyen/TN335/2005	agtgatgccccattccttgatcggcttcgccgagatcag aggtccctaaggggaagaggcaatactc	-	-
CY015600	Human H3N2 IAVs	Human	H3N2		2005	New_Zealand	A/Waikato/3/2005	agtgatgccccattccttgatcggcttcgccgagatcag aggtccctaaggggaagaggcaatactc	-	-
CY104272	Human H3N2 IAVs	Human	H3N2		2005	Viet_Nam	A/HaNoi/Q427/2005	agtgatgccccattccttgatcggcttcgccgagatcag aggtccctaaggggaagaggcaatactc	-	-
CY118862	Human H3N2 IAVs	Human	H3N2		2005	Malaysia	A/Malaysia/32226/2005	agtgatgccccattccttgatcggcttcgccgagatcag aggtccctaaggggaagaggcaatactc	-	-
CY017111	Human H3N2 IAVs	Human	H3N2		2005	Australia	A/Western_Australia/79/2005	agtgatgccccattccttgatcggcttcgccgagatcag aggtccctaaggggaagaggcaatactc	-	-
CY118846	Human H3N2 IAVs	Human	H3N2		2005	Malaysia	A/Malaysia/32171/2005	agtgatgccccattccttgatcggcttcgccgagatcag aggtccctaaggggaagaggcaatactc	-	-
CY039243	Human H3N2 IAVs	Human	H3N2		2005	Hong_Kong	A/Hong_Kong/HKU64/2005	agtgatgccccattccttgatcggcttcgccgagatcag aggtccctaaggggaagaggcaatactc	-	-
CY018929	Human H3N2 IAVs	Human	H3N2		2005	Australia	A/Western_Australia/67/2005	agtgatgccccattccttgatcggcttcgccgagatcag aggtccctaaggggaagaggcaatactc	-	-
CY039187	Human H3N2 IAVs	Human	H3N2		2005	Hong_Kong	A/Hong_Kong/HKU48/2005	agtgatgccccattccttgatcggcttcgccgagatcag aggtccctaaggggaagaggcaatactc	-	-
CY038731	Human H3N2 IAVs	Human	H3N2		2005	Hong_Kong	A/Hong_Kong/HKU69/2005	agtgatgccccattccttgatcggcttcgccgagatcag aggtccctaaggggaagaggcaatactc	-	-

CY040358	Human H3N2 IAVs	Human	H3N2		2005	Hong_Kong	A/Hong_Kong/HKU77/2005	agtgatgccccattccttgatcggcttcgccgagatcag aggtccctaaggggaagaggcaatactc	-	-
CY017623	Human H3N2 IAVs	Human	H3N2		2005	Australia	A/Queensland/57/2005	agtgatgccccattccttgatcggcttcgccgagatcag aggtccctaaggggaagaggcaatactc	-	-
CY091425	Human H3N2 IAVs	Human	H3N2		2005	Australia	A/Australia/NHRC0001/2005	agtgatgccccattccttgatcggcttcgccgagatcag aggtccctaaggggaagaggcaatactc	-	-
CY091417	Human H3N2 IAVs	Human	H3N2		2005	Australia	A/Australia/NHRC0002/2005	agtgatgccccattccttgatcggcttcgccgagatcag aggtccctaaggggaagaggcaatactc	-	-
CY091409	Human H3N2 IAVs	Human	H3N2		2005	Australia	A/Australia/NHRC0003/2005	agtgatgccccattccttgatcggcttcgccgagatcag aggtccctaaggggaagaggcaatactc	-	-
CY091401	Human H3N2 IAVs	Human	H3N2		2005	Australia	A/Australia/NHRC0004/2005	agtgatgccccattccttgatcggcttcgccgagatcag aggtccctaaggggaagaggcaatactc	-	-
CY091393	Human H3N2 IAVs	Human	H3N2		2005	Australia	A/Australia/NHRC0005/2005	agtgatgccccattccttgatcggcttcgccgagatcag aggtccctaaggggaagaggcaatactc	-	-
CY007959	Human H3N2 IAVs	Human	H3N2		2005	New_Zealand	A/Canterbury/16/2005	agtgatgccccattccttgatcggcttcgccgagatcag aggtccctaaggggaagaggcaatactc	-	-
CY008656	Human H3N2 IAVs	Human	H3N2		2005	New_Zealand	A/Canterbury/270/2005	agtgatgccccattccttgatcggcttcgccgagatcag aggtccctaaggggaagaggcaatactc	-	-
CY014131	Human H3N2 IAVs	Human	H3N2		2005	New_Zealand	A/Otago/3/2005	agtgatgccccattccttgatcggcttcgccgagatcag aggtccctaaggggaagaggcaatactc	-	-
CY014123	Human H3N2 IAVs	Human	H3N2		2005	New_Zealand	A/Otago/4/2005	agtgatgccccattccttgatcggcttcgccgagatcag aggtccctaaggggaagaggcaatactc	-	-
CY016599	Human H3N2 IAVs	Human	H3N2		2005	Australia	A/South_Australia/18/2005	agtgatgccccattccttgatcggcttcgccgagatcag aggtccctaaggggaagaggcaatactc	-	-
CY018945	Human H3N2 IAVs	Human	H3N2		2005	Australia	A/South_Australia/42/2005	agtgatgccccattccttgatcggcttcgccgagatcag aggtccctaaggggaagaggcaatactc	-	-
CY015632	Human H3N2 IAVs	Human	H3N2		2005	New_Zealand	A/Waikato/15/2005	agtgatgccccattccttgatcggcttcgccgagatcag aggtccctaaggggaagaggcaatactc	-	-
CY014107	Human H3N2 IAVs	Human	H3N2		2005	New_Zealand	A/Wellington/9/2005	agtgatgccccattccttgatcggcttcgccgagatcag aggtccctaaggggaagaggcaatactc	-	-
CY016000	Human H3N2 IAVs	Human	H3N2		2005	Australia	A/Western_Australia/66/2005	agtgatgccccattccttgatcggcttcgccgagatcag aggtccctaaggggaagaggcaatactc	-	-
CY016008	Human H3N2 IAVs	Human	H3N2		2005	Australia	A/Western_Australia/68/2005	agtgatgccccattccttgatcggcttcgccgagatcag aggtccctaaggggaagaggcaatactc	-	-
CY016024	Human H3N2 IAVs	Human	H3N2		2005	Australia	A/Western_Australia/73/2005	agtgatgccccattccttgatcggcttcgccgagatcag aggtccctaaggggaagaggcaatactc	-	-
CY016032	Human H3N2 IAVs	Human	H3N2		2005	Australia	A/Western_Australia/74/2005	agtgatgccccattccttgatcggcttcgccgagatcag aggtccctaaggggaagaggcaatactc	-	-
CY016048	Human H3N2 IAVs	Human	H3N2		2005	Australia	A/Western_Australia/78/2005	agtgatgccccattccttgatcggcttcgccgagatcag aggtccctaaggggaagaggcaatactc	-	-
CY016663	Human H3N2 IAVs	Human	H3N2		2005	Australia	A/South_Australia/23/2005	agtgatgccccattccttgatcggcttcgccgagatcag aggtccctaaggggaagaggcaatactc	-	-
CY016016	Human H3N2 IAVs	Human	H3N2		2005	Australia	A/Western_Australia/70/2005	agtgatgccccattccttgatcggcttcgccgagatcag aggtccctaaggggaagaggcaatactc	-	-
CY008608	Human H3N2 IAVs	Human	H3N2		2005	New_Zealand	A/Canterbury/253/2005	agtgatgccccattccttgatcggcttcgccgagatcag aggtccctaaggggaagaggcaatactc	-	-
CY008440	Human H3N2 IAVs	Human	H3N2		2005	New_Zealand	A/Canterbury/260/2005	agtgatgccccattccttgatcggcttcgccgagatcag aggtccctaaggggaagaggcaatactc	-	-
CY017825	Human H3N2 IAVs	Human	H3N2		2005	Australia	A/South_Australia/32/2005	agtgatgccccattccttgatcggcttcgccgagatcag aggtccctaaggggaagaggcaatactc	-	-

CY019033	Human H3N2 IAVs	Human	H3N2		2005	Australia	A/Queensland/56/2005	agtgatgccccattccttgatcggcttcgccgagatcag aggtccctaaggggaagaggcaatactc	-	-
CY015608	Human H3N2 IAVs	Human	H3N2		2005	New_Zealand	A/Wellington/4/2005	agtgatgccccattccttgatcggcttcgccgagatcag aggtccctaaggggaagaggcaatactc	-	-
CY092221	Human H3N2 IAVs	Human	H3N2		2005	USA	A/New_Jersey/NHRC0001/2005	agtgatgccccattccttgatcggcttcgccgagatcag aggtccctaaggggaagaggcaatactc	-	-
CY016607	Human H3N2 IAVs	Human	H3N2		2005	Australia	A/South_Australia/20/2005	agtgatgccccattccttgatcggcttcgccgagatcag aggtccctaaggggaagaggcaatactc	-	-
CY014059	Human H3N2 IAVs	Human	H3N2		2005	New_Zealand	A/Southland/5/2005	agtgatgccccattccttgatcggcttcgccgagatcag aggtccctaaggggaagaggcaatactc	-	-
CY016479	Human H3N2 IAVs	Human	H3N2		2005	New_Zealand	A/Wellington/7/2005	agtgatgccccattccttgatcggcttcgccgagatcag aggtccctaaggggaagaggcaatactc	-	-
CY014099	Human H3N2 IAVs	Human	H3N2		2005	New_Zealand	A/Wellington/8/2005	agtgatgccccattccttgatcggcttcgccgagatcag aggtccctaaggggaagaggcaatactc	-	-
CY008400	Human H3N2 IAVs	Human	H3N2		2005	New_Zealand	A/Canterbury/235/2005	agtgatgccccattccttgatcggcttcgccgagatcag aggtccctaaggggaagaggcaatactc	-	-
CY008648	Human H3N2 IAVs	Human	H3N2		2005	New_Zealand	A/Canterbury/266/2005	agtgatgccccattccttgatcggcttcgccgagatcag aggtccctaaggggaagaggcaatactc	-	-
CY008448	Human H3N2 IAVs	Human	H3N2		2005	New_Zealand	A/Canterbury/269/2005	agtgatgccccattccttgatcggcttcgccgagatcag aggtccctaaggggaagaggcaatactc	-	-
CY008352	Human H3N2 IAVs	Human	H3N2		2005	New_Zealand	A/Canterbury/64/2005	agtgatgccccattccttgatcggcttcgccgagatcag aggtccctaaggggaagaggcaatactc	-	-
CY008103	Human H3N2 IAVs	Human	H3N2		2005	New_Zealand	A/Canterbury/212/2005	agtgatgccccattccttgatcggcttcgccgagatcag aggtccctaaggggaagaggcaatactc	-	-
CY008592	Human H3N2 IAVs	Human	H3N2		2005	New_Zealand	A/Canterbury/248/2005	agtgatgccccattccttgatcggcttcgccgagatcag aggtccctaaggggaagaggcaatactc	-	-
CY008616	Human H3N2 IAVs	Human	H3N2		2005	New_Zealand	A/Canterbury/255/2005	agtgatgccccattccttgatcggcttcgccgagatcag aggtccctaaggggaagaggcaatactc	-	-
CY008063	Human H3N2 IAVs	Human	H3N2		2005	New_Zealand	A/Canterbury/67/2005	agtgatgccccattccttgatcggcttcgccgagatcag aggtccctaaggggaagaggcaatactc	-	-
CY010088	Human H3N2 IAVs	Human	H3N2		2005	New_Zealand	A/Canterbury/250/2005	agtgatgccccattccttgatcggcttcgccgagatcag aggtccctaaggggaagaggcaatactc	-	-
CY043772	Human H3N2 IAVs	Human	H3N2		2005	Hong_Kong	A/Hong_Kong/HKU78/2005	agtgatgccccattccttgatcggcttcgccgagatcag aggtccctaaggggaagaggcaatactc	-	-
CY007799	Human H3N2 IAVs	Human	H3N2		2005	New_Zealand	A/Canterbury/01/2005	agtgatgccccattccttgatcggcttcgccgagatcag aggtccctaaggggaagaggcaatactc	-	-
CY007807	Human H3N2 IAVs	Human	H3N2		2005	New_Zealand	A/Canterbury/02/2005	agtgatgccccattccttgatcggcttcgccgagatcag aggtccctaaggggaagaggcaatactc	-	-
CY007815	Human H3N2 IAVs	Human	H3N2		2005	New_Zealand	A/Canterbury/03/2005	agtgatgccccattccttgatcggcttcgccgagatcag aggtccctaaggggaagaggcaatactc	-	-
CY019025	Human H3N2 IAVs	Human	H3N2		2005	Australia	A/Queensland/54/2005	agtgatgccccattccttgatcggcttcgccgagatcag aggtccctaaggggaagaggcaatactc	-	-
CY034120	Human H3N2 IAVs	Human	H3N2		2005	USA	A/Wisconsin/67/2005	agtgatgccccattccttgatcggcttcgccgagatcag aggtccctaaggggaagaggcaatactc	-	-
CY039059	Human H3N2 IAVs	Human	H3N2		2005	Hong_Kong	A/Hong_Kong/HKU45/2005	agtgatgccccattccttgatcggcttcgccgagatcag aggtccctaaggggaagaggcaatactc	-	-
CY009936	Human H3N2 IAVs	Human	H3N2		2005	New_Zealand	A/Canterbury/125/2005	agtgatgccccattccttgatcggcttcgccgagatcag aggtccctaaggggaagaggcaatactc	-	-
CY008079	Human H3N2 IAVs	Human	H3N2		2005	New_Zealand	A/Canterbury/129/2005	agtgatgccccattccttgatcggcttcgccgagatcag aggtccctaaggggaagaggcaatactc	-	-

CY008344	Human H3N2 IAVs	Human	H3N2		2005	New_Zealand	A/Canterbury/20/2005	agtgatgccccattccttgatcggcttcgccgagatcag aggtccctaaggggaagaggcaatactc	-	-
CY009032	Human H3N2 IAVs	Human	H3N2		2005	New_Zealand	A/Canterbury/26/2005	agtgatgccccattccttgatcggcttcgccgagatcag aggtccctaaggggaagaggcaatactc	-	-
CY009040	Human H3N2 IAVs	Human	H3N2		2005	New_Zealand	A/Canterbury/34/2005	agtgatgccccattccttgatcggcttcgccgagatcag aggtccctaaggggaagaggcaatactc	-	-
CY039523	Human H3N2 IAVs	Human	H3N2		2005	Hong_Kong	A/Hong_Kong/HKU74/2005	agtgatgccccattccttgatcggcttcgccgagatcag aggtccctaaggggaagaggcaatactc	-	-
CY105418	Human H3N2 IAVs	Human	H3N2		2005	Viet_Nam	A/TayNguyen/TN380/2005	agtgatgccccattccttgatcggcttcgccgagatcag aggtccctaaggggaagaggcaatactc	-	-
CY105442	Human H3N2 IAVs	Human	H3N2		2005	Viet_Nam	A/TayNguyen/TN403/2005	agtgatgccccattccttgatcggcttcgccgagatcag aggtccctaaggggaagaggcaatactc	-	-
CY104256	Human H3N2 IAVs	Human	H3N2		2005	Viet_Nam	A/TayNguyen/TN381/2005	agtgatgccccattccttgatcggcttcgccgagatcag aggtccctaaggggaagaggcaatactc	-	-
CY038683	Human H3N2 IAVs	Human	H3N2		2005	Hong_Kong	A/Hong_Kong/HKU42/2005	agtgatgccccattccttgatcggcttcgccgagatcag aggtccctaaggggaagaggcaatactc	-	-
CY090993	Human H3N2 IAVs	Human	H3N2		2005	USA	A/South_Carolina/NHRC0001/2005	agtgatgccccattccttgatcggcttcgccgagatcag aggtccctaaggggaagaggcaatactc	-	-
CY006135	Human H3N2 IAVs	Human	H3N2		2005	USA	A/New_York/206/2005	agtgatgccccattccttgatcggcttcgccgagatcag aggtccctaaggggaagaggcaatactc	-	-
CY002740	Human H3N2 IAVs	Human	H3N2		2005	USA	A/New_York/394/2005	agtgatgccccattccttgatcggcttcgccgagatcag aggtccctaaggggaagaggcaatactc	-	-
CY002036	Human H3N2 IAVs	Human	H3N2		2005	USA	A/New_York/382/2005	agtgatgccccattccttgatcggcttcgccgagatcag aggtccctaaggggaagaggcaatactc	-	-
CY038739	Human H3N2 IAVs	Human	H3N2		2005	Hong_Kong	A/Hong_Kong/HKU70/2005	agtgatgccccattccttgatcggcttcgccgagatcag aggtccctaaggggaagaggcaatactc	-	-
CY040350	Human H3N2 IAVs	Human	H3N2		2005	Hong_Kong	A/Hong_Kong/HKU76/2005	agtgatgccccattccttgatcggcttcgccgagatcag aggtccctaaggggaagaggcaatactc	-	-
CY091017	Human H3N2 IAVs	Human	H3N2		2005	USA	A/California/NHRC0001/2005	agtgatgccccattccttgatcggcttcgccgagatcag aggtccctaaggggaagaggcaatactc	-	-
CY039235	Human H3N2 IAVs	Human	H3N2		2005	Hong_Kong	A/Hong_Kong/HKU63/2005	agtgatgccccattccttgatcggcttcgccgagatcag aggtccctaaggggaagaggcaatactc	-	-
CY091537	Human H3N2 IAVs	Human	H3N2		2005	USA	A/California/NHRC0007/2005	agtgatgccccattccttgatcggcttcgccgagatcag aggtccctaaggggaagaggcaatactc	-	-
CY172203	Human H3N2 IAVs	Human	H3N2		2005	USA	A/New_York/1002/2005	agtgatgccccattccttgatcggcttcgccgagatcag aggtccctaaggggaagaggcaatactc	-	-
CY091433	Human H3N2 IAVs	Human	H3N2		2005	USA	A/California/NHRC0002/2005	agtgatgccccattccttgatcggcttcgccgagatcag aggtccctaaggggaagaggcaatactc	-	-
CY105434	Human H3N2 IAVs	Human	H3N2		2005	Viet_Nam	A/TayNguyen/TN393/2005	agtgatgccccattccttgatcggcttcgccgagatcag aggtccctaaggggaagaggcaatactc	-	-
CY091009	Human H3N2 IAVs	Human	H3N2		2005	USA	A/Illinois/NHRC0001/2005	agtgatgccccattccttgatcggcttcgccgagatcag aggtccctaaggggaagaggcaatactc	-	-
CY008071	Human H3N2 IAVs	Human	H3N2		2005	New_Zealand	A/Canterbury/127/2005	agtgatgccccattccttgatcggcttcgccgagatcag aggtccctaaggggaagaggcaatactc	-	-
CY008047	Human H3N2 IAVs	Human	H3N2		2005	New_Zealand	A/Canterbury/24/2005	agtgatgccccattccttgatcggcttcgccgagatcag aggtccctaaggggaagaggcaatactc	-	-
CY091033	Human H3N2 IAVs	Human	H3N2		2005	USA	A/Illinois/NHRC0002/2005	agtgatgccccattccttgatcggcttcgccgagatcag aggtccctaaggggaagaggcaatactc	-	-
CY172187	Human H3N2 IAVs	Human	H3N2		2005	USA	A/New_York/1000/2005	agtgatgccccattccttgatcggcttcgccgagatcag aggtccctaaggggaagaggcaatactc	-	-

CY172195	Human H3N2 IAVs	Human	H3N2		2005	USA	A/New_York/1001/2005	agtgatgccccattccttgatcggcttcgccgagatcag<br>aggtccctaaggggaagaggcaatactc	-	-
CY173603	Human H3N2 IAVs	Human	H3N2		2005	USA	A/New_York/1003/2005	agtgatgccccattccttgatcggcttcgccgagatcag<br>aggtccctaaggggaagaggcaatactc	-	-
EU597804	Human H3N2 IAVs	Human	H3N2		2005	Japan	A/Hiroshima/52/2005	agtgatgccccattccttgatcggcttcgccgagatcag<br>aggtccctaaggggaagaggcaatactc	-	-
CY022577	Human H3N2 IAVs	Human	H3N2		2005	New_Zealand	A/Auckland/616/2005	agtgatgccccattccttgatcggcttcgccgagatcag<br>aggtccctaaggggaagaggcaatactc	-	-
CY023086	Human H3N2 IAVs	Human	H3N2		2005	New_Zealand	A/Auckland/617/2005	agtgatgccccattccttgatcggcttcgccgagatcag<br>aggtccctaaggggaagaggcaatactc	-	-
CY015640	Human H3N2 IAVs	Human	H3N2		2005	New_Zealand	A/Waikato/10/2005	agtgatgccccattccttgatcggcttcgccgagatcag<br>aggtccctaaggggaagaggcaatactc	-	-
CY014139	Human H3N2 IAVs	Human	H3N2		2005	New_Zealand	A/Waikato/12/2005	agtgatgccccattccttgatcggcttcgccgagatcag<br>aggtccctaaggggaagaggcaatactc	-	-
CY009048	Human H3N2 IAVs	Human	H3N2		2005	New_Zealand	A/Canterbury/124/2005	agtgatgccccattccttgatcggcttcgccgagatcag<br>aggtccctaaggggaagaggcaatactc	-	-
CY008368	Human H3N2 IAVs	Human	H3N2		2005	New_Zealand	A/Canterbury/186/2005	agtgatgccccattccttgatcggcttcgccgagatcag<br>aggtccctaaggggaagaggcaatactc	-	-
CY008376	Human H3N2 IAVs	Human	H3N2		2005	New_Zealand	A/Canterbury/204/2005	agtgatgccccattccttgatcggcttcgccgagatcag<br>aggtccctaaggggaagaggcaatactc	-	-
CY008576	Human H3N2 IAVs	Human	H3N2		2005	New_Zealand	A/Canterbury/230/2005	agtgatgccccattccttgatcggcttcgccgagatcag<br>aggtccctaaggggaagaggcaatactc	-	-
CY013244	Human H3N2 IAVs	Human	H3N2		2005	New_Zealand	A/Canterbury/232/2005	agtgatgccccattccttgatcggcttcgccgagatcag<br>aggtccctaaggggaagaggcaatactc	-	-
CY019017	Human H3N2 IAVs	Human	H3N2		2005	Australia	A/Queensland/53/2005	agtgatgccccattccttgatcggcttcgccgagatcag<br>aggtccctaaggggaagaggcaatactc	-	-
CY021969	Human H3N2 IAVs	Human	H3N2		2005	New_Zealand	A/Waikato/6/2005	agtgatgccccattccttgatcggcttcgccgagatcag<br>aggtccctaaggggaagaggcaatactc	-	-
CY014083	Human H3N2 IAVs	Human	H3N2		2005	New_Zealand	A/Waikato/16/2005	agtgatgccccattccttgatcggcttcgccgagatcag<br>aggtccctaaggggaagaggcaatactc	-	-
CY016471	Human H3N2 IAVs	Human	H3N2		2005	New_Zealand	A/Waikato/18/2005	agtgatgccccattccttgatcggcttcgccgagatcag<br>aggtccctaaggggaagaggcaatactc	-	-
CY014115	Human H3N2 IAVs	Human	H3N2		2005	New_Zealand	A/Waikato/9/2005	agtgatgccccattccttgatcggcttcgccgagatcag<br>aggtccctaaggggaagaggcaatactc	-	-
CY014027	Human H3N2 IAVs	Human	H3N2		2005	New_Zealand	A/Wellington/1/2005	agtgatgccccattccttgatcggcttcgccgagatcag<br>aggtccctaaggggaagaggcaatactc	-	-
CY015616	Human H3N2 IAVs	Human	H3N2		2005	New_Zealand	A/Wellington/5/2005	agtgatgccccattccttgatcggcttcgccgagatcag<br>aggtccctaaggggaagaggcaatactc	-	-
CY015624	Human H3N2 IAVs	Human	H3N2		2005	New_Zealand	A/Wellington/6/2005	agtgatgccccattccttgatcggcttcgccgagatcag<br>aggtccctaaggggaagaggcaatactc	-	-
CY014091	Human H3N2 IAVs	Human	H3N2		2005	New_Zealand	A/Otago/2/2005	agtgatgccccattccttgatcggcttcgccgagatcag<br>aggtccctaaggggaagaggcaatactc	-	-
CY008600	Human H3N2 IAVs	Human	H3N2		2005	New_Zealand	A/Canterbury/251/2005	agtgatgccccattccttgatcggcttcgccgagatcag<br>aggtccctaaggggaagaggcaatactc	-	-
CY038755	Human H3N2 IAVs	Human	H3N2		2005	Hong_Kong	A/Hong_Kong/HKU79/2005	agtgatgccccattccttgatcggcttcgccgagatcag<br>aggtccctaaggggaagaggcaatactc	-	-
CY008640	Human H3N2 IAVs	Human	H3N2		2005	New_Zealand	A/Canterbury/258/2005	agtgatgccccattccttgatcggcttcgccgagatcag<br>aggtccctaaggggaagaggcaatactc	-	-
CY016983	Human H3N2 IAVs	Human	H3N2		2005	Australia	A/Western_Australia/69/2005	agtgatgccccattccttgatcggcttcgccgagatcag<br>aggtccctaaggggaagaggcaatactc	-	-

CY016991	Human H3N2 IAVs	Human	H3N2		2005	Australia	A/Western_Australia/72/2005	agtgatgccccattccttgatcggcttcgccgagatcag<br>aggtccctaaggggaagaggcaatactc	-	-
CY091545	Human H3N2 IAVs	Human	H3N2		2005	USA	A/California/NHRC0008/2005	agtgatgccccattccttgatcggcttcgccgagatcag<br>aggtccctaaggggaagaggcaatactc	-	-
KJ855415	Human H3N2 IAVs	Human	H3N2		2005	Mexico	A/Mexico/DIF2160/2005	agtgatgccccattccttgatcggcttcgccgagatcag<br>aggtccctaaggggaagaggcaatactc	-	-
CY100566	Human H3N2 IAVs	Human	H3N2		2005	Mexico	A/Mexico/InDRE2160/2005	agtgatgccccattccttgatcggcttcgccgagatcag<br>aggtccctaaggggaagaggcaatactc	-	-
CY040342	Human H3N2 IAVs	Human	H3N2		2005	Hong_Kong	A/Hong_Kong/HKU75/2005	agtgatgccccattccttgatcggcttcgccgagatcag<br>aggtccctaaggggaagaggcaatactc	-	-
CY091385	Human H3N2 IAVs	Human	H3N2		2005	Australia	A/Australia/NHRC0006/2005	agtgatgccccattccttgatcggcttcgccgagatcag<br>aggtccctaaggggaagaggcaatactc	-	-
CY091377	Human H3N2 IAVs	Human	H3N2		2005	Australia	A/Australia/NHRC0007/2005	agtgatgccccattccttgatcggcttcgccgagatcag<br>aggtccctaaggggaagaggcaatactc	-	-
CY091369	Human H3N2 IAVs	Human	H3N2		2005	Australia	A/Australia/NHRC0008/2005	agtgatgccccattccttgatcggcttcgccgagatcag<br>aggtccctaaggggaagaggcaatactc	-	-
CY091361	Human H3N2 IAVs	Human	H3N2		2005	Australia	A/Australia/NHRC0009/2005	agtgatgccccattccttgatcggcttcgccgagatcag<br>aggtccctaaggggaagaggcaatactc	-	-
CY092237	Human H3N2 IAVs	Human	H3N2		2005	Australia	A/Australia/NHRC0010/2005	agtgatgccccattccttgatcggcttcgccgagatcag<br>aggtccctaaggggaagaggcaatactc	-	-
CY091345	Human H3N2 IAVs	Human	H3N2		2005	Australia	A/Australia/NHRC0012/2005	agtgatgccccattccttgatcggcttcgccgagatcag<br>aggtccctaaggggaagaggcaatactc	-	-
CY091337	Human H3N2 IAVs	Human	H3N2		2005	Australia	A/Australia/NHRC0013/2005	agtgatgccccattccttgatcggcttcgccgagatcag<br>aggtccctaaggggaagaggcaatactc	-	-
CY091321	Human H3N2 IAVs	Human	H3N2		2005	Australia	A/Australia/NHRC0015/2005	agtgatgccccattccttgatcggcttcgccgagatcag<br>aggtccctaaggggaagaggcaatactc	-	-
CY008624	Human H3N2 IAVs	Human	H3N2		2005	New_Zealand	A/Canterbury/256/2005	agtgatgccccattccttgatcggcttcgccgagatcag<br>aggtccctaaggggaagaggcaatactc	-	-
CY016040	Human H3N2 IAVs	Human	H3N2		2005	Australia	A/Western_Australia/75/2005	agtgatgccccattccttgatcggcttcgccgagatcag<br>aggtccctaaggggaagaggcaatactc	-	-
CY017615	Human H3N2 IAVs	Human	H3N2		2005	Australia	A/Queensland/55/2005	agtgatgccccattccttgatcggcttcgccgagatcag<br>aggtccctaaggggaagaggcaatactc	-	-
CY091353	Human H3N2 IAVs	Human	H3N2		2005	Australia	A/Australia/NHRC0011/2005	agtgatgccccattccttgatcggcttcgccgagatcag<br>aggtccctaaggggaagaggcaatactc	-	-
CY019751	Human H3N2 IAVs	Human	H3N2		2005	Australia	A/Queensland/60/2005	agtgatgccccattccttgatcggcttcgccgagatcag<br>aggtccctaaggggaagaggcaatactc	-	-
CY091329	Human H3N2 IAVs	Human	H3N2		2005	Australia	A/Australia/NHRC0014/2005	agtgatgccccattccttgatcggcttcgccgagatcag<br>aggtccctaaggggaagaggcaatactc	-	-
CY021769	Human H3N2 IAVs	Human	H3N2		2005	Australia	A/South_Australia/53/2005	agtgatgccccattccttgatcggcttcgccgagatcag<br>aggtccctaaggggaagaggcaatactc	-	-
CY105378	Human H3N2 IAVs	Human	H3N2		2005	Viet_Nam	A/TayNguyen/TN348/2005	agtgatgccccattccttgatcggcttcgccgagatcag<br>aggtccctaaggggaagaggcaatactc	-	-
CY104224	Human H3N2 IAVs	Human	H3N2		2005	Viet_Nam	A/TayNguyen/TN340/2005	agtgatgccccattccttgatcggcttcgccgagatcag<br>aggtccctaaggggaagaggcaatactc	-	-
CY100558	Human H3N2 IAVs	Human	H3N2		2005	Mexico	A/Mexico/InDRE2118/2005	agtgatgccccattccttgatcggcttcgccgagatcag<br>aggtccctaaggggaagaggcaatactc	-	-
CY039203	Human H3N2 IAVs	Human	H3N2		2005	Hong_Kong	A/Hong_Kong/HKU57/2005	agtgatgccccattccttgatcggcttcgccgagatcag<br>aggtccctaaggggaagatgcaatactc	-	-
CY039179	Human H3N2 IAVs	Human	H3N2		2005	Hong_Kong	A/Hong_Kong/HKU44/2005	agtgatgccccattccttgatcggcttcgccgagatcag<br>aggtccttaaggggaagaggcaatactc	+	+

EU247845	Human H3N2 IAVs	Human	H3N2		2005	France	A/Poitiers/484/2005	agtgatgccccattccttgatcggcttcgccgggatcag aggtccctaagaggaagaggcaatactc	-	*
CY091529	Human H3N2 IAVs	Human	H3N2		2005	USA	A/California/NHRC0006/2005	agtgatgccccatttcttgatcggcttcgccgagatcag aggtccctaaggggaagaggcaatactc	-	-
EU273790	Human H3N2 IAVs	Swine	H3N2		2005	China	A/Swine/Heilongjiang/1/05	ggtgatgccccattccttgatcggcttcgccgagatcag aagtccctaaggggaagaggcagcactc	+	+
CY118894	Human H3N2 IAVs	Human	H3N2		2006	Malaysia	A/Malaysia/33561/2006	agtgatgccccattccttgatcggcttcgccgagatcag aggtccctaaggagaagaggcaatactc	-	*
EU273791	Human H3N2 IAVs	Swine	H3N2		2006	China	A/Swine/Guangdong/164/06	agtgatgccccattccttgatcggcttcgccgagatcag aggtccctaaggggaagaggcaacactc	-	-
GU086093	Human H3N2 IAVs	Swine	H3N2		2006	China	A/swine/Guangdong/7/2006	agtgatgccccattccttgatcggcttcgccgagatcag aggtccctaaggggaagaggcaacactc	-	-
EU273792	Human H3N2 IAVs	Swine	H3N2		2006	China	A/Swine/Guangdong/165/06	agtgatgccccattccttgatcggcttcgccgagatcag aggtccctaaggggaagaggcaacactc	-	-
EU273793	Human H3N2 IAVs	Swine	H3N2		2006	China	A/Swine/Guangdong/166/06	agtgatgccccattccttgatcggcttcgccgagatcag aggtccctaaggggaagaggcaacactc	-	-
EU655689	Human H3N2 IAVs	Swine	H3N2		2006	China	A/swine/Sichuan/01/2006	agtgatgccccattccttgatcggcttcgccgagatcag aggtccctaaggggaagaggcaacactc	-	-
CY172339	Human H3N2 IAVs	Human	H3N2		2006	USA	A/New_York/1022/2006	agtgatgccccattccttgatcggcttcgccgagatcag aggtccctaaggggaagaggcaacactc	-	-
CY172355	Human H3N2 IAVs	Human	H3N2		2006	USA	A/New_York/1024/2006	agtgatgccccattccttgatcggcttcgccgagatcag aggtccctaaggggaagaggcaacactc	-	-
CY172363	Human H3N2 IAVs	Human	H3N2		2006	USA	A/New_York/1025/2006	agtgatgccccattccttgatcggcttcgccgagatcag aggtccctaaggggaagaggcaacactc	-	-
CY172371	Human H3N2 IAVs	Human	H3N2		2006	USA	A/New_York/1026/2006	agtgatgccccattccttgatcggcttcgccgagatcag aggtccctaaggggaagaggcaacactc	-	-
CY172379	Human H3N2 IAVs	Human	H3N2		2006	USA	A/New_York/1027/2006	agtgatgccccattccttgatcggcttcgccgagatcag aggtccctaaggggaagaggcaacactc	-	-
CY172395	Human H3N2 IAVs	Human	H3N2		2006	USA	A/New_York/1029/2006	agtgatgccccattccttgatcggcttcgccgagatcag aggtccctaaggggaagaggcaacactc	-	-
CY172427	Human H3N2 IAVs	Human	H3N2		2006	USA	A/New_York/1033/2006	agtgatgccccattccttgatcggcttcgccgagatcag aggtccctaaggggaagaggcaacactc	-	-
CY172443	Human H3N2 IAVs	Human	H3N2		2006	USA	A/New_York/1035/2006	agtgatgccccattccttgatcggcttcgccgagatcag aggtccctaaggggaagaggcaacactc	-	-
CY172483	Human H3N2 IAVs	Human	H3N2		2006	USA	A/New_York/1041/2006	agtgatgccccattccttgatcggcttcgccgagatcag aggtccctaaggggaagaggcaacactc	-	-
CY172491	Human H3N2 IAVs	Human	H3N2		2006	USA	A/New_York/1042/2006	agtgatgccccattccttgatcggcttcgccgagatcag aggtccctaaggggaagaggcaacactc	-	-
CY013236	Human H3N2 IAVs	Human	H3N2		2006	USA	A/New_York/5/2006	agtgatgccccattccttgatcggcttcgccgagatcag aggtccctaaggggaagaggcaacactc	-	-
CY172267	Human H3N2 IAVs	Human	H3N2		2006	USA	A/New_York/1013/2006	agtgatgccccattccttgatcggcttcgccgagatcag aggtccctaaggggaagaggcaacactc	-	-
CY091073	Human H3N2 IAVs	Human	H3N2		2006	USA	A/Texas/NHRC0002/2006	agtgatgccccattccttgatcggcttcgccgagatcag aggtccctaaggggaagaggcaacactc	-	-
CY172323	Human H3N2 IAVs	Human	H3N2		2006	USA	A/New_York/1020/2006	agtgatgccccattccttgatcggcttcgccgagatcag aggtccctaaggggaagaggcaacactc	-	-
FJ769887	Human H3N2 IAVs	Human	H3N2		2006	Iran	A/Esfahan/413/2006	agtgatgccccattccttgatcggcttcgccgagatcag aggtccctaaggggaagaggcaatactc	-	-
AB442000	Human H3N2 IAVs	Human	H3N2		2006	Japan	A/Sendai_H/041/2006	agtgatgccccattccttgatcggcttcgccgagatcag aggtccctaaggggaagaggcaatactc	-	-

AB442001	Human H3N2 IAVs	Human	H3N2		2006	Japan	A/Sendai_H/F005/2006	agtgatgccccattccttgatcggcttcgccgagatcag<br>aggtccctaaggggaagaggcaatactc	-	-
AB442003	Human H3N2 IAVs	Human	H3N2		2006	LAB	A/Sendai_H/401/2006	agtgatgccccattccttgatcggcttcgccgagatcag<br>aggtccctaaggggaagaggcaatactc	-	-
CY114393	Human H3N2 IAVs	Human	H3N2		2006	Netherlands	A/Netherlands/42/2006	agtgatgccccattccttgatcggcttcgccgagatcag<br>aggtccctaaggggaagaggcaatactc	-	-
AB442002	Human H3N2 IAVs	Human	H3N2		2006	Japan	A/Sendai_H/132/2006	agtgatgccccattccttgatcggcttcgccgagatcag<br>aggtccctaaggggaagaggcaatactc	-	-
CY118878	Human H3N2 IAVs	Human	H3N2		2006	Malaysia	A/Malaysia/33464/2006	agtgatgccccattccttgatcggcttcgccgagatcag<br>aggtccctaaggggaagaggcaatactc	-	-
CY118886	Human H3N2 IAVs	Human	H3N2		2006	Malaysia	A/Malaysia/33827/2006	agtgatgccccattccttgatcggcttcgccgagatcag<br>aggtccctaaggggaagaggcaatactc	-	-
CY172211	Human H3N2 IAVs	Human	H3N2		2006	USA	A/New_York/1005/2006	agtgatgccccattccttgatcggcttcgccgagatcag<br>aggtccctaaggggaagaggcaatactc	-	-
CY172283	Human H3N2 IAVs	Human	H3N2		2006	USA	A/New_York/1015/2006	agtgatgccccattccttgatcggcttcgccgagatcag<br>aggtccctaaggggaagaggcaatactc	-	-
CY012796	Human H3N2 IAVs	Human	H3N2		2006	USA	A/New_York/4/2006	agtgatgccccattccttgatcggcttcgccgagatcag<br>aggtccctaaggggaagaggcaatactc	-	-
CY091049	Human H3N2 IAVs	Human	H3N2		2006	USA	A/Illinois/NHRC0001/2006	agtgatgccccattccttgatcggcttcgccgagatcag<br>aggtccctaaggggaagaggcaatactc	-	-
CY172411	Human H3N2 IAVs	Human	H3N2		2006	USA	A/New_York/1031/2006	agtgatgccccattccttgatcggcttcgccgagatcag<br>aggtccctaaggggaagaggcaatactc	-	-
CY019329	Human H3N2 IAVs	Human	H3N2		2006	USA	A/New_York/928/2006	agtgatgccccattccttgatcggcttcgccgagatcag<br>aggtccctaaggggaagaggcaatactc	-	-
CY020097	Human H3N2 IAVs	Human	H3N2		2006	USA	A/New_York/928/2006	agtgatgccccattccttgatcggcttcgccgagatcag<br>aggtccctaaggggaagaggcaatactc	-	-
CY020089	Human H3N2 IAVs	Human	H3N2		2006	USA	A/New_York/928/2006	agtgatgccccattccttgatcggcttcgccgagatcag<br>aggtccctaaggggaagaggcaatactc	-	-
CY019943	Human H3N2 IAVs	Human	H3N2		2006	USA	A/New_York/928/2006	agtgatgccccattccttgatcggcttcgccgagatcag<br>aggtccctaaggggaagaggcaatactc	-	-
CY019839	Human H3N2 IAVs	Human	H3N2		2006	USA	A/New_York/928/2006	agtgatgccccattccttgatcggcttcgccgagatcag<br>aggtccctaaggggaagaggcaatactc	-	-
CY172435	Human H3N2 IAVs	Human	H3N2		2006	USA	A/New_York/1034/2006	agtgatgccccattccttgatcggcttcgccgagatcag<br>aggtccctaaggggaagaggcaatactc	-	-
CY172499	Human H3N2 IAVs	Human	H3N2		2006	USA	A/New_York/1043/2006	agtgatgccccattccttgatcggcttcgccgagatcag<br>aggtccctaaggggaagaggcaatactc	-	-
CY172227	Human H3N2 IAVs	Human	H3N2		2006	USA	A/New_York/1008/2006	agtgatgccccattccttgatcggcttcgccgagatcag<br>aggtccctaaggggaagaggcaatactc	-	-
CY121572	Human H3N2 IAVs	Human	H3N2		2006	Australia	A/Brisbane/09/2006	agtgatgccccattccttgatcggcttcgccgagatcag<br>aggtccctaaggggaagaggcaatactc	-	-
CY118918	Human H3N2 IAVs	Human	H3N2		2006	Malaysia	A/Malaysia/1665090/2006	agtgatgccccattccttgatcggcttcgccgagatcag<br>aggtccctaaggggaagaggcaatactc	-	-
CY118032	Human H3N2 IAVs	Human	H3N2		2006	Malaysia	A/Malaysia/1666665/2006	agtgatgccccattccttgatcggcttcgccgagatcag<br>aggtccctaaggggaagaggcaatactc	-	-
CY118040	Human H3N2 IAVs	Human	H3N2		2006	Malaysia	A/Malaysia/1666666/2006	agtgatgccccattccttgatcggcttcgccgagatcag<br>aggtccctaaggggaagaggcaatactc	-	-
FJ912954	Human H3N2 IAVs	Human	H3N2		2006	Thailand	A/Thailand/CU231/2006	agtgatgccccattccttgatcggcttcgccgagatcag<br>aggtccctaaggggaagaggcaatactc	-	-
CY118926	Human H3N2 IAVs	Human	H3N2		2006	Malaysia	A/Malaysia/1674395/2006	agtgatgccccattccttgatcggcttcgccgagatcag<br>aggtccctaaggggaagaggcaatactc	-	-

CY118934	Human H3N2 IAVs	Human	H3N2		2006	Malaysia	A/Malaysia/1676558/2006	agtgatgccccattccttgatcggcttcgccgagatcag aggtccctaaggggaagaggcaatactc	-	-
CY118942	Human H3N2 IAVs	Human	H3N2		2006	Malaysia	A/Malaysia/1681079/2006	agtgatgccccattccttgatcggcttcgccgagatcag aggtccctaaggggaagaggcaatactc	-	-
CY091089	Human H3N2 IAVs	Human	H3N2		2006	USA	A/Illinois/NHRC0002/2006	agtgatgccccattccttgatcggcttcgccgagatcag aggtccctaaggggaagaggcaatactc	-	-
CY091081	Human H3N2 IAVs	Human	H3N2		2006	USA	A/Missouri/NHRC0001/2006	agtgatgccccattccttgatcggcttcgccgagatcag aggtccctaaggggaagaggcaatactc	-	-
CY019337	Human H3N2 IAVs	Human	H3N2		2006	USA	A/New_York/933/2006	agtgatgccccattccttgatcggcttcgccgagatcag aggtccctaaggggaagaggcaatactc	-	-
CY019855	Human H3N2 IAVs	Human	H3N2		2006	USA	A/New_York/933/2006	agtgatgccccattccttgatcggcttcgccgagatcag aggtccctaaggggaagaggcaatactc	-	-
CY019847	Human H3N2 IAVs	Human	H3N2		2006	USA	A/New_York/933/2006	agtgatgccccattccttgatcggcttcgccgagatcag aggtccctaaggggaagaggcaatactc	-	-
CY172219	Human H3N2 IAVs	Human	H3N2		2006	USA	A/New_York/1006/2006	agtgatgccccattccttgatcggcttcgccgagatcag aggtccctaaggggaagaggcaatactc	-	-
CY172307	Human H3N2 IAVs	Human	H3N2		2006	USA	A/New_York/1018/2006	agtgatgccccattccttgatcggcttcgccgagatcag aggtccctaaggggaagaggcaatactc	-	-
CY172347	Human H3N2 IAVs	Human	H3N2		2006	USA	A/New_York/1023/2006	agtgatgccccattccttgatcggcttcgccgagatcag aggtccctaaggggaagaggcaatactc	-	-
CY172387	Human H3N2 IAVs	Human	H3N2		2006	USA	A/New_York/1028/2006	agtgatgccccattccttgatcggcttcgccgagatcag aggtccctaaggggaagaggcaatactc	-	-
CY172419	Human H3N2 IAVs	Human	H3N2		2006	USA	A/New_York/1032/2006	agtgatgccccattccttgatcggcttcgccgagatcag aggtccctaaggggaagaggcaatactc	-	-
CY172459	Human H3N2 IAVs	Human	H3N2		2006	USA	A/New_York/1037/2006	agtgatgccccattccttgatcggcttcgccgagatcag aggtccctaaggggaagaggcaatactc	-	-
CY172507	Human H3N2 IAVs	Human	H3N2		2006	USA	A/New_York/1044/2006	agtgatgccccattccttgatcggcttcgccgagatcag aggtccctaaggggaagaggcaatactc	-	-
CY172515	Human H3N2 IAVs	Human	H3N2		2006	USA	A/New_York/1045/2006	agtgatgccccattccttgatcggcttcgccgagatcag aggtccctaaggggaagaggcaatactc	-	-
CY172523	Human H3N2 IAVs	Human	H3N2		2006	USA	A/New_York/1046/2006	agtgatgccccattccttgatcggcttcgccgagatcag aggtccctaaggggaagaggcaatactc	-	-
CY172531	Human H3N2 IAVs	Human	H3N2		2006	USA	A/New_York/1048/2006	agtgatgccccattccttgatcggcttcgccgagatcag aggtccctaaggggaagaggcaatactc	-	-
CY020113	Human H3N2 IAVs	Human	H3N2		2006	USA	A/New_York/933/2006	agtgatgccccattccttgatcggcttcgccgagatcag aggtccctaaggggaagaggcaatactc	-	-
CY020105	Human H3N2 IAVs	Human	H3N2		2006	USA	A/New_York/933/2006	agtgatgccccattccttgatcggcttcgccgagatcag aggtccctaaggggaagaggcaatactc	-	-
CY172451	Human H3N2 IAVs	Human	H3N2		2006	USA	A/New_York/1036/2006	agtgatgccccattccttgatcggcttcgccgagatcag aggtccctaaggggaagaggcaatactc	-	-
CY091065	Human H3N2 IAVs	Human	H3N2		2006	USA	A/South_Carolina/NHRC0001/2006	agtgatgccccattccttgatcggcttcgccgagatcag aggtccctaaggggaagaggcaatactc	-	-
FJ912924	Human H3N2 IAVs	Human	H3N2		2006	Thailand	A/Thailand/CU46/2006	agtgatgccccattccttgatcggcttcgccgagatcag aggtccctaaggggaagaggcaatactc	-	-
AB434128	Human H3N2 IAVs	Human	H3N2		2006	Japan	A/Morioka/18/2006	agtgatgccccattccttgatcggcttcgccgagatcag aggtccctaaggggaagaggcaatactc	-	-
CY091041	Human H3N2 IAVs	Human	H3N2		2006	USA	A/Texas/NHRC0001/2006	agtgatgccccattccttgatcggcttcgccgagatcag aggtccctaaggggaagaggcaatactc	-	-
CY091057	Human H3N2 IAVs	Human	H3N2		2006	USA	A/California/NHRC0001/2006	agtgatgccccattccttgatcggcttcgccgagatcag aggtccctaaggggaagaggcaatactc	-	-

CY020137	Human H3N2 IAVs	Human	H3N2		2006	USA	A/New_York/938/2006	agtgatgccccattccttgatcggcttcgccgagatcagaggtccctaaggggaagaggcaatactc	-	-
CY020129	Human H3N2 IAVs	Human	H3N2		2006	USA	A/New_York/938/2006	agtgatgccccattccttgatcggcttcgccgagatcagaggtccctaaggggaagaggcaatactc	-	-
CY020121	Human H3N2 IAVs	Human	H3N2		2006	USA	A/New_York/938/2006	agtgatgccccattccttgatcggcttcgccgagatcagaggtccctaaggggaagaggcaatactc	-	-
CY020369	Human H3N2 IAVs	Human	H3N2		2006	USA	A/New_York/938/2006	agtgatgccccattccttgatcggcttcgccgagatcagaggtccctaaggggaagaggcaatactc	-	-
CY019863	Human H3N2 IAVs	Human	H3N2		2006	USA	A/New_York/938/2006	agtgatgccccattccttgatcggcttcgccgagatcagaggtccctaaggggaagaggcaatactc	-	-
CY172299	Human H3N2 IAVs	Human	H3N2		2006	USA	A/New_York/1017/2006	agtgatgccccattccttgatcggcttcgccgagatcagaggtccctaaggggaagaggcaatactc	-	-
CY172331	Human H3N2 IAVs	Human	H3N2		2006	USA	A/New_York/1021/2006	agtgatgccccattccttgatcggcttcgccgagatcagaggtccctaaggggaagaggcaatactc	-	-
CY016999	Human H3N2 IAVs	Human	H3N2		2006	USA	A/New_York/6/2006	agtgatgccccattccttgatcggcttcgccgagatcagaggtccctaaggggaagaggcaatactc	-	-
CY014163	Human H3N2 IAVs	Human	H3N2		2006	USA	A/New_York/7/2006	agtgatgccccattccttgatcggcttcgccgagatcagaggtccctaaggggaagaggcaatactc	-	-
AB434127	Human H3N2 IAVs	Human	H3N2		2006	Japan	A/Morioka/17/2006	agtgatgccccattccttgatcggcttcgccgagatcagaggtccctaaggggaagaggcaatactc	-	-
CY091145	Human H3N2 IAVs	Human	H3N2		2006	USA	A/Georgia/NHRC0001/2006	agtgatgccccattccttgatcggcttcgccgagatcagaggtccctaaggggaagaggcaatactc	-	-
CY172235	Human H3N2 IAVs	Human	H3N2		2006	USA	A/New_York/1009/2006	agtgatgccccattccttgatcggcttcgccgagatcagaggtccctaaggggaagaggcaatactc	-	-
CY172259	Human H3N2 IAVs	Human	H3N2		2006	USA	A/New_York/1012/2006	agtgatgccccattccttgatcggcttcgccgagatcagaggtccctaaggggaagaggcaatactc	-	-
CY172243	Human H3N2 IAVs	Human	H3N2		2006	USA	A/New_York/1010/2006	agtgatgccccattccttgatcggcttcgccgagatcagaggtccctaaggggaagaggcaatactc	-	-
CY172275	Human H3N2 IAVs	Human	H3N2		2006	USA	A/New_York/1014/2006	agtgatgccccattccttgatcggcttcgccgagatcagaggtccctaaggggaagaggcaatactc	-	-
CY172251	Human H3N2 IAVs	Human	H3N2		2006	USA	A/New_York/1011/2006	agtgatgccccattccttgatcggcttcgccgagatcagaggtccctaaggggaagaggcaatactc	-	-
CY172403	Human H3N2 IAVs	Human	H3N2		2006	USA	A/New_York/1030/2006	agtgatgccccattccttgatcggcttcgccgagatcagaggtccctaaggggaagaggcaatactc	-	-
CY020073	Human H3N2 IAVs	Human	H3N2		2006	USA	A/New_York/923/2006	agtgatgccccattccttgatcggcttcgccgagatcagaggtccctaaggggaagaggcaatactc	-	-
CY019831	Human H3N2 IAVs	Human	H3N2		2006	USA	A/New_York/923/2006	agtgatgccccattccttgatcggcttcgccgagatcagaggtccctaaggggaagaggcaatactc	-	-
CY020081	Human H3N2 IAVs	Human	H3N2		2006	USA	A/New_York/923/2006	agtgatgccccattccttgatcggcttcgccgagatcagaggtccctaaggggaagaggcaatactc	-	-
CY020065	Human H3N2 IAVs	Human	H3N2		2006	USA	A/New_York/923/2006	agtgatgccccattccttgatcggcttcgccgagatcagaggtccctaaggggaagaggcaatactc	-	-
CY020361	Human H3N2 IAVs	Human	H3N2		2006	USA	A/New_York/923/2006	agtgatgccccattccttgatcggcttcgccgagatcagaggtccctaaggggaagaggcaatactc	-	-
FJ912906	Human H3N2 IAVs	Human	H3N2		2006	Thailand	A/Thailand/CU23/2006	agtgatgccccattccttgatcggcttcgccgagatcagaggtccctaaggggaagaggcaatactc	-	-
CY118008	Human H3N2 IAVs	Human	H3N2		2006	Malaysia	A/Malaysia/33817/2006	agtgatgccccattccttgatcggcttcgccgagatcagaggtccctaaggggaagaggcaatactc	-	-
CY172315	Human H3N2 IAVs	Human	H3N2		2006	USA	A/New_York/1019/2006	agtgatgccccattccttgatcggcttcgccgagatcagaggtccctaaggggaagaggcaatactc	-	-

KJ855367	Human H3N2 IAVs	Human	H3N2		2006	Mexico	A/Mexico/DIF29/2006	agtgatgccccattccttgatcggcttcgccgagatcag<br>aggtccctaaggggaagaggcaatactc	-	-
CY100606	Human H3N2 IAVs	Human	H3N2		2006	Mexico	A/Mexico/InDRE29/2006	agtgatgccccattccttgatcggcttcgccgagatcag<br>aggtccctaaggggaagaggcaatactc	-	-
CY118902	Human H3N2 IAVs	Human	H3N2		2006	Malaysia	A/Malaysia/34015/2006	agtgatgccccattccttgatcggcttcgccgagatcag<br>aggtccctaaggggaagaggcaatactc	-	-
CY105882	Human H3N2 IAVs	Human	H3N2		2006	Viet_Nam	A/TayNguyen/HCM52/2006	agtgatgccccattccttgatcggcttcgccgagatcag<br>aggtccctaaggggaagaggcaatactc	-	-
AB434129	Human H3N2 IAVs	Human	H3N2		2006	Japan	A/Morioka/22/2006	agtgatgccccattccttgatcggcttcgccgagatcag<br>aggtccctaaggggaagaggcagtactc	-	-
AB434130	Human H3N2 IAVs	Human	H3N2		2006	Japan	A/Morioka/29/2006	agtgatgccccattccttgatcggcttcgccgagatcag<br>aggtccctaaggggaagaggcagtactc	-	-
CY118910	Human H3N2 IAVs	Human	H3N2		2006	Malaysia	A/Malaysia/1657167/2006	agtgatgccccattccttgatcggcttcgccgagatcag<br>aggtccctaaggggtagaggcaatactc	-	-
CY172291	Human H3N2 IAVs	Human	H3N2		2006	USA	A/New_York/1016/2006	agtgatgccccattccttgatcggcttcgccgggatcag<br>aggtccctaaggggaagaggcaatactc	-	-
CY114401	Human H3N2 IAVs	Human	H3N2		2006	Netherlands	A/Netherlands/363/2006	agtgatgccccattccttgatcgggttcgccgagatca<br>gaggtccctaaggggaagaggcaacactc	-	-
FJ912960	Human H3N2 IAVs	Human	H3N2		2006	Thailand	A/Thailand/CU260/2006	agtgatgccccattccttgatcgggttcgccgagatca<br>gaggtccctaaggggaagaggcaacactc	-	-
FJ912948	Human H3N2 IAVs	Human	H3N2		2006	Thailand	A/Thailand/CU228/2006	agtgatgccccattccttgatcgggttcgccgagatca<br>gaggtccctaaggggaagaggcaacactc	-	-
CY121516	Human H3N2 IAVs	Human	H3N2		2006	Nepal	A/Nepal/921/2006	agtgatgccccattccttgatcgggttcgccgagatca<br>gaggtccctaaggggaagaggcaacactc	-	-
CY172467	Human H3N2 IAVs	Human	H3N2		2006	USA	A/New_York/1039/2006	agtgatgccccccttccttgatcggcttcgccgagatcag<br>aggtccctaaggggaagaggcaatactc	-	-
CY172475	Human H3N2 IAVs	Human	H3N2		2006	USA	A/New_York/1040/2006	agtgatgccccccttccttgatcggcttcgccgagatcag<br>aggtccctaaggggaagaggcaatactc	-	-
FJ769886	Human H3N2 IAVs	Human	H3N2		2006	Iran	A/Boshehr/388/2006	ggtgatgccccattccttgatcggcttcgccgagacca<br>gaggtccctaaggggaagaggcagcactc	-	-
GU086125	Human H3N2 IAVs	Swine	H3N2		2006	China	A/swine/Guangdong/211/2006	ggtgatgccccattccttgatcggcttcgccgagatcag<br>aagtccctaaggggaagaggcagcactc	+	+
GU086133	Human H3N2 IAVs	Swine	H3N2		2006	China	A/swine/Guangdong/223/2006	ggtgatgccccattccttgatcggcttcgccgagatcag<br>aagtccctaaggggaagaggcagcactc	+	+
GU086149	Human H3N2 IAVs	Swine	H3N2		2006	China	A/swine/Guangdong/811/2006	ggtgatgccccattccttgatcggcttcgccgagatcag<br>aagtccctaaggggaagaggcagcactc	+	+
GU086141	Human H3N2 IAVs	Swine	H3N2		2006	China	A/swine/Guangdong/423/2006	ggtgatgccccattccttgatcggcttcgccgagatcag<br>aagtccctaaggggaagaggcagcactc	+	+
GU086157	Human H3N2 IAVs	Swine	H3N2		2006	China	A/swine/Guangdong/968/2006	ggtgatgccccattccttgatcggcttcgccgagatcag<br>aagtccctaaggggaagaggcagcactc	+	+
CY025847	Human H3N2 IAVs	Human	H3N2		2007	USA	A/New_York/UR06_0510/2007	agtgatgccccattcctcgatcggcttcgccgagatca<br>gaggtccctaaggggaagaggcaatactc	-	-
CY026671	Human H3N2 IAVs	Human	H3N2		2007	USA	A/Colorado/UR06_0279/2007	agtgatgccccattccttgatcggcttcgccgagacca<br>gaggtccctaaggggaagaggcaatactc	-	-
CY104480	Human H3N2 IAVs	Human	H3N2		2007	Viet_Nam	A/HaNoi/Q582/2007	agtgatgccccattccttgatcggcttcgccgagacca<br>gaggtccctaaggggaagaggcaatactc	-	-
CY172875	Human H3N2 IAVs	Human	H3N2		2007	USA	A/New_York/1098/2007	agtgatgccccattccttgatcggcttcgccgagatcag<br>aggtccctaaagggaagaggcaatactc	+	+
CY025735	Human H3N2 IAVs	Human	H3N2		2007	USA	A/New_York/UR06_0529/2007	agtgatgccccattccttgatcggcttcgccgagatcag<br>aggtccctaaagggaagaggcaatactc	+	+

GU215030	Human H3N2 IAVs	Swine	H3N2		2007	China	A/swine/Jilin/19/2007	agtgatgccccattccttgatcggcttcgccgagatcag aggtccctaaggggaagaggcaacactc	-	-
GU215022	Human H3N2 IAVs	Swine	H3N2		2007	China	A/swine/Jilin/5/2007	agtgatgccccattccttgatcggcttcgccgagatcag aggtccctaaggggaagaggcaacactc	-	-
CY025489	Human H3N2 IAVs	Human	H3N2		2007	USA	A/New_York/UR06_0515/2007	agtgatgccccattccttgatcggcttcgccgagatcag aggtccctaaggggaagaggcaacactc	-	-
CY027503	Human H3N2 IAVs	Human	H3N2		2007	USA	A/Ohio/UR06_0410/2007	agtgatgccccattccttgatcggcttcgccgagatcag aggtccctaaggggaagaggcaataatc	+	+
CY026927	Human H3N2 IAVs	Human	H3N2		2007	USA	A/Ohio/UR06_0494/2007	agtgatgccccattccttgatcggcttcgccgagatcag aggtccctaaggggaagaggcaataatc	+	+
CY026279	Human H3N2 IAVs	Human	H3N2		2007	USA	A/Texas/UR06_0566/2007	agtgatgccccattccttgatcggcttcgccgagatcag aggtccctaaggggaagaggcaataccc	-	-
CY026039	Human H3N2 IAVs	Human	H3N2		2007	USA	A/Alabama/UR06_0482/2007	agtgatgccccattccttgatcggcttcgccgagatcag aggtccctaaggggaagaggcaatactc	-	-
KF836396	Human H3N2 IAVs	Human	H3N2		2007	Singapore	A/Singapore/37C/2007	agtgatgccccattccttgatcggcttcgccgagatcag aggtccctaaggggaagaggcaatactc	-	-
CY044496	Human H3N2 IAVs	Human	H3N2		2007	USA	A/Boston/14/2007	agtgatgccccattccttgatcggcttcgccgagatcag aggtccctaaggggaagaggcaatactc	-	-
CY026247	Human H3N2 IAVs	Human	H3N2		2007	USA	A/California/UR06_0547/2007	agtgatgccccattccttgatcggcttcgccgagatcag aggtccctaaggggaagaggcaatactc	-	-
CY025345	Human H3N2 IAVs	Human	H3N2		2007	USA	A/Florida/UR06_0597/2007	agtgatgccccattccttgatcggcttcgccgagatcag aggtccctaaggggaagaggcaatactc	-	-
CY105482	Human H3N2 IAVs	Human	H3N2		2007	Viet_Nam	A/HaNoi/BM171/2007	agtgatgccccattccttgatcggcttcgccgagatcag aggtccctaaggggaagaggcaatactc	-	-
CY105506	Human H3N2 IAVs	Human	H3N2		2007	Viet_Nam	A/HaNoi/BT251/2007	agtgatgccccattccttgatcggcttcgccgagatcag aggtccctaaggggaagaggcaatactc	-	-
CY104472	Human H3N2 IAVs	Human	H3N2		2007	Viet_Nam	A/HaNoi/Q502/2007	agtgatgccccattccttgatcggcttcgccgagatcag aggtccctaaggggaagaggcaatactc	-	-
CY105746	Human H3N2 IAVs	Human	H3N2		2007	Viet_Nam	A/HaNoi/Q656/2007	agtgatgccccattccttgatcggcttcgccgagatcag aggtccctaaggggaagaggcaatactc	-	-
CY104496	Human H3N2 IAVs	Human	H3N2		2007	Viet_Nam	A/HaNoi/Q672/2007	agtgatgccccattccttgatcggcttcgccgagatcag aggtccctaaggggaagaggcaatactc	-	-
CY105754	Human H3N2 IAVs	Human	H3N2		2007	Viet_Nam	A/HaNoi/Q769/2007	agtgatgccccattccttgatcggcttcgccgagatcag aggtccctaaggggaagaggcaatactc	-	-
CY104536	Human H3N2 IAVs	Human	H3N2		2007	Viet_Nam	A/HaNoi/TX322/2007	agtgatgccccattccttgatcggcttcgccgagatcag aggtccctaaggggaagaggcaatactc	-	-
CY104544	Human H3N2 IAVs	Human	H3N2		2007	Viet_Nam	A/HaNoi/TX327/2007	agtgatgccccattccttgatcggcttcgccgagatcag aggtccctaaggggaagaggcaatactc	-	-
CY105530	Human H3N2 IAVs	Human	H3N2		2007	Viet_Nam	A/Hue/H511/2007	agtgatgccccattccttgatcggcttcgccgagatcag aggtccctaaggggaagaggcaatactc	-	-
CY105538	Human H3N2 IAVs	Human	H3N2		2007	Viet_Nam	A/Hue/H515/2007	agtgatgccccattccttgatcggcttcgccgagatcag aggtccctaaggggaagaggcaatactc	-	-
CY105570	Human H3N2 IAVs	Human	H3N2		2007	Viet_Nam	A/LangSon/LS218/2007	agtgatgccccattccttgatcggcttcgccgagatcag aggtccctaaggggaagaggcaatactc	-	-
CY118950	Human H3N2 IAVs	Human	H3N2		2007	Malaysia	A/Malaysia/1755590/2007	agtgatgccccattccttgatcggcttcgccgagatcag aggtccctaaggggaagaggcaatactc	-	-
CY118958	Human H3N2 IAVs	Human	H3N2		2007	Malaysia	A/Malaysia/1756502/2007	agtgatgccccattccttgatcggcttcgccgagatcag aggtccctaaggggaagaggcaatactc	-	-
CY118966	Human H3N2 IAVs	Human	H3N2		2007	Malaysia	A/Malaysia/1756937/2007	agtgatgccccattccttgatcggcttcgccgagatcag aggtccctaaggggaagaggcaatactc	-	-

CY118063	Human H3N2 IAVs	Human	H3N2		2007	Malaysia	A/Malaysia/1769312/2007	agtgatgccccattccttgatcggcttcgccgagatcag aggtccctaaggggaagaggcaatactc	-	-
CY119054	Human H3N2 IAVs	Human	H3N2		2007	Malaysia	A/Malaysia/1770921/2007	agtgatgccccattccttgatcggcttcgccgagatcag aggtccctaaggggaagaggcaatactc	-	-
CY118990	Human H3N2 IAVs	Human	H3N2		2007	Malaysia	A/Malaysia/1773914/2007	agtgatgccccattccttgatcggcttcgccgagatcag aggtccctaaggggaagaggcaatactc	-	-
CY118071	Human H3N2 IAVs	Human	H3N2		2007	Malaysia	A/Malaysia/1777545/2007	agtgatgccccattccttgatcggcttcgccgagatcag aggtccctaaggggaagaggcaatactc	-	-
CY118998	Human H3N2 IAVs	Human	H3N2		2007	Malaysia	A/Malaysia/1779036/2007	agtgatgccccattccttgatcggcttcgccgagatcag aggtccctaaggggaagaggcaatactc	-	-
CY119006	Human H3N2 IAVs	Human	H3N2		2007	Malaysia	A/Malaysia/1781328/2007	agtgatgccccattccttgatcggcttcgccgagatcag aggtccctaaggggaagaggcaatactc	-	-
CY032441	Human H3N2 IAVs	Human	H3N2		2007	Nicaragua	A/Managua/25/2007	agtgatgccccattccttgatcggcttcgccgagatcag aggtccctaaggggaagaggcaatactc	-	-
CY040222	Human H3N2 IAVs	Human	H3N2		2007	Nicaragua	A/Managua/3012.01/2007	agtgatgccccattccttgatcggcttcgccgagatcag aggtccctaaggggaagaggcaatactc	-	-
CY038531	Human H3N2 IAVs	Human	H3N2		2007	Nicaragua	A/Managua/4127.01/2007	agtgatgccccattccttgatcggcttcgccgagatcag aggtccctaaggggaagaggcaatactc	-	-
CY040214	Human H3N2 IAVs	Human	H3N2		2007	Nicaragua	A/Managua/469.01/2007	agtgatgccccattccttgatcggcttcgccgagatcag aggtccctaaggggaagaggcaatactc	-	-
CY037363	Human H3N2 IAVs	Human	H3N2		2007	Nicaragua	A/Managua/4902.01/2007	agtgatgccccattccttgatcggcttcgccgagatcag aggtccctaaggggaagaggcaatactc	-	-
CY034096	Human H3N2 IAVs	Human	H3N2		2007	Nicaragua	A/Managua/5/2007	agtgatgccccattccttgatcggcttcgccgagatcag aggtccctaaggggaagaggcaatactc	-	-
CY032465	Human H3N2 IAVs	Human	H3N2		2007	Nicaragua	A/Managua/15/2007	agtgatgccccattccttgatcggcttcgccgagatcag aggtccctaaggggaagaggcaatactc	-	-
CY032513	Human H3N2 IAVs	Human	H3N2		2007	Nicaragua	A/Managua/2/2007	agtgatgccccattccttgatcggcttcgccgagatcag aggtccctaaggggaagaggcaatactc	-	-
CY035106	Human H3N2 IAVs	Human	H3N2		2007	Nicaragua	A/Managua/21/2007	agtgatgccccattccttgatcggcttcgccgagatcag aggtccctaaggggaagaggcaatactc	-	-
CY032537	Human H3N2 IAVs	Human	H3N2		2007	Nicaragua	A/Managua/28/2007	agtgatgccccattccttgatcggcttcgccgagatcag aggtccctaaggggaagaggcaatactc	-	-
CY032481	Human H3N2 IAVs	Human	H3N2		2007	Nicaragua	A/Managua/29/2007	agtgatgccccattccttgatcggcttcgccgagatcag aggtccctaaggggaagaggcaatactc	-	-
CY038515	Human H3N2 IAVs	Human	H3N2		2007	Nicaragua	A/Managua/3616.01/2007	agtgatgccccattccttgatcggcttcgccgagatcag aggtccctaaggggaagaggcaatactc	-	-
CY032545	Human H3N2 IAVs	Human	H3N2		2007	Nicaragua	A/Managua/7/2007	agtgatgccccattccttgatcggcttcgccgagatcag aggtccctaaggggaagaggcaatactc	-	-
CY035886	Human H3N2 IAVs	Human	H3N2		2007	USA	A/Mississippi/UR06_0197/2007	agtgatgccccattccttgatcggcttcgccgagatcag aggtccctaaggggaagaggcaatactc	-	-
CY105762	Human H3N2 IAVs	Human	H3N2		2007	Viet_Nam	A/TayNguyen/TN341/2007	agtgatgccccattccttgatcggcttcgccgagatcag aggtccctaaggggaagaggcaatactc	-	-
CY105770	Human H3N2 IAVs	Human	H3N2		2007	Viet_Nam	A/TayNguyen/TN367/2007	agtgatgccccattccttgatcggcttcgccgagatcag aggtccctaaggggaagaggcaatactc	-	-
CY121636	Human H3N2 IAVs	Human	H3N2		2007	Uruguay	A/Uruguay/716/2007	agtgatgccccattccttgatcggcttcgccgagatcag aggtccctaaggggaagaggcaatactc	-	-
CY031559	Human H3N2 IAVs	Human	H3N2		2007	USA	A/Kentucky/UR06_0571/2007	agtgatgccccattccttgatcggcttcgccgagatcag aggtccctaaggggaagaggcaatactc	-	-
CY027799	Human H3N2 IAVs	Human	H3N2		2007	USA	A/Oregon/UR06_0200/2007	agtgatgccccattccttgatcggcttcgccgagatcag aggtccctaaggggaagaggcaatactc	-	-

CY035098	Human H3N2 IAVs	Human	H3N2		2007	Nicaragua	A/Managua/26/2007	agtgatgccccattccttgatcggcttcgccgagatcag<br>aggtccctaaggggaagaggcaatactc	-	-
CY025425	Human H3N2 IAVs	Human	H3N2		2007	USA	A/California/UR06_0347/2007	agtgatgccccattccttgatcggcttcgccgagatcag<br>aggtccctaaggggaagaggcaatactc	-	-
CY027551	Human H3N2 IAVs	Human	H3N2		2007	USA	A/Colorado/UR06_0024/2007	agtgatgccccattccttgatcggcttcgccgagatcag<br>aggtccctaaggggaagaggcaatactc	-	-
CY025353	Human H3N2 IAVs	Human	H3N2		2007	USA	A/Colorado/UR06_0206/2007	agtgatgccccattccttgatcggcttcgccgagatcag<br>aggtccctaaggggaagaggcaatactc	-	-
CY027543	Human H3N2 IAVs	Human	H3N2		2007	USA	A/Colorado/UR06_022/2007	agtgatgccccattccttgatcggcttcgccgagatcag<br>aggtccctaaggggaagaggcaatactc	-	-
CY030057	Human H3N2 IAVs	Human	H3N2		2007	USA	A/Colorado/UR06_0452/2007	agtgatgccccattccttgatcggcttcgccgagatcag<br>aggtccctaaggggaagaggcaatactc	-	-
CY025935	Human H3N2 IAVs	Human	H3N2		2007	USA	A/Colorado/UR06_0453/2007	agtgatgccccattccttgatcggcttcgccgagatcag<br>aggtccctaaggggaagaggcaatactc	-	-
CY025727	Human H3N2 IAVs	Human	H3N2		2007	USA	A/Colorado/UR06_0531/2007	agtgatgccccattccttgatcggcttcgccgagatcag<br>aggtccctaaggggaagaggcaatactc	-	-
CY027575	Human H3N2 IAVs	Human	H3N2		2007	USA	A/Colorado/UR06_0534/2007	agtgatgccccattccttgatcggcttcgccgagatcag<br>aggtccctaaggggaagaggcaatactc	-	-
CY026167	Human H3N2 IAVs	Human	H3N2		2007	USA	A/Colorado/UR06_0535/2007	agtgatgccccattccttgatcggcttcgccgagatcag<br>aggtccctaaggggaagaggcaatactc	-	-
CY025615	Human H3N2 IAVs	Human	H3N2		2007	USA	A/Colorado/UR06_0557/2007	agtgatgccccattccttgatcggcttcgccgagatcag<br>aggtccctaaggggaagaggcaatactc	-	-
CY025711	Human H3N2 IAVs	Human	H3N2		2007	USA	A/Illinois/UR06_0478/2007	agtgatgccccattccttgatcggcttcgccgagatcag<br>aggtccctaaggggaagaggcaatactc	-	-
CY028447	Human H3N2 IAVs	Human	H3N2		2007	USA	A/Kentucky/UR06_0044/2007	agtgatgccccattccttgatcggcttcgccgagatcag<br>aggtccctaaggggaagaggcaatactc	-	-
CY025647	Human H3N2 IAVs	Human	H3N2		2007	USA	A/New_York/UR06_0040/2007	agtgatgccccattccttgatcggcttcgccgagatcag<br>aggtccctaaggggaagaggcaatactc	-	-
CY034418	Human H3N2 IAVs	Human	H3N2		2007	USA	A/New_York/UR06_0607/2007	agtgatgccccattccttgatcggcttcgccgagatcag<br>aggtccctaaggggaagaggcaatactc	-	-
CY028303	Human H3N2 IAVs	Human	H3N2		2007	USA	A/Ohio/UR06_0256/2007	agtgatgccccattccttgatcggcttcgccgagatcag<br>aggtccctaaggggaagaggcaatactc	-	-
CY027687	Human H3N2 IAVs	Human	H3N2		2007	USA	A/Oregon/UR06_0202/2007	agtgatgccccattccttgatcggcttcgccgagatcag<br>aggtccctaaggggaagaggcaatactc	-	-
CY026831	Human H3N2 IAVs	Human	H3N2		2007	USA	A/Oregon/UR06_0450/2007	agtgatgccccattccttgatcggcttcgccgagatcag<br>aggtccctaaggggaagaggcaatactc	-	-
CY027567	Human H3N2 IAVs	Human	H3N2		2007	USA	A/Texas/UR06_0358/2007	agtgatgccccattccttgatcggcttcgccgagatcag<br>aggtccctaaggggaagaggcaatactc	-	-
CY025855	Human H3N2 IAVs	Human	H3N2		2007	USA	A/Texas/UR06_0418/2007	agtgatgccccattccttgatcggcttcgccgagatcag<br>aggtccctaaggggaagaggcaatactc	-	-
CY028479	Human H3N2 IAVs	Human	H3N2		2007	USA	A/Texas/UR06_0603/2007	agtgatgccccattccttgatcggcttcgccgagatcag<br>aggtccctaaggggaagaggcaatactc	-	-
CY025607	Human H3N2 IAVs	Human	H3N2		2007	USA	A/Vermont/UR06_0471/2007	agtgatgccccattccttgatcggcttcgccgagatcag<br>aggtccctaaggggaagaggcaatactc	-	-
CY026775	Human H3N2 IAVs	Human	H3N2		2007	USA	A/Washington/UR06_0252/2007	agtgatgccccattccttgatcggcttcgccgagatcag<br>aggtccctaaggggaagaggcaatactc	-	-
CY026255	Human H3N2 IAVs	Human	H3N2		2007	USA	A/Colorado/UR06_0558/2007	agtgatgccccattccttgatcggcttcgccgagatcag<br>aggtccctaaggggaagaggcaatactc	-	-
CY172739	Human H3N2 IAVs	Human	H3N2		2007	USA	A/New_York/1077/2007	agtgatgccccattccttgatcggcttcgccgagatcag<br>aggtccctaaggggaagaggcaatactc	-	-

CY172747	Human H3N2 IAVs	Human	H3N2		2007	USA	A/New_York/1078/2007	agtgatgccccattccttgatcggcttcgccgagatcag aggtccctaaggggaagaggcaatactc	-	-
CY172795	Human H3N2 IAVs	Human	H3N2		2007	USA	A/New_York/1085/2007	agtgatgccccattccttgatcggcttcgccgagatcag aggtccctaaggggaagaggcaatactc	-	-
CY172811	Human H3N2 IAVs	Human	H3N2		2007	USA	A/New_York/1087/2007	agtgatgccccattccttgatcggcttcgccgagatcag aggtccctaaggggaagaggcaatactc	-	-
CY172827	Human H3N2 IAVs	Human	H3N2		2007	USA	A/New_York/1089/2007	agtgatgccccattccttgatcggcttcgccgagatcag aggtccctaaggggaagaggcaatactc	-	-
CY172843	Human H3N2 IAVs	Human	H3N2		2007	USA	A/New_York/1093/2007	agtgatgccccattccttgatcggcttcgccgagatcag aggtccctaaggggaagaggcaatactc	-	-
CY027871	Human H3N2 IAVs	Human	H3N2		2007	USA	A/Virginia/UR06_0580/2007	agtgatgccccattccttgatcggcttcgccgagatcag aggtccctaaggggaagaggcaatactc	-	-
CY026607	Human H3N2 IAVs	Human	H3N2		2007	USA	A/Washington/UR06_0225/2007	agtgatgccccattccttgatcggcttcgccgagatcag aggtccctaaggggaagaggcaatactc	-	-
KF836399	Human H3N2 IAVs	Human	H3N2		2007	Singapore	A/Singapore/105L/2007	agtgatgccccattccttgatcggcttcgccgagatcag aggtccctaaggggaagaggcaatactc	-	-
KF836394	Human H3N2 IAVs	Human	H3N2		2007	Singapore	A/Singapore/25Z/2007	agtgatgccccattccttgatcggcttcgccgagatcag aggtccctaaggggaagaggcaatactc	-	-
FJ769889	Human H3N2 IAVs	Human	H3N2		2007	Iran	A/Esfahan/501/2007	agtgatgccccattccttgatcggcttcgccgagatcag aggtccctaaggggaagaggcaatactc	-	-
FJ769894	Human H3N2 IAVs	Human	H3N2		2007	Iran	A/Kermanshah/687/2007	agtgatgccccattccttgatcggcttcgccgagatcag aggtccctaaggggaagaggcaatactc	-	-
FJ769888	Human H3N2 IAVs	Human	H3N2		2007	Iran	A/Tehran/487/2007	agtgatgccccattccttgatcggcttcgccgagatcag aggtccctaaggggaagaggcaatactc	-	-
FJ769893	Human H3N2 IAVs	Human	H3N2		2007	Iran	A/Tehran/631/2007	agtgatgccccattccttgatcggcttcgccgagatcag aggtccctaaggggaagaggcaatactc	-	-
CY104682	Human H3N2 IAVs	Human	H3N2		2007	Viet_Nam	A/TayNguyen/HCM955/2007	agtgatgccccattccttgatcggcttcgccgagatcag aggtccctaaggggaagaggcaatactc	-	-
CY105490	Human H3N2 IAVs	Human	H3N2		2007	Viet_Nam	A/HaNoi/BM238/2007	agtgatgccccattccttgatcggcttcgccgagatcag aggtccctaaggggaagaggcaatactc	-	-
CY026751	Human H3N2 IAVs	Human	H3N2		2007	USA	A/Illinois/UR06_0528/2007	agtgatgccccattccttgatcggcttcgccgagatcag aggtccctaaggggaagaggcaatactc	-	-
CY026263	Human H3N2 IAVs	Human	H3N2		2007	USA	A/Illinois/UR06_0555/2007	agtgatgccccattccttgatcggcttcgccgagatcag aggtccctaaggggaagaggcaatactc	-	-
FJ009458	Human H3N2 IAVs	Human	H3N2		2007	South_Korea	A/Cheongju/H383/2007	agtgatgccccattccttgatcggcttcgccgagatcag aggtccctaaggggaagaggcaatactc	-	-
FJ009460	Human H3N2 IAVs	Human	H3N2		2007	South_Korea	A/Cheongju/H395/2007	agtgatgccccattccttgatcggcttcgccgagatcag aggtccctaaggggaagaggcaatactc	-	-
CY105818	Human H3N2 IAVs	Human	H3N2		2007	Viet_Nam	A/HaNoi/Q924/2007	agtgatgccccattccttgatcggcttcgccgagatcag aggtccctaaggggaagaggcaatactc	-	-
FJ009459	Human H3N2 IAVs	Human	H3N2		2007	South_Korea	A/Cheongju/H393/2007	agtgatgccccattccttgatcggcttcgccgagatcag aggtccctaaggggaagaggcaatactc	-	-
CY118055	Human H3N2 IAVs	Human	H3N2		2007	Malaysia	A/Malaysia/1768409/2007	agtgatgccccattccttgatcggcttcgccgagatcag aggtccctaaggggaagaggcaatactc	-	-
CY026559	Human H3N2 IAVs	Human	H3N2		2007	USA	A/Texas/UR06_0356/2007	agtgatgccccattccttgatcggcttcgccgagatcag aggtccctaaggggaagaggcaatactc	-	-
CY104368	Human H3N2 IAVs	Human	H3N2		2007	Viet_Nam	A/HoaBinh/HB409/2007	agtgatgccccattccttgatcggcttcgccgagatcag aggtccctaaggggaagaggcaatactc	-	-
AB442007	Human H3N2 IAVs	Human	H3N2		2007	Japan	A/Sendai_H/F193/2007	agtgatgccccattccttgatcggcttcgccgagatcag aggtccctaaggggaagaggcaatactc	-	-

CY104504	Human H3N2 IAVs	Human	H3N2		2007	Viet_Nam	A/HaNoi/Q748/2007	agtgatgccccattccttgatcggcttcgccgagatcag<br>aggtccctaaggggaagaggcaatactc	-	-
CY172819	Human H3N2 IAVs	Human	H3N2		2007	USA	A/New_York/1088/2007	agtgatgccccattccttgatcggcttcgccgagatcag<br>aggtccctaaggggaagaggcaatactc	-	-
CY025281	Human H3N2 IAVs	Human	H3N2		2007	USA	A/Illinois/UR06_0567/2007	agtgatgccccattccttgatcggcttcgccgagatcag<br>aggtccctaaggggaagaggcaatactc	-	-
CY104448	Human H3N2 IAVs	Human	H3N2		2007	Viet_Nam	A/HaNoi/Q118/2007	agtgatgccccattccttgatcggcttcgccgagatcag<br>aggtccctaaggggaagaggcaatactc	-	-
CY105714	Human H3N2 IAVs	Human	H3N2		2007	Viet_Nam	A/HaNoi/Q499/2007	agtgatgccccattccttgatcggcttcgccgagatcag<br>aggtccctaaggggaagaggcaatactc	-	-
CY104528	Human H3N2 IAVs	Human	H3N2		2007	Viet_Nam	A/HaNoi/TX320/2007	agtgatgccccattccttgatcggcttcgccgagatcag<br>aggtccctaaggggaagaggcaatactc	-	-
CY040206	Human H3N2 IAVs	Human	H3N2		2007	Nicaragua	A/Managua/2347.01/2007	agtgatgccccattccttgatcggcttcgccgagatcag<br>aggtccctaaggggaagaggcaatactc	-	-
CY032553	Human H3N2 IAVs	Human	H3N2		2007	Nicaragua	A/Managua/31/2007	agtgatgccccattccttgatcggcttcgccgagatcag<br>aggtccctaaggggaagaggcaatactc	-	-
CY032521	Human H3N2 IAVs	Human	H3N2		2007	Nicaragua	A/Managua/33/2007	agtgatgccccattccttgatcggcttcgccgagatcag<br>aggtccctaaggggaagaggcaatactc	-	-
CY032497	Human H3N2 IAVs	Human	H3N2		2007	Nicaragua	A/Managua/34/2007	agtgatgccccattccttgatcggcttcgccgagatcag<br>aggtccctaaggggaagaggcaatactc	-	-
KJ855399	Human H3N2 IAVs	Human	H3N2		2007	Mexico	A/Mexico/NAY2090/2007	agtgatgccccattccttgatcggcttcgccgagatcag<br>aggtccctaaggggaagaggcaatactc	-	-
CY092245	Human H3N2 IAVs	Human	H3N2		2007	USA	A/California/NHRC0001/2007	agtgatgccccattccttgatcggcttcgccgagatcag<br>aggtccctaaggggaagaggcaatactc	-	-
CY026791	Human H3N2 IAVs	Human	H3N2		2007	USA	A/California/UR06_0565/2007	agtgatgccccattccttgatcggcttcgccgagatcag<br>aggtccctaaggggaagaggcaatactc	-	-
CY025481	Human H3N2 IAVs	Human	H3N2		2007	USA	A/Illinois/UR06_0334/2007	agtgatgccccattccttgatcggcttcgccgagatcag<br>aggtccctaaggggaagaggcaatactc	-	-
CY027719	Human H3N2 IAVs	Human	H3N2		2007	USA	A/Illinois/UR06_0402/2007	agtgatgccccattccttgatcggcttcgccgagatcag<br>aggtccctaaggggaagaggcaatactc	-	-
CY027591	Human H3N2 IAVs	Human	H3N2		2007	USA	A/Illinois/UR06_0546/2007	agtgatgccccattccttgatcggcttcgccgagatcag<br>aggtccctaaggggaagaggcaatactc	-	-
CY172803	Human H3N2 IAVs	Human	H3N2		2007	USA	A/New_York/1086/2007	agtgatgccccattccttgatcggcttcgccgagatcag<br>aggtccctaaggggaagaggcaatactc	-	-
CY026711	Human H3N2 IAVs	Human	H3N2		2007	USA	A/Colorado/UR06_0454/2007	agtgatgccccattccttgatcggcttcgccgagatcag<br>aggtccctaaggggaagaggcaatactc	-	-
CY070148	Human H3N2 IAVs	Human	H3N2		2007	Peru	A/Peru/WRAIR1508P/2007	agtgatgccccattccttgatcggcttcgccgagatcag<br>aggtccctaaggggaagaggcaatactc	-	-
FJ769891	Human H3N2 IAVs	Human	H3N2		2007	Iran	A/Kermanshah/580/2007	agtgatgccccattccttgatcggcttcgccgagatcag<br>aggtccctaaggggaagaggcaatactc	-	-
CY040126	Human H3N2 IAVs	Human	H3N2		2007	Taiwan	A/Taiwan/72106/2007	agtgatgccccattccttgatcggcttcgccgagatcag<br>aggtccctaaggggaagaggcaatactc	-	-
CY032489	Human H3N2 IAVs	Human	H3N2		2007	Nicaragua	A/Managua/22/2007	agtgatgccccattccttgatcggcttcgccgagatcag<br>aggtccctaaggggaagaggcaatactc	-	-
CY172907	Human H3N2 IAVs	Human	H3N2		2007	USA	A/New_York/1103/2007	agtgatgccccattccttgatcggcttcgccgagatcag<br>aggtccctaaggggaagaggcaatactc	-	-
CY028375	Human H3N2 IAVs	Human	H3N2		2007	USA	A/Oregon/UR06_0221/2007	agtgatgccccattccttgatcggcttcgccgagatcag<br>aggtccctaaggggaagaggcaatactc	-	-
CY027127	Human H3N2 IAVs	Human	H3N2		2007	USA	A/Oregon/UR06_0272/2007	agtgatgccccattccttgatcggcttcgccgagatcag<br>aggtccctaaggggaagaggcaatactc	-	-

CY027079	Human H3N2 IAVs	Human	H3N2		2007	USA	A/Oregon/UR06_0273/2007	agtgatgccccattccttgatcggcttcgccgagatcag aggtccctaaggggaagaggcaatactc	-	-
CY026847	Human H3N2 IAVs	Human	H3N2		2007	USA	A/Oregon/UR06_0289/2007	agtgatgccccattccttgatcggcttcgccgagatcag aggtccctaaggggaagaggcaatactc	-	-
CY027111	Human H3N2 IAVs	Human	H3N2		2007	USA	A/Oregon/UR06_0389/2007	agtgatgccccattccttgatcggcttcgccgagatcag aggtccctaaggggaagaggcaatactc	-	-
CY105706	Human H3N2 IAVs	Human	H3N2		2007	Viet_Nam	A/HaNoi/Q492/2007	agtgatgccccattccttgatcggcttcgccgagatcag aggtccctaaggggaagaggcaatactc	-	-
CY105730	Human H3N2 IAVs	Human	H3N2		2007	Viet_Nam	A/HaNoi/Q578/2007	agtgatgccccattccttgatcggcttcgccgagatcag aggtccctaaggggaagaggcaatactc	-	-
CY172867	Human H3N2 IAVs	Human	H3N2		2007	USA	A/New_York/1097/2007	agtgatgccccattccttgatcggcttcgccgagatcag aggtccctaaggggaagaggcaatactc	-	-
CY033461	Human H3N2 IAVs	Human	H3N2		2007	USA	A/Vermont/UR06_0329/2007	agtgatgccccattccttgatcggcttcgccgagatcag aggtccctaaggggaagaggcaatactc	-	-
CY027199	Human H3N2 IAVs	Human	H3N2		2007	USA	A/Vermont/UR06_0469/2007	agtgatgccccattccttgatcggcttcgccgagatcag aggtccctaaggggaagaggcaatactc	-	-
CY034410	Human H3N2 IAVs	Human	H3N2		2007	USA	A/Vermont/UR06_0512/2007	agtgatgccccattccttgatcggcttcgccgagatcag aggtccctaaggggaagaggcaatactc	-	-
CY025544	Human H3N2 IAVs	Human	H3N2		2007	USA	A/Vermont/UR06_0448/2007	agtgatgccccattccttgatcggcttcgccgagatcag aggtccctaaggggaagaggcaatactc	-	-
CY025879	Human H3N2 IAVs	Human	H3N2		2007	USA	A/Vermont/UR06_0470/2007	agtgatgccccattccttgatcggcttcgccgagatcag aggtccctaaggggaagaggcaatactc	-	-
CY025871	Human H3N2 IAVs	Human	H3N2		2007	USA	A/Vermont/UR06_0483/2007	agtgatgccccattccttgatcggcttcgccgagatcag aggtccctaaggggaagaggcaatactc	-	-
CY025887	Human H3N2 IAVs	Human	H3N2		2007	USA	A/Vermont/UR06_0486/2007	agtgatgccccattccttgatcggcttcgccgagatcag aggtccctaaggggaagaggcaatactc	-	-
CY025417	Human H3N2 IAVs	Human	H3N2		2007	USA	A/Vermont/UR06_0524/2007	agtgatgccccattccttgatcggcttcgccgagatcag aggtccctaaggggaagaggcaatactc	-	-
CY025903	Human H3N2 IAVs	Human	H3N2		2007	USA	A/Illinois/UR06_0436/2007	agtgatgccccattccttgatcggcttcgccgagatcag aggtccctaaggggaagaggcaatactc	-	-
CY025505	Human H3N2 IAVs	Human	H3N2		2007	USA	A/Vermont/UR06_0484/2007	agtgatgccccattccttgatcggcttcgccgagatcag aggtccctaaggggaagaggcaatactc	-	-
CY033149	Human H3N2 IAVs	Human	H3N2		2007	Nicaragua	A/Managua/30/2007	agtgatgccccattccttgatcggcttcgccgagatcag aggtccctaaggggaagaggcaatactc	-	-
CY038915	Human H3N2 IAVs	Human	H3N2		2007	Nicaragua	A/Managua/419.01/2007	agtgatgccccattccttgatcggcttcgccgagatcag aggtccctaaggggaagaggcaatactc	-	-
CY039443	Human H3N2 IAVs	Human	H3N2		2007	Nicaragua	A/Managua/4348.01/2007	agtgatgccccattccttgatcggcttcgccgagatcag aggtccctaaggggaagaggcaatactc	-	-
CY105626	Human H3N2 IAVs	Human	H3N2		2007	Viet_Nam	A/HaNoi/N317/2007	agtgatgccccattccttgatcggcttcgccgagatcag aggtccctaaggggaagaggcaatactc	-	-
CY118974	Human H3N2 IAVs	Human	H3N2		2007	Malaysia	A/Malaysia/1767091/2007	agtgatgccccattccttgatcggcttcgccgagatcag aggtccctaaggggaagaggcaatactc	-	-
CY034088	Human H3N2 IAVs	Human	H3N2		2007	Nicaragua	A/Managua/17/2007	agtgatgccccattccttgatcggcttcgccgagatcag aggtccctaaggggaagaggcaatactc	-	-
CY105722	Human H3N2 IAVs	Human	H3N2		2007	Viet_Nam	A/HaNoi/Q545/2007	agtgatgccccattccttgatcggcttcgccgagatcag aggtccctaaggggaagaggcaatactc	-	-
FJ769892	Human H3N2 IAVs	Human	H3N2		2007	Iran	A/Tehran/630/2007	agtgatgccccattccttgatcggcttcgccgagatcag aggtccctaaggggaagaggcaatactc	-	-
FJ769895	Human H3N2 IAVs	Human	H3N2		2007	Iran	A/Tehran/762/2007	agtgatgccccattccttgatcggcttcgccgagatcag aggtccctaaggggaagaggcaatactc	-	-

CY039091	Human H3N2 IAVs	Human	H3N2		2007	Australia	A/Brisbane/10/2007	agtgatgccccattccttgatcggcttcgccgagatcag<br>aggtccctaaggggaagaggcaatactc	-	-
CY035026	Human H3N2 IAVs	Human	H3N2		2007	Australia	A/Brisbane/10/2007	agtgatgccccattccttgatcggcttcgccgagatcag<br>aggtccctaaggggaagaggcaatactc	-	-
CY105578	Human H3N2 IAVs	Human	H3N2		2007	Viet_Nam	A/LangSon/LS281/2007	agtgatgccccattccttgatcggcttcgccgagatcag<br>aggtccctaaggggaagaggcaatactc	-	-
CY032529	Human H3N2 IAVs	Human	H3N2		2007	Nicaragua	A/Managua/14/2007	agtgatgccccattccttgatcggcttcgccgagatcag<br>aggtccctaaggggaagaggcaatactc	-	-
CY032473	Human H3N2 IAVs	Human	H3N2		2007	Nicaragua	A/Managua/16/2007	agtgatgccccattccttgatcggcttcgccgagatcag<br>aggtccctaaggggaagaggcaatactc	-	-
CY032505	Human H3N2 IAVs	Human	H3N2		2007	Nicaragua	A/Managua/18/2007	agtgatgccccattccttgatcggcttcgccgagatcag<br>aggtccctaaggggaagaggcaatactc	-	-
CY038563	Human H3N2 IAVs	Human	H3N2		2007	Nicaragua	A/Managua/1867.01/2007	agtgatgccccattccttgatcggcttcgccgagatcag<br>aggtccctaaggggaagaggcaatactc	-	-
CY032433	Human H3N2 IAVs	Human	H3N2		2007	Nicaragua	A/Managua/20/2007	agtgatgccccattccttgatcggcttcgccgagatcag<br>aggtccctaaggggaagaggcaatactc	-	-
CY032449	Human H3N2 IAVs	Human	H3N2		2007	Nicaragua	A/Managua/27/2007	agtgatgccccattccttgatcggcttcgccgagatcag<br>aggtccctaaggggaagaggcaatactc	-	-
CY032457	Human H3N2 IAVs	Human	H3N2		2007	Nicaragua	A/Managua/32/2007	agtgatgccccattccttgatcggcttcgccgagatcag<br>aggtccctaaggggaagaggcaatactc	-	-
CY038891	Human H3N2 IAVs	Human	H3N2		2007	Nicaragua	A/Managua/3556.01/2007	agtgatgccccattccttgatcggcttcgccgagatcag<br>aggtccctaaggggaagaggcaatactc	-	-
CY040198	Human H3N2 IAVs	Human	H3N2		2007	Nicaragua	A/Managua/68.01/2007	agtgatgccccattccttgatcggcttcgccgagatcag<br>aggtccctaaggggaagaggcaatactc	-	-
CY035090	Human H3N2 IAVs	Human	H3N2		2007	Nicaragua	A/Managua/3/2007	agtgatgccccattccttgatcggcttcgccgagatcag<br>aggtccctaaggggaagaggcaatactc	-	-
CY038523	Human H3N2 IAVs	Human	H3N2		2007	Nicaragua	A/Managua/112.01/2007	agtgatgccccattccttgatcggcttcgccgagatcag<br>aggtccctaaggggaagaggcaatactc	-	-
CY038547	Human H3N2 IAVs	Human	H3N2		2007	Nicaragua	A/Managua/3243.01/2007	agtgatgccccattccttgatcggcttcgccgagatcag<br>aggtccctaaggggaagaggcaatactc	-	-
CY092253	Human H3N2 IAVs	Human	H3N2		2007	USA	A/California/NHRC0002/2007	agtgatgccccattccttgatcggcttcgccgagatcag<br>aggtccctaaggggaagaggcaatactc	-	-
CY105698	Human H3N2 IAVs	Human	H3N2		2007	Viet_Nam	A/HaNoi/Q464/2007	agtgatgccccattccttgatcggcttcgccgagatcag<br>aggtccctaaggggaagaggcaatactc	-	-
CY026887	Human H3N2 IAVs	Human	H3N2		2007	USA	A/Kentucky/UR06_0158/2007	agtgatgccccattccttgatcggcttcgccgagatcag<br>aggtccctaaggggaagaggcaatactc	-	-
CY026311	Human H3N2 IAVs	Human	H3N2		2007	USA	A/Virginia/UR06_0021/2007	agtgatgccccattccttgatcggcttcgccgagatcag<br>aggtccctaaggggaagaggcaatactc	-	-
CY105738	Human H3N2 IAVs	Human	H3N2		2007	Viet_Nam	A/HaNoi/Q638/2007	agtgatgccccattccttgatcggcttcgccgagatcag<br>aggtccctaaggggaagaggcaatactc	-	-
CY105514	Human H3N2 IAVs	Human	H3N2		2007	Viet_Nam	A/HaNoi/GS0737/2007	agtgatgccccattccttgatcggcttcgccgagatcag<br>aggtccctaaggggaagaggcaatactc	-	-
CY027583	Human H3N2 IAVs	Human	H3N2		2007	USA	A/California/UR06_0463/2007	agtgatgccccattccttgatcggcttcgccgagatcag<br>aggtccctaaggggaagaggcaatactc	-	-
CY026031	Human H3N2 IAVs	Human	H3N2		2007	USA	A/Illinois/UR06_0030/2007	agtgatgccccattccttgatcggcttcgccgagatcag<br>aggtccctaaggggaagaggcaatactc	-	-
CY026023	Human H3N2 IAVs	Human	H3N2		2007	USA	A/Illinois/UR06_0036/2007	agtgatgccccattccttgatcggcttcgccgagatcag<br>aggtccctaaggggaagaggcaatactc	-	-
CY104344	Human H3N2 IAVs	Human	H3N2		2007	Viet_Nam	A/HaNoi/GS0740/2007	agtgatgccccattccttgatcggcttcgccgagatcag<br>aggtccctaaggggaagaggcaatactc	-	-

CY104360	Human H3N2 IAVs	Human	H3N2		2007	Viet_Nam	A/HoaBinh/HB378/2007	agtgatgccccattccttgatcggcttcgccgagatcag aggtccctaaggggaagaggcaatactc	-	-
CY028744	Human H3N2 IAVs	Human	H3N2		2007	USA	A/Virginia/UR06_0489/2007	agtgatgccccattccttgatcggcttcgccgagatcag aggtccctaaggggaagaggcaatactc	-	-
FJ769885	Human H3N2 IAVs	Human	H3N2		2007	Iran	A/Tehran/379/2007	agtgatgccccattccttgatcggcttcgccgagatcag aggtccctaaggggaagaggcaatactc	-	-
CY172763	Human H3N2 IAVs	Human	H3N2		2007	USA	A/New_York/1080/2007	agtgatgccccattccttgatcggcttcgccgagatcag aggtccctaaggggaagaggcaatactc	-	-
CY114417	Human H3N2 IAVs	Human	H3N2		2007	Netherlands	A/Netherlands/348/2007	agtgatgccccattccttgatcggcttcgccgagatcag aggtccctaaggggaagaggcaatactc	-	-
CY172779	Human H3N2 IAVs	Human	H3N2		2007	USA	A/New_York/1083/2007	agtgatgccccattccttgatcggcttcgccgagatcag aggtccctaaggggaagaggcaatactc	-	-
CY025719	Human H3N2 IAVs	Human	H3N2		2007	USA	A/New_York/UR06_0437/2007	agtgatgccccattccttgatcggcttcgccgagatcag aggtccctaaggggaagaggcaatactc	-	-
CY114409	Human H3N2 IAVs	Human	H3N2		2007	Netherlands	A/Netherlands/69/2007	agtgatgccccattccttgatcggcttcgccgagatcag aggtccctaaggggaagaggcaatactc	-	-
CY105522	Human H3N2 IAVs	Human	H3N2		2007	Viet_Nam	A/HaNoi/GS0738/2007	agtgatgccccattccttgatcggcttcgccgagatcag aggtccctaaggggaagaggcaatactc	-	-
CY025759	Human H3N2 IAVs	Human	H3N2		2007	USA	A/Florida/UR06_0150/2007	agtgatgccccattccttgatcggcttcgccgagatcag aggtccctaaggggaagaggcaatactc	-	-
CY104464	Human H3N2 IAVs	Human	H3N2		2007	Viet_Nam	A/HaNoi/Q430/2007	agtgatgccccattccttgatcggcttcgccgagatcag aggtccctaaggggaagaggcaatactc	-	-
CY172891	Human H3N2 IAVs	Human	H3N2		2007	USA	A/New_York/1100/2007	agtgatgccccattccttgatcggcttcgccgagatcag aggtccctaaggggaagaggcaatactc	-	-
CY104488	Human H3N2 IAVs	Human	H3N2		2007	Viet_Nam	A/HaNoi/Q664/2007	agtgatgccccattccttgatcggcttcgccgagatcag aggtccctaaggggaagaggcaatactc	-	-
CY105546	Human H3N2 IAVs	Human	H3N2		2007	Viet_Nam	A/Hue/H519/2007	agtgatgccccattccttgatcggcttcgccgagatcag aggtccctaaggggaagaggcaatactc	-	-
CY105554	Human H3N2 IAVs	Human	H3N2		2007	Viet_Nam	A/Hue/H521/2007	agtgatgccccattccttgatcggcttcgccgagatcag aggtccctaaggggaagaggcaatactc	-	-
CY172851	Human H3N2 IAVs	Human	H3N2		2007	USA	A/New_York/1095/2007	agtgatgccccattccttgatcggcttcgccgagatcag aggtccctaaggggaagaggcaatactc	-	-
CY121532	Human H3N2 IAVs	Human	H3N2		2007	USA	A/Wisconsin/03/2007	agtgatgccccattccttgatcggcttcgccgagatcag aggtccctaaggggaagaggcaatactc	-	-
CY172859	Human H3N2 IAVs	Human	H3N2		2007	USA	A/New_York/1096/2007	agtgatgccccattccttgatcggcttcgccgagatcag aggtccctaaggggaagaggcaatactc	-	-
HQ853532	Human H3N2 IAVs	Human	H3N2		2007	India	A/KOL/1075/2007	agtgatgccccattccttgatcggcttcgccgagatcag aggtccctaaggggaagaggcaatactc	-	-
HQ853528	Human H3N2 IAVs	Human	H3N2		2007	India	A/KOL/595/2007	agtgatgccccattccttgatcggcttcgccgagatcag aggtccctaaggggaagaggcaatactc	-	-
CY172883	Human H3N2 IAVs	Human	H3N2		2007	USA	A/New_York/1099/2007	agtgatgccccattccttgatcggcttcgccgagatcag aggtccctaaggggaagaggcaatactc	-	-
CY118119	Human H3N2 IAVs	Human	H3N2		2007	Malaysia	A/Malaysia/1912341/2007	agtgatgccccattccttgatcggcttcgccgagatcag aggtccctaaggggaagaggcaatactc	-	-
CY119014	Human H3N2 IAVs	Human	H3N2		2007	Malaysia	A/Malaysia/1912346/2007	agtgatgccccattccttgatcggcttcgccgagatcag aggtccctaaggggaagaggcaatactc	-	-
CY104336	Human H3N2 IAVs	Human	H3N2		2007	Viet_Nam	A/HaNoi/BT262/2007	agtgatgccccattccttgatcggcttcgccgagatcag aggtccctaaggggaagaggcaatactc	-	-
CY040070	Human H3N2 IAVs	Human	H3N2		2007	Taiwan	A/Taiwan/2361/2007	agtgatgccccattccttgatcggcttcgccgagatcag aggtccctaaggggaagaggcaatactc	-	-

CY118127	Human H3N2 IAVs	Human	H3N2		2007	Malaysia	A/Malaysia/1918618/2007	agtgatgccccattccttgatcggcttcgccgagatcag<br>aggtccctaaggggaagaggcaatactc	-	-
CY025743	Human H3N2 IAVs	Human	H3N2		2007	USA	A/California/UR06_0118/2007	agtgatgccccattccttgatcggcttcgccgagatcag<br>aggtccctaaggggaagaggcaatactc	-	-
CY105610	Human H3N2 IAVs	Human	H3N2		2007	Viet_Nam	A/HaNoi/N080/2007	agtgatgccccattccttgatcggcttcgccgagatcag<br>aggtccctaaggggaagaggcaatactc	-	-
CY172899	Human H3N2 IAVs	Human	H3N2		2007	USA	A/New_York/1102/2007	agtgatgccccattccttgatcggcttcgccgagatcag<br>aggtccctaaggggaagaggcaatactc	-	-
CY039411	Human H3N2 IAVs	Human	H3N2		2007	Taiwan	A/Taiwan/70113/2007	agtgatgccccattccttgatcggcttcgccgagatcag<br>aggtccctaaggggaagaggcaatactc	-	-
CY026151	Human H3N2 IAVs	Human	H3N2		2007	USA	A/Colorado/UR06_0023/2007	agtgatgccccattccttgatcggcttcgccgagatcag<br>aggtccctaaggggaagaggcaatactc	-	-
HQ853527	Human H3N2 IAVs	Human	H3N2		2007	India	A/KOL/524/2007	agtgatgccccattccttgatcggcttcgccgagatcag<br>aggtccctaaggggaagaggcaatactc	-	-
HQ853529	Human H3N2 IAVs	Human	H3N2		2007	India	A/KOL/960/2007	agtgatgccccattccttgatcggcttcgccgagatcag<br>aggtccctaaggggaagaggcaatactc	-	-
HQ853530	Human H3N2 IAVs	Human	H3N2		2007	India	A/KOL/989/2007	agtgatgccccattccttgatcggcttcgccgagatcag<br>aggtccctaaggggaagaggcaatactc	-	-
CY105498	Human H3N2 IAVs	Human	H3N2		2007	Viet_Nam	A/HaNoi/BM38/2007	agtgatgccccattccttgatcggcttcgccgagatcag<br>aggtccctaaggggaagaggcaatactc	-	-
CY040054	Human H3N2 IAVs	Human	H3N2		2007	Taiwan	A/Taiwan/70307/2007	agtgatgccccattccttgatcggcttcgccgagatcag<br>aggtccctaaggggaagaggcaatactc	-	-
HQ853526	Human H3N2 IAVs	Human	H3N2		2007	India	A/KOL/600/2007	agtgatgccccattccttgatcggcttcgccgagatcag<br>aggtccctaaggggaagaggcaatactc	-	-
HQ853531	Human H3N2 IAVs	Human	H3N2		2007	India	A/KOL/994/2007	agtgatgccccattccttgatcggcttcgccgagatcag<br>aggtccctaaggggaagaggcaatactc	-	-
CY049752	Human H3N2 IAVs	Human	H3N2		2007	Japan	A/Tokyo/Ut_Sk_1/2007	agtgatgccccattccttgatcggcttcgccgagatcag<br>aggtccctaaggggaagaggcaatactc	-	-
AB442009	Human H3N2 IAVs	Human	H3N2		2007	Japan	A/Sendai_H/441/2007	agtgatgccccattccttgatcggcttcgccgagatcag<br>aggtccctaaggggaagaggcaatactc	-	-
CY104352	Human H3N2 IAVs	Human	H3N2		2007	Viet_Nam	A/Hue/H020/2007	agtgatgccccattccttgatcggcttcgccgagatcag<br>aggtccctaaggggaagaggcaatactc	-	-
CY039403	Human H3N2 IAVs	Human	H3N2		2007	Taiwan	A/Taiwan/70002/2007	agtgatgccccattccttgatcggcttcgccgagatcag<br>aggtccctaaggggaagaggcaatactc	-	-
AB442005	Human H3N2 IAVs	Human	H3N2		2007	Japan	A/Sendai_H/F093/2007	agtgatgccccattccttgatcggcttcgccgagatcag<br>aggtccctaaggggaagaggcaatactc	-	-
AB442004	Human H3N2 IAVs	Human	H3N2		2007	Japan	A/Sendai_H/115/2007	agtgatgccccattccttgatcggcttcgccgagatcag<br>aggtccctaaggggaagaggcaatactc	-	-
KF836398	Human H3N2 IAVs	Human	H1N1		2007	Singapore	A/Singapore/66L/2007	agtgatgccccattccttgatcggcttcgccgagatcag<br>aggtccctaaggggaagaggcaatactc	-	-
CY040134	Human H3N2 IAVs	Human	H3N2		2007	Taiwan	A/Taiwan/1452/2007	agtgatgccccattccttgatcggcttcgccgagatcag<br>aggtccctaaggggaagaggcaatactc	-	-
FJ360625	Human H3N2 IAVs	Human	H3N2		2007	China	A/Guangzhou/84/2007	agtgatgccccattccttgatcggcttcgccgagatcag<br>aggtccctaaggggagggaggaatattt	-	-
CY105810	Human H3N2 IAVs	Human	H3N2		2007	Viet_Nam	A/HaNoi/TX072/2007	agtgatgccccattccttgatcgggttcgacgagatca<br>gaggtccctaaggggaagaggcaacactc	-	-
CY030201	Human H3N2 IAVs	Human	H3N2		2007	USA	A/Illinois/UR06_0600/2007	agtgatgccccattccttgatcgggttcgccgagatca<br>gaggtcactaaggggaagaggcaacactc	+	+
CY025863	Human H3N2 IAVs	Human	H3N2		2007	USA	A/Alabama/UR06_0545/2007	agtgatgccccattccttgatcgggttcgccgagatca<br>gaggtccctaaggggaagaggcaaaactc	-	-

CY172771	Human H3N2 IAVs	Human	H3N2		2007	USA	A/New_York/1082/2007	agtgatgccccattccttgatcgggttcgccgagatca gaggtccctaaggggaagaggcaaaactc	-	-
CY172787	Human H3N2 IAVs	Human	H3N2		2007	USA	A/New_York/1084/2007	agtgatgccccattccttgatcgggttcgccgagatca gaggtccctaaggggaagaggcaacactc	-	-
CY172835	Human H3N2 IAVs	Human	H3N2		2007	USA	A/New_York/1090/2007	agtgatgccccattccttgatcgggttcgccgagatca gaggtccctaaggggaagaggcaacactc	-	-
CY031567	Human H3N2 IAVs	Human	H3N2		2007	USA	A/California/UR06_0588/2007	agtgatgccccattccttgatcgggttcgccgagatca gaggtccctaaggggaagaggcaacactc	-	-
CY026199	Human H3N2 IAVs	Human	H3N2		2007	USA	A/California/UR06_0589/2007	agtgatgccccattccttgatcgggttcgccgagatca gaggtccctaaggggaagaggcaacactc	-	-
CY025751	Human H3N2 IAVs	Human	H3N2		2007	USA	A/Texas/UR06_0480/2007	agtgatgccccattccttgatcgggttcgccgagatca gaggtccctaaggggaagaggcaacactc	-	-
CY105682	Human H3N2 IAVs	Human	H3N2		2007	Viet_Nam	A/HaNam/Q187/2007	agtgatgccccattccttgatcgggttcgccgagatca gaggtccctaaggggaagaggcaacactc	-	-
CY104328	Human H3N2 IAVs	Human	H3N2		2007	Viet_Nam	A/HaNoi/BT17/2007	agtgatgccccattccttgatcgggttcgccgagatca gaggtccctaaggggaagaggcaacactc	-	-
CY104392	Human H3N2 IAVs	Human	H3N2		2007	Viet_Nam	A/HaNoi/N015/2007	agtgatgccccattccttgatcgggttcgccgagatca gaggtccctaaggggaagaggcaacactc	-	-
CY105602	Human H3N2 IAVs	Human	H3N2		2007	Viet_Nam	A/HaNoi/N073/2007	agtgatgccccattccttgatcgggttcgccgagatca gaggtccctaaggggaagaggcaacactc	-	-
CY104416	Human H3N2 IAVs	Human	H3N2		2007	Viet_Nam	A/HaNoi/N078/2007	agtgatgccccattccttgatcgggttcgccgagatca gaggtccctaaggggaagaggcaacactc	-	-
CY104424	Human H3N2 IAVs	Human	H3N2		2007	Viet_Nam	A/HaNoi/Q011/2007	agtgatgccccattccttgatcgggttcgccgagatca gaggtccctaaggggaagaggcaacactc	-	-
CY104432	Human H3N2 IAVs	Human	H3N2		2007	Viet_Nam	A/HaNoi/Q039/2007	agtgatgccccattccttgatcgggttcgccgagatca gaggtccctaaggggaagaggcaacactc	-	-
CY104440	Human H3N2 IAVs	Human	H3N2		2007	Viet_Nam	A/HaNoi/Q097/2007	agtgatgccccattccttgatcgggttcgccgagatca gaggtccctaaggggaagaggcaacactc	-	-
CY105786	Human H3N2 IAVs	Human	H3N2		2007	Viet_Nam	A/HaNoi/TX022/2007	agtgatgccccattccttgatcgggttcgccgagatca gaggtccctaaggggaagaggcaacactc	-	-
CY105794	Human H3N2 IAVs	Human	H3N2		2007	Viet_Nam	A/HaNoi/TX023/2007	agtgatgccccattccttgatcgggttcgccgagatca gaggtccctaaggggaagaggcaacactc	-	-
CY104512	Human H3N2 IAVs	Human	H3N2		2007	Viet_Nam	A/HaNoi/TX050/2007	agtgatgccccattccttgatcgggttcgccgagatca gaggtccctaaggggaagaggcaacactc	-	-
CY118982	Human H3N2 IAVs	Human	H3N2		2007	Malaysia	A/Malaysia/1768035/2007	agtgatgccccattccttgatcgggttcgccgagatca gaggtccctaaggggaagaggcaacactc	-	-
CY030209	Human H3N2 IAVs	Human	H3N2		2007	USA	A/Virginia/UR06_0605/2007	agtgatgccccattccttgatcgggttcgccgagatca gaggtccctaaggggaagaggcaacactc	-	-
AB442008	Human H3N2 IAVs	Human	H3N2		2007	LAB	A/Sendai_H/271/2007	agtgatgccccattccttgatcgggttcgccgagatca gaggtccctaaggggaagaggcaacactc	-	-
CY105618	Human H3N2 IAVs	Human	H3N2		2007	Viet_Nam	A/HaNoi/N210/2007	agtgatgccccattccttgatcgggttcgccgagatca gaggtccctaaggggaagaggcaacactc	-	-
CY025839	Human H3N2 IAVs	Human	H3N2		2007	USA	A/New_York/UR06_0373/2007	agtgatgccccattccttgatcgggttcgccgagatca gaggtccctaaggggaagaggcaacactc	-	-
CY104320	Human H3N2 IAVs	Human	H3N2		2007	Viet_Nam	A/HaNoi/BM061/2007	agtgatgccccattccttgatcgggttcgccgagatca gaggtccctaaggggaagaggcaacactc	-	-
CY105634	Human H3N2 IAVs	Human	H3N2		2007	Viet_Nam	A/HaNoi/N71/2007	agtgatgccccattccttgatcgggttcgccgagatca gaggtccctaaggggaagaggcaacactc	-	-
CY104456	Human H3N2 IAVs	Human	H3N2		2007	Viet_Nam	A/HaNoi/Q163/2007	agtgatgccccattccttgatcgggttcgccgagatca gaggtccctaaggggaagaggcaacactc	-	-

CY104384	Human H3N2 IAVs	Human	H3N2		2007	Viet_Nam	A/LangSon/LS156/2007	agtgatgccccattccttgatcgggttcgccgagatca<br>gaggtccctaaggggaagaggcaacactc	-	-
CY104408	Human H3N2 IAVs	Human	H3N2		2007	Viet_Nam	A/HaNoi/N064/2007	agtgatgccccattccttgatcgggttcgccgagatca<br>gaggtccctaaggggaagaggcaacactc	-	-
CY105802	Human H3N2 IAVs	Human	H3N2		2007	Viet_Nam	A/HaNoi/TX067/2007	agtgatgccccattccttgatcgggttcgccgagatca<br>gaggtccctaaggggaagaggcaacactc	-	-
CY104520	Human H3N2 IAVs	Human	H3N2		2007	Viet_Nam	A/HaNoi/TX058/2007	agtgatgccccattccttgatcgggttcgccgagatca<br>gaggtccctaaggggaagaggcaacactc	-	-
CY104400	Human H3N2 IAVs	Human	H3N2		2007	Viet_Nam	A/HaNoi/N027/2007	agtgatgccccattccttgatcgggttcgccgagatca<br>gaggtccctaaggggaagaggcaacactc	-	-
CY105594	Human H3N2 IAVs	Human	H3N2		2007	Viet_Nam	A/HaNoi/N062/2007	agtgatgccccattccttgatcgggttcgccgagatca<br>gaggtccctaaggggaagaggcaacactc	-	-
CY105642	Human H3N2 IAVs	Human	H3N2		2007	Viet_Nam	A/HaNoi/Q031/2007	agtgatgccccattccttgatcgggttcgccgagatca<br>gaggtccctaaggggaagaggcaacactc	-	-
CY105650	Human H3N2 IAVs	Human	H3N2		2007	Viet_Nam	A/HaNoi/Q071/2007	agtgatgccccattccttgatcgggttcgccgagatca<br>gaggtccctaaggggaagaggcaacactc	-	-
FJ912966	Human H3N2 IAVs	Human	H3N2		2007	Thailand	A/Thailand/CU280/2007	agtgatgccccattccttgatcgggttcgccgagatca<br>gaggtccctaaggggaagaggcaacactc	-	-
CY105658	Human H3N2 IAVs	Human	H3N2		2007	Viet_Nam	A/HaNoi/Q094/2007	agtgatgccccattccttgatcgggttcgccgagatca<br>gaggtccctaaggggaagaggcaacactc	-	-
FJ912972	Human H3N2 IAVs	Human	H3N2		2007	Thailand	A/Thailand/CU282/2007	agtgatgccccattccttgatcgggttcgccgagatca<br>gaggtccctaaggggaagaggcaacactc	-	-
CY105666	Human H3N2 IAVs	Human	H3N2		2007	Viet_Nam	A/HaNoi/Q099/2007	agtgatgccccattccttgatcgggttcgccgagatca<br>gaggtccctaaggggaagaggcaacactc	-	-
CY105690	Human H3N2 IAVs	Human	H3N2		2007	Viet_Nam	A/HaNoi/Q220/2007	agtgatgccccattccttgatcgggttcgccgagatca<br>gaggtccctaaggggaagaggcaacactc	-	-
CY105562	Human H3N2 IAVs	Human	H3N2		2007	Viet_Nam	A/HungYen/HY6/2007	agtgatgccccattccttgatcgggttcgccgagatca<br>gaggtccctaaggggaagaggcaacactc	-	-
CY104376	Human H3N2 IAVs	Human	H3N2		2007	Viet_Nam	A/HungYen/HY3/2007	agtgatgccccattccttgatcgggttcgccgagatca<br>gaggtccctaaggggaagaggcaacactc	-	-
CY105674	Human H3N2 IAVs	Human	H3N2		2007	Viet_Nam	A/HaNoi/Q148/2007	agtgatgccccattccttgatcgggttcgccgagatca<br>gaggtccctaaggggaagaggcaacactc	-	-
AB442006	Human H3N2 IAVs	Human	H3N2		2007	Japan	A/Sendai_H/F150/2007	agtgatgccccattccttgatcgggttcgccgagatca<br>gaggtccctaaggggaagaggcaacactc	-	-
CY105778	Human H3N2 IAVs	Human	H3N2		2007	Viet_Nam	A/HaNoi/TX019/2007	agtgatgccccattccttgatcgggttcgccgagatca<br>gaggtccctaaggggaagaggcaacactc	-	-
CY105586	Human H3N2 IAVs	Human	H3N2		2007	Viet_Nam	A/HaNoi/N035/2007	agtgatgccccattccttgatcgggttcgccgagatca<br>gaggtccctaaggggaagaggcaacactc	-	-
CY025911	Human H3N2 IAVs	Human	H3N2		2007	USA	A/Kentucky/UR06_0370/2007	agtgatgccccattccttgatcgggttcgccgagatca<br>gaggtccctaaggggaagaggcaacactc	-	-
CY104552	Human H3N2 IAVs	Human	H3N2		2007	Viet_Nam	A/BacNinh/N52/2007	agtgatgccccattccttgatcgggttcgccgagatca<br>gaggtccctaaggggaagaggcaacactc	-	-
KF836400	Human H3N2 IAVs	Human	H3N2		2007	Singapore	A/Singapore/139N/2007	agtgatgccccattccttgatcgggttcgccgagatca<br>gaggtccctaaggggaagaggcaacactc	-	-
CY118079	Human H3N2 IAVs	Human	H3N2		2007	Malaysia	A/Malaysia/1779027/2007	agtgatgcccccttccttgatcggcttcgccgagatcag<br>aggtccctaaggggaagaggcaatactc	-	-
CY172755	Human H3N2 IAVs	Human	H3N2		2007	USA	A/New_York/1079/2007	agtgatgcccccttccttgatcggcttcgccgagatcag<br>aggtccctaaggggaagaggcaatactc	-	-
JN105977	Human H3N2 IAVs	Swine	H3N2		2007	China	A/swine/Fujian/F2/2007	ggtgatgccccattccttgatcggcttcgccgagatcag<br>aagtccctaaggggaagaggcagcactc	+	+

CY104626	Human H3N2 IAVs	Human	H3N2		2008	Viet_Nam	A/DaNang/DN446/2008	aatgatgccccattccttgatcggcttcgccgagatcagaggtccctaaggggaagaggcaatactc	-	-
HQ853541	Human H3N2 IAVs	Human	H3N2		2008	India	A/KOL/1810/2008	agcgatgccccattccttgatcggcttcgccgagatcagaggtccctaaggggaagaggcaatactc	-	-
CY038819	Human H3N2 IAVs	Human	H3N2		2008	USA	A/California/UR07_0067/2008	agcgatgccccattccttgatcggcttcgccgagatcagaggtccctaaggggaagaggcaatactc	-	-
CY147631	Human H3N2 IAVs	Human	H3N2		2008	Mexico	A/Mexico/24009/2008	agcgatgccccattccttgatcggcttcgccgagatcagaggtccctaaggggaagaggcaatactc	-	-
CY037571	Human H3N2 IAVs	Human	H3N2		2008	USA	A/Kentucky/UR07_0081/2008	agtgatgccccattccttgatcggcttcgacgagatcagaggtccctaaggggaagaggcaatactc	+	+
CY044712	Human H3N2 IAVs	Human	H3N2		2008	USA	A/Boston/57/2008	agtgatgccccattccttgatcggcttcgccgagatcaaaggtccctaaggggaagaggcaatactc	-	-
CY104634	Human H3N2 IAVs	Human	H3N2		2008	Viet_Nam	A/KhanhHoa/KH466/2008	agtgatgccccattccttgatcggcttcgccgagatcaaaggtccctaaggggaagaggcaatactc	-	-
CY037315	Human H3N2 IAVs	Human	H3N2		2008	Kuwait	A/Kuwait/AF03/2008	agtgatgccccattccttgatcggcttcgccgagatcagaggtccctaagaggaagaggcaatactc	+	+
JX138513	Human H3N2 IAVs	Swine	H1N2		2008	China	A/swine/Zhejiang/01/2008	agtgatgccccattccttgatcggcttcgccgagatcagaggtccctaaggggaagaggcaacactc	-	-
GU646034	Human H3N2 IAVs	Swine	H1N1		2008	China	A/swine/Shandong/1123/2008	agtgatgccccattccttgatcggcttcgccgagatcagaggtccctaaggggaagaggcaacactc	-	-
CY037715	Human H3N2 IAVs	Human	H3N2		2008	USA	A/New_York/UR07_0093/2008	agtgatgccccattccttgatcggcttcgccgagatcagaggtccctaaggggaagaggcaacactc	-	-
CY118135	Human H3N2 IAVs	Human	H3N2		2008	Malaysia	A/Malaysia/1959476/2008	agtgatgccccattccttgatcggcttcgccgagatcagaggtccctaaggggaagaggcaacactc	-	-
CY119038	Human H3N2 IAVs	Human	H3N2		2008	Malaysia	A/Malaysia/1963888/2008	agtgatgccccattccttgatcggcttcgccgagatcagaggtccctaaggggaagaggcaacactc	-	-
CY119046	Human H3N2 IAVs	Human	H3N2		2008	Malaysia	A/Malaysia/1965896/2008	agtgatgccccattccttgatcggcttcgccgagatcagaggtccctaaggggaagaggcaacactc	-	-
CY092328	Human H3N2 IAVs	Swine	H3N2		2008	Argentina	A/swine/Argentina/CIP051_A2/2008	agtgatgccccattccttgatcggcttcgccgagatcagaggtccctaaggggaagaggcaacactc	-	-
FJ913002	Human H3N2 IAVs	Human	H3N2		2008	Thailand	A/Thailand/CU_1101/2008	agtgatgccccattccttgatcggcttcgccgagatcagaggtccctaaggggaagaggcaatactc	-	-
HQ853539	Human H3N2 IAVs	Human	H3N2		2008	India	A/KOL/1762/2008	agtgatgccccattccttgatcggcttcgccgagatcagaggtccctaaggggaagaggcaatactc	-	-
CY044433	Human H3N2 IAVs	Human	H3N2		2008	USA	A/Boston/1/2008	agtgatgccccattccttgatcggcttcgccgagatcagaggtccctaaggggaagaggcaatactc	-	-
CY044504	Human H3N2 IAVs	Human	H3N2		2008	USA	A/Boston/19/2008	agtgatgccccattccttgatcggcttcgccgagatcagaggtccctaaggggaagaggcaatactc	-	-
CY044441	Human H3N2 IAVs	Human	H3N2		2008	USA	A/Boston/2/2008	agtgatgccccattccttgatcggcttcgccgagatcagaggtccctaaggggaagaggcaatactc	-	-
CY044512	Human H3N2 IAVs	Human	H3N2		2008	USA	A/Boston/20/2008	agtgatgccccattccttgatcggcttcgccgagatcagaggtccctaaggggaagaggcaatactc	-	-
CY044528	Human H3N2 IAVs	Human	H3N2		2008	USA	A/Boston/23/2008	agtgatgccccattccttgatcggcttcgccgagatcagaggtccctaaggggaagaggcaatactc	-	-
CY044544	Human H3N2 IAVs	Human	H3N2		2008	USA	A/Boston/29/2008	agtgatgccccattccttgatcggcttcgccgagatcagaggtccctaaggggaagaggcaatactc	-	-
CY044552	Human H3N2 IAVs	Human	H3N2		2008	USA	A/Boston/31/2008	agtgatgccccattccttgatcggcttcgccgagatcagaggtccctaaggggaagaggcaatactc	-	-
CY044592	Human H3N2 IAVs	Human	H3N2		2008	USA	A/Boston/39/2008	agtgatgccccattccttgatcggcttcgccgagatcagaggtccctaaggggaagaggcaatactc	-	-

CY044600	Human H3N2 IAVs	Human	H3N2		2008	USA	A/Boston/40/2008	agtgatgccccattccttgatcggcttcgccgagatcag aggtccctaaggggaagaggcaatactc	-	-
CY044608	Human H3N2 IAVs	Human	H3N2		2008	USA	A/Boston/42/2008	agtgatgccccattccttgatcggcttcgccgagatcag aggtccctaaggggaagaggcaatactc	-	-
CY044648	Human H3N2 IAVs	Human	H3N2		2008	USA	A/Boston/47/2008	agtgatgccccattccttgatcggcttcgccgagatcag aggtccctaaggggaagaggcaatactc	-	-
CY044672	Human H3N2 IAVs	Human	H3N2		2008	USA	A/Boston/50/2008	agtgatgccccattccttgatcggcttcgccgagatcag aggtccctaaggggaagaggcaatactc	-	-
CY044680	Human H3N2 IAVs	Human	H3N2		2008	USA	A/Boston/51/2008	agtgatgccccattccttgatcggcttcgccgagatcag aggtccctaaggggaagaggcaatactc	-	-
CY044696	Human H3N2 IAVs	Human	H3N2		2008	USA	A/Boston/55/2008	agtgatgccccattccttgatcggcttcgccgagatcag aggtccctaaggggaagaggcaatactc	-	-
CY044704	Human H3N2 IAVs	Human	H3N2		2008	USA	A/Boston/56/2008	agtgatgccccattccttgatcggcttcgccgagatcag aggtccctaaggggaagaggcaatactc	-	-
CY044744	Human H3N2 IAVs	Human	H3N2		2008	USA	A/Boston/61/2008	agtgatgccccattccttgatcggcttcgccgagatcag aggtccctaaggggaagaggcaatactc	-	-
CY044768	Human H3N2 IAVs	Human	H3N2		2008	USA	A/Boston/64/2008	agtgatgccccattccttgatcggcttcgccgagatcag aggtccctaaggggaagaggcaatactc	-	-
CY044792	Human H3N2 IAVs	Human	H3N2		2008	USA	A/Boston/71/2008	agtgatgccccattccttgatcggcttcgccgagatcag aggtccctaaggggaagaggcaatactc	-	-
CY044800	Human H3N2 IAVs	Human	H3N2		2008	USA	A/Boston/78/2008	agtgatgccccattccttgatcggcttcgccgagatcag aggtccctaaggggaagaggcaatactc	-	-
CY044848	Human H3N2 IAVs	Human	H3N2		2008	USA	A/Boston/96/2008	agtgatgccccattccttgatcggcttcgccgagatcag aggtccctaaggggaagaggcaatactc	-	-
CY038795	Human H3N2 IAVs	Human	H3N2		2008	USA	A/California/UR07_0017/2008	agtgatgccccattccttgatcggcttcgccgagatcag aggtccctaaggggaagaggcaatactc	-	-
CY037507	Human H3N2 IAVs	Human	H3N2		2008	USA	A/California/UR07_0019/2008	agtgatgccccattccttgatcggcttcgccgagatcag aggtccctaaggggaagaggcaatactc	-	-
CY038803	Human H3N2 IAVs	Human	H3N2		2008	USA	A/Florida/UR07_0021/2008	agtgatgccccattccttgatcggcttcgccgagatcag aggtccctaaggggaagaggcaatactc	-	-
CY037731	Human H3N2 IAVs	Human	H3N2		2008	USA	A/Florida/UR07_0102/2008	agtgatgccccattccttgatcggcttcgccgagatcag aggtccctaaggggaagaggcaatactc	-	-
CY038875	Human H3N2 IAVs	Human	H3N2		2008	USA	A/Florida/UR07_0150/2008	agtgatgccccattccttgatcggcttcgccgagatcag aggtccctaaggggaagaggcaatactc	-	-
CY069361	Human H3N2 IAVs	Human	H3N2		2008	Japan	A/Japan/WRAIR1059P/2008	agtgatgccccattccttgatcggcttcgccgagatcag aggtccctaaggggaagaggcaatactc	-	-
CY037491	Human H3N2 IAVs	Human	H3N2		2008	USA	A/Kansas/UR07_0007/2008	agtgatgccccattccttgatcggcttcgccgagatcag aggtccctaaggggaagaggcaatactc	-	-
CY037811	Human H3N2 IAVs	Human	H3N2		2008	USA	A/Kansas/UR07_0047/2008	agtgatgccccattccttgatcggcttcgccgagatcag aggtccctaaggggaagaggcaatactc	-	-
CY038851	Human H3N2 IAVs	Human	H3N2		2008	USA	A/Kansas/UR07_0096/2008	agtgatgccccattccttgatcggcttcgccgagatcag aggtccctaaggggaagaggcaatactc	-	-
CY036995	Human H3N2 IAVs	Human	H3N2		2008	USA	A/Kansas/UR07_0135/2008	agtgatgccccattccttgatcggcttcgccgagatcag aggtccctaaggggaagaggcaatactc	-	-
CY037851	Human H3N2 IAVs	Human	H3N2		2008	USA	A/Kansas/UR07_0136/2008	agtgatgccccattccttgatcggcttcgccgagatcag aggtccctaaggggaagaggcaatactc	-	-
CY037619	Human H3N2 IAVs	Human	H3N2		2008	USA	A/Kansas/UR07_0137/2008	agtgatgccccattccttgatcggcttcgccgagatcag aggtccctaaggggaagaggcaatactc	-	-
CY037675	Human H3N2 IAVs	Human	H3N2		2008	USA	A/Kentucky/UR07_0015/2008	agtgatgccccattccttgatcggcttcgccgagatcag aggtccctaaggggaagaggcaatactc	-	-

CY037819	Human H3N2 IAVs	Human	H3N2		2008	USA	A/Kentucky/UR07_0049/2008	agtgatgccccattccttgatcggcttcgccgagatcag<br>aggtccctaaggggaagaggcaatactc	-	-
CY038827	Human H3N2 IAVs	Human	H3N2		2008	USA	A/Kentucky/UR07_0069/2008	agtgatgccccattccttgatcggcttcgccgagatcag<br>aggtccctaaggggaagaggcaatactc	-	-
CY037707	Human H3N2 IAVs	Human	H3N2		2008	USA	A/Kentucky/UR07_0072/2008	agtgatgccccattccttgatcggcttcgccgagatcag<br>aggtccctaaggggaagaggcaatactc	-	-
CY037587	Human H3N2 IAVs	Human	H3N2		2008	USA	A/Kentucky/UR07_0107/2008	agtgatgccccattccttgatcggcttcgccgagatcag<br>aggtccctaaggggaagaggcaatactc	-	-
CY037739	Human H3N2 IAVs	Human	H3N2		2008	USA	A/Kentucky/UR07_0109/2008	agtgatgccccattccttgatcggcttcgccgagatcag<br>aggtccctaaggggaagaggcaatactc	-	-
CY044640	Human H3N2 IAVs	Human	H3N2		2008	USA	A/Boston/46/2008	agtgatgccccattccttgatcggcttcgccgagatcag<br>aggtccctaaggggaagaggcaatactc	-	-
CY044728	Human H3N2 IAVs	Human	H3N2		2008	USA	A/Boston/59/2008	agtgatgccccattccttgatcggcttcgccgagatcag<br>aggtccctaaggggaagaggcaatactc	-	-
CY037835	Human H3N2 IAVs	Human	H3N2		2008	USA	A/Kentucky/UR07_0124/2008	agtgatgccccattccttgatcggcttcgccgagatcag<br>aggtccctaaggggaagaggcaatactc	-	-
CY038867	Human H3N2 IAVs	Human	H3N2		2008	USA	A/Kentucky/UR07_0149/2008	agtgatgccccattccttgatcggcttcgccgagatcag<br>aggtccctaaggggaagaggcaatactc	-	-
CY037643	Human H3N2 IAVs	Human	H3N2		2008	USA	A/Kentucky/UR07_0162/2008	agtgatgccccattccttgatcggcttcgccgagatcag<br>aggtccctaaggggaagaggcaatactc	-	-
CY093323	Human H3N2 IAVs	Human	H3N2		2008	Kyrgyzstan	A/Kyrgyzstan/WRAIR1256P/2008	agtgatgccccattccttgatcggcttcgccgagatcag<br>aggtccctaaggggaagaggcaatactc	-	-
CY073903	Human H3N2 IAVs	Human	H3N2		2008	Mexico	A/Mexico/UASLP_010/2008	agtgatgccccattccttgatcggcttcgccgagatcag<br>aggtccctaaggggaagaggcaatactc	-	-
CY036955	Human H3N2 IAVs	Human	H3N2		2008	USA	A/Mississippi/UR07_0004/2008	agtgatgccccattccttgatcggcttcgccgagatcag<br>aggtccctaaggggaagaggcaatactc	-	-
CY037515	Human H3N2 IAVs	Human	H3N2		2008	USA	A/Mississippi/UR07_0025/2008	agtgatgccccattccttgatcggcttcgccgagatcag<br>aggtccctaaggggaagaggcaatactc	-	-
CY037611	Human H3N2 IAVs	Human	H3N2		2008	USA	A/New_York/UR07_0111/2008	agtgatgccccattccttgatcggcttcgccgagatcag<br>aggtccctaaggggaagaggcaatactc	-	-
CY044385	Human H3N2 IAVs	Human	H3N2		2008	USA	A/New_York/UR07_0160/2008	agtgatgccccattccttgatcggcttcgccgagatcag<br>aggtccctaaggggaagaggcaatactc	-	-
CY037859	Human H3N2 IAVs	Human	H3N2		2008	USA	A/Ohio/UR07_0023/2008	agtgatgccccattccttgatcggcttcgccgagatcag<br>aggtccctaaggggaagaggcaatactc	-	-
CY037803	Human H3N2 IAVs	Human	H3N2		2008	USA	A/Ohio/UR07_0035/2008	agtgatgccccattccttgatcggcttcgccgagatcag<br>aggtccctaaggggaagaggcaatactc	-	-
CY044624	Human H3N2 IAVs	Human	H3N2		2008	USA	A/Boston/44/2008	agtgatgccccattccttgatcggcttcgccgagatcag<br>aggtccctaaggggaagaggcaatactc	-	-
CY044752	Human H3N2 IAVs	Human	H3N2		2008	USA	A/Boston/62/2008	agtgatgccccattccttgatcggcttcgccgagatcag<br>aggtccctaaggggaagaggcaatactc	-	-
CY044776	Human H3N2 IAVs	Human	H3N2		2008	USA	A/Boston/66/2008	agtgatgccccattccttgatcggcttcgccgagatcag<br>aggtccctaaggggaagaggcaatactc	-	-
CY037883	Human H3N2 IAVs	Human	H3N2		2008	USA	A/Kentucky/UR07_0083/2008	agtgatgccccattccttgatcggcttcgccgagatcag<br>aggtccctaaggggaagaggcaatactc	-	-
CY037323	Human H3N2 IAVs	Human	H3N2		2008	South_Korea	A/Korea/AF05/2008	agtgatgccccattccttgatcggcttcgccgagatcag<br>aggtccctaaggggaagaggcaatactc	-	-
CY037747	Human H3N2 IAVs	Human	H3N2		2008	USA	A/New_York/UR07_0133/2008	agtgatgccccattccttgatcggcttcgccgagatcag<br>aggtccctaaggggaagaggcaatactc	-	-
CY037635	Human H3N2 IAVs	Human	H3N2		2008	USA	A/New_York/UR07_0153/2008	agtgatgccccattccttgatcggcttcgccgagatcag<br>aggtccctaaggggaagaggcaatactc	-	-

CY039099	Human H3N2 IAVs	Human	H3N2		2008	USA	A/Ohio/UR07_0034/2008	agtgatgccccattccttgatcggcttcgccgagatcag aggtccctaaggggaagaggcaatactc	-	-
CY036963	Human H3N2 IAVs	Human	H3N2		2008	USA	A/Ohio/UR07_0043/2008	agtgatgccccattccttgatcggcttcgccgagatcag aggtccctaaggggaagaggcaatactc	-	-
CY038835	Human H3N2 IAVs	Human	H3N2		2008	USA	A/Ohio/UR07_0073/2008	agtgatgccccattccttgatcggcttcgccgagatcag aggtccctaaggggaagaggcaatactc	-	-
CY036979	Human H3N2 IAVs	Human	H3N2		2008	USA	A/Ohio/UR07_0089/2008	agtgatgccccattccttgatcggcttcgccgagatcag aggtccctaaggggaagaggcaatactc	-	-
CY037003	Human H3N2 IAVs	Human	H3N2		2008	USA	A/Ohio/UR07_0140/2008	agtgatgccccattccttgatcggcttcgccgagatcag aggtccctaaggggaagaggcaatactc	-	-
CY044361	Human H3N2 IAVs	Human	H3N2		2008	USA	A/Pennsylvania/PIT34/2008	agtgatgccccattccttgatcggcttcgccgagatcag aggtccctaaggggaagaggcaatactc	-	-
CY073923	Human H3N2 IAVs	Human	H3N2		2008	Mexico	A/Mexico/UASLP_013/2008	agtgatgccccattccttgatcggcttcgccgagatcag aggtccctaaggggaagaggcaatactc	-	-
CY044337	Human H3N2 IAVs	Human	H3N2		2008	USA	A/Texas/AF01/2007	agtgatgccccattccttgatcggcttcgccgagatcag aggtccctaaggggaagaggcaatactc	-	-
CY044465	Human H3N2 IAVs	Human	H3N2		2008	USA	A/Boston/5/2008	agtgatgccccattccttgatcggcttcgccgagatcag aggtccctaaggggaagaggcaatactc	-	-
CY038499	Human H3N2 IAVs	Human	H3N2		2008	USA	A/Pennsylvania/PIT39/2008	agtgatgccccattccttgatcggcttcgccgagatcag aggtccctaaggggaagaggcaatactc	-	-
CY037563	Human H3N2 IAVs	Human	H3N2		2008	USA	A/Kentucky/UR07_0068/2008	agtgatgccccattccttgatcggcttcgccgagatcag aggtccctaaggggaagaggcaatactc	-	-
CY037651	Human H3N2 IAVs	Human	H3N2		2008	USA	A/Kentucky/UR07_0164/2008	agtgatgccccattccttgatcggcttcgccgagatcag aggtccctaaggggaagaggcaatactc	-	-
CY041462	Human H3N2 IAVs	Human	H3N2		2008	USA	A/Ohio/UR07_0013/2008	agtgatgccccattccttgatcggcttcgccgagatcag aggtccctaaggggaagaggcaatactc	-	-
CY105858	Human H3N2 IAVs	Human	H3N2		2008	Viet_Nam	A/KhanhHoa/KH363/2008	agtgatgccccattccttgatcggcttcgccgagatcag aggtccctaaggggaagaggcaatactc	-	-
CY147583	Human H3N2 IAVs	Human	H3N2		2008	Mexico	A/Mexico/24003/2008	agtgatgccccattccttgatcggcttcgccgagatcag aggtccctaaggggaagaggcaatactc	-	-
CY147663	Human H3N2 IAVs	Human	H3N2		2008	Mexico	A/Mexico/24013/2008	agtgatgccccattccttgatcggcttcgccgagatcag aggtccctaaggggaagaggcaatactc	-	-
CY039107	Human H3N2 IAVs	Human	H3N2		2008	USA	A/Mississippi/UR07_0042/2008	agtgatgccccattccttgatcggcttcgccgagatcag aggtccctaaggggaagaggcaatactc	-	-
CY173131	Human H3N2 IAVs	Human	H3N2		2008	USA	A/New_York/1134/2008	agtgatgccccattccttgatcggcttcgccgagatcag aggtccctaaggggaagaggcaatactc	-	-
CY173147	Human H3N2 IAVs	Human	H3N2		2008	USA	A/New_York/1136/2008	agtgatgccccattccttgatcggcttcgccgagatcag aggtccctaaggggaagaggcaatactc	-	-
CY173187	Human H3N2 IAVs	Human	H3N2		2008	USA	A/New_York/1142/2008	agtgatgccccattccttgatcggcttcgccgagatcag aggtccctaaggggaagaggcaatactc	-	-
CY173195	Human H3N2 IAVs	Human	H3N2		2008	USA	A/New_York/1143/2008	agtgatgccccattccttgatcggcttcgccgagatcag aggtccctaaggggaagaggcaatactc	-	-
CY173203	Human H3N2 IAVs	Human	H3N2		2008	USA	A/New_York/1144/2008	agtgatgccccattccttgatcggcttcgccgagatcag aggtccctaaggggaagaggcaatactc	-	-
CY173211	Human H3N2 IAVs	Human	H3N2		2008	USA	A/New_York/1145/2008	agtgatgccccattccttgatcggcttcgccgagatcag aggtccctaaggggaagaggcaatactc	-	-
CY173219	Human H3N2 IAVs	Human	H3N2		2008	USA	A/New_York/1146/2008	agtgatgccccattccttgatcggcttcgccgagatcag aggtccctaaggggaagaggcaatactc	-	-
CY173243	Human H3N2 IAVs	Human	H3N2		2008	USA	A/New_York/1150/2008	agtgatgccccattccttgatcggcttcgccgagatcag aggtccctaaggggaagaggcaatactc	-	-

CY173251	Human H3N2 IAVs	Human	H3N2		2008	USA	A/New_York/1151/2008	agtgatgccccattccttgatcggcttcgccgagatcag aggtccctaaggggaagaggcaatactc	-	-
CY173259	Human H3N2 IAVs	Human	H3N2		2008	USA	A/New_York/1152/2008	agtgatgccccattccttgatcggcttcgccgagatcag aggtccctaaggggaagaggcaatactc	-	-
CY173275	Human H3N2 IAVs	Human	H3N2		2008	USA	A/New_York/1154/2008	agtgatgccccattccttgatcggcttcgccgagatcag aggtccctaaggggaagaggcaatactc	-	-
FJ913008	Human H3N2 IAVs	Human	H3N2		2008	Thailand	A/Thailand/CU_1102/2008	agtgatgccccattccttgatcggcttcgccgagatcag aggtccctaaggggaagaggcaatactc	-	-
CY073951	Human H3N2 IAVs	Human	H3N2		2008	Mexico	A/Mexico/UASLP_014/2008	agtgatgccccattccttgatcggcttcgccgagatcag aggtccctaaggggaagaggcaatactc	-	-
FJ912996	Human H3N2 IAVs	Human	H3N2		2008	Thailand	A/Thailand/CU379/2008	agtgatgccccattccttgatcggcttcgccgagatcag aggtccctaaggggaagaggcaatactc	-	-
CY044632	Human H3N2 IAVs	Human	H3N2		2008	USA	A/Boston/45/2008	agtgatgccccattccttgatcggcttcgccgagatcag aggtccctaaggggaagaggcaatactc	-	-
CY044656	Human H3N2 IAVs	Human	H3N2		2008	USA	A/Boston/48/2008	agtgatgccccattccttgatcggcttcgccgagatcag aggtccctaaggggaagaggcaatactc	-	-
CY044720	Human H3N2 IAVs	Human	H3N2		2008	USA	A/Boston/58/2008	agtgatgccccattccttgatcggcttcgccgagatcag aggtccctaaggggaagaggcaatactc	-	-
CY044784	Human H3N2 IAVs	Human	H3N2		2008	USA	A/Boston/68/2008	agtgatgccccattccttgatcggcttcgccgagatcag aggtccctaaggggaagaggcaatactc	-	-
CY037547	Human H3N2 IAVs	Human	H3N2		2008	USA	A/California/UR07_0053/2008	agtgatgccccattccttgatcggcttcgccgagatcag aggtccctaaggggaagaggcaatactc	-	-
CY037627	Human H3N2 IAVs	Human	H3N2		2008	USA	A/Florida/UR07_0146/2008	agtgatgccccattccttgatcggcttcgccgagatcag aggtccctaaggggaagaggcaatactc	-	-
CY037603	Human H3N2 IAVs	Human	H3N2		2008	USA	A/Kansas/UR07_0110/2008	agtgatgccccattccttgatcggcttcgccgagatcag aggtccctaaggggaagaggcaatactc	-	-
CY037843	Human H3N2 IAVs	Human	H3N2		2008	USA	A/Kansas/UR07_0129/2008	agtgatgccccattccttgatcggcttcgccgagatcag aggtccctaaggggaagaggcaatactc	-	-
CY039435	Human H3N2 IAVs	Human	H3N2		2008	USA	A/Kentucky/UR07_0116/2008	agtgatgccccattccttgatcggcttcgccgagatcag aggtccctaaggggaagaggcaatactc	-	-
CY044816	Human H3N2 IAVs	Human	H3N2		2008	USA	A/Boston/81/2008	agtgatgccccattccttgatcggcttcgccgagatcag aggtccctaaggggaagaggcaatactc	-	-
CY037691	Human H3N2 IAVs	Human	H3N2		2008	USA	A/Kentucky/UR07_0060/2008	agtgatgccccattccttgatcggcttcgccgagatcag aggtccctaaggggaagaggcaatactc	-	-
CY044856	Human H3N2 IAVs	Human	H3N2		2008	USA	A/Boston/97/2008	agtgatgccccattccttgatcggcttcgccgagatcag aggtccctaaggggaagaggcaatactc	-	-
CY039115	Human H3N2 IAVs	Human	H3N2		2008	USA	A/Ohio/UR07_0045/2008	agtgatgccccattccttgatcggcttcgccgagatcag aggtccctaaggggaagaggcaatactc	-	-
CY087315	Human H3N2 IAVs	Human	H3N2		2008	Uganda	A/Uganda/MUWRP_001/2008	agtgatgccccattccttgatcggcttcgccgagatcag aggtccctaaggggaagaggcaatactc	-	-
CY087488	Human H3N2 IAVs	Human	H3N2		2008	Uganda	A/Uganda/MUWRP_025/2008	agtgatgccccattccttgatcggcttcgccgagatcag aggtccctaaggggaagaggcaatactc	-	-
CY087563	Human H3N2 IAVs	Human	H3N2		2008	Uganda	A/Uganda/MUWRP_036/2008	agtgatgccccattccttgatcggcttcgccgagatcag aggtccctaaggggaagaggcaatactc	-	-
CY087686	Human H3N2 IAVs	Human	H3N2		2008	Uganda	A/Uganda/MUWRP_052/2008	agtgatgccccattccttgatcggcttcgccgagatcag aggtccctaaggggaagaggcaatactc	-	-
CY087416	Human H3N2 IAVs	Human	H3N2		2008	Uganda	A/Uganda/MUWRP_015/2008	agtgatgccccattccttgatcggcttcgccgagatcag aggtccctaaggggaagaggcaatactc	-	-
CY087527	Human H3N2 IAVs	Human	H3N2		2008	Uganda	A/Uganda/MUWRP_031/2008	agtgatgccccattccttgatcggcttcgccgagatcag aggtccctaaggggaagaggcaatactc	-	-

CY087543	Human H3N2 IAVs	Human	H3N2		2008	Uganda	A/Uganda/MUWRP_033/2008	agtgatgccccattccttgatcggcttcgccgagatcag aggtccctaaggggaagaggcaatactc	-	-
CY087586	Human H3N2 IAVs	Human	H3N2		2008	Uganda	A/Uganda/MUWRP_039/2008	agtgatgccccattccttgatcggcttcgccgagatcag aggtccctaaggggaagaggcaatactc	-	-
CY041478	Human H3N2 IAVs	Human	H3N2		2008	USA	A/Florida/UR07_0154/2008	agtgatgccccattccttgatcggcttcgccgagatcag aggtccctaaggggaagaggcaatactc	-	-
FJ009465	Human H3N2 IAVs	Human	H3N2		2008	South_Korea	A/Cheongju/H471/2008	agtgatgccccattccttgatcggcttcgccgagatcag aggtccctaaggggaagaggcaatactc	-	-
CY037499	Human H3N2 IAVs	Human	H3N2		2008	USA	A/Ohio/UR07_0016/2008	agtgatgccccattccttgatcggcttcgccgagatcag aggtccctaaggggaagaggcaatactc	-	-
CY037891	Human H3N2 IAVs	Human	H3N2		2008	USA	A/Ohio/UR07_0126/2008	agtgatgccccattccttgatcggcttcgccgagatcag aggtccctaaggggaagaggcaatactc	-	-
CY044616	Human H3N2 IAVs	Human	H3N2		2008	USA	A/Boston/43/2008	agtgatgccccattccttgatcggcttcgccgagatcag aggtccctaaggggaagaggcaatactc	-	-
CY037579	Human H3N2 IAVs	Human	H3N2		2008	USA	A/Ohio/UR07_0105/2008	agtgatgccccattccttgatcggcttcgccgagatcag aggtccctaaggggaagaggcaatactc	-	-
FJ009461	Human H3N2 IAVs	Human	H3N2		2008	South_Korea	A/Cheongju/H397/2008	agtgatgccccattccttgatcggcttcgccgagatcag aggtccctaaggggaagaggcaatactc	-	-
FJ009462	Human H3N2 IAVs	Human	H3N2		2008	South_Korea	A/Cheongju/H398/2008	agtgatgccccattccttgatcggcttcgccgagatcag aggtccctaaggggaagaggcaatactc	-	-
FJ009463	Human H3N2 IAVs	Human	H3N2		2008	South_Korea	A/Cheongju/H407/2008	agtgatgccccattccttgatcggcttcgccgagatcag aggtccctaaggggaagaggcaatactc	-	-
FJ009464	Human H3N2 IAVs	Human	H3N2		2008	South_Korea	A/Cheongju/H412/2008	agtgatgccccattccttgatcggcttcgccgagatcag aggtccctaaggggaagaggcaatactc	-	-
CY037555	Human H3N2 IAVs	Human	H3N2		2008	USA	A/Ohio/UR07_0058/2008	agtgatgccccattccttgatcggcttcgccgagatcag aggtccctaaggggaagaggcaatactc	-	-
CY037867	Human H3N2 IAVs	Human	H3N2		2008	USA	A/California/UR07_0063/2008	agtgatgccccattccttgatcggcttcgccgagatcag aggtccctaaggggaagaggcaatactc	-	-
CY173155	Human H3N2 IAVs	Human	H3N2		2008	USA	A/New_York/1138/2008	agtgatgccccattccttgatcggcttcgccgagatcag aggtccctaaggggaagaggcaatactc	-	-
CY104618	Human H3N2 IAVs	Human	H3N2		2008	Viet_Nam	A/DaNang/DN434/2008	agtgatgccccattccttgatcggcttcgccgagatcag aggtccctaaggggaagaggcaatactc	-	-
CY037539	Human H3N2 IAVs	Human	H3N2		2008	USA	A/Kentucky/UR07_0041/2008	agtgatgccccattccttgatcggcttcgccgagatcag aggtccctaaggggaagaggcaatactc	-	-
CY119022	Human H3N2 IAVs	Human	H3N2		2008	Malaysia	A/Malaysia/1922209/2008	agtgatgccccattccttgatcggcttcgccgagatcag aggtccctaaggggaagaggcaatactc	-	-
CY105826	Human H3N2 IAVs	Human	H3N2		2008	Viet_Nam	A/DaNang/DN453/2008	agtgatgccccattccttgatcggcttcgccgagatcag aggtccctaaggggaagaggcaatactc	-	-
CY119030	Human H3N2 IAVs	Human	H3N2		2008	Malaysia	A/Malaysia/1959864/2008	agtgatgccccattccttgatcggcttcgccgagatcag aggtccctaaggggaagaggcaatactc	-	-
CY069409	Human H3N2 IAVs	Human	H3N2		2008	Kyrgyzstan	A/Kyrgyzstan/WRAIR1121P/2008	agtgatgccccattccttgatcggcttcgccgagatcag aggtccctaaggggaagaggcaatactc	-	-
CY093315	Human H3N2 IAVs	Human	H3N2		2008	Kyrgyzstan	A/Kyrgyzstan/WRAIR1255P/2008	agtgatgccccattccttgatcggcttcgccgagatcag aggtccctaaggggaagaggcaatactc	-	-
CY036987	Human H3N2 IAVs	Human	H3N2		2008	USA	A/Florida/UR07_0101/2008	agtgatgccccattccttgatcggcttcgccgagatcag aggtccctaaggggaagaggcaatactc	-	-
CY118155	Human H3N2 IAVs	Human	H3N2		2008	Malaysia	A/Malaysia/1997829/2008	agtgatgccccattccttgatcggcttcgccgagatcag aggtccctaaggggaagaggcaatactc	-	-
CY118163	Human H3N2 IAVs	Human	H3N2		2008	Malaysia	A/Malaysia/1997831/2008	agtgatgccccattccttgatcggcttcgccgagatcag aggtccctaaggggaagaggcaatactc	-	-

CY044584	Human H3N2 IAVs	Human	H3N2		2008	USA	A/Boston/38/2008	agtgatgccccattccttgatcggcttcgccgagatcag aggtccctaaggggaagaggcaatactc	-	-
CY040102	Human H3N2 IAVs	Human	H3N2		2008	Taiwan	A/Taiwan/70120/2008	agtgatgccccattccttgatcggcttcgccgagatcag aggtccctaaggggaagaggcaatactc	-	-
CY044449	Human H3N2 IAVs	Human	H3N2		2008	USA	A/Boston/3/2008	agtgatgccccattccttgatcggcttcgccgagatcag aggtccctaaggggaagaggcaatactc	-	-
CY035178	Human H3N2 IAVs	Human	H3N2		2008	USA	A/Pennsylvania/PIT25/2008	agtgatgccccattccttgatcggcttcgccgagatcag aggtccctaaggggaagaggcaatactc	-	-
CY044457	Human H3N2 IAVs	Human	H3N2		2008	USA	A/Boston/4/2008	agtgatgccccattccttgatcggcttcgccgagatcag aggtccctaaggggaagaggcaatactc	-	-
CY038787	Human H3N2 IAVs	Human	H3N2		2008	USA	A/Mississippi/UR07_0003/2008	agtgatgccccattccttgatcggcttcgccgagatcag aggtccctaaggggaagaggcaatactc	-	-
CY104610	Human H3N2 IAVs	Human	Mixed		2008	Viet_Nam	A/KhanhHoa/KH37/2008	agtgatgccccattccttgatcggcttcgccgagatcag aggtccctaaggggaagaggcaatactc	-	-
CY037523	Human H3N2 IAVs	Human	H3N2		2008	USA	A/Ohio/UR07_0031/2008	agtgatgccccattccttgatcggcttcgccgagatcag aggtccctaaggggaagaggcaatactc	-	-
CY037531	Human H3N2 IAVs	Human	H3N2		2008	USA	A/Kentucky/UR07_0037/2008	agtgatgccccattccttgatcggcttcgccgagatcag aggtccctaaggggaagaggcaatactc	-	-
CY044520	Human H3N2 IAVs	Human	H3N2		2008	USA	A/Boston/21/2008	agtgatgccccattccttgatcggcttcgccgagatcag aggtccctaaggggaagaggcaatactc	-	-
CY044576	Human H3N2 IAVs	Human	H3N2		2008	USA	A/Boston/36/2008	agtgatgccccattccttgatcggcttcgccgagatcag aggtccctaaggggaagaggcaatactc	-	-
CY044760	Human H3N2 IAVs	Human	H3N2		2008	USA	A/Boston/63/2008	agtgatgccccattccttgatcggcttcgccgagatcag aggtccctaaggggaagaggcaatactc	-	-
CY044824	Human H3N2 IAVs	Human	H3N2		2008	USA	A/Boston/88/2008	agtgatgccccattccttgatcggcttcgccgagatcag aggtccctaaggggaagaggcaatactc	-	-
CY039427	Human H3N2 IAVs	Human	H3N2		2008	USA	A/Kentucky/UR07_0038/2008	agtgatgccccattccttgatcggcttcgccgagatcag aggtccctaaggggaagaggcaatactc	-	-
CY035042	Human H3N2 IAVs	Human	H3N2		2008	USA	A/Pennsylvania/PIT02/2008	agtgatgccccattccttgatcggcttcgccgagatcag aggtccctaaggggaagaggcaatactc	-	-
CY036931	Human H3N2 IAVs	Human	H3N2		2008	USA	A/Pennsylvania/PIT04/2008	agtgatgccccattccttgatcggcttcgccgagatcag aggtccctaaggggaagaggcaatactc	-	-
CY035162	Human H3N2 IAVs	Human	H3N2		2008	USA	A/Pennsylvania/PIT12/2008	agtgatgccccattccttgatcggcttcgccgagatcag aggtccctaaggggaagaggcaatactc	-	-
CY035058	Human H3N2 IAVs	Human	H3N2		2008	USA	A/Pennsylvania/PIT08/2008	agtgatgccccattccttgatcggcttcgccgagatcag aggtccctaaggggaagaggcaatactc	-	-
CY036939	Human H3N2 IAVs	Human	H3N2		2008	USA	A/Pennsylvania/PIT42/2008	agtgatgccccattccttgatcggcttcgccgagatcag aggtccctaaggggaagaggcaatactc	-	-
CY038491	Human H3N2 IAVs	Human	H3N2		2008	USA	A/Pennsylvania/PIT37/2008	agtgatgccccattccttgatcggcttcgccgagatcag aggtccctaaggggaagaggcaatactc	-	-
CY035194	Human H3N2 IAVs	Human	H3N2		2008	USA	A/Pennsylvania/PIT43/2008	agtgatgccccattccttgatcggcttcgccgagatcag aggtccctaaggggaagaggcaatactc	-	-
CY035154	Human H3N2 IAVs	Human	H3N2		2008	USA	A/Pennsylvania/PIT05/2008	agtgatgccccattccttgatcggcttcgccgagatcag aggtccctaaggggaagaggcaatactc	-	-
CY035050	Human H3N2 IAVs	Human	H3N2		2008	USA	A/Pennsylvania/PIT06/2008	agtgatgccccattccttgatcggcttcgccgagatcag aggtccctaaggggaagaggcaatactc	-	-
CY035170	Human H3N2 IAVs	Human	H3N2		2008	USA	A/Pennsylvania/PIT20/2008	agtgatgccccattccttgatcggcttcgccgagatcag aggtccctaaggggaagaggcaatactc	-	-
CY035066	Human H3N2 IAVs	Human	H3N2		2008	USA	A/Pennsylvania/PIT22/2008	agtgatgccccattccttgatcggcttcgccgagatcag aggtccctaaggggaagaggcaatactc	-	-

CY035186	Human H3N2 IAVs	Human	H3N2		2008	USA	A/Pennsylvania/PIT31/2008	agtgatgccccattccttgatcggcttcgccgagatcag aggtccctaaggggaagaggcaatactc	-	-
CY044832	Human H3N2 IAVs	Human	H3N2		2008	USA	A/Boston/92/2008	agtgatgccccattccttgatcggcttcgccgagatcag aggtccctaaggggaagaggcaatactc	-	-
CY044736	Human H3N2 IAVs	Human	H3N2		2008	USA	A/Boston/60/2008	agtgatgccccattccttgatcggcttcgccgagatcag aggtccctaaggggaagaggcaatactc	-	-
CY037795	Human H3N2 IAVs	Human	H3N2		2008	USA	A/Kentucky/UR07_0028/2008	agtgatgccccattccttgatcggcttcgccgagatcag aggtccctaaggggaagaggcaatactc	-	-
CY173115	Human H3N2 IAVs	Human	H3N2		2008	USA	A/New_York/1131/2008	agtgatgccccattccttgatcggcttcgccgagatcag aggtccctaaggggaagaggcaatactc	-	-
CY173283	Human H3N2 IAVs	Human	H3N2		2008	USA	A/New_York/1155/2008	agtgatgccccattccttgatcggcttcgccgagatcag aggtccctaaggggaagaggcaatactc	-	-
CY173235	Human H3N2 IAVs	Human	H3N2		2008	USA	A/New_York/1148/2008	agtgatgccccattccttgatcggcttcgccgagatcag aggtccctaaggggaagaggcaatactc	-	-
CY039419	Human H3N2 IAVs	Human	H3N2		2008	USA	A/Mississippi/UR07_0024/2008	agtgatgccccattccttgatcggcttcgccgagatcag aggtccctaaggggaagaggcaatactc	-	-
CY173123	Human H3N2 IAVs	Human	H3N2		2008	USA	A/New_York/1133/2008	agtgatgccccattccttgatcggcttcgccgagatcag aggtccctaaggggaagaggcaatactc	-	-
CY044480	Human H3N2 IAVs	Human	H3N2		2008	USA	A/Boston/10/2008	agtgatgccccattccttgatcggcttcgccgagatcag aggtccctaaggggaagaggcaatactc	-	-
CY044840	Human H3N2 IAVs	Human	H3N2		2008	USA	A/Boston/94/2008	agtgatgccccattccttgatcggcttcgccgagatcag aggtccctaaggggaagaggcaatactc	-	-
CY087346	Human H3N2 IAVs	Human	H3N2		2008	Uganda	A/Uganda/MUWRP_006/2008	agtgatgccccattccttgatcggcttcgccgagatcag aggtccctaaggggaagaggcaatactc	-	-
CY087472	Human H3N2 IAVs	Human	H3N2		2008	Uganda	A/Uganda/MUWRP_023/2008	agtgatgccccattccttgatcggcttcgccgagatcag aggtccctaaggggaagaggcaatactc	-	-
CY087519	Human H3N2 IAVs	Human	H3N2		2008	Uganda	A/Uganda/MUWRP_029/2008	agtgatgccccattccttgatcggcttcgccgagatcag aggtccctaaggggaagaggcaatactc	-	-
CY087610	Human H3N2 IAVs	Human	H3N2		2008	Uganda	A/Uganda/MUWRP_042/2008	agtgatgccccattccttgatcggcttcgccgagatcag aggtccctaaggggaagaggcaatactc	-	-
CY087624	Human H3N2 IAVs	Human	H3N2		2008	Uganda	A/Uganda/MUWRP_044/2008	agtgatgccccattccttgatcggcttcgccgagatcag aggtccctaaggggaagaggcaatactc	-	-
FJ912980	Human H3N2 IAVs	Human	H3N2		2008	Thailand	A/Thailand/CU356/2008	agtgatgccccattccttgatcggcttcgccgagatcag aggtccctaaggggaagaggcaatactc	-	-
FJ912988	Human H3N2 IAVs	Human	H3N2		2008	Thailand	A/Thailand/CU370/2008	agtgatgccccattccttgatcggcttcgccgagatcag aggtccctaaggggaagaggcaatactc	-	-
CY147639	Human H3N2 IAVs	Human	H3N2		2008	Mexico	A/Mexico/24010/2008	agtgatgccccattccttgatcggcttcgccgagatcag aggtccctaaggggaagaggcaatactc	-	-
CY147647	Human H3N2 IAVs	Human	H3N2		2008	Mexico	A/Mexico/24011/2008	agtgatgccccattccttgatcggcttcgccgagatcag aggtccctaaggggaagaggcaatactc	-	-
CY073916	Human H3N2 IAVs	Human	H3N2		2008	Mexico	A/Mexico/UASLP_012/2008	agtgatgccccattccttgatcggcttcgccgagatcag aggtccctaaggggaagaggcaatactc	-	-
CY038843	Human H3N2 IAVs	Human	H3N2		2008	USA	A/California/UR07_0076/2008	agtgatgccccattccttgatcggcttcgccgagatcag aggtccctaaggggaagaggcaatactc	-	-
CY147671	Human H3N2 IAVs	Human	H3N2		2008	Mexico	A/Mexico/24014/2008	agtgatgccccattccttgatcggcttcgccgagatcag aggtccctaaggggaagaggcaatactc	-	-
CY036971	Human H3N2 IAVs	Human	H3N2		2008	USA	A/New_York/UR07_0080/2008	agtgatgccccattccttgatcggcttcgccgagatcag aggtccctaaggggaagaggcaatactc	-	-
CY104567	Human H3N2 IAVs	Human	H1N1		2008	Viet_Nam	A/DaNang/DN311/2008	agtgatgccccattccttgatcggcttcgccgagatcag aggtccctaaggggaagaggcaatactc	-	-

KJ473726	Human H3N2 IAVs	Human	H3N2		2008	China	A/Guangdong/ST798/2008	agtgatgccccattccttgatcggcttcgccgagatcag aggtccctaaggggaagaggcaatactc	-	-
CY037595	Human H3N2 IAVs	Human	H3N2		2008	USA	A/Kentucky/UR07_0108/2008	agtgatgccccattccttgatcggcttcgccgagatcag aggtccctaaggggaagaggcaatactc	-	-
CY173299	Human H3N2 IAVs	Human	H3N2		2008	USA	A/New_York/1157/2008	agtgatgccccattccttgatcggcttcgccgagatcag aggtccctaaggggaagaggcaatactc	-	-
CY173099	Human H3N2 IAVs	Human	H3N2		2008	USA	A/New_York/1129/2008	agtgatgccccattccttgatcggcttcgccgagatcag aggtccctaaggggaagaggcaatactc	-	-
CY173163	Human H3N2 IAVs	Human	H3N2		2008	USA	A/New_York/1139/2008	agtgatgccccattccttgatcggcttcgccgagatcag aggtccctaaggggaagaggcaatactc	-	-
CY173171	Human H3N2 IAVs	Human	H3N2		2008	USA	A/New_York/1140/2008	agtgatgccccattccttgatcggcttcgccgagatcag aggtccctaaggggaagaggcaatactc	-	-
CY173179	Human H3N2 IAVs	Human	H3N2		2008	USA	A/New_York/1141/2008	agtgatgccccattccttgatcggcttcgccgagatcag aggtccctaaggggaagaggcaatactc	-	-
CY173227	Human H3N2 IAVs	Human	H3N2		2008	USA	A/New_York/1147/2008	agtgatgccccattccttgatcggcttcgccgagatcag aggtccctaaggggaagaggcaatactc	-	-
CY037723	Human H3N2 IAVs	Human	H3N2		2008	USA	A/Mississippi/UR07_0100/2008	agtgatgccccattccttgatcggcttcgccgagatcag aggtccctaaggggaagaggcaatactc	-	-
CY118179	Human H3N2 IAVs	Human	H3N2		2008	Malaysia	A/Malaysia/2075556/2008	agtgatgccccattccttgatcggcttcgccgagatcag aggtccctaaggggaagaggcaatactc	-	-
CY173291	Human H3N2 IAVs	Human	H3N2		2008	USA	A/New_York/1156/2008	agtgatgccccattccttgatcggcttcgccgagatcag aggtccctaaggggaagaggcaatactc	-	-
CY105866	Human H3N2 IAVs	Human	H3N2		2008	Viet_Nam	A/KhanhHoa/KH474/2008	agtgatgccccattccttgatcggcttcgccgagatcag aggtccctaaggggaagaggcaatactc	-	-
CY104642	Human H3N2 IAVs	Human	H3N2		2008	Viet_Nam	A/KhanhHoa/KH488/2008	agtgatgccccattccttgatcggcttcgccgagatcag aggtccctaaggggaagaggcaatactc	-	-
CY173091	Human H3N2 IAVs	Human	H3N2		2008	USA	A/New_York/1128/2008	agtgatgccccattccttgatcggcttcgccgagatcag aggtccctaaggggaagaggcaatactc	-	-
CY172179	Human H3N2 IAVs	Human	H3N2		2008	USA	A/New_York/1149/2008	agtgatgccccattccttgatcggcttcgccgagatcag aggtccctaaggggaagaggcaatactc	-	-
CY044808	Human H3N2 IAVs	Human	H3N2		2008	USA	A/Boston/79/2008	agtgatgccccattccttgatcggcttcgccgagatcag aggtccctaaggggaagaggcaatactc	-	-
CY173107	Human H3N2 IAVs	Human	H3N2		2008	USA	A/New_York/1130/2008	agtgatgccccattccttgatcggcttcgccgagatcag aggtccctaaggggaagaggcaatactc	-	-
CY037483	Human H3N2 IAVs	Human	H3N2		2008	USA	A/Ohio/UR07_0005/2008	agtgatgccccattccttgatcggcttcgccgagatcag aggtccctaaggggaagaggcaatactc	-	-
CY037875	Human H3N2 IAVs	Human	H3N2		2008	USA	A/Kentucky/UR07_0082/2008	agtgatgccccattccttgatcggcttcgccgagatcag aggtccctaaggggaagaggcaatactc	-	-
CY173139	Human H3N2 IAVs	Human	H3N2		2008	USA	A/New_York/1135/2008	agtgatgccccattccttgatcggcttcgccgagatcag aggtccctaaggggaagaggcaatactc	-	-
CY173267	Human H3N2 IAVs	Human	H3N2		2008	USA	A/New_York/1153/2008	agtgatgccccattccttgatcggcttcgccgagatcag aggtccctaaggggaagaggcaatactc	-	-
CY105834	Human H3N2 IAVs	Human	H3N2		2008	Viet_Nam	A/Hue/H05/2008	agtgatgccccattccttgatcggcttcgccgagatcag aggtccctaaggggaagaggcaatactc	-	-
CY105842	Human H3N2 IAVs	Human	H3N2		2008	Viet_Nam	A/Hue/H207/2008	agtgatgccccattccttgatcggcttcgccgagatcag aggtccctaaggggaagaggcaatactc	-	-
CY105850	Human H3N2 IAVs	Human	H3N2		2008	Viet_Nam	A/Hue/H39/2008	agtgatgccccattccttgatcggcttcgccgagatcag aggtccctaaggggaagaggcaatactc	-	-
CY038859	Human H3N2 IAVs	Human	H3N2		2008	USA	A/Kentucky/UR07_0148/2008	agtgatgccccattccttgatcggcttcgccgagatcag aggtccctaaggggaagaggcaatactc	-	-

CY087354	Human H3N2 IAVs	Human	H3N2		2008	Uganda	A/Uganda/MUWRP_007/2008	agtgatgccccattccttgatcggcttcgccgagatcagaggtccctaaggggaagaggcaatactc	-	-
CY087393	Human H3N2 IAVs	Human	H3N2		2008	Uganda	A/Uganda/MUWRP_012/2008	agtgatgccccattccttgatcggcttcgccgagatcagaggtccctaaggggaagaggcaatactc	-	-
CY087594	Human H3N2 IAVs	Human	H3N2		2008	Uganda	A/Uganda/MUWRP_040/2008	agtgatgccccattccttgatcggcttcgccgagatcagaggtccctaaggggaagaggcaatactc	-	-
CY087322	Human H3N2 IAVs	Human	H3N2		2008	Uganda	A/Uganda/MUWRP_002/2008	agtgatgccccattccttgatcggcttcgccgagatcagaggtccctaaggggaagaggcaatactc	-	-
CY087618	Human H3N2 IAVs	Human	H3N2		2008	Uganda	A/Uganda/MUWRP_043/2008	agtgatgccccattccttgatcggcttcgccgagatcagaggtccctaaggggaagaggcaatactc	-	-
CY087656	Human H3N2 IAVs	Human	H3N2		2008	Uganda	A/Uganda/MUWRP_048/2008	agtgatgccccattccttgatcggcttcgccgagatcagaggtccctaaggggaagaggcaatactc	-	-
CY087424	Human H3N2 IAVs	Human	H3N2		2008	Uganda	A/Uganda/MUWRP_016/	agtgatgccccattccttgatcggcttcgccgagatcagaggtccctaaggggaagaggcaatactc	-	-
CY087330	Human H3N2 IAVs	Human	H3N2		2008	Uganda	A/Uganda/MUWRP_003/2008	agtgatgccccattccttgatcggcttcgccgagatcagaggtccctaaggggaagaggcaatactc	-	-
CY087362	Human H3N2 IAVs	Human	H3N2		2008	Uganda	A/Uganda/MUWRP_008/2008	agtgatgccccattccttgatcggcttcgccgagatcagaggtccctaaggggaagaggcaatactc	-	-
CY087369	Human H3N2 IAVs	Human	H3N2		2008	Uganda	A/Uganda/MUWRP_009/2008	agtgatgccccattccttgatcggcttcgccgagatcagaggtccctaaggggaagaggcaatactc	-	-
CY087377	Human H3N2 IAVs	Human	H3N2		2008	Uganda	A/Uganda/MUWRP_010/2008	agtgatgccccattccttgatcggcttcgccgagatcagaggtccctaaggggaagaggcaatactc	-	-
CY087401	Human H3N2 IAVs	Human	H3N2		2008	Uganda	A/Uganda/MUWRP_013/2008	agtgatgccccattccttgatcggcttcgccgagatcagaggtccctaaggggaagaggcaatactc	-	-
CY087408	Human H3N2 IAVs	Human	H3N2		2008	Uganda	A/Uganda/MUWRP_014/2008	agtgatgccccattccttgatcggcttcgccgagatcagaggtccctaaggggaagaggcaatactc	-	-
CY087432	Human H3N2 IAVs	Human	H3N2		2008	Uganda	A/Uganda/MUWRP_017/2008	agtgatgccccattccttgatcggcttcgccgagatcagaggtccctaaggggaagaggcaatactc	-	-
CY087438	Human H3N2 IAVs	Human	H3N2		2008	Uganda	A/Uganda/MUWRP_018/2008	agtgatgccccattccttgatcggcttcgccgagatcagaggtccctaaggggaagaggcaatactc	-	-
CY087446	Human H3N2 IAVs	Human	H3N2		2008	Uganda	A/Uganda/MUWRP_019/2008	agtgatgccccattccttgatcggcttcgccgagatcagaggtccctaaggggaagaggcaatactc	-	-
CY087680	Human H3N2 IAVs	Human	H3N2		2008	Uganda	A/Uganda/MUWRP_051/2008	agtgatgccccattccttgatcggcttcgccgagatcagaggtccctaaggggaagaggcaatactc	-	-
CY087496	Human H3N2 IAVs	Human	H3N2		2008	Uganda	A/Uganda/MUWRP_026/2008	agtgatgccccattccttgatcggcttcgccgagatcagaggtccctaaggggaagaggcaatactc	-	-
CY087461	Human H3N2 IAVs	Human	H3N2		2008	Uganda	A/Uganda/MUWRP_021/2008	agtgatgccccattccttgatcggcttcgccgagatcagaggtccctaaggggaagaggcaatactc	-	-
CY087664	Human H3N2 IAVs	Human	H3N2		2008	Uganda	A/Uganda/MUWRP_049/2008	agtgatgccccattccttgatcggcttcgccgagatcagaggtccctaaggggaagaggcaatactc	-	-
CY087480	Human H3N2 IAVs	Human	H3N2		2008	Uganda	A/Uganda/MUWRP_024/2008	agtgatgccccattccttgatcggcttcgccgagatcagaggtccctaaggggaagaggcaatactc	-	-
CY087632	Human H3N2 IAVs	Human	H3N2		2008	Uganda	A/Uganda/MUWRP_045/2008	agtgatgccccattccttgatcggcttcgccgagatcagaggtccctaaggggaagaggcaatactc	-	-
CY087640	Human H3N2 IAVs	Human	H3N2		2008	Uganda	A/Uganda/MUWRP_046/2008	agtgatgccccattccttgatcggcttcgccgagatcagaggtccctaaggggaagaggcaatactc	-	-
HQ853538	Human H3N2 IAVs	Human	H3N2		2008	India	A/KOL/1728/2008	agtgatgccccattccttgatcggcttcgccgagatcagaggtccctaaggggaagaggcaatactc	-	-
HQ853533	Human H3N2 IAVs	Human	H3N2		2008	India	A/KOL/1537/2008	agtgatgccccattccttgatcggcttcgccgagatcagaggtccctaaggggaagaggcaatactc	-	-

HQ853536	Human H3N2 IAVs	Human	H3N2		2008	India	A/KOL/1691/2008	agtgatgccccattccttgatcggcttcgccgagatcag<br>aggtccctaaggggaagaggcaatactc	-	-
HQ853540	Human H3N2 IAVs	Human	H3N2		2008	India	A/KOL/1822/2008	agtgatgccccattccttgatcggcttcgccgagatcag<br>aggtccctaaggggaagaggcaatactc	-	-
HQ853534	Human H3N2 IAVs	Human	H3N2		2008	India	A/KOL/1687/2008	agtgatgccccattccttgatcggcttcgccgagatcag<br>aggtccctaaggggaagaggcaatactc	-	-
HQ853537	Human H3N2 IAVs	Human	H3N2		2008	India	A/KOL/1708/2008	agtgatgccccattccttgatcggcttcgccgagatcag<br>aggtccctaaggggaagaggcaatactc	-	-
FJ913014	Human H3N2 IAVs	Human	H3N2		2008	Thailand	A/Thailand/CU_1103/2008	agtgatgccccattccttgatcggcttcgccgagatcag<br>aggtccctaaggggaagaggcaatactc	-	-
HQ853535	Human H3N2 IAVs	Human	H3N2		2008	India	A/KOL/1690/2008	agtgatgccccattccttgatcggcttcgccgagatcag<br>aggtccctaaggggaagaggcaatactc	-	-
CY147655	Human H3N2 IAVs	Human	H3N2		2008	Mexico	A/Mexico/24012/2008	agtgatgccccattccttgatcggcttcgccgagatcag<br>aggtccctaaggggaagaggcaatactc	-	-
CY037307	Human H3N2 IAVs	Human	H3N2		2008	Qatar	A/Qatar/AF02/2008	agtgatgccccattccttgatcggcttcgccgagatcag<br>aggtccctaaggggaagaggcaatactc	-	-
CY105874	Human H3N2 IAVs	Human	H3N2		2008	Viet_Nam	A/KhanhHoa/KH475/2008	agtgatgccccattccttgatcggcttcgccgagatcag<br>aggtccctaaggggaagaggcaatactc	-	-
CY104650	Human H3N2 IAVs	Human	H3N2		2008	Viet_Nam	A/KhanhHoa/KH494/2008	agtgatgccccattccttgatcggcttcgccgagatcag<br>aggtccctaaggggaagaggcaatactc	-	-
CY113017	Human H3N2 IAVs	Human	H3N2		2008	Netherlands	A/Netherlands/377/2008	agtgatgccccattccttgatcggcttcgccgagatcag<br>aggtccctaaggggaagaggcaatactc	-	-
CY044860	Human H3N2 IAVs	Human	H3N2		2008	USA	A/Boston/9/2008	agtgatgccccattccttgatcggcttcgccgagatcag<br>aggtccctaaggggaagaggcaatactc	-	-
CY087503	Human H3N2 IAVs	Human	H3N2		2008	Uganda	A/Uganda/MUWRP_027/2008	agtgatgccccattccttgatcggcttcgccgagatcag<br>aggtccctaaggggaaggggcaatactc	-	-
CY087511	Human H3N2 IAVs	Human	H3N2		2008	Uganda	A/Uganda/MUWRP_028/2008	agtgatgccccattccttgatcggcttcgccgagatcag<br>aggtccctaaggggaaggggcaatactc	-	-
CY087578	Human H3N2 IAVs	Human	H3N2		2008	Uganda	A/Uganda/MUWRP_038/2008	agtgatgccccattccttgatcggcttcgtcgagatcag<br>aggtccctaaggggaagaggcaacactc	-	-
CY087602	Human H3N2 IAVs	Human	H3N2		2008	Uganda	A/Uganda/MUWRP_041/2008	agtgatgccccattccttgatcggcttcgtcgagatcag<br>aggtccctaaggggaagaggcaacactc	-	-
CY041470	Human H3N2 IAVs	Human	H3N2		2008	USA	A/Florida/UR07_0095/2008	agtgatgccccattccttgatcggcttcgtcgagatcag<br>aggtccctaaggggaagaggcaatactc	+	+
CY087338	Human H3N2 IAVs	Human	H3N2		2008	Uganda	A/Uganda/MUWRP_005/2008	agtgatgccccattccttgatcggcttcgtcgagatcag<br>aggtccctaaggggaagaggcaatactc	+	+
CY087385	Human H3N2 IAVs	Human	H3N2		2008	Uganda	A/Uganda/MUWRP_011/2008	agtgatgccccattccttgatcggcttcgtcgagatcag<br>aggtccctaaggggaagaggcaatactc	+	+
CY087453	Human H3N2 IAVs	Human	H3N2		2008	Uganda	A/Uganda/MUWRP_020/2008	agtgatgccccattccttgatcggcttcgtcgagatcag<br>aggtccctaaggggaagaggcaatactc	+	+
CY087570	Human H3N2 IAVs	Human	H3N2		2008	Uganda	A/Uganda/MUWRP_037/2008	agtgatgccccattccttgatcggcttcgtcgagatcag<br>aggtccctaaggggaagaggcaatactc	+	+
CY087535	Human H3N2 IAVs	Human	H3N2		2008	Uganda	A/Uganda/MUWRP_032/2008	agtgatgccccattccttgatcggcttcgtcgagatcag<br>aggtccctaaggggaagaggcaatactc	+	+
CY037827	Human H3N2 IAVs	Human	H3N2		2008	USA	A/Kentucky/UR07_0085/2008	agtgatgccccattccttgatcggctttgccgagatcag<br>aggtccctaaggggaagaggcaatactc	-	-
KC620458	Human H3N2 IAVs	Swine	H3N2		2008	China	A/swine/HuNan/01/2008	ggtgatgccccattccttgatcggcttcgccgagatcag<br>aagtccctaaggggaagaggcagcactc	+	+
JX138529	Human H3N2 IAVs	Swine	H3N2		2008	China	A/swine/Hunan/3/2008	ggtgatgccccattccttgatcggcttcgccgagatcag<br>aagtccctaaggggaagaggcagcactc	+	+

GU271978	Human H3N2 IAVs	Human	H3N2		2009	Thailand	A/Thailand/CU_B1672/2009	aatgatgccccattccttgatcggcttcgccgagatca gaggtccctaaagggaagaggcaatactc	-	-
CY158749	Human H3N2 IAVs	Swine	H3N2		2009	Canada	A/swine/Saskatchewan/02903/2009	aatgatgctccattccttgatcggcttcgccgagatcag aggtccctaatgggaagaggcaacactc	+	+
CY068445	Human H3N2 IAVs	Human	H3N2		2009	USA	A/California/VRDL293/2009	agcgatgccccattccttgatcggcttcgccgagatca gaggtccctaaggggaagaggcaatactc	-	-
CY068181	Human H3N2 IAVs	Human	H3N2		2009	USA	A/California/VRDL166/2009	agtgatgccccattcctcgatcggcttcgccgagatca gaggtccctaaggggaagaggcaatactc	-	-
CY080669	Human H3N2 IAVs	Human	H3N2		2009	USA	A/Boston/16/2009	agtgatgccccattccttgatcggcttcgccgagatca aaggtccctaaggggaagaggcaatactc	-	-
CY089737	Human H3N2 IAVs	Human	H3N2		2009	USA	A/Boston/40/2009	agtgatgccccattccttgatcggcttcgccgagatca aaggtccctaaggggaagaggcaatactc	-	-
CY064883	Human H3N2 IAVs	Human	H3N2		2009	USA	A/California/VRDL147/2009	agtgatgccccattccttgatcggcttcgccgagatca aaggtccctaaggggaagaggcaatactc	-	-
CY068157	Human H3N2 IAVs	Human	H3N2		2009	USA	A/California/VRDL161/2009	agtgatgccccattccttgatcggcttcgccgagatca aaggtccctaaggggaagaggcaatactc	-	-
CY068189	Human H3N2 IAVs	Human	H3N2		2009	USA	A/California/VRDL168/2009	agtgatgccccattccttgatcggcttcgccgagatca aaggtccctaaggggaagaggcaatactc	-	-
CY068213	Human H3N2 IAVs	Human	H3N2		2009	USA	A/California/VRDL172/2009	agtgatgccccattccttgatcggcttcgccgagatca aaggtccctaaggggaagaggcaatactc	-	-
CY068229	Human H3N2 IAVs	Human	H3N2		2009	USA	A/California/VRDL188/2009	agtgatgccccattccttgatcggcttcgccgagatca aaggtccctaaggggaagaggcaatactc	-	-
CY068237	Human H3N2 IAVs	Human	H3N2		2009	USA	A/California/VRDL211/2009	agtgatgccccattccttgatcggcttcgccgagatca aaggtccctaaggggaagaggcaatactc	-	-
CY068341	Human H3N2 IAVs	Human	H3N2		2009	USA	A/California/VRDL261/2009	agtgatgccccattccttgatcggcttcgccgagatca aaggtccctaaggggaagaggcaatactc	-	-
CY068818	Human H3N2 IAVs	Human	H3N2		2009	USA	A/California/VRDL269/2009	agtgatgccccattccttgatcggcttcgccgagatca aaggtccctaaggggaagaggcaatactc	-	-
CY068485	Human H3N2 IAVs	Human	H3N2		2009	USA	A/California/VRDL310/2009	agtgatgccccattccttgatcggcttcgccgagatca aaggtccctaaggggaagaggcaatactc	-	-
CY068493	Human H3N2 IAVs	Human	H3N2		2009	USA	A/California/VRDL312/2009	agtgatgccccattccttgatcggcttcgccgagatca aaggtccctaaggggaagaggcaatactc	-	-
CY068541	Human H3N2 IAVs	Human	H3N2		2009	USA	A/California/VRDL321/2009	agtgatgccccattccttgatcggcttcgccgagatca aaggtccctaaggggaagaggcaatactc	-	-
CY068882	Human H3N2 IAVs	Human	H3N2		2009	USA	A/California/VRDL393/2009	agtgatgccccattccttgatcggcttcgccgagatca aaggtccctaaggggaagaggcaatactc	-	-
CY093283	Human H3N2 IAVs	Human	H3N2		2009	USA	A/Colorado/WRAIR1246P/2009	agtgatgccccattccttgatcggcttcgccgagatca aaggtccctaaggggaagaggcaatactc	-	-
CY069321	Human H3N2 IAVs	Human	H3N2		2009	Japan	A/Japan/WRAIR1037P/2009	agtgatgccccattccttgatcggcttcgccgagatca aaggtccctaaggggaagaggcaatactc	-	-
CY050640	Human H3N2 IAVs	Human	H3N2		2009	USA	A/New_York/3146/2009	agtgatgccccattccttgatcggcttcgccgagatca aaggtccctaaggggaagaggcaatactc	-	-
CY058768	Human H3N2 IAVs	Human	H3N2		2009	USA	A/New_York/3360/2009	agtgatgccccattccttgatcggcttcgccgagatca aaggtccctaaggggaagaggcaatactc	-	-
CY068714	Human H3N2 IAVs	Human	H3N2		2009	USA	A/California/VRDL185/2009	agtgatgccccattccttgatcggcttcgccgagatca aaggtccctaaggggaagaggcaatactc	-	-
CY068778	Human H3N2 IAVs	Human	H3N2		2009	USA	A/California/VRDL205/2009	agtgatgccccattccttgatcggcttcgccgagatca aaggtccctaaggggaagaggcaatactc	-	-
CY072202	Human H3N2 IAVs	Human	H3N2		2009	USA	A/California/VRDL385/2009	agtgatgccccattccttgatcggcttcgccgagatca aaggtccctaaggggaagaggcaatactc	-	-

CY147623	Human H3N2 IAVs	Human	H3N2		2009	Mexico	A/Mexico/24008/2009	agtgatgccccattccttgatcggcttcgccgagatca<br>aaggtccctaaggggaagaggcaatactc	-	-
CY068389	Human H3N2 IAVs	Human	H3N2		2009	USA	A/California/VRDL271/2009	agtgatgccccattccttgatcggcttcgccgagatca<br>aaggtccctaaggggaagaggcaatactc	-	-
CY068682	Human H3N2 IAVs	Human	H3N2		2009	USA	A/California/VRDL178/2009	agtgatgccccattccttgatcggcttcgccgagatca<br>aaggtccctaaggggaagaggcaatactc	-	-
CY068794	Human H3N2 IAVs	Human	H3N2		2009	USA	A/California/VRDL208/2009	agtgatgccccattccttgatcggcttcgccgagatca<br>aaggtccctaaggggaagaggcaatactc	-	-
CY068261	Human H3N2 IAVs	Human	H3N2		2009	USA	A/California/VRDL230/2009	agtgatgccccattccttgatcggcttcgccgagatca<br>aaggtccctaaggggaagaggcaatactc	-	-
CY068557	Human H3N2 IAVs	Human	H3N2		2009	USA	A/California/VRDL325/2009	agtgatgccccattccttgatcggcttcgccgagatca<br>aaggtccctaaggggaagaggcaatactc	-	-
CY092365	Human H3N2 IAVs	Human	H3N2		2009	USA	A/California/VRDL242/2009	agtgatgccccattccttgatcggcttcgccgagatca<br>aaggtccctaaggggaagaggcaatactc	-	-
CY068309	Human H3N2 IAVs	Human	H3N2		2009	USA	A/California/VRDL250/2009	agtgatgccccattccttgatcggcttcgccgagatca<br>aaggtccctaaggggaagaggcaatactc	-	-
CY068637	Human H3N2 IAVs	Human	H3N2		2009	USA	A/California/VRDL394/2009	agtgatgccccattccttgatcggcttcgccgagatca<br>aaggtccctaaggggaagaggcaatactc	-	-
CY068738	Human H3N2 IAVs	Human	H3N2		2009	USA	A/California/VRDL198/2009	agtgatgccccattccttgatcggcttcgccgagatca<br>aaggtccctaaggggaagaggcaatactc	-	-
CY068293	Human H3N2 IAVs	Human	H3N2		2009	USA	A/California/VRDL247/2009	agtgatgccccattccttgatcggcttcgccgagatca<br>aaggtccctaaggggaagaggcaatactc	-	-
CY067209	Human H3N2 IAVs	Human	H3N2		2009	USA	A/California/VRDL291/2009	agtgatgccccattccttgatcggcttcgccgagatca<br>aaggtccctaaggggaagaggcaatactc	-	-
CY068754	Human H3N2 IAVs	Human	H3N2		2009	USA	A/California/VRDL201/2009	agtgatgccccattccttgatcggcttcgccgagatca<br>aaggtccctaaggggaagaggcaatactc	-	-
CY068245	Human H3N2 IAVs	Human	H3N2		2009	USA	A/California/VRDL212/2009	agtgatgccccattccttgatcggcttcgccgagatca<br>aaggtccctaaggggaagaggcaatactc	-	-
CY068850	Human H3N2 IAVs	Human	H3N2		2009	USA	A/California/VRDL387/2009	agtgatgccccattccttgatcggcttcgccgagatca<br>aaggtccctaaggggaagaggcaatactc	-	-
CY093355	Human H3N2 IAVs	Human	H3N2		2009	Kuwait	A/Kuwait/WRAIR1561P/2009	agtgatgccccattccttgatcggcttcgccgagatcag<br>agatccctaaggggaagaggcaatactc	-	*
CY089397	Human H3N2 IAVs	Human	H3N2		2009	USA	A/Boston/5/2009	agtgatgccccattccttgatcggcttcgccgagatcag<br>aggtccataaggggaagaggcaatactc	+	+
CY093299	Human H3N2 IAVs	Human	H3N2		2009	USA	A/Arkansas/WRAIR1251P/2009	agtgatgccccattccttgatcggcttcgccgagatcag<br>aggtccctaaagggaagaggcaatactc	+	+
CY072210	Human H3N2 IAVs	Human	H3N2		2009	USA	A/California/VRDL386/2009	agtgatgccccattccttgatcggcttcgccgagatcag<br>aggtccctaaggggaagaggcaacactc	-	-
CY068085	Human H3N2 IAVs	Human	H3N2		2009	USA	A/California/VRDL362/2009	agtgatgccccattccttgatcggcttcgccgagatcag<br>aggtccctaaggggaagaggcaacactc	-	-
CY113041	Human H3N2 IAVs	Human	H3N2		2009	Netherlands	A/Netherlands/761/2009	agtgatgccccattccttgatcggcttcgccgagatcag<br>aggtccctaaggggaagaggcaacactc	-	-
JQ340010	Human H3N2 IAVs	Human	H1N2		2009	India	A/Eastern_India/N_1289/2009	agtgatgccccattccttgatcggcttcgccgagatcag<br>aggtccctaaggggaagaggcaacactc	-	-
CY093267	Human H3N2 IAVs	Human	H3N2		2009	USA	A/Alabama/WRAIR1242P/2009	agtgatgccccattccttgatcggcttcgccgagatcag<br>aggtccctaaggggaagaggcaatactc	-	-
CY093363	Human H3N2 IAVs	Human	H3N2		2009	USA	A/Arizona/WRAIR1562P/2009	agtgatgccccattccttgatcggcttcgccgagatcag<br>aggtccctaaggggaagaggcaatactc	-	-
CY089745	Human H3N2 IAVs	Human	H3N2		2009	USA	A/Boston/73/2009	agtgatgccccattccttgatcggcttcgccgagatcag<br>aggtccctaaggggaagaggcaatactc	-	-

CY068730	Human H3N2 IAVs	Human	H3N2		2009	USA	A/California/VRDL196/2009	agtgatgccccattccttgatcggcttcgccgagatcag aggtccctaaggggaagaggcaatactc	-	-
CY069505	Human H3N2 IAVs	Human	H3N2		2009	USA	A/South_Dakota/WRAIR1177P/2009	agtgatgccccattccttgatcggcttcgccgagatcag aggtccctaaggggaagaggcaatactc	-	-
CY064827	Human H3N2 IAVs	Human	H3N2		2009	USA	A/California/VRDL138/2009	agtgatgccccattccttgatcggcttcgccgagatcag aggtccctaaggggaagaggcaatactc	-	-
CY066523	Human H3N2 IAVs	Human	H3N2		2009	USA	A/California/VRDL156/2009	agtgatgccccattccttgatcggcttcgccgagatcag aggtccctaaggggaagaggcaatactc	-	-
CY068381	Human H3N2 IAVs	Human	H3N2		2009	USA	A/California/VRDL268/2009	agtgatgccccattccttgatcggcttcgccgagatcag aggtccctaaggggaagaggcaatactc	-	-
CY068421	Human H3N2 IAVs	Human	H3N2		2009	USA	A/California/VRDL278/2009	agtgatgccccattccttgatcggcttcgccgagatcag aggtccctaaggggaagaggcaatactc	-	-
CY068405	Human H3N2 IAVs	Human	H3N2		2009	USA	A/California/VRDL274/2009	agtgatgccccattccttgatcggcttcgccgagatcag aggtccctaaggggaagaggcaatactc	-	-
CY064819	Human H3N2 IAVs	Human	H3N2		2009	USA	A/California/VRDL136/2009	agtgatgccccattccttgatcggcttcgccgagatcag aggtccctaaggggaagaggcaatactc	-	-
CY068165	Human H3N2 IAVs	Human	H3N2		2009	USA	A/California/VRDL164/2009	agtgatgccccattccttgatcggcttcgccgagatcag aggtccctaaggggaagaggcaatactc	-	-
CY067225	Human H3N2 IAVs	Human	H3N2		2009	USA	A/California/VRDL302/2009	agtgatgccccattccttgatcggcttcgccgagatcag aggtccctaaggggaagaggcaatactc	-	-
CY068149	Human H3N2 IAVs	Human	H3N2		2009	USA	A/California/VRDL160/2009	agtgatgccccattccttgatcggcttcgccgagatcag aggtccctaaggggaagaggcaatactc	-	-
CY068205	Human H3N2 IAVs	Human	H3N2		2009	USA	A/California/VRDL170/2009	agtgatgccccattccttgatcggcttcgccgagatcag aggtccctaaggggaagaggcaatactc	-	-
CY173515	Human H3N2 IAVs	Human	H3N2		2009	USA	A/New_York/1188/2009	agtgatgccccattccttgatcggcttcgccgagatcag aggtccctaaggggaagaggcaatactc	-	-
CY173531	Human H3N2 IAVs	Human	H3N2		2009	USA	A/New_York/1190/2009	agtgatgccccattccttgatcggcttcgccgagatcag aggtccctaaggggaagaggcaatactc	-	-
CY068365	Human H3N2 IAVs	Human	H3N2		2009	USA	A/California/VRDL265/2009	agtgatgccccattccttgatcggcttcgccgagatcag aggtccctaaggggaagaggcaatactc	-	-
CY068597	Human H3N2 IAVs	Human	H3N2		2009	USA	A/California/VRDL333/2009	agtgatgccccattccttgatcggcttcgccgagatcag aggtccctaaggggaagaggcaatactc	-	-
CY089753	Human H3N2 IAVs	Human	H3N2		2009	USA	A/Boston/75/2009	agtgatgccccattccttgatcggcttcgccgagatcag aggtccctaaggggaagaggcaatactc	-	-
CY173563	Human H3N2 IAVs	Human	H3N2		2009	USA	A/New_York/1194/2009	agtgatgccccattccttgatcggcttcgccgagatcag aggtccctaaggggaagaggcaatactc	-	-
CY068802	Human H3N2 IAVs	Human	H3N2		2009	USA	A/California/VRDL210/2009	agtgatgccccattccttgatcggcttcgccgagatcag aggtccctaaggggaagaggcaatactc	-	-
CY113025	Human H3N2 IAVs	Human	H3N2		2009	Netherlands	A/Netherlands/69/2009	agtgatgccccattccttgatcggcttcgccgagatcag aggtccctaaggggaagaggcaatactc	-	-
CY173539	Human H3N2 IAVs	Human	H3N2		2009	USA	A/New_York/1191/2009	agtgatgccccattccttgatcggcttcgccgagatcag aggtccctaaggggaagaggcaatactc	-	-
CY173579	Human H3N2 IAVs	Human	H3N2		2009	USA	A/New_York/1196/2009	agtgatgccccattccttgatcggcttcgccgagatcag aggtccctaaggggaagaggcaatactc	-	-
CY121756	Human H3N2 IAVs	Human	H3N2		2009	Finland	A/Finland/97/2009	agtgatgccccattccttgatcggcttcgccgagatcag aggtccctaaggggaagaggcaatactc	-	-
CY121780	Human H3N2 IAVs	Human	H3N2		2009	Finland	A/Finland/97/2009	agtgatgccccattccttgatcggcttcgccgagatcag aggtccctaaggggaagaggcaatactc	-	-
CY064859	Human H3N2 IAVs	Human	H3N2		2009	USA	A/California/VRDL144/2009	agtgatgccccattccttgatcggcttcgccgagatcag aggtccctaaggggaagaggcaatactc	-	-

CY068866	Human H3N2 IAVs	Human	H3N2		2009	USA	A/California/VRDL391/2009	agtgatgccccattccttgatcggcttcgccgagatcag aggtccctaaggggaagaggcaatactc	-	-
CY067233	Human H3N2 IAVs	Human	H3N2		2009	USA	A/California/VRDL304/2009	agtgatgccccattccttgatcggcttcgccgagatcag aggtccctaaggggaagaggcaatactc	-	-
CY068469	Human H3N2 IAVs	Human	H3N2		2009	USA	A/California/VRDL308/2009	agtgatgccccattccttgatcggcttcgccgagatcag aggtccctaaggggaagaggcaatactc	-	-
CY068549	Human H3N2 IAVs	Human	H3N2		2009	USA	A/California/VRDL322/2009	agtgatgccccattccttgatcggcttcgccgagatcag aggtccctaaggggaagaggcaatactc	-	-
CY067989	Human H3N2 IAVs	Human	H3N2		2009	USA	A/California/VRDL342/2009	agtgatgccccattccttgatcggcttcgccgagatcag aggtccctaaggggaagaggcaatactc	-	-
CY067257	Human H3N2 IAVs	Human	H3N2		2009	USA	A/California/VRDL316/2009	agtgatgccccattccttgatcggcttcgccgagatcag aggtccctaaggggaagaggcaatactc	-	-
CY068834	Human H3N2 IAVs	Human	H3N2		2009	USA	A/California/VRDL383/2009	agtgatgccccattccttgatcggcttcgccgagatcag aggtccctaaggggaagaggcaatactc	-	-
CY092357	Human H3N2 IAVs	Human	H3N2		2009	USA	A/California/VRDL197/2009	agtgatgccccattccttgatcggcttcgccgagatcag aggtccctaaggggaagaggcaatactc	-	-
KJ855463	Human H3N2 IAVs	Human	H3N2		2009	Mexico	A/Mexico/JAL25206/2009	agtgatgccccattccttgatcggcttcgccgagatcag aggtccctaaggggaagaggcaatactc	-	-
CY093291	Human H3N2 IAVs	Human	H3N2		2009	USA	A/New_Jersey/WRAIR1248P/2009	agtgatgccccattccttgatcggcttcgccgagatcag aggtccctaaggggaagaggcaatactc	-	-
CY093244	Human H3N2 IAVs	Human	H3N2		2009	South_Korea	A/South_Korea/WRAIR1171P/2009	agtgatgccccattccttgatcggcttcgccgagatcag aggtccctaaggggaagaggcaatactc	-	-
CY173547	Human H3N2 IAVs	Human	H3N2		2009	USA	A/New_York/1192/2009	agtgatgccccattccttgatcggcttcgccgagatcag aggtccctaaggggaagaggcaatactc	-	-
CY068101	Human H3N2 IAVs	Human	H3N2		2009	USA	A/California/VRDL368/2009	agtgatgccccattccttgatcggcttcgccgagatcag aggtccctaaggggaagaggcaatactc	-	-
KJ855471	Human H3N2 IAVs	Human	H3N2		2009	Mexico	A/Mexico/JAL25216/2009	agtgatgccccattccttgatcggcttcgccgagatcag aggtccctaaggggaagaggcaatactc	-	-
CY092373	Human H3N2 IAVs	Human	H3N2		2009	USA	A/California/VRDL255/2009	agtgatgccccattccttgatcggcttcgccgagatcag aggtccctaaggggaagaggcaatactc	-	-
CY068762	Human H3N2 IAVs	Human	H3N2		2009	USA	A/California/VRDL203/2009	agtgatgccccattccttgatcggcttcgccgagatcag aggtccctaaggggaagaggcaatactc	-	-
CY173571	Human H3N2 IAVs	Human	H3N2		2009	USA	A/New_York/1195/2009	agtgatgccccattccttgatcggcttcgccgagatcag aggtccctaaggggaagaggcaatactc	-	-
CY069481	Human H3N2 IAVs	Human	H3N2		2009	Iraq	A/Iraq/WRAIR1157P/2009	agtgatgccccattccttgatcggcttcgccgagatcag aggtccctaaggggaagaggcaatactc	-	-
CY069561	Human H3N2 IAVs	Human	H3N2		2009	South_Korea	A/Korea/WRAIR1391P/2009	agtgatgccccattccttgatcggcttcgccgagatcag aggtccctaaggggaagaggcaatactc	-	-
CY069337	Human H3N2 IAVs	Human	H3N2		2009	USA	A/Florida/WRAIR1039P/2009	agtgatgccccattccttgatcggcttcgccgagatcag aggtccctaaggggaagaggcaatactc	-	-
CY069473	Human H3N2 IAVs	Human	H3N2		2009	USA	A/New_Jersey/WRAIR1146P/2009	agtgatgccccattccttgatcggcttcgccgagatcag aggtccctaaggggaagaggcaatactc	-	-
CY147727	Human H3N2 IAVs	Human	H3N2		2009	Mexico	A/Mexico/24025/2009	agtgatgccccattccttgatcggcttcgccgagatcag aggtccctaaggggaagaggcaatactc	-	-
CY068786	Human H3N2 IAVs	Human	H3N2		2009	USA	A/California/VRDL207/2009	agtgatgccccattccttgatcggcttcgccgagatcag aggtccctaaggggaagaggcaatactc	-	-
CY089777	Human H3N2 IAVs	Human	H3N2		2009	USA	A/Boston/99/2009	agtgatgccccattccttgatcggcttcgccgagatcag aggtccctaaggggaagaggcaatactc	-	-
CY069433	Human H3N2 IAVs	Human	H3N2		2009	Japan	A/Japan/WRAIR1140P/2009	agtgatgccccattccttgatcggcttcgccgagatcag aggtccctaaggggaagaggcaatactc	-	-

CY050496	Human H3N2 IAVs	Human	H3N2		2009	USA	A/New_York/3060/2009	agtgatgccccattccttgatcggcttcgccgagatcag aggtccctaaggggaagaggcaatactc	-	-
CY173587	Human H3N2 IAVs	Human	H3N2		2009	USA	A/New_York/1197/2009	agtgatgccccattccttgatcggcttcgccgagatcag aggtccctaaggggaagaggcaatactc	-	-
CY173595	Human H3N2 IAVs	Human	H3N2		2009	USA	A/New_York/1198/2009	agtgatgccccattccttgatcggcttcgccgagatcag aggtccctaaggggaagaggcaatactc	-	-
CY093339	Human H3N2 IAVs	Human	H3N2		2009	Guam	A/Guam/WRAIR1557P/2009	agtgatgccccattccttgatcggcttcgccgagatcag aggtccctaaggggaagaggcaatactc	-	-
CY093371	Human H3N2 IAVs	Human	H3N2		2009	Guam	A/Guam/WRAIR1670P/2009	agtgatgccccattccttgatcggcttcgccgagatcag aggtccctaaggggaagaggcaatactc	-	-
CY069441	Human H3N2 IAVs	Human	H3N2		2009	USA	A/Arizona/WRAIR1142P/2009	agtgatgccccattccttgatcggcttcgccgagatcag aggtccctaaggggaagaggcaatactc	-	-
CY067933	Human H3N2 IAVs	Human	H3N2		2009	USA	A/California/VRDL177/2009	agtgatgccccattccttgatcggcttcgccgagatcag aggtccctaaggggaagaggcaatactc	-	-
CY068197	Human H3N2 IAVs	Human	H3N2		2009	USA	A/California/VRDL169/2009	agtgatgccccattccttgatcggcttcgccgagatcag aggtccctaaggggaagaggcaatactc	-	-
CY068629	Human H3N2 IAVs	Human	H3N2		2009	USA	A/California/VRDL338/2009	agtgatgccccattccttgatcggcttcgccgagatcag aggtccctaaggggaagaggcaatactc	-	-
CY092793	Human H3N2 IAVs	Human	mixed		2009	USA	A/California/VRDL364/2009	agtgatgccccattccttgatcggcttcgccgagatcag aggtccctaaggggaagaggcaatactc	-	-
CY068842	Human H3N2 IAVs	Human	H3N2		2009	USA	A/California/VRDL384/2009	agtgatgccccattccttgatcggcttcgccgagatcag aggtccctaaggggaagaggcaatactc	-	-
HQ853545	Human H3N2 IAVs	Human	H3N2		2009	India	A/KOL/1525/2009	agtgatgccccattccttgatcggcttcgccgagatcag aggtccctaaggggaagaggcaatactc	-	-
HQ853546	Human H3N2 IAVs	Human	H3N2		2009	India	A/KOL/2309/2009	agtgatgccccattccttgatcggcttcgccgagatcag aggtccctaaggggaagaggcaatactc	-	-
HQ853547	Human H3N2 IAVs	Human	H3N2		2009	India	A/KOL/2312/2009	agtgatgccccattccttgatcggcttcgccgagatcag aggtccctaaggggaagaggcaatactc	-	-
HQ853548	Human H3N2 IAVs	Human	H3N2		2009	India	A/KOL/2321/2009	agtgatgccccattccttgatcggcttcgccgagatcag aggtccctaaggggaagaggcaatactc	-	-
HQ853549	Human H3N2 IAVs	Human	H3N2		2009	India	A/KOL/2404/2009	agtgatgccccattccttgatcggcttcgccgagatcag aggtccctaaggggaagaggcaatactc	-	-
HQ853542	Human H3N2 IAVs	Human	H3N2		2009	India	A/KOL/402/2009	agtgatgccccattccttgatcggcttcgccgagatcag aggtccctaaggggaagaggcaatactc	-	-
HQ853543	Human H3N2 IAVs	Human	H3N2		2009	India	A/KOL/779/2009	agtgatgccccattccttgatcggcttcgccgagatcag aggtccctaaggggaagaggcaatactc	-	-
HQ853544	Human H3N2 IAVs	Human	H3N2		2009	India	A/KOL/857/2009	agtgatgccccattccttgatcggcttcgccgagatcag aggtccctaaggggaagaggcaatactc	-	-
GU907116	Human H3N2 IAVs	Human	H3N2		2009	China	A/Nanjing/1/2009	agtgatgccccattccttgatcggcttcgccgagatcag aggtccctaaggggaagaggcaatactc	-	-
CY050568	Human H3N2 IAVs	Human	H3N2		2009	USA	A/New_York/3104/2009	agtgatgccccattccttgatcggcttcgccgagatcag aggtccctaaggggaagaggcaatactc	-	-
CY050712	Human H3N2 IAVs	Human	H3N2		2009	USA	A/New_York/3280/2009	agtgatgccccattccttgatcggcttcgccgagatcag aggtccctaaggggaagaggcaatactc	-	-
CY106932	Human H3N2 IAVs	Human	H3N2		2009	Hong_Kong	A/Hong_Kong/H090_756_V10/2009	agtgatgccccattccttgatcggcttcgccgagatcag aggtccctaaggggaagaggcaatactc	-	-
CY115788	Human H3N2 IAVs	Human	H3N2		2009	Hong_Kong	A/Hong_Kong/H090_756_V10/2009	agtgatgccccattccttgatcggcttcgccgagatcag aggtccctaaggggaagaggcaatactc	-	-
CY050704	Human H3N2 IAVs	Human	H3N2		2009	USA	A/New_York/3279/2009	agtgatgccccattccttgatcggcttcgccgagatcag aggtccctaaggggaagaggcaatactc	-	-

GQ902829	Human H3N2 IAVs	Human	H3N2		2009	Thailand	A/Thailand/CU_B657/2009	agtgatgccccattccttgatcggcttcgccgagatcag aggtccctaaggggaagaggcaatactc	-	-
CY087694	Human H3N2 IAVs	Human	H3N2		2009	Uganda	A/Uganda/MUWRP_058/2009	agtgatgccccattccttgatcggcttcgccgagatcag aggtccctaaggggaagaggcaatactc	-	-
CY087701	Human H3N2 IAVs	Human	H3N2		2009	Uganda	A/Uganda/MUWRP_061/2009	agtgatgccccattccttgatcggcttcgccgagatcag aggtccctaaggggaagaggcaatactc	-	-
CY087716	Human H3N2 IAVs	Human	H3N2		2009	Uganda	A/Uganda/MUWRP_065/2009	agtgatgccccattccttgatcggcttcgccgagatcag aggtccctaaggggaagaggcaatactc	-	-
CY087722	Human H3N2 IAVs	Human	H3N2		2009	Uganda	A/Uganda/MUWRP_070/2009	agtgatgccccattccttgatcggcttcgccgagatcag aggtccctaaggggaagaggcaatactc	-	-
CY087728	Human H3N2 IAVs	Human	H3N2		2009	Uganda	A/Uganda/MUWRP_074/2009	agtgatgccccattccttgatcggcttcgccgagatcag aggtccctaaggggaagaggcaatactc	-	-
CY087744	Human H3N2 IAVs	Human	H3N2		2009	Uganda	A/Uganda/MUWRP_079/2009	agtgatgccccattccttgatcggcttcgccgagatcag aggtccctaaggggaagaggcaatactc	-	-
CY087751	Human H3N2 IAVs	Human	H3N2		2009	Uganda	A/Uganda/MUWRP_086/2009	agtgatgccccattccttgatcggcttcgccgagatcag aggtccctaaggggaagaggcaatactc	-	-
CY084338	Human H3N2 IAVs	Human	H3N2		2009	USA	A/New_York/6630/2009	agtgatgccccattccttgatcggcttcgccgagatcag aggtccctaaggggaagaggcaatactc	-	-
CY087736	Human H3N2 IAVs	Human	H3N2		2009	Uganda	A/Uganda/MUWRP_075/2009	agtgatgccccattccttgatcggcttcgccgagatcag aggtccctaaggggaagaggcaatactc	-	-
GQ902797	Human H3N2 IAVs	Human	H3N2		2009	Thailand	A/Thailand/CU_B4/2009	agtgatgccccattccttgatcggcttcgccgagatcag aggtccctaaggggaagaggcaatactc	-	-
CY061902	Human H3N2 IAVs	Human	H3N2		2009	Australia	A/Australia/19/2009	agtgatgccccattccttgatcggcttcgccgagatcag aggtccctaaggggaagaggcaatactc	-	-
CY073761	Human H3N2 IAVs	Human	H3N2		2009	USA	A/California/VRDL363/2009	agtgatgccccattccttgatcggcttcgccgagatcag aggtccctaaggggaagaggcaatactc	-	-
CY080479	Human H3N2 IAVs	Human	H3N2		2009	USA	A/New_York/3519/2009	agtgatgccccattccttgatcggcttcgccgagatcag aggtccctaaggggaagaggcaatactc	-	-
CY106596	Human H3N2 IAVs	Human	H3N2		2009	Hong_Kong	A/Hong_Kong/H090_671_V10/2009	agtgatgccccattccttgatcggcttcgccgagatcag aggtccctaaggggaagaggcaatactc	-	-
CY115484	Human H3N2 IAVs	Human	H3N2		2009	Hong_Kong	A/Hong_Kong/H090_671_V10/2009	agtgatgccccattccttgatcggcttcgccgagatcag aggtccctaaggggaagaggcaatactc	-	-
CY106708	Human H3N2 IAVs	Human	H3N2		2009	Hong_Kong	A/Hong_Kong/H090_720_V10/2009	agtgatgccccattccttgatcggcttcgccgagatcag aggtccctaaggggaagaggcaatactc	-	-
CY115588	Human H3N2 IAVs	Human	H3N2		2009	Hong_Kong	A/Hong_Kong/H090_720_V10/2009	agtgatgccccattccttgatcggcttcgccgagatcag aggtccctaaggggaagaggcaatactc	-	-
CY115596	Human H3N2 IAVs	Human	H3N2		2009	Hong_Kong	A/Hong_Kong/H090_720_V20/2009	agtgatgccccattccttgatcggcttcgccgagatcag aggtccctaaggggaagaggcaatactc	-	-
CY106716	Human H3N2 IAVs	Human	H3N2		2009	Hong_Kong	A/Hong_Kong/H090_720_V20/2009	agtgatgccccattccttgatcggcttcgccgagatcag aggtccctaaggggaagaggcaatactc	-	-
CY115604	Human H3N2 IAVs	Human	H3N2		2009	Hong_Kong	A/Hong_Kong/H090_720_V21/2009	agtgatgccccattccttgatcggcttcgccgagatcag aggtccctaaggggaagaggcaatactc	-	-
CY106724	Human H3N2 IAVs	Human	H3N2		2009	Hong_Kong	A/Hong_Kong/H090_720_V21/2009	agtgatgccccattccttgatcggcttcgccgagatcag aggtccctaaggggaagaggcaatactc	-	-
CY106732	Human H3N2 IAVs	Human	H3N2		2009	Hong_Kong	A/Hong_Kong/H090_720_V31/2009	agtgatgccccattccttgatcggcttcgccgagatcag aggtccctaaggggaagaggcaatactc	-	-
CY115612	Human H3N2 IAVs	Human	H3N2		2009	Hong_Kong	A/Hong_Kong/H090_720_V31/2009	agtgatgccccattccttgatcggcttcgccgagatcag aggtccctaaggggaagaggcaatactc	-	-
CY115564	Human H3N2 IAVs	Human	H3N2		2009	Hong_Kong	A/Hong_Kong/H090_707_V10/2009	agtgatgccccattccttgatcggcttcgccgagatcag aggtccctaaggggaagaggcaatactc	-	-

CY106684	Human H3N2 IAVs	Human	H3N2		2009	Hong_Kong	A/Hong_Kong/H090_707_V10/2009	agtgatgccccattccttgatcggcttcgccgagatcag aggtccctaaggggaagaggcaatactc	-	-
CY106988	Human H3N2 IAVs	Human	H3N2		2009	Hong_Kong	A/Hong_Kong/H090_781_V10/2009	agtgatgccccattccttgatcggcttcgccgagatcag aggtccctaaggggaagaggcaatactc	-	-
CY115460	Human H3N2 IAVs	Human	Mixed		2009	Hong_Kong	A/Hong_Kong/H090_781_V10/2009	agtgatgccccattccttgatcggcttcgccgagatcag aggtccctaaggggaagaggcaatactc	-	-
CY093275	Human H3N2 IAVs	Human	H3N2		2009	Iraq	A/Iraq/WRAIR1244P/2009	agtgatgccccattccttgatcggcttcgccgagatcag aggtccctaaggggaagaggcaatactc	-	-
CY093307	Human H3N2 IAVs	Human	H3N2		2009	Iraq	A/Iraq/WRAIR1252P/2009	agtgatgccccattccttgatcggcttcgccgagatcag aggtccctaaggggaagaggcaatactc	-	-
CY068453	Human H3N2 IAVs	Human	H3N2		2009	USA	A/California/VRDL294/2009	agtgatgccccattccttgatcggcttcgccgagatcag aggtccctaaggggaagaggcaatactc	-	-
CY069449	Human H3N2 IAVs	Human	H3N2		2009	Kuwait	A/Kuwait/WRAIR1143P/2009	agtgatgccccattccttgatcggcttcgccgagatcag aggtccctaaggggaagaggcaatactc	-	-
CY069425	Human H3N2 IAVs	Human	H3N2		2009	USA	A/New_Mexico/WRAIR1139P/2009	agtgatgccccattccttgatcggcttcgccgagatcag aggtccctaaggggaagaggcaatactc	-	-
CY066515	Human H3N2 IAVs	Human	H3N2		2009	Australia	A/Australia/18/2009	agtgatgccccattccttgatcggcttcgccgagatcag aggtccctaaggggaagaggcaatactc	-	-
CY061894	Human H3N2 IAVs	Human	H3N2		2009	Australia	A/Australia/2/2009	agtgatgccccattccttgatcggcttcgccgagatcag aggtccctaaggggaagaggcaatactc	-	-
CY080543	Human H3N2 IAVs	Human	H3N2		2009	Australia	A/Australia/22/2009	agtgatgccccattccttgatcggcttcgccgagatcag aggtccctaaggggaagaggcaatactc	-	-
CY080559	Human H3N2 IAVs	Human	H3N2		2009	Australia	A/Australia/34/2009	agtgatgccccattccttgatcggcttcgccgagatcag aggtccctaaggggaagaggcaatactc	-	-
JF316723	Human H3N2 IAVs	Human	H3N2		2009	China	A/Beijing/080302/2009	agtgatgccccattccttgatcggcttcgccgagatcag aggtccctaaggggaagaggcaatactc	-	-
CY068141	Human H3N2 IAVs	Human	H3N2		2009	USA	A/California/VRDL159/2009	agtgatgccccattccttgatcggcttcgccgagatcag aggtccctaaggggaagaggcaatactc	-	-
CY068221	Human H3N2 IAVs	Human	H3N2		2009	USA	A/California/VRDL173/2009	agtgatgccccattccttgatcggcttcgccgagatcag aggtccctaaggggaagaggcaatactc	-	-
CY068690	Human H3N2 IAVs	Human	H3N2		2009	USA	A/California/VRDL181/2009	agtgatgccccattccttgatcggcttcgccgagatcag aggtccctaaggggaagaggcaatactc	-	-
CY068770	Human H3N2 IAVs	Human	H3N2		2009	USA	A/California/VRDL204/2009	agtgatgccccattccttgatcggcttcgccgagatcag aggtccctaaggggaagaggcaatactc	-	-
CY068810	Human H3N2 IAVs	Human	H3N2		2009	USA	A/California/VRDL246/2009	agtgatgccccattccttgatcggcttcgccgagatcag aggtccctaaggggaagaggcaatactc	-	-
CY068357	Human H3N2 IAVs	Human	H3N2		2009	USA	A/California/VRDL263/2009	agtgatgccccattccttgatcggcttcgccgagatcag aggtccctaaggggaagaggcaatactc	-	-
CY067949	Human H3N2 IAVs	Human	H3N2		2009	USA	A/California/VRDL287/2009	agtgatgccccattccttgatcggcttcgccgagatcag aggtccctaaggggaagaggcaatactc	-	-
CY067957	Human H3N2 IAVs	Human	H3N2		2009	USA	A/California/VRDL288/2009	agtgatgccccattccttgatcggcttcgccgagatcag aggtccctaaggggaagaggcaatactc	-	-
CY067217	Human H3N2 IAVs	Human	H3N2		2009	USA	A/California/VRDL296/2009	agtgatgccccattccttgatcggcttcgccgagatcag aggtccctaaggggaagaggcaatactc	-	-
CY068461	Human H3N2 IAVs	Human	H3N2		2009	USA	A/California/VRDL305/2009	agtgatgccccattccttgatcggcttcgccgagatcag aggtccctaaggggaagaggcaatactc	-	-
CY067249	Human H3N2 IAVs	Human	H3N2		2009	USA	A/California/VRDL314/2009	agtgatgccccattccttgatcggcttcgccgagatcag aggtccctaaggggaagaggcaatactc	-	-
CY067973	Human H3N2 IAVs	Human	H3N2		2009	USA	A/California/VRDL339/2009	agtgatgccccattccttgatcggcttcgccgagatcag aggtccctaaggggaagaggcaatactc	-	-

CY068061	Human H3N2 IAVs	Human	H3N2		2009	USA	A/California/VRDL357/2009	agtgatgccccattccttgatcggcttcgccgagatcag aggtccctaaggggaagaggcaatactc	-	-
CY068093	Human H3N2 IAVs	Human	H3N2		2009	USA	A/California/VRDL367/2009	agtgatgccccattccttgatcggcttcgccgagatcag aggtccctaaggggaagaggcaatactc	-	-
CY068117	Human H3N2 IAVs	Human	H3N2		2009	USA	A/California/VRDL374/2009	agtgatgccccattccttgatcggcttcgccgagatcag aggtccctaaggggaagaggcaatactc	-	-
CY068125	Human H3N2 IAVs	Human	H3N2		2009	USA	A/California/VRDL375/2009	agtgatgccccattccttgatcggcttcgccgagatcag aggtccctaaggggaagaggcaatactc	-	-
CY068858	Human H3N2 IAVs	Human	H3N2		2009	USA	A/California/VRDL390/2009	agtgatgccccattccttgatcggcttcgccgagatcag aggtccctaaggggaagaggcaatactc	-	-
CY092807	Human H3N2 IAVs	Human	mixed		2009	USA	A/California/VRDL395/2009	agtgatgccccattccttgatcggcttcgccgagatcag aggtccctaaggggaagaggcaatactc	-	-
CY068133	Human H3N2 IAVs	Human	H3N2		2009	USA	A/California/VRDL396/2009	agtgatgccccattccttgatcggcttcgccgagatcag aggtccctaaggggaagaggcaatactc	-	-
CY069305	Human H3N2 IAVs	Human	H3N2		2009	Senegal	A/Dakar/WRAIR0011T/2009	agtgatgccccattccttgatcggcttcgccgagatcag aggtccctaaggggaagaggcaatactc	-	-
CY093220	Human H3N2 IAVs	Human	H3N2		2009	Senegal	A/Dakar/WRAIR0013N/2009	agtgatgccccattccttgatcggcttcgccgagatcag aggtccctaaggggaagaggcaatactc	-	-
CY106580	Human H3N2 IAVs	Human	H3N2		2009	Hong_Kong	A/Hong_Kong/H090_662_V10/2009	agtgatgccccattccttgatcggcttcgccgagatcag aggtccctaaggggaagaggcaatactc	-	-
CY115516	Human H3N2 IAVs	Human	H3N2		2009	Hong_Kong	A/Hong_Kong/H090_689_V10/2009	agtgatgccccattccttgatcggcttcgccgagatcag aggtccctaaggggaagaggcaatactc	-	-
CY106628	Human H3N2 IAVs	Human	H3N2		2009	Hong_Kong	A/Hong_Kong/H090_689_V10/2009	agtgatgccccattccttgatcggcttcgccgagatcag aggtccctaaggggaagaggcaatactc	-	-
CY115540	Human H3N2 IAVs	Human	H3N2		2009	Hong_Kong	A/Hong_Kong/H090_692_V10/2009	agtgatgccccattccttgatcggcttcgccgagatcag aggtccctaaggggaagaggcaatactc	-	-
CY115620	Human H3N2 IAVs	Human	H3N2		2009	Hong_Kong	A/Hong_Kong/H090_724_V10/2009	agtgatgccccattccttgatcggcttcgccgagatcag aggtccctaaggggaagaggcaatactc	-	-
CY106748	Human H3N2 IAVs	Human	H3N2		2009	Hong_Kong	A/Hong_Kong/H090_724_V20/2009	agtgatgccccattccttgatcggcttcgccgagatcag aggtccctaaggggaagaggcaatactc	-	-
CY106900	Human H3N2 IAVs	Human	H3N2		2009	Hong_Kong	A/Hong_Kong/H090_750_V10/2009	agtgatgccccattccttgatcggcttcgccgagatcag aggtccctaaggggaagaggcaatactc	-	-
CY106996	Human H3N2 IAVs	Human	H3N2		2009	Hong_Kong	A/Hong_Kong/H090_783_V10/2009	agtgatgccccattccttgatcggcttcgccgagatcag aggtccctaaggggaagaggcaatactc	-	-
CY173555	Human H3N2 IAVs	Human	H3N2		2009	USA	A/New_York/1193/2009	agtgatgccccattccttgatcggcttcgccgagatcag aggtccctaaggggaagaggcaatactc	-	-
CY050584	Human H3N2 IAVs	Human	H3N2		2009	USA	A/New_York/3135/2009	agtgatgccccattccttgatcggcttcgccgagatcag aggtccctaaggggaagaggcaatactc	-	-
CY050736	Human H3N2 IAVs	Human	H3N2		2009	USA	A/New_York/3309/2009	agtgatgccccattccttgatcggcttcgccgagatcag aggtccctaaggggaagaggcaatactc	-	-
CY050816	Human H3N2 IAVs	Human	H3N2		2009	USA	A/New_York/3536/2009	agtgatgccccattccttgatcggcttcgccgagatcag aggtccctaaggggaagaggcaatactc	-	-
CY058800	Human H3N2 IAVs	Human	H3N2		2009	USA	A/New_York/3626/2009	agtgatgccccattccttgatcggcttcgccgagatcag aggtccctaaggggaagaggcaatactc	-	-
CY081045	Human H3N2 IAVs	Human	mixed		2009	USA	A/New_York/3647/2009	agtgatgccccattccttgatcggcttcgccgagatcag aggtccctaaggggaagaggcaatactc	-	-
KF014732	Human H3N2 IAVs	Human	H3N2		2009	Singapore	A/Singapore/C2009.485b/2009	agtgatgccccattccttgatcggcttcgccgagatcag aggtccctaaggggaagaggcaatactc	-	-
KF014660	Human H3N2 IAVs	Human	H3N2		2009	Singapore	A/Singapore/C2009.485b/2009	agtgatgccccattccttgatcggcttcgccgagatcag aggtccctaaggggaagaggcaatactc	-	-

KF014733	Human H3N2 IAVs	Human	H3N2		2009	Singapore	A/Singapore/C2009.515/2009	agtgatgccccattccttgatcggcttcgccgagatcag aggtccctaaggggaagaggcaatactc	-	-
KF014661	Human H3N2 IAVs	Human	H3N2		2009	Singapore	A/Singapore/C2009.515/2009	agtgatgccccattccttgatcggcttcgccgagatcag aggtccctaaggggaagaggcaatactc	-	-
JX437769	Human H3N2 IAVs	Human	H3N2		2009	Singapore	A/Singapore/C2009.518V/2009	agtgatgccccattccttgatcggcttcgccgagatcag aggtccctaaggggaagaggcaatactc	-	-
KM070035	Human H3N2 IAVs	Human	H3N2		2009	Singapore	A/Singapore/C2009.677/2009	agtgatgccccattccttgatcggcttcgccgagatcag aggtccctaaggggaagaggcaatactc	-	-
JX437770	Human H3N2 IAVs	Human	H3N2		2009	Singapore	A/Singapore/C2009.715V/2009	agtgatgccccattccttgatcggcttcgccgagatcag aggtccctaaggggaagaggcaatactc	-	-
KM070039	Human H3N2 IAVs	Human	H3N2		2009	Singapore	A/Singapore/C2009.803a/2009	agtgatgccccattccttgatcggcttcgccgagatcag aggtccctaaggggaagaggcaatactc	-	-
JX437771	Human H3N2 IAVs	Human	H3N2		2009	Singapore	A/Singapore/C2009.803bV/2009	agtgatgccccattccttgatcggcttcgccgagatcag aggtccctaaggggaagaggcaatactc	-	-
JX437888	Human H3N2 IAVs	Human	H3N2		2009	Singapore	A/Singapore/H2009.332C/2009	agtgatgccccattccttgatcggcttcgccgagatcag aggtccctaaggggaagaggcaatactc	-	-
KF014687	Human H3N2 IAVs	Human	H3N2		2009	Singapore	A/Singapore/H2009.332C/2009	agtgatgccccattccttgatcggcttcgccgagatcag aggtccctaaggggaagaggcaatactc	-	-
KF014761	Human H3N2 IAVs	Human	H3N2		2009	Singapore	A/Singapore/H2009.389b/2009	agtgatgccccattccttgatcggcttcgccgagatcag aggtccctaaggggaagaggcaatactc	-	-
KF014685	Human H3N2 IAVs	Human	H3N2		2009	Singapore	A/Singapore/H2009.389b/2009	agtgatgccccattccttgatcggcttcgccgagatcag aggtccctaaggggaagaggcaatactc	-	-
KF014763	Human H3N2 IAVs	Human	H3N2		2009	Singapore	A/Singapore/H2009.510/2009	agtgatgccccattccttgatcggcttcgccgagatcag aggtccctaaggggaagaggcaatactc	-	-
KF014690	Human H3N2 IAVs	Human	H3N2		2009	Singapore	A/Singapore/H2009.510/2009	agtgatgccccattccttgatcggcttcgccgagatcag aggtccctaaggggaagaggcaatactc	-	-
KF014691	Human H3N2 IAVs	Human	H3N2		2009	Singapore	A/Singapore/H2009.518/2009	agtgatgccccattccttgatcggcttcgccgagatcag aggtccctaaggggaagaggcaatactc	-	-
CY121064	Human H3N2 IAVs	Human	H3N2		2009	China	A/Guangdong_Luohu/1256/2009	agtgatgccccattccttgatcggcttcgccgagatcag aggtccctaaggggaagaggcaatactc	-	-
CY115468	Human H3N2 IAVs	Human	H3N2		2009	Hong_Kong	A/Hong_Kong/H090_662_V10/2009	agtgatgccccattccttgatcggcttcgccgagatcag aggtccctaaggggaagaggcaatactc	-	-
CY106604	Human H3N2 IAVs	Human	H3N2		2009	Hong_Kong	A/Hong_Kong/H090_672_V10/2009	agtgatgccccattccttgatcggcttcgccgagatcag aggtccctaaggggaagaggcaatactc	-	-
CY115492	Human H3N2 IAVs	Human	H3N2		2009	Hong_Kong	A/Hong_Kong/H090_672_V10/2009	agtgatgccccattccttgatcggcttcgccgagatcag aggtccctaaggggaagaggcaatactc	-	-
CY115508	Human H3N2 IAVs	Human	H3N2		2009	Hong_Kong	A/Hong_Kong/H090_688_V10/2009	agtgatgccccattccttgatcggcttcgccgagatcag aggtccctaaggggaagaggcaatactc	-	-
CY106620	Human H3N2 IAVs	Human	H3N2		2009	Hong_Kong	A/Hong_Kong/H090_688_V10/2009	agtgatgccccattccttgatcggcttcgccgagatcag aggtccctaaggggaagaggcaatactc	-	-
CY115524	Human H3N2 IAVs	Human	H3N2		2009	Hong_Kong	A/Hong_Kong/H090_689_V20/2009	agtgatgccccattccttgatcggcttcgccgagatcag aggtccctaaggggaagaggcaatactc	-	-
CY106636	Human H3N2 IAVs	Human	H3N2		2009	Hong_Kong	A/Hong_Kong/H090_689_V20/2009	agtgatgccccattccttgatcggcttcgccgagatcag aggtccctaaggggaagaggcaatactc	-	-
CY115532	Human H3N2 IAVs	Human	H3N2		2009	Hong_Kong	A/Hong_Kong/H090_689_V22/2009	agtgatgccccattccttgatcggcttcgccgagatcag aggtccctaaggggaagaggcaatactc	-	-
CY106644	Human H3N2 IAVs	Human	H3N2		2009	Hong_Kong	A/Hong_Kong/H090_689_V22/2009	agtgatgccccattccttgatcggcttcgccgagatcag aggtccctaaggggaagaggcaatactc	-	-
CY106652	Human H3N2 IAVs	Human	H3N2		2009	Hong_Kong	A/Hong_Kong/H090_692_V10/2009	agtgatgccccattccttgatcggcttcgccgagatcag aggtccctaaggggaagaggcaatactc	-	-

CY106660	Human H3N2 IAVs	Human	H3N2		2009	Hong_Kong	A/Hong_Kong/H090_692_V12/2009	agtgatgccccattccttgatcggcttcgccgagatcagaggtccctaaggggaagaggcaatactc	-	-
CY115548	Human H3N2 IAVs	Human	H3N2		2009	Hong_Kong	A/Hong_Kong/H090_695_V10/2009	agtgatgccccattccttgatcggcttcgccgagatcagaggtccctaaggggaagaggcaatactc	-	-
CY106668	Human H3N2 IAVs	Human	H3N2		2009	Hong_Kong	A/Hong_Kong/H090_695_V10/2009	agtgatgccccattccttgatcggcttcgccgagatcagaggtccctaaggggaagaggcaatactc	-	-
CY106676	Human H3N2 IAVs	Human	H3N2		2009	Hong_Kong	A/Hong_Kong/H090_703_V10/2009	agtgatgccccattccttgatcggcttcgccgagatcagaggtccctaaggggaagaggcaatactc	-	-
CY115556	Human H3N2 IAVs	Human	H3N2		2009	Hong_Kong	A/Hong_Kong/H090_703_V10/2009	agtgatgccccattccttgatcggcttcgccgagatcagaggtccctaaggggaagaggcaatactc	-	-
CY115580	Human H3N2 IAVs	Human	H3N2		2009	Hong_Kong	A/Hong_Kong/H090_714_V10/2009	agtgatgccccattccttgatcggcttcgccgagatcagaggtccctaaggggaagaggcaatactc	-	-
CY106700	Human H3N2 IAVs	Human	H3N2		2009	Hong_Kong	A/Hong_Kong/H090_714_V10/2009	agtgatgccccattccttgatcggcttcgccgagatcagaggtccctaaggggaagaggcaatactc	-	-
CY106740	Human H3N2 IAVs	Human	H3N2		2009	Hong_Kong	A/Hong_Kong/H090_724_V10/2009	agtgatgccccattccttgatcggcttcgccgagatcagaggtccctaaggggaagaggcaatactc	-	-
CY115628	Human H3N2 IAVs	Human	H3N2		2009	Hong_Kong	A/Hong_Kong/H090_724_V20/2009	agtgatgccccattccttgatcggcttcgccgagatcagaggtccctaaggggaagaggcaatactc	-	-
CY106764	Human H3N2 IAVs	Human	H3N2		2009	Hong_Kong	A/Hong_Kong/H090_726_V10/2009	agtgatgccccattccttgatcggcttcgccgagatcagaggtccctaaggggaagaggcaatactc	-	-
CY115644	Human H3N2 IAVs	Human	H3N2		2009	Hong_Kong	A/Hong_Kong/H090_726_V10/2009	agtgatgccccattccttgatcggcttcgccgagatcagaggtccctaaggggaagaggcaatactc	-	-
CY106772	Human H3N2 IAVs	Human	H3N2		2009	Hong_Kong	A/Hong_Kong/H090_727_V10/2009	agtgatgccccattccttgatcggcttcgccgagatcagaggtccctaaggggaagaggcaatactc	-	-
CY106780	Human H3N2 IAVs	Human	H3N2		2009	Hong_Kong	A/Hong_Kong/H090_729_V10/2009	agtgatgccccattccttgatcggcttcgccgagatcagaggtccctaaggggaagaggcaatactc	-	-
CY115652	Human H3N2 IAVs	Human	H3N2		2009	Hong_Kong	A/Hong_Kong/H090_729_V10/2009	agtgatgccccattccttgatcggcttcgccgagatcagaggtccctaaggggaagaggcaatactc	-	-
CY106820	Human H3N2 IAVs	Human	H3N2		2009	Hong_Kong	A/Hong_Kong/H090_736_V10/2009	agtgatgccccattccttgatcggcttcgccgagatcagaggtccctaaggggaagaggcaatactc	-	-
CY115692	Human H3N2 IAVs	Human	H3N2		2009	Hong_Kong	A/Hong_Kong/H090_736_V10/2009	agtgatgccccattccttgatcggcttcgccgagatcagaggtccctaaggggaagaggcaatactc	-	-
CY106828	Human H3N2 IAVs	Human	H3N2		2009	Hong_Kong	A/Hong_Kong/H090_737_V10/2009	agtgatgccccattccttgatcggcttcgccgagatcagaggtccctaaggggaagaggcaatactc	-	-
CY115700	Human H3N2 IAVs	Human	H3N2		2009	Hong_Kong	A/Hong_Kong/H090_737_V10/2009	agtgatgccccattccttgatcggcttcgccgagatcagaggtccctaaggggaagaggcaatactc	-	-
CY115740	Human H3N2 IAVs	Human	H3N2		2009	Hong_Kong	A/Hong_Kong/H090_741_V10/2009	agtgatgccccattccttgatcggcttcgccgagatcagaggtccctaaggggaagaggcaatactc	-	-
CY106868	Human H3N2 IAVs	Human	H3N2		2009	Hong_Kong	A/Hong_Kong/H090_741_V10/2009	agtgatgccccattccttgatcggcttcgccgagatcagaggtccctaaggggaagaggcaatactc	-	-
CY115764	Human H3N2 IAVs	Human	H3N2		2009	Hong_Kong	A/Hong_Kong/H090_750_V10/2009	agtgatgccccattccttgatcggcttcgccgagatcagaggtccctaaggggaagaggcaatactc	-	-
CY106916	Human H3N2 IAVs	Human	H3N2		2009	Hong_Kong	A/Hong_Kong/H090_755_V20/2009	agtgatgccccattccttgatcggcttcgccgagatcagaggtccctaaggggaagaggcaatactc	-	-
CY115780	Human H3N2 IAVs	Human	H3N2		2009	Hong_Kong	A/Hong_Kong/H090_755_V31/2009	agtgatgccccattccttgatcggcttcgccgagatcagaggtccctaaggggaagaggcaatactc	-	-
CY106924	Human H3N2 IAVs	Human	H3N2		2009	Hong_Kong	A/Hong_Kong/H090_755_V31/2009	agtgatgccccattccttgatcggcttcgccgagatcagaggtccctaaggggaagaggcaatactc	-	-
CY084434	Human H3N2 IAVs	Human	mixed		2009	USA	A/New_York/3305/2009	agtgatgccccattccttgatcggcttcgccgagatcagaggtccctaaggggaagaggcaatactc	-	-

KF014764	Human H3N2 IAVs	Human	H3N2		2009	Singapore	A/Singapore/H2009.518/2009	agtgatgccccattccttgatcggcttcgccgagatcag aggtccctaaggggaagaggcaatactc	-	-
KF014692	Human H3N2 IAVs	Human	H3N2		2009	Singapore	A/Singapore/H2009.679/2009	agtgatgccccattccttgatcggcttcgccgagatcag aggtccctaaggggaagaggcaatactc	-	-
KF014765	Human H3N2 IAVs	Human	H3N2		2009	Singapore	A/Singapore/H2009.679/2009	agtgatgccccattccttgatcggcttcgccgagatcag aggtccctaaggggaagaggcaatactc	-	-
KM070022	Human H3N2 IAVs	Human	H3N2		2009	Singapore	A/Singapore/S2009.329/2009	agtgatgccccattccttgatcggcttcgccgagatcag aggtccctaaggggaagaggcaatactc	-	-
KM070024	Human H3N2 IAVs	Human	H3N2		2009	Singapore	A/Singapore/S2009.359/2009	agtgatgccccattccttgatcggcttcgccgagatcag aggtccctaaggggaagaggcaatactc	-	-
KM070025	Human H3N2 IAVs	Human	H3N2		2009	Singapore	A/Singapore/S2009.362b/2009	agtgatgccccattccttgatcggcttcgccgagatcag aggtccctaaggggaagaggcaatactc	-	-
KM070026	Human H3N2 IAVs	Human	H3N2		2009	Singapore	A/Singapore/S2009.362c/2009	agtgatgccccattccttgatcggcttcgccgagatcag aggtccctaaggggaagaggcaatactc	-	-
CY121081	Human H3N2 IAVs	Human	H3N2		2009	Australia	A/Victoria/210/2009	agtgatgccccattccttgatcggcttcgccgagatcag aggtccctaaggggaagaggcaatactc	-	-
CY050808	Human H3N2 IAVs	Human	H3N2		2009	USA	A/New_York/3542/2009	agtgatgccccattccttgatcggcttcgccgagatcag aggtccctaaggggaagaggcaatactc	-	-
CY093251	Human H3N2 IAVs	Human	H3N2		2009	USA	A/Texas/WRAIR1239P/2009	agtgatgccccattccttgatcggcttcgccgagatcag aggtccctaaggggaagaggcaatactc	-	-
CY069353	Human H3N2 IAVs	Human	H3N2		2009	USA	A/Washington/WRAIR1057P/2009	agtgatgccccattccttgatcggcttcgccgagatcag aggtccctaaggggaagaggcaatactc	-	-
CY074946	Human H3N2 IAVs	Human	H3N2		2009	Thailand	A/Thailand/CU_H1071/2009	agtgatgccccattccttgatcggcttcgccgagatcag aggtccctaaggggaagaggcaatactc	-	-
GQ902821	Human H3N2 IAVs	Human	H3N2		2009	Thailand	A/Thailand/CU_B590/2009	agtgatgccccattccttgatcggcttcgccgagatcag aggtccctaaggggaagaggcaatactc	-	-
CY068413	Human H3N2 IAVs	Human	H3N2		2009	USA	A/California/VRDL277/2009	agtgatgccccattccttgatcggcttcgccgagatcag aggtccctaaggggaagaggcaatactc	-	-
CY050696	Human H3N2 IAVs	Human	H3N2		2009	USA	A/New_York/3274/2009	agtgatgccccattccttgatcggcttcgccgagatcag aggtccctaaggggaagaggcaatactc	-	-
CY106964	Human H3N2 IAVs	Human	H3N2		2009	Hong_Kong	A/Hong_Kong/H090_768_V10/2009	agtgatgccccattccttgatcggcttcgccgagatcag aggtccctaaggggaagaggcaatactc	-	-
GQ983552	Human H3N2 IAVs	Human	H3N2		2009	Thailand	A/Thailand/CU_B106/2009	agtgatgccccattccttgatcggcttcgccgagatcag aggtccctaaggggaagaggcaatactc	-	-
CY106788	Human H3N2 IAVs	Human	H3N2		2009	Hong_Kong	A/Hong_Kong/H090_733_V10/2009	agtgatgccccattccttgatcggcttcgccgagatcag aggtccctaaggggaagaggcaatactc	-	-
CY115660	Human H3N2 IAVs	Human	H3N2		2009	Hong_Kong	A/Hong_Kong/H090_733_V10/2009	agtgatgccccattccttgatcggcttcgccgagatcag aggtccctaaggggaagaggcaatactc	-	-
JX437889	Human H3N2 IAVs	Human	H3N2		2009	Singapore	A/Singapore/H2009.334C/2009	agtgatgccccattccttgatcggcttcgccgagatcag aggtccctaaggggaagaggcaatactc	-	-
KF014686	Human H3N2 IAVs	Human	H3N2		2009	Singapore	A/Singapore/H2009.334C/2009	agtgatgccccattccttgatcggcttcgccgagatcag aggtccctaaggggaagaggcaatactc	-	-
CY092381	Human H3N2 IAVs	Human	H3N2		2009	USA	A/California/VRDL257/2009	agtgatgccccattccttgatcggcttcgccgagatcag aggtccctaaggggaagaggcaatactc	-	-
CY106908	Human H3N2 IAVs	Human	H3N2		2009	Hong_Kong	A/Hong_Kong/H090_752_V10/2009	agtgatgccccattccttgatcggcttcgccgagatcag aggtccctaaggggaagaggcaatactc	-	-
CY115772	Human H3N2 IAVs	Human	H3N2		2009	Hong_Kong	A/Hong_Kong/H090_752_V10/2009	agtgatgccccattccttgatcggcttcgccgagatcag aggtccctaaggggaagaggcaatactc	-	-
CY106956	Human H3N2 IAVs	Human	H3N2		2009	Hong_Kong	A/Hong_Kong/H090_764_V10/2009	agtgatgccccattccttgatcggcttcgccgagatcag aggtccctaaggggaagaggcaatactc	-	-

CY115812	Human H3N2 IAVs	Human	H3N2		2009	Hong_Kong	A/Hong_Kong/H090_764_V10/2009	agtgatgccccattccttgatcggcttcgccgagatcagaggtccctaaggggaagaggcaatactc	-	-
CY050624	Human H3N2 IAVs	Human	H3N2		2009	USA	A/New_York/3137/2009	agtgatgccccattccttgatcggcttcgccgagatcagaggtccctaaggggaagaggcaatactc	-	-
CY106796	Human H3N2 IAVs	Human	H3N2		2009	Hong_Kong	A/Hong_Kong/H090_734_V10/2009	agtgatgccccattccttgatcggcttcgccgagatcagaggtccctaaggggaagaggcaatactc	-	-
CY115668	Human H3N2 IAVs	Human	H3N2		2009	Hong_Kong	A/Hong_Kong/H090_734_V10/2009	agtgatgccccattccttgatcggcttcgccgagatcagaggtccctaaggggaagaggcaatactc	-	-
CY106804	Human H3N2 IAVs	Human	H3N2		2009	Hong_Kong	A/Hong_Kong/H090_734_V20/2009	agtgatgccccattccttgatcggcttcgccgagatcagaggtccctaaggggaagaggcaatactc	-	-
CY115676	Human H3N2 IAVs	Human	H3N2		2009	Hong_Kong	A/Hong_Kong/H090_734_V20/2009	agtgatgccccattccttgatcggcttcgccgagatcagaggtccctaaggggaagaggcaatactc	-	-
CY115684	Human H3N2 IAVs	Human	H3N2		2009	Hong_Kong	A/Hong_Kong/H090_734_V32/2009	agtgatgccccattccttgatcggcttcgccgagatcagaggtccctaaggggaagaggcaatactc	-	-
CY106812	Human H3N2 IAVs	Human	H3N2		2009	Hong_Kong	A/Hong_Kong/H090_734_V32/2009	agtgatgccccattccttgatcggcttcgccgagatcagaggtccctaaggggaagaggcaatactc	-	-
CY093259	Human H3N2 IAVs	Human	H3N2		2009	USA	A/Florida/WRAIR1241P/2009	agtgatgccccattccttgatcggcttcgccgagatcagaggtccctaaggggaagaggcaatactc	-	-
CY069329	Human H3N2 IAVs	Human	H3N2		2009	South_Korea	A/Korea/WRAIR1038P/2009	agtgatgccccattccttgatcggcttcgccgagatcagaggtccctaaggggaagaggcaatactc	-	-
CY115636	Human H3N2 IAVs	Human	H3N2		2009	Hong_Kong	A/Hong_Kong/H090_725_V10/2009	agtgatgccccattccttgatcggcttcgccgagatcagaggtccctaaggggaagaggcaatactc	-	-
CY106756	Human H3N2 IAVs	Human	H3N2		2009	Hong_Kong	A/Hong_Kong/H090_725_V10/2009	agtgatgccccattccttgatcggcttcgccgagatcagaggtccctaaggggaagaggcaatactc	-	-
JF316726	Human H3N2 IAVs	Human	H3N2		2009	China	A/Beijing/073103/2009	agtgatgccccattccttgatcggcttcgccgagatcagaggtccctaaggggaagaggcaatactc	-	-
GU271994	Human H3N2 IAVs	Human	H3N2		2009	Thailand	A/Thailand/CU_H16/2009	agtgatgccccattccttgatcggcttcgccgagatcagaggtccctaaggggaagaggcaatactc	-	-
CY068722	Human H3N2 IAVs	Human	H3N2		2009	USA	A/California/VRDL187/2009	agtgatgccccattccttgatcggcttcgccgagatcagaggtccctaaggggaagaggcaatactc	-	-
CY068269	Human H3N2 IAVs	Human	H3N2		2009	USA	A/California/VRDL231/2009	agtgatgccccattccttgatcggcttcgccgagatcagaggtccctaaggggaagaggcaatactc	-	-
CY068285	Human H3N2 IAVs	Human	H3N2		2009	USA	A/California/VRDL243/2009	agtgatgccccattccttgatcggcttcgccgagatcagaggtccctaaggggaagaggcaatactc	-	-
CY077429	Human H3N2 IAVs	Human	H3N2		2009	USA	A/California/VRDL356/2009	agtgatgccccattccttgatcggcttcgccgagatcagaggtccctaaggggaagaggcaatactc	-	-
CY067201	Human H3N2 IAVs	Human	H3N2		2009	USA	A/California/VRDL241/2009	agtgatgccccattccttgatcggcttcgccgagatcagaggtccctaaggggaagaggcaatactc	-	-
CY121046	Human H3N2 IAVs	Human	H3N2		2009	USA	A/Wisconsin/15/2009	agtgatgccccattccttgatcggcttcgccgagatcagaggtccctaaggggaagaggcaatactc	-	-
KF014658	Human H3N2 IAVs	Human	H3N2		2009	Singapore	A/Singapore/C2009.496/2009	agtgatgccccattccttgatcggcttcgccgagatcagaggtccctaaggggaagaggcaatactc	-	-
KF014729	Human H3N2 IAVs	Human	H3N2		2009	Singapore	A/Singapore/C2009.496/2009	agtgatgccccattccttgatcggcttcgccgagatcagaggtccctaaggggaagaggcaatactc	-	-
CY064867	Human H3N2 IAVs	Human	H3N2		2009	USA	A/California/VRDL145/2009	agtgatgccccattccttgatcggcttcgccgagatcagaggtccctaaggggaagaggcaatactc	-	-
CY068173	Human H3N2 IAVs	Human	H3N2		2009	USA	A/California/VRDL165/2009	agtgatgccccattccttgatcggcttcgccgagatcagaggtccctaaggggaagaggcaatactc	-	-
CY068706	Human H3N2 IAVs	Human	H3N2		2009	USA	A/California/VRDL184/2009	agtgatgccccattccttgatcggcttcgccgagatcagaggtccctaaggggaagaggcaatactc	-	-

CY068301	Human H3N2 IAVs	Human	H3N2		2009	USA	A/California/VRDL248/2009	agtgatgccccattccttgatcggcttcgccgagatcag aggtccctaaggggaagaggcaatactc	-	-
CY068333	Human H3N2 IAVs	Human	H3N2		2009	USA	A/California/VRDL260/2009	agtgatgccccattccttgatcggcttcgccgagatcag aggtccctaaggggaagaggcaatactc	-	-
CY068397	Human H3N2 IAVs	Human	H3N2		2009	USA	A/California/VRDL272/2009	agtgatgccccattccttgatcggcttcgccgagatcag aggtccctaaggggaagaggcaatactc	-	-
CY068501	Human H3N2 IAVs	Human	H3N2		2009	USA	A/California/VRDL313/2009	agtgatgccccattccttgatcggcttcgccgagatcag aggtccctaaggggaagaggcaatactc	-	-
CY068509	Human H3N2 IAVs	Human	H3N2		2009	USA	A/California/VRDL315/2009	agtgatgccccattccttgatcggcttcgccgagatcag aggtccctaaggggaagaggcaatactc	-	-
CY068517	Human H3N2 IAVs	Human	H3N2		2009	USA	A/California/VRDL317/2009	agtgatgccccattccttgatcggcttcgccgagatcag aggtccctaaggggaagaggcaatactc	-	-
CY068533	Human H3N2 IAVs	Human	H3N2		2009	USA	A/California/VRDL319/2009	agtgatgccccattccttgatcggcttcgccgagatcag aggtccctaaggggaagaggcaatactc	-	-
CY068573	Human H3N2 IAVs	Human	H3N2		2009	USA	A/California/VRDL329/2009	agtgatgccccattccttgatcggcttcgccgagatcag aggtccctaaggggaagaggcaatactc	-	-
CY068605	Human H3N2 IAVs	Human	H3N2		2009	USA	A/California/VRDL334/2009	agtgatgccccattccttgatcggcttcgccgagatcag aggtccctaaggggaagaggcaatactc	-	-
CY067981	Human H3N2 IAVs	Human	H3N2		2009	USA	A/California/VRDL340/2009	agtgatgccccattccttgatcggcttcgccgagatcag aggtccctaaggggaagaggcaatactc	-	-
CY068109	Human H3N2 IAVs	Human	H3N2		2009	USA	A/California/VRDL373/2009	agtgatgccccattccttgatcggcttcgccgagatcag aggtccctaaggggaagaggcaatactc	-	-
CY068874	Human H3N2 IAVs	Human	H3N2		2009	USA	A/California/VRDL392/2009	agtgatgccccattccttgatcggcttcgccgagatcag aggtccctaaggggaagaggcaatactc	-	-
CY068746	Human H3N2 IAVs	Human	H3N2		2009	USA	A/California/VRDL199/2009	agtgatgccccattccttgatcggcttcgccgagatcag aggtccctaaggggaagaggcaatactc	-	-
CY106692	Human H3N2 IAVs	Human	H3N2		2009	Hong_Kong	A/Hong_Kong/H090_710_V10/2009	agtgatgccccattccttgatcggcttcgccgagatcag aggtccctaaggggaagaggcaatactc	-	-
CY115572	Human H3N2 IAVs	Human	H3N2		2009	Hong_Kong	A/Hong_Kong/H090_710_V10/2009	agtgatgccccattccttgatcggcttcgccgagatcag aggtccctaaggggaagaggcaatactc	-	-
CY067997	Human H3N2 IAVs	Human	H3N2		2009	USA	A/California/VRDL343/2009	agtgatgccccattccttgatcggcttcgccgagatcag aggtccctaaggggaagaggcaatactc	-	-
CY115828	Human H3N2 IAVs	Human	H3N2		2009	Hong_Kong	A/Hong_Kong/H090_770_V10/2009	agtgatgccccattccttgatcggcttcgccgagatcag aggtccctaaggggaagaggcaatactc	-	-
CY068437	Human H3N2 IAVs	Human	H3N2		2009	USA	A/California/VRDL285/2009	agtgatgccccattccttgatcggcttcgccgagatcag aggtccctaaggggaagaggcaatactc	-	-
CY068525	Human H3N2 IAVs	Human	H3N2		2009	USA	A/California/VRDL318/2009	agtgatgccccattccttgatcggcttcgccgagatcag aggtccctaaggggaagaggcaatactc	-	-
CY068581	Human H3N2 IAVs	Human	H3N2		2009	USA	A/California/VRDL331/2009	agtgatgccccattccttgatcggcttcgccgagatcag aggtccctaaggggaagaggcaatactc	-	-
CY068005	Human H3N2 IAVs	Human	H3N2		2009	USA	A/California/VRDL344/2009	agtgatgccccattccttgatcggcttcgccgagatcag aggtccctaaggggaagaggcaatactc	-	-
CY068037	Human H3N2 IAVs	Human	H3N2		2009	USA	A/California/VRDL352/2009	agtgatgccccattccttgatcggcttcgccgagatcag aggtccctaaggggaagaggcaatactc	-	-
CY068053	Human H3N2 IAVs	Human	H3N2		2009	USA	A/California/VRDL355/2009	agtgatgccccattccttgatcggcttcgccgagatcag aggtccctaaggggaagaggcaatactc	-	-
CY067925	Human H3N2 IAVs	Human	H3N2		2009	USA	A/California/VRDL176/2009	agtgatgccccattccttgatcggcttcgccgagatcag aggtccctaaggggaagaggcaatactc	-	-
CY067241	Human H3N2 IAVs	Human	H3N2		2009	USA	A/California/VRDL307/2009	agtgatgccccattccttgatcggcttcgccgagatcag aggtccctaaggggaagaggcaatactc	-	-

CY068565	Human H3N2 IAVs	Human	H3N2		2009	USA	A/California/VRDL327/2009	agtgatgccccattccttgatcggcttcgccgagatcag aggtccctaaggggaagaggcaatactc	-	-
CY068069	Human H3N2 IAVs	Human	H3N2		2009	USA	A/California/VRDL360/2009	agtgatgccccattccttgatcggcttcgccgagatcag aggtccctaaggggaagaggcaatactc	-	-
CY068826	Human H3N2 IAVs	Human	H3N2		2009	USA	A/California/VRDL381/2009	agtgatgccccattccttgatcggcttcgccgagatcag aggtccctaaggggaagaggcaatactc	-	-
CY081466	Human H3N2 IAVs	Human	H3N2		2009	Cambodia	A/Cambodia/NHRCC00001/2009	agtgatgccccattccttgatcggcttcgccgagatcag aggtccctaaggggaagaggcaatactc	-	-
CY081474	Human H3N2 IAVs	Human	H3N2		2009	Cambodia	A/Cambodia/NHRCC00002/2009	agtgatgccccattccttgatcggcttcgccgagatcag aggtccctaaggggaagaggcaatactc	-	-
CY081434	Human H3N2 IAVs	Human	H3N2		2009	Cambodia	A/Cambodia/NHRCC00003/2009	agtgatgccccattccttgatcggcttcgccgagatcag aggtccctaaggggaagaggcaatactc	-	-
CY081562	Human H3N2 IAVs	Human	H3N2		2009	Cambodia	A/Cambodia/NHRCC00004/2009	agtgatgccccattccttgatcggcttcgccgagatcag aggtccctaaggggaagaggcaatactc	-	-
CY081482	Human H3N2 IAVs	Human	H3N2		2009	Cambodia	A/Cambodia/NHRCC00006/2009	agtgatgccccattccttgatcggcttcgccgagatcag aggtccctaaggggaagaggcaatactc	-	-
CY081442	Human H3N2 IAVs	Human	H3N2		2009	Cambodia	A/Cambodia/NHRCC00007/2009	agtgatgccccattccttgatcggcttcgccgagatcag aggtccctaaggggaagaggcaatactc	-	-
CY119062	Human H3N2 IAVs	Human	H3N2		2009	Malaysia	A/Malaysia/2205714/2009	agtgatgccccattccttgatcggcttcgccgagatcag aggtccctaaggggaagaggcaatactc	-	-
CY068045	Human H3N2 IAVs	Human	H3N2		2009	USA	A/California/VRDL354/2009	agtgatgccccattccttgatcggcttcgccgagatcag aggtccctaaggggaagaggcaatactc	-	-
CY068077	Human H3N2 IAVs	Human	H3N2		2009	USA	A/California/VRDL361/2009	agtgatgccccattccttgatcggcttcgccgagatcag aggtccctaaggggaagaggcaatactc	-	-
CY068589	Human H3N2 IAVs	Human	H3N2		2009	USA	A/California/VRDL332/2009	agtgatgccccattccttgatcggcttcgccgagatcag aggtccctaaggggaagaggcaatactc	-	-
CY115796	Human H3N2 IAVs	Human	H3N2		2009	Hong_Kong	A/Hong_Kong/H090_763_V10/2009	agtgatgccccattccttgatcggcttcgccgagatcag aggtccctaaggggaagaggcaatactc	-	-
CY106940	Human H3N2 IAVs	Human	H3N2		2009	Hong_Kong	A/Hong_Kong/H090_763_V10/2009	agtgatgccccattccttgatcggcttcgccgagatcag aggtccctaaggggaagaggcaatactc	-	-
CY106948	Human H3N2 IAVs	Human	H3N2		2009	Hong_Kong	A/Hong_Kong/H090_763_V23/2009	agtgatgccccattccttgatcggcttcgccgagatcag aggtccctaaggggaagaggcaatactc	-	-
CY115804	Human H3N2 IAVs	Human	H3N2		2009	Hong_Kong	A/Hong_Kong/H090_763_V23/2009	agtgatgccccattccttgatcggcttcgccgagatcag aggtccctaaggggaagaggcaatactc	-	-
CY121740	Human H3N2 IAVs	Human	H3N2		2009	Hong_Kong	A/Hong_Kong/26560/2009	agtgatgccccattccttgatcggcttcgccgagatcag aggtccctaaggggaagaggcaatactc	-	-
CY106876	Human H3N2 IAVs	Human	H3N2		2009	Hong_Kong	A/Hong_Kong/H090_747_V10/2009	agtgatgccccattccttgatcggcttcgccgagatcag aggtccctaaggggaagaggcaatactc	-	-
CY115748	Human H3N2 IAVs	Human	H3N2		2009	Hong_Kong	A/Hong_Kong/H090_747_V10/2009	agtgatgccccattccttgatcggcttcgccgagatcag aggtccctaaggggaagaggcaatactc	-	-
CY106884	Human H3N2 IAVs	Human	H3N2		2009	Hong_Kong	A/Hong_Kong/H090_747_V20/2009	agtgatgccccattccttgatcggcttcgccgagatcag aggtccctaaggggaagaggcaatactc	-	-
CY106892	Human H3N2 IAVs	Human	H3N2		2009	Hong_Kong	A/Hong_Kong/H090_747_V22/2009	agtgatgccccattccttgatcggcttcgccgagatcag aggtccctaaggggaagaggcaatactc	-	-
CY115756	Human H3N2 IAVs	Human	H3N2		2009	Hong_Kong	A/Hong_Kong/H090_747_V22/2009	agtgatgccccattccttgatcggcttcgccgagatcag aggtccctaaggggaagaggcaatactc	-	-
CY115820	Human H3N2 IAVs	Human	H3N2		2009	Hong_Kong	A/Hong_Kong/H090_769_V10/2009	agtgatgccccattccttgatcggcttcgccgagatcag aggtccctaaggggaagaggcaatactc	-	-
CY106972	Human H3N2 IAVs	Human	H3N2		2009	Hong_Kong	A/Hong_Kong/H090_769_V10/2009	agtgatgccccattccttgatcggcttcgccgagatcag aggtccctaaggggaagaggcaatactc	-	-

CY115708	Human H3N2 IAVs	Human	H3N2		2009	Hong_Kong	A/Hong_Kong/H090_739_V10/2009	agtgatgccccattccttgatcggcttcgccgagatcagaggtccctaaggggaagaggcaatactc	-	-
CY106836	Human H3N2 IAVs	Human	H3N2		2009	Hong_Kong	A/Hong_Kong/H090_739_V10/2009	agtgatgccccattccttgatcggcttcgccgagatcagaggtccctaaggggaagaggcaatactc	-	-
CY106844	Human H3N2 IAVs	Human	H3N2		2009	Hong_Kong	A/Hong_Kong/H090_739_V22/2009	agtgatgccccattccttgatcggcttcgccgagatcagaggtccctaaggggaagaggcaatactc	-	-
CY115716	Human H3N2 IAVs	Human	H3N2		2009	Hong_Kong	A/Hong_Kong/H090_739_V22/2009	agtgatgccccattccttgatcggcttcgccgagatcagaggtccctaaggggaagaggcaatactc	-	-
CY115724	Human H3N2 IAVs	Human	H3N2		2009	Hong_Kong	A/Hong_Kong/H090_739_V23/2009	agtgatgccccattccttgatcggcttcgccgagatcagaggtccctaaggggaagaggcaatactc	-	-
CY106852	Human H3N2 IAVs	Human	H3N2		2009	Hong_Kong	A/Hong_Kong/H090_739_V23/2009	agtgatgccccattccttgatcggcttcgccgagatcagaggtccctaaggggaagaggcaatactc	-	-
CY115732	Human H3N2 IAVs	Human	H3N2		2009	Hong_Kong	A/Hong_Kong/H090_739_V33/2009	agtgatgccccattccttgatcggcttcgccgagatcagaggtccctaaggggaagaggcaatactc	-	-
CY106860	Human H3N2 IAVs	Human	H3N2		2009	Hong_Kong	A/Hong_Kong/H090_739_V33/2009	agtgatgccccattccttgatcggcttcgccgagatcagaggtccctaaggggaagaggcaatactc	-	-
CY080567	Human H3N2 IAVs	Human	H3N2		2009	Australia	A/Australia/55/2009	agtgatgccccattccttgatcggcttcgccgagatcagaggtccctaaggggaagaggcaatactc	-	-
CY080495	Human H3N2 IAVs	Human	H3N2		2009	USA	A/New_York/3932/2009	agtgatgccccattccttgatcggcttcgccgagatcagaggtccctaaggggaagaggcaatactc	-	-
CY089544	Human H3N2 IAVs	Human	H3N2		2009	USA	A/California/VRDL359/2009	agtgatgccccattccttgatcggcttcgccgagatcagaggtccctaaggggaagaggcaatactc	-	-
CY082996	Human H3N2 IAVs	Human	H3N2		2009	Cambodia	A/Cambodia/NHRCC00011/2009	agtgatgccccattccttgatcggcttcgccgagatcagaggtccctaaggggaagaggcaatactc	-	-
CY050592	Human H3N2 IAVs	Human	H3N2		2009	USA	A/New_York/3006/2009	agtgatgccccattccttgatcggcttcgccgagatcagaggtccctaaggggaagaggcaatactc	-	-
CY058752	Human H3N2 IAVs	Human	H3N2		2009	USA	A/New_York/3061/2009	agtgatgccccattccttgatcggcttcgccgagatcagaggtccctaaggggaagaggcaatactc	-	-
CY050520	Human H3N2 IAVs	Human	H3N2		2009	USA	A/New_York/3066/2009	agtgatgccccattccttgatcggcttcgccgagatcagaggtccctaaggggaagaggcaatactc	-	-
CY050528	Human H3N2 IAVs	Human	H3N2		2009	USA	A/New_York/3078/2009	agtgatgccccattccttgatcggcttcgccgagatcagaggtccctaaggggaagaggcaatactc	-	-
CY050832	Human H3N2 IAVs	Human	H3N2		2009	USA	A/New_York/3752/2009	agtgatgccccattccttgatcggcttcgccgagatcagaggtccctaaggggaagaggcaatactc	-	-
JX905403	Human H3N2 IAVs	Human	H3N2		2009	USA	A/Florida/22/2009	agtgatgccccattccttgatcggcttcgccgagatcagaggtccctaaggggaagaggcaatactc	-	-
CY050720	Human H3N2 IAVs	Human	H3N2		2009	USA	A/New_York/3297/2009	agtgatgccccattccttgatcggcttcgccgagatcagaggtccctaaggggaagaggcaatactc	-	-
CY080463	Human H3N2 IAVs	Human	H3N2		2009	USA	A/New_York/3421/2009	agtgatgccccattccttgatcggcttcgccgagatcagaggtccctaaggggaagaggcaatactc	-	-
CY080471	Human H3N2 IAVs	Human	H3N2		2009	USA	A/New_York/3470/2009	agtgatgccccattccttgatcggcttcgccgagatcagaggtccctaaggggaagaggcaatactc	-	-
CY080487	Human H3N2 IAVs	Human	H3N2		2009	USA	A/New_York/3644/2009	agtgatgccccattccttgatcggcttcgccgagatcagaggtccctaaggggaagaggcaatactc	-	-
CY089633	Human H3N2 IAVs	Human	H3N2		2009	USA	A/New_York/3750/2009	agtgatgccccattccttgatcggcttcgccgagatcagaggtccctaaggggaagaggcaatactc	-	-
CY084346	Human H3N2 IAVs	Human	H3N2		2009	USA	A/New_York/4017/2009	agtgatgccccattccttgatcggcttcgccgagatcagaggtccctaaggggaagaggcaatactc	-	-
CY050456	Human H3N2 IAVs	Human	H3N2		2009	USA	A/New_York/1670/2009	agtgatgccccattccttgatcggcttcgccgagatcagaggtccctaaggggaagaggcaatactc	-	-

CY050464	Human H3N2 IAVs	Human	H3N2		2009	USA	A/New_York/1671/2009	agtgatgccccattccttgatcggcttcgccgagatcag<br>aggtccctaaggggaagaggcaatactc	-	-
CY050472	Human H3N2 IAVs	Human	H3N2		2009	USA	A/New_York/1685/2009	agtgatgccccattccttgatcggcttcgccgagatcag<br>aggtccctaaggggaagaggcaatactc	-	-
CY050608	Human H3N2 IAVs	Human	H3N2		2009	USA	A/New_York/3018/2009	agtgatgccccattccttgatcggcttcgccgagatcag<br>aggtccctaaggggaagaggcaatactc	-	-
CY050504	Human H3N2 IAVs	Human	H3N2		2009	USA	A/New_York/3062/2009	agtgatgccccattccttgatcggcttcgccgagatcag<br>aggtccctaaggggaagaggcaatactc	-	-
CY050512	Human H3N2 IAVs	Human	H3N2		2009	USA	A/New_York/3064/2009	agtgatgccccattccttgatcggcttcgccgagatcag<br>aggtccctaaggggaagaggcaatactc	-	-
CY050560	Human H3N2 IAVs	Human	H3N2		2009	USA	A/New_York/3103/2009	agtgatgccccattccttgatcggcttcgccgagatcag<br>aggtccctaaggggaagaggcaatactc	-	-
CY050576	Human H3N2 IAVs	Human	H3N2		2009	USA	A/New_York/3127/2009	agtgatgccccattccttgatcggcttcgccgagatcag<br>aggtccctaaggggaagaggcaatactc	-	-
CY050648	Human H3N2 IAVs	Human	H3N2		2009	USA	A/New_York/3147/2009	agtgatgccccattccttgatcggcttcgccgagatcag<br>aggtccctaaggggaagaggcaatactc	-	-
CY050680	Human H3N2 IAVs	Human	H3N2		2009	USA	A/New_York/3272/2009	agtgatgccccattccttgatcggcttcgccgagatcag<br>aggtccctaaggggaagaggcaatactc	-	-
CY084397	Human H3N2 IAVs	Human	H3N2		2009	USA	A/New_York/3417/2009	agtgatgccccattccttgatcggcttcgccgagatcag<br>aggtccctaaggggaagaggcaatactc	-	-
CY050784	Human H3N2 IAVs	Human	H3N2		2009	USA	A/New_York/3471/2009	agtgatgccccattccttgatcggcttcgccgagatcag<br>aggtccctaaggggaagaggcaatactc	-	-
CY058776	Human H3N2 IAVs	Human	H3N2		2009	USA	A/New_York/3494/2009	agtgatgccccattccttgatcggcttcgccgagatcag<br>aggtccctaaggggaagaggcaatactc	-	-
CY050800	Human H3N2 IAVs	Human	H3N2		2009	USA	A/New_York/3496/2009	agtgatgccccattccttgatcggcttcgccgagatcag<br>aggtccctaaggggaagaggcaatactc	-	-
CY055103	Human H3N2 IAVs	Human	H3N2		2009	USA	A/New_York/3743/2009	agtgatgccccattccttgatcggcttcgccgagatcag<br>aggtccctaaggggaagaggcaatactc	-	-
CY050600	Human H3N2 IAVs	Human	H3N2		2009	USA	A/New_York/3011/2009	agtgatgccccattccttgatcggcttcgccgagatcag<br>aggtccctaaggggaagaggcaatactc	-	-
CY050632	Human H3N2 IAVs	Human	H3N2		2009	USA	A/New_York/3139/2009	agtgatgccccattccttgatcggcttcgccgagatcag<br>aggtccctaaggggaagaggcaatactc	-	-
CY050656	Human H3N2 IAVs	Human	H3N2		2009	USA	A/New_York/3148/2009	agtgatgccccattccttgatcggcttcgccgagatcag<br>aggtccctaaggggaagaggcaatactc	-	-
CY050672	Human H3N2 IAVs	Human	H3N2		2009	USA	A/New_York/3164/2009	agtgatgccccattccttgatcggcttcgccgagatcag<br>aggtccctaaggggaagaggcaatactc	-	-
CY050688	Human H3N2 IAVs	Human	H3N2		2009	USA	A/New_York/3273/2009	agtgatgccccattccttgatcggcttcgccgagatcag<br>aggtccctaaggggaagaggcaatactc	-	-
CY058760	Human H3N2 IAVs	Human	H3N2		2009	USA	A/New_York/3316/2009	agtgatgccccattccttgatcggcttcgccgagatcag<br>aggtccctaaggggaagaggcaatactc	-	-
CY050744	Human H3N2 IAVs	Human	H3N2		2009	USA	A/New_York/3317/2009	agtgatgccccattccttgatcggcttcgccgagatcag<br>aggtccctaaggggaagaggcaatactc	-	-
CY084389	Human H3N2 IAVs	Human	H3N2		2009	USA	A/New_York/3318/2009	agtgatgccccattccttgatcggcttcgccgagatcag<br>aggtccctaaggggaagaggcaatactc	-	-
CY050840	Human H3N2 IAVs	Human	H3N2		2009	USA	A/New_York/3487/2009	agtgatgccccattccttgatcggcttcgccgagatcag<br>aggtccctaaggggaagaggcaatactc	-	-
CY084405	Human H3N2 IAVs	Human	H3N2		2009	USA	A/New_York/3904/2009	agtgatgccccattccttgatcggcttcgccgagatcag<br>aggtccctaaggggaagaggcaatactc	-	-
CY058808	Human H3N2 IAVs	Human	H3N2		2009	USA	A/New_York/4255/2009	agtgatgccccattccttgatcggcttcgccgagatcag<br>aggtccctaaggggaagaggcaatactc	-	-

CY084421	Human H3N2 IAVs	Human	H3N2		2009	USA	A/New_York/4362/2009	agtgatgccccattccttgatcggcttcgccgagatcag<br>aggtccctaaggggaagaggcaatactc	-	-
CY081053	Human H3N2 IAVs	Human	mixed		2009	USA	A/New_York/3720/2009	agtgatgccccattccttgatcggcttcgccgagatcag<br>aggtccctaaggggaagaggcaatactc	-	-
CY050488	Human H3N2 IAVs	Human	H3N2		2009	USA	A/New_York/3051/2009	agtgatgccccattccttgatcggcttcgccgagatcag<br>aggtccctaaggggaagaggcaatactc	-	-
CY058792	Human H3N2 IAVs	Human	H3N2		2009	USA	A/New_York/3896/2009	agtgatgccccattccttgatcggcttcgccgagatcag<br>aggtccctaaggggaagaggcaatactc	-	-
CY084413	Human H3N2 IAVs	Human	H3N2		2009	USA	A/New_York/3918/2009	agtgatgccccattccttgatcggcttcgccgagatcag<br>aggtccctaaggggaagaggcaatactc	-	-
CY058784	Human H3N2 IAVs	Human	H3N2		2009	USA	A/New_York/3527/2009	agtgatgccccattccttgatcggcttcgccgagatcag<br>aggtccctaaggggaagaggcaatactc	-	-
CY050728	Human H3N2 IAVs	Human	H3N2		2009	USA	A/New_York/3143/2009	agtgatgccccattccttgatcggcttcgccgagatcag<br>aggtccctaaggggaagaggcaatactc	-	-
CY050824	Human H3N2 IAVs	Human	H3N2		2009	USA	A/New_York/3738/2009	agtgatgccccattccttgatcggcttcgccgagatcag<br>aggtccctaaggggaagaggcaatactc	-	-
CY055095	Human H3N2 IAVs	Human	H3N2		2009	USA	A/New_York/4165/2009	agtgatgccccattccttgatcggcttcgccgagatcag<br>aggtccctaaggggaagaggcaatactc	-	-
CY055087	Human H3N2 IAVs	Human	H3N2		2009	USA	A/New_York/3687/2009	agtgatgccccattccttgatcggcttcgccgagatcag<br>aggtccctaaggggaagaggcaatactc	-	-
CY089769	Human H3N2 IAVs	Human	H3N2		2009	USA	A/Boston/97/2009	agtgatgccccattccttgatcggcttcgccgagatcag<br>aggtccctaaggggaagaggcaatactc	-	-
GQ902813	Human H3N2 IAVs	Human	H3N2		2009	Thailand	A/Thailand/CU_B110/2009	agtgatgccccattccttgatcggcttcgccgagatcag<br>aggtccctaaggggaagaggcaatactc	-	-
CY050792	Human H3N2 IAVs	Human	H3N2		2009	USA	A/New_York/3491/2009	agtgatgccccattccttgatcggcttcgccgagatcag<br>aggtccctaaggggaagaggcaatactc	-	-
CY068325	Human H3N2 IAVs	Human	H3N2		2009	USA	A/California/VRDL259/2009	agtgatgccccattccttgatcggcttcgccgagatcag<br>aggtccctaaggggaagaggcaatactc	-	-
KF014731	Human H3N2 IAVs	Human	H3N2		2009	Singapore	A/Singapore/C2009.485a/2009	agtgatgccccattccttgatcggcttcgccgagatcag<br>aggtccctaaggggaagaggcaatactc	-	-
KF014659	Human H3N2 IAVs	Human	H3N2		2009	Singapore	A/Singapore/C2009.485a/2009	agtgatgccccattccttgatcggcttcgccgagatcag<br>aggtccctaaggggaagaggcaatactc	-	-
CY068013	Human H3N2 IAVs	Human	H3N2		2009	USA	A/California/VRDL345/2009	agtgatgccccattccttgatcggcttcgccgagatcag<br>aggtccctaaggggaagaggcaatactc	-	-
CY068021	Human H3N2 IAVs	Human	H3N2		2009	USA	A/California/VRDL349/2009	agtgatgccccattccttgatcggcttcgccgagatcag<br>aggtccctaaggggaagaggcaatactc	-	-
CY067965	Human H3N2 IAVs	Human	H3N2		2009	USA	A/California/VRDL328/2009	agtgatgccccattccttgatcggcttcgccgagatcag<br>aggtccctaaggggaagaggcaatactc	-	-
CY070923	Human H3N2 IAVs	Human	H3N2		2009	USA	A/California/VRDL330/2009_H1N1<br>_	agtgatgccccattccttgatcggcttcgccgagatcag<br>aggtccctaaggggaagaggcaatactc	-	-
CY121087	Human H3N2 IAVs	Human	unknown		2009	Philippines	A/Philippines/219/2009	agtgatgccccattccttgatcggcttcgccgagatcag<br>aggtccctaaggggaagaggcaatactc	-	-
KF014730	Human H3N2 IAVs	Human	H3N2		2009	Singapore	A/Singapore/C2009.458V/2009	agtgatgccccattccttgatcggcttcgccgagatcag<br>aggtccctaaggggaagaggcaatactc	-	-
JX437768	Human H3N2 IAVs	Human	H3N2		2009	Singapore	A/Singapore/C2009.458V/2009	agtgatgccccattccttgatcggcttcgccgagatcag<br>aggtccctaaggggaagaggcaatactc	-	-
JX437890	Human H3N2 IAVs	Human	H3N2		2009	Singapore	A/Singapore/H2009.471C/2009	agtgatgccccattccttgatcggcttcgccgagatcag<br>aggtccctaaggggaagaggcaatactc	-	-
KF014689	Human H3N2 IAVs	Human	H3N2		2009	Singapore	A/Singapore/H2009.471C/2009	agtgatgccccattccttgatcggcttcgccgagatcag<br>aggtccctaaggggaagaggcaatactc	-	-

KF014662	Human H3N2 IAVs	Human	H3N2		2009	Singapore	A/Singapore/C2009.863/2009	agtgatgccccattccttgatcggcttcgccgagatcagaggtccctaaggggaagaggcaatactc	-	-
KF014734	Human H3N2 IAVs	Human	H3N2		2009	Singapore	A/Singapore/C2009.863/2009	agtgatgccccattccttgatcggcttcgccgagatcagaggtccctaaggggaagaggcaatactc	-	-
KF014762	Human H3N2 IAVs	Human	H3N2		2009	Singapore	A/Singapore/H2009.485/2009	agtgatgccccattccttgatcggcttcgccgagatcagaggtccctaaggggaagaggcaatactc	-	-
KF014688	Human H3N2 IAVs	Human	H3N2		2009	Singapore	A/Singapore/H2009.485/2009	agtgatgccccattccttgatcggcttcgccgagatcagaggtccctaaggggaagaggcaatactc	-	-
CY081458	Human H3N2 IAVs	Human	H3N2		2009	Cambodia	A/Cambodia/NHRCC00010/2009	agtgatgccccattccttgatcggcttcgccgagatcagaggtccctaaggggaagaggcaatactc	-	-
CY082972	Human H3N2 IAVs	Human	H3N2		2009	Cambodia	A/Cambodia/NHRCC00005/2009	agtgatgccccattccttgatcggcttcgccgagatcagaggtccctaaggggaagaggcaatactc	-	-
CY115476	Human H3N2 IAVs	Human	H3N2		2009	Hong_Kong	A/Hong_Kong/H090_669_V10/2009	agtgatgccccattccttgatcggcttcgccgagatcagaggtccctaaggggaagaggcaatactc	-	-
CY106588	Human H3N2 IAVs	Human	H3N2		2009	Hong_Kong	A/Hong_Kong/H090_669_V10/2009	agtgatgccccattccttgatcggcttcgccgagatcagaggtccctaaggggaagaggcaatactc	-	-
GU271986	Human H3N2 IAVs	Human	H3N2		2009	Thailand	A/Thailand/CU_B1697/2009	agtgatgccccattccttgatcggcttcgccgagatcagaggtccctaaggggaagaggcaatactc	-	-
CY092777	Human H3N2 IAVs	Human	mixed		2009	USA	A/California/VRDL353/2009	agtgatgccccattccttgatcggcttcgccgagatcagaggtccctaaggggaagaggcaatactc	-	-
CY067941	Human H3N2 IAVs	Human	H3N2		2009	USA	A/California/VRDL286/2009	agtgatgccccattccttgatcggcttcgccgagatcagaggtccctaaggggaagaggcaatactc	-	-
CY080535	Human H3N2 IAVs	Human	H3N2		2009	Australia	A/Australia/46/2009	agtgatgccccattccttgatcggcttcgccgagatcagaggtccctaaggggaagaggcaatactc	-	-
CY069465	Human H3N2 IAVs	Human	H3N2		2009	USA	A/Alaska/WRAIR1145P/2009	agtgatgccccattccttgatcggcttcgccgagatcagaggtccctaaggggaagaggcaatactc	-	-
CY069497	Human H3N2 IAVs	Human	H3N2		2009	USA	A/Alaska/WRAIR1172P/2009	agtgatgccccattccttgatcggcttcgccgagatcagaggtccctaaggggaagaggcaatactc	-	-
CY072194	Human H3N2 IAVs	Human	H3N2		2009	USA	A/California/VRDL347/2009_H1N1_	agtgatgccccattccttgatcggcttcgccgagatcagaggtccctaaggggaagaggcaatactc	-	-
CY068029	Human H3N2 IAVs	Human	H3N2		2009	USA	A/California/VRDL351/2009	agtgatgccccattccttgatcggcttcgccgagatcagaggtccctaaggggaagaggcaatactc	-	-
CY064851	Human H3N2 IAVs	Human	H3N2		2009	USA	A/California/VRDL143/2009	agtgatgccccattccttgatcggcttcgccgagatcagaggtccctaaggggaagaggcaatactc	-	-
CY092333	Human H3N2 IAVs	Human	H3N2		2009	USA	A/California/VRDL182/2009	agtgatgccccattccttgatcggcttcgccgagatcagaggtccctaaggggaagaggcaatactc	-	-
CY068349	Human H3N2 IAVs	Human	H3N2		2009	USA	A/California/VRDL262/2009	agtgatgccccattccttgatcggcttcgccgagatcagaggtccctaaggggaagaggcaatactc	-	-
CY068253	Human H3N2 IAVs	Human	H3N2		2009	USA	A/California/VRDL216/2009	agtgatgccccattccttgatcggcttcgccgagatcagaggtccctaaggggaagaggcaatactc	-	-
CY068477	Human H3N2 IAVs	Human	H3N2		2009	USA	A/California/VRDL309/2009	agtgatgccccattccttgatcggcttcgccgagatcagaggtccctaaggggaagaggcaatactc	-	-
CY050544	Human H3N2 IAVs	Human	H3N2		2009	USA	A/New_York/3087/2009	agtgatgccccattccttgatcggcttcgccgagatcagaggtccctaaggggaagaggcaatactc	-	-
CY064891	Human H3N2 IAVs	Human	H3N2		2009	USA	A/California/VRDL158/2009	agtgatgccccattccttgatcggcttcgccgagatcagaggtccctaaggggaagaggcaatactc	-	-
CY068698	Human H3N2 IAVs	Human	H3N2		2009	USA	A/California/VRDL183/2009	agtgatgccccattccttgatcggcttcgccgagatcagaggtccctaaggggaagaggcaatactc	-	-
CY068429	Human H3N2 IAVs	Human	H3N2		2009	USA	A/California/VRDL279/2009	agtgatgccccattccttgatcggcttcgccgagatcagaggtccctaaggggaagaggcaatactc	-	-

CY069457	Human H3N2 IAVs	Human	H3N2		2009	USA	A/Nevada/WRAIR1144P/2009	agtgatgccccattccttgatcggcttcgccgagatcag aggtccctaaggggaagaggcaatactc	-	-
CY093331	Human H3N2 IAVs	Human	H3N2		2009	USA	A/Nevada/WRAIR1258P/2009	agtgatgccccattccttgatcggcttcgccgagatcag aggtccctaaggggaagaggcaatactc	-	-
CY050536	Human H3N2 IAVs	Human	H3N2		2009	USA	A/New_York/3079/2009	agtgatgccccattccttgatcggcttcgccgagatcag aggtccctaaggggaagaggcaatactc	-	-
CY068317	Human H3N2 IAVs	Human	H3N2		2009	USA	A/California/VRDL253/2009	agtgatgccccattccttgatcggcttcgccgagatcag aggtccctaaggggaagaggcaatactc	-	-
CY080551	Human H3N2 IAVs	Human	H3N2		2009	Australia	A/Australia/30/2009	agtgatgccccattccttgatcggcttcgccgagatcag aggtccctaaggggaagaggcaatactc	-	-
CY068277	Human H3N2 IAVs	Human	H3N2		2009	USA	A/California/VRDL233/2009	agtgatgccccattccttgatcggcttcgccgagatcag aggtccctaaggggaagaggcaatactc	-	-
CY068613	Human H3N2 IAVs	Human	H3N2		2009	USA	A/California/VRDL335/2009	agtgatgccccattccttgatcggcttcgccgagatcag aggtccctaaggggaagaggcaatactc	-	-
CY173523	Human H3N2 IAVs	Human	H3N2		2009	USA	A/New_York/1189/2009	agtgatgccccattccttgatcggcttcgccgagatcag aggtccctaaggggaagaggcaatactc	-	-
CY080454	Human H3N2 IAVs	Human	H3N2		2009	USA	A/New_York/3745/2009	agtgatgccccattccttgatcggcttcgccgagatcag aggtccctaaggggaagaggcaatactc	-	-
CY068621	Human H3N2 IAVs	Human	H3N2		2009	USA	A/California/VRDL336/2009	agtgatgccccattccttgatcggcttcgccgagatcag aggtccctacggggaagaggcaatactc	-	-
CY106612	Human H3N2 IAVs	Human	H3N2		2009	Hong_Kong	A/Hong_Kong/H090_674_V10/2009	agtgatgccccgttccttgatcggcttcgccgagatcag aggtccctaaagggaagaggcaatactc	-	-
CY115500	Human H3N2 IAVs	Human	H3N2		2009	Hong_Kong	A/Hong_Kong/H090_674_V10/2009	agtgatgccccgttccttgatcggcttcgccgagatcag aggtccctaaagggaagaggcaatactc	-	-
CY093347	Human H3N2 IAVs	Human	H3N2		2009	USA	A/Texas/WRAIR1558P/2009	agtgatgctccattccttgatcggcttcgccgagatcag aggtccctaaggggaagaggcaatactc	-	-
CY073873	Human H3N2 IAVs	Human	H3N2		2010	Nicaragua	A/Managua/1422.03/2010	agtgataccccattccttgatcggcttcgccgagatca gaggtccctaaggggaagaggcaatactc	-	-
CY074954	Human H3N2 IAVs	Human	H3N2		2010	Thailand	A/Thailand/CU_H1285/2010	agtgatgccccattccttgataggcttcgccgagatca gaggtccctaaggggaagaggcaatactc	+	+
CY074962	Human H3N2 IAVs	Human	H3N2		2010	Thailand	A/Thailand/CU_H1443/2010	agtgatgccccattccttgataggcttcgccgagatca gaggtccctaaggggaagaggcaatactc	+	+
CY074970	Human H3N2 IAVs	Human	H3N2		2010	Thailand	A/Thailand/CU_H1817/2010	agtgatgccccattccttgataggcttcgccgagatca gaggtccctaaggggaagaggcaatactc	+	+
CY088935	Human H3N2 IAVs	Human	H3N2		2010	Nicaragua	A/Managua/4747.04/2010	agtgatgccccattccttgatcggcttcgccgagacca gaggtccctaaggggaagaggcaatactc	-	-
CY161796	Human H3N2 IAVs	Human	H3N2		2010	Peru	A/Peru/PER191/2010	agtgatgccccattccttgatcggcttcgccgagatcag agatccctaaggggaagaggcaatactc	-	*
KM070057	Human H3N2 IAVs	Human	H3N2		2010	Singapore	A/Singapore/C2010.416/2010	agtgatgccccattccttgatcggcttcgccgagatcag agatccctaaggggaagaggcaatactc	-	*
KF014768	Human H3N2 IAVs	Human	H3N2		2010	Singapore	A/Singapore/H2010.384/2010	agtgatgccccattccttgatcggcttcgccgagatcag agatccctaaggggaagaggcaatactc	-	*
KF014697	Human H3N2 IAVs	Human	H3N2		2010	Singapore	A/Singapore/H2010.384/2010	agtgatgccccattccttgatcggcttcgccgagatcag agatccctaaggggaagaggcaatactc	-	*
JX437895	Human H3N2 IAVs	Human	H3N2		2010	Singapore	A/Singapore/H2010.564C/2010	agtgatgccccattccttgatcggcttcgccgagatcag agatccctaaggggaagaggcaatactc	-	*
KF014701	Human H3N2 IAVs	Human	H3N2		2010	Singapore	A/Singapore/H2010.564C/2010	agtgatgccccattccttgatcggcttcgccgagatcag agatccctaaggggaagaggcaatactc	-	*
KM070066	Human H3N2 IAVs	Human	H3N2		2010	Singapore	A/Singapore/H2010.586/2010	agtgatgccccattccttgatcggcttcgccgagatcag agatccctaaggggaagaggcaatactc	-	*

KM070050	Human H3N2 IAVs	Human	H3N2		2010	Singapore	A/Singapore/S2010.345c/2010	agtgatgccccattccttgatcggcttcgccgagatcag<br>agatccctaaggggaagaggcaatactc	-	*
KM070064	Human H3N2 IAVs	Human	H3N2		2010	Singapore	A/Singapore/H2010.570/2010	agtgatgccccattccttgatcggcttcgccgagatcag<br>agatccctaaggggaagaggcaatactc	-	*
CY160292	Human H3N2 IAVs	Human	H3N2		2010	Peru	A/Peru/PER001/2010	agtgatgccccattccttgatcggcttcgccgagatcag<br>aggtccctaagggaaagaggcaacactc	+	+
CY160876	Human H3N2 IAVs	Human	H3N2		2010	Peru	A/Peru/PER074/2010	agtgatgccccattccttgatcggcttcgccgagatcag<br>aggtccctaagggaaagaggcaacactc	+	+
CY161716	Human H3N2 IAVs	Human	H3N2		2010	Peru	A/Peru/PER181/2010	agtgatgccccattccttgatcggcttcgccgagatcag<br>aggtccctaagggaaagaggcaacactc	+	+
KF550962	Human H3N2 IAVs	Swine	H3N2		2010	China	A/swine/Henan/1/2010	agtgatgccccattccttgatcggcttcgccgagatcag<br>aggtccctaaggggaagaggcaacactc	-	-
CY070971	Human H3N2 IAVs	Human	H3N2		2010	USA	A/New_York/20342/2010	agtgatgccccattccttgatcggcttcgccgagatcag<br>aggtccctaaggggaagaggcaacactc	-	-
CY160285	Human H3N2 IAVs	Human	H3N2		2010	Peru	A/Peru/PER261/2010	agtgatgccccattccttgatcggcttcgccgagatcag<br>aggtccctaaggggaagaggcaacactc	-	-
CY160668	Human H3N2 IAVs	Human	H3N2		2010	Peru	A/Peru/PER048/2010	agtgatgccccattccttgatcggcttcgccgagatcag<br>aggtccctaaggggaagaggcaacactc	-	-
CY161564	Human H3N2 IAVs	Human	H3N2		2010	Peru	A/Peru/PER162/2010	agtgatgccccattccttgatcggcttcgccgagatcag<br>aggtccctaaggggaagaggcaacactc	-	-
CY161724	Human H3N2 IAVs	Human	H3N2		2010	Peru	A/Peru/PER182/2010	agtgatgccccattccttgatcggcttcgccgagatcag<br>aggtccctaaggggaagaggcaacactc	-	-
CY162132	Human H3N2 IAVs	Human	H3N2		2010	Peru	A/Peru/PER233/2010	agtgatgccccattccttgatcggcttcgccgagatcag<br>aggtccctaaggggaagaggcaacactc	-	-
CY162220	Human H3N2 IAVs	Human	H3N2		2010	Peru	A/Peru/PER245/2010	agtgatgccccattccttgatcggcttcgccgagatcag<br>aggtccctaaggggaagaggcaacactc	-	-
CY163004	Human H3N2 IAVs	Human	H3N2		2010	Peru	A/Peru/PER347/2010	agtgatgccccattccttgatcggcttcgccgagatcag<br>aggtccctaaggggaagaggcaacactc	-	-
CY163236	Human H3N2 IAVs	Human	H3N2		2010	Peru	A/Peru/PER379/2010	agtgatgccccattccttgatcggcttcgccgagatcag<br>aggtccctaaggggaagaggcaacactc	-	-
CY093427	Human H3N2 IAVs	Human	H3N2		2010	Dominican_Republic	A/Santo_Domingo/WRAIR3514T/2<br>010	agtgatgccccattccttgatcggcttcgccgagatcag<br>aggtccctaaggggaagaggcaacactc	-	-
CY093435	Human H3N2 IAVs	Human	H3N2		2010	Dominican_Republic	A/Santo_Domingo/WRAIR3516N/2<br>010	agtgatgccccattccttgatcggcttcgccgagatcag<br>aggtccctaaggggaagaggcaacactc	-	-
CY093443	Human H3N2 IAVs	Human	H3N2		2010	Dominican_Republic	A/Santo_Domingo/WRAIR3516T/2<br>010	agtgatgccccattccttgatcggcttcgccgagatcag<br>aggtccctaaggggaagaggcaacactc	-	-
CY088903	Human H3N2 IAVs	Human	H3N2		2010	Nicaragua	A/Managua/1155.01/2010	agtgatgccccattccttgatcggcttcgccgagatcag<br>aggtccctaaggggaagaggcaataccc	-	-
CY093379	Human H3N2 IAVs	Human	H3N2		2010	Mexico	A/Mexico_City/WRAIR1752T/2010	agtgatgccccattccttgatcggcttcgccgagatcag<br>aggtccctaaggggaagaggcaatactc	-	-
CY090881	Human H3N2 IAVs	Human	H3N2		2010	Australia	A/Sydney/DD2_02/2010	agtgatgccccattccttgatcggcttcgccgagatcag<br>aggtccctaaggggaagaggcaatactc	-	-
CY160316	Human H3N2 IAVs	Human	H3N2		2010	Peru	A/Peru/PER004/2010	agtgatgccccattccttgatcggcttcgccgagatcag<br>aggtccctaaggggaagaggcaatactc	-	-
CY160324	Human H3N2 IAVs	Human	H3N2		2010	Peru	A/Peru/PER005/2010	agtgatgccccattccttgatcggcttcgccgagatcag<br>aggtccctaaggggaagaggcaatactc	-	-
CY160340	Human H3N2 IAVs	Human	H3N2		2010	Peru	A/Peru/PER007/2010	agtgatgccccattccttgatcggcttcgccgagatcag<br>aggtccctaaggggaagaggcaatactc	-	-
CY160380	Human H3N2 IAVs	Human	H3N2		2010	Peru	A/Peru/PER012/2010	agtgatgccccattccttgatcggcttcgccgagatcag<br>aggtccctaaggggaagaggcaatactc	-	-

CY160460	Human H3N2 IAVs	Human	H3N2		2010	Peru	A/Peru/PER022/2010	agtgatgccccattccttgatcggcttcgccgagatcag aggtccctaaggggaagaggcaatactc	-	-
CY160468	Human H3N2 IAVs	Human	H3N2		2010	Peru	A/Peru/PER023/2010	agtgatgccccattccttgatcggcttcgccgagatcag aggtccctaaggggaagaggcaatactc	-	-
CY160476	Human H3N2 IAVs	Human	H3N2		2010	Peru	A/Peru/PER024/2010	agtgatgccccattccttgatcggcttcgccgagatcag aggtccctaaggggaagaggcaatactc	-	-
CY160484	Human H3N2 IAVs	Human	H3N2		2010	Peru	A/Peru/PER025/2010	agtgatgccccattccttgatcggcttcgccgagatcag aggtccctaaggggaagaggcaatactc	-	-
CY160548	Human H3N2 IAVs	Human	H3N2		2010	Peru	A/Peru/PER033/2010	agtgatgccccattccttgatcggcttcgccgagatcag aggtccctaaggggaagaggcaatactc	-	-
CY160652	Human H3N2 IAVs	Human	H3N2		2010	Peru	A/Peru/PER046/2010	agtgatgccccattccttgatcggcttcgccgagatcag aggtccctaaggggaagaggcaatactc	-	-
CY160700	Human H3N2 IAVs	Human	H3N2		2010	Peru	A/Peru/PER052/2010	agtgatgccccattccttgatcggcttcgccgagatcag aggtccctaaggggaagaggcaatactc	-	-
CY160716	Human H3N2 IAVs	Human	H3N2		2010	Peru	A/Peru/PER054/2010	agtgatgccccattccttgatcggcttcgccgagatcag aggtccctaaggggaagaggcaatactc	-	-
CY160740	Human H3N2 IAVs	Human	H3N2		2010	Peru	A/Peru/PER057/2010	agtgatgccccattccttgatcggcttcgccgagatcag aggtccctaaggggaagaggcaatactc	-	-
CY160748	Human H3N2 IAVs	Human	H3N2		2010	Peru	A/Peru/PER058/2010	agtgatgccccattccttgatcggcttcgccgagatcag aggtccctaaggggaagaggcaatactc	-	-
CY160756	Human H3N2 IAVs	Human	H3N2		2010	Peru	A/Peru/PER059/2010	agtgatgccccattccttgatcggcttcgccgagatcag aggtccctaaggggaagaggcaatactc	-	-
CY160764	Human H3N2 IAVs	Human	H3N2		2010	Peru	A/Peru/PER060/2010	agtgatgccccattccttgatcggcttcgccgagatcag aggtccctaaggggaagaggcaatactc	-	-
CY160828	Human H3N2 IAVs	Human	H3N2		2010	Peru	A/Peru/PER068/2010	agtgatgccccattccttgatcggcttcgccgagatcag aggtccctaaggggaagaggcaatactc	-	-
CY160868	Human H3N2 IAVs	Human	H3N2		2010	Peru	A/Peru/PER073/2010	agtgatgccccattccttgatcggcttcgccgagatcag aggtccctaaggggaagaggcaatactc	-	-
CY160892	Human H3N2 IAVs	Human	H3N2		2010	Peru	A/Peru/PER076/2010	agtgatgccccattccttgatcggcttcgccgagatcag aggtccctaaggggaagaggcaatactc	-	-
CY160948	Human H3N2 IAVs	Human	H3N2		2010	Peru	A/Peru/PER083/2010	agtgatgccccattccttgatcggcttcgccgagatcag aggtccctaaggggaagaggcaatactc	-	-
CY160956	Human H3N2 IAVs	Human	H3N2		2010	Peru	A/Peru/PER084/2010	agtgatgccccattccttgatcggcttcgccgagatcag aggtccctaaggggaagaggcaatactc	-	-
CY161020	Human H3N2 IAVs	Human	H3N2		2010	Peru	A/Peru/PER093/2010	agtgatgccccattccttgatcggcttcgccgagatcag aggtccctaaggggaagaggcaatactc	-	-
CY161108	Human H3N2 IAVs	Human	H3N2		2010	Peru	A/Peru/PER104/2010	agtgatgccccattccttgatcggcttcgccgagatcag aggtccctaaggggaagaggcaatactc	-	-
CY161132	Human H3N2 IAVs	Human	H3N2		2010	Peru	A/Peru/PER107/2010	agtgatgccccattccttgatcggcttcgccgagatcag aggtccctaaggggaagaggcaatactc	-	-
CY161236	Human H3N2 IAVs	Human	H3N2		2010	Peru	A/Peru/PER120/2010	agtgatgccccattccttgatcggcttcgccgagatcag aggtccctaaggggaagaggcaatactc	-	-
CY161332	Human H3N2 IAVs	Human	H3N2		2010	Peru	A/Peru/PER133/2010	agtgatgccccattccttgatcggcttcgccgagatcag aggtccctaaggggaagaggcaatactc	-	-
CY161444	Human H3N2 IAVs	Human	H3N2		2010	Peru	A/Peru/PER147/2010	agtgatgccccattccttgatcggcttcgccgagatcag aggtccctaaggggaagaggcaatactc	-	-
CY161500	Human H3N2 IAVs	Human	H3N2		2010	Peru	A/Peru/PER154/2010	agtgatgccccattccttgatcggcttcgccgagatcag aggtccctaaggggaagaggcaatactc	-	-
CY161524	Human H3N2 IAVs	Human	H3N2		2010	Peru	A/Peru/PER157/2010	agtgatgccccattccttgatcggcttcgccgagatcag aggtccctaaggggaagaggcaatactc	-	-

CY161692	Human H3N2 IAVs	Human	H3N2		2010	Peru	A/Peru/PER178/2010	agtgatgccccattccttgatcggcttcgccgagatcag<br>aggtccctaaggggaagaggcaatactc	-	-
CY161740	Human H3N2 IAVs	Human	H3N2		2010	Peru	A/Peru/PER184/2010	agtgatgccccattccttgatcggcttcgccgagatcag<br>aggtccctaaggggaagaggcaatactc	-	-
CY161788	Human H3N2 IAVs	Human	H3N2		2010	Peru	A/Peru/PER190/2010	agtgatgccccattccttgatcggcttcgccgagatcag<br>aggtccctaaggggaagaggcaatactc	-	-
CY161812	Human H3N2 IAVs	Human	H3N2		2010	Peru	A/Peru/PER193/2010	agtgatgccccattccttgatcggcttcgccgagatcag<br>aggtccctaaggggaagaggcaatactc	-	-
CY161828	Human H3N2 IAVs	Human	H3N2		2010	Peru	A/Peru/PER195/2010	agtgatgccccattccttgatcggcttcgccgagatcag<br>aggtccctaaggggaagaggcaatactc	-	-
CY161836	Human H3N2 IAVs	Human	H3N2		2010	Peru	A/Peru/PER196/2010	agtgatgccccattccttgatcggcttcgccgagatcag<br>aggtccctaaggggaagaggcaatactc	-	-
CY161948	Human H3N2 IAVs	Human	H3N2		2010	Peru	A/Peru/PER210/2010	agtgatgccccattccttgatcggcttcgccgagatcag<br>aggtccctaaggggaagaggcaatactc	-	-
CY161988	Human H3N2 IAVs	Human	H3N2		2010	Peru	A/Peru/PER215/2010	agtgatgccccattccttgatcggcttcgccgagatcag<br>aggtccctaaggggaagaggcaatactc	-	-
CY162092	Human H3N2 IAVs	Human	H3N2		2010	Peru	A/Peru/PER228/2010	agtgatgccccattccttgatcggcttcgccgagatcag<br>aggtccctaaggggaagaggcaatactc	-	-
CY162180	Human H3N2 IAVs	Human	H3N2		2010	Peru	A/Peru/PER239/2010	agtgatgccccattccttgatcggcttcgccgagatcag<br>aggtccctaaggggaagaggcaatactc	-	-
CY162276	Human H3N2 IAVs	Human	H3N2		2010	Peru	A/Peru/PER253/2010	agtgatgccccattccttgatcggcttcgccgagatcag<br>aggtccctaaggggaagaggcaatactc	-	-
CY162620	Human H3N2 IAVs	Human	H3N2		2010	Peru	A/Peru/PER299/2010	agtgatgccccattccttgatcggcttcgccgagatcag<br>aggtccctaaggggaagaggcaatactc	-	-
CY162916	Human H3N2 IAVs	Human	H3N2		2010	Peru	A/Peru/PER336/2010	agtgatgccccattccttgatcggcttcgccgagatcag<br>aggtccctaaggggaagaggcaatactc	-	-
CY163020	Human H3N2 IAVs	Human	H3N2		2010	Peru	A/Peru/PER350/2010	agtgatgccccattccttgatcggcttcgccgagatcag<br>aggtccctaaggggaagaggcaatactc	-	-
CY163180	Human H3N2 IAVs	Human	H3N2		2010	Peru	A/Peru/PER372/2010	agtgatgccccattccttgatcggcttcgccgagatcag<br>aggtccctaaggggaagaggcaatactc	-	-
CY162228	Human H3N2 IAVs	Human	H3N2		2010	Peru	A/Peru/PER246/2010	agtgatgccccattccttgatcggcttcgccgagatcag<br>aggtccctaaggggaagaggcaatactc	-	-
CY162316	Human H3N2 IAVs	Human	H3N2		2010	Peru	A/Peru/PER258/2010	agtgatgccccattccttgatcggcttcgccgagatcag<br>aggtccctaaggggaagaggcaatactc	-	-
CY163268	Human H3N2 IAVs	Human	H3N2		2010	Peru	A/Peru/PER383/2010	agtgatgccccattccttgatcggcttcgccgagatcag<br>aggtccctaaggggaagaggcaatactc	-	-
CY161468	Human H3N2 IAVs	Human	H3N2		2010	Peru	A/Peru/PER150/2010	agtgatgccccattccttgatcggcttcgccgagatcag<br>aggtccctaaggggaagaggcaatactc	-	-
CY114513	Human H3N2 IAVs	Human	H3N2		2010	Netherlands	A/Netherlands/034/2010	agtgatgccccattccttgatcggcttcgccgagatcag<br>aggtccctaaggggaagaggcaatactc	-	-
CY160804	Human H3N2 IAVs	Human	H3N2		2010	Peru	A/Peru/PER065/2010	agtgatgccccattccttgatcggcttcgccgagatcag<br>aggtccctaaggggaagaggcaatactc	-	-
JX437772	Human H3N2 IAVs	Human	H3N2		2010	Singapore	A/Singapore/C2010.036V/2010	agtgatgccccattccttgatcggcttcgccgagatcag<br>aggtccctaaggggaagaggcaatactc	-	-
KM070044	Human H3N2 IAVs	Human	H3N2		2010	Singapore	A/Singapore/C2010.307/2010	agtgatgccccattccttgatcggcttcgccgagatcag<br>aggtccctaaggggaagaggcaatactc	-	-
KM070046	Human H3N2 IAVs	Human	H3N2		2010	Singapore	A/Singapore/H2010.310/2010	agtgatgccccattccttgatcggcttcgccgagatcag<br>aggtccctaaggggaagaggcaatactc	-	-
KF014696	Human H3N2 IAVs	Human	H3N2		2010	Singapore	A/Singapore/H2010.370C/2010	agtgatgccccattccttgatcggcttcgccgagatcag<br>aggtccctaaggggaagaggcaatactc	-	-

JX437892	Human H3N2 IAVs	Human	H3N2		2010	Singapore	A/Singapore/H2010.370C/2010	agtgatgccccattccttgatcggcttcgccgagatcag aggtccctaaggggaagaggcaatactc	-	-
KF014698	Human H3N2 IAVs	Human	H3N2		2010	Singapore	A/Singapore/H2010.389/2010	agtgatgccccattccttgatcggcttcgccgagatcag aggtccctaaggggaagaggcaatactc	-	-
KF014769	Human H3N2 IAVs	Human	H3N2		2010	Singapore	A/Singapore/H2010.389/2010	agtgatgccccattccttgatcggcttcgccgagatcag aggtccctaaggggaagaggcaatactc	-	-
KF014699	Human H3N2 IAVs	Human	H3N2		2010	Singapore	A/Singapore/H2010.471C/2010	agtgatgccccattccttgatcggcttcgccgagatcag aggtccctaaggggaagaggcaatactc	-	-
JX437893	Human H3N2 IAVs	Human	H3N2		2010	Singapore	A/Singapore/H2010.471C/2010	agtgatgccccattccttgatcggcttcgccgagatcag aggtccctaaggggaagaggcaatactc	-	-
KM070058	Human H3N2 IAVs	Human	H3N2		2010	Singapore	A/Singapore/S2010.416a/2010	agtgatgccccattccttgatcggcttcgccgagatcag aggtccctaaggggaagaggcaatactc	-	-
JX437774	Human H3N2 IAVs	Human	H3N2		2010	Singapore	A/Singapore/C2010.362V/2010	agtgatgccccattccttgatcggcttcgccgagatcag aggtccctaaggggaagaggcaatactc	-	-
CY162748	Human H3N2 IAVs	Human	H3N2		2010	Peru	A/Peru/PER315/2010	agtgatgccccattccttgatcggcttcgccgagatcag aggtccctaaggggaagaggcaatactc	-	-
KC883230	Human H3N2 IAVs	Human	H3N2		2010	USA	A/Massachusetts/06/2010	agtgatgccccattccttgatcggcttcgccgagatcag aggtccctaaggggaagaggcaatactc	-	-
CY072218	Human H3N2 IAVs	Human	H3N2		2010	USA	A/New_York/20343/2010	agtgatgccccattccttgatcggcttcgccgagatcag aggtccctaaggggaagaggcaatactc	-	-
KM070055	Human H3N2 IAVs	Human	H3N2		2010	Singapore	A/Singapore/C2010.384/2010	agtgatgccccattccttgatcggcttcgccgagatcag aggtccctaaggggaagaggcaatactc	-	-
JX437775	Human H3N2 IAVs	Human	H3N2		2010	Singapore	A/Singapore/C2010.471aV/2010	agtgatgccccattccttgatcggcttcgccgagatcag aggtccctaaggggaagaggcaatactc	-	-
KF014767	Human H3N2 IAVs	Human	H3N2		2010	Singapore	A/Singapore/H2010.301/2010	agtgatgccccattccttgatcggcttcgccgagatcag aggtccctaaggggaagaggcaatactc	-	-
KF014694	Human H3N2 IAVs	Human	H3N2		2010	Singapore	A/Singapore/H2010.301/2010	agtgatgccccattccttgatcggcttcgccgagatcag aggtccctaaggggaagaggcaatactc	-	-
KF014695	Human H3N2 IAVs	Human	H3N2		2010	Singapore	A/Singapore/H2010.321C/2010	agtgatgccccattccttgatcggcttcgccgagatcag aggtccctaaggggaagaggcaatactc	-	-
JX437891	Human H3N2 IAVs	Human	H3N2		2010	Singapore	A/Singapore/H2010.321C/2010	agtgatgccccattccttgatcggcttcgccgagatcag aggtccctaaggggaagaggcaatactc	-	-
KM070056	Human H3N2 IAVs	Human	H3N2		2010	Singapore	A/Singapore/H2010.400/2010	agtgatgccccattccttgatcggcttcgccgagatcag aggtccctaaggggaagaggcaatactc	-	-
JX437896	Human H3N2 IAVs	Human	H3N2		2010	Singapore	A/Singapore/H2010.666C/2010	agtgatgccccattccttgatcggcttcgccgagatcag aggtccctaaggggaagaggcaatactc	-	-
KF014703	Human H3N2 IAVs	Human	H3N2		2010	Singapore	A/Singapore/H2010.666C/2010	agtgatgccccattccttgatcggcttcgccgagatcag aggtccctaaggggaagaggcaatactc	-	-
KM070049	Human H3N2 IAVs	Human	H3N2		2010	Singapore	A/Singapore/S2010.345b/2010	agtgatgccccattccttgatcggcttcgccgagatcag aggtccctaaggggaagaggcaatactc	-	-
KM070052	Human H3N2 IAVs	Human	H3N2		2010	Singapore	A/Singapore/S2010.359a/2010	agtgatgccccattccttgatcggcttcgccgagatcag aggtccctaaggggaagaggcaatactc	-	-
KM070062	Human H3N2 IAVs	Human	H3N2		2010	Singapore	A/Singapore/C2010.471b/2010	agtgatgccccattccttgatcggcttcgccgagatcag aggtccctaaggggaagaggcaatactc	-	-
CY121804	Human H3N2 IAVs	Human	H3N2		2010	USA	A/Rhode_Island/01/2010	agtgatgccccattccttgatcggcttcgccgagatcag aggtccctaaggggaagaggcaatactc	-	-
KM070041	Human H3N2 IAVs	Human	H3N2		2010	Singapore	A/Singapore/C2010.049/2010	agtgatgccccattccttgatcggcttcgccgagatcag aggtccctaaggggaagaggcaatactc	-	-
CY090873	Human H3N2 IAVs	Human	H3N2		2010	Australia	A/Sydney/DD2_01/2010	agtgatgccccattccttgatcggcttcgccgagatcag aggtccctaaggggaagaggcaatactc	-	-

JX437773	Human H3N2 IAVs	Human	H3N2		2010	Singapore	A/Singapore/C2010.304V/2010	agtgatgccccattccttgatcggcttcgccgagatcag aggtccctaaggggaagaggcaatactc	-	-
KM070068	Human H3N2 IAVs	Human	H3N2		2010	Singapore	A/Singapore/C2010.614/2010	agtgatgccccattccttgatcggcttcgccgagatcag aggtccctaaggggaagaggcaatactc	-	-
CY074847	Human H3N2 IAVs	Human	H3N2		2010	Nicaragua	A/Managua/1166.03/2010	agtgatgccccattccttgatcggcttcgccgagatcag aggtccctaaggggaagaggcaatactc	-	-
CY074751	Human H3N2 IAVs	Human	H3N2		2010	Nicaragua	A/Managua/1167.03/2010	agtgatgccccattccttgatcggcttcgccgagatcag aggtccctaaggggaagaggcaatactc	-	-
CY070955	Human H3N2 IAVs	Human	H3N2		2010	Nicaragua	A/Managua/1181.03/2010	agtgatgccccattccttgatcggcttcgccgagatcag aggtccctaaggggaagaggcaatactc	-	-
CY088895	Human H3N2 IAVs	Human	H3N2		2010	Nicaragua	A/Managua/1260.02/2010	agtgatgccccattccttgatcggcttcgccgagatcag aggtccctaaggggaagaggcaatactc	-	-
CY074783	Human H3N2 IAVs	Human	H3N2		2010	Nicaragua	A/Managua/1535.03/2010	agtgatgccccattccttgatcggcttcgccgagatcag aggtccctaaggggaagaggcaatactc	-	-
CY098085	Human H3N2 IAVs	Human	H3N2		2010	Nicaragua	A/Managua/1645.03/2010	agtgatgccccattccttgatcggcttcgccgagatcag aggtccctaaggggaagaggcaatactc	-	-
CY074799	Human H3N2 IAVs	Human	H3N2		2010	Nicaragua	A/Managua/1784.03/2010	agtgatgccccattccttgatcggcttcgccgagatcag aggtccctaaggggaagaggcaatactc	-	-
CY070947	Human H3N2 IAVs	Human	H3N2		2010	Nicaragua	A/Managua/1807.03/2010	agtgatgccccattccttgatcggcttcgccgagatcag aggtccctaaggggaagaggcaatactc	-	-
CY074767	Human H3N2 IAVs	Human	H3N2		2010	Nicaragua	A/Managua/1856.03/2010	agtgatgccccattccttgatcggcttcgccgagatcag aggtccctaaggggaagaggcaatactc	-	-
CY074735	Human H3N2 IAVs	Human	H3N2		2010	Nicaragua	A/Managua/194.01/2010	agtgatgccccattccttgatcggcttcgccgagatcag aggtccctaaggggaagaggcaatactc	-	-
CY088919	Human H3N2 IAVs	Human	H3N2		2010	Nicaragua	A/Managua/2145.01/2010	agtgatgccccattccttgatcggcttcgccgagatcag aggtccctaaggggaagaggcaatactc	-	-
CY074919	Human H3N2 IAVs	Human	H3N2		2010	Nicaragua	A/Managua/2614.02/2010	agtgatgccccattccttgatcggcttcgccgagatcag aggtccctaaggggaagaggcaatactc	-	-
CY088855	Human H3N2 IAVs	Human	H3N2		2010	Nicaragua	A/Managua/2654.01/2010	agtgatgccccattccttgatcggcttcgccgagatcag aggtccctaaggggaagaggcaatactc	-	-
CY070963	Human H3N2 IAVs	Human	H3N2		2010	Nicaragua	A/Managua/2867.01/2010	agtgatgccccattccttgatcggcttcgccgagatcag aggtccctaaggggaagaggcaatactc	-	-
CY074743	Human H3N2 IAVs	Human	H3N2		2010	Nicaragua	A/Managua/2912.04/2010	agtgatgccccattccttgatcggcttcgccgagatcag aggtccctaaggggaagaggcaatactc	-	-
CY088911	Human H3N2 IAVs	Human	H3N2		2010	Nicaragua	A/Managua/3039.01/2010	agtgatgccccattccttgatcggcttcgccgagatcag aggtccctaaggggaagaggcaatactc	-	-
CY074703	Human H3N2 IAVs	Human	H3N2		2010	Nicaragua	A/Managua/3192.01/2010	agtgatgccccattccttgatcggcttcgccgagatcag aggtccctaaggggaagaggcaatactc	-	-
CY088975	Human H3N2 IAVs	Human	H3N2		2010	Nicaragua	A/Managua/3209.01/2010	agtgatgccccattccttgatcggcttcgccgagatcag aggtccctaaggggaagaggcaatactc	-	-
CY088847	Human H3N2 IAVs	Human	H3N2		2010	Nicaragua	A/Managua/3345.02/2010	agtgatgccccattccttgatcggcttcgccgagatcag aggtccctaaggggaagaggcaatactc	-	-
CY088983	Human H3N2 IAVs	Human	H3N2		2010	Nicaragua	A/Managua/3408.02/2010	agtgatgccccattccttgatcggcttcgccgagatcag aggtccctaaggggaagaggcaatactc	-	-
CY074831	Human H3N2 IAVs	Human	H3N2		2010	Nicaragua	A/Managua/3433.02/2010	agtgatgccccattccttgatcggcttcgccgagatcag aggtccctaaggggaagaggcaatactc	-	-
CY074879	Human H3N2 IAVs	Human	H3N2		2010	Nicaragua	A/Managua/3527.02/2010	agtgatgccccattccttgatcggcttcgccgagatcag aggtccctaaggggaagaggcaatactc	-	-
CY074895	Human H3N2 IAVs	Human	H3N2		2010	Nicaragua	A/Managua/3570.04/2010	agtgatgccccattccttgatcggcttcgccgagatcag aggtccctaaggggaagaggcaatactc	-	-

CY098077	Human H3N2 IAVs	Human	H3N2		2010	Nicaragua	A/Managua/3587.02/2010	agtgatgccccattccttgatcggcttcgccgagatcag aggtccctaaggggaagaggcaatactc	-	-
CY088959	Human H3N2 IAVs	Human	H3N2		2010	Nicaragua	A/Managua/3622.02/2010	agtgatgccccattccttgatcggcttcgccgagatcag aggtccctaaggggaagaggcaatactc	-	-
CY088991	Human H3N2 IAVs	Human	H3N2		2010	Nicaragua	A/Managua/3659.02/2010	agtgatgccccattccttgatcggcttcgccgagatcag aggtccctaaggggaagaggcaatactc	-	-
CY088871	Human H3N2 IAVs	Human	H3N2		2010	Nicaragua	A/Managua/3704.06/2010	agtgatgccccattccttgatcggcttcgccgagatcag aggtccctaaggggaagaggcaatactc	-	-
CY074807	Human H3N2 IAVs	Human	H3N2		2010	Nicaragua	A/Managua/3715.02/2010	agtgatgccccattccttgatcggcttcgccgagatcag aggtccctaaggggaagaggcaatactc	-	-
CY088943	Human H3N2 IAVs	Human	H3N2		2010	Nicaragua	A/Managua/38.01/2010	agtgatgccccattccttgatcggcttcgccgagatcag aggtccctaaggggaagaggcaatactc	-	-
CY074711	Human H3N2 IAVs	Human	H3N2		2010	Nicaragua	A/Managua/4009.02/2010	agtgatgccccattccttgatcggcttcgccgagatcag aggtccctaaggggaagaggcaatactc	-	-
CY089007	Human H3N2 IAVs	Human	H3N2		2010	Nicaragua	A/Managua/4076.04/2010	agtgatgccccattccttgatcggcttcgccgagatcag aggtccctaaggggaagaggcaatactc	-	-
CY074839	Human H3N2 IAVs	Human	H3N2		2010	Nicaragua	A/Managua/410.02/2010	agtgatgccccattccttgatcggcttcgccgagatcag aggtccctaaggggaagaggcaatactc	-	-
CY074871	Human H3N2 IAVs	Human	H3N2		2010	Nicaragua	A/Managua/4134.04/2010	agtgatgccccattccttgatcggcttcgccgagatcag aggtccctaaggggaagaggcaatactc	-	-
CY074903	Human H3N2 IAVs	Human	H3N2		2010	Nicaragua	A/Managua/4203.02/2010	agtgatgccccattccttgatcggcttcgccgagatcag aggtccctaaggggaagaggcaatactc	-	-
CY074823	Human H3N2 IAVs	Human	H3N2		2010	Nicaragua	A/Managua/4218.02/2010	agtgatgccccattccttgatcggcttcgccgagatcag aggtccctaaggggaagaggcaatactc	-	-
CY088887	Human H3N2 IAVs	Human	H3N2		2010	Nicaragua	A/Managua/4355.03/2010	agtgatgccccattccttgatcggcttcgccgagatcag aggtccctaaggggaagaggcaatactc	-	-
CY089015	Human H3N2 IAVs	Human	H3N2		2010	Nicaragua	A/Managua/4604.04/2010	agtgatgccccattccttgatcggcttcgccgagatcag aggtccctaaggggaagaggcaatactc	-	-
CY089031	Human H3N2 IAVs	Human	H3N2		2010	Nicaragua	A/Managua/4855.03/2010	agtgatgccccattccttgatcggcttcgccgagatcag aggtccctaaggggaagaggcaatactc	-	-
CY074863	Human H3N2 IAVs	Human	H3N2		2010	Nicaragua	A/Managua/506.02/2010	agtgatgccccattccttgatcggcttcgccgagatcag aggtccctaaggggaagaggcaatactc	-	-
CY088879	Human H3N2 IAVs	Human	H3N2		2010	Nicaragua	A/Managua/5099.03/2010	agtgatgccccattccttgatcggcttcgccgagatcag aggtccctaaggggaagaggcaatactc	-	-
CY088778	Human H3N2 IAVs	Human	H3N2		2010	Nicaragua	A/Managua/511.03/2010	agtgatgccccattccttgatcggcttcgccgagatcag aggtccctaaggggaagaggcaatactc	-	-
CY074911	Human H3N2 IAVs	Human	H3N2		2010	Nicaragua	A/Managua/5259.02/2010	agtgatgccccattccttgatcggcttcgccgagatcag aggtccctaaggggaagaggcaatactc	-	-
CY088863	Human H3N2 IAVs	Human	H3N2		2010	Nicaragua	A/Managua/5271.04/2010	agtgatgccccattccttgatcggcttcgccgagatcag aggtccctaaggggaagaggcaatactc	-	-
CY088794	Human H3N2 IAVs	Human	H3N2		2010	Nicaragua	A/Managua/5427.01/2010	agtgatgccccattccttgatcggcttcgccgagatcag aggtccctaaggggaagaggcaatactc	-	-
CY088951	Human H3N2 IAVs	Human	H3N2		2010	Nicaragua	A/Managua/5444.02/2010	agtgatgccccattccttgatcggcttcgccgagatcag aggtccctaaggggaagaggcaatactc	-	-
CY074855	Human H3N2 IAVs	Human	H3N2		2010	Nicaragua	A/Managua/5543.01/2010	agtgatgccccattccttgatcggcttcgccgagatcag aggtccctaaggggaagaggcaatactc	-	-
CY088786	Human H3N2 IAVs	Human	H3N2		2010	Nicaragua	A/Managua/5664.02/2010	agtgatgccccattccttgatcggcttcgccgagatcag aggtccctaaggggaagaggcaatactc	-	-
CY074815	Human H3N2 IAVs	Human	H3N2		2010	Nicaragua	A/Managua/5674.05/2010	agtgatgccccattccttgatcggcttcgccgagatcag aggtccctaaggggaagaggcaatactc	-	-

CY093121	Human H3N2 IAVs	Human	H3N2		2010	Nicaragua	A/Managua/5744.03/2010	agtgatgccccattccttgatcggcttcgccgagatcag<br>aggtccctaaggggaagaggcaatactc	-	-
CY088927	Human H3N2 IAVs	Human	H3N2		2010	Nicaragua	A/Managua/5801.02/2010	agtgatgccccattccttgatcggcttcgccgagatcag<br>aggtccctaaggggaagaggcaatactc	-	-
CY098069	Human H3N2 IAVs	Human	H3N2		2010	Nicaragua	A/Managua/5828.02/2010	agtgatgccccattccttgatcggcttcgccgagatcag<br>aggtccctaaggggaagaggcaatactc	-	-
CY074887	Human H3N2 IAVs	Human	H3N2		2010	Nicaragua	A/Managua/5863.02/2010	agtgatgccccattccttgatcggcttcgccgagatcag<br>aggtccctaaggggaagaggcaatactc	-	-
CY074687	Human H3N2 IAVs	Human	H3N2		2010	Nicaragua	A/Managua/5906.01/2010	agtgatgccccattccttgatcggcttcgccgagatcag<br>aggtccctaaggggaagaggcaatactc	-	-
CY103795	Human H3N2 IAVs	Human	H3N2		2010	Nicaragua	A/Managua/615.02/2010	agtgatgccccattccttgatcggcttcgccgagatcag<br>aggtccctaaggggaagaggcaatactc	-	-
CY074759	Human H3N2 IAVs	Human	H3N2		2010	Nicaragua	A/Managua/80.1/2010	agtgatgccccattccttgatcggcttcgccgagatcag<br>aggtccctaaggggaagaggcaatactc	-	-
CY074791	Human H3N2 IAVs	Human	H3N2		2010	Nicaragua	A/Managua/90.01/2010	agtgatgccccattccttgatcggcttcgccgagatcag<br>aggtccctaaggggaagaggcaatactc	-	-
CY074727	Human H3N2 IAVs	Human	H3N2		2010	Nicaragua	A/Managua/905.02/2010	agtgatgccccattccttgatcggcttcgccgagatcag<br>aggtccctaaggggaagaggcaatactc	-	-
KM070048	Human H3N2 IAVs	Human	H3N2		2010	Singapore	A/Singapore/C2010.345/2010	agtgatgccccattccttgatcggcttcgccgagatcag<br>aggtccctaaggggaagaggcaatactc	-	-
CY074927	Human H3N2 IAVs	Human	H3N2		2010	Nicaragua	A/Managua/4456.02/2010	agtgatgccccattccttgatcggcttcgccgagatcag<br>aggtccctaaggggaagaggcaatactc	-	-
CY088967	Human H3N2 IAVs	Human	H3N2		2010	Nicaragua	A/Managua/5898.02/2010	agtgatgccccattccttgatcggcttcgccgagatcag<br>aggtccctaaggggaagaggcaatactc	-	-
CY074775	Human H3N2 IAVs	Human	H3N2		2010	Nicaragua	A/Managua/3244.02/2010	agtgatgccccattccttgatcggcttcgccgagatcag<br>aggtccctaaggggaagaggcaatactc	-	-
CY070939	Human H3N2 IAVs	Human	H3N2		2010	Nicaragua	A/Managua/4131.04/2010	agtgatgccccattccttgatcggcttcgccgagatcag<br>aggtccctaaggggaagaggcaatactc	-	-
CY088999	Human H3N2 IAVs	Human	H3N2		2010	Nicaragua	A/Managua/1685.01/2010	agtgatgccccattccttgatcggcttcgccgagatcag<br>aggtccctaaggggaagaggcaatactc	-	-
CY074935	Human H3N2 IAVs	Human	H3N2		2010	Nicaragua	A/Managua/305_10/2010	agtgatgccccattccttgatcggcttcgccgagatcag<br>aggtccctaaggggaagaggcaatactc	-	-
CY070931	Human H3N2 IAVs	Human	H3N2		2010	Nicaragua	A/Managua/5806.01/2010	agtgatgccccattccttgatcggcttcgccgagatcag<br>aggtccctaaggggaagaggcaatactc	-	-
CY089023	Human H3N2 IAVs	Human	H3N2		2010	Nicaragua	A/Managua/5870.02/2010	agtgatgccccattccttgatcggcttcgccgagatcag<br>aggtccctaaggggaagaggcaatactc	-	-
CY093451	Human H3N2 IAVs	Human	H3N2		2010	El_Salvador	A/San_Salvador/WRAIR3537T/201<br>0	agtgatgccccattccttgatcggcttcgccgagatcag<br>aggtccctaaggggaagaggcaatactc	-	-
CY074679	Human H3N2 IAVs	Human	H3N2		2010	Nicaragua	A/Managua/395.02/2010	agtgatgccccattccttgatcggcttcgccgagatcag<br>aggtccctaaggggaagaggcaatactc	-	-
CY074695	Human H3N2 IAVs	Human	H3N2		2010	Nicaragua	A/Managua/4763.03/2010	agtgatgccccattccttgatcggcttcgccgagatcag<br>aggtccctaaggggaagaggcaatactc	-	-
CY074719	Human H3N2 IAVs	Human	H3N2		2010	Nicaragua	A/Managua/5871.02/2010	agtgatgccccattccttgatcggcttcgccgagatcag<br>aggtccctaaggggaagaggcaatactc	-	-
CY093228	Human H3N2 IAVs	Human	H3N2		2010	USA	A/District_of_Columbia/WRAIR030<br>0/2010	agtgatgccccattccttgatcggcttcgccgagatcag<br>aggtccctaaggggaagaggcaatactc	-	-
CY160708	Human H3N2 IAVs	Human	H3N2		2010	Peru	A/Peru/PER053/2010	agtgatgccccattccttgatcggcttcgccgagatcag<br>aggtccctaaggggaagaggcaatactc	-	-
CY161684	Human H3N2 IAVs	Human	H3N2		2010	Peru	A/Peru/PER177/2010	agtgatgccccattccttgatcggcttcgccgagatcag<br>aggtccctaaggggaagaggcaatactc	-	-

CY162052	Human H3N2 IAVs	Human	H3N2		2010	Peru	A/Peru/PER223/2010	agtgatgccccattccttgatcggcttcgccgagatcag<br>aggtccctaaggggaagaggcaatactc	-	-
CY160308	Human H3N2 IAVs	Human	H3N2		2010	Peru	A/Peru/PER003/2010	agtgatgccccattccttgatcggcttcgccgagatcag<br>aggtccctaaggggaagaggcaatactc	-	-
CY160348	Human H3N2 IAVs	Human	H3N2		2010	Peru	A/Peru/PER008/2010	agtgatgccccattccttgatcggcttcgccgagatcag<br>aggtccctaaggggaagaggcaatactc	-	-
CY160388	Human H3N2 IAVs	Human	H3N2		2010	Peru	A/Peru/PER013/2010	agtgatgccccattccttgatcggcttcgccgagatcag<br>aggtccctaaggggaagaggcaatactc	-	-
CY160436	Human H3N2 IAVs	Human	H3N2		2010	Peru	A/Peru/PER019/2010	agtgatgccccattccttgatcggcttcgccgagatcag<br>aggtccctaaggggaagaggcaatactc	-	-
CY160452	Human H3N2 IAVs	Human	H3N2		2010	Peru	A/Peru/PER021/2010	agtgatgccccattccttgatcggcttcgccgagatcag<br>aggtccctaaggggaagaggcaatactc	-	-
CY160492	Human H3N2 IAVs	Human	H3N2		2010	Peru	A/Peru/PER026/2010	agtgatgccccattccttgatcggcttcgccgagatcag<br>aggtccctaaggggaagaggcaatactc	-	-
CY160516	Human H3N2 IAVs	Human	H3N2		2010	Peru	A/Peru/PER029/2010	agtgatgccccattccttgatcggcttcgccgagatcag<br>aggtccctaaggggaagaggcaatactc	-	-
CY160524	Human H3N2 IAVs	Human	H3N2		2010	Peru	A/Peru/PER030/2010	agtgatgccccattccttgatcggcttcgccgagatcag<br>aggtccctaaggggaagaggcaatactc	-	-
CY160604	Human H3N2 IAVs	Human	H3N2		2010	Peru	A/Peru/PER040/2010	agtgatgccccattccttgatcggcttcgccgagatcag<br>aggtccctaaggggaagaggcaatactc	-	-
CY160684	Human H3N2 IAVs	Human	H3N2		2010	Peru	A/Peru/PER050/2010	agtgatgccccattccttgatcggcttcgccgagatcag<br>aggtccctaaggggaagaggcaatactc	-	-
CY160924	Human H3N2 IAVs	Human	H3N2		2010	Peru	A/Peru/PER080/2010	agtgatgccccattccttgatcggcttcgccgagatcag<br>aggtccctaaggggaagaggcaatactc	-	-
CY160996	Human H3N2 IAVs	Human	H3N2		2010	Peru	A/Peru/PER089/2010	agtgatgccccattccttgatcggcttcgccgagatcag<br>aggtccctaaggggaagaggcaatactc	-	-
CY161004	Human H3N2 IAVs	Human	H3N2		2010	Peru	A/Peru/PER090/2010	agtgatgccccattccttgatcggcttcgccgagatcag<br>aggtccctaaggggaagaggcaatactc	-	-
CY161204	Human H3N2 IAVs	Human	H3N2		2010	Peru	A/Peru/PER116/2010	agtgatgccccattccttgatcggcttcgccgagatcag<br>aggtccctaaggggaagaggcaatactc	-	-
CY161644	Human H3N2 IAVs	Human	H3N2		2010	Peru	A/Peru/PER172/2010	agtgatgccccattccttgatcggcttcgccgagatcag<br>aggtccctaaggggaagaggcaatactc	-	-
CY161652	Human H3N2 IAVs	Human	H3N2		2010	Peru	A/Peru/PER173/2010	agtgatgccccattccttgatcggcttcgccgagatcag<br>aggtccctaaggggaagaggcaatactc	-	-
CY161972	Human H3N2 IAVs	Human	H3N2		2010	Peru	A/Peru/PER213/2010	agtgatgccccattccttgatcggcttcgccgagatcag<br>aggtccctaaggggaagaggcaatactc	-	-
CY162012	Human H3N2 IAVs	Human	H3N2		2010	Peru	A/Peru/PER218/2010	agtgatgccccattccttgatcggcttcgccgagatcag<br>aggtccctaaggggaagaggcaatactc	-	-
CY162244	Human H3N2 IAVs	Human	H3N2		2010	Peru	A/Peru/PER248/2010	agtgatgccccattccttgatcggcttcgccgagatcag<br>aggtccctaaggggaagaggcaatactc	-	-
CY162260	Human H3N2 IAVs	Human	H3N2		2010	Peru	A/Peru/PER251/2010	agtgatgccccattccttgatcggcttcgccgagatcag<br>aggtccctaaggggaagaggcaatactc	-	-
CY162372	Human H3N2 IAVs	Human	H3N2		2010	Peru	A/Peru/PER266/2010	agtgatgccccattccttgatcggcttcgccgagatcag<br>aggtccctaaggggaagaggcaatactc	-	-
CY162404	Human H3N2 IAVs	Human	H3N2		2010	Peru	A/Peru/PER270/2010	agtgatgccccattccttgatcggcttcgccgagatcag<br>aggtccctaaggggaagaggcaatactc	-	-
CY162452	Human H3N2 IAVs	Human	H3N2		2010	Peru	A/Peru/PER276/2010	agtgatgccccattccttgatcggcttcgccgagatcag<br>aggtccctaaggggaagaggcaatactc	-	-
CY162460	Human H3N2 IAVs	Human	H3N2		2010	Peru	A/Peru/PER277/2010	agtgatgccccattccttgatcggcttcgccgagatcag<br>aggtccctaaggggaagaggcaatactc	-	-

CY162604	Human H3N2 IAVs	Human	H3N2		2010	Peru	A/Peru/PER297/2010	agtgatgccccattccttgatcggcttcgccgagatcag<br>aggtccctaaggggaagaggcaatactc	-	-
CY162692	Human H3N2 IAVs	Human	H3N2		2010	Peru	A/Peru/PER308/2010	agtgatgccccattccttgatcggcttcgccgagatcag<br>aggtccctaaggggaagaggcaatactc	-	-
CY162772	Human H3N2 IAVs	Human	H3N2		2010	Peru	A/Peru/PER318/2010	agtgatgccccattccttgatcggcttcgccgagatcag<br>aggtccctaaggggaagaggcaatactc	-	-
CY162788	Human H3N2 IAVs	Human	H3N2		2010	Peru	A/Peru/PER320/2010	agtgatgccccattccttgatcggcttcgccgagatcag<br>aggtccctaaggggaagaggcaatactc	-	-
CY162828	Human H3N2 IAVs	Human	H3N2		2010	Peru	A/Peru/PER325/2010	agtgatgccccattccttgatcggcttcgccgagatcag<br>aggtccctaaggggaagaggcaatactc	-	-
CY162844	Human H3N2 IAVs	Human	H3N2		2010	Peru	A/Peru/PER327/2010	agtgatgccccattccttgatcggcttcgccgagatcag<br>aggtccctaaggggaagaggcaatactc	-	-
CY162868	Human H3N2 IAVs	Human	H3N2		2010	Peru	A/Peru/PER330/2010	agtgatgccccattccttgatcggcttcgccgagatcag<br>aggtccctaaggggaagaggcaatactc	-	-
CY162964	Human H3N2 IAVs	Human	H3N2		2010	Peru	A/Peru/PER342/2010	agtgatgccccattccttgatcggcttcgccgagatcag<br>aggtccctaaggggaagaggcaatactc	-	-
CY163164	Human H3N2 IAVs	Human	H3N2		2010	Peru	A/Peru/PER370/2010	agtgatgccccattccttgatcggcttcgccgagatcag<br>aggtccctaaggggaagaggcaatactc	-	-
CY163204	Human H3N2 IAVs	Human	H3N2		2010	Peru	A/Peru/PER375/2010	agtgatgccccattccttgatcggcttcgccgagatcag<br>aggtccctaaggggaagaggcaatactc	-	-
CY162812	Human H3N2 IAVs	Human	H3N2		2010	Peru	A/Peru/PER323/2010	agtgatgccccattccttgatcggcttcgccgagatcag<br>aggtccctaaggggaagaggcaatactc	-	-
CY162932	Human H3N2 IAVs	Human	H3N2		2010	Peru	A/Peru/PER338/2010	agtgatgccccattccttgatcggcttcgccgagatcag<br>aggtccctaaggggaagaggcaatactc	-	-
CY160940	Human H3N2 IAVs	Human	H3N2		2010	Peru	A/Peru/PER082/2010	agtgatgccccattccttgatcggcttcgccgagatcag<br>aggtccctaaggggaagaggcaatactc	-	-
CY161036	Human H3N2 IAVs	Human	H3N2		2010	Peru	A/Peru/PER095/2010	agtgatgccccattccttgatcggcttcgccgagatcag<br>aggtccctaaggggaagaggcaatactc	-	-
CY161956	Human H3N2 IAVs	Human	H3N2		2010	Peru	A/Peru/PER211/2010	agtgatgccccattccttgatcggcttcgccgagatcag<br>aggtccctaaggggaagaggcaatactc	-	-
KM070061	Human H3N2 IAVs	Human	H3N2		2010	Singapore	A/Singapore/C2010.441/2010	agtgatgccccattccttgatcggcttcgccgagatcag<br>aggtccctaaggggaagaggcaatactc	-	-
CY121796	Human H3N2 IAVs	Human	H3N2		2010	Australia	A/Brisbane/11/2010	agtgatgccccattccttgatcggcttcgccgagatcag<br>aggtccctaaggggaagaggcaatactc	-	-
CY162660	Human H3N2 IAVs	Human	H3N2		2010	Peru	A/Peru/PER304/2010	agtgatgccccattccttgatcggcttcgccgagatcag<br>aggtccctaaggggaagaggcaatactc	-	-
KM070065	Human H3N2 IAVs	Human	H3N2		2010	Singapore	A/Singapore/S2010.570/2010	agtgatgccccattccttgatcggcttcgccgagatcag<br>aggtccctaaggggaagaggcaatactc	-	-
CY161172	Human H3N2 IAVs	Human	H3N2		2010	Peru	A/Peru/PER112/2010	agtgatgccccattccttgatcggcttcgccgagatcag<br>aggtccctaaggggaagaggcaatactc	-	-
KM070074	Human H3N2 IAVs	Human	H3N2		2010	Singapore	A/Singapore/C2010.663/2010	agtgatgccccattccttgatcggcttcgccgagatcag<br>aggtccctaaggggaagaggcaatactc	-	-
KM070073	Human H3N2 IAVs	Human	H3N2		2010	Singapore	A/Singapore/S2010.655b/2010	agtgatgccccattccttgatcggcttcgccgagatcag<br>aggtccctaaggggaagaggcaatactc	-	-
CY098192	Human H3N2 IAVs	Human	mixed		2010	Australia	A/Sydney/DD3_33/2010_mixed_	agtgatgccccattccttgatcggcttcgccgagatcag<br>aggtccctaaggggaagaggcaatactc	-	-
CY093411	Human H3N2 IAVs	Human	H3N2		2010	Colombia	A/Bogota/WRAIR3457N/2010	agtgatgccccattccttgatcggcttcgccgagatcag<br>aggtccctaaggggaagaggcaatactc	-	-
CY093419	Human H3N2 IAVs	Human	H3N2		2010	Colombia	A/Bogota/WRAIR3457T/2010	agtgatgccccattccttgatcggcttcgccgagatcag<br>aggtccctaaggggaagaggcaatactc	-	-

CY092317	Human H3N2 IAVs	Human	H3N2		2010	Nicaragua	A/Managua/3424.01/2010	agtgatgccccattccttgatcggcttcgccgagatcag aggtccctaaggggaagaggcaatactc	-	-
CY093475	Human H3N2 IAVs	Human	H3N2		2010	Mexico	A/Mexico_City/WRAIR3569N/2010	agtgatgccccattccttgatcggcttcgccgagatcag aggtccctaaggggaagaggcaatactc	-	-
CY093483	Human H3N2 IAVs	Human	H3N2		2010	Mexico	A/Mexico_City/WRAIR3569T/2010	agtgatgccccattccttgatcggcttcgccgagatcag aggtccctaaggggaagaggcaatactc	-	-
CY093539	Human H3N2 IAVs	Human	H3N2		2010	Mexico	A/Mexico_City/WRAIR3579N/2010	agtgatgccccattccttgatcggcttcgccgagatcag aggtccctaaggggaagaggcaatactc	-	-
CY093547	Human H3N2 IAVs	Human	H3N2		2010	Mexico	A/Mexico_City/WRAIR3579T/2010	agtgatgccccattccttgatcggcttcgccgagatcag aggtccctaaggggaagaggcaatactc	-	-
CY114505	Human H3N2 IAVs	Human	H3N2		2010	Netherlands	A/Netherlands/009/2010	agtgatgccccattccttgatcggcttcgccgagatcag aggtccctaaggggaagaggcaatactc	-	-
CY121500	Human H3N2 IAVs	Human	H3N2		2010	Australia	A/Perth/10/2010	agtgatgccccattccttgatcggcttcgccgagatcag aggtccctaaggggaagaggcaatactc	-	-
CY160988	Human H3N2 IAVs	Human	H3N2		2010	Peru	A/Peru/PER088/2010	agtgatgccccattccttgatcggcttcgccgagatcag aggtccctaaggggaagaggcaatactc	-	-
CY162028	Human H3N2 IAVs	Human	H3N2		2010	Peru	A/Peru/PER220/2010	agtgatgccccattccttgatcggcttcgccgagatcag aggtccctaaggggaagaggcaatactc	-	-
CY163244	Human H3N2 IAVs	Human	H3N2		2010	Peru	A/Peru/PER380/2010	agtgatgccccattccttgatcggcttcgccgagatcag aggtccctaaggggaagaggcaatactc	-	-
CY163348	Human H3N2 IAVs	Human	H3N2		2010	Peru	A/Peru/PER393/2010	agtgatgccccattccttgatcggcttcgccgagatcag aggtccctaaggggaagaggcaatactc	-	-
KM070042	Human H3N2 IAVs	Human	H3N2		2010	Singapore	A/Singapore/C2010.241/2010	agtgatgccccattccttgatcggcttcgccgagatcag aggtccctaaggggaagaggcaatactc	-	-
KM070045	Human H3N2 IAVs	Human	H3N2		2010	Singapore	A/Singapore/C2010.310/2010	agtgatgccccattccttgatcggcttcgccgagatcag aggtccctaaggggaagaggcaatactc	-	-
KM070060	Human H3N2 IAVs	Human	H3N2		2010	Singapore	A/Singapore/H2010.422/2010	agtgatgccccattccttgatcggcttcgccgagatcag aggtccctaaggggaagaggcaatactc	-	-
KF014702	Human H3N2 IAVs	Human	H3N2		2010	Singapore	A/Singapore/H2010.619/2010	agtgatgccccattccttgatcggcttcgccgagatcag aggtccctaaggggaagaggcaatactc	-	-
KF014770	Human H3N2 IAVs	Human	H3N2		2010	Singapore	A/Singapore/H2010.619/2010	agtgatgccccattccttgatcggcttcgccgagatcag aggtccctaaggggaagaggcaatactc	-	-
KF014771	Human H3N2 IAVs	Human	H3N2		2010	Singapore	A/Singapore/H2010.797/2010	agtgatgccccattccttgatcggcttcgccgagatcag aggtccctaaggggaagaggcaatactc	-	-
KF014704	Human H3N2 IAVs	Human	H3N2		2010	Singapore	A/Singapore/H2010.797/2010	agtgatgccccattccttgatcggcttcgccgagatcag aggtccctaaggggaagaggcaatactc	-	-
KM070054	Human H3N2 IAVs	Human	H3N2		2010	Singapore	A/Singapore/S2010.364/2010	agtgatgccccattccttgatcggcttcgccgagatcag aggtccctaaggggaagaggcaatactc	-	-
KM070059	Human H3N2 IAVs	Human	H3N2		2010	Singapore	A/Singapore/S2010.416b/2010	agtgatgccccattccttgatcggcttcgccgagatcag aggtccctaaggggaagaggcaatactc	-	-
KM070067	Human H3N2 IAVs	Human	H3N2		2010	Singapore	A/Singapore/S2010.611a/2010	agtgatgccccattccttgatcggcttcgccgagatcag aggtccctaaggggaagaggcaatactc	-	-
KM070071	Human H3N2 IAVs	Human	H3N2		2010	Singapore	A/Singapore/S2010.627a/2010	agtgatgccccattccttgatcggcttcgccgagatcag aggtccctaaggggaagaggcaatactc	-	-
KM070072	Human H3N2 IAVs	Human	H3N2		2010	Singapore	A/Singapore/S2010.627b/2010	agtgatgccccattccttgatcggcttcgccgagatcag aggtccctaaggggaagaggcaatactc	-	-
CY093507	Human H3N2 IAVs	Human	H3N2		2010	Mexico	A/Mexico_City/WRAIR3571N/2010	agtgatgccccattccttgatcggcttcgccgagatcag aggtccctaaggggaagaggcaatactc	-	-
CY093515	Human H3N2 IAVs	Human	H3N2		2010	Mexico	A/Mexico_City/WRAIR3577T/2010	agtgatgccccattccttgatcggcttcgccgagatcag aggtccctaaggggaagaggcaatactc	-	-

CY121217	Human H3N2 IAVs	Human	H3N2		2010	USA	A/Iowa/119/2010	agtgatgccccattccttgatcggcttcgccgagatcag aggtccctaaggggaagaggcaatactc	-	-
KC882444	Human H3N2 IAVs	Human	H3N2		2010	USA	A/Iowa/19/2010	agtgatgccccattccttgatcggcttcgccgagatcag aggtccctaaggggaagaggcaatactc	-	-
KM070069	Human H3N2 IAVs	Human	H3N2		2010	Singapore	A/Singapore/S2010.614/2010	agtgatgccccattccttgatcggcttcgccgagatcag aggtccctaaggggaagaggcaatactc	-	-
CY160564	Human H3N2 IAVs	Human	H3N2		2010	Peru	A/Peru/PER035/2010	agtgatgccccattccttgatcggcttcgccgagatcag aggtccctaaggggaagaggcaatactc	-	-
CY163188	Human H3N2 IAVs	Human	H3N2		2010	Peru	A/Peru/PER373/2010	agtgatgccccattccttgatcggcttcgccgagatcag aggtccctaaggggaagaggcaatactc	-	-
CY093387	Human H3N2 IAVs	Human	H3N2		2010	USA	A/District_of_Columbia/WRAIR1753P/2010	agtgatgccccattccttgatcggcttcgccgagatcag aggtccctaaggggaagaggcaatactc	-	-
KM070078	Human H3N2 IAVs	Human	H3N2		2010	Singapore	A/Singapore/C2010.784b/2010	agtgatgccccattccttgatcggcttcgccgagatcag aggtccctaaggggaagaggcaatactc	-	-
JX437777	Human H3N2 IAVs	Human	H3N2		2010	Singapore	A/Singapore/C2010.803V/2010	agtgatgccccattccttgatcggcttcgccgagatcag aggtccctaaggggaagaggcaatactc	-	-
KF014700	Human H3N2 IAVs	Human	H3N2		2010	Singapore	A/Singapore/H2010.559C/2010	agtgatgccccattccttgatcggcttcgccgagatcag aggtccctaaggggaagaggcaatactc	-	-
JX437894	Human H3N2 IAVs	Human	H3N2		2010	Singapore	A/Singapore/H2010.559C/2010	agtgatgccccattccttgatcggcttcgccgagatcag aggtccctaaggggaagaggcaatactc	-	-
KM070079	Human H3N2 IAVs	Human	H3N2		2010	Singapore	A/Singapore/S2010.836/2010	agtgatgccccattccttgatcggcttcgccgagatcag aggtccctaaggggaagaggcaatactc	-	-
CY092309	Human H3N2 IAVs	Human	H3N2		2010	Nicaragua	A/Managua/2492.04/2010	agtgatgccccattccttgatcggcttcgccgagatcag aggtccctaaggggaagaggcaatactc	-	-
CY160356	Human H3N2 IAVs	Human	H3N2		2010	Peru	A/Peru/PER009/2010	agtgatgccccattccttgatcggcttcgccgagatcag aggtccctaaggggaagaggcaatactc	-	-
CY160372	Human H3N2 IAVs	Human	H3N2		2010	Peru	A/Peru/PER011/2010	agtgatgccccattccttgatcggcttcgccgagatcag aggtccctaaggggaagaggcaatactc	-	-
CY160540	Human H3N2 IAVs	Human	H3N2		2010	Peru	A/Peru/PER032/2010	agtgatgccccattccttgatcggcttcgccgagatcag aggtccctaaggggaagaggcaatactc	-	-
CY160588	Human H3N2 IAVs	Human	H3N2		2010	Peru	A/Peru/PER038/2010	agtgatgccccattccttgatcggcttcgccgagatcag aggtccctaaggggaagaggcaatactc	-	-
CY161028	Human H3N2 IAVs	Human	H3N2		2010	Peru	A/Peru/PER094/2010	agtgatgccccattccttgatcggcttcgccgagatcag aggtccctaaggggaagaggcaatactc	-	-
CY161100	Human H3N2 IAVs	Human	H3N2		2010	Peru	A/Peru/PER103/2010	agtgatgccccattccttgatcggcttcgccgagatcag aggtccctaaggggaagaggcaatactc	-	-
CY161148	Human H3N2 IAVs	Human	H3N2		2010	Peru	A/Peru/PER109/2010	agtgatgccccattccttgatcggcttcgccgagatcag aggtccctaaggggaagaggcaatactc	-	-
CY161228	Human H3N2 IAVs	Human	H3N2		2010	Peru	A/Peru/PER119/2010	agtgatgccccattccttgatcggcttcgccgagatcag aggtccctaaggggaagaggcaatactc	-	-
CY161276	Human H3N2 IAVs	Human	H3N2		2010	Peru	A/Peru/PER125/2010	agtgatgccccattccttgatcggcttcgccgagatcag aggtccctaaggggaagaggcaatactc	-	-
CY161316	Human H3N2 IAVs	Human	H3N2		2010	Peru	A/Peru/PER131/2010	agtgatgccccattccttgatcggcttcgccgagatcag aggtccctaaggggaagaggcaatactc	-	-
CY161372	Human H3N2 IAVs	Human	H3N2		2010	Peru	A/Peru/PER138/2010	agtgatgccccattccttgatcggcttcgccgagatcag aggtccctaaggggaagaggcaatactc	-	-
CY161580	Human H3N2 IAVs	Human	H3N2		2010	Peru	A/Peru/PER164/2010	agtgatgccccattccttgatcggcttcgccgagatcag aggtccctaaggggaagaggcaatactc	-	-
CY161708	Human H3N2 IAVs	Human	H3N2		2010	Peru	A/Peru/PER180/2010	agtgatgccccattccttgatcggcttcgccgagatcag aggtccctaaggggaagaggcaatactc	-	-

CY161884	Human H3N2 IAVs	Human	H3N2		2010	Peru	A/Peru/PER202/2010	agtgatgccccattccttgatcggcttcgccgagatcag<br>aggtccctaaggggaagaggcaatactc	-	-
CY161964	Human H3N2 IAVs	Human	H3N2		2010	Peru	A/Peru/PER212/2010	agtgatgccccattccttgatcggcttcgccgagatcag<br>aggtccctaaggggaagaggcaatactc	-	-
CY161980	Human H3N2 IAVs	Human	H3N2		2010	Peru	A/Peru/PER214/2010	agtgatgccccattccttgatcggcttcgccgagatcag<br>aggtccctaaggggaagaggcaatactc	-	-
CY162068	Human H3N2 IAVs	Human	H3N2		2010	Peru	A/Peru/PER225/2010	agtgatgccccattccttgatcggcttcgccgagatcag<br>aggtccctaaggggaagaggcaatactc	-	-
CY162108	Human H3N2 IAVs	Human	H3N2		2010	Peru	A/Peru/PER230/2010	agtgatgccccattccttgatcggcttcgccgagatcag<br>aggtccctaaggggaagaggcaatactc	-	-
CY162188	Human H3N2 IAVs	Human	H3N2		2010	Peru	A/Peru/PER241/2010	agtgatgccccattccttgatcggcttcgccgagatcag<br>aggtccctaaggggaagaggcaatactc	-	-
CY162252	Human H3N2 IAVs	Human	H3N2		2010	Peru	A/Peru/PER250/2010	agtgatgccccattccttgatcggcttcgccgagatcag<br>aggtccctaaggggaagaggcaatactc	-	-
CY162468	Human H3N2 IAVs	Human	H3N2		2010	Peru	A/Peru/PER278/2010	agtgatgccccattccttgatcggcttcgccgagatcag<br>aggtccctaaggggaagaggcaatactc	-	-
CY162884	Human H3N2 IAVs	Human	H3N2		2010	Peru	A/Peru/PER332/2010	agtgatgccccattccttgatcggcttcgccgagatcag<br>aggtccctaaggggaagaggcaatactc	-	-
CY162948	Human H3N2 IAVs	Human	H3N2		2010	Peru	A/Peru/PER340/2010	agtgatgccccattccttgatcggcttcgccgagatcag<br>aggtccctaaggggaagaggcaatactc	-	-
CY163100	Human H3N2 IAVs	Human	H3N2		2010	Peru	A/Peru/PER360/2010	agtgatgccccattccttgatcggcttcgccgagatcag<br>aggtccctaaggggaagaggcaatactc	-	-
CY163148	Human H3N2 IAVs	Human	H3N2		2010	Peru	A/Peru/PER367/2010	agtgatgccccattccttgatcggcttcgccgagatcag<br>aggtccctaaggggaagaggcaatactc	-	-
CY163260	Human H3N2 IAVs	Human	H3N2		2010	Peru	A/Peru/PER382/2010	agtgatgccccattccttgatcggcttcgccgagatcag<br>aggtccctaaggggaagaggcaatactc	-	-
CY163300	Human H3N2 IAVs	Human	H3N2		2010	Peru	A/Peru/PER387/2010	agtgatgccccattccttgatcggcttcgccgagatcag<br>aggtccctaaggggaagaggcaatactc	-	-
CY161380	Human H3N2 IAVs	Human	H3N2		2010	Peru	A/Peru/PER139/2010	agtgatgccccattccttgatcggcttcgccgagatcag<br>aggtccctaaggggaagaggcaatactc	-	-
CY162980	Human H3N2 IAVs	Human	H3N2		2010	Peru	A/Peru/PER344/2010	agtgatgccccattccttgatcggcttcgccgagatcag<br>aggtccctaaggggaagaggcaatactc	-	-
CY160900	Human H3N2 IAVs	Human	H3N2		2010	Peru	A/Peru/PER077/2010	agtgatgccccattccttgatcggcttcgccgagatcag<br>aggtccctaaggggaagaggcaatactc	-	-
CY162764	Human H3N2 IAVs	Human	H3N2		2010	Peru	A/Peru/PER317/2010	agtgatgccccattccttgatcggcttcgccgagatcag<br>aggtccctaaggggaagaggcaatactc	-	-
CY161284	Human H3N2 IAVs	Human	H3N2		2010	Peru	A/Peru/PER127/2010	agtgatgccccattccttgatcggcttcgccgagatcag<br>aggtccctaaggggaagaggcaatactc	-	-
CY163388	Human H3N2 IAVs	Human	H3N2		2010	Peru	A/Peru/PER398/2010	agtgatgccccattccttgatcggcttcgccgagatcag<br>aggtccctaaggggaagaggcaatactc	-	-
CY161156	Human H3N2 IAVs	Human	H3N2		2010	Peru	A/Peru/PER110/2010	agtgatgccccattccttgatcggcttcgccgagatcag<br>aggtccctaaggggaagaggcaatactc	-	-
CY161628	Human H3N2 IAVs	Human	H3N2		2010	Peru	A/Peru/PER170/2010	agtgatgccccattccttgatcggcttcgccgagatcag<br>aggtccctaaggggaagaggcaatactc	-	-
JX437778	Human H3N2 IAVs	Human	H3N2		2010	Singapore	A/Singapore/C2010.937V/2010	agtgatgccccattccttgatcggcttcgccgagatcag<br>aggtccctaaggggaagaggcaatactc	-	-
KF014735	Human H3N2 IAVs	Human	H3N2		2010	Singapore	A/Singapore/C2010.937V/2010	agtgatgccccattccttgatcggcttcgccgagatcag<br>aggtccctaaggggaagaggcaatactc	-	-
CY161188	Human H3N2 IAVs	Human	H3N2		2010	Peru	A/Peru/PER114/2010	agtgatgccccattccttgatcggcttcgccgagatcag<br>aggtccctaaggggaagaggcaatactc	-	-

CY161388	Human H3N2 IAVs	Human	H3N2		2010	Peru	A/Peru/PER140/2010	agtgatgccccattccttgatcggcttcgccgagatcag aggtccctaaggggaagaggcaatactc	-	-
CY161412	Human H3N2 IAVs	Human	H3N2		2010	Peru	A/Peru/PER143/2010	agtgatgccccattccttgatcggcttcgccgagatcag aggtccctaaggggaagaggcaatactc	-	-
CY162284	Human H3N2 IAVs	Human	H3N2		2010	Peru	A/Peru/PER254/2010	agtgatgccccattccttgatcggcttcgccgagatcag aggtccctaaggggaagaggcaatactc	-	-
CY162380	Human H3N2 IAVs	Human	H3N2		2010	Peru	A/Peru/PER267/2010	agtgatgccccattccttgatcggcttcgccgagatcag aggtccctaaggggaagaggcaatactc	-	-
CY161660	Human H3N2 IAVs	Human	H3N2		2010	Peru	A/Peru/PER174/2010	agtgatgccccattccttgatcggcttcgccgagatcag aggtccctaaggggaagaggcaatactc	-	-
CY093563	Human H3N2 IAVs	Human	H3N2		2010	Mexico	A/Mexico_City/WRAIR4139N/2010	agtgatgccccattccttgatcggcttcgccgagatcag aggtccctaaggggaagaggcaatactc	-	-
KF014705	Human H3N2 IAVs	Human	H3N2		2010	Singapore	A/Singapore/H2010.822/2010	agtgatgccccattccttgatcggcttcgccgagatcag aggtccctaaggggaagaggcaatactc	-	-
KF014772	Human H3N2 IAVs	Human	H3N2		2010	Singapore	A/Singapore/H2010.822/2010	agtgatgccccattccttgatcggcttcgccgagatcag aggtccctaaggggaagaggcaatactc	-	-
KC882472	Human H3N2 IAVs	Human	H3N2		2010	USA	A/Texas/24/2010	agtgatgccccattccttgatcggcttcgccgagatcag aggtccctaaggggaagaggcaatactc	-	-
KM070075	Human H3N2 IAVs	Human	H3N2		2010	Singapore	A/Singapore/S2010.682/2010	agtgatgccccattccttgatcggcttcgccgagatcag aggtccctaaggggaagaggcaatactc	-	-
CY080295	Human H3N2 IAVs	Human	H3N2		2010	Mongolia	A/Ulaanbaatar/3815/2010	agtgatgccccattccttgatcggcttcgccgagatcag aggtccctaaggggaagaggcaatactc	-	-
CY093395	Human H3N2 IAVs	Human	H3N2		2010	Serbia	A/Belgrade/WRAIR2379N/2010	agtgatgccccattccttgatcggcttcgccgagatcag aggtccctaaggggaagaggcaatactc	-	-
CY093403	Human H3N2 IAVs	Human	H3N2		2010	Serbia	A/Belgrade/WRAIR2956T/2010	agtgatgccccattccttgatcggcttcgccgagatcag aggtccctaaggggaagaggcaatactc	-	-
HQ664921	Human H3N2 IAVs	Human	H3N2		2010	China	A/Nanjing/1655/2010	agtgatgccccattccttgatcggcttcgccgagatcag aggtccctaaggggaagaggcaatactc	-	-
HQ703376	Human H3N2 IAVs	Human	H3N2		2010	Japan	A/Niigata/1144/2010	agtgatgccccattccttgatcggcttcgccgagatcag aggtccctaaggggaagaggcaatactc	-	-
HQ703377	Human H3N2 IAVs	Human	H3N2		2010	Japan	A/Niigata/1146/2010	agtgatgccccattccttgatcggcttcgccgagatcag aggtccctaaggggaagaggcaatactc	-	-
HQ703378	Human H3N2 IAVs	Human	H3N2		2010	Japan	A/Niigata/1147/2010	agtgatgccccattccttgatcggcttcgccgagatcag aggtccctaaggggaagaggcaatactc	-	-
HQ703379	Human H3N2 IAVs	Human	H3N2		2010	Japan	A/Niigata/1148/2010	agtgatgccccattccttgatcggcttcgccgagatcag aggtccctaaggggaagaggcaatactc	-	-
HQ703380	Human H3N2 IAVs	Human	H3N2		2010	Japan	A/Niigata/1149/2010	agtgatgccccattccttgatcggcttcgccgagatcag aggtccctaaggggaagaggcaatactc	-	-
HQ703381	Human H3N2 IAVs	Human	H3N2		2010	Japan	A/Niigata/1150/2010	agtgatgccccattccttgatcggcttcgccgagatcag aggtccctaaggggaagaggcaatactc	-	-
HQ664935	Human H3N2 IAVs	Human	H3N2		2010	China	A/Nanjing/1663/2010	agtgatgccccattccttgatcggcttcgccgagatcag aggtccctaaggggaagaggcaatactc	-	-
HQ703375	Human H3N2 IAVs	Human	H3N2		2010	Japan	A/Niigata/1143/2010	agtgatgccccattccttgatcggcttcgccgagatcag aggtccctaaggggaagaggcaatactc	-	-
HQ664928	Human H3N2 IAVs	Human	H3N2		2010	China	A/Nanjing/1654/2010	agtgatgccccattccttgatcggcttcgccgagatcag aggtccctaaggggaagaggcaatactc	-	-
CY160844	Human H3N2 IAVs	Human	H3N2		2010	Peru	A/Peru/PER070/2010	agtgatgccccattccttgatcggcttcgccgagatcag aggtccctaatgggaagaggcaatactc	-	-
CY163332	Human H3N2 IAVs	Human	H3N2		2010	Peru	A/Peru/PER391/2010	agtgatgccccattccttgatcggcttcgccgagatcag aggtccctaatgggaagaggcaatactc	-	-

CY161084	Human H3N2 IAVs	Human	H3N2		2010	Peru	A/Peru/PER101/2010	agtgatgccccattccttgatcggcttcgccgagatcag aggtccttaaggggaagaggcaatactc	+	+
CY093236	Human H3N2 IAVs	Human	H3N2		2010	USA	A/District_of_Columbia/WRAIR0301/2010	agtgatgccccattccttgatcggcttcgccgagatcag aggtctctaaggggaagaggcaatactc	+	+
KM070070	Human H3N2 IAVs	Human	H3N2		2010	Singapore	A/Singapore/C2010.625/2010	agtgatgccccattccttgatcggcttcgccgagatcag aggtctctaaggggaagaggcaatactc	+	+
JX437776	Human H3N2 IAVs	Human	H3N2		2010	Singapore	A/Singapore/C2010.682V/2010	agtgatgccccattccttgatcggcttcgccgagatcag aggtctctaaggggaagaggcaatactc	+	+
KM070076	Human H3N2 IAVs	Human	H3N2		2010	Singapore	A/Singapore/C2010.721/2010	agtgatgccccattccttgatcggcttcgccgagatcag aggtctctaaggggaagaggcaatactc	+	+
CY161260	Human H3N2 IAVs	Human	H3N2		2010	Peru	A/Peru/PER123/2010	agtgatgccccattccttgatcggcttcgccgagatcag aggtgcctaaagggaagaggcaatactc	+	+
CY162436	Human H3N2 IAVs	Human	H3N2		2010	Peru	A/Peru/PER274/2010	agtgatgccccattccttgatcggcttcgccgagatcag aggtgcctaagaggaagaggcaatactc	+	+
CY160364	Human H3N2 IAVs	Human	H3N2		2010	Peru	A/Peru/PER010/2010	agtgatgccccattccttgatcggcttcgccgagatcag aggtgcctaaggggaagaggcaatactc	+	+
CY093523	Human H3N2 IAVs	Human	H3N2		2010	Mexico	A/Mexico_City/WRAIR3578N/2010	agtgatgccccattccttgatcggcttcgccgagatcag aggtgcctaaggggaagaggcaatactc	+	+
CY093531	Human H3N2 IAVs	Human	H3N2		2010	Mexico	A/Mexico_City/WRAIR3578T/2010	agtgatgccccattccttgatcggcttcgccgagatcag aggtgcctaaggggaagaggcaatactc	+	+
CY093555	Human H3N2 IAVs	Human	H3N2		2010	Mexico	A/Mexico_City/WRAIR3580T/2010	agtgatgccccattccttgatcggcttcgccgagatcag aggtgcctaaggggaagaggcaatactc	+	+
CY160300	Human H3N2 IAVs	Human	H3N2		2010	Peru	A/Peru/PER002/2010	agtgatgccccattccttgatcggcttcgccgagatcag aggtgcctaaggggaagaggcaatactc	+	+
CY162300	Human H3N2 IAVs	Human	H3N2		2010	Peru	A/Peru/PER256/2010	agtgatgccccattccttgatcggcttcgccgagatcag aggtgcctaaggggaagaggcaatactc	+	+
CY160404	Human H3N2 IAVs	Human	H3N2		2010	Peru	A/Peru/PER015/2010	agtgatgccccattccttgatcggcttcgccgagatcag aggtgcctaaggggaagaggcaatactc	+	+
CY160780	Human H3N2 IAVs	Human	H3N2		2010	Peru	A/Peru/PER062/2010	agtgatgccccattccttgatcggcttcgccgagatcag aggtgcctaaggggaagaggcaatactc	+	+
CY160796	Human H3N2 IAVs	Human	H3N2		2010	Peru	A/Peru/PER064/2010	agtgatgccccattccttgatcggcttcgccgagatcag aggtgcctaaggggaagaggcaatactc	+	+
CY160852	Human H3N2 IAVs	Human	H3N2		2010	Peru	A/Peru/PER071/2010	agtgatgccccattccttgatcggcttcgccgagatcag aggtgcctaaggggaagaggcaatactc	+	+
CY160884	Human H3N2 IAVs	Human	H3N2		2010	Peru	A/Peru/PER075/2010	agtgatgccccattccttgatcggcttcgccgagatcag aggtgcctaaggggaagaggcaatactc	+	+
CY160916	Human H3N2 IAVs	Human	H3N2		2010	Peru	A/Peru/PER079/2010	agtgatgccccattccttgatcggcttcgccgagatcag aggtgcctaaggggaagaggcaatactc	+	+
CY161300	Human H3N2 IAVs	Human	H3N2		2010	Peru	A/Peru/PER129/2010	agtgatgccccattccttgatcggcttcgccgagatcag aggtgcctaaggggaagaggcaatactc	+	+
CY161348	Human H3N2 IAVs	Human	H3N2		2010	Peru	A/Peru/PER135/2010	agtgatgccccattccttgatcggcttcgccgagatcag aggtgcctaaggggaagaggcaatactc	+	+
CY161396	Human H3N2 IAVs	Human	H3N2		2010	Peru	A/Peru/PER141/2010	agtgatgccccattccttgatcggcttcgccgagatcag aggtgcctaaggggaagaggcaatactc	+	+
CY161428	Human H3N2 IAVs	Human	H3N2		2010	Peru	A/Peru/PER145/2010	agtgatgccccattccttgatcggcttcgccgagatcag aggtgcctaaggggaagaggcaatactc	+	+
CY161436	Human H3N2 IAVs	Human	H3N2		2010	Peru	A/Peru/PER146/2010	agtgatgccccattccttgatcggcttcgccgagatcag aggtgcctaaggggaagaggcaatactc	+	+
CY161548	Human H3N2 IAVs	Human	H3N2		2010	Peru	A/Peru/PER160/2010	agtgatgccccattccttgatcggcttcgccgagatcag aggtgcctaaggggaagaggcaatactc	+	+

CY161588	Human H3N2 IAVs	Human	H3N2		2010	Peru	A/Peru/PER165/2010	agtgatgccccattccttgatcggcttcgccgagatcag aggtgcctaaggggaagaggcaatactc	+	+
CY161612	Human H3N2 IAVs	Human	H3N2		2010	Peru	A/Peru/PER168/2010	agtgatgccccattccttgatcggcttcgccgagatcag aggtgcctaaggggaagaggcaatactc	+	+
CY161620	Human H3N2 IAVs	Human	H3N2		2010	Peru	A/Peru/PER169/2010	agtgatgccccattccttgatcggcttcgccgagatcag aggtgcctaaggggaagaggcaatactc	+	+
CY161636	Human H3N2 IAVs	Human	H3N2		2010	Peru	A/Peru/PER171/2010	agtgatgccccattccttgatcggcttcgccgagatcag aggtgcctaaggggaagaggcaatactc	+	+
CY161732	Human H3N2 IAVs	Human	H3N2		2010	Peru	A/Peru/PER183/2010	agtgatgccccattccttgatcggcttcgccgagatcag aggtgcctaaggggaagaggcaatactc	+	+
CY161772	Human H3N2 IAVs	Human	H3N2		2010	Peru	A/Peru/PER188/2010	agtgatgccccattccttgatcggcttcgccgagatcag aggtgcctaaggggaagaggcaatactc	+	+
CY161852	Human H3N2 IAVs	Human	H3N2		2010	Peru	A/Peru/PER198/2010	agtgatgccccattccttgatcggcttcgccgagatcag aggtgcctaaggggaagaggcaatactc	+	+
CY161924	Human H3N2 IAVs	Human	H3N2		2010	Peru	A/Peru/PER207/2010	agtgatgccccattccttgatcggcttcgccgagatcag aggtgcctaaggggaagaggcaatactc	+	+
CY161932	Human H3N2 IAVs	Human	H3N2		2010	Peru	A/Peru/PER208/2010	agtgatgccccattccttgatcggcttcgccgagatcag aggtgcctaaggggaagaggcaatactc	+	+
CY162060	Human H3N2 IAVs	Human	H3N2		2010	Peru	A/Peru/PER224/2010	agtgatgccccattccttgatcggcttcgccgagatcag aggtgcctaaggggaagaggcaatactc	+	+
CY162100	Human H3N2 IAVs	Human	H3N2		2010	Peru	A/Peru/PER229/2010	agtgatgccccattccttgatcggcttcgccgagatcag aggtgcctaaggggaagaggcaatactc	+	+
CY162140	Human H3N2 IAVs	Human	H3N2		2010	Peru	A/Peru/PER234/2010	agtgatgccccattccttgatcggcttcgccgagatcag aggtgcctaaggggaagaggcaatactc	+	+
CY162196	Human H3N2 IAVs	Human	H3N2		2010	Peru	A/Peru/PER242/2010	agtgatgccccattccttgatcggcttcgccgagatcag aggtgcctaaggggaagaggcaatactc	+	+
CY162268	Human H3N2 IAVs	Human	H3N2		2010	Peru	A/Peru/PER252/2010	agtgatgccccattccttgatcggcttcgccgagatcag aggtgcctaaggggaagaggcaatactc	+	+
CY162308	Human H3N2 IAVs	Human	H3N2		2010	Peru	A/Peru/PER257/2010	agtgatgccccattccttgatcggcttcgccgagatcag aggtgcctaaggggaagaggcaatactc	+	+
CY162324	Human H3N2 IAVs	Human	H3N2		2010	Peru	A/Peru/PER259/2010	agtgatgccccattccttgatcggcttcgccgagatcag aggtgcctaaggggaagaggcaatactc	+	+
CY162348	Human H3N2 IAVs	Human	H3N2		2010	Peru	A/Peru/PER263/2010	agtgatgccccattccttgatcggcttcgccgagatcag aggtgcctaaggggaagaggcaatactc	+	+
CY162420	Human H3N2 IAVs	Human	H3N2		2010	Peru	A/Peru/PER272/2010	agtgatgccccattccttgatcggcttcgccgagatcag aggtgcctaaggggaagaggcaatactc	+	+
CY162476	Human H3N2 IAVs	Human	H3N2		2010	Peru	A/Peru/PER279/2010	agtgatgccccattccttgatcggcttcgccgagatcag aggtgcctaaggggaagaggcaatactc	+	+
CY162524	Human H3N2 IAVs	Human	H3N2		2010	Peru	A/Peru/PER285/2010	agtgatgccccattccttgatcggcttcgccgagatcag aggtgcctaaggggaagaggcaatactc	+	+
CY162532	Human H3N2 IAVs	Human	H3N2		2010	Peru	A/Peru/PER286/2010	agtgatgccccattccttgatcggcttcgccgagatcag aggtgcctaaggggaagaggcaatactc	+	+
CY162548	Human H3N2 IAVs	Human	H3N2		2010	Peru	A/Peru/PER288/2010	agtgatgccccattccttgatcggcttcgccgagatcag aggtgcctaaggggaagaggcaatactc	+	+
CY162572	Human H3N2 IAVs	Human	H3N2		2010	Peru	A/Peru/PER291/2010	agtgatgccccattccttgatcggcttcgccgagatcag aggtgcctaaggggaagaggcaatactc	+	+
CY162580	Human H3N2 IAVs	Human	H3N2		2010	Peru	A/Peru/PER292/2010	agtgatgccccattccttgatcggcttcgccgagatcag aggtgcctaaggggaagaggcaatactc	+	+
CY162588	Human H3N2 IAVs	Human	H3N2		2010	Peru	A/Peru/PER294/2010	agtgatgccccattccttgatcggcttcgccgagatcag aggtgcctaaggggaagaggcaatactc	+	+

CY162596	Human H3N2 IAVs	Human	H3N2		2010	Peru	A/Peru/PER295/2010	agtgatgccccattccttgatcggcttcgccgagatcag aggtgcctaaggggaagaggcaatactc	+	+
CY162628	Human H3N2 IAVs	Human	H3N2		2010	Peru	A/Peru/PER300/2010	agtgatgccccattccttgatcggcttcgccgagatcag aggtgcctaaggggaagaggcaatactc	+	+
CY162652	Human H3N2 IAVs	Human	H3N2		2010	Peru	A/Peru/PER303/2010	agtgatgccccattccttgatcggcttcgccgagatcag aggtgcctaaggggaagaggcaatactc	+	+
CY162676	Human H3N2 IAVs	Human	H3N2		2010	Peru	A/Peru/PER306/2010	agtgatgccccattccttgatcggcttcgccgagatcag aggtgcctaaggggaagaggcaatactc	+	+
CY162708	Human H3N2 IAVs	Human	H3N2		2010	Peru	A/Peru/PER310/2010	agtgatgccccattccttgatcggcttcgccgagatcag aggtgcctaaggggaagaggcaatactc	+	+
CY162716	Human H3N2 IAVs	Human	H3N2		2010	Peru	A/Peru/PER311/2010	agtgatgccccattccttgatcggcttcgccgagatcag aggtgcctaaggggaagaggcaatactc	+	+
CY162780	Human H3N2 IAVs	Human	H3N2		2010	Peru	A/Peru/PER319/2010	agtgatgccccattccttgatcggcttcgccgagatcag aggtgcctaaggggaagaggcaatactc	+	+
CY162796	Human H3N2 IAVs	Human	H3N2		2010	Peru	A/Peru/PER321/2010	agtgatgccccattccttgatcggcttcgccgagatcag aggtgcctaaggggaagaggcaatactc	+	+
CY162860	Human H3N2 IAVs	Human	H3N2		2010	Peru	A/Peru/PER329/2010	agtgatgccccattccttgatcggcttcgccgagatcag aggtgcctaaggggaagaggcaatactc	+	+
CY162940	Human H3N2 IAVs	Human	H3N2		2010	Peru	A/Peru/PER339/2010	agtgatgccccattccttgatcggcttcgccgagatcag aggtgcctaaggggaagaggcaatactc	+	+
CY163012	Human H3N2 IAVs	Human	H3N2		2010	Peru	A/Peru/PER349/2010	agtgatgccccattccttgatcggcttcgccgagatcag aggtgcctaaggggaagaggcaatactc	+	+
CY163076	Human H3N2 IAVs	Human	H3N2		2010	Peru	A/Peru/PER357/2010	agtgatgccccattccttgatcggcttcgccgagatcag aggtgcctaaggggaagaggcaatactc	+	+
CY163140	Human H3N2 IAVs	Human	H3N2		2010	Peru	A/Peru/PER365/2010	agtgatgccccattccttgatcggcttcgccgagatcag aggtgcctaaggggaagaggcaatactc	+	+
CY163212	Human H3N2 IAVs	Human	H3N2		2010	Peru	A/Peru/PER376/2010	agtgatgccccattccttgatcggcttcgccgagatcag aggtgcctaaggggaagaggcaatactc	+	+
CY163220	Human H3N2 IAVs	Human	H3N2		2010	Peru	A/Peru/PER377/2010	agtgatgccccattccttgatcggcttcgccgagatcag aggtgcctaaggggaagaggcaatactc	+	+
CY163252	Human H3N2 IAVs	Human	H3N2		2010	Peru	A/Peru/PER381/2010	agtgatgccccattccttgatcggcttcgccgagatcag aggtgcctaaggggaagaggcaatactc	+	+
CY163292	Human H3N2 IAVs	Human	H3N2		2010	Peru	A/Peru/PER386/2010	agtgatgccccattccttgatcggcttcgccgagatcag aggtgcctaaggggaagaggcaatactc	+	+
CY163324	Human H3N2 IAVs	Human	H3N2		2010	Peru	A/Peru/PER390/2010	agtgatgccccattccttgatcggcttcgccgagatcag aggtgcctaaggggaagaggcaatactc	+	+
CY161292	Human H3N2 IAVs	Human	H3N2		2010	Peru	A/Peru/PER128/2010	agtgatgccccattccttgatcggcttcgccgagatcag aggtgcctaaggggaagaggcaatactc	+	+
CY163028	Human H3N2 IAVs	Human	H3N2		2010	Peru	A/Peru/PER351/2010	agtgatgccccattccttgatcggcttcgccgagatcag aggtgcctaaggggaagaggcaatactc	+	+
CY161452	Human H3N2 IAVs	Human	H3N2		2010	Peru	A/Peru/PER148/2010	agtgatgccccattccttgatcggcttcgccgagatcag aggtgcctaaggggaagaggcaatactc	+	+
CY161804	Human H3N2 IAVs	Human	H3N2		2010	Peru	A/Peru/PER192/2010	agtgatgccccattccttgatcggcttcgccgagatcag aggtgcctaaggggaagaggcaatactc	+	+
CY161876	Human H3N2 IAVs	Human	H3N2		2010	Peru	A/Peru/PER201/2010	agtgatgccccattccttgatcggcttcgccgagatcag aggtgcctaaggggaagaggcaatactc	+	+
CY162164	Human H3N2 IAVs	Human	H3N2		2010	Peru	A/Peru/PER237/2010	agtgatgccccattccttgatcggcttcgccgagatcag aggtgcctaaggggaagaggcaatactc	+	+
CY162724	Human H3N2 IAVs	Human	H3N2		2010	Peru	A/Peru/PER312/2010	agtgatgccccattccttgatcggcttcgccgagatcag aggtgcctaaggggaagaggcaatactc	+	+

CY162908	Human H3N2 IAVs	Human	H3N2		2010	Peru	A/Peru/PER335/2010	agtgatgccccattccttgatcggcttcgccgagatcag aggtgcctaaggggaagaggcaatactc	+	+
CY163108	Human H3N2 IAVs	Human	H3N2		2010	Peru	A/Peru/PER361/2010	agtgatgccccattccttgatcggcttcgccgagatcag aggtgcctaaggggaagaggcaatactc	+	+
CY163156	Human H3N2 IAVs	Human	H3N2		2010	Peru	A/Peru/PER369/2010	agtgatgccccattccttgatcggcttcgccgagatcag aggtgcctaaggggaagaggcaatactc	+	+
CY163340	Human H3N2 IAVs	Human	H3N2		2010	Peru	A/Peru/PER392/2010	agtgatgccccattccttgatcggcttcgccgagatcag aggtgcctaaggggaagaggcaatactc	+	+
CY161900	Human H3N2 IAVs	Human	H3N2		2010	Peru	A/Peru/PER204/2010	agtgatgccccattccttgatcggcttcgccgagatcag aggtgcctaaggggaagaggcaatactc	+	+
KF014766	Human H3N2 IAVs	Human	H3N2		2010	Singapore	A/Singapore/H2010.211/2010	agtgatgccccccttccttgatcggcttcgccgagatcag aggtccctaaagggaagaggcaatactc	-	-
KF014693	Human H3N2 IAVs	Human	H3N2		2010	Singapore	A/Singapore/H2010.211/2010	agtgatgccccccttccttgatcggcttcgccgagatcag aggtccctaaagggaagaggcaatactc	-	-
KM070063	Human H3N2 IAVs	Human	H3N2		2010	Singapore	A/Singapore/C2010.567/2010	agtgatgccccccttccttgatcggcttcgccgagatcag aggtccctaaggggaagaggcaatactc	-	-
KM070077	Human H3N2 IAVs	Human	H3N2		2010	Singapore	A/Singapore/C2010.742/2010	artgatgccccattccttgatcggcttcgccgagatcag aggtccctaaggggaagaggcaatactc	-	-
JX494718	Human H3N2 IAVs	Swine	H3N2		2010	China	A/swine/Guangdong/L23/2010	ggtgatgccccattccttgatcggcttcgccgagatcag aagtccctaaggggaagaggcagcactc	+	+
CY167321	Human H3N2 IAVs	Human	H3N2		2011	USA	A/Tennessee/F2092c90/2011	aatgatgccccattccttgatcggcttcgccgagatca gaggtccctaaggggaagaggcaatactc	-	-
CY111250	Human H3N2 IAVs	Human	H3N2		2011	USA	A/Boston/DOA20/2011	agcgatgccccattccttgatcggcttcgccgagatca gaggtccctaaggggaagaggcaatactc	-	-
KF014742	Human H3N2 IAVs	Human	H3N2		2011	Singapore	A/Singapore/C2011.411/2011	agtgatgccccattccttgataggcttcgccgagatca gaggtccctaaggggaagaggcaatactc	+	+
KF014667	Human H3N2 IAVs	Human	H3N2		2011	Singapore	A/Singapore/C2011.411/2011	agtgatgccccattccttgataggcttcgccgagatca gaggtccctaaggggaagaggcaatactc	+	+
CY162900	Human H3N2 IAVs	Human	H3N2		2011	Peru	A/Peru/PER334/2011	agtgatgccccattccttgatcggattcgccgagatca gaggtccctaaggggaagaggcaatactc	-	-
CY163364	Human H3N2 IAVs	Human	H3N2		2011	Peru	A/Peru/PER395/2011	agtgatgccccattccttgatcggattcgccgagatca gaggtccctaaggggaagaggcaatactc	-	-
KF014751	Human H3N2 IAVs	Human	H3N2		2011	Singapore	A/Singapore/C2011.512/2011	agtgatgccccattccttgatcggctgcgccgagatca gaggtccctaaggggaagaggcaatactc	-	-
KF014675	Human H3N2 IAVs	Human	H3N2		2011	Singapore	A/Singapore/C2011.512/2011	agtgatgccccattccttgatcggctgcgccgagatca gaggtccctaaggggaagaggcaatactc	-	-
KF014681	Human H3N2 IAVs	Human	H3N2		2011	Singapore	A/Singapore/C2011.641/2011	agtgatgccccattccttgatcggctgcgccgagatca gaggtccctaaggggaagaggcaatactc	-	-
KF014757	Human H3N2 IAVs	Human	H3N2		2011	Singapore	A/Singapore/C2011.641/2011	agtgatgccccattccttgatcggctgcgccgagatca gaggtccctaaggggaagaggcaatactc	-	-
KF014784	Human H3N2 IAVs	Human	H3N2		2011	Singapore	A/Singapore/H2011.507/2011	agtgatgccccattccttgatcggctgcgccgagatca gaggtccctaaggggaagaggcaatactc	-	-
KF014720	Human H3N2 IAVs	Human	H3N2		2011	Singapore	A/Singapore/H2011.507/2011	agtgatgccccattccttgatcggctgcgccgagatca gaggtccctaaggggaagaggcaatactc	-	-
KM070088	Human H3N2 IAVs	Human	H3N2		2011	Singapore	A/Singapore/H2011.748/2011	agtgatgccccattccttgatcggctgcgccgagatca gaggtccctaaggggaagaggcaatactc	-	-
KM070083	Human H3N2 IAVs	Human	H3N2		2011	Singapore	A/Singapore/S2011.611b/2011	agtgatgccccattccttgatcggctgcgccgagatca gaggtccctaaggggaagaggcaatactc	-	-
KF014663	Human H3N2 IAVs	Human	H3N2		2011	Singapore	A/Singapore/C2011.173/2011	agtgatgccccattccttgatcggcttcgccgagacca gaggtccctaaggggaagaggcaatactc	-	-

KF014737	Human H3N2 IAVs	Human	H3N2		2011	Singapore	A/Singapore/C2011.173/2011	agtgatgccccattccttgatcggcttcgccgagacca<br>gaggtccctaaggggaagaggcaatactc	-	-
JX437897	Human H3N2 IAVs	Human	H3N2		2011	Singapore	A/Singapore/H2011.140C/2011	agtgatgccccattccttgatcggcttcgccgagacca<br>gaggtccctaaggggaagaggcaatactc	-	-
KF014706	Human H3N2 IAVs	Human	H3N2		2011	Singapore	A/Singapore/H2011.140C/2011	agtgatgccccattccttgatcggcttcgccgagacca<br>gaggtccctaaggggaagaggcaatactc	-	-
KM070094	Human H3N2 IAVs	Human	H3N2		2011	Singapore	A/Singapore/H2011.904/2011	agtgatgccccattccttgatcggcttcgccgagacca<br>gaggtccctaatgggaagaggcaatactc	-	-
KJ855335	Human H3N2 IAVs	Human	H3N2		2011	Mexico	A/Mexico/VER40/2011	agtgatgccccattccttgatcggcttcgccgagatca<br>aaggtccctaaggggaagaggcaatactc	-	-
CY181917	Human H3N2 IAVs	Human	H3N2		2011	Nicaragua	A/Nicaragua/AGA2_49/2011	agtgatgccccattccttgatcggcttcgccgagatca<br>aaggtccctaaggggaagaggcaatactc	-	-
CY181989	Human H3N2 IAVs	Human	H3N2		2011	Nicaragua	A/Nicaragua/AGA2_59/2011	agtgatgccccattccttgatcggcttcgccgagatca<br>aaggtccctaaggggaagaggcaatactc	-	-
CY182029	Human H3N2 IAVs	Human	H3N2		2011	Nicaragua	A/Nicaragua/AGA2_65/2011	agtgatgccccattccttgatcggcttcgccgagatca<br>aaggtccctaaggggaagaggcaatactc	-	-
CY182045	Human H3N2 IAVs	Human	H3N2		2011	Nicaragua	A/Nicaragua/AGA2_67/2011	agtgatgccccattccttgatcggcttcgccgagatca<br>aaggtccctaaggggaagaggcaatactc	-	-
CY181909	Human H3N2 IAVs	Human	H3N2		2011	Nicaragua	A/Nicaragua/AGA2_48/2011	agtgatgccccattccttgatcggcttcgccgagatca<br>aaggtccctaaggggaagaggcaatactc	-	-
CY116711	Human H3N2 IAVs	Human	H3N2		2011	USA	A/Boston/DOA24/2011	agtgatgccccattccttgatcggcttcgccgagatcag<br>aggtccctaaagggaagaggcaatactc	+	+
KM070092	Human H3N2 IAVs	Human	H3N2		2011	Singapore	A/Singapore/C2011.863/2011	agtgatgccccattccttgatcggcttcgccgagatcag<br>aggtccctaaggggaagaggcaacactc	-	-
CY111386	Human H3N2 IAVs	Human	H3N2		2011	USA	A/Boston/DOA71/2011	agtgatgccccattccttgatcggcttcgccgagatcag<br>aggtccctaaggggaagaggcaacactc	-	-
CY161572	Human H3N2 IAVs	Human	H3N2		2011	Peru	A/Peru/PER163/2011	agtgatgccccattccttgatcggcttcgccgagatcag<br>aggtccctaaggggaagaggcaacactc	-	-
CY114557	Human H3N2 IAVs	Human	H3N2		2011	Russia	A/Astrakhan/RII65/2011	agtgatgccccattccttgatcggcttcgccgagatcag<br>aggtccctaaggggaagaggcaatactc	-	-
CY161476	Human H3N2 IAVs	Human	H3N2		2011	Peru	A/Peru/PER151/2011	agtgatgccccattccttgatcggcttcgccgagatcag<br>aggtccctaaggggaagaggcaatactc	-	-
CY161780	Human H3N2 IAVs	Human	H3N2		2011	Peru	A/Peru/PER189/2011	agtgatgccccattccttgatcggcttcgccgagatcag<br>aggtccctaaggggaagaggcaatactc	-	-
CY163116	Human H3N2 IAVs	Human	H3N2		2011	Peru	A/Peru/PER362/2011	agtgatgccccattccttgatcggcttcgccgagatcag<br>aggtccctaaggggaagaggcaatactc	-	-
CY163196	Human H3N2 IAVs	Human	H3N2		2011	Peru	A/Peru/PER374/2011	agtgatgccccattccttgatcggcttcgccgagatcag<br>aggtccctaaggggaagaggcaatactc	-	-
CY160396	Human H3N2 IAVs	Human	H3N2		2011	Peru	A/Peru/PER014/2011	agtgatgccccattccttgatcggcttcgccgagatcag<br>aggtccctaaggggaagaggcaatactc	-	-
CY160508	Human H3N2 IAVs	Human	H3N2		2011	Peru	A/Peru/PER028/2011	agtgatgccccattccttgatcggcttcgccgagatcag<br>aggtccctaaggggaagaggcaatactc	-	-
CY160612	Human H3N2 IAVs	Human	H3N2		2011	Peru	A/Peru/PER041/2011	agtgatgccccattccttgatcggcttcgccgagatcag<br>aggtccctaaggggaagaggcaatactc	-	-
CY160812	Human H3N2 IAVs	Human	H3N2		2011	Peru	A/Peru/PER066/2011	agtgatgccccattccttgatcggcttcgccgagatcag<br>aggtccctaaggggaagaggcaatactc	-	-
CY161244	Human H3N2 IAVs	Human	H3N2		2011	Peru	A/Peru/PER121/2011	agtgatgccccattccttgatcggcttcgccgagatcag<br>aggtccctaaggggaagaggcaatactc	-	-
CY161356	Human H3N2 IAVs	Human	H3N2		2011	Peru	A/Peru/PER136/2011	agtgatgccccattccttgatcggcttcgccgagatcag<br>aggtccctaaggggaagaggcaatactc	-	-

CY161596	Human H3N2 IAVs	Human	H3N2		2011	Peru	A/Peru/PER166/2011	agtgatgccccattccttgatcggcttcgccgagatcag<br>aggtccctaaggggaagaggcaatactc	-	-
CY161844	Human H3N2 IAVs	Human	H3N2		2011	Peru	A/Peru/PER197/2011	agtgatgccccattccttgatcggcttcgccgagatcag<br>aggtccctaaggggaagaggcaatactc	-	-
CY162684	Human H3N2 IAVs	Human	H3N2		2011	Peru	A/Peru/PER307/2011	agtgatgccccattccttgatcggcttcgccgagatcag<br>aggtccctaaggggaagaggcaatactc	-	-
CY162924	Human H3N2 IAVs	Human	H3N2		2011	Peru	A/Peru/PER337/2011	agtgatgccccattccttgatcggcttcgccgagatcag<br>aggtccctaaggggaagaggcaatactc	-	-
CY162972	Human H3N2 IAVs	Human	H3N2		2011	Peru	A/Peru/PER343/2011	agtgatgccccattccttgatcggcttcgccgagatcag<br>aggtccctaaggggaagaggcaatactc	-	-
CY162996	Human H3N2 IAVs	Human	H3N2		2011	Peru	A/Peru/PER346/2011	agtgatgccccattccttgatcggcttcgccgagatcag<br>aggtccctaaggggaagaggcaatactc	-	-
CY160420	Human H3N2 IAVs	Human	H3N2		2011	Peru	A/Peru/PER017/2011	agtgatgccccattccttgatcggcttcgccgagatcag<br>aggtccctaaggggaagaggcaatactc	-	-
KF014678	Human H3N2 IAVs	Human	H3N2		2011	Singapore	A/Singapore/C2011.564/2011	agtgatgccccattccttgatcggcttcgccgagatcag<br>aggtccctaaggggaagaggcaatactc	-	-
KF014753	Human H3N2 IAVs	Human	H3N2		2011	Singapore	A/Singapore/C2011.564/2011	agtgatgccccattccttgatcggcttcgccgagatcag<br>aggtccctaaggggaagaggcaatactc	-	-
KM070082	Human H3N2 IAVs	Human	H3N2		2011	Singapore	A/Singapore/S2011.575/2011	agtgatgccccattccttgatcggcttcgccgagatcag<br>aggtccctaaggggaagaggcaatactc	-	-
KM070084	Human H3N2 IAVs	Human	H3N2		2011	Singapore	A/Singapore/H2011.625/2011	agtgatgccccattccttgatcggcttcgccgagatcag<br>aggtccctaaggggaagaggcaatactc	-	-
CY161764	Human H3N2 IAVs	Human	H3N2		2011	Peru	A/Peru/PER187/2011	agtgatgccccattccttgatcggcttcgccgagatcag<br>aggtccctaaggggaagaggcaatactc	-	-
CY161012	Human H3N2 IAVs	Human	H3N2		2011	Peru	A/Peru/PER092/2011	agtgatgccccattccttgatcggcttcgccgagatcag<br>aggtccctaaggggaagaggcaatactc	-	-
KF014669	Human H3N2 IAVs	Human	H3N2		2011	Singapore	A/Singapore/C2011.452/2011	agtgatgccccattccttgatcggcttcgccgagatcag<br>aggtccctaaggggaagaggcaatactc	-	-
KF014744	Human H3N2 IAVs	Human	H3N2		2011	Singapore	A/Singapore/C2011.452/2011	agtgatgccccattccttgatcggcttcgccgagatcag<br>aggtccctaaggggaagaggcaatactc	-	-
KF014674	Human H3N2 IAVs	Human	H3N2		2011	Singapore	A/Singapore/C2011.496/2011	agtgatgccccattccttgatcggcttcgccgagatcag<br>aggtccctaaggggaagaggcaatactc	-	-
KF014749	Human H3N2 IAVs	Human	H3N2		2011	Singapore	A/Singapore/C2011.496/2011	agtgatgccccattccttgatcggcttcgccgagatcag<br>aggtccctaaggggaagaggcaatactc	-	-
KF014752	Human H3N2 IAVs	Human	H3N2		2011	Singapore	A/Singapore/C2011.518/2011	agtgatgccccattccttgatcggcttcgccgagatcag<br>aggtccctaaggggaagaggcaatactc	-	-
KF014676	Human H3N2 IAVs	Human	H3N2		2011	Singapore	A/Singapore/C2011.518/2011	agtgatgccccattccttgatcggcttcgccgagatcag<br>aggtccctaaggggaagaggcaatactc	-	-
KF014754	Human H3N2 IAVs	Human	H3N2		2011	Singapore	A/Singapore/C2011.573/2011	agtgatgccccattccttgatcggcttcgccgagatcag<br>aggtccctaaggggaagaggcaatactc	-	-
KF014680	Human H3N2 IAVs	Human	H3N2		2011	Singapore	A/Singapore/C2011.614/2011	agtgatgccccattccttgatcggcttcgccgagatcag<br>aggtccctaaggggaagaggcaatactc	-	-
KF014756	Human H3N2 IAVs	Human	H3N2		2011	Singapore	A/Singapore/C2011.614/2011	agtgatgccccattccttgatcggcttcgccgagatcag<br>aggtccctaaggggaagaggcaatactc	-	-
KF014682	Human H3N2 IAVs	Human	H3N2		2011	Singapore	A/Singapore/C2011.647/2011	agtgatgccccattccttgatcggcttcgccgagatcag<br>aggtccctaaggggaagaggcaatactc	-	-
KF014758	Human H3N2 IAVs	Human	H3N2		2011	Singapore	A/Singapore/C2011.647/2011	agtgatgccccattccttgatcggcttcgccgagatcag<br>aggtccctaaggggaagaggcaatactc	-	-
KF014719	Human H3N2 IAVs	Human	H3N2		2011	Singapore	A/Singapore/H2011.504C/2011	agtgatgccccattccttgatcggcttcgccgagatcag<br>aggtccctaaggggaagaggcaatactc	-	-

JX437899	Human H3N2 IAVs	Human	H3N2		2011	Singapore	A/Singapore/H2011.504C/2011	agtgatgccccattccttgatcggcttcgccgagatcag aggtccctaaggggaagaggcaatactc	-	-
KF014723	Human H3N2 IAVs	Human	H3N2		2011	Singapore	A/Singapore/H2011.570/2011	agtgatgccccattccttgatcggcttcgccgagatcag aggtccctaaggggaagaggcaatactc	-	-
KF014787	Human H3N2 IAVs	Human	H3N2		2011	Singapore	A/Singapore/H2011.570/2011	agtgatgccccattccttgatcggcttcgccgagatcag aggtccctaaggggaagaggcaatactc	-	-
KF014679	Human H3N2 IAVs	Human	H3N2		2011	Singapore	A/Singapore/C2011.573/2011	agtgatgccccattccttgatcggcttcgccgagatcag aggtccctaaggggaagaggcaatactc	-	-
KF014786	Human H3N2 IAVs	Human	H3N2		2011	Singapore	A/Singapore/H2011.518/2011	agtgatgccccattccttgatcggcttcgccgagatcag aggtccctaaggggaagaggcaatactc	-	-
KF014722	Human H3N2 IAVs	Human	H3N2		2011	Singapore	A/Singapore/H2011.518/2011	agtgatgccccattccttgatcggcttcgccgagatcag aggtccctaaggggaagaggcaatactc	-	-
KM070081	Human H3N2 IAVs	Human	H3N2		2011	Singapore	A/Singapore/H2011.521/2011	agtgatgccccattccttgatcggcttcgccgagatcag aggtccctaaggggaagaggcaatactc	-	-
KM070097	Human H3N2 IAVs	Human	H3N2		2011	Singapore	A/Singapore/C2011.992/2011	agtgatgccccattccttgatcggcttcgccgagatcag aggtccctaaggggaagaggcaatactc	-	-
KM070096	Human H3N2 IAVs	Human	H3N2		2011	Singapore	A/Singapore/H2011.923/2011	agtgatgccccattccttgatcggcttcgccgagatcag aggtccctaaggggaagaggcaatactc	-	-
CY111146	Human H3N2 IAVs	Human	H3N2		2011	USA	A/Boston/DOA03/2011	agtgatgccccattccttgatcggcttcgccgagatcag aggtccctaaggggaagaggcaatactc	-	-
CY111178	Human H3N2 IAVs	Human	H3N2		2011	USA	A/Boston/DOA07/2011	agtgatgccccattccttgatcggcttcgccgagatcag aggtccctaaggggaagaggcaatactc	-	-
CY111242	Human H3N2 IAVs	Human	H3N2		2011	USA	A/Boston/DOA12/2011	agtgatgccccattccttgatcggcttcgccgagatcag aggtccctaaggggaagaggcaatactc	-	-
CY134533	Human H3N2 IAVs	Human	H3N2		2011	USA	A/Boston/DOA41/2011	agtgatgccccattccttgatcggcttcgccgagatcag aggtccctaaggggaagaggcaatactc	-	-
CY111306	Human H3N2 IAVs	Human	H3N2		2011	USA	A/Boston/DOA48/2011	agtgatgccccattccttgatcggcttcgccgagatcag aggtccctaaggggaagaggcaatactc	-	-
CY111346	Human H3N2 IAVs	Human	H3N2		2011	USA	A/Boston/DOA61/2011	agtgatgccccattccttgatcggcttcgccgagatcag aggtccctaaggggaagaggcaatactc	-	-
CY134613	Human H3N2 IAVs	Human	H3N2		2011	USA	A/Boston/DOA63/2011	agtgatgccccattccttgatcggcttcgccgagatcag aggtccctaaggggaagaggcaatactc	-	-
CY111362	Human H3N2 IAVs	Human	H3N2		2011	USA	A/Boston/DOA67/2011	agtgatgccccattccttgatcggcttcgccgagatcag aggtccctaaggggaagaggcaatactc	-	-
CY111218	Human H3N2 IAVs	Human	H3N2		2011	USA	A/Boston/DOA13/2011	agtgatgccccattccttgatcggcttcgccgagatcag aggtccctaaggggaagaggcaatactc	-	-
CY134549	Human H3N2 IAVs	Human	H3N2		2011	USA	A/Boston/DOA45/2011	agtgatgccccattccttgatcggcttcgccgagatcag aggtccctaaggggaagaggcaatactc	-	-
CY111314	Human H3N2 IAVs	Human	H3N2		2011	USA	A/Boston/DOA51/2011	agtgatgccccattccttgatcggcttcgccgagatcag aggtccctaaggggaagaggcaatactc	-	-
CY111330	Human H3N2 IAVs	Human	H3N2		2011	USA	A/Boston/DOA55/2011	agtgatgccccattccttgatcggcttcgccgagatcag aggtccctaaggggaagaggcaatactc	-	-
CY111354	Human H3N2 IAVs	Human	H3N2		2011	USA	A/Boston/DOA66/2011	agtgatgccccattccttgatcggcttcgccgagatcag aggtccctaaggggaagaggcaatactc	-	-
CY111426	Human H3N2 IAVs	Human	H3N2		2011	USA	A/Boston/DOA79/2011	agtgatgccccattccttgatcggcttcgccgagatcag aggtccctaaggggaagaggcaatactc	-	-
KC882559	Human H3N2 IAVs	Human	H3N2		2011	USA	A/New_Jersey/01/2011	agtgatgccccattccttgatcggcttcgccgagatcag aggtccctaaggggaagaggcaatactc	-	-
CY091569	Human H3N2 IAVs	Human	H3N2		2011	USA	A/South_Carolina/NHRC0001/2011	agtgatgccccattccttgatcggcttcgccgagatcag aggtccctaaggggaagaggcaatactc	-	-

CY167624	Human H3N2 IAVs	Human	H3N2		2011	USA	A/Tennessee/F2054A/2011	agtgatgccccattccttgatcggcttcgccgagatcag<br>aggtccctaaggggaagaggcaatactc	-	-
CY167632	Human H3N2 IAVs	Human	H3N2		2011	USA	A/Tennessee/F2057c54A/2011	agtgatgccccattccttgatcggcttcgccgagatcag<br>aggtccctaaggggaagaggcaatactc	-	-
CY111394	Human H3N2 IAVs	Human	H3N2		2011	USA	A/Boston/DOA64/2011	agtgatgccccattccttgatcggcttcgccgagatcag<br>aggtccctaaggggaagaggcaatactc	-	-
CY139590	Human H3N2 IAVs	Human	H3N2		2011	USA	A/Boston/DOA18/2011	agtgatgccccattccttgatcggcttcgccgagatcag<br>aggtccctaaggggaagaggcaatactc	-	-
CY111186	Human H3N2 IAVs	Human	H3N2		2011	USA	A/Boston/DOA09/2011	agtgatgccccattccttgatcggcttcgccgagatcag<br>aggtccctaaggggaagaggcaatactc	-	-
CY117585	Human H3N2 IAVs	Human	H3N2		2011	USA	A/Boston/DOA42/2011	agtgatgccccattccttgatcggcttcgccgagatcag<br>aggtccctaaggggaagaggcaatactc	-	-
CY111458	Human H3N2 IAVs	Human	H3N2		2011	USA	A/Boston/DOA85/2011	agtgatgccccattccttgatcggcttcgccgagatcag<br>aggtccctaaggggaagaggcaatactc	-	-
KF014665	Human H3N2 IAVs	Human	H3N2		2011	Singapore	A/Singapore/C2011.301/2011	agtgatgccccattccttgatcggcttcgccgagatcag<br>aggtccctaaggggaagaggcaatactc	-	-
KF014739	Human H3N2 IAVs	Human	H3N2		2011	Singapore	A/Singapore/C2011.301/2011	agtgatgccccattccttgatcggcttcgccgagatcag<br>aggtccctaaggggaagaggcaatactc	-	-
KF014668	Human H3N2 IAVs	Human	H3N2		2011	Singapore	A/Singapore/C2011.422/2011	agtgatgccccattccttgatcggcttcgccgagatcag<br>aggtccctaaggggaagaggcaatactc	-	-
KF014743	Human H3N2 IAVs	Human	H3N2		2011	Singapore	A/Singapore/C2011.422/2011	agtgatgccccattccttgatcggcttcgccgagatcag<br>aggtccctaaggggaagaggcaatactc	-	-
KF014745	Human H3N2 IAVs	Human	H3N2		2011	Singapore	A/Singapore/C2011.458/2011	agtgatgccccattccttgatcggcttcgccgagatcag<br>aggtccctaaggggaagaggcaatactc	-	-
KF014670	Human H3N2 IAVs	Human	H3N2		2011	Singapore	A/Singapore/C2011.458/2011	agtgatgccccattccttgatcggcttcgccgagatcag<br>aggtccctaaggggaagaggcaatactc	-	-
KF014671	Human H3N2 IAVs	Human	H3N2		2011	Singapore	A/Singapore/C2011.471/2011	agtgatgccccattccttgatcggcttcgccgagatcag<br>aggtccctaaggggaagaggcaatactc	-	-
KF014746	Human H3N2 IAVs	Human	H3N2		2011	Singapore	A/Singapore/C2011.471/2011	agtgatgccccattccttgatcggcttcgccgagatcag<br>aggtccctaaggggaagaggcaatactc	-	-
KF014747	Human H3N2 IAVs	Human	H3N2		2011	Singapore	A/Singapore/C2011.477/2011	agtgatgccccattccttgatcggcttcgccgagatcag<br>aggtccctaaggggaagaggcaatactc	-	-
KF014672	Human H3N2 IAVs	Human	H3N2		2011	Singapore	A/Singapore/C2011.477/2011	agtgatgccccattccttgatcggcttcgccgagatcag<br>aggtccctaaggggaagaggcaatactc	-	-
KF014748	Human H3N2 IAVs	Human	H3N2		2011	Singapore	A/Singapore/C2011.493/2011	agtgatgccccattccttgatcggcttcgccgagatcag<br>aggtccctaaggggaagaggcaatactc	-	-
JX437781	Human H3N2 IAVs	Human	H3N2		2011	Singapore	A/Singapore/C2011.507V/2011	agtgatgccccattccttgatcggcttcgccgagatcag<br>aggtccctaaggggaagaggcaatactc	-	-
KF014750	Human H3N2 IAVs	Human	H3N2		2011	Singapore	A/Singapore/C2011.507V/2011	agtgatgccccattccttgatcggcttcgccgagatcag<br>aggtccctaaggggaagaggcaatactc	-	-
JX437902	Human H3N2 IAVs	Human	H3N2		2011	Singapore	A/Singapore/C2011.545C/2011	agtgatgccccattccttgatcggcttcgccgagatcag<br>aggtccctaaggggaagaggcaatactc	-	-
KF014677	Human H3N2 IAVs	Human	H3N2		2011	Singapore	A/Singapore/C2011.545C/2011	agtgatgccccattccttgatcggcttcgccgagatcag<br>aggtccctaaggggaagaggcaatactc	-	-
JX437782	Human H3N2 IAVs	Human	H3N2		2011	Singapore	A/Singapore/C2011.584V/2011	agtgatgccccattccttgatcggcttcgccgagatcag<br>aggtccctaaggggaagaggcaatactc	-	-
KF014755	Human H3N2 IAVs	Human	H3N2		2011	Singapore	A/Singapore/C2011.584V/2011	agtgatgccccattccttgatcggcttcgccgagatcag<br>aggtccctaaggggaagaggcaatactc	-	-
KF014774	Human H3N2 IAVs	Human	H3N2		2011	Singapore	A/Singapore/H2011.460a/2011	agtgatgccccattccttgatcggcttcgccgagatcag<br>aggtccctaaggggaagaggcaatactc	-	-

KF014708	Human H3N2 IAVs	Human	H3N2		2011	Singapore	A/Singapore/H2011.460a/2011	agtgatgccccattccttgatcggcttcgccgagatcag aggtccctaaggggaagaggcaatactc	-	-
KF014709	Human H3N2 IAVs	Human	H3N2		2011	Singapore	A/Singapore/H2011.460cC/2011	agtgatgccccattccttgatcggcttcgccgagatcag aggtccctaaggggaagaggcaatactc	-	-
JX437898	Human H3N2 IAVs	Human	H3N2		2011	Singapore	A/Singapore/H2011.460cC/2011	agtgatgccccattccttgatcggcttcgccgagatcag aggtccctaaggggaagaggcaatactc	-	-
KF014710	Human H3N2 IAVs	Human	H3N2		2011	Singapore	A/Singapore/H2011.463/2011	agtgatgccccattccttgatcggcttcgccgagatcag aggtccctaaggggaagaggcaatactc	-	-
KF014775	Human H3N2 IAVs	Human	H3N2		2011	Singapore	A/Singapore/H2011.463/2011	agtgatgccccattccttgatcggcttcgccgagatcag aggtccctaaggggaagaggcaatactc	-	-
KF014711	Human H3N2 IAVs	Human	H3N2		2011	Singapore	A/Singapore/H2011.471a/2011	agtgatgccccattccttgatcggcttcgccgagatcag aggtccctaaggggaagaggcaatactc	-	-
KF014776	Human H3N2 IAVs	Human	H3N2		2011	Singapore	A/Singapore/H2011.471a/2011	agtgatgccccattccttgatcggcttcgccgagatcag aggtccctaaggggaagaggcaatactc	-	-
KF014712	Human H3N2 IAVs	Human	H3N2		2011	Singapore	A/Singapore/H2011.471b/2011	agtgatgccccattccttgatcggcttcgccgagatcag aggtccctaaggggaagaggcaatactc	-	-
KF014777	Human H3N2 IAVs	Human	H3N2		2011	Singapore	A/Singapore/H2011.471b/2011	agtgatgccccattccttgatcggcttcgccgagatcag aggtccctaaggggaagaggcaatactc	-	-
KF014778	Human H3N2 IAVs	Human	H3N2		2011	Singapore	A/Singapore/H2011.471c/2011	agtgatgccccattccttgatcggcttcgccgagatcag aggtccctaaggggaagaggcaatactc	-	-
KF014713	Human H3N2 IAVs	Human	H3N2		2011	Singapore	A/Singapore/H2011.471c/2011	agtgatgccccattccttgatcggcttcgccgagatcag aggtccctaaggggaagaggcaatactc	-	-
KF014781	Human H3N2 IAVs	Human	H3N2		2011	Singapore	A/Singapore/H2011.482/2011	agtgatgccccattccttgatcggcttcgccgagatcag aggtccctaaggggaagaggcaatactc	-	-
KF014716	Human H3N2 IAVs	Human	H3N2		2011	Singapore	A/Singapore/H2011.482/2011	agtgatgccccattccttgatcggcttcgccgagatcag aggtccctaaggggaagaggcaatactc	-	-
KF014782	Human H3N2 IAVs	Human	H3N2		2011	Singapore	A/Singapore/H2011.496/2011	agtgatgccccattccttgatcggcttcgccgagatcag aggtccctaaggggaagaggcaatactc	-	-
KF014717	Human H3N2 IAVs	Human	H3N2		2011	Singapore	A/Singapore/H2011.496/2011	agtgatgccccattccttgatcggcttcgccgagatcag aggtccctaaggggaagaggcaatactc	-	-
KF014721	Human H3N2 IAVs	Human	H3N2		2011	Singapore	A/Singapore/H2011.515/2011	agtgatgccccattccttgatcggcttcgccgagatcag aggtccctaaggggaagaggcaatactc	-	-
KF014785	Human H3N2 IAVs	Human	H3N2		2011	Singapore	A/Singapore/H2011.515/2011	agtgatgccccattccttgatcggcttcgccgagatcag aggtccctaaggggaagaggcaatactc	-	-
KM070087	Human H3N2 IAVs	Human	H3N2		2011	Singapore	A/Singapore/S2011.740/2011	agtgatgccccattccttgatcggcttcgccgagatcag aggtccctaaggggaagaggcaatactc	-	-
KF014673	Human H3N2 IAVs	Human	H3N2		2011	Singapore	A/Singapore/C2011.493/2011	agtgatgccccattccttgatcggcttcgccgagatcag aggtccctaaggggaagaggcaatactc	-	-
JX437780	Human H3N2 IAVs	Human	H3N2		2011	Singapore	A/Singapore/C2011.362V/2011	agtgatgccccattccttgatcggcttcgccgagatcag aggtccctaaggggaagaggcaatactc	-	-
KF014740	Human H3N2 IAVs	Human	H3N2		2011	Singapore	A/Singapore/C2011.362V/2011	agtgatgccccattccttgatcggcttcgccgagatcag aggtccctaaggggaagaggcaatactc	-	-
KF014666	Human H3N2 IAVs	Human	H3N2		2011	Singapore	A/Singapore/C2011.384/2011	agtgatgccccattccttgatcggcttcgccgagatcag aggtccctaaggggaagaggcaatactc	-	-
KF014741	Human H3N2 IAVs	Human	H3N2		2011	Singapore	A/Singapore/C2011.384/2011	agtgatgccccattccttgatcggcttcgccgagatcag aggtccctaaggggaagaggcaatactc	-	-
KF014718	Human H3N2 IAVs	Human	H3N2		2011	Singapore	A/Singapore/H2011.499a/2011	agtgatgccccattccttgatcggcttcgccgagatcag aggtccctaaggggaagaggcaatactc	-	-
KF014783	Human H3N2 IAVs	Human	H3N2		2011	Singapore	A/Singapore/H2011.499a/2011	agtgatgccccattccttgatcggcttcgccgagatcag aggtccctaaggggaagaggcaatactc	-	-

KF014724	Human H3N2 IAVs	Human	H3N2		2011	Singapore	A/Singapore/H2011.704C/2011	agtgatgccccattccttgatcggcttcgccgagatcag aggtccctaaggggaagaggcaatactc	-	-
JX437900	Human H3N2 IAVs	Human	H3N2		2011	Singapore	A/Singapore/H2011.704C/2011	agtgatgccccattccttgatcggcttcgccgagatcag aggtccctaaggggaagaggcaatactc	-	-
KM070089	Human H3N2 IAVs	Human	H3N2		2011	Singapore	A/Singapore/H2011.795/2011	agtgatgccccattccttgatcggcttcgccgagatcag aggtccctaaggggaagaggcaatactc	-	-
KF014780	Human H3N2 IAVs	Human	H3N2		2011	Singapore	A/Singapore/H2011.479/2011	agtgatgccccattccttgatcggcttcgccgagatcag aggtccctaaggggaagaggcaatactc	-	-
KF014715	Human H3N2 IAVs	Human	H3N2		2011	Singapore	A/Singapore/H2011.479/2011	agtgatgccccattccttgatcggcttcgccgagatcag aggtccctaaggggaagaggcaatactc	-	-
KF014683	Human H3N2 IAVs	Human	H3N2		2011	Singapore	A/Singapore/C2011.803/2011	agtgatgccccattccttgatcggcttcgccgagatcag aggtccctaaggggaagaggcaatactc	-	-
KF014759	Human H3N2 IAVs	Human	H3N2		2011	Singapore	A/Singapore/C2011.803/2011	agtgatgccccattccttgatcggcttcgccgagatcag aggtccctaaggggaagaggcaatactc	-	-
KF014790	Human H3N2 IAVs	Human	H3N2		2011	Singapore	A/Singapore/H2011.808a/2011	agtgatgccccattccttgatcggcttcgccgagatcag aggtccctaaggggaagaggcaatactc	-	-
KF014727	Human H3N2 IAVs	Human	H3N2		2011	Singapore	A/Singapore/H2011.808a/2011	agtgatgccccattccttgatcggcttcgccgagatcag aggtccctaaggggaagaggcaatactc	-	-
KF014728	Human H3N2 IAVs	Human	H3N2		2011	Singapore	A/Singapore/H2011.808bC/2011	agtgatgccccattccttgatcggcttcgccgagatcag aggtccctaaggggaagaggcaatactc	-	-
JX437901	Human H3N2 IAVs	Human	H3N2		2011	Singapore	A/Singapore/H2011.808bC/2011	agtgatgccccattccttgatcggcttcgccgagatcag aggtccctaaggggaagaggcaatactc	-	-
KM070090	Human H3N2 IAVs	Human	H3N2		2011	Singapore	A/Singapore/H2011.814/2011	agtgatgccccattccttgatcggcttcgccgagatcag aggtccctaaggggaagaggcaatactc	-	-
KM070093	Human H3N2 IAVs	Human	H3N2		2011	Singapore	A/Singapore/H2011.871/2011	agtgatgccccattccttgatcggcttcgccgagatcag aggtccctaaggggaagaggcaatactc	-	-
KF014738	Human H3N2 IAVs	Human	H3N2		2011	Singapore	A/Singapore/C2011.244/2011	agtgatgccccattccttgatcggcttcgccgagatcag aggtccctaaggggaagaggcaatactc	-	-
KF014664	Human H3N2 IAVs	Human	H3N2		2011	Singapore	A/Singapore/C2011.244/2011	agtgatgccccattccttgatcggcttcgccgagatcag aggtccctaaggggaagaggcaatactc	-	-
KF014707	Human H3N2 IAVs	Human	H3N2		2011	Singapore	A/Singapore/H2011.447/2011	agtgatgccccattccttgatcggcttcgccgagatcag aggtccctaaggggaagaggcaatactc	-	-
KF014773	Human H3N2 IAVs	Human	H3N2		2011	Singapore	A/Singapore/H2011.447/2011	agtgatgccccattccttgatcggcttcgccgagatcag aggtccctaaggggaagaggcaatactc	-	-
KM070085	Human H3N2 IAVs	Human	H3N2		2011	Singapore	A/Singapore/S2011.701a/2011	agtgatgccccattccttgatcggcttcgccgagatcag aggtccctaaggggaagaggcaatactc	-	-
CY092293	Human H3N2 IAVs	Human	H3N2		2011	Nicaragua	A/Managua/38_11/2011	agtgatgccccattccttgatcggcttcgccgagatcag aggtccctaaggggaagaggcaatactc	-	-
CY092301	Human H3N2 IAVs	Human	H3N2		2011	Nicaragua	A/Managua/58_11/2011	agtgatgccccattccttgatcggcttcgccgagatcag aggtccctaaggggaagaggcaatactc	-	-
CY111322	Human H3N2 IAVs	Human	H3N2		2011	USA	A/Boston/DOA52/2011	agtgatgccccattccttgatcggcttcgccgagatcag aggtccctaaggggaagaggcaatactc	-	-
KC882907	Human H3N2 IAVs	Human	H3N2		2011	USA	A/California/16/2011	agtgatgccccattccttgatcggcttcgccgagatcag aggtccctaaggggaagaggcaatactc	-	-
KF014726	Human H3N2 IAVs	Human	H3N2		2011	Singapore	A/Singapore/H2011.797/2011	agtgatgccccattccttgatcggcttcgccgagatcag aggtccctaaggggaagaggcaatactc	-	-
KF014789	Human H3N2 IAVs	Human	H3N2		2011	Singapore	A/Singapore/H2011.797/2011	agtgatgccccattccttgatcggcttcgccgagatcag aggtccctaaggggaagaggcaatactc	-	-
CY134605	Human H3N2 IAVs	Human	H3N2		2011	USA	A/Boston/DOA60/2011	agtgatgccccattccttgatcggcttcgccgagatcag aggtccctaaggggaagaggcaatactc	-	-

CY111450	Human H3N2 IAVs	Human	H3N2		2011	USA	A/Boston/DOA82/2011	agtgatgccccattccttgatcggcttcgccgagatcag aggtccctaaggggaagaggcaatactc	-	-
KJ855343	Human H3N2 IAVs	Human	H3N2		2011	Mexico	A/Mexico/VER60/2011	agtgatgccccattccttgatcggcttcgccgagatcag aggtccctaaggggaagaggcaatactc	-	-
CY134597	Human H3N2 IAVs	Human	H3N2		2011	USA	A/Boston/DOA54/2011	agtgatgccccattccttgatcggcttcgccgagatcag aggtccctaaggggaagaggcaatactc	-	-
CY111138	Human H3N2 IAVs	Human	H3N2		2011	USA	A/Boston/DOA02/2011	agtgatgccccattccttgatcggcttcgccgagatcag aggtccctaaggggaagaggcaatactc	-	-
KF014779	Human H3N2 IAVs	Human	H3N2		2011	Singapore	A/Singapore/H2011.474/2011	agtgatgccccattccttgatcggcttcgccgagatcag aggtccctaaggggaagaggcaatactc	-	-
KF014714	Human H3N2 IAVs	Human	H3N2		2011	Singapore	A/Singapore/H2011.474/2011	agtgatgccccattccttgatcggcttcgccgagatcag aggtccctaaggggaagaggcaatactc	-	-
CY134509	Human H3N2 IAVs	Human	H3N2		2011	USA	A/Boston/DOA36/2011	agtgatgccccattccttgatcggcttcgccgagatcag aggtccctaaggggaagaggcaatactc	-	-
KM070091	Human H3N2 IAVs	Human	H3N2		2011	Singapore	A/Singapore/C2011.860/2011	agtgatgccccattccttgatcggcttcgccgagatcag aggtccctaaggggaagaggcaatactc	-	-
KM070086	Human H3N2 IAVs	Human	H3N2		2011	Singapore	A/Singapore/S2011.737/2011	agtgatgccccattccttgatcggcttcgccgagatcag aggtccctaaggggaagaggcaatactc	-	-
KJ942684	Human H3N2 IAVs	Human	H3N2		2011	Australia	A/Victoria/361/2011	agtgatgccccattccttgatcggcttcgccgagatcag aggtccctaaggggaagaggcaatactc	-	-
CY111234	Human H3N2 IAVs	Human	H3N2		2011	USA	A/Boston/DOA11/2011	agtgatgccccattccttgatcggcttcgccgagatcag aggtccctaaggggaagaggcaatactc	-	-
CY117593	Human H3N2 IAVs	Human	H3N2		2011	USA	A/Boston/DOA74/2011	agtgatgccccattccttgatcggcttcgccgagatcag aggtccctaaggggaagaggcaatactc	-	-
CY167568	Human H3N2 IAVs	Human	H3N2		2011	USA	A/Tennessee/F2031A/2011	agtgatgccccattccttgatcggcttcgccgagatcag aggtccctaaggggaagaggcaatactc	-	-
CY134565	Human H3N2 IAVs	Human	H3N2		2011	USA	A/Boston/DOA47/2011	agtgatgccccattccttgatcggcttcgccgagatcag aggtccctaaggggaagaggcaatactc	-	-
KF014760	Human H3N2 IAVs	Human	H3N2		2011	Singapore	A/Singapore/C2011.825/2011	agtgatgccccattccttgatcggcttcgccgagatcag aggtccctaaggggaagaggcaatactc	-	-
KF014684	Human H3N2 IAVs	Human	H3N2		2011	Singapore	A/Singapore/C2011.825/2011	agtgatgccccattccttgatcggcttcgccgagatcag aggtccctaaggggaagaggcaatactc	-	-
KF014725	Human H3N2 IAVs	Human	H3N2		2011	Singapore	A/Singapore/H2011.751/2011	agtgatgccccattccttgatcggcttcgccgagatcag aggtccctaaggggaagaggcaatactc	-	-
KF014788	Human H3N2 IAVs	Human	H3N2		2011	Singapore	A/Singapore/H2011.751/2011	agtgatgccccattccttgatcggcttcgccgagatcag aggtccctaaggggaagaggcaatactc	-	-
KM070080	Human H3N2 IAVs	Human	H3N2		2011	Singapore	A/Singapore/S2011.090/2011	agtgatgccccattccttgatcggcttcgccgagatcag aggtccctaaggggaagaggcaatactc	-	-
KC883399	Human H3N2 IAVs	Human	H3N2		2011	USA	A/Alaska/15/2011	agtgatgccccattccttgatcggcttcgccgagatcag aggtccctaaggggaagaggcaatactc	-	-
CY111338	Human H3N2 IAVs	Human	H3N2		2011	USA	A/Boston/DOA57/2011	agtgatgccccattccttgatcggcttcgccgagatcag aggtccctaaggggaagaggcaatactc	-	-
CY111482	Human H3N2 IAVs	Human	H3N2		2011	USA	A/Boston/DOA75/2011	agtgatgccccattccttgatcggcttcgccgagatcag aggtccctaaggggaagaggcaatactc	-	-
CY111466	Human H3N2 IAVs	Human	H3N2		2011	USA	A/Boston/DOA86/2011	agtgatgccccattccttgatcggcttcgccgagatcag aggtccctaaggggaagaggcaatactc	-	-
CY091585	Human H3N2 IAVs	Human	H3N2		2011	USA	A/California/NHRC0001/2011	agtgatgccccattccttgatcggcttcgccgagatcag aggtccctaaggggaagaggcaatactc	-	-
CY092269	Human H3N2 IAVs	Human	H3N2		2011	USA	A/California/NHRC0003/2011	agtgatgccccattccttgatcggcttcgccgagatcag aggtccctaaggggaagaggcaatactc	-	-

CY093571	Human H3N2 IAVs	Human	H3N2		2011	Russia	A/Moscow/WRAIR4307N/2011	agtgatgccccattccttgatcggcttcgccgagatcag aggtccctaaggggaagaggcaatactc	-	-
CY093579	Human H3N2 IAVs	Human	H3N2		2011	Russia	A/Moscow/WRAIR4307T/2011	agtgatgccccattccttgatcggcttcgccgagatcag aggtccctaaggggaagaggcaatactc	-	-
KC883243	Human H3N2 IAVs	Human	H3N2		2011	USA	A/North_Carolina/05/2011	agtgatgccccattccttgatcggcttcgccgagatcag aggtccctaaggggaagaggcaatactc	-	-
KF612209	Human H3N2 IAVs	Human	H3N2		2011	Bolivia	A/Santa_Cruz/174/2011	agtgatgccccattccttgatcggcttcgccgagatcag aggtccctaaggggaagaggcaatactc	-	-
KC882635	Human H3N2 IAVs	Human	H3N2		2011	USA	A/Texas/06/2011	agtgatgccccattccttgatcggcttcgccgagatcag aggtccctaaggggaagaggcaatactc	-	-
JX437779	Human H3N2 IAVs	Human	H3N2		2011	Singapore	A/Singapore/C2011.027V/2011	agtgatgccccattccttgatcggcttcgccgagatcag aggtccctaaggggaagaggcaatactc	-	-
KF014736	Human H3N2 IAVs	Human	H3N2		2011	Singapore	A/Singapore/C2011.027V/2011	agtgatgccccattccttgatcggcttcgccgagatcag aggtccctaaggggaagaggcaatactc	-	-
CY167520	Human H3N2 IAVs	Human	H3N2		2011	USA	A/Tennessee/F2019/2011	agtgatgccccattccttgatcggcttcgccgagatcag aggtccctaaggggaagaggcaatactc	-	-
CY167536	Human H3N2 IAVs	Human	H3N2		2011	USA	A/Tennessee/F2021/2011	agtgatgccccattccttgatcggcttcgccgagatcag aggtccctaaggggaagaggcaatactc	-	-
CY167544	Human H3N2 IAVs	Human	H3N2		2011	USA	A/Tennessee/F2021B/2011	agtgatgccccattccttgatcggcttcgccgagatcag aggtccctaaggggaagaggcaatactc	-	-
CY167600	Human H3N2 IAVs	Human	H3N2		2011	USA	A/Tennessee/F2048/2011	agtgatgccccattccttgatcggcttcgccgagatcag aggtccctaaggggaagaggcaatactc	-	-
CY167608	Human H3N2 IAVs	Human	H3N2		2011	USA	A/Tennessee/F2048A/2011	agtgatgccccattccttgatcggcttcgccgagatcag aggtccctaaggggaagaggcaatactc	-	-
CY167672	Human H3N2 IAVs	Human	H3N2		2011	USA	A/Tennessee/F2078/2011	agtgatgccccattccttgatcggcttcgccgagatcag aggtccctaaggggaagaggcaatactc	-	-
CY167680	Human H3N2 IAVs	Human	H3N2		2011	USA	A/Tennessee/F2078A/2011	agtgatgccccattccttgatcggcttcgccgagatcag aggtccctaaggggaagaggcaatactc	-	-
CY167688	Human H3N2 IAVs	Human	H3N2		2011	USA	A/Tennessee/F2079/2011	agtgatgccccattccttgatcggcttcgccgagatcag aggtccctaaggggaagaggcaatactc	-	-
CY167696	Human H3N2 IAVs	Human	H3N2		2011	USA	A/Tennessee/F2079A/2011	agtgatgccccattccttgatcggcttcgccgagatcag aggtccctaaggggaagaggcaatactc	-	-
CY167704	Human H3N2 IAVs	Human	H3N2		2011	USA	A/Tennessee/F2079B/2011	agtgatgccccattccttgatcggcttcgccgagatcag aggtccctaaggggaagaggcaatactc	-	-
CY167712	Human H3N2 IAVs	Human	H3N2		2011	USA	A/Tennessee/F2080c7879/2011	agtgatgccccattccttgatcggcttcgccgagatcag aggtccctaaggggaagaggcaatactc	-	-
CY167744	Human H3N2 IAVs	Human	H3N2		2011	USA	A/Tennessee/F2084c83A/2011	agtgatgccccattccttgatcggcttcgccgagatcag aggtccctaaggggaagaggcaatactc	-	-
CY167768	Human H3N2 IAVs	Human	H3N2		2011	USA	A/Tennessee/F2090B/2011	agtgatgccccattccttgatcggcttcgccgagatcag aggtccctaaggggaagaggcaatactc	-	-
CY092285	Human H3N2 IAVs	Human	H3N2		2011	USA	A/California/NHRC0002/2011	agtgatgccccattccttgatcggcttcgccgagatcag aggtccctaaggggaagaggcaatactc	-	-
CY092277	Human H3N2 IAVs	Human	H3N2		2011	USA	A/California/NHRC0004/2011	agtgatgccccattccttgatcggcttcgccgagatcag aggtccctaaggggaagaggcaatactc	-	-
KF612211	Human H3N2 IAVs	Human	H3N2		2011	Bolivia	A/Santa_Cruz/637/2011	agtgatgccccattccttgatcggcttcgccgagatcag aggtccctaaggggaagaggcaatactc	-	-
CY091561	Human H3N2 IAVs	Human	H3N2		2011	USA	A/Texas/NHRC0001/2011	agtgatgccccattccttgatcggcttcgccgagatcag aggtccctaaggggaagaggcaatactc	-	-
CY167648	Human H3N2 IAVs	Human	H3N2		2011	USA	A/Tennessee/F2065/2011	agtgatgccccattccttgatcggcttcgccgagatcag aggtccctaaggggaagaggcaatactc	-	-

CY167656	Human H3N2 IAVs	Human	H3N2		2011	USA	A/Tennessee/F2065B/2011	agtgatgccccattccttgatcggcttcgccgagatcag<br>aggtccctaaggggaagaggcaatactc	-	-
CY111410	Human H3N2 IAVs	Human	H3N2		2011	USA	A/Boston/DOA77/2011	agtgatgccccattccttgatcggcttcgccgagatcag<br>aggtccctaaggggaagaggcaatactc	-	-
KC883052	Human H3N2 IAVs	Human	H3N2		2011	USA	A/Wisconsin/22/2011	agtgatgccccattccttgatcggcttcgccgagatcag<br>aggtccctaaggggaagaggcaatactc	-	-
CY111226	Human H3N2 IAVs	Human	H3N2		2011	USA	A/Boston/DOA10/2011	agtgatgccccattccttgatcggcttcgccgagatcag<br>aggtccctaaggggaagaggcaatactc	-	-
KJ942796	Human H3N2 IAVs	Human	H3N2		2011	Australia	A/Brisbane/299/2011	agtgatgccccattccttgatcggcttcgccgagatcag<br>aggtccctaaggggaagaggcaatactc	-	-
CY116703	Human H3N2 IAVs	Human	H3N2		2011	USA	A/Boston/DOA16/2011	agtgatgccccattccttgatcggcttcgccgagatcag<br>aggtccctaaggggaagaggcaatactc	-	-
CY111370	Human H3N2 IAVs	Human	H3N2		2011	USA	A/Boston/DOA69/2011	agtgatgccccattccttgatcggcttcgccgagatcag<br>aggtccctaaggggaagaggcaatactc	-	-
CY111474	Human H3N2 IAVs	Human	H3N2		2011	USA	A/Boston/DOA87/2011	agtgatgccccattccttgatcggcttcgccgagatcag<br>aggtccctaaggggaagaggcaatactc	-	-
CY134581	Human H3N2 IAVs	Human	H3N2		2011	USA	A/Boston/DOA50/2011	agtgatgccccattccttgatcggcttcgccgagatcag<br>aggtccctaaggggaagaggcaatactc	-	-
CY111442	Human H3N2 IAVs	Human	H3N2		2011	USA	A/Boston/DOA81/2011	agtgatgccccattccttgatcggcttcgccgagatcag<br>aggtccctaaggggaagaggcaatactc	-	-
CY111154	Human H3N2 IAVs	Human	H3N2		2011	USA	A/Boston/DOA04/2011	agtgatgccccattccttgatcggcttcgccgagatcag<br>aggtccctaaggggaagaggcaatactc	-	-
CY111130	Human H3N2 IAVs	Human	H3N2		2011	USA	A/Boston/DOA01/2011	agtgatgccccattccttgatcggcttcgccgagatcag<br>aggtccctaaggggaagaggcaatactc	-	-
CY111162	Human H3N2 IAVs	Human	H3N2		2011	USA	A/Boston/DOA05/2011	agtgatgccccattccttgatcggcttcgccgagatcag<br>aggtccctaaggggaagaggcaatactc	-	-
CY116719	Human H3N2 IAVs	Human	H3N2		2011	USA	A/Boston/DOA68/2011	agtgatgccccattccttgatcggcttcgccgagatcag<br>aggtccctaaggggaagaggcaatactc	-	-
CY111434	Human H3N2 IAVs	Human	H3N2		2011	USA	A/Boston/DOA80/2011	agtgatgccccattccttgatcggcttcgccgagatcag<br>aggtccctaaggggaagaggcaatactc	-	-
CY134517	Human H3N2 IAVs	Human	H3N2		2011	USA	A/Boston/DOA38/2011	agtgatgccccattccttgatcggcttcgccgagatcag<br>aggtccctaaggggaagaggcaatactc	-	-
CY111418	Human H3N2 IAVs	Human	H3N2		2011	USA	A/Boston/DOA78/2011	agtgatgccccattccttgatcggcttcgccgagatcag<br>aggtccctaaggggaagaggcaatactc	-	-
CY160332	Human H3N2 IAVs	Human	H3N2		2011	Peru	A/Peru/PER006/2011	agtgatgccccattccttgatcggcttcgccgagatcag<br>aggtccctaaggggaagaggcaatactc	-	-
CY160556	Human H3N2 IAVs	Human	H3N2		2011	Peru	A/Peru/PER034/2011	agtgatgccccattccttgatcggcttcgccgagatcag<br>aggtccctaaggggaagaggcaatactc	-	-
CY160628	Human H3N2 IAVs	Human	H3N2		2011	Peru	A/Peru/PER043/2011	agtgatgccccattccttgatcggcttcgccgagatcag<br>aggtccctaaggggaagaggcaatactc	-	-
CY160732	Human H3N2 IAVs	Human	H3N2		2011	Peru	A/Peru/PER056/2011	agtgatgccccattccttgatcggcttcgccgagatcag<br>aggtccctaaggggaagaggcaatactc	-	-
CY160772	Human H3N2 IAVs	Human	H3N2		2011	Peru	A/Peru/PER061/2011	agtgatgccccattccttgatcggcttcgccgagatcag<br>aggtccctaaggggaagaggcaatactc	-	-
CY161060	Human H3N2 IAVs	Human	H3N2		2011	Peru	A/Peru/PER098/2011	agtgatgccccattccttgatcggcttcgccgagatcag<br>aggtccctaaggggaagaggcaatactc	-	-
CY161308	Human H3N2 IAVs	Human	H3N2		2011	Peru	A/Peru/PER130/2011	agtgatgccccattccttgatcggcttcgccgagatcag<br>aggtccctaaggggaagaggcaatactc	-	-
CY161340	Human H3N2 IAVs	Human	H3N2		2011	Peru	A/Peru/PER134/2011	agtgatgccccattccttgatcggcttcgccgagatcag<br>aggtccctaaggggaagaggcaatactc	-	-

CY161364	Human H3N2 IAVs	Human	H3N2		2011	Peru	A/Peru/PER137/2011	agtgatgccccattccttgatcggcttcgccgagatcag aggtccctaaggggaagaggcaatactc	-	-
CY161404	Human H3N2 IAVs	Human	H3N2		2011	Peru	A/Peru/PER142/2011	agtgatgccccattccttgatcggcttcgccgagatcag aggtccctaaggggaagaggcaatactc	-	-
CY161460	Human H3N2 IAVs	Human	H3N2		2011	Peru	A/Peru/PER149/2011	agtgatgccccattccttgatcggcttcgccgagatcag aggtccctaaggggaagaggcaatactc	-	-
CY161484	Human H3N2 IAVs	Human	H3N2		2011	Peru	A/Peru/PER152/2011	agtgatgccccattccttgatcggcttcgccgagatcag aggtccctaaggggaagaggcaatactc	-	-
CY161532	Human H3N2 IAVs	Human	H3N2		2011	Peru	A/Peru/PER158/2011	agtgatgccccattccttgatcggcttcgccgagatcag aggtccctaaggggaagaggcaatactc	-	-
CY161700	Human H3N2 IAVs	Human	H3N2		2011	Peru	A/Peru/PER179/2011	agtgatgccccattccttgatcggcttcgccgagatcag aggtccctaaggggaagaggcaatactc	-	-
CY161748	Human H3N2 IAVs	Human	H3N2		2011	Peru	A/Peru/PER185/2011	agtgatgccccattccttgatcggcttcgccgagatcag aggtccctaaggggaagaggcaatactc	-	-
CY161892	Human H3N2 IAVs	Human	H3N2		2011	Peru	A/Peru/PER203/2011	agtgatgccccattccttgatcggcttcgccgagatcag aggtccctaaggggaagaggcaatactc	-	-
CY161908	Human H3N2 IAVs	Human	H3N2		2011	Peru	A/Peru/PER205/2011	agtgatgccccattccttgatcggcttcgccgagatcag aggtccctaaggggaagaggcaatactc	-	-
CY162116	Human H3N2 IAVs	Human	H3N2		2011	Peru	A/Peru/PER231/2011	agtgatgccccattccttgatcggcttcgccgagatcag aggtccctaaggggaagaggcaatactc	-	-
CY162148	Human H3N2 IAVs	Human	H3N2		2011	Peru	A/Peru/PER235/2011	agtgatgccccattccttgatcggcttcgccgagatcag aggtccctaaggggaagaggcaatactc	-	-
CY162204	Human H3N2 IAVs	Human	H3N2		2011	Peru	A/Peru/PER243/2011	agtgatgccccattccttgatcggcttcgccgagatcag aggtccctaaggggaagaggcaatactc	-	-
CY162212	Human H3N2 IAVs	Human	H3N2		2011	Peru	A/Peru/PER244/2011	agtgatgccccattccttgatcggcttcgccgagatcag aggtccctaaggggaagaggcaatactc	-	-
CY162292	Human H3N2 IAVs	Human	H3N2		2011	Peru	A/Peru/PER255/2011	agtgatgccccattccttgatcggcttcgccgagatcag aggtccctaaggggaagaggcaatactc	-	-
CY162364	Human H3N2 IAVs	Human	H3N2		2011	Peru	A/Peru/PER265/2011	agtgatgccccattccttgatcggcttcgccgagatcag aggtccctaaggggaagaggcaatactc	-	-
CY162388	Human H3N2 IAVs	Human	H3N2		2011	Peru	A/Peru/PER268/2011	agtgatgccccattccttgatcggcttcgccgagatcag aggtccctaaggggaagaggcaatactc	-	-
CY162396	Human H3N2 IAVs	Human	H3N2		2011	Peru	A/Peru/PER269/2011	agtgatgccccattccttgatcggcttcgccgagatcag aggtccctaaggggaagaggcaatactc	-	-
CY162412	Human H3N2 IAVs	Human	H3N2		2011	Peru	A/Peru/PER271/2011	agtgatgccccattccttgatcggcttcgccgagatcag aggtccctaaggggaagaggcaatactc	-	-
CY162500	Human H3N2 IAVs	Human	H3N2		2011	Peru	A/Peru/PER282/2011	agtgatgccccattccttgatcggcttcgccgagatcag aggtccctaaggggaagaggcaatactc	-	-
CY162508	Human H3N2 IAVs	Human	H3N2		2011	Peru	A/Peru/PER283/2011	agtgatgccccattccttgatcggcttcgccgagatcag aggtccctaaggggaagaggcaatactc	-	-
CY162540	Human H3N2 IAVs	Human	H3N2		2011	Peru	A/Peru/PER287/2011	agtgatgccccattccttgatcggcttcgccgagatcag aggtccctaaggggaagaggcaatactc	-	-
CY162556	Human H3N2 IAVs	Human	H3N2		2011	Peru	A/Peru/PER289/2011	agtgatgccccattccttgatcggcttcgccgagatcag aggtccctaaggggaagaggcaatactc	-	-
CY162612	Human H3N2 IAVs	Human	H3N2		2011	Peru	A/Peru/PER298/2011	agtgatgccccattccttgatcggcttcgccgagatcag aggtccctaaggggaagaggcaatactc	-	-
CY162852	Human H3N2 IAVs	Human	H3N2		2011	Peru	A/Peru/PER328/2011	agtgatgccccattccttgatcggcttcgccgagatcag aggtccctaaggggaagaggcaatactc	-	-
CY162988	Human H3N2 IAVs	Human	H3N2		2011	Peru	A/Peru/PER345/2011	agtgatgccccattccttgatcggcttcgccgagatcag aggtccctaaggggaagaggcaatactc	-	-

CY163132	Human H3N2 IAVs	Human	H3N2		2011	Peru	A/Peru/PER364/2011	agtgatgccccattccttgatcggcttcgccgagatcag aggtccctaaggggaagaggcaatactc	-	-
CY163228	Human H3N2 IAVs	Human	H3N2		2011	Peru	A/Peru/PER378/2011	agtgatgccccattccttgatcggcttcgccgagatcag aggtccctaaggggaagaggcaatactc	-	-
CY163356	Human H3N2 IAVs	Human	H3N2		2011	Peru	A/Peru/PER394/2011	agtgatgccccattccttgatcggcttcgccgagatcag aggtccctaaggggaagaggcaatactc	-	-
CY163380	Human H3N2 IAVs	Human	H3N2		2011	Peru	A/Peru/PER397/2011	agtgatgccccattccttgatcggcttcgccgagatcag aggtccctaaggggaagaggcaatactc	-	-
CY161540	Human H3N2 IAVs	Human	H3N2		2011	Peru	A/Peru/PER159/2011	agtgatgccccattccttgatcggcttcgccgagatcag aggtccctaaggggaagaggcaatactc	-	-
CY162804	Human H3N2 IAVs	Human	H3N2		2011	Peru	A/Peru/PER322/2011	agtgatgccccattccttgatcggcttcgccgagatcag aggtccctaaggggaagaggcaatactc	-	-
CY163284	Human H3N2 IAVs	Human	H3N2		2011	Peru	A/Peru/PER385/2011	agtgatgccccattccttgatcggcttcgccgagatcag aggtccctaaggggaagaggcaatactc	-	-
CY111170	Human H3N2 IAVs	Human	H3N2		2011	USA	A/Boston/DOA06/2011	agtgatgccccattccttgatcggcttcgccgagatcag aggtccctaaggggaagaggcaatactc	-	-
CY111194	Human H3N2 IAVs	Human	H3N2		2011	USA	A/Boston/DOA17/2011	agtgatgccccattccttgatcggcttcgccgagatcag aggtccctaaggggaagaggcaatactc	-	-
CY134485	Human H3N2 IAVs	Human	H3N2		2011	USA	A/Boston/DOA29/2011	agtgatgccccattccttgatcggcttcgccgagatcag aggtccctaaggggaagaggcaatactc	-	-
CY117563	Human H3N2 IAVs	Human	Mixed		2011	USA	A/Boston/DOA31/2011_mixed_	agtgatgccccattccttgatcggcttcgccgagatcag aggtccctaaggggaagaggcaatactc	-	-
CY117601	Human H3N2 IAVs	Human	H3N2		2011	USA	A/Boston/DOA83/2011	agtgatgccccattccttgatcggcttcgccgagatcag aggtccctaaggggaagaggcaatactc	-	-
CY160980	Human H3N2 IAVs	Human	H3N2		2011	Peru	A/Peru/PER087/2011	agtgatgccccattccttgatcggcttcgccgagatcag aggtccctaaggggaagaggcaatactc	-	-
CY162444	Human H3N2 IAVs	Human	H3N2		2011	Peru	A/Peru/PER275/2011	agtgatgccccattccttgatcggcttcgccgagatcag aggtccctaaggggaagaggcaatactc	-	-
CY161052	Human H3N2 IAVs	Human	H3N2		2011	Peru	A/Peru/PER097/2011	agtgatgccccattccttgatcggcttcgccgagatcag aggtccctaaggggaagaggcaatactc	-	-
CY161860	Human H3N2 IAVs	Human	H3N2		2011	Peru	A/Peru/PER199/2011	agtgatgccccattccttgatcggcttcgccgagatcag aggtccctaaggggaagaggcaatactc	-	-
CY162564	Human H3N2 IAVs	Human	H3N2		2011	Peru	A/Peru/PER290/2011	agtgatgccccattccttgatcggcttcgccgagatcag aggtccctaaggggaagaggcaatactc	-	-
CY162892	Human H3N2 IAVs	Human	H3N2		2011	Peru	A/Peru/PER333/2011	agtgatgccccattccttgatcggcttcgccgagatcag aggtccctaaggggaagaggcaatactc	-	-
CY163060	Human H3N2 IAVs	Human	H3N2		2011	Peru	A/Peru/PER355/2011	agtgatgccccattccttgatcggcttcgccgagatcag aggtccctaaggggaagaggcaatactc	-	-
CY162356	Human H3N2 IAVs	Human	H3N2		2011	Peru	A/Peru/PER264/2011	agtgatgccccattccttgatcggcttcgccgagatcag aggtccctaaggggaagaggcaatactc	-	-
CY111202	Human H3N2 IAVs	Human	H3N2		2011	USA	A/Boston/DOA15/2011	agtgatgccccattccttgatcggcttcgccgagatcag aggtccctaaggggaagaggcaatactc	-	-
CY161220	Human H3N2 IAVs	Human	H3N2		2011	Peru	A/Peru/PER118/2011	agtgatgccccattccttgatcggcttcgccgagatcag aggtccctaaggggaagaggcaatactc	-	-
CY162956	Human H3N2 IAVs	Human	H3N2		2011	Peru	A/Peru/PER341/2011	agtgatgccccattccttgatcggcttcgccgagatcag aggtccctaaggggaagaggcaatactc	-	-
CY167576	Human H3N2 IAVs	Human	H3N2		2011	USA	A/Tennessee/F2034/2011	agtgatgccccattccttgatcggcttcgccgagatcag aggtccctaaggggaagaggcaatactc	-	-
CY167616	Human H3N2 IAVs	Human	H3N2		2011	USA	A/Tennessee/F2053/2011	agtgatgccccattccttgatcggcttcgccgagatcag aggtccctaaggggaagaggcaatactc	-	-

CY167488	Human H3N2 IAVs	Human	H3N2		2011	USA	A/Tennessee/F2011/2011	agtgatgccccattccttgatcggcttcgccgagatcag aggtccctaaggggaagaggcaatactc	-	-
CY167504	Human H3N2 IAVs	Human	H3N2		2011	USA	A/Tennessee/F2018/2011	agtgatgccccattccttgatcggcttcgccgagatcag aggtccctaaggggaagaggcaatactc	-	-
CY167512	Human H3N2 IAVs	Human	H3N2		2011	USA	A/Tennessee/F2018A/2011	agtgatgccccattccttgatcggcttcgccgagatcag aggtccctaaggggaagaggcaatactc	-	-
CY167528	Human H3N2 IAVs	Human	H3N2		2011	USA	A/Tennessee/F2019A/2011	agtgatgccccattccttgatcggcttcgccgagatcag aggtccctaaggggaagaggcaatactc	-	-
CY167640	Human H3N2 IAVs	Human	H3N2		2011	USA	A/Tennessee/F2059/2011	agtgatgccccattccttgatcggcttcgccgagatcag aggtccctaaggggaagaggcaatactc	-	-
CY160412	Human H3N2 IAVs	Human	H3N2		2011	Peru	A/Peru/PER016/2011	agtgatgccccattccttgatcggcttcgccgagatcag aggtccctaaggggaagaggcaatactc	-	-
CY160444	Human H3N2 IAVs	Human	H3N2		2011	Peru	A/Peru/PER020/2011	agtgatgccccattccttgatcggcttcgccgagatcag aggtccctaaggggaagaggcaatactc	-	-
CY160500	Human H3N2 IAVs	Human	H3N2		2011	Peru	A/Peru/PER027/2011	agtgatgccccattccttgatcggcttcgccgagatcag aggtccctaaggggaagaggcaatactc	-	-
CY160596	Human H3N2 IAVs	Human	H3N2		2011	Peru	A/Peru/PER039/2011	agtgatgccccattccttgatcggcttcgccgagatcag aggtccctaaggggaagaggcaatactc	-	-
CY160636	Human H3N2 IAVs	Human	H3N2		2011	Peru	A/Peru/PER044/2011	agtgatgccccattccttgatcggcttcgccgagatcag aggtccctaaggggaagaggcaatactc	-	-
CY160660	Human H3N2 IAVs	Human	H3N2		2011	Peru	A/Peru/PER047/2011	agtgatgccccattccttgatcggcttcgccgagatcag aggtccctaaggggaagaggcaatactc	-	-
CY160820	Human H3N2 IAVs	Human	H3N2		2011	Peru	A/Peru/PER067/2011	agtgatgccccattccttgatcggcttcgccgagatcag aggtccctaaggggaagaggcaatactc	-	-
CY160836	Human H3N2 IAVs	Human	H3N2		2011	Peru	A/Peru/PER069/2011	agtgatgccccattccttgatcggcttcgccgagatcag aggtccctaaggggaagaggcaatactc	-	-
CY160972	Human H3N2 IAVs	Human	H3N2		2011	Peru	A/Peru/PER086/2011	agtgatgccccattccttgatcggcttcgccgagatcag aggtccctaaggggaagaggcaatactc	-	-
CY161124	Human H3N2 IAVs	Human	H3N2		2011	Peru	A/Peru/PER106/2011	agtgatgccccattccttgatcggcttcgccgagatcag aggtccctaaggggaagaggcaatactc	-	-
CY161252	Human H3N2 IAVs	Human	H3N2		2011	Peru	A/Peru/PER122/2011	agtgatgccccattccttgatcggcttcgccgagatcag aggtccctaaggggaagaggcaatactc	-	-
CY161324	Human H3N2 IAVs	Human	H3N2		2011	Peru	A/Peru/PER132/2011	agtgatgccccattccttgatcggcttcgccgagatcag aggtccctaaggggaagaggcaatactc	-	-
CY161508	Human H3N2 IAVs	Human	H3N2		2011	Peru	A/Peru/PER155/2011	agtgatgccccattccttgatcggcttcgccgagatcag aggtccctaaggggaagaggcaatactc	-	-
CY161676	Human H3N2 IAVs	Human	H3N2		2011	Peru	A/Peru/PER176/2011	agtgatgccccattccttgatcggcttcgccgagatcag aggtccctaaggggaagaggcaatactc	-	-
CY161820	Human H3N2 IAVs	Human	H3N2		2011	Peru	A/Peru/PER194/2011	agtgatgccccattccttgatcggcttcgccgagatcag aggtccctaaggggaagaggcaatactc	-	-
CY161916	Human H3N2 IAVs	Human	H3N2		2011	Peru	A/Peru/PER206/2011	agtgatgccccattccttgatcggcttcgccgagatcag aggtccctaaggggaagaggcaatactc	-	-
CY162084	Human H3N2 IAVs	Human	H3N2		2011	Peru	A/Peru/PER227/2011	agtgatgccccattccttgatcggcttcgccgagatcag aggtccctaaggggaagaggcaatactc	-	-
CY162236	Human H3N2 IAVs	Human	H3N2		2011	Peru	A/Peru/PER247/2011	agtgatgccccattccttgatcggcttcgccgagatcag aggtccctaaggggaagaggcaatactc	-	-
CY162428	Human H3N2 IAVs	Human	H3N2		2011	Peru	A/Peru/PER273/2011	agtgatgccccattccttgatcggcttcgccgagatcag aggtccctaaggggaagaggcaatactc	-	-
CY162516	Human H3N2 IAVs	Human	H3N2		2011	Peru	A/Peru/PER284/2011	agtgatgccccattccttgatcggcttcgccgagatcag aggtccctaaggggaagaggcaatactc	-	-

CY162820	Human H3N2 IAVs	Human	H3N2		2011	Peru	A/Peru/PER324/2011	agtgatgccccattccttgatcggcttcgccgagatcag<br>aggtccctaaggggaagaggcaatactc	-	-
CY163036	Human H3N2 IAVs	Human	H3N2		2011	Peru	A/Peru/PER352/2011	agtgatgccccattccttgatcggcttcgccgagatcag<br>aggtccctaaggggaagaggcaatactc	-	-
CY163372	Human H3N2 IAVs	Human	H3N2		2011	Peru	A/Peru/PER396/2011	agtgatgccccattccttgatcggcttcgccgagatcag<br>aggtccctaaggggaagaggcaatactc	-	-
CY160572	Human H3N2 IAVs	Human	H3N2		2011	Peru	A/Peru/PER036/2011	agtgatgccccattccttgatcggcttcgccgagatcag<br>aggtccctaaggggaagaggcaatactc	-	-
CY160964	Human H3N2 IAVs	Human	H3N2		2011	Peru	A/Peru/PER085/2011	agtgatgccccattccttgatcggcttcgccgagatcag<br>aggtccctaaggggaagaggcaatactc	-	-
CY161196	Human H3N2 IAVs	Human	H3N2		2011	Peru	A/Peru/PER115/2011	agtgatgccccattccttgatcggcttcgccgagatcag<br>aggtccctaaggggaagaggcaatactc	-	-
CY162172	Human H3N2 IAVs	Human	H3N2		2011	Peru	A/Peru/PER238/2011	agtgatgccccattccttgatcggcttcgccgagatcag<br>aggtccctaaggggaagaggcaatactc	-	-
CY162492	Human H3N2 IAVs	Human	H3N2		2011	Peru	A/Peru/PER281/2011	agtgatgccccattccttgatcggcttcgccgagatcag<br>aggtccctaaggggaagaggcaatactc	-	-
CY161556	Human H3N2 IAVs	Human	H3N2		2011	Peru	A/Peru/PER161/2011	agtgatgccccattccttgatcggcttcgccgagatcag<br>aggtccctaaggggaagaggcaatactc	-	-
CY162644	Human H3N2 IAVs	Human	H3N2		2011	Peru	A/Peru/PER302/2011	agtgatgccccattccttgatcggcttcgccgagatcag<br>aggtccctaaggggaagaggcaatactc	-	-
CY091577	Human H3N2 IAVs	Human	H3N2		2011	USA	A/Illinois/NHRC0002/2011	agtgatgccccattccttgatcggcttcgccgagatcag<br>aggtccctaaggggaagaggcaatactc	-	-
CY125721	Human H3N2 IAVs	Human	H3N2		2011	USA	A/Boston/DOA27/2011	agtgatgccccattccttgatcggcttcgccgagatcag<br>aggtccctaaggggaagaggcaatactc	-	-
CY181733	Human H3N2 IAVs	Human	H3N2		2011	Nicaragua	A/Nicaragua/AGA2_23/2011	agtgatgccccattccttgatcggcttcgccgagatcag<br>aggtccctaaggggaagaggcaatactc	-	-
CY181749	Human H3N2 IAVs	Human	H3N2		2011	Nicaragua	A/Nicaragua/AGA2_25/2011	agtgatgccccattccttgatcggcttcgccgagatcag<br>aggtccctaaggggaagaggcaatactc	-	-
CY181789	Human H3N2 IAVs	Human	H3N2		2011	Nicaragua	A/Nicaragua/AGA2_30/2011	agtgatgccccattccttgatcggcttcgccgagatcag<br>aggtccctaaggggaagaggcaatactc	-	-
CY182021	Human H3N2 IAVs	Human	H3N2		2011	Nicaragua	A/Nicaragua/AGA2_64/2011	agtgatgccccattccttgatcggcttcgccgagatcag<br>aggtccctaaggggaagaggcaatactc	-	-
CY181797	Human H3N2 IAVs	Human	H3N2		2011	Nicaragua	A/Nicaragua/AGA2_32/2011	agtgatgccccattccttgatcggcttcgccgagatcag<br>aggtccctaaggggaagaggcaatactc	-	-
CY181845	Human H3N2 IAVs	Human	H3N2		2011	Nicaragua	A/Nicaragua/AGA2_40/2011	agtgatgccccattccttgatcggcttcgccgagatcag<br>aggtccctaaggggaagaggcaatactc	-	-
CY111274	Human H3N2 IAVs	Human	H3N2		2011	USA	A/Boston/DOA32/2011	agtgatgccccattccttgatcggcttcgccgagatcag<br>aggtccctaaggggaagaggcaatactc	-	-
KJ855351	Human H3N2 IAVs	Human	H3N2		2011	Mexico	A/Mexico/VER59/2011	agtgatgccccattccttgatcggcttcgccgagatcag<br>aggtccctaaggggaagaggcaatactc	-	-
CY114425	Human H3N2 IAVs	Human	H3N2		2011	Netherlands	A/Netherlands/063/2011	agtgatgccccattccttgatcggcttcgccgagatcag<br>aggtccctaaggggaagaggcaatactc	-	-
CY160676	Human H3N2 IAVs	Human	H3N2		2011	Peru	A/Peru/PER049/2011	agtgatgccccattccttgatcggcttcgccgagatcag<br>aggtccctaaggggaagaggtaatactc	-	-
KC883072	Human H3N2 IAVs	Human	H3N2		2011	USA	A/Illinois/03/2011	agtgatgccccattccttgatcggcttcgccgagatcag<br>aggtctctaaggggaagaggcaatactc	+	+
KC882989	Human H3N2 IAVs	Human	H3N2		2011	USA	A/Illinois/04/2011	agtgatgccccattccttgatcggcttcgccgagatcag<br>aggtctctaaggggaagaggcaatactc	+	+
CY167736	Human H3N2 IAVs	Human	H3N2		2011	USA	A/Tennessee/F2084c83/2011	agtgatgccccattccttgatcggcttcgccgagatcag<br>aggtctctaaggggaagaggcaatactc	+	+

CY160724	Human H3N2 IAVs	Human	H3N2		2011	Peru	A/Peru/PER055/2011	agtgatgccccattccttgatcggcttcgccgagatcag aggtgcctaaggggaagaggcaatactc	+	+
CY162076	Human H3N2 IAVs	Human	H3N2		2011	Peru	A/Peru/PER226/2011	agtgatgccccattccttgatcggcttcgccgagatcag aggtgcctaaggggaagaggcaatactc	+	+
CY111550	Human H3N2 IAVs	Human	H3N2		2011	USA	A/Boston/DOA33/2011	agtgatgccccattccttgatcggcttcgtcgagatcag aggtccctaaggggaagaggcaatactc	+	+
KM070095	Human H3N2 IAVs	Human	H3N2		2011	Singapore	A/Singapore/H2011.907/2011	agtgatgccccattccttgatcggcttckccgagatcag aggtccctaaggggaagaggcaatactc	-	-
CY163308	Human H3N2 IAVs	Human	H3N2		2011	Peru	A/Peru/PER388/2011	agtgatgccccccttccttgatcggcttcgccgagatcag aggtccctaaggggaagaggcaatactc	-	-
JX913071	Human H3N2 IAVs	Human	H3N2		2012	Czech_Republic	A/Czech_Republic/114/2012	aatgatgccccattccttgatcggcttcgccgagatca gaggtccctaaggggaagaggcaatactc	-	-
CY148296	Human H3N2 IAVs	Human	H3N2		2012	USA	A/Boston/DOA2_095/2012	agcgatgccccattcctcgatcggcttcgccgagatca gaggtccctaaggggaagaggcaatactc	-	-
CY148304	Human H3N2 IAVs	Human	H3N2		2012	USA	A/Boston/DOA2_097/2012	agcgatgccccattcctcgatcggcttcgccgagatca gaggtccctaaggggaagaggcaatactc	-	-
CY148312	Human H3N2 IAVs	Human	H3N2		2012	USA	A/Boston/DOA2_098/2012	agcgatgccccattcctcgatcggcttcgccgagatca gaggtccctaaggggaagaggcaatactc	-	-
CY148400	Human H3N2 IAVs	Human	H3N2		2012	USA	A/Boston/DOA2_109/2012	agcgatgccccattcctcgatcggcttcgccgagatca gaggtccctaaggggaagaggcaatactc	-	-
CY148408	Human H3N2 IAVs	Human	H3N2		2012	USA	A/Boston/DOA2_110/2012	agcgatgccccattcctcgatcggcttcgccgagatca gaggtccctaaggggaagaggcaatactc	-	-
CY148432	Human H3N2 IAVs	Human	H3N2		2012	USA	A/Boston/DOA2_113/2012	agcgatgccccattcctcgatcggcttcgccgagatca gaggtccctaaggggaagaggcaatactc	-	-
CY148456	Human H3N2 IAVs	Human	H3N2		2012	USA	A/Boston/DOA2_116/2012	agcgatgccccattcctcgatcggcttcgccgagatca gaggtccctaaggggaagaggcaatactc	-	-
CY148544	Human H3N2 IAVs	Human	H3N2		2012	USA	A/Boston/DOA2_128/2012	agcgatgccccattcctcgatcggcttcgccgagatca gaggtccctaaggggaagaggcaatactc	-	-
CY148632	Human H3N2 IAVs	Human	H3N2		2012	USA	A/Boston/DOA2_140/2012	agcgatgccccattcctcgatcggcttcgccgagatca gaggtccctaaggggaagaggcaatactc	-	-
CY167891	Human H3N2 IAVs	Human	H3N2		2012	USA	A/Boston/YGA_00002/2012	agcgatgccccattcctcgatcggcttcgccgagatca gaggtccctaaggggaagaggcaatactc	-	-
CY168659	Human H3N2 IAVs	Human	H3N2		2012	USA	A/Boston/YGA_01018/2012	agcgatgccccattcctcgatcggcttcgccgagatca gaggtccctaaggggaagaggcaatactc	-	-
CY168939	Human H3N2 IAVs	Human	H3N2		2012	USA	A/Boston/YGA_01058/2012	agcgatgccccattcctcgatcggcttcgccgagatca gaggtccctaaggggaagaggcaatactc	-	-
CY167899	Human H3N2 IAVs	Human	H3N2		2012	USA	A/Boston/YGA_00003/2012	agcgatgccccattcctcgatcggcttcgccgagatca gaggtccctaaggggaagaggcaatactc	-	-
CY168475	Human H3N2 IAVs	Human	H3N2		2012	USA	A/Boston/YGA_00093/2012	agcgatgccccattcctcgatcggcttcgccgagatca gaggtccctaaggggaagaggcaatactc	-	-
CY148424	Human H3N2 IAVs	Human	H3N2		2012	USA	A/Boston/DOA2_112/2012	agcgatgccccattcctcgatcggcttcgccgagatca gaggtccctaaggggaagaggcaatactc	-	-
KM070111	Human H3N2 IAVs	Human	H3N2		2012	Singapore	A/Singapore/H2012.567/2012	agtgacgccccattccttgatcggcttcgccgagatca aaggtccctaaggggaagaggcaatactc	-	-
KJ943070	Human H3N2 IAVs	Human	H3N2		2012	Chile	A/Santiago/p19d0/2012	agtgataccccattccttgatcggcttcgccgagatca gaggtccctaaggggaagaggcaatactc	-	-
KJ942955	Human H3N2 IAVs	Human	H3N2		2012	Chile	A/Santiago/p19d2/2012	agtgataccccattccttgatcggcttcgccgagatca gaggtccctaaggggaagaggcaatactc	-	-
KJ942612	Human H3N2 IAVs	Human	H3N2		2012	USA	A/Hawaii/22/2012	agtgatgccccattcctcgatcggcttcgccgagatca gaggtccctaaggggaagaggcaatactc	-	-

CY168955	Human H3N2 IAVs	Human	H3N2		2012	USA	A/Boston/YGA_01060/2012	agtgatgccccattccttgaccggcttcgccgagatca gaggtccctaaggggaagaggcaatactc	-	-
KC892834	Human H3N2 IAVs	Human	H3N2		2012	USA	A/Nevada/12/2012	agtgatgccccattccttgataggcttcgccgagatca aaggtccctaaggggaagaggcaatactc	-	-
CY171059	Human H3N2 IAVs	Human	H3N2		2012	USA	A/Chicago/YGA_04002/2012	agtgatgccccattccttgatcggattcgccgagatca gaggtccctaaggggaagaggcaatactc	-	-
CY135016	Human H3N2 IAVs	Human	H3N2		2012	USA	A/Texas/JMM_39/2012	agtgatgccccattccttgatcggctacgccgagatca gaggtccctaaggggaagaggcaatactc	-	-
KF928643	Human H3N2 IAVs	Human	H3N2		2012	USA	A/Illinois/NHRC384214/2012	agtgatgccccattccttgatcggcttcgcagagatca gaggtccctaaggggaagaggcaatactc	+	+
CY114537	Human H3N2 IAVs	Human	H3N2		2012	Russia	A/Petrozavodsk/RII01/2012	agtgatgccccattccttgatcggcttcgccgagatca aaggtccctaaggggaagaggcaatactc	-	-
KC892826	Human H3N2 IAVs	Human	H3N2		2012	USA	A/New_Mexico/07/2012	agtgatgccccattccttgatcggcttcgccgagatca aaggtccctaaggggaagaggcaatactc	-	-
KJ855359	Human H3N2 IAVs	Human	H3N2		2012	Mexico	A/Mexico/VER58/2012	agtgatgccccattccttgatcggcttcgccgagatca aaggtccctaaggggaagaggcaatactc	-	-
CY182709	Human H3N2 IAVs	Human	H3N2		2012	USA	A/Houston/JMM_63/2012	agtgatgccccattccttgatcggcttcgccgagatca aaggtccctaaggggaagaggcgatactc	-	-
CY162756	Human H3N2 IAVs	Human	H3N2		2012	Peru	A/Peru/PER316/2012	agtgatgccccattccttgatcggcttcgccgagatca aaggtgcataaggggaagaggcaatactc	-	-
KJ943097	Human H3N2 IAVs	Human	H3N2		2012	Chile	A/Santiago/p38_2012d1/2012	agtgatgccccattccttgatcggcttcgccgagatcag aagtccctaaggggaagaggcaatactc	+	+
KJ943244	Human H3N2 IAVs	Human	H3N2		2012	Chile	A/Santiago/p38_2012d3/2012	agtgatgccccattccttgatcggcttcgccgagatcag aagtccctaaggggaagaggcaatactc	+	+
JX913063	Human H3N2 IAVs	Human	H3N2		2012	Czech_Republic	A/Czech_Republic/126/2012	agtgatgccccattccttgatcggcttcgccgagatcag agatccctaaggggaagaggcaatactc	-	*
CY161212	Human H3N2 IAVs	Human	H3N2		2012	Peru	A/Peru/PER117/2012	agtgatgccccattccttgatcggcttcgccgagatcag agggccctaaggggaagaggcaatactc	-	-
CY148744	Human H3N2 IAVs	Human	H3N2		2012	USA	A/Boston/DOA2_155/2012	agtgatgccccattccttgatcggcttcgccgagatcag aggtccctaaagggaagaggcaataccc	-	-
CY160860	Human H3N2 IAVs	Human	H3N2		2012	Peru	A/Peru/PER072/2012	agtgatgccccattccttgatcggcttcgccgagatcag aggtccctaaagggaagaggcaatactc	+	+
CY161940	Human H3N2 IAVs	Human	H3N2		2012	Peru	A/Peru/PER209/2012	agtgatgccccattccttgatcggcttcgccgagatcag aggtccctaaagggaagaggcaatactc	+	+
KM070110	Human H3N2 IAVs	Human	H3N2		2012	Singapore	A/Singapore/H2012.562a/2012	agtgatgccccattccttgatcggcttcgccgagatcag aggtccctaagaggaagaggcaatactc	+	+
KJ577161	Human H3N2 IAVs	Human	H3N2		2012	Thailand	A/Thailand/VIROAF2/2012	agtgatgccccattccttgatcggcttcgccgagatcag aggtccctaagaggaagaggcaatactc	+	+
KC892833	Human H3N2 IAVs	Human	H3N2		2012	USA	A/California/22/2012	agtgatgccccattccttgatcggcttcgccgagatcag aggtccctaagaggaagaggcaatactc	+	+
CY148480	Human H3N2 IAVs	Human	H3N2		2012	USA	A/Boston/DOA2_120/2012	agtgatgccccattccttgatcggcttcgccgagatcag aggtccctaaggagaagaggcaatactc	-	*
KM070107	Human H3N2 IAVs	Human	H3N2		2012	Singapore	A/Singapore/C2012.389/2012	agtgatgccccattccttgatcggcttcgccgagatcag aggtccctaaggggaagaggcaacactc	-	-
CY168675	Human H3N2 IAVs	Human	H3N2		2012	USA	A/Boston/YGA_01022/2012	agtgatgccccattccttgatcggcttcgccgagatcag aggtccctaaggggaagaggcaacactc	-	-
CY182285	Human H3N2 IAVs	Human	H3N2		2012	Nicaragua	A/Nicaragua/AGA2_104/2012	agtgatgccccattccttgatcggcttcgccgagatcag aggtccctaaggggaagaggcaataccc	-	-
CY181685	Human H3N2 IAVs	Human	H3N2		2012	Nicaragua	A/Nicaragua/AGA2_14/2012	agtgatgccccattccttgatcggcttcgccgagatcag aggtccctaaggggaagaggcaataccc	-	-

CY181717	Human H3N2 IAVs	Human	H3N2		2012	Nicaragua	A/Nicaragua/AGA2_20/2012	agtgatgccccattccttgatcggcttcgccgagatcag aggtccctaaggggaagaggcaataccc	-	-
CY182205	Human H3N2 IAVs	Human	H3N2		2012	Nicaragua	A/Nicaragua/AGA2_92/2012	agtgatgccccattccttgatcggcttcgccgagatcag aggtccctaaggggaagaggcaataccc	-	-
JX913023	Human H3N2 IAVs	Human	H3N2		2012	Czech_Republic	A/Czech_Republic/119/2012	agtgatgccccattccttgatcggcttcgccgagatcag aggtccctaaggggaagaggcaatactc	-	-
JX913031	Human H3N2 IAVs	Human	H3N2		2012	Czech_Republic	A/Czech_Republic/120/2012	agtgatgccccattccttgatcggcttcgccgagatcag aggtccctaaggggaagaggcaatactc	-	-
KJ942708	Human H3N2 IAVs	Human	H3N2		2012	USA	A/Ohio/2/2012	agtgatgccccattccttgatcggcttcgccgagatcag aggtccctaaggggaagaggcaatactc	-	-
CY160532	Human H3N2 IAVs	Human	H3N2		2012	Peru	A/Peru/PER031/2012	agtgatgccccattccttgatcggcttcgccgagatcag aggtccctaaggggaagaggcaatactc	-	-
CY160620	Human H3N2 IAVs	Human	H3N2		2012	Peru	A/Peru/PER042/2012	agtgatgccccattccttgatcggcttcgccgagatcag aggtccctaaggggaagaggcaatactc	-	-
CY161180	Human H3N2 IAVs	Human	H3N2		2012	Peru	A/Peru/PER113/2012	agtgatgccccattccttgatcggcttcgccgagatcag aggtccctaaggggaagaggcaatactc	-	-
CY161492	Human H3N2 IAVs	Human	H3N2		2012	Peru	A/Peru/PER153/2012	agtgatgccccattccttgatcggcttcgccgagatcag aggtccctaaggggaagaggcaatactc	-	-
CY162004	Human H3N2 IAVs	Human	H3N2		2012	Peru	A/Peru/PER217/2012	agtgatgccccattccttgatcggcttcgccgagatcag aggtccctaaggggaagaggcaatactc	-	-
CY162020	Human H3N2 IAVs	Human	H3N2		2012	Peru	A/Peru/PER219/2012	agtgatgccccattccttgatcggcttcgccgagatcag aggtccctaaggggaagaggcaatactc	-	-
CY162036	Human H3N2 IAVs	Human	H3N2		2012	Peru	A/Peru/PER221/2012	agtgatgccccattccttgatcggcttcgccgagatcag aggtccctaaggggaagaggcaatactc	-	-
CY162340	Human H3N2 IAVs	Human	H3N2		2012	Peru	A/Peru/PER262/2012	agtgatgccccattccttgatcggcttcgccgagatcag aggtccctaaggggaagaggcaatactc	-	-
CY162668	Human H3N2 IAVs	Human	H3N2		2012	Peru	A/Peru/PER305/2012	agtgatgccccattccttgatcggcttcgccgagatcag aggtccctaaggggaagaggcaatactc	-	-
CY162700	Human H3N2 IAVs	Human	H3N2		2012	Peru	A/Peru/PER309/2012	agtgatgccccattccttgatcggcttcgccgagatcag aggtccctaaggggaagaggcaatactc	-	-
CY162836	Human H3N2 IAVs	Human	H3N2		2012	Peru	A/Peru/PER326/2012	agtgatgccccattccttgatcggcttcgccgagatcag aggtccctaaggggaagaggcaatactc	-	-
CY163052	Human H3N2 IAVs	Human	H3N2		2012	Peru	A/Peru/PER354/2012	agtgatgccccattccttgatcggcttcgccgagatcag aggtccctaaggggaagaggcaatactc	-	-
CY163084	Human H3N2 IAVs	Human	H3N2		2012	Peru	A/Peru/PER358/2012	agtgatgccccattccttgatcggcttcgccgagatcag aggtccctaaggggaagaggcaatactc	-	-
CY163124	Human H3N2 IAVs	Human	H3N2		2012	Peru	A/Peru/PER363/2012	agtgatgccccattccttgatcggcttcgccgagatcag aggtccctaaggggaagaggcaatactc	-	-
CY114547	Human H3N2 IAVs	Human	H3N2		2012	Russia	A/Saint_Petersburg/RII02/2012	agtgatgccccattccttgatcggcttcgccgagatcag aggtccctaaggggaagaggcaatactc	-	-
CY114552	Human H3N2 IAVs	Human	H3N2		2012	Russia	A/Saint_Petersburg/RII03/2012	agtgatgccccattccttgatcggcttcgccgagatcag aggtccctaaggggaagaggcaatactc	-	-
KM070098	Human H3N2 IAVs	Human	H3N2		2012	Singapore	A/Singapore/C2012.011/2012	agtgatgccccattccttgatcggcttcgccgagatcag aggtccctaaggggaagaggcaatactc	-	-
KC892842	Human H3N2 IAVs	Human	H3N2		2012	USA	A/Michigan/03/2012	agtgatgccccattccttgatcggcttcgccgagatcag aggtccctaaggggaagaggcaatactc	-	-
CY162484	Human H3N2 IAVs	Human	H3N2		2012	Peru	A/Peru/PER280/2012	agtgatgccccattccttgatcggcttcgccgagatcag aggtccctaaggggaagaggcaatactc	-	-
CY163172	Human H3N2 IAVs	Human	H3N2		2012	Peru	A/Peru/PER371/2012	agtgatgccccattccttgatcggcttcgccgagatcag aggtccctaaggggaagaggcaatactc	-	-

CY162044	Human H3N2 IAVs	Human	H3N2		2012	Peru	A/Peru/PER222/2012	agtgatgccccattccttgatcggcttcgccgagatcag<br>aggtccctaaggggaagaggcaatactc	-	-
CY163092	Human H3N2 IAVs	Human	H3N2		2012	Peru	A/Peru/PER359/2012	agtgatgccccattccttgatcggcttcgccgagatcag<br>aggtccctaaggggaagaggcaatactc	-	-
CY160580	Human H3N2 IAVs	Human	H3N2		2012	Peru	A/Peru/PER037/2012	agtgatgccccattccttgatcggcttcgccgagatcag<br>aggtccctaaggggaagaggcaatactc	-	-
CY160788	Human H3N2 IAVs	Human	H3N2		2012	Peru	A/Peru/PER063/2012	agtgatgccccattccttgatcggcttcgccgagatcag<br>aggtccctaaggggaagaggcaatactc	-	-
CY161516	Human H3N2 IAVs	Human	H3N2		2012	Peru	A/Peru/PER156/2012	agtgatgccccattccttgatcggcttcgccgagatcag<br>aggtccctaaggggaagaggcaatactc	-	-
CY161604	Human H3N2 IAVs	Human	H3N2		2012	Peru	A/Peru/PER167/2012	agtgatgccccattccttgatcggcttcgccgagatcag<br>aggtccctaaggggaagaggcaatactc	-	-
KM070099	Human H3N2 IAVs	Human	H3N2		2012	Singapore	A/Singapore/S2012.014/2012	agtgatgccccattccttgatcggcttcgccgagatcag<br>aggtccctaaggggaagaggcaatactc	-	-
CY161668	Human H3N2 IAVs	Human	H3N2		2012	Peru	A/Peru/PER175/2012	agtgatgccccattccttgatcggcttcgccgagatcag<br>aggtccctaaggggaagaggcaatactc	-	-
CY148336	Human H3N2 IAVs	Human	H3N2		2012	USA	A/Boston/DOA2_101/2012	agtgatgccccattccttgatcggcttcgccgagatcag<br>aggtccctaaggggaagaggcaatactc	-	-
CY169011	Human H3N2 IAVs	Human	H3N2		2012	USA	A/Boston/YGA_01067/2012	agtgatgccccattccttgatcggcttcgccgagatcag<br>aggtccctaaggggaagaggcaatactc	-	-
CY169099	Human H3N2 IAVs	Human	H3N2		2012	USA	A/Boston/YGA_01079/2012	agtgatgccccattccttgatcggcttcgccgagatcag<br>aggtccctaaggggaagaggcaatactc	-	-
CY169315	Human H3N2 IAVs	Human	H3N2		2012	USA	A/Boston/YGA_01109/2012	agtgatgccccattccttgatcggcttcgccgagatcag<br>aggtccctaaggggaagaggcaatactc	-	-
JX913039	Human H3N2 IAVs	Human	H3N2		2012	Czech_Republic	A/Czech_Republic/121/2012	agtgatgccccattccttgatcggcttcgccgagatcag<br>aggtccctaaggggaagaggcaatactc	-	-
KM070115	Human H3N2 IAVs	Human	H3N2		2012	Singapore	A/Singapore/H2012.779/2012	agtgatgccccattccttgatcggcttcgccgagatcag<br>aggtccctaaggggaagaggcaatactc	-	-
JX913047	Human H3N2 IAVs	Human	H3N2		2012	Czech_Republic	A/Czech_Republic/76/2012	agtgatgccccattccttgatcggcttcgccgagatcag<br>aggtccctaaggggaagaggcaatactc	-	-
CY168987	Human H3N2 IAVs	Human	H3N2		2012	USA	A/Boston/YGA_01064/2012	agtgatgccccattccttgatcggcttcgccgagatcag<br>aggtccctaaggggaagaggcaatactc	-	-
CY169075	Human H3N2 IAVs	Human	H3N2		2012	USA	A/Boston/YGA_01076/2012	agtgatgccccattccttgatcggcttcgccgagatcag<br>aggtccctaaggggaagaggcaatactc	-	-
KM070112	Human H3N2 IAVs	Human	H3N2		2012	Singapore	A/Singapore/S2012.579b/2012	agtgatgccccattccttgatcggcttcgccgagatcag<br>aggtccctaaggggaagaggcaatactc	-	-
KM070104	Human H3N2 IAVs	Human	H3N2		2012	Singapore	A/Singapore/C2012.274/2012	agtgatgccccattccttgatcggcttcgccgagatcag<br>aggtccctaaggggaagaggcaatactc	-	-
KM070109	Human H3N2 IAVs	Human	H3N2		2012	Singapore	A/Singapore/H2012.490/2012	agtgatgccccattccttgatcggcttcgccgagatcag<br>aggtccctaaggggaagaggcaatactc	-	-
KM070108	Human H3N2 IAVs	Human	H3N2		2012	Singapore	A/Singapore/S2012.389/2012	agtgatgccccattccttgatcggcttcgccgagatcag<br>aggtccctaaggggaagaggcaatactc	-	-
CY114562	Human H3N2 IAVs	Human	H3N2		2012	Russia	A/Novosibirsk/RII09/2012	agtgatgccccattccttgatcggcttcgccgagatcag<br>aggtccctaaggggaagaggcaatactc	-	-
KJ942620	Human H3N2 IAVs	Human	H3N2		2012	USA	A/Texas/50/2012	agtgatgccccattccttgatcggcttcgccgagatcag<br>aggtccctaaggggaagaggcaatactc	-	-
KJ943755	Human H3N2 IAVs	Human	H3N2		2012	Chile	A/Santiago/p25d2/2012	agtgatgccccattccttgatcggcttcgccgagatcag<br>aggtccctaaggggaagaggcaatactc	-	-
KJ943697	Human H3N2 IAVs	Human	H3N2		2012	Chile	A/Santiago/p25d5/2012	agtgatgccccattccttgatcggcttcgccgagatcag<br>aggtccctaaggggaagaggcaatactc	-	-

KJ943676	Human H3N2 IAVs	Human	H3N2		2012	Chile	A/Santiago/p25d7/2012	agtgatgccccattccttgatcggcttcgccgagatcag aggtccctaaggggaagaggcaatactc	-	-
KJ943569	Human H3N2 IAVs	Human	H3N2		2012	Chile	A/Santiago/p2d2/2012	agtgatgccccattccttgatcggcttcgccgagatcag aggtccctaaggggaagaggcaatactc	-	-
KJ943577	Human H3N2 IAVs	Human	H3N2		2012	Chile	A/Santiago/p3d0/2012	agtgatgccccattccttgatcggcttcgccgagatcag aggtccctaaggggaagaggcaatactc	-	-
KJ943771	Human H3N2 IAVs	Human	H3N2		2012	Chile	A/Santiago/p2d1/2012	agtgatgccccattccttgatcggcttcgccgagatcag aggtccctaaggggaagaggcaatactc	-	-
KJ943662	Human H3N2 IAVs	Human	H3N2		2012	Chile	A/Santiago/p3d2/2012	agtgatgccccattccttgatcggcttcgccgagatcag aggtccctaaggggaagaggcaatactc	-	-
KJ943748	Human H3N2 IAVs	Human	N2		2012	Chile	A/Santiago/p2d7/2012	agtgatgccccattccttgatcggcttcgccgagatcag aggtccctaaggggaagaggcaatactc	-	-
KJ943724	Human H3N2 IAVs	Human	H3N2		2012	Chile	A/Santiago/p18d0/2012	agtgatgccccattccttgatcggcttcgccgagatcag aggtccctaaggggaagaggcaatactc	-	-
KJ943630	Human H3N2 IAVs	Human	H3N2		2012	Chile	A/Santiago/p18d00/2012	agtgatgccccattccttgatcggcttcgccgagatcag aggtccctaaggggaagaggcaatactc	-	-
KM070100	Human H3N2 IAVs	Human	H3N2		2012	Singapore	A/Singapore/S2012.181/2012	agtgatgccccattccttgatcggcttcgccgagatcag aggtccctaaggggaagaggcaatactc	-	-
CY114542	Human H3N2 IAVs	Human	H3N2		2012	Russia	A/Novosibirsk/RII08/2012	agtgatgccccattccttgatcggcttcgccgagatcag aggtccctaaggggaagaggcaatactc	-	-
CY114567	Human H3N2 IAVs	Human	H3N2		2012	Russia	A/Saint_Petersburg/RII01/2012	agtgatgccccattccttgatcggcttcgccgagatcag aggtccctaaggggaagaggcaatactc	-	-
CY162636	Human H3N2 IAVs	Human	H3N2		2012	Peru	A/Peru/PER301/2012	agtgatgccccattccttgatcggcttcgccgagatcag aggtccctaaggggaagaggcaatactc	-	-
KC892448	Human H3N2 IAVs	Human	H3N2		2012	USA	A/Washington/16/2012	agtgatgccccattccttgatcggcttcgccgagatcag aggtccctaaggggaagaggcaatactc	-	-
CY162732	Human H3N2 IAVs	Human	H3N2		2012	Peru	A/Peru/PER313/2012	agtgatgccccattccttgatcggcttcgccgagatcag aggtccctaaggggaagaggcaatactc	-	-
CY182765	Human H3N2 IAVs	Human	H3N2		2012	USA	A/Houston/JMM_70/2012	agtgatgccccattccttgatcggcttcgccgagatcag aggtccctaaggggaagaggcaatactc	-	-
CY134952	Human H3N2 IAVs	Human	H3N2		2012	USA	A/Texas/JMM_31/2012	agtgatgccccattccttgatcggcttcgccgagatcag aggtccctaaggggaagaggcaatactc	-	-
CY135024	Human H3N2 IAVs	Human	H3N2		2012	USA	A/Texas/JMM_40/2012	agtgatgccccattccttgatcggcttcgccgagatcag aggtccctaaggggaagaggcaatactc	-	-
CY135032	Human H3N2 IAVs	Human	H3N2		2012	USA	A/Texas/JMM_41/2012	agtgatgccccattccttgatcggcttcgccgagatcag aggtccctaaggggaagaggcaatactc	-	-
CY182773	Human H3N2 IAVs	Human	H3N2		2012	USA	A/Houston/JMM_71/2012	agtgatgccccattccttgatcggcttcgccgagatcag aggtccctaaggggaagaggcaatactc	-	-
CY134800	Human H3N2 IAVs	Human	H3N2		2012	USA	A/Texas/JMM_11/2012	agtgatgccccattccttgatcggcttcgccgagatcag aggtccctaaggggaagaggcaatactc	-	-
CY134912	Human H3N2 IAVs	Human	H3N2		2012	USA	A/Texas/JMM_25/2012	agtgatgccccattccttgatcggcttcgccgagatcag aggtccctaaggggaagaggcaatactc	-	-
CY134808	Human H3N2 IAVs	Human	H3N2		2012	USA	A/Texas/JMM_12/2012	agtgatgccccattccttgatcggcttcgccgagatcag aggtccctaaggggaagaggcaatactc	-	-
CY134824	Human H3N2 IAVs	Human	H3N2		2012	USA	A/Texas/JMM_14/2012	agtgatgccccattccttgatcggcttcgccgagatcag aggtccctaaggggaagaggcaatactc	-	-
CY135008	Human H3N2 IAVs	Human	H3N2		2012	USA	A/Texas/JMM_38/2012	agtgatgccccattccttgatcggcttcgccgagatcag aggtccctaaggggaagaggcaatactc	-	-
CY135080	Human H3N2 IAVs	Human	H3N2		2012	USA	A/Texas/JMM_48/2012	agtgatgccccattccttgatcggcttcgccgagatcag aggtccctaaggggaagaggcaatactc	-	-

CY135168	Human H3N2 IAVs	Human	H3N2		2012	USA	A/Texas/JMM_60/2012	agtgatgccccattccttgatcggcttcgccgagatcag aggtccctaaggggaagaggcaatactc	-	-
CY134928	Human H3N2 IAVs	Human	H3N2		2012	USA	A/Texas/JMM_27/2012	agtgatgccccattccttgatcggcttcgccgagatcag aggtccctaaggggaagaggcaatactc	-	-
CY182901	Human H3N2 IAVs	Human	H3N2		2012	USA	A/Houston/JMM_88/2012	agtgatgccccattccttgatcggcttcgccgagatcag aggtccctaaggggaagaggcaatactc	-	-
CY134992	Human H3N2 IAVs	Human	H3N2		2012	USA	A/Texas/JMM_36/2012	agtgatgccccattccttgatcggcttcgccgagatcag aggtccctaaggggaagaggcaatactc	-	-
KM070101	Human H3N2 IAVs	Human	H3N2		2012	Singapore	A/Singapore/C2012.197/2012	agtgatgccccattccttgatcggcttcgccgagatcag aggtccctaaggggaagaggcaatactc	-	-
KM070102	Human H3N2 IAVs	Human	H3N2		2012	Singapore	A/Singapore/C2012.208/2012	agtgatgccccattccttgatcggcttcgccgagatcag aggtccctaaggggaagaggcaatactc	-	-
CY148328	Human H3N2 IAVs	Human	H3N2		2012	USA	A/Boston/DOA2_100/2012	agtgatgccccattccttgatcggcttcgccgagatcag aggtccctaaggggaagaggcaatactc	-	-
CY148376	Human H3N2 IAVs	Human	H3N2		2012	USA	A/Boston/DOA2_106/2012	agtgatgccccattccttgatcggcttcgccgagatcag aggtccctaaggggaagaggcaatactc	-	-
CY148384	Human H3N2 IAVs	Human	H3N2		2012	USA	A/Boston/DOA2_107/2012	agtgatgccccattccttgatcggcttcgccgagatcag aggtccctaaggggaagaggcaatactc	-	-
CY148392	Human H3N2 IAVs	Human	H3N2		2012	USA	A/Boston/DOA2_108/2012	agtgatgccccattccttgatcggcttcgccgagatcag aggtccctaaggggaagaggcaatactc	-	-
CY148488	Human H3N2 IAVs	Human	H3N2		2012	USA	A/Boston/DOA2_121/2012	agtgatgccccattccttgatcggcttcgccgagatcag aggtccctaaggggaagaggcaatactc	-	-
CY148512	Human H3N2 IAVs	Human	H3N2		2012	USA	A/Boston/DOA2_124/2012	agtgatgccccattccttgatcggcttcgccgagatcag aggtccctaaggggaagaggcaatactc	-	-
CY148536	Human H3N2 IAVs	Human	H3N2		2012	USA	A/Boston/DOA2_127/2012	agtgatgccccattccttgatcggcttcgccgagatcag aggtccctaaggggaagaggcaatactc	-	-
CY148552	Human H3N2 IAVs	Human	H3N2		2012	USA	A/Boston/DOA2_129/2012	agtgatgccccattccttgatcggcttcgccgagatcag aggtccctaaggggaagaggcaatactc	-	-
CY148560	Human H3N2 IAVs	Human	H3N2		2012	USA	A/Boston/DOA2_130/2012	agtgatgccccattccttgatcggcttcgccgagatcag aggtccctaaggggaagaggcaatactc	-	-
CY148568	Human H3N2 IAVs	Human	H3N2		2012	USA	A/Boston/DOA2_131/2012	agtgatgccccattccttgatcggcttcgccgagatcag aggtccctaaggggaagaggcaatactc	-	-
CY148584	Human H3N2 IAVs	Human	H3N2		2012	USA	A/Boston/DOA2_133/2012	agtgatgccccattccttgatcggcttcgccgagatcag aggtccctaaggggaagaggcaatactc	-	-
CY148648	Human H3N2 IAVs	Human	H3N2		2012	USA	A/Boston/DOA2_142/2012	agtgatgccccattccttgatcggcttcgccgagatcag aggtccctaaggggaagaggcaatactc	-	-
CY148848	Human H3N2 IAVs	Human	H3N2		2012	USA	A/Boston/DOA2_171/2012	agtgatgccccattccttgatcggcttcgccgagatcag aggtccctaaggggaagaggcaatactc	-	-
CY148286	Human H3N2 IAVs	Human	N2		2012	USA	A/Boston/DOA2_211/2012	agtgatgccccattccttgatcggcttcgccgagatcag aggtccctaaggggaagaggcaatactc	-	-
CY149160	Human H3N2 IAVs	Human	H3N2		2012	USA	A/Boston/DOA2_222/2012	agtgatgccccattccttgatcggcttcgccgagatcag aggtccctaaggggaagaggcaatactc	-	-
CY167923	Human H3N2 IAVs	Human	H3N2		2012	USA	A/Boston/YGA_00007/2012	agtgatgccccattccttgatcggcttcgccgagatcag aggtccctaaggggaagaggcaatactc	-	-
CY168443	Human H3N2 IAVs	Human	H3N2		2012	USA	A/Boston/YGA_00089/2012	agtgatgccccattccttgatcggcttcgccgagatcag aggtccctaaggggaagaggcaatactc	-	-
CY168451	Human H3N2 IAVs	Human	H3N2		2012	USA	A/Boston/YGA_00090/2012	agtgatgccccattccttgatcggcttcgccgagatcag aggtccctaaggggaagaggcaatactc	-	-
CY168571	Human H3N2 IAVs	Human	H3N2		2012	USA	A/Boston/YGA_01006/2012	agtgatgccccattccttgatcggcttcgccgagatcag aggtccctaaggggaagaggcaatactc	-	-

CY168611	Human H3N2 IAVs	Human	H3N2		2012	USA	A/Boston/YGA_01012/2012	agtgatgccccattccttgatcggcttcgccgagatcag aggtccctaaggggaagaggcaatactc	-	-
CY168627	Human H3N2 IAVs	Human	H3N2		2012	USA	A/Boston/YGA_01014/2012	agtgatgccccattccttgatcggcttcgccgagatcag aggtccctaaggggaagaggcaatactc	-	-
CY168683	Human H3N2 IAVs	Human	H3N2		2012	USA	A/Boston/YGA_01023/2012	agtgatgccccattccttgatcggcttcgccgagatcag aggtccctaaggggaagaggcaatactc	-	-
CY168699	Human H3N2 IAVs	Human	H3N2		2012	USA	A/Boston/YGA_01025/2012	agtgatgccccattccttgatcggcttcgccgagatcag aggtccctaaggggaagaggcaatactc	-	-
CY168715	Human H3N2 IAVs	Human	H3N2		2012	USA	A/Boston/YGA_01027/2012	agtgatgccccattccttgatcggcttcgccgagatcag aggtccctaaggggaagaggcaatactc	-	-
CY168779	Human H3N2 IAVs	Human	H3N2		2012	USA	A/Boston/YGA_01037/2012	agtgatgccccattccttgatcggcttcgccgagatcag aggtccctaaggggaagaggcaatactc	-	-
CY168803	Human H3N2 IAVs	Human	H3N2		2012	USA	A/Boston/YGA_01040/2012	agtgatgccccattccttgatcggcttcgccgagatcag aggtccctaaggggaagaggcaatactc	-	-
CY168827	Human H3N2 IAVs	Human	H3N2		2012	USA	A/Boston/YGA_01043/2012	agtgatgccccattccttgatcggcttcgccgagatcag aggtccctaaggggaagaggcaatactc	-	-
CY168899	Human H3N2 IAVs	Human	H3N2		2012	USA	A/Boston/YGA_01052/2012	agtgatgccccattccttgatcggcttcgccgagatcag aggtccctaaggggaagaggcaatactc	-	-
CY168971	Human H3N2 IAVs	Human	H3N2		2012	USA	A/Boston/YGA_01062/2012	agtgatgccccattccttgatcggcttcgccgagatcag aggtccctaaggggaagaggcaatactc	-	-
CY169171	Human H3N2 IAVs	Human	H3N2		2012	USA	A/Boston/YGA_01090/2012	agtgatgccccattccttgatcggcttcgccgagatcag aggtccctaaggggaagaggcaatactc	-	-
CY169259	Human H3N2 IAVs	Human	H3N2		2012	USA	A/Boston/YGA_01102/2012	agtgatgccccattccttgatcggcttcgccgagatcag aggtccctaaggggaagaggcaatactc	-	-
CY169275	Human H3N2 IAVs	Human	H3N2		2012	USA	A/Boston/YGA_01104/2012	agtgatgccccattccttgatcggcttcgccgagatcag aggtccctaaggggaagaggcaatactc	-	-
CY169291	Human H3N2 IAVs	Human	H3N2		2012	USA	A/Boston/YGA_01106/2012	agtgatgccccattccttgatcggcttcgccgagatcag aggtccctaaggggaagaggcaatactc	-	-
CY169299	Human H3N2 IAVs	Human	H3N2		2012	USA	A/Boston/YGA_01107/2012	agtgatgccccattccttgatcggcttcgccgagatcag aggtccctaaggggaagaggcaatactc	-	-
CY169323	Human H3N2 IAVs	Human	H3N2		2012	USA	A/Boston/YGA_01110/2012	agtgatgccccattccttgatcggcttcgccgagatcag aggtccctaaggggaagaggcaatactc	-	-
CY170107	Human H3N2 IAVs	Human	H3N2		2012	USA	A/Boston/YGA_02001/2012	agtgatgccccattccttgatcggcttcgccgagatcag aggtccctaaggggaagaggcaatactc	-	-
CY170131	Human H3N2 IAVs	Human	H3N2		2012	USA	A/Boston/YGA_02004/2012	agtgatgccccattccttgatcggcttcgccgagatcag aggtccctaaggggaagaggcaatactc	-	-
CY170179	Human H3N2 IAVs	Human	H3N2		2012	USA	A/Boston/YGA_02012/2012	agtgatgccccattccttgatcggcttcgccgagatcag aggtccctaaggggaagaggcaatactc	-	-
KF928621	Human H3N2 IAVs	Human	H3N2		2012	USA	A/California/NHRC382595/2012	agtgatgccccattccttgatcggcttcgccgagatcag aggtccctaaggggaagaggcaatactc	-	-
CY134904	Human H3N2 IAVs	Human	H3N2		2012	USA	A/Texas/JMM_24/2012	agtgatgccccattccttgatcggcttcgccgagatcag aggtccctaaggggaagaggcaatactc	-	-
CY168547	Human H3N2 IAVs	Human	H3N2		2012	USA	A/Boston/YGA_01003/2012	agtgatgccccattccttgatcggcttcgccgagatcag aggtccctaaggggaagaggcaatactc	-	-
CY168619	Human H3N2 IAVs	Human	H3N2		2012	USA	A/Boston/YGA_01013/2012	agtgatgccccattccttgatcggcttcgccgagatcag aggtccctaaggggaagaggcaatactc	-	-
CY168907	Human H3N2 IAVs	Human	H3N2		2012	USA	A/Boston/YGA_01053/2012	agtgatgccccattccttgatcggcttcgccgagatcag aggtccctaaggggaagaggcaatactc	-	-
CY148416	Human H3N2 IAVs	Human	H3N2		2012	USA	A/Boston/DOA2_111/2012	agtgatgccccattccttgatcggcttcgccgagatcag aggtccctaaggggaagaggcaatactc	-	-

CY168739	Human H3N2 IAVs	Human	H3N2		2012	USA	A/Boston/YGA_01030/2012	agtgatgccccattccttgatcggcttcgccgagatcag aggtccctaaggggaagaggcaatactc	-	-
CY169211	Human H3N2 IAVs	Human	H3N2		2012	USA	A/Boston/YGA_01096/2012	agtgatgccccattccttgatcggcttcgccgagatcag aggtccctaaggggaagaggcaatactc	-	-
CY168963	Human H3N2 IAVs	Human	H3N2		2012	USA	A/Boston/YGA_01061/2012	agtgatgccccattccttgatcggcttcgccgagatcag aggtccctaaggggaagaggcaatactc	-	-
CY134880	Human H3N2 IAVs	Human	H3N2		2012	USA	A/Texas/JMM_21/2012	agtgatgccccattccttgatcggcttcgccgagatcag aggtccctaaggggaagaggcaatactc	-	-
CY170123	Human H3N2 IAVs	Human	H3N2		2012	USA	A/Boston/YGA_02003/2012	agtgatgccccattccttgatcggcttcgccgagatcag aggtccctaaggggaagaggcaatactc	-	-
CY170147	Human H3N2 IAVs	Human	H3N2		2012	USA	A/Boston/YGA_02006/2012	agtgatgccccattccttgatcggcttcgccgagatcag aggtccctaaggggaagaggcaatactc	-	-
CY170155	Human H3N2 IAVs	Human	H3N2		2012	USA	A/Boston/YGA_02008/2012	agtgatgccccattccttgatcggcttcgccgagatcag aggtccctaaggggaagaggcaatactc	-	-
KM070113	Human H3N2 IAVs	Human	H3N2		2012	Singapore	A/Singapore/C2012.669/2012	agtgatgccccattccttgatcggcttcgccgagatcag aggtccctaaggggaagaggcaatactc	-	-
CY148344	Human H3N2 IAVs	Human	H3N2		2012	USA	A/Boston/DOA2_102/2012	agtgatgccccattccttgatcggcttcgccgagatcag aggtccctaaggggaagaggcaatactc	-	-
CY148496	Human H3N2 IAVs	Human	H3N2		2012	USA	A/Boston/DOA2_122/2012	agtgatgccccattccttgatcggcttcgccgagatcag aggtccctaaggggaagaggcaatactc	-	-
CY148504	Human H3N2 IAVs	Human	H3N2		2012	USA	A/Boston/DOA2_123/2012	agtgatgccccattccttgatcggcttcgccgagatcag aggtccctaaggggaagaggcaatactc	-	-
CY148520	Human H3N2 IAVs	Human	H3N2		2012	USA	A/Boston/DOA2_125/2012	agtgatgccccattccttgatcggcttcgccgagatcag aggtccctaaggggaagaggcaatactc	-	-
CY148792	Human H3N2 IAVs	Human	H3N2		2012	USA	A/Boston/DOA2_162/2012	agtgatgccccattccttgatcggcttcgccgagatcag aggtccctaaggggaagaggcaatactc	-	-
CY149056	Human H3N2 IAVs	Human	H3N2		2012	USA	A/Boston/DOA2_203/2012	agtgatgccccattccttgatcggcttcgccgagatcag aggtccctaaggggaagaggcaatactc	-	-
CY168643	Human H3N2 IAVs	Human	H3N2		2012	USA	A/Boston/YGA_01016/2012	agtgatgccccattccttgatcggcttcgccgagatcag aggtccctaaggggaagaggcaatactc	-	-
CY169003	Human H3N2 IAVs	Human	H3N2		2012	USA	A/Boston/YGA_01066/2012	agtgatgccccattccttgatcggcttcgccgagatcag aggtccctaaggggaagaggcaatactc	-	-
CY169091	Human H3N2 IAVs	Human	H3N2		2012	USA	A/Boston/YGA_01078/2012	agtgatgccccattccttgatcggcttcgccgagatcag aggtccctaaggggaagaggcaatactc	-	-
CY169243	Human H3N2 IAVs	Human	H3N2		2012	USA	A/Boston/YGA_01100/2012	agtgatgccccattccttgatcggcttcgccgagatcag aggtccctaaggggaagaggcaatactc	-	-
CY169219	Human H3N2 IAVs	Human	H3N2		2012	USA	A/Boston/YGA_01097/2012	agtgatgccccattccttgatcggcttcgccgagatcag aggtccctaaggggaagaggcaatactc	-	-
CY148528	Human H3N2 IAVs	Human	H3N2		2012	USA	A/Boston/DOA2_126/2012	agtgatgccccattccttgatcggcttcgccgagatcag aggtccctaaggggaagaggcaatactc	-	-
CY168731	Human H3N2 IAVs	Human	H3N2		2012	USA	A/Boston/YGA_01029/2012	agtgatgccccattccttgatcggcttcgccgagatcag aggtccctaaggggaagaggcaatactc	-	-
CY148616	Human H3N2 IAVs	Human	H3N2		2012	USA	A/Boston/DOA2_137/2012	agtgatgccccattccttgatcggcttcgccgagatcag aggtccctaaggggaagaggcaatactc	-	-
CY149080	Human H3N2 IAVs	Human	H3N2		2012	USA	A/Boston/DOA2_209/2012	agtgatgccccattccttgatcggcttcgccgagatcag aggtccctaaggggaagaggcaatactc	-	-
CY148472	Human H3N2 IAVs	Human	H3N2		2012	USA	A/Boston/DOA2_119/2012	agtgatgccccattccttgatcggcttcgccgagatcag aggtccctaaggggaagaggcaatactc	-	-
CY169059	Human H3N2 IAVs	Human	H3N2		2012	USA	A/Boston/YGA_01074/2012	agtgatgccccattccttgatcggcttcgccgagatcag aggtccctaaggggaagaggcaatactc	-	-

CY168635	Human H3N2 IAVs	Human	H3N2		2012	USA	A/Boston/YGA_01015/2012	agtgatgccccattccttgatcggcttcgccgagatcag<br>aggtccctaaggggaagaggcaatactc	-	-
CY148448	Human H3N2 IAVs	Human	H3N2		2012	USA	A/Boston/DOA2_115/2012	agtgatgccccattccttgatcggcttcgccgagatcag<br>aggtccctaaggggaagaggcaatactc	-	-
CY148832	Human H3N2 IAVs	Human	H3N2		2012	USA	A/Boston/DOA2_169/2012	agtgatgccccattccttgatcggcttcgccgagatcag<br>aggtccctaaggggaagaggcaatactc	-	-
CY167931	Human H3N2 IAVs	Human	H3N2		2012	USA	A/Boston/YGA_00008/2012	agtgatgccccattccttgatcggcttcgccgagatcag<br>aggtccctaaggggaagaggcaatactc	-	-
CY167971	Human H3N2 IAVs	Human	H3N2		2012	USA	A/Boston/YGA_00014/2012	agtgatgccccattccttgatcggcttcgccgagatcag<br>aggtccctaaggggaagaggcaatactc	-	-
CY168003	Human H3N2 IAVs	Human	H3N2		2012	USA	A/Boston/YGA_00019/2012	agtgatgccccattccttgatcggcttcgccgagatcag<br>aggtccctaaggggaagaggcaatactc	-	-
CY168531	Human H3N2 IAVs	Human	H3N2		2012	USA	A/Boston/YGA_01001/2012	agtgatgccccattccttgatcggcttcgccgagatcag<br>aggtccctaaggggaagaggcaatactc	-	-
CY168563	Human H3N2 IAVs	Human	H3N2		2012	USA	A/Boston/YGA_01005/2012	agtgatgccccattccttgatcggcttcgccgagatcag<br>aggtccctaaggggaagaggcaatactc	-	-
CY168723	Human H3N2 IAVs	Human	H3N2		2012	USA	A/Boston/YGA_01028/2012	agtgatgccccattccttgatcggcttcgccgagatcag<br>aggtccctaaggggaagaggcaatactc	-	-
CY168771	Human H3N2 IAVs	Human	H3N2		2012	USA	A/Boston/YGA_01036/2012	agtgatgccccattccttgatcggcttcgccgagatcag<br>aggtccctaaggggaagaggcaatactc	-	-
CY168795	Human H3N2 IAVs	Human	H3N2		2012	USA	A/Boston/YGA_01039/2012	agtgatgccccattccttgatcggcttcgccgagatcag<br>aggtccctaaggggaagaggcaatactc	-	-
CY168835	Human H3N2 IAVs	Human	H3N2		2012	USA	A/Boston/YGA_01044/2012	agtgatgccccattccttgatcggcttcgccgagatcag<br>aggtccctaaggggaagaggcaatactc	-	-
CY168851	Human H3N2 IAVs	Human	H3N2		2012	USA	A/Boston/YGA_01046/2012	agtgatgccccattccttgatcggcttcgccgagatcag<br>aggtccctaaggggaagaggcaatactc	-	-
CY168979	Human H3N2 IAVs	Human	H3N2		2012	USA	A/Boston/YGA_01063/2012	agtgatgccccattccttgatcggcttcgccgagatcag<br>aggtccctaaggggaagaggcaatactc	-	-
CY169019	Human H3N2 IAVs	Human	H3N2		2012	USA	A/Boston/YGA_01068/2012	agtgatgccccattccttgatcggcttcgccgagatcag<br>aggtccctaaggggaagaggcaatactc	-	-
CY169051	Human H3N2 IAVs	Human	H3N2		2012	USA	A/Boston/YGA_01073/2012	agtgatgccccattccttgatcggcttcgccgagatcag<br>aggtccctaaggggaagaggcaatactc	-	-
CY169067	Human H3N2 IAVs	Human	H3N2		2012	USA	A/Boston/YGA_01075/2012	agtgatgccccattccttgatcggcttcgccgagatcag<br>aggtccctaaggggaagaggcaatactc	-	-
CY169131	Human H3N2 IAVs	Human	H3N2		2012	USA	A/Boston/YGA_01084/2012	agtgatgccccattccttgatcggcttcgccgagatcag<br>aggtccctaaggggaagaggcaatactc	-	-
CY169139	Human H3N2 IAVs	Human	H3N2		2012	USA	A/Boston/YGA_01085/2012	agtgatgccccattccttgatcggcttcgccgagatcag<br>aggtccctaaggggaagaggcaatactc	-	-
CY169179	Human H3N2 IAVs	Human	H3N2		2012	USA	A/Boston/YGA_01091/2012	agtgatgccccattccttgatcggcttcgccgagatcag<br>aggtccctaaggggaagaggcaatactc	-	-
CY169187	Human H3N2 IAVs	Human	H3N2		2012	USA	A/Boston/YGA_01092/2012	agtgatgccccattccttgatcggcttcgccgagatcag<br>aggtccctaaggggaagaggcaatactc	-	-
CY169235	Human H3N2 IAVs	Human	H3N2		2012	USA	A/Boston/YGA_01099/2012	agtgatgccccattccttgatcggcttcgccgagatcag<br>aggtccctaaggggaagaggcaatactc	-	-
CY169307	Human H3N2 IAVs	Human	H3N2		2012	USA	A/Boston/YGA_01108/2012	agtgatgccccattccttgatcggcttcgccgagatcag<br>aggtccctaaggggaagaggcaatactc	-	-
CY170163	Human H3N2 IAVs	Human	H3N2		2012	USA	A/Boston/YGA_02009/2012	agtgatgccccattccttgatcggcttcgccgagatcag<br>aggtccctaaggggaagaggcaatactc	-	-
CY170171	Human H3N2 IAVs	Human	H3N2		2012	USA	A/Boston/YGA_02010/2012	agtgatgccccattccttgatcggcttcgccgagatcag<br>aggtccctaaggggaagaggcaatactc	-	-

CY171075	Human H3N2 IAVs	Human	H3N2		2012	USA	A/Chicago/YGA_04007/2012	agtgatgccccattccttgatcggcttcgccgagatcag<br>aggtccctaaggggaagaggcaatactc	-	-
CY171083	Human H3N2 IAVs	Human	H3N2		2012	USA	A/Chicago/YGA_04008/2012	agtgatgccccattccttgatcggcttcgccgagatcag<br>aggtccctaaggggaagaggcaatactc	-	-
CY171091	Human H3N2 IAVs	Human	H3N2		2012	USA	A/Chicago/YGA_04009/2012	agtgatgccccattccttgatcggcttcgccgagatcag<br>aggtccctaaggggaagaggcaatactc	-	-
CY171123	Human H3N2 IAVs	Human	H3N2		2012	USA	A/Chicago/YGA_04013/2012	agtgatgccccattccttgatcggcttcgccgagatcag<br>aggtccctaaggggaagaggcaatactc	-	-
CY171171	Human H3N2 IAVs	Human	H3N2		2012	USA	A/Chicago/YGA_04020/2012	agtgatgccccattccttgatcggcttcgccgagatcag<br>aggtccctaaggggaagaggcaatactc	-	-
CY171179	Human H3N2 IAVs	Human	H3N2		2012	USA	A/Chicago/YGA_04035/2012	agtgatgccccattccttgatcggcttcgccgagatcag<br>aggtccctaaggggaagaggcaatactc	-	-
CY171203	Human H3N2 IAVs	Human	H3N2		2012	USA	A/Chicago/YGA_04038/2012	agtgatgccccattccttgatcggcttcgccgagatcag<br>aggtccctaaggggaagaggcaatactc	-	-
CY171243	Human H3N2 IAVs	Human	H3N2		2012	USA	A/Chicago/YGA_04053/2012	agtgatgccccattccttgatcggcttcgccgagatcag<br>aggtccctaaggggaagaggcaatactc	-	-
CY171331	Human H3N2 IAVs	Human	H3N2		2012	USA	A/Chicago/YGA_04089/2012	agtgatgccccattccttgatcggcttcgccgagatcag<br>aggtccctaaggggaagaggcaatactc	-	-
CY171339	Human H3N2 IAVs	Human	H3N2		2012	USA	A/Chicago/YGA_04090/2012	agtgatgccccattccttgatcggcttcgccgagatcag<br>aggtccctaaggggaagaggcaatactc	-	-
CY171347	Human H3N2 IAVs	Human	H3N2		2012	USA	A/Chicago/YGA_04092/2012	agtgatgccccattccttgatcggcttcgccgagatcag<br>aggtccctaaggggaagaggcaatactc	-	-
CY171395	Human H3N2 IAVs	Human	H3N2		2012	USA	A/Chicago/YGA_04099/2012	agtgatgccccattccttgatcggcttcgccgagatcag<br>aggtccctaaggggaagaggcaatactc	-	-
CY171403	Human H3N2 IAVs	Human	H3N2		2012	USA	A/Chicago/YGA_04102/2012	agtgatgccccattccttgatcggcttcgccgagatcag<br>aggtccctaaggggaagaggcaatactc	-	-
CY171411	Human H3N2 IAVs	Human	H3N2		2012	USA	A/Chicago/YGA_04103/2012	agtgatgccccattccttgatcggcttcgccgagatcag<br>aggtccctaaggggaagaggcaatactc	-	-
CY171419	Human H3N2 IAVs	Human	H3N2		2012	USA	A/Chicago/YGA_04104/2012	agtgatgccccattccttgatcggcttcgccgagatcag<br>aggtccctaaggggaagaggcaatactc	-	-
CY171539	Human H3N2 IAVs	Human	H3N2		2012	USA	A/Chicago/YGA_04122/2012	agtgatgccccattccttgatcggcttcgccgagatcag<br>aggtccctaaggggaagaggcaatactc	-	-
CY182701	Human H3N2 IAVs	Human	H3N2		2012	USA	A/Houston/JMM_62/2012	agtgatgccccattccttgatcggcttcgccgagatcag<br>aggtccctaaggggaagaggcaatactc	-	-
CY182949	Human H3N2 IAVs	Human	H3N2		2012	USA	A/Houston/JMM_95/2012	agtgatgccccattccttgatcggcttcgccgagatcag<br>aggtccctaaggggaagaggcaatactc	-	-
CY182965	Human H3N2 IAVs	Human	H3N2		2012	USA	A/Houston/JMM_97/2012	agtgatgccccattccttgatcggcttcgccgagatcag<br>aggtccctaaggggaagaggcaatactc	-	-
KF928625	Human H3N2 IAVs	Human	H3N2		2012	USA	A/Oklahoma/NHRC375600/2012	agtgatgccccattccttgatcggcttcgccgagatcag<br>aggtccctaaggggaagaggcaatactc	-	-
CY170907	Human H3N2 IAVs	Human	H3N2		2012	USA	A/Santa_Clara/YGA_03062/2012	agtgatgccccattccttgatcggcttcgccgagatcag<br>aggtccctaaggggaagaggcaatactc	-	-
KM070106	Human H3N2 IAVs	Human	H3N2		2012	Singapore	A/Singapore/H2012.375/2012	agtgatgccccattccttgatcggcttcgccgagatcag<br>aggtccctaaggggaagaggcaatactc	-	-
KM070114	Human H3N2 IAVs	Human	H3N2		2012	Singapore	A/Singapore/H2012.705/2012	agtgatgccccattccttgatcggcttcgccgagatcag<br>aggtccctaaggggaagaggcaatactc	-	-
KM070122	Human H3N2 IAVs	Human	H3N2		2012	Singapore	A/Singapore/H2012.956/2012	agtgatgccccattccttgatcggcttcgccgagatcag<br>aggtccctaaggggaagaggcaatactc	-	-
CY135000	Human H3N2 IAVs	Human	H3N2		2012	USA	A/Texas/JMM_37/2012	agtgatgccccattccttgatcggcttcgccgagatcag<br>aggtccctaaggggaagaggcaatactc	-	-

KJ577153	Human H3N2 IAVs	Human	H3N2		2012	Thailand	A/Thailand/VIROAF1/2012	agtgatgccccattccttgatcggcttcgccgagatcag aggtccctaaggggaagaggcaatactc	-	-
KJ577193	Human H3N2 IAVs	Human	H3N2		2012	Thailand	A/Thailand/VIROAF6/2012	agtgatgccccattccttgatcggcttcgccgagatcag aggtccctaaggggaagaggcaatactc	-	-
CY168867	Human H3N2 IAVs	Human	H3N2		2012	USA	A/Boston/YGA_01048/2012	agtgatgccccattccttgatcggcttcgccgagatcag aggtccctaaggggaagaggcaatactc	-	-
CY182981	Human H3N2 IAVs	Human	H3N2		2012	USA	A/Houston/JMM_99/2012	agtgatgccccattccttgatcggcttcgccgagatcag aggtccctaaggggaagaggcaatactc	-	-
CY169267	Human H3N2 IAVs	Human	H3N2		2012	USA	A/Boston/YGA_01103/2012	agtgatgccccattccttgatcggcttcgccgagatcag aggtccctaaggggaagaggcaatactc	-	-
CY171531	Human H3N2 IAVs	Human	H3N2		2012	USA	A/Chicago/YGA_04121/2012	agtgatgccccattccttgatcggcttcgccgagatcag aggtccctaaggggaagaggcaatactc	-	-
CY170955	Human H3N2 IAVs	Human	H3N2		2012	USA	A/Santa_Clara/YGA_03068/2012	agtgatgccccattccttgatcggcttcgccgagatcag aggtccctaaggggaagaggcaatactc	-	-
KM070120	Human H3N2 IAVs	Human	H3N2		2012	Singapore	A/Singapore/H2012.923/2012	agtgatgccccattccttgatcggcttcgccgagatcag aggtccctaaggggaagaggcaatactc	-	-
CY171307	Human H3N2 IAVs	Human	H3N2		2012	USA	A/Chicago/YGA_04084/2012	agtgatgccccattccttgatcggcttcgccgagatcag aggtccctaaggggaagaggcaatactc	-	-
CY134888	Human H3N2 IAVs	Human	H3N2		2012	USA	A/Texas/JMM_22/2012	agtgatgccccattccttgatcggcttcgccgagatcag aggtccctaaggggaagaggcaatactc	-	-
CY167963	Human H3N2 IAVs	Human	H3N2		2012	USA	A/Boston/YGA_00012/2012	agtgatgccccattccttgatcggcttcgccgagatcag aggtccctaaggggaagaggcaatactc	-	-
CY171139	Human H3N2 IAVs	Human	H3N2		2012	USA	A/Chicago/YGA_04015/2012	agtgatgccccattccttgatcggcttcgccgagatcag aggtccctaaggggaagaggcaatactc	-	-
CY169283	Human H3N2 IAVs	Human	H3N2		2012	USA	A/Boston/YGA_01105/2012	agtgatgccccattccttgatcggcttcgccgagatcag aggtccctaaggggaagaggcaatactc	-	-
CY171115	Human H3N2 IAVs	Human	H3N2		2012	USA	A/Chicago/YGA_04012/2012	agtgatgccccattccttgatcggcttcgccgagatcag aggtccctaaggggaagaggcaatactc	-	-
CY171235	Human H3N2 IAVs	Human	H3N2		2012	USA	A/Chicago/YGA_04048/2012	agtgatgccccattccttgatcggcttcgccgagatcag aggtccctaaggggaagaggcaatactc	-	-
CY168891	Human H3N2 IAVs	Human	H3N2		2012	USA	A/Boston/YGA_01051/2012	agtgatgccccattccttgatcggcttcgccgagatcag aggtccctaaggggaagaggcaatactc	-	-
CY169027	Human H3N2 IAVs	Human	H3N2		2012	USA	A/Boston/YGA_01070/2012	agtgatgccccattccttgatcggcttcgccgagatcag aggtccctaaggggaagaggcaatactc	-	-
CY168747	Human H3N2 IAVs	Human	H3N2		2012	USA	A/Boston/YGA_01031/2012	agtgatgccccattccttgatcggcttcgccgagatcag aggtccctaaggggaagaggcaatactc	-	-
CY171051	Human H3N2 IAVs	Human	H3N2		2012	USA	A/Chicago/YGA_04001/2012	agtgatgccccattccttgatcggcttcgccgagatcag aggtccctaaggggaagaggcaatactc	-	-
CY168019	Human H3N2 IAVs	Human	H3N2		2012	USA	A/Boston/YGA_00021/2012	agtgatgccccattccttgatcggcttcgccgagatcag aggtccctaaggggaagaggcaatactc	-	-
CY168667	Human H3N2 IAVs	Human	H3N2		2012	USA	A/Boston/YGA_01020/2012	agtgatgccccattccttgatcggcttcgccgagatcag aggtccctaaggggaagaggcaatactc	-	-
KM070116	Human H3N2 IAVs	Human	H3N2		2012	Singapore	A/Singapore/C2012.801/2012	agtgatgccccattccttgatcggcttcgccgagatcag aggtccctaaggggaagaggcaatactc	-	-
KM070118	Human H3N2 IAVs	Human	H3N2		2012	Singapore	A/Singapore/S2012.833/2012	agtgatgccccattccttgatcggcttcgccgagatcag aggtccctaaggggaagaggcaatactc	-	-
KM070117	Human H3N2 IAVs	Human	H3N2		2012	Singapore	A/Singapore/H2012.828/2012	agtgatgccccattccttgatcggcttcgccgagatcag aggtccctaaggggaagaggcaatactc	-	-
KM070119	Human H3N2 IAVs	Human	H3N2		2012	Singapore	A/Singapore/H2012.855/2012	agtgatgccccattccttgatcggcttcgccgagatcag aggtccctaaggggaagaggcaatactc	-	-

KM070121	Human H3N2 IAVs	Human	H3N2		2012	Singapore	A/Singapore/H2012.934/2012	agtgatgccccattccttgatcggcttcgccgagatcag aggtccctaaggggaagaggcaatactc	-	-
CY167947	Human H3N2 IAVs	Human	H3N2		2012	USA	A/Boston/YGA_00010/2012	agtgatgccccattccttgatcggcttcgccgagatcag aggtccctaaggggaagaggcaatactc	-	-
CY148592	Human H3N2 IAVs	Human	H3N2		2012	USA	A/Boston/DOA2_134/2012	agtgatgccccattccttgatcggcttcgccgagatcag aggtccctaaggggaagaggcaatactc	-	-
CY167915	Human H3N2 IAVs	Human	H3N2		2012	USA	A/Boston/YGA_00006/2012	agtgatgccccattccttgatcggcttcgccgagatcag aggtccctaaggggaagaggcaatactc	-	-
CY169251	Human H3N2 IAVs	Human	H3N2		2012	USA	A/Boston/YGA_01101/2012	agtgatgccccattccttgatcggcttcgccgagatcag aggtccctaaggggaagaggcaatactc	-	-
CY171131	Human H3N2 IAVs	Human	H3N2		2012	USA	A/Chicago/YGA_04014/2012	agtgatgccccattccttgatcggcttcgccgagatcag aggtccctaaggggaagaggcaatactc	-	-
CY171467	Human H3N2 IAVs	Human	H3N2		2012	USA	A/Chicago/YGA_04112/2012	agtgatgccccattccttgatcggcttcgccgagatcag aggtccctaaggggaagaggcaatactc	-	-
CY182837	Human H3N2 IAVs	Human	H3N2		2012	USA	A/Houston/JMM_79/2012	agtgatgccccattccttgatcggcttcgccgagatcag aggtccctaaggggaagaggcaatactc	-	-
CY148360	Human H3N2 IAVs	Human	H3N2		2012	USA	A/Boston/DOA2_104/2012	agtgatgccccattccttgatcggcttcgccgagatcag aggtccctaaggggaagaggcaatactc	-	-
CY148576	Human H3N2 IAVs	Human	H3N2		2012	USA	A/Boston/DOA2_132/2012	agtgatgccccattccttgatcggcttcgccgagatcag aggtccctaaggggaagaggcaatactc	-	-
CY148816	Human H3N2 IAVs	Human	H3N2		2012	USA	A/Boston/DOA2_167/2012	agtgatgccccattccttgatcggcttcgccgagatcag aggtccctaaggggaagaggcaatactc	-	-
CY148824	Human H3N2 IAVs	Human	H3N2		2012	USA	A/Boston/DOA2_168/2012	agtgatgccccattccttgatcggcttcgccgagatcag aggtccctaaggggaagaggcaatactc	-	-
CY149168	Human H3N2 IAVs	Human	H3N2		2012	USA	A/Boston/DOA2_223/2012	agtgatgccccattccttgatcggcttcgccgagatcag aggtccctaaggggaagaggcaatactc	-	-
CY167939	Human H3N2 IAVs	Human	H3N2		2012	USA	A/Boston/YGA_00009/2012	agtgatgccccattccttgatcggcttcgccgagatcag aggtccctaaggggaagaggcaatactc	-	-
CY167995	Human H3N2 IAVs	Human	H3N2		2012	USA	A/Boston/YGA_00017/2012	agtgatgccccattccttgatcggcttcgccgagatcag aggtccctaaggggaagaggcaatactc	-	-
CY168555	Human H3N2 IAVs	Human	H3N2		2012	USA	A/Boston/YGA_01004/2012	agtgatgccccattccttgatcggcttcgccgagatcag aggtccctaaggggaagaggcaatactc	-	-
CY168579	Human H3N2 IAVs	Human	H3N2		2012	USA	A/Boston/YGA_01008/2012	agtgatgccccattccttgatcggcttcgccgagatcag aggtccctaaggggaagaggcaatactc	-	-
CY168595	Human H3N2 IAVs	Human	H3N2		2012	USA	A/Boston/YGA_01010/2012	agtgatgccccattccttgatcggcttcgccgagatcag aggtccctaaggggaagaggcaatactc	-	-
CY168691	Human H3N2 IAVs	Human	H3N2		2012	USA	A/Boston/YGA_01024/2012	agtgatgccccattccttgatcggcttcgccgagatcag aggtccctaaggggaagaggcaatactc	-	-
CY168755	Human H3N2 IAVs	Human	H3N2		2012	USA	A/Boston/YGA_01033/2012	agtgatgccccattccttgatcggcttcgccgagatcag aggtccctaaggggaagaggcaatactc	-	-
CY168915	Human H3N2 IAVs	Human	H3N2		2012	USA	A/Boston/YGA_01054/2012	agtgatgccccattccttgatcggcttcgccgagatcag aggtccctaaggggaagaggcaatactc	-	-
CY169035	Human H3N2 IAVs	Human	H3N2		2012	USA	A/Boston/YGA_01071/2012	agtgatgccccattccttgatcggcttcgccgagatcag aggtccctaaggggaagaggcaatactc	-	-
CY169115	Human H3N2 IAVs	Human	H3N2		2012	USA	A/Boston/YGA_01082/2012	agtgatgccccattccttgatcggcttcgccgagatcag aggtccctaaggggaagaggcaatactc	-	-
CY169123	Human H3N2 IAVs	Human	H3N2		2012	USA	A/Boston/YGA_01083/2012	agtgatgccccattccttgatcggcttcgccgagatcag aggtccctaaggggaagaggcaatactc	-	-
CY171067	Human H3N2 IAVs	Human	H3N2		2012	USA	A/Chicago/YGA_04006/2012	agtgatgccccattccttgatcggcttcgccgagatcag aggtccctaaggggaagaggcaatactc	-	-

CY171099	Human H3N2 IAVs	Human	H3N2		2012	USA	A/Chicago/YGA_04010/2012	agtgatgccccattccttgatcggcttcgccgagatcag aggtccctaaggggaagaggcaatactc	-	-
CY171107	Human H3N2 IAVs	Human	H3N2		2012	USA	A/Chicago/YGA_04011/2012	agtgatgccccattccttgatcggcttcgccgagatcag aggtccctaaggggaagaggcaatactc	-	-
CY171147	Human H3N2 IAVs	Human	H3N2		2012	USA	A/Chicago/YGA_04017/2012	agtgatgccccattccttgatcggcttcgccgagatcag aggtccctaaggggaagaggcaatactc	-	-
CY171195	Human H3N2 IAVs	Human	H3N2		2012	USA	A/Chicago/YGA_04037/2012	agtgatgccccattccttgatcggcttcgccgagatcag aggtccctaaggggaagaggcaatactc	-	-
CY171211	Human H3N2 IAVs	Human	H3N2		2012	USA	A/Chicago/YGA_04039/2012	agtgatgccccattccttgatcggcttcgccgagatcag aggtccctaaggggaagaggcaatactc	-	-
CY171251	Human H3N2 IAVs	Human	H3N2		2012	USA	A/Chicago/YGA_04054/2012	agtgatgccccattccttgatcggcttcgccgagatcag aggtccctaaggggaagaggcaatactc	-	-
CY171267	Human H3N2 IAVs	Human	H3N2		2012	USA	A/Chicago/YGA_04056/2012	agtgatgccccattccttgatcggcttcgccgagatcag aggtccctaaggggaagaggcaatactc	-	-
CY171275	Human H3N2 IAVs	Human	H3N2		2012	USA	A/Chicago/YGA_04064/2012	agtgatgccccattccttgatcggcttcgccgagatcag aggtccctaaggggaagaggcaatactc	-	-
CY171291	Human H3N2 IAVs	Human	H3N2		2012	USA	A/Chicago/YGA_04066/2012	agtgatgccccattccttgatcggcttcgccgagatcag aggtccctaaggggaagaggcaatactc	-	-
CY171371	Human H3N2 IAVs	Human	H3N2		2012	USA	A/Chicago/YGA_04096/2012	agtgatgccccattccttgatcggcttcgccgagatcag aggtccctaaggggaagaggcaatactc	-	-
CY171379	Human H3N2 IAVs	Human	H3N2		2012	USA	A/Chicago/YGA_04097/2012	agtgatgccccattccttgatcggcttcgccgagatcag aggtccctaaggggaagaggcaatactc	-	-
CY171387	Human H3N2 IAVs	Human	H3N2		2012	USA	A/Chicago/YGA_04098/2012	agtgatgccccattccttgatcggcttcgccgagatcag aggtccctaaggggaagaggcaatactc	-	-
CY169155	Human H3N2 IAVs	Human	H3N2		2012	USA	A/Boston/YGA_01088/2012	agtgatgccccattccttgatcggcttcgccgagatcag aggtccctaaggggaagaggcaatactc	-	-
CY171155	Human H3N2 IAVs	Human	H3N2		2012	USA	A/Chicago/YGA_04018/2012	agtgatgccccattccttgatcggcttcgccgagatcag aggtccctaaggggaagaggcaatactc	-	-
CY171259	Human H3N2 IAVs	Human	H3N2		2012	USA	A/Chicago/YGA_04055/2012	agtgatgccccattccttgatcggcttcgccgagatcag aggtccctaaggggaagaggcaatactc	-	-
CY171299	Human H3N2 IAVs	Human	H3N2		2012	USA	A/Chicago/YGA_04081/2012	agtgatgccccattccttgatcggcttcgccgagatcag aggtccctaaggggaagaggcaatactc	-	-
CY171363	Human H3N2 IAVs	Human	H3N2		2012	USA	A/Chicago/YGA_04095/2012	agtgatgccccattccttgatcggcttcgccgagatcag aggtccctaaggggaagaggcaatactc	-	-
CY171427	Human H3N2 IAVs	Human	H3N2		2012	USA	A/Chicago/YGA_04105/2012	agtgatgccccattccttgatcggcttcgccgagatcag aggtccctaaggggaagaggcaatactc	-	-
CY171435	Human H3N2 IAVs	Human	H3N2		2012	USA	A/Chicago/YGA_04106/2012	agtgatgccccattccttgatcggcttcgccgagatcag aggtccctaaggggaagaggcaatactc	-	-
CY171443	Human H3N2 IAVs	Human	H3N2		2012	USA	A/Chicago/YGA_04107/2012	agtgatgccccattccttgatcggcttcgccgagatcag aggtccctaaggggaagaggcaatactc	-	-
CY171475	Human H3N2 IAVs	Human	H3N2		2012	USA	A/Chicago/YGA_04113/2012	agtgatgccccattccttgatcggcttcgccgagatcag aggtccctaaggggaagaggcaatactc	-	-
CY171491	Human H3N2 IAVs	Human	H3N2		2012	USA	A/Chicago/YGA_04115/2012	agtgatgccccattccttgatcggcttcgccgagatcag aggtccctaaggggaagaggcaatactc	-	-
CY171499	Human H3N2 IAVs	Human	H3N2		2012	USA	A/Chicago/YGA_04117/2012	agtgatgccccattccttgatcggcttcgccgagatcag aggtccctaaggggaagaggcaatactc	-	-
CY171515	Human H3N2 IAVs	Human	H3N2		2012	USA	A/Chicago/YGA_04119/2012	agtgatgccccattccttgatcggcttcgccgagatcag aggtccctaaggggaagaggcaatactc	-	-
CY171563	Human H3N2 IAVs	Human	H3N2		2012	USA	A/Chicago/YGA_04126/2012	agtgatgccccattccttgatcggcttcgccgagatcag aggtccctaaggggaagaggcaatactc	-	-

CY182821	Human H3N2 IAVs	Human	H3N2		2012	USA	A/Houston/JMM_77/2012	agtgatgccccattccttgatcggcttcgccgagatcag aggtccctaaggggaagaggcaatactc	-	-
CY182853	Human H3N2 IAVs	Human	H3N2		2012	USA	A/Houston/JMM_81/2012	agtgatgccccattccttgatcggcttcgccgagatcag aggtccctaaggggaagaggcaatactc	-	-
KF789762	Human H3N2 IAVs	Human	H3N2		2012	USA	A/Michigan/32/2012	agtgatgccccattccttgatcggcttcgccgagatcag aggtccctaaggggaagaggcaatactc	-	-
CY170819	Human H3N2 IAVs	Human	H3N2		2012	USA	A/Santa_Clara/YGA_03049/2012	agtgatgccccattccttgatcggcttcgccgagatcag aggtccctaaggggaagaggcaatactc	-	-
CY170947	Human H3N2 IAVs	Human	H3N2		2012	USA	A/Santa_Clara/YGA_03067/2012	agtgatgccccattccttgatcggcttcgccgagatcag aggtccctaaggggaagaggcaatactc	-	-
CY135056	Human H3N2 IAVs	Human	H3N2		2012	USA	A/Texas/JMM_45/2012	agtgatgccccattccttgatcggcttcgccgagatcag aggtccctaaggggaagaggcaatactc	-	-
CY182749	Human H3N2 IAVs	Human	H3N2		2012	USA	A/Houston/JMM_68/2012	agtgatgccccattccttgatcggcttcgccgagatcag aggtccctaaggggaagaggcaatactc	-	-
CY168819	Human H3N2 IAVs	Human	H3N2		2012	USA	A/Boston/YGA_01042/2012	agtgatgccccattccttgatcggcttcgccgagatcag aggtccctaaggggaagaggcaatactc	-	-
CY168859	Human H3N2 IAVs	Human	H3N2		2012	USA	A/Boston/YGA_01047/2012	agtgatgccccattccttgatcggcttcgccgagatcag aggtccctaaggggaagaggcaatactc	-	-
CY171459	Human H3N2 IAVs	Human	H3N2		2012	USA	A/Chicago/YGA_04111/2012	agtgatgccccattccttgatcggcttcgccgagatcag aggtccctaaggggaagaggcaatactc	-	-
CY168651	Human H3N2 IAVs	Human	H3N2		2012	USA	A/Boston/YGA_01017/2012	agtgatgccccattccttgatcggcttcgccgagatcag aggtccctaaggggaagaggcaatactc	-	-
CY168995	Human H3N2 IAVs	Human	H3N2		2012	USA	A/Boston/YGA_01065/2012	agtgatgccccattccttgatcggcttcgccgagatcag aggtccctaaggggaagaggcaatactc	-	-
CY169083	Human H3N2 IAVs	Human	H3N2		2012	USA	A/Boston/YGA_01077/2012	agtgatgccccattccttgatcggcttcgccgagatcag aggtccctaaggggaagaggcaatactc	-	-
CY169043	Human H3N2 IAVs	Human	H3N2		2012	USA	A/Boston/YGA_01072/2012	agtgatgccccattccttgatcggcttcgccgagatcag aggtccctaaggggaagaggcaatactc	-	-
CY171219	Human H3N2 IAVs	Human	H3N2		2012	USA	A/Chicago/YGA_04046/2012	agtgatgccccattccttgatcggcttcgccgagatcag aggtccctaaggggaagaggcaatactc	-	-
CY169107	Human H3N2 IAVs	Human	H3N2		2012	USA	A/Boston/YGA_01081/2012	agtgatgccccattccttgatcggcttcgccgagatcag aggtccctaaggggaagaggcaatactc	-	-
CY170187	Human H3N2 IAVs	Human	H3N2		2012	USA	A/Boston/YGA_02013/2012	agtgatgccccattccttgatcggcttcgccgagatcag aggtccctaaggggaagaggcaatactc	-	-
CY182909	Human H3N2 IAVs	Human	H3N2		2012	USA	A/Houston/JMM_90/2012	agtgatgccccattccttgatcggcttcgccgagatcag aggtccctaaggggaagaggcaatactc	-	-
CY168923	Human H3N2 IAVs	Human	H3N2		2012	USA	A/Boston/YGA_01055/2012	agtgatgccccattccttgatcggcttcgccgagatcag aggtccctaaggggaagaggcaatactc	-	-
CY171507	Human H3N2 IAVs	Human	H3N2		2012	USA	A/Chicago/YGA_04118/2012	agtgatgccccattccttgatcggcttcgccgagatcag aggtccctaaggggaagaggcaatactc	-	-
CY134840	Human H3N2 IAVs	Human	H3N2		2012	USA	A/Texas/JMM_16/2012	agtgatgccccattccttgatcggcttcgccgagatcag aggtccctaaggggaagaggcaatactc	-	-
CY148688	Human H3N2 IAVs	Human	H3N2		2012	USA	A/Boston/DOA2_147/2012	agtgatgccccattccttgatcggcttcgccgagatcag aggtccctaaggggaagaggcaatactc	-	-
CY170211	Human H3N2 IAVs	Human	H3N2		2012	USA	A/Boston/YGA_02017/2012	agtgatgccccattccttgatcggcttcgccgagatcag aggtccctaaggggaagaggcaatactc	-	-
KF928617	Human H3N2 IAVs	Human	H3N2		2012	USA	A/Illinois/NHRC383545/2012	agtgatgccccattccttgatcggcttcgccgagatcag aggtccctaaggggaagaggcaatactc	-	-
CY148368	Human H3N2 IAVs	Human	H3N2		2012	USA	A/Boston/DOA2_105/2012	agtgatgccccattccttgatcggcttcgccgagatcag aggtccctaaggggaagaggcaatactc	-	-

CY148600	Human H3N2 IAVs	Human	H3N2		2012	USA	A/Boston/DOA2_135/2012	agtgatgccccattccttgatcggcttcgccgagatcag aggtccctaaggggaagaggcaatactc	-	-
CY149136	Human H3N2 IAVs	Human	H3N2		2012	USA	A/Boston/DOA2_219/2012	agtgatgccccattccttgatcggcttcgccgagatcag aggtccctaaggggaagaggcaatactc	-	-
CY168603	Human H3N2 IAVs	Human	H3N2		2012	USA	A/Boston/YGA_01011/2012	agtgatgccccattccttgatcggcttcgccgagatcag aggtccctaaggggaagaggcaatactc	-	-
CY168947	Human H3N2 IAVs	Human	H3N2		2012	USA	A/Boston/YGA_01059/2012	agtgatgccccattccttgatcggcttcgccgagatcag aggtccctaaggggaagaggcaatactc	-	-
CY167955	Human H3N2 IAVs	Human	H3N2		2012	USA	A/Boston/YGA_00011/2012	agtgatgccccattccttgatcggcttcgccgagatcag aggtccctaaggggaagaggcaatactc	-	-
CY148352	Human H3N2 IAVs	Human	H3N2		2012	USA	A/Boston/DOA2_103/2012	agtgatgccccattccttgatcggcttcgccgagatcag aggtccctaaggggaagaggcaatactc	-	-
CY168763	Human H3N2 IAVs	Human	H3N2		2012	USA	A/Boston/YGA_01035/2012	agtgatgccccattccttgatcggcttcgccgagatcag aggtccctaaggggaagaggcaatactc	-	-
CY169203	Human H3N2 IAVs	Human	H3N2		2012	USA	A/Boston/YGA_01095/2012	agtgatgccccattccttgatcggcttcgccgagatcag aggtccctaaggggaagaggcaatactc	-	-
CY148440	Human H3N2 IAVs	Human	H3N2		2012	USA	A/Boston/DOA2_114/2012	agtgatgccccattccttgatcggcttcgccgagatcag aggtccctaaggggaagaggcaatactc	-	-
CY148464	Human H3N2 IAVs	Human	H3N2		2012	USA	A/Boston/DOA2_117/2012	agtgatgccccattccttgatcggcttcgccgagatcag aggtccctaaggggaagaggcaatactc	-	-
CY168587	Human H3N2 IAVs	Human	H3N2		2012	USA	A/Boston/YGA_01009/2012	agtgatgccccattccttgatcggcttcgccgagatcag aggtccctaaggggaagaggcaatactc	-	-
CY168875	Human H3N2 IAVs	Human	H3N2		2012	USA	A/Boston/YGA_01049/2012	agtgatgccccattccttgatcggcttcgccgagatcag aggtccctaaggggaagaggcaatactc	-	-
CY182741	Human H3N2 IAVs	Human	H3N2		2012	USA	A/Houston/JMM_67/2012	agtgatgccccattccttgatcggcttcgccgagatcag aggtccctaaggggaagaggcaatactc	-	-
CY182781	Human H3N2 IAVs	Human	H3N2		2012	USA	A/Houston/JMM_72/2012	agtgatgccccattccttgatcggcttcgccgagatcag aggtccctaaggggaagaggcaatactc	-	-
CY182829	Human H3N2 IAVs	Human	H3N2		2012	USA	A/Houston/JMM_78/2012	agtgatgccccattccttgatcggcttcgccgagatcag aggtccctaaggggaagaggcaatactc	-	-
CY182885	Human H3N2 IAVs	Human	H3N2		2012	USA	A/Houston/JMM_86/2012	agtgatgccccattccttgatcggcttcgccgagatcag aggtccctaaggggaagaggcaatactc	-	-
CY134792	Human H3N2 IAVs	Human	H3N2		2012	USA	A/Texas/JMM_10/2012	agtgatgccccattccttgatcggcttcgccgagatcag aggtccctaaggggaagaggcaatactc	-	-
CY134832	Human H3N2 IAVs	Human	H3N2		2012	USA	A/Texas/JMM_15/2012	agtgatgccccattccttgatcggcttcgccgagatcag aggtccctaaggggaagaggcaatactc	-	-
CY134920	Human H3N2 IAVs	Human	H3N2		2012	USA	A/Texas/JMM_26/2012	agtgatgccccattccttgatcggcttcgccgagatcag aggtccctaaggggaagaggcaatactc	-	-
CY134936	Human H3N2 IAVs	Human	H3N2		2012	USA	A/Texas/JMM_29/2012	agtgatgccccattccttgatcggcttcgccgagatcag aggtccctaaggggaagaggcaatactc	-	-
CY134960	Human H3N2 IAVs	Human	H3N2		2012	USA	A/Texas/JMM_32/2012	agtgatgccccattccttgatcggcttcgccgagatcag aggtccctaaggggaagaggcaatactc	-	-
CY134976	Human H3N2 IAVs	Human	H3N2		2012	USA	A/Texas/JMM_34/2012	agtgatgccccattccttgatcggcttcgccgagatcag aggtccctaaggggaagaggcaatactc	-	-
CY135128	Human H3N2 IAVs	Human	H3N2		2012	USA	A/Texas/JMM_54/2012	agtgatgccccattccttgatcggcttcgccgagatcag aggtccctaaggggaagaggcaatactc	-	-
CY135136	Human H3N2 IAVs	Human	H3N2		2012	USA	A/Texas/JMM_56/2012	agtgatgccccattccttgatcggcttcgccgagatcag aggtccctaaggggaagaggcaatactc	-	-
CY135144	Human H3N2 IAVs	Human	H3N2		2012	USA	A/Texas/JMM_57/2012	agtgatgccccattccttgatcggcttcgccgagatcag aggtccctaaggggaagaggcaatactc	-	-

CY134784	Human H3N2 IAVs	Human	H3N2		2012	USA	A/Texas/JMM_9/2012	agtgatgccccattccttgatcggcttcgccgagatcag aggtccctaaggggaagaggcaatactc	-	-
CY182805	Human H3N2 IAVs	Human	H3N2		2012	USA	A/Houston/JMM_75/2012	agtgatgccccattccttgatcggcttcgccgagatcag aggtccctaaggggaagaggcaatactc	-	-
CY182813	Human H3N2 IAVs	Human	H3N2		2012	USA	A/Houston/JMM_76/2012	agtgatgccccattccttgatcggcttcgccgagatcag aggtccctaaggggaagaggcaatactc	-	-
CY182845	Human H3N2 IAVs	Human	H3N2		2012	USA	A/Houston/JMM_80/2012	agtgatgccccattccttgatcggcttcgccgagatcag aggtccctaaggggaagaggcaatactc	-	-
CY182893	Human H3N2 IAVs	Human	H3N2		2012	USA	A/Houston/JMM_87/2012	agtgatgccccattccttgatcggcttcgccgagatcag aggtccctaaggggaagaggcaatactc	-	-
CY182941	Human H3N2 IAVs	Human	H3N2		2012	USA	A/Houston/JMM_94/2012	agtgatgccccattccttgatcggcttcgccgagatcag aggtccctaaggggaagaggcaatactc	-	-
CY134816	Human H3N2 IAVs	Human	H3N2		2012	USA	A/Texas/JMM_13/2012	agtgatgccccattccttgatcggcttcgccgagatcag aggtccctaaggggaagaggcaatactc	-	-
CY134856	Human H3N2 IAVs	Human	H3N2		2012	USA	A/Texas/JMM_18/2012	agtgatgccccattccttgatcggcttcgccgagatcag aggtccctaaggggaagaggcaatactc	-	-
CY134968	Human H3N2 IAVs	Human	H3N2		2012	USA	A/Texas/JMM_33/2012	agtgatgccccattccttgatcggcttcgccgagatcag aggtccctaaggggaagaggcaatactc	-	-
CY134760	Human H3N2 IAVs	Human	H3N2		2012	USA	A/Texas/JMM_4/2012	agtgatgccccattccttgatcggcttcgccgagatcag aggtccctaaggggaagaggcaatactc	-	-
CY135048	Human H3N2 IAVs	Human	H3N2		2012	USA	A/Texas/JMM_44/2012	agtgatgccccattccttgatcggcttcgccgagatcag aggtccctaaggggaagaggcaatactc	-	-
CY182797	Human H3N2 IAVs	Human	H3N2		2012	USA	A/Houston/JMM_74/2012	agtgatgccccattccttgatcggcttcgccgagatcag aggtccctaaggggaagaggcaatactc	-	-
CY135096	Human H3N2 IAVs	Human	H3N2		2012	USA	A/Texas/JMM_50/2012	agtgatgccccattccttgatcggcttcgccgagatcag aggtccctaaggggaagaggcaatactc	-	-
CY148608	Human H3N2 IAVs	Human	H3N2		2012	USA	A/Boston/DOA2_136/2012	agtgatgccccattccttgatcggcttcgccgagatcag aggtccctaaggggaagaggcaatactc	-	-
CY135152	Human H3N2 IAVs	Human	H3N2		2012	USA	A/Texas/JMM_58/2012	agtgatgccccattccttgatcggcttcgccgagatcag aggtccctaaggggaagaggcaatactc	-	-
CY187630	Human H3N2 IAVs	Human	H3N2		2012	USA	A/Houston/JMM_42/2012	agtgatgccccattccttgatcggcttcgccgagatcag aggtccctaaggggaagaggcaatactc	-	-
CY182957	Human H3N2 IAVs	Human	H3N2		2012	USA	A/Houston/JMM_96/2012	agtgatgccccattccttgatcggcttcgccgagatcag aggtccctaaggggaagaggcaatactc	-	-
CY182725	Human H3N2 IAVs	Human	H3N2		2012	USA	A/Houston/JMM_65/2012	agtgatgccccattccttgatcggcttcgccgagatcag aggtccctaaggggaagaggcaatactc	-	-
CY182877	Human H3N2 IAVs	Human	H3N2		2012	USA	A/Houston/JMM_85/2012	agtgatgccccattccttgatcggcttcgccgagatcag aggtccctaaggggaagaggcaatactc	-	-
CY169195	Human H3N2 IAVs	Human	H3N2		2012	USA	A/Boston/YGA_01093/2012	agtgatgccccattccttgatcggcttcgccgagatcag aggtccctaaggggaagaggcaatactc	-	-
CY182869	Human H3N2 IAVs	Human	H3N2		2012	USA	A/Houston/JMM_84/2012	agtgatgccccattccttgatcggcttcgccgagatcag aggtccctaaggggaagaggcaatactc	-	-
CY134848	Human H3N2 IAVs	Human	H3N2		2012	USA	A/Texas/JMM_17/2012	agtgatgccccattccttgatcggcttcgccgagatcag aggtccctaaggggaagaggcaatactc	-	-
CY182861	Human H3N2 IAVs	Human	H3N2		2012	USA	A/Houston/JMM_82/2012	agtgatgccccattccttgatcggcttcgccgagatcag aggtccctaaggggaagaggcaatactc	-	-
CY135072	Human H3N2 IAVs	Human	H3N2		2012	USA	A/Texas/JMM_47/2012	agtgatgccccattccttgatcggcttcgccgagatcag aggtccctaaggggaagaggcaatactc	-	-
CY134744	Human H3N2 IAVs	Human	H3N2		2012	USA	A/Texas/JMM_2/2012	agtgatgccccattccttgatcggcttcgccgagatcag aggtccctaaggggaagaggcaatactc	-	-

CY182693	Human H3N2 IAVs	Human	H3N2		2012	USA	A/Houston/JMM_61/2012	agtgatgccccattccttgatcggcttcgccgagatcag<br>aggtccctaaggggaagaggcaatactc	-	-
CY135064	Human H3N2 IAVs	Human	H3N2		2012	USA	A/Texas/JMM_46/2012	agtgatgccccattccttgatcggcttcgccgagatcag<br>aggtccctaaggggaagaggcaatactc	-	-
CY182789	Human H3N2 IAVs	Human	H3N2		2012	USA	A/Houston/JMM_73/2012	agtgatgccccattccttgatcggcttcgccgagatcag<br>aggtccctaaggggaagaggcaatactc	-	-
KJ943105	Human H3N2 IAVs	Human	H3N2		2012	Chile	A/Santiago/op20d1/2012	agtgatgccccattccttgatcggcttcgccgagatcag<br>aggtccctaaggggaagaggcaatactc	-	-
KJ942995	Human H3N2 IAVs	Human	H3N2		2012	Chile	A/Santiago/p37d0/2012	agtgatgccccattccttgatcggcttcgccgagatcag<br>aggtccctaaggggaagaggcaatactc	-	-
KJ942963	Human H3N2 IAVs	Human	H3N2		2012	Chile	A/Santiago/p37d1/2012	agtgatgccccattccttgatcggcttcgccgagatcag<br>aggtccctaaggggaagaggcaatactc	-	-
KJ943158	Human H3N2 IAVs	Human	H3N2		2012	Chile	A/Santiago/p37d3/2012	agtgatgccccattccttgatcggcttcgccgagatcag<br>aggtccctaaggggaagaggcaatactc	-	-
CY168931	Human H3N2 IAVs	Human	H3N2		2012	USA	A/Boston/YGA_01056/2012	agtgatgccccattccttgatcggcttcgccgagatcag<br>aggtccctaaggggaagaggcaatactc	-	-
CY171355	Human H3N2 IAVs	Human	H3N2		2012	USA	A/Chicago/YGA_04094/2012	agtgatgccccattccttgatcggcttcgccgagatcag<br>aggtccctaaggggaagaggcaatactc	-	-
CY134768	Human H3N2 IAVs	Human	H3N2		2012	USA	A/Texas/JMM_5/2012	agtgatgccccattccttgatcggcttcgccgagatcag<br>aggtccctaaggggaagaggcaatactc	-	-
CY134864	Human H3N2 IAVs	Human	H3N2		2012	USA	A/Texas/JMM_19/2012	agtgatgccccattccttgatcggcttcgccgagatcag<br>aggtccctaaggggaagaggcaatactc	-	-
CY134872	Human H3N2 IAVs	Human	H3N2		2012	USA	A/Texas/JMM_20/2012	agtgatgccccattccttgatcggcttcgccgagatcag<br>aggtccctaaggggaagaggcaatactc	-	-
CY134896	Human H3N2 IAVs	Human	H3N2		2012	USA	A/Texas/JMM_23/2012	agtgatgccccattccttgatcggcttcgccgagatcag<br>aggtccctaaggggaagaggcaatactc	-	-
CY135104	Human H3N2 IAVs	Human	H3N2		2012	USA	A/Texas/JMM_51/2012	agtgatgccccattccttgatcggcttcgccgagatcag<br>aggtccctaaggggaagaggcaatactc	-	-
CY182917	Human H3N2 IAVs	Human	H3N2		2012	USA	A/Houston/JMM_91/2012	agtgatgccccattccttgatcggcttcgccgagatcag<br>aggtccctaaggggaagaggcaatactc	-	-
CY161164	Human H3N2 IAVs	Human	H3N2		2012	Peru	A/Peru/PER111/2012	agtgatgccccattccttgatcggcttcgccgagatcag<br>aggtccctaaggggaagaggcaatactc	-	-
CY161268	Human H3N2 IAVs	Human	H3N2		2012	Peru	A/Peru/PER124/2012	agtgatgccccattccttgatcggcttcgccgagatcag<br>aggtccctaaggggaagaggcaatactc	-	-
CY161868	Human H3N2 IAVs	Human	H3N2		2012	Peru	A/Peru/PER200/2012	agtgatgccccattccttgatcggcttcgccgagatcag<br>aggtccctaaggggaagaggcaatactc	-	-
CY162332	Human H3N2 IAVs	Human	H3N2		2012	Peru	A/Peru/PER260/2012	agtgatgccccattccttgatcggcttcgccgagatcag<br>aggtccctaaggggaagaggcaatactc	-	-
CY163276	Human H3N2 IAVs	Human	H3N2		2012	Peru	A/Peru/PER384/2012	agtgatgccccattccttgatcggcttcgccgagatcag<br>aggtccctaaggggaagaggcaatactc	-	-
KM070103	Human H3N2 IAVs	Human	H3N2		2012	Singapore	A/Singapore/C2012.247/2012	agtgatgccccattccttgatcggcttcgccgagatcag<br>aggtccctaaggggaagaggcaatactc	-	-
CY148656	Human H3N2 IAVs	Human	H3N2		2012	USA	A/Boston/DOA2_143/2012	agtgatgccccattccttgatcggcttcgccgagatcag<br>aggtccctaaggggaagaggcaatactc	-	-
CY148640	Human H3N2 IAVs	Human	H3N2		2012	USA	A/Boston/DOA2_141/2012	agtgatgccccattccttgatcggcttcgccgagatcag<br>aggtccctaaggggaagaggcaatactc	-	-
CY148624	Human H3N2 IAVs	Human	H3N2		2012	USA	A/Boston/DOA2_138/2012	agtgatgccccattccttgatcggcttcgccgagatcag<br>aggtccctaaggggaagaggcaatactc	-	-
CY167907	Human H3N2 IAVs	Human	H3N2		2012	USA	A/Boston/YGA_00004/2012	agtgatgccccattccttgatcggcttcgccgagatcag<br>aggtccctaaggggaagaggcaatactc	-	-

CY168011	Human H3N2 IAVs	Human	H3N2		2012	USA	A/Boston/YGA_00020/2012	agtgatgccccattccttgatcggcttcgccgagatcag aggtccctaaggggaagaggcaatactc	-	-
CY168843	Human H3N2 IAVs	Human	H3N2		2012	USA	A/Boston/YGA_01045/2012	agtgatgccccattccttgatcggcttcgccgagatcag aggtccctaaggggaagaggcaatactc	-	-
CY169227	Human H3N2 IAVs	Human	H3N2		2012	USA	A/Boston/YGA_01098/2012	agtgatgccccattccttgatcggcttcgccgagatcag aggtccctaaggggaagaggcaatactc	-	-
CY170139	Human H3N2 IAVs	Human	H3N2		2012	USA	A/Boston/YGA_02005/2012	agtgatgccccattccttgatcggcttcgccgagatcag aggtccctaaggggaagaggcaatactc	-	-
CY170195	Human H3N2 IAVs	Human	H3N2		2012	USA	A/Boston/YGA_02014/2012	agtgatgccccattccttgatcggcttcgccgagatcag aggtccctaaggggaagaggcaatactc	-	-
CY170203	Human H3N2 IAVs	Human	H3N2		2012	USA	A/Boston/YGA_02015/2012	agtgatgccccattccttgatcggcttcgccgagatcag aggtccctaaggggaagaggcaatactc	-	-
CY168707	Human H3N2 IAVs	Human	H3N2		2012	USA	A/Boston/YGA_01026/2012	agtgatgccccattccttgatcggcttcgccgagatcag aggtccctaaggggaagaggcaatactc	-	-
CY170115	Human H3N2 IAVs	Human	H3N2		2012	USA	A/Boston/YGA_02002/2012	agtgatgccccattccttgatcggcttcgccgagatcag aggtccctaaggggaagaggcaatactc	-	-
CY182973	Human H3N2 IAVs	Human	H3N2		2012	USA	A/Houston/JMM_98/2012	agtgatgccccattccttgatcggcttcgccgagatcag aggtccctaaggggaagaggcaatactc	-	-
CY171451	Human H3N2 IAVs	Human	H3N2		2012	USA	A/Chicago/YGA_04110/2012	agtgatgccccattccttgatcggcttcgccgagatcag aggtccctaaggggaagaggcaatactc	-	-
CY181605	Human H3N2 IAVs	Human	H3N2		2012	Nicaragua	A/Nicaragua/AGA2_03/2012	agtgatgccccattccttgatcggcttcgccgagatcag aggtccctaaggggaagaggcaatactc	-	-
CY181613	Human H3N2 IAVs	Human	H3N2		2012	Nicaragua	A/Nicaragua/AGA2_04/2012	agtgatgccccattccttgatcggcttcgccgagatcag aggtccctaaggggaagaggcaatactc	-	-
CY181621	Human H3N2 IAVs	Human	H3N2		2012	Nicaragua	A/Nicaragua/AGA2_05/2012	agtgatgccccattccttgatcggcttcgccgagatcag aggtccctaaggggaagaggcaatactc	-	-
CY181629	Human H3N2 IAVs	Human	H3N2		2012	Nicaragua	A/Nicaragua/AGA2_06/2012	agtgatgccccattccttgatcggcttcgccgagatcag aggtccctaaggggaagaggcaatactc	-	-
CY181637	Human H3N2 IAVs	Human	H3N2		2012	Nicaragua	A/Nicaragua/AGA2_07/2012	agtgatgccccattccttgatcggcttcgccgagatcag aggtccctaaggggaagaggcaatactc	-	-
CY181645	Human H3N2 IAVs	Human	H3N2		2012	Nicaragua	A/Nicaragua/AGA2_08/2012	agtgatgccccattccttgatcggcttcgccgagatcag aggtccctaaggggaagaggcaatactc	-	-
CY181653	Human H3N2 IAVs	Human	H3N2		2012	Nicaragua	A/Nicaragua/AGA2_09/2012	agtgatgccccattccttgatcggcttcgccgagatcag aggtccctaaggggaagaggcaatactc	-	-
CY182253	Human H3N2 IAVs	Human	H3N2		2012	Nicaragua	A/Nicaragua/AGA2_100/2012	agtgatgccccattccttgatcggcttcgccgagatcag aggtccctaaggggaagaggcaatactc	-	-
CY182269	Human H3N2 IAVs	Human	H3N2		2012	Nicaragua	A/Nicaragua/AGA2_102/2012	agtgatgccccattccttgatcggcttcgccgagatcag aggtccctaaggggaagaggcaatactc	-	-
CY182277	Human H3N2 IAVs	Human	H3N2		2012	Nicaragua	A/Nicaragua/AGA2_103/2012	agtgatgccccattccttgatcggcttcgccgagatcag aggtccctaaggggaagaggcaatactc	-	-
CY182293	Human H3N2 IAVs	Human	H3N2		2012	Nicaragua	A/Nicaragua/AGA2_105/2012	agtgatgccccattccttgatcggcttcgccgagatcag aggtccctaaggggaagaggcaatactc	-	-
CY182301	Human H3N2 IAVs	Human	H3N2		2012	Nicaragua	A/Nicaragua/AGA2_106/2012	agtgatgccccattccttgatcggcttcgccgagatcag aggtccctaaggggaagaggcaatactc	-	-
CY182309	Human H3N2 IAVs	Human	H3N2		2012	Nicaragua	A/Nicaragua/AGA2_107/2012	agtgatgccccattccttgatcggcttcgccgagatcag aggtccctaaggggaagaggcaatactc	-	-
CY182317	Human H3N2 IAVs	Human	H3N2		2012	Nicaragua	A/Nicaragua/AGA2_108/2012	agtgatgccccattccttgatcggcttcgccgagatcag aggtccctaaggggaagaggcaatactc	-	-
CY182325	Human H3N2 IAVs	Human	H3N2		2012	Nicaragua	A/Nicaragua/AGA2_109/2012	agtgatgccccattccttgatcggcttcgccgagatcag aggtccctaaggggaagaggcaatactc	-	-

CY181661	Human H3N2 IAVs	Human	H3N2		2012	Nicaragua	A/Nicaragua/AGA2_11/2012	agtgatgccccattccttgatcggcttcgccgagatcag aggtccctaaggggaagaggcaatactc	-	-
CY181677	Human H3N2 IAVs	Human	H3N2		2012	Nicaragua	A/Nicaragua/AGA2_13/2012	agtgatgccccattccttgatcggcttcgccgagatcag aggtccctaaggggaagaggcaatactc	-	-
CY181701	Human H3N2 IAVs	Human	H3N2		2012	Nicaragua	A/Nicaragua/AGA2_18/2012	agtgatgccccattccttgatcggcttcgccgagatcag aggtccctaaggggaagaggcaatactc	-	-
CY181709	Human H3N2 IAVs	Human	H3N2		2012	Nicaragua	A/Nicaragua/AGA2_19/2012	agtgatgccccattccttgatcggcttcgccgagatcag aggtccctaaggggaagaggcaatactc	-	-
CY181725	Human H3N2 IAVs	Human	H3N2		2012	Nicaragua	A/Nicaragua/AGA2_21/2012	agtgatgccccattccttgatcggcttcgccgagatcag aggtccctaaggggaagaggcaatactc	-	-
CY182077	Human H3N2 IAVs	Human	H3N2		2012	Nicaragua	A/Nicaragua/AGA2_72/2012	agtgatgccccattccttgatcggcttcgccgagatcag aggtccctaaggggaagaggcaatactc	-	-
CY182093	Human H3N2 IAVs	Human	H3N2		2012	Nicaragua	A/Nicaragua/AGA2_76/2012	agtgatgccccattccttgatcggcttcgccgagatcag aggtccctaaggggaagaggcaatactc	-	-
CY182133	Human H3N2 IAVs	Human	H3N2		2012	Nicaragua	A/Nicaragua/AGA2_81/2012	agtgatgccccattccttgatcggcttcgccgagatcag aggtccctaaggggaagaggcaatactc	-	-
CY182149	Human H3N2 IAVs	Human	H3N2		2012	Nicaragua	A/Nicaragua/AGA2_84/2012	agtgatgccccattccttgatcggcttcgccgagatcag aggtccctaaggggaagaggcaatactc	-	-
CY182157	Human H3N2 IAVs	Human	H3N2		2012	Nicaragua	A/Nicaragua/AGA2_85/2012	agtgatgccccattccttgatcggcttcgccgagatcag aggtccctaaggggaagaggcaatactc	-	-
CY182173	Human H3N2 IAVs	Human	H3N2		2012	Nicaragua	A/Nicaragua/AGA2_88/2012	agtgatgccccattccttgatcggcttcgccgagatcag aggtccctaaggggaagaggcaatactc	-	-
CY182181	Human H3N2 IAVs	Human	H3N2		2012	Nicaragua	A/Nicaragua/AGA2_89/2012	agtgatgccccattccttgatcggcttcgccgagatcag aggtccctaaggggaagaggcaatactc	-	-
CY182189	Human H3N2 IAVs	Human	H3N2		2012	Nicaragua	A/Nicaragua/AGA2_90/2012	agtgatgccccattccttgatcggcttcgccgagatcag aggtccctaaggggaagaggcaatactc	-	-
CY182197	Human H3N2 IAVs	Human	H3N2		2012	Nicaragua	A/Nicaragua/AGA2_91/2012	agtgatgccccattccttgatcggcttcgccgagatcag aggtccctaaggggaagaggcaatactc	-	-
CY182213	Human H3N2 IAVs	Human	H3N2		2012	Nicaragua	A/Nicaragua/AGA2_93/2012	agtgatgccccattccttgatcggcttcgccgagatcag aggtccctaaggggaagaggcaatactc	-	-
CY182229	Human H3N2 IAVs	Human	H3N2		2012	Nicaragua	A/Nicaragua/AGA2_95/2012	agtgatgccccattccttgatcggcttcgccgagatcag aggtccctaaggggaagaggcaatactc	-	-
CY182237	Human H3N2 IAVs	Human	H3N2		2012	Nicaragua	A/Nicaragua/AGA2_96/2012	agtgatgccccattccttgatcggcttcgccgagatcag aggtccctaaggggaagaggcaatactc	-	-
CY181669	Human H3N2 IAVs	Human	H3N2		2012	Nicaragua	A/Nicaragua/AGA2_12/2012	agtgatgccccattccttgatcggcttcgccgagatcag aggtccctaaggggaagaggcaatactc	-	-
CY181693	Human H3N2 IAVs	Human	H3N2		2012	Nicaragua	A/Nicaragua/AGA2_17/2012	agtgatgccccattccttgatcggcttcgccgagatcag aggtccctaaggggaagaggcaatactc	-	-
CY181589	Human H3N2 IAVs	Human	H3N2		2012	Nicaragua	A/Nicaragua/AGA2_01/2012	agtgatgccccattccttgatcggcttcgccgagatcag aggtccctaaggggaagaggcaatactc	-	-
CY181597	Human H3N2 IAVs	Human	H3N2		2012	Nicaragua	A/Nicaragua/AGA2_02/2012	agtgatgccccattccttgatcggcttcgccgagatcag aggtccctaaggggaagaggcaatactc	-	-
CY182261	Human H3N2 IAVs	Human	H3N2		2012	Nicaragua	A/Nicaragua/AGA2_101/2012	agtgatgccccattccttgatcggcttcgccgagatcag aggtccctaaggggaagaggcaatactc	-	-
CY182333	Human H3N2 IAVs	Human	H3N2		2012	Nicaragua	A/Nicaragua/AGA2_110/2012	agtgatgccccattccttgatcggcttcgccgagatcag aggtccctaaggggaagaggcaatactc	-	-
CY182165	Human H3N2 IAVs	Human	H3N2		2012	Nicaragua	A/Nicaragua/AGA2_86/2012	agtgatgccccattccttgatcggcttcgccgagatcag aggtccctaaggggaagaggcaatactc	-	-
CY182221	Human H3N2 IAVs	Human	H3N2		2012	Nicaragua	A/Nicaragua/AGA2_94/2012	agtgatgccccattccttgatcggcttcgccgagatcag aggtccctaaggggaagaggcaatactc	-	-

KF612208	Human H3N2 IAVs	Human	H3N2		2012	Bolivia	A/Santa_Cruz/1391/2012	agtgatgccccattccttgatcggcttcgccgagatcag aggtccctaaggggaagaggcaatactc	-	-
CY168787	Human H3N2 IAVs	Human	H3N2		2012	USA	A/Boston/YGA_01038/2012	agtgatgccccattccttgatcggcttcgccgagatcag aggtccctaaggggaagaggcaatactc	-	-
CY135040	Human H3N2 IAVs	Human	H3N2		2012	USA	A/Texas/JMM_43/2012	agtgatgccccattccttgatcggcttcgccgagatcag aggtccctaaggggaagaggcaatactc	-	-
CY135160	Human H3N2 IAVs	Human	H3N2		2012	USA	A/Texas/JMM_59/2012	agtgatgccccattccttgatcggcttcgccgagatcag aggtccctaaggggaagaggcaatactc	-	-
CY171227	Human H3N2 IAVs	Human	H3N2		2012	USA	A/Chicago/YGA_04047/2012	agtgatgccccattccttgatcggcttcgccgagatcag aggtccctaaggggaagaggcaatactc	-	-
CY167979	Human H3N2 IAVs	Human	H3N2		2012	USA	A/Boston/YGA_00015/2012	agtgatgccccattccttgatcggcttcgccgagatcag aggtccctaaggggaagaggcaatactc	-	-
CY171187	Human H3N2 IAVs	Human	H3N2		2012	USA	A/Chicago/YGA_04036/2012	agtgatgccccattccttgatcggcttcgccgagatcag aggtccctaaggggaagaggcaatactc	-	-
CY171283	Human H3N2 IAVs	Human	H3N2		2012	USA	A/Chicago/YGA_04065/2012	agtgatgccccattccttgatcggcttcgccgagatcag aggtccctaaggggaagaggcaatactc	-	-
CY171555	Human H3N2 IAVs	Human	H3N2		2012	USA	A/Chicago/YGA_04124/2012	agtgatgccccattccttgatcggcttcgccgagatcag aggtccctaaggggaagaggcaatactc	-	-
CY171523	Human H3N2 IAVs	Human	H3N2		2012	USA	A/Chicago/YGA_04120/2012	agtgatgccccattccttgatcggcttcgccgagatcag aggtccctaaggggaagaggcaatactc	-	-
CY163044	Human H3N2 IAVs	Human	H3N2		2012	Peru	A/Peru/PER353/2012	agtgatgccccattccttgatcggcttcgccgagatcag aggtccctaaggggaagaggcaatactc	-	-
KJ943690	Human H3N2 IAVs	Human	H3N2		2012	Chile	A/Santiago/p1d1/2012	agtgatgccccattccttgatcggcttcgccgagatcag aggtccctaaggggaagaggcaatactc	-	-
KJ943613	Human H3N2 IAVs	Human	H3N2		2012	Chile	A/Santiago/p1d2/2012	agtgatgccccattccttgatcggcttcgccgagatcag aggtccctaaggggaagaggcaatactc	-	-
KJ943682	Human H3N2 IAVs	Human	H3N2		2012	Chile	A/Santiago/p20d0/2012	agtgatgccccattccttgatcggcttcgccgagatcag aggtccctaaggggaagaggcaatactc	-	-
KJ943601	Human H3N2 IAVs	Human	H3N2		2012	Chile	A/Santiago/p6d1/2012	agtgatgccccattccttgatcggcttcgccgagatcag aggtccctaaggggaagaggcaatactc	-	-
KJ943741	Human H3N2 IAVs	Human	H3N2		2012	Chile	A/Santiago/p6d2/2012	agtgatgccccattccttgatcggcttcgccgagatcag aggtccctaaggggaagaggcaatactc	-	-
KJ943763	Human H3N2 IAVs	Human	H3N2		2012	Chile	A/Santiago/p6d3/2012	agtgatgccccattccttgatcggcttcgccgagatcag aggtccctaaggggaagaggcaatactc	-	-
KJ943779	Human H3N2 IAVs	Human	H3N2		2012	Chile	A/Santiago/p6d5/2012	agtgatgccccattccttgatcggcttcgccgagatcag aggtccctaaggggaagaggcaatactc	-	-
CY160692	Human H3N2 IAVs	Human	H3N2		2012	Peru	A/Peru/PER051/2012	agtgatgccccattccttgatcggcttcgccgagatcag aggtccctaaggggaagaggcaatactc	-	-
CY161092	Human H3N2 IAVs	Human	H3N2		2012	Peru	A/Peru/PER102/2012	agtgatgccccattccttgatcggcttcgccgagatcag aggtccctaaggggaagaggcaatactc	-	-
KM070105	Human H3N2 IAVs	Human	H3N2		2012	Singapore	A/Singapore/H2012.301/2012	agtgatgccccattccttgatcggcttcgccgagatcag aggtccctaaggggaagaggcaatactc	-	-
KJ943787	Human H3N2 IAVs	Human	H3N2		2012	Chile	A/Santiago/p4d0/2012	agtgatgccccattccttgatcggcttcgccgagatcag aggtccctaaggggaagaggcaatactc	-	-
KJ943733	Human H3N2 IAVs	Human	H3N2		2012	Chile	A/Santiago/p4d1/2012	agtgatgccccattccttgatcggcttcgccgagatcag aggtccctaaggggaagaggcaatactc	-	-
KJ943716	Human H3N2 IAVs	Human	H3N2		2012	Chile	A/Santiago/p4d2/2012	agtgatgccccattccttgatcggcttcgccgagatcag aggtccctaaggggaagaggcaatactc	-	-
KJ943638	Human H3N2 IAVs	Human	H3N2		2012	Chile	A/Santiago/p4d3/2012	agtgatgccccattccttgatcggcttcgccgagatcag aggtccctaaggggaagaggcaatactc	-	-

KJ943142	Human H3N2 IAVs	Human	H3N2		2012	Chile	A/Santiago/p5d0/2012	agtgatgccccattccttgatcggcttcgccgagatcag aggtccctaaggggaagaggcaatactc	-	-
KJ943150	Human H3N2 IAVs	Human	H3N2		2012	Chile	A/Santiago/p5d1/2012	agtgatgccccattccttgatcggcttcgccgagatcag aggtccctaaggggaagaggcaatactc	-	-
KJ942915	Human H3N2 IAVs	Human	H3N2		2012	Chile	A/Santiago/p5d2/2012	agtgatgccccattccttgatcggcttcgccgagatcag aggtccctaaggggaagaggcaatactc	-	-
KJ943113	Human H3N2 IAVs	Human	H3N2		2012	Chile	A/Santiago/p5d3/2012	agtgatgccccattccttgatcggcttcgccgagatcag aggtccctaaggggaagaggcaatactc	-	-
KJ943187	Human H3N2 IAVs	Human	H3N2		2012	Chile	A/Santiago/op7d1/2012	agtgatgccccattccttgatcggcttcgccgagatcag aggtccctaaggggaagaggcaatactc	-	-
KC892441	Human H3N2 IAVs	Human	H3N2		2012	USA	A/California/17/2012	agtgatgccccattccttgatcggcttcgccgagatcag aggtccctaaggggaagaggcaatactc	-	-
CY171483	Human H3N2 IAVs	Human	H3N2		2012	USA	A/Chicago/YGA_04114/2012	agtgatgccccattccttgatcggcttcgccgagatcag aggtccctaaggggaagaggcaatactc	-	-
KF789772	Human H3N2 IAVs	Human	H3N2		2012	USA	A/Michigan/33/2012	agtgatgccccattccttgatcggcttcgccgagatcag aggtccctaaggggaagaggcaatactc	-	-
JX913015	Human H3N2 IAVs	Human	H3N2		2012	Czech_Republic	A/Czech_Republic/130/2012	agtgatgccccattccttgatcggcttcgccgagatcag aggtccctaaggggaagaggcaatactc	-	-
JX913007	Human H3N2 IAVs	Human	H3N2		2012	Czech_Republic	A/Czech_Republic/20/2012	agtgatgccccattccttgatcggcttcgccgagatcag aggtccctaaggggaagaggcaatactc	-	-
JX913055	Human H3N2 IAVs	Human	H3N2		2012	Czech_Republic	A/Czech_Republic/98/2012	agtgatgccccattccttgatcggcttcgccgagatcag aggtccctaaggggaagaggcaatactc	-	-
CY161756	Human H3N2 IAVs	Human	H3N2		2012	Peru	A/Peru/PER186/2012	agtgatgccccattccttgatcggcttcgccgagatcag aggtccctaaggggaagaggcaatactc	-	-
CY160428	Human H3N2 IAVs	Human	H3N2		2012	Peru	A/Peru/PER018/2012	agtgatgccccattccttgatcggcttcgccgagatcag aggtccctaaggggaagaggcaatactc	-	-
CY161068	Human H3N2 IAVs	Human	H3N2		2012	Peru	A/Peru/PER099/2012	agtgatgccccattccttgatcggcttcgccgagatcag aggtccctaaggggaagaggcaatactc	-	-
CY162740	Human H3N2 IAVs	Human	H3N2		2012	Peru	A/Peru/PER314/2012	agtgatgccccattccttgatcggcttcgccgagatcag aggtccctaaggggaagaggcaatactc	-	-
CY163068	Human H3N2 IAVs	Human	H3N2		2012	Peru	A/Peru/PER356/2012	agtgatgccccattccttgatcggcttcgccgagatcag aggtccctaaggggaagaggcaatactc	-	-
CY163404	Human H3N2 IAVs	Human	H3N2		2012	Peru	A/Peru/PER400/2012	agtgatgccccattccttgatcggcttcgccgagatcag aggtccctaaggggaagaggcaatactc	-	-
CY160932	Human H3N2 IAVs	Human	H3N2		2012	Peru	A/Peru/PER081/2012	agtgatgccccattccttgatcggcttcgccgagatcag aggtccctaaggggaagaggcaatactc	-	-
CY161420	Human H3N2 IAVs	Human	H3N2		2012	Peru	A/Peru/PER144/2012	agtgatgccccattccttgatcggcttcgccgagatcag aggtccctaaggggaagaggcaatactc	-	-
CY163396	Human H3N2 IAVs	Human	H3N2		2012	Peru	A/Peru/PER399/2012	agtgatgccccattccttgatcggcttcgccgagatcag aggtccctaaggggaagaggcaatactc	-	-
CY160908	Human H3N2 IAVs	Human	H3N2		2012	Peru	A/Peru/PER078/2012	agtgatgccccattccttgatcggcttcgccgagatcag aggtccctaaggggaagaggcaatactc	-	-
CY162156	Human H3N2 IAVs	Human	H3N2		2012	Peru	A/Peru/PER236/2012	agtgatgccccattccttgatcggcttcgccgagatcag aggtccctaaggggaagaggcaatactc	-	-
CY161116	Human H3N2 IAVs	Human	H3N2		2012	Peru	A/Peru/PER105/2012	agtgatgccccattccttgatcggcttcgccgagatcag aggtccctaaggggaagaggcaatactc	-	-
CY163316	Human H3N2 IAVs	Human	H3N2		2012	Peru	A/Peru/PER389/2012	agtgatgccccattccttgatcggcttcgccgagatcag aggtccctaaggggaagaggcaatactc	-	-
CY161044	Human H3N2 IAVs	Human	H3N2		2012	Peru	A/Peru/PER096/2012	agtgatgccccattccttgatcggcttcgccgagatcag aggtccctaaggggaagaggcaatactc	-	-

KJ943252	Human H3N2 IAVs	Human	H3N2		2012	Chile	A/Santiago/op17d0/2012	agtgatgccccattccttgatcggcttcgccgagatcag aggtccctaaggggaagaggcaatactc	-	-
KJ942923	Human H3N2 IAVs	Human	H3N2		2012	Chile	A/Santiago/op5d1/2012	agtgatgccccattccttgatcggcttcgccgagatcag aggtccctaaggggaagaggcaatactc	-	-
CY182245	Human H3N2 IAVs	Human	H3N2		2012	Nicaragua	A/Nicaragua/AGA2_97/2012	agtgatgccccattccttgatcggcttcgccgagatcag aggtccctaaggggaagaggcaatactc	-	-
KF928629	Human H3N2 IAVs	Human	H3N2		2012	USA	A/South_Carolina/NHRC382368/2012	agtgatgccccattccttgatcggcttcgccgagatcag aggtccctaaggggaagaggcaatactc	-	-
CY135088	Human H3N2 IAVs	Human	H3N2		2012	USA	A/Texas/JMM_49/2012	agtgatgccccattccttgatcggcttcgccgagatcag aggtccctaaggggaagaggcaatactc	-	-
CY182925	Human H3N2 IAVs	Human	H3N2		2012	USA	A/Houston/JMM_92/2012	agtgatgccccattccttgatcggcttcgccgagatcag aggtccctaaggggaagaggcaatactc	-	-
CY167987	Human H3N2 IAVs	Human	H3N2		2012	USA	A/Boston/YGA_00016/2012	agtgatgccccattccttgatcggcttcgccgagatcag aggtccctaaggggaagaggcaatactc	-	-
KJ943646	Human H3N2 IAVs	Human	H3N2		2012	Chile	A/Santiago/p9d0/2012	agtgatgccccattccttgatcggcttcgccgagatcag aggtccctaaggggaagaggcaatactc	-	-
KJ943593	Human H3N2 IAVs	Human	H3N2		2012	Chile	A/Santiago/p9d1/2012	agtgatgccccattccttgatcggcttcgccgagatcag aggtccctaaggggaagaggcaatactc	-	-
KJ943668	Human H3N2 IAVs	Human	H3N2		2012	Chile	A/Santiago/p9d2/2012	agtgatgccccattccttgatcggcttcgccgagatcag aggtccctaaggggaagaggcaatactc	-	-
KJ943585	Human H3N2 IAVs	Human	H3N2		2012	Chile	A/Santiago/p9d3/2012	agtgatgccccattccttgatcggcttcgccgagatcag aggtccctaaggggaagaggcaatactc	-	-
KJ943703	Human H3N2 IAVs	Human	H3N2		2012	Chile	A/Santiago/p9d5/2012	agtgatgccccattccttgatcggcttcgccgagatcag aggtccctaaggggaagaggcaatactc	-	-
CY169147	Human H3N2 IAVs	Human	H3N2		2012	USA	A/Boston/YGA_01086/2012	agtgatgccccattccttgatcggcttcgccgagatcag aggtccctaaggggaagaggcaatactc	-	-
CY161140	Human H3N2 IAVs	Human	H3N2		2012	Peru	A/Peru/PER108/2012	agtgatgccccattccttgatcggcttcgccgagatcag aggtccctaaggggaagaggcaatactc	-	-
CY125795	Human H3N2 IAVs	Human	H3N2		2012	USA	A/Boston/DOA94/2012	agtgatgccccattccttgatcggcttcgccgagatcag aggtccctacggggaagaggcaatactc	-	-
CY161076	Human H3N2 IAVs	Human	H3N2		2012	Peru	A/Peru/PER100/2012	agtgatgccccattccttgatcggcttcgccgagatcag aggtccttaaggggaagaggcaatactc	+	+
CY162124	Human H3N2 IAVs	Human	H3N2		2012	Peru	A/Peru/PER232/2012	agtgatgccccattccttgatcggcttcgccgagatcag aggtccttaaggggaagaggcaatactc	+	+
CY162876	Human H3N2 IAVs	Human	H3N2		2012	Peru	A/Peru/PER331/2012	agtgatgccccattccttgatcggcttcgccgagatcag aggtccttaaggggaagaggcaatactc	+	+
CY169163	Human H3N2 IAVs	Human	H3N2		2012	USA	A/Boston/YGA_01089/2012	agtgatgccccattccttgatcggcttcgccgatatcag aggtccctaaggggaagaggcaatactc	-	-
KJ943622	Human H3N2 IAVs	Human	H3N2		2012	Chile	A/Santiago/p24d1/2012	agtgatgccccattccttgatcggcttcgccgggatcag aggtccctaaggggaagaggcagtactc	-	-
KJ943561	Human H3N2 IAVs	Human	H3N2		2012	Chile	A/Santiago/p24d2/2012	agtgatgccccattccttgatcggcttcgccgggatcag aggtccctaaggggaagaggcagtactc	-	-
KJ943654	Human H3N2 IAVs	Human	H3N2		2012	Chile	A/Santiago/p24d5/2012	agtgatgccccattccttgatcggcttcgccgggatcag aggtccctaaggggaagaggcagtactc	-	-
JX913083	Human H3N2 IAVs	Human	H3N2		2012	Czech_Republic	A/Czech_Republic/138/2012	agtgatgccccattccttgatcggcttcgtcgagatcag aggtccctaaggggaagaggcaatactc	+	+
KF612210	Human H3N2 IAVs	Human	H3N2		2012	Bolivia	A/Santa_Cruz/2821/2012	agtgatgccccatttcttgatcggcttcgccgagatcag aggtccctaaggggaagaggcaatactc	-	-
CY182733	Human H3N2 IAVs	Human	H3N2		2012	USA	A/Houston/JMM_66/2012	agtgatgccccatttcttgatcggcttcgccgagatcag aggtccctaaggggaagaggcaatactc	-	-

CY182757	Human H3N2 IAVs	Human	H3N2		2012	USA	A/Houston/JMM_69/2012	agtgatgccccatttcttgatcggcttcgccgagatcag<br>aggtccctaaggggaagaggcaatactc	-	-
CY134736	Human H3N2 IAVs	Human	H3N2		2012	USA	A/Texas/JMM_1/2012	agtgatgccccatttcttgatcggcttcgccgagatcag<br>aggtccctaaggggaagaggcaatactc	-	-
CY134752	Human H3N2 IAVs	Human	H3N2		2012	USA	A/Texas/JMM_3/2012	agtgatgccccatttcttgatcggcttcgccgagatcag<br>aggtccctaaggggaagaggcaatactc	-	-
CY134944	Human H3N2 IAVs	Human	H3N2		2012	USA	A/Texas/JMM_30/2012	agtgatgccccatttcttgatcggcttcgccgagatcag<br>aggtccctaaggggaagaggcaatactc	-	-
CY134984	Human H3N2 IAVs	Human	H3N2		2012	USA	A/Texas/JMM_35/2012	agtgatgccccatttcttgatcggcttcgccgagatcag<br>aggtccctaaggggaagaggcaatactc	-	-
CY134776	Human H3N2 IAVs	Human	H3N2		2012	USA	A/Texas/JMM_8/2012	agtgatgccccatttcttgatcggcttcgccgagatcag<br>aggtccctaaggggaagaggcaatactc	-	-
CY170875	Human H3N2 IAVs	Human	H3N2		2012	USA	A/Santa_Clara/YGA_03057/2012	agtgatgtcccattccttgatcggcttcgccgagatcag<br>aggtccctaaggggaagaggcaatactc	+	+
CY171315	Human H3N2 IAVs	Human	H3N2		2012	USA	A/Chicago/YGA_04085/2012	agtgatgtcccattccttgatcggcttcgccgagatcag<br>aggtccctaaggggaagaggcaatactc	+	+
CY171323	Human H3N2 IAVs	Human	H3N2		2012	USA	A/Chicago/YGA_04086/2012	agtgatgtcccattccttgatcggcttcgccgagatcag<br>aggtccctaaggggaagaggcaatactc	+	+
CY161996	Human H3N2 IAVs	Human	H3N2		2012	Peru	A/Peru/PER216/2012	agtgattccccattccttgatcggcttcgccgagatcag<br>aggtccctaagggggagaggcaatactc	-	-
CY168155	Human H3N2 IAVs	Human	H3N2		2013	USA	A/Boston/YGA_00044/2013	aatgatgccccattccttgatcggcttcgccgagatca<br>gaggtccctaaggggaagaggcaatactc	-	-
CY148856	Human H3N2 IAVs	Human	H3N2		2013	USA	A/Boston/DOA2_172/2013	agcgatgccccattcctcgatcggcttcgccgagatca<br>gaggtccctaaggggaagaggcaatactc	-	-
CY169763	Human H3N2 IAVs	Human	H3N2		2013	USA	A/Boston/YGA_01169/2013	agcgatgccccattcctcgatcggcttcgccgagatca<br>gaggtccctaaggggaagaggcaatactc	-	-
CY170227	Human H3N2 IAVs	Human	H3N2		2013	USA	A/Boston/YGA_02019/2013	agcgatgccccattcctcgatcggcttcgccgagatca<br>gaggtccctaaggggaagaggcaatactc	-	-
CY170267	Human H3N2 IAVs	Human	H3N2		2013	USA	A/Boston/YGA_02025/2013	agcgatgccccattcctcgatcggcttcgccgagatca<br>gaggtccctaaggggaagaggcaatactc	-	-
CY186199	Human H3N2 IAVs	Human	H3N2		2013	USA	A/Houston/JMM_172/2013	agcgatgccccattcctcgatcggcttcgccgagatca<br>gaggtccctaaggggaagaggcaatactc	-	-
CY170755	Human H3N2 IAVs	Human	H3N2		2013	USA	A/Santa_Clara/YGA_03040/2013	agcgatgccccattcctcgatcggcttcgccgagatca<br>gaggtccctaaggggaagaggcaatactc	-	-
CY170851	Human H3N2 IAVs	Human	H3N2		2013	USA	A/Santa_Clara/YGA_03054/2013	agcgatgccccattcctcgatcggcttcgccgagatca<br>gaggtccctaaggggaagaggcaatactc	-	-
CY168283	Human H3N2 IAVs	Human	H3N2		2013	USA	A/Boston/YGA_00062/2013	agcgatgccccattcctcgatcggcttcgccgagatca<br>gaggtctctaaggggaagaggcaatactc	-	-
KM070131	Human H3N2 IAVs	Human	H3N2		2013	Singapore	A/Singapore/H2013.430a/2013	agcgatgccccattccttgatcggcttcgccgagatca<br>gaggtccctaaggggaagaggcaatactc	-	-
CY170075	Human H3N2 IAVs	Human	H3N2		2013	USA	A/Boston/YGA_01216/2013	agtgatgccccattcctcgatcggcttcaccgagatca<br>gaggtccctaaggggaagaggcaatactc	-	-
CY170611	Human H3N2 IAVs	Human	H3N2		2013	USA	A/Santa_Clara/YGA_03021/2013	agtgatgccccattcctcgatcggcttcgccgagatca<br>gaggtccctaaggggaagaggcaatactc	-	-
CY186127	Human H3N2 IAVs	Human	H3N2		2013	USA	A/Houston/JMM_162/2013	agtgatgccccattccttgatcgacttcgccgagatca<br>gaggtccctaaggggaagaggcaatactc	-	-
KF805644	Human H3N2 IAVs	Human	H3N2		2013	Finland	A/Helsinki/798/2013	agtgatgccccattccttgatcggctacgccgagatca<br>aaagtccttaaaaggaagaggcaacaccc	-	-
KF805652	Human H3N2 IAVs	Human	H3N2		2013	Finland	A/Helsinki/823/2013	agtgatgccccattccttgatcggctacgccgagatca<br>aaagtccttaaaaggaagaggcaacaccc	-	-

KF805676	Human H3N2 IAVs	Human	H3N2		2013	Finland	A/Helsinki/897/2013	agtgatgccccattccttgatcggctccgccgagatca<br>aaagtccttaaaaggaagaggcaacaccc	-	*
KF805636	Human H3N2 IAVs	Human	H3N2		2013	Finland	A/Helsinki/716/2013	agtgatgccccattccttgatcggctccgccgagatca<br>aaggtccctaaggggaagaggcaacaccc	-	*
KF805684	Human H3N2 IAVs	Human	H3N2		2013	Finland	A/Helsinki/941/2013	agtgatgccccattccttgatcggcttcgccgagatca<br>aaagtccctaaggggaagaggcaacaccc	-	-
CY169659	Human H3N2 IAVs	Human	H3N2		2013	USA	A/Boston/YGA_01156/2013	agtgatgccccattccttgatcggcttcgccgagatcag<br>aggtcactaaggggaagaggcaatactc	-	-
CY170787	Human H3N2 IAVs	Human	H3N2		2013	USA	A/Santa_Clara/YGA_03045/2013	agtgatgccccattccttgatcggcttcgccgagatcag<br>aggtccctaaagggaagaggcaatactc	+	+
CY168171	Human H3N2 IAVs	Human	H3N2		2013	USA	A/Boston/YGA_00046/2013	agtgatgccccattccttgatcggcttcgccgagatcag<br>aggtccctaagaggaagaggcaatactc	+	+
CY148696	Human H3N2 IAVs	Human	H3N2		2013	USA	A/Boston/DOA2_148/2013	agtgatgccccattccttgatcggcttcgccgagatcag<br>aggtccctaaggagaagaggcaatactc	-	*
KF805708	Human H3N2 IAVs	Human	H3N2		2013	Finland	A/Helsinki/991/2013	agtgatgccccattccttgatcggcttcgccgagatcag<br>aggtccctaaggggaagaggcaacactc	-	-
CY183069	Human H3N2 IAVs	Human	H3N2		2013	USA	A/Houston/JMM_111/2013	agtgatgccccattccttgatcggcttcgccgagatcag<br>aggtccctaaggggaagaggcaatactc	-	-
CY170435	Human H3N2 IAVs	Human	H3N2		2013	USA	A/Boston/YGA_02055/2013	agtgatgccccattccttgatcggcttcgccgagatcag<br>aggtccctaaggggaagaggcaatactc	-	-
CY183029	Human H3N2 IAVs	Human	H3N2		2013	USA	A/Houston/JMM_106/2013	agtgatgccccattccttgatcggcttcgccgagatcag<br>aggtccctaaggggaagaggcaatactc	-	-
CY168411	Human H3N2 IAVs	Human	H3N2		2013	USA	A/Boston/YGA_00084/2013	agtgatgccccattccttgatcggcttcgccgagatcag<br>aggtccctaaggggaagaggcaatactc	-	-
CY183013	Human H3N2 IAVs	Human	H3N2		2013	USA	A/Houston/JMM_104/2013	agtgatgccccattccttgatcggcttcgccgagatcag<br>aggtccctaaggggaagaggcaatactc	-	-
CY183061	Human H3N2 IAVs	Human	H3N2		2013	USA	A/Houston/JMM_110/2013	agtgatgccccattccttgatcggcttcgccgagatcag<br>aggtccctaaggggaagaggcaatactc	-	-
CY183141	Human H3N2 IAVs	Human	H3N2		2013	USA	A/Houston/JMM_121/2013	agtgatgccccattccttgatcggcttcgccgagatcag<br>aggtccctaaggggaagaggcaatactc	-	-
KM064432	Human H3N2 IAVs	Human	H3N2		2013	USA	A/Arkansas/13/2013	agtgatgccccattccttgatcggcttcgccgagatcag<br>aggtccctaaggggaagaggcaatactc	-	-
CY183317	Human H3N2 IAVs	Human	H3N2		2013	USA	A/Houston/JMM_146/2013	agtgatgccccattccttgatcggcttcgccgagatcag<br>aggtccctaaggggaagaggcaatactc	-	-
CY170491	Human H3N2 IAVs	Human	H3N2		2013	USA	A/Santa_Clara/YGA_03006/2013	agtgatgccccattccttgatcggcttcgccgagatcag<br>aggtccctaaggggaagaggcaatactc	-	-
KM070132	Human H3N2 IAVs	Human	H3N2		2013	Singapore	A/Singapore/H2013.430b/2013	agtgatgccccattccttgatcggcttcgccgagatcag<br>aggtccctaaggggaagaggcaatactc	-	-
CY148712	Human H3N2 IAVs	Human	H3N2		2013	USA	A/Boston/DOA2_150/2013	agtgatgccccattccttgatcggcttcgccgagatcag<br>aggtccctaaggggaagaggcaatactc	-	-
CY148888	Human H3N2 IAVs	Human	H3N2		2013	USA	A/Boston/DOA2_176/2013	agtgatgccccattccttgatcggcttcgccgagatcag<br>aggtccctaaggggaagaggcaatactc	-	-
CY148912	Human H3N2 IAVs	Human	H3N2		2013	USA	A/Boston/DOA2_179/2013	agtgatgccccattccttgatcggcttcgccgagatcag<br>aggtccctaaggggaagaggcaatactc	-	-
CY148920	Human H3N2 IAVs	Human	H3N2		2013	USA	A/Boston/DOA2_180/2013	agtgatgccccattccttgatcggcttcgccgagatcag<br>aggtccctaaggggaagaggcaatactc	-	-
CY148928	Human H3N2 IAVs	Human	H3N2		2013	USA	A/Boston/DOA2_181/2013	agtgatgccccattccttgatcggcttcgccgagatcag<br>aggtccctaaggggaagaggcaatactc	-	-
CY148984	Human H3N2 IAVs	Human	H3N2		2013	USA	A/Boston/DOA2_189/2013	agtgatgccccattccttgatcggcttcgccgagatcag<br>aggtccctaaggggaagaggcaatactc	-	-

CY148279	Human H3N2 IAVs	Human	H3N2		2013	USA	A/Boston/DOA2_193/2013	agtgatgccccattccttgatcggcttcgccgagatcagaggtccctaaggggaagaggcaatactc	-	-
CY149040	Human H3N2 IAVs	Human	H3N2		2013	USA	A/Boston/DOA2_201/2013	agtgatgccccattccttgatcggcttcgccgagatcagaggtccctaaggggaagaggcaatactc	-	-
CY149112	Human H3N2 IAVs	Human	H3N2		2013	USA	A/Boston/DOA2_215/2013	agtgatgccccattccttgatcggcttcgccgagatcagaggtccctaaggggaagaggcaatactc	-	-
CY149192	Human H3N2 IAVs	Human	H3N2		2013	USA	A/Boston/DOA2_226/2013	agtgatgccccattccttgatcggcttcgccgagatcagaggtccctaaggggaagaggcaatactc	-	-
CY149200	Human H3N2 IAVs	Human	H3N2		2013	USA	A/Boston/DOA2_227/2013	agtgatgccccattccttgatcggcttcgccgagatcagaggtccctaaggggaagaggcaatactc	-	-
CY149224	Human H3N2 IAVs	Human	H3N2		2013	USA	A/Boston/DOA2_230/2013	agtgatgccccattccttgatcggcttcgccgagatcagaggtccctaaggggaagaggcaatactc	-	-
CY149256	Human H3N2 IAVs	Human	H3N2		2013	USA	A/Boston/DOA2_236/2013	agtgatgccccattccttgatcggcttcgccgagatcagaggtccctaaggggaagaggcaatactc	-	-
CY149320	Human H3N2 IAVs	Human	H3N2		2013	USA	A/Boston/DOA2_245/2013	agtgatgccccattccttgatcggcttcgccgagatcagaggtccctaaggggaagaggcaatactc	-	-
CY168075	Human H3N2 IAVs	Human	H3N2		2013	USA	A/Boston/YGA_00030/2013	agtgatgccccattccttgatcggcttcgccgagatcagaggtccctaaggggaagaggcaatactc	-	-
CY168091	Human H3N2 IAVs	Human	H3N2		2013	USA	A/Boston/YGA_00035/2013	agtgatgccccattccttgatcggcttcgccgagatcagaggtccctaaggggaagaggcaatactc	-	-
CY168179	Human H3N2 IAVs	Human	H3N2		2013	USA	A/Boston/YGA_00047/2013	agtgatgccccattccttgatcggcttcgccgagatcagaggtccctaaggggaagaggcaatactc	-	-
CY168187	Human H3N2 IAVs	Human	H3N2		2013	USA	A/Boston/YGA_00048/2013	agtgatgccccattccttgatcggcttcgccgagatcagaggtccctaaggggaagaggcaatactc	-	-
CY168275	Human H3N2 IAVs	Human	H3N2		2013	USA	A/Boston/YGA_00061/2013	agtgatgccccattccttgatcggcttcgccgagatcagaggtccctaaggggaagaggcaatactc	-	-
CY168379	Human H3N2 IAVs	Human	H3N2		2013	USA	A/Boston/YGA_00080/2013	agtgatgccccattccttgatcggcttcgccgagatcagaggtccctaaggggaagaggcaatactc	-	-
CY168387	Human H3N2 IAVs	Human	H3N2		2013	USA	A/Boston/YGA_00081/2013	agtgatgccccattccttgatcggcttcgccgagatcagaggtccctaaggggaagaggcaatactc	-	-
CY169347	Human H3N2 IAVs	Human	H3N2		2013	USA	A/Boston/YGA_01113/2013	agtgatgccccattccttgatcggcttcgccgagatcagaggtccctaaggggaagaggcaatactc	-	-
CY167808	Human H3N2 IAVs	Human	H3N2		2013	USA	A/Boston/YGA_01116/2013	agtgatgccccattccttgatcggcttcgccgagatcagaggtccctaaggggaagaggcaatactc	-	-
CY169395	Human H3N2 IAVs	Human	H3N2		2013	USA	A/Boston/YGA_01122/2013	agtgatgccccattccttgatcggcttcgccgagatcagaggtccctaaggggaagaggcaatactc	-	-
CY169403	Human H3N2 IAVs	Human	H3N2		2013	USA	A/Boston/YGA_01123/2013	agtgatgccccattccttgatcggcttcgccgagatcagaggtccctaaggggaagaggcaatactc	-	-
CY169435	Human H3N2 IAVs	Human	H3N2		2013	USA	A/Boston/YGA_01127/2013	agtgatgccccattccttgatcggcttcgccgagatcagaggtccctaaggggaagaggcaatactc	-	-
CY169443	Human H3N2 IAVs	Human	H3N2		2013	USA	A/Boston/YGA_01128/2013	agtgatgccccattccttgatcggcttcgccgagatcagaggtccctaaggggaagaggcaatactc	-	-
CY169603	Human H3N2 IAVs	Human	H3N2		2013	USA	A/Boston/YGA_01149/2013	agtgatgccccattccttgatcggcttcgccgagatcagaggtccctaaggggaagaggcaatactc	-	-
CY169683	Human H3N2 IAVs	Human	H3N2		2013	USA	A/Boston/YGA_01159/2013	agtgatgccccattccttgatcggcttcgccgagatcagaggtccctaaggggaagaggcaatactc	-	-
CY169707	Human H3N2 IAVs	Human	H3N2		2013	USA	A/Boston/YGA_01162/2013	agtgatgccccattccttgatcggcttcgccgagatcagaggtccctaaggggaagaggcaatactc	-	-
CY169723	Human H3N2 IAVs	Human	H3N2		2013	USA	A/Boston/YGA_01164/2013	agtgatgccccattccttgatcggcttcgccgagatcagaggtccctaaggggaagaggcaatactc	-	-

CY169779	Human H3N2 IAVs	Human	H3N2		2013	USA	A/Boston/YGA_01171/2013	agtgatgccccattccttgatcggcttcgccgagatcag aggtccctaaggggaagaggcaatactc	-	-
CY169819	Human H3N2 IAVs	Human	H3N2		2013	USA	A/Boston/YGA_01176/2013	agtgatgccccattccttgatcggcttcgccgagatcag aggtccctaaggggaagaggcaatactc	-	-
CY170323	Human H3N2 IAVs	Human	H3N2		2013	USA	A/Boston/YGA_02035/2013	agtgatgccccattccttgatcggcttcgccgagatcag aggtccctaaggggaagaggcaatactc	-	-
CY170419	Human H3N2 IAVs	Human	H3N2		2013	USA	A/Boston/YGA_02053/2013	agtgatgccccattccttgatcggcttcgccgagatcag aggtccctaaggggaagaggcaatactc	-	-
CY188301	Human H3N2 IAVs	Human	H3N2		2013	Nicaragua	A/Nicaragua/3360_03/2013	agtgatgccccattccttgatcggcttcgccgagatcag aggtccctaaggggaagaggcaatactc	-	-
CY187909	Human H3N2 IAVs	Human	H3N2		2013	Nicaragua	A/Nicaragua/3442_07/2013	agtgatgccccattccttgatcggcttcgccgagatcag aggtccctaaggggaagaggcaatactc	-	-
CY188125	Human H3N2 IAVs	Human	H3N2		2013	Nicaragua	A/Nicaragua/4236_02/2013	agtgatgccccattccttgatcggcttcgccgagatcag aggtccctaaggggaagaggcaatactc	-	-
CY187877	Human H3N2 IAVs	Human	H3N2		2013	Nicaragua	A/Nicaragua/4430_02/2013	agtgatgccccattccttgatcggcttcgccgagatcag aggtccctaaggggaagaggcaatactc	-	-
CY188085	Human H3N2 IAVs	Human	H3N2		2013	Nicaragua	A/Nicaragua/4860_12/2013	agtgatgccccattccttgatcggcttcgccgagatcag aggtccctaaggggaagaggcaatactc	-	-
CY188021	Human H3N2 IAVs	Human	H3N2		2013	Nicaragua	A/Nicaragua/4871_07/2013	agtgatgccccattccttgatcggcttcgccgagatcag aggtccctaaggggaagaggcaatactc	-	-
CY188149	Human H3N2 IAVs	Human	H3N2		2013	Nicaragua	A/Nicaragua/5026_03/2013	agtgatgccccattccttgatcggcttcgccgagatcag aggtccctaaggggaagaggcaatactc	-	-
CY187941	Human H3N2 IAVs	Human	H3N2		2013	Nicaragua	A/Nicaragua/5300_01/2013	agtgatgccccattccttgatcggcttcgccgagatcag aggtccctaaggggaagaggcaatactc	-	-
CY188061	Human H3N2 IAVs	Human	H3N2		2013	Nicaragua	A/Nicaragua/5805_08/2013	agtgatgccccattccttgatcggcttcgccgagatcag aggtccctaaggggaagaggcaatactc	-	-
CY188045	Human H3N2 IAVs	Human	H3N2		2013	Nicaragua	A/Nicaragua/6104_06/2013	agtgatgccccattccttgatcggcttcgccgagatcag aggtccctaaggggaagaggcaatactc	-	-
CY187925	Human H3N2 IAVs	Human	H3N2		2013	Nicaragua	A/Nicaragua/6201_05/2013	agtgatgccccattccttgatcggcttcgccgagatcag aggtccctaaggggaagaggcaatactc	-	-
CY188141	Human H3N2 IAVs	Human	H3N2		2013	Nicaragua	A/Nicaragua/6480_07/2013	agtgatgccccattccttgatcggcttcgccgagatcag aggtccctaaggggaagaggcaatactc	-	-
CY187981	Human H3N2 IAVs	Human	H3N2		2013	Nicaragua	A/Nicaragua/6496_12/2013	agtgatgccccattccttgatcggcttcgccgagatcag aggtccctaaggggaagaggcaatactc	-	-
CY188405	Human H3N2 IAVs	Human	H3N2		2013	Nicaragua	A/Nicaragua/6854_05/2013	agtgatgccccattccttgatcggcttcgccgagatcag aggtccctaaggggaagaggcaatactc	-	-
CY188133	Human H3N2 IAVs	Human	H3N2		2013	Nicaragua	A/Nicaragua/6890_09/2013	agtgatgccccattccttgatcggcttcgccgagatcag aggtccctaaggggaagaggcaatactc	-	-
CY188029	Human H3N2 IAVs	Human	H3N2		2013	Nicaragua	A/Nicaragua/6958_04/2013	agtgatgccccattccttgatcggcttcgccgagatcag aggtccctaaggggaagaggcaatactc	-	-
CY187973	Human H3N2 IAVs	Human	H3N2		2013	Nicaragua	A/Nicaragua/6965_03/2013	agtgatgccccattccttgatcggcttcgccgagatcag aggtccctaaggggaagaggcaatactc	-	-
CY188181	Human H3N2 IAVs	Human	H3N2		2013	Nicaragua	A/Nicaragua/7607_03/2013	agtgatgccccattccttgatcggcttcgccgagatcag aggtccctaaggggaagaggcaatactc	-	-
CY188333	Human H3N2 IAVs	Human	H3N2		2013	Nicaragua	A/Nicaragua/7637_03/2013	agtgatgccccattccttgatcggcttcgccgagatcag aggtccctaaggggaagaggcaatactc	-	-
CY188277	Human H3N2 IAVs	Human	H3N2		2013	Nicaragua	A/Nicaragua/7655_01/2013	agtgatgccccattccttgatcggcttcgccgagatcag aggtccctaaggggaagaggcaatactc	-	-
CY182341	Human H3N2 IAVs	Human	H3N2		2013	Nicaragua	A/Nicaragua/AGA2_112/2013	agtgatgccccattccttgatcggcttcgccgagatcag aggtccctaaggggaagaggcaatactc	-	-

CY182349	Human H3N2 IAVs	Human	H3N2		2013	Nicaragua	A/Nicaragua/AGA2_113/2013	agtgatgccccattccttgatcggcttcgccgagatcagaggtccctaaggggaagaggcaatactc	-	-
CY182357	Human H3N2 IAVs	Human	H3N2		2013	Nicaragua	A/Nicaragua/AGA2_114/2013	agtgatgccccattccttgatcggcttcgccgagatcagaggtccctaaggggaagaggcaatactc	-	-
CY170603	Human H3N2 IAVs	Human	H3N2		2013	USA	A/Santa_Clara/YGA_03020/2013	agtgatgccccattccttgatcggcttcgccgagatcagaggtccctaaggggaagaggcaatactc	-	-
CY170747	Human H3N2 IAVs	Human	H3N2		2013	USA	A/Santa_Clara/YGA_03039/2013	agtgatgccccattccttgatcggcttcgccgagatcagaggtccctaaggggaagaggcaatactc	-	-
CY149144	Human H3N2 IAVs	Human	H3N2		2013	USA	A/Boston/DOA2_220/2013	agtgatgccccattccttgatcggcttcgccgagatcagaggtccctaaggggaagaggcaatactc	-	-
CY169547	Human H3N2 IAVs	Human	H3N2		2013	USA	A/Boston/YGA_01142/2013	agtgatgccccattccttgatcggcttcgccgagatcagaggtccctaaggggaagaggcaatactc	-	-
CY169651	Human H3N2 IAVs	Human	H3N2		2013	USA	A/Boston/YGA_01155/2013	agtgatgccccattccttgatcggcttcgccgagatcagaggtccctaaggggaagaggcaatactc	-	-
CY183301	Human H3N2 IAVs	Human	H3N2		2013	USA	A/Houston/JMM_144/2013	agtgatgccccattccttgatcggcttcgccgagatcagaggtccctaaggggaagaggcaatactc	-	-
CY149208	Human H3N2 IAVs	Human	H3N2		2013	USA	A/Boston/DOA2_228/2013	agtgatgccccattccttgatcggcttcgccgagatcagaggtccctaaggggaagaggcaatactc	-	-
CY149248	Human H3N2 IAVs	Human	H3N2		2013	USA	A/Boston/DOA2_235/2013	agtgatgccccattccttgatcggcttcgccgagatcagaggtccctaaggggaagaggcaatactc	-	-
CY168035	Human H3N2 IAVs	Human	H3N2		2013	USA	A/Boston/YGA_00024/2013	agtgatgccccattccttgatcggcttcgccgagatcagaggtccctaaggggaagaggcaatactc	-	-
CY149232	Human H3N2 IAVs	Human	H3N2		2013	USA	A/Boston/DOA2_232/2013	agtgatgccccattccttgatcggcttcgccgagatcagaggtccctaaggggaagaggcaatactc	-	-
CY168195	Human H3N2 IAVs	Human	H3N2		2013	USA	A/Boston/YGA_00049/2013	agtgatgccccattccttgatcggcttcgccgagatcagaggtccctaaggggaagaggcaatactc	-	-
CY169475	Human H3N2 IAVs	Human	H3N2		2013	USA	A/Boston/YGA_01132/2013	agtgatgccccattccttgatcggcttcgccgagatcagaggtccctaaggggaagaggcaatactc	-	-
CY169675	Human H3N2 IAVs	Human	H3N2		2013	USA	A/Boston/YGA_01158/2013	agtgatgccccattccttgatcggcttcgccgagatcagaggtccctaaggggaagaggcaatactc	-	-
CY183021	Human H3N2 IAVs	Human	H3N2		2013	USA	A/Houston/JMM_105/2013	agtgatgccccattccttgatcggcttcgccgagatcagaggtccctaaggggaagaggcaatactc	-	-
CY188093	Human H3N2 IAVs	Human	H3N2		2013	Nicaragua	A/Nicaragua/5349_05/2013	agtgatgccccattccttgatcggcttcgccgagatcagaggtccctaaggggaagaggcaatactc	-	-
CY183077	Human H3N2 IAVs	Human	H3N2		2013	USA	A/Houston/JMM_112/2013	agtgatgccccattccttgatcggcttcgccgagatcagaggtccctaaggggaagaggcaatactc	-	-
CY187949	Human H3N2 IAVs	Human	H3N2		2013	Nicaragua	A/Nicaragua/6426_03/2013	agtgatgccccattccttgatcggcttcgccgagatcagaggtccctaaggggaagaggcaatactc	-	-
CY169715	Human H3N2 IAVs	Human	H3N2		2013	USA	A/Boston/YGA_01163/2013	agtgatgccccattccttgatcggcttcgccgagatcagaggtccctaaggggaagaggcaatactc	-	-
CY148760	Human H3N2 IAVs	Human	H3N2		2013	USA	A/Boston/DOA2_157/2013	agtgatgccccattccttgatcggcttcgccgagatcagaggtccctaaggggaagaggcaatactc	-	-
CY148952	Human H3N2 IAVs	Human	H3N2		2013	USA	A/Boston/DOA2_184/2013	agtgatgccccattccttgatcggcttcgccgagatcagaggtccctaaggggaagaggcaatactc	-	-
CY148976	Human H3N2 IAVs	Human	H3N2		2013	USA	A/Boston/DOA2_187/2013	agtgatgccccattccttgatcggcttcgccgagatcagaggtccctaaggggaagaggcaatactc	-	-
CY169947	Human H3N2 IAVs	Human	H3N2		2013	USA	A/Boston/YGA_01198/2013	agtgatgccccattccttgatcggcttcgccgagatcagaggtccctaaggggaagaggcaatactc	-	-
CY188381	Human H3N2 IAVs	Human	H3N2		2013	Nicaragua	A/Nicaragua/6249_03/2013	agtgatgccccattccttgatcggcttcgccgagatcagaggtccctaaggggaagaggcaatactc	-	-

CY188349	Human H3N2 IAVs	Human	H3N2		2013	Nicaragua	A/Nicaragua/5605_09/2013	agtgatgccccattccttgatcggcttcgccgagatcag aggtccctaaggggaagaggcaatactc	-	-
CY188309	Human H3N2 IAVs	Human	H3N2		2013	Nicaragua	A/Nicaragua/6397_14/2013	agtgatgccccattccttgatcggcttcgccgagatcag aggtccctaaggggaagaggcaatactc	-	-
CY187989	Human H3N2 IAVs	Human	H3N2		2013	Nicaragua	A/Nicaragua/6823_15/2013	agtgatgccccattccttgatcggcttcgccgagatcag aggtccctaaggggaagaggcaatactc	-	-
CY188037	Human H3N2 IAVs	Human	H3N2		2013	Nicaragua	A/Nicaragua/6889_07/2013	agtgatgccccattccttgatcggcttcgccgagatcag aggtccctaaggggaagaggcaatactc	-	-
CY188325	Human H3N2 IAVs	Human	H3N2		2013	Nicaragua	A/Nicaragua/6971_03/2013	agtgatgccccattccttgatcggcttcgccgagatcag aggtccctaaggggaagaggcaatactc	-	-
CY187933	Human H3N2 IAVs	Human	H3N2		2013	Nicaragua	A/Nicaragua/7525_05/2013	agtgatgccccattccttgatcggcttcgccgagatcag aggtccctaaggggaagaggcaatactc	-	-
CY187997	Human H3N2 IAVs	Human	H3N2		2013	Nicaragua	A/Nicaragua/7622_01/2013	agtgatgccccattccttgatcggcttcgccgagatcag aggtccctaaggggaagaggcaatactc	-	-
CY187957	Human H3N2 IAVs	Human	H3N2		2013	Nicaragua	A/Nicaragua/7699_01/2013	agtgatgccccattccttgatcggcttcgccgagatcag aggtccctaaggggaagaggcaatactc	-	-
CY188197	Human H3N2 IAVs	Human	H3N2		2013	Nicaragua	A/Nicaragua/6228_04/2013	agtgatgccccattccttgatcggcttcgccgagatcag aggtccctaaggggaagaggcaatactc	-	-
CY188373	Human H3N2 IAVs	Human	H3N2		2013	Nicaragua	A/Nicaragua/6840_02/2013	agtgatgccccattccttgatcggcttcgccgagatcag aggtccctaaggggaagaggcaatactc	-	-
CY187965	Human H3N2 IAVs	Human	H3N2		2013	Nicaragua	A/Nicaragua/6909_03/2013	agtgatgccccattccttgatcggcttcgccgagatcag aggtccctaaggggaagaggcaatactc	-	-
CY188117	Human H3N2 IAVs	Human	H3N2		2013	Nicaragua	A/Nicaragua/6118_03/2013	agtgatgccccattccttgatcggcttcgccgagatcag aggtccctaaggggaagaggcaatactc	-	-
CY188269	Human H3N2 IAVs	Human	H3N2		2013	Nicaragua	A/Nicaragua/7660_03/2013	agtgatgccccattccttgatcggcttcgccgagatcag aggtccctaaggggaagaggcaatactc	-	-
CY187885	Human H3N2 IAVs	Human	H3N2		2013	Nicaragua	A/Nicaragua/6596_07/2013	agtgatgccccattccttgatcggcttcgccgagatcag aggtccctaaggggaagaggcaatactc	-	-
CY187893	Human H3N2 IAVs	Human	H3N2		2013	Nicaragua	A/Nicaragua/7477_04/2013	agtgatgccccattccttgatcggcttcgccgagatcag aggtccctaaggggaagaggcaatactc	-	-
CY148872	Human H3N2 IAVs	Human	H3N2		2013	USA	A/Boston/DOA2_174/2013	agtgatgccccattccttgatcggcttcgccgagatcag aggtccctaaggggaagaggcaatactc	-	-
CY168523	Human H3N2 IAVs	Human	H3N2		2013	USA	A/Boston/YGA_00099/2013	agtgatgccccattccttgatcggcttcgccgagatcag aggtccctaaggggaagaggcaatactc	-	-
CY149032	Human H3N2 IAVs	Human	H3N2		2013	USA	A/Boston/DOA2_199/2013	agtgatgccccattccttgatcggcttcgccgagatcag aggtccctaaggggaagaggcaatactc	-	-
CY149104	Human H3N2 IAVs	Human	H3N2		2013	USA	A/Boston/DOA2_214/2013	agtgatgccccattccttgatcggcttcgccgagatcag aggtccctaaggggaagaggcaatactc	-	-
CY148840	Human H3N2 IAVs	Human	H3N2		2013	USA	A/Boston/DOA2_170/2013	agtgatgccccattccttgatcggcttcgccgagatcag aggtccctaaggggaagaggcaatactc	-	-
CY148880	Human H3N2 IAVs	Human	H3N2		2013	USA	A/Boston/DOA2_175/2013	agtgatgccccattccttgatcggcttcgccgagatcag aggtccctaaggggaagaggcaatactc	-	-
CY148968	Human H3N2 IAVs	Human	H3N2		2013	USA	A/Boston/DOA2_186/2013	agtgatgccccattccttgatcggcttcgccgagatcag aggtccctaaggggaagaggcaatactc	-	-
CY149072	Human H3N2 IAVs	Human	H3N2		2013	USA	A/Boston/DOA2_207/2013	agtgatgccccattccttgatcggcttcgccgagatcag aggtccctaaggggaagaggcaatactc	-	-
CY149184	Human H3N2 IAVs	Human	H3N2		2013	USA	A/Boston/DOA2_225/2013	agtgatgccccattccttgatcggcttcgccgagatcag aggtccctaaggggaagaggcaatactc	-	-
CY149240	Human H3N2 IAVs	Human	H3N2		2013	USA	A/Boston/DOA2_233/2013	agtgatgccccattccttgatcggcttcgccgagatcag aggtccctaaggggaagaggcaatactc	-	-

CY149288	Human H3N2 IAVs	Human	H3N2		2013	USA	A/Boston/DOA2_241/2013	agtgatgccccattccttgatcggcttcgccgagatcag<br>aggtccctaaggggaagaggcaatactc	-	-
CY168131	Human H3N2 IAVs	Human	H3N2		2013	USA	A/Boston/YGA_00041/2013	agtgatgccccattccttgatcggcttcgccgagatcag<br>aggtccctaaggggaagaggcaatactc	-	-
CY168331	Human H3N2 IAVs	Human	H3N2		2013	USA	A/Boston/YGA_00071/2013	agtgatgccccattccttgatcggcttcgccgagatcag<br>aggtccctaaggggaagaggcaatactc	-	-
CY168403	Human H3N2 IAVs	Human	H3N2		2013	USA	A/Boston/YGA_00083/2013	agtgatgccccattccttgatcggcttcgccgagatcag<br>aggtccctaaggggaagaggcaatactc	-	-
CY169371	Human H3N2 IAVs	Human	H3N2		2013	USA	A/Boston/YGA_01118/2013	agtgatgccccattccttgatcggcttcgccgagatcag<br>aggtccctaaggggaagaggcaatactc	-	-
CY169515	Human H3N2 IAVs	Human	H3N2		2013	USA	A/Boston/YGA_01137/2013	agtgatgccccattccttgatcggcttcgccgagatcag<br>aggtccctaaggggaagaggcaatactc	-	-
CY169611	Human H3N2 IAVs	Human	H3N2		2013	USA	A/Boston/YGA_01150/2013	agtgatgccccattccttgatcggcttcgccgagatcag<br>aggtccctaaggggaagaggcaatactc	-	-
CY170019	Human H3N2 IAVs	Human	H3N2		2013	USA	A/Boston/YGA_01207/2013	agtgatgccccattccttgatcggcttcgccgagatcag<br>aggtccctaaggggaagaggcaatactc	-	-
CY170283	Human H3N2 IAVs	Human	H3N2		2013	USA	A/Boston/YGA_02027/2013	agtgatgccccattccttgatcggcttcgccgagatcag<br>aggtccctaaggggaagaggcaatactc	-	-
CY170379	Human H3N2 IAVs	Human	H3N2		2013	USA	A/Boston/YGA_02043/2013	agtgatgccccattccttgatcggcttcgccgagatcag<br>aggtccctaaggggaagaggcaatactc	-	-
CY170443	Human H3N2 IAVs	Human	H3N2		2013	USA	A/Boston/YGA_02058/2013	agtgatgccccattccttgatcggcttcgccgagatcag<br>aggtccctaaggggaagaggcaatactc	-	-
CY183173	Human H3N2 IAVs	Human	H3N2		2013	USA	A/Houston/JMM_125/2013	agtgatgccccattccttgatcggcttcgccgagatcag<br>aggtccctaaggggaagaggcaatactc	-	-
CY169379	Human H3N2 IAVs	Human	H3N2		2013	USA	A/Boston/YGA_01120/2013	agtgatgccccattccttgatcggcttcgccgagatcag<br>aggtccctaaggggaagaggcaatactc	-	-
CY170387	Human H3N2 IAVs	Human	H3N2		2013	USA	A/Boston/YGA_02045/2013	agtgatgccccattccttgatcggcttcgccgagatcag<br>aggtccctaaggggaagaggcaatactc	-	-
CY169627	Human H3N2 IAVs	Human	H3N2		2013	USA	A/Boston/YGA_01152/2013	agtgatgccccattccttgatcggcttcgccgagatcag<br>aggtccctaaggggaagaggcaatactc	-	-
CY169731	Human H3N2 IAVs	Human	H3N2		2013	USA	A/Boston/YGA_01165/2013	agtgatgccccattccttgatcggcttcgccgagatcag<br>aggtccctaaggggaagaggcaatactc	-	-
CY148904	Human H3N2 IAVs	Human	H3N2		2013	USA	A/Boston/DOA2_178/2013	agtgatgccccattccttgatcggcttcgccgagatcag<br>aggtccctaaggggaagaggcaatactc	-	-
CY168371	Human H3N2 IAVs	Human	H3N2		2013	USA	A/Boston/YGA_00079/2013	agtgatgccccattccttgatcggcttcgccgagatcag<br>aggtccctaaggggaagaggcaatactc	-	-
CY168307	Human H3N2 IAVs	Human	H3N2		2013	USA	A/Boston/YGA_00067/2013	agtgatgccccattccttgatcggcttcgccgagatcag<br>aggtccctaaggggaagaggcaatactc	-	-
CY170251	Human H3N2 IAVs	Human	H3N2		2013	USA	A/Boston/YGA_02023/2013	agtgatgccccattccttgatcggcttcgccgagatcag<br>aggtccctaaggggaagaggcaatactc	-	-
CY168123	Human H3N2 IAVs	Human	H3N2		2013	USA	A/Boston/YGA_00040/2013	agtgatgccccattccttgatcggcttcgccgagatcag<br>aggtccctaaggggaagaggcaatactc	-	-
CY148704	Human H3N2 IAVs	Human	H3N2		2013	USA	A/Boston/DOA2_149/2013	agtgatgccccattccttgatcggcttcgccgagatcag<br>aggtccctaaggggaagaggcaatactc	-	-
CY149304	Human H3N2 IAVs	Human	H3N2		2013	USA	A/Boston/DOA2_243/2013	agtgatgccccattccttgatcggcttcgccgagatcag<br>aggtccctaaggggaagaggcaatactc	-	-
CY168467	Human H3N2 IAVs	Human	H3N2		2013	USA	A/Boston/YGA_00092/2013	agtgatgccccattccttgatcggcttcgccgagatcag<br>aggtccctaaggggaagaggcaatactc	-	-
CY168235	Human H3N2 IAVs	Human	H3N2		2013	USA	A/Boston/YGA_00054/2013	agtgatgccccattccttgatcggcttcgccgagatcag<br>aggtccctaaggggaagaggcaatactc	-	-

CY168491	Human H3N2 IAVs	Human	H3N2		2013	USA	A/Boston/YGA_00095/2013	agtgatgccccattccttgatcggcttcgccgagatcag aggtccctaaggggaagaggcaatactc	-	-
CY170427	Human H3N2 IAVs	Human	H3N2		2013	USA	A/Boston/YGA_02054/2013	agtgatgccccattccttgatcggcttcgccgagatcag aggtccctaaggggaagaggcaatactc	-	-
CY169467	Human H3N2 IAVs	Human	H3N2		2013	USA	A/Boston/YGA_01131/2013	agtgatgccccattccttgatcggcttcgccgagatcag aggtccctaaggggaagaggcaatactc	-	-
CY169803	Human H3N2 IAVs	Human	H3N2		2013	USA	A/Boston/YGA_01174/2013	agtgatgccccattccttgatcggcttcgccgagatcag aggtccctaaggggaagaggcaatactc	-	-
CY148936	Human H3N2 IAVs	Human	H3N2		2013	USA	A/Boston/DOA2_182/2013	agtgatgccccattccttgatcggcttcgccgagatcag aggtccctaaggggaagaggcaatactc	-	-
CY168299	Human H3N2 IAVs	Human	H3N2		2013	USA	A/Boston/YGA_00066/2013	agtgatgccccattccttgatcggcttcgccgagatcag aggtccctaaggggaagaggcaatactc	-	-
CY183229	Human H3N2 IAVs	Human	H3N2		2013	USA	A/Houston/JMM_132/2013	agtgatgccccattccttgatcggcttcgccgagatcag aggtccctaaggggaagaggcaatactc	-	-
CY169699	Human H3N2 IAVs	Human	H3N2		2013	USA	A/Boston/YGA_01161/2013	agtgatgccccattccttgatcggcttcgccgagatcag aggtccctaaggggaagaggcaatactc	-	-
CY148720	Human H3N2 IAVs	Human	H3N2		2013	USA	A/Boston/DOA2_151/2013	agtgatgccccattccttgatcggcttcgccgagatcag aggtccctaaggggaagaggcaatactc	-	-
CY148776	Human H3N2 IAVs	Human	H3N2		2013	USA	A/Boston/DOA2_159/2013	agtgatgccccattccttgatcggcttcgccgagatcag aggtccctaaggggaagaggcaatactc	-	-
CY148960	Human H3N2 IAVs	Human	H3N2		2013	USA	A/Boston/DOA2_185/2013	agtgatgccccattccttgatcggcttcgccgagatcag aggtccctaaggggaagaggcaatactc	-	-
CY149128	Human H3N2 IAVs	Human	H3N2		2013	USA	A/Boston/DOA2_218/2013	agtgatgccccattccttgatcggcttcgccgagatcag aggtccctaaggggaagaggcaatactc	-	-
CY168083	Human H3N2 IAVs	Human	H3N2		2013	USA	A/Boston/YGA_00031/2013	agtgatgccccattccttgatcggcttcgccgagatcag aggtccctaaggggaagaggcaatactc	-	-
CY168139	Human H3N2 IAVs	Human	H3N2		2013	USA	A/Boston/YGA_00042/2013	agtgatgccccattccttgatcggcttcgccgagatcag aggtccctaaggggaagaggcaatactc	-	-
CY168211	Human H3N2 IAVs	Human	H3N2		2013	USA	A/Boston/YGA_00051/2013	agtgatgccccattccttgatcggcttcgccgagatcag aggtccctaaggggaagaggcaatactc	-	-
CY168219	Human H3N2 IAVs	Human	H3N2		2013	USA	A/Boston/YGA_00052/2013	agtgatgccccattccttgatcggcttcgccgagatcag aggtccctaaggggaagaggcaatactc	-	-
CY168267	Human H3N2 IAVs	Human	H3N2		2013	USA	A/Boston/YGA_00060/2013	agtgatgccccattccttgatcggcttcgccgagatcag aggtccctaaggggaagaggcaatactc	-	-
CY168291	Human H3N2 IAVs	Human	H3N2		2013	USA	A/Boston/YGA_00064/2013	agtgatgccccattccttgatcggcttcgccgagatcag aggtccctaaggggaagaggcaatactc	-	-
CY168323	Human H3N2 IAVs	Human	H3N2		2013	USA	A/Boston/YGA_00070/2013	agtgatgccccattccttgatcggcttcgccgagatcag aggtccctaaggggaagaggcaatactc	-	-
CY168355	Human H3N2 IAVs	Human	H3N2		2013	USA	A/Boston/YGA_00077/2013	agtgatgccccattccttgatcggcttcgccgagatcag aggtccctaaggggaagaggcaatactc	-	-
CY168363	Human H3N2 IAVs	Human	H3N2		2013	USA	A/Boston/YGA_00078/2013	agtgatgccccattccttgatcggcttcgccgagatcag aggtccctaaggggaagaggcaatactc	-	-
CY168419	Human H3N2 IAVs	Human	H3N2		2013	USA	A/Boston/YGA_00086/2013	agtgatgccccattccttgatcggcttcgccgagatcag aggtccctaaggggaagaggcaatactc	-	-
CY168435	Human H3N2 IAVs	Human	H3N2		2013	USA	A/Boston/YGA_00088/2013	agtgatgccccattccttgatcggcttcgccgagatcag aggtccctaaggggaagaggcaatactc	-	-
CY168483	Human H3N2 IAVs	Human	H3N2		2013	USA	A/Boston/YGA_00094/2013	agtgatgccccattccttgatcggcttcgccgagatcag aggtccctaaggggaagaggcaatactc	-	-
CY168499	Human H3N2 IAVs	Human	H3N2		2013	USA	A/Boston/YGA_00096/2013	agtgatgccccattccttgatcggcttcgccgagatcag aggtccctaaggggaagaggcaatactc	-	-

CY168507	Human H3N2 IAVs	Human	H3N2		2013	USA	A/Boston/YGA_00097/2013	agtgatgccccattccttgatcggcttcgccgagatcag<br>aggtccctaaggggaagaggcaatactc	-	-
CY168515	Human H3N2 IAVs	Human	H3N2		2013	USA	A/Boston/YGA_00098/2013	agtgatgccccattccttgatcggcttcgccgagatcag<br>aggtccctaaggggaagaggcaatactc	-	-
CY169363	Human H3N2 IAVs	Human	H3N2		2013	USA	A/Boston/YGA_01115/2013	agtgatgccccattccttgatcggcttcgccgagatcag<br>aggtccctaaggggaagaggcaatactc	-	-
CY169427	Human H3N2 IAVs	Human	H3N2		2013	USA	A/Boston/YGA_01126/2013	agtgatgccccattccttgatcggcttcgccgagatcag<br>aggtccctaaggggaagaggcaatactc	-	-
CY169483	Human H3N2 IAVs	Human	H3N2		2013	USA	A/Boston/YGA_01133/2013	agtgatgccccattccttgatcggcttcgccgagatcag<br>aggtccctaaggggaagaggcaatactc	-	-
CY169507	Human H3N2 IAVs	Human	H3N2		2013	USA	A/Boston/YGA_01136/2013	agtgatgccccattccttgatcggcttcgccgagatcag<br>aggtccctaaggggaagaggcaatactc	-	-
CY169571	Human H3N2 IAVs	Human	H3N2		2013	USA	A/Boston/YGA_01145/2013	agtgatgccccattccttgatcggcttcgccgagatcag<br>aggtccctaaggggaagaggcaatactc	-	-
CY169619	Human H3N2 IAVs	Human	H3N2		2013	USA	A/Boston/YGA_01151/2013	agtgatgccccattccttgatcggcttcgccgagatcag<br>aggtccctaaggggaagaggcaatactc	-	-
CY169635	Human H3N2 IAVs	Human	H3N2		2013	USA	A/Boston/YGA_01153/2013	agtgatgccccattccttgatcggcttcgccgagatcag<br>aggtccctaaggggaagaggcaatactc	-	-
CY169691	Human H3N2 IAVs	Human	H3N2		2013	USA	A/Boston/YGA_01160/2013	agtgatgccccattccttgatcggcttcgccgagatcag<br>aggtccctaaggggaagaggcaatactc	-	-
CY169739	Human H3N2 IAVs	Human	H3N2		2013	USA	A/Boston/YGA_01166/2013	agtgatgccccattccttgatcggcttcgccgagatcag<br>aggtccctaaggggaagaggcaatactc	-	-
CY169747	Human H3N2 IAVs	Human	H3N2		2013	USA	A/Boston/YGA_01167/2013	agtgatgccccattccttgatcggcttcgccgagatcag<br>aggtccctaaggggaagaggcaatactc	-	-
CY169795	Human H3N2 IAVs	Human	H3N2		2013	USA	A/Boston/YGA_01173/2013	agtgatgccccattccttgatcggcttcgccgagatcag<br>aggtccctaaggggaagaggcaatactc	-	-
CY169883	Human H3N2 IAVs	Human	H3N2		2013	USA	A/Boston/YGA_01189/2013	agtgatgccccattccttgatcggcttcgccgagatcag<br>aggtccctaaggggaagaggcaatactc	-	-
CY169907	Human H3N2 IAVs	Human	H3N2		2013	USA	A/Boston/YGA_01192/2013	agtgatgccccattccttgatcggcttcgccgagatcag<br>aggtccctaaggggaagaggcaatactc	-	-
CY169915	Human H3N2 IAVs	Human	H3N2		2013	USA	A/Boston/YGA_01193/2013	agtgatgccccattccttgatcggcttcgccgagatcag<br>aggtccctaaggggaagaggcaatactc	-	-
CY169955	Human H3N2 IAVs	Human	H3N2		2013	USA	A/Boston/YGA_01199/2013	agtgatgccccattccttgatcggcttcgccgagatcag<br>aggtccctaaggggaagaggcaatactc	-	-
CY169987	Human H3N2 IAVs	Human	H3N2		2013	USA	A/Boston/YGA_01203/2013	agtgatgccccattccttgatcggcttcgccgagatcag<br>aggtccctaaggggaagaggcaatactc	-	-
CY169995	Human H3N2 IAVs	Human	H3N2		2013	USA	A/Boston/YGA_01204/2013	agtgatgccccattccttgatcggcttcgccgagatcag<br>aggtccctaaggggaagaggcaatactc	-	-
CY170003	Human H3N2 IAVs	Human	H3N2		2013	USA	A/Boston/YGA_01205/2013	agtgatgccccattccttgatcggcttcgccgagatcag<br>aggtccctaaggggaagaggcaatactc	-	-
CY170011	Human H3N2 IAVs	Human	H3N2		2013	USA	A/Boston/YGA_01206/2013	agtgatgccccattccttgatcggcttcgccgagatcag<br>aggtccctaaggggaagaggcaatactc	-	-
CY170027	Human H3N2 IAVs	Human	H3N2		2013	USA	A/Boston/YGA_01208/2013	agtgatgccccattccttgatcggcttcgccgagatcag<br>aggtccctaaggggaagaggcaatactc	-	-
CY170067	Human H3N2 IAVs	Human	H3N2		2013	USA	A/Boston/YGA_01214/2013	agtgatgccccattccttgatcggcttcgccgagatcag<br>aggtccctaaggggaagaggcaatactc	-	-
CY170219	Human H3N2 IAVs	Human	H3N2		2013	USA	A/Boston/YGA_02018/2013	agtgatgccccattccttgatcggcttcgccgagatcag<br>aggtccctaaggggaagaggcaatactc	-	-
CY170235	Human H3N2 IAVs	Human	H3N2		2013	USA	A/Boston/YGA_02021/2013	agtgatgccccattccttgatcggcttcgccgagatcag<br>aggtccctaaggggaagaggcaatactc	-	-

CY170339	Human H3N2 IAVs	Human	H3N2		2013	USA	A/Boston/YGA_02037/2013	agtgatgccccattccttgatcggcttcgccgagatcag aggtccctaaggggaagaggcaatactc	-	-
CY170395	Human H3N2 IAVs	Human	H3N2		2013	USA	A/Boston/YGA_02047/2013	agtgatgccccattccttgatcggcttcgccgagatcag aggtccctaaggggaagaggcaatactc	-	-
CY171619	Human H3N2 IAVs	Human	H3N2		2013	USA	A/Chicago/YGA_04133/2013	agtgatgccccattccttgatcggcttcgccgagatcag aggtccctaaggggaagaggcaatactc	-	-
CY171635	Human H3N2 IAVs	Human	H3N2		2013	USA	A/Chicago/YGA_04135/2013	agtgatgccccattccttgatcggcttcgccgagatcag aggtccctaaggggaagaggcaatactc	-	-
CY171651	Human H3N2 IAVs	Human	H3N2		2013	USA	A/Chicago/YGA_04142/2013	agtgatgccccattccttgatcggcttcgccgagatcag aggtccctaaggggaagaggcaatactc	-	-
CY186223	Human H3N2 IAVs	Human	H3N2		2013	USA	A/Houston/JMM_176/2013	agtgatgccccattccttgatcggcttcgccgagatcag aggtccctaaggggaagaggcaatactc	-	-
CY170467	Human H3N2 IAVs	Human	H3N2		2013	USA	A/Santa_Clara/YGA_03003/2013	agtgatgccccattccttgatcggcttcgccgagatcag aggtccctaaggggaagaggcaatactc	-	-
CY170555	Human H3N2 IAVs	Human	H3N2		2013	USA	A/Santa_Clara/YGA_03014/2013	agtgatgccccattccttgatcggcttcgccgagatcag aggtccctaaggggaagaggcaatactc	-	-
CY170595	Human H3N2 IAVs	Human	H3N2		2013	USA	A/Santa_Clara/YGA_03019/2013	agtgatgccccattccttgatcggcttcgccgagatcag aggtccctaaggggaagaggcaatactc	-	-
CY170619	Human H3N2 IAVs	Human	H3N2		2013	USA	A/Santa_Clara/YGA_03022/2013	agtgatgccccattccttgatcggcttcgccgagatcag aggtccctaaggggaagaggcaatactc	-	-
CY170659	Human H3N2 IAVs	Human	H3N2		2013	USA	A/Santa_Clara/YGA_03027/2013	agtgatgccccattccttgatcggcttcgccgagatcag aggtccctaaggggaagaggcaatactc	-	-
CY170827	Human H3N2 IAVs	Human	H3N2		2013	USA	A/Santa_Clara/YGA_03050/2013	agtgatgccccattccttgatcggcttcgccgagatcag aggtccctaaggggaagaggcaatactc	-	-
CY170859	Human H3N2 IAVs	Human	H3N2		2013	USA	A/Santa_Clara/YGA_03055/2013	agtgatgccccattccttgatcggcttcgccgagatcag aggtccctaaggggaagaggcaatactc	-	-
CY170883	Human H3N2 IAVs	Human	H3N2		2013	USA	A/Santa_Clara/YGA_03059/2013	agtgatgccccattccttgatcggcttcgccgagatcag aggtccctaaggggaagaggcaatactc	-	-
CY170995	Human H3N2 IAVs	Human	H3N2		2013	USA	A/Santa_Clara/YGA_03074/2013	agtgatgccccattccttgatcggcttcgccgagatcag aggtccctaaggggaagaggcaatactc	-	-
CY171043	Human H3N2 IAVs	Human	H3N2		2013	USA	A/Santa_Clara/YGA_03080/2013	agtgatgccccattccttgatcggcttcgccgagatcag aggtccctaaggggaagaggcaatactc	-	-
KJ943062	Human H3N2 IAVs	Human	H3N2		2013	Chile	A/Santiago/p27d0/2013	agtgatgccccattccttgatcggcttcgccgagatcag aggtccctaaggggaagaggcaatactc	-	-
KJ943166	Human H3N2 IAVs	Human	H3N2		2013	Chile	A/Santiago/p27d3/2013	agtgatgccccattccttgatcggcttcgccgagatcag aggtccctaaggggaagaggcaatactc	-	-
KF789843	Human H3N2 IAVs	Human	H3N2		2013	USA	A/Washington/07/2013	agtgatgccccattccttgatcggcttcgccgagatcag aggtccctaaggggaagaggcaatactc	-	-
CY168347	Human H3N2 IAVs	Human	H3N2		2013	USA	A/Boston/YGA_00076/2013	agtgatgccccattccttgatcggcttcgccgagatcag aggtccctaaggggaagaggcaatactc	-	-
CY183005	Human H3N2 IAVs	Human	H3N2		2013	USA	A/Houston/JMM_103/2013	agtgatgccccattccttgatcggcttcgccgagatcag aggtccctaaggggaagaggcaatactc	-	-
CY183285	Human H3N2 IAVs	Human	H3N2		2013	USA	A/Houston/JMM_141/2013	agtgatgccccattccttgatcggcttcgccgagatcag aggtccctaaggggaagaggcaatactc	-	-
CY183293	Human H3N2 IAVs	Human	H3N2		2013	USA	A/Houston/JMM_142/2013	agtgatgccccattccttgatcggcttcgccgagatcag aggtccctaaggggaagaggcaatactc	-	-
CY168243	Human H3N2 IAVs	Human	H3N2		2013	USA	A/Boston/YGA_00055/2013	agtgatgccccattccttgatcggcttcgccgagatcag aggtccctaaggggaagaggcaatactc	-	-
CY148752	Human H3N2 IAVs	Human	H3N2		2013	USA	A/Boston/DOA2_156/2013	agtgatgccccattccttgatcggcttcgccgagatcag aggtccctaaggggaagaggcaatactc	-	-

CY168315	Human H3N2 IAVs	Human	H3N2		2013	USA	A/Boston/YGA_00068/2013	agtgatgccccattccttgatcggcttcgccgagatcag aggtccctaaggggaagaggcaatactc	-	-
CY170779	Human H3N2 IAVs	Human	H3N2		2013	USA	A/Santa_Clara/YGA_03044/2013	agtgatgccccattccttgatcggcttcgccgagatcag aggtccctaaggggaagaggcaatactc	-	-
CY171587	Human H3N2 IAVs	Human	H3N2		2013	USA	A/Chicago/YGA_04129/2013	agtgatgccccattccttgatcggcttcgccgagatcag aggtccctaaggggaagaggcaatactc	-	-
KJ561742	Human H3N2 IAVs	Human	H3N2		2013	Czech_Republic	A/Czech_Republic/200/2013	agtgatgccccattccttgatcggcttcgccgagatcag aggtccctaaggggaagaggcaatactc	-	-
CY171643	Human H3N2 IAVs	Human	H3N2		2013	USA	A/Chicago/YGA_04136/2013	agtgatgccccattccttgatcggcttcgccgagatcag aggtccctaaggggaagaggcaatactc	-	-
KM070123	Human H3N2 IAVs	Human	H3N2		2013	Singapore	A/Singapore/S2013.044/2013	agtgatgccccattccttgatcggcttcgccgagatcag aggtccctaaggggaagaggcaatactc	-	-
CY170587	Human H3N2 IAVs	Human	H3N2		2013	USA	A/Santa_Clara/YGA_03018/2013	agtgatgccccattccttgatcggcttcgccgagatcag aggtccctaaggggaagaggcaatactc	-	-
CY170739	Human H3N2 IAVs	Human	H3N2		2013	USA	A/Santa_Clara/YGA_03038/2013	agtgatgccccattccttgatcggcttcgccgagatcag aggtccctaaggggaagaggcaatactc	-	-
CY170275	Human H3N2 IAVs	Human	H3N2		2013	USA	A/Boston/YGA_02026/2013	agtgatgccccattccttgatcggcttcgccgagatcag aggtccctaaggggaagaggcaatactc	-	-
CY170291	Human H3N2 IAVs	Human	H3N2		2013	USA	A/Boston/YGA_02031/2013	agtgatgccccattccttgatcggcttcgccgagatcag aggtccctaaggggaagaggcaatactc	-	-
CY170563	Human H3N2 IAVs	Human	H3N2		2013	USA	A/Santa_Clara/YGA_03015/2013	agtgatgccccattccttgatcggcttcgccgagatcag aggtccctaaggggaagaggcaatactc	-	-
KJ561758	Human H3N2 IAVs	Human	H3N2		2013	Czech_Republic	A/Czech_Republic/166/2013	agtgatgccccattccttgatcggcttcgccgagatcag aggtccctaaggggaagaggcaatactc	-	-
CY171019	Human H3N2 IAVs	Human	H3N2		2013	USA	A/Santa_Clara/YGA_03077/2013	agtgatgccccattccttgatcggcttcgccgagatcag aggtccctaaggggaagaggcaatactc	-	-
CY170499	Human H3N2 IAVs	Human	H3N2		2013	USA	A/Santa_Clara/YGA_03007/2013	agtgatgccccattccttgatcggcttcgccgagatcag aggtccctaaggggaagaggcaatactc	-	-
KF034883	Human H3N2 IAVs	Human	H3N2		2013	China	A/Suzhou/1275/2013	agtgatgccccattccttgatcggcttcgccgagatcag aggtccctaaggggaagaggcaatactc	-	-
CY168059	Human H3N2 IAVs	Human	H3N2		2013	USA	A/Boston/YGA_00027/2013	agtgatgccccattccttgatcggcttcgccgagatcag aggtccctaaggggaagaggcaatactc	-	-
CY186079	Human H3N2 IAVs	Human	H3N2		2013	USA	A/Houston/JMM_156/2013	agtgatgccccattccttgatcggcttcgccgagatcag aggtccctaaggggaagaggcaatactc	-	-
CY169971	Human H3N2 IAVs	Human	H3N2		2013	USA	A/Boston/YGA_01201/2013	agtgatgccccattccttgatcggcttcgccgagatcag aggtccctaaggggaagaggcaatactc	-	-
CY170723	Human H3N2 IAVs	Human	H3N2		2013	USA	A/Santa_Clara/YGA_03036/2013	agtgatgccccattccttgatcggcttcgccgagatcag aggtccctaaggggaagaggcaatactc	-	-
CY170731	Human H3N2 IAVs	Human	H3N2		2013	USA	A/Santa_Clara/YGA_03037/2013	agtgatgccccattccttgatcggcttcgccgagatcag aggtccctaaggggaagaggcaatactc	-	-
CY168027	Human H3N2 IAVs	Human	H3N2		2013	USA	A/Boston/YGA_00022/2013	agtgatgccccattccttgatcggcttcgccgagatcag aggtccctaaggggaagaggcaatactc	-	-
CY168043	Human H3N2 IAVs	Human	H3N2		2013	USA	A/Boston/YGA_00025/2013	agtgatgccccattccttgatcggcttcgccgagatcag aggtccctaaggggaagaggcaatactc	-	-
CY169451	Human H3N2 IAVs	Human	H3N2		2013	USA	A/Boston/YGA_01129/2013	agtgatgccccattccttgatcggcttcgccgagatcag aggtccctaaggggaagaggcaatactc	-	-
CY183181	Human H3N2 IAVs	Human	H3N2		2013	USA	A/Houston/JMM_126/2013	agtgatgccccattccttgatcggcttcgccgagatcag aggtccctaaggggaagaggcaatactc	-	-
KJ943030	Human H3N2 IAVs	Human	H3N2		2013	Chile	A/Santiago/p34d0/2013	agtgatgccccattccttgatcggcttcgccgagatcag aggtccctaaggggaagaggcaatactc	-	-

KJ943195	Human H3N2 IAVs	Human	H3N2		2013	Chile	A/Santiago/p34d1/2013	agtgatgccccattccttgatcggcttcgccgagatcag aggtccctaaggggaagaggcaatactc	-	-
KJ943129	Human H3N2 IAVs	Human	H3N2		2013	Chile	A/Santiago/p34d7/2013	agtgatgccccattccttgatcggcttcgccgagatcag aggtccctaaggggaagaggcaatactc	-	-
KJ942947	Human H3N2 IAVs	Human	H3N2		2013	Chile	A/Santiago/p36d0/2013	agtgatgccccattccttgatcggcttcgccgagatcag aggtccctaaggggaagaggcaatactc	-	-
KJ942939	Human H3N2 IAVs	Human	H3N2		2013	Chile	A/Santiago/p36d1/2013	agtgatgccccattccttgatcggcttcgccgagatcag aggtccctaaggggaagaggcaatactc	-	-
KJ943078	Human H3N2 IAVs	Human	H3N2		2013	Chile	A/Santiago/p36d2/2013	agtgatgccccattccttgatcggcttcgccgagatcag aggtccctaaggggaagaggcaatactc	-	-
KJ943220	Human H3N2 IAVs	Human	H3N2		2013	Chile	A/Santiago/p36d5/2013	agtgatgccccattccttgatcggcttcgccgagatcag aggtccctaaggggaagaggcaatactc	-	-
CY168203	Human H3N2 IAVs	Human	H3N2		2013	USA	A/Boston/YGA_00050/2013	agtgatgccccattccttgatcggcttcgccgagatcag aggtccctaaggggaagaggcaatactc	-	-
CY171691	Human H3N2 IAVs	Human	H3N2		2013	USA	A/Chicago/YGA_04183/2013	agtgatgccccattccttgatcggcttcgccgagatcag aggtccctaaggggaagaggcaatactc	-	-
KF432087	Human H3N2 IAVs	Human	H3N2		2013	Singapore	A/Singapore/H2013.060/2013	agtgatgccccattccttgatcggcttcgccgagatcag aggtccctaaggggaagaggcaatactc	-	-
CY170411	Human H3N2 IAVs	Human	H3N2		2013	USA	A/Boston/YGA_02051/2013	agtgatgccccattccttgatcggcttcgccgagatcag aggtccctaaggggaagaggcaatactc	-	-
CY170635	Human H3N2 IAVs	Human	H3N2		2013	USA	A/Santa_Clara/YGA_03024/2013	agtgatgccccattccttgatcggcttcgccgagatcag aggtccctaaggggaagaggcaatactc	-	-
CY170763	Human H3N2 IAVs	Human	H3N2		2013	USA	A/Santa_Clara/YGA_03041/2013	agtgatgccccattccttgatcggcttcgccgagatcag aggtccctaaggggaagaggcaatactc	-	-
KM070126	Human H3N2 IAVs	Human	H3N2		2013	Singapore	A/Singapore/H2013.411/2013	agtgatgccccattccttgatcggcttcgccgagatcag aggtccctaaggggaagaggcaatactc	-	-
KM070135	Human H3N2 IAVs	Human	H3N2		2013	Singapore	A/Singapore/H2013.529/2013	agtgatgccccattccttgatcggcttcgccgagatcag aggtccctaaggggaagaggcaatactc	-	-
KM064106	Human H3N2 IAVs	Human	H3N2		2013	USA	A/Utah/07/2013	agtgatgccccattccttgatcggcttcgccgagatcag aggtccctaaggggaagaggcaatactc	-	-
KM070143	Human H3N2 IAVs	Human	H3N2		2013	Singapore	A/Singapore/H2013.778/2013	agtgatgccccattccttgatcggcttcgccgagatcag aggtccctaaggggaagaggcaatactc	-	-
CY170307	Human H3N2 IAVs	Human	H3N2		2013	USA	A/Boston/YGA_02033/2013	agtgatgccccattccttgatcggcttcgccgagatcag aggtccctaaggggaagaggcaatactc	-	-
CY148768	Human H3N2 IAVs	Human	H3N2		2013	USA	A/Boston/DOA2_158/2013	agtgatgccccattccttgatcggcttcgccgagatcag aggtccctaaggggaagaggcaatactc	-	-
KF928633	Human H3N2 IAVs	Human	H3N2		2013	USA	A/California/NHRC384259/2013	agtgatgccccattccttgatcggcttcgccgagatcag aggtccctaaggggaagaggcaatactc	-	-
CY171659	Human H3N2 IAVs	Human	H3N2		2013	USA	A/Chicago/YGA_04149/2013	agtgatgccccattccttgatcggcttcgccgagatcag aggtccctaaggggaagaggcaatactc	-	-
CY171667	Human H3N2 IAVs	Human	H3N2		2013	USA	A/Chicago/YGA_04150/2013	agtgatgccccattccttgatcggcttcgccgagatcag aggtccctaaggggaagaggcaatactc	-	-
CY149328	Human H3N2 IAVs	Human	H3N2		2013	USA	A/Boston/DOA2_246/2013	agtgatgccccattccttgatcggcttcgccgagatcag aggtccctaaggggaagaggcaatactc	-	-
CY148808	Human H3N2 IAVs	Human	H3N2		2013	USA	A/Boston/DOA2_166/2013	agtgatgccccattccttgatcggcttcgccgagatcag aggtccctaaggggaagaggcaatactc	-	-
CY148864	Human H3N2 IAVs	Human	H3N2		2013	USA	A/Boston/DOA2_173/2013	agtgatgccccattccttgatcggcttcgccgagatcag aggtccctaaggggaagaggcaatactc	-	-
CY148896	Human H3N2 IAVs	Human	H3N2		2013	USA	A/Boston/DOA2_177/2013	agtgatgccccattccttgatcggcttcgccgagatcag aggtccctaaggggaagaggcaatactc	-	-

CY148944	Human H3N2 IAVs	Human	H3N2		2013	USA	A/Boston/DOA2_183/2013	agtgatgccccattccttgatcggcttcgccgagatcag<br>aggtccctaaggggaagaggcaatactc	-	-
CY149000	Human H3N2 IAVs	Human	H3N2		2013	USA	A/Boston/DOA2_191/2013	agtgatgccccattccttgatcggcttcgccgagatcag<br>aggtccctaaggggaagaggcaatactc	-	-
CY149008	Human H3N2 IAVs	Human	H3N2		2013	USA	A/Boston/DOA2_192/2013	agtgatgccccattccttgatcggcttcgccgagatcag<br>aggtccctaaggggaagaggcaatactc	-	-
CY149024	Human H3N2 IAVs	Human	H3N2		2013	USA	A/Boston/DOA2_196/2013	agtgatgccccattccttgatcggcttcgccgagatcag<br>aggtccctaaggggaagaggcaatactc	-	-
CY149048	Human H3N2 IAVs	Human	H3N2		2013	USA	A/Boston/DOA2_202/2013	agtgatgccccattccttgatcggcttcgccgagatcag<br>aggtccctaaggggaagaggcaatactc	-	-
CY149064	Human H3N2 IAVs	Human	H3N2		2013	USA	A/Boston/DOA2_206/2013	agtgatgccccattccttgatcggcttcgccgagatcag<br>aggtccctaaggggaagaggcaatactc	-	-
CY149216	Human H3N2 IAVs	Human	H3N2		2013	USA	A/Boston/DOA2_229/2013	agtgatgccccattccttgatcggcttcgccgagatcag<br>aggtccctaaggggaagaggcaatactc	-	-
CY149272	Human H3N2 IAVs	Human	H3N2		2013	USA	A/Boston/DOA2_239/2013	agtgatgccccattccttgatcggcttcgccgagatcag<br>aggtccctaaggggaagaggcaatactc	-	-
CY149280	Human H3N2 IAVs	Human	H3N2		2013	USA	A/Boston/DOA2_240/2013	agtgatgccccattccttgatcggcttcgccgagatcag<br>aggtccctaaggggaagaggcaatactc	-	-
CY149296	Human H3N2 IAVs	Human	H3N2		2013	USA	A/Boston/DOA2_242/2013	agtgatgccccattccttgatcggcttcgccgagatcag<br>aggtccctaaggggaagaggcaatactc	-	-
CY168147	Human H3N2 IAVs	Human	H3N2		2013	USA	A/Boston/YGA_00043/2013	agtgatgccccattccttgatcggcttcgccgagatcag<br>aggtccctaaggggaagaggcaatactc	-	-
CY168163	Human H3N2 IAVs	Human	H3N2		2013	USA	A/Boston/YGA_00045/2013	agtgatgccccattccttgatcggcttcgccgagatcag<br>aggtccctaaggggaagaggcaatactc	-	-
CY168339	Human H3N2 IAVs	Human	H3N2		2013	USA	A/Boston/YGA_00074/2013	agtgatgccccattccttgatcggcttcgccgagatcag<br>aggtccctaaggggaagaggcaatactc	-	-
CY168459	Human H3N2 IAVs	Human	H3N2		2013	USA	A/Boston/YGA_00091/2013	agtgatgccccattccttgatcggcttcgccgagatcag<br>aggtccctaaggggaagaggcaatactc	-	-
CY169355	Human H3N2 IAVs	Human	H3N2		2013	USA	A/Boston/YGA_01114/2013	agtgatgccccattccttgatcggcttcgccgagatcag<br>aggtccctaaggggaagaggcaatactc	-	-
CY169387	Human H3N2 IAVs	Human	H3N2		2013	USA	A/Boston/YGA_01121/2013	agtgatgccccattccttgatcggcttcgccgagatcag<br>aggtccctaaggggaagaggcaatactc	-	-
CY169411	Human H3N2 IAVs	Human	H3N2		2013	USA	A/Boston/YGA_01124/2013	agtgatgccccattccttgatcggcttcgccgagatcag<br>aggtccctaaggggaagaggcaatactc	-	-
CY169459	Human H3N2 IAVs	Human	H3N2		2013	USA	A/Boston/YGA_01130/2013	agtgatgccccattccttgatcggcttcgccgagatcag<br>aggtccctaaggggaagaggcaatactc	-	-
CY169555	Human H3N2 IAVs	Human	H3N2		2013	USA	A/Boston/YGA_01143/2013	agtgatgccccattccttgatcggcttcgccgagatcag<br>aggtccctaaggggaagaggcaatactc	-	-
CY169563	Human H3N2 IAVs	Human	H3N2		2013	USA	A/Boston/YGA_01144/2013	agtgatgccccattccttgatcggcttcgccgagatcag<br>aggtccctaaggggaagaggcaatactc	-	-
CY169771	Human H3N2 IAVs	Human	H3N2		2013	USA	A/Boston/YGA_01170/2013	agtgatgccccattccttgatcggcttcgccgagatcag<br>aggtccctaaggggaagaggcaatactc	-	-
CY169827	Human H3N2 IAVs	Human	H3N2		2013	USA	A/Boston/YGA_01180/2013	agtgatgccccattccttgatcggcttcgccgagatcag<br>aggtccctaaggggaagaggcaatactc	-	-
CY169835	Human H3N2 IAVs	Human	H3N2		2013	USA	A/Boston/YGA_01181/2013	agtgatgccccattccttgatcggcttcgccgagatcag<br>aggtccctaaggggaagaggcaatactc	-	-
CY169843	Human H3N2 IAVs	Human	H3N2		2013	USA	A/Boston/YGA_01182/2013	agtgatgccccattccttgatcggcttcgccgagatcag<br>aggtccctaaggggaagaggcaatactc	-	-
CY169891	Human H3N2 IAVs	Human	H3N2		2013	USA	A/Boston/YGA_01190/2013	agtgatgccccattccttgatcggcttcgccgagatcag<br>aggtccctaaggggaagaggcaatactc	-	-

CY169899	Human H3N2 IAVs	Human	H3N2		2013	USA	A/Boston/YGA_01191/2013	agtgatgccccattccttgatcggcttcgccgagatcag aggtccctaaggggaagaggcaatactc	-	-
CY169923	Human H3N2 IAVs	Human	H3N2		2013	USA	A/Boston/YGA_01194/2013	agtgatgccccattccttgatcggcttcgccgagatcag aggtccctaaggggaagaggcaatactc	-	-
CY169939	Human H3N2 IAVs	Human	H3N2		2013	USA	A/Boston/YGA_01197/2013	agtgatgccccattccttgatcggcttcgccgagatcag aggtccctaaggggaagaggcaatactc	-	-
CY170035	Human H3N2 IAVs	Human	H3N2		2013	USA	A/Boston/YGA_01209/2013	agtgatgccccattccttgatcggcttcgccgagatcag aggtccctaaggggaagaggcaatactc	-	-
CY170051	Human H3N2 IAVs	Human	H3N2		2013	USA	A/Boston/YGA_01212/2013	agtgatgccccattccttgatcggcttcgccgagatcag aggtccctaaggggaagaggcaatactc	-	-
CY170243	Human H3N2 IAVs	Human	H3N2		2013	USA	A/Boston/YGA_02022/2013	agtgatgccccattccttgatcggcttcgccgagatcag aggtccctaaggggaagaggcaatactc	-	-
CY170299	Human H3N2 IAVs	Human	H3N2		2013	USA	A/Boston/YGA_02032/2013	agtgatgccccattccttgatcggcttcgccgagatcag aggtccctaaggggaagaggcaatactc	-	-
CY170331	Human H3N2 IAVs	Human	H3N2		2013	USA	A/Boston/YGA_02036/2013	agtgatgccccattccttgatcggcttcgccgagatcag aggtccctaaggggaagaggcaatactc	-	-
CY170355	Human H3N2 IAVs	Human	H3N2		2013	USA	A/Boston/YGA_02040/2013	agtgatgccccattccttgatcggcttcgccgagatcag aggtccctaaggggaagaggcaatactc	-	-
CY170363	Human H3N2 IAVs	Human	H3N2		2013	USA	A/Boston/YGA_02041/2013	agtgatgccccattccttgatcggcttcgccgagatcag aggtccctaaggggaagaggcaatactc	-	-
CY170403	Human H3N2 IAVs	Human	H3N2		2013	USA	A/Boston/YGA_02049/2013	agtgatgccccattccttgatcggcttcgccgagatcag aggtccctaaggggaagaggcaatactc	-	-
CY149016	Human H3N2 IAVs	Human	H3N2		2013	USA	A/Boston/DOA2_194/2013	agtgatgccccattccttgatcggcttcgccgagatcag aggtccctaaggggaagaggcaatactc	-	-
CY149312	Human H3N2 IAVs	Human	H3N2		2013	USA	A/Boston/DOA2_244/2013	agtgatgccccattccttgatcggcttcgccgagatcag aggtccctaaggggaagaggcaatactc	-	-
CY167800	Human H3N2 IAVs	Human	H3N2		2013	USA	A/Boston/YGA_00073/2013	agtgatgccccattccttgatcggcttcgccgagatcag aggtccctaaggggaagaggcaatactc	-	-
CY171579	Human H3N2 IAVs	Human	H3N2		2013	USA	A/Chicago/YGA_04128/2013	agtgatgccccattccttgatcggcttcgccgagatcag aggtccctaaggggaagaggcaatactc	-	-
CY171675	Human H3N2 IAVs	Human	H3N2		2013	USA	A/Chicago/YGA_04164/2013	agtgatgccccattccttgatcggcttcgccgagatcag aggtccctaaggggaagaggcaatactc	-	-
CY171707	Human H3N2 IAVs	Human	H3N2		2013	USA	A/Chicago/YGA_04187/2013	agtgatgccccattccttgatcggcttcgccgagatcag aggtccctaaggggaagaggcaatactc	-	-
KF805700	Human H3N2 IAVs	Human	H3N2		2013	Finland	A/Helsinki/951/2013	agtgatgccccattccttgatcggcttcgccgagatcag aggtccctaaggggaagaggcaatactc	-	-
CY183213	Human H3N2 IAVs	Human	H3N2		2013	USA	A/Houston/JMM_130/2013	agtgatgccccattccttgatcggcttcgccgagatcag aggtccctaaggggaagaggcaatactc	-	-
CY183253	Human H3N2 IAVs	Human	H3N2		2013	USA	A/Houston/JMM_136/2013	agtgatgccccattccttgatcggcttcgccgagatcag aggtccctaaggggaagaggcaatactc	-	-
CY186183	Human H3N2 IAVs	Human	H3N2		2013	USA	A/Houston/JMM_170/2013	agtgatgccccattccttgatcggcttcgccgagatcag aggtccctaaggggaagaggcaatactc	-	-
CY186207	Human H3N2 IAVs	Human	H3N2		2013	USA	A/Houston/JMM_174/2013	agtgatgccccattccttgatcggcttcgccgagatcag aggtccctaaggggaagaggcaatactc	-	-
CY186215	Human H3N2 IAVs	Human	H3N2		2013	USA	A/Houston/JMM_175/2013	agtgatgccccattccttgatcggcttcgccgagatcag aggtccctaaggggaagaggcaatactc	-	-
CY186231	Human H3N2 IAVs	Human	H3N2		2013	USA	A/Houston/JMM_177/2013	agtgatgccccattccttgatcggcttcgccgagatcag aggtccctaaggggaagaggcaatactc	-	-
CY186247	Human H3N2 IAVs	Human	H3N2		2013	USA	A/Houston/JMM_179/2013	agtgatgccccattccttgatcggcttcgccgagatcag aggtccctaaggggaagaggcaatactc	-	-

CY187917	Human H3N2 IAVs	Human	H3N2		2013	Nicaragua	A/Nicaragua/6676_09/2013	agtgatgccccattccttgatcggcttcgccgagatcag aggtccctaaggggaagaggcaatactc	-	-
CY188101	Human H3N2 IAVs	Human	H3N2		2013	Nicaragua	A/Nicaragua/7228_01/2013	agtgatgccccattccttgatcggcttcgccgagatcag aggtccctaaggggaagaggcaatactc	-	-
CY170515	Human H3N2 IAVs	Human	H3N2		2013	USA	A/Santa_Clara/YGA_03009/2013	agtgatgccccattccttgatcggcttcgccgagatcag aggtccctaaggggaagaggcaatactc	-	-
CY170531	Human H3N2 IAVs	Human	H3N2		2013	USA	A/Santa_Clara/YGA_03011/2013	agtgatgccccattccttgatcggcttcgccgagatcag aggtccctaaggggaagaggcaatactc	-	-
CY170539	Human H3N2 IAVs	Human	H3N2		2013	USA	A/Santa_Clara/YGA_03012/2013	agtgatgccccattccttgatcggcttcgccgagatcag aggtccctaaggggaagaggcaatactc	-	-
CY170579	Human H3N2 IAVs	Human	H3N2		2013	USA	A/Santa_Clara/YGA_03017/2013	agtgatgccccattccttgatcggcttcgccgagatcag aggtccctaaggggaagaggcaatactc	-	-
CY170707	Human H3N2 IAVs	Human	H3N2		2013	USA	A/Santa_Clara/YGA_03034/2013	agtgatgccccattccttgatcggcttcgccgagatcag aggtccctaaggggaagaggcaatactc	-	-
CY170795	Human H3N2 IAVs	Human	H3N2		2013	USA	A/Santa_Clara/YGA_03046/2013	agtgatgccccattccttgatcggcttcgccgagatcag aggtccctaaggggaagaggcaatactc	-	-
CY170835	Human H3N2 IAVs	Human	H3N2		2013	USA	A/Santa_Clara/YGA_03052/2013	agtgatgccccattccttgatcggcttcgccgagatcag aggtccctaaggggaagaggcaatactc	-	-
CY170867	Human H3N2 IAVs	Human	H3N2		2013	USA	A/Santa_Clara/YGA_03056/2013	agtgatgccccattccttgatcggcttcgccgagatcag aggtccctaaggggaagaggcaatactc	-	-
CY170891	Human H3N2 IAVs	Human	H3N2		2013	USA	A/Santa_Clara/YGA_03060/2013	agtgatgccccattccttgatcggcttcgccgagatcag aggtccctaaggggaagaggcaatactc	-	-
CY170923	Human H3N2 IAVs	Human	H3N2		2013	USA	A/Santa_Clara/YGA_03064/2013	agtgatgccccattccttgatcggcttcgccgagatcag aggtccctaaggggaagaggcaatactc	-	-
CY170939	Human H3N2 IAVs	Human	H3N2		2013	USA	A/Santa_Clara/YGA_03066/2013	agtgatgccccattccttgatcggcttcgccgagatcag aggtccctaaggggaagaggcaatactc	-	-
CY170987	Human H3N2 IAVs	Human	H3N2		2013	USA	A/Santa_Clara/YGA_03073/2013	agtgatgccccattccttgatcggcttcgccgagatcag aggtccctaaggggaagaggcaatactc	-	-
CY171027	Human H3N2 IAVs	Human	H3N2		2013	USA	A/Santa_Clara/YGA_03078/2013	agtgatgccccattccttgatcggcttcgccgagatcag aggtccctaaggggaagaggcaatactc	-	-
KM070124	Human H3N2 IAVs	Human	H3N2		2013	Singapore	A/Singapore/H2013.181/2013	agtgatgccccattccttgatcggcttcgccgagatcag aggtccctaaggggaagaggcaatactc	-	-
KM070127	Human H3N2 IAVs	Human	H3N2		2013	Singapore	A/Singapore/H2013.422a/2013	agtgatgccccattccttgatcggcttcgccgagatcag aggtccctaaggggaagaggcaatactc	-	-
KM070134	Human H3N2 IAVs	Human	H3N2		2013	Singapore	A/Singapore/H2013.463/2013	agtgatgccccattccttgatcggcttcgccgagatcag aggtccctaaggggaagaggcaatactc	-	-
CY171571	Human H3N2 IAVs	Human	H3N2		2013	USA	A/Chicago/YGA_04127/2013	agtgatgccccattccttgatcggcttcgccgagatcag aggtccctaaggggaagaggcaatactc	-	-
CY183205	Human H3N2 IAVs	Human	H3N2		2013	USA	A/Houston/JMM_129/2013	agtgatgccccattccttgatcggcttcgccgagatcag aggtccctaaggggaagaggcaatactc	-	-
CY168395	Human H3N2 IAVs	Human	H3N2		2013	USA	A/Boston/YGA_00082/2013	agtgatgccccattccttgatcggcttcgccgagatcag aggtccctaaggggaagaggcaatactc	-	-
CY148736	Human H3N2 IAVs	Human	H3N2		2013	USA	A/Boston/DOA2_154/2013	agtgatgccccattccttgatcggcttcgccgagatcag aggtccctaaggggaagaggcaatactc	-	-
CY170043	Human H3N2 IAVs	Human	H3N2		2013	USA	A/Boston/YGA_01210/2013	agtgatgccccattccttgatcggcttcgccgagatcag aggtccctaaggggaagaggcaatactc	-	-
KM070125	Human H3N2 IAVs	Human	H3N2		2013	Singapore	A/Singapore/H2013.384/2013	agtgatgccccattccttgatcggcttcgccgagatcag aggtccctaaggggaagaggcaatactc	-	-
KM070130	Human H3N2 IAVs	Human	H3N2		2013	Singapore	A/Singapore/H2013.425/2013	agtgatgccccattccttgatcggcttcgccgagatcag aggtccctaaggggaagaggcaatactc	-	-

CY170371	Human H3N2 IAVs	Human	H3N2		2013	USA	A/Boston/YGA_02042/2013	agtgatgccccattccttgatcggcttcgccgagatcag<br>aggtccctaaggggaagaggcaatactc	-	-
CY182997	Human H3N2 IAVs	Human	H3N2		2013	USA	A/Houston/JMM_102/2013	agtgatgccccattccttgatcggcttcgccgagatcag<br>aggtccctaaggggaagaggcaatactc	-	-
CY186095	Human H3N2 IAVs	Human	H3N2		2013	USA	A/Houston/JMM_158/2013	agtgatgccccattccttgatcggcttcgccgagatcag<br>aggtccctaaggggaagaggcaatactc	-	-
CY148664	Human H3N2 IAVs	Human	H3N2		2013	USA	A/Boston/DOA2_144/2013	agtgatgccccattccttgatcggcttcgccgagatcag<br>aggtccctaaggggaagaggcaatactc	-	-
CY186175	Human H3N2 IAVs	Human	H3N2		2013	USA	A/Houston/JMM_169/2013	agtgatgccccattccttgatcggcttcgccgagatcag<br>aggtccctaaggggaagaggcaatactc	-	-
CY149096	Human H3N2 IAVs	Human	H3N2		2013	USA	A/Boston/DOA2_213/2013	agtgatgccccattccttgatcggcttcgccgagatcag<br>aggtccctaaggggaagaggcaatactc	-	-
CY149152	Human H3N2 IAVs	Human	H3N2		2013	USA	A/Boston/DOA2_221/2013	agtgatgccccattccttgatcggcttcgccgagatcag<br>aggtccctaaggggaagaggcaatactc	-	-
CY168051	Human H3N2 IAVs	Human	H3N2		2013	USA	A/Boston/YGA_00026/2013	agtgatgccccattccttgatcggcttcgccgagatcag<br>aggtccctaaggggaagaggcaatactc	-	-
CY170899	Human H3N2 IAVs	Human	H3N2		2013	USA	A/Santa_Clara/YGA_03061/2013	agtgatgccccattccttgatcggcttcgccgagatcag<br>aggtccctaaggggaagaggcaatactc	-	-
CY169579	Human H3N2 IAVs	Human	H3N2		2013	USA	A/Boston/YGA_01146/2013	agtgatgccccattccttgatcggcttcgccgagatcag<br>aggtccctaaggggaagaggcaatactc	-	-
CY169587	Human H3N2 IAVs	Human	H3N2		2013	USA	A/Boston/YGA_01147/2013	agtgatgccccattccttgatcggcttcgccgagatcag<br>aggtccctaaggggaagaggcaatactc	-	-
CY170507	Human H3N2 IAVs	Human	H3N2		2013	USA	A/Santa_Clara/YGA_03008/2013	agtgatgccccattccttgatcggcttcgccgagatcag<br>aggtccctaaggggaagaggcaatactc	-	-
CY169643	Human H3N2 IAVs	Human	H3N2		2013	USA	A/Boston/YGA_01154/2013	agtgatgccccattccttgatcggcttcgccgagatcag<br>aggtccctaaggggaagaggcaatactc	-	-
CY169963	Human H3N2 IAVs	Human	H3N2		2013	USA	A/Boston/YGA_01200/2013	agtgatgccccattccttgatcggcttcgccgagatcag<br>aggtccctaaggggaagaggcaatactc	-	-
CY170059	Human H3N2 IAVs	Human	H3N2		2013	USA	A/Boston/YGA_01213/2013	agtgatgccccattccttgatcggcttcgccgagatcag<br>aggtccctaaggggaagaggcaatactc	-	-
CY171595	Human H3N2 IAVs	Human	H3N2		2013	USA	A/Chicago/YGA_04130/2013	agtgatgccccattccttgatcggcttcgccgagatcag<br>aggtccctaaggggaagaggcaatactc	-	-
CY186119	Human H3N2 IAVs	Human	H3N2		2013	USA	A/Houston/JMM_161/2013	agtgatgccccattccttgatcggcttcgccgagatcag<br>aggtccctaaggggaagaggcaatactc	-	-
CY170459	Human H3N2 IAVs	Human	H3N2		2013	USA	A/Santa_Clara/YGA_03002/2013	agtgatgccccattccttgatcggcttcgccgagatcag<br>aggtccctaaggggaagaggcaatactc	-	-
CY170523	Human H3N2 IAVs	Human	H3N2		2013	USA	A/Santa_Clara/YGA_03010/2013	agtgatgccccattccttgatcggcttcgccgagatcag<br>aggtccctaaggggaagaggcaatactc	-	-
CY170667	Human H3N2 IAVs	Human	H3N2		2013	USA	A/Santa_Clara/YGA_03028/2013	agtgatgccccattccttgatcggcttcgccgagatcag<br>aggtccctaaggggaagaggcaatactc	-	-
CY169755	Human H3N2 IAVs	Human	H3N2		2013	USA	A/Boston/YGA_01168/2013	agtgatgccccattccttgatcggcttcgccgagatcag<br>aggtccctaaggggaagaggcaatactc	-	-
CY169859	Human H3N2 IAVs	Human	H3N2		2013	USA	A/Boston/YGA_01184/2013	agtgatgccccattccttgatcggcttcgccgagatcag<br>aggtccctaaggggaagaggcaatactc	-	-
CY169931	Human H3N2 IAVs	Human	H3N2		2013	USA	A/Boston/YGA_01196/2013	agtgatgccccattccttgatcggcttcgccgagatcag<br>aggtccctaaggggaagaggcaatactc	-	-
CY170715	Human H3N2 IAVs	Human	H3N2		2013	USA	A/Santa_Clara/YGA_03035/2013	agtgatgccccattccttgatcggcttcgccgagatcag<br>aggtccctaaggggaagaggcaatactc	-	-
CY170627	Human H3N2 IAVs	Human	H3N2		2013	USA	A/Santa_Clara/YGA_03023/2013	agtgatgccccattccttgatcggcttcgccgagatcag<br>aggtccctaaggggaagaggcaatactc	-	-

CY170811	Human H3N2 IAVs	Human	H3N2		2013	USA	A/Santa_Clara/YGA_03048/2013	agtgatgccccattccttgatcggcttcgccgagatcag<br>aggtccctaaggggaagaggcaatactc	-	-
CY148728	Human H3N2 IAVs	Human	H3N2		2013	USA	A/Boston/DOA2_153/2013	agtgatgccccattccttgatcggcttcgccgagatcag<br>aggtccctaaggggaagaggcaatactc	-	-
CY168251	Human H3N2 IAVs	Human	H3N2		2013	USA	A/Boston/YGA_00056/2013	agtgatgccccattccttgatcggcttcgccgagatcag<br>aggtccctaaggggaagaggcaatactc	-	-
CY169339	Human H3N2 IAVs	Human	H3N2		2013	USA	A/Boston/YGA_01112/2013	agtgatgccccattccttgatcggcttcgccgagatcag<br>aggtccctaaggggaagaggcaatactc	-	-
CY171699	Human H3N2 IAVs	Human	H3N2		2013	USA	A/Chicago/YGA_04184/2013	agtgatgccccattccttgatcggcttcgccgagatcag<br>aggtccctaaggggaagaggcaatactc	-	-
CY169875	Human H3N2 IAVs	Human	H3N2		2013	USA	A/Boston/YGA_01186/2013	agtgatgccccattccttgatcggcttcgccgagatcag<br>aggtccctaaggggaagaggcaatactc	-	-
CY167872	Human H3N2 IAVs	Human	H3N2		2013	USA	A/Santa_Clara/YGA_03071/2013	agtgatgccccattccttgatcggcttcgccgagatcag<br>aggtccctaaggggaagaggcaatactc	-	-
CY171723	Human H3N2 IAVs	Human	H3N2		2013	USA	A/Chicago/YGA_04193/2013	agtgatgccccattccttgatcggcttcgccgagatcag<br>aggtccctaaggggaagaggcaatactc	-	-
CY183133	Human H3N2 IAVs	Human	H3N2		2013	USA	A/Houston/JMM_120/2013	agtgatgccccattccttgatcggcttcgccgagatcag<br>aggtccctaaggggaagaggcaatactc	-	-
CY168115	Human H3N2 IAVs	Human	H3N2		2013	USA	A/Boston/YGA_00039/2013	agtgatgccccattccttgatcggcttcgccgagatcag<br>aggtccctaaggggaagaggcaatactc	-	-
CY169491	Human H3N2 IAVs	Human	H3N2		2013	USA	A/Boston/YGA_01134/2013	agtgatgccccattccttgatcggcttcgccgagatcag<br>aggtccctaaggggaagaggcaatactc	-	-
KM070128	Human H3N2 IAVs	Human	H3N2		2013	Singapore	A/Singapore/H2013.422b/2013	agtgatgccccattccttgatcggcttcgccgagatcag<br>aggtccctaaggggaagaggcaatactc	-	-
KM070137	Human H3N2 IAVs	Human	H3N2		2013	Singapore	A/Singapore/H2013.696/2013	agtgatgccccattccttgatcggcttcgccgagatcag<br>aggtccctaaggggaagaggcaatactc	-	-
KM070129	Human H3N2 IAVs	Human	H3N2		2013	Singapore	A/Singapore/H2013.422c/2013	agtgatgccccattccttgatcggcttcgccgagatcag<br>aggtccctaaggggaagaggcaatactc	-	-
KM070133	Human H3N2 IAVs	Human	H3N2		2013	Singapore	A/Singapore/H2013.447/2013	agtgatgccccattccttgatcggcttcgccgagatcag<br>aggtccctaaggggaagaggcaatactc	-	-
KM070142	Human H3N2 IAVs	Human	H3N2		2013	Singapore	A/Singapore/H2013.751/2013	agtgatgccccattccttgatcggcttcgccgagatcag<br>aggtccctaaggggaagaggcaatactc	-	-
CY170547	Human H3N2 IAVs	Human	H3N2		2013	USA	A/Santa_Clara/YGA_03013/2013	agtgatgccccattccttgatcggcttcgccgagatcag<br>aggtccctaaggggaagaggcaatactc	-	-
CY148680	Human H3N2 IAVs	Human	H3N2		2013	USA	A/Boston/DOA2_146/2013	agtgatgccccattccttgatcggcttcgccgagatcag<br>aggtccctaaggggaagaggcaatactc	-	-
CY170347	Human H3N2 IAVs	Human	H3N2		2013	USA	A/Boston/YGA_02039/2013	agtgatgccccattccttgatcggcttcgccgagatcag<br>aggtccctaaggggaagaggcaatactc	-	-
CY168227	Human H3N2 IAVs	Human	H3N2		2013	USA	A/Boston/YGA_00053/2013	agtgatgccccattccttgatcggcttcgccgagatcag<br>aggtccctaaggggaagaggcaatactc	-	-
CY169811	Human H3N2 IAVs	Human	H3N2		2013	USA	A/Boston/YGA_01175/2013	agtgatgccccattccttgatcggcttcgccgagatcag<br>aggtccctaaggggaagaggcaatactc	-	-
KM070136	Human H3N2 IAVs	Human	H3N2		2013	Singapore	A/Singapore/H2013.545/2013	agtgatgccccattccttgatcggcttcgccgagatcag<br>aggtccctaaggggaagaggcaatactc	-	-
KM070138	Human H3N2 IAVs	Human	H3N2		2013	Singapore	A/Singapore/H2013.718a/2013	agtgatgccccattccttgatcggcttcgccgagatcag<br>aggtccctaaggggaagaggcaatactc	-	-
KM070139	Human H3N2 IAVs	Human	H3N2		2013	Singapore	A/Singapore/H2013.718b/2013	agtgatgccccattccttgatcggcttcgccgagatcag<br>aggtccctaaggggaagaggcaatactc	-	-
KM070140	Human H3N2 IAVs	Human	H3N2		2013	Singapore	A/Singapore/H2013.721c/2013	agtgatgccccattccttgatcggcttcgccgagatcag<br>aggtccctaaggggaagaggcaatactc	-	-

KM070141	Human H3N2 IAVs	Human	H3N2		2013	Singapore	A/Singapore/H2013.721e/2013	agtgatgccccattccttgatcggcttcgccgagatcag<br>aggtccctaaggggaagaggcaatactc	-	-
CY168067	Human H3N2 IAVs	Human	H3N2		2013	USA	A/Boston/YGA_00028/2013	agtgatgccccattccttgatcggcttcgccgagatcag<br>aggtccctaaggggaagaggcaatactc	-	-
CY148784	Human H3N2 IAVs	Human	H3N2		2013	USA	A/Boston/DOA2_161/2013	agtgatgccccattccttgatcggcttcgccgagatcag<br>aggtccctaaggggaagaggcaatactc	-	-
CY149120	Human H3N2 IAVs	Human	H3N2		2013	USA	A/Boston/DOA2_217/2013	agtgatgccccattccttgatcggcttcgccgagatcag<br>aggtccctaaggggaagaggcaatactc	-	-
CY148992	Human H3N2 IAVs	Human	H3N2		2013	USA	A/Boston/DOA2_190/2013	agtgatgccccattccttgatcggcttcgccgagatcag<br>aggtccctaaggggaagaggcaatactc	-	-
CY169531	Human H3N2 IAVs	Human	H3N2		2013	USA	A/Boston/YGA_01140/2013	agtgatgccccattccttgatcggcttcgccgagatcag<br>aggtccctaaggggaagaggcaatactc	-	-
CY149088	Human H3N2 IAVs	Human	H3N2		2013	USA	A/Boston/DOA2_212/2013	agtgatgccccattccttgatcggcttcgccgagatcag<br>aggtccctaaggggaagaggcaatactc	-	-
CY169539	Human H3N2 IAVs	Human	H3N2		2013	USA	A/Boston/YGA_01141/2013	agtgatgccccattccttgatcggcttcgccgagatcag<br>aggtccctaaggggaagaggcaatactc	-	-
CY170483	Human H3N2 IAVs	Human	H3N2		2013	USA	A/Santa_Clara/YGA_03005/2013	agtgatgccccattccttgatcggcttcgccgagatcag<br>aggtccctaaggggaagaggcaatactc	-	-
CY170651	Human H3N2 IAVs	Human	H3N2		2013	USA	A/Santa_Clara/YGA_03026/2013	agtgatgccccattccttgatcggcttcgccgagatcag<br>aggtccctaaggggaagaggcaatactc	-	-
CY170771	Human H3N2 IAVs	Human	H3N2		2013	USA	A/Santa_Clara/YGA_03042/2013	agtgatgccccattccttgatcggcttcgccgagatcag<br>aggtccctaaggggaagaggcaatactc	-	-
CY170971	Human H3N2 IAVs	Human	H3N2		2013	USA	A/Santa_Clara/YGA_03070/2013	agtgatgccccattccttgatcggcttcgccgagatcag<br>aggtccctaaggggaagaggcaatactc	-	-
CY149264	Human H3N2 IAVs	Human	H3N2		2013	USA	A/Boston/DOA2_238/2013	agtgatgccccattccttgatcggcttcgccgagatcag<br>aggtccctaaggggaagaggcaatactc	-	-
CY183045	Human H3N2 IAVs	Human	H3N2		2013	USA	A/Houston/JMM_108/2013	agtgatgccccattccttgatcggcttcgccgagatcag<br>aggtccctaaggggaagaggcaatactc	-	-
CY183085	Human H3N2 IAVs	Human	H3N2		2013	USA	A/Houston/JMM_113/2013	agtgatgccccattccttgatcggcttcgccgagatcag<br>aggtccctaaggggaagaggcaatactc	-	-
CY183189	Human H3N2 IAVs	Human	H3N2		2013	USA	A/Houston/JMM_127/2013	agtgatgccccattccttgatcggcttcgccgagatcag<br>aggtccctaaggggaagaggcaatactc	-	-
CY186023	Human H3N2 IAVs	Human	H3N2		2013	USA	A/Houston/JMM_149/2013	agtgatgccccattccttgatcggcttcgccgagatcag<br>aggtccctaaggggaagaggcaatactc	-	-
CY186055	Human H3N2 IAVs	Human	H3N2		2013	USA	A/Houston/JMM_153/2013	agtgatgccccattccttgatcggcttcgccgagatcag<br>aggtccctaaggggaagaggcaatactc	-	-
CY186063	Human H3N2 IAVs	Human	H3N2		2013	USA	A/Houston/JMM_154/2013	agtgatgccccattccttgatcggcttcgccgagatcag<br>aggtccctaaggggaagaggcaatactc	-	-
CY186111	Human H3N2 IAVs	Human	H3N2		2013	USA	A/Houston/JMM_160/2013	agtgatgccccattccttgatcggcttcgccgagatcag<br>aggtccctaaggggaagaggcaatactc	-	-
CY182685	Human H3N2 IAVs	Human	H3N2		2013	USA	A/Houston/JMM_165/2013	agtgatgccccattccttgatcggcttcgccgagatcag<br>aggtccctaaggggaagaggcaatactc	-	-
CY186239	Human H3N2 IAVs	Human	H3N2		2013	USA	A/Houston/JMM_178/2013	agtgatgccccattccttgatcggcttcgccgagatcag<br>aggtccctaaggggaagaggcaatactc	-	-
CY170571	Human H3N2 IAVs	Human	H3N2		2013	USA	A/Santa_Clara/YGA_03016/2013	agtgatgccccattccttgatcggcttcgccgagatcag<br>aggtccctaaggggaagaggcaatactc	-	-
CY170915	Human H3N2 IAVs	Human	H3N2		2013	USA	A/Santa_Clara/YGA_03063/2013	agtgatgccccattccttgatcggcttcgccgagatcag<br>aggtccctaaggggaagaggcaatactc	-	-
CY170963	Human H3N2 IAVs	Human	H3N2		2013	USA	A/Santa_Clara/YGA_03069/2013	agtgatgccccattccttgatcggcttcgccgagatcag<br>aggtccctaaggggaagaggcaatactc	-	-

CY183053	Human H3N2 IAVs	Human	H3N2		2013	USA	A/Houston/JMM_109/2013	agtgatgccccattccttgatcggcttcgccgagatcag<br>aggtccctaaggggaagaggcaatactc	-	-
CY183101	Human H3N2 IAVs	Human	H3N2		2013	USA	A/Houston/JMM_116/2013	agtgatgccccattccttgatcggcttcgccgagatcag<br>aggtccctaaggggaagaggcaatactc	-	-
CY183157	Human H3N2 IAVs	Human	H3N2		2013	USA	A/Houston/JMM_123/2013	agtgatgccccattccttgatcggcttcgccgagatcag<br>aggtccctaaggggaagaggcaatactc	-	-
CY183197	Human H3N2 IAVs	Human	H3N2		2013	USA	A/Houston/JMM_128/2013	agtgatgccccattccttgatcggcttcgccgagatcag<br>aggtccctaaggggaagaggcaatactc	-	-
CY183037	Human H3N2 IAVs	Human	H3N2		2013	USA	A/Houston/JMM_107/2013	agtgatgccccattccttgatcggcttcgccgagatcag<br>aggtccctaaggggaagaggcaatactc	-	-
CY186039	Human H3N2 IAVs	Human	H3N2		2013	USA	A/Houston/JMM_151/2013	agtgatgccccattccttgatcggcttcgccgagatcag<br>aggtccctaaggggaagaggcaatactc	-	-
CY170691	Human H3N2 IAVs	Human	H3N2		2013	USA	A/Santa_Clara/YGA_03032/2013	agtgatgccccattccttgatcggcttcgccgagatcag<br>aggtccctaaggggaagaggcaatactc	-	-
CY168259	Human H3N2 IAVs	Human	H3N2		2013	USA	A/Boston/YGA_00059/2013	agtgatgccccattccttgatcggcttcgccgagatcag<br>aggtccctaaggggaagaggcaatactc	-	-
KF805668	Human H3N2 IAVs	Human	H3N2		2013	Finland	A/Helsinki/879/2013	agtgatgccccattccttgatcggcttcgccgagatcag<br>aggtccctaaggggaagaggcaatactc	-	-
CY183165	Human H3N2 IAVs	Human	H3N2		2013	USA	A/Houston/JMM_124/2013	agtgatgccccattccttgatcggcttcgccgagatcag<br>aggtccctaaggggaagaggcaatactc	-	-
CY183277	Human H3N2 IAVs	Human	H3N2		2013	USA	A/Houston/JMM_140/2013	agtgatgccccattccttgatcggcttcgccgagatcag<br>aggtccctaaggggaagaggcaatactc	-	-
CY183093	Human H3N2 IAVs	Human	H3N2		2013	USA	A/Houston/JMM_115/2013	agtgatgccccattccttgatcggcttcgccgagatcag<br>aggtccctaaggggaagaggcaatactc	-	-
CY186031	Human H3N2 IAVs	Human	H3N2		2013	USA	A/Houston/JMM_150/2013	agtgatgccccattccttgatcggcttcgccgagatcag<br>aggtccctaaggggaagaggcaatactc	-	-
CY186167	Human H3N2 IAVs	Human	H3N2		2013	USA	A/Houston/JMM_168/2013	agtgatgccccattccttgatcggcttcgccgagatcag<br>aggtccctaaggggaagaggcaatactc	-	-
CY183117	Human H3N2 IAVs	Human	H3N2		2013	USA	A/Houston/JMM_118/2013	agtgatgccccattccttgatcggcttcgccgagatcag<br>aggtccctaaggggaagaggcaatactc	-	-
CY183245	Human H3N2 IAVs	Human	H3N2		2013	USA	A/Houston/JMM_135/2013	agtgatgccccattccttgatcggcttcgccgagatcag<br>aggtccctaaggggaagaggcaatactc	-	-
CY186143	Human H3N2 IAVs	Human	H3N2		2013	USA	A/Houston/JMM_164/2013	agtgatgccccattccttgatcggcttcgccgagatcag<br>aggtccctaaggggaagaggcaatactc	-	-
CY170803	Human H3N2 IAVs	Human	H3N2		2013	USA	A/Santa_Clara/YGA_03047/2013	agtgatgccccattccttgatcggcttcgccgagatcag<br>aggtccctaaggggaagaggcaatactc	-	-
KM064141	Human H3N2 IAVs	Human	H3N2		2013	USA	A/Massachusetts/11/2013	agtgatgccccattccttgatcggcttcgccgagatcag<br>aggtccctaaggggaagaggcaatactc	-	-
CY169499	Human H3N2 IAVs	Human	H3N2		2013	USA	A/Boston/YGA_01135/2013	agtgatgccccattccttgatcggcttcgccgagatcag<br>aggtccctaaggggaagaggcaatactc	-	-
CY170979	Human H3N2 IAVs	Human	H3N2		2013	USA	A/Santa_Clara/YGA_03072/2013	agtgatgccccattccttgatcggcttcgccgagatcag<br>aggtccctaaggggaagaggcaatactc	-	-
CY169851	Human H3N2 IAVs	Human	H3N2		2013	USA	A/Boston/YGA_01183/2013	agtgatgccccattccttgatcggcttcgccgagatcag<br>aggtccctaaggggaagaggcaatactc	-	-
CY170643	Human H3N2 IAVs	Human	H3N2		2013	USA	A/Santa_Clara/YGA_03025/2013	agtgatgccccattccttgatcggcttcgccgagatcag<br>aggtccctaaggggaagaggcaatactc	-	-
CY170451	Human H3N2 IAVs	Human	H3N2		2013	USA	A/Santa_Clara/YGA_03001/2013	agtgatgccccattccttgatcggcttcgccgagatcag<br>aggtccctaaggggaagaggcaatactc	-	-
CY171035	Human H3N2 IAVs	Human	H3N2		2013	USA	A/Santa_Clara/YGA_03079/2013	agtgatgccccattccttgatcggcttcgccgagatcag<br>aggtccctaaggggaagaggcaatactc	-	-

CY170315	Human H3N2 IAVs	Human	H3N2		2013	USA	A/Boston/YGA_02034/2013	agtgatgccccattccttgatcggcttcgccgagatcag aggtccctaaggggaagaggcaatactc	-	-
CY182989	Human H3N2 IAVs	Human	H3N2		2013	USA	A/Houston/JMM_101/2013	agtgatgccccattccttgatcggcttcgccgagatcag aggtccctaaggggaagaggcaatactc	-	-
CY183309	Human H3N2 IAVs	Human	H3N2		2013	USA	A/Houston/JMM_145/2013	agtgatgccccattccttgatcggcttcgccgagatcag aggtccctaaggggaagaggcaatactc	-	-
CY170475	Human H3N2 IAVs	Human	H3N2		2013	USA	A/Santa_Clara/YGA_03004/2013	agtgatgccccattccttgatcggcttcgccgagatcag aggtccctaaggggaagaggcaatactc	-	-
CY186087	Human H3N2 IAVs	Human	H3N2		2013	USA	A/Houston/JMM_157/2013	agtgatgccccattccttgatcggcttcgccgagatcag aggtccctaaggggaagaggcaatactc	-	-
KF789784	Human H3N2 IAVs	Human	H3N2		2013	USA	A/Alaska/02/2013	agtgatgccccattccttgatcggcttcgccgagatcag aggtccctaaggggaagaggcaatactc	-	-
KF790321	Human H3N2 IAVs	Human	H3N2		2013	USA	A/Alaska/02/2013	agtgatgccccattccttgatcggcttcgccgagatcag aggtccctaaggggaagaggcaatactc	-	-
CY168107	Human H3N2 IAVs	Human	H3N2		2013	USA	A/Boston/YGA_00038/2013	agtgatgccccattccttgatcggcttcgccgagatcag aggtccctaaggggaagaggcaatactc	-	-
CY169331	Human H3N2 IAVs	Human	H3N2		2013	USA	A/Boston/YGA_01111/2013	agtgatgccccattccttgatcggcttcgccgagatcag aggtccctaaggggaagaggcaatactc	-	-
CY169419	Human H3N2 IAVs	Human	H3N2		2013	USA	A/Boston/YGA_01125/2013	agtgatgccccattccttgatcggcttcgccgagatcag aggtccctaaggggaagaggcaatactc	-	-
CY169595	Human H3N2 IAVs	Human	H3N2		2013	USA	A/Boston/YGA_01148/2013	agtgatgccccattccttgatcggcttcgccgagatcag aggtccctaaggggaagaggcaatactc	-	-
CY171683	Human H3N2 IAVs	Human	H3N2		2013	USA	A/Chicago/YGA_04165/2013	agtgatgccccattccttgatcggcttcgccgagatcag aggtccctaaggggaagaggcaatactc	-	-
CY171011	Human H3N2 IAVs	Human	H3N2		2013	USA	A/Santa_Clara/YGA_03076/2013	agtgatgccccattccttgatcggcttcgccgagatcag aggtccctaaggggaagaggcaatactc	-	-
CY186255	Human H3N2 IAVs	Human	H3N2		2013	USA	A/Houston/JMM_180/2013	agtgatgccccattccttgatcggcttcgccgagatcag aggtccctaaggggaagaggcaatactc	-	-
CY171003	Human H3N2 IAVs	Human	H3N2		2013	USA	A/Santa_Clara/YGA_03075/2013	agtgatgccccattccttgatcggcttcgccgagatcag aggtccctaaggggaagaggcaatactc	-	-
CY183237	Human H3N2 IAVs	Human	H3N2		2013	USA	A/Houston/JMM_134/2013	agtgatgccccattccttgatcggcttcgccgagatcag aggtccctaaggggaagaggcaatactc	-	-
CY148672	Human H3N2 IAVs	Human	H3N2		2013	USA	A/Boston/DOA2_145/2013	agtgatgccccattccttgatcggcttcgccgagatcag aggtccctaaggggaagaggcaatactc	-	-
CY183109	Human H3N2 IAVs	Human	H3N2		2013	USA	A/Houston/JMM_117/2013	agtgatgccccattccttgatcggcttcgccgagatcag aggtccctaaggggaagaggcaatactc	-	-
CY169523	Human H3N2 IAVs	Human	H3N2		2013	USA	A/Boston/YGA_01139/2013	agtgatgccccattccttgatcggcttcgccgagatcag aggtccctaaggggaagaggcaatactc	-	-
CY169667	Human H3N2 IAVs	Human	H3N2		2013	USA	A/Boston/YGA_01157/2013	agtgatgccccattccttgatcggcttcgccgagatcag aggtccctaaggggaagaggcaatactc	-	-
CY171611	Human H3N2 IAVs	Human	H3N2		2013	USA	A/Chicago/YGA_04132/2013	agtgatgccccattccttgatcggcttcgccgagatcag aggtccctaaggggaagaggcaatactc	-	-
CY171715	Human H3N2 IAVs	Human	H3N2		2013	USA	A/Chicago/YGA_04188/2013	agtgatgccccattccttgatcggcttcgccgagatcag aggtccctaaggggaagaggcaatactc	-	-
CY171739	Human H3N2 IAVs	Human	H3N2		2013	USA	A/Chicago/YGA_04200/2013	agtgatgccccattccttgatcggcttcgccgagatcag aggtccctaaggggaagaggcaatactc	-	-
CY148800	Human H3N2 IAVs	Human	H3N2		2013	USA	A/Boston/DOA2_163/2013	agtgatgccccattccttgatcggcttcgccgagatcag aggtccctaaggggaagaggcaatactc	-	-
CY171603	Human H3N2 IAVs	Human	H3N2		2013	USA	A/Chicago/YGA_04131/2013	agtgatgccccattccttgatcggcttcgccgagatcag aggtccctaaggggaagaggcaatactc	-	-

CY171627	Human H3N2 IAVs	Human	H3N2		2013	USA	A/Chicago/YGA_04134/2013	agtgatgccccattccttgatcggcttcgccgagatcag aggtccctaaggggaagaggcaatactc	-	-
CY183325	Human H3N2 IAVs	Human	H3N2		2013	USA	A/Houston/JMM_147/2013	agtgatgccccattccttgatcggcttcgccgagatcag aggtccctaaggggaagaggcaatactc	-	-
CY183125	Human H3N2 IAVs	Human	H3N2		2013	USA	A/Houston/JMM_119/2013	agtgatgccccattccttgatcggcttcgccgagatcag aggtccctaaggggaagaggcaatactc	-	-
KF805660	Human H3N2 IAVs	Human	H3N2		2013	Finland	A/Helsinki/824/2013	agtgatgccccattccttgatcggcttcgccgagatcag aggtccctaaggggaagaggcaatactc	-	-
KF805692	Human H3N2 IAVs	Human	H3N2		2013	Finland	A/Helsinki/942/2013	agtgatgccccattccttgatcggcttcgccgagatcag aggtccctaaggggaagaggcaatactc	-	-
CY169787	Human H3N2 IAVs	Human	H3N2		2013	USA	A/Boston/YGA_01172/2013	agtgatgccccattccttgatcggcttcgccgagatcag aggtccctaaggggaagaggcaatgctc	-	-
KJ880922	Human H3N2 IAVs	Human	H3N2		2013	Czech_Republic	A/Czech_Republic/218/2013	agtgatgccccattccttgatcggcttcgccgagatcag aggtccctaaggggaagaggcactactc	-	-
CY149176	Human H3N2 IAVs	Human	H3N2		2013	USA	A/Boston/DOA2_224/2013	agtgatgccccatttcttgatcggcttcgccgagatcag aggtccctaaggggaagaggcaatactc	-	-
CY183261	Human H3N2 IAVs	Human	H3N2		2013	USA	A/Houston/JMM_137/2013	agtgatgccccatttcttgatcggcttcgccgagatcag aggtccctaaggggaagaggcaatactc	-	-
CY183269	Human H3N2 IAVs	Human	H3N2		2013	USA	A/Houston/JMM_139/2013	agtgatgccccatttcttgatcggcttcgccgagatcag aggtccctaaggggaagaggcaatactc	-	-
CY186151	Human H3N2 IAVs	Human	H3N2		2013	USA	A/Houston/JMM_166/2013	agtgatgccccatttcttgatcggcttcgccgagatcag aggtccctaaggggaagaggcaatactc	-	-
CY186159	Human H3N2 IAVs	Human	H3N2		2013	USA	A/Houston/JMM_167/2013	agtgatgccccatttcttgatcggcttcgccgagatcag aggtccctaaggggaagaggcaatactc	-	-
CY171731	Human H3N2 IAVs	Human	H3N2		2013	USA	A/Chicago/YGA_04198/2013	agtgatgtcccattccttgatcggcttcgccgagatcag aggtccctaaggggaagaggcaatactc	+	+
KJ561710	Human H3N2 IAVs	Human	H3N2		2014	Czech_Republic	A/Czech_Republic/1/2014	ggtgatgccccattccttgatcggcttcgccgagatcag aggtccctaaggggaagaggcaatactc	-	-
CY146821	Human H3N2 IAVs	Human	H1N2		NA	Unknown	A/PAL/unknown	ggtgatgccccattccttgatcggcttcgccgagatcag aagtccctaaggggaagaggcagcactc	+	+
CY147394	Human H3N2 IAVs	Human	H3N2		NA	NA	A/Georgia/JY2/unknown	ggtgatgccccattccttgatcggcttcgccgagatcag aagtccctaaggggaagaggcagcactc	+	+
CY062951	Human H1N1pdm2009 IAVs	Human	H1N1	pdm	2009	Spain	A/Terrassa/INS173/2009	agtgatgccccattccttgatcggctccgccgagatca aaagtccttaaaaggaagaggcaacaccc	-	*
CY062807	Human H1N1pdm2009 IAVs	Human	H1N1	pdm	2009	Austria	A/Vienna/INS142/2009	agtgatgccccattccttgatcggctccgccgagatca aaagtccttaaaaggaagaggcaacaccc	-	*
JN187339	Human H1N1pdm2009 IAVs	Human	H1N1	pdm	2009	Taiwan	A/Taiwan/11706/2009	agtgatgccccattccttgatcggctccgccgagatca aaagtccttaaaaggaagaggcaacaccc	-	*
CY123134	Human H1N1pdm2009 IAVs	Human	H1N1	pdm	2009	Singapore	A/Singapore/GP3278/2009	agtgatgccccattccttgatcggctccgccgagatca aaagtccttaaaaggaagaggcaacaccc	-	*
CY123705	Human H1N1pdm2009 IAVs	Human	H1N1	pdm	2009	Singapore	A/Singapore/ON2009/2009	agtgatgccccattccttgatcggctccgccgagatca aaagtccttaaaaggaagaggcaacaccc	-	*
CY124127	Human H1N1pdm2009 IAVs	Human	H1N1	pdm	2009	Singapore	A/Singapore/ON812/2009	agtgatgccccattccttgatcggctccgccgagatca aaagtccttaaaaggaagaggcaacaccc	-	*
CY066611	Human H1N1pdm2009 IAVs	Human	H1N1	pdm	2009	Greece	A/Athens/INS273/2009	agtgatgccccattccttgatcggctccgccgagatca aaagtccttaaaaggaagaggcaacaccc	-	*
JQ173152	Human H1N1pdm2009 IAVs	Human	H1N1		2009	Finland	A/Helsinki/P14/2009	agtgatgccccattccttgatcggctccgccgagatca aaagtccttaaaaggaagaggcaacaccc	-	*
CY123178	Human H1N1pdm2009 IAVs	Human	H1N1	pdm	2009	Singapore	A/Singapore/GP3710/2009	ggagatgccccattccttgatcggctccgccgagatca aaagtccttaaaaggaagaggcaacaccc	-	*

CY055514	Human H1N1pdm2009 IAVs	Human	H1N1	pdm	2009	USA	A/California/VRDL76/2009	ggcgatgccccattccttgatcggctccgccgagatca aaaatccttaaaaggaagaggcaaaaccc	-	*
CY066435	Human H1N1pdm2009 IAVs	Human	H1N1	pdm	2009	USA	A/California/VRDL132/2009	ggcgatgccccattccttgatcggctccgccgagatca aaagtccttaaaaggaagaggcaacaccc	-	*
CY056016	Human H1N1pdm2009 IAVs	Human	H1N1	pdm	2009	USA	A/San_Diego/INS11/2009	ggcgatgccccattccttgatcggctccgccgagatca aaagtccttaaaaggaagaggcaacaccc	-	*
CY083673	Human H1N1pdm2009 IAVs	Human	H1N1	pdm	2009	USA	A/San_Diego/INS62/2009	ggcgatgccccattccttgatcggctccgccgagatca aaagtccttaaaaggaagaggcaacaccc	-	*
CY065804	Human H1N1pdm2009 IAVs	Human	H1N1	pdm	2009	Netherlands	A/Netherlands/2445b/2009	ggcgatgccccattccttgatcggctccgccgagatca aaagtccttaaaaggaagaggcaacaccc	-	*
GU136015	Human H1N1pdm2009 IAVs	Human	H1N1	pdm	2009	Japan	A/Niigata/690/2009	ggtgacgccccattccttgatcggctccgccgagatca aaagtccttaaaaggaagaggcaacaccc	-	*
JX625415	Human H1N1pdm2009 IAVs	Human	H1N1	pdm	2009	United_Kingdom	A/England/92880002/2009	ggtgatgcaccattccttgatcggctccgccgagatca aaagtccttaaaaggaagaggcaacaccc	-	*
GQ168856	Human H1N1pdm2009 IAVs	Human	H1N1	pdm	2009	USA	A/Kansas/02/2009	ggtgatgccccactccttgatcggctccgccgagatca aaagtccttaaaaggaagaggcaacaccc	-	*
GQ117030	Human H1N1pdm2009 IAVs	Human	H1N1	pdm	2009	USA	A/Texas/09/2009	ggtgatgccccactccttgatcggctccgccgagatca aaagtccttaaaaggaagaggcaacaccc	-	*
GQ160572	Human H1N1pdm2009 IAVs	Human	H1N1		2009	USA	A/Texas/22/2009	ggtgatgccccactccttgatcggctccgccgagatca aaagtccttaaaaggaagaggcaacaccc	-	*
CY075552	Human H1N1pdm2009 IAVs	Human	H1N1		2009	USA	A/Boston/648/2009	ggtgatgccccattcattgatcggctccgccgagatca aaagtccttaaaaggaagaggcaacaccc	-	-
CY075616	Human H1N1pdm2009 IAVs	Human	H1N1	pdm	2009	USA	A/Boston/682/2009	ggtgatgccccattcattgatcggctccgccgagatca aaagtccttaaaaggaagaggcaacaccc	-	-
CY062054	Human H1N1pdm2009 IAVs	Human	H1N1	pdm	2009	USA	A/New_York/0357/2009	ggtgatgccccattcattgatcggctccgccgagatca aaagtccttaaaaggaagaggcaacaccc	-	-
CY056559	Human H1N1pdm2009 IAVs	Human	H1N1	pdm	2009	USA	A/New_York/5199/2009	ggtgatgccccattcattgatcggctccgccgagatca aaagtccttaaaaggaagaggcaacaccc	-	-
CY056679	Human H1N1pdm2009 IAVs	Human	H1N1	pdm	2009	USA	A/New_York/6214/2009	ggtgatgccccattcattgatcggctccgccgagatca aaagtccttaaaaggaagaggcaacaccc	-	-
CY056735	Human H1N1pdm2009 IAVs	Human	H1N1	pdm	2009	USA	A/New_York/6546/2009	ggtgatgccccattcattgatcggctccgccgagatca aaagtccttaaaaggaagaggcaacaccc	-	-
CY056775	Human H1N1pdm2009 IAVs	Human	H1N1	pdm	2009	USA	A/New_York/6669/2009	ggtgatgccccattcattgatcggctccgccgagatca aaagtccttaaaaggaagaggcaacaccc	-	-
CY056823	Human H1N1pdm2009 IAVs	Human	H1N1	pdm	2009	USA	A/New_York/6810/2009	ggtgatgccccattcattgatcggctccgccgagatca aaagtccttaaaaggaagaggcaacaccc	-	-
CY072338	Human H1N1pdm2009 IAVs	Human	H1N1	pdm	2009	USA	A/Newark/INS319/2009	ggtgatgccccattcattgatcggctccgccgagatca aaagtccttaaaaggaagaggcaacaccc	-	-
CY062967	Human H1N1pdm2009 IAVs	Human	H1N1	pdm	2009	Spain	A/Terrassa/INS175/2009	ggtgatgccccattcattgatcggctccgccgagatca aaagtccttaaaaggaagaggcaacaccc	-	-
CY061023	Human H1N1pdm2009 IAVs	Human	H1N1	pdm	2009	USA	A/Texas/JMS388/2009	ggtgatgccccattcattgatcggctccgccgagatca aaagtccttaaaaggaagaggcaacaccc	-	-
KC781309	Human H1N1pdm2009 IAVs	Human	H1N1	pdm	2009	USA	A/Wisconsin/66/2009	ggtgatgccccattcctagatcggctccgccgagatca aaagtccttaaaaggaagaggcaacaccc	-	*
CY083938	Human H1N1pdm2009 IAVs	Human	H1N1	pdm	2009	Poland	A/Warsaw/INS149/2009	ggtgatgccccattcctagatcggctccgccgagatca aaagtccttaaaaggaagaggcaacaccc	-	*
CY072610	Human H1N1pdm2009 IAVs	Human	H1N1	pdm	2009	Nicaragua	A/Managua/2155.01/2009	ggtgatgccccattccttgaccggctccgccgagatca aaagtccttaaaaggaagaggcaacaccc	-	*
CY040027	Human H1N1pdm2009 IAVs	Human	H1N1	pdm	2009	USA	A/New_York/3049/2009	ggtgatgccccattccttgaccggctccgccgagatca aaagtccttaaaaggaagaggcaacaccc	-	*

CY051611	Human H1N1pdm2009 IAVs	Human	H1N1	pdm	2009	USA	A/New_York/4567/2009	ggtgatgccccattccttgaccggctccgccgagatca aaagtccttaaaaggaagaggcaacaccc	-	*
CY041794	Human H1N1pdm2009 IAVs	Human	H1N1	pdm	2009	USA	A/New_York/3463/2009	ggtgatgccccattccttgaccggctccgccgagatca aaagtccttaaaaggaagaggcaacaccc	-	*
CY072850	Human H1N1pdm2009 IAVs	Human	H1N1	pdm	2009	Nicaragua	A/Managua/4702.04/2009	ggtgatgccccattccttgaccggctccgccgagatca aaagtccttaaaaggaagaggcaacaccc	-	*
CY072706	Human H1N1pdm2009 IAVs	Human	H1N1	pdm	2009	Nicaragua	A/Managua/5036.01/2009	ggtgatgccccattccttgaccggctccgccgagatca aaagtccttaaaaggaagaggcaacaccc	-	*
CY069278	Human H1N1pdm2009 IAVs	Human	H1N1	pdm	2009	Nicaragua	A/Managua/5212.04/2009	ggtgatgccccattccttgaccggctccgccgagatca aaagtccttaaaaggaagaggcaacaccc	-	*
CY061263	Human H1N1pdm2009 IAVs	Human	H1N1	pdm	2009	USA	A/San_Diego/INS105/2009	ggtgatgccccattccttgaccggctccgccgagatca aaagtccttaaaaggaagaggcaacaccc	-	*
CY065706	Human H1N1pdm2009 IAVs	Human	H1N1	pdm	2009	United_Kingdom	A/England/430/2009	ggtgatgccccattccttgataggctccgccgagatca aaagtccttaaaaggaagaggcaacaccc	-	*
HM189591	Human H1N1pdm2009 IAVs	Swine	H1N1		2009	South_Korea	A/swine/Korea/SCJ01/2009	ggtgatgccccattccttgatcgactccgccgagatca aaagtccttaaaaggaagaggcaacaccc	-	-
HM189592	Human H1N1pdm2009 IAVs	Swine	H1N1		2009	South_Korea	A/swine/Korea/SCJ02/2009	ggtgatgccccattccttgatcgactccgccgagatca aaagtccttaaaaggaagaggcaacaccc	-	-
HM189595	Human H1N1pdm2009 IAVs	Swine	H1N1		2009	South_Korea	A/swine/Korea/SCJ05/2009	ggtgatgccccattccttgatcgactccgccgagatca aaagtccttaaaaggaagaggcaacaccc	-	-
HM189596	Human H1N1pdm2009 IAVs	Swine	H1N1		2009	South_Korea	A/swine/Korea/SCJ06/2009	ggtgatgccccattccttgatcgactccgccgagatca aaagtccttaaaaggaagaggcaacaccc	-	-
HM189593	Human H1N1pdm2009 IAVs	Swine	H1N1		2009	South_Korea	A/swine/Korea/SCJ03/2009	ggtgatgccccattccttgatcgactccgccgagatca aaagtccttaaaaggaagaggcaacaccc	-	-
HM189594	Human H1N1pdm2009 IAVs	Swine	H1N1		2009	South_Korea	A/swine/Korea/SCJ04/2009	ggtgatgccccattccttgatcgactccgccgagatca aaagtccttaaaaggaagaggcaacaccc	-	-
HM189597	Human H1N1pdm2009 IAVs	Swine	H1N1		2009	South_Korea	A/swine/Korea/SCJ07/2009	ggtgatgccccattccttgatcgactccgccgagatca aaagtccttaaaaggaagaggcaacaccc	-	-
CY100497	Human H1N1pdm2009 IAVs	Human	H1N1	pdm	2009	Mexico	A/Mexico_City/INER15/2009	ggtgatgccccattccttgatcggccnnnncgagatc aaaagtccttaaaaggaagaggcaacaccc	+	+
KJ023096	Human H1N1pdm2009 IAVs	Human	H1N1	pdm	2009	India	A/Goa/006/2009	ggtgatgccccattccttgatcggctacgccgagatca aaagtccttaaaaggaagaggcaacaccc	-	*
KJ023095	Human H1N1pdm2009 IAVs	Human	H1N1	pdm	2009	India	A/Uttar_Pradesh/005/2009	ggtgatgccccattccttgatcggctacgccgagatca aaagtccttaaaaggaagaggcaacaccc	-	*
KJ023097	Human H1N1pdm2009 IAVs	Human	H1N1	pdm	2009	India	A/Punjab/007/2009	ggtgatgccccattccttgatcggctacgccgagatca aaagtccttaaaaggaagaggcaacaccc	-	*
KJ023098	Human H1N1pdm2009 IAVs	Human	H1N1	pdm	2009	India	A/Gujarat/008/2009	ggtgatgccccattccttgatcggctacgccgagatca aaagtccttaaaaggaagaggcaacaccc	-	*
KJ023104	Human H1N1pdm2009 IAVs	Human	H1N1	pdm	2009	India	A/Uttrakhand/014/2009	ggtgatgccccattccttgatcggctacgccgagatca aaagtccttaaaaggaagaggcaacaccc	-	*
CY066211	Human H1N1pdm2009 IAVs	Human	H1N1	pdm	2009	USA	A/California/VRDL104/2009	ggtgatgccccattccttgatcggctccgcagagatca aaagtccttaaaaggaagaggcaacaccc	+	+
CY055968	Human H1N1pdm2009 IAVs	Human	H1N1	pdm	2009	USA	A/San_Diego/INS03/2009	ggtgatgccccattccttgatcggctccgcagagatca aaagtccttaaaaggaagaggcaacaccc	+	+
CY066531	Human H1N1pdm2009 IAVs	Human	H1N1	pdm	2009	USA	A/San_Diego/INS193/2009	ggtgatgccccattccttgatcggctccgcagagatca aaagtccttaaaaggaagaggcaacaccc	+	+
CY066547	Human H1N1pdm2009 IAVs	Human	H1N1	pdm	2009	USA	A/San_Diego/INS197/2009	ggtgatgccccattccttgatcggctccgcagagatca aaagtccttaaaaggaagaggcaacaccc	+	+
CY066803	Human H1N1pdm2009 IAVs	Human	H1N1	pdm	2009	USA	A/San_Diego/INS216/2009	ggtgatgccccattccttgatcggctccgcagagatca aaagtccttaaaaggaagaggcaacaccc	+	+

CY056232	Human H1N1pdm2009 IAVs	Human	H1N1		2009	USA	A/San_Diego/INS69/2009	ggtgatgccccattccttgatcggctccgcagagatca aaagtccttaaaaggaagaggcaacaccc	+	+
CY058472	Human H1N1pdm2009 IAVs	Human	H1N1		2009	USA	A/Wisconsin/629_D00337/2009	ggtgatgccccattccttgatcggctccgcagagatca aaagtccttaaaaggaagaggcaacaccc	+	+
CY083842	Human H1N1pdm2009 IAVs	Human	H1N1		2009	USA	A/San_Diego/INS14/2009	ggtgatgccccattccttgatcggctccgcagagatca aaagtccttaaaaggaagaggcaacaccc	+	+
KC782165	Human H1N1pdm2009 IAVs	Human	H1N1	pdm	2009	USA	A/New_Mexico/16/2009	ggtgatgccccattccttgatcggctccgcagagatca aaagtccttaaaaggaagaggcaacaccc	+	+
KC781516	Human H1N1pdm2009 IAVs	Human	H1N1	pdm	2009	USA	A/Utah/51/2009	ggtgatgccccattccttgatcggctccgcagagatca aaagtccttaaaaggaagaggcaacaccc	+	+
KJ023103	Human H1N1pdm2009 IAVs	Human	H1N1	pdm	2009	India	A/Jammu_and_Kashmir/013/2009	ggtgatgccccattccttgatcggctccgcagagatca aaagtccttaaaaggaagaggcaacaccc	+	+
CY111615	Human H1N1pdm2009 IAVs	Human	H1N1	pdm	2009	Hong_Kong	A/Hong_Kong/H090_661_V10/2009	ggtgatgccccattccttgatcggctccgccgaaatca aaagtccttaaaaggaagaggcaacaccc	-	*
CY120497	Human H1N1pdm2009 IAVs	Human	H1N1	pdm	2009	Hong_Kong	A/Hong_Kong/H090_661_V10/2009	ggtgatgccccattccttgatcggctccgccgaaatca aaagtccttaaaaggaagaggcaacaccc	-	*
CY054687	Human H1N1pdm2009 IAVs	Human	H1N1	pdm	2009	USA	A/Wisconsin/629_D00589/2009	ggtgatgccccattccttgatcggctccgccgaaatca aaagtccttaaaaggaagaggcaacaccc	-	*
CY090101	Human H1N1pdm2009 IAVs	Human	H1N1	pdm	2009	USA	A/Boston/706/2009	ggtgatgccccattccttgatcggctccgccgagatca aaaatccttaaaaggaagaggcaacaccc	-	*
CY089932	Human H1N1pdm2009 IAVs	Human	H1N1		2009	USA	A/Wisconsin/629_D00889/2009	ggtgatgccccattccttgatcggctccgccgagatca aaaatccttaaaaggaagaggcaacaccc	-	*
CY067035	Human H1N1pdm2009 IAVs	Human	H1N1	pdm	2009	Denmark	A/Aarhus/INS251/2009	ggtgatgccccattccttgatcggctccgccgagatca aaagtcattaaaaggaagaggcaacaccc	-	*
CY072258	Human H1N1pdm2009 IAVs	Human	H1N1	pdm	2009	Denmark	A/Odense/INS308/2009	ggtgatgccccattccttgatcggctccgccgagatca aaagtcattaaaaggaagaggcaacaccc	-	*
CY088677	Human H1N1pdm2009 IAVs	Human	H1N1	pdm	2009	India	A/Delhi/NIV3610/2009	ggtgatgccccattccttgatcggctccgccgagatca aaagtcattaaaaggaagaggcaacaccc	-	*
HM567740	Human H1N1pdm2009 IAVs	Human	H1N1	pdm	2009	United_Kingdom	A/England/366/2009	ggtgatgccccattccttgatcggctccgccgagatca aaagtcattaaaaggaagaggcaacaccc	-	*
HM567828	Human H1N1pdm2009 IAVs	Human	H1N1	pdm	2009	Ireland	A/Ireland/10/2009	ggtgatgccccattccttgatcggctccgccgagatca aaagtcattaaaaggaagaggcaacaccc	-	*
HM567820	Human H1N1pdm2009 IAVs	Human	H1N1	pdm	2009	Ireland	A/Ireland/7/2009	ggtgatgccccattccttgatcggctccgccgagatca aaagtcattaaaaggaagaggcaacaccc	-	*
HM773214	Human H1N1pdm2009 IAVs	Human	H1N1	pdm	2009	China	A/Jiangsu/2/2009	ggtgatgccccattccttgatcggctccgccgagatca aaagtcattaaaaggaagaggcaacaccc	-	*
HQ652624	Human H1N1pdm2009 IAVs	Human	H1N1	pdm	2009	China	A/Jiangsu/S61/2009	ggtgatgccccattccttgatcggctccgccgagatca aaagtcattaaaaggaagaggcaacaccc	-	*
HQ652625	Human H1N1pdm2009 IAVs	Human	H1N1	pdm	2009	China	A/Jiangsu/S62/2009	ggtgatgccccattccttgatcggctccgccgagatca aaagtcattaaaaggaagaggcaacaccc	-	*
HM754230	Human H1N1pdm2009 IAVs	Human	H1N1	pdm	2009	China	A/Jiangsu/1/2009	ggtgatgccccattccttgatcggctccgccgagatca aaagtcattaaaaggaagaggcaacaccc	-	*
HM855263	Human H1N1pdm2009 IAVs	Human	H1N1	pdm	2009	Kenya	A/Mombasa/148/2009	ggtgatgccccattccttgatcggctccgccgagatca aaagtcattaaaaggaagaggcaacaccc	-	*
CY095895	Human H1N1pdm2009 IAVs	Human	H1N1	pdm	2009	China	A/Zhejiang/78/2009	ggtgatgccccattccttgatcggctccgccgagatca aaagtccataaaaggaagaggcaacaccc	-	*
CY055649	Human H1N1pdm2009 IAVs	Human	H1N1	pdm	2009	Australia	A/Australia/26/2009	ggtgatgccccattccttgatcggctccgccgagatca aaagtccctaaaaggaagaggcaacaccc	-	*
CY069761	Human H1N1pdm2009 IAVs	Human	H1N1	pdm	2009	United_Kingdom	A/England/633/2009	ggtgatgccccattccttgatcggctccgccgagatca aaagtccctaaaaggaagaggcaacaccc	-	*

CY065178	Human H1N1pdm2009 IAVs	Human	H1N1	pdm	2009	United_Kingdom	A/England/634/2009	ggtgatgccccattccttgatcggctccgccgagatca<br>aaagtccctaaaaggaagaggcaacaccc	-	*
JX625431	Human H1N1pdm2009 IAVs	Human	H1N1	pdm	2009	United_Kingdom	A/England/92920018/2009	ggtgatgccccattccttgatcggctccgccgagatca<br>aaagtccctaaaaggaagaggcaacaccc	-	*
CY123510	Human H1N1pdm2009 IAVs	Human	H1N1	pdm	2009	Singapore	A/Singapore/ON1760/2009	ggtgatgccccattccttgatcggctccgccgagatca<br>aaagtccctaaaaggaagaggcaacaccc	-	*
CY051715	Human H1N1pdm2009 IAVs	Human	H1N1	pdm	2009	USA	A/New_York/4787/2009	ggtgatgccccattccttgatcggctccgccgagatca<br>aaagtccctaaaaggaagaggcaacaccc	-	*
CY051731	Human H1N1pdm2009 IAVs	Human	H1N1	pdm	2009	USA	A/New_York/4790/2009	ggtgatgccccattccttgatcggctccgccgagatca<br>aaagtccctaaaaggaagaggcaacaccc	-	*
CY051739	Human H1N1pdm2009 IAVs	Human	H1N1	pdm	2009	USA	A/New_York/4791/2009	ggtgatgccccattccttgatcggctccgccgagatca<br>aaagtccctaaaaggaagaggcaacaccc	-	*
CY051747	Human H1N1pdm2009 IAVs	Human	H1N1	pdm	2009	USA	A/New_York/4810/2009	ggtgatgccccattccttgatcggctccgccgagatca<br>aaagtccctaaaaggaagaggcaacaccc	-	*
CY041641	Human H1N1pdm2009 IAVs	Human	H1N1	pdm	2009	USA	A/New_York/3307/2009	ggtgatgccccattccttgatcggctccgccgagatca<br>aaagtccttaaaaagaagaggcaacaccc	-	*
CY053732	Human H1N1pdm2009 IAVs	Human	H1N1		2009	Russia	A/Russia/74/2009	ggtgatgccccattccttgatcggctccgccgagatca<br>aaagtccttaaaaagaagaggcaacaccc	-	*
GQ122093	Human H1N1pdm2009 IAVs	Human	H1N1	pdm	2009	USA	A/Texas/15/2009	ggtgatgccccattccttgatcggctccgccgagatca<br>aaagtccttaaaaagaagaggcaacaccc	-	*
CY058544	Human H1N1pdm2009 IAVs	Human	H1N1	pdm	2009	Nicaragua	A/Managua/462.01/2009	ggtgatgccccattccttgatcggctccgccgagatca<br>aaagtccttaaaaggaagaagcaacaccc	-	-
CY072986	Human H1N1pdm2009 IAVs	Human	H1N1	pdm	2009	Nicaragua	A/Managua/5273.02/2009	ggtgatgccccattccttgatcggctccgccgagatca<br>aaagtccttaaaaggaagaagcaacaccc	-	-
CY066971	Human H1N1pdm2009 IAVs	Human	H1N1	pdm	2009	Denmark	A/Aarhus/INS241/2009	ggtgatgccccattccttgatcggctccgccgagatca<br>aaagtccttaaaaggaagaagcaacaccc	-	-
CY064744	Human H1N1pdm2009 IAVs	Human	H1N1	pdm	2009	Mexico	A/Mexico_City/024/2009	ggtgatgccccattccttgatcggctccgccgagatca<br>aaagtccttaaaaggaagaggaaacaccc	-	*
GQ463204	Human H1N1pdm2009 IAVs	Human	H1N1	pdm	2009	China	A/Hunan/SWL3/2009	ggtgatgccccattccttgatcggctccgccgagatca<br>aaagtccttaaaaggaagaggcaaaaccc	-	*
JN375247	Human H1N1pdm2009 IAVs	Swine	H1N1		2009	China	A/swine/Guangdong/106/2009	ggtgatgccccattccttgatcggctccgccgagatca<br>aaagtccttaaaaggaagaggcaacaacc	-	*
JN375248	Human H1N1pdm2009 IAVs	Swine	H1N1		2009	China	A/swine/Guangdong/114/2009	ggtgatgccccattccttgatcggctccgccgagatca<br>aaagtccttaaaaggaagaggcaacaacc	-	*
JN375246	Human H1N1pdm2009 IAVs	Swine	H1N1		2009	China	A/swine/Guangdong/94/2009	ggtgatgccccattccttgatcggctccgccgagatca<br>aaagtccttaaaaggaagaggcaacaacc	-	*
GQ323539	Human H1N1pdm2009 IAVs	Human	H1N1	pdm	2009	USA	A/Texas/35/2009	ggtgatgccccattccttgatcggctccgccgagatca<br>aaagtccttaaaaggaagaggcaacaacc	-	*
CY051051	Human H1N1pdm2009 IAVs	Human	H1N1	pdm	2009	USA	A/Wisconsin/629_D00008/2009	ggtgatgccccattccttgatcggctccgccgagatca<br>aaagtccttaaaaggaagaggcaacacca	-	*
CY046503	Human H1N1pdm2009 IAVs	Human	H1N1	pdm	2009	USA	A/Wisconsin/629_D00378/2009	ggtgatgccccattccttgatcggctccgccgagatca<br>aaagtccttaaaaggaagaggcaacacca	-	*
CY046887	Human H1N1pdm2009 IAVs	Human	H1N1	pdm	2009	USA	A/Wisconsin/629_D00498/2009	ggtgatgccccattccttgatcggctccgccgagatca<br>aaagtccttaaaaggaagaggcaacacca	-	*
CY046215	Human H1N1pdm2009 IAVs	Human	H1N1	pdm	2009	USA	A/Wisconsin/629_D00869/2009	ggtgatgccccattccttgatcggctccgccgagatca<br>aaagtccttaaaaggaagaggcaacacca	-	*
CY046687	Human H1N1pdm2009 IAVs	Human	H1N1	pdm	2009	USA	A/Wisconsin/629_D00978/2009	ggtgatgccccattccttgatcggctccgccgagatca<br>aaagtccttaaaaggaagaggcaacacca	-	*
CY050384	Human H1N1pdm2009 IAVs	Human	H1N1	pdm	2009	USA	A/Wisconsin/629_D01295/2009	ggtgatgccccattccttgatcggctccgccgagatca<br>aaagtccttaaaaggaagaggcaacacca	-	*

CY046599	Human H1N1pdm2009 IAVs	Human	H1N1	pdm	2009	USA	A/Wisconsin/629_D01482/2009	ggtgatgccccattccttgatcggctccgccgagatca aaagtccttaaaaggaagaggcaacacca	-	*
CY050448	Human H1N1pdm2009 IAVs	Human	H1N1	pdm	2009	USA	A/Wisconsin/629_D01482/2009	ggtgatgccccattccttgatcggctccgccgagatca aaagtccttaaaaggaagaggcaacacca	-	*
CY046359	Human H1N1pdm2009 IAVs	Human	H1N1	pdm	2009	USA	A/Wisconsin/629_D01529/2009	ggtgatgccccattccttgatcggctccgccgagatca aaagtccttaaaaggaagaggcaacacca	-	*
CY046239	Human H1N1pdm2009 IAVs	Human	H1N1	pdm	2009	USA	A/Wisconsin/629_D01735/2009	ggtgatgccccattccttgatcggctccgccgagatca aaagtccttaaaaggaagaggcaacacca	-	*
CY046711	Human H1N1pdm2009 IAVs	Human	H1N1	pdm	2009	USA	A/Wisconsin/629_D01787/2009	ggtgatgccccattccttgatcggctccgccgagatca aaagtccttaaaaggaagaggcaacacca	-	*
CY046295	Human H1N1pdm2009 IAVs	Human	H1N1	pdm	2009	USA	A/Wisconsin/629_D01817/2009	ggtgatgccccattccttgatcggctccgccgagatca aaagtccttaaaaggaagaggcaacacca	-	*
CY050392	Human H1N1pdm2009 IAVs	Human	H1N1		2009	USA	A/Wisconsin/629_D01839/2009	ggtgatgccccattccttgatcggctccgccgagatca aaagtccttaaaaggaagaggcaacacca	-	*
CY046631	Human H1N1pdm2009 IAVs	Human	H1N1	pdm	2009	USA	A/Wisconsin/629_D01839/2009	ggtgatgccccattccttgatcggctccgccgagatca aaagtccttaaaaggaagaggcaacacca	-	*
CY051427	Human H1N1pdm2009 IAVs	Human	H1N1	pdm	2009	USA	A/Wisconsin/629_S0279/2009	ggtgatgccccattccttgatcggctccgccgagatca aaagtccttaaaaggaagaggcaacacca	-	*
CY050146	Human H1N1pdm2009 IAVs	Human	H1N1	pdm	2009	USA	A/Wisconsin/629_D01058/2009	ggtgatgccccattccttgatcggctccgccgagatca aaagtccttaaaaggaagaggcaacacca	-	*
CY123897	Human H1N1pdm2009 IAVs	Human	H1N1	pdm	2009	Singapore	A/Singapore/ON2416/2009	ggtgatgccccattccttgatcggctccgccgagatca aaagtccttaaaaggaagaggcaacacca	-	*
CY083914	Human H1N1pdm2009 IAVs	Human	H1N1	pdm	2009	Denmark	A/Aalborg/INS132/2009	ggtgatgccccattccttgatcggctccgccgagatca aaagtccttaaaaggaagaggcaacaccc	-	*
CY062703	Human H1N1pdm2009 IAVs	Human	H1N1	pdm	2009	Denmark	A/Aarhus/INS118/2009	ggtgatgccccattccttgatcggctccgccgagatca aaagtccttaaaaggaagaggcaacaccc	-	*
CY066979	Human H1N1pdm2009 IAVs	Human	H1N1	pdm	2009	Denmark	A/Aarhus/INS242/2009	ggtgatgccccattccttgatcggctccgccgagatca aaagtccttaaaaggaagaggcaacaccc	-	*
CY071083	Human H1N1pdm2009 IAVs	Human	H1N1	pdm	2009	Germany	A/Bochum/INS375/2009	ggtgatgccccattccttgatcggctccgccgagatca aaagtccttaaaaggaagaggcaacaccc	-	*
CY071091	Human H1N1pdm2009 IAVs	Human	H1N1	pdm	2009	Germany	A/Bochum/INS376/2009	ggtgatgccccattccttgatcggctccgccgagatca aaagtccttaaaaggaagaggcaacaccc	-	*
CY083563	Human H1N1pdm2009 IAVs	Human	H1N1	pdm	2009	Hungary	A/Budapest/WRAIR2411N/2009	ggtgatgccccattccttgatcggctccgccgagatca aaagtccttaaaaggaagaggcaacaccc	-	*
CY069222	Human H1N1pdm2009 IAVs	Human	H1N1		2009	Germany	A/Munich/INS363/2009	ggtgatgccccattccttgatcggctccgccgagatca aaagtccttaaaaggaagaggcaacaccc	-	*
CY072250	Human H1N1pdm2009 IAVs	Human	H1N1	pdm	2009	Denmark	A/Odense/INS307/2009	ggtgatgccccattccttgatcggctccgccgagatca aaagtccttaaaaggaagaggcaacaccc	-	*
CY088625	Human H1N1pdm2009 IAVs	Human	H1N1	pdm	2009	Denmark	A/Odense/INS176/2009	ggtgatgccccattccttgatcggctccgccgagatca aaagtccttaaaaggaagaggcaacaccc	-	*
CY072226	Human H1N1pdm2009 IAVs	Human	H1N1	pdm	2009	Germany	A/Frankfurt/INS302/2009	ggtgatgccccattccttgatcggctccgccgagatca aaagtccttaaaaggaagaggcaacaccc	-	*
CY069230	Human H1N1pdm2009 IAVs	Human	H1N1	pdm	2009	Germany	A/Munich/INS364/2009	ggtgatgccccattccttgatcggctccgccgagatca aaagtccttaaaaggaagaggcaacaccc	-	*
CY123801	Human H1N1pdm2009 IAVs	Human	H1N1	pdm	2009	Singapore	A/Singapore/ON2180/2009	ggtgatgccccattccttgatcggctccgccgagatca aaagtccttaaaaggaagaggcaacaccc	-	*
CY118271	Human H1N1pdm2009 IAVs	Human	H1N1	pdm	2009	Malaysia	A/Malaysia/2205822/2009	ggtgatgccccattccttgatcggctccgccgagatca aaagtccttaaaaggaagaggcaacaccc	-	*
CY045238	Human H1N1pdm2009 IAVs	Human	H1N1	pdm	2009	Taiwan	A/Taiwan/126/2009	ggtgatgccccattccttgatcggctccgccgagatca aaagtccttaaaaggaagaggcaacaccc	-	*

CY069623	Human H1N1pdm2009 IAVs	Human	H1N1	pdm	2009	Singapore	A/Singapore/276/2009	ggtgatgccccattccttgatcggctccgccgagatca aaagtccttaaaaggaagaggcaacaccc	-	*
CY158261	Human H1N1pdm2009 IAVs	Swine	H1N1		2009	USA	A/swine/Arkansas/SG1321/2009	ggtgatgccccattccttgatcggctccgccgagatca aaagtccttaaaaggaagaggcaacaccc	-	*
CY071067	Human H1N1pdm2009 IAVs	Human	H1N1	pdm	2009	Estonia	A/Tallinn/INS372/2009	ggtgatgccccattccttgatcggctccgccgagatca aaagtccttaaaaggaagaggcaacaccc	-	*
CY052899	Human H1N1pdm2009 IAVs	Human	H1N1	pdm	2009	USA	A/Texas/45102952/2009	ggtgatgccccattccttgatcggctccgccgagatca aaagtccttaaaaggaagaggcaacaccc	-	*
CY052835	Human H1N1pdm2009 IAVs	Human	H1N1	pdm	2009	USA	A/Texas/45131305/2009	ggtgatgccccattccttgatcggctccgccgagatca aaagtccttaaaaggaagaggcaacaccc	-	*
CY069190	Human H1N1pdm2009 IAVs	Human	H1N1	pdm	2009	Greece	A/Athens/INS357/2009	ggtgatgccccattccttgatcggctccgccgagatca aaagtccttaaaaggaagaggcaacaccc	-	*
CY066587	Human H1N1pdm2009 IAVs	Human	H1N1	pdm	2009	USA	A/San_Diego/INS203/2009	ggtgatgccccattccttgatcggctccgccgagatca aaagtccttaaaaggaagaggcaacaccc	-	*
KF411265	Human H1N1pdm2009 IAVs	Human	H1N1	pdm	2009	China	A/Qingdao/1364/2009	ggtgatgccccattccttgatcggctccgccgagatca aaagtccttaaaaggaagaggcaacaccc	-	*
KF411269	Human H1N1pdm2009 IAVs	Human	H1N1	pdm	2009	China	A/Qingdao/1577/2009	ggtgatgccccattccttgatcggctccgccgagatca aaagtccttaaaaggaagaggcaacaccc	-	*
HQ541692	Human H1N1pdm2009 IAVs	Swine	H1N1		2009	China	A/swine/Heilongjiang/44/2009	ggtgatgccccattccttgatcggctccgccgagatca aaagtccttaaaaggaagaggcaacaccc	-	*
HM855244	Human H1N1pdm2009 IAVs	Human	H1N1	pdm	2009	Kenya	A/Eldoret/120/2009	ggtgatgccccattccttgatcggctccgccgagatca aaagtccttaaaaggaagaggcaacaccc	-	*
CY061087	Human H1N1pdm2009 IAVs	Human	H1N1	pdm	2009	USA	A/Texas/JMS397/2009	ggtgatgccccattccttgatcggctccgccgagatca aaagtccttaaaaggaagaggcaacaccc	-	*
CY061095	Human H1N1pdm2009 IAVs	Human	H1N1	pdm	2009	USA	A/Texas/JMS398/2009	ggtgatgccccattccttgatcggctccgccgagatca aaagtccttaaaaggaagaggcaacaccc	-	*
CY061103	Human H1N1pdm2009 IAVs	Human	H1N1	pdm	2009	USA	A/Texas/JMS399/2009	ggtgatgccccattccttgatcggctccgccgagatca aaagtccttaaaaggaagaggcaacaccc	-	*
CY061574	Human H1N1pdm2009 IAVs	Human	H1N1	pdm	2009	USA	A/Texas/JMS400/2009	ggtgatgccccattccttgatcggctccgccgagatca aaagtccttaaaaggaagaggcaacaccc	-	*
CY069254	Human H1N1pdm2009 IAVs	Human	H1N1	pdm	2009	Nicaragua	A/Managua/4943.09/2009	ggtgatgccccattccttgatcggctccgccgagatca aaagtccttaaaaggaagaggcaacaccc	-	*
HM855247	Human H1N1pdm2009 IAVs	Human	H1N1	pdm	2009	Kenya	A/Kisumu/61/2009	ggtgatgccccattccttgatcggctccgccgagatca aaagtccttaaaaggaagaggcaacaccc	-	*
HQ165791	Human H1N1pdm2009 IAVs	Human	H1N1	pdm	2009	Kenya	A/Nairobi/64/2009	ggtgatgccccattccttgatcggctccgccgagatca aaagtccttaaaaggaagaggcaacaccc	-	*
CY073485	Human H1N1pdm2009 IAVs	Human	H1N1	pdm	2009	Germany	A/Bochum/INS248/2009	ggtgatgccccattccttgatcggctccgccgagatca aaagtccttaaaaggaagaggcaacaccc	-	*
CY062743	Human H1N1pdm2009 IAVs	Human	H1N1	pdm	2009	Germany	A/Bonn/INS127/2009	ggtgatgccccattccttgatcggctccgccgagatca aaagtccttaaaaggaagaggcaacaccc	-	*
CY064480	Human H1N1pdm2009 IAVs	Human	H1N1	pdm	2009	USA	A/Boston/106/2009	ggtgatgccccattccttgatcggctccgccgagatca aaagtccttaaaaggaagaggcaacaccc	-	*
CY064576	Human H1N1pdm2009 IAVs	Human	H1N1	pdm	2009	USA	A/Boston/125/2009	ggtgatgccccattccttgatcggctccgccgagatca aaagtccttaaaaggaagaggcaacaccc	-	*
CY064648	Human H1N1pdm2009 IAVs	Human	H1N1	pdm	2009	USA	A/Boston/135/2009	ggtgatgccccattccttgatcggctccgccgagatca aaagtccttaaaaggaagaggcaacaccc	-	*
CY064656	Human H1N1pdm2009 IAVs	Human	H1N1	pdm	2009	USA	A/Boston/137/2009	ggtgatgccccattccttgatcggctccgccgagatca aaagtccttaaaaggaagaggcaacaccc	-	*
CY064520	Human H1N1pdm2009 IAVs	Human	H1N1	pdm	2009	USA	A/Boston/117/2009	ggtgatgccccattccttgatcggctccgccgagatca aaagtccttaaaaggaagaggcaacaccc	-	*

CY058592	Human H1N1pdm2009 IAVs	Human	H1N1		2009	Nicaragua	A/Managua/65.02/2009	ggtgatgccccattccttgatcggctccgccgagatca aaagtccttaaaaggaagaggcaacaccc	-	*
CY120727	Human H1N1pdm2009 IAVs	Human	H1N1	pdm	2009	Brazil	A/Brazil/AVS04/2009	ggtgatgccccattccttgatcggctccgccgagatca aaagtccttaaaaggaagaggcaacaccc	-	*
CY120767	Human H1N1pdm2009 IAVs	Human	H1N1	pdm	2009	Brazil	A/Brazil/AVS05/2009	ggtgatgccccattccttgatcggctccgccgagatca aaagtccttaaaaggaagaggcaacaccc	-	*
CY052230	Human H1N1pdm2009 IAVs	Human	H1N1	pdm	2009	USA	A/Texas/44302551/2009	ggtgatgccccattccttgatcggctccgccgagatca aaagtccttaaaaggaagaggcaacaccc	-	*
CY052819	Human H1N1pdm2009 IAVs	Human	H1N1	pdm	2009	USA	A/Texas/45072656/2009	ggtgatgccccattccttgatcggctccgccgagatca aaagtccttaaaaggaagaggcaacaccc	-	*
HQ695924	Human H1N1pdm2009 IAVs	Human	H1N1	pdm	2009	Taiwan	A/Taiwan/14/2009	ggtgatgccccattccttgatcggctccgccgagatca aaagtccttaaaaggaagaggcaacaccc	-	*
GQ323440	Human H1N1pdm2009 IAVs	Human	H1N1	pdm	2009	USA	A/Texas/19/2009	ggtgatgccccattccttgatcggctccgccgagatca aaagtccttaaaaggaagaggcaacaccc	-	*
CY058704	Human H1N1pdm2009 IAVs	Human	H1N1	pdm	2009	USA	A/Texas/46192760/2009	ggtgatgccccattccttgatcggctccgccgagatca aaagtccttaaaaggaagaggcaacaccc	-	*
KC780856	Human H1N1pdm2009 IAVs	Human	H1N1	pdm	2009	USA	A/Wyoming/16/2009	ggtgatgccccattccttgatcggctccgccgagatca aaagtccttaaaaggaagaggcaacaccc	-	*
CY060690	Human H1N1pdm2009 IAVs	Human	H1N1	pdm	2009	Canada	A/Ontario/315181/2009	ggtgatgccccattccttgatcggctccgccgagatca aaagtccttaaaaggaagaggcaacaccc	-	*
CY122591	Human H1N1pdm2009 IAVs	Human	H1N1	pdm	2009	Singapore	A/Singapore/GP1092/2009	ggtgatgccccattccttgatcggctccgccgagatca aaagtccttaaaaggaagaggcaacaccc	-	*
CY122599	Human H1N1pdm2009 IAVs	Human	H1N1	pdm	2009	Singapore	A/Singapore/GP1094/2009	ggtgatgccccattccttgatcggctccgccgagatca aaagtccttaaaaggaagaggcaacaccc	-	*
CY122727	Human H1N1pdm2009 IAVs	Human	H1N1	pdm	2009	Singapore	A/Singapore/GP1162/2009	ggtgatgccccattccttgatcggctccgccgagatca aaagtccttaaaaggaagaggcaacaccc	-	*
CY123426	Human H1N1pdm2009 IAVs	Human	H1N1	pdm	2009	Singapore	A/Singapore/ON1095/2009	ggtgatgccccattccttgatcggctccgccgagatca aaagtccttaaaaggaagaggcaacaccc	-	*
CY124055	Human H1N1pdm2009 IAVs	Human	H1N1	pdm	2009	Singapore	A/Singapore/ON800/2009	ggtgatgccccattccttgatcggctccgccgagatca aaagtccttaaaaggaagaggcaacaccc	-	*
CY061737	Human H1N1pdm2009 IAVs	Swine	H1N1		2009	Hong_Kong	A/swine/Hong_Kong/2299/2009	ggtgatgccccattccttgatcggctccgccgagatca aaagtccttaaaaggaagaggcaacaccc	-	*
CY061729	Human H1N1pdm2009 IAVs	Swine	H1N1		2009	Hong_Kong	A/swine/Hong_Kong/NS1583/2009	ggtgatgccccattccttgatcggctccgccgagatca aaagtccttaaaaggaagaggcaacaccc	-	*
CY056152	Human H1N1pdm2009 IAVs	Human	H1N1		2009	USA	A/Pensacola/INS37/2009	ggtgatgccccattccttgatcggctccgccgagatca aaagtccttaaaaggaagaggcaacaccc	-	*
CY073122	Human H1N1pdm2009 IAVs	Human	H1N1		2009	Russia	A/Moscow/WRAIR1632N/2009	ggtgatgccccattccttgatcggctccgccgagatca aaagtccttaaaaggaagaggcaacaccc	-	*
CY063610	Human H1N1pdm2009 IAVs	Human	H1N1	pdm	2009	Denmark	A/Aalborg/INS133/2009	ggtgatgccccattccttgatcggctccgccgagatca aaagtccttaaaaggaagaggcaacaccc	-	*
CY066379	Human H1N1pdm2009 IAVs	Human	H1N1	pdm	2009	USA	A/California/VRDL125/2009	ggtgatgccccattccttgatcggctccgccgagatca aaagtccttaaaaggaagaggcaacaccc	-	*
CY055023	Human H1N1pdm2009 IAVs	Human	H1N1	pdm	2009	USA	A/California/VRDL67/2009	ggtgatgccccattccttgatcggctccgccgagatca aaagtccttaaaaggaagaggcaacaccc	-	*
CY055482	Human H1N1pdm2009 IAVs	Human	H1N1		2009	USA	A/California/VRDL72/2009	ggtgatgccccattccttgatcggctccgccgagatca aaagtccttaaaaggaagaggcaacaccc	-	*
CY063023	Human H1N1pdm2009 IAVs	Human	H1N1	pdm	2009	Spain	A/Madrid/INS184/2009	ggtgatgccccattccttgatcggctccgccgagatca aaagtccttaaaaggaagaggcaacaccc	-	*
CY066843	Human H1N1pdm2009 IAVs	Human	H1N1	pdm	2009	Spain	A/Madrid/INS223/2009	ggtgatgccccattccttgatcggctccgccgagatca aaagtccttaaaaggaagaggcaacaccc	-	*

CY107243	Human H1N1pdm2009 IAVs	Human	H1N1	pdm	2009	United_Kingdom	A/Scotland/Aberdeen_13/2009	ggtgatgccccattccttgatcggctccgccgagatca aaagtccttaaaaggaagaggcaacaccc	-	*
CY107250	Human H1N1pdm2009 IAVs	Human	H1N1	pdm	2009	United_Kingdom	A/Scotland/Aberdeen_14/2009	ggtgatgccccattccttgatcggctccgccgagatca aaagtccttaaaaggaagaggcaacaccc	-	*
CY107344	Human H1N1pdm2009 IAVs	Human	H1N1	pdm	2009	United_Kingdom	A/Scotland/Dundee_09V500203/2009	ggtgatgccccattccttgatcggctccgccgagatca aaagtccttaaaaggaagaggcaacaccc	-	*
CY107724	Human H1N1pdm2009 IAVs	Human	H1N1	pdm	2009	United_Kingdom	A/Scotland/Glasgow_444590/2009	ggtgatgccccattccttgatcggctccgccgagatca aaagtccttaaaaggaagaggcaacaccc	-	*
CY107506	Human H1N1pdm2009 IAVs	Human	H1N1	pdm	2009	United_Kingdom	A/Scotland/Livingston_20263/2009	ggtgatgccccattccttgatcggctccgccgagatca aaagtccttaaaaggaagaggcaacaccc	-	*
CY058432	Human H1N1pdm2009 IAVs	Human	H1N1		2009	USA	A/Wisconsin/629_D01752/2009	ggtgatgccccattccttgatcggctccgccgagatca aaagtccttaaaaggaagaggcaacaccc	-	*
CY073729	Human H1N1pdm2009 IAVs	Human	H1N1	pdm	2009	Denmark	A/Aalborg/INS283/2009	ggtgatgccccattccttgatcggctccgccgagatca aaagtccttaaaaggaagaggcaacaccc	-	*
CY066963	Human H1N1pdm2009 IAVs	Human	H1N1	pdm	2009	Denmark	A/Aarhus/INS240/2009	ggtgatgccccattccttgatcggctccgccgagatca aaagtccttaaaaggaagaggcaacaccc	-	*
CY067043	Human H1N1pdm2009 IAVs	Human	H1N1	pdm	2009	Denmark	A/Aarhus/INS252/2009	ggtgatgccccattccttgatcggctccgccgagatca aaagtccttaaaaggaagaggcaacaccc	-	*
CY066827	Human H1N1pdm2009 IAVs	Human	H1N1	pdm	2009	Belgium	A/Antwerp/INS221/2009	ggtgatgccccattccttgatcggctccgccgagatca aaagtccttaaaaggaagaggcaacaccc	-	*
CY047807	Human H1N1pdm2009 IAVs	Human	H1N1	pdm	2009	Argentina	A/Argentina/7953/2009	ggtgatgccccattccttgatcggctccgccgagatca aaagtccttaaaaggaagaggcaacaccc	-	*
CY047815	Human H1N1pdm2009 IAVs	Human	H1N1	pdm	2009	Argentina	A/Argentina/7967/2009	ggtgatgccccattccttgatcggctccgccgagatca aaagtccttaaaaggaagaggcaacaccc	-	*
CY047823	Human H1N1pdm2009 IAVs	Human	H1N1	pdm	2009	Argentina	A/Argentina/7980/2009	ggtgatgccccattccttgatcggctccgccgagatca aaagtccttaaaaggaagaggcaacaccc	-	*
CY047831	Human H1N1pdm2009 IAVs	Human	H1N1	pdm	2009	Argentina	A/Argentina/8019/2009	ggtgatgccccattccttgatcggctccgccgagatca aaagtccttaaaaggaagaggcaacaccc	-	*
CY047839	Human H1N1pdm2009 IAVs	Human	H1N1	pdm	2009	Argentina	A/Argentina/8551/2009	ggtgatgccccattccttgatcggctccgccgagatca aaagtccttaaaaggaagaggcaacaccc	-	*
CY047847	Human H1N1pdm2009 IAVs	Human	H1N1	pdm	2009	Argentina	A/Argentina/8574/2009	ggtgatgccccattccttgatcggctccgccgagatca aaagtccttaaaaggaagaggcaacaccc	-	*
CY047863	Human H1N1pdm2009 IAVs	Human	H1N1	pdm	2009	Argentina	A/Argentina/8989/2009	ggtgatgccccattccttgatcggctccgccgagatca aaagtccttaaaaggaagaggcaacaccc	-	*
CY047871	Human H1N1pdm2009 IAVs	Human	H1N1	pdm	2009	Argentina	A/Argentina/8994/2009	ggtgatgccccattccttgatcggctccgccgagatca aaagtccttaaaaggaagaggcaacaccc	-	*
CY047879	Human H1N1pdm2009 IAVs	Human	H1N1	pdm	2009	Argentina	A/Argentina/9004/2009	ggtgatgccccattccttgatcggctccgccgagatca aaagtccttaaaaggaagaggcaacaccc	-	*
CY047959	Human H1N1pdm2009 IAVs	Human	H1N1	pdm	2009	Argentina	A/Argentina/9705/2009	ggtgatgccccattccttgatcggctccgccgagatca aaagtccttaaaaggaagaggcaacaccc	-	*
CY047975	Human H1N1pdm2009 IAVs	Human	H1N1	pdm	2009	Argentina	A/Argentina/9721/2009	ggtgatgccccattccttgatcggctccgccgagatca aaagtccttaaaaggaagaggcaacaccc	-	*
CY073978	Human H1N1pdm2009 IAVs	Human	H1N1	pdm	2009	Argentina	A/Argentina/HNRG104/2009	ggtgatgccccattccttgatcggctccgccgagatca aaagtccttaaaaggaagaggcaacaccc	-	*
CY073999	Human H1N1pdm2009 IAVs	Human	H1N1	pdm	2009	Argentina	A/Argentina/HNRG107/2009	ggtgatgccccattccttgatcggctccgccgagatca aaagtccttaaaaggaagaggcaacaccc	-	*
CY074004	Human H1N1pdm2009 IAVs	Human	H1N1	pdm	2009	Argentina	A/Argentina/HNRG14/2009	ggtgatgccccattccttgatcggctccgccgagatca aaagtccttaaaaggaagaggcaacaccc	-	*
CY074014	Human H1N1pdm2009 IAVs	Human	H1N1	pdm	2009	Argentina	A/Argentina/HNRG21/2009	ggtgatgccccattccttgatcggctccgccgagatca aaagtccttaaaaggaagaggcaacaccc	-	*

CY074024	Human H1N1pdm2009 IAVs	Human	H1N1	pdm	2009	Argentina	A/Argentina/HNRG32/2009	ggtgatgccccattccttgatcggctccgccgagatca aaagtccttaaaaggaagaggcaacaccc	-	*
CY074029	Human H1N1pdm2009 IAVs	Human	H1N1	pdm	2009	Argentina	A/Argentina/HNRG33/2009	ggtgatgccccattccttgatcggctccgccgagatca aaagtccttaaaaggaagaggcaacaccc	-	*
CY074034	Human H1N1pdm2009 IAVs	Human	H1N1	pdm	2009	Argentina	A/Argentina/HNRG36/2009	ggtgatgccccattccttgatcggctccgccgagatca aaagtccttaaaaggaagaggcaacaccc	-	*
CY074044	Human H1N1pdm2009 IAVs	Human	H1N1	pdm	2009	Argentina	A/Argentina/HNRG45/2009	ggtgatgccccattccttgatcggctccgccgagatca aaagtccttaaaaggaagaggcaacaccc	-	*
CY062719	Human H1N1pdm2009 IAVs	Human	H1N1	pdm	2009	Greece	A/Athens/INS124/2009	ggtgatgccccattccttgatcggctccgccgagatca aaagtccttaaaaggaagaggcaacaccc	-	*
CY088611	Human H1N1pdm2009 IAVs	Human	H1N1	pdm	2009	Greece	A/Athens/INS152/2009	ggtgatgccccattccttgatcggctccgccgagatca aaagtccttaaaaggaagaggcaacaccc	-	*
CY062879	Human H1N1pdm2009 IAVs	Human	H1N1	pdm	2009	Greece	A/Athens/INS161/2009	ggtgatgccccattccttgatcggctccgccgagatca aaagtccttaaaaggaagaggcaacaccc	-	*
CY062895	Human H1N1pdm2009 IAVs	Human	H1N1	pdm	2009	Greece	A/Athens/INS163/2009	ggtgatgccccattccttgatcggctccgccgagatca aaagtccttaaaaggaagaggcaacaccc	-	*
CY072346	Human H1N1pdm2009 IAVs	Human	H1N1	pdm	2009	Greece	A/Athens/INS320/2009	ggtgatgccccattccttgatcggctccgccgagatca aaagtccttaaaaggaagaggcaacaccc	-	*
CY072386	Human H1N1pdm2009 IAVs	Human	H1N1	pdm	2009	Greece	A/Athens/INS331/2009	ggtgatgccccattccttgatcggctccgccgagatca aaagtccttaaaaggaagaggcaacaccc	-	*
CY072418	Human H1N1pdm2009 IAVs	Human	H1N1	pdm	2009	Greece	A/Athens/INS335/2009	ggtgatgccccattccttgatcggctccgccgagatca aaagtccttaaaaggaagaggcaacaccc	-	*
CY072426	Human H1N1pdm2009 IAVs	Human	H1N1	pdm	2009	Greece	A/Athens/INS336/2009	ggtgatgccccattccttgatcggctccgccgagatca aaagtccttaaaaggaagaggcaacaccc	-	*
CY072474	Human H1N1pdm2009 IAVs	Human	H1N1	pdm	2009	Greece	A/Athens/INS342/2009	ggtgatgccccattccttgatcggctccgccgagatca aaagtccttaaaaggaagaggcaacaccc	-	*
CY072490	Human H1N1pdm2009 IAVs	Human	H1N1	pdm	2009	Greece	A/Athens/INS345/2009	ggtgatgccccattccttgatcggctccgccgagatca aaagtccttaaaaggaagaggcaacaccc	-	*
CY073509	Human H1N1pdm2009 IAVs	Human	H1N1	pdm	2009	Greece	A/Athens/INS350/2009	ggtgatgccccattccttgatcggctccgccgagatca aaagtccttaaaaggaagaggcaacaccc	-	*
CY069166	Human H1N1pdm2009 IAVs	Human	H1N1	pdm	2009	Greece	A/Athens/INS353/2009	ggtgatgccccattccttgatcggctccgccgagatca aaagtccttaaaaggaagaggcaacaccc	-	*
CY069198	Human H1N1pdm2009 IAVs	Human	H1N1	pdm	2009	Greece	A/Athens/INS358/2009	ggtgatgccccattccttgatcggctccgccgagatca aaagtccttaaaaggaagaggcaacaccc	-	*
CY055530	Human H1N1pdm2009 IAVs	Human	H1N1	pdm	2009	Australia	A/Australia/1/2009	ggtgatgccccattccttgatcggctccgccgagatca aaagtccttaaaaggaagaggcaacaccc	-	*
CY055554	Human H1N1pdm2009 IAVs	Human	H1N1	pdm	2009	Australia	A/Australia/10/2009	ggtgatgccccattccttgatcggctccgccgagatca aaagtccttaaaaggaagaggcaacaccc	-	*
CY055585	Human H1N1pdm2009 IAVs	Human	H1N1	pdm	2009	Australia	A/Australia/14/2009	ggtgatgccccattccttgatcggctccgccgagatca aaagtccttaaaaggaagaggcaacaccc	-	*
CY055609	Human H1N1pdm2009 IAVs	Human	H1N1	pdm	2009	Australia	A/Australia/20/2009	ggtgatgccccattccttgatcggctccgccgagatca aaagtccttaaaaggaagaggcaacaccc	-	*
CY055617	Human H1N1pdm2009 IAVs	Human	H1N1	pdm	2009	Australia	A/Australia/21/2009	ggtgatgccccattccttgatcggctccgccgagatca aaagtccttaaaaggaagaggcaacaccc	-	*
CY055665	Human H1N1pdm2009 IAVs	Human	H1N1	pdm	2009	Australia	A/Australia/28/2009	ggtgatgccccattccttgatcggctccgccgagatca aaagtccttaaaaggaagaggcaacaccc	-	*
CY055672	Human H1N1pdm2009 IAVs	Human	H1N1		2009	Australia	A/Australia/29/2009	ggtgatgccccattccttgatcggctccgccgagatca aaagtccttaaaaggaagaggcaacaccc	-	*
CY055680	Human H1N1pdm2009 IAVs	Human	H1N1	pdm	2009	Australia	A/Australia/31/2009	ggtgatgccccattccttgatcggctccgccgagatca aaagtccttaaaaggaagaggcaacaccc	-	*

CY055696	Human H1N1pdm2009 IAVs	Human	H1N1	pdm	2009	Australia	A/Australia/35/2009	ggtgatgccccattccttgatcggctccgccgagatca aaagtccttaaaaggaagaggcaacaccc	-	*
CY055704	Human H1N1pdm2009 IAVs	Human	H1N1	pdm	2009	Australia	A/Australia/36/2009	ggtgatgccccattccttgatcggctccgccgagatca aaagtccttaaaaggaagaggcaacaccc	-	*
CY055712	Human H1N1pdm2009 IAVs	Human	H1N1		2009	Australia	A/Australia/37/2009	ggtgatgccccattccttgatcggctccgccgagatca aaagtccttaaaaggaagaggcaacaccc	-	*
CY055720	Human H1N1pdm2009 IAVs	Human	H1N1	pdm	2009	Australia	A/Australia/39/2009	ggtgatgccccattccttgatcggctccgccgagatca aaagtccttaaaaggaagaggcaacaccc	-	*
CY055071	Human H1N1pdm2009 IAVs	Human	H1N1	pdm	2009	Australia	A/Australia/4/2009	ggtgatgccccattccttgatcggctccgccgagatca aaagtccttaaaaggaagaggcaacaccc	-	*
CY055728	Human H1N1pdm2009 IAVs	Human	H1N1	pdm	2009	Australia	A/Australia/40/2009	ggtgatgccccattccttgatcggctccgccgagatca aaagtccttaaaaggaagaggcaacaccc	-	*
CY055736	Human H1N1pdm2009 IAVs	Human	H1N1	pdm	2009	Australia	A/Australia/42/2009	ggtgatgccccattccttgatcggctccgccgagatca aaagtccttaaaaggaagaggcaacaccc	-	*
CY055744	Human H1N1pdm2009 IAVs	Human	H1N1	pdm	2009	Australia	A/Australia/43/2009	ggtgatgccccattccttgatcggctccgccgagatca aaagtccttaaaaggaagaggcaacaccc	-	*
CY055760	Human H1N1pdm2009 IAVs	Human	H1N1	pdm	2009	Australia	A/Australia/45/2009	ggtgatgccccattccttgatcggctccgccgagatca aaagtccttaaaaggaagaggcaacaccc	-	*
CY055784	Human H1N1pdm2009 IAVs	Human	H1N1	pdm	2009	Australia	A/Australia/51/2009	ggtgatgccccattccttgatcggctccgccgagatca aaagtccttaaaaggaagaggcaacaccc	-	*
CY055792	Human H1N1pdm2009 IAVs	Human	H1N1		2009	Australia	A/Australia/53/2009	ggtgatgccccattccttgatcggctccgccgagatca aaagtccttaaaaggaagaggcaacaccc	-	*
CY055808	Human H1N1pdm2009 IAVs	Human	H1N1	pdm	2009	Australia	A/Australia/56/2009	ggtgatgccccattccttgatcggctccgccgagatca aaagtccttaaaaggaagaggcaacaccc	-	*
CY055832	Human H1N1pdm2009 IAVs	Human	H1N1		2009	Australia	A/Australia/59/2009	ggtgatgccccattccttgatcggctccgccgagatca aaagtccttaaaaggaagaggcaacaccc	-	*
CY092189	Human H1N1pdm2009 IAVs	Human	H1N1	pdm	2009	Australia	A/Australia/17/2009	ggtgatgccccattccttgatcggctccgccgagatca aaagtccttaaaaggaagaggcaacaccc	-	*
CY055079	Human H1N1pdm2009 IAVs	Human	H1N1	pdm	2009	Australia	A/Australia/6/2009	ggtgatgccccattccttgatcggctccgccgagatca aaagtccttaaaaggaagaggcaacaccc	-	*
CY055864	Human H1N1pdm2009 IAVs	Human	H1N1	pdm	2009	Australia	A/Australia/64/2009	ggtgatgccccattccttgatcggctccgccgagatca aaagtccttaaaaggaagaggcaacaccc	-	*
CY055872	Human H1N1pdm2009 IAVs	Human	H1N1		2009	Australia	A/Australia/65/2009	ggtgatgccccattccttgatcggctccgccgagatca aaagtccttaaaaggaagaggcaacaccc	-	*
CY055880	Human H1N1pdm2009 IAVs	Human	H1N1	pdm	2009	Australia	A/Australia/66/2009	ggtgatgccccattccttgatcggctccgccgagatca aaagtccttaaaaggaagaggcaacaccc	-	*
CY055538	Human H1N1pdm2009 IAVs	Human	H1N1	pdm	2009	Australia	A/Australia/7/2009	ggtgatgccccattccttgatcggctccgccgagatca aaagtccttaaaaggaagaggcaacaccc	-	*
CY055904	Human H1N1pdm2009 IAVs	Human	H1N1	pdm	2009	Australia	A/Australia/72/2009	ggtgatgccccattccttgatcggctccgccgagatca aaagtccttaaaaggaagaggcaacaccc	-	*
CY055928	Human H1N1pdm2009 IAVs	Human	H1N1	pdm	2009	Australia	A/Australia/80/2009	ggtgatgccccattccttgatcggctccgccgagatca aaagtccttaaaaggaagaggcaacaccc	-	*
CY055546	Human H1N1pdm2009 IAVs	Human	H1N1	pdm	2009	Australia	A/Australia/9/2009	ggtgatgccccattccttgatcggctccgccgagatca aaagtccttaaaaggaagaggcaacaccc	-	*
GQ396554	Human H1N1pdm2009 IAVs	Human	H1N1	pdm	2009	Spain	A/Baleares/GP500/2009	ggtgatgccccattccttgatcggctccgccgagatca aaagtccttaaaaggaagaggcaacaccc	-	*
CY045507	Human H1N1pdm2009 IAVs	Human	H1N1	pdm	2009	Germany	A/Bayern/66/2009	ggtgatgccccattccttgatcggctccgccgagatca aaagtccttaaaaggaagaggcaacaccc	-	*
CY064776	Human H1N1pdm2009 IAVs	Human	H1N1	pdm	2009	Germany	A/Berlin/INS170/2009	ggtgatgccccattccttgatcggctccgccgagatca aaagtccttaaaaggaagaggcaacaccc	-	*

CY083801	Human H1N1pdm2009 IAVs	Human	H1N1	pdm	2009	Germany	A/Berlin/INS362/2009	ggtgatgccccattccttgatcggctccgccgagatca aaagtccttaaaaggaagaggcaacaccc	-	*
HQ263277	Human H1N1pdm2009 IAVs	Human	H1N1	pdm	2009	USA	A/Bethesda/NIH106_D14/2009	ggtgatgccccattccttgatcggctccgccgagatca aaagtccttaaaaggaagaggcaacaccc	-	*
HQ263284	Human H1N1pdm2009 IAVs	Human	H1N1	pdm	2009	USA	A/Bethesda/NIH107_D0/2009	ggtgatgccccattccttgatcggctccgccgagatca aaagtccttaaaaggaagaggcaacaccc	-	*
CY067019	Human H1N1pdm2009 IAVs	Human	H1N1	pdm	2009	Germany	A/Bochum/INS249/2009	ggtgatgccccattccttgatcggctccgccgagatca aaagtccttaaaaggaagaggcaacaccc	-	*
CY067027	Human H1N1pdm2009 IAVs	Human	H1N1	pdm	2009	Germany	A/Bochum/INS250/2009	ggtgatgccccattccttgatcggctccgccgagatca aaagtccttaaaaggaagaggcaacaccc	-	*
CY063602	Human H1N1pdm2009 IAVs	Human	H1N1		2009	Germany	A/Bonn/INS128/2009	ggtgatgccccattccttgatcggctccgccgagatca aaagtccttaaaaggaagaggcaacaccc	-	*
CY066635	Human H1N1pdm2009 IAVs	Human	H1N1	pdm	2009	Germany	A/Bonn/INS277/2009	ggtgatgccccattccttgatcggctccgccgagatca aaagtccttaaaaggaagaggcaacaccc	-	*
CY066643	Human H1N1pdm2009 IAVs	Human	H1N1	pdm	2009	Germany	A/Bonn/INS278/2009	ggtgatgccccattccttgatcggctccgccgagatca aaagtccttaaaaggaagaggcaacaccc	-	*
CY067171	Human H1N1pdm2009 IAVs	Human	H1N1	pdm	2009	Germany	A/Bonn/INS280/2009	ggtgatgccccattccttgatcggctccgccgagatca aaagtccttaaaaggaagaggcaacaccc	-	*
CY089721	Human H1N1pdm2009 IAVs	Human	H1N1	pdm	2009	USA	A/Boston/107/2009	ggtgatgccccattccttgatcggctccgccgagatca aaagtccttaaaaggaagaggcaacaccc	-	*
CY063498	Human H1N1pdm2009 IAVs	Human	H1N1	pdm	2009	USA	A/Boston/112/2009	ggtgatgccccattccttgatcggctccgccgagatca aaagtccttaaaaggaagaggcaacaccc	-	*
CY064560	Human H1N1pdm2009 IAVs	Human	H1N1	pdm	2009	USA	A/Boston/123/2009	ggtgatgccccattccttgatcggctccgccgagatca aaagtccttaaaaggaagaggcaacaccc	-	*
CY064592	Human H1N1pdm2009 IAVs	Human	H1N1		2009	USA	A/Boston/127/2009	ggtgatgccccattccttgatcggctccgccgagatca aaagtccttaaaaggaagaggcaacaccc	-	*
CY064640	Human H1N1pdm2009 IAVs	Human	H1N1	pdm	2009	USA	A/Boston/134/2009	ggtgatgccccattccttgatcggctccgccgagatca aaagtccttaaaaggaagaggcaacaccc	-	*
CY064680	Human H1N1pdm2009 IAVs	Human	H1N1	pdm	2009	USA	A/Boston/141/2009	ggtgatgccccattccttgatcggctccgccgagatca aaagtccttaaaaggaagaggcaacaccc	-	*
CY066187	Human H1N1pdm2009 IAVs	Human	H1N1	pdm	2009	USA	A/Boston/144/2009	ggtgatgccccattccttgatcggctccgccgagatca aaagtccttaaaaggaagaggcaacaccc	-	*
CY063514	Human H1N1pdm2009 IAVs	Human	H1N1	pdm	2009	USA	A/Boston/145/2009	ggtgatgccccattccttgatcggctccgccgagatca aaagtccttaaaaggaagaggcaacaccc	-	*
CY063538	Human H1N1pdm2009 IAVs	Human	H1N1	pdm	2009	USA	A/Boston/148/2009	ggtgatgccccattccttgatcggctccgccgagatca aaagtccttaaaaggaagaggcaacaccc	-	*
CY063546	Human H1N1pdm2009 IAVs	Human	H1N1	pdm	2009	USA	A/Boston/149/2009	ggtgatgccccattccttgatcggctccgccgagatca aaagtccttaaaaggaagaggcaacaccc	-	*
CY063562	Human H1N1pdm2009 IAVs	Human	H1N1		2009	USA	A/Boston/152/2009	ggtgatgccccattccttgatcggctccgccgagatca aaagtccttaaaaggaagaggcaacaccc	-	*
CY075536	Human H1N1pdm2009 IAVs	Human	H1N1	pdm	2009	USA	A/Boston/612/2009	ggtgatgccccattccttgatcggctccgccgagatca aaagtccttaaaaggaagaggcaacaccc	-	*
CY075664	Human H1N1pdm2009 IAVs	Human	H1N1	pdm	2009	USA	A/Boston/628/2009	ggtgatgccccattccttgatcggctccgccgagatca aaagtccttaaaaggaagaggcaacaccc	-	*
CY089223	Human H1N1pdm2009 IAVs	Human	H1N1	pdm	2009	USA	A/Boston/630/2009	ggtgatgccccattccttgatcggctccgccgagatca aaagtccttaaaaggaagaggcaacaccc	-	*
CY089255	Human H1N1pdm2009 IAVs	Human	H1N1	pdm	2009	USA	A/Boston/637/2009	ggtgatgccccattccttgatcggctccgccgagatca aaagtccttaaaaggaagaggcaacaccc	-	*
CY075584	Human H1N1pdm2009 IAVs	Human	H1N1	pdm	2009	USA	A/Boston/663/2009	ggtgatgccccattccttgatcggctccgccgagatca aaagtccttaaaaggaagaggcaacaccc	-	*

CY075592	Human H1N1pdm2009 IAVs	Human	H1N1		2009	USA	A/Boston/666/2009	ggtgatgccccattccttgatcggctccgccgagatca aaagtccttaaaaggaagaggcaacaccc	-	*
CY089295	Human H1N1pdm2009 IAVs	Human	H1N1	pdm	2009	USA	A/Boston/670/2009	ggtgatgccccattccttgatcggctccgccgagatca aaagtccttaaaaggaagaggcaacaccc	-	*
CY089327	Human H1N1pdm2009 IAVs	Human	H1N1	pdm	2009	USA	A/Boston/680/2009	ggtgatgccccattccttgatcggctccgccgagatca aaagtccttaaaaggaagaggcaacaccc	-	*
CY075608	Human H1N1pdm2009 IAVs	Human	H1N1	pdm	2009	USA	A/Boston/681/2009	ggtgatgccccattccttgatcggctccgccgagatca aaagtccttaaaaggaagaggcaacaccc	-	*
CY089367	Human H1N1pdm2009 IAVs	Human	H1N1	pdm	2009	USA	A/Boston/691/2009	ggtgatgccccattccttgatcggctccgccgagatca aaagtccttaaaaggaagaggcaacaccc	-	*
CY088589	Human H1N1pdm2009 IAVs	Human	H1N1	pdm	2009	USA	A/Boston/694/2009	ggtgatgccccattccttgatcggctccgccgagatca aaagtccttaaaaggaagaggcaacaccc	-	*
CY075648	Human H1N1pdm2009 IAVs	Human	H1N1	pdm	2009	USA	A/Boston/704/2009	ggtgatgccccattccttgatcggctccgccgagatca aaagtccttaaaaggaagaggcaacaccc	-	*
CY083757	Human H1N1pdm2009 IAVs	Human	H1N1	pdm	2009	Belgium	A/Brussels/INS205/2009	ggtgatgccccattccttgatcggctccgccgagatca aaagtccttaaaaggaagaggcaacaccc	-	*
CY066987	Human H1N1pdm2009 IAVs	Human	H1N1	pdm	2009	Belgium	A/Brussels/INS243/2009	ggtgatgccccattccttgatcggctccgccgagatca aaagtccttaaaaggaagaggcaacaccc	-	*
GQ117038	Human H1N1pdm2009 IAVs	Human	H1N1	pdm	2009	USA	A/California/14/2009	ggtgatgccccattccttgatcggctccgccgagatca aaagtccttaaaaggaagaggcaacaccc	-	*
CY063159	Human H1N1pdm2009 IAVs	Human	H1N1	pdm	2009	USA	A/California/VRDL100/2009	ggtgatgccccattccttgatcggctccgccgagatca aaagtccttaaaaggaagaggcaacaccc	-	*
CY066203	Human H1N1pdm2009 IAVs	Human	H1N1	pdm	2009	USA	A/California/VRDL103/2009	ggtgatgccccattccttgatcggctccgccgagatca aaagtccttaaaaggaagaggcaacaccc	-	*
CY066267	Human H1N1pdm2009 IAVs	Human	H1N1	pdm	2009	USA	A/California/VRDL111/2009	ggtgatgccccattccttgatcggctccgccgagatca aaagtccttaaaaggaagaggcaacaccc	-	*
CY066275	Human H1N1pdm2009 IAVs	Human	H1N1	pdm	2009	USA	A/California/VRDL112/2009	ggtgatgccccattccttgatcggctccgccgagatca aaagtccttaaaaggaagaggcaacaccc	-	*
CY066291	Human H1N1pdm2009 IAVs	Human	H1N1	pdm	2009	USA	A/California/VRDL114/2009	ggtgatgccccattccttgatcggctccgccgagatca aaagtccttaaaaggaagaggcaacaccc	-	*
CY066323	Human H1N1pdm2009 IAVs	Human	H1N1	pdm	2009	USA	A/California/VRDL118/2009	ggtgatgccccattccttgatcggctccgccgagatca aaagtccttaaaaggaagaggcaacaccc	-	*
CY066331	Human H1N1pdm2009 IAVs	Human	H1N1	pdm	2009	USA	A/California/VRDL119/2009	ggtgatgccccattccttgatcggctccgccgagatca aaagtccttaaaaggaagaggcaacaccc	-	*
CY066355	Human H1N1pdm2009 IAVs	Human	H1N1	pdm	2009	USA	A/California/VRDL122/2009	ggtgatgccccattccttgatcggctccgccgagatca aaagtccttaaaaggaagaggcaacaccc	-	*
CY066363	Human H1N1pdm2009 IAVs	Human	H1N1	pdm	2009	USA	A/California/VRDL123/2009	ggtgatgccccattccttgatcggctccgccgagatca aaagtccttaaaaggaagaggcaacaccc	-	*
CY066427	Human H1N1pdm2009 IAVs	Human	H1N1	pdm	2009	USA	A/California/VRDL131/2009	ggtgatgccccattccttgatcggctccgccgagatca aaagtccttaaaaggaagaggcaacaccc	-	*
CY055467	Human H1N1pdm2009 IAVs	Human	H1N1	pdm	2009	USA	A/California/VRDL14/2009	ggtgatgccccattccttgatcggctccgccgagatca aaagtccttaaaaggaagaggcaacaccc	-	*
CY054759	Human H1N1pdm2009 IAVs	Human	H1N1	pdm	2009	USA	A/California/VRDL19/2009	ggtgatgccccattccttgatcggctccgccgagatca aaagtccttaaaaggaagaggcaacaccc	-	*
CY054783	Human H1N1pdm2009 IAVs	Human	H1N1	pdm	2009	USA	A/California/VRDL22/2009	ggtgatgccccattccttgatcggctccgccgagatca aaagtccttaaaaggaagaggcaacaccc	-	*
CY054791	Human H1N1pdm2009 IAVs	Human	H1N1	pdm	2009	USA	A/California/VRDL23/2009	ggtgatgccccattccttgatcggctccgccgagatca aaagtccttaaaaggaagaggcaacaccc	-	*
CY054799	Human H1N1pdm2009 IAVs	Human	H1N1	pdm	2009	USA	A/California/VRDL25/2009	ggtgatgccccattccttgatcggctccgccgagatca aaagtccttaaaaggaagaggcaacaccc	-	*

CY054815	Human H1N1pdm2009 IAVs	Human	H1N1	pdm	2009	USA	A/California/VRDL27/2009	ggtgatgccccattccttgatcggctccgccgagatca aaagtccttaaaaggaagaggcaacaccc	-	*
CY054863	Human H1N1pdm2009 IAVs	Human	H1N1	pdm	2009	USA	A/California/VRDL33/2009	ggtgatgccccattccttgatcggctccgccgagatca aaagtccttaaaaggaagaggcaacaccc	-	*
CY054871	Human H1N1pdm2009 IAVs	Human	H1N1	pdm	2009	USA	A/California/VRDL34/2009	ggtgatgccccattccttgatcggctccgccgagatca aaagtccttaaaaggaagaggcaacaccc	-	*
CY092776	Human H1N1pdm2009 IAVs	Human	mixed	pdm	2009	USA	A/California/VRDL353/2009	ggtgatgccccattccttgatcggctccgccgagatca aaagtccttaaaaggaagaggcaacaccc	-	*
CY054711	Human H1N1pdm2009 IAVs	Human	H1N1	pdm	2009	USA	A/California/VRDL4/2009	ggtgatgccccattccttgatcggctccgccgagatca aaagtccttaaaaggaagaggcaacaccc	-	*
CY054919	Human H1N1pdm2009 IAVs	Human	H1N1	pdm	2009	USA	A/California/VRDL41/2009	ggtgatgccccattccttgatcggctccgccgagatca aaagtccttaaaaggaagaggcaacaccc	-	*
CY054951	Human H1N1pdm2009 IAVs	Human	H1N1	pdm	2009	USA	A/California/VRDL50/2009	ggtgatgccccattccttgatcggctccgccgagatca aaagtccttaaaaggaagaggcaacaccc	-	*
CY054959	Human H1N1pdm2009 IAVs	Human	H1N1	pdm	2009	USA	A/California/VRDL51/2009	ggtgatgccccattccttgatcggctccgccgagatca aaagtccttaaaaggaagaggcaacaccc	-	*
CY054967	Human H1N1pdm2009 IAVs	Human	H1N1	pdm	2009	USA	A/California/VRDL52/2009	ggtgatgccccattccttgatcggctccgccgagatca aaagtccttaaaaggaagaggcaacaccc	-	*
CY054975	Human H1N1pdm2009 IAVs	Human	H1N1	pdm	2009	USA	A/California/VRDL53/2009	ggtgatgccccattccttgatcggctccgccgagatca aaagtccttaaaaggaagaggcaacaccc	-	*
CY054983	Human H1N1pdm2009 IAVs	Human	H1N1	pdm	2009	USA	A/California/VRDL55/2009	ggtgatgccccattccttgatcggctccgccgagatca aaagtccttaaaaggaagaggcaacaccc	-	*
CY055007	Human H1N1pdm2009 IAVs	Human	H1N1	pdm	2009	USA	A/California/VRDL64/2009	ggtgatgccccattccttgatcggctccgccgagatca aaagtccttaaaaggaagaggcaacaccc	-	*
CY055015	Human H1N1pdm2009 IAVs	Human	H1N1	pdm	2009	USA	A/California/VRDL65/2009	ggtgatgccccattccttgatcggctccgccgagatca aaagtccttaaaaggaagaggcaacaccc	-	*
CY055031	Human H1N1pdm2009 IAVs	Human	H1N1	pdm	2009	USA	A/California/VRDL68/2009	ggtgatgccccattccttgatcggctccgccgagatca aaagtccttaaaaggaagaggcaacaccc	-	*
CY054735	Human H1N1pdm2009 IAVs	Human	H1N1	pdm	2009	USA	A/California/VRDL7/2009	ggtgatgccccattccttgatcggctccgccgagatca aaagtccttaaaaggaagaggcaacaccc	-	*
CY063119	Human H1N1pdm2009 IAVs	Human	H1N1	pdm	2009	USA	A/California/VRDL93/2009	ggtgatgccccattccttgatcggctccgccgagatca aaagtccttaaaaggaagaggcaacaccc	-	*
CY063127	Human H1N1pdm2009 IAVs	Human	H1N1	pdm	2009	USA	A/California/VRDL96/2009	ggtgatgccccattccttgatcggctccgccgagatca aaagtccttaaaaggaagaggcaacaccc	-	*
CY063135	Human H1N1pdm2009 IAVs	Human	H1N1	pdm	2009	USA	A/California/VRDL97/2009	ggtgatgccccattccttgatcggctccgccgagatca aaagtccttaaaaggaagaggcaacaccc	-	*
CY063151	Human H1N1pdm2009 IAVs	Human	H1N1	pdm	2009	USA	A/California/VRDL99/2009	ggtgatgccccattccttgatcggctccgccgagatca aaagtccttaaaaggaagaggcaacaccc	-	*
GQ465721	Human H1N1pdm2009 IAVs	Human	H1N1	pdm	2009	Canada	A/Canada_AB/RV1531/2009	ggtgatgccccattccttgatcggctccgccgagatca aaagtccttaaaaggaagaggcaacaccc	-	*
GQ132169	Human H1N1pdm2009 IAVs	Human	H1N1	pdm	2009	Canada	A/Canada_AB/RV1532/2009	ggtgatgccccattccttgatcggctccgccgagatca aaagtccttaaaaggaagaggcaacaccc	-	*
GQ132171	Human H1N1pdm2009 IAVs	Human	H1N1	pdm	2009	Canada	A/Canada_ON/RV1526/2009	ggtgatgccccattccttgatcggctccgccgagatca aaagtccttaaaaggaagaggcaacaccc	-	*
GQ132168	Human H1N1pdm2009 IAVs	Human	H1N1	pdm	2009	Canada	A/Canada_ON/RV1529/2009	ggtgatgccccattccttgatcggctccgccgagatca aaagtccttaaaaggaagaggcaacaccc	-	*
GQ465726	Human H1N1pdm2009 IAVs	Human	H1N1	pdm	2009	Canada	A/Canada_ON/RV1589/2009	ggtgatgccccattccttgatcggctccgccgagatca aaagtccttaaaaggaagaggcaacaccc	-	*
GQ402275	Human H1N1pdm2009 IAVs	Human	H1N1	pdm	2009	Canada	A/Canada_QC/RV1759/2009	ggtgatgccccattccttgatcggctccgccgagatca aaagtccttaaaaggaagaggcaacaccc	-	*

CY075159	Human H1N1pdm2009 IAVs	Human	H1N1	pdm	2009	Chile	A/Chile/158/2009	ggtgatgccccattccttgatcggctccgccgagatca aaagtccttaaaaggaagaggcaacaccc	-	*
CY075175	Human H1N1pdm2009 IAVs	Human	H1N1	pdm	2009	Chile	A/Chile/1586/2009	ggtgatgccccattccttgatcggctccgccgagatca aaagtccttaaaaggaagaggcaacaccc	-	*
CY075199	Human H1N1pdm2009 IAVs	Human	H1N1	pdm	2009	Chile	A/Chile/1600/2009	ggtgatgccccattccttgatcggctccgccgagatca aaagtccttaaaaggaagaggcaacaccc	-	*
CY075207	Human H1N1pdm2009 IAVs	Human	H1N1	pdm	2009	Chile	A/Chile/1603/2009	ggtgatgccccattccttgatcggctccgccgagatca aaagtccttaaaaggaagaggcaacaccc	-	*
CY075215	Human H1N1pdm2009 IAVs	Human	H1N1	pdm	2009	Chile	A/Chile/1624/2009	ggtgatgccccattccttgatcggctccgccgagatca aaagtccttaaaaggaagaggcaacaccc	-	*
CY075167	Human H1N1pdm2009 IAVs	Human	H1N1	pdm	2009	Chile	A/Chile/180/2009	ggtgatgccccattccttgatcggctccgccgagatca aaagtccttaaaaggaagaggcaacaccc	-	*
CY089183	Human H1N1pdm2009 IAVs	Human	H1N1	pdm	2009	Chile	A/Chile/19/2009	ggtgatgccccattccttgatcggctccgccgagatca aaagtccttaaaaggaagaggcaacaccc	-	*
CY075223	Human H1N1pdm2009 IAVs	Human	H1N1	pdm	2009	Chile	A/Chile/2009/2009	ggtgatgccccattccttgatcggctccgccgagatca aaagtccttaaaaggaagaggcaacaccc	-	*
CY075231	Human H1N1pdm2009 IAVs	Human	H1N1	pdm	2009	Chile	A/Chile/2239/2009	ggtgatgccccattccttgatcggctccgccgagatca aaagtccttaaaaggaagaggcaacaccc	-	*
CY075247	Human H1N1pdm2009 IAVs	Human	H1N1	pdm	2009	Chile	A/Chile/2362/2009	ggtgatgccccattccttgatcggctccgccgagatca aaagtccttaaaaggaagaggcaacaccc	-	*
FJ998221	Human H1N1pdm2009 IAVs	Human	H1N1	pdm	2009	Canada	A/Canada_ON/RV1527/2009	ggtgatgccccattccttgatcggctccgccgagatca aaagtccttaaaaggaagaggcaacaccc	-	*
CY075087	Human H1N1pdm2009 IAVs	Human	H1N1	pdm	2009	Chile	A/Chile/28/2009	ggtgatgccccattccttgatcggctccgccgagatca aaagtccttaaaaggaagaggcaacaccc	-	*
CY075239	Human H1N1pdm2009 IAVs	Human	H1N1	pdm	2009	Chile	A/Chile/2851/2009	ggtgatgccccattccttgatcggctccgccgagatca aaagtccttaaaaggaagaggcaacaccc	-	*
CY075255	Human H1N1pdm2009 IAVs	Human	H1N1	pdm	2009	Chile	A/Chile/2909/2009	ggtgatgccccattccttgatcggctccgccgagatca aaagtccttaaaaggaagaggcaacaccc	-	*
CY075263	Human H1N1pdm2009 IAVs	Human	H1N1	pdm	2009	Chile	A/Chile/2911/2009	ggtgatgccccattccttgatcggctccgccgagatca aaagtccttaaaaggaagaggcaacaccc	-	*
CY075271	Human H1N1pdm2009 IAVs	Human	H1N1	pdm	2009	Chile	A/Chile/2994/2009	ggtgatgccccattccttgatcggctccgccgagatca aaagtccttaaaaggaagaggcaacaccc	-	*
CY075287	Human H1N1pdm2009 IAVs	Human	H1N1	pdm	2009	Chile	A/Chile/3056/2009	ggtgatgccccattccttgatcggctccgccgagatca aaagtccttaaaaggaagaggcaacaccc	-	*
CY075095	Human H1N1pdm2009 IAVs	Human	H1N1	pdm	2009	Chile	A/Chile/31/2009	ggtgatgccccattccttgatcggctccgccgagatca aaagtccttaaaaggaagaggcaacaccc	-	*
CY075295	Human H1N1pdm2009 IAVs	Human	H1N1	pdm	2009	Chile	A/Chile/3123/2009	ggtgatgccccattccttgatcggctccgccgagatca aaagtccttaaaaggaagaggcaacaccc	-	*
CY075103	Human H1N1pdm2009 IAVs	Human	H1N1	pdm	2009	Chile	A/Chile/32/2009	ggtgatgccccattccttgatcggctccgccgagatca aaagtccttaaaaggaagaggcaacaccc	-	*
CY075303	Human H1N1pdm2009 IAVs	Human	H1N1	pdm	2009	Chile	A/Chile/3220/2009	ggtgatgccccattccttgatcggctccgccgagatca aaagtccttaaaaggaagaggcaacaccc	-	*
CY075319	Human H1N1pdm2009 IAVs	Human	H1N1	pdm	2009	Chile	A/Chile/3244/2009	ggtgatgccccattccttgatcggctccgccgagatca aaagtccttaaaaggaagaggcaacaccc	-	*
CY075327	Human H1N1pdm2009 IAVs	Human	H1N1	pdm	2009	Chile	A/Chile/3295/2009	ggtgatgccccattccttgatcggctccgccgagatca aaagtccttaaaaggaagaggcaacaccc	-	*
CY075343	Human H1N1pdm2009 IAVs	Human	H1N1	pdm	2009	Chile	A/Chile/3361/2009	ggtgatgccccattccttgatcggctccgccgagatca aaagtccttaaaaggaagaggcaacaccc	-	*
CY075367	Human H1N1pdm2009 IAVs	Human	H1N1	pdm	2009	Chile	A/Chile/3467/2009	ggtgatgccccattccttgatcggctccgccgagatca aaagtccttaaaaggaagaggcaacaccc	-	*

CY075375	Human H1N1pdm2009 IAVs	Human	H1N1	pdm	2009	Chile	A/Chile/3553/2009	ggtgatgccccattccttgatcggctccgccgagatca<br>aaagtccttaaaaggaagaggcaacaccc	-	*
CY075383	Human H1N1pdm2009 IAVs	Human	H1N1	pdm	2009	Chile	A/Chile/3586/2009	ggtgatgccccattccttgatcggctccgccgagatca<br>aaagtccttaaaaggaagaggcaacaccc	-	*
CY075111	Human H1N1pdm2009 IAVs	Human	H1N1	pdm	2009	Chile	A/Chile/36/2009	ggtgatgccccattccttgatcggctccgccgagatca<br>aaagtccttaaaaggaagaggcaacaccc	-	*
CY075391	Human H1N1pdm2009 IAVs	Human	H1N1	pdm	2009	Chile	A/Chile/3760/2009	ggtgatgccccattccttgatcggctccgccgagatca<br>aaagtccttaaaaggaagaggcaacaccc	-	*
CY075407	Human H1N1pdm2009 IAVs	Human	H1N1	pdm	2009	Chile	A/Chile/3819/2009	ggtgatgccccattccttgatcggctccgccgagatca<br>aaagtccttaaaaggaagaggcaacaccc	-	*
CY075415	Human H1N1pdm2009 IAVs	Human	H1N1	pdm	2009	Chile	A/Chile/3905/2009	ggtgatgccccattccttgatcggctccgccgagatca<br>aaagtccttaaaaggaagaggcaacaccc	-	*
CY075423	Human H1N1pdm2009 IAVs	Human	H1N1	pdm	2009	Chile	A/Chile/3935/2009	ggtgatgccccattccttgatcggctccgccgagatca<br>aaagtccttaaaaggaagaggcaacaccc	-	*
CY075119	Human H1N1pdm2009 IAVs	Human	H1N1	pdm	2009	Chile	A/Chile/40/2009	ggtgatgccccattccttgatcggctccgccgagatca<br>aaagtccttaaaaggaagaggcaacaccc	-	*
CY081530	Human H1N1pdm2009 IAVs	Human	H1N1	pdm	2009	Cambodia	A/Cambodia/NHRCC00006/2009	ggtgatgccccattccttgatcggctccgccgagatca<br>aaagtccttaaaaggaagaggcaacaccc	-	*
CY075431	Human H1N1pdm2009 IAVs	Human	H1N1	pdm	2009	Chile	A/Chile/4064/2009	ggtgatgccccattccttgatcggctccgccgagatca<br>aaagtccttaaaaggaagaggcaacaccc	-	*
CY075127	Human H1N1pdm2009 IAVs	Human	H1N1	pdm	2009	Chile	A/Chile/42/2009	ggtgatgccccattccttgatcggctccgccgagatca<br>aaagtccttaaaaggaagaggcaacaccc	-	*
CY075455	Human H1N1pdm2009 IAVs	Human	H1N1	pdm	2009	Chile	A/Chile/4257/2009	ggtgatgccccattccttgatcggctccgccgagatca<br>aaagtccttaaaaggaagaggcaacaccc	-	*
CY075471	Human H1N1pdm2009 IAVs	Human	H1N1	pdm	2009	Chile	A/Chile/4438/2009	ggtgatgccccattccttgatcggctccgccgagatca<br>aaagtccttaaaaggaagaggcaacaccc	-	*
CY075135	Human H1N1pdm2009 IAVs	Human	H1N1	pdm	2009	Chile	A/Chile/56/2009	ggtgatgccccattccttgatcggctccgccgagatca<br>aaagtccttaaaaggaagaggcaacaccc	-	*
CY075143	Human H1N1pdm2009 IAVs	Human	H1N1	pdm	2009	Chile	A/Chile/88/2009	ggtgatgccccattccttgatcggctccgccgagatca<br>aaagtccttaaaaggaagaggcaacaccc	-	*
CY075151	Human H1N1pdm2009 IAVs	Human	H1N1	pdm	2009	Chile	A/Chile/95/2009	ggtgatgccccattccttgatcggctccgccgagatca<br>aaagtccttaaaaggaagaggcaacaccc	-	*
GU234177	Human H1N1pdm2009 IAVs	Human	H1N1	pdm	2009	Russia	A/Chita/01/2009	ggtgatgccccattccttgatcggctccgccgagatca<br>aaagtccttaaaaggaagaggcaacaccc	-	*
HM189316	Human H1N1pdm2009 IAVs	Human	H1N1	pdm	2009	Russia	A/Chita/CRIE_8/2009	ggtgatgccccattccttgatcggctccgccgagatca<br>aaagtccttaaaaggaagaggcaacaccc	-	*
CY083060	Human H1N1pdm2009 IAVs	Human	H1N1	pdm	2009	USA	A/Colorado/WRAIR0353S/2009	ggtgatgccccattccttgatcggctccgccgagatca<br>aaagtccttaaaaggaagaggcaacaccc	-	*
CY056064	Human H1N1pdm2009 IAVs	Human	H1N1	pdm	2009	USA	A/District_of_Columbia/INS20/2009	ggtgatgccccattccttgatcggctccgccgagatca<br>aaagtccttaaaaggaagaggcaacaccc	-	*
CY066859	Human H1N1pdm2009 IAVs	Human	H1N1	pdm	2009	USA	A/District_of_Columbia/INS226/2009	ggtgatgccccattccttgatcggctccgccgagatca<br>aaagtccttaaaaggaagaggcaacaccc	-	*
CY056080	Human H1N1pdm2009 IAVs	Human	H1N1	pdm	2009	USA	A/District_of_Columbia/INS24/2009	ggtgatgccccattccttgatcggctccgccgagatca<br>aaagtccttaaaaggaagaggcaacaccc	-	*
CY056096	Human H1N1pdm2009 IAVs	Human	H1N1	pdm	2009	USA	A/District_of_Columbia/INS27/2009	ggtgatgccccattccttgatcggctccgccgagatca<br>aaagtccttaaaaggaagaggcaacaccc	-	*
CY056128	Human H1N1pdm2009 IAVs	Human	H1N1	pdm	2009	USA	A/District_of_Columbia/INS33/2009	ggtgatgccccattccttgatcggctccgccgagatca<br>aaagtccttaaaaggaagaggcaacaccc	-	*
GQ485660	Human H1N1pdm2009 IAVs	Human	H1N1	pdm	2009	Russia	A/Ekaterinburg/01/2009	ggtgatgccccattccttgatcggctccgccgagatca<br>aaagtccttaaaaggaagaggcaacaccc	-	*

JX625556	Human H1N1pdm2009 IAVs	Human	H1N1	pdm	2009	United_Kingdom	A/England/1397/2009	ggtgatgccccattccttgatcggctccgccgagatcaaaagtccttaaaaggaagaggcaacaccc	-	*
JX625391	Human H1N1pdm2009 IAVs	Human	H1N1	pdm	2009	United_Kingdom	A/England/506/2009	ggtgatgccccattccttgatcggctccgccgagatcaaaagtccttaaaaggaagaggcaacaccc	-	*
JX625423	Human H1N1pdm2009 IAVs	Human	H1N1	pdm	2009	United_Kingdom	A/England/667/2009	ggtgatgccccattccttgatcggctccgccgagatcaaaagtccttaaaaggaagaggcaacaccc	-	*
JX625471	Human H1N1pdm2009 IAVs	Human	H1N1	pdm	2009	United_Kingdom	A/England/92980479/2009	ggtgatgccccattccttgatcggctccgccgagatcaaaagtccttaaaaggaagaggcaacaccc	-	*
JX625580	Human H1N1pdm2009 IAVs	Human	H1N1	pdm	2009	United_Kingdom	A/England/94580011/2009	ggtgatgccccattccttgatcggctccgccgagatcaaaagtccttaaaaggaagaggcaacaccc	-	*
CY073501	Human H1N1pdm2009 IAVs	Human	H1N1	pdm	2009	Germany	A/Frankfurt/INS304/2009	ggtgatgccccattccttgatcggctccgccgagatcaaaagtccttaaaaggaagaggcaacaccc	-	*
CY071203	Human H1N1pdm2009 IAVs	Human	H1N1	pdm	2009	Germany	A/Frankfurt/INS401/2009	ggtgatgccccattccttgatcggctccgccgagatcaaaagtccttaaaaggaagaggcaacaccc	-	*
CY083443	Human H1N1pdm2009 IAVs	Human	H1N1	pdm	2009	USA	A/Ft.Benning/WRAIR1669P/2009	ggtgatgccccattccttgatcggctccgccgagatcaaaagtccttaaaaggaagaggcaacaccc	-	*
GQ225369	Human H1N1pdm2009 IAVs	Human	H1N1	pdm	2009	China	A/Fujian/1/2009	ggtgatgccccattccttgatcggctccgccgagatcaaaagtccttaaaaggaagaggcaacaccc	-	*
GQ288376	Human H1N1pdm2009 IAVs	Human	H1N1	pdm	2009	China	A/Fuzhou/01/2009	ggtgatgccccattccttgatcggctccgccgagatcaaaagtccttaaaaggaagaggcaacaccc	-	*
CY068942	Human H1N1pdm2009 IAVs	Human	H1N1		2009	Guam	A/Guam/NHRC0008/2009	ggtgatgccccattccttgatcggctccgccgagatcaaaagtccttaaaaggaagaggcaacaccc	-	*
CY068990	Human H1N1pdm2009 IAVs	Human	H1N1	pdm	2009	Guam	A/Guam/NHRC0013/2009	ggtgatgccccattccttgatcggctccgccgagatcaaaagtccttaaaaggaagaggcaacaccc	-	*
CY069006	Human H1N1pdm2009 IAVs	Human	H1N1	pdm	2009	Guam	A/Guam/NHRC0015/2009	ggtgatgccccattccttgatcggctccgccgagatcaaaagtccttaaaaggaagaggcaacaccc	-	*
CY069030	Human H1N1pdm2009 IAVs	Human	H1N1	pdm	2009	Guam	A/Guam/NHRC0018/2009	ggtgatgccccattccttgatcggctccgccgagatcaaaagtccttaaaaggaagaggcaacaccc	-	*
CY069046	Human H1N1pdm2009 IAVs	Human	H1N1	pdm	2009	Guam	A/Guam/NHRC0020/2009	ggtgatgccccattccttgatcggctccgccgagatcaaaagtccttaaaaggaagaggcaacaccc	-	*
CY069062	Human H1N1pdm2009 IAVs	Human	H1N1		2009	Guam	A/Guam/NHRC0022/2009	ggtgatgccccattccttgatcggctccgccgagatcaaaagtccttaaaaggaagaggcaacaccc	-	*
CY069102	Human H1N1pdm2009 IAVs	Human	H1N1		2009	Guam	A/Guam/NHRC0027/2009	ggtgatgccccattccttgatcggctccgccgagatcaaaagtccttaaaaggaagaggcaacaccc	-	*
CY071011	Human H1N1pdm2009 IAVs	Human	H1N1	pdm	2009	Guam	A/Guam/NHRC0031/2009	ggtgatgccccattccttgatcggctccgccgagatcaaaagtccttaaaaggaagaggcaacaccc	-	*
AB704426	Human H1N1pdm2009 IAVs	Human	H1N1	pdm	2009	Japan	A/Gunma/262/2009	ggtgatgccccattccttgatcggctccgccgagatcaaaagtccttaaaaggaagaggcaacaccc	-	*
AB704434	Human H1N1pdm2009 IAVs	Human	H1N1	pdm	2009	Japan	A/Gunma/263/2009	ggtgatgccccattccttgatcggctccgccgagatcaaaagtccttaaaaggaagaggcaacaccc	-	*
AB704442	Human H1N1pdm2009 IAVs	Human	H1N1		2009	Japan	A/Gunma/267/2009	ggtgatgccccattccttgatcggctccgccgagatcaaaagtccttaaaaggaagaggcaacaccc	-	*
GU480942	Human H1N1pdm2009 IAVs	Human	H1N1		2009	Russia	A/Habarovsk/01/2009	ggtgatgccccattccttgatcggctccgccgagatcaaaagtccttaaaaggaagaggcaacaccc	-	*
JQ409130	Human H1N1pdm2009 IAVs	Human	H1N1	pdm	2009	Finland	A/Helsinki/Vi1/2009	ggtgatgccccattccttgatcggctccgccgagatcaaaagtccttaaaaggaagaggcaacaccc	-	*
JQ409138	Human H1N1pdm2009 IAVs	Human	H1N1	pdm	2009	Finland	A/Helsinki/Vi2/2009	ggtgatgccccattccttgatcggctccgccgagatcaaaagtccttaaaaggaagaggcaacaccc	-	*
JQ409146	Human H1N1pdm2009 IAVs	Human	H1N1	pdm	2009	Finland	A/Helsinki/Vi3/2009	ggtgatgccccattccttgatcggctccgccgagatcaaaagtccttaaaaggaagaggcaacaccc	-	*

CY053115	Human H1N1pdm2009 IAVs	Human	H1N1	pdm	2009	USA	A/Houston/16OS/2009	ggtgatgccccattccttgatcggctccgccgagatca aaagtccttaaaaggaagaggcaacaccc	-	*
CY053123	Human H1N1pdm2009 IAVs	Human	H1N1	pdm	2009	USA	A/Houston/17OS/2009	ggtgatgccccattccttgatcggctccgccgagatca aaagtccttaaaaggaagaggcaacaccc	-	*
GQ505857	Human H1N1pdm2009 IAVs	Human	H1N1	pdm	2009	Russia	A/Irkutsk/02/2009	ggtgatgccccattccttgatcggctccgccgagatca aaagtccttaaaaggaagaggcaacaccc	-	*
CY041975	Human H1N1pdm2009 IAVs	Human	H1N1	pdm	2009	Israel	A/Israel/277/2009	ggtgatgccccattccttgatcggctccgccgagatca aaagtccttaaaaggaagaggcaacaccc	-	*
GQ283479	Human H1N1pdm2009 IAVs	Human	H1N1	pdm	2009	Italy	A/Italy/49/2009	ggtgatgccccattccttgatcggctccgccgagatca aaagtccttaaaaggaagaggcaacaccc	-	*
CY043082	Human H1N1pdm2009 IAVs	Human	H1N1		2009	Japan	A/Japan/1070/2009	ggtgatgccccattccttgatcggctccgccgagatca aaagtccttaaaaggaagaggcaacaccc	-	*
HM568084	Human H1N1pdm2009 IAVs	Human	H1N1	pdm	2009	United_Kingdom	A/England/1213/2009	ggtgatgccccattccttgatcggctccgccgagatca aaagtccttaaaaggaagaggcaacaccc	-	*
HM568108	Human H1N1pdm2009 IAVs	Human	H1N1	pdm	2009	United_Kingdom	A/England/797/2009	ggtgatgccccattccttgatcggctccgccgagatca aaagtccttaaaaggaagaggcaacaccc	-	*
HM568012	Human H1N1pdm2009 IAVs	Human	H1N1		2009	United_Kingdom	A/England/854/2009	ggtgatgccccattccttgatcggctccgccgagatca aaagtccttaaaaggaagaggcaacaccc	-	*
HM568028	Human H1N1pdm2009 IAVs	Human	H1N1	pdm	2009	United_Kingdom	A/England/862/2009	ggtgatgccccattccttgatcggctccgccgagatca aaagtccttaaaaggaagaggcaacaccc	-	*
HM568020	Human H1N1pdm2009 IAVs	Human	H1N1	pdm	2009	United_Kingdom	A/England/872/2009	ggtgatgccccattccttgatcggctccgccgagatca aaagtccttaaaaggaagaggcaacaccc	-	*
HM568036	Human H1N1pdm2009 IAVs	Human	H1N1	pdm	2009	United_Kingdom	A/England/902/2009	ggtgatgccccattccttgatcggctccgccgagatca aaagtccttaaaaggaagaggcaacaccc	-	*
HM568148	Human H1N1pdm2009 IAVs	Human	H1N1	pdm	2009	United_Kingdom	A/England/H093780034/2009	ggtgatgccccattccttgatcggctccgccgagatca aaagtccttaaaaggaagaggcaacaccc	-	*
HM568076	Human H1N1pdm2009 IAVs	Human	H1N1	pdm	2009	United_Kingdom	A/England/H094220579/2009	ggtgatgccccattccttgatcggctccgccgagatca aaagtccttaaaaggaagaggcaacaccc	-	*
CY120193	Human H1N1pdm2009 IAVs	Human	H1N1	pdm	2009	Hong_Kong	A/Hong_Kong/H090_698_V20/2009	ggtgatgccccattccttgatcggctccgccgagatca aaagtccttaaaaggaagaggcaacaccc	-	*
CY111943	Human H1N1pdm2009 IAVs	Human	H1N1	pdm	2009	Hong_Kong	A/Hong_Kong/H090_757_V10/2009	ggtgatgccccattccttgatcggctccgccgagatca aaagtccttaaaaggaagaggcaacaccc	-	*
HM189503	Human H1N1pdm2009 IAVs	Human	H1N1	pdm	2009	South_Korea	A/Korea/CJ09/2009	ggtgatgccccattccttgatcggctccgccgagatca aaagtccttaaaaggaagaggcaacaccc	-	*
HM189504	Human H1N1pdm2009 IAVs	Human	H1N1	pdm	2009	South_Korea	A/Korea/CJ15/2009	ggtgatgccccattccttgatcggctccgccgagatca aaagtccttaaaaggaagaggcaacaccc	-	*
HM189506	Human H1N1pdm2009 IAVs	Human	H1N1	pdm	2009	South_Korea	A/Korea/CJ24/2009	ggtgatgccccattccttgatcggctccgccgagatca aaagtccttaaaaggaagaggcaacaccc	-	*
JF714146	Human H1N1pdm2009 IAVs	Human	H1N1	pdm	2009	South_Korea	A/Korea/NAP_1/2009	ggtgatgccccattccttgatcggctccgccgagatca aaagtccttaaaaggaagaggcaacaccc	-	*
CY050371	Human H1N1pdm2009 IAVs	Human	H1N1	pdm	2009	South_Korea	A/Korea/S1/2009	ggtgatgccccattccttgatcggctccgccgagatca aaagtccttaaaaggaagaggcaacaccc	-	*
CY083052	Human H1N1pdm2009 IAVs	Human	H1N1		2009	Peru	A/Lima/WRAIR0285F/2009	ggtgatgccccattccttgatcggctccgccgagatca aaagtccttaaaaggaagaggcaacaccc	-	*
GQ396558	Human H1N1pdm2009 IAVs	Human	H1N1	pdm	2009	Spain	A/Madrid/GP575/2009	ggtgatgccccattccttgatcggctccgccgagatca aaagtccttaaaaggaagaggcaacaccc	-	*
CY062775	Human H1N1pdm2009 IAVs	Human	H1N1	pdm	2009	Spain	A/Madrid/INS135/2009	ggtgatgccccattccttgatcggctccgccgagatca aaagtccttaaaaggaagaggcaacaccc	-	*
CY063039	Human H1N1pdm2009 IAVs	Human	H1N1	pdm	2009	Spain	A/Madrid/INS186/2009	ggtgatgccccattccttgatcggctccgccgagatca aaagtccttaaaaggaagaggcaacaccc	-	*

CY069126	Human H1N1pdm2009 IAVs	Human	H1N1	pdm	2009	Spain	A/Madrid/INS298/2009	ggtgatgccccattccttgatcggctccgccgagatca aaagtccttaaaaggaagaggcaacaccc	-	*
CY055283	Human H1N1pdm2009 IAVs	Human	H1N1	pdm	2009	Malaysia	A/Malaysia/12617/2009	ggtgatgccccattccttgatcggctccgccgagatca aaagtccttaaaaggaagaggcaacaccc	-	*
CY119358	Human H1N1pdm2009 IAVs	Human	H1N1	pdm	2009	Malaysia	A/Malaysia/2050837/2009	ggtgatgccccattccttgatcggctccgccgagatca aaagtccttaaaaggaagaggcaacaccc	-	*
CY118203	Human H1N1pdm2009 IAVs	Human	H1N1	pdm	2009	Malaysia	A/Malaysia/2076212/2009	ggtgatgccccattccttgatcggctccgccgagatca aaagtccttaaaaggaagaggcaacaccc	-	*
CY119406	Human H1N1pdm2009 IAVs	Human	H1N1	pdm	2009	Malaysia	A/Malaysia/2086505/2009	ggtgatgccccattccttgatcggctccgccgagatca aaagtccttaaaaggaagaggcaacaccc	-	*
CY119414	Human H1N1pdm2009 IAVs	Human	H1N1	pdm	2009	Malaysia	A/Malaysia/2089841/2009	ggtgatgccccattccttgatcggctccgccgagatca aaagtccttaaaaggaagaggcaacaccc	-	*
CY119454	Human H1N1pdm2009 IAVs	Human	H1N1	pdm	2009	Malaysia	A/Malaysia/2097717/2009	ggtgatgccccattccttgatcggctccgccgagatca aaagtccttaaaaggaagaggcaacaccc	-	*
CY118214	Human H1N1pdm2009 IAVs	Human	Mixed	pdm	2009	Malaysia	A/Malaysia/2097724/2009	ggtgatgccccattccttgatcggctccgccgagatca aaagtccttaaaaggaagaggcaacaccc	-	*
CY119518	Human H1N1pdm2009 IAVs	Human	H1N1	pdm	2009	Malaysia	A/Malaysia/2097960/2009	ggtgatgccccattccttgatcggctccgccgagatca aaagtccttaaaaggaagaggcaacaccc	-	*
CY119430	Human H1N1pdm2009 IAVs	Human	H1N1	pdm	2009	Malaysia	A/Malaysia/2098001/2009	ggtgatgccccattccttgatcggctccgccgagatca aaagtccttaaaaggaagaggcaacaccc	-	*
CY119438	Human H1N1pdm2009 IAVs	Human	H1N1	pdm	2009	Malaysia	A/Malaysia/2098044/2009	ggtgatgccccattccttgatcggctccgccgagatca aaagtccttaaaaggaagaggcaacaccc	-	*
CY119446	Human H1N1pdm2009 IAVs	Human	H1N1	pdm	2009	Malaysia	A/Malaysia/2098053/2009	ggtgatgccccattccttgatcggctccgccgagatca aaagtccttaaaaggaagaggcaacaccc	-	*
CY119462	Human H1N1pdm2009 IAVs	Human	H1N1		2009	Malaysia	A/Malaysia/2126061/2009	ggtgatgccccattccttgatcggctccgccgagatca aaagtccttaaaaggaagaggcaacaccc	-	*
CY119470	Human H1N1pdm2009 IAVs	Human	H1N1	pdm	2009	Malaysia	A/Malaysia/2135565/2009	ggtgatgccccattccttgatcggctccgccgagatca aaagtccttaaaaggaagaggcaacaccc	-	*
CY119334	Human H1N1pdm2009 IAVs	Human	H1N1	pdm	2009	Malaysia	A/Malaysia/2142295/2009	ggtgatgccccattccttgatcggctccgccgagatca aaagtccttaaaaggaagaggcaacaccc	-	*
CY118248	Human H1N1pdm2009 IAVs	Human	Mixed	pdm	2009	Malaysia	A/Malaysia/2156787/2009	ggtgatgccccattccttgatcggctccgccgagatca aaagtccttaaaaggaagaggcaacaccc	-	*
CY119486	Human H1N1pdm2009 IAVs	Human	H1N1	pdm	2009	Malaysia	A/Malaysia/2156796/2009	ggtgatgccccattccttgatcggctccgccgagatca aaagtccttaaaaggaagaggcaacaccc	-	*
CY055291	Human H1N1pdm2009 IAVs	Human	H1N1	pdm	2009	Malaysia	A/Malaysia/5259/2009	ggtgatgccccattccttgatcggctccgccgagatca aaagtccttaaaaggaagaggcaacaccc	-	*
CY048929	Human H1N1pdm2009 IAVs	Human	H1N1	pdm	2009	Malaysia	A/Malaysia/820/2009	ggtgatgccccattccttgatcggctccgccgagatca aaagtccttaaaaggaagaggcaacaccc	-	*
CY072890	Human H1N1pdm2009 IAVs	Human	H1N1	pdm	2009	Nicaragua	A/Managua/12.01/2009	ggtgatgccccattccttgatcggctccgccgagatca aaagtccttaaaaggaagaggcaacaccc	-	*
CY058264	Human H1N1pdm2009 IAVs	Human	H1N1	pdm	2009	Nicaragua	A/Managua/164.01/2009	ggtgatgccccattccttgatcggctccgccgagatca aaagtccttaaaaggaagaggcaacaccc	-	*
CY072834	Human H1N1pdm2009 IAVs	Human	H1N1	pdm	2009	Nicaragua	A/Managua/1909.02/2009	ggtgatgccccattccttgatcggctccgccgagatca aaagtccttaaaaggaagaggcaacaccc	-	*
CY072794	Human H1N1pdm2009 IAVs	Human	H1N1	pdm	2009	Nicaragua	A/Managua/1984.02/2009	ggtgatgccccattccttgatcggctccgccgagatca aaagtccttaaaaggaagaggcaacaccc	-	*
CY058616	Human H1N1pdm2009 IAVs	Human	H1N1	pdm	2009	Nicaragua	A/Managua/2323.02/2009	ggtgatgccccattccttgatcggctccgccgagatca aaagtccttaaaaggaagaggcaacaccc	-	*
CY072666	Human H1N1pdm2009 IAVs	Human	H1N1	pdm	2009	Nicaragua	A/Managua/2339.03/2009	ggtgatgccccattccttgatcggctccgccgagatca aaagtccttaaaaggaagaggcaacaccc	-	*

CY072770	Human H1N1pdm2009 IAVs	Human	H1N1	pdm	2009	Nicaragua	A/Managua/287.01/2009	ggtgatgccccattccttgatcggctccgccgagatca aaagtccttaaaaggaagaggcaacaccc	-	*
CY072722	Human H1N1pdm2009 IAVs	Human	H1N1		2009	Nicaragua	A/Managua/3359.01/2009	ggtgatgccccattccttgatcggctccgccgagatca aaagtccttaaaaggaagaggcaacaccc	-	*
CY072930	Human H1N1pdm2009 IAVs	Human	H1N1	pdm	2009	Nicaragua	A/Managua/4063.01/2009	ggtgatgccccattccttgatcggctccgccgagatca aaagtccttaaaaggaagaggcaacaccc	-	*
CY120105	Human H1N1pdm2009 IAVs	Human	H1N1	pdm	2009	Hong_Kong	A/Hong_Kong/H090_663_V10/2009	ggtgatgccccattccttgatcggctccgccgagatca aaagtccttaaaaggaagaggcaacaccc	-	*
CY111623	Human H1N1pdm2009 IAVs	Human	H1N1	pdm	2009	Hong_Kong	A/Hong_Kong/H090_663_V10/2009	ggtgatgccccattccttgatcggctccgccgagatca aaagtccttaaaaggaagaggcaacaccc	-	*
CY111655	Human H1N1pdm2009 IAVs	Human	H1N1	pdm	2009	Hong_Kong	A/Hong_Kong/H090_678_V10/2009	ggtgatgccccattccttgatcggctccgccgagatca aaagtccttaaaaggaagaggcaacaccc	-	*
CY120129	Human H1N1pdm2009 IAVs	Human	H1N1	pdm	2009	Hong_Kong	A/Hong_Kong/H090_678_V10/2009	ggtgatgccccattccttgatcggctccgccgagatca aaagtccttaaaaggaagaggcaacaccc	-	*
CY111687	Human H1N1pdm2009 IAVs	Human	H1N1	pdm	2009	Hong_Kong	A/Hong_Kong/H090_683_V10/2009	ggtgatgccccattccttgatcggctccgccgagatca aaagtccttaaaaggaagaggcaacaccc	-	*
CY120153	Human H1N1pdm2009 IAVs	Human	H1N1	pdm	2009	Hong_Kong	A/Hong_Kong/H090_683_V10/2009	ggtgatgccccattccttgatcggctccgccgagatca aaagtccttaaaaggaagaggcaacaccc	-	*
CY111695	Human H1N1pdm2009 IAVs	Human	H1N1	pdm	2009	Hong_Kong	A/Hong_Kong/H090_683_V20/2009	ggtgatgccccattccttgatcggctccgccgagatca aaagtccttaaaaggaagaggcaacaccc	-	*
CY120161	Human H1N1pdm2009 IAVs	Human	H1N1	pdm	2009	Hong_Kong	A/Hong_Kong/H090_683_V20/2009	ggtgatgccccattccttgatcggctccgccgagatca aaagtccttaaaaggaagaggcaacaccc	-	*
CY111703	Human H1N1pdm2009 IAVs	Human	H1N1	pdm	2009	Hong_Kong	A/Hong_Kong/H090_684_V10/2009	ggtgatgccccattccttgatcggctccgccgagatca aaagtccttaaaaggaagaggcaacaccc	-	*
CY120169	Human H1N1pdm2009 IAVs	Human	H1N1	pdm	2009	Hong_Kong	A/Hong_Kong/H090_684_V10/2009	ggtgatgccccattccttgatcggctccgccgagatca aaagtccttaaaaggaagaggcaacaccc	-	*
CY120185	Human H1N1pdm2009 IAVs	Human	H1N1	pdm	2009	Hong_Kong	A/Hong_Kong/H090_693_V10/2009	ggtgatgccccattccttgatcggctccgccgagatca aaagtccttaaaaggaagaggcaacaccc	-	*
CY111719	Human H1N1pdm2009 IAVs	Human	H1N1	pdm	2009	Hong_Kong	A/Hong_Kong/H090_693_V10/2009	ggtgatgccccattccttgatcggctccgccgagatca aaagtccttaaaaggaagaggcaacaccc	-	*
CY111727	Human H1N1pdm2009 IAVs	Human	H1N1	pdm	2009	Hong_Kong	A/Hong_Kong/H090_693_V20/2009	ggtgatgccccattccttgatcggctccgccgagatca aaagtccttaaaaggaagaggcaacaccc	-	*
CY111735	Human H1N1pdm2009 IAVs	Human	H1N1	pdm	2009	Hong_Kong	A/Hong_Kong/H090_698_V10/2009	ggtgatgccccattccttgatcggctccgccgagatca aaagtccttaaaaggaagaggcaacaccc	-	*
CY111807	Human H1N1pdm2009 IAVs	Human	H1N1	pdm	2009	Hong_Kong	A/Hong_Kong/H090_715_V10/2009	ggtgatgccccattccttgatcggctccgccgagatca aaagtccttaaaaggaagaggcaacaccc	-	*
CY111815	Human H1N1pdm2009 IAVs	Human	H1N1	pdm	2009	Hong_Kong	A/Hong_Kong/H090_721_V10/2009	ggtgatgccccattccttgatcggctccgccgagatca aaagtccttaaaaggaagaggcaacaccc	-	*
CY120257	Human H1N1pdm2009 IAVs	Human	H1N1	pdm	2009	Hong_Kong	A/Hong_Kong/H090_721_V10/2009	ggtgatgccccattccttgatcggctccgccgagatca aaagtccttaaaaggaagaggcaacaccc	-	*
CY120305	Human H1N1pdm2009 IAVs	Human	H1N1	pdm	2009	Hong_Kong	A/Hong_Kong/H090_751_V10/2009	ggtgatgccccattccttgatcggctccgccgagatca aaagtccttaaaaggaagaggcaacaccc	-	*
CY111895	Human H1N1pdm2009 IAVs	Human	H1N1	pdm	2009	Hong_Kong	A/Hong_Kong/H090_751_V10/2009	ggtgatgccccattccttgatcggctccgccgagatca aaagtccttaaaaggaagaggcaacaccc	-	*
CY120313	Human H1N1pdm2009 IAVs	Human	H1N1	pdm	2009	Hong_Kong	A/Hong_Kong/H090_751_V20/2009	ggtgatgccccattccttgatcggctccgccgagatca aaagtccttaaaaggaagaggcaacaccc	-	*
CY111903	Human H1N1pdm2009 IAVs	Human	H1N1	pdm	2009	Hong_Kong	A/Hong_Kong/H090_751_V20/2009	ggtgatgccccattccttgatcggctccgccgagatca aaagtccttaaaaggaagaggcaacaccc	-	*
CY111911	Human H1N1pdm2009 IAVs	Human	H1N1	pdm	2009	Hong_Kong	A/Hong_Kong/H090_751_V23/2009	ggtgatgccccattccttgatcggctccgccgagatca aaagtccttaaaaggaagaggcaacaccc	-	*

CY120321	Human H1N1pdm2009 IAVs	Human	H1N1	pdm	2009	Hong_Kong	A/Hong_Kong/H090_751_V23/2009	ggtgatgccccattccttgatcggctccgccgagatcaaaagtccttaaaaggaagaggcaacaccc	-	*
CY120329	Human H1N1pdm2009 IAVs	Human	H1N1	pdm	2009	Hong_Kong	A/Hong_Kong/H090_751_V24/2009	ggtgatgccccattccttgatcggctccgccgagatcaaaagtccttaaaaggaagaggcaacaccc	-	*
CY111919	Human H1N1pdm2009 IAVs	Human	H1N1	pdm	2009	Hong_Kong	A/Hong_Kong/H090_751_V24/2009	ggtgatgccccattccttgatcggctccgccgagatcaaaagtccttaaaaggaagaggcaacaccc	-	*
CY120337	Human H1N1pdm2009 IAVs	Human	H1N1	pdm	2009	Hong_Kong	A/Hong_Kong/H090_751_V31/2009	ggtgatgccccattccttgatcggctccgccgagatcaaaagtccttaaaaggaagaggcaacaccc	-	*
CY111927	Human H1N1pdm2009 IAVs	Human	H1N1	pdm	2009	Hong_Kong	A/Hong_Kong/H090_751_V31/2009	ggtgatgccccattccttgatcggctccgccgagatcaaaagtccttaaaaggaagaggcaacaccc	-	*
CY120345	Human H1N1pdm2009 IAVs	Human	H1N1	pdm	2009	Hong_Kong	A/Hong_Kong/H090_751_V34/2009	ggtgatgccccattccttgatcggctccgccgagatcaaaagtccttaaaaggaagaggcaacaccc	-	*
HM567908	Human H1N1pdm2009 IAVs	Human	H1N1	pdm	2009	United_Kingdom	A/England/435/2009	ggtgatgccccattccttgatcggctccgccgagatcaaaagtccttaaaaggaagaggcaacaccc	-	*
CY111935	Human H1N1pdm2009 IAVs	Human	H1N1	pdm	2009	Hong_Kong	A/Hong_Kong/H090_751_V34/2009	ggtgatgccccattccttgatcggctccgccgagatcaaaagtccttaaaaggaagaggcaacaccc	-	*
CY120353	Human H1N1pdm2009 IAVs	Human	H1N1	pdm	2009	Hong_Kong	A/Hong_Kong/H090_757_V10/2009	ggtgatgccccattccttgatcggctccgccgagatcaaaagtccttaaaaggaagaggcaacaccc	-	*
CY111951	Human H1N1pdm2009 IAVs	Human	H1N1	pdm	2009	Hong_Kong	A/Hong_Kong/H090_758_V10/2009	ggtgatgccccattccttgatcggctccgccgagatcaaaagtccttaaaaggaagaggcaacaccc	-	*
CY120361	Human H1N1pdm2009 IAVs	Human	H1N1	pdm	2009	Hong_Kong	A/Hong_Kong/H090_758_V10/2009	ggtgatgccccattccttgatcggctccgccgagatcaaaagtccttaaaaggaagaggcaacaccc	-	*
CY120369	Human H1N1pdm2009 IAVs	Human	H1N1	pdm	2009	Hong_Kong	A/Hong_Kong/H090_759_V10/2009	ggtgatgccccattccttgatcggctccgccgagatcaaaagtccttaaaaggaagaggcaacaccc	-	*
HM567588	Human H1N1pdm2009 IAVs	Human	H1N1	pdm	2009	United_Kingdom	A/England/303/2009	ggtgatgccccattccttgatcggctccgccgagatcaaaagtccttaaaaggaagaggcaacaccc	-	*
HM567644	Human H1N1pdm2009 IAVs	Human	H1N1	pdm	2009	United_Kingdom	A/England/343/2009	ggtgatgccccattccttgatcggctccgccgagatcaaaagtccttaaaaggaagaggcaacaccc	-	*
HM567788	Human H1N1pdm2009 IAVs	Human	H1N1	pdm	2009	United_Kingdom	A/England/393/2009	ggtgatgccccattccttgatcggctccgccgagatcaaaagtccttaaaaggaagaggcaacaccc	-	*
HM567876	Human H1N1pdm2009 IAVs	Human	H1N1	pdm	2009	United_Kingdom	A/England/421/2009	ggtgatgccccattccttgatcggctccgccgagatcaaaagtccttaaaaggaagaggcaacaccc	-	*
HM567884	Human H1N1pdm2009 IAVs	Human	H1N1	pdm	2009	United_Kingdom	A/England/424/2009	ggtgatgccccattccttgatcggctccgccgagatcaaaagtccttaaaaggaagaggcaacaccc	-	*
HM567892	Human H1N1pdm2009 IAVs	Human	H1N1		2009	United_Kingdom	A/England/429/2009	ggtgatgccccattccttgatcggctccgccgagatcaaaagtccttaaaaggaagaggcaacaccc	-	*
HM567932	Human H1N1pdm2009 IAVs	Human	H1N1		2009	United_Kingdom	A/England/446/2009	ggtgatgccccattccttgatcggctccgccgagatcaaaagtccttaaaaggaagaggcaacaccc	-	*
HM567940	Human H1N1pdm2009 IAVs	Human	H1N1	pdm	2009	United_Kingdom	A/England/450/2009	ggtgatgccccattccttgatcggctccgccgagatcaaaagtccttaaaaggaagaggcaacaccc	-	*
HM567948	Human H1N1pdm2009 IAVs	Human	H1N1	pdm	2009	United_Kingdom	A/England/451/2009	ggtgatgccccattccttgatcggctccgccgagatcaaaagtccttaaaaggaagaggcaacaccc	-	*
CY111959	Human H1N1pdm2009 IAVs	Human	H1N1	pdm	2009	Hong_Kong	A/Hong_Kong/H090_759_V10/2009	ggtgatgccccattccttgatcggctccgccgagatcaaaagtccttaaaaggaagaggcaacaccc	-	*
CY120377	Human H1N1pdm2009 IAVs	Human	H1N1	pdm	2009	Hong_Kong	A/Hong_Kong/H090_761_V10/2009	ggtgatgccccattccttgatcggctccgccgagatcaaaagtccttaaaaggaagaggcaacaccc	-	*
CY111967	Human H1N1pdm2009 IAVs	Human	H1N1	pdm	2009	Hong_Kong	A/Hong_Kong/H090_761_V10/2009	ggtgatgccccattccttgatcggctccgccgagatcaaaagtccttaaaaggaagaggcaacaccc	-	*
CY120401	Human H1N1pdm2009 IAVs	Human	H1N1	pdm	2009	Hong_Kong	A/Hong_Kong/H090_770_V22/2009	ggtgatgccccattccttgatcggctccgccgagatcaaaagtccttaaaaggaagaggcaacaccc	-	*

CY111991	Human H1N1pdm2009 IAVs	Human	H1N1	pdm	2009	Hong_Kong	A/Hong_Kong/H090_770_V22/2009	ggtgatgccccattccttgatcggctccgccgagatcaaaagtccttaaaaggaagaggcaacaccc	-	*
CY112023	Human H1N1pdm2009 IAVs	Human	H1N1	pdm	2009	Hong_Kong	A/Hong_Kong/H090_774_V10/2009	ggtgatgccccattccttgatcggctccgccgagatcaaaagtccttaaaaggaagaggcaacaccc	-	*
CY120425	Human H1N1pdm2009 IAVs	Human	H1N1	pdm	2009	Hong_Kong	A/Hong_Kong/H090_774_V10/2009	ggtgatgccccattccttgatcggctccgccgagatcaaaagtccttaaaaggaagaggcaacaccc	-	*
CY112079	Human H1N1pdm2009 IAVs	Human	H1N1	pdm	2009	Hong_Kong	A/Hong_Kong/H090_787_V10/2009	ggtgatgccccattccttgatcggctccgccgagatcaaaagtccttaaaaggaagaggcaacaccc	-	*
CY120473	Human H1N1pdm2009 IAVs	Human	H1N1	pdm	2009	Hong_Kong	A/Hong_Kong/H090_787_V10/2009	ggtgatgccccattccttgatcggctccgccgagatcaaaagtccttaaaaggaagaggcaacaccc	-	*
HM567812	Human H1N1pdm2009 IAVs	Human	H1N1		2009	Ireland	A/Ireland/2/2009	ggtgatgccccattccttgatcggctccgccgagatcaaaagtccttaaaaggaagaggcaacaccc	-	*
HM567836	Human H1N1pdm2009 IAVs	Human	H1N1	pdm	2009	Ireland	A/Ireland/3/2009	ggtgatgccccattccttgatcggctccgccgagatcaaaagtccttaaaaggaagaggcaacaccc	-	*
GQ246615	Human H1N1pdm2009 IAVs	Human	H1N1	pdm	2009	Russia	A/Moscow/01/2009	ggtgatgccccattccttgatcggctccgccgagatcaaaagtccttaaaaggaagaggcaacaccc	-	*
CY089620	Human H1N1pdm2009 IAVs	Human	H1N1	pdm	2009	China	A/Nanchang/8008/2009	ggtgatgccccattccttgatcggctccgccgagatcaaaagtccttaaaaggaagaggcaacaccc	-	*
CY041486	Human H1N1pdm2009 IAVs	Human	H1N1	pdm	2009	USA	A/New_York/3189/2009	ggtgatgccccattccttgatcggctccgccgagatcaaaagtccttaaaaggaagaggcaacaccc	-	*
CY040665	Human H1N1pdm2009 IAVs	Human	H1N1	pdm	2009	USA	A/New_York/3202/2009	ggtgatgccccattccttgatcggctccgccgagatcaaaagtccttaaaaggaagaggcaacaccc	-	*
CY040673	Human H1N1pdm2009 IAVs	Human	H1N1	pdm	2009	USA	A/New_York/3218/2009	ggtgatgccccattccttgatcggctccgccgagatcaaaagtccttaaaaggaagaggcaacaccc	-	*
CY040681	Human H1N1pdm2009 IAVs	Human	H1N1	pdm	2009	USA	A/New_York/3222/2009	ggtgatgccccattccttgatcggctccgccgagatcaaaagtccttaaaaggaagaggcaacaccc	-	*
CY040697	Human H1N1pdm2009 IAVs	Human	H1N1	pdm	2009	USA	A/New_York/3232/2009	ggtgatgccccattccttgatcggctccgccgagatcaaaagtccttaaaaggaagaggcaacaccc	-	*
CY086973	Human H1N1pdm2009 IAVs	Human	H1N1	pdm	2009	USA	A/New_York/3198/2009	ggtgatgccccattccttgatcggctccgccgagatcaaaagtccttaaaaggaagaggcaacaccc	-	*
CY040721	Human H1N1pdm2009 IAVs	Human	H1N1	pdm	2009	USA	A/New_York/3238/2009	ggtgatgccccattccttgatcggctccgccgagatcaaaagtccttaaaaggaagaggcaacaccc	-	*
CY040842	Human H1N1pdm2009 IAVs	Human	H1N1		2009	USA	A/New_York/3262/2009	ggtgatgccccattccttgatcggctccgccgagatcaaaagtccttaaaaggaagaggcaacaccc	-	*
CY047354	Human H1N1pdm2009 IAVs	Human	H1N1	pdm	2009	USA	A/New_York/3365/2009	ggtgatgccccattccttgatcggctccgccgagatcaaaagtccttaaaaggaagaggcaacaccc	-	*
JX625548	Human H1N1pdm2009 IAVs	Human	H1N1	pdm	2009	United_Kingdom	A/Northern_Ireland/94480417/2009	ggtgatgccccattccttgatcggctccgccgagatcaaaagtccttaaaaggaagaggcaacaccc	-	*
GQ329096	Human H1N1pdm2009 IAVs	Human	H1N1	pdm	2009	France	A/Paris/2709/2009	ggtgatgccccattccttgatcggctccgccgagatcaaaagtccttaaaaggaagaggcaacaccc	-	*
KF411266	Human H1N1pdm2009 IAVs	Human	H1N1	pdm	2009	China	A/Qingdao/1508/2009	ggtgatgccccattccttgatcggctccgccgagatcaaaagtccttaaaaggaagaggcaacaccc	-	*
KF411273	Human H1N1pdm2009 IAVs	Human	H1N1	pdm	2009	China	A/Qingdao/1631/2009	ggtgatgccccattccttgatcggctccgccgagatcaaaagtccttaaaaggaagaggcaacaccc	-	*
FN434464	Human H1N1pdm2009 IAVs	Human	H1N1	pdm	2009	Canada	A/Quebec/144147/2009	ggtgatgccccattccttgatcggctccgccgagatcaaaagtccttaaaaggaagaggcaacaccc	-	*
FN434472	Human H1N1pdm2009 IAVs	Human	H1N1		2009	Canada	A/Quebec/144180/2009	ggtgatgccccattccttgatcggctccgccgagatcaaaagtccttaaaaggaagaggcaacaccc	-	*
CY083116	Human H1N1pdm2009 IAVs	Human	H1N1	pdm	2009	Ecuador	A/Quito/WRAIR0617N/2009	ggtgatgccccattccttgatcggctccgccgagatcaaaagtccttaaaaggaagaggcaacaccc	-	*

GQ329068	Human H1N1pdm2009 IAVs	Human	H1N1	pdm	2009	France	A/Rennes/2671/2009	ggtgatgccccattccttgatcggctccgccgagatca aaagtccttaaaaggaagaggcaacaccc	-	*
JQ431329	Human H1N1pdm2009 IAVs	Human	H1N1	pdm	2009	France	A/Reunion/2378_1_M1E/2009	ggtgatgccccattccttgatcggctccgccgagatca aaagtccttaaaaggaagaggcaacaccc	-	*
JQ431333	Human H1N1pdm2009 IAVs	Human	H1N1	pdm	2009	France	A/Reunion/2956_1_M2E/2009	ggtgatgccccattccttgatcggctccgccgagatca aaagtccttaaaaggaagaggcaacaccc	-	*
JQ431334	Human H1N1pdm2009 IAVs	Human	H1N1	pdm	2009	France	A/Reunion/2956_2_M1E/2009	ggtgatgccccattccttgatcggctccgccgagatca aaagtccttaaaaggaagaggcaacaccc	-	*
CY083946	Human H1N1pdm2009 IAVs	Human	H1N1	pdm	2009	USA	A/San_Diego/INS194/2009	ggtgatgccccattccttgatcggctccgccgagatca aaagtccttaaaaggaagaggcaacaccc	-	*
CY107236	Human H1N1pdm2009 IAVs	Human	H1N1	pdm	2009	United_Kingdom	A/Scotland/Aberdeen_12/2009	ggtgatgccccattccttgatcggctccgccgagatca aaagtccttaaaaggaagaggcaacaccc	-	*
CY107318	Human H1N1pdm2009 IAVs	Human	H1N1	pdm	2009	United_Kingdom	A/Scotland/Aberdeen_8/2009	ggtgatgccccattccttgatcggctccgccgagatca aaagtccttaaaaggaagaggcaacaccc	-	*
CY107462	Human H1N1pdm2009 IAVs	Human	H1N1		2009	United_Kingdom	A/Scotland/Dumfries_425403/2009	ggtgatgccccattccttgatcggctccgccgagatca aaagtccttaaaaggaagaggcaacaccc	-	*
CY107372	Human H1N1pdm2009 IAVs	Human	H1N1		2009	United_Kingdom	A/Scotland/Dundee_09V500266/2009	ggtgatgccccattccttgatcggctccgccgagatca aaagtccttaaaaggaagaggcaacaccc	-	*
CY107379	Human H1N1pdm2009 IAVs	Human	H1N1	pdm	2009	United_Kingdom	A/Scotland/Dundee_09V500321/2009	ggtgatgccccattccttgatcggctccgccgagatca aaagtccttaaaaggaagaggcaacaccc	-	*
CY107815	Human H1N1pdm2009 IAVs	Human	H1N1	pdm	2009	United_Kingdom	A/Scotland/EastKilbride_425383/2009	ggtgatgccccattccttgatcggctccgccgagatca aaagtccttaaaaggaagaggcaacaccc	-	*
CY107512	Human H1N1pdm2009 IAVs	Human	H1N1		2009	United_Kingdom	A/Scotland/Edinburgh_20308/2009	ggtgatgccccattccttgatcggctccgccgagatca aaagtccttaaaaggaagaggcaacaccc	-	*
CY107524	Human H1N1pdm2009 IAVs	Human	H1N1	pdm	2009	United_Kingdom	A/Scotland/Edinburgh_20665/2009	ggtgatgccccattccttgatcggctccgccgagatca aaagtccttaaaaggaagaggcaacaccc	-	*
CY107537	Human H1N1pdm2009 IAVs	Human	H1N1	pdm	2009	United_Kingdom	A/Scotland/Edinburgh_23151/2009	ggtgatgccccattccttgatcggctccgccgagatca aaagtccttaaaaggaagaggcaacaccc	-	*
CY045179	Human H1N1pdm2009 IAVs	Human	H1N1	pdm	2009	USA	A/New_York/3959/2009	ggtgatgccccattccttgatcggctccgccgagatca aaagtccttaaaaggaagaggcaacaccc	-	*
CY047386	Human H1N1pdm2009 IAVs	Human	H1N1	pdm	2009	USA	A/New_York/4100/2009	ggtgatgccccattccttgatcggctccgccgagatca aaagtccttaaaaggaagaggcaacaccc	-	*
GU290051	Human H1N1pdm2009 IAVs	Human	H1N1	pdm	2009	Czech_Republic	A/Prague/197_81395/2009	ggtgatgccccattccttgatcggctccgccgagatca aaagtccttaaaaggaagaggcaacaccc	-	*
CY107469	Human H1N1pdm2009 IAVs	Human	H1N1	pdm	2009	United_Kingdom	A/Scotland/Fife_17712/2009	ggtgatgccccattccttgatcggctccgccgagatca aaagtccttaaaaggaagaggcaacaccc	-	*
CY107610	Human H1N1pdm2009 IAVs	Human	H1N1	pdm	2009	United_Kingdom	A/Scotland/Glasgow_413608/2009	ggtgatgccccattccttgatcggctccgccgagatca aaagtccttaaaaggaagaggcaacaccc	-	*
CY107709	Human H1N1pdm2009 IAVs	Human	H1N1	pdm	2009	United_Kingdom	A/Scotland/Glasgow_443734/2009	ggtgatgccccattccttgatcggctccgccgagatca aaagtccttaaaaggaagaggcaacaccc	-	*
CY107257	Human H1N1pdm2009 IAVs	Human	H1N1	pdm	2009	United_Kingdom	A/Scotland/Inverness_15/2009	ggtgatgccccattccttgatcggctccgccgagatca aaagtccttaaaaggaagaggcaacaccc	-	*
CY107761	Human H1N1pdm2009 IAVs	Human	H1N1	pdm	2009	United_Kingdom	A/Scotland/Paisley_415110/2009	ggtgatgccccattccttgatcggctccgccgagatca aaagtccttaaaaggaagaggcaacaccc	-	*
CY107351	Human H1N1pdm2009 IAVs	Human	H1N1	pdm	2009	United_Kingdom	A/Scotland/StAndrews_09V500252/2009	ggtgatgccccattccttgatcggctccgccgagatca aaagtccttaaaaggaagaggcaacaccc	-	*
CY107393	Human H1N1pdm2009 IAVs	Human	H1N1	pdm	2009	United_Kingdom	A/Scotland/StAndrews_09V500630/2009	ggtgatgccccattccttgatcggctccgccgagatca aaagtccttaaaaggaagaggcaacaccc	-	*
CY072866	Human H1N1pdm2009 IAVs	Human	H1N1	pdm	2009	Nicaragua	A/Managua/4147.01/2009	ggtgatgccccattccttgatcggctccgccgagatca aaagtccttaaaaggaagaggcaacaccc	-	*

CY058568	Human H1N1pdm2009 IAVs	Human	H1N1	pdm	2009	Nicaragua	A/Managua/4218.01/2009	ggtgatgccccattccttgatcggctccgccgagatca aaagtccttaaaaggaagaggcaacaccc	-	*
CY073605	Human H1N1pdm2009 IAVs	Human	H1N1	pdm	2009	Nicaragua	A/Managua/4451.02/2009	ggtgatgccccattccttgatcggctccgccgagatca aaagtccttaaaaggaagaggcaacaccc	-	*
CY072658	Human H1N1pdm2009 IAVs	Human	H1N1	pdm	2009	Nicaragua	A/Managua/4570.01/2009	ggtgatgccccattccttgatcggctccgccgagatca aaagtccttaaaaggaagaggcaacaccc	-	*
CY073533	Human H1N1pdm2009 IAVs	Human	H1N1	pdm	2009	Nicaragua	A/Managua/466.01/2009	ggtgatgccccattccttgatcggctccgccgagatca aaagtccttaaaaggaagaggcaacaccc	-	*
CY090069	Human H1N1pdm2009 IAVs	Human	H1N1	pdm	2009	Nicaragua	A/Managua/4877.02/2009	ggtgatgccccattccttgatcggctccgccgagatca aaagtccttaaaaggaagaggcaacaccc	-	*
CY072954	Human H1N1pdm2009 IAVs	Human	H1N1	pdm	2009	Nicaragua	A/Managua/5258.02/2009	ggtgatgccccattccttgatcggctccgccgagatca aaagtccttaaaaggaagaggcaacaccc	-	*
CY072786	Human H1N1pdm2009 IAVs	Human	H1N1	pdm	2009	Nicaragua	A/Managua/5401.01/2009	ggtgatgccccattccttgatcggctccgccgagatca aaagtccttaaaaggaagaggcaacaccc	-	*
CY083491	Human H1N1pdm2009 IAVs	Human	H1N1	pdm	2009	Mexico	A/Mexico_City/WRAIR1691N/2009	ggtgatgccccattccttgatcggctccgccgagatca aaagtccttaaaaggaagaggcaacaccc	-	*
CY069238	Human H1N1pdm2009 IAVs	Human	H1N1	pdm	2009	Germany	A/Munich/INS365/2009	ggtgatgccccattccttgatcggctccgccgagatca aaagtccttaaaaggaagaggcaacaccc	-	*
CY065908	Human H1N1pdm2009 IAVs	Human	H1N1	pdm	2009	Netherlands	A/Netherlands/1083/2009	ggtgatgccccattccttgatcggctccgccgagatca aaagtccttaaaaggaagaggcaacaccc	-	*
CY065916	Human H1N1pdm2009 IAVs	Human	H1N1	pdm	2009	Netherlands	A/Netherlands/1084/2009	ggtgatgccccattccttgatcggctccgccgagatca aaagtccttaaaaggaagaggcaacaccc	-	*
CY065884	Human H1N1pdm2009 IAVs	Human	H1N1	pdm	2009	Netherlands	A/Netherlands/1093/2009	ggtgatgccccattccttgatcggctccgccgagatca aaagtccttaaaaggaagaggcaacaccc	-	*
GQ202724	Human H1N1pdm2009 IAVs	Human	H1N1	pdm	2009	Russia	A/Moscow/IIV01/2009	ggtgatgccccattccttgatcggctccgccgagatca aaagtccttaaaaggaagaggcaacaccc	-	*
GQ247725	Human H1N1pdm2009 IAVs	Human	H1N1	pdm	2009	Russia	A/Moscow/IIV02/2009	ggtgatgccccattccttgatcggctccgccgagatca aaagtccttaaaaggaagaggcaacaccc	-	*
GQ330647	Human H1N1pdm2009 IAVs	Human	H1N1	pdm	2009	Russia	A/Moscow/IIV03/2009	ggtgatgccccattccttgatcggctccgccgagatca aaagtccttaaaaggaagaggcaacaccc	-	*
CY065135	Human H1N1pdm2009 IAVs	Human	H1N1	pdm	2009	Netherlands	A/Netherlands/2143/2009	ggtgatgccccattccttgatcggctccgccgagatca aaagtccttaaaaggaagaggcaacaccc	-	*
FJ984339	Human H1N1pdm2009 IAVs	Human	H1N1	pdm	2009	USA	A/New_York/06/2009	ggtgatgccccattccttgatcggctccgccgagatca aaagtccttaaaaggaagaggcaacaccc	-	*
FJ984370	Human H1N1pdm2009 IAVs	Human	H1N1	pdm	2009	USA	A/New_York/10/2009	ggtgatgccccattccttgatcggctccgccgagatca aaagtccttaaaaggaagaggcaacaccc	-	*
FJ984343	Human H1N1pdm2009 IAVs	Human	H1N1	pdm	2009	USA	A/New_York/11/2009	ggtgatgccccattccttgatcggctccgccgagatca aaagtccttaaaaggaagaggcaacaccc	-	*
FJ984334	Human H1N1pdm2009 IAVs	Human	H1N1	pdm	2009	USA	A/New_York/12/2009	ggtgatgccccattccttgatcggctccgccgagatca aaagtccttaaaaggaagaggcaacaccc	-	*
FJ984377	Human H1N1pdm2009 IAVs	Human	H1N1	pdm	2009	USA	A/New_York/15/2009	ggtgatgccccattccttgatcggctccgccgagatca aaagtccttaaaaggaagaggcaacaccc	-	*
FJ984389	Human H1N1pdm2009 IAVs	Human	H1N1	pdm	2009	USA	A/New_York/19/2009	ggtgatgccccattccttgatcggctccgccgagatca aaagtccttaaaaggaagaggcaacaccc	-	*
FJ984361	Human H1N1pdm2009 IAVs	Human	H1N1	pdm	2009	USA	A/New_York/23/2009	ggtgatgccccattccttgatcggctccgccgagatca aaagtccttaaaaggaagaggcaacaccc	-	*
CY041617	Human H1N1pdm2009 IAVs	Human	H1N1	pdm	2009	USA	A/New_York/3002/2009	ggtgatgccccattccttgatcggctccgccgagatca aaagtccttaaaaggaagaggcaacaccc	-	*
CY040046	Human H1N1pdm2009 IAVs	Human	H1N1	pdm	2009	USA	A/New_York/3007/2009	ggtgatgccccattccttgatcggctccgccgagatca aaagtccttaaaaggaagaggcaacaccc	-	*

CY040003	Human H1N1pdm2009 IAVs	Human	H1N1	pdm	2009	USA	A/New_York/3008/2009	ggtgatgccccattccttgatcggctccgccgagatcaaaagtccttaaaaggaagaggcaacaccc	-	*
CY040011	Human H1N1pdm2009 IAVs	Human	H1N1	pdm	2009	USA	A/New_York/3012/2009	ggtgatgccccattccttgatcggctccgccgagatcaaaagtccttaaaaggaagaggcaacaccc	-	*
CY040019	Human H1N1pdm2009 IAVs	Human	H1N1	pdm	2009	USA	A/New_York/3014/2009	ggtgatgccccattccttgatcggctccgccgagatcaaaagtccttaaaaggaagaggcaacaccc	-	*
FJ984356	Human H1N1pdm2009 IAVs	Human	H1N1	pdm	2009	USA	A/New_York/31/2009	ggtgatgccccattccttgatcggctccgccgagatcaaaagtccttaaaaggaagaggcaacaccc	-	*
CY047322	Human H1N1pdm2009 IAVs	Human	H1N1		2009	USA	A/New_York/3124/2009	ggtgatgccccattccttgatcggctccgccgagatcaaaagtccttaaaaggaagaggcaacaccc	-	*
CY041633	Human H1N1pdm2009 IAVs	Human	H1N1	pdm	2009	USA	A/New_York/3149/2009	ggtgatgccccattccttgatcggctccgccgagatcaaaagtccttaaaaggaagaggcaacaccc	-	*
CY041054	Human H1N1pdm2009 IAVs	Human	H1N1	pdm	2009	USA	A/New_York/3166/2009	ggtgatgccccattccttgatcggctccgccgagatcaaaagtccttaaaaggaagaggcaacaccc	-	*
CY041062	Human H1N1pdm2009 IAVs	Human	H1N1		2009	USA	A/New_York/3168/2009	ggtgatgccccattccttgatcggctccgccgagatcaaaagtccttaaaaggaagaggcaacaccc	-	*
CY041070	Human H1N1pdm2009 IAVs	Human	H1N1	pdm	2009	USA	A/New_York/3169/2009	ggtgatgccccattccttgatcggctccgccgagatcaaaagtccttaaaaggaagaggcaacaccc	-	*
CY041086	Human H1N1pdm2009 IAVs	Human	H1N1	pdm	2009	USA	A/New_York/3172/2009	ggtgatgccccattccttgatcggctccgccgagatcaaaagtccttaaaaggaagaggcaacaccc	-	*
CY041102	Human H1N1pdm2009 IAVs	Human	H1N1		2009	USA	A/New_York/3176/2009	ggtgatgccccattccttgatcggctccgccgagatcaaaagtccttaaaaggaagaggcaacaccc	-	*
CY041166	Human H1N1pdm2009 IAVs	Human	H1N1	pdm	2009	USA	A/New_York/3185/2009	ggtgatgccccattccttgatcggctccgccgagatcaaaagtccttaaaaggaagaggcaacaccc	-	*
CY041174	Human H1N1pdm2009 IAVs	Human	H1N1	pdm	2009	USA	A/New_York/3186/2009	ggtgatgccccattccttgatcggctccgccgagatcaaaagtccttaaaaggaagaggcaacaccc	-	*
CY041190	Human H1N1pdm2009 IAVs	Human	H1N1	pdm	2009	USA	A/New_York/3188/2009	ggtgatgccccattccttgatcggctccgccgagatcaaaagtccttaaaaggaagaggcaacaccc	-	*
CY041198	Human H1N1pdm2009 IAVs	Human	H1N1	pdm	2009	USA	A/New_York/3190/2009	ggtgatgccccattccttgatcggctccgccgagatcaaaagtccttaaaaggaagaggcaacaccc	-	*
CY040649	Human H1N1pdm2009 IAVs	Human	H1N1	pdm	2009	USA	A/New_York/3194/2009	ggtgatgccccattccttgatcggctccgccgagatcaaaagtccttaaaaggaagaggcaacaccc	-	*
CY041502	Human H1N1pdm2009 IAVs	Human	H1N1		2009	USA	A/New_York/3195/2009	ggtgatgccccattccttgatcggctccgccgagatcaaaagtccttaaaaggaagaggcaacaccc	-	*
CY041510	Human H1N1pdm2009 IAVs	Human	H1N1	pdm	2009	USA	A/New_York/3196/2009	ggtgatgccccattccttgatcggctccgccgagatcaaaagtccttaaaaggaagaggcaacaccc	-	*
CY041206	Human H1N1pdm2009 IAVs	Human	H1N1	pdm	2009	USA	A/New_York/3199/2009	ggtgatgccccattccttgatcggctccgccgagatcaaaagtccttaaaaggaagaggcaacaccc	-	*
CY040657	Human H1N1pdm2009 IAVs	Human	H1N1	pdm	2009	USA	A/New_York/3201/2009	ggtgatgccccattccttgatcggctccgccgagatcaaaagtccttaaaaggaagaggcaacaccc	-	*
CY041526	Human H1N1pdm2009 IAVs	Human	H1N1	pdm	2009	USA	A/New_York/3204/2009	ggtgatgccccattccttgatcggctccgccgagatcaaaagtccttaaaaggaagaggcaacaccc	-	*
CY041534	Human H1N1pdm2009 IAVs	Human	H1N1	pdm	2009	USA	A/New_York/3205/2009	ggtgatgccccattccttgatcggctccgccgagatcaaaagtccttaaaaggaagaggcaacaccc	-	*
CY041738	Human H1N1pdm2009 IAVs	Human	H1N1	pdm	2009	USA	A/New_York/3206/2009	ggtgatgccccattccttgatcggctccgccgagatcaaaagtccttaaaaggaagaggcaacaccc	-	*
CY041609	Human H1N1pdm2009 IAVs	Human	H1N1	pdm	2009	USA	A/New_York/3207/2009	ggtgatgccccattccttgatcggctccgccgagatcaaaagtccttaaaaggaagaggcaacaccc	-	*
CY041545	Human H1N1pdm2009 IAVs	Human	H1N1	pdm	2009	USA	A/New_York/3209/2009	ggtgatgccccattccttgatcggctccgccgagatcaaaagtccttaaaaggaagaggcaacaccc	-	*

CY041553	Human H1N1pdm2009 IAVs	Human	H1N1	pdm	2009	USA	A/New_York/3210/2009	ggtgatgccccattccttgatcggctccgccgagatca aaagtccttaaaaggaagaggcaacaccc	-	*
CY041561	Human H1N1pdm2009 IAVs	Human	H1N1	pdm	2009	USA	A/New_York/3211/2009	ggtgatgccccattccttgatcggctccgccgagatca aaagtccttaaaaggaagaggcaacaccc	-	*
CY041126	Human H1N1pdm2009 IAVs	Human	H1N1	pdm	2009	USA	A/New_York/3214/2009	ggtgatgccccattccttgatcggctccgccgagatca aaagtccttaaaaggaagaggcaacaccc	-	*
CY041134	Human H1N1pdm2009 IAVs	Human	H1N1	pdm	2009	USA	A/New_York/3215/2009	ggtgatgccccattccttgatcggctccgccgagatca aaagtccttaaaaggaagaggcaacaccc	-	*
CY040609	Human H1N1pdm2009 IAVs	Human	H1N1	pdm	2009	USA	A/New_York/3219/2009	ggtgatgccccattccttgatcggctccgccgagatca aaagtccttaaaaggaagaggcaacaccc	-	*
CY040617	Human H1N1pdm2009 IAVs	Human	H1N1	pdm	2009	USA	A/New_York/3220/2009	ggtgatgccccattccttgatcggctccgccgagatca aaagtccttaaaaggaagaggcaacaccc	-	*
CY040625	Human H1N1pdm2009 IAVs	Human	H1N1	pdm	2009	USA	A/New_York/3221/2009	ggtgatgccccattccttgatcggctccgccgagatca aaagtccttaaaaggaagaggcaacaccc	-	*
CY041569	Human H1N1pdm2009 IAVs	Human	H1N1	pdm	2009	USA	A/New_York/3225/2009	ggtgatgccccattccttgatcggctccgccgagatca aaagtccttaaaaggaagaggcaacaccc	-	*
CY041577	Human H1N1pdm2009 IAVs	Human	H1N1	pdm	2009	USA	A/New_York/3227/2009	ggtgatgccccattccttgatcggctccgccgagatca aaagtccttaaaaggaagaggcaacaccc	-	*
CY041585	Human H1N1pdm2009 IAVs	Human	H1N1	pdm	2009	USA	A/New_York/3230/2009	ggtgatgccccattccttgatcggctccgccgagatca aaagtccttaaaaggaagaggcaacaccc	-	*
CY041746	Human H1N1pdm2009 IAVs	Human	H1N1	pdm	2009	USA	A/New_York/3231/2009	ggtgatgccccattccttgatcggctccgccgagatca aaagtccttaaaaggaagaggcaacaccc	-	*
CY040633	Human H1N1pdm2009 IAVs	Human	H1N1	pdm	2009	USA	A/New_York/3234/2009	ggtgatgccccattccttgatcggctccgccgagatca aaagtccttaaaaggaagaggcaacaccc	-	*
CY040705	Human H1N1pdm2009 IAVs	Human	H1N1	pdm	2009	USA	A/New_York/3235/2009	ggtgatgccccattccttgatcggctccgccgagatca aaagtccttaaaaggaagaggcaacaccc	-	*
CY041593	Human H1N1pdm2009 IAVs	Human	H1N1	pdm	2009	USA	A/New_York/3236/2009	ggtgatgccccattccttgatcggctccgccgagatca aaagtccttaaaaggaagaggcaacaccc	-	*
CY040729	Human H1N1pdm2009 IAVs	Human	H1N1	pdm	2009	USA	A/New_York/3239/2009	ggtgatgccccattccttgatcggctccgccgagatca aaagtccttaaaaggaagaggcaacaccc	-	*
CY040746	Human H1N1pdm2009 IAVs	Human	H1N1	pdm	2009	USA	A/New_York/3242/2009	ggtgatgccccattccttgatcggctccgccgagatca aaagtccttaaaaggaagaggcaacaccc	-	*
CY040754	Human H1N1pdm2009 IAVs	Human	H1N1	pdm	2009	USA	A/New_York/3243/2009	ggtgatgccccattccttgatcggctccgccgagatca aaagtccttaaaaggaagaggcaacaccc	-	*
CY040770	Human H1N1pdm2009 IAVs	Human	H1N1	pdm	2009	USA	A/New_York/3245/2009	ggtgatgccccattccttgatcggctccgccgagatca aaagtccttaaaaggaagaggcaacaccc	-	*
CY040778	Human H1N1pdm2009 IAVs	Human	H1N1	pdm	2009	USA	A/New_York/3246/2009	ggtgatgccccattccttgatcggctccgccgagatca aaagtccttaaaaggaagaggcaacaccc	-	*
CY041730	Human H1N1pdm2009 IAVs	Human	H1N1	pdm	2009	USA	A/New_York/3247/2009	ggtgatgccccattccttgatcggctccgccgagatca aaagtccttaaaaggaagaggcaacaccc	-	*
CY086997	Human H1N1pdm2009 IAVs	Human	H1N1	pdm	2009	USA	A/New_York/3251/2009	ggtgatgccccattccttgatcggctccgccgagatca aaagtccttaaaaggaagaggcaacaccc	-	*
CY040786	Human H1N1pdm2009 IAVs	Human	H1N1	pdm	2009	USA	A/New_York/3252/2009	ggtgatgccccattccttgatcggctccgccgagatca aaagtccttaaaaggaagaggcaacaccc	-	*
CY040794	Human H1N1pdm2009 IAVs	Human	H1N1	pdm	2009	USA	A/New_York/3253/2009	ggtgatgccccattccttgatcggctccgccgagatca aaagtccttaaaaggaagaggcaacaccc	-	*
CY040802	Human H1N1pdm2009 IAVs	Human	H1N1		2009	USA	A/New_York/3255/2009	ggtgatgccccattccttgatcggctccgccgagatca aaagtccttaaaaggaagaggcaacaccc	-	*
CY040810	Human H1N1pdm2009 IAVs	Human	H1N1	pdm	2009	USA	A/New_York/3257/2009	ggtgatgccccattccttgatcggctccgccgagatca aaagtccttaaaaggaagaggcaacaccc	-	*

CY041142	Human H1N1pdm2009 IAVs	Human	H1N1		2009	USA	A/New_York/3258/2009	ggtgatgccccattccttgatcggctccgccgagatca<br>aaagtccttaaaaggaagaggcaacaccc	-	*
CY040818	Human H1N1pdm2009 IAVs	Human	H1N1	pdm	2009	USA	A/New_York/3259/2009	ggtgatgccccattccttgatcggctccgccgagatca<br>aaagtccttaaaaggaagaggcaacaccc	-	*
CY040826	Human H1N1pdm2009 IAVs	Human	H1N1	pdm	2009	USA	A/New_York/3260/2009	ggtgatgccccattccttgatcggctccgccgagatca<br>aaagtccttaaaaggaagaggcaacaccc	-	*
CY040858	Human H1N1pdm2009 IAVs	Human	H1N1	pdm	2009	USA	A/New_York/3264/2009	ggtgatgccccattccttgatcggctccgccgagatca<br>aaagtccttaaaaggaagaggcaacaccc	-	*
CY040866	Human H1N1pdm2009 IAVs	Human	H1N1	pdm	2009	USA	A/New_York/3265/2009	ggtgatgccccattccttgatcggctccgccgagatca<br>aaagtccttaaaaggaagaggcaacaccc	-	*
CY041649	Human H1N1pdm2009 IAVs	Human	H1N1	pdm	2009	USA	A/New_York/3313/2009	ggtgatgccccattccttgatcggctccgccgagatca<br>aaagtccttaaaaggaagaggcaacaccc	-	*
CY047346	Human H1N1pdm2009 IAVs	Human	H1N1	pdm	2009	USA	A/New_York/3345/2009	ggtgatgccccattccttgatcggctccgccgagatca<br>aaagtccttaaaaggaagaggcaacaccc	-	*
CY041770	Human H1N1pdm2009 IAVs	Human	H1N1	pdm	2009	USA	A/New_York/3354/2009	ggtgatgccccattccttgatcggctccgccgagatca<br>aaagtccttaaaaggaagaggcaacaccc	-	*
CY047338	Human H1N1pdm2009 IAVs	Human	H1N1	pdm	2009	USA	A/New_York/3364/2009	ggtgatgccccattccttgatcggctccgccgagatca<br>aaagtccttaaaaggaagaggcaacaccc	-	*
CY041810	Human H1N1pdm2009 IAVs	Human	H1N1	pdm	2009	USA	A/New_York/3408/2009	ggtgatgccccattccttgatcggctccgccgagatca<br>aaagtccttaaaaggaagaggcaacaccc	-	*
CY041762	Human H1N1pdm2009 IAVs	Human	H1N1		2009	USA	A/New_York/3413/2009	ggtgatgccccattccttgatcggctccgccgagatca<br>aaagtccttaaaaggaagaggcaacaccc	-	*
CY043159	Human H1N1pdm2009 IAVs	Human	H1N1	pdm	2009	USA	A/New_York/3418/2009	ggtgatgccccattccttgatcggctccgccgagatca<br>aaagtccttaaaaggaagaggcaacaccc	-	*
CY043215	Human H1N1pdm2009 IAVs	Human	H1N1	pdm	2009	USA	A/New_York/3420/2009	ggtgatgccccattccttgatcggctccgccgagatca<br>aaagtccttaaaaggaagaggcaacaccc	-	*
CY041802	Human H1N1pdm2009 IAVs	Human	H1N1		2009	USA	A/New_York/3468/2009	ggtgatgccccattccttgatcggctccgccgagatca<br>aaagtccttaaaaggaagaggcaacaccc	-	*
CY043295	Human H1N1pdm2009 IAVs	Human	H1N1	pdm	2009	USA	A/New_York/3469/2009	ggtgatgccccattccttgatcggctccgccgagatca<br>aaagtccttaaaaggaagaggcaacaccc	-	*
CY043183	Human H1N1pdm2009 IAVs	Human	H1N1	pdm	2009	USA	A/New_York/3488/2009	ggtgatgccccattccttgatcggctccgccgagatca<br>aaagtccttaaaaggaagaggcaacaccc	-	*
CY043239	Human H1N1pdm2009 IAVs	Human	H1N1	pdm	2009	USA	A/New_York/3502/2009	ggtgatgccccattccttgatcggctccgccgagatca<br>aaagtccttaaaaggaagaggcaacaccc	-	*
CY043247	Human H1N1pdm2009 IAVs	Human	H1N1	pdm	2009	USA	A/New_York/3509/2009	ggtgatgccccattccttgatcggctccgccgagatca<br>aaagtccttaaaaggaagaggcaacaccc	-	*
CY044132	Human H1N1pdm2009 IAVs	Human	H1N1		2009	USA	A/New_York/3516/2009	ggtgatgccccattccttgatcggctccgccgagatca<br>aaagtccttaaaaggaagaggcaacaccc	-	*
CY044140	Human H1N1pdm2009 IAVs	Human	H1N1	pdm	2009	USA	A/New_York/3528/2009	ggtgatgccccattccttgatcggctccgccgagatca<br>aaagtccttaaaaggaagaggcaacaccc	-	*
CY043143	Human H1N1pdm2009 IAVs	Human	H1N1	pdm	2009	USA	A/New_York/3529/2009	ggtgatgccccattccttgatcggctccgccgagatca<br>aaagtccttaaaaggaagaggcaacaccc	-	*
CY041778	Human H1N1pdm2009 IAVs	Human	H1N1	pdm	2009	USA	A/New_York/3532/2009	ggtgatgccccattccttgatcggctccgccgagatca<br>aaagtccttaaaaggaagaggcaacaccc	-	*
CY043311	Human H1N1pdm2009 IAVs	Human	H1N1	pdm	2009	USA	A/New_York/3534/2009	ggtgatgccccattccttgatcggctccgccgagatca<br>aaagtccttaaaaggaagaggcaacaccc	-	*
CY043263	Human H1N1pdm2009 IAVs	Human	H1N1	pdm	2009	USA	A/New_York/3552/2009	ggtgatgccccattccttgatcggctccgccgagatca<br>aaagtccttaaaaggaagaggcaacaccc	-	*
CY043271	Human H1N1pdm2009 IAVs	Human	H1N1	pdm	2009	USA	A/New_York/3563/2009	ggtgatgccccattccttgatcggctccgccgagatca<br>aaagtccttaaaaggaagaggcaacaccc	-	*

CY087012	Human H1N1pdm2009 IAVs	Human	H1N1		2009	USA	A/New_York/3565/2009	ggtgatgccccattccttgatcggctccgccgagatca aaagtccttaaaaggaagaggcaacaccc	-	*
CY044052	Human H1N1pdm2009 IAVs	Human	H1N1		2009	USA	A/New_York/3566/2009	ggtgatgccccattccttgatcggctccgccgagatca aaagtccttaaaaggaagaggcaacaccc	-	*
CY044060	Human H1N1pdm2009 IAVs	Human	H1N1	pdm	2009	USA	A/New_York/3567/2009	ggtgatgccccattccttgatcggctccgccgagatca aaagtccttaaaaggaagaggcaacaccc	-	*
CY044068	Human H1N1pdm2009 IAVs	Human	H1N1	pdm	2009	USA	A/New_York/3568/2009	ggtgatgccccattccttgatcggctccgccgagatca aaagtccttaaaaggaagaggcaacaccc	-	*
CY044873	Human H1N1pdm2009 IAVs	Human	H1N1	pdm	2009	USA	A/New_York/3571/2009	ggtgatgccccattccttgatcggctccgccgagatca aaagtccttaaaaggaagaggcaacaccc	-	*
CY044889	Human H1N1pdm2009 IAVs	Human	H1N1	pdm	2009	USA	A/New_York/3580/2009	ggtgatgccccattccttgatcggctccgccgagatca aaagtccttaaaaggaagaggcaacaccc	-	*
CY044897	Human H1N1pdm2009 IAVs	Human	H1N1	pdm	2009	USA	A/New_York/3582/2009	ggtgatgccccattccttgatcggctccgccgagatca aaagtccttaaaaggaagaggcaacaccc	-	*
CY044905	Human H1N1pdm2009 IAVs	Human	H1N1	pdm	2009	USA	A/New_York/3611/2009	ggtgatgccccattccttgatcggctccgccgagatca aaagtccttaaaaggaagaggcaacaccc	-	*
CY044921	Human H1N1pdm2009 IAVs	Human	H1N1	pdm	2009	USA	A/New_York/3613/2009	ggtgatgccccattccttgatcggctccgccgagatca aaagtccttaaaaggaagaggcaacaccc	-	*
CY044929	Human H1N1pdm2009 IAVs	Human	H1N1	pdm	2009	USA	A/New_York/3617/2009	ggtgatgccccattccttgatcggctccgccgagatca aaagtccttaaaaggaagaggcaacaccc	-	*
CY044937	Human H1N1pdm2009 IAVs	Human	H1N1	pdm	2009	USA	A/New_York/3623/2009	ggtgatgccccattccttgatcggctccgccgagatca aaagtccttaaaaggaagaggcaacaccc	-	*
CY044945	Human H1N1pdm2009 IAVs	Human	H1N1	pdm	2009	USA	A/New_York/3625/2009	ggtgatgccccattccttgatcggctccgccgagatca aaagtccttaaaaggaagaggcaacaccc	-	*
CY044953	Human H1N1pdm2009 IAVs	Human	H1N1	pdm	2009	USA	A/New_York/3627/2009	ggtgatgccccattccttgatcggctccgccgagatca aaagtccttaaaaggaagaggcaacaccc	-	*
CY044961	Human H1N1pdm2009 IAVs	Human	H1N1	pdm	2009	USA	A/New_York/3629/2009	ggtgatgccccattccttgatcggctccgccgagatca aaagtccttaaaaggaagaggcaacaccc	-	*
CY043279	Human H1N1pdm2009 IAVs	Human	H1N1	pdm	2009	USA	A/New_York/3651/2009	ggtgatgccccattccttgatcggctccgccgagatca aaagtccttaaaaggaagaggcaacaccc	-	*
CY044084	Human H1N1pdm2009 IAVs	Human	H1N1	pdm	2009	USA	A/New_York/3715/2009	ggtgatgccccattccttgatcggctccgccgagatca aaagtccttaaaaggaagaggcaacaccc	-	*
CY044865	Human H1N1pdm2009 IAVs	Human	H1N1	pdm	2009	USA	A/New_York/3726/2009	ggtgatgccccattccttgatcggctccgccgagatca aaagtccttaaaaggaagaggcaacaccc	-	*
CY044092	Human H1N1pdm2009 IAVs	Human	H1N1		2009	USA	A/New_York/3740/2009	ggtgatgccccattccttgatcggctccgccgagatca aaagtccttaaaaggaagaggcaacaccc	-	*
CY044977	Human H1N1pdm2009 IAVs	Human	H1N1	pdm	2009	USA	A/New_York/3744/2009	ggtgatgccccattccttgatcggctccgccgagatca aaagtccttaaaaggaagaggcaacaccc	-	*
CY044985	Human H1N1pdm2009 IAVs	Human	H1N1	pdm	2009	USA	A/New_York/3747/2009	ggtgatgccccattccttgatcggctccgccgagatca aaagtccttaaaaggaagaggcaacaccc	-	*
CY045025	Human H1N1pdm2009 IAVs	Human	H1N1	pdm	2009	USA	A/New_York/3803/2009	ggtgatgccccattccttgatcggctccgccgagatca aaagtccttaaaaggaagaggcaacaccc	-	*
CY045033	Human H1N1pdm2009 IAVs	Human	H1N1	pdm	2009	USA	A/New_York/3812/2009	ggtgatgccccattccttgatcggctccgccgagatca aaagtccttaaaaggaagaggcaacaccc	-	*
CY045163	Human H1N1pdm2009 IAVs	Human	H1N1	pdm	2009	USA	A/New_York/3818/2009	ggtgatgccccattccttgatcggctccgccgagatca aaagtccttaaaaggaagaggcaacaccc	-	*
CY045171	Human H1N1pdm2009 IAVs	Human	H1N1	pdm	2009	USA	A/New_York/3819/2009	ggtgatgccccattccttgatcggctccgccgagatca aaagtccttaaaaggaagaggcaacaccc	-	*
CY045001	Human H1N1pdm2009 IAVs	Human	H1N1	pdm	2009	USA	A/New_York/3828/2009	ggtgatgccccattccttgatcggctccgccgagatca aaagtccttaaaaggaagaggcaacaccc	-	*

CY044108	Human H1N1pdm2009 IAVs	Human	H1N1	pdm	2009	USA	A/New_York/3898/2009	ggtgatgccccattccttgatcggctccgccgagatca aaagtccttaaaaggaagaggcaacaccc	-	*
CY045187	Human H1N1pdm2009 IAVs	Human	H1N1	pdm	2009	USA	A/New_York/3960/2009	ggtgatgccccattccttgatcggctccgccgagatca aaagtccttaaaaggaagaggcaacaccc	-	*
CY052134	Human H1N1pdm2009 IAVs	Human	H1N1	pdm	2009	USA	A/New_York/3976/2009	ggtgatgccccattccttgatcggctccgccgagatca aaagtccttaaaaggaagaggcaacaccc	-	*
CY052142	Human H1N1pdm2009 IAVs	Human	H1N1		2009	USA	A/New_York/3980/2009	ggtgatgccccattccttgatcggctccgccgagatca aaagtccttaaaaggaagaggcaacaccc	-	*
CY044116	Human H1N1pdm2009 IAVs	Human	H1N1	pdm	2009	USA	A/New_York/3992/2009	ggtgatgccccattccttgatcggctccgccgagatca aaagtccttaaaaggaagaggcaacaccc	-	*
CY045057	Human H1N1pdm2009 IAVs	Human	H1N1	pdm	2009	USA	A/New_York/4052/2009	ggtgatgccccattccttgatcggctccgccgagatca aaagtccttaaaaggaagaggcaacaccc	-	*
CY045219	Human H1N1pdm2009 IAVs	Human	H1N1	pdm	2009	USA	A/New_York/4074/2009	ggtgatgccccattccttgatcggctccgccgagatca aaagtccttaaaaggaagaggcaacaccc	-	*
CY050162	Human H1N1pdm2009 IAVs	Human	H1N1		2009	USA	A/New_York/4095/2009	ggtgatgccccattccttgatcggctccgccgagatca aaagtccttaaaaggaagaggcaacaccc	-	*
CY045065	Human H1N1pdm2009 IAVs	Human	H1N1	pdm	2009	USA	A/New_York/4099/2009	ggtgatgccccattccttgatcggctccgccgagatca aaagtccttaaaaggaagaggcaacaccc	-	*
CY051531	Human H1N1pdm2009 IAVs	Human	H1N1	pdm	2009	USA	A/New_York/4400/2009	ggtgatgccccattccttgatcggctccgccgagatca aaagtccttaaaaggaagaggcaacaccc	-	*
CY052158	Human H1N1pdm2009 IAVs	Human	H1N1	pdm	2009	USA	A/New_York/4401/2009	ggtgatgccccattccttgatcggctccgccgagatca aaagtccttaaaaggaagaggcaacaccc	-	*
CY051539	Human H1N1pdm2009 IAVs	Human	H1N1	pdm	2009	USA	A/New_York/4402/2009	ggtgatgccccattccttgatcggctccgccgagatca aaagtccttaaaaggaagaggcaacaccc	-	*
CY050186	Human H1N1pdm2009 IAVs	Human	H1N1	pdm	2009	USA	A/New_York/4438/2009	ggtgatgccccattccttgatcggctccgccgagatca aaagtccttaaaaggaagaggcaacaccc	-	*
CY051587	Human H1N1pdm2009 IAVs	Human	H1N1	pdm	2009	USA	A/New_York/4556/2009	ggtgatgccccattccttgatcggctccgccgagatca aaagtccttaaaaggaagaggcaacaccc	-	*
CY051619	Human H1N1pdm2009 IAVs	Human	H1N1	pdm	2009	USA	A/New_York/4568/2009	ggtgatgccccattccttgatcggctccgccgagatca aaagtccttaaaaggaagaggcaacaccc	-	*
CY050194	Human H1N1pdm2009 IAVs	Human	H1N1	pdm	2009	USA	A/New_York/4576/2009	ggtgatgccccattccttgatcggctccgccgagatca aaagtccttaaaaggaagaggcaacaccc	-	*
CY051643	Human H1N1pdm2009 IAVs	Human	H1N1	pdm	2009	USA	A/New_York/4677/2009	ggtgatgccccattccttgatcggctccgccgagatca aaagtccttaaaaggaagaggcaacaccc	-	*
CY051675	Human H1N1pdm2009 IAVs	Human	H1N1	pdm	2009	USA	A/New_York/4738/2009	ggtgatgccccattccttgatcggctccgccgagatca aaagtccttaaaaggaagaggcaacaccc	-	*
CY051683	Human H1N1pdm2009 IAVs	Human	H1N1	pdm	2009	USA	A/New_York/4747/2009	ggtgatgccccattccttgatcggctccgccgagatca aaagtccttaaaaggaagaggcaacaccc	-	*
CY051691	Human H1N1pdm2009 IAVs	Human	H1N1	pdm	2009	USA	A/New_York/4761/2009	ggtgatgccccattccttgatcggctccgccgagatca aaagtccttaaaaggaagaggcaacaccc	-	*
CY087020	Human H1N1pdm2009 IAVs	Human	H1N1	pdm	2009	USA	A/New_York/4764/2009	ggtgatgccccattccttgatcggctccgccgagatca aaagtccttaaaaggaagaggcaacaccc	-	*
CY056431	Human H1N1pdm2009 IAVs	Human	H1N1	pdm	2009	USA	A/New_York/4981/2009	ggtgatgccccattccttgatcggctccgccgagatca aaagtccttaaaaggaagaggcaacaccc	-	*
CY056439	Human H1N1pdm2009 IAVs	Human	H1N1	pdm	2009	USA	A/New_York/4984/2009	ggtgatgccccattccttgatcggctccgccgagatca aaagtccttaaaaggaagaggcaacaccc	-	*
CY056447	Human H1N1pdm2009 IAVs	Human	H1N1	pdm	2009	USA	A/New_York/4985/2009	ggtgatgccccattccttgatcggctccgccgagatca aaagtccttaaaaggaagaggcaacaccc	-	*
CY056495	Human H1N1pdm2009 IAVs	Human	H1N1	pdm	2009	USA	A/New_York/5074/2009	ggtgatgccccattccttgatcggctccgccgagatca aaagtccttaaaaggaagaggcaacaccc	-	*

CY056543	Human H1N1pdm2009 IAVs	Human	H1N1	pdm	2009	USA	A/New_York/5150/2009	ggtgatgccccattccttgatcggctccgccgagatca aaagtccttaaaaggaagaggcaacaccc	-	*
CY057250	Human H1N1pdm2009 IAVs	Human	H1N1	pdm	2009	USA	A/New_York/5161/2009	ggtgatgccccattccttgatcggctccgccgagatca aaagtccttaaaaggaagaggcaacaccc	-	*
CY057298	Human H1N1pdm2009 IAVs	Human	H1N1	pdm	2009	USA	A/New_York/5276/2009	ggtgatgccccattccttgatcggctccgccgagatca aaagtccttaaaaggaagaggcaacaccc	-	*
CY057322	Human H1N1pdm2009 IAVs	Human	H1N1		2009	USA	A/New_York/5667/2009	ggtgatgccccattccttgatcggctccgccgagatca aaagtccttaaaaggaagaggcaacaccc	-	*
CY056575	Human H1N1pdm2009 IAVs	Human	H1N1	pdm	2009	USA	A/New_York/5755/2009	ggtgatgccccattccttgatcggctccgccgagatca aaagtccttaaaaggaagaggcaacaccc	-	*
CY057330	Human H1N1pdm2009 IAVs	Human	H1N1	pdm	2009	USA	A/New_York/6072/2009	ggtgatgccccattccttgatcggctccgccgagatca aaagtccttaaaaggaagaggcaacaccc	-	*
CY056703	Human H1N1pdm2009 IAVs	Human	H1N1	pdm	2009	USA	A/New_York/6293/2009	ggtgatgccccattccttgatcggctccgccgagatca aaagtccttaaaaggaagaggcaacaccc	-	*
CY056711	Human H1N1pdm2009 IAVs	Human	H1N1	pdm	2009	USA	A/New_York/6300/2009	ggtgatgccccattccttgatcggctccgccgagatca aaagtccttaaaaggaagaggcaacaccc	-	*
CY056719	Human H1N1pdm2009 IAVs	Human	H1N1	pdm	2009	USA	A/New_York/6346/2009	ggtgatgccccattccttgatcggctccgccgagatca aaagtccttaaaaggaagaggcaacaccc	-	*
CY084466	Human H1N1pdm2009 IAVs	Human	H1N1	pdm	2009	USA	A/New_York/6537/2009	ggtgatgccccattccttgatcggctccgccgagatca aaagtccttaaaaggaagaggcaacaccc	-	*
CY056767	Human H1N1pdm2009 IAVs	Human	H1N1	pdm	2009	USA	A/New_York/6668/2009	ggtgatgccccattccttgatcggctccgccgagatca aaagtccttaaaaggaagaggcaacaccc	-	*
CY061990	Human H1N1pdm2009 IAVs	Human	H1N1	pdm	2009	USA	A/New_York/7019/2009	ggtgatgccccattccttgatcggctccgccgagatca aaagtccttaaaaggaagaggcaacaccc	-	*
CY062006	Human H1N1pdm2009 IAVs	Human	H1N1	pdm	2009	USA	A/New_York/7036/2009	ggtgatgccccattccttgatcggctccgccgagatca aaagtccttaaaaggaagaggcaacaccc	-	*
CY062014	Human H1N1pdm2009 IAVs	Human	H1N1	pdm	2009	USA	A/New_York/7107/2009	ggtgatgccccattccttgatcggctccgccgagatca aaagtccttaaaaggaagaggcaacaccc	-	*
CY065095	Human H1N1pdm2009 IAVs	Human	H1N1	pdm	2009	USA	A/New_York/7425/2009	ggtgatgccccattccttgatcggctccgccgagatca aaagtccttaaaaggaagaggcaacaccc	-	*
CY065103	Human H1N1pdm2009 IAVs	Human	H1N1	pdm	2009	USA	A/New_York/7426/2009	ggtgatgccccattccttgatcggctccgccgagatca aaagtccttaaaaggaagaggcaacaccc	-	*
CY063634	Human H1N1pdm2009 IAVs	Human	H1N1	pdm	2009	USA	A/New_York/INS151/2009	ggtgatgccccattccttgatcggctccgccgagatca aaagtccttaaaaggaagaggcaacaccc	-	*
CY071035	Human H1N1pdm2009 IAVs	Human	H1N1	pdm	2009	USA	A/New_York/NHRC0002/2009	ggtgatgccccattccttgatcggctccgccgagatca aaagtccttaaaaggaagaggcaacaccc	-	*
CY071051	Human H1N1pdm2009 IAVs	Human	H1N1	pdm	2009	USA	A/New_York/NHRC0004/2009	ggtgatgccccattccttgatcggctccgccgagatca aaagtccttaaaaggaagaggcaacaccc	-	*
CY072330	Human H1N1pdm2009 IAVs	Human	H1N1	pdm	2009	USA	A/Newark/INS318/2009	ggtgatgccccattccttgatcggctccgccgagatca aaagtccttaaaaggaagaggcaacaccc	-	*
CY062975	Human H1N1pdm2009 IAVs	Human	H1N1	pdm	2009	Denmark	A/Odense/INS177/2009	ggtgatgccccattccttgatcggctccgccgagatca aaagtccttaaaaggaagaggcaacaccc	-	*
FJ984399	Human H1N1pdm2009 IAVs	Human	H1N1	pdm	2009	USA	A/Ohio/07/2009	ggtgatgccccattccttgatcggctccgccgagatca aaagtccttaaaaggaagaggcaacaccc	-	*
CY061287	Human H1N1pdm2009 IAVs	Human	H1N1	pdm	2009	USA	A/Pensacola/INS108/2009	ggtgatgccccattccttgatcggctccgccgagatca aaagtccttaaaaggaagaggcaacaccc	-	*
CY062671	Human H1N1pdm2009 IAVs	Human	H1N1	pdm	2009	USA	A/Pensacola/INS109/2009	ggtgatgccccattccttgatcggctccgccgagatca aaagtccttaaaaggaagaggcaacaccc	-	*
CY066763	Human H1N1pdm2009 IAVs	Human	H1N1	pdm	2009	USA	A/Pensacola/INS211/2009	ggtgatgccccattccttgatcggctccgccgagatca aaagtccttaaaaggaagaggcaacaccc	-	*

CY066915	Human H1N1pdm2009 IAVs	Human	H1N1	pdm	2009	USA	A/Pensacola/INS233/2009	ggtgatgccccattccttgatcggctccgccgagatca<br>aaagtccttaaaaggaagaggcaacaccc	-	*
CY056160	Human H1N1pdm2009 IAVs	Human	H1N1	pdm	2009	USA	A/Pensacola/INS38/2009	ggtgatgccccattccttgatcggctccgccgagatca<br>aaagtccttaaaaggaagaggcaacaccc	-	*
CY056168	Human H1N1pdm2009 IAVs	Human	H1N1	pdm	2009	USA	A/Pensacola/INS40/2009	ggtgatgccccattccttgatcggctccgccgagatca<br>aaagtccttaaaaggaagaggcaacaccc	-	*
CY083124	Human H1N1pdm2009 IAVs	Human	H1N1	pdm	2009	Ecuador	A/Quito/WRAIR0617T/2009	ggtgatgccccattccttgatcggctccgccgagatca<br>aaagtccttaaaaggaagaggcaacaccc	-	*
CY055952	Human H1N1pdm2009 IAVs	Human	H1N1		2009	USA	A/San_Diego/INS01/2009	ggtgatgccccattccttgatcggctccgccgagatca<br>aaagtccttaaaaggaagaggcaacaccc	-	*
CY055976	Human H1N1pdm2009 IAVs	Human	H1N1	pdm	2009	USA	A/San_Diego/INS04/2009	ggtgatgccccattccttgatcggctccgccgagatca<br>aaagtccttaaaaggaagaggcaacaccc	-	*
CY055984	Human H1N1pdm2009 IAVs	Human	H1N1	pdm	2009	USA	A/San_Diego/INS05/2009	ggtgatgccccattccttgatcggctccgccgagatca<br>aaagtccttaaaaggaagaggcaacaccc	-	*
CY061239	Human H1N1pdm2009 IAVs	Human	H1N1	pdm	2009	USA	A/San_Diego/INS102/2009	ggtgatgccccattccttgatcggctccgccgagatca<br>aaagtccttaaaaggaagaggcaacaccc	-	*
CY061247	Human H1N1pdm2009 IAVs	Human	H1N1	pdm	2009	USA	A/San_Diego/INS103/2009	ggtgatgccccattccttgatcggctccgccgagatca<br>aaagtccttaaaaggaagaggcaacaccc	-	*
CY066539	Human H1N1pdm2009 IAVs	Human	H1N1	pdm	2009	USA	A/San_Diego/INS195/2009	ggtgatgccccattccttgatcggctccgccgagatca<br>aaagtccttaaaaggaagaggcaacaccc	-	*
CY066571	Human H1N1pdm2009 IAVs	Human	H1N1	pdm	2009	USA	A/San_Diego/INS201/2009	ggtgatgccccattccttgatcggctccgccgagatca<br>aaagtccttaaaaggaagaggcaacaccc	-	*
CY056136	Human H1N1pdm2009 IAVs	Human	H1N1	pdm	2009	USA	A/San_Diego/INS34/2009	ggtgatgccccattccttgatcggctccgccgagatca<br>aaagtccttaaaaggaagaggcaacaccc	-	*
CY083665	Human H1N1pdm2009 IAVs	Human	H1N1	pdm	2009	USA	A/San_Diego/INS41/2009	ggtgatgccccattccttgatcggctccgccgagatca<br>aaagtccttaaaaggaagaggcaacaccc	-	*
CY056176	Human H1N1pdm2009 IAVs	Human	H1N1	pdm	2009	USA	A/San_Diego/INS42/2009	ggtgatgccccattccttgatcggctccgccgagatca<br>aaagtccttaaaaggaagaggcaacaccc	-	*
CY056200	Human H1N1pdm2009 IAVs	Human	H1N1	pdm	2009	USA	A/San_Diego/INS48/2009	ggtgatgccccattccttgatcggctccgccgagatca<br>aaagtccttaaaaggaagaggcaacaccc	-	*
CY056879	Human H1N1pdm2009 IAVs	Human	H1N1	pdm	2009	USA	A/San_Diego/INS51/2009	ggtgatgccccattccttgatcggctccgccgagatca<br>aaagtccttaaaaggaagaggcaacaccc	-	*
CY056208	Human H1N1pdm2009 IAVs	Human	H1N1	pdm	2009	USA	A/San_Diego/INS52/2009	ggtgatgccccattccttgatcggctccgccgagatca<br>aaagtccttaaaaggaagaggcaacaccc	-	*
CY056216	Human H1N1pdm2009 IAVs	Human	H1N1	pdm	2009	USA	A/San_Diego/INS53/2009	ggtgatgccccattccttgatcggctccgccgagatca<br>aaagtccttaaaaggaagaggcaacaccc	-	*
GU433037	Human H1N1pdm2009 IAVs	Human	H1N1	pdm	2009	Russia	A/Novosibirsk/02/2009	ggtgatgccccattccttgatcggctccgccgagatca<br>aaagtccttaaaaggaagaggcaacaccc	-	*
CY083834	Human H1N1pdm2009 IAVs	Human	H1N1	pdm	2009	USA	A/San_Diego/INS07/2009	ggtgatgccccattccttgatcggctccgccgagatca<br>aaagtccttaaaaggaagaggcaacaccc	-	*
CY083874	Human H1N1pdm2009 IAVs	Human	H1N1	pdm	2009	USA	A/San_Diego/INS49/2009	ggtgatgccccattccttgatcggctccgccgagatca<br>aaagtccttaaaaggaagaggcaacaccc	-	*
CY056895	Human H1N1pdm2009 IAVs	Human	H1N1	pdm	2009	USA	A/San_Diego/INS55/2009	ggtgatgccccattccttgatcggctccgccgagatca<br>aaagtccttaaaaggaagaggcaacaccc	-	*
CY056903	Human H1N1pdm2009 IAVs	Human	H1N1	pdm	2009	USA	A/San_Diego/INS57/2009	ggtgatgccccattccttgatcggctccgccgagatca<br>aaagtccttaaaaggaagaggcaacaccc	-	*
CY056911	Human H1N1pdm2009 IAVs	Human	H1N1	pdm	2009	USA	A/San_Diego/INS60/2009	ggtgatgccccattccttgatcggctccgccgagatca<br>aaagtccttaaaaggaagaggcaacaccc	-	*
CY056927	Human H1N1pdm2009 IAVs	Human	H1N1	pdm	2009	USA	A/San_Diego/INS63/2009	ggtgatgccccattccttgatcggctccgccgagatca<br>aaagtccttaaaaggaagaggcaacaccc	-	*

CY056935	Human H1N1pdm2009 IAVs	Human	H1N1	pdm	2009	USA	A/San_Diego/INS65/2009	ggtgatgccccattccttgatcggctccgccgagatca aaagtccttaaaaggaagaggcaacaccc	-	*
CY056240	Human H1N1pdm2009 IAVs	Human	H1N1	pdm	2009	USA	A/San_Diego/INS70/2009	ggtgatgccccattccttgatcggctccgccgagatca aaagtccttaaaaggaagaggcaacaccc	-	*
CY062655	Human H1N1pdm2009 IAVs	Human	H1N1	pdm	2009	USA	A/San_Diego/INS74/2009	ggtgatgccccattccttgatcggctccgccgagatca aaagtccttaaaaggaagaggcaacaccc	-	*
CY057346	Human H1N1pdm2009 IAVs	Human	H1N1	pdm	2009	USA	A/San_Diego/INS75/2009	ggtgatgccccattccttgatcggctccgccgagatca aaagtccttaaaaggaagaggcaacaccc	-	*
KF612172	Human H1N1pdm2009 IAVs	Human	H1N1		2009	Bolivia	A/Santa_Cruz/12541/2009	ggtgatgccccattccttgatcggctccgccgagatca aaagtccttaaaaggaagaggcaacaccc	-	*
KF612180	Human H1N1pdm2009 IAVs	Human	H1N1	pdm	2009	Bolivia	A/Santa_Cruz/97831/2009	ggtgatgccccattccttgatcggctccgccgagatca aaagtccttaaaaggaagaggcaacaccc	-	*
CY107823	Human H1N1pdm2009 IAVs	Human	H1N1	pdm	2009	United_Kingdom	A/Scotland/StAndrews_17693/2009	ggtgatgccccattccttgatcggctccgccgagatca aaagtccttaaaaggaagaggcaacaccc	-	*
GQ253502	Human H1N1pdm2009 IAVs	Human	H1N1		2009	China	A/Shanghai/71T/2009	ggtgatgccccattccttgatcggctccgccgagatca aaagtccttaaaaggaagaggcaacaccc	-	*
AB539743	Human H1N1pdm2009 IAVs	Human	H1N1	pdm	2009	China	A/Shanghai/P1/2009	ggtgatgccccattccttgatcggctccgccgagatca aaagtccttaaaaggaagaggcaacaccc	-	*
CY049104	Human H1N1pdm2009 IAVs	Human	H1N1	pdm	2009	Singapore	A/Singapore/ON511/2009	ggtgatgccccattccttgatcggctccgccgagatca aaagtccttaaaaggaagaggcaacaccc	-	*
CY097888	Human H1N1pdm2009 IAVs	Human	H1N1	pdm	2009	USA	A/South_Carolina/WRAIR1645P/2009	ggtgatgccccattccttgatcggctccgccgagatca aaagtccttaaaaggaagaggcaacaccc	-	*
CY083284	Human H1N1pdm2009 IAVs	Human	H1N1	pdm	2009	USA	A/South_Carolina/WRAIR1645P/2009	ggtgatgccccattccttgatcggctccgccgagatca aaagtccttaaaaggaagaggcaacaccc	-	*
GQ359768	Human H1N1pdm2009 IAVs	Human	H1N1	pdm	2009	Sweden	A/Stockholm/31/2009	ggtgatgccccattccttgatcggctccgccgagatca aaagtccttaaaaggaagaggcaacaccc	-	*
GQ360065	Human H1N1pdm2009 IAVs	Human	H1N1	pdm	2009	Sweden	A/Stockholm/32/2009	ggtgatgccccattccttgatcggctccgccgagatca aaagtccttaaaaggaagaggcaacaccc	-	*
GQ365686	Human H1N1pdm2009 IAVs	Human	H1N1	pdm	2009	Sweden	A/Stockholm/44/2009	ggtgatgccccattccttgatcggctccgccgagatca aaagtccttaaaaggaagaggcaacaccc	-	*
GQ329109	Human H1N1pdm2009 IAVs	Human	H1N1	pdm	2009	France	A/Strasbourg/2609/2009	ggtgatgccccattccttgatcggctccgccgagatca aaagtccttaaaaggaagaggcaacaccc	-	*
GQ329091	Human H1N1pdm2009 IAVs	Human	H1N1	pdm	2009	France	A/Strasbourg/2611/2009	ggtgatgccccattccttgatcggctccgccgagatca aaagtccttaaaaggaagaggcaacaccc	-	*
CY075860	Human H1N1pdm2009 IAVs	Swine	H1N1		2009	Argentina	A/swine/Argentina/CIP051_BsAs76/2009	ggtgatgccccattccttgatcggctccgccgagatca aaagtccttaaaaggaagaggcaacaccc	-	*
CY061942	Human H1N1pdm2009 IAVs	Human	H1N1		2009	USA	A/New_York/6941/2009	ggtgatgccccattccttgatcggctccgccgagatca aaagtccttaaaaggaagaggcaacaccc	-	*
CY047330	Human H1N1pdm2009 IAVs	Human	H1N1	pdm	2009	USA	A/New_York/3167/2009	ggtgatgccccattccttgatcggctccgccgagatca aaagtccttaaaaggaagaggcaacaccc	-	*
CY066923	Human H1N1pdm2009 IAVs	Human	H1N1	pdm	2009	USA	A/Pensacola/INS234/2009	ggtgatgccccattccttgatcggctccgccgagatca aaagtccttaaaaggaagaggcaacaccc	-	*
CY066931	Human H1N1pdm2009 IAVs	Human	H1N1	pdm	2009	USA	A/Pensacola/INS235/2009	ggtgatgccccattccttgatcggctccgccgagatca aaagtccttaaaaggaagaggcaacaccc	-	*
CY057338	Human H1N1pdm2009 IAVs	Human	H1N1	pdm	2009	USA	A/San_Diego/INS08/2009	ggtgatgccccattccttgatcggctccgccgagatca aaagtccttaaaaggaagaggcaacaccc	-	*
GQ360063	Human H1N1pdm2009 IAVs	Human	H1N1	pdm	2009	Sweden	A/Stockholm/33/2009	ggtgatgccccattccttgatcggctccgccgagatca aaagtccttaaaaggaagaggcaacaccc	-	*
GQ365681	Human H1N1pdm2009 IAVs	Human	H1N1	pdm	2009	Sweden	A/Stockholm/38/2009	ggtgatgccccattccttgatcggctccgccgagatca aaagtccttaaaaggaagaggcaacaccc	-	*

GQ369280	Human H1N1pdm2009 IAVs	Human	H1N1	pdm	2009	Sweden	A/Stockholm/41/2009	ggtgatgccccattccttgatcggctccgccgagatca aaagtccttaaaaggaagaggcaacaccc	-	*
GQ365369	Human H1N1pdm2009 IAVs	Human	H1N1	pdm	2009	Sweden	A/Stockholm/35/2009	ggtgatgccccattccttgatcggctccgccgagatca aaagtccttaaaaggaagaggcaacaccc	-	*
CY053623	Human H1N1pdm2009 IAVs	Swine	H1N1		2009	Italy	A/swine/Italy/290271/2009	ggtgatgccccattccttgatcggctccgccgagatca aaagtccttaaaaggaagaggcaacaccc	-	*
JF951853	Human H1N1pdm2009 IAVs	Swine	H1N1		2009	China	A/swine/Shandong/731/2009	ggtgatgccccattccttgatcggctccgccgagatca aaagtccttaaaaggaagaggcaacaccc	-	*
JQ695891	Human H1N1pdm2009 IAVs	Swine	H1N1		2009	China	A/swine/Shandong/811/2009	ggtgatgccccattccttgatcggctccgccgagatca aaagtccttaaaaggaagaggcaacaccc	-	*
JQ695899	Human H1N1pdm2009 IAVs	Swine	H1N1		2009	China	A/swine/Shandong/854/2009	ggtgatgccccattccttgatcggctccgccgagatca aaagtccttaaaaggaagaggcaacaccc	-	*
JN187335	Human H1N1pdm2009 IAVs	Human	H1N1	pdm	2009	Taiwan	A/Taiwan/7418/2009	ggtgatgccccattccttgatcggctccgccgagatca aaagtccttaaaaggaagaggcaacaccc	-	*
JN187334	Human H1N1pdm2009 IAVs	Human	H1N1	pdm	2009	Taiwan	A/Taiwan/8542/2009	ggtgatgccccattccttgatcggctccgccgagatca aaagtccttaaaaggaagaggcaacaccc	-	*
JN187331	Human H1N1pdm2009 IAVs	Human	H1N1	pdm	2009	Taiwan	A/Taiwan/940/2009	ggtgatgccccattccttgatcggctccgccgagatca aaagtccttaaaaggaagaggcaacaccc	-	*
CY062783	Human H1N1pdm2009 IAVs	Human	H1N1	pdm	2009	Belgium	A/Tessenderlo/INS138/2009	ggtgatgccccattccttgatcggctccgccgagatca aaagtccttaaaaggaagaggcaacaccc	-	*
CY066675	Human H1N1pdm2009 IAVs	Human	H1N1	pdm	2009	Belgium	A/Tessenderlo/INS287/2009	ggtgatgccccattccttgatcggctccgccgagatca aaagtccttaaaaggaagaggcaacaccc	-	*
CY052270	Human H1N1pdm2009 IAVs	Human	H1N1	pdm	2009	USA	A/Texas/42102708/2009	ggtgatgccccattccttgatcggctccgccgagatca aaagtccttaaaaggaagaggcaacaccc	-	*
CY051827	Human H1N1pdm2009 IAVs	Human	H1N1	pdm	2009	USA	A/Texas/43242018/2009	ggtgatgccccattccttgatcggctccgccgagatca aaagtccttaaaaggaagaggcaacaccc	-	*
CY052619	Human H1N1pdm2009 IAVs	Human	H1N1	pdm	2009	USA	A/Texas/44302533/2009	ggtgatgccccattccttgatcggctccgccgagatca aaagtccttaaaaggaagaggcaacaccc	-	*
CY052342	Human H1N1pdm2009 IAVs	Human	H1N1		2009	USA	A/Texas/44312415/2009	ggtgatgccccattccttgatcggctccgccgagatca aaagtccttaaaaggaagaggcaacaccc	-	*
CY052683	Human H1N1pdm2009 IAVs	Human	H1N1	pdm	2009	USA	A/Texas/45010998/2009	ggtgatgccccattccttgatcggctccgccgagatca aaagtccttaaaaggaagaggcaacaccc	-	*
CY052595	Human H1N1pdm2009 IAVs	Human	H1N1	pdm	2009	USA	A/Texas/45033774/2009	ggtgatgccccattccttgatcggctccgccgagatca aaagtccttaaaaggaagaggcaacaccc	-	*
CY052923	Human H1N1pdm2009 IAVs	Human	H1N1	pdm	2009	USA	A/Texas/45052569/2009	ggtgatgccccattccttgatcggctccgccgagatca aaagtccttaaaaggaagaggcaacaccc	-	*
CY051899	Human H1N1pdm2009 IAVs	Human	H1N1	pdm	2009	USA	A/Texas/45062633/2009	ggtgatgccccattccttgatcggctccgccgagatca aaagtccttaaaaggaagaggcaacaccc	-	*
CY083694	Human H1N1pdm2009 IAVs	Human	H1N1	pdm	2009	Spain	A/Terrassa/INS94/2009	ggtgatgccccattccttgatcggctccgccgagatca aaagtccttaaaaggaagaggcaacaccc	-	*
CY052811	Human H1N1pdm2009 IAVs	Human	H1N1	pdm	2009	USA	A/Texas/45071344/2009	ggtgatgccccattccttgatcggctccgccgagatca aaagtccttaaaaggaagaggcaacaccc	-	*
CY052827	Human H1N1pdm2009 IAVs	Human	H1N1	pdm	2009	USA	A/Texas/45072128/2009	ggtgatgccccattccttgatcggctccgccgagatca aaagtccttaaaaggaagaggcaacaccc	-	*
CY052699	Human H1N1pdm2009 IAVs	Human	H1N1	pdm	2009	USA	A/Texas/45083819/2009	ggtgatgccccattccttgatcggctccgccgagatca aaagtccttaaaaggaagaggcaacaccc	-	*
CY069638	Human H1N1pdm2009 IAVs	Human	H1N1	pdm	2009	Singapore	A/Singapore/478/2009	ggtgatgccccattccttgatcggctccgccgagatca aaagtccttaaaaggaagaggcaacaccc	-	*
CY052667	Human H1N1pdm2009 IAVs	Human	H1N1	pdm	2009	USA	A/Texas/45093670/2009	ggtgatgccccattccttgatcggctccgccgagatca aaagtccttaaaaggaagaggcaacaccc	-	*

CY052747	Human H1N1pdm2009 IAVs	Human	H1N1	pdm	2009	USA	A/Texas/45101424/2009	ggtgatgccccattccttgatcggctccgccgagatca aaagtccttaaaaggaagaggcaacaccc	-	*
CY052651	Human H1N1pdm2009 IAVs	Human	H1N1	pdm	2009	USA	A/Texas/45103259/2009	ggtgatgccccattccttgatcggctccgccgagatca aaagtccttaaaaggaagaggcaacaccc	-	*
CY052515	Human H1N1pdm2009 IAVs	Human	H1N1	pdm	2009	USA	A/Texas/45103737/2009	ggtgatgccccattccttgatcggctccgccgagatca aaagtccttaaaaggaagaggcaacaccc	-	*
CY052507	Human H1N1pdm2009 IAVs	Human	H1N1	pdm	2009	USA	A/Texas/45104026/2009	ggtgatgccccattccttgatcggctccgccgagatca aaagtccttaaaaggaagaggcaacaccc	-	*
CY052555	Human H1N1pdm2009 IAVs	Human	H1N1	pdm	2009	USA	A/Texas/45122774/2009	ggtgatgccccattccttgatcggctccgccgagatca aaagtccttaaaaggaagaggcaacaccc	-	*
CY052947	Human H1N1pdm2009 IAVs	Human	H1N1	pdm	2009	USA	A/Texas/45122886/2009	ggtgatgccccattccttgatcggctccgccgagatca aaagtccttaaaaggaagaggcaacaccc	-	*
CY052547	Human H1N1pdm2009 IAVs	Human	H1N1	pdm	2009	USA	A/Texas/45132214/2009	ggtgatgccccattccttgatcggctccgccgagatca aaagtccttaaaaggaagaggcaacaccc	-	*
CY058050	Human H1N1pdm2009 IAVs	Human	H1N1	pdm	2009	USA	A/Texas/46172734/2009	ggtgatgccccattccttgatcggctccgccgagatca aaagtccttaaaaggaagaggcaacaccc	-	*
CY060759	Human H1N1pdm2009 IAVs	Human	H1N1	pdm	2009	USA	A/Texas/46193632/2009	ggtgatgccccattccttgatcggctccgccgagatca aaagtccttaaaaggaagaggcaacaccc	-	*
CY060799	Human H1N1pdm2009 IAVs	Human	H1N1	pdm	2009	USA	A/Texas/46223444/2009	ggtgatgccccattccttgatcggctccgccgagatca aaagtccttaaaaggaagaggcaacaccc	-	*
CY060807	Human H1N1pdm2009 IAVs	Human	H1N1	pdm	2009	USA	A/Texas/46223582/2009	ggtgatgccccattccttgatcggctccgccgagatca aaagtccttaaaaggaagaggcaacaccc	-	*
CY060823	Human H1N1pdm2009 IAVs	Human	H1N1	pdm	2009	USA	A/Texas/46233104/2009	ggtgatgccccattccttgatcggctccgccgagatca aaagtccttaaaaggaagaggcaacaccc	-	*
CY060879	Human H1N1pdm2009 IAVs	Human	H1N1	pdm	2009	USA	A/Texas/JMS362/2009	ggtgatgccccattccttgatcggctccgccgagatca aaagtccttaaaaggaagaggcaacaccc	-	*
CY060935	Human H1N1pdm2009 IAVs	Human	H1N1	pdm	2009	USA	A/Texas/JMS372/2009	ggtgatgccccattccttgatcggctccgccgagatca aaagtccttaaaaggaagaggcaacaccc	-	*
CY061031	Human H1N1pdm2009 IAVs	Human	H1N1	pdm	2009	USA	A/Texas/JMS389/2009	ggtgatgccccattccttgatcggctccgccgagatca aaagtccttaaaaggaagaggcaacaccc	-	*
CY074978	Human H1N1pdm2009 IAVs	Human	H1N1	pdm	2009	Thailand	A/Thailand/CU_C161/2009	ggtgatgccccattccttgatcggctccgccgagatca aaagtccttaaaaggaagaggcaacaccc	-	*
CY074994	Human H1N1pdm2009 IAVs	Human	H1N1	pdm	2009	Thailand	A/Thailand/CU_H567/2009	ggtgatgccccattccttgatcggctccgccgagatca aaagtccttaaaaggaagaggcaacaccc	-	*
CY075002	Human H1N1pdm2009 IAVs	Human	H1N1		2009	Thailand	A/Thailand/CU_H572/2009	ggtgatgccccattccttgatcggctccgccgagatca aaagtccttaaaaggaagaggcaacaccc	-	*
CY075010	Human H1N1pdm2009 IAVs	Human	H1N1	pdm	2009	Thailand	A/Thailand/CU_H847/2009	ggtgatgccccattccttgatcggctccgccgagatca aaagtccttaaaaggaagaggcaacaccc	-	*
AB704474	Human H1N1pdm2009 IAVs	Human	H1N1	pdm	2009	Japan	A/Tochigi/445/2009	ggtgatgccccattccttgatcggctccgccgagatca aaagtccttaaaaggaagaggcaacaccc	-	*
GQ396565	Human H1N1pdm2009 IAVs	Human	H1N1	pdm	2009	Spain	A/Valencia/GP272/2009	ggtgatgccccattccttgatcggctccgccgagatca aaagtccttaaaaggaagaggcaacaccc	-	*
CY128073	Human H1N1pdm2009 IAVs	Human	N1	pdm	2009	Viet_Nam	A/Viet_Nam/001_1016/2009	ggtgatgccccattccttgatcggctccgccgagatca aaagtccttaaaaggaagaggcaacaccc	-	*
CY128067	Human H1N1pdm2009 IAVs	Human	H1N1	pdm	2009	Viet_Nam	A/Viet_Nam/001_1890/2009	ggtgatgccccattccttgatcggctccgccgagatca aaagtccttaaaaggaagaggcaacaccc	-	*
CY128295	Human H1N1pdm2009 IAVs	Human	H1N1	pdm	2009	Viet_Nam	A/Viet_Nam/13032074/2009	ggtgatgccccattccttgatcggctccgccgagatca aaagtccttaaaaggaagaggcaacaccc	-	*
CY128447	Human H1N1pdm2009 IAVs	Human	H1N1	pdm	2009	Viet_Nam	A/Viet_Nam/001_1716/2009	ggtgatgccccattccttgatcggctccgccgagatca aaagtccttaaaaggaagaggcaacaccc	-	*

CY128455	Human H1N1pdm2009 IAVs	Human	H1N1	pdm	2009	Viet_Nam	A/Viet_Nam/001_1880/2009	ggtgatgccccattccttgatcggctccgccgagatca aaagtccttaaaaggaagaggcaacaccc	-	*
CY128463	Human H1N1pdm2009 IAVs	Human	H1N1	pdm	2009	Viet_Nam	A/Viet_Nam/001_1889/2009	ggtgatgccccattccttgatcggctccgccgagatca aaagtccttaaaaggaagaggcaacaccc	-	*
CY128375	Human H1N1pdm2009 IAVs	Human	H1N1	pdm	2009	Viet_Nam	A/Viet_Nam/11032001/2009	ggtgatgccccattccttgatcggctccgccgagatca aaagtccttaaaaggaagaggcaacaccc	-	*
CY128383	Human H1N1pdm2009 IAVs	Human	H1N1	pdm	2009	Viet_Nam	A/Viet_Nam/11032006/2009	ggtgatgccccattccttgatcggctccgccgagatca aaagtccttaaaaggaagaggcaacaccc	-	*
CY128135	Human H1N1pdm2009 IAVs	Human	H1N1	pdm	2009	Viet_Nam	A/Viet_Nam/13032001/2009	ggtgatgccccattccttgatcggctccgccgagatca aaagtccttaaaaggaagaggcaacaccc	-	*
CY128143	Human H1N1pdm2009 IAVs	Human	H1N1	pdm	2009	Viet_Nam	A/Viet_Nam/13032002/2009	ggtgatgccccattccttgatcggctccgccgagatca aaagtccttaaaaggaagaggcaacaccc	-	*
CY128151	Human H1N1pdm2009 IAVs	Human	H1N1	pdm	2009	Viet_Nam	A/Viet_Nam/13032008/2009	ggtgatgccccattccttgatcggctccgccgagatca aaagtccttaaaaggaagaggcaacaccc	-	*
CY128159	Human H1N1pdm2009 IAVs	Human	H1N1	pdm	2009	Viet_Nam	A/Viet_Nam/13032009/2009	ggtgatgccccattccttgatcggctccgccgagatca aaagtccttaaaaggaagaggcaacaccc	-	*
CY128175	Human H1N1pdm2009 IAVs	Human	H1N1	pdm	2009	Viet_Nam	A/Viet_Nam/13032015/2009	ggtgatgccccattccttgatcggctccgccgagatca aaagtccttaaaaggaagaggcaacaccc	-	*
CY128191	Human H1N1pdm2009 IAVs	Human	H1N1	pdm	2009	Viet_Nam	A/Viet_Nam/13032019/2009	ggtgatgccccattccttgatcggctccgccgagatca aaagtccttaaaaggaagaggcaacaccc	-	*
CY128199	Human H1N1pdm2009 IAVs	Human	H1N1	pdm	2009	Viet_Nam	A/Viet_Nam/13032030/2009	ggtgatgccccattccttgatcggctccgccgagatca aaagtccttaaaaggaagaggcaacaccc	-	*
CY128207	Human H1N1pdm2009 IAVs	Human	H1N1	pdm	2009	Viet_Nam	A/Viet_Nam/13032035/2009	ggtgatgccccattccttgatcggctccgccgagatca aaagtccttaaaaggaagaggcaacaccc	-	*
CY128215	Human H1N1pdm2009 IAVs	Human	H1N1	pdm	2009	Viet_Nam	A/Viet_Nam/13032036/2009	ggtgatgccccattccttgatcggctccgccgagatca aaagtccttaaaaggaagaggcaacaccc	-	*
CY128223	Human H1N1pdm2009 IAVs	Human	H1N1	pdm	2009	Viet_Nam	A/Viet_Nam/13032037/2009	ggtgatgccccattccttgatcggctccgccgagatca aaagtccttaaaaggaagaggcaacaccc	-	*
CY128231	Human H1N1pdm2009 IAVs	Human	H1N1	pdm	2009	Viet_Nam	A/Viet_Nam/13032040/2009	ggtgatgccccattccttgatcggctccgccgagatca aaagtccttaaaaggaagaggcaacaccc	-	*
CY128239	Human H1N1pdm2009 IAVs	Human	H1N1	pdm	2009	Viet_Nam	A/Viet_Nam/13032044/2009	ggtgatgccccattccttgatcggctccgccgagatca aaagtccttaaaaggaagaggcaacaccc	-	*
CY128263	Human H1N1pdm2009 IAVs	Human	H1N1	pdm	2009	Viet_Nam	A/Viet_Nam/13032059/2009	ggtgatgccccattccttgatcggctccgccgagatca aaagtccttaaaaggaagaggcaacaccc	-	*
CY128271	Human H1N1pdm2009 IAVs	Human	H1N1	pdm	2009	Viet_Nam	A/Viet_Nam/13032060/2009	ggtgatgccccattccttgatcggctccgccgagatca aaagtccttaaaaggaagaggcaacaccc	-	*
CY128287	Human H1N1pdm2009 IAVs	Human	H1N1	pdm	2009	Viet_Nam	A/Viet_Nam/13032071/2009	ggtgatgccccattccttgatcggctccgccgagatca aaagtccttaaaaggaagaggcaacaccc	-	*
CY128303	Human H1N1pdm2009 IAVs	Human	H1N1	pdm	2009	Viet_Nam	A/Viet_Nam/13032076/2009	ggtgatgccccattccttgatcggctccgccgagatca aaagtccttaaaaggaagaggcaacaccc	-	*
CY128311	Human H1N1pdm2009 IAVs	Human	H1N1	pdm	2009	Viet_Nam	A/Viet_Nam/13032079/2009	ggtgatgccccattccttgatcggctccgccgagatca aaagtccttaaaaggaagaggcaacaccc	-	*
CY128319	Human H1N1pdm2009 IAVs	Human	H1N1	pdm	2009	Viet_Nam	A/Viet_Nam/13032088/2009	ggtgatgccccattccttgatcggctccgccgagatca aaagtccttaaaaggaagaggcaacaccc	-	*
CY128327	Human H1N1pdm2009 IAVs	Human	H1N1	pdm	2009	Viet_Nam	A/Viet_Nam/13032093/2009	ggtgatgccccattccttgatcggctccgccgagatca aaagtccttaaaaggaagaggcaacaccc	-	*
CY072314	Human H1N1pdm2009 IAVs	Human	H1N1	pdm	2009	Poland	A/Warsaw/INS316/2009	ggtgatgccccattccttgatcggctccgccgagatca aaagtccttaaaaggaagaggcaacaccc	-	*
CY057394	Human H1N1pdm2009 IAVs	Human	H1N1	pdm	2009	USA	A/Wisconsin/629_D00036/2009	ggtgatgccccattccttgatcggctccgccgagatca aaagtccttaaaaggaagaggcaacaccc	-	*

CY063295	Human H1N1pdm2009 IAVs	Human	H1N1	pdm	2009	USA	A/Wisconsin/629_D00292/2009	ggtgatgccccattccttgatcggctccgccgagatca aaagtccttaaaaggaagaggcaacaccc	-	*
CY057858	Human H1N1pdm2009 IAVs	Human	H1N1	pdm	2009	USA	A/Wisconsin/629_D00295/2009	ggtgatgccccattccttgatcggctccgccgagatca aaagtccttaaaaggaagaggcaacaccc	-	*
CY087212	Human H1N1pdm2009 IAVs	Human	H1N1		2009	USA	A/Texas/JMS368/2009	ggtgatgccccattccttgatcggctccgccgagatca aaagtccttaaaaggaagaggcaacaccc	-	*
GU211247	Human H1N1pdm2009 IAVs	Human	H1N1	pdm	2009	Russia	A/Tomsk/02/2009	ggtgatgccccattccttgatcggctccgccgagatca aaagtccttaaaaggaagaggcaacaccc	-	*
CY057490	Human H1N1pdm2009 IAVs	Human	H1N1	pdm	2009	USA	A/Wisconsin/629_D00346/2009	ggtgatgccccattccttgatcggctccgccgagatca aaagtccttaaaaggaagaggcaacaccc	-	*
CY057031	Human H1N1pdm2009 IAVs	Human	H1N1	pdm	2009	USA	A/Wisconsin/629_D00357/2009	ggtgatgccccattccttgatcggctccgccgagatca aaagtccttaaaaggaagaggcaacaccc	-	*
CY058026	Human H1N1pdm2009 IAVs	Human	H1N1	pdm	2009	USA	A/Wisconsin/629_D00401/2009	ggtgatgccccattccttgatcggctccgccgagatca aaagtccttaaaaggaagaggcaacaccc	-	*
CY057514	Human H1N1pdm2009 IAVs	Human	H1N1	pdm	2009	USA	A/Wisconsin/629_D00410/2009	ggtgatgccccattccttgatcggctccgccgagatca aaagtccttaaaaggaagaggcaacaccc	-	*
CY057890	Human H1N1pdm2009 IAVs	Human	H1N1	pdm	2009	USA	A/Wisconsin/629_D00533/2009	ggtgatgccccattccttgatcggctccgccgagatca aaagtccttaaaaggaagaggcaacaccc	-	*
CY057610	Human H1N1pdm2009 IAVs	Human	H1N1	pdm	2009	USA	A/Wisconsin/629_D00557/2009	ggtgatgccccattccttgatcggctccgccgagatca aaagtccttaaaaggaagaggcaacaccc	-	*
CY058368	Human H1N1pdm2009 IAVs	Human	H1N1	pdm	2009	USA	A/Wisconsin/629_D00689/2009	ggtgatgccccattccttgatcggctccgccgagatca aaagtccttaaaaggaagaggcaacaccc	-	*
CY063255	Human H1N1pdm2009 IAVs	Human	H1N1	pdm	2009	USA	A/Wisconsin/629_D00692/2009	ggtgatgccccattccttgatcggctccgccgagatca aaagtccttaaaaggaagaggcaacaccc	-	*
CY056999	Human H1N1pdm2009 IAVs	Human	H1N1	pdm	2009	USA	A/Wisconsin/629_D00734/2009	ggtgatgccccattccttgatcggctccgccgagatca aaagtccttaaaaggaagaggcaacaccc	-	*
CY057458	Human H1N1pdm2009 IAVs	Human	H1N1	pdm	2009	USA	A/Wisconsin/629_D00748/2009	ggtgatgccccattccttgatcggctccgccgagatca aaagtccttaaaaggaagaggcaacaccc	-	*
CY063247	Human H1N1pdm2009 IAVs	Human	H1N1	pdm	2009	USA	A/Wisconsin/629_D00830/2009	ggtgatgccccattccttgatcggctccgccgagatca aaagtccttaaaaggaagaggcaacaccc	-	*
CY057466	Human H1N1pdm2009 IAVs	Human	H1N1	pdm	2009	USA	A/Wisconsin/629_D00839/2009	ggtgatgccccattccttgatcggctccgccgagatca aaagtccttaaaaggaagaggcaacaccc	-	*
CY063303	Human H1N1pdm2009 IAVs	Human	H1N1	pdm	2009	USA	A/Wisconsin/629_D00859/2009	ggtgatgccccattccttgatcggctccgccgagatca aaagtccttaaaaggaagaggcaacaccc	-	*
CY057834	Human H1N1pdm2009 IAVs	Human	H1N1	pdm	2009	USA	A/Wisconsin/629_D00911/2009	ggtgatgccccattccttgatcggctccgccgagatca aaagtccttaaaaggaagaggcaacaccc	-	*
CY057826	Human H1N1pdm2009 IAVs	Human	H1N1	pdm	2009	USA	A/Wisconsin/629_D00965/2009	ggtgatgccccattccttgatcggctccgccgagatca aaagtccttaaaaggaagaggcaacaccc	-	*
CY058464	Human H1N1pdm2009 IAVs	Human	H1N1	pdm	2009	USA	A/Wisconsin/629_D00996/2009	ggtgatgccccattccttgatcggctccgccgagatca aaagtccttaaaaggaagaggcaacaccc	-	*
CY058280	Human H1N1pdm2009 IAVs	Human	H1N1	pdm	2009	USA	A/Wisconsin/629_D01015/2009	ggtgatgccccattccttgatcggctccgccgagatca aaagtccttaaaaggaagaggcaacaccc	-	*
CY058408	Human H1N1pdm2009 IAVs	Human	H1N1	pdm	2009	USA	A/Wisconsin/629_D01038/2009	ggtgatgccccattccttgatcggctccgccgagatca aaagtccttaaaaggaagaggcaacaccc	-	*
CY058288	Human H1N1pdm2009 IAVs	Human	H1N1	pdm	2009	USA	A/Wisconsin/629_D01102/2009	ggtgatgccccattccttgatcggctccgccgagatca aaagtccttaaaaggaagaggcaacaccc	-	*
CY057450	Human H1N1pdm2009 IAVs	Human	H1N1	pdm	2009	USA	A/Wisconsin/629_D01355/2009	ggtgatgccccattccttgatcggctccgccgagatca aaagtccttaaaaggaagaggcaacaccc	-	*
CY057794	Human H1N1pdm2009 IAVs	Human	H1N1	pdm	2009	USA	A/Wisconsin/629_D01369/2009	ggtgatgccccattccttgatcggctccgccgagatca aaagtccttaaaaggaagaggcaacaccc	-	*

CY057970	Human H1N1pdm2009 IAVs	Human	H1N1	pdm	2009	USA	A/Wisconsin/629_D01528/2009	ggtgatgccccattccttgatcggctccgccgagatca aaagtccttaaaaggaagaggcaacaccc	-	*
CY057754	Human H1N1pdm2009 IAVs	Human	H1N1	pdm	2009	USA	A/Wisconsin/629_D01570/2009	ggtgatgccccattccttgatcggctccgccgagatca aaagtccttaaaaggaagaggcaacaccc	-	*
CY058352	Human H1N1pdm2009 IAVs	Human	H1N1		2009	USA	A/Wisconsin/629_D01582/2009	ggtgatgccccattccttgatcggctccgccgagatca aaagtccttaaaaggaagaggcaacaccc	-	*
CY057874	Human H1N1pdm2009 IAVs	Human	H1N1	pdm	2009	USA	A/Wisconsin/629_D01661/2009	ggtgatgccccattccttgatcggctccgccgagatca aaagtccttaaaaggaagaggcaacaccc	-	*
CY090044	Human H1N1pdm2009 IAVs	Human	H1N1	pdm	2009	USA	A/Wisconsin/629_D01667/2009	ggtgatgccccattccttgatcggctccgccgagatca aaagtccttaaaaggaagaggcaacaccc	-	*
CY057586	Human H1N1pdm2009 IAVs	Human	H1N1	pdm	2009	USA	A/Wisconsin/629_D01749/2009	ggtgatgccccattccttgatcggctccgccgagatca aaagtccttaaaaggaagaggcaacaccc	-	*
GQ359770	Human H1N1pdm2009 IAVs	Human	H1N1	pdm	2009	Sweden	A/Stockholm/30/2009	ggtgatgccccattccttgatcggctccgccgagatca aaagtccttaaaaggaagaggcaacaccc	-	*
CY057378	Human H1N1pdm2009 IAVs	Human	H1N1	pdm	2009	USA	A/Wisconsin/629_D01850/2009	ggtgatgccccattccttgatcggctccgccgagatca aaagtccttaaaaggaagaggcaacaccc	-	*
CY057434	Human H1N1pdm2009 IAVs	Human	H1N1	pdm	2009	USA	A/Wisconsin/629_D01935/2009	ggtgatgccccattccttgatcggctccgccgagatca aaagtccttaaaaggaagaggcaacaccc	-	*
CY058656	Human H1N1pdm2009 IAVs	Human	H1N1	pdm	2009	USA	A/Wisconsin/629_D02002/2009	ggtgatgccccattccttgatcggctccgccgagatca aaagtccttaaaaggaagaggcaacaccc	-	*
CY057015	Human H1N1pdm2009 IAVs	Human	H1N1	pdm	2009	USA	A/Wisconsin/629_D02041/2009	ggtgatgccccattccttgatcggctccgccgagatca aaagtccttaaaaggaagaggcaacaccc	-	*
CY057594	Human H1N1pdm2009 IAVs	Human	H1N1	pdm	2009	USA	A/Wisconsin/629_D02090/2009	ggtgatgccccattccttgatcggctccgccgagatca aaagtccttaaaaggaagaggcaacaccc	-	*
CY057023	Human H1N1pdm2009 IAVs	Human	H1N1	pdm	2009	USA	A/Wisconsin/629_D02133/2009	ggtgatgccccattccttgatcggctccgccgagatca aaagtccttaaaaggaagaggcaacaccc	-	*
CY090020	Human H1N1pdm2009 IAVs	Human	H1N1	pdm	2009	USA	A/Wisconsin/629_D02330/2009	ggtgatgccccattccttgatcggctccgccgagatca aaagtccttaaaaggaagaggcaacaccc	-	*
CY057650	Human H1N1pdm2009 IAVs	Human	H1N1	pdm	2009	USA	A/Wisconsin/629_D02342/2009	ggtgatgccccattccttgatcggctccgccgagatca aaagtccttaaaaggaagaggcaacaccc	-	*
CY057762	Human H1N1pdm2009 IAVs	Human	H1N1		2009	USA	A/Wisconsin/629_D02370/2009	ggtgatgccccattccttgatcggctccgccgagatca aaagtccttaaaaggaagaggcaacaccc	-	*
CY063239	Human H1N1pdm2009 IAVs	Human	H1N1	pdm	2009	USA	A/Wisconsin/629_D02442/2009	ggtgatgccccattccttgatcggctccgccgagatca aaagtccttaaaaggaagaggcaacaccc	-	*
CY057618	Human H1N1pdm2009 IAVs	Human	H1N1	pdm	2009	USA	A/Wisconsin/629_S1309/2009	ggtgatgccccattccttgatcggctccgccgagatca aaagtccttaaaaggaagaggcaacaccc	-	*
CY057642	Human H1N1pdm2009 IAVs	Human	H1N1		2009	USA	A/Wisconsin/629_S1350/2009	ggtgatgccccattccttgatcggctccgccgagatca aaagtccttaaaaggaagaggcaacaccc	-	*
CY057714	Human H1N1pdm2009 IAVs	Human	H1N1	pdm	2009	USA	A/Wisconsin/629_S1407/2009	ggtgatgccccattccttgatcggctccgccgagatca aaagtccttaaaaggaagaggcaacaccc	-	*
CY058320	Human H1N1pdm2009 IAVs	Human	H1N1	pdm	2009	USA	A/Wisconsin/629_S1414/2009	ggtgatgccccattccttgatcggctccgccgagatca aaagtccttaaaaggaagaggcaacaccc	-	*
CY058312	Human H1N1pdm2009 IAVs	Human	H1N1		2009	USA	A/Wisconsin/629_S1417/2009	ggtgatgccccattccttgatcggctccgccgagatca aaagtccttaaaaggaagaggcaacaccc	-	*
CY058640	Human H1N1pdm2009 IAVs	Human	H1N1	pdm	2009	USA	A/Wisconsin/629_S1425/2009	ggtgatgccccattccttgatcggctccgccgagatca aaagtccttaaaaggaagaggcaacaccc	-	*
CY058632	Human H1N1pdm2009 IAVs	Human	H1N1		2009	USA	A/Wisconsin/629_S1431/2009	ggtgatgccccattccttgatcggctccgccgagatca aaagtccttaaaaggaagaggcaacaccc	-	*
AB704530	Human H1N1pdm2009 IAVs	Human	H1N1	pdm	2009	Japan	A/Yamaguchi/248/2009	ggtgatgccccattccttgatcggctccgccgagatca aaagtccttaaaaggaagaggcaacaccc	-	*

GQ293080	Human H1N1pdm2009 IAVs	Human	H1N1	pdm	2009	China	A/Zhejiang/2/2009	ggtgatgccccattccttgatcggctccgccgagatcaaaagtccttaaaaggaagaggcaacaccc	-	*
GU112094	Human H1N1pdm2009 IAVs	Human	H1N1	pdm	2009	China	A/Zhejiang/DTID_ZJU02/2009	ggtgatgccccattccttgatcggctccgccgagatcaaaagtccttaaaaggaagaggcaacaccc	-	*
GU189653	Human H1N1pdm2009 IAVs	Human	H1N1	pdm	2009	China	A/Zhejiang/DTID_ZJU03/2009	ggtgatgccccattccttgatcggctccgccgagatcaaaagtccttaaaaggaagaggcaacaccc	-	*
HQ165794	Human H1N1pdm2009 IAVs	Human	H1N1	pdm	2009	Kenya	A/Mombasa/179/2009	ggtgatgccccattccttgatcggctccgccgagatcaaaagtccttaaaaggaagaggcaacaccc	-	*
CY049376	Human H1N1pdm2009 IAVs	Human	H1N1	pdm	2009	Singapore	A/Singapore/ON355/2009	ggtgatgccccattccttgatcggctccgccgagatcaaaagtccttaaaaggaagaggcaacaccc	-	*
CY128439	Human H1N1pdm2009 IAVs	Human	H1N1	pdm	2009	Viet_Nam	A/Viet_Nam/001_1424/2009	ggtgatgccccattccttgatcggctccgccgagatcaaaagtccttaaaaggaagaggcaacaccc	-	*
CY128041	Human H1N1pdm2009 IAVs	Human	H1	pdm	2009	Viet_Nam	A/Viet_Nam/13032042/2009	ggtgatgccccattccttgatcggctccgccgagatcaaaagtccttaaaaggaagaggcaacaccc	-	*
CY083930	Human H1N1pdm2009 IAVs	Human	H1N1	pdm	2009	Poland	A/Warsaw/INS148/2009	ggtgatgccccattccttgatcggctccgccgagatcaaaagtccttaaaaggaagaggcaacaccc	-	*
GU135996	Human H1N1pdm2009 IAVs	Human	H1N1	pdm	2009	Japan	A/Aichi/202/2009	ggtgatgccccattccttgatcggctccgccgagatcaaaagtccttaaaaggaagaggcaacaccc	-	*
GQ365414	Human H1N1pdm2009 IAVs	Human	H1N1	pdm	2009	Japan	A/Akita/1/2009	ggtgatgccccattccttgatcggctccgccgagatcaaaagtccttaaaaggaagaggcaacaccc	-	*
KC780103	Human H1N1pdm2009 IAVs	Human	H1N1	pdm	2009	USA	A/Alaska/38/2009	ggtgatgccccattccttgatcggctccgccgagatcaaaagtccttaaaaggaagaggcaacaccc	-	*
HM569663	Human H1N1pdm2009 IAVs	Human	H1N1	pdm	2009	Argentina	A/Argentina/07_09GP/2009	ggtgatgccccattccttgatcggctccgccgagatcaaaagtccttaaaaggaagaggcaacaccc	-	*
HM569679	Human H1N1pdm2009 IAVs	Human	H1N1	pdm	2009	Argentina	A/Argentina/19527/2009	ggtgatgccccattccttgatcggctccgccgagatcaaaagtccttaaaaggaagaggcaacaccc	-	*
HM569687	Human H1N1pdm2009 IAVs	Human	H1N1	pdm	2009	Argentina	A/Argentina/19618/2009	ggtgatgccccattccttgatcggctccgccgagatcaaaagtccttaaaaggaagaggcaacaccc	-	*
KC780769	Human H1N1pdm2009 IAVs	Human	H1N1	pdm	2009	USA	A/Arkansas/09/2009	ggtgatgccccattccttgatcggctccgccgagatcaaaagtccttaaaaggaagaggcaacaccc	-	*
CY065766	Human H1N1pdm2009 IAVs	Human	H1N1	pdm	2009	Canada	A/British_Columbia/GFA0401/2009	ggtgatgccccattccttgatcggctccgccgagatcaaaagtccttaaaaggaagaggcaacaccc	-	*
JN032410	Human H1N1pdm2009 IAVs	Human	H1N1	pdm	2009	China	A/Changchun/01/2009	ggtgatgccccattccttgatcggctccgccgagatcaaaagtccttaaaaggaagaggcaacaccc	-	*
KJ023100	Human H1N1pdm2009 IAVs	Human	H1N1	pdm	2009	India	A/Chattisgarh/010/2009	ggtgatgccccattccttgatcggctccgccgagatcaaaagtccttaaaaggaagaggcaacaccc	-	*
GU135997	Human H1N1pdm2009 IAVs	Human	H1N1	pdm	2009	Japan	A/Chiba_C/48/2009	ggtgatgccccattccttgatcggctccgccgagatcaaaagtccttaaaaggaagaggcaacaccc	-	*
GU135998	Human H1N1pdm2009 IAVs	Human	H1N1	pdm	2009	Japan	A/Chiba_C/51/2009	ggtgatgccccattccttgatcggctccgccgagatcaaaagtccttaaaaggaagaggcaacaccc	-	*
KC780925	Human H1N1pdm2009 IAVs	Human	H1N1	pdm	2009	USA	A/Colorado/13/2009	ggtgatgccccattccttgatcggctccgccgagatcaaaagtccttaaaaggaagaggcaacaccc	-	*
KJ023108	Human H1N1pdm2009 IAVs	Human	H1N1	pdm	2009	India	A/Delhi/018/2009	ggtgatgccccattccttgatcggctccgccgagatcaaaagtccttaaaaggaagaggcaacaccc	-	*
CY065234	Human H1N1pdm2009 IAVs	Human	H1N1	pdm	2009	United_Kingdom	A/England/200/2009	ggtgatgccccattccttgatcggctccgccgagatcaaaagtccttaaaaggaagaggcaacaccc	-	*
CY065194	Human H1N1pdm2009 IAVs	Human	H1N1	pdm	2009	United_Kingdom	A/England/202/2009	ggtgatgccccattccttgatcggctccgccgagatcaaaagtccttaaaaggaagaggcaacaccc	-	*
CY065226	Human H1N1pdm2009 IAVs	Human	H1N1	pdm	2009	United_Kingdom	A/England/207/2009	ggtgatgccccattccttgatcggctccgccgagatcaaaagtccttaaaaggaagaggcaacaccc	-	*

CY065274	Human H1N1pdm2009 IAVs	Human	H1N1	pdm	2009	United_Kingdom	A/England/214/2009	ggtgatgccccattccttgatcggctccgccgagatca aaagtccttaaaaggaagaggcaacaccc	-	*
CY065346	Human H1N1pdm2009 IAVs	Human	H1N1	pdm	2009	United_Kingdom	A/England/223/2009	ggtgatgccccattccttgatcggctccgccgagatca aaagtccttaaaaggaagaggcaacaccc	-	*
CY065450	Human H1N1pdm2009 IAVs	Human	H1N1	pdm	2009	United_Kingdom	A/England/259/2009	ggtgatgccccattccttgatcggctccgccgagatca aaagtccttaaaaggaagaggcaacaccc	-	*
CY069857	Human H1N1pdm2009 IAVs	Human	H1N1	pdm	2009	United_Kingdom	A/England/260/2009	ggtgatgccccattccttgatcggctccgccgagatca aaagtccttaaaaggaagaggcaacaccc	-	*
CY065482	Human H1N1pdm2009 IAVs	Human	H1N1		2009	United_Kingdom	A/England/263/2009	ggtgatgccccattccttgatcggctccgccgagatca aaagtccttaaaaggaagaggcaacaccc	-	*
CY065490	Human H1N1pdm2009 IAVs	Human	H1N1	pdm	2009	United_Kingdom	A/England/264/2009	ggtgatgccccattccttgatcggctccgccgagatca aaagtccttaaaaggaagaggcaacaccc	-	*
CY065498	Human H1N1pdm2009 IAVs	Human	H1N1	pdm	2009	United_Kingdom	A/England/265/2009	ggtgatgccccattccttgatcggctccgccgagatca aaagtccttaaaaggaagaggcaacaccc	-	*
CY069873	Human H1N1pdm2009 IAVs	Human	H1N1	pdm	2009	United_Kingdom	A/England/271/2009	ggtgatgccccattccttgatcggctccgccgagatca aaagtccttaaaaggaagaggcaacaccc	-	*
CY065602	Human H1N1pdm2009 IAVs	Human	H1N1		2009	United_Kingdom	A/England/389/2009	ggtgatgccccattccttgatcggctccgccgagatca aaagtccttaaaaggaagaggcaacaccc	-	*
CY065610	Human H1N1pdm2009 IAVs	Human	H1N1	pdm	2009	United_Kingdom	A/England/392/2009	ggtgatgccccattccttgatcggctccgccgagatca aaagtccttaaaaggaagaggcaacaccc	-	*
CY069905	Human H1N1pdm2009 IAVs	Human	H1N1	pdm	2009	United_Kingdom	A/England/393/2009	ggtgatgccccattccttgatcggctccgccgagatca aaagtccttaaaaggaagaggcaacaccc	-	*
CY065634	Human H1N1pdm2009 IAVs	Human	H1N1	pdm	2009	United_Kingdom	A/England/398/2009	ggtgatgccccattccttgatcggctccgccgagatca aaagtccttaaaaggaagaggcaacaccc	-	*
CY065650	Human H1N1pdm2009 IAVs	Human	H1N1	pdm	2009	United_Kingdom	A/England/402/2009	ggtgatgccccattccttgatcggctccgccgagatca aaagtccttaaaaggaagaggcaacaccc	-	*
CY065658	Human H1N1pdm2009 IAVs	Human	H1N1	pdm	2009	United_Kingdom	A/England/411/2009	ggtgatgccccattccttgatcggctccgccgagatca aaagtccttaaaaggaagaggcaacaccc	-	*
CY069920	Human H1N1pdm2009 IAVs	Human	H1N1	pdm	2009	United_Kingdom	A/England/413/2009	ggtgatgccccattccttgatcggctccgccgagatca aaagtccttaaaaggaagaggcaacaccc	-	*
CY065674	Human H1N1pdm2009 IAVs	Human	H1N1	pdm	2009	United_Kingdom	A/England/418/2009	ggtgatgccccattccttgatcggctccgccgagatca aaagtccttaaaaggaagaggcaacaccc	-	*
JX625986	Human H1N1pdm2009 IAVs	Human	H1N1	pdm	2009	United_Kingdom	A/England/421/2009	ggtgatgccccattccttgatcggctccgccgagatca aaagtccttaaaaggaagaggcaacaccc	-	*
CY065730	Human H1N1pdm2009 IAVs	Human	H1N1	pdm	2009	United_Kingdom	A/England/434/2009	ggtgatgccccattccttgatcggctccgccgagatca aaagtccttaaaaggaagaggcaacaccc	-	*
JX625399	Human H1N1pdm2009 IAVs	Human	H1N1	pdm	2009	United_Kingdom	A/England/527/2009	ggtgatgccccattccttgatcggctccgccgagatca aaagtccttaaaaggaagaggcaacaccc	-	*
CY069753	Human H1N1pdm2009 IAVs	Human	H1N1	pdm	2009	United_Kingdom	A/England/527/2009	ggtgatgccccattccttgatcggctccgccgagatca aaagtccttaaaaggaagaggcaacaccc	-	*
JX625455	Human H1N1pdm2009 IAVs	Human	H1N1	pdm	2009	United_Kingdom	A/England/671/2009	ggtgatgccccattccttgatcggctccgccgagatca aaagtccttaaaaggaagaggcaacaccc	-	*
CY065186	Human H1N1pdm2009 IAVs	Human	H1N1	pdm	2009	United_Kingdom	A/England/675/2009	ggtgatgccccattccttgatcggctccgccgagatca aaagtccttaaaaggaagaggcaacaccc	-	*
JX625447	Human H1N1pdm2009 IAVs	Human	H1N1	pdm	2009	United_Kingdom	A/England/687/2009	ggtgatgccccattccttgatcggctccgccgagatca aaagtccttaaaaggaagaggcaacaccc	-	*
CY065170	Human H1N1pdm2009 IAVs	Human	H1N1	pdm	2009	United_Kingdom	A/England/92860032/2009	ggtgatgccccattccttgatcggctccgccgagatca aaagtccttaaaaggaagaggcaacaccc	-	*
JX625487	Human H1N1pdm2009 IAVs	Human	H1N1	pdm	2009	United_Kingdom	A/England/93320016/2009	ggtgatgccccattccttgatcggctccgccgagatca aaagtccttaaaaggaagaggcaacaccc	-	*

JX625494	Human H1N1pdm2009 IAVs	Human	H1N1	pdm	2009	United_Kingdom	A/England/93520038/2009	ggtgatgccccattccttgatcggctccgccgagatca aaagtccttaaaaggaagaggcaacaccc	-	*
JX625602	Human H1N1pdm2009 IAVs	Human	H1N1		2009	United_Kingdom	A/England/94720002/2009	ggtgatgccccattccttgatcggctccgccgagatca aaagtccttaaaaggaagaggcaacaccc	-	*
CY065458	Human H1N1pdm2009 IAVs	Human	H1N1	pdm	2009	United_Kingdom	A/England/XFL00418/2009	ggtgatgccccattccttgatcggctccgccgagatca aaagtccttaaaaggaagaggcaacaccc	-	*
CY065474	Human H1N1pdm2009 IAVs	Human	H1N1	pdm	2009	United_Kingdom	A/England/XFL00473/2009	ggtgatgccccattccttgatcggctccgccgagatca aaagtccttaaaaggaagaggcaacaccc	-	*
CY065506	Human H1N1pdm2009 IAVs	Human	H1N1	pdm	2009	United_Kingdom	A/England/XFL00481/2009	ggtgatgccccattccttgatcggctccgccgagatca aaagtccttaaaaggaagaggcaacaccc	-	*
CY065514	Human H1N1pdm2009 IAVs	Human	H1N1	pdm	2009	United_Kingdom	A/England/XFL00482/2009	ggtgatgccccattccttgatcggctccgccgagatca aaagtccttaaaaggaagaggcaacaccc	-	*
CY065530	Human H1N1pdm2009 IAVs	Human	H1N1	pdm	2009	United_Kingdom	A/England/XFL00532/2009	ggtgatgccccattccttgatcggctccgccgagatca aaagtccttaaaaggaagaggcaacaccc	-	*
GQ365422	Human H1N1pdm2009 IAVs	Human	H1N1		2009	Japan	A/Fukushima/1/2009	ggtgatgccccattccttgatcggctccgccgagatca aaagtccttaaaaggaagaggcaacaccc	-	*
GQ200239	Human H1N1pdm2009 IAVs	Human	H1N1	pdm	2009	USA	A/Georgia/01/2009	ggtgatgccccattccttgatcggctccgccgagatca aaagtccttaaaaggaagaggcaacaccc	-	*
KC781692	Human H1N1pdm2009 IAVs	Human	H1N1		2009	USA	A/Georgia/08/2009	ggtgatgccccattccttgatcggctccgccgagatca aaagtccttaaaaggaagaggcaacaccc	-	*
GU135999	Human H1N1pdm2009 IAVs	Human	H1N1	pdm	2009	Japan	A/Gifu_C/67/2009	ggtgatgccccattccttgatcggctccgccgagatca aaagtccttaaaaggaagaggcaacaccc	-	*
GU136000	Human H1N1pdm2009 IAVs	Human	H1N1	pdm	2009	Japan	A/Hiroshima/200/2009	ggtgatgccccattccttgatcggctccgccgagatca aaagtccttaaaaggaagaggcaacaccc	-	*
GU136001	Human H1N1pdm2009 IAVs	Human	H1N1	pdm	2009	Japan	A/Hiroshima/201/2009	ggtgatgccccattccttgatcggctccgccgagatca aaagtccttaaaaggaagaggcaacaccc	-	*
GU136002	Human H1N1pdm2009 IAVs	Human	H1N1		2009	Japan	A/Hiroshima/207/2009	ggtgatgccccattccttgatcggctccgccgagatca aaagtccttaaaaggaagaggcaacaccc	-	*
CY095866	Human H1N1pdm2009 IAVs	Human	H1N1	pdm	2009	China	A/Hubei/74/2009	ggtgatgccccattccttgatcggctccgccgagatca aaagtccttaaaaggaagaggcaacaccc	-	*
GQ375884	Human H1N1pdm2009 IAVs	Human	H1N1	pdm	2009	Japan	A/Iwate/1/2009	ggtgatgccccattccttgatcggctccgccgagatca aaagtccttaaaaggaagaggcaacaccc	-	*
GU136006	Human H1N1pdm2009 IAVs	Human	H1N1	pdm	2009	Japan	A/Kanagawa/137/2009	ggtgatgccccattccttgatcggctccgccgagatca aaagtccttaaaaggaagaggcaacaccc	-	*
KC780970	Human H1N1pdm2009 IAVs	Human	H1N1	pdm	2009	USA	A/Kansas/23/2009	ggtgatgccccattccttgatcggctccgccgagatca aaagtccttaaaaggaagaggcaacaccc	-	*
JX875046	Human H1N1pdm2009 IAVs	Human	H1N1	pdm	2009	USA	A/Kentucky/104/2009	ggtgatgccccattccttgatcggctccgccgagatca aaagtccttaaaaggaagaggcaacaccc	-	*
JX875018	Human H1N1pdm2009 IAVs	Human	H1N1	pdm	2009	USA	A/Kentucky/99/2009	ggtgatgccccattccttgatcggctccgccgagatca aaagtccttaaaaggaagaggcaacaccc	-	*
HM189510	Human H1N1pdm2009 IAVs	Human	H1N1	pdm	2009	South_Korea	A/Korea/CJ109/2009	ggtgatgccccattccttgatcggctccgccgagatca aaagtccttaaaaggaagaggcaacaccc	-	*
HM189511	Human H1N1pdm2009 IAVs	Human	H1N1	pdm	2009	South_Korea	A/Korea/CJ112/2009	ggtgatgccccattccttgatcggctccgccgagatca aaagtccttaaaaggaagaggcaacaccc	-	*
HM189507	Human H1N1pdm2009 IAVs	Human	H1N1	pdm	2009	South_Korea	A/Korea/CJ40/2009	ggtgatgccccattccttgatcggctccgccgagatca aaagtccttaaaaggaagaggcaacaccc	-	*
HM189509	Human H1N1pdm2009 IAVs	Human	H1N1	pdm	2009	South_Korea	A/Korea/CJ68/2009	ggtgatgccccattccttgatcggctccgccgagatca aaagtccttaaaaggaagaggcaacaccc	-	*
FN423709	Human H1N1pdm2009 IAVs	Human	H1N1	pdm	2009	Luxembourg	A/Luxembourg/43/2009	ggtgatgccccattccttgatcggctccgccgagatca aaagtccttaaaaggaagaggcaacaccc	-	*

GQ323499	Human H1N1pdm2009 IAVs	Human	H1N1	pdm	2009	USA	A/Michigan/06/2009	ggtgatgccccattccttgatcggctccgccgagatca aaagtccttaaaaggaagaggcaacaccc	-	*
GU136008	Human H1N1pdm2009 IAVs	Human	H1N1	pdm	2009	Japan	A/Mie/41/2009	ggtgatgccccattccttgatcggctccgccgagatca aaagtccttaaaaggaagaggcaacaccc	-	*
GU136009	Human H1N1pdm2009 IAVs	Human	H1N1	pdm	2009	Japan	A/Mie/52/2009	ggtgatgccccattccttgatcggctccgccgagatca aaagtccttaaaaggaagaggcaacaccc	-	*
KC782244	Human H1N1pdm2009 IAVs	Human	H1N1	pdm	2009	USA	A/Minnesota/40/2009	ggtgatgccccattccttgatcggctccgccgagatca aaagtccttaaaaggaagaggcaacaccc	-	*
KC781960	Human H1N1pdm2009 IAVs	Human	H1N1	pdm	2009	USA	A/Mississippi/05/2009	ggtgatgccccattccttgatcggctccgccgagatca aaagtccttaaaaggaagaggcaacaccc	-	*
GQ377042	Human H1N1pdm2009 IAVs	Human	H1N1		2009	USA	A/Montana/07/2009	ggtgatgccccattccttgatcggctccgccgagatca aaagtccttaaaaggaagaggcaacaccc	-	*
GU136012	Human H1N1pdm2009 IAVs	Human	H1N1		2009	Myanmar	A/Myanmar/JP101/2009	ggtgatgccccattccttgatcggctccgccgagatca aaagtccttaaaaggaagaggcaacaccc	-	*
GQ160598	Human H1N1pdm2009 IAVs	Human	H1N1	pdm	2009	USA	A/Nebraska/03/2009	ggtgatgccccattccttgatcggctccgccgagatca aaagtccttaaaaggaagaggcaacaccc	-	*
KC780397	Human H1N1pdm2009 IAVs	Human	H1N1	pdm	2009	USA	A/New_Mexico/06/2009	ggtgatgccccattccttgatcggctccgccgagatca aaagtccttaaaaggaagaggcaacaccc	-	*
KC780118	Human H1N1pdm2009 IAVs	Human	H1N1	pdm	2009	USA	A/New_Mexico/07/2009	ggtgatgccccattccttgatcggctccgccgagatca aaagtccttaaaaggaagaggcaacaccc	-	*
GQ377096	Human H1N1pdm2009 IAVs	Human	H1N1	pdm	2009	USA	A/New_York/09/2009	ggtgatgccccattccttgatcggctccgccgagatca aaagtccttaaaaggaagaggcaacaccc	-	*
GQ168882	Human H1N1pdm2009 IAVs	Human	H1N1		2009	USA	A/New_York/13/2009	ggtgatgccccattccttgatcggctccgccgagatca aaagtccttaaaaggaagaggcaacaccc	-	*
GQ168862	Human H1N1pdm2009 IAVs	Human	H1N1		2009	USA	A/New_York/20/2009	ggtgatgccccattccttgatcggctccgccgagatca aaagtccttaaaaggaagaggcaacaccc	-	*
GQ200252	Human H1N1pdm2009 IAVs	Human	H1N1		2009	USA	A/New_York/21/2009	ggtgatgccccattccttgatcggctccgccgagatca aaagtccttaaaaggaagaggcaacaccc	-	*
GQ168846	Human H1N1pdm2009 IAVs	Human	H1N1	pdm	2009	USA	A/New_York/22/2009	ggtgatgccccattccttgatcggctccgccgagatca aaagtccttaaaaggaagaggcaacaccc	-	*
GQ232048	Human H1N1pdm2009 IAVs	Human	H1N1	pdm	2009	USA	A/New_York/25/2009	ggtgatgccccattccttgatcggctccgccgagatca aaagtccttaaaaggaagaggcaacaccc	-	*
CY041078	Human H1N1pdm2009 IAVs	Human	H1N1	pdm	2009	USA	A/New_York/3170/2009	ggtgatgccccattccttgatcggctccgccgagatca aaagtccttaaaaggaagaggcaacaccc	-	*
CY045049	Human H1N1pdm2009 IAVs	Human	H1N1	pdm	2009	USA	A/New_York/4006/2009	ggtgatgccccattccttgatcggctccgccgagatca aaagtccttaaaaggaagaggcaacaccc	-	*
KC781480	Human H1N1pdm2009 IAVs	Human	H1N1	pdm	2009	USA	A/New_York/96/2009	ggtgatgccccattccttgatcggctccgccgagatca aaagtccttaaaaggaagaggcaacaccc	-	*
GU136017	Human H1N1pdm2009 IAVs	Human	H1N1	pdm	2009	Japan	A/Niigata/717/2009	ggtgatgccccattccttgatcggctccgccgagatca aaagtccttaaaaggaagaggcaacaccc	-	*
KC781745	Human H1N1pdm2009 IAVs	Human	H1N1	pdm	2009	USA	A/North_Carolina/37/2009	ggtgatgccccattccttgatcggctccgccgagatca aaagtccttaaaaggaagaggcaacaccc	-	*
KC781053	Human H1N1pdm2009 IAVs	Human	H1N1	pdm	2009	USA	A/North_Carolina/44/2009	ggtgatgccccattccttgatcggctccgccgagatca aaagtccttaaaaggaagaggcaacaccc	-	*
GQ377098	Human H1N1pdm2009 IAVs	Human	H1N1	pdm	2009	USA	A/Ohio/07/2009	ggtgatgccccattccttgatcggctccgccgagatca aaagtccttaaaaggaagaggcaacaccc	-	*
GQ168872	Human H1N1pdm2009 IAVs	Human	H1N1		2009	USA	A/Ohio/07/2009	ggtgatgccccattccttgatcggctccgccgagatca aaagtccttaaaaggaagaggcaacaccc	-	*
CY060570	Human H1N1pdm2009 IAVs	Human	H1N1	pdm	2009	Canada	A/Ontario/296008/2009	ggtgatgccccattccttgatcggctccgccgagatca aaagtccttaaaaggaagaggcaacaccc	-	*

CY060650	Human H1N1pdm2009 IAVs	Human	H1N1	pdm	2009	Canada	A/Ontario/314603/2009	ggtgatgccccattccttgatcggctccgccgagatca aaagtccttaaaaggaagaggcaacaccc	-	*
CY060682	Human H1N1pdm2009 IAVs	Human	H1N1		2009	Canada	A/Ontario/315107/2009	ggtgatgccccattccttgatcggctccgccgagatca aaagtccttaaaaggaagaggcaacaccc	-	*
CY060738	Human H1N1pdm2009 IAVs	Human	H1N1	pdm	2009	Canada	A/Ontario/35273/2009	ggtgatgccccattccttgatcggctccgccgagatca aaagtccttaaaaggaagaggcaacaccc	-	*
CY054666	Human H1N1pdm2009 IAVs	Human	H1N1	pdm	2009	Russia	A/Russia/100/2009	ggtgatgccccattccttgatcggctccgccgagatca aaagtccttaaaaggaagaggcaacaccc	-	*
CY054658	Human H1N1pdm2009 IAVs	Human	H1N1	pdm	2009	Russia	A/Russia/61/2009	ggtgatgccccattccttgatcggctccgccgagatca aaagtccttaaaaggaagaggcaacaccc	-	*
GU136019	Human H1N1pdm2009 IAVs	Human	H1N1	pdm	2009	Japan	A/Saitama/85/2009	ggtgatgccccattccttgatcggctccgccgagatca aaagtccttaaaaggaagaggcaacaccc	-	*
GQ365450	Human H1N1pdm2009 IAVs	Human	H1N1	pdm	2009	Japan	A/Sapporo/1/2009	ggtgatgccccattccttgatcggctccgccgagatca aaagtccttaaaaggaagaggcaacaccc	-	*
CY065146	Human H1N1pdm2009 IAVs	Human	H1N1	pdm	2009	United_Kingdom	A/Scotland/91800797/2009	ggtgatgccccattccttgatcggctccgccgagatca aaagtccttaaaaggaagaggcaacaccc	-	*
CY065154	Human H1N1pdm2009 IAVs	Human	H1N1	pdm	2009	United_Kingdom	A/Scotland/91800799/2009	ggtgatgccccattccttgatcggctccgccgagatca aaagtccttaaaaggaagaggcaacaccc	-	*
GQ324568	Human H1N1pdm2009 IAVs	Human	H1N1	pdm	2009	Japan	A/Shiga/3/2009	ggtgatgccccattccttgatcggctccgccgagatca aaagtccttaaaaggaagaggcaacaccc	-	*
GQ334350	Human H1N1pdm2009 IAVs	Human	H1N1	pdm	2009	Japan	A/Shizuoka/759/2009	ggtgatgccccattccttgatcggctccgccgagatca aaagtccttaaaaggaagaggcaacaccc	-	*
CY055307	Human H1N1pdm2009 IAVs	Human	H1N1	pdm	2009	Singapore	A/Singapore/GN285/2009	ggtgatgccccattccttgatcggctccgccgagatca aaagtccttaaaaggaagaggcaacaccc	-	*
CY122559	Human H1N1pdm2009 IAVs	Human	H1N1	pdm	2009	Singapore	A/Singapore/GP1020/2009	ggtgatgccccattccttgatcggctccgccgagatca aaagtccttaaaaggaagaggcaacaccc	-	*
CY122575	Human H1N1pdm2009 IAVs	Human	H1N1	pdm	2009	Singapore	A/Singapore/GP1087/2009	ggtgatgccccattccttgatcggctccgccgagatca aaagtccttaaaaggaagaggcaacaccc	-	*
CY122583	Human H1N1pdm2009 IAVs	Human	H1N1	pdm	2009	Singapore	A/Singapore/GP1090/2009	ggtgatgccccattccttgatcggctccgccgagatca aaagtccttaaaaggaagaggcaacaccc	-	*
CY122607	Human H1N1pdm2009 IAVs	Human	H1N1	pdm	2009	Singapore	A/Singapore/GP1097/2009	ggtgatgccccattccttgatcggctccgccgagatca aaagtccttaaaaggaagaggcaacaccc	-	*
CY122623	Human H1N1pdm2009 IAVs	Human	H1N1	pdm	2009	Singapore	A/Singapore/GP1102/2009	ggtgatgccccattccttgatcggctccgccgagatca aaagtccttaaaaggaagaggcaacaccc	-	*
CY122627	Human H1N1pdm2009 IAVs	Human	H1N1	pdm	2009	Singapore	A/Singapore/GP1107/2009	ggtgatgccccattccttgatcggctccgccgagatca aaagtccttaaaaggaagaggcaacaccc	-	*
CY122651	Human H1N1pdm2009 IAVs	Human	H1N1	pdm	2009	Singapore	A/Singapore/GP1122/2009	ggtgatgccccattccttgatcggctccgccgagatca aaagtccttaaaaggaagaggcaacaccc	-	*
CY122659	Human H1N1pdm2009 IAVs	Human	H1N1	pdm	2009	Singapore	A/Singapore/GP1124/2009	ggtgatgccccattccttgatcggctccgccgagatca aaagtccttaaaaggaagaggcaacaccc	-	*
CY122683	Human H1N1pdm2009 IAVs	Human	H1N1	pdm	2009	Singapore	A/Singapore/GP1136/2009	ggtgatgccccattccttgatcggctccgccgagatca aaagtccttaaaaggaagaggcaacaccc	-	*
CY122691	Human H1N1pdm2009 IAVs	Human	H1N1	pdm	2009	Singapore	A/Singapore/GP1137/2009	ggtgatgccccattccttgatcggctccgccgagatca aaagtccttaaaaggaagaggcaacaccc	-	*
CY122707	Human H1N1pdm2009 IAVs	Human	H1N1	pdm	2009	Singapore	A/Singapore/GP1146/2009	ggtgatgccccattccttgatcggctccgccgagatca aaagtccttaaaaggaagaggcaacaccc	-	*
CY122723	Human H1N1pdm2009 IAVs	Human	H1N1	pdm	2009	Singapore	A/Singapore/GP1160/2009	ggtgatgccccattccttgatcggctccgccgagatca aaagtccttaaaaggaagaggcaacaccc	-	*
CY122751	Human H1N1pdm2009 IAVs	Human	H1N1	pdm	2009	Singapore	A/Singapore/GP1167/2009	ggtgatgccccattccttgatcggctccgccgagatca aaagtccttaaaaggaagaggcaacaccc	-	*

CY122759	Human H1N1pdm2009 IAVs	Human	H1N1	pdm	2009	Singapore	A/Singapore/GP1169/2009	ggtgatgccccattccttgatcggctccgccgagatca aaagtccttaaaaggaagaggcaacaccc	-	*
CY122775	Human H1N1pdm2009 IAVs	Human	H1N1	pdm	2009	Singapore	A/Singapore/GP1178/2009	ggtgatgccccattccttgatcggctccgccgagatca aaagtccttaaaaggaagaggcaacaccc	-	*
CY122783	Human H1N1pdm2009 IAVs	Human	H1N1	pdm	2009	Singapore	A/Singapore/GP1182/2009	ggtgatgccccattccttgatcggctccgccgagatca aaagtccttaaaaggaagaggcaacaccc	-	*
CY122863	Human H1N1pdm2009 IAVs	Human	H1N1	pdm	2009	Singapore	A/Singapore/GP2290/2009	ggtgatgccccattccttgatcggctccgccgagatca aaagtccttaaaaggaagaggcaacaccc	-	*
CY122875	Human H1N1pdm2009 IAVs	Human	H1N1	pdm	2009	Singapore	A/Singapore/GP2297/2009	ggtgatgccccattccttgatcggctccgccgagatca aaagtccttaaaaggaagaggcaacaccc	-	*
CY122931	Human H1N1pdm2009 IAVs	Human	H1N1	pdm	2009	Singapore	A/Singapore/GP2680/2009	ggtgatgccccattccttgatcggctccgccgagatca aaagtccttaaaaggaagaggcaacaccc	-	*
CY122939	Human H1N1pdm2009 IAVs	Human	H1N1	pdm	2009	Singapore	A/Singapore/GP2693/2009	ggtgatgccccattccttgatcggctccgccgagatca aaagtccttaaaaggaagaggcaacaccc	-	*
CY123015	Human H1N1pdm2009 IAVs	Human	H1N1	pdm	2009	Singapore	A/Singapore/GP3040/2009	ggtgatgccccattccttgatcggctccgccgagatca aaagtccttaaaaggaagaggcaacaccc	-	*
CY123023	Human H1N1pdm2009 IAVs	Human	H1N1	pdm	2009	Singapore	A/Singapore/GP3044/2009	ggtgatgccccattccttgatcggctccgccgagatca aaagtccttaaaaggaagaggcaacaccc	-	*
CY123031	Human H1N1pdm2009 IAVs	Human	H1N1	pdm	2009	Singapore	A/Singapore/GP3069/2009	ggtgatgccccattccttgatcggctccgccgagatca aaagtccttaaaaggaagaggcaacaccc	-	*
CY123039	Human H1N1pdm2009 IAVs	Human	H1N1	pdm	2009	Singapore	A/Singapore/GP3074/2009	ggtgatgccccattccttgatcggctccgccgagatca aaagtccttaaaaggaagaggcaacaccc	-	*
CY123063	Human H1N1pdm2009 IAVs	Human	H1N1	pdm	2009	Singapore	A/Singapore/GP3104/2009	ggtgatgccccattccttgatcggctccgccgagatca aaagtccttaaaaggaagaggcaacaccc	-	*
CY123087	Human H1N1pdm2009 IAVs	Human	H1N1	pdm	2009	Singapore	A/Singapore/GP3179/2009	ggtgatgccccattccttgatcggctccgccgagatca aaagtccttaaaaggaagaggcaacaccc	-	*
CY123110	Human H1N1pdm2009 IAVs	Human	H1N1	pdm	2009	Singapore	A/Singapore/GP3191/2009	ggtgatgccccattccttgatcggctccgccgagatca aaagtccttaaaaggaagaggcaacaccc	-	*
CY123118	Human H1N1pdm2009 IAVs	Human	H1N1	pdm	2009	Singapore	A/Singapore/GP3205/2009	ggtgatgccccattccttgatcggctccgccgagatca aaagtccttaaaaggaagaggcaacaccc	-	*
CY123126	Human H1N1pdm2009 IAVs	Human	H1N1	pdm	2009	Singapore	A/Singapore/GP3212/2009	ggtgatgccccattccttgatcggctccgccgagatca aaagtccttaaaaggaagaggcaacaccc	-	*
CY123272	Human H1N1pdm2009 IAVs	Human	H1N1		2009	Singapore	A/Singapore/GP4419/2009	ggtgatgccccattccttgatcggctccgccgagatca aaagtccttaaaaggaagaggcaacaccc	-	*
CY123370	Human H1N1pdm2009 IAVs	Human	H1N1	pdm	2009	Singapore	A/Singapore/ON1048/2009	ggtgatgccccattccttgatcggctccgccgagatca aaagtccttaaaaggaagaggcaacaccc	-	*
CY123378	Human H1N1pdm2009 IAVs	Human	H1N1	pdm	2009	Singapore	A/Singapore/ON1058/2009	ggtgatgccccattccttgatcggctccgccgagatca aaagtccttaaaaggaagaggcaacaccc	-	*
CY123418	Human H1N1pdm2009 IAVs	Human	H1N1	pdm	2009	Singapore	A/Singapore/ON1091/2009	ggtgatgccccattccttgatcggctccgccgagatca aaagtccttaaaaggaagaggcaacaccc	-	*
CY123434	Human H1N1pdm2009 IAVs	Human	H1N1	pdm	2009	Singapore	A/Singapore/ON1102/2009	ggtgatgccccattccttgatcggctccgccgagatca aaagtccttaaaaggaagaggcaacaccc	-	*
CY123450	Human H1N1pdm2009 IAVs	Human	H1N1	pdm	2009	Singapore	A/Singapore/ON1114/2009	ggtgatgccccattccttgatcggctccgccgagatca aaagtccttaaaaggaagaggcaacaccc	-	*
CY123498	Human H1N1pdm2009 IAVs	Human	H1N1	pdm	2009	Singapore	A/Singapore/ON1747/2009	ggtgatgccccattccttgatcggctccgccgagatca aaagtccttaaaaggaagaggcaacaccc	-	*
CY123536	Human H1N1pdm2009 IAVs	Human	H1N1	pdm	2009	Singapore	A/Singapore/ON1808/2009	ggtgatgccccattccttgatcggctccgccgagatca aaagtccttaaaaggaagaggcaacaccc	-	*
CY123570	Human H1N1pdm2009 IAVs	Human	H1N1	pdm	2009	Singapore	A/Singapore/ON1858/2009	ggtgatgccccattccttgatcggctccgccgagatca aaagtccttaaaaggaagaggcaacaccc	-	*

CY123588	Human H1N1pdm2009 IAVs	Human	H1N1	pdm	2009	Singapore	A/Singapore/ON1892/2009	ggtgatgccccattccttgatcggctccgccgagatca aaagtccttaaaaggaagaggcaacaccc	-	*
CY123596	Human H1N1pdm2009 IAVs	Human	H1N1	pdm	2009	Singapore	A/Singapore/ON1896/2009	ggtgatgccccattccttgatcggctccgccgagatca aaagtccttaaaaggaagaggcaacaccc	-	*
CY123604	Human H1N1pdm2009 IAVs	Human	H1N1	pdm	2009	Singapore	A/Singapore/ON1900/2009	ggtgatgccccattccttgatcggctccgccgagatca aaagtccttaaaaggaagaggcaacaccc	-	*
CY123628	Human H1N1pdm2009 IAVs	Human	H1N1	pdm	2009	Singapore	A/Singapore/ON1914/2009	ggtgatgccccattccttgatcggctccgccgagatca aaagtccttaaaaggaagaggcaacaccc	-	*
CY123644	Human H1N1pdm2009 IAVs	Human	H1N1	pdm	2009	Singapore	A/Singapore/ON1924/2009	ggtgatgccccattccttgatcggctccgccgagatca aaagtccttaaaaggaagaggcaacaccc	-	*
CY123671	Human H1N1pdm2009 IAVs	Human	H1N1	pdm	2009	Singapore	A/Singapore/ON1943/2009	ggtgatgccccattccttgatcggctccgccgagatca aaagtccttaaaaggaagaggcaacaccc	-	*
CY123695	Human H1N1pdm2009 IAVs	Human	H1N1	pdm	2009	Singapore	A/Singapore/ON199/2009	ggtgatgccccattccttgatcggctccgccgagatca aaagtccttaaaaggaagaggcaacaccc	-	*
CY123713	Human H1N1pdm2009 IAVs	Human	H1N1	pdm	2009	Singapore	A/Singapore/ON201/2009	ggtgatgccccattccttgatcggctccgccgagatca aaagtccttaaaaggaagaggcaacaccc	-	*
CY123749	Human H1N1pdm2009 IAVs	Human	H1N1	pdm	2009	Singapore	A/Singapore/ON2081/2009	ggtgatgccccattccttgatcggctccgccgagatca aaagtccttaaaaggaagaggcaacaccc	-	*
CY123813	Human H1N1pdm2009 IAVs	Human	H1N1	pdm	2009	Singapore	A/Singapore/ON2224/2009	ggtgatgccccattccttgatcggctccgccgagatca aaagtccttaaaaggaagaggcaacaccc	-	*
CY123853	Human H1N1pdm2009 IAVs	Human	H1N1	pdm	2009	Singapore	A/Singapore/ON2313/2009	ggtgatgccccattccttgatcggctccgccgagatca aaagtccttaaaaggaagaggcaacaccc	-	*
CY123883	Human H1N1pdm2009 IAVs	Human	H1N1	pdm	2009	Singapore	A/Singapore/ON2383/2009	ggtgatgccccattccttgatcggctccgccgagatca aaagtccttaaaaggaagaggcaacaccc	-	*
CY123925	Human H1N1pdm2009 IAVs	Human	H1N1	pdm	2009	Singapore	A/Singapore/ON313/2009	ggtgatgccccattccttgatcggctccgccgagatca aaagtccttaaaaggaagaggcaacaccc	-	*
CY123957	Human H1N1pdm2009 IAVs	Human	H1N1	pdm	2009	Singapore	A/Singapore/ON392/2009	ggtgatgccccattccttgatcggctccgccgagatca aaagtccttaaaaggaagaggcaacaccc	-	*
CY123984	Human H1N1pdm2009 IAVs	Human	H1N1	pdm	2009	Singapore	A/Singapore/ON477/2009	ggtgatgccccattccttgatcggctccgccgagatca aaagtccttaaaaggaagaggcaacaccc	-	*
CY123992	Human H1N1pdm2009 IAVs	Human	H1N1		2009	Singapore	A/Singapore/ON479/2009	ggtgatgccccattccttgatcggctccgccgagatca aaagtccttaaaaggaagaggcaacaccc	-	*
CY124000	Human H1N1pdm2009 IAVs	Human	H1N1	pdm	2009	Singapore	A/Singapore/ON496/2009	ggtgatgccccattccttgatcggctccgccgagatca aaagtccttaaaaggaagaggcaacaccc	-	*
CY041094	Human H1N1pdm2009 IAVs	Human	H1N1	pdm	2009	USA	A/New_York/3173/2009	ggtgatgccccattccttgatcggctccgccgagatca aaagtccttaaaaggaagaggcaacaccc	-	*
CY124008	Human H1N1pdm2009 IAVs	Human	H1N1	pdm	2009	Singapore	A/Singapore/ON506/2009	ggtgatgccccattccttgatcggctccgccgagatca aaagtccttaaaaggaagaggcaacaccc	-	*
CY124071	Human H1N1pdm2009 IAVs	Human	H1N1	pdm	2009	Singapore	A/Singapore/ON803/2009	ggtgatgccccattccttgatcggctccgccgagatca aaagtccttaaaaggaagaggcaacaccc	-	*
CY124083	Human H1N1pdm2009 IAVs	Human	H1N1	pdm	2009	Singapore	A/Singapore/ON805/2009	ggtgatgccccattccttgatcggctccgccgagatca aaagtccttaaaaggaagaggcaacaccc	-	*
CY124099	Human H1N1pdm2009 IAVs	Human	H1N1	pdm	2009	Singapore	A/Singapore/ON807/2009	ggtgatgccccattccttgatcggctccgccgagatca aaagtccttaaaaggaagaggcaacaccc	-	*
CY124103	Human H1N1pdm2009 IAVs	Human	H1N1	pdm	2009	Singapore	A/Singapore/ON808/2009	ggtgatgccccattccttgatcggctccgccgagatca aaagtccttaaaaggaagaggcaacaccc	-	*
CY124111	Human H1N1pdm2009 IAVs	Human	H1N1	pdm	2009	Singapore	A/Singapore/ON809/2009	ggtgatgccccattccttgatcggctccgccgagatca aaagtccttaaaaggaagaggcaacaccc	-	*
CY124150	Human H1N1pdm2009 IAVs	Human	H1N1	pdm	2009	Singapore	A/Singapore/ON822/2009	ggtgatgccccattccttgatcggctccgccgagatca aaagtccttaaaaggaagaggcaacaccc	-	*

CY045934	Human H1N1pdm2009 IAVs	Human	H1N1	pdm	2009	Canada	A/Toronto/3184/2009	ggtgatgccccattccttgatcggctccgccgagatca aaagtccttaaaaggaagaggcaacaccc	-	*
JX625439	Human H1N1pdm2009 IAVs	Human	H1N1	pdm	2009	United_Kingdom	A/England/92960004/2009	ggtgatgccccattccttgatcggctccgccgagatca aaagtccttaaaaggaagaggcaacaccc	-	*
JN617978	Human H1N1pdm2009 IAVs	Swine	H1N1		2009	Canada	A/swine/Alberta/25/2009	ggtgatgccccattccttgatcggctccgccgagatca aaagtccttaaaaggaagaggcaacaccc	-	*
GU136023	Human H1N1pdm2009 IAVs	Human	H1N1	pdm	2009	Japan	A/Yamaguchi/21/2009	ggtgatgccccattccttgatcggctccgccgagatca aaagtccttaaaaggaagaggcaacaccc	-	*
GU136024	Human H1N1pdm2009 IAVs	Human	H1N1	pdm	2009	Japan	A/Yamaguchi/22/2009	ggtgatgccccattccttgatcggctccgccgagatca aaagtccttaaaaggaagaggcaacaccc	-	*
GQ324580	Human H1N1pdm2009 IAVs	Human	H1N1	pdm	2009	Japan	A/Yokohama/1/2009	ggtgatgccccattccttgatcggctccgccgagatca aaagtccttaaaaggaagaggcaacaccc	-	*
HQ840294	Human H1N1pdm2009 IAVs	Swine	H1N1		2009	USA	A/swine/Minnesota/074A/2009	ggtgatgccccattccttgatcggctccgccgagatca aaagtccttaaaaggaagaggcaacaccc	-	*
HQ840308	Human H1N1pdm2009 IAVs	Swine	H1N1		2009	USA	A/swine/Minnesota/130A/2009	ggtgatgccccattccttgatcggctccgccgagatca aaagtccttaaaaggaagaggcaacaccc	-	*
HQ840313	Human H1N1pdm2009 IAVs	Swine	H1N1		2009	USA	A/swine/Minnesota/136B/2009	ggtgatgccccattccttgatcggctccgccgagatca aaagtccttaaaaggaagaggcaacaccc	-	*
HQ840321	Human H1N1pdm2009 IAVs	Swine	H1N1		2009	USA	A/swine/Minnesota/165A/2009	ggtgatgccccattccttgatcggctccgccgagatca aaagtccttaaaaggaagaggcaacaccc	-	*
CY045230	Human H1N1pdm2009 IAVs	Human	H1N1	pdm	2009	Taiwan	A/Taiwan/115/2009	ggtgatgccccattccttgatcggctccgccgagatca aaagtccttaaaaggaagaggcaacaccc	-	*
KC780987	Human H1N1pdm2009 IAVs	Human	H1N1	pdm	2009	USA	A/Texas/66/2009	ggtgatgccccattccttgatcggctccgccgagatca aaagtccttaaaaggaagaggcaacaccc	-	*
KC780873	Human H1N1pdm2009 IAVs	Human	H1N1	pdm	2009	USA	A/Texas/67/2009	ggtgatgccccattccttgatcggctccgccgagatca aaagtccttaaaaggaagaggcaacaccc	-	*
GQ457459	Human H1N1pdm2009 IAVs	Human	H1N1	pdm	2009	USA	A/Utah/05/2009	ggtgatgccccattccttgatcggctccgccgagatca aaagtccttaaaaggaagaggcaacaccc	-	*
GQ457476	Human H1N1pdm2009 IAVs	Human	H1N1	pdm	2009	USA	A/Utah/07/2009	ggtgatgccccattccttgatcggctccgccgagatca aaagtccttaaaaggaagaggcaacaccc	-	*
KC781889	Human H1N1pdm2009 IAVs	Human	H1N1	pdm	2009	USA	A/Utah/21/2009	ggtgatgccccattccttgatcggctccgccgagatca aaagtccttaaaaggaagaggcaacaccc	-	*
KC781504	Human H1N1pdm2009 IAVs	Human	H1N1	pdm	2009	USA	A/Utah/22/2009	ggtgatgccccattccttgatcggctccgccgagatca aaagtccttaaaaggaagaggcaacaccc	-	*
KC781436	Human H1N1pdm2009 IAVs	Human	H1N1	pdm	2009	USA	A/Utah/27/2009	ggtgatgccccattccttgatcggctccgccgagatca aaagtccttaaaaggaagaggcaacaccc	-	*
KC781925	Human H1N1pdm2009 IAVs	Human	H1N1	pdm	2009	USA	A/Virginia/24/2009	ggtgatgccccattccttgatcggctccgccgagatca aaagtccttaaaaggaagaggcaacaccc	-	*
KC780379	Human H1N1pdm2009 IAVs	Human	H1N1	pdm	2009	USA	A/Washington/62/2009	ggtgatgccccattccttgatcggctccgccgagatca aaagtccttaaaaggaagaggcaacaccc	-	*
KC782311	Human H1N1pdm2009 IAVs	Human	H1N1	pdm	2009	USA	A/Wisconsin/53/2009	ggtgatgccccattccttgatcggctccgccgagatca aaagtccttaaaaggaagaggcaacaccc	-	*
KC782058	Human H1N1pdm2009 IAVs	Human	H1N1	pdm	2009	USA	A/Wisconsin/55/2009	ggtgatgccccattccttgatcggctccgccgagatca aaagtccttaaaaggaagaggcaacaccc	-	*
KC781737	Human H1N1pdm2009 IAVs	Human	H1N1	pdm	2009	USA	A/Wyoming/08/2009	ggtgatgccccattccttgatcggctccgccgagatca aaagtccttaaaaggaagaggcaacaccc	-	*
CY095931	Human H1N1pdm2009 IAVs	Human	H1N1	pdm	2009	China	A/Zhejiang/82/2009	ggtgatgccccattccttgatcggctccgccgagatca aaagtccttaaaaggaagaggcaacaccc	-	*
CY056008	Human H1N1pdm2009 IAVs	Human	H1N1	pdm	2009	USA	A/San_Diego/INS10/2009	ggtgatgccccattccttgatcggctccgccgagatca aaagtccttaaaaggaagaggcaacaccc	-	*

GQ365367	Human H1N1pdm2009 IAVs	Human	H1N1	pdm	2009	Sweden	A/Stockholm/36/2009	ggtgatgccccattccttgatcggctccgccgagatca aaagtccttaaaaggaagaggcaacaccc	-	*
GQ365364	Human H1N1pdm2009 IAVs	Human	H1N1	pdm	2009	Sweden	A/Stockholm/42/2009	ggtgatgccccattccttgatcggctccgccgagatca aaagtccttaaaaggaagaggcaacaccc	-	*
KJ690530	Human H1N1pdm2009 IAVs	Human	H1N1	pdm	2009	Uganda	A/Uganda/MUWRP_137/2009	ggtgatgccccattccttgatcggctccgccgagatca aaagtccttaaaaggaagaggcaacaccc	-	*
GU108490	Human H1N1pdm2009 IAVs	Human	H1N1	pdm	2009	China	A/Zhejiang_Yiwu/11/2009	ggtgatgccccattccttgatcggctccgccgagatca aaagtccttaaaaggaagaggcaacaccc	-	*
CY066371	Human H1N1pdm2009 IAVs	Human	H1N1	pdm	2009	USA	A/California/VRDL124/2009	ggtgatgccccattccttgatcggctccgccgagatca aaagtccttaaaaggaagaggcaacaccc	-	*
GQ223439	Human H1N1pdm2009 IAVs	Human	H1N1	pdm	2009	Finland	A/Finland/553/2009	ggtgatgccccattccttgatcggctccgccgagatca aaagtccttaaaaggaagaggcaacaccc	-	*
HM855243	Human H1N1pdm2009 IAVs	Human	H1N1	pdm	2009	Kenya	A/Keiyo/96/2009	ggtgatgccccattccttgatcggctccgccgagatca aaagtccttaaaaggaagaggcaacaccc	-	*
GQ375288	Human H1N1pdm2009 IAVs	Human	H1N1	pdm	2009	Russia	A/Moscow/03/2009	ggtgatgccccattccttgatcggctccgccgagatca aaagtccttaaaaggaagaggcaacaccc	-	*
CY048937	Human H1N1pdm2009 IAVs	Human	H1N1	pdm	2009	Malaysia	A/Malaysia/854/2009	ggtgatgccccattccttgatcggctccgccgagatca aaagtccttaaaaggaagaggcaacaccc	-	*
HM855251	Human H1N1pdm2009 IAVs	Human	H1N1	pdm	2009	Kenya	A/Meru/456/2009	ggtgatgccccattccttgatcggctccgccgagatca aaagtccttaaaaggaagaggcaacaccc	-	*
CY041182	Human H1N1pdm2009 IAVs	Human	H1N1		2009	USA	A/New_York/3187/2009	ggtgatgccccattccttgatcggctccgccgagatca aaagtccttaaaaggaagaggcaacaccc	-	*
CY060530	Human H1N1pdm2009 IAVs	Human	H1N1	pdm	2009	Canada	A/Ontario/235657/2009	ggtgatgccccattccttgatcggctccgccgagatca aaagtccttaaaaggaagaggcaacaccc	-	*
CY086952	Human H1N1pdm2009 IAVs	Swine	H1N1		2009	Thailand	A/swine/Thailand/CU_M8_2/2009	ggtgatgccccattccttgatcggctccgccgagatca aaagtccttaaaaggaagaggcaacaccc	-	*
CY045246	Human H1N1pdm2009 IAVs	Human	H1N1	pdm	2009	Taiwan	A/Taiwan/137/2009	ggtgatgccccattccttgatcggctccgccgagatca aaagtccttaaaaggaagaggcaacaccc	-	*
CY083379	Human H1N1pdm2009 IAVs	Human	H1N1	pdm	2009	USA	A/Pendleton/WRAIR1661P/2009	ggtgatgccccattccttgatcggctccgccgagatca aaagtccttaaaaggaagaggcaacaccc	-	*
CY083348	Human H1N1pdm2009 IAVs	Human	H1N1	pdm	2009	USA	A/San_Diego/WRAIR1656P/2009	ggtgatgccccattccttgatcggctccgccgagatca aaagtccttaaaaggaagaggcaacaccc	-	*
CY083411	Human H1N1pdm2009 IAVs	Human	H1N1	pdm	2009	USA	A/San_Diego/WRAIR1665P/2009	ggtgatgccccattccttgatcggctccgccgagatca aaagtccttaaaaggaagaggcaacaccc	-	*
CY083188	Human H1N1pdm2009 IAVs	Human	H1N1	pdm	2009	USA	A/South_Carolina/WRAIR1501P/2 009	ggtgatgccccattccttgatcggctccgccgagatca aaagtccttaaaaggaagaggcaacaccc	-	*
CY083371	Human H1N1pdm2009 IAVs	Human	H1N1	pdm	2009	USA	A/South_Carolina/WRAIR1660P/2 009	ggtgatgccccattccttgatcggctccgccgagatca aaagtccttaaaaggaagaggcaacaccc	-	*
CY122983	Human H1N1pdm2009 IAVs	Human	H1N1	pdm	2009	Singapore	A/Singapore/GP3009/2009	ggtgatgccccattccttgatcggctccgccgagatca aaagtccttaaaaggaagaggcaacaccc	-	*
CY123458	Human H1N1pdm2009 IAVs	Human	H1N1	pdm	2009	Singapore	A/Singapore/ON1121/2009	ggtgatgccccattccttgatcggctccgccgagatca aaagtccttaaaaggaagaggcaacaccc	-	*
JF327344	Human H1N1pdm2009 IAVs	Human	H1N1	pdm	2009	Finland	A/Finland/671/2009	ggtgatgccccattccttgatcggctccgccgagatca aaagtccttaaaaggaagaggcaacaccc	-	*
GQ168868	Human H1N1pdm2009 IAVs	Human	H1N1	pdm	2009	USA	A/New_York/20/2009	ggtgatgccccattccttgatcggctccgccgagatca aaagtccttaaaaggaagaggcaacaccc	-	*
CY044151	Human H1N1pdm2009 IAVs	Human	H1N1	pdm	2009	Colombia	A/Bogota/0466N/2009	ggtgatgccccattccttgatcggctccgccgagatca aaagtccttaaaaggaagaggcaacaccc	-	*
CY073258	Human H1N1pdm2009 IAVs	Human	H1N1	pdm	2009	Greece	A/Athens/WRAIR2962N/2009	ggtgatgccccattccttgatcggctccgccgagatca aaagtccttaaaaggaagaggcaacaccc	-	*

CY071478	Human H1N1pdm2009 IAVs	Human	H1N1	pdm	2009	USA	A/California/WR1313P/2009	ggtgatgccccattccttgatcggctccgccgagatca aaagtccttaaaaggaagaggcaacaccc	-	*
CY071550	Human H1N1pdm2009 IAVs	Human	H1N1	pdm	2009	USA	A/California/WR1323P/2009	ggtgatgccccattccttgatcggctccgccgagatca aaagtccttaaaaggaagaggcaacaccc	-	*
CY071582	Human H1N1pdm2009 IAVs	Human	H1N1		2009	USA	A/South_Carolina/WR1326P/2009	ggtgatgccccattccttgatcggctccgccgagatca aaagtccttaaaaggaagaggcaacaccc	-	*
CY071526	Human H1N1pdm2009 IAVs	Human	H1N1	pdm	2009	USA	A/California/WR1319P/2009	ggtgatgccccattccttgatcggctccgccgagatca aaagtccttaaaaggaagaggcaacaccc	-	*
CY071790	Human H1N1pdm2009 IAVs	Human	H1N1	pdm	2009	Mexico	A/Mexico_City/WR1765N/2009	ggtgatgccccattccttgatcggctccgccgagatca aaagtccttaaaaggaagaggcaacaccc	-	*
CY071566	Human H1N1pdm2009 IAVs	Human	H1N1	pdm	2009	USA	A/South_Carolina/WR1324P/2009	ggtgatgccccattccttgatcggctccgccgagatca aaagtccttaaaaggaagaggcaacaccc	-	*
CY049895	Human H1N1pdm2009 IAVs	Human	H1N1	pdm	2009	El_Salvador	A/San_Salvador/0169T/2009	ggtgatgccccattccttgatcggctccgccgagatca aaagtccttaaaaggaagaggcaacaccc	-	*
CY044200	Human H1N1pdm2009 IAVs	Human	H1N1	pdm	2009	Taiwan	A/Taiwan/T0724/2009	ggtgatgccccattccttgatcggctccgccgagatca aaagtccttaaaaggaagaggcaacaccc	-	*
CY044232	Human H1N1pdm2009 IAVs	Human	H1N1		2009	Taiwan	A/Taiwan/T1821/2009	ggtgatgccccattccttgatcggctccgccgagatca aaagtccttaaaaggaagaggcaacaccc	-	*
CY044216	Human H1N1pdm2009 IAVs	Human	H1N1	pdm	2009	Taiwan	A/Taiwan/T1339/2009	ggtgatgccccattccttgatcggctccgccgagatca aaagtccttaaaaggaagaggcaacaccc	-	*
HM189502	Human H1N1pdm2009 IAVs	Human	H1N1		2009	South_Korea	A/Korea/CJ04/2009	ggtgatgccccattccttgatcggctccgccgagatca aaagtccttaaaaggaagaggcaacaccc	-	*
CY062501	Human H1N1pdm2009 IAVs	Human	H1N1	pdm	2009	Mexico	A/Mexico_city/CIA2/2009	ggtgatgccccattccttgatcggctccgccgagatca aaagtccttaaaaggaagaggcaacaccc	-	*
CY054879	Human H1N1pdm2009 IAVs	Human	H1N1	pdm	2009	USA	A/California/VRDL35/2009	ggtgatgccccattccttgatcggctccgccgagatca aaagtccttaaaaggaagaggcaacaccc	-	*
CY107386	Human H1N1pdm2009 IAVs	Human	H1N1	pdm	2009	United_Kingdom	A/Scotland/Dundee_09V500624/2009	ggtgatgccccattccttgatcggctccgccgagatca aaagtccttaaaaggaagaggcaacaccc	-	*
CY055657	Human H1N1pdm2009 IAVs	Human	H1N1	pdm	2009	Australia	A/Australia/27/2009	ggtgatgccccattccttgatcggctccgccgagatca aaagtccttaaaaggaagaggcaacaccc	-	*
CY055912	Human H1N1pdm2009 IAVs	Human	H1N1		2009	Australia	A/Australia/73/2009	ggtgatgccccattccttgatcggctccgccgagatca aaagtccttaaaaggaagaggcaacaccc	-	*
GQ351294	Human H1N1pdm2009 IAVs	Human	H1N1	pdm	2009	Italy	A/Italy/85/2009	ggtgatgccccattccttgatcggctccgccgagatca aaagtccttaaaaggaagaggcaacaccc	-	*
CY073597	Human H1N1pdm2009 IAVs	Human	H1N1	pdm	2009	Nicaragua	A/Managua/5295.02/2009	ggtgatgccccattccttgatcggctccgccgagatca aaagtccttaaaaggaagaggcaacaccc	-	*
CY043255	Human H1N1pdm2009 IAVs	Human	H1N1	pdm	2009	USA	A/New_York/3545/2009	ggtgatgccccattccttgatcggctccgccgagatca aaagtccttaaaaggaagaggcaacaccc	-	*
CY052102	Human H1N1pdm2009 IAVs	Human	H1N1		2009	USA	A/New_York/4238/2009	ggtgatgccccattccttgatcggctccgccgagatca aaagtccttaaaaggaagaggcaacaccc	-	*
CY051627	Human H1N1pdm2009 IAVs	Human	H1N1	pdm	2009	USA	A/New_York/4607/2009	ggtgatgccccattccttgatcggctccgccgagatca aaagtccttaaaaggaagaggcaacaccc	-	*
CY057242	Human H1N1pdm2009 IAVs	Human	H1N1		2009	USA	A/New_York/5158/2009	ggtgatgccccattccttgatcggctccgccgagatca aaagtccttaaaaggaagaggcaacaccc	-	*
CY056831	Human H1N1pdm2009 IAVs	Human	H1N1	pdm	2009	USA	A/New_York/6846/2009	ggtgatgccccattccttgatcggctccgccgagatca aaagtccttaaaaggaagaggcaacaccc	-	*
CY071027	Human H1N1pdm2009 IAVs	Human	H1N1	pdm	2009	USA	A/New_York/NHRC0001/2009	ggtgatgccccattccttgatcggctccgccgagatca aaagtccttaaaaggaagaggcaacaccc	-	*
CY107569	Human H1N1pdm2009 IAVs	Human	H1N1	pdm	2009	United_Kingdom	A/Scotland/Glasgow_412286/2009	ggtgatgccccattccttgatcggctccgccgagatca aaagtccttaaaaggaagaggcaacaccc	-	*

CY107551	Human H1N1pdm2009 IAVs	Human	H1N1	pdm	2009	United_Kingdom	A/Scotland/Livingston_412757/2009	ggtgatgccccattccttgatcggctccgccgagatca aaagtccttaaaaggaagaggcaacaccc	-	*
CY107618	Human H1N1pdm2009 IAVs	Human	H1N1	pdm	2009	United_Kingdom	A/Scotland/Paisley_413739/2009	ggtgatgccccattccttgatcggctccgccgagatca aaagtccttaaaaggaagaggcaacaccc	-	*
CY052787	Human H1N1pdm2009 IAVs	Human	H1N1	pdm	2009	USA	A/Texas/45043852/2009	ggtgatgccccattccttgatcggctccgccgagatca aaagtccttaaaaggaagaggcaacaccc	-	*
CY052723	Human H1N1pdm2009 IAVs	Human	H1N1	pdm	2009	USA	A/Texas/45091402/2009	ggtgatgccccattccttgatcggctccgccgagatca aaagtccttaaaaggaagaggcaacaccc	-	*
CY052731	Human H1N1pdm2009 IAVs	Human	H1N1	pdm	2009	USA	A/Texas/45091405/2009	ggtgatgccccattccttgatcggctccgccgagatca aaagtccttaaaaggaagaggcaacaccc	-	*
CY052739	Human H1N1pdm2009 IAVs	Human	H1N1	pdm	2009	USA	A/Texas/45091417/2009	ggtgatgccccattccttgatcggctccgccgagatca aaagtccttaaaaggaagaggcaacaccc	-	*
CY052763	Human H1N1pdm2009 IAVs	Human	H1N1	pdm	2009	USA	A/Texas/45101422/2009	ggtgatgccccattccttgatcggctccgccgagatca aaagtccttaaaaggaagaggcaacaccc	-	*
CY052755	Human H1N1pdm2009 IAVs	Human	H1N1	pdm	2009	USA	A/Texas/45103759/2009	ggtgatgccccattccttgatcggctccgccgagatca aaagtccttaaaaggaagaggcaacaccc	-	*
CY052803	Human H1N1pdm2009 IAVs	Human	H1N1	pdm	2009	USA	A/Texas/45131576/2009	ggtgatgccccattccttgatcggctccgccgagatca aaagtccttaaaaggaagaggcaacaccc	-	*
CY058042	Human H1N1pdm2009 IAVs	Human	H1N1		2009	USA	A/Texas/46172731/2009	ggtgatgccccattccttgatcggctccgccgagatca aaagtccttaaaaggaagaggcaacaccc	-	*
CY060775	Human H1N1pdm2009 IAVs	Human	H1N1	pdm	2009	USA	A/Texas/46214103/2009	ggtgatgccccattccttgatcggctccgccgagatca aaagtccttaaaaggaagaggcaacaccc	-	*
CY060903	Human H1N1pdm2009 IAVs	Human	H1N1	pdm	2009	USA	A/Texas/JMS365/2009	ggtgatgccccattccttgatcggctccgccgagatca aaagtccttaaaaggaagaggcaacaccc	-	*
AB704506	Human H1N1pdm2009 IAVs	Human	H1N1	pdm	2009	Japan	A/Yamagata/674/2009	ggtgatgccccattccttgatcggctccgccgagatca aaagtccttaaaaggaagaggcaacaccc	-	*
HM567628	Human H1N1pdm2009 IAVs	Human	H1N1	pdm	2009	United_Kingdom	A/England/341/2009	ggtgatgccccattccttgatcggctccgccgagatca aaagtccttaaaaggaagaggcaacaccc	-	*
HM567660	Human H1N1pdm2009 IAVs	Human	H1N1	pdm	2009	United_Kingdom	A/England/346/2009	ggtgatgccccattccttgatcggctccgccgagatca aaagtccttaaaaggaagaggcaacaccc	-	*
HM567596	Human H1N1pdm2009 IAVs	Human	H1N1	pdm	2009	Ireland	A/Ireland/1/2009	ggtgatgccccattccttgatcggctccgccgagatca aaagtccttaaaaggaagaggcaacaccc	-	*
HM567716	Human H1N1pdm2009 IAVs	Human	H1N1	pdm	2009	United_Kingdom	A/Scotland/8/2009	ggtgatgccccattccttgatcggctccgccgagatca aaagtccttaaaaggaagaggcaacaccc	-	*
HM014328	Human H1N1pdm2009 IAVs	Human	H1N1	pdm	2009	China	A/Guangzhou/GIRD07/2009	ggtgatgccccattccttgatcggctccgccgagatca aaagtccttaaaaggaagaggcaacaccc	-	*
KC781987	Human H1N1pdm2009 IAVs	Human	H1N1	pdm	2009	USA	A/Kansas/22/2009	ggtgatgccccattccttgatcggctccgccgagatca aaagtccttaaaaggaagaggcaacaccc	-	*
KC781708	Human H1N1pdm2009 IAVs	Human	H1N1	pdm	2009	USA	A/New_York/98/2009	ggtgatgccccattccttgatcggctccgccgagatca aaagtccttaaaaggaagaggcaacaccc	-	*
KC782093	Human H1N1pdm2009 IAVs	Human	H1N1	pdm	2009	USA	A/Rhode_Island/15/2009	ggtgatgccccattccttgatcggctccgccgagatca aaagtccttaaaaggaagaggcaacaccc	-	*
CY071502	Human H1N1pdm2009 IAVs	Human	H1N1		2009	USA	A/California/WR1316P/2009	ggtgatgccccattccttgatcggctccgccgagatca aaagtccttaaaaggaagaggcaacaccc	-	*
CY071518	Human H1N1pdm2009 IAVs	Human	H1N1	pdm	2009	USA	A/California/WR1318P/2009	ggtgatgccccattccttgatcggctccgccgagatca aaagtccttaaaaggaagaggcaacaccc	-	*
CY107197	Human H1N1pdm2009 IAVs	Human	H1N1	pdm	2009	United_Kingdom	A/Scotland/Paisley_417557/2009	ggtgatgccccattccttgatcggctccgccgagatca aaagtccttaaaaggaagaggcaacaccc	-	*
CY066795	Human H1N1pdm2009 IAVs	Human	H1N1	pdm	2009	USA	A/San_Diego/INS215/2009	ggtgatgccccattccttgatcggctccgccgagatca aaagtccttaaaaggaagaggcaacaccc	-	*

CY065796	Human H1N1pdm2009 IAVs	Human	H1N1	pdm	2009	Netherlands	A/Netherlands/1039/2009	ggtgatgccccattccttgatcggctccgccgagatca<br>aaagtccttaaaaggaagaggcaacaccc	-	*
CY051571	Human H1N1pdm2009 IAVs	Human	H1N1	pdm	2009	USA	A/New_York/4476/2009	ggtgatgccccattccttgatcggctccgccgagatca<br>aaagtccttaaaaggaagaggcaacaccc	-	*
CY052150	Human H1N1pdm2009 IAVs	Human	H1N1	pdm	2009	USA	A/New_York/4398/2009	ggtgatgccccattccttgatcggctccgccgagatca<br>aaagtccttaaaaggaagaggcaacaccc	-	*
CY089612	Human H1N1pdm2009 IAVs	Human	H1N1		2009	China	A/Nanchang/8002/2009	ggtgatgccccattccttgatcggctccgccgagatca<br>aaagtccttaaaaggaagaggcaacaccc	-	*
HM230705	Human H1N1pdm2009 IAVs	Human	H1N1	pdm	2009	China	A/Zhoushan/52/2009	ggtgatgccccattccttgatcggctccgccgagatca<br>aaagtccttaaaaggaagaggcaacaccc	-	*
CY107544	Human H1N1pdm2009 IAVs	Human	H1N1	pdm	2009	United_Kingdom	A/Scotland/Fife_17690/2009	ggtgatgccccattccttgatcggctccgccgagatca<br>aaagtccttaaaaggaagaggcaacaccc	-	*
AB704498	Human H1N1pdm2009 IAVs	Human	H1N1	pdm	2009	Japan	A/Yamagata/473/2009	ggtgatgccccattccttgatcggctccgccgagatca<br>aaagtccttaaaaggaagaggcaacaccc	-	*
CY063626	Human H1N1pdm2009 IAVs	Human	H1N1	pdm	2009	USA	A/New_York/INS150/2009	ggtgatgccccattccttgatcggctccgccgagatca<br>aaagtccttaaaaggaagaggcaacaccc	-	*
KC780299	Human H1N1pdm2009 IAVs	Human	H1N1	pdm	2009	USA	A/Pennsylvania/31/2009	ggtgatgccccattccttgatcggctccgccgagatca<br>aaagtccttaaaaggaagaggcaacaccc	-	*
CY096015	Human H1N1pdm2009 IAVs	Human	H1N1	pdm	2009	China	A/Zhejiang/94/2009	ggtgatgccccattccttgatcggctccgccgagatca<br>aaagtccttaaaaggaagaggcaacaccc	-	*
CY064899	Human H1N1pdm2009 IAVs	Human	H1N1	pdm	2009	USA	A/Boston/136/2009	ggtgatgccccattccttgatcggctccgccgagatca<br>aaagtccttaaaaggaagaggcaacaccc	-	*
CY063570	Human H1N1pdm2009 IAVs	Human	H1N1	pdm	2009	USA	A/Boston/153/2009	ggtgatgccccattccttgatcggctccgccgagatca<br>aaagtccttaaaaggaagaggcaacaccc	-	*
CY043231	Human H1N1pdm2009 IAVs	Human	H1N1	pdm	2009	USA	A/New_York/3501/2009	ggtgatgccccattccttgatcggctccgccgagatca<br>aaagtccttaaaaggaagaggcaacaccc	-	*
CY057986	Human H1N1pdm2009 IAVs	Human	H1N1	pdm	2009	USA	A/Wisconsin/629_D01412/2009	ggtgatgccccattccttgatcggctccgccgagatca<br>aaagtccttaaaaggaagaggcaacaccc	-	*
CY057978	Human H1N1pdm2009 IAVs	Human	H1N1	pdm	2009	USA	A/Wisconsin/629_D01434/2009	ggtgatgccccattccttgatcggctccgccgagatca<br>aaagtccttaaaaggaagaggcaacaccc	-	*
CY054991	Human H1N1pdm2009 IAVs	Human	H1N1	pdm	2009	USA	A/California/VRDL58/2009	ggtgatgccccattccttgatcggctccgccgagatca<br>aaagtccttaaaaggaagaggcaacaccc	-	*
CY055593	Human H1N1pdm2009 IAVs	Human	H1N1	pdm	2009	Australia	A/Australia/15/2009	ggtgatgccccattccttgatcggctccgccgagatca<br>aaagtccttaaaaggaagaggcaacaccc	-	*
CY055752	Human H1N1pdm2009 IAVs	Human	H1N1		2009	Australia	A/Australia/44/2009	ggtgatgccccattccttgatcggctccgccgagatca<br>aaagtccttaaaaggaagaggcaacaccc	-	*
CY075335	Human H1N1pdm2009 IAVs	Human	H1N1	pdm	2009	Chile	A/Chile/3349/2009	ggtgatgccccattccttgatcggctccgccgagatca<br>aaagtccttaaaaggaagaggcaacaccc	-	*
CY067639	Human H1N1pdm2009 IAVs	Human	H1N1	pdm	2009	Norway	A/Oslo/INS219/2009	ggtgatgccccattccttgatcggctccgccgagatca<br>aaagtccttaaaaggaagaggcaacaccc	-	*
CY066563	Human H1N1pdm2009 IAVs	Human	H1N1	pdm	2009	USA	A/San_Diego/INS199/2009	ggtgatgccccattccttgatcggctccgccgagatca<br>aaagtccttaaaaggaagaggcaacaccc	-	*
CY066819	Human H1N1pdm2009 IAVs	Human	H1N1	pdm	2009	USA	A/San_Diego/INS218/2009	ggtgatgccccattccttgatcggctccgccgagatca<br>aaagtccttaaaaggaagaggcaacaccc	-	*
CY122791	Human H1N1pdm2009 IAVs	Human	H1N1	pdm	2009	Singapore	A/Singapore/GP1358/2009	ggtgatgccccattccttgatcggctccgccgagatca<br>aaagtccttaaaaggaagaggcaacaccc	-	*
CY056687	Human H1N1pdm2009 IAVs	Human	H1N1	pdm	2009	USA	A/New_York/6257/2009	ggtgatgccccattccttgatcggctccgccgagatca<br>aaagtccttaaaaggaagaggcaacaccc	-	*
HM567860	Human H1N1pdm2009 IAVs	Human	H1N1	pdm	2009	United_Kingdom	A/England/415/2009	ggtgatgccccattccttgatcggctccgccgagatca<br>aaagtccttaaaaggaagaggcaacaccc	-	*

GU290059	Human H1N1pdm2009 IAVs	Human	H1N1	pdm	2009	Czech_Republic	A/Prague/196_81511/2009	ggtgatgccccattccttgatcggctccgccgagatca aaagtccttaaaaggaagaggcaacaccc	-	*
CY040834	Human H1N1pdm2009 IAVs	Human	H1N1	pdm	2009	USA	A/New_York/3261/2009	ggtgatgccccattccttgatcggctccgccgagatca aaagtccttaaaaggaagaggcaacaccc	-	*
CY123835	Human H1N1pdm2009 IAVs	Human	H1N1	pdm	2009	Singapore	A/Singapore/ON2269/2009	ggtgatgccccattccttgatcggctccgccgagatca aaagtccttaaaaggaagaggcaacaccc	-	*
JN187332	Human H1N1pdm2009 IAVs	Human	H1N1		2009	Taiwan	A/Taiwan/8575/2009	ggtgatgccccattccttgatcggctccgccgagatca aaagtccttaaaaggaagaggcaacaccc	-	*
CY071115	Human H1N1pdm2009 IAVs	Human	H1N1	pdm	2009	Germany	A/Wurzburg/INS381/2009	ggtgatgccccattccttgatcggctccgccgagatca aaagtccttaaaaggaagaggcaacaccc	-	*
GU136018	Human H1N1pdm2009 IAVs	Human	H1N1	pdm	2009	Japan	A/Niigata/749/2009	ggtgatgccccattccttgatcggctccgccgagatca aaagtccttaaaaggaagaggcaacaccc	-	*
GQ232089	Human H1N1pdm2009 IAVs	Human	H1N1	pdm	2009	China	A/Guangdong/02/2009	ggtgatgccccattccttgatcggctccgccgagatca aaagtccttaaaaggaagaggcaacaccc	-	*
GQ244325	Human H1N1pdm2009 IAVs	Human	H1N1	pdm	2009	China	A/Guangdong/02/2009	ggtgatgccccattccttgatcggctccgccgagatca aaagtccttaaaaggaagaggcaacaccc	-	*
GQ227549	Human H1N1pdm2009 IAVs	Human	H1N1	pdm	2009	China	A/Guangdong/03/2009	ggtgatgccccattccttgatcggctccgccgagatca aaagtccttaaaaggaagaggcaacaccc	-	*
HQ011422	Human H1N1pdm2009 IAVs	Human	H1N1		2009	China	A/Guangdong/45/2009	ggtgatgccccattccttgatcggctccgccgagatca aaagtccttaaaaggaagaggcaacaccc	-	*
CY067051	Human H1N1pdm2009 IAVs	Human	H1N1	pdm	2009	Denmark	A/Aarhus/INS253/2009	ggtgatgccccattccttgatcggctccgccgagatca aaagtccttaaaaggaagaggcaacaccc	-	*
CY062847	Human H1N1pdm2009 IAVs	Human	H1N1	pdm	2009	Greece	A/Athens/INS156/2009	ggtgatgccccattccttgatcggctccgccgagatca aaagtccttaaaaggaagaggcaacaccc	-	*
CY072362	Human H1N1pdm2009 IAVs	Human	H1N1		2009	Greece	A/Athens/INS325/2009	ggtgatgccccattccttgatcggctccgccgagatca aaagtccttaaaaggaagaggcaacaccc	-	*
CY065714	Human H1N1pdm2009 IAVs	Human	H1N1	pdm	2009	United_Kingdom	A/England/431/2009	ggtgatgccccattccttgatcggctccgccgagatca aaagtccttaaaaggaagaggcaacaccc	-	*
CY051043	Human H1N1pdm2009 IAVs	Human	H1N1	pdm	2009	USA	A/Wisconsin/629_D00725/2009	ggtgatgccccattccttgatcggctccgccgagatca aaagtccttaaaaggaagaggcaacaccc	-	*
CY041110	Human H1N1pdm2009 IAVs	Human	H1N1	pdm	2009	USA	A/New_York/3178/2009	ggtgatgccccattccttgatcggctccgccgagatca aaagtccttaaaaggaagaggcaacaccc	-	*
CY040762	Human H1N1pdm2009 IAVs	Human	H1N1		2009	USA	A/New_York/3244/2009	ggtgatgccccattccttgatcggctccgccgagatca aaagtccttaaaaggaagaggcaacaccc	-	*
CY056647	Human H1N1pdm2009 IAVs	Human	H1N1	pdm	2009	USA	A/New_York/6079/2009	ggtgatgccccattccttgatcggctccgccgagatca aaagtccttaaaaggaagaggcaacaccc	-	*
CY061215	Human H1N1pdm2009 IAVs	Human	H1N1	pdm	2009	USA	A/New_York/6771/2009	ggtgatgccccattccttgatcggctccgccgagatca aaagtccttaaaaggaagaggcaacaccc	-	*
GQ396561	Human H1N1pdm2009 IAVs	Human	H1N1	pdm	2009	Spain	A/Andalucia/GP286/2009	ggtgatgccccattccttgatcggctccgccgagatca aaagtccttaaaaggaagaggcaacaccc	-	*
GQ396549	Human H1N1pdm2009 IAVs	Human	H1N1	pdm	2009	Spain	A/Andalucia/GP327/2009	ggtgatgccccattccttgatcggctccgccgagatca aaagtccttaaaaggaagaggcaacaccc	-	*
GQ396533	Human H1N1pdm2009 IAVs	Human	H1N1	pdm	2009	Spain	A/CastillaLaMancha/GP369/2009	ggtgatgccccattccttgatcggctccgccgagatca aaagtccttaaaaggaagaggcaacaccc	-	*
GQ396588	Human H1N1pdm2009 IAVs	Human	H1N1	pdm	2009	Spain	A/Madrid/GP62/2009	ggtgatgccccattccttgatcggctccgccgagatca aaagtccttaaaaggaagaggcaacaccc	-	*
CY060642	Human H1N1pdm2009 IAVs	Human	H1N1		2009	Canada	A/Ontario/314137/2009	ggtgatgccccattccttgatcggctccgccgagatca aaagtccttaaaaggaagaggcaacaccc	-	*
CY060722	Human H1N1pdm2009 IAVs	Human	H1N1		2009	Canada	A/Ontario/320266/2009	ggtgatgccccattccttgatcggctccgccgagatca aaagtccttaaaaggaagaggcaacaccc	-	*

CY058664	Human H1N1pdm2009 IAVs	Human	H1N1	pdm	2009	USA	A/Wisconsin/629_D01913/2009	ggtgatgccccattccttgatcggctccgccgagatcaaaagtccttaaaaggaagaggcaacaccc	-	*
CY058648	Human H1N1pdm2009 IAVs	Human	H1N1	pdm	2009	USA	A/Wisconsin/629_D02329/2009	ggtgatgccccattccttgatcggctccgccgagatcaaaagtccttaaaaggaagaggcaacaccc	-	*
CY052467	Human H1N1pdm2009 IAVs	Human	H1N1	pdm	2009	USA	A/Texas/45121606/2009	ggtgatgccccattccttgatcggctccgccgagatcaaaagtccttaaaaggaagaggcaacaccc	-	*
CY128359	Human H1N1pdm2009 IAVs	Human	H1N1	pdm	2009	Viet_Nam	A/Viet_Nam/15032003/2009	ggtgatgccccattccttgatcggctccgccgagatcaaaagtccttaaaaggaagaggcaacaccc	-	*
CY083148	Human H1N1pdm2009 IAVs	Human	H1N1	pdm	2009	Kyrgyzstan	A/Bishkek/WRAIR0883N/2009	ggtgatgccccattccttgatcggctccgccgagatcaaaagtccttaaaaggaagaggcaacaccc	-	*
CY055475	Human H1N1pdm2009 IAVs	Human	H1N1	pdm	2009	USA	A/California/VRDL18/2009	ggtgatgccccattccttgatcggctccgccgagatcaaaagtccttaaaaggaagaggcaacaccc	-	*
HM567972	Human H1N1pdm2009 IAVs	Human	H1N1		2009	United_Kingdom	A/England/876/2009	ggtgatgccccattccttgatcggctccgccgagatcaaaagtccttaaaaggaagaggcaacaccc	-	*
HM568004	Human H1N1pdm2009 IAVs	Human	H1N1	pdm	2009	United_Kingdom	A/England/900/2009	ggtgatgccccattccttgatcggctccgccgagatcaaaagtccttaaaaggaagaggcaacaccc	-	*
HM567980	Human H1N1pdm2009 IAVs	Human	H1N1	pdm	2009	United_Kingdom	A/England/1111/2009	ggtgatgccccattccttgatcggctccgccgagatcaaaagtccttaaaaggaagaggcaacaccc	-	*
CY071131	Human H1N1pdm2009 IAVs	Human	H1N1	pdm	2009	Greece	A/Athens/INS383/2009	ggtgatgccccattccttgatcggctccgccgagatcaaaagtccttaaaaggaagaggcaacaccc	-	*
CY075191	Human H1N1pdm2009 IAVs	Human	H1N1	pdm	2009	Chile	A/Chile/1599/2009	ggtgatgccccattccttgatcggctccgccgagatcaaaagtccttaaaaggaagaggcaacaccc	-	*
CY072802	Human H1N1pdm2009 IAVs	Human	H1N1		2009	Nicaragua	A/Managua/432.01/2009	ggtgatgccccattccttgatcggctccgccgagatcaaaagtccttaaaaggaagaggcaacaccc	-	*
CY043346	Human H1N1pdm2009 IAVs	Human	H1N1	pdm	2009	Denmark	A/Denmark/524/2009	ggtgatgccccattccttgatcggctccgccgagatcaaaagtccttaaaaggaagaggcaacaccc	-	*
CY043354	Human H1N1pdm2009 IAVs	Human	H1N1	pdm	2009	Denmark	A/Denmark/528/2009	ggtgatgccccattccttgatcggctccgccgagatcaaaagtccttaaaaggaagaggcaacaccc	-	*
CY122831	Human H1N1pdm2009 IAVs	Human	H1N1	pdm	2009	Singapore	A/Singapore/GP2238/2009	ggtgatgccccattccttgatcggctccgccgagatcaaaagtccttaaaaggaagaggcaacaccc	-	*
CY122871	Human H1N1pdm2009 IAVs	Human	H1N1	pdm	2009	Singapore	A/Singapore/GP2293/2009	ggtgatgccccattccttgatcggctccgccgagatcaaaagtccttaaaaggaagaggcaacaccc	-	*
CY122891	Human H1N1pdm2009 IAVs	Human	H1N1	pdm	2009	Singapore	A/Singapore/GP2317/2009	ggtgatgccccattccttgatcggctccgccgagatcaaaagtccttaaaaggaagaggcaacaccc	-	*
CY055601	Human H1N1pdm2009 IAVs	Human	H1N1	pdm	2009	Australia	A/Australia/16/2009	ggtgatgccccattccttgatcggctccgccgagatcaaaagtccttaaaaggaagaggcaacaccc	-	*
CY055063	Human H1N1pdm2009 IAVs	Human	H1N1	pdm	2009	Australia	A/Australia/3/2009	ggtgatgccccattccttgatcggctccgccgagatcaaaagtccttaaaaggaagaggcaacaccc	-	*
CY092197	Human H1N1pdm2009 IAVs	Human	H1N1	pdm	2009	Australia	A/Australia/38/2009	ggtgatgccccattccttgatcggctccgccgagatcaaaagtccttaaaaggaagaggcaacaccc	-	*
CY055768	Human H1N1pdm2009 IAVs	Human	H1N1	pdm	2009	Australia	A/Australia/47/2009	ggtgatgccccattccttgatcggctccgccgagatcaaaagtccttaaaaggaagaggcaacaccc	-	*
HM780480	Human H1N1pdm2009 IAVs	Human	H1N1	pdm	2009	China	A/Guangdong/0752/2009	ggtgatgccccattccttgatcggctccgccgagatcaaaagtccttaaaaggaagaggcaacaccc	-	*
GQ392025	Human H1N1pdm2009 IAVs	Human	H1N1	pdm	2009	Russia	A/Moscow/IIV04/2009	ggtgatgccccattccttgatcggctccgccgagatcaaaagtccttaaaaggaagaggcaacaccc	-	*
CY052491	Human H1N1pdm2009 IAVs	Human	H1N1	pdm	2009	USA	A/Texas/45122036/2009	ggtgatgccccattccttgatcggctccgccgagatcaaaagtccttaaaaggaagaggcaacaccc	-	*
CY052563	Human H1N1pdm2009 IAVs	Human	H1N1	pdm	2009	USA	A/Texas/45122722/2009	ggtgatgccccattccttgatcggctccgccgagatcaaaagtccttaaaaggaagaggcaacaccc	-	*

CY060815	Human H1N1pdm2009 IAVs	Human	H1N1	pdm	2009	USA	A/Texas/46224042/2009	ggtgatgccccattccttgatcggctccgccgagatca aaagtccttaaaaggaagaggcaacaccc	-	*
CY120225	Human H1N1pdm2009 IAVs	Human	H1N1	pdm	2009	Hong_Kong	A/Hong_Kong/H090_712_V10/2009	ggtgatgccccattccttgatcggctccgccgagatca aaagtccttaaaaggaagaggcaacaccc	-	*
CY111775	Human H1N1pdm2009 IAVs	Human	H1N1	pdm	2009	Hong_Kong	A/Hong_Kong/H090_712_V10/2009	ggtgatgccccattccttgatcggctccgccgagatca aaagtccttaaaaggaagaggcaacaccc	-	*
CY120233	Human H1N1pdm2009 IAVs	Human	H1N1	pdm	2009	Hong_Kong	A/Hong_Kong/H090_712_V14/2009	ggtgatgccccattccttgatcggctccgccgagatca aaagtccttaaaaggaagaggcaacaccc	-	*
CY111783	Human H1N1pdm2009 IAVs	Human	H1N1	pdm	2009	Hong_Kong	A/Hong_Kong/H090_712_V1_4/2009	ggtgatgccccattccttgatcggctccgccgagatca aaagtccttaaaaggaagaggcaacaccc	-	*
CY120241	Human H1N1pdm2009 IAVs	Human	H1N1	pdm	2009	Hong_Kong	A/Hong_Kong/H090_712_V20/2009	ggtgatgccccattccttgatcggctccgccgagatca aaagtccttaaaaggaagaggcaacaccc	-	*
CY111791	Human H1N1pdm2009 IAVs	Human	H1N1	pdm	2009	Hong_Kong	A/Hong_Kong/H090_712_V20/2009	ggtgatgccccattccttgatcggctccgccgagatca aaagtccttaaaaggaagaggcaacaccc	-	*
CY120249	Human H1N1pdm2009 IAVs	Human	H1N1	pdm	2009	Hong_Kong	A/Hong_Kong/H090_712_V24/2009	ggtgatgccccattccttgatcggctccgccgagatca aaagtccttaaaaggaagaggcaacaccc	-	*
CY111799	Human H1N1pdm2009 IAVs	Human	H1N1	pdm	2009	Hong_Kong	A/Hong_Kong/H090_712_V24/2009	ggtgatgccccattccttgatcggctccgccgagatca aaagtccttaaaaggaagaggcaacaccc	-	*
JN375249	Human H1N1pdm2009 IAVs	Swine	H1N1		2009	China	A/swine/Guangdong/176/2009	ggtgatgccccattccttgatcggctccgccgagatca aaagtccttaaaaggaagaggcaacaccc	-	*
JN375250	Human H1N1pdm2009 IAVs	Swine	H1N1		2009	China	A/swine/Guangdong/213/2009	ggtgatgccccattccttgatcggctccgccgagatca aaagtccttaaaaggaagaggcaacaccc	-	*
KF411261	Human H1N1pdm2009 IAVs	Human	H1N1	pdm	2009	China	A/Qingdao/1008/2009	ggtgatgccccattccttgatcggctccgccgagatca aaagtccttaaaaggaagaggcaacaccc	-	*
CY066715	Human H1N1pdm2009 IAVs	Human	H1N1	pdm	2009	Austria	A/Vienna/INS293/2009	ggtgatgccccattccttgatcggctccgccgagatca aaagtccttaaaaggaagaggcaacaccc	-	*
JN375251	Human H1N1pdm2009 IAVs	Swine	H1N1		2009	China	A/swine/Guangdong/221/2009	ggtgatgccccattccttgatcggctccgccgagatca aaagtccttaaaaggaagaggcaacaccc	-	*
HM568060	Human H1N1pdm2009 IAVs	Human	H1N1	pdm	2009	United_Kingdom	A/England/1116/2009	ggtgatgccccattccttgatcggctccgccgagatca aaagtccttaaaaggaagaggcaacaccc	-	*
CY107292	Human H1N1pdm2009 IAVs	Human	H1N1		2009	United_Kingdom	A/Scotland/Aberdeen_4/2009	ggtgatgccccattccttgatcggctccgccgagatca aaagtccttaaaaggaagaggcaacaccc	-	*
CY107305	Human H1N1pdm2009 IAVs	Human	H1N1	pdm	2009	United_Kingdom	A/Scotland/Aberdeen_6/2009	ggtgatgccccattccttgatcggctccgccgagatca aaagtccttaaaaggaagaggcaacaccc	-	*
CY107312	Human H1N1pdm2009 IAVs	Human	H1N1		2009	United_Kingdom	A/Scotland/Aberdeen_7/2009	ggtgatgccccattccttgatcggctccgccgagatca aaagtccttaaaaggaagaggcaacaccc	-	*
CY107407	Human H1N1pdm2009 IAVs	Human	H1N1	pdm	2009	United_Kingdom	A/Scotland/Dundee_09V500800/2009	ggtgatgccccattccttgatcggctccgccgagatca aaagtccttaaaaggaagaggcaacaccc	-	*
CY107422	Human H1N1pdm2009 IAVs	Human	H1N1		2009	United_Kingdom	A/Scotland/Dundee_09V502215/2009	ggtgatgccccattccttgatcggctccgccgagatca aaagtccttaaaaggaagaggcaacaccc	-	*
CY107450	Human H1N1pdm2009 IAVs	Human	H1N1	pdm	2009	United_Kingdom	A/Scotland/Dundee_09V502666/2009	ggtgatgccccattccttgatcggctccgccgagatca aaagtccttaaaaggaagaggcaacaccc	-	*
CY107358	Human H1N1pdm2009 IAVs	Human	H1N1	pdm	2009	United_Kingdom	A/Scotland/Perth_09V500257/2009	ggtgatgccccattccttgatcggctccgccgagatca aaagtccttaaaaggaagaggcaacaccc	-	*
CY107365	Human H1N1pdm2009 IAVs	Human	H1N1	pdm	2009	United_Kingdom	A/Scotland/Perth_09V500259/2009	ggtgatgccccattccttgatcggctccgccgagatca aaagtccttaaaaggaagaggcaacaccc	-	*
CY107443	Human H1N1pdm2009 IAVs	Human	H1N1	pdm	2009	United_Kingdom	A/Scotland/Perth_09V502475/2009	ggtgatgccccattccttgatcggctccgccgagatca aaagtccttaaaaggaagaggcaacaccc	-	*
JX625610	Human H1N1pdm2009 IAVs	Human	H1N1	pdm	2009	United_Kingdom	A/Northern_Ireland/94940020/2009	ggtgatgccccattccttgatcggctccgccgagatca aaagtccttaaaaggaagaggcaacaccc	-	*

JX625626	Human H1N1pdm2009 IAVs	Human	H1N1	pdm	2009	United_Kingdom	A/Northern_Ireland/94940033/2009	ggtgatgccccattccttgatcggctccgccgagatcaaaagtccttaaaaggaagaggcaacaccc	-	*
CY069630	Human H1N1pdm2009 IAVs	Human	H1N1	pdm	2009	Singapore	A/Singapore/471/2009	ggtgatgccccattccttgatcggctccgccgagatcaaaagtccttaaaaggaagaggcaacaccc	-	*
CY069645	Human H1N1pdm2009 IAVs	Human	H1N1	pdm	2009	Singapore	A/Singapore/527/2009	ggtgatgccccattccttgatcggctccgccgagatcaaaagtccttaaaaggaagaggcaacaccc	-	*
CY049224	Human H1N1pdm2009 IAVs	Human	H1N1	pdm	2009	Singapore	A/Singapore/ON639/2009	ggtgatgccccattccttgatcggctccgccgagatcaaaagtccttaaaaggaagaggcaacaccc	-	*
CY122767	Human H1N1pdm2009 IAVs	Human	H1N1	pdm	2009	Singapore	A/Singapore/GP1174/2009	ggtgatgccccattccttgatcggctccgccgagatcaaaagtccttaaaaggaagaggcaacaccc	-	*
CY122815	Human H1N1pdm2009 IAVs	Human	H1N1	pdm	2009	Singapore	A/Singapore/GP2223/2009	ggtgatgccccattccttgatcggctccgccgagatcaaaagtccttaaaaggaagaggcaacaccc	-	*
CY122851	Human H1N1pdm2009 IAVs	Human	H1N1	pdm	2009	Singapore	A/Singapore/GP2266/2009	ggtgatgccccattccttgatcggctccgccgagatcaaaagtccttaaaaggaagaggcaacaccc	-	*
CY122899	Human H1N1pdm2009 IAVs	Human	H1N1	pdm	2009	Singapore	A/Singapore/GP2640/2009	ggtgatgccccattccttgatcggctccgccgagatcaaaagtccttaaaaggaagaggcaacaccc	-	*
CY123402	Human H1N1pdm2009 IAVs	Human	H1N1		2009	Singapore	A/Singapore/ON1074/2009	ggtgatgccccattccttgatcggctccgccgagatcaaaagtccttaaaaggaagaggcaacaccc	-	*
CY123903	Human H1N1pdm2009 IAVs	Human	H1N1	pdm	2009	Singapore	A/Singapore/ON279/2009	ggtgatgccccattccttgatcggctccgccgagatcaaaagtccttaaaaggaagaggcaacaccc	-	*
CY123916	Human H1N1pdm2009 IAVs	Human	H1N1	pdm	2009	Singapore	A/Singapore/ON305/2009	ggtgatgccccattccttgatcggctccgccgagatcaaaagtccttaaaaggaagaggcaacaccc	-	*
CY123933	Human H1N1pdm2009 IAVs	Human	H1N1	pdm	2009	Singapore	A/Singapore/ON355/2009	ggtgatgccccattccttgatcggctccgccgagatcaaaagtccttaaaaggaagaggcaacaccc	-	*
CY123937	Human H1N1pdm2009 IAVs	Human	H1N1	pdm	2009	Singapore	A/Singapore/ON360/2009	ggtgatgccccattccttgatcggctccgccgagatcaaaagtccttaaaaggaagaggcaacaccc	-	*
CY123941	Human H1N1pdm2009 IAVs	Human	H1N1	pdm	2009	Singapore	A/Singapore/ON361/2009	ggtgatgccccattccttgatcggctccgccgagatcaaaagtccttaaaaggaagaggcaacaccc	-	*
CY123945	Human H1N1pdm2009 IAVs	Human	H1N1	pdm	2009	Singapore	A/Singapore/ON362/2009	ggtgatgccccattccttgatcggctccgccgagatcaaaagtccttaaaaggaagaggcaacaccc	-	*
CY123949	Human H1N1pdm2009 IAVs	Human	H1N1	pdm	2009	Singapore	A/Singapore/ON368/2009	ggtgatgccccattccttgatcggctccgccgagatcaaaagtccttaaaaggaagaggcaacaccc	-	*
CY123961	Human H1N1pdm2009 IAVs	Human	H1N1	pdm	2009	Singapore	A/Singapore/ON396/2009	ggtgatgccccattccttgatcggctccgccgagatcaaaagtccttaaaaggaagaggcaacaccc	-	*
CY123968	Human H1N1pdm2009 IAVs	Human	H1N1	pdm	2009	Singapore	A/Singapore/ON402/2009	ggtgatgccccattccttgatcggctccgccgagatcaaaagtccttaaaaggaagaggcaacaccc	-	*
CY123976	Human H1N1pdm2009 IAVs	Human	H1N1	pdm	2009	Singapore	A/Singapore/ON457/2009	ggtgatgccccattccttgatcggctccgccgagatcaaaagtccttaaaaggaagaggcaacaccc	-	*
CY124016	Human H1N1pdm2009 IAVs	Human	H1N1	pdm	2009	Singapore	A/Singapore/ON520/2009	ggtgatgccccattccttgatcggctccgccgagatcaaaagtccttaaaaggaagaggcaacaccc	-	*
CY045958	Human H1N1pdm2009 IAVs	Human	H1N1	pdm	2009	Canada	A/Toronto/T0106/2009	ggtgatgccccattccttgatcggctccgccgagatcaaaagtccttaaaaggaagaggcaacaccc	-	*
CY123047	Human H1N1pdm2009 IAVs	Human	H1N1	pdm	2009	Singapore	A/Singapore/GP3081/2009	ggtgatgccccattccttgatcggctccgccgagatcaaaagtccttaaaaggaagaggcaacaccc	-	*
CY049064	Human H1N1pdm2009 IAVs	Human	H1N1	pdm	2009	Singapore	A/Singapore/ON305/2009	ggtgatgccccattccttgatcggctccgccgagatcaaaagtccttaaaaggaagaggcaacaccc	-	*
CY062198	Human H1N1pdm2009 IAVs	Human	H1N1	pdm	2009	USA	A/California/VRDL79/2009	ggtgatgccccattccttgatcggctccgccgagatcaaaagtccttaaaaggaagaggcaacaccc	-	*
CY083483	Human H1N1pdm2009 IAVs	Human	H1N1	pdm	2009	USA	A/Chicago/WRAIR1690P/2009	ggtgatgccccattccttgatcggctccgccgagatcaaaagtccttaaaaggaagaggcaacaccc	-	*

CY107716	Human H1N1pdm2009 IAVs	Human	H1N1	pdm	2009	United_Kingdom	A/Scotland/Glasgow_444125/2009	ggtgatgccccattccttgatcggctccgccgagatca aaagtccttaaaaggaagaggcaacaccc	-	*
CY053154	Human H1N1pdm2009 IAVs	Human	H1N1	pdm	2009	USA	A/Houston/22H/2009	ggtgatgccccattccttgatcggctccgccgagatca aaagtccttaaaaggaagaggcaacaccc	-	*
CY053146	Human H1N1pdm2009 IAVs	Human	H1N1	pdm	2009	USA	A/Houston/22OS/2009	ggtgatgccccattccttgatcggctccgccgagatca aaagtccttaaaaggaagaggcaacaccc	-	*
CY072674	Human H1N1pdm2009 IAVs	Human	H1N1	pdm	2009	Nicaragua	A/Managua/1054.02/2009	ggtgatgccccattccttgatcggctccgccgagatca aaagtccttaaaaggaagaggcaacaccc	-	*
CY072602	Human H1N1pdm2009 IAVs	Human	H1N1		2009	Nicaragua	A/Managua/1077.04/2009	ggtgatgccccattccttgatcggctccgccgagatca aaagtccttaaaaggaagaggcaacaccc	-	*
CY072618	Human H1N1pdm2009 IAVs	Human	H1N1	pdm	2009	Nicaragua	A/Managua/2604.02/2009	ggtgatgccccattccttgatcggctccgccgagatca aaagtccttaaaaggaagaggcaacaccc	-	*
CY072746	Human H1N1pdm2009 IAVs	Human	H1N1	pdm	2009	Nicaragua	A/Managua/2958.01/2009	ggtgatgccccattccttgatcggctccgccgagatca aaagtccttaaaaggaagaggcaacaccc	-	*
CY058184	Human H1N1pdm2009 IAVs	Human	H1N1	pdm	2009	Nicaragua	A/Managua/3189.01/2009	ggtgatgccccattccttgatcggctccgccgagatca aaagtccttaaaaggaagaggcaacaccc	-	*
CY069286	Human H1N1pdm2009 IAVs	Human	H1N1	pdm	2009	Nicaragua	A/Managua/3467.01/2009	ggtgatgccccattccttgatcggctccgccgagatca aaagtccttaaaaggaagaggcaacaccc	-	*
CY090085	Human H1N1pdm2009 IAVs	Human	H1N1	pdm	2009	Nicaragua	A/Managua/4032.03/2009	ggtgatgccccattccttgatcggctccgccgagatca aaagtccttaaaaggaagaggcaacaccc	-	*
CY072586	Human H1N1pdm2009 IAVs	Human	H1N1	pdm	2009	Nicaragua	A/Managua/4186.02/2009	ggtgatgccccattccttgatcggctccgccgagatca aaagtccttaaaaggaagaggcaacaccc	-	*
CY073669	Human H1N1pdm2009 IAVs	Human	H1N1	pdm	2009	Nicaragua	A/Managua/4209.04/2009	ggtgatgccccattccttgatcggctccgccgagatca aaagtccttaaaaggaagaggcaacaccc	-	*
CY072778	Human H1N1pdm2009 IAVs	Human	H1N1	pdm	2009	Nicaragua	A/Managua/5235.03/2009	ggtgatgccccattccttgatcggctccgccgagatca aaagtccttaaaaggaagaggcaacaccc	-	*
CY058232	Human H1N1pdm2009 IAVs	Human	H1N1		2009	Nicaragua	A/Managua/5363.02/2009	ggtgatgccccattccttgatcggctccgccgagatca aaagtccttaaaaggaagaggcaacaccc	-	*
CY073693	Human H1N1pdm2009 IAVs	Human	H1N1	pdm	2009	Nicaragua	A/Managua/5953.01/2009	ggtgatgccccattccttgatcggctccgccgagatca aaagtccttaaaaggaagaggcaacaccc	-	*
CY058208	Human H1N1pdm2009 IAVs	Human	H1N1	pdm	2009	Nicaragua	A/Managua/791.03/2009	ggtgatgccccattccttgatcggctccgccgagatca aaagtccttaaaaggaagaggcaacaccc	-	*
CY073629	Human H1N1pdm2009 IAVs	Human	H1N1	pdm	2009	Nicaragua	A/Managua/5595.03/2009	ggtgatgccccattccttgatcggctccgccgagatca aaagtccttaaaaggaagaggcaacaccc	-	*
CY046975	Human H1N1pdm2009 IAVs	Human	H1N1	pdm	2009	USA	A/New_York/3443/2009	ggtgatgccccattccttgatcggctccgccgagatca aaagtccttaaaaggaagaggcaacaccc	-	*
CY043175	Human H1N1pdm2009 IAVs	Human	H1N1	pdm	2009	USA	A/New_York/3460/2009	ggtgatgccccattccttgatcggctccgccgagatca aaagtccttaaaaggaagaggcaacaccc	-	*
KJ023106	Human H1N1pdm2009 IAVs	Human	H1N1	pdm	2009	India	A/Delhi/016/2009	ggtgatgccccattccttgatcggctccgccgagatca aaagtccttaaaaggaagaggcaacaccc	-	*
KJ023107	Human H1N1pdm2009 IAVs	Human	H1N1	pdm	2009	India	A/Delhi/017/2009	ggtgatgccccattccttgatcggctccgccgagatca aaagtccttaaaaggaagaggcaacaccc	-	*
GQ396527	Human H1N1pdm2009 IAVs	Human	H1N1	pdm	2009	Spain	A/Madrid/GP523/2009	ggtgatgccccattccttgatcggctccgccgagatca aaagtccttaaaaggaagaggcaacaccc	-	*
CY073517	Human H1N1pdm2009 IAVs	Human	H1N1	pdm	2009	Nicaragua	A/Managua/3014.02/2009	ggtgatgccccattccttgatcggctccgccgagatca aaagtccttaaaaggaagaggcaacaccc	-	*
GQ168848	Human H1N1pdm2009 IAVs	Human	H1N1	pdm	2009	USA	A/Massachusetts/06/2009	ggtgatgccccattccttgatcggctccgccgagatca aaagtccttaaaaggaagaggcaacaccc	-	*
CY058696	Human H1N1pdm2009 IAVs	Human	H1N1	pdm	2009	USA	A/Texas/461917783/2009	ggtgatgccccattccttgatcggctccgccgagatca aaagtccttaaaaggaagaggcaacaccc	-	*

GQ168874	Human H1N1pdm2009 IAVs	Human	H1N1	pdm	2009	USA	A/Massachusetts/07/2009	ggtgatgccccattccttgatcggctccgccgagatca<br>aaagtccttaaaaggaagaggcaacaccc	-	*
CY057746	Human H1N1pdm2009 IAVs	Human	H1N1	pdm	2009	USA	A/Wisconsin/629_D00151/2009	ggtgatgccccattccttgatcggctccgccgagatca<br>aaagtccttaaaaggaagaggcaacaccc	-	*
CY063223	Human H1N1pdm2009 IAVs	Human	H1N1	pdm	2009	USA	A/Wisconsin/629_D01351/2009	ggtgatgccccattccttgatcggctccgccgagatca<br>aaagtccttaaaaggaagaggcaacaccc	-	*
CY057682	Human H1N1pdm2009 IAVs	Human	H1N1		2009	USA	A/Wisconsin/629_S1384/2009	ggtgatgccccattccttgatcggctccgccgagatca<br>aaagtccttaaaaggaagaggcaacaccc	-	*
CY057802	Human H1N1pdm2009 IAVs	Human	H1N1		2009	USA	A/Wisconsin/629_D01466/2009	ggtgatgccccattccttgatcggctccgccgagatca<br>aaagtccttaaaaggaagaggcaacaccc	-	*
CY058304	Human H1N1pdm2009 IAVs	Human	H1N1	pdm	2009	USA	A/Wisconsin/629_D02018/2009	ggtgatgccccattccttgatcggctccgccgagatca<br>aaagtccttaaaaggaagaggcaacaccc	-	*
CY057810	Human H1N1pdm2009 IAVs	Human	H1N1	pdm	2009	USA	A/Wisconsin/629_D02392/2009	ggtgatgccccattccttgatcggctccgccgagatca<br>aaagtccttaaaaggaagaggcaacaccc	-	*
CY083680	Human H1N1pdm2009 IAVs	Human	H1N1	pdm	2009	Spain	A/Madrid/INS77/2009	ggtgatgccccattccttgatcggctccgccgagatca<br>aaagtccttaaaaggaagaggcaacaccc	-	*
CY064704	Human H1N1pdm2009 IAVs	Human	H1N1	pdm	2009	Mexico	A/Mexico_City/019/2009	ggtgatgccccattccttgatcggctccgccgagatca<br>aaagtccttaaaaggaagaggcaacaccc	-	*
CY107603	Human H1N1pdm2009 IAVs	Human	H1N1	pdm	2009	United_Kingdom	A/Scotland/Glasgow_413364/2009	ggtgatgccccattccttgatcggctccgccgagatca<br>aaagtccttaaaaggaagaggcaacaccc	-	*
CY056631	Human H1N1pdm2009 IAVs	Human	H1N1	pdm	2009	USA	A/New_York/6018/2009	ggtgatgccccattccttgatcggctccgccgagatca<br>aaagtccttaaaaggaagaggcaacaccc	-	*
CY045097	Human H1N1pdm2009 IAVs	Human	H1N1	pdm	2009	USA	A/New_York/3614/2009	ggtgatgccccattccttgatcggctccgccgagatca<br>aaagtccttaaaaggaagaggcaacaccc	-	*
CY062759	Human H1N1pdm2009 IAVs	Human	H1N1	pdm	2009	Spain	A/Madrid/INS131/2009	ggtgatgccccattccttgatcggctccgccgagatca<br>aaagtccttaaaaggaagaggcaacaccc	-	*
CY071259	Human H1N1pdm2009 IAVs	Human	H1N1	pdm	2009	Spain	A/Madrid/INS408/2009	ggtgatgccccattccttgatcggctccgccgagatca<br>aaagtccttaaaaggaagaggcaacaccc	-	*
CY065780	Human H1N1pdm2009 IAVs	Human	H1N1	pdm	2009	Netherlands	A/Netherlands/2635b/2009	ggtgatgccccattccttgatcggctccgccgagatca<br>aaagtccttaaaaggaagaggcaacaccc	-	*
CY058728	Human H1N1pdm2009 IAVs	Human	H1N1	pdm	2009	USA	A/Texas/JMS376/2009	ggtgatgccccattccttgatcggctccgccgagatca<br>aaagtccttaaaaggaagaggcaacaccc	-	*
CY066771	Human H1N1pdm2009 IAVs	Human	H1N1	pdm	2009	USA	A/Pensacola/INS212/2009	ggtgatgccccattccttgatcggctccgccgagatca<br>aaagtccttaaaaggaagaggcaacaccc	-	*
CY119374	Human H1N1pdm2009 IAVs	Human	H1N1	pdm	2009	Malaysia	A/Malaysia/2076204/2009	ggtgatgccccattccttgatcggctccgccgagatca<br>aaagtccttaaaaggaagaggcaacaccc	-	*
CY060983	Human H1N1pdm2009 IAVs	Human	H1N1	pdm	2009	USA	A/Texas/JMS383/2009	ggtgatgccccattccttgatcggctccgccgagatca<br>aaagtccttaaaaggaagaggcaacaccc	-	*
CY072266	Human H1N1pdm2009 IAVs	Human	H1N1	pdm	2009	Poland	A/Warsaw/INS310/2009	ggtgatgccccattccttgatcggctccgccgagatca<br>aaagtccttaaaaggaagaggcaacaccc	-	*
CY052707	Human H1N1pdm2009 IAVs	Human	H1N1	pdm	2009	USA	A/Texas/45093214/2009	ggtgatgccccattccttgatcggctccgccgagatca<br>aaagtccttaaaaggaagaggcaacaccc	-	*
CY060887	Human H1N1pdm2009 IAVs	Human	H1N1	pdm	2009	USA	A/Texas/JMS363/2009	ggtgatgccccattccttgatcggctccgccgagatca<br>aaagtccttaaaaggaagaggcaacaccc	-	*
JN187330	Human H1N1pdm2009 IAVs	Human	H1N1	pdm	2009	Taiwan	A/Taiwan/937/2009	ggtgatgccccattccttgatcggctccgccgagatca<br>aaagtccttaaaaggaagaggcaacaccc	-	*
CY052779	Human H1N1pdm2009 IAVs	Human	H1N1	pdm	2009	USA	A/Texas/45021632/2009	ggtgatgccccattccttgatcggctccgccgagatca<br>aaagtccttaaaaggaagaggcaacaccc	-	*
CY123560	Human H1N1pdm2009 IAVs	Human	H1N1	pdm	2009	Singapore	A/Singapore/ON1852/2009	ggtgatgccccattccttgatcggctccgccgagatca<br>aaagtccttaaaaggaagaggcaacaccc	-	*

CY049999	Human H1N1pdm2009 IAVs	Human	H1N1	pdm	2009	Mexico	A/Mexico_City/WR1100N/2009	ggtgatgccccattccttgatcggctccgccgagatca aaagtccttaaaaggaagaggcaacaccc	-	*
CY083172	Human H1N1pdm2009 IAVs	Human	H1N1		2009	USA	A/New_Jersey/WRAIR1499P/2009	ggtgatgccccattccttgatcggctccgccgagatca aaagtccttaaaaggaagaggcaacaccc	-	*
CY083212	Human H1N1pdm2009 IAVs	Human	H1N1		2009	USA	A/New_Jersey/WRAIR1504P/2009	ggtgatgccccattccttgatcggctccgccgagatca aaagtccttaaaaggaagaggcaacaccc	-	*
CY083220	Human H1N1pdm2009 IAVs	Human	H1N1	pdm	2009	USA	A/New_Jersey/WRAIR1506P/2009	ggtgatgccccattccttgatcggctccgccgagatca aaagtccttaaaaggaagaggcaacaccc	-	*
CY074039	Human H1N1pdm2009 IAVs	Human	H1N1	pdm	2009	Argentina	A/Argentina/HNRG40/2009	ggtgatgccccattccttgatcggctccgccgagatca aaagtccttaaaaggaagaggcaacaccc	-	*
CY075439	Human H1N1pdm2009 IAVs	Human	H1N1	pdm	2009	Chile	A/Chile/4181/2009	ggtgatgccccattccttgatcggctccgccgagatca aaagtccttaaaaggaagaggcaacaccc	-	*
CY075447	Human H1N1pdm2009 IAVs	Human	H1N1	pdm	2009	Chile	A/Chile/4182/2009	ggtgatgccccattccttgatcggctccgccgagatca aaagtccttaaaaggaagaggcaacaccc	-	*
CY063578	Human H1N1pdm2009 IAVs	Human	H1N1	pdm	2009	USA	A/Boston/154/2009	ggtgatgccccattccttgatcggctccgccgagatca aaagtccttaaaaggaagaggcaacaccc	-	*
CY052110	Human H1N1pdm2009 IAVs	Human	H1N1	pdm	2009	USA	A/Wisconsin/629_D00739/2009	ggtgatgccccattccttgatcggctccgccgagatca aaagtccttaaaaggaagaggcaacaccc	-	*
CY051155	Human H1N1pdm2009 IAVs	Human	H1N1	pdm	2009	USA	A/Wisconsin/629_D01147/2009	ggtgatgccccattccttgatcggctccgccgagatca aaagtccttaaaaggaagaggcaacaccc	-	*
CY046703	Human H1N1pdm2009 IAVs	Human	H1N1	pdm	2009	USA	A/Wisconsin/629_D01445/2009	ggtgatgccccattccttgatcggctccgccgagatca aaagtccttaaaaggaagaggcaacaccc	-	*
CY071718	Human H1N1pdm2009 IAVs	Human	H1N1	pdm	2009	Mexico	A/Mexico_City/WR1695N/2009	ggtgatgccccattccttgatcggctccgccgagatca aaagtccttaaaaggaagaggcaacaccc	-	*
CY063175	Human H1N1pdm2009 IAVs	Human	H1N1	pdm	2009	USA	A/California/VRDL102/2009	ggtgatgccccattccttgatcggctccgccgagatca aaagtccttaaaaggaagaggcaacaccc	-	*
CY066347	Human H1N1pdm2009 IAVs	Human	H1N1	pdm	2009	USA	A/California/VRDL121/2009	ggtgatgccccattccttgatcggctccgccgagatca aaagtccttaaaaggaagaggcaacaccc	-	*
CY120735	Human H1N1pdm2009 IAVs	Human	H1N1	pdm	2009	Brazil	A/Brazil/AVS06/2009	ggtgatgccccattccttgatcggctccgccgagatca aaagtccttaaaaggaagaggcaacaccc	-	*
CY061801	Human H1N1pdm2009 IAVs	Swine	H1N1		2009	Hong_Kong	A/swine/Hong_Kong/2995/2009	ggtgatgccccattccttgatcggctccgccgagatca aaagtccttaaaaggaagaggcaacaccc	-	*
CY056120	Human H1N1pdm2009 IAVs	Human	H1N1	pdm	2009	USA	A/District_of_Columbia/INS31/2009	ggtgatgccccattccttgatcggctccgccgagatca aaagtccttaaaaggaagaggcaacaccc	-	*
CY044881	Human H1N1pdm2009 IAVs	Human	H1N1	pdm	2009	USA	A/New_York/3573/2009	ggtgatgccccattccttgatcggctccgccgagatca aaagtccttaaaaggaagaggcaacaccc	-	*
CY046967	Human H1N1pdm2009 IAVs	Human	H1N1	pdm	2009	USA	A/New_York/3576/2009	ggtgatgccccattccttgatcggctccgccgagatca aaagtccttaaaaggaagaggcaacaccc	-	*
CY045195	Human H1N1pdm2009 IAVs	Human	H1N1	pdm	2009	USA	A/New_York/4014/2009	ggtgatgccccattccttgatcggctccgccgagatca aaagtccttaaaaggaagaggcaacaccc	-	*
CY087037	Human H1N1pdm2009 IAVs	Human	H1N1	pdm	2009	USA	A/New_York/4631/2009	ggtgatgccccattccttgatcggctccgccgagatca aaagtccttaaaaggaagaggcaacaccc	-	*
CY055936	Human H1N1pdm2009 IAVs	Human	H1N1	pdm	2009	Australia	A/Australia/81/2009	ggtgatgccccattccttgatcggctccgccgagatca aaagtccttaaaaggaagaggcaacaccc	-	*
CY055944	Human H1N1pdm2009 IAVs	Human	H1N1	pdm	2009	Australia	A/Australia/82/2009	ggtgatgccccattccttgatcggctccgccgagatca aaagtccttaaaaggaagaggcaacaccc	-	*
CY066243	Human H1N1pdm2009 IAVs	Human	H1N1	pdm	2009	USA	A/California/VRDL108/2009	ggtgatgccccattccttgatcggctccgccgagatca aaagtccttaaaaggaagaggcaacaccc	-	*
CY066315	Human H1N1pdm2009 IAVs	Human	H1N1	pdm	2009	USA	A/California/VRDL117/2009	ggtgatgccccattccttgatcggctccgccgagatca aaagtccttaaaaggaagaggcaacaccc	-	*

CY072826	Human H1N1pdm2009 IAVs	Human	H1N1	pdm	2009	Nicaragua	A/Managua/5115.01/2009	ggtgatgccccattccttgatcggctccgccgagatcaaaagtccttaaaaggaagaggcaacaccc	-	*
CY057946	Human H1N1pdm2009 IAVs	Human	H1N1	pdm	2009	USA	A/Wisconsin/629_D00629/2009	ggtgatgccccattccttgatcggctccgccgagatcaaaagtccttaaaaggaagaggcaacaccc	-	*
CY083809	Human H1N1pdm2009 IAVs	Human	H1N1	pdm	2009	Spain	A/Madrid/INS377/2009	ggtgatgccccattccttgatcggctccgccgagatcaaaagtccttaaaaggaagaggcaacaccc	-	*
CY065860	Human H1N1pdm2009 IAVs	Human	H1N1	pdm	2009	Netherlands	A/Netherlands/2229/2009	ggtgatgccccattccttgatcggctccgccgagatcaaaagtccttaaaaggaagaggcaacaccc	-	*
CY083791	Human H1N1pdm2009 IAVs	Human	H1N1	pdm	2009	Denmark	A/Odense/INS309/2009	ggtgatgccccattccttgatcggctccgccgagatcaaaagtccttaaaaggaagaggcaacaccc	-	*
HQ263291	Human H1N1pdm2009 IAVs	Human	H1N1	pdm	2009	USA	A/Bethesda/NIH107_D31/2009	ggtgatgccccattccttgatcggctccgccgagatcaaaagtccttaaaaggaagaggcaacaccc	-	*
CY064664	Human H1N1pdm2009 IAVs	Human	H1N1	pdm	2009	USA	A/Boston/138/2009	ggtgatgccccattccttgatcggctccgccgagatcaaaagtccttaaaaggaagaggcaacaccc	-	*
CY055055	Human H1N1pdm2009 IAVs	Human	H1N1	pdm	2009	USA	A/California/VRDL71/2009	ggtgatgccccattccttgatcggctccgccgagatcaaaagtccttaaaaggaagaggcaacaccc	-	*
CY065682	Human H1N1pdm2009 IAVs	Human	H1N1		2009	United_Kingdom	A/England/419/2009	ggtgatgccccattccttgatcggctccgccgagatcaaaagtccttaaaaggaagaggcaacaccc	-	*
CY122667	Human H1N1pdm2009 IAVs	Human	H1N1	pdm	2009	Singapore	A/Singapore/GP1127/2009	ggtgatgccccattccttgatcggctccgccgagatcaaaagtccttaaaaggaagaggcaacaccc	-	*
HM189508	Human H1N1pdm2009 IAVs	Human	H1N1	pdm	2009	South_Korea	A/Korea/CJ63/2009	ggtgatgccccattccttgatcggctccgccgagatcaaaagtccttaaaaggaagaggcaacaccc	-	*
JX625517	Human H1N1pdm2009 IAVs	Human	H1N1	pdm	2009	United_Kingdom	A/England/1072/2009	ggtgatgccccattccttgatcggctccgccgagatcaaaagtccttaaaaggaagaggcaacaccc	-	*
GQ494351	Human H1N1pdm2009 IAVs	Human	H1N1	pdm	2009	Russia	A/Moscow/IIV05/2009	ggtgatgccccattccttgatcggctccgccgagatcaaaagtccttaaaaggaagaggcaacaccc	-	*
KF411259	Human H1N1pdm2009 IAVs	Human	H1N1	pdm	2009	China	A/Qingdao/1006/2009	ggtgatgccccattccttgatcggctccgccgagatcaaaagtccttaaaaggaagaggcaacaccc	-	*
KF411260	Human H1N1pdm2009 IAVs	Human	H1N1	pdm	2009	China	A/Qingdao/1020/2009	ggtgatgccccattccttgatcggctccgccgagatcaaaagtccttaaaaggaagaggcaacaccc	-	*
CY123757	Human H1N1pdm2009 IAVs	Human	H1N1	pdm	2009	Singapore	A/Singapore/ON2095/2009	ggtgatgccccattccttgatcggctccgccgagatcaaaagtccttaaaaggaagaggcaacaccc	-	*
CY083850	Human H1N1pdm2009 IAVs	Human	H1N1	pdm	2009	USA	A/District_of_Columbia/INS23/2009	ggtgatgccccattccttgatcggctccgccgagatcaaaagtccttaaaaggaagaggcaacaccc	-	*
GQ360059	Human H1N1pdm2009 IAVs	Human	H1N1	pdm	2009	Sweden	A/Stockholm/34/2009	ggtgatgccccattccttgatcggctccgccgagatcaaaagtccttaaaaggaagaggcaacaccc	-	*
KC780792	Human H1N1pdm2009 IAVs	Human	H1N1		2009	USA	A/Maryland/31/2009	ggtgatgccccattccttgatcggctccgccgagatcaaaagtccttaaaaggaagaggcaacaccc	-	*
JF327346	Human H1N1pdm2009 IAVs	Human	H1N1	pdm	2009	Finland	A/Finland/690/2009	ggtgatgccccattccttgatcggctccgccgagatcaaaagtccttaaaaggaagaggcaacaccc	-	*
CY071195	Human H1N1pdm2009 IAVs	Human	H1N1	pdm	2009	Germany	A/Frankfurt/INS399/2009	ggtgatgccccattccttgatcggctccgccgagatcaaaagtccttaaaaggaagaggcaacaccc	-	*
CY063031	Human H1N1pdm2009 IAVs	Human	H1N1	pdm	2009	Spain	A/Madrid/INS185/2009	ggtgatgccccattccttgatcggctccgccgagatcaaaagtccttaaaaggaagaggcaacaccc	-	*
CY121872	Human H1N1pdm2009 IAVs	Swine	H1N1		2009	Thailand	A/swine/Thailand/CU_M6_1/2009	ggtgatgccccattccttgatcggctccgccgagatcaaaagtccttaaaaggaagaggcaacaccc	-	*
CY066723	Human H1N1pdm2009 IAVs	Human	H1N1	pdm	2009	Spain	A/Madrid/INS295/2009	ggtgatgccccattccttgatcggctccgccgagatcaaaagtccttaaaaggaagaggcaacaccc	-	*
CY051843	Human H1N1pdm2009 IAVs	Human	H1N1	pdm	2009	USA	A/Texas/42123701/2009	ggtgatgccccattccttgatcggctccgccgagatcaaaagtccttaaaaggaagaggcaacaccc	-	*

CY051851	Human H1N1pdm2009 IAVs	Human	H1N1	pdm	2009	USA	A/Texas/42191653/2009	ggtgatgccccattccttgatcggctccgccgagatca aaagtccttaaaaggaagaggcaacaccc	-	*
CY052643	Human H1N1pdm2009 IAVs	Human	H1N1	pdm	2009	USA	A/Texas/45062584/2009	ggtgatgccccattccttgatcggctccgccgagatca aaagtccttaaaaggaagaggcaacaccc	-	*
CY055047	Human H1N1pdm2009 IAVs	Human	H1N1	pdm	2009	USA	A/California/VRDL70/2009	ggtgatgccccattccttgatcggctccgccgagatca aaagtccttaaaaggaagaggcaacaccc	-	*
CY120265	Human H1N1pdm2009 IAVs	Human	H1N1	pdm	2009	Hong_Kong	A/Hong_Kong/H090_722_V10/2009	ggtgatgccccattccttgatcggctccgccgagatca aaagtccttaaaaggaagaggcaacaccc	-	*
CY111823	Human H1N1pdm2009 IAVs	Human	H1N1	pdm	2009	Hong_Kong	A/Hong_Kong/H090_722_V10/2009	ggtgatgccccattccttgatcggctccgccgagatca aaagtccttaaaaggaagaggcaacaccc	-	*
CY111831	Human H1N1pdm2009 IAVs	Human	H1N1	pdm	2009	Hong_Kong	A/Hong_Kong/H090_722_V20/2009	ggtgatgccccattccttgatcggctccgccgagatca aaagtccttaaaaggaagaggcaacaccc	-	*
CY062214	Human H1N1pdm2009 IAVs	Human	H1N1	pdm	2009	USA	A/California/VRDL81/2009	ggtgatgccccattccttgatcggctccgccgagatca aaagtccttaaaaggaagaggcaacaccc	-	*
CY119534	Human H1N1pdm2009 IAVs	Human	H1N1	pdm	2009	Malaysia	A/Malaysia/2072040/2009	ggtgatgccccattccttgatcggctccgccgagatca aaagtccttaaaaggaagaggcaacaccc	-	*
CY056032	Human H1N1pdm2009 IAVs	Human	H1N1		2009	USA	A/San_Diego/INS16/2009	ggtgatgccccattccttgatcggctccgccgagatca aaagtccttaaaaggaagaggcaacaccc	-	*
CY089215	Human H1N1pdm2009 IAVs	Human	H1N1	pdm	2009	USA	A/Boston/618/2009	ggtgatgccccattccttgatcggctccgccgagatca aaagtccttaaaaggaagaggcaacaccc	-	*
CY056727	Human H1N1pdm2009 IAVs	Human	H1N1	pdm	2009	USA	A/New_York/6473/2009	ggtgatgccccattccttgatcggctccgccgagatca aaagtccttaaaaggaagaggcaacaccc	-	*
CY083547	Human H1N1pdm2009 IAVs	Human	H1N1	pdm	2009	Hungary	A/Budapest/WRAIR2393N/2009	ggtgatgccccattccttgatcggctccgccgagatca aaagtccttaaaaggaagaggcaacaccc	-	*
CY083555	Human H1N1pdm2009 IAVs	Human	H1N1	pdm	2009	Hungary	A/Budapest/WRAIR2393T/2009	ggtgatgccccattccttgatcggctccgccgagatca aaagtccttaaaaggaagaggcaacaccc	-	*
CY128063	Human H1N1pdm2009 IAVs	Human	H1	pdm	2009	Viet_Nam	A/Viet_Nam/001_1711/2009	ggtgatgccccattccttgatcggctccgccgagatca aaagtccttaaaaggaagaggcaacaccc	-	*
CY107271	Human H1N1pdm2009 IAVs	Human	H1N1	pdm	2009	United_Kingdom	A/Scotland/Inverness_18/2009	ggtgatgccccattccttgatcggctccgccgagatca aaagtccttaaaaggaagaggcaacaccc	-	*
CY118224	Human H1N1pdm2009 IAVs	Human	H1N1	pdm	2009	Malaysia	A/Malaysia/2098038/2009	ggtgatgccccattccttgatcggctccgccgagatca aaagtccttaaaaggaagaggcaacaccc	-	*
GU136016	Human H1N1pdm2009 IAVs	Human	H1N1	pdm	2009	Japan	A/Niigata/700/2009	ggtgatgccccattccttgatcggctccgccgagatca aaagtccttaaaaggaagaggcaacaccc	-	*
GQ334359	Human H1N1pdm2009 IAVs	Human	H1N1	pdm	2009	Japan	A/Utsunomiya/1/2009	ggtgatgccccattccttgatcggctccgccgagatca aaagtccttaaaaggaagaggcaacaccc	-	*
GQ365459	Human H1N1pdm2009 IAVs	Human	H1N1	pdm	2009	Japan	A/Utsunomiya/2/2009	ggtgatgccccattccttgatcggctccgccgagatca aaagtccttaaaaggaagaggcaacaccc	-	*
CY123972	Human H1N1pdm2009 IAVs	Human	H1N1		2009	Singapore	A/Singapore/ON406/2009	ggtgatgccccattccttgatcggctccgccgagatca aaagtccttaaaaggaagaggcaacaccc	-	*
CY107736	Human H1N1pdm2009 IAVs	Human	H1N1	pdm	2009	United_Kingdom	A/Scotland/Glasgow_445236/2009	ggtgatgccccattccttgatcggctccgccgagatca aaagtccttaaaaggaagaggcaacaccc	-	*
KC780777	Human H1N1pdm2009 IAVs	Human	H1N1	pdm	2009	USA	A/Alaska/47/2009	ggtgatgccccattccttgatcggctccgccgagatca aaagtccttaaaaggaagaggcaacaccc	-	*
CY123793	Human H1N1pdm2009 IAVs	Human	H1N1	pdm	2009	Singapore	A/Singapore/ON2168/2009	ggtgatgccccattccttgatcggctccgccgagatca aaagtccttaaaaggaagaggcaacaccc	-	*
CY056695	Human H1N1pdm2009 IAVs	Human	H1N1	pdm	2009	USA	A/New_York/6292/2009	ggtgatgccccattccttgatcggctccgccgagatca aaagtccttaaaaggaagaggcaacaccc	-	*
CY061015	Human H1N1pdm2009 IAVs	Human	H1N1	pdm	2009	USA	A/Texas/JMS387/2009	ggtgatgccccattccttgatcggctccgccgagatca aaagtccttaaaaggaagaggcaacaccc	-	*

HM569671	Human H1N1pdm2009 IAVs	Human	H1N1	pdm	2009	Argentina	A/Argentina/08AR/2009	ggtgatgccccattccttgatcggctccgccgagatcaaaagtccttaaaaggaagaggcaacaccc	-	*
CY060618	Human H1N1pdm2009 IAVs	Human	H1N1	pdm	2009	Canada	A/Ontario/309862/2009	ggtgatgccccattccttgatcggctccgccgagatcaaaagtccttaaaaggaagaggcaacaccc	-	*
CY060658	Human H1N1pdm2009 IAVs	Human	H1N1	pdm	2009	Canada	A/Ontario/315003/2009	ggtgatgccccattccttgatcggctccgccgagatcaaaagtccttaaaaggaagaggcaacaccc	-	*
CY107429	Human H1N1pdm2009 IAVs	Human	H1N1	pdm	2009	United_Kingdom	A/Scotland/Dundee_09V502385/2009	ggtgatgccccattccttgatcggctccgccgagatcaaaagtccttaaaaggaagaggcaacaccc	-	*
CY060855	Human H1N1pdm2009 IAVs	Human	H1N1	pdm	2009	USA	A/Texas/JMS358/2009	ggtgatgccccattccttgatcggctccgccgagatcaaaagtccttaaaaggaagaggcaacaccc	-	*
CY117645	Human H1N1pdm2009 IAVs	Human	H1N1	pdm	2009	Mexico	A/Mexico/UASLP_023/2009	ggtgatgccccattccttgatcggctccgccgagatcaaaagtccttaaaaggaagaggcaacaccc	-	*
CY117664	Human H1N1pdm2009 IAVs	Human	H1N1	pdm	2009	Mexico	A/Mexico/UASLP_026/2009	ggtgatgccccattccttgatcggctccgccgagatcaaaagtccttaaaaggaagaggcaacaccc	-	*
CY117667	Human H1N1pdm2009 IAVs	Human	H1N1	pdm	2009	Mexico	A/Mexico/UASLP_028/2009	ggtgatgccccattccttgatcggctccgccgagatcaaaagtccttaaaaggaagaggcaacaccc	-	*
CY055888	Human H1N1pdm2009 IAVs	Human	H1N1	pdm	2009	Australia	A/Australia/69/2009	ggtgatgccccattccttgatcggctccgccgagatcaaaagtccttaaaaggaagaggcaacaccc	-	*
GQ329073	Human H1N1pdm2009 IAVs	Human	H1N1	pdm	2009	France	A/Caen/2716/2009	ggtgatgccccattccttgatcggctccgccgagatcaaaagtccttaaaaggaagaggcaacaccc	-	*
CY053170	Human H1N1pdm2009 IAVs	Human	H1N1	pdm	2009	USA	A/Houston/24OS/2009	ggtgatgccccattccttgatcggctccgccgagatcaaaagtccttaaaaggaagaggcaacaccc	-	*
CY045203	Human H1N1pdm2009 IAVs	Human	H1N1	pdm	2009	USA	A/New_York/4048/2009	ggtgatgccccattccttgatcggctccgccgagatcaaaagtccttaaaaggaagaggcaacaccc	-	*
CY058680	Human H1N1pdm2009 IAVs	Human	H1N1	pdm	2009	USA	A/Wisconsin/629_D00938/2009	ggtgatgccccattccttgatcggctccgccgagatcaaaagtccttaaaaggaagaggcaacaccc	-	*
CY089287	Human H1N1pdm2009 IAVs	Human	H1N1	pdm	2009	USA	A/Boston/667/2009	ggtgatgccccattccttgatcggctccgccgagatcaaaagtccttaaaaggaagaggcaacaccc	-	*
CY072322	Human H1N1pdm2009 IAVs	Human	H1N1		2009	USA	A/New_York/INS317/2009	ggtgatgccccattccttgatcggctccgccgagatcaaaagtccttaaaaggaagaggcaacaccc	-	*
JX875025	Human H1N1pdm2009 IAVs	Human	H1N1	pdm	2009	USA	A/Kentucky/136/2009	ggtgatgccccattccttgatcggctccgccgagatcaaaagtccttaaaaggaagaggcaacaccc	-	*
JX875010	Human H1N1pdm2009 IAVs	Human	H1N1	pdm	2009	USA	A/Kentucky/80/2009	ggtgatgccccattccttgatcggctccgccgagatcaaaagtccttaaaaggaagaggcaacaccc	-	*
KJ023094	Human H1N1pdm2009 IAVs	Human	H1N1	pdm	2009	India	A/West_Bengal/004/2009	ggtgatgccccattccttgatcggctccgccgagatcaaaagtccttaaaaggaagaggcaacaccc	-	*
HQ728098	Human H1N1pdm2009 IAVs	Swine	H1N1		2009	Taiwan	A/swine/Taiwan/HL_1125/2009	ggtgatgccccattccttgatcggctccgccgagatcaaaagtccttaaaaggaagaggcaacaccc	-	*
HQ728106	Human H1N1pdm2009 IAVs	Swine	H1N1		2009	Taiwan	A/swine/Taiwan/PT_1204/2009	ggtgatgccccattccttgatcggctccgccgagatcaaaagtccttaaaaggaagaggcaacaccc	-	*
CY066307	Human H1N1pdm2009 IAVs	Human	H1N1	pdm	2009	USA	A/California/VRDL116/2009	ggtgatgccccattccttgatcggctccgccgagatcaaaagtccttaaaaggaagaggcaacaccc	-	*
CY072282	Human H1N1pdm2009 IAVs	Human	H1N1		2009	Poland	A/Warsaw/INS312/2009	ggtgatgccccattccttgatcggctccgccgagatcaaaagtccttaaaaggaagaggcaacaccc	-	*
CY054927	Human H1N1pdm2009 IAVs	Human	H1N1	pdm	2009	USA	A/California/VRDL43/2009	ggtgatgccccattccttgatcggctccgccgagatcaaaagtccttaaaaggaagaggcaacaccc	-	*
CY052691	Human H1N1pdm2009 IAVs	Human	H1N1	pdm	2009	USA	A/Texas/45071524/2009	ggtgatgccccattccttgatcggctccgccgagatcaaaagtccttaaaaggaagaggcaacaccc	-	*
CY052531	Human H1N1pdm2009 IAVs	Human	H1N1	pdm	2009	USA	A/Texas/45103998/2009	ggtgatgccccattccttgatcggctccgccgagatcaaaagtccttaaaaggaagaggcaacaccc	-	*

CY066875	Human H1N1pdm2009 IAVs	Human	H1N1	pdm	2009	USA	A/District_of_Columbia/INS228/2009	ggtgatgccccattccttgatcggctccgccgagatca aaagtccttaaaaggaagaggcaacaccc	-	*
CY058384	Human H1N1pdm2009 IAVs	Human	H1N1	pdm	2009	USA	A/Wisconsin/629_D00780/2009	ggtgatgccccattccttgatcggctccgccgagatca aaagtccttaaaaggaagaggcaacaccc	-	*
CY045073	Human H1N1pdm2009 IAVs	Human	H1N1	pdm	2009	USA	A/New_York/4290/2009	ggtgatgccccattccttgatcggctccgccgagatca aaagtccttaaaaggaagaggcaacaccc	-	*
CY051603	Human H1N1pdm2009 IAVs	Human	H1N1	pdm	2009	USA	A/New_York/4566/2009	ggtgatgccccattccttgatcggctccgccgagatca aaagtccttaaaaggaagaggcaacaccc	-	*
CY062959	Human H1N1pdm2009 IAVs	Human	H1N1	pdm	2009	Spain	A/Terrassa/INS174/2009	ggtgatgccccattccttgatcggctccgccgagatca aaagtccttaaaaggaagaggcaacaccc	-	*
CY052771	Human H1N1pdm2009 IAVs	Human	H1N1	pdm	2009	USA	A/Texas/44282651/2009	ggtgatgccccattccttgatcggctccgccgagatca aaagtccttaaaaggaagaggcaacaccc	-	*
GQ267854	Human H1N1pdm2009 IAVs	Human	H1N1	pdm	2009	Japan	A/Shiga/1/2009	ggtgatgccccattccttgatcggctccgccgagatca aaagtccttaaaaggaagaggcaacaccc	-	*
CY063095	Human H1N1pdm2009 IAVs	Human	H1N1	pdm	2009	USA	A/California/VRDL90/2009	ggtgatgccccattccttgatcggctccgccgagatca aaagtccttaaaaggaagaggcaacaccc	-	*
CY056048	Human H1N1pdm2009 IAVs	Human	H1N1	pdm	2009	USA	A/District_of_Columbia/INS18/2009	ggtgatgccccattccttgatcggctccgccgagatca aaagtccttaaaaggaagaggcaacaccc	-	*
JF714048	Human H1N1pdm2009 IAVs	Swine	H1N1		2009	Canada	A/swine/MB/25_3/2009	ggtgatgccccattccttgatcggctccgccgagatca aaagtccttaaaaggaagaggcaacaccc	-	*
AB538393	Human H1N1pdm2009 IAVs	Human	H1N1	pdm	2009	Japan	A/Nagano/RC1/2009	ggtgatgccccattccttgatcggctccgccgagatca aaagtccttaaaaggaagaggcaacaccc	-	*
CY040850	Human H1N1pdm2009 IAVs	Human	H1N1	pdm	2009	USA	A/New_York/3263/2009	ggtgatgccccattccttgatcggctccgccgagatca aaagtccttaaaaggaagaggcaacaccc	-	*
CY058018	Human H1N1pdm2009 IAVs	Human	H1N1	pdm	2009	USA	A/Wisconsin/629_D00672/2009	ggtgatgccccattccttgatcggctccgccgagatca aaagtccttaaaaggaagaggcaacaccc	-	*
CY069086	Human H1N1pdm2009 IAVs	Human	H1N1	pdm	2009	Guam	A/Guam/NHRC0025/2009	ggtgatgccccattccttgatcggctccgccgagatca aaagtccttaaaaggaagaggcaacaccc	-	*
CY069110	Human H1N1pdm2009 IAVs	Human	H1N1	pdm	2009	Guam	A/Guam/NHRC0028/2009	ggtgatgccccattccttgatcggctccgccgagatca aaagtccttaaaaggaagaggcaacaccc	-	*
CY065991	Human H1N1pdm2009 IAVs	Human	H1N1	pdm	2009	Mongolia	A/Ulaanbaatar/6525/2009	ggtgatgccccattccttgatcggctccgccgagatca aaagtccttaaaaggaagaggcaacaccc	-	*
CY046607	Human H1N1pdm2009 IAVs	Human	H1N1	pdm	2009	USA	A/Wisconsin/629_D00487/2009	ggtgatgccccattccttgatcggctccgccgagatca aaagtccttaaaaggaagaggcaacaccc	-	*
CY075463	Human H1N1pdm2009 IAVs	Human	H1N1	pdm	2009	Chile	A/Chile/4406/2009	ggtgatgccccattccttgatcggctccgccgagatca aaagtccttaaaaggaagaggcaacaccc	-	*
CY071043	Human H1N1pdm2009 IAVs	Human	H1N1	pdm	2009	USA	A/New_York/NHRC0003/2009	ggtgatgccccattccttgatcggctccgccgagatca aaagtccttaaaaggaagaggcaacaccc	-	*
CY083717	Human H1N1pdm2009 IAVs	Human	H1N1	pdm	2009	Spain	A/Madrid/INS112/2009	ggtgatgccccattccttgatcggctccgccgagatca aaagtccttaaaaggaagaggcaacaccc	-	*
CY062767	Human H1N1pdm2009 IAVs	Human	H1N1	pdm	2009	Spain	A/Madrid/INS134/2009	ggtgatgccccattccttgatcggctccgccgagatca aaagtccttaaaaggaagaggcaacaccc	-	*
CY066851	Human H1N1pdm2009 IAVs	Human	H1N1	pdm	2009	Spain	A/Madrid/INS225/2009	ggtgatgccccattccttgatcggctccgccgagatca aaagtccttaaaaggaagaggcaacaccc	-	*
CY069134	Human H1N1pdm2009 IAVs	Human	H1N1	pdm	2009	Spain	A/Madrid/INS299/2009	ggtgatgccccattccttgatcggctccgccgagatca aaagtccttaaaaggaagaggcaacaccc	-	*
CY069142	Human H1N1pdm2009 IAVs	Human	H1N1		2009	Spain	A/Madrid/INS300/2009	ggtgatgccccattccttgatcggctccgccgagatca aaagtccttaaaaggaagaggcaacaccc	-	*
CY069118	Human H1N1pdm2009 IAVs	Human	H1N1	pdm	2009	Spain	A/Madrid/INS296/2009	ggtgatgccccattccttgatcggctccgccgagatca aaagtccttaaaaggaagaggcaacaccc	-	*

CY046343	Human H1N1pdm2009 IAVs	Human	H1N1	pdm	2009	USA	A/Wisconsin/629_D02455/2009	ggtgatgccccattccttgatcggctccgccgagatcaaaagtccttaaaaggaagaggcaacaccc	-	*
CY066579	Human H1N1pdm2009 IAVs	Human	H1N1	pdm	2009	USA	A/San_Diego/INS202/2009	ggtgatgccccattccttgatcggctccgccgagatcaaaagtccttaaaaggaagaggcaacaccc	-	*
CY062679	Human H1N1pdm2009 IAVs	Human	H1N1	pdm	2009	Spain	A/Madrid/INS113/2009	ggtgatgccccattccttgatcggctccgccgagatcaaaagtccttaaaaggaagaggcaacaccc	-	*
CY072714	Human H1N1pdm2009 IAVs	Human	H1N1	pdm	2009	Nicaragua	A/Managua/5307.01/2009	ggtgatgccccattccttgatcggctccgccgagatcaaaagtccttaaaaggaagaggcaacaccc	-	*
CY100460	Human H1N1pdm2009 IAVs	Human	H1N1	pdm	2009	Mexico	A/Mexico_City/IBT23/2009	ggtgatgccccattccttgatcggctccgccgagatcaaaagtccttaaaaggaagaggcaacaccc	-	*
CY057386	Human H1N1pdm2009 IAVs	Human	H1N1	pdm	2009	USA	A/Wisconsin/629_D02192/2009	ggtgatgccccattccttgatcggctccgccgagatcaaaagtccttaaaaggaagaggcaacaccc	-	*
CY063506	Human H1N1pdm2009 IAVs	Human	H1N1	pdm	2009	USA	A/Boston/113/2009	ggtgatgccccattccttgatcggctccgccgagatcaaaagtccttaaaaggaagaggcaacaccc	-	*
KC780345	Human H1N1pdm2009 IAVs	Human	H1N1	pdm	2009	USA	A/New_York/102/2009	ggtgatgccccattccttgatcggctccgccgagatcaaaagtccttaaaaggaagaggcaacaccc	-	*
CY107208	Human H1N1pdm2009 IAVs	Human	H1N1	pdm	2009	United_Kingdom	A/Scotland/Paisley_425827/2009	ggtgatgccccattccttgatcggctccgccgagatcaaaagtccttaaaaggaagaggcaacaccc	-	*
CY054943	Human H1N1pdm2009 IAVs	Human	H1N1	pdm	2009	USA	A/California/VRDL48/2009	ggtgatgccccattccttgatcggctccgccgagatcaaaagtccttaaaaggaagaggcaacaccc	-	*
CY088605	Human H1N1pdm2009 IAVs	Human	H1N1	pdm	2009	USA	A/Boston/705/2009	ggtgatgccccattccttgatcggctccgccgagatcaaaagtccttaaaaggaagaggcaacaccc	-	*
CY058560	Human H1N1pdm2009 IAVs	Human	H1N1	pdm	2009	Nicaragua	A/Managua/2093.01/2009	ggtgatgccccattccttgatcggctccgccgagatcaaaagtccttaaaaggaagaggcaacaccc	-	*
CY072698	Human H1N1pdm2009 IAVs	Human	H1N1	pdm	2009	Nicaragua	A/Managua/3013.01/2009	ggtgatgccccattccttgatcggctccgccgagatcaaaagtccttaaaaggaagaggcaacaccc	-	*
CY072690	Human H1N1pdm2009 IAVs	Human	H1N1	pdm	2009	Nicaragua	A/Managua/554.01/2009	ggtgatgccccattccttgatcggctccgccgagatcaaaagtccttaaaaggaagaggcaacaccc	-	*
KJ690428	Human H1N1pdm2009 IAVs	Human	H1N1	pdm	2009	Uganda	A/Uganda/MUWRP_059/2009	ggtgatgccccattccttgatcggctccgccgagatcaaaagtccttaaaaggaagaggcaacaccc	-	*
KJ690412	Human H1N1pdm2009 IAVs	Human	H1N1		2009	Uganda	A/Uganda/MUWRP_066/2009	ggtgatgccccattccttgatcggctccgccgagatcaaaagtccttaaaaggaagaggcaacaccc	-	*
KJ690507	Human H1N1pdm2009 IAVs	Human	H1N1	pdm	2009	Uganda	A/Uganda/MUWRP_067/2009	ggtgatgccccattccttgatcggctccgccgagatcaaaagtccttaaaaggaagaggcaacaccc	-	*
KJ690467	Human H1N1pdm2009 IAVs	Human	H1N1	pdm	2009	Uganda	A/Uganda/MUWRP_069/2009	ggtgatgccccattccttgatcggctccgccgagatcaaaagtccttaaaaggaagaggcaacaccc	-	*
KJ690460	Human H1N1pdm2009 IAVs	Human	H1N1	pdm	2009	Uganda	A/Uganda/MUWRP_111/2009	ggtgatgccccattccttgatcggctccgccgagatcaaaagtccttaaaaggaagaggcaacaccc	-	*
KJ690483	Human H1N1pdm2009 IAVs	Human	H1N1	pdm	2009	Uganda	A/Uganda/MUWRP_098/2009	ggtgatgccccattccttgatcggctccgccgagatcaaaagtccttaaaaggaagaggcaacaccc	-	*
CY058256	Human H1N1pdm2009 IAVs	Human	H1N1	pdm	2009	Nicaragua	A/Managua/3244.01/2009	ggtgatgccccattccttgatcggctccgccgagatcaaaagtccttaaaaggaagaggcaacaccc	-	*
CY056399	Human H1N1pdm2009 IAVs	Human	H1N1	pdm	2009	USA	A/New_York/4925/2009	ggtgatgccccattccttgatcggctccgccgagatcaaaagtccttaaaaggaagaggcaacaccc	-	*
FN434447	Human H1N1pdm2009 IAVs	Human	H1N1	pdm	2009	Canada	A/Quebec/147023/2009	ggtgatgccccattccttgatcggctccgccgagatcaaaagtccttaaaaggaagaggcaacaccc	-	*
FN434455	Human H1N1pdm2009 IAVs	Human	H1N1	pdm	2009	Canada	A/Quebec/147365/2009	ggtgatgccccattccttgatcggctccgccgagatcaaaagtccttaaaaggaagaggcaacaccc	-	*
CY057842	Human H1N1pdm2009 IAVs	Human	H1N1		2009	USA	A/Wisconsin/629_D00128/2009	ggtgatgccccattccttgatcggctccgccgagatcaaaagtccttaaaaggaagaggcaacaccc	-	*

CY062038	Human H1N1pdm2009 IAVs	Human	H1N1	pdm	2009	USA	A/New_York/0259/2009	ggtgatgccccattccttgatcggctccgccgagatca aaagtccttaaaaggaagaggcaacaccc	-	*
CY061982	Human H1N1pdm2009 IAVs	Human	H1N1		2009	USA	A/New_York/6977/2009	ggtgatgccccattccttgatcggctccgccgagatca aaagtccttaaaaggaagaggcaacaccc	-	*
CY062030	Human H1N1pdm2009 IAVs	Human	H1N1	pdm	2009	USA	A/New_York/7236/2009	ggtgatgccccattccttgatcggctccgccgagatca aaagtccttaaaaggaagaggcaacaccc	-	*
CY089199	Human H1N1pdm2009 IAVs	Human	H1N1	pdm	2009	USA	A/Boston/593/2009	ggtgatgccccattccttgatcggctccgccgagatca aaagtccttaaaaggaagaggcaacaccc	-	*
CY089239	Human H1N1pdm2009 IAVs	Human	H1N1	pdm	2009	USA	A/Boston/633/2009	ggtgatgccccattccttgatcggctccgccgagatca aaagtccttaaaaggaagaggcaacaccc	-	*
CY075568	Human H1N1pdm2009 IAVs	Human	H1N1	pdm	2009	USA	A/Boston/655/2009	ggtgatgccccattccttgatcggctccgccgagatca aaagtccttaaaaggaagaggcaacaccc	-	*
CY089279	Human H1N1pdm2009 IAVs	Human	H1N1	pdm	2009	USA	A/Boston/665/2009	ggtgatgccccattccttgatcggctccgccgagatca aaagtccttaaaaggaagaggcaacaccc	-	*
CY066899	Human H1N1pdm2009 IAVs	Human	H1N1	pdm	2009	USA	A/District_of_Columbia/INS231/2009	ggtgatgccccattccttgatcggctccgccgagatca aaagtccttaaaaggaagaggcaacaccc	-	*
CY056088	Human H1N1pdm2009 IAVs	Human	H1N1	pdm	2009	USA	A/District_of_Columbia/INS25/2009	ggtgatgccccattccttgatcggctccgccgagatca aaagtccttaaaaggaagaggcaacaccc	-	*
CY056104	Human H1N1pdm2009 IAVs	Human	H1N1	pdm	2009	USA	A/District_of_Columbia/INS28/2009	ggtgatgccccattccttgatcggctccgccgagatca aaagtccttaaaaggaagaggcaacaccc	-	*
CY056871	Human H1N1pdm2009 IAVs	Human	H1N1	pdm	2009	USA	A/District_of_Columbia/INS44/2009	ggtgatgccccattccttgatcggctccgccgagatca aaagtccttaaaaggaagaggcaacaccc	-	*
CY062647	Human H1N1pdm2009 IAVs	Human	H1N1	pdm	2009	USA	A/District_of_Columbia/INS45/2009	ggtgatgccccattccttgatcggctccgccgagatca aaagtccttaaaaggaagaggcaacaccc	-	*
CY083866	Human H1N1pdm2009 IAVs	Human	H1N1	pdm	2009	USA	A/District_of_Columbia/INS47/2009	ggtgatgccccattccttgatcggctccgccgagatca aaagtccttaaaaggaagaggcaacaccc	-	*
CY056527	Human H1N1pdm2009 IAVs	Human	H1N1	pdm	2009	USA	A/New_York/5130/2009	ggtgatgccccattccttgatcggctccgccgagatca aaagtccttaaaaggaagaggcaacaccc	-	*
CY056583	Human H1N1pdm2009 IAVs	Human	H1N1	pdm	2009	USA	A/New_York/5782/2009	ggtgatgccccattccttgatcggctccgccgagatca aaagtccttaaaaggaagaggcaacaccc	-	*
CY056591	Human H1N1pdm2009 IAVs	Human	H1N1	pdm	2009	USA	A/New_York/5886/2009	ggtgatgccccattccttgatcggctccgccgagatca aaagtccttaaaaggaagaggcaacaccc	-	*
CY056615	Human H1N1pdm2009 IAVs	Human	H1N1	pdm	2009	USA	A/New_York/5976/2009	ggtgatgccccattccttgatcggctccgccgagatca aaagtccttaaaaggaagaggcaacaccc	-	*
CY056743	Human H1N1pdm2009 IAVs	Human	H1N1	pdm	2009	USA	A/New_York/6571/2009	ggtgatgccccattccttgatcggctccgccgagatca aaagtccttaaaaggaagaggcaacaccc	-	*
CY056791	Human H1N1pdm2009 IAVs	Human	H1N1	pdm	2009	USA	A/New_York/6775/2009	ggtgatgccccattccttgatcggctccgccgagatca aaagtccttaaaaggaagaggcaacaccc	-	*
CY056847	Human H1N1pdm2009 IAVs	Human	H1N1	pdm	2009	USA	A/New_York/6868/2009	ggtgatgccccattccttgatcggctccgccgagatca aaagtccttaaaaggaagaggcaacaccc	-	*
KC780207	Human H1N1pdm2009 IAVs	Human	H1N1	pdm	2009	USA	A/Maine/13/2009	ggtgatgccccattccttgatcggctccgccgagatca aaagtccttaaaaggaagaggcaacaccc	-	*
KC780558	Human H1N1pdm2009 IAVs	Human	H1N1	pdm	2009	USA	A/West_Virginia/12/2009	ggtgatgccccattccttgatcggctccgccgagatca aaagtccttaaaaggaagaggcaacaccc	-	*
KC780473	Human H1N1pdm2009 IAVs	Human	H1N1	pdm	2009	USA	A/Florida/25/2009	ggtgatgccccattccttgatcggctccgccgagatca aaagtccttaaaaggaagaggcaacaccc	-	*
CY084442	Human H1N1pdm2009 IAVs	Human	H1N1		2009	USA	A/New_York/6064/2009	ggtgatgccccattccttgatcggctccgccgagatca aaagtccttaaaaggaagaggcaacaccc	-	*
CY066891	Human H1N1pdm2009 IAVs	Human	H1N1	pdm	2009	USA	A/District_of_Columbia/INS230/2009	ggtgatgccccattccttgatcggctccgccgagatca aaagtccttaaaaggaagaggcaacaccc	-	*

CY066907	Human H1N1pdm2009 IAVs	Human	H1N1	pdm	2009	USA	A/District_of_Columbia/INS232/2009	ggtgatgccccattccttgatcggctccgccgagatcaaaagtccttaaaaggaagaggcaacaccc	-	*
CY057282	Human H1N1pdm2009 IAVs	Human	H1N1		2009	USA	A/New_York/5221/2009	ggtgatgccccattccttgatcggctccgccgagatcaaaagtccttaaaaggaagaggcaacaccc	-	*
CY083651	Human H1N1pdm2009 IAVs	Human	H1N1	pdm	2009	USA	A/District_of_Columbia/INS22/2009	ggtgatgccccattccttgatcggctccgccgagatcaaaagtccttaaaaggaagaggcaacaccc	-	*
CY056663	Human H1N1pdm2009 IAVs	Human	H1N1	pdm	2009	USA	A/New_York/6182/2009	ggtgatgccccattccttgatcggctccgccgagatcaaaagtccttaaaaggaagaggcaacaccc	-	*
CY061910	Human H1N1pdm2009 IAVs	Human	H1N1	pdm	2009	USA	A/New_York/6903/2009	ggtgatgccccattccttgatcggctccgccgagatcaaaagtccttaaaaggaagaggcaacaccc	-	*
CY065079	Human H1N1pdm2009 IAVs	Human	H1N1	pdm	2009	USA	A/New_York/7421/2009	ggtgatgccccattccttgatcggctccgccgagatcaaaagtccttaaaaggaagaggcaacaccc	-	*
CY056639	Human H1N1pdm2009 IAVs	Human	H1N1	pdm	2009	USA	A/New_York/6019/2009	ggtgatgccccattccttgatcggctccgccgagatcaaaagtccttaaaaggaagaggcaacaccc	-	*
CY056799	Human H1N1pdm2009 IAVs	Human	H1N1	pdm	2009	USA	A/New_York/6776/2009	ggtgatgccccattccttgatcggctccgccgagatcaaaagtccttaaaaggaagaggcaacaccc	-	*
CY061918	Human H1N1pdm2009 IAVs	Human	H1N1	pdm	2009	USA	A/New_York/6907/2009	ggtgatgccccattccttgatcggctccgccgagatcaaaagtccttaaaaggaagaggcaacaccc	-	*
CY054855	Human H1N1pdm2009 IAVs	Human	H1N1	pdm	2009	USA	A/California/VRDL32/2009	ggtgatgccccattccttgatcggctccgccgagatcaaaagtccttaaaaggaagaggcaacaccc	-	*
CY054895	Human H1N1pdm2009 IAVs	Human	H1N1	pdm	2009	USA	A/California/VRDL38/2009	ggtgatgccccattccttgatcggctccgccgagatcaaaagtccttaaaaggaagaggcaacaccc	-	*
CY054935	Human H1N1pdm2009 IAVs	Human	H1N1	pdm	2009	USA	A/California/VRDL45/2009	ggtgatgccccattccttgatcggctccgccgagatcaaaagtccttaaaaggaagaggcaacaccc	-	*
CY069070	Human H1N1pdm2009 IAVs	Human	H1N1	pdm	2009	Guam	A/Guam/NHRC0023/2009	ggtgatgccccattccttgatcggctccgccgagatcaaaagtccttaaaaggaagaggcaacaccc	-	*
CY119478	Human H1N1pdm2009 IAVs	Human	H1N1	pdm	2009	Malaysia	A/Malaysia/2139971/2009	ggtgatgccccattccttgatcggctccgccgagatcaaaagtccttaaaaggaagaggcaacaccc	-	*
CY072818	Human H1N1pdm2009 IAVs	Human	H1N1	pdm	2009	Nicaragua	A/Managua/3421.02/2009	ggtgatgccccattccttgatcggctccgccgagatcaaaagtccttaaaaggaagaggcaacaccc	-	*
CY066755	Human H1N1pdm2009 IAVs	Human	H1N1	pdm	2009	USA	A/Pensacola/INS210/2009	ggtgatgccccattccttgatcggctccgccgagatcaaaagtccttaaaaggaagaggcaacaccc	-	*
CY052635	Human H1N1pdm2009 IAVs	Human	H1N1	pdm	2009	USA	A/Texas/45061670/2009	ggtgatgccccattccttgatcggctccgccgagatcaaaagtccttaaaaggaagaggcaacaccc	-	*
CY052955	Human H1N1pdm2009 IAVs	Human	H1N1	pdm	2009	USA	A/Texas/45061755/2009	ggtgatgccccattccttgatcggctccgccgagatcaaaagtccttaaaaggaagaggcaacaccc	-	*
CY061566	Human H1N1pdm2009 IAVs	Human	H1N1	pdm	2009	USA	A/Texas/JMS367/2009	ggtgatgccccattccttgatcggctccgccgagatcaaaagtccttaaaaggaagaggcaacaccc	-	*
CY128167	Human H1N1pdm2009 IAVs	Human	H1N1	pdm	2009	Viet_Nam	A/Viet_Nam/13032011/2009	ggtgatgccccattccttgatcggctccgccgagatcaaaagtccttaaaaggaagaggcaacaccc	-	*
CY128047	Human H1N1pdm2009 IAVs	Human	H1N1	pdm	2009	Viet_Nam	A/Viet_Nam/15032002/2009	ggtgatgccccattccttgatcggctccgccgagatcaaaagtccttaaaaggaagaggcaacaccc	-	*
CY057914	Human H1N1pdm2009 IAVs	Human	H1N1	pdm	2009	USA	A/Wisconsin/629_D00134/2009	ggtgatgccccattccttgatcggctccgccgagatcaaaagtccttaaaaggaagaggcaacaccc	-	*
CY057007	Human H1N1pdm2009 IAVs	Human	H1N1	pdm	2009	USA	A/Wisconsin/629_D02367/2009	ggtgatgccccattccttgatcggctccgccgagatcaaaagtccttaaaaggaagaggcaacaccc	-	*
CY057362	Human H1N1pdm2009 IAVs	Human	H1N1		2009	USA	A/Wisconsin/629_D02485/2009	ggtgatgccccattccttgatcggctccgccgagatcaaaagtccttaaaaggaagaggcaacaccc	-	*
CY066659	Human H1N1pdm2009 IAVs	Human	H1N1	pdm	2009	Spain	A/Madrid/INS281/2009	ggtgatgccccattccttgatcggctccgccgagatcaaaagtccttaaaaggaagaggcaacaccc	-	*

CY083435	Human H1N1pdm2009 IAVs	Human	H1N1	pdm	2009	USA	A/San_Diego/WRAIR1668P/2009	ggtgatgccccattccttgatcggctccgccgagatca aaagtccttaaaaggaagaggcaacaccc	-	*
AB704522	Human H1N1pdm2009 IAVs	Human	H1N1		2009	Japan	A/Yamaguchi/247/2009	ggtgatgccccattccttgatcggctccgccgagatca aaagtccttaaaaggaagaggcaacaccc	-	*
CY071838	Human H1N1pdm2009 IAVs	Human	H1N1	pdm	2009	USA	A/South_Carolina/WRSP520/2009	ggtgatgccccattccttgatcggctccgccgagatca aaagtccttaaaaggaagaggcaacaccc	-	*
CY083403	Human H1N1pdm2009 IAVs	Human	H1N1	pdm	2009	USA	A/Great_Lakes/WRAIR1664P/2009	ggtgatgccccattccttgatcggctccgccgagatca aaagtccttaaaaggaagaggcaacaccc	-	*
CY054903	Human H1N1pdm2009 IAVs	Human	H1N1	pdm	2009	USA	A/California/VRDL39/2009	ggtgatgccccattccttgatcggctccgccgagatca aaagtccttaaaaggaagaggcaacaccc	-	*
CY118235	Human H1N1pdm2009 IAVs	Human	Mixed	pdm	2009	Malaysia	A/Malaysia/2124650/2009	ggtgatgccccattccttgatcggctccgccgagatca aaagtccttaaaaggaagaggcaacaccc	-	*
CY054719	Human H1N1pdm2009 IAVs	Human	H1N1	pdm	2009	USA	A/California/VRDL5/2009	ggtgatgccccattccttgatcggctccgccgagatca aaagtccttaaaaggaagaggcaacaccc	-	*
CY044076	Human H1N1pdm2009 IAVs	Human	H1N1	pdm	2009	USA	A/New_York/3702/2009	ggtgatgccccattccttgatcggctccgccgagatca aaagtccttaaaaggaagaggcaacaccc	-	*
CY051699	Human H1N1pdm2009 IAVs	Human	H1N1	pdm	2009	USA	A/New_York/4777/2009	ggtgatgccccattccttgatcggctccgccgagatca aaagtccttaaaaggaagaggcaacaccc	-	*
CY050851	Human H1N1pdm2009 IAVs	Human	H1N1	pdm	2009	Mexico	A/Mexico_City/007/2009	ggtgatgccccattccttgatcggctccgccgagatca aaagtccttaaaaggaagaggcaacaccc	-	*
CY062532	Human H1N1pdm2009 IAVs	Human	H1N1		2009	Mexico	A/Mexico_city/CIA7/2009	ggtgatgccccattccttgatcggctccgccgagatca aaagtccttaaaaggaagaggcaacaccc	-	*
CY062494	Human H1N1pdm2009 IAVs	Human	H1N1	pdm	2009	Mexico	A/Mexico_city/CIA1/2009	ggtgatgccccattccttgatcggctccgccgagatca aaagtccttaaaaggaagaggcaacaccc	-	*
CY064414	Human H1N1pdm2009 IAVs	Human	H1N1	pdm	2009	Mexico	A/Mexico_city/CIA10/2009	ggtgatgccccattccttgatcggctccgccgagatca aaagtccttaaaaggaagaggcaacaccc	-	*
CY064421	Human H1N1pdm2009 IAVs	Human	H1N1	pdm	2009	Mexico	A/Mexico_city/CIA11/2009	ggtgatgccccattccttgatcggctccgccgagatca aaagtccttaaaaggaagaggcaacaccc	-	*
CY062544	Human H1N1pdm2009 IAVs	Human	H1N1	pdm	2009	Mexico	A/Mexico_city/CIA9/2009	ggtgatgccccattccttgatcggctccgccgagatca aaagtccttaaaaggaagaggcaacaccc	-	*
CY045155	Human H1N1pdm2009 IAVs	Human	H1N1	pdm	2009	USA	A/New_York/3655/2009	ggtgatgccccattccttgatcggctccgccgagatca aaagtccttaaaaggaagaggcaacaccc	-	*
CY056024	Human H1N1pdm2009 IAVs	Human	H1N1	pdm	2009	USA	A/San_Diego/INS12/2009	ggtgatgccccattccttgatcggctccgccgagatca aaagtccttaaaaggaagaggcaacaccc	-	*
CY122847	Human H1N1pdm2009 IAVs	Human	H1N1	pdm	2009	Singapore	A/Singapore/GP2252/2009	ggtgatgccccattccttgatcggctccgccgagatca aaagtccttaaaaggaagaggcaacaccc	-	*
CY122971	Human H1N1pdm2009 IAVs	Human	H1N1	pdm	2009	Singapore	A/Singapore/GP2982/2009	ggtgatgccccattccttgatcggctccgccgagatca aaagtccttaaaaggaagaggcaacaccc	-	*
CY123296	Human H1N1pdm2009 IAVs	Human	H1N1	pdm	2009	Singapore	A/Singapore/GP4556/2009	ggtgatgccccattccttgatcggctccgccgagatca aaagtccttaaaaggaagaggcaacaccc	-	*
CY062206	Human H1N1pdm2009 IAVs	Human	H1N1	pdm	2009	USA	A/California/VRDL80/2009	ggtgatgccccattccttgatcggctccgccgagatca aaagtccttaaaaggaagaggcaacaccc	-	*
JN596863	Human H1N1pdm2009 IAVs	Human	H1N1	pdm	2009	Russia	A/Orenburg/01/2009	ggtgatgccccattccttgatcggctccgccgagatca aaagtccttaaaaggaagaggcaacaccc	-	*
GU211231	Human H1N1pdm2009 IAVs	Human	H1N1	pdm	2009	Russia	A/Russia/01/2009	ggtgatgccccattccttgatcggctccgccgagatca aaagtccttaaaaggaagaggcaacaccc	-	*
GU560012	Human H1N1pdm2009 IAVs	Human	H1N1		2009	Russia	A/Tomsk/05/2009	ggtgatgccccattccttgatcggctccgccgagatca aaagtccttaaaaggaagaggcaacaccc	-	*
GU560020	Human H1N1pdm2009 IAVs	Human	H1N1	pdm	2009	Russia	A/Tomsk/06/2009	ggtgatgccccattccttgatcggctccgccgagatca aaagtccttaaaaggaagaggcaacaccc	-	*

KC782218	Human H1N1pdm2009 IAVs	Human	H1N1	pdm	2009	USA	A/South_Carolina/18/2009	ggtgatgccccattccttgatcggctccgccgagatca aaagtccttaaaaggaagaggcaacaccc	-	*
KC782203	Human H1N1pdm2009 IAVs	Human	H1N1	pdm	2009	USA	A/South_Carolina/18/2009	ggtgatgccccattccttgatcggctccgccgagatca aaagtccttaaaaggaagaggcaacaccc	-	*
KC780077	Human H1N1pdm2009 IAVs	Human	H1N1	pdm	2009	USA	A/South_Carolina/18/2009	ggtgatgccccattccttgatcggctccgccgagatca aaagtccttaaaaggaagaggcaacaccc	-	*
CY047748	Human H1N1pdm2009 IAVs	Human	H1N1	pdm	2009	Taiwan	A/Taiwan/526/2009	ggtgatgccccattccttgatcggctccgccgagatca aaagtccttaaaaggaagaggcaacaccc	-	*
CY057690	Human H1N1pdm2009 IAVs	Human	H1N1	pdm	2009	USA	A/Wisconsin/629_S1388/2009	ggtgatgccccattccttgatcggctccgccgagatca aaagtccttaaaaggaagaggcaacaccc	-	*
CY088691	Human H1N1pdm2009 IAVs	Human	H1N1	pdm	2009	India	A/Mum/NIV5442/2009	ggtgatgccccattccttgatcggctccgccgagatca aaagtccttaaaaggaagaggcaacaccc	-	*
CY095767	Human H1N1pdm2009 IAVs	Human	H1N1	pdm	2009	China	A/Zhejiang/3/2009	ggtgatgccccattccttgatcggctccgccgagatca aaagtccttaaaaggaagaggcaacaccc	-	*
KF411270	Human H1N1pdm2009 IAVs	Human	H1N1	pdm	2009	China	A/Qingdao/1609/2009	ggtgatgccccattccttgatcggctccgccgagatca aaagtccttaaaaggaagaggcaacaccc	-	*
KF411271	Human H1N1pdm2009 IAVs	Human	H1N1	pdm	2009	China	A/Qingdao/1610/2009	ggtgatgccccattccttgatcggctccgccgagatca aaagtccttaaaaggaagaggcaacaccc	-	*
HM569695	Human H1N1pdm2009 IAVs	Human	H1N1	pdm	2009	Argentina	A/Argentina/19656/2009	ggtgatgccccattccttgatcggctccgccgagatca aaagtccttaaaaggaagaggcaacaccc	-	*
CY047855	Human H1N1pdm2009 IAVs	Human	H1N1	pdm	2009	Argentina	A/Argentina/8673/2009	ggtgatgccccattccttgatcggctccgccgagatca aaagtccttaaaaggaagaggcaacaccc	-	*
CY064768	Human H1N1pdm2009 IAVs	Human	H1N1	pdm	2009	USA	A/District_of_Columbia/INS32/2009	ggtgatgccccattccttgatcggctccgccgagatca aaagtccttaaaaggaagaggcaacaccc	-	*
CY047362	Human H1N1pdm2009 IAVs	Human	H1N1		2009	USA	A/New_York/3741/2009	ggtgatgccccattccttgatcggctccgccgagatca aaagtccttaaaaggaagaggcaacaccc	-	*
CY051811	Human H1N1pdm2009 IAVs	Human	H1N1	pdm	2009	USA	A/New_York/4870/2009	ggtgatgccccattccttgatcggctccgccgagatca aaagtccttaaaaggaagaggcaacaccc	-	*
CY056623	Human H1N1pdm2009 IAVs	Human	H1N1	pdm	2009	USA	A/New_York/5988/2009	ggtgatgccccattccttgatcggctccgccgagatca aaagtccttaaaaggaagaggcaacaccc	-	*
CY061958	Human H1N1pdm2009 IAVs	Human	H1N1	pdm	2009	USA	A/New_York/6945/2009	ggtgatgccccattccttgatcggctccgccgagatca aaagtccttaaaaggaagaggcaacaccc	-	*
CY047378	Human H1N1pdm2009 IAVs	Human	H1N1	pdm	2009	USA	A/New_York/3753/2009	ggtgatgccccattccttgatcggctccgccgagatca aaagtccttaaaaggaagaggcaacaccc	-	*
CY122915	Human H1N1pdm2009 IAVs	Human	H1N1	pdm	2009	Singapore	A/Singapore/GP2658/2009	ggtgatgccccattccttgatcggctccgccgagatca aaagtccttaaaaggaagaggcaacaccc	-	*
CY089191	Human H1N1pdm2009 IAVs	Human	H1N1	pdm	2009	USA	A/Boston/583/2009	ggtgatgccccattccttgatcggctccgccgagatca aaagtccttaaaaggaagaggcaacaccc	-	*
CY089311	Human H1N1pdm2009 IAVs	Human	H1N1	pdm	2009	USA	A/Boston/673/2009	ggtgatgccccattccttgatcggctccgccgagatca aaagtccttaaaaggaagaggcaacaccc	-	*
CY075624	Human H1N1pdm2009 IAVs	Human	H1N1	pdm	2009	USA	A/Boston/698/2009	ggtgatgccccattccttgatcggctccgccgagatca aaagtccttaaaaggaagaggcaacaccc	-	*
CY092792	Human H1N1pdm2009 IAVs	Human	mixed		2009	USA	A/California/VRDL364/2009	ggtgatgccccattccttgatcggctccgccgagatca aaagtccttaaaaggaagaggcaacaccc	-	*
CY056192	Human H1N1pdm2009 IAVs	Human	H1N1		2009	USA	A/District_of_Columbia/INS46/2009	ggtgatgccccattccttgatcggctccgccgagatca aaagtccttaaaaggaagaggcaacaccc	-	*
CY056487	Human H1N1pdm2009 IAVs	Human	H1N1	pdm	2009	USA	A/New_York/4995/2009	ggtgatgccccattccttgatcggctccgccgagatca aaagtccttaaaaggaagaggcaacaccc	-	*
CY056839	Human H1N1pdm2009 IAVs	Human	H1N1	pdm	2009	USA	A/New_York/6864/2009	ggtgatgccccattccttgatcggctccgccgagatca aaagtccttaaaaggaagaggcaacaccc	-	*

CY056855	Human H1N1pdm2009 IAVs	Human	H1N1	pdm	2009	USA	A/New_York/6909/2009	ggtgatgccccattccttgatcggctccgccgagatca aaagtccttaaaaggaagaggcaacaccc	-	*
KC781039	Human H1N1pdm2009 IAVs	Human	H1N1	pdm	2009	USA	A/Massachusetts/31/2009	ggtgatgccccattccttgatcggctccgccgagatca aaagtccttaaaaggaagaggcaacaccc	-	*
CY056783	Human H1N1pdm2009 IAVs	Human	H1N1	pdm	2009	USA	A/New_York/6675/2009	ggtgatgccccattccttgatcggctccgccgagatca aaagtccttaaaaggaagaggcaacaccc	-	*
HM567764	Human H1N1pdm2009 IAVs	Human	H1N1	pdm	2009	United_Kingdom	A/England/380/2009	ggtgatgccccattccttgatcggctccgccgagatca aaagtccttaaaaggaagaggcaacaccc	-	*
CY111751	Human H1N1pdm2009 IAVs	Human	H1N1	pdm	2009	Hong_Kong	A/Hong_Kong/H090_701_V10/2009	ggtgatgccccattccttgatcggctccgccgagatca aaagtccttaaaaggaagaggcaacaccc	-	*
CY120201	Human H1N1pdm2009 IAVs	Human	H1N1	pdm	2009	Hong_Kong	A/Hong_Kong/H090_701_V10/2009	ggtgatgccccattccttgatcggctccgccgagatca aaagtccttaaaaggaagaggcaacaccc	-	*
CY106980	Human H1N1pdm2009 IAVs	Human	H1N1	pdm	2009	Hong_Kong	A/Hong_Kong/H090_770_V10/2009	ggtgatgccccattccttgatcggctccgccgagatca aaagtccttaaaaggaagaggcaacaccc	-	*
CY120409	Human H1N1pdm2009 IAVs	Human	H1N1	pdm	2009	Hong_Kong	A/Hong_Kong/H090_771_V10/2009	ggtgatgccccattccttgatcggctccgccgagatca aaagtccttaaaaggaagaggcaacaccc	-	*
CY111999	Human H1N1pdm2009 IAVs	Human	H1N1	pdm	2009	Hong_Kong	A/Hong_Kong/H090_771_V10/2009	ggtgatgccccattccttgatcggctccgccgagatca aaagtccttaaaaggaagaggcaacaccc	-	*
CY112007	Human H1N1pdm2009 IAVs	Human	H1N1	pdm	2009	Hong_Kong	A/Hong_Kong/H090_771_V20/2009	ggtgatgccccattccttgatcggctccgccgagatca aaagtccttaaaaggaagaggcaacaccc	-	*
HM189601	Human H1N1pdm2009 IAVs	Swine	H1N1		2009	South_Korea	A/swine/Korea/SCJ11/2009	ggtgatgccccattccttgatcggctccgccgagatca aaagtccttaaaaggaagaggcaacaccc	-	*
HM189602	Human H1N1pdm2009 IAVs	Swine	H1N1		2009	South_Korea	A/swine/Korea/SCJ12/2009	ggtgatgccccattccttgatcggctccgccgagatca aaagtccttaaaaggaagaggcaacaccc	-	*
HM189603	Human H1N1pdm2009 IAVs	Swine	H1N1		2009	South_Korea	A/swine/Korea/SCJ13/2009	ggtgatgccccattccttgatcggctccgccgagatca aaagtccttaaaaggaagaggcaacaccc	-	*
HM189604	Human H1N1pdm2009 IAVs	Swine	H1N1		2009	South_Korea	A/swine/Korea/SCJ20/2009	ggtgatgccccattccttgatcggctccgccgagatca aaagtccttaaaaggaagaggcaacaccc	-	*
HM189605	Human H1N1pdm2009 IAVs	Swine	H1N1		2009	South_Korea	A/swine/Korea/SCJ26/2009	ggtgatgccccattccttgatcggctccgccgagatca aaagtccttaaaaggaagaggcaacaccc	-	*
CY119422	Human H1N1pdm2009 IAVs	Human	H1N1		2009	Malaysia	A/Malaysia/2089888/2009	ggtgatgccccattccttgatcggctccgccgagatca aaagtccttaaaaggaagaggcaacaccc	-	*
CY072738	Human H1N1pdm2009 IAVs	Human	H1N1	pdm	2009	Nicaragua	A/Managua/1419.02/2009	ggtgatgccccattccttgatcggctccgccgagatca aaagtccttaaaaggaagaggcaacaccc	-	*
CY072922	Human H1N1pdm2009 IAVs	Human	H1N1		2009	Nicaragua	A/Managua/4972.02/2009	ggtgatgccccattccttgatcggctccgccgagatca aaagtccttaaaaggaagaggcaacaccc	-	*
CY072242	Human H1N1pdm2009 IAVs	Human	H1N1		2009	Germany	A/Frankfurt/INS305/2009	ggtgatgccccattccttgatcggctccgccgagatca aaagtccttaaaaggaagaggcaacaccc	-	*
CY088617	Human H1N1pdm2009 IAVs	Human	H1N1	pdm	2009	Germany	A/Frankfurt/INS93/2009	ggtgatgccccattccttgatcggctccgccgagatca aaagtccttaaaaggaagaggcaacaccc	-	*
CY055896	Human H1N1pdm2009 IAVs	Human	H1N1	pdm	2009	Australia	A/Australia/70/2009	ggtgatgccccattccttgatcggctccgccgagatca aaagtccttaaaaggaagaggcaacaccc	-	*
CY064488	Human H1N1pdm2009 IAVs	Human	H1N1	pdm	2009	USA	A/Boston/108/2009	ggtgatgccccattccttgatcggctccgccgagatca aaagtccttaaaaggaagaggcaacaccc	-	*
CY069928	Human H1N1pdm2009 IAVs	Human	H1N1	pdm	2009	United_Kingdom	A/England/417/2009	ggtgatgccccattccttgatcggctccgccgagatca aaagtccttaaaaggaagaggcaacaccc	-	*
CY069936	Human H1N1pdm2009 IAVs	Human	H1N1	pdm	2009	United_Kingdom	A/England/425/2009	ggtgatgccccattccttgatcggctccgccgagatca aaagtccttaaaaggaagaggcaacaccc	-	*
CY069944	Human H1N1pdm2009 IAVs	Human	H1N1	pdm	2009	United_Kingdom	A/England/438/2009	ggtgatgccccattccttgatcggctccgccgagatca aaagtccttaaaaggaagaggcaacaccc	-	*

CY069952	Human H1N1pdm2009 IAVs	Human	H1N1		2009	United_Kingdom	A/England/444/2009	ggtgatgccccattccttgatcggctccgccgagatca aaagtccttaaaaggaagaggcaacaccc	-	*
CY112031	Human H1N1pdm2009 IAVs	Human	H1N1	pdm	2009	Hong_Kong	A/Hong_Kong/H090_779_V10/2009	ggtgatgccccattccttgatcggctccgccgagatca aaagtccttaaaaggaagaggcaacaccc	-	*
CY120433	Human H1N1pdm2009 IAVs	Human	H1N1	pdm	2009	Hong_Kong	A/Hong_Kong/H090_779_V10/2009	ggtgatgccccattccttgatcggctccgccgagatca aaagtccttaaaaggaagaggcaacaccc	-	*
CY112039	Human H1N1pdm2009 IAVs	Human	H1N1	pdm	2009	Hong_Kong	A/Hong_Kong/H090_779_V12/2009	ggtgatgccccattccttgatcggctccgccgagatca aaagtccttaaaaggaagaggcaacaccc	-	*
CY120441	Human H1N1pdm2009 IAVs	Human	H1N1	pdm	2009	Hong_Kong	A/Hong_Kong/H090_779_V12/2009	ggtgatgccccattccttgatcggctccgccgagatca aaagtccttaaaaggaagaggcaacaccc	-	*
CY112047	Human H1N1pdm2009 IAVs	Human	H1N1	pdm	2009	Hong_Kong	A/Hong_Kong/H090_779_V20/2009	ggtgatgccccattccttgatcggctccgccgagatca aaagtccttaaaaggaagaggcaacaccc	-	*
CY120096	Human H1N1pdm2009 IAVs	Human	H1N1	pdm	2009	Hong_Kong	A/Hong_Kong/H090_779_V20/2009	ggtgatgccccattccttgatcggctccgccgagatca aaagtccttaaaaggaagaggcaacaccc	-	*
CY120449	Human H1N1pdm2009 IAVs	Human	H1N1	pdm	2009	Hong_Kong	A/Hong_Kong/H090_779_V21/2009	ggtgatgccccattccttgatcggctccgccgagatca aaagtccttaaaaggaagaggcaacaccc	-	*
CY112055	Human H1N1pdm2009 IAVs	Human	H1N1	pdm	2009	Hong_Kong	A/Hong_Kong/H090_779_V21/2009	ggtgatgccccattccttgatcggctccgccgagatca aaagtccttaaaaggaagaggcaacaccc	-	*
CY112063	Human H1N1pdm2009 IAVs	Human	H1N1	pdm	2009	Hong_Kong	A/Hong_Kong/H090_779_V22/2009	ggtgatgccccattccttgatcggctccgccgagatca aaagtccttaaaaggaagaggcaacaccc	-	*
CY120457	Human H1N1pdm2009 IAVs	Human	H1N1	pdm	2009	Hong_Kong	A/Hong_Kong/H090_779_V22/2009	ggtgatgccccattccttgatcggctccgccgagatca aaagtccttaaaaggaagaggcaacaccc	-	*
HM567844	Human H1N1pdm2009 IAVs	Human	H1N1	pdm	2009	Ireland	A/Ireland/4/2009	ggtgatgccccattccttgatcggctccgccgagatca aaagtccttaaaaggaagaggcaacaccc	-	*
CY057706	Human H1N1pdm2009 IAVs	Human	H1N1	pdm	2009	USA	A/Wisconsin/629_S1397/2009	ggtgatgccccattccttgatcggctccgccgagatca aaagtccttaaaaggaagaggcaacaccc	-	*
CY057698	Human H1N1pdm2009 IAVs	Human	H1N1	pdm	2009	USA	A/Wisconsin/629_S1398/2009	ggtgatgccccattccttgatcggctccgccgagatca aaagtccttaaaaggaagaggcaacaccc	-	*
CY060538	Human H1N1pdm2009 IAVs	Human	H1N1	pdm	2009	Canada	A/Ontario/237882/2009	ggtgatgccccattccttgatcggctccgccgagatca aaagtccttaaaaggaagaggcaacaccc	-	*
CY064696	Human H1N1pdm2009 IAVs	Human	H1N1	pdm	2009	USA	A/Boston/143/2009	ggtgatgccccattccttgatcggctccgccgagatca aaagtccttaaaaggaagaggcaacaccc	-	*
CY052883	Human H1N1pdm2009 IAVs	Human	H1N1	pdm	2009	USA	A/Texas/45024243/2009	ggtgatgccccattccttgatcggctccgccgagatca aaagtccttaaaaggaagaggcaacaccc	-	*
CY063287	Human H1N1pdm2009 IAVs	Human	H1N1	pdm	2009	USA	A/Wisconsin/629_D02008/2009	ggtgatgccccattccttgatcggctccgccgagatca aaagtccttaaaaggaagaggcaacaccc	-	*
CY147719	Human H1N1pdm2009 IAVs	Human	H1N1	pdm	2009	Mexico	A/Mexico/24024/2009	ggtgatgccccattccttgatcggctccgccgagatca aaagtccttaaaaggaagaggcaacaccc	-	*
CY046695	Human H1N1pdm2009 IAVs	Human	H1N1	pdm	2009	USA	A/Wisconsin/629_D00434/2009	ggtgatgccccattccttgatcggctccgccgagatca aaagtccttaaaaggaagaggcaacaccc	-	*
CY051147	Human H1N1pdm2009 IAVs	Human	H1N1	pdm	2009	USA	A/Wisconsin/629_D01734/2009	ggtgatgccccattccttgatcggctccgccgagatca aaagtccttaaaaggaagaggcaacaccc	-	*
CY046767	Human H1N1pdm2009 IAVs	Human	H1N1	pdm	2009	USA	A/Wisconsin/629_D02298/2009	ggtgatgccccattccttgatcggctccgccgagatca aaagtccttaaaaggaagaggcaacaccc	-	*
GQ232059	Human H1N1pdm2009 IAVs	Human	H1N1	pdm	2009	USA	A/North_Dakota/04/2009	ggtgatgccccattccttgatcggctccgccgagatca aaagtccttaaaaggaagaggcaacaccc	-	*
CY083627	Human H1N1pdm2009 IAVs	Human	H1N1	pdm	2009	Peru	A/Trujillo/WRAIR9309F/2009	ggtgatgccccattccttgatcggctccgccgagatca aaagtccttaaaaggaagaggcaacaccc	-	*
KC781322	Human H1N1pdm2009 IAVs	Human	H1N1		2009	USA	A/Mississippi/01/2009	ggtgatgccccattccttgatcggctccgccgagatca aaagtccttaaaaggaagaggcaacaccc	-	*

CY057498	Human H1N1pdm2009 IAVs	Human	H1N1	pdm	2009	USA	A/Wisconsin/629_D00287/2009	ggtgatgccccattccttgatcggctccgccgagatca aaagtccttaaaaggaagaggcaacaccc	-	*
CY057474	Human H1N1pdm2009 IAVs	Human	H1N1	pdm	2009	USA	A/Wisconsin/629_D00402/2009	ggtgatgccccattccttgatcggctccgccgagatca aaagtccttaaaaggaagaggcaacaccc	-	*
CY057426	Human H1N1pdm2009 IAVs	Human	H1N1	pdm	2009	USA	A/Wisconsin/629_D00485/2009	ggtgatgccccattccttgatcggctccgccgagatca aaagtccttaaaaggaagaggcaacaccc	-	*
CY057562	Human H1N1pdm2009 IAVs	Human	H1N1		2009	USA	A/Wisconsin/629_D00690/2009	ggtgatgccccattccttgatcggctccgccgagatca aaagtccttaaaaggaagaggcaacaccc	-	*
CY057410	Human H1N1pdm2009 IAVs	Human	H1N1	pdm	2009	USA	A/Wisconsin/629_D00832/2009	ggtgatgccccattccttgatcggctccgccgagatca aaagtccttaaaaggaagaggcaacaccc	-	*
CY057402	Human H1N1pdm2009 IAVs	Human	H1N1		2009	USA	A/Wisconsin/629_D01469/2009	ggtgatgccccattccttgatcggctccgccgagatca aaagtccttaaaaggaagaggcaacaccc	-	*
CY058624	Human H1N1pdm2009 IAVs	Human	H1N1	pdm	2009	USA	A/Wisconsin/629_S1433/2009	ggtgatgccccattccttgatcggctccgccgagatca aaagtccttaaaaggaagaggcaacaccc	-	*
CY057506	Human H1N1pdm2009 IAVs	Human	H1N1	pdm	2009	USA	A/Wisconsin/629_D00565/2009	ggtgatgccccattccttgatcggctccgccgagatca aaagtccttaaaaggaagaggcaacaccc	-	*
CY083068	Human H1N1pdm2009 IAVs	Human	H1N1	pdm	2009	Colombia	A/Bogota/WRAIR0435N/2009	ggtgatgccccattccttgatcggctccgccgagatca aaagtccttaaaaggaagaggcaacaccc	-	*
CY083076	Human H1N1pdm2009 IAVs	Human	H1N1	pdm	2009	Colombia	A/Bogota/WRAIR0435T/2009	ggtgatgccccattccttgatcggctccgccgagatca aaagtccttaaaaggaagaggcaacaccc	-	*
CY083132	Human H1N1pdm2009 IAVs	Human	H1N1		2009	Peru	A/Lima/WRAIR0672F/2009	ggtgatgccccattccttgatcggctccgccgagatca aaagtccttaaaaggaagaggcaacaccc	-	*
CY083108	Human H1N1pdm2009 IAVs	Human	H1N1	pdm	2009	Peru	A/Piura/WRAIR0603F/2009	ggtgatgccccattccttgatcggctccgccgagatca aaagtccttaaaaggaagaggcaacaccc	-	*
CY083515	Human H1N1pdm2009 IAVs	Human	H1N1	pdm	2009	Peru	A/Piura/WRAIR1694P/2009	ggtgatgccccattccttgatcggctccgccgagatca aaagtccttaaaaggaagaggcaacaccc	-	*
CY051763	Human H1N1pdm2009 IAVs	Human	H1N1	pdm	2009	USA	A/New_York/4823/2009	ggtgatgccccattccttgatcggctccgccgagatca aaagtccttaaaaggaagaggcaacaccc	-	*
CY051771	Human H1N1pdm2009 IAVs	Human	H1N1	pdm	2009	USA	A/New_York/4824/2009	ggtgatgccccattccttgatcggctccgccgagatca aaagtccttaaaaggaagaggcaacaccc	-	*
CY051779	Human H1N1pdm2009 IAVs	Human	H1N1	pdm	2009	USA	A/New_York/4841/2009	ggtgatgccccattccttgatcggctccgccgagatca aaagtccttaaaaggaagaggcaacaccc	-	*
CY052174	Human H1N1pdm2009 IAVs	Human	H1N1	pdm	2009	USA	A/New_York/4855/2009	ggtgatgccccattccttgatcggctccgccgagatca aaagtccttaaaaggaagaggcaacaccc	-	*
CY051795	Human H1N1pdm2009 IAVs	Human	H1N1	pdm	2009	USA	A/New_York/4856/2009	ggtgatgccccattccttgatcggctccgccgagatca aaagtccttaaaaggaagaggcaacaccc	-	*
CY052182	Human H1N1pdm2009 IAVs	Human	H1N1		2009	USA	A/New_York/4881/2009	ggtgatgccccattccttgatcggctccgccgagatca aaagtccttaaaaggaagaggcaacaccc	-	*
CY055427	Human H1N1pdm2009 IAVs	Human	H1N1	pdm	2009	USA	A/New_York/4887/2009	ggtgatgccccattccttgatcggctccgccgagatca aaagtccttaaaaggaagaggcaacaccc	-	*
CY056407	Human H1N1pdm2009 IAVs	Human	H1N1	pdm	2009	USA	A/New_York/4944/2009	ggtgatgccccattccttgatcggctccgccgagatca aaagtccttaaaaggaagaggcaacaccc	-	*
CY056455	Human H1N1pdm2009 IAVs	Human	H1N1	pdm	2009	USA	A/New_York/4986/2009	ggtgatgccccattccttgatcggctccgccgagatca aaagtccttaaaaggaagaggcaacaccc	-	*
CY056471	Human H1N1pdm2009 IAVs	Human	H1N1	pdm	2009	USA	A/New_York/4988/2009	ggtgatgccccattccttgatcggctccgccgagatca aaagtccttaaaaggaagaggcaacaccc	-	*
CY057234	Human H1N1pdm2009 IAVs	Human	H1N1	pdm	2009	USA	A/New_York/5045/2009	ggtgatgccccattccttgatcggctccgccgagatca aaagtccttaaaaggaagaggcaacaccc	-	*
CY055443	Human H1N1pdm2009 IAVs	Human	H1N1	pdm	2009	USA	A/New_York/4979/2009	ggtgatgccccattccttgatcggctccgccgagatca aaagtccttaaaaggaagaggcaacaccc	-	*

CY056415	Human H1N1pdm2009 IAVs	Human	H1N1	pdm	2009	USA	A/New_York/4945/2009	ggtgatgccccattccttgatcggctccgccgagatca aaagtccttaaaaggaagaggcaacaccc	-	*
CY083531	Human H1N1pdm2009 IAVs	Human	H1N1	pdm	2009	Peru	A/Lima/WRAIR1696P/2009	ggtgatgccccattccttgatcggctccgccgagatca aaagtccttaaaaggaagaggcaacaccc	-	*
CY083619	Human H1N1pdm2009 IAVs	Human	H1N1	pdm	2009	Peru	A/Lima/WRAIR9202F/2009	ggtgatgccccattccttgatcggctccgccgagatca aaagtccttaaaaggaagaggcaacaccc	-	*
CY083084	Human H1N1pdm2009 IAVs	Human	H1N1	pdm	2009	Colombia	A/Bogota/WRAIR0440T/2009	ggtgatgccccattccttgatcggctccgccgagatca aaagtccttaaaaggaagaggcaacaccc	-	*
CY083092	Human H1N1pdm2009 IAVs	Human	H1N1		2009	Colombia	A/Bogota/WRAIR0442T/2009	ggtgatgccccattccttgatcggctccgccgagatca aaagtccttaaaaggaagaggcaacaccc	-	*
CY055522	Human H1N1pdm2009 IAVs	Human	H1N1		2009	USA	A/California/VRDL78/2009	ggtgatgccccattccttgatcggctccgccgagatca aaagtccttaaaaggaagaggcaacaccc	-	*
CY083140	Human H1N1pdm2009 IAVs	Human	H1N1	pdm	2009	Peru	A/Lima/WRAIR0681F/2009	ggtgatgccccattccttgatcggctccgccgagatca aaagtccttaaaaggaagaggcaacaccc	-	*
CY083579	Human H1N1pdm2009 IAVs	Human	H1N1	pdm	2009	Peru	A/Lima/WRAIR8648F/2009	ggtgatgccccattccttgatcggctccgccgagatca aaagtccttaaaaggaagaggcaacaccc	-	*
CY089335	Human H1N1pdm2009 IAVs	Human	H1N1	pdm	2009	USA	A/Boston/683/2009	ggtgatgccccattccttgatcggctccgccgagatca aaagtccttaaaaggaagaggcaacaccc	-	*
CY089351	Human H1N1pdm2009 IAVs	Human	H1N1	pdm	2009	USA	A/Boston/686/2009	ggtgatgccccattccttgatcggctccgccgagatca aaagtccttaaaaggaagaggcaacaccc	-	*
CY066811	Human H1N1pdm2009 IAVs	Human	H1N1	pdm	2009	USA	A/San_Diego/INS217/2009	ggtgatgccccattccttgatcggctccgccgagatca aaagtccttaaaaggaagaggcaacaccc	-	*
CY068934	Human H1N1pdm2009 IAVs	Human	H1N1	pdm	2009	Guam	A/Guam/NHRC0005/2009	ggtgatgccccattccttgatcggctccgccgagatca aaagtccttaaaaggaagaggcaacaccc	-	*
CY068998	Human H1N1pdm2009 IAVs	Human	H1N1	pdm	2009	Guam	A/Guam/NHRC0014/2009	ggtgatgccccattccttgatcggctccgccgagatca aaagtccttaaaaggaagaggcaacaccc	-	*
CY068950	Human H1N1pdm2009 IAVs	Human	H1N1	pdm	2009	Japan	A/Japan/NHRC0004/2009	ggtgatgccccattccttgatcggctccgccgagatca aaagtccttaaaaggaagaggcaacaccc	-	*
CY069014	Human H1N1pdm2009 IAVs	Human	H1N1	pdm	2009	Guam	A/Guam/NHRC0016/2009	ggtgatgccccattccttgatcggctccgccgagatca aaagtccttaaaaggaagaggcaacaccc	-	*
CY075183	Human H1N1pdm2009 IAVs	Human	H1N1	pdm	2009	Chile	A/Chile/1598/2009	ggtgatgccccattccttgatcggctccgccgagatca aaagtccttaaaaggaagaggcaacaccc	-	*
CY075311	Human H1N1pdm2009 IAVs	Human	H1N1	pdm	2009	Chile	A/Chile/3242/2009	ggtgatgccccattccttgatcggctccgccgagatca aaagtccttaaaaggaagaggcaacaccc	-	*
CY176378	Human H1N1pdm2009 IAVs	Human	Unknow	pdm	2009	Denmark	A/Odense/INS3_631/2009	ggtgatgccccattccttgatcggctccgccgagatca aaagtccttaaaaggaagaggcaacaccc	-	*
CY083228	Human H1N1pdm2009 IAVs	Human	H1N1	pdm	2009	USA	A/California/WRAIR1507P/2009	ggtgatgccccattccttgatcggctccgccgagatca aaagtccttaaaaggaagaggcaacaccc	-	*
CY065940	Human H1N1pdm2009 IAVs	Human	H1N1	pdm	2009	Guam	A/Guam/NHRC0002/2009	ggtgatgccccattccttgatcggctccgccgagatca aaagtccttaaaaggaagaggcaacaccc	-	*
CY065948	Human H1N1pdm2009 IAVs	Human	H1N1	pdm	2009	Guam	A/Guam/NHRC0003/2009	ggtgatgccccattccttgatcggctccgccgagatca aaagtccttaaaaggaagaggcaacaccc	-	*
CY070979	Human H1N1pdm2009 IAVs	Human	H1N1	pdm	2009	Guam	A/Guam/NHRC0006/2009	ggtgatgccccattccttgatcggctccgccgagatca aaagtccttaaaaggaagaggcaacaccc	-	*
CY070987	Human H1N1pdm2009 IAVs	Human	H1N1	pdm	2009	Guam	A/Guam/NHRC0007/2009	ggtgatgccccattccttgatcggctccgccgagatca aaagtccttaaaaggaagaggcaacaccc	-	*
CY068958	Human H1N1pdm2009 IAVs	Human	H1N1	pdm	2009	Guam	A/Guam/NHRC0009/2009	ggtgatgccccattccttgatcggctccgccgagatca aaagtccttaaaaggaagaggcaacaccc	-	*
CY068966	Human H1N1pdm2009 IAVs	Human	H1N1	pdm	2009	Guam	A/Guam/NHRC0010/2009	ggtgatgccccattccttgatcggctccgccgagatca aaagtccttaaaaggaagaggcaacaccc	-	*

CY068982	Human H1N1pdm2009 IAVs	Human	H1N1		2009	Guam	A/Guam/NHRC0012/2009	ggtgatgccccattccttgatcggctccgccgagatca aaagtccttaaaaggaagaggcaacaccc	-	*
CY069022	Human H1N1pdm2009 IAVs	Human	H1N1		2009	Guam	A/Guam/NHRC0017/2009	ggtgatgccccattccttgatcggctccgccgagatca aaagtccttaaaaggaagaggcaacaccc	-	*
CY069054	Human H1N1pdm2009 IAVs	Human	H1N1	pdm	2009	Guam	A/Guam/NHRC0021/2009	ggtgatgccccattccttgatcggctccgccgagatca aaagtccttaaaaggaagaggcaacaccc	-	*
CY083236	Human H1N1pdm2009 IAVs	Human	H1N1	pdm	2009	Guam	A/Guam/WRAIR1510P/2009	ggtgatgccccattccttgatcggctccgccgagatca aaagtccttaaaaggaagaggcaacaccc	-	*
CY120072	Human H1N1pdm2009 IAVs	Human	H1N1		2009	Hong_Kong	A/Hong_Kong/H090_693_V20/2009	ggtgatgccccattccttgatcggctccgccgagatca aaagtccttaaaaggaagaggcaacaccc	-	*
HM189505	Human H1N1pdm2009 IAVs	Human	H1N1	pdm	2009	South_Korea	A/Korea/CJ19/2009	ggtgatgccccattccttgatcggctccgccgagatca aaagtccttaaaaggaagaggcaacaccc	-	*
CY123721	Human H1N1pdm2009 IAVs	Human	H1N1	pdm	2009	Singapore	A/Singapore/ON202/2009	ggtgatgccccattccttgatcggctccgccgagatca aaagtccttaaaaggaagaggcaacaccc	-	*
GU136021	Human H1N1pdm2009 IAVs	Human	H1N1	pdm	2009	Japan	A/Tokushima/2/2009	ggtgatgccccattccttgatcggctccgccgagatca aaagtccttaaaaggaagaggcaacaccc	-	*
CY122635	Human H1N1pdm2009 IAVs	Human	H1N1	pdm	2009	Singapore	A/Singapore/GP1114/2009	ggtgatgccccattccttgatcggctccgccgagatca aaagtccttaaaaggaagaggcaacaccc	-	*
CY122675	Human H1N1pdm2009 IAVs	Human	H1N1	pdm	2009	Singapore	A/Singapore/GP1132/2009	ggtgatgccccattccttgatcggctccgccgagatca aaagtccttaaaaggaagaggcaacaccc	-	*
CY122715	Human H1N1pdm2009 IAVs	Human	H1N1	pdm	2009	Singapore	A/Singapore/GP1147/2009	ggtgatgccccattccttgatcggctccgccgagatca aaagtccttaaaaggaagaggcaacaccc	-	*
CY122799	Human H1N1pdm2009 IAVs	Human	H1N1	pdm	2009	Singapore	A/Singapore/GP2215/2009	ggtgatgccccattccttgatcggctccgccgagatca aaagtccttaaaaggaagaggcaacaccc	-	*
CY123787	Human H1N1pdm2009 IAVs	Human	H1N1	pdm	2009	Singapore	A/Singapore/ON2145/2009	ggtgatgccccattccttgatcggctccgccgagatca aaagtccttaaaaggaagaggcaacaccc	-	*
CY123661	Human H1N1pdm2009 IAVs	Human	H1N1	pdm	2009	Singapore	A/Singapore/ON1934/2009	ggtgatgccccattccttgatcggctccgccgagatca aaagtccttaaaaggaagaggcaacaccc	-	*
CY124024	Human H1N1pdm2009 IAVs	Human	H1N1	pdm	2009	Singapore	A/Singapore/ON547/2009	ggtgatgccccattccttgatcggctccgccgagatca aaagtccttaaaaggaagaggcaacaccc	-	*
CY124039	Human H1N1pdm2009 IAVs	Human	H1N1	pdm	2009	Singapore	A/Singapore/ON580/2009	ggtgatgccccattccttgatcggctccgccgagatca aaagtccttaaaaggaagaggcaacaccc	-	*
CY124063	Human H1N1pdm2009 IAVs	Human	H1N1	pdm	2009	Singapore	A/Singapore/ON802/2009	ggtgatgccccattccttgatcggctccgccgagatca aaagtccttaaaaggaagaggcaacaccc	-	*
CY124142	Human H1N1pdm2009 IAVs	Human	H1N1		2009	Singapore	A/Singapore/ON819/2009	ggtgatgccccattccttgatcggctccgccgagatca aaagtccttaaaaggaagaggcaacaccc	-	*
CY095902	Human H1N1pdm2009 IAVs	Human	H1N1		2009	China	A/Zhejiang/79/2009	ggtgatgccccattccttgatcggctccgccgagatca aaagtccttaaaaggaagaggcaacaccc	-	*
CY083725	Human H1N1pdm2009 IAVs	Human	H1N1	pdm	2009	USA	A/District_of_Columbia/INS115/2009	ggtgatgccccattccttgatcggctccgccgagatca aaagtccttaaaaggaagaggcaacaccc	-	*
CY119382	Human H1N1pdm2009 IAVs	Human	H1N1		2009	Malaysia	A/Malaysia/2076039/2009	ggtgatgccccattccttgatcggctccgccgagatca aaagtccttaaaaggaagaggcaacaccc	-	*
GU136010	Human H1N1pdm2009 IAVs	Human	H1N1	pdm	2009	Myanmar	A/Myanmar/60/2009	ggtgatgccccattccttgatcggctccgccgagatca aaagtccttaaaaggaagaggcaacaccc	-	*
CY122567	Human H1N1pdm2009 IAVs	Human	H1N1	pdm	2009	Singapore	A/Singapore/GP1022/2009	ggtgatgccccattccttgatcggctccgccgagatca aaagtccttaaaaggaagaggcaacaccc	-	*
CY122743	Human H1N1pdm2009 IAVs	Human	H1N1	pdm	2009	Singapore	A/Singapore/GP1164/2009	ggtgatgccccattccttgatcggctccgccgagatca aaagtccttaaaaggaagaggcaacaccc	-	*
CY122807	Human H1N1pdm2009 IAVs	Human	H1N1	pdm	2009	Singapore	A/Singapore/GP2221/2009	ggtgatgccccattccttgatcggctccgccgagatca aaagtccttaaaaggaagaggcaacaccc	-	*

CY122839	Human H1N1pdm2009 IAVs	Human	H1N1	pdm	2009	Singapore	A/Singapore/GP2242/2009	ggtgatgccccattccttgatcggctccgccgagatca<br>aaagtccttaaaaggaagaggcaacaccc	-	*
CY122883	Human H1N1pdm2009 IAVs	Human	H1N1	pdm	2009	Singapore	A/Singapore/GP2315/2009	ggtgatgccccattccttgatcggctccgccgagatca<br>aaagtccttaaaaggaagaggcaacaccc	-	*
CY122963	Human H1N1pdm2009 IAVs	Human	H1N1	pdm	2009	Singapore	A/Singapore/GP2714/2009	ggtgatgccccattccttgatcggctccgccgagatca<br>aaagtccttaaaaggaagaggcaacaccc	-	*
CY123079	Human H1N1pdm2009 IAVs	Human	H1N1	pdm	2009	Singapore	A/Singapore/GP3178/2009	ggtgatgccccattccttgatcggctccgccgagatca<br>aaagtccttaaaaggaagaggcaacaccc	-	*
CY123103	Human H1N1pdm2009 IAVs	Human	H1N1	pdm	2009	Singapore	A/Singapore/GP3187/2009	ggtgatgccccattccttgatcggctccgccgagatca<br>aaagtccttaaaaggaagaggcaacaccc	-	*
CY123158	Human H1N1pdm2009 IAVs	Human	H1N1	pdm	2009	Singapore	A/Singapore/GP3545/2009	ggtgatgccccattccttgatcggctccgccgagatca<br>aaagtccttaaaaggaagaggcaacaccc	-	*
CY123350	Human H1N1pdm2009 IAVs	Human	H1N1	pdm	2009	Singapore	A/Singapore/GP687/2009	ggtgatgccccattccttgatcggctccgccgagatca<br>aaagtccttaaaaggaagaggcaacaccc	-	*
CY123366	Human H1N1pdm2009 IAVs	Human	H1N1	pdm	2009	Singapore	A/Singapore/GP875/2009	ggtgatgccccattccttgatcggctccgccgagatca<br>aaagtccttaaaaggaagaggcaacaccc	-	*
CY123552	Human H1N1pdm2009 IAVs	Human	H1N1		2009	Singapore	A/Singapore/ON1813/2009	ggtgatgccccattccttgatcggctccgccgagatca<br>aaagtccttaaaaggaagaggcaacaccc	-	*
CY123620	Human H1N1pdm2009 IAVs	Human	H1N1	pdm	2009	Singapore	A/Singapore/ON1911/2009	ggtgatgccccattccttgatcggctccgccgagatca<br>aaagtccttaaaaggaagaggcaacaccc	-	*
CY123687	Human H1N1pdm2009 IAVs	Human	H1N1	pdm	2009	Singapore	A/Singapore/ON1964/2009	ggtgatgccccattccttgatcggctccgccgagatca<br>aaagtccttaaaaggaagaggcaacaccc	-	*
CY124032	Human H1N1pdm2009 IAVs	Human	H1N1		2009	Singapore	A/Singapore/ON575/2009	ggtgatgccccattccttgatcggctccgccgagatca<br>aaagtccttaaaaggaagaggcaacaccc	-	*
CY124091	Human H1N1pdm2009 IAVs	Human	H1N1	pdm	2009	Singapore	A/Singapore/ON806/2009	ggtgatgccccattccttgatcggctccgccgagatca<br>aaagtccttaaaaggaagaggcaacaccc	-	*
CY124135	Human H1N1pdm2009 IAVs	Human	H1N1	pdm	2009	Singapore	A/Singapore/ON816/2009	ggtgatgccccattccttgatcggctccgccgagatca<br>aaagtccttaaaaggaagaggcaacaccc	-	*
CY122991	Human H1N1pdm2009 IAVs	Human	H1N1	pdm	2009	Singapore	A/Singapore/GP3012/2009	ggtgatgccccattccttgatcggctccgccgagatca<br>aaagtccttaaaaggaagaggcaacaccc	-	*
CY122823	Human H1N1pdm2009 IAVs	Human	H1N1	pdm	2009	Singapore	A/Singapore/GP2224/2009	ggtgatgccccattccttgatcggctccgccgagatca<br>aaagtccttaaaaggaagaggcaacaccc	-	*
CY122855	Human H1N1pdm2009 IAVs	Human	H1N1	pdm	2009	Singapore	A/Singapore/GP2286/2009	ggtgatgccccattccttgatcggctccgccgagatca<br>aaagtccttaaaaggaagaggcaacaccc	-	*
CY122955	Human H1N1pdm2009 IAVs	Human	H1N1	pdm	2009	Singapore	A/Singapore/GP2709/2009	ggtgatgccccattccttgatcggctccgccgagatca<br>aaagtccttaaaaggaagaggcaacaccc	-	*
CY123821	Human H1N1pdm2009 IAVs	Human	H1N1	pdm	2009	Singapore	A/Singapore/ON2250/2009	ggtgatgccccattccttgatcggctccgccgagatca<br>aaagtccttaaaaggaagaggcaacaccc	-	*
CY122923	Human H1N1pdm2009 IAVs	Human	H1N1	pdm	2009	Singapore	A/Singapore/GP2672/2009	ggtgatgccccattccttgatcggctccgccgagatca<br>aaagtccttaaaaggaagaggcaacaccc	-	*
CY122979	Human H1N1pdm2009 IAVs	Human	H1N1	pdm	2009	Singapore	A/Singapore/GP2987/2009	ggtgatgccccattccttgatcggctccgccgagatca<br>aaagtccttaaaaggaagaggcaacaccc	-	*
CY041494	Human H1N1pdm2009 IAVs	Human	H1N1	pdm	2009	USA	A/New_York/3184/2009	ggtgatgccccattccttgatcggctccgccgagatca<br>aaagtccttaaaaggaagaggcaacaccc	-	*
CY086981	Human H1N1pdm2009 IAVs	Human	H1N1	pdm	2009	USA	A/New_York/3217/2009	ggtgatgccccattccttgatcggctccgccgagatca<br>aaagtccttaaaaggaagaggcaacaccc	-	*
CY052891	Human H1N1pdm2009 IAVs	Human	H1N1	pdm	2009	USA	A/Texas/45062346/2009	ggtgatgccccattccttgatcggctccgccgagatca<br>aaagtccttaaaaggaagaggcaacaccc	-	*
CY123055	Human H1N1pdm2009 IAVs	Human	H1N1	pdm	2009	Singapore	A/Singapore/GP3093/2009	ggtgatgccccattccttgatcggctccgccgagatca<br>aaagtccttaaaaggaagaggcaacaccc	-	*

CY072634	Human H1N1pdm2009 IAVs	Human	H1N1	pdm	2009	Nicaragua	A/Managua/1836.02/2009	ggtgatgccccattccttgatcggctccgccgagatca aaagtccttaaaaggaagaggcaacaccc	-	*
CY073549	Human H1N1pdm2009 IAVs	Human	H1N1	pdm	2009	Nicaragua	A/Managua/3083.01/2009	ggtgatgccccattccttgatcggctccgccgagatca aaagtccttaaaaggaagaggcaacaccc	-	*
CY052286	Human H1N1pdm2009 IAVs	Human	H1N1	pdm	2009	USA	A/Texas/43292238/2009	ggtgatgccccattccttgatcggctccgccgagatca aaagtccttaaaaggaagaggcaacaccc	-	*
GQ225353	Human H1N1pdm2009 IAVs	Human	H1N1	pdm	2009	China	A/Zhejiang/1/2009	ggtgatgccccattccttgatcggctccgccgagatca aaagtccttaaaaggaagaggcaacaccc	-	*
CY061255	Human H1N1pdm2009 IAVs	Human	H1N1	pdm	2009	USA	A/San_Diego/INS104/2009	ggtgatgccccattccttgatcggctccgccgagatca aaagtccttaaaaggaagaggcaacaccc	-	*
CY065932	Human H1N1pdm2009 IAVs	Human	H1N1		2009	Guam	A/Guam/NHRC0001/2009	ggtgatgccccattccttgatcggctccgccgagatca aaagtccttaaaaggaagaggcaacaccc	-	*
CY068974	Human H1N1pdm2009 IAVs	Human	H1N1	pdm	2009	Guam	A/Guam/NHRC0011/2009	ggtgatgccccattccttgatcggctccgccgagatca aaagtccttaaaaggaagaggcaacaccc	-	*
CY071003	Human H1N1pdm2009 IAVs	Human	H1N1	pdm	2009	Guam	A/Guam/NHRC0030/2009	ggtgatgccccattccttgatcggctccgccgagatca aaagtccttaaaaggaagaggcaacaccc	-	*
CY069094	Human H1N1pdm2009 IAVs	Human	H1N1	pdm	2009	Guam	A/Guam/NHRC0026/2009	ggtgatgccccattccttgatcggctccgccgagatca aaagtccttaaaaggaagaggcaacaccc	-	*
CY051787	Human H1N1pdm2009 IAVs	Human	H1N1	pdm	2009	USA	A/New_York/4844/2009	ggtgatgccccattccttgatcggctccgccgagatca aaagtccttaaaaggaagaggcaacaccc	-	*
CY055577	Human H1N1pdm2009 IAVs	Human	H1N1	pdm	2009	Australia	A/Australia/13/2009	ggtgatgccccattccttgatcggctccgccgagatca aaagtccttaaaaggaagaggcaacaccc	-	*
CY055824	Human H1N1pdm2009 IAVs	Human	H1N1	pdm	2009	Australia	A/Australia/58/2009	ggtgatgccccattccttgatcggctccgccgagatca aaagtccttaaaaggaagaggcaacaccc	-	*
CY055856	Human H1N1pdm2009 IAVs	Human	H1N1	pdm	2009	Australia	A/Australia/63/2009	ggtgatgccccattccttgatcggctccgccgagatca aaagtccttaaaaggaagaggcaacaccc	-	*
CY066867	Human H1N1pdm2009 IAVs	Human	H1N1	pdm	2009	USA	A/District_of_Columbia/INS227/2009	ggtgatgccccattccttgatcggctccgccgagatca aaagtccttaaaaggaagaggcaacaccc	-	*
CY120177	Human H1N1pdm2009 IAVs	Human	H1N1	pdm	2009	Hong_Kong	A/Hong_Kong/H090_684_V23/2009	ggtgatgccccattccttgatcggctccgccgagatca aaagtccttaaaaggaagaggcaacaccc	-	*
CY111711	Human H1N1pdm2009 IAVs	Human	H1N1	pdm	2009	Hong_Kong	A/Hong_Kong/H090_684_V23/2009	ggtgatgccccattccttgatcggctccgccgagatca aaagtccttaaaaggaagaggcaacaccc	-	*
CY041518	Human H1N1pdm2009 IAVs	Human	H1N1	pdm	2009	USA	A/New_York/3203/2009	ggtgatgccccattccttgatcggctccgccgagatca aaagtccttaaaaggaagaggcaacaccc	-	*
CY080428	Human H1N1pdm2009 IAVs	Swine	H1N1		2009	Australia	A/swine/VIC/09_02767_01/2009	ggtgatgccccattccttgatcggctccgccgagatca aaagtccttaaaaggaagaggcaacaccc	-	*
CY080436	Human H1N1pdm2009 IAVs	Swine	H1N1		2009	Australia	A/swine/VIC/09_02797_62/2009	ggtgatgccccattccttgatcggctccgccgagatca aaagtccttaaaaggaagaggcaacaccc	-	*
CY057570	Human H1N1pdm2009 IAVs	Human	H1N1	pdm	2009	USA	A/Wisconsin/629_D00834/2009	ggtgatgccccattccttgatcggctccgccgagatca aaagtccttaaaaggaagaggcaacaccc	-	*
KJ130200	Human H1N1pdm2009 IAVs	Human	H1N1	pdm	2009	New_Zealand	A/New_Zealand/2047/2009	ggtgatgccccattccttgatcggctccgccgagatca aaagtccttaaaaggaagaggcaacaccc	-	*
CY119390	Human H1N1pdm2009 IAVs	Human	H1N1	pdm	2009	Malaysia	A/Malaysia/2077462/2009	ggtgatgccccattccttgatcggctccgccgagatca aaagtccttaaaaggaagaggcaacaccc	-	*
CY123007	Human H1N1pdm2009 IAVs	Human	H1N1	pdm	2009	Singapore	A/Singapore/GP3026/2009	ggtgatgccccattccttgatcggctccgccgagatca aaagtccttaaaaggaagaggcaacaccc	-	*
GU136004	Human H1N1pdm2009 IAVs	Human	H1N1	pdm	2009	Japan	A/Hiroshima/230/2009	ggtgatgccccattccttgatcggctccgccgagatca aaagtccttaaaaggaagaggcaacaccc	-	*
CY123653	Human H1N1pdm2009 IAVs	Human	H1N1	pdm	2009	Singapore	A/Singapore/ON193/2009	ggtgatgccccattccttgatcggctccgccgagatca aaagtccttaaaaggaagaggcaacaccc	-	*

CY081490	Human H1N1pdm2009 IAVs	Human	H1N1	pdm	2009	Cambodia	A/Cambodia/NHRCC00001/2009	ggtgatgccccattccttgatcggctccgccgagatcaaaagtccttaaaaggaagaggcaacaccc	-	*
CY081498	Human H1N1pdm2009 IAVs	Human	H1N1	pdm	2009	Cambodia	A/Cambodia/NHRCC00002/2009	ggtgatgccccattccttgatcggctccgccgagatcaaaagtccttaaaaggaagaggcaacaccc	-	*
CY081506	Human H1N1pdm2009 IAVs	Human	H1N1	pdm	2009	Cambodia	A/Cambodia/NHRCC00003/2009	ggtgatgccccattccttgatcggctccgccgagatcaaaagtccttaaaaggaagaggcaacaccc	-	*
CY081514	Human H1N1pdm2009 IAVs	Human	H1N1	pdm	2009	Cambodia	A/Cambodia/NHRCC00004/2009	ggtgatgccccattccttgatcggctccgccgagatcaaaagtccttaaaaggaagaggcaacaccc	-	*
CY081522	Human H1N1pdm2009 IAVs	Human	H1N1		2009	Cambodia	A/Cambodia/NHRCC00005/2009	ggtgatgccccattccttgatcggctccgccgagatcaaaagtccttaaaaggaagaggcaacaccc	-	*
CY081538	Human H1N1pdm2009 IAVs	Human	H1N1	pdm	2009	Cambodia	A/Cambodia/NHRCC00007/2009	ggtgatgccccattccttgatcggctccgccgagatcaaaagtccttaaaaggaagaggcaacaccc	-	*
CY081546	Human H1N1pdm2009 IAVs	Human	H1N1	pdm	2009	Cambodia	A/Cambodia/NHRCC00008/2009	ggtgatgccccattccttgatcggctccgccgagatcaaaagtccttaaaaggaagaggcaacaccc	-	*
CY120217	Human H1N1pdm2009 IAVs	Human	H1N1	pdm	2009	Hong_Kong	A/Hong_Kong/H090_708_V10/2009	ggtgatgccccattccttgatcggctccgccgagatcaaaagtccttaaaaggaagaggcaacaccc	-	*
CY111767	Human H1N1pdm2009 IAVs	Human	H1N1	pdm	2009	Hong_Kong	A/Hong_Kong/H090_708_V10/2009	ggtgatgccccattccttgatcggctccgccgagatcaaaagtccttaaaaggaagaggcaacaccc	-	*
CY111839	Human H1N1pdm2009 IAVs	Human	H1N1	pdm	2009	Hong_Kong	A/Hong_Kong/H090_738_V10/2009	ggtgatgccccattccttgatcggctccgccgagatcaaaagtccttaaaaggaagaggcaacaccc	-	*
CY120273	Human H1N1pdm2009 IAVs	Human	H1N1	pdm	2009	Hong_Kong	A/Hong_Kong/H090_738_V10/2009	ggtgatgccccattccttgatcggctccgccgagatcaaaagtccttaaaaggaagaggcaacaccc	-	*
CY120281	Human H1N1pdm2009 IAVs	Human	H1N1	pdm	2009	Hong_Kong	A/Hong_Kong/H090_742_V10/2009	ggtgatgccccattccttgatcggctccgccgagatcaaaagtccttaaaaggaagaggcaacaccc	-	*
CY111847	Human H1N1pdm2009 IAVs	Human	H1N1	pdm	2009	Hong_Kong	A/Hong_Kong/H090_742_V10/2009	ggtgatgccccattccttgatcggctccgccgagatcaaaagtccttaaaaggaagaggcaacaccc	-	*
CY120289	Human H1N1pdm2009 IAVs	Human	H1N1	pdm	2009	Hong_Kong	A/Hong_Kong/H090_742_V33/2009	ggtgatgccccattccttgatcggctccgccgagatcaaaagtccttaaaaggaagaggcaacaccc	-	*
CY111855	Human H1N1pdm2009 IAVs	Human	H1N1	pdm	2009	Hong_Kong	A/Hong_Kong/H090_742_V33/2009	ggtgatgccccattccttgatcggctccgccgagatcaaaagtccttaaaaggaagaggcaacaccc	-	*
CY120088	Human H1N1pdm2009 IAVs	Human	H1N1	pdm	2009	Hong_Kong	A/Hong_Kong/H090_745_V10/2009	ggtgatgccccattccttgatcggctccgccgagatcaaaagtccttaaaaggaagaggcaacaccc	-	*
CY111863	Human H1N1pdm2009 IAVs	Human	H1N1	pdm	2009	Hong_Kong	A/Hong_Kong/H090_745_V10/2009	ggtgatgccccattccttgatcggctccgccgagatcaaaagtccttaaaaggaagaggcaacaccc	-	*
CY111871	Human H1N1pdm2009 IAVs	Human	H1N1	pdm	2009	Hong_Kong	A/Hong_Kong/H090_745_V24/2009	ggtgatgccccattccttgatcggctccgccgagatcaaaagtccttaaaaggaagaggcaacaccc	-	*
CY120297	Human H1N1pdm2009 IAVs	Human	H1N1	pdm	2009	Hong_Kong	A/Hong_Kong/H090_746_V10/2009	ggtgatgccccattccttgatcggctccgccgagatcaaaagtccttaaaaggaagaggcaacaccc	-	*
CY111879	Human H1N1pdm2009 IAVs	Human	H1N1	pdm	2009	Hong_Kong	A/Hong_Kong/H090_746_V10/2009	ggtgatgccccattccttgatcggctccgccgagatcaaaagtccttaaaaggaagaggcaacaccc	-	*
CY111887	Human H1N1pdm2009 IAVs	Human	H1N1	pdm	2009	Hong_Kong	A/Hong_Kong/H090_746_V20/2009	ggtgatgccccattccttgatcggctccgccgagatcaaaagtccttaaaaggaagaggcaacaccc	-	*
CY111975	Human H1N1pdm2009 IAVs	Human	H1N1	pdm	2009	Hong_Kong	A/Hong_Kong/H090_762_V10/2009	ggtgatgccccattccttgatcggctccgccgagatcaaaagtccttaaaaggaagaggcaacaccc	-	*
CY120385	Human H1N1pdm2009 IAVs	Human	H1N1	pdm	2009	Hong_Kong	A/Hong_Kong/H090_762_V10/2009	ggtgatgccccattccttgatcggctccgccgagatcaaaagtccttaaaaggaagaggcaacaccc	-	*
CY112071	Human H1N1pdm2009 IAVs	Human	H1N1	pdm	2009	Hong_Kong	A/Hong_Kong/H090_785_V10/2009	ggtgatgccccattccttgatcggctccgccgagatcaaaagtccttaaaaggaagaggcaacaccc	-	*
CY120465	Human H1N1pdm2009 IAVs	Human	H1N1	pdm	2009	Hong_Kong	A/Hong_Kong/H090_785_V10/2009	ggtgatgccccattccttgatcggctccgccgagatcaaaagtccttaaaaggaagaggcaacaccc	-	*

CY055275	Human H1N1pdm2009 IAVs	Human	H1N1	pdm	2009	Malaysia	A/Malaysia/10226/2009	ggtgatgccccattccttgatcggctccgccgagatca aaagtccttaaaaggaagaggcaacaccc	-	*
CY112015	Human H1N1pdm2009 IAVs	Human	H1N1	pdm	2009	Hong_Kong	A/Hong_Kong/H090_772_V10/2009	ggtgatgccccattccttgatcggctccgccgagatca aaagtccttaaaaggaagaggcaacaccc	-	*
CY120417	Human H1N1pdm2009 IAVs	Human	H1N1	pdm	2009	Hong_Kong	A/Hong_Kong/H090_772_V10/2009	ggtgatgccccattccttgatcggctccgccgagatca aaagtccttaaaaggaagaggcaacaccc	-	*
CY119366	Human H1N1pdm2009 IAVs	Human	H1N1	pdm	2009	Malaysia	A/Malaysia/2068631/2009	ggtgatgccccattccttgatcggctccgccgagatca aaagtccttaaaaggaagaggcaacaccc	-	*
CY119526	Human H1N1pdm2009 IAVs	Human	H1N1	pdm	2009	Malaysia	A/Malaysia/2076756/2009	ggtgatgccccattccttgatcggctccgccgagatca aaagtccttaaaaggaagaggcaacaccc	-	*
CY119342	Human H1N1pdm2009 IAVs	Human	H1N1		2009	Malaysia	A/Malaysia/2142299/2009	ggtgatgccccattccttgatcggctccgccgagatca aaagtccttaaaaggaagaggcaacaccc	-	*
CY119350	Human H1N1pdm2009 IAVs	Human	H1N1	pdm	2009	Malaysia	A/Malaysia/2143696/2009	ggtgatgccccattccttgatcggctccgccgagatca aaagtccttaaaaggaagaggcaacaccc	-	*
CY123071	Human H1N1pdm2009 IAVs	Human	H1N1	pdm	2009	Singapore	A/Singapore/GP3175/2009	ggtgatgccccattccttgatcggctccgccgagatca aaagtccttaaaaggaagaggcaacaccc	-	*
CY123212	Human H1N1pdm2009 IAVs	Human	H1N1		2009	Singapore	A/Singapore/GP4000/2009	ggtgatgccccattccttgatcggctccgccgagatca aaagtccttaaaaggaagaggcaacaccc	-	*
CY119542	Human H1N1pdm2009 IAVs	Human	H1N1		2009	Malaysia	A/Malaysia/2126082/2009	ggtgatgccccattccttgatcggctccgccgagatca aaagtccttaaaaggaagaggcaacaccc	-	*
CY055960	Human H1N1pdm2009 IAVs	Human	H1N1	pdm	2009	USA	A/San_Diego/INS02/2009	ggtgatgccccattccttgatcggctccgccgagatca aaagtccttaaaaggaagaggcaacaccc	-	*
CY055992	Human H1N1pdm2009 IAVs	Human	H1N1		2009	USA	A/San_Diego/INS06/2009	ggtgatgccccattccttgatcggctccgccgagatca aaagtccttaaaaggaagaggcaacaccc	-	*
CY120080	Human H1N1pdm2009 IAVs	Human	H1N1	pdm	2009	Hong_Kong	A/Hong_Kong/H090_698_V10/2009	ggtgatgccccattccttgatcggctccgccgagatca aaagtccttaaaaggaagaggcaacaccc	-	*
CY111743	Human H1N1pdm2009 IAVs	Human	H1N1	pdm	2009	Hong_Kong	A/Hong_Kong/H090_698_V20/2009	ggtgatgccccattccttgatcggctccgccgagatca aaagtccttaaaaggaagaggcaacaccc	-	*
CY064512	Human H1N1pdm2009 IAVs	Human	H1N1		2009	USA	A/Boston/116/2009	ggtgatgccccattccttgatcggctccgccgagatca aaagtccttaaaaggaagaggcaacaccc	-	*
CY120743	Human H1N1pdm2009 IAVs	Human	H1N1	pdm	2009	Brazil	A/Brazil/AVS07/2009	ggtgatgccccattccttgatcggctccgccgagatca aaagtccttaaaaggaagaggcaacaccc	-	*
CY054767	Human H1N1pdm2009 IAVs	Human	H1N1	pdm	2009	USA	A/California/VRDL20/2009	ggtgatgccccattccttgatcggctccgccgagatca aaagtccttaaaaggaagaggcaacaccc	-	*
CY047370	Human H1N1pdm2009 IAVs	Human	H1N1	pdm	2009	USA	A/New_York/3795/2009	ggtgatgccccattccttgatcggctccgccgagatca aaagtccttaaaaggaagaggcaacaccc	-	*
CY045017	Human H1N1pdm2009 IAVs	Human	H1N1	pdm	2009	USA	A/New_York/3796/2009	ggtgatgccccattccttgatcggctccgccgagatca aaagtccttaaaaggaagaggcaacaccc	-	*
CY072962	Human H1N1pdm2009 IAVs	Human	H1N1		2009	Nicaragua	A/Managua/585.01/2009	ggtgatgccccattccttgatcggctccgccgagatca aaagtccttaaaaggaagaggcaacaccc	-	*
CY060594	Human H1N1pdm2009 IAVs	Human	H1N1	pdm	2009	Canada	A/Ontario/305139/2009	ggtgatgccccattccttgatcggctccgccgagatca aaagtccttaaaaggaagaggcaacaccc	-	*
CY060602	Human H1N1pdm2009 IAVs	Human	H1N1		2009	Canada	A/Ontario/305598/2009	ggtgatgccccattccttgatcggctccgccgagatca aaagtccttaaaaggaagaggcaacaccc	-	*
CY060610	Human H1N1pdm2009 IAVs	Human	H1N1	pdm	2009	Canada	A/Ontario/308054/2009	ggtgatgccccattccttgatcggctccgccgagatca aaagtccttaaaaggaagaggcaacaccc	-	*
CY060626	Human H1N1pdm2009 IAVs	Human	H1N1	pdm	2009	Canada	A/Ontario/313762/2009	ggtgatgccccattccttgatcggctccgccgagatca aaagtccttaaaaggaagaggcaacaccc	-	*
CY060634	Human H1N1pdm2009 IAVs	Human	H1N1	pdm	2009	Canada	A/Ontario/314095/2009	ggtgatgccccattccttgatcggctccgccgagatca aaagtccttaaaaggaagaggcaacaccc	-	*

CY060666	Human H1N1pdm2009 IAVs	Human	H1N1	pdm	2009	Canada	A/Ontario/315015/2009	ggtgatgccccattccttgatcggctccgccgagatca aaagtccttaaaaggaagaggcaacaccc	-	*
CY060674	Human H1N1pdm2009 IAVs	Human	H1N1	pdm	2009	Canada	A/Ontario/315047/2009	ggtgatgccccattccttgatcggctccgccgagatca aaagtccttaaaaggaagaggcaacaccc	-	*
CY060698	Human H1N1pdm2009 IAVs	Human	H1N1	pdm	2009	Canada	A/Ontario/315187/2009	ggtgatgccccattccttgatcggctccgccgagatca aaagtccttaaaaggaagaggcaacaccc	-	*
CY060706	Human H1N1pdm2009 IAVs	Human	H1N1	pdm	2009	Canada	A/Ontario/315613/2009	ggtgatgccccattccttgatcggctccgccgagatca aaagtccttaaaaggaagaggcaacaccc	-	*
CY060714	Human H1N1pdm2009 IAVs	Human	H1N1	pdm	2009	Canada	A/Ontario/315637/2009	ggtgatgccccattccttgatcggctccgccgagatca aaagtccttaaaaggaagaggcaacaccc	-	*
CY111639	Human H1N1pdm2009 IAVs	Human	H1N1	pdm	2009	Hong_Kong	A/Hong_Kong/H090_667_V10/2009	ggtgatgccccattccttgatcggctccgccgagatca aaagtccttaaaaggaagaggcaacaccc	-	*
CY120121	Human H1N1pdm2009 IAVs	Human	H1N1	pdm	2009	Hong_Kong	A/Hong_Kong/H090_667_V10/2009	ggtgatgccccattccttgatcggctccgccgagatca aaagtccttaaaaggaagaggcaacaccc	-	*
CY120065	Human H1N1pdm2009 IAVs	Human	H1N1	pdm	2009	Hong_Kong	A/Hong_Kong/H090_667_V23/2009	ggtgatgccccattccttgatcggctccgccgagatca aaagtccttaaaaggaagaggcaacaccc	-	*
CY111647	Human H1N1pdm2009 IAVs	Human	H1N1	pdm	2009	Hong_Kong	A/Hong_Kong/H090_667_V23/2009	ggtgatgccccattccttgatcggctccgccgagatca aaagtccttaaaaggaagaggcaacaccc	-	*
HM189599	Human H1N1pdm2009 IAVs	Swine	H1N1		2009	South_Korea	A/swine/Korea/SCJ09/2009	ggtgatgccccattccttgatcggctccgccgagatca aaagtccttaaaaggaagaggcaacaccc	-	*
HM189600	Human H1N1pdm2009 IAVs	Swine	H1N1		2009	South_Korea	A/swine/Korea/SCJ10/2009	ggtgatgccccattccttgatcggctccgccgagatca aaagtccttaaaaggaagaggcaacaccc	-	*
CY063642	Human H1N1pdm2009 IAVs	Human	H1N1		2009	Greece	A/Athens/INS153/2009	ggtgatgccccattccttgatcggctccgccgagatca aaagtccttaaaaggaagaggcaacaccc	-	*
CY063658	Human H1N1pdm2009 IAVs	Human	H1N1	pdm	2009	Greece	A/Athens/INS157/2009	ggtgatgccccattccttgatcggctccgccgagatca aaagtccttaaaaggaagaggcaacaccc	-	*
CY067067	Human H1N1pdm2009 IAVs	Human	H1N1	pdm	2009	Greece	A/Athens/INS257/2009	ggtgatgccccattccttgatcggctccgccgagatca aaagtccttaaaaggaagaggcaacaccc	-	*
CY072482	Human H1N1pdm2009 IAVs	Human	H1N1		2009	Greece	A/Athens/INS344/2009	ggtgatgccccattccttgatcggctccgccgagatca aaagtccttaaaaggaagaggcaacaccc	-	*
CY069158	Human H1N1pdm2009 IAVs	Human	H1N1	pdm	2009	Greece	A/Athens/INS352/2009	ggtgatgccccattccttgatcggctccgccgagatca aaagtccttaaaaggaagaggcaacaccc	-	*
CY069206	Human H1N1pdm2009 IAVs	Human	H1N1	pdm	2009	Greece	A/Athens/INS359/2009	ggtgatgccccattccttgatcggctccgccgagatca aaagtccttaaaaggaagaggcaacaccc	-	*
CY062839	Human H1N1pdm2009 IAVs	Human	H1N1	pdm	2009	Greece	A/Athens/INS155/2009	ggtgatgccccattccttgatcggctccgccgagatca aaagtccttaaaaggaagaggcaacaccc	-	*
CY072354	Human H1N1pdm2009 IAVs	Human	H1N1	pdm	2009	Greece	A/Athens/INS322/2009	ggtgatgccccattccttgatcggctccgccgagatca aaagtccttaaaaggaagaggcaacaccc	-	*
CY072450	Human H1N1pdm2009 IAVs	Human	H1N1	pdm	2009	Greece	A/Athens/INS339/2009	ggtgatgccccattccttgatcggctccgccgagatca aaagtccttaaaaggaagaggcaacaccc	-	*
CY062855	Human H1N1pdm2009 IAVs	Human	H1N1	pdm	2009	Greece	A/Athens/INS158/2009	ggtgatgccccattccttgatcggctccgccgagatca aaagtccttaaaaggaagaggcaacaccc	-	*
CY083772	Human H1N1pdm2009 IAVs	Human	H1N1		2009	Greece	A/Athens/INS256/2009	ggtgatgccccattccttgatcggctccgccgagatca aaagtccttaaaaggaagaggcaacaccc	-	*
CY092916	Human H1N1pdm2009 IAVs	Human	H1N1	pdm	2009	USA	A/Maryland/NHRC0001/2009	ggtgatgccccattccttgatcggctccgccgagatca aaagtccttaaaaggaagaggcaacaccc	-	*
CY092924	Human H1N1pdm2009 IAVs	Human	H1N1	pdm	2009	USA	A/Maryland/NHRC0002/2009	ggtgatgccccattccttgatcggctccgccgagatca aaagtccttaaaaggaagaggcaacaccc	-	*
CY092932	Human H1N1pdm2009 IAVs	Human	H1N1		2009	USA	A/Maryland/NHRC0003/2009	ggtgatgccccattccttgatcggctccgccgagatca aaagtccttaaaaggaagaggcaacaccc	-	*

CY092940	Human H1N1pdm2009 IAVs	Human	H1N1	pdm	2009	USA	A/Maryland/NHRC0004/2009	ggtgatgccccattccttgatcggctccgccgagatca aaagtccttaaaaggaagaggcaacaccc	-	*
CY052023	Human H1N1pdm2009 IAVs	Human	H1N1	pdm	2009	Norway	A/Norway/3206_3/2009	ggtgatgccccattccttgatcggctccgccgagatca aaagtccttaaaaggaagaggcaacaccc	-	*
CY123242	Human H1N1pdm2009 IAVs	Human	H1N1		2009	Singapore	A/Singapore/GP4218/2009	ggtgatgccccattccttgatcggctccgccgagatca aaagtccttaaaaggaagaggcaacaccc	-	*
CY083898	Human H1N1pdm2009 IAVs	Human	H1N1	pdm	2009	USA	A/San_Diego/INS73/2009	ggtgatgccccattccttgatcggctccgccgagatca aaagtccttaaaaggaagaggcaacaccc	-	*
CY055498	Human H1N1pdm2009 IAVs	Human	H1N1	pdm	2009	USA	A/California/VRDL74/2009	ggtgatgccccattccttgatcggctccgccgagatca aaagtccttaaaaggaagaggcaacaccc	-	*
CY055506	Human H1N1pdm2009 IAVs	Human	H1N1	pdm	2009	USA	A/California/VRDL75/2009	ggtgatgccccattccttgatcggctccgccgagatca aaagtccttaaaaggaagaggcaacaccc	-	*
CY066595	Human H1N1pdm2009 IAVs	Human	H1N1	pdm	2009	USA	A/San_Diego/INS204/2009	ggtgatgccccattccttgatcggctccgccgagatca aaagtccttaaaaggaagaggcaacaccc	-	*
CY054823	Human H1N1pdm2009 IAVs	Human	H1N1	pdm	2009	USA	A/California/VRDL28/2009	ggtgatgccccattccttgatcggctccgccgagatca aaagtccttaaaaggaagaggcaacaccc	-	*
CY065956	Human H1N1pdm2009 IAVs	Human	H1N1	pdm	2009	Japan	A/Japan/NHRC0001/2009	ggtgatgccccattccttgatcggctccgccgagatca aaagtccttaaaaggaagaggcaacaccc	-	*
CY065964	Human H1N1pdm2009 IAVs	Human	H1N1	pdm	2009	Japan	A/Japan/NHRC0002/2009	ggtgatgccccattccttgatcggctccgccgagatca aaagtccttaaaaggaagaggcaacaccc	-	*
CY068918	Human H1N1pdm2009 IAVs	Human	H1N1	pdm	2009	Japan	A/Japan/NHRC0003/2009	ggtgatgccccattccttgatcggctccgccgagatca aaagtccttaaaaggaagaggcaacaccc	-	*
CY062062	Human H1N1pdm2009 IAVs	Human	H1N1		2009	USA	A/New_York/0461/2009	ggtgatgccccattccttgatcggctccgccgagatca aaagtccttaaaaggaagaggcaacaccc	-	*
CY065999	Human H1N1pdm2009 IAVs	Human	H1N1	pdm	2009	Mongolia	A/Ulgii/9911/2009	ggtgatgccccattccttgatcggctccgccgagatca aaagtccttaaaaggaagaggcaacaccc	-	*
CY089359	Human H1N1pdm2009 IAVs	Human	H1N1	pdm	2009	USA	A/Boston/690/2009	ggtgatgccccattccttgatcggctccgccgagatca aaagtccttaaaaggaagaggcaacaccc	-	*
CY066251	Human H1N1pdm2009 IAVs	Human	H1N1	pdm	2009	USA	A/California/VRDL109/2009	ggtgatgccccattccttgatcggctccgccgagatca aaagtccttaaaaggaagaggcaacaccc	-	*
CY055459	Human H1N1pdm2009 IAVs	Human	H1N1	pdm	2009	USA	A/California/VRDL12/2009	ggtgatgccccattccttgatcggctccgccgagatca aaagtccttaaaaggaagaggcaacaccc	-	*
CY057538	Human H1N1pdm2009 IAVs	Human	H1N1	pdm	2009	USA	A/Wisconsin/629_D00250/2009	ggtgatgccccattccttgatcggctccgccgagatca aaagtccttaaaaggaagaggcaacaccc	-	*
CY051579	Human H1N1pdm2009 IAVs	Human	H1N1	pdm	2009	USA	A/New_York/4500/2009	ggtgatgccccattccttgatcggctccgccgagatca aaagtccttaaaaggaagaggcaacaccc	-	*
CY128183	Human H1N1pdm2009 IAVs	Human	H1N1	pdm	2009	Viet_Nam	A/Viet_Nam/13032016/2009	ggtgatgccccattccttgatcggctccgccgagatca aaagtccttaaaaggaagaggcaacaccc	-	*
CY066955	Human H1N1pdm2009 IAVs	Human	H1N1	pdm	2009	Denmark	A/Aarhus/INS238/2009	ggtgatgccccattccttgatcggctccgccgagatca aaagtccttaaaaggaagaggcaacaccc	-	*
GQ253493	Human H1N1pdm2009 IAVs	Human	H1N1	pdm	2009	China	A/Shanghai/37T/2009	ggtgatgccccattccttgatcggctccgccgagatca aaagtccttaaaaggaagaggcaacaccc	-	*
GQ290437	Human H1N1pdm2009 IAVs	Human	H1N1	pdm	2009	China	A/Shanghai/60T/2009	ggtgatgccccattccttgatcggctccgccgagatca aaagtccttaaaaggaagaggcaacaccc	-	*
CY055625	Human H1N1pdm2009 IAVs	Human	H1N1	pdm	2009	Australia	A/Australia/23/2009	ggtgatgccccattccttgatcggctccgccgagatca aaagtccttaaaaggaagaggcaacaccc	-	*
CY055848	Human H1N1pdm2009 IAVs	Human	H1N1	pdm	2009	Australia	A/Australia/61/2009	ggtgatgccccattccttgatcggctccgccgagatca aaagtccttaaaaggaagaggcaacaccc	-	*
HQ712177	Human H1N1pdm2009 IAVs	Human	H1N1	pdm	2009	Australia	A/Brisbane/2015/2009	ggtgatgccccattccttgatcggctccgccgagatca aaagtccttaaaaggaagaggcaacaccc	-	*

CY111983	Human H1N1pdm2009 IAVs	Human	H1N1	pdm	2009	Hong_Kong	A/Hong_Kong/H090_765_V10/2009	ggtgatgccccattccttgatcggctccgccgagatcaaaagtccttaaaaggaagaggcaacaccc	-	*
CY120393	Human H1N1pdm2009 IAVs	Human	H1N1	pdm	2009	Hong_Kong	A/Hong_Kong/H090_765_V10/2009	ggtgatgccccattccttgatcggctccgccgagatcaaaagtccttaaaaggaagaggcaacaccc	-	*
CY080444	Human H1N1pdm2009 IAVs	Swine	H1N1		2009	Australia	A/swine/QLD/09_02865_07/2009	ggtgatgccccattccttgatcggctccgccgagatcaaaagtccttaaaaggaagaggcaacaccc	-	*
KJ130152	Human H1N1pdm2009 IAVs	Human	H1N1		2009	New_Zealand	A/New_Zealand/1212c/2009	ggtgatgccccattccttgatcggctccgccgagatcaaaagtccttaaaaggaagaggcaacaccc	-	*
KJ130208	Human H1N1pdm2009 IAVs	Human	H1N1	pdm	2009	New_Zealand	A/New_Zealand/1212e/2009	ggtgatgccccattccttgatcggctccgccgagatcaaaagtccttaaaaggaagaggcaacaccc	-	*
KJ130192	Human H1N1pdm2009 IAVs	Human	H1N1		2009	New_Zealand	A/New_Zealand/1212f/2009	ggtgatgccccattccttgatcggctccgccgagatcaaaagtccttaaaaggaagaggcaacaccc	-	*
GU136022	Human H1N1pdm2009 IAVs	Human	H1N1		2009	Japan	A/Wakayama/57/2009	ggtgatgccccattccttgatcggctccgccgagatcaaaagtccttaaaaggaagaggcaacaccc	-	*
KJ130184	Human H1N1pdm2009 IAVs	Human	H1N1	pdm	2009	New_Zealand	A/New_Zealand/1212d/2009	ggtgatgccccattccttgatcggctccgccgagatcaaaagtccttaaaaggaagaggcaacaccc	-	*
CY123342	Human H1N1pdm2009 IAVs	Human	H1N1		2009	Singapore	A/Singapore/GP674/2009	ggtgatgccccattccttgatcggctccgccgagatcaaaagtccttaaaaggaagaggcaacaccc	-	*
CY063784	Human H1N1pdm2009 IAVs	Human	H1N1	pdm	2009	Singapore	A/Singapore/ON0801/2009	ggtgatgccccattccttgatcggctccgccgagatcaaaagtccttaaaaggaagaggcaacaccc	-	*
CY123394	Human H1N1pdm2009 IAVs	Human	H1N1	pdm	2009	Singapore	A/Singapore/ON1069/2009	ggtgatgccccattccttgatcggctccgccgagatcaaaagtccttaaaaggaagaggcaacaccc	-	*
CY124075	Human H1N1pdm2009 IAVs	Human	H1N1	pdm	2009	Singapore	A/Singapore/ON804/2009	ggtgatgccccattccttgatcggctccgccgagatcaaaagtccttaaaaggaagaggcaacaccc	-	*
CY123358	Human H1N1pdm2009 IAVs	Human	H1N1	pdm	2009	Singapore	A/Singapore/GP835/2009	ggtgatgccccattccttgatcggctccgccgagatcaaaagtccttaaaaggaagaggcaacaccc	-	*
KC833445	Human H1N1pdm2009 IAVs	Swine	H1N1		2009	Thailand	A/swine/Thailand/NS393/2009	ggtgatgccccattccttgatcggctccgccgagatcaaaagtccttaaaaggaagaggcaacaccc	-	*
KC782318	Human H1N1pdm2009 IAVs	Human	H1N1	pdm	2009	USA	A/Minnesota/16/2009	ggtgatgccccattccttgatcggctccgccgagatcaaaagtccttaaaaggaagaggcaacaccc	-	*
JF714013	Human H1N1pdm2009 IAVs	Swine	H1N1		2009	Canada	A/SW/SK/11_16/2009	ggtgatgccccattccttgatcggctccgccgagatcaaaagtccttaaaaggaagaggcaacaccc	-	*
JF714005	Human H1N1pdm2009 IAVs	Swine	H1N1		2009	Canada	A/SW/SK/12_71/2009	ggtgatgccccattccttgatcggctccgccgagatcaaaagtccttaaaaggaagaggcaacaccc	-	*
CY124047	Human H1N1pdm2009 IAVs	Human	H1N1	pdm	2009	Singapore	A/Singapore/ON799/2009	ggtgatgccccattccttgatcggctccgccgagatcaaaagtccttaaaaggaagaggcaacaccc	-	*
CY095732	Human H1N1pdm2009 IAVs	Human	H1N1		2009	China	A/Zhejiang/10/2009	ggtgatgccccattccttgatcggctccgccgagatcaaaagtccttaaaaggaagaggcaacaccc	-	*
CY095752	Human H1N1pdm2009 IAVs	Human	H1N1		2009	China	A/Zhejiang/X2/2009	ggtgatgccccattccttgatcggctccgccgagatcaaaagtccttaaaaggaagaggcaacaccc	-	*
GQ866963	Human H1N1pdm2009 IAVs	Human	H1N1	pdm	2009	Thailand	A/Thailand/CU_H9/2009	ggtgatgccccattccttgatcggctccgccgagatcaaaagtccttaaaaggaagaggcaacaccc	-	*
CY055800	Human H1N1pdm2009 IAVs	Human	H1N1	pdm	2009	Australia	A/Australia/54/2009	ggtgatgccccattccttgatcggctccgccgagatcaaaagtccttaaaaggaagaggcaacaccc	-	*
CY083252	Human H1N1pdm2009 IAVs	Human	H1N1		2009	Russia	A/Moscow/WRAIR1627T/2009	ggtgatgccccattccttgatcggctccgccgagatcaaaagtccttaaaaggaagaggcaacaccc	-	*
CY071510	Human H1N1pdm2009 IAVs	Human	H1N1	pdm	2009	USA	A/California/WR1317P/2009	ggtgatgccccattccttgatcggctccgccgagatcaaaagtccttaaaaggaagaggcaacaccc	-	*
KC780559	Human H1N1pdm2009 IAVs	Human	H1N1	pdm	2009	USA	A/Minnesota/15/2009	ggtgatgccccattccttgatcggctccgccgagatcaaaagtccttaaaaggaagaggcaacaccc	-	*

CY083363	Human H1N1pdm2009 IAVs	Human	H1N1	pdm	2009	USA	A/San_Diego/WRAIR1658P/2009	ggtgatgccccattccttgatcggctccgccgagatca aaagtccttaaaaggaagaggcaacaccc	-	*
CY095845	Human H1N1pdm2009 IAVs	Human	H1N1	pdm	2009	China	A/Zhejiang/7/2009	ggtgatgccccattccttgatcggctccgccgagatca aaagtccttaaaaggaagaggcaacaccc	-	*
JN207844	Human H1N1pdm2009 IAVs	Human	H1N1	pdm	2009	China	A/Zhejiang/89w/2009	ggtgatgccccattccttgatcggctccgccgagatca aaagtccttaaaaggaagaggcaacaccc	-	*
CY095983	Human H1N1pdm2009 IAVs	Human	H1N1	pdm	2009	China	A/Zhejiang/9/2009	ggtgatgccccattccttgatcggctccgccgagatca aaagtccttaaaaggaagaggcaacaccc	-	*
CY075018	Human H1N1pdm2009 IAVs	Human	H1N1	pdm	2009	Thailand	A/Thailand/CU_H910/2009	ggtgatgccccattccttgatcggctccgccgagatca aaagtccttaaaaggaagaggcaacaccc	-	*
CY062815	Human H1N1pdm2009 IAVs	Human	H1N1	pdm	2009	Denmark	A/Odense/INS143/2009	ggtgatgccccattccttgatcggctccgccgagatca aaagtccttaaaaggaagaggcaacaccc	-	*
CY123410	Human H1N1pdm2009 IAVs	Human	H1N1	pdm	2009	Singapore	A/Singapore/ON1079/2009	ggtgatgccccattccttgatcggctccgccgagatca aaagtccttaaaaggaagaggcaacaccc	-	*
GU136013	Human H1N1pdm2009 IAVs	Human	H1N1	pdm	2009	Myanmar	A/Myanmar/JP131/2009	ggtgatgccccattccttgatcggctccgccgagatca aaagtccttaaaaggaagaggcaacaccc	-	*
GU136014	Human H1N1pdm2009 IAVs	Human	H1N1	pdm	2009	Myanmar	A/Myanmar/JP133/2009	ggtgatgccccattccttgatcggctccgccgagatca aaagtccttaaaaggaagaggcaacaccc	-	*
CY056655	Human H1N1pdm2009 IAVs	Human	H1N1	pdm	2009	USA	A/New_York/6110/2009	ggtgatgccccattccttgatcggctccgccgagatca aaagtccttaaaaggaagaggcaacaccc	-	*
CY083244	Human H1N1pdm2009 IAVs	Human	H1N1	pdm	2009	USA	A/Virginia/WRAIR1511P/2009	ggtgatgccccattccttgatcggctccgccgagatca aaagtccttaaaaggaagaggcaacaccc	-	*
KC782334	Human H1N1pdm2009 IAVs	Human	H1N1	pdm	2009	USA	A/Georgia/25/2009	ggtgatgccccattccttgatcggctccgccgagatca aaagtccttaaaaggaagaggcaacaccc	-	*
CY062230	Human H1N1pdm2009 IAVs	Human	H1N1	pdm	2009	USA	A/California/VRDL83/2009	ggtgatgccccattccttgatcggctccgccgagatca aaagtccttaaaaggaagaggcaacaccc	-	*
CY052851	Human H1N1pdm2009 IAVs	Human	H1N1	pdm	2009	USA	A/Texas/44152535/2009	ggtgatgccccattccttgatcggctccgccgagatca aaagtccttaaaaggaagaggcaacaccc	-	*
GU136011	Human H1N1pdm2009 IAVs	Human	H1N1	pdm	2009	Myanmar	A/Myanmar/JP90/2009	ggtgatgccccattccttgatcggctccgccgagatca aaagtccttaaaaggaagaggcaacaccc	-	*
CY061231	Human H1N1pdm2009 IAVs	Human	H1N1	pdm	2009	USA	A/San_Diego/INS101/2009	ggtgatgccccattccttgatcggctccgccgagatca aaagtccttaaaaggaagaggcaacaccc	-	*
CY075600	Human H1N1pdm2009 IAVs	Human	H1N1	pdm	2009	USA	A/Boston/672/2009	ggtgatgccccattccttgatcggctccgccgagatca aaagtccttaaaaggaagaggcaacaccc	-	*
CY120719	Human H1N1pdm2009 IAVs	Human	H1N1	pdm	2009	Brazil	A/Brazil/AVS03/2009	ggtgatgccccattccttgatcggctccgccgagatca aaagtccttaaaaggaagaggcaacaccc	-	*
GQ132166	Human H1N1pdm2009 IAVs	Human	H1N1	pdm	2009	Canada	A/Canada_NS/RV1538/2009	ggtgatgccccattccttgatcggctccgccgagatca aaagtccttaaaaggaagaggcaacaccc	-	*
HM568044	Human H1N1pdm2009 IAVs	Human	H1N1	pdm	2009	United_Kingdom	A/England/898/2009	ggtgatgccccattccttgatcggctccgccgagatca aaagtccttaaaaggaagaggcaacaccc	-	*
CY057778	Human H1N1pdm2009 IAVs	Human	H1N1	pdm	2009	USA	A/Wisconsin/629_D00802/2009	ggtgatgccccattccttgatcggctccgccgagatca aaagtccttaaaaggaagaggcaacaccc	-	*
GQ465720	Human H1N1pdm2009 IAVs	Human	H1N1	pdm	2009	Canada	A/Canada_SK/RV2486/2009	ggtgatgccccattccttgatcggctccgccgagatca aaagtccttaaaaggaagaggcaacaccc	-	*
CY072730	Human H1N1pdm2009 IAVs	Human	H1N1	pdm	2009	Nicaragua	A/Managua/4407.02/2009	ggtgatgccccattccttgatcggctccgccgagatca aaagtccttaaaaggaagaggcaacaccc	-	*
CY060730	Human H1N1pdm2009 IAVs	Human	H1N1	pdm	2009	Canada	A/Ontario/328474/2009	ggtgatgccccattccttgatcggctccgccgagatca aaagtccttaaaaggaagaggcaacaccc	-	*
CY055561	Human H1N1pdm2009 IAVs	Human	H1N1	pdm	2009	Australia	A/Australia/11/2009	ggtgatgccccattccttgatcggctccgccgagatca aaagtccttaaaaggaagaggcaacaccc	-	*

CY071019	Human H1N1pdm2009 IAVs	Human	H1N1	pdm	2009	Guam	A/Guam/NHRC0032/2009	ggtgatgccccattccttgatcggctccgccgagatca aaagtccttaaaaggaagaggcaacaccc	-	*
CY057770	Human H1N1pdm2009 IAVs	Human	H1N1	pdm	2009	USA	A/Wisconsin/629_D01346/2009	ggtgatgccccattccttgatcggctccgccgagatca aaagtccttaaaaggaagaggcaacaccc	-	*
KC780431	Human H1N1pdm2009 IAVs	Human	H1N1	pdm	2009	USA	A/Texas/71/2009	ggtgatgccccattccttgatcggctccgccgagatca aaagtccttaaaaggaagaggcaacaccc	-	*
CY073645	Human H1N1pdm2009 IAVs	Human	H1N1	pdm	2009	Nicaragua	A/Managua/1973.02/2009	ggtgatgccccattccttgatcggctccgccgagatca aaagtccttaaaaggaagaggcaacaccc	-	*
CY058112	Human H1N1pdm2009 IAVs	Human	H1N1		2009	Nicaragua	A/Managua/2152.01/2009	ggtgatgccccattccttgatcggctccgccgagatca aaagtccttaaaaggaagaggcaacaccc	-	*
CY072898	Human H1N1pdm2009 IAVs	Human	H1N1	pdm	2009	Nicaragua	A/Managua/3214.04/2009	ggtgatgccccattccttgatcggctccgccgagatca aaagtccttaaaaggaagaggcaacaccc	-	*
CY072578	Human H1N1pdm2009 IAVs	Human	H1N1	pdm	2009	Nicaragua	A/Managua/3231.02/2009	ggtgatgccccattccttgatcggctccgccgagatca aaagtccttaaaaggaagaggcaacaccc	-	*
CY058152	Human H1N1pdm2009 IAVs	Human	H1N1		2009	Nicaragua	A/Managua/4085.02/2009	ggtgatgccccattccttgatcggctccgccgagatca aaagtccttaaaaggaagaggcaacaccc	-	*
CY073637	Human H1N1pdm2009 IAVs	Human	H1N1	pdm	2009	Nicaragua	A/Managua/4960.01/2009	ggtgatgccccattccttgatcggctccgccgagatca aaagtccttaaaaggaagaggcaacaccc	-	*
CY058224	Human H1N1pdm2009 IAVs	Human	H1N1	pdm	2009	Nicaragua	A/Managua/5221.03/2009	ggtgatgccccattccttgatcggctccgccgagatca aaagtccttaaaaggaagaggcaacaccc	-	*
CY058128	Human H1N1pdm2009 IAVs	Human	H1N1	pdm	2009	Nicaragua	A/Managua/5453.01/2009	ggtgatgccccattccttgatcggctccgccgagatca aaagtccttaaaaggaagaggcaacaccc	-	*
CY073557	Human H1N1pdm2009 IAVs	Human	H1N1	pdm	2009	Nicaragua	A/Managua/2978.04/2009	ggtgatgccccattccttgatcggctccgccgagatca aaagtccttaaaaggaagaggcaacaccc	-	*
FJ984349	Human H1N1pdm2009 IAVs	Human	H1N1	pdm	2009	USA	A/New_York/18/2009	ggtgatgccccattccttgatcggctccgccgagatca aaagtccttaaaaggaagaggcaacaccc	-	*
GQ232063	Human H1N1pdm2009 IAVs	Human	H1N1	pdm	2009	USA	A/New_York/18/2009	ggtgatgccccattccttgatcggctccgccgagatca aaagtccttaaaaggaagaggcaacaccc	-	*
CY041118	Human H1N1pdm2009 IAVs	Human	H1N1	pdm	2009	USA	A/New_York/3193/2009	ggtgatgccccattccttgatcggctccgccgagatca aaagtccttaaaaggaagaggcaacaccc	-	*
CY052262	Human H1N1pdm2009 IAVs	Human	H1N1		2009	USA	A/Texas/42221280/2009	ggtgatgccccattccttgatcggctccgccgagatca aaagtccttaaaaggaagaggcaacaccc	-	*
CY058066	Human H1N1pdm2009 IAVs	Human	H1N1	pdm	2009	USA	A/Texas/46181292/2009	ggtgatgccccattccttgatcggctccgccgagatca aaagtccttaaaaggaagaggcaacaccc	-	*
CY054807	Human H1N1pdm2009 IAVs	Human	H1N1	pdm	2009	USA	A/California/VRDL26/2009	ggtgatgccccattccttgatcggctccgccgagatca aaagtccttaaaaggaagaggcaacaccc	-	*
JN187328	Human H1N1pdm2009 IAVs	Human	H1N1	pdm	2009	Taiwan	A/Taiwan/2826/2009	ggtgatgccccattccttgatcggctccgccgagatca aaagtccttaaaaggaagaggcaacaccc	-	*
KC780134	Human H1N1pdm2009 IAVs	Human	H1N1	pdm	2009	USA	A/New_Mexico/08/2009	ggtgatgccccattccttgatcggctccgccgagatca aaagtccttaaaaggaagaggcaacaccc	-	*
CY052795	Human H1N1pdm2009 IAVs	Human	H1N1	pdm	2009	USA	A/Texas/45072273/2009	ggtgatgccccattccttgatcggctccgccgagatca aaagtccttaaaaggaagaggcaacaccc	-	*
CY052915	Human H1N1pdm2009 IAVs	Human	H1N1	pdm	2009	USA	A/Texas/45113911/2009	ggtgatgccccattccttgatcggctccgccgagatca aaagtccttaaaaggaagaggcaacaccc	-	*
CY060831	Human H1N1pdm2009 IAVs	Human	H1N1	pdm	2009	USA	A/Texas/46240925/2009	ggtgatgccccattccttgatcggctccgccgagatca aaagtccttaaaaggaagaggcaacaccc	-	*
CY057866	Human H1N1pdm2009 IAVs	Human	H1N1	pdm	2009	USA	A/Wisconsin/629_D00936/2009	ggtgatgccccattccttgatcggctccgccgagatca aaagtccttaaaaggaagaggcaacaccc	-	*
CY052443	Human H1N1pdm2009 IAVs	Human	H1N1	pdm	2009	USA	A/Texas/45122538/2009	ggtgatgccccattccttgatcggctccgccgagatca aaagtccttaaaaggaagaggcaacaccc	-	*

CY062022	Human H1N1pdm2009 IAVs	Human	H1N1		2009	USA	A/New_York/7216/2009	ggtgatgccccattccttgatcggctccgccgagatca aaagtccttaaaaggaagaggcaacaccc	-	*
CY051219	Human H1N1pdm2009 IAVs	Human	H1N1	pdm	2009	USA	A/Wisconsin/629_D02292/2009	ggtgatgccccattccttgatcggctccgccgagatca aaagtccttaaaaggaagaggcaacaccc	-	*
CY051467	Human H1N1pdm2009 IAVs	Human	H1N1	pdm	2009	USA	A/Wisconsin/629_S0314/2009	ggtgatgccccattccttgatcggctccgccgagatca aaagtccttaaaaggaagaggcaacaccc	-	*
GQ214154	Human H1N1pdm2009 IAVs	Human	H1N1	pdm	2009	France	A/Paris/2573/2009	ggtgatgccccattccttgatcggctccgccgagatca aaagtccttaaaaggaagaggcaacaccc	-	*
CY100452	Human H1N1pdm2009 IAVs	Human	H1N1		2009	Mexico	A/Mexico_City/IBT22/2009	ggtgatgccccattccttgatcggctccgccgagatca aaagtccttaaaaggaagaggcaacaccc	-	*
CY147565	Human H1N1pdm2009 IAVs	Human	H1	pdm	2009	Mexico	A/Mexico/24041/2009	ggtgatgccccattccttgatcggctccgccgagatca aaagtccttaaaaggaagaggcaacaccc	-	*
CY077599	Human H1N1pdm2009 IAVs	Human	H1N1	pdm	2009	Mexico	A/Mexico/LaGloria_3/2009	ggtgatgccccattccttgatcggctccgccgagatca aaagtccttaaaaggaagaggcaacaccc	-	*
CY077615	Human H1N1pdm2009 IAVs	Human	H1N1	pdm	2009	Mexico	A/Mexico/LaGloria_8/2009	ggtgatgccccattccttgatcggctccgccgagatca aaagtccttaaaaggaagaggcaacaccc	-	*
CY077607	Human H1N1pdm2009 IAVs	Human	H1N1	pdm	2009	Mexico	A/Mexico/LaGloria_4/2009	ggtgatgccccattccttgatcggctccgccgagatca aaagtccttaaaaggaagaggcaacaccc	-	*
CY062222	Human H1N1pdm2009 IAVs	Human	H1N1		2009	USA	A/California/VRDL82/2009	ggtgatgccccattccttgatcggctccgccgagatca aaagtccttaaaaggaagaggcaacaccc	-	*
CY057906	Human H1N1pdm2009 IAVs	Human	H1N1	pdm	2009	USA	A/Wisconsin/629_D02448/2009	ggtgatgccccattccttgatcggctccgccgagatca aaagtccttaaaaggaagaggcaacaccc	-	*
HM189598	Human H1N1pdm2009 IAVs	Swine	H1N1		2009	South_Korea	A/swine/Korea/SCJ08/2009	ggtgatgccccattccttgatcggctccgccgagatca aaagtccttaaaaggaagaggcaacaccc	-	*
CY055569	Human H1N1pdm2009 IAVs	Human	H1N1	pdm	2009	Australia	A/Australia/12/2009	ggtgatgccccattccttgatcggctccgccgagatca aaagtccttaaaaggaagaggcaacaccc	-	*
CY055688	Human H1N1pdm2009 IAVs	Human	H1N1	pdm	2009	Australia	A/Australia/32/2009	ggtgatgccccattccttgatcggctccgccgagatca aaagtccttaaaaggaagaggcaacaccc	-	*
CY054999	Human H1N1pdm2009 IAVs	Human	H1N1	pdm	2009	USA	A/California/VRDL61/2009	ggtgatgccccattccttgatcggctccgccgagatca aaagtccttaaaaggaagaggcaacaccc	-	*
CY122735	Human H1N1pdm2009 IAVs	Human	H1N1	pdm	2009	Singapore	A/Singapore/GP1163/2009	ggtgatgccccattccttgatcggctccgccgagatca aaagtccttaaaaggaagaggcaacaccc	-	*
CY055776	Human H1N1pdm2009 IAVs	Human	H1N1	pdm	2009	Australia	A/Australia/48/2009	ggtgatgccccattccttgatcggctccgccgagatca aaagtccttaaaaggaagaggcaacaccc	-	*
CY061039	Human H1N1pdm2009 IAVs	Human	H1N1	pdm	2009	USA	A/Texas/JMS390/2009	ggtgatgccccattccttgatcggctccgccgagatca aaagtccttaaaaggaagaggcaacaccc	-	*
HM230697	Human H1N1pdm2009 IAVs	Human	H1N1	pdm	2009	China	A/Zhoushan/1/2009	ggtgatgccccattccttgatcggctccgccgagatca aaagtccttaaaaggaagaggcaacaccc	-	*
CY119494	Human H1N1pdm2009 IAVs	Human	H1N1	pdm	2009	Malaysia	A/Malaysia/2173411/2009	ggtgatgccccattccttgatcggctccgccgagatca aaagtccttaaaaggaagaggcaacaccc	-	*
CY058744	Human H1N1pdm2009 IAVs	Human	H1N1	pdm	2009	USA	A/Texas/JMS378/2009	ggtgatgccccattccttgatcggctccgccgagatca aaagtccttaaaaggaagaggcaacaccc	-	*
CY060951	Human H1N1pdm2009 IAVs	Human	H1N1	pdm	2009	USA	A/Texas/JMS379/2009	ggtgatgccccattccttgatcggctccgccgagatca aaagtccttaaaaggaagaggcaacaccc	-	*
CY075512	Human H1N1pdm2009 IAVs	Human	H1N1		2009	USA	A/Boston/595/2009	ggtgatgccccattccttgatcggctccgccgagatca aaagtccttaaaaggaagaggcaacaccc	-	*
CY075520	Human H1N1pdm2009 IAVs	Human	H1N1	pdm	2009	USA	A/Boston/602/2009	ggtgatgccccattccttgatcggctccgccgagatca aaagtccttaaaaggaagaggcaacaccc	-	*
CY089263	Human H1N1pdm2009 IAVs	Human	H1N1	pdm	2009	USA	A/Boston/650/2009	ggtgatgccccattccttgatcggctccgccgagatca aaagtccttaaaaggaagaggcaacaccc	-	*

CY089271	Human H1N1pdm2009 IAVs	Human	H1N1	pdm	2009	USA	A/Boston/657/2009	ggtgatgccccattccttgatcggctccgccgagatcaaaagtccttaaaaggaagaggcaacaccc	-	*
CY089303	Human H1N1pdm2009 IAVs	Human	H1N1	pdm	2009	USA	A/Boston/671/2009	ggtgatgccccattccttgatcggctccgccgagatcaaaagtccttaaaaggaagaggcaacaccc	-	*
CY075632	Human H1N1pdm2009 IAVs	Human	H1N1		2009	USA	A/Boston/701/2009	ggtgatgccccattccttgatcggctccgccgagatcaaaagtccttaaaaggaagaggcaacaccc	-	*
CY057290	Human H1N1pdm2009 IAVs	Human	H1N1	pdm	2009	USA	A/New_York/5271/2009	ggtgatgccccattccttgatcggctccgccgagatcaaaagtccttaaaaggaagaggcaacaccc	-	*
CY061998	Human H1N1pdm2009 IAVs	Human	H1N1	pdm	2009	USA	A/New_York/7020/2009	ggtgatgccccattccttgatcggctccgccgagatcaaaagtccttaaaaggaagaggcaacaccc	-	*
CY060791	Human H1N1pdm2009 IAVs	Human	H1N1	pdm	2009	USA	A/Texas/46222134/2009	ggtgatgccccattccttgatcggctccgccgagatcaaaagtccttaaaaggaagaggcaacaccc	-	*
CY058002	Human H1N1pdm2009 IAVs	Human	H1N1		2009	USA	A/Wisconsin/629_D02299/2009	ggtgatgccccattccttgatcggctccgccgagatcaaaagtccttaaaaggaagaggcaacaccc	-	*
KC780715	Human H1N1pdm2009 IAVs	Human	H1N1	pdm	2009	USA	A/Rhode_Island/18/2009	ggtgatgccccattccttgatcggctccgccgagatcaaaagtccttaaaaggaagaggcaacaccc	-	*
AB704466	Human H1N1pdm2009 IAVs	Human	H1N1	pdm	2009	Japan	A/Tochigi/350/2009	ggtgatgccccattccttgatcggctccgccgagatcaaaagtccttaaaaggaagaggcaacaccc	-	*
CY065820	Human H1N1pdm2009 IAVs	Human	H1N1	pdm	2009	Netherlands	A/Netherlands/2442b/2009	ggtgatgccccattccttgatcggctccgccgagatcaaaagtccttaaaaggaagaggcaacaccc	-	*
CY095739	Human H1N1pdm2009 IAVs	Human	H1N1	pdm	2009	China	A/Hubei/100/2009	ggtgatgccccattccttgatcggctccgccgagatcaaaagtccttaaaaggaagaggcaacaccc	-	*
HQ840298	Human H1N1pdm2009 IAVs	Swine	H1N1		2009	USA	A/swine/Minnesota/074A/2009	ggtgatgccccattccttgatcggctccgccgagatcaaaagtccttaaaaggaagaggcaacaccc	-	*
HQ840329	Human H1N1pdm2009 IAVs	Swine	H1N1		2009	USA	A/swine/Minnesota/165A/2009	ggtgatgccccattccttgatcggctccgccgagatcaaaagtccttaaaaggaagaggcaacaccc	-	*
KC780994	Human H1N1pdm2009 IAVs	Human	H1N1	pdm	2009	USA	A/Texas/66/2009	ggtgatgccccattccttgatcggctccgccgagatcaaaagtccttaaaaggaagaggcaacaccc	-	*
KC781022	Human H1N1pdm2009 IAVs	Human	H1N1		2009	USA	A/North_Carolina/43/2009	ggtgatgccccattccttgatcggctccgccgagatcaaaagtccttaaaaggaagaggcaacaccc	-	*
KC781728	Human H1N1pdm2009 IAVs	Human	H1N1	pdm	2009	USA	A/Oregon/30/2009	ggtgatgccccattccttgatcggctccgccgagatcaaaagtccttaaaaggaagaggcaacaccc	-	*
JQ612503	Human H1N1pdm2009 IAVs	Human	H1N1	pdm	2009	Hungary	A/Hungary/40/2009	ggtgatgccccattccttgatcggctccgccgagatcaaaagtccttaaaaggaagaggcaacaccc	-	*
CY053649	Human H1N1pdm2009 IAVs	Swine	H1N1		2009	Mexico	A/swine/4/Mexico/2009	ggtgatgccccattccttgatcggctccgccgagatcaaaagtccttaaaaggaagaggcaacaccc	-	*
KC781789	Human H1N1pdm2009 IAVs	Human	H1N1	pdm	2009	USA	A/Wisconsin/26/2009	ggtgatgccccattccttgatcggctccgccgagatcaaaagtccttaaaaggaagaggcaacaccc	-	*
GQ232008	Human H1N1pdm2009 IAVs	Human	H1N1	pdm	2009	USA	A/Kentucky/05/2009	ggtgatgccccattccttgatcggctccgccgagatcaaaagtccttaaaaggaagaggcaacaccc	-	*
CY066219	Human H1N1pdm2009 IAVs	Human	H1N1	pdm	2009	USA	A/California/VRDL105/2009	ggtgatgccccattccttgatcggctccgccgagatcaaaagtccttaaaaggaagaggcaacaccc	-	*
CY066387	Human H1N1pdm2009 IAVs	Human	H1N1	pdm	2009	USA	A/California/VRDL126/2009	ggtgatgccccattccttgatcggctccgccgagatcaaaagtccttaaaaggaagaggcaacaccc	-	*
CY063103	Human H1N1pdm2009 IAVs	Human	H1N1	pdm	2009	USA	A/California/VRDL91/2009	ggtgatgccccattccttgatcggctccgccgagatcaaaagtccttaaaaggaagaggcaacaccc	-	*
CY056919	Human H1N1pdm2009 IAVs	Human	H1N1	pdm	2009	USA	A/San_Diego/INS61/2009	ggtgatgccccattccttgatcggctccgccgagatcaaaagtccttaaaaggaagaggcaacaccc	-	*
CY060839	Human H1N1pdm2009 IAVs	Human	H1N1	pdm	2009	USA	A/Texas/46241654/2009	ggtgatgccccattccttgatcggctccgccgagatcaaaagtccttaaaaggaagaggcaacaccc	-	*

CY062254	Human H1N1pdm2009 IAVs	Human	H1N1	pdm	2009	USA	A/California/VRDL86/2009	ggtgatgccccattccttgatcggctccgccgagatcaaaagtccttaaaaggaagaggcaacaccc	-	*
CY050947	Human H1N1pdm2009 IAVs	Human	H1N1	pdm	2009	USA	A/Wisconsin/629_D00374/2009	ggtgatgccccattccttgatcggctccgccgagatcaaaagtccttaaaaggaagaggcaacaccc	-	*
CY050987	Human H1N1pdm2009 IAVs	Human	H1N1	pdm	2009	USA	A/Wisconsin/629_D00592/2009	ggtgatgccccattccttgatcggctccgccgagatcaaaagtccttaaaaggaagaggcaacaccc	-	*
CY072842	Human H1N1pdm2009 IAVs	Human	H1N1		2009	Nicaragua	A/Managua/265.01/2009	ggtgatgccccattccttgatcggctccgccgagatcaaaagtccttaaaaggaagaggcaacaccc	-	*
CY073661	Human H1N1pdm2009 IAVs	Human	H1N1	pdm	2009	Nicaragua	A/Managua/3225.03/2009	ggtgatgccccattccttgatcggctccgccgagatcaaaagtccttaaaaggaagaggcaacaccc	-	*
CY072626	Human H1N1pdm2009 IAVs	Human	H1N1	pdm	2009	Nicaragua	A/Managua/5339.04/2009	ggtgatgccccattccttgatcggctccgccgagatcaaaagtccttaaaaggaagaggcaacaccc	-	*
CY060959	Human H1N1pdm2009 IAVs	Human	H1N1	pdm	2009	USA	A/Texas/JMS380/2009	ggtgatgccccattccttgatcggctccgccgagatcaaaagtccttaaaaggaagaggcaacaccc	-	*
CY072594	Human H1N1pdm2009 IAVs	Human	H1N1	pdm	2009	Nicaragua	A/Managua/1356.03/2009	ggtgatgccccattccttgatcggctccgccgagatcaaaagtccttaaaaggaagaggcaacaccc	-	*
CY090077	Human H1N1pdm2009 IAVs	Human	H1N1	pdm	2009	Nicaragua	A/Managua/4076.03/2009	ggtgatgccccattccttgatcggctccgccgagatcaaaagtccttaaaaggaagaggcaacaccc	-	*
CY073677	Human H1N1pdm2009 IAVs	Human	H1N1	pdm	2009	Nicaragua	A/Managua/5248.03/2009	ggtgatgccccattccttgatcggctccgccgagatcaaaagtccttaaaaggaagaggcaacaccc	-	*
KC782287	Human H1N1pdm2009 IAVs	Human	H1N1	pdm	2009	USA	A/Texas/48/2009	ggtgatgccccattccttgatcggctccgccgagatcaaaagtccttaaaaggaagaggcaacaccc	-	*
HM855241	Human H1N1pdm2009 IAVs	Human	H1N1	pdm	2009	Kenya	A/Kisumu/56/2009	ggtgatgccccattccttgatcggctccgccgagatcaaaagtccttaaaaggaagaggcaacaccc	-	*
CY053258	Human H1N1pdm2009 IAVs	Human	H1N1	pdm	2009	USA	A/Brownsville/36H/2009	ggtgatgccccattccttgatcggctccgccgagatcaaaagtccttaaaaggaagaggcaacaccc	-	*
CY051091	Human H1N1pdm2009 IAVs	Human	H1N1	pdm	2009	USA	A/Wisconsin/629_D01844/2009	ggtgatgccccattccttgatcggctccgccgagatcaaaagtccttaaaaggaagaggcaacaccc	-	*
GQ465725	Human H1N1pdm2009 IAVs	Human	H1N1	pdm	2009	Canada	A/Canada_PQ/RV1586/2009	ggtgatgccccattccttgatcggctccgccgagatcaaaagtccttaaaaggaagaggcaacaccc	-	*
CY056056	Human H1N1pdm2009 IAVs	Human	H1N1	pdm	2009	USA	A/District_of_Columbia/INS19/2009	ggtgatgccccattccttgatcggctccgccgagatcaaaagtccttaaaaggaagaggcaacaccc	-	*
GQ323466	Human H1N1pdm2009 IAVs	Human	H1N1	pdm	2009	USA	A/Kentucky/07/2009	ggtgatgccccattccttgatcggctccgccgagatcaaaagtccttaaaaggaagaggcaacaccc	-	*
CY065746	Human H1N1pdm2009 IAVs	Human	H1N1	pdm	2009	United_Kingdom	A/Scotland/10/2009	ggtgatgccccattccttgatcggctccgccgagatcaaaagtccttaaaaggaagaggcaacaccc	-	*
CY107596	Human H1N1pdm2009 IAVs	Human	H1N1	pdm	2009	United_Kingdom	A/Scotland/EastKilbride_412770/2009	ggtgatgccccattccttgatcggctccgccgagatcaaaagtccttaaaaggaagaggcaacaccc	-	*
CY107776	Human H1N1pdm2009 IAVs	Human	H1N1	pdm	2009	United_Kingdom	A/Scotland/EastKilbride_419809/2009	ggtgatgccccattccttgatcggctccgccgagatcaaaagtccttaaaaggaagaggcaacaccc	-	*
CY107783	Human H1N1pdm2009 IAVs	Human	H1N1	pdm	2009	United_Kingdom	A/Scotland/EastKilbride_420703/2009	ggtgatgccccattccttgatcggctccgccgagatcaaaagtccttaaaaggaagaggcaacaccc	-	*
CY107787	Human H1N1pdm2009 IAVs	Human	H1N1	pdm	2009	United_Kingdom	A/Scotland/EastKilbride_420874/2009	ggtgatgccccattccttgatcggctccgccgagatcaaaagtccttaaaaggaagaggcaacaccc	-	*
CY107794	Human H1N1pdm2009 IAVs	Human	H1N1	pdm	2009	United_Kingdom	A/Scotland/EastKilbride_420875/2009	ggtgatgccccattccttgatcggctccgccgagatcaaaagtccttaaaaggaagaggcaacaccc	-	*
CY107801	Human H1N1pdm2009 IAVs	Human	H1N1	pdm	2009	United_Kingdom	A/Scotland/EastKilbride_420880/2009	ggtgatgccccattccttgatcggctccgccgagatcaaaagtccttaaaaggaagaggcaacaccc	-	*
CY107808	Human H1N1pdm2009 IAVs	Human	H1N1	pdm	2009	United_Kingdom	A/Scotland/EastKilbride_420881/2009	ggtgatgccccattccttgatcggctccgccgagatcaaaagtccttaaaaggaagaggcaacaccc	-	*

CY107330	Human H1N1pdm2009 IAVs	Human	H1N1	pdm	2009	United_Kingdom	A/Scotland/Edinburgh_17715/2009	ggtgatgccccattccttgatcggctccgccgagatcaaaagtccttaaaaggaagaggcaacaccc	-	*
CY107562	Human H1N1pdm2009 IAVs	Human	H1N1		2009	United_Kingdom	A/Scotland/Glasgow_412118/2009	ggtgatgccccattccttgatcggctccgccgagatcaaaagtccttaaaaggaagaggcaacaccc	-	*
CY107574	Human H1N1pdm2009 IAVs	Human	H1N1	pdm	2009	United_Kingdom	A/Scotland/Glasgow_412311/2009	ggtgatgccccattccttgatcggctccgccgagatcaaaagtccttaaaaggaagaggcaacaccc	-	*
CY107582	Human H1N1pdm2009 IAVs	Human	H1N1		2009	United_Kingdom	A/Scotland/Glasgow_412313/2009	ggtgatgccccattccttgatcggctccgccgagatcaaaagtccttaaaaggaagaggcaacaccc	-	*
CY107604	Human H1N1pdm2009 IAVs	Human	H1N1	pdm	2009	United_Kingdom	A/Scotland/Glasgow_413375/2009	ggtgatgccccattccttgatcggctccgccgagatcaaaagtccttaaaaggaagaggcaacaccc	-	*
CY107625	Human H1N1pdm2009 IAVs	Human	H1N1	pdm	2009	United_Kingdom	A/Scotland/Glasgow_413848/2009	ggtgatgccccattccttgatcggctccgccgagatcaaaagtccttaaaaggaagaggcaacaccc	-	*
CY107639	Human H1N1pdm2009 IAVs	Human	H1N1	pdm	2009	United_Kingdom	A/Scotland/Glasgow_413886/2009	ggtgatgccccattccttgatcggctccgccgagatcaaaagtccttaaaaggaagaggcaacaccc	-	*
CY107646	Human H1N1pdm2009 IAVs	Human	H1N1	pdm	2009	United_Kingdom	A/Scotland/Glasgow_413941/2009	ggtgatgccccattccttgatcggctccgccgagatcaaaagtccttaaaaggaagaggcaacaccc	-	*
CY107652	Human H1N1pdm2009 IAVs	Human	H1N1		2009	United_Kingdom	A/Scotland/Glasgow_414159/2009	ggtgatgccccattccttgatcggctccgccgagatcaaaagtccttaaaaggaagaggcaacaccc	-	*
CY107658	Human H1N1pdm2009 IAVs	Human	H1N1	pdm	2009	United_Kingdom	A/Scotland/Glasgow_414473/2009	ggtgatgccccattccttgatcggctccgccgagatcaaaagtccttaaaaggaagaggcaacaccc	-	*
CY107665	Human H1N1pdm2009 IAVs	Human	H1N1	pdm	2009	United_Kingdom	A/Scotland/Glasgow_419707/2009	ggtgatgccccattccttgatcggctccgccgagatcaaaagtccttaaaaggaagaggcaacaccc	-	*
CY107672	Human H1N1pdm2009 IAVs	Human	H1N1		2009	United_Kingdom	A/Scotland/Glasgow_419865/2009	ggtgatgccccattccttgatcggctccgccgagatcaaaagtccttaaaaggaagaggcaacaccc	-	*
CY107679	Human H1N1pdm2009 IAVs	Human	H1N1	pdm	2009	United_Kingdom	A/Scotland/Glasgow_419977/2009	ggtgatgccccattccttgatcggctccgccgagatcaaaagtccttaaaaggaagaggcaacaccc	-	*
CY107686	Human H1N1pdm2009 IAVs	Human	H1N1	pdm	2009	United_Kingdom	A/Scotland/Glasgow_420755/2009	ggtgatgccccattccttgatcggctccgccgagatcaaaagtccttaaaaggaagaggcaacaccc	-	*
CY107690	Human H1N1pdm2009 IAVs	Human	H1N1	pdm	2009	United_Kingdom	A/Scotland/Glasgow_420768/2009	ggtgatgccccattccttgatcggctccgccgagatcaaaagtccttaaaaggaagaggcaacaccc	-	*
CY107697	Human H1N1pdm2009 IAVs	Human	H1N1	pdm	2009	United_Kingdom	A/Scotland/Glasgow_422823/2009	ggtgatgccccattccttgatcggctccgccgagatcaaaagtccttaaaaggaagaggcaacaccc	-	*
CY107555	Human H1N1pdm2009 IAVs	Human	H1N1	pdm	2009	United_Kingdom	A/Scotland/Livingston_416138/2009	ggtgatgccccattccttgatcggctccgccgagatcaaaagtccttaaaaggaagaggcaacaccc	-	*
CY107748	Human H1N1pdm2009 IAVs	Human	H1N1	pdm	2009	United_Kingdom	A/Scotland/Paisley_412182/2009	ggtgatgccccattccttgatcggctccgccgagatcaaaagtccttaaaaggaagaggcaacaccc	-	*
CY107589	Human H1N1pdm2009 IAVs	Human	H1N1	pdm	2009	United_Kingdom	A/Scotland/Paisley_412666/2009	ggtgatgccccattccttgatcggctccgccgagatcaaaagtccttaaaaggaagaggcaacaccc	-	*
CY107631	Human H1N1pdm2009 IAVs	Human	H1N1	pdm	2009	United_Kingdom	A/Scotland/Paisley_413869/2009	ggtgatgccccattccttgatcggctccgccgagatcaaaagtccttaaaaggaagaggcaacaccc	-	*
CY107755	Human H1N1pdm2009 IAVs	Human	H1N1	pdm	2009	United_Kingdom	A/Scotland/Paisley_413960/2009	ggtgatgccccattccttgatcggctccgccgagatcaaaagtccttaaaaggaagaggcaacaccc	-	*
CY051659	Human H1N1pdm2009 IAVs	Human	H1N1	pdm	2009	USA	A/New_York/4728/2009	ggtgatgccccattccttgatcggctccgccgagatcaaaagtccttaaaaggaagaggcaacaccc	-	*
CY123202	Human H1N1pdm2009 IAVs	Human	H1N1		2009	Singapore	A/Singapore/GP3912/2009	ggtgatgccccattccttgatcggctccgccgagatcaaaagtccttaaaaggaagaggcaacaccc	-	*
CY107202	Human H1N1pdm2009 IAVs	Human	H1N1		2009	United_Kingdom	A/Scotland/Paisley_419540/2009	ggtgatgccccattccttgatcggctccgccgagatcaaaagtccttaaaaggaagaggcaacaccc	-	*
KC222640	Human H1N1pdm2009 IAVs	Human	H1N1	pdm	2009	Germany	A/Jena/VI5258/2009	ggtgatgccccattccttgatcggctccgccgagatcaaaagtccttaaaaggaagaggcaacaccc	-	*

GQ339883	Human H1N1pdm2009 IAVs	Human	H1N1	pdm	2009	Mexico	A/Mexico/4283/2009	ggtgatgccccattccttgatcggctccgccgagatca aaagtccttaaaaggaagaggcaacaccc	-	*
GQ303343	Human H1N1pdm2009 IAVs	Human	H1N1	pdm	2009	Mexico	A/Mexico/4595/2009	ggtgatgccccattccttgatcggctccgccgagatca aaagtccttaaaaggaagaggcaacaccc	-	*
KC780900	Human H1N1pdm2009 IAVs	Human	H1N1	pdm	2009	USA	A/Wisconsin/51/2009	ggtgatgccccattccttgatcggctccgccgagatca aaagtccttaaaaggaagaggcaacaccc	-	*
CY058376	Human H1N1pdm2009 IAVs	Human	H1N1	pdm	2009	USA	A/Wisconsin/629_D01244/2009	ggtgatgccccattccttgatcggctccgccgagatca aaagtccttaaaaggaagaggcaacaccc	-	*
CY058416	Human H1N1pdm2009 IAVs	Human	H1N1	pdm	2009	USA	A/Wisconsin/629_D00459/2009	ggtgatgccccattccttgatcggctccgccgagatca aaagtccttaaaaggaagaggcaacaccc	-	*
CY057938	Human H1N1pdm2009 IAVs	Human	H1N1	pdm	2009	USA	A/Wisconsin/629_D00968/2009	ggtgatgccccattccttgatcggctccgccgagatca aaagtccttaaaaggaagaggcaacaccc	-	*
CY058440	Human H1N1pdm2009 IAVs	Human	H1N1	pdm	2009	USA	A/Wisconsin/629_D01556/2009	ggtgatgccccattccttgatcggctccgccgagatca aaagtccttaaaaggaagaggcaacaccc	-	*
CY058456	Human H1N1pdm2009 IAVs	Human	H1N1	pdm	2009	USA	A/Wisconsin/629_D01846/2009	ggtgatgccccattccttgatcggctccgccgagatca aaagtccttaaaaggaagaggcaacaccc	-	*
CY057882	Human H1N1pdm2009 IAVs	Human	H1N1		2009	USA	A/Wisconsin/629_D01987/2009	ggtgatgccccattccttgatcggctccgccgagatca aaagtccttaaaaggaagaggcaacaccc	-	*
CY057578	Human H1N1pdm2009 IAVs	Human	H1N1	pdm	2009	USA	A/Wisconsin/629_S1252/2009	ggtgatgccccattccttgatcggctccgccgagatca aaagtccttaaaaggaagaggcaacaccc	-	*
CY057626	Human H1N1pdm2009 IAVs	Human	H1N1	pdm	2009	USA	A/Wisconsin/629_S1313/2009	ggtgatgccccattccttgatcggctccgccgagatca aaagtccttaaaaggaagaggcaacaccc	-	*
CY090012	Human H1N1pdm2009 IAVs	Human	H1N1		2009	USA	A/Wisconsin/629_S1368/2009	ggtgatgccccattccttgatcggctccgccgagatca aaagtccttaaaaggaagaggcaacaccc	-	*
CY057738	Human H1N1pdm2009 IAVs	Human	H1N1	pdm	2009	USA	A/Wisconsin/629_D01140/2009	ggtgatgccccattccttgatcggctccgccgagatca aaagtccttaaaaggaagaggcaacaccc	-	*
CY057818	Human H1N1pdm2009 IAVs	Human	H1N1	pdm	2009	USA	A/Wisconsin/629_D01826/2009	ggtgatgccccattccttgatcggctccgccgagatca aaagtccttaaaaggaagaggcaacaccc	-	*
CY128407	Human H1N1pdm2009 IAVs	Human	H1N1	pdm	2009	Viet_Nam	A/Viet_Nam/11032017/2009	ggtgatgccccattccttgatcggctccgccgagatca aaagtccttaaaaggaagaggcaacaccc	-	*
CY044175	Human H1N1pdm2009 IAVs	Human	H1N1	pdm	2009	USA	A/Bethesda/SP506/2009	ggtgatgccccattccttgatcggctccgccgagatca aaagtccttaaaaggaagaggcaacaccc	-	*
CY043122	Human H1N1pdm2009 IAVs	Human	H1N1		2009	USA	A/Bethesda/SP508/2009	ggtgatgccccattccttgatcggctccgccgagatca aaagtccttaaaaggaagaggcaacaccc	-	*
CY045211	Human H1N1pdm2009 IAVs	Human	H1N1	pdm	2009	USA	A/New_York/4057/2009	ggtgatgccccattccttgatcggctccgccgagatca aaagtccttaaaaggaagaggcaacaccc	-	*
CY056000	Human H1N1pdm2009 IAVs	Human	H1N1	pdm	2009	USA	A/San_Diego/INS09/2009	ggtgatgccccattccttgatcggctccgccgagatca aaagtccttaaaaggaagaggcaacaccc	-	*
CY051803	Human H1N1pdm2009 IAVs	Human	H1N1	pdm	2009	USA	A/New_York/4866/2009	ggtgatgccccattccttgatcggctccgccgagatca aaagtccttaaaaggaagaggcaacaccc	-	*
CY052659	Human H1N1pdm2009 IAVs	Human	H1N1	pdm	2009	USA	A/Texas/45093846/2009	ggtgatgccccattccttgatcggctccgccgagatca aaagtccttaaaaggaagaggcaacaccc	-	*
CY055633	Human H1N1pdm2009 IAVs	Human	H1N1	pdm	2009	Australia	A/Australia/24/2009	ggtgatgccccattccttgatcggctccgccgagatca aaagtccttaaaaggaagaggcaacaccc	-	*
KC780049	Human H1N1pdm2009 IAVs	Human	H1N1	pdm	2009	USA	A/Washington/75/2009	ggtgatgccccattccttgatcggctccgccgagatca aaagtccttaaaaggaagaggcaacaccc	-	*
CY083100	Human H1N1pdm2009 IAVs	Human	H1N1	pdm	2009	Peru	A/Lima/WRAIR0519F/2009	ggtgatgccccattccttgatcggctccgccgagatca aaagtccttaaaaggaagaggcaacaccc	-	*
CY052843	Human H1N1pdm2009 IAVs	Human	H1N1	pdm	2009	USA	A/Texas/45132202/2009	ggtgatgccccattccttgatcggctccgccgagatca aaagtccttaaaaggaagaggcaacaccc	-	*

CY122907	Human H1N1pdm2009 IAVs	Human	H1N1	pdm	2009	Singapore	A/Singapore/GP2649/2009	ggtgatgccccattccttgatcggctccgccgagatcaaaagtccttaaaaggaagaggcaacaccc	-	*
CY123222	Human H1N1pdm2009 IAVs	Human	H1N1		2009	Singapore	A/Singapore/GP4012/2009	ggtgatgccccattccttgatcggctccgccgagatcaaaagtccttaaaaggaagaggcaacaccc	-	*
CY123679	Human H1N1pdm2009 IAVs	Human	H1N1	pdm	2009	Singapore	A/Singapore/ON1955/2009	ggtgatgccccattccttgatcggctccgccgagatcaaaagtccttaaaaggaagaggcaacaccc	-	*
CY123442	Human H1N1pdm2009 IAVs	Human	H1N1		2009	Singapore	A/Singapore/ON1107/2009	ggtgatgccccattccttgatcggctccgccgagatcaaaagtccttaaaaggaagaggcaacaccc	-	*
HQ165795	Human H1N1pdm2009 IAVs	Human	H1N1	pdm	2009	Kenya	A/Kapenguria/181/2009	ggtgatgccccattccttgatcggctccgccgagatcaaaagtccttaaaaggaagaggcaacaccc	-	*
CY044100	Human H1N1pdm2009 IAVs	Human	H1N1	pdm	2009	USA	A/New_York/3895/2009	ggtgatgccccattccttgatcggctccgccgagatcaaaagtccttaaaaggaagaggcaacaccc	-	*
CY073985	Human H1N1pdm2009 IAVs	Human	H1N1	pdm	2009	Argentina	A/Argentina/HNRG105/2009	ggtgatgccccattccttgatcggctccgccgagatcaaaagtccttaaaaggaagaggcaacaccc	-	*
CY064536	Human H1N1pdm2009 IAVs	Human	H1N1	pdm	2009	USA	A/Boston/120/2009	ggtgatgccccattccttgatcggctccgccgagatcaaaagtccttaaaaggaagaggcaacaccc	-	*
CY064544	Human H1N1pdm2009 IAVs	Human	H1N1	pdm	2009	USA	A/Boston/121/2009	ggtgatgccccattccttgatcggctccgccgagatcaaaagtccttaaaaggaagaggcaacaccc	-	*
CY111759	Human H1N1pdm2009 IAVs	Human	H1N1	pdm	2009	Hong_Kong	A/Hong_Kong/H090_704_V10/2009	ggtgatgccccattccttgatcggctccgccgagatcaaaagtccttaaaaggaagaggcaacaccc	-	*
CY120209	Human H1N1pdm2009 IAVs	Human	H1N1	pdm	2009	Hong_Kong	A/Hong_Kong/H090_704_V10/2009	ggtgatgccccattccttgatcggctccgccgagatcaaaagtccttaaaaggaagaggcaacaccc	-	*
GU371262	Human H1N1pdm2009 IAVs	Human	H1N1		2009	Russia	A/Orenburg/IIV2974/2009	ggtgatgccccattccttgatcggctccgccgagatcaaaagtccttaaaaggaagaggcaacaccc	-	*
CY061047	Human H1N1pdm2009 IAVs	Human	H1N1	pdm	2009	USA	A/Texas/JMS391/2009	ggtgatgccccattccttgatcggctccgccgagatcaaaagtccttaaaaggaagaggcaacaccc	-	*
CY040713	Human H1N1pdm2009 IAVs	Human	H1N1	pdm	2009	USA	A/New_York/3237/2009	ggtgatgccccattccttgatcggctccgccgagatcaaaagtccttaaaaggaagaggcaacaccc	-	*
KJ690404	Human H1N1pdm2009 IAVs	Human	H1N1	pdm	2009	Uganda	A/Uganda/MUWRP_093/2009	ggtgatgccccattccttgatcggctccgccgagatcaaaagtccttaaaaggaagaggcaacaccc	-	*
KJ690546	Human H1N1pdm2009 IAVs	Human	H1N1	pdm	2009	Uganda	A/Uganda/MUWRP_102/2009	ggtgatgccccattccttgatcggctccgccgagatcaaaagtccttaaaaggaagaggcaacaccc	-	*
CY058424	Human H1N1pdm2009 IAVs	Human	H1N1	pdm	2009	USA	A/Wisconsin/629_D01904/2009	ggtgatgccccattccttgatcggctccgccgagatcaaaagtccttaaaaggaagaggcaacaccc	-	*
CY071542	Human H1N1pdm2009 IAVs	Human	H1N1		2009	USA	A/California/WR1321P/2009	ggtgatgccccattccttgatcggctccgccgagatcaaaagtccttaaaaggaagaggcaacaccc	-	*
KC780181	Human H1N1pdm2009 IAVs	Human	H1N1	pdm	2009	USA	A/Montana/22/2009	ggtgatgccccattccttgatcggctccgccgagatcaaaagtccttaaaaggaagaggcaacaccc	-	*
CY046959	Human H1N1pdm2009 IAVs	Human	H1N1	pdm	2009	USA	A/New_York/3654/2009	ggtgatgccccattccttgatcggctccgccgagatcaaaagtccttaaaaggaagaggcaacaccc	-	*
CY052715	Human H1N1pdm2009 IAVs	Human	H1N1	pdm	2009	USA	A/Texas/45140902/2009	ggtgatgccccattccttgatcggctccgccgagatcaaaagtccttaaaaggaagaggcaacaccc	-	*
CY060919	Human H1N1pdm2009 IAVs	Human	H1N1	pdm	2009	USA	A/Texas/JMS370/2009	ggtgatgccccattccttgatcggctccgccgagatcaaaagtccttaaaaggaagaggcaacaccc	-	*
CY061063	Human H1N1pdm2009 IAVs	Human	H1N1	pdm	2009	USA	A/Texas/JMS393/2009	ggtgatgccccattccttgatcggctccgccgagatcaaaagtccttaaaaggaagaggcaacaccc	-	*
CY044993	Human H1N1pdm2009 IAVs	Human	H1N1	pdm	2009	USA	A/New_York/3751/2009	ggtgatgccccattccttgatcggctccgccgagatcaaaagtccttaaaaggaagaggcaacaccc	-	*
GQ214142	Human H1N1pdm2009 IAVs	Human	H1N1		2009	France	A/Paris/2590/2009	ggtgatgccccattccttgatcggctccgccgagatcaaaagtccttaaaaggaagaggcaacaccc	-	*

KC781011	Human H1N1pdm2009 IAVs	Human	H1N1	pdm	2009	USA	A/Arizona/20/2009	ggtgatgccccattccttgatcggctccgccgagatca aaagtccttaaaaggaagaggcaacaccc	-	*
CY065578	Human H1N1pdm2009 IAVs	Human	H1N1	pdm	2009	United_Kingdom	A/England/385/2009	ggtgatgccccattccttgatcggctccgccgagatca aaagtccttaaaaggaagaggcaacaccc	-	*
JX625540	Human H1N1pdm2009 IAVs	Human	H1N1	pdm	2009	United_Kingdom	A/Northern_Ireland/94480404/2009	ggtgatgccccattccttgatcggctccgccgagatca aaagtccttaaaaggaagaggcaacaccc	-	*
KC261335	Human H1N1pdm2009 IAVs	Human	H1N1	pdm	2009	Mexico	A/Mexico_City/INERPHAC2/2009	ggtgatgccccattccttgatcggctccgccgagatca aaagtccttaaaaggaagaggcaacaccc	-	*
CY095916	Human H1N1pdm2009 IAVs	Human	H1N1	pdm	2009	China	A/Zhejiang/80/2009	ggtgatgccccattccttgatcggctccgccgagatca aaagtccttaaaaggaagaggcaacaccc	-	*
CY095924	Human H1N1pdm2009 IAVs	Human	H1N1	pdm	2009	China	A/Zhejiang/81/2009	ggtgatgccccattccttgatcggctccgccgagatca aaagtccttaaaaggaagaggcaacaccc	-	*
CY096020	Human H1N1pdm2009 IAVs	Human	H1N1	pdm	2009	China	A/Zhejiang/95/2009	ggtgatgccccattccttgatcggctccgccgagatca aaagtccttaaaaggaagaggcaacaccc	-	*
CY096034	Human H1N1pdm2009 IAVs	Human	H1N1	pdm	2009	China	A/Zhejiang/97/2009	ggtgatgccccattccttgatcggctccgccgagatca aaagtccttaaaaggaagaggcaacaccc	-	*
CY075496	Human H1N1pdm2009 IAVs	Human	H1N1	pdm	2009	USA	A/Boston/584/2009	ggtgatgccccattccttgatcggctccgccgagatca aaagtccttaaaaggaagaggcaacaccc	-	*
CY066227	Human H1N1pdm2009 IAVs	Human	H1N1	pdm	2009	USA	A/California/VRDL106/2009	ggtgatgccccattccttgatcggctccgccgagatca aaagtccttaaaaggaagaggcaacaccc	-	*
CY062823	Human H1N1pdm2009 IAVs	Human	H1N1	pdm	2009	Denmark	A/Copenhagen/INS144/2009	ggtgatgccccattccttgatcggctccgccgagatca aaagtccttaaaaggaagaggcaacaccc	-	*
CY056967	Human H1N1pdm2009 IAVs	Human	H1N1	pdm	2009	Denmark	A/Copenhagen/INS95/2009	ggtgatgccccattccttgatcggctccgccgagatca aaagtccttaaaaggaagaggcaacaccc	-	*
CY056184	Human H1N1pdm2009 IAVs	Human	H1N1	pdm	2009	USA	A/District_of_Columbia/INS43/2009	ggtgatgccccattccttgatcggctccgccgagatca aaagtccttaaaaggaagaggcaacaccc	-	*
CY066691	Human H1N1pdm2009 IAVs	Human	H1N1	pdm	2009	Denmark	A/Hvidovre/INS289/2009	ggtgatgccccattccttgatcggctccgccgagatca aaagtccttaaaaggaagaggcaacaccc	-	*
CY056519	Human H1N1pdm2009 IAVs	Human	H1N1	pdm	2009	USA	A/New_York/5086/2009	ggtgatgccccattccttgatcggctccgccgagatca aaagtccttaaaaggaagaggcaacaccc	-	*
CY056607	Human H1N1pdm2009 IAVs	Human	H1N1	pdm	2009	USA	A/New_York/5931/2009	ggtgatgccccattccttgatcggctccgccgagatca aaagtccttaaaaggaagaggcaacaccc	-	*
CY057658	Human H1N1pdm2009 IAVs	Human	H1N1	pdm	2009	USA	A/Wisconsin/629_D00544/2009	ggtgatgccccattccttgatcggctccgccgagatca aaagtccttaaaaggaagaggcaacaccc	-	*
CY057546	Human H1N1pdm2009 IAVs	Human	H1N1	pdm	2009	USA	A/Wisconsin/629_D00853/2009	ggtgatgccccattccttgatcggctccgccgagatca aaagtccttaaaaggaagaggcaacaccc	-	*
CY057850	Human H1N1pdm2009 IAVs	Human	H1N1	pdm	2009	USA	A/Wisconsin/629_D02060/2009	ggtgatgccccattccttgatcggctccgccgagatca aaagtccttaaaaggaagaggcaacaccc	-	*
CY057554	Human H1N1pdm2009 IAVs	Human	H1N1	pdm	2009	USA	A/Wisconsin/629_D02337/2009	ggtgatgccccattccttgatcggctccgccgagatca aaagtccttaaaaggaagaggcaacaccc	-	*
CY058328	Human H1N1pdm2009 IAVs	Human	H1N1	pdm	2009	USA	A/Wisconsin/629_S1416/2009	ggtgatgccccattccttgatcggctccgccgagatca aaagtccttaaaaggaagaggcaacaccc	-	*
CY057730	Human H1N1pdm2009 IAVs	Human	H1N1	pdm	2009	USA	A/Wisconsin/629_D01915/2009	ggtgatgccccattccttgatcggctccgccgagatca aaagtccttaaaaggaagaggcaacaccc	-	*
CY063263	Human H1N1pdm2009 IAVs	Human	H1N1	pdm	2009	USA	A/Wisconsin/629_D00740/2009	ggtgatgccccattccttgatcggctccgccgagatca aaagtccttaaaaggaagaggcaacaccc	-	*
CY058344	Human H1N1pdm2009 IAVs	Human	H1N1	pdm	2009	USA	A/Wisconsin/629_D00503/2009	ggtgatgccccattccttgatcggctccgccgagatca aaagtccttaaaaggaagaggcaacaccc	-	*
CY063111	Human H1N1pdm2009 IAVs	Human	H1N1	pdm	2009	USA	A/California/VRDL92/2009	ggtgatgccccattccttgatcggctccgccgagatca aaagtccttaaaaggaagaggcaacaccc	-	*

CY063143	Human H1N1pdm2009 IAVs	Human	H1N1	pdm	2009	USA	A/California/VRDL98/2009	ggtgatgccccattccttgatcggctccgccgagatca<br>aaagtccttaaaaggaagaggcaacaccc	-	*
CY083451	Human H1N1pdm2009 IAVs	Human	H1N1	pdm	2009	Mexico	A/Mexico_City/WRAIR1679N/2009	ggtgatgccccattccttgatcggctccgccgagatca<br>aaagtccttaaaaggaagaggcaacaccc	-	*
CY058688	Human H1N1pdm2009 IAVs	Human	H1N1	pdm	2009	USA	A/Texas/46182018/2009	ggtgatgccccattccttgatcggctccgccgagatca<br>aaagtccttaaaaggaagaggcaacaccc	-	*
CY061558	Human H1N1pdm2009 IAVs	Human	H1N1	pdm	2009	USA	A/Texas/JMS366/2009	ggtgatgccccattccttgatcggctccgccgagatca<br>aaagtccttaaaaggaagaggcaacaccc	-	*
CY063271	Human H1N1pdm2009 IAVs	Human	H1N1	pdm	2009	USA	A/Wisconsin/629_D02082/2009	ggtgatgccccattccttgatcggctccgccgagatca<br>aaagtccttaaaaggaagaggcaacaccc	-	*
CY123636	Human H1N1pdm2009 IAVs	Human	H1N1	pdm	2009	Singapore	A/Singapore/ON1921/2009	ggtgatgccccattccttgatcggctccgccgagatca<br>aaagtccttaaaaggaagaggcaacaccc	-	*
CY063279	Human H1N1pdm2009 IAVs	Human	H1N1	pdm	2009	USA	A/Wisconsin/629_D00888/2009	ggtgatgccccattccttgatcggctccgccgagatca<br>aaagtccttaaaaggaagaggcaacaccc	-	*
CY057898	Human H1N1pdm2009 IAVs	Human	H1N1	pdm	2009	USA	A/Wisconsin/629_D00908/2009	ggtgatgccccattccttgatcggctccgccgagatca<br>aaagtccttaaaaggaagaggcaacaccc	-	*
CY058448	Human H1N1pdm2009 IAVs	Human	H1N1	pdm	2009	USA	A/Wisconsin/629_D02073/2009	ggtgatgccccattccttgatcggctccgccgagatca<br>aaagtccttaaaaggaagaggcaacaccc	-	*
CY083658	Human H1N1pdm2009 IAVs	Human	H1N1	pdm	2009	USA	A/San_Diego/INS36/2009	ggtgatgccccattccttgatcggctccgccgagatca<br>aaagtccttaaaaggaagaggcaacaccc	-	*
CY071694	Human H1N1pdm2009 IAVs	Human	H1N1	pdm	2009	Mexico	A/Mexico_City/WR1683T/2009	ggtgatgccccattccttgatcggctccgccgagatca<br>aaagtccttaaaaggaagaggcaacaccc	-	*
CY071630	Human H1N1pdm2009 IAVs	Human	H1N1	pdm	2009	Taiwan	A/Taipei/WR1472T/2009	ggtgatgccccattccttgatcggctccgccgagatca<br>aaagtccttaaaaggaagaggcaacaccc	-	*
JF713997	Human H1N1pdm2009 IAVs	Swine	H1N1		2009	Canada	A/SW/SK/59_6/2009	ggtgatgccccattccttgatcggctccgccgagatca<br>aaagtccttaaaaggaagaggcaacaccc	-	*
CY066411	Human H1N1pdm2009 IAVs	Human	H1N1	pdm	2009	USA	A/California/VRDL129/2009	ggtgatgccccattccttgatcggctccgccgagatca<br>aaagtccttaaaaggaagaggcaacaccc	-	*
CY060578	Human H1N1pdm2009 IAVs	Human	H1N1	pdm	2009	Canada	A/Ontario/29801/2009	ggtgatgccccattccttgatcggctccgccgagatca<br>aaagtccttaaaaggaagaggcaacaccc	-	*
CY128335	Human H1N1pdm2009 IAVs	Human	H1N1	pdm	2009	Viet_Nam	A/Viet_Nam/13032096/2009	ggtgatgccccattccttgatcggctccgccgagatca<br>aaagtccttaaaaggaagaggcaacaccc	-	*
CY128343	Human H1N1pdm2009 IAVs	Human	H1N1	pdm	2009	Viet_Nam	A/Viet_Nam/13032097/2009	ggtgatgccccattccttgatcggctccgccgagatca<br>aaagtccttaaaaggaagaggcaacaccc	-	*
KC780667	Human H1N1pdm2009 IAVs	Human	H1N1	pdm	2009	USA	A/Nebraska/23/2009	ggtgatgccccattccttgatcggctccgccgagatca<br>aaagtccttaaaaggaagaggcaacaccc	-	*
CY066339	Human H1N1pdm2009 IAVs	Human	H1N1	pdm	2009	USA	A/California/VRDL120/2009	ggtgatgccccattccttgatcggctccgccgagatca<br>aaagtccttaaaaggaagaggcaacaccc	-	*
CY066787	Human H1N1pdm2009 IAVs	Human	H1N1	pdm	2009	USA	A/San_Diego/INS214/2009	ggtgatgccccattccttgatcggctccgccgagatca<br>aaagtccttaaaaggaagaggcaacaccc	-	*
JX625502	Human H1N1pdm2009 IAVs	Human	H1N1		2009	United_Kingdom	A/England/93760756/2009	ggtgatgccccattccttgatcggctccgccgagatca<br>aaagtccttaaaaggaagaggcaacaccc	-	*
HM567868	Human H1N1pdm2009 IAVs	Human	H1N1	pdm	2009	United_Kingdom	A/England/416/2009	ggtgatgccccattccttgatcggctccgccgagatca<br>aaagtccttaaaaggaagaggcaacaccc	-	*
HQ165790	Human H1N1pdm2009 IAVs	Human	H1N1	pdm	2009	Kenya	A/Nairobi/59/2009	ggtgatgccccattccttgatcggctccgccgagatca<br>aaagtccttaaaaggaagaggcaacaccc	-	*
CY060911	Human H1N1pdm2009 IAVs	Human	H1N1	pdm	2009	USA	A/Texas/JMS369/2009	ggtgatgccccattccttgatcggctccgccgagatca<br>aaagtccttaaaaggaagaggcaacaccc	-	*
CY058720	Human H1N1pdm2009 IAVs	Human	H1N1	pdm	2009	USA	A/Texas/JMS375/2009	ggtgatgccccattccttgatcggctccgccgagatca<br>aaagtccttaaaaggaagaggcaacaccc	-	*

CY058736	Human H1N1pdm2009 IAVs	Human	H1N1	pdm	2009	USA	A/Texas/JMS377/2009	ggtgatgccccattccttgatcggctccgccgagatca aaagtccttaaaaggaagaggcaacaccc	-	*
CY060991	Human H1N1pdm2009 IAVs	Human	H1N1	pdm	2009	USA	A/Texas/JMS384/2009	ggtgatgccccattccttgatcggctccgccgagatca aaagtccttaaaaggaagaggcaacaccc	-	*
CY057634	Human H1N1pdm2009 IAVs	Human	H1N1	pdm	2009	USA	A/Wisconsin/629_S1348/2009	ggtgatgccccattccttgatcggctccgccgagatca aaagtccttaaaaggaagaggcaacaccc	-	*
CY067083	Human H1N1pdm2009 IAVs	Human	H1N1	pdm	2009	Greece	A/Athens/INS260/2009	ggtgatgccccattccttgatcggctccgccgagatca aaagtccttaaaaggaagaggcaacaccc	-	*
CY067123	Human H1N1pdm2009 IAVs	Human	H1N1	pdm	2009	Greece	A/Athens/INS266/2009	ggtgatgccccattccttgatcggctccgccgagatca aaagtccttaaaaggaagaggcaacaccc	-	*
CY072370	Human H1N1pdm2009 IAVs	Human	H1N1	pdm	2009	Greece	A/Athens/INS327/2009	ggtgatgccccattccttgatcggctccgccgagatca aaagtccttaaaaggaagaggcaacaccc	-	*
CY073010	Human H1N1pdm2009 IAVs	Human	H1N1	pdm	2009	Greece	A/Athens/INS330/2009	ggtgatgccccattccttgatcggctccgccgagatca aaagtccttaaaaggaagaggcaacaccc	-	*
CY072498	Human H1N1pdm2009 IAVs	Human	H1N1	pdm	2009	Greece	A/Athens/INS346/2009	ggtgatgccccattccttgatcggctccgccgagatca aaagtccttaaaaggaagaggcaacaccc	-	*
CY069174	Human H1N1pdm2009 IAVs	Human	H1N1	pdm	2009	Greece	A/Athens/INS354/2009	ggtgatgccccattccttgatcggctccgccgagatca aaagtccttaaaaggaagaggcaacaccc	-	*
HM780486	Human H1N1pdm2009 IAVs	Human	H1N1	pdm	2009	China	A/Guangdong/0862/2009	ggtgatgccccattccttgatcggctccgccgagatca aaagtccttaaaaggaagaggcaacaccc	-	*
CY100476	Human H1N1pdm2009 IAVs	Human	H1N1	pdm	2009	Mexico	A/Mexico_City/INER12/2009	ggtgatgccccattccttgatcggctccgccgagatca aaagtccttaaaaggaagaggcaacaccc	-	*
HQ840300	Human H1N1pdm2009 IAVs	Swine	H1N1		2009	USA	A/swine/Minnesota/130A/2009	ggtgatgccccattccttgatcggctccgccgagatca aaagtccttaaaaggaagaggcaacaccc	-	*
HM855245	Human H1N1pdm2009 IAVs	Human	H1N1	pdm	2009	Kenya	A/Trans_nzoia/168/2009	ggtgatgccccattccttgatcggctccgccgagatca aaagtccttaaaaggaagaggcaacaccc	-	*
CY071251	Human H1N1pdm2009 IAVs	Human	H1N1	pdm	2009	Spain	A/Madrid/INS407/2009	ggtgatgccccattccttgatcggctccgccgagatca aaagtccttaaaaggaagaggcaacaccc	-	*
CY044913	Human H1N1pdm2009 IAVs	Human	H1N1	pdm	2009	USA	A/New_York/3612/2009	ggtgatgccccattccttgatcggctccgccgagatca aaagtccttaaaaggaagaggcaacaccc	-	*
CY057674	Human H1N1pdm2009 IAVs	Human	H1N1	pdm	2009	USA	A/Wisconsin/629_D00132/2009	ggtgatgccccattccttgatcggctccgccgagatca aaagtccttaaaaggaagaggcaacaccc	-	*
CY058672	Human H1N1pdm2009 IAVs	Human	H1N1		2009	USA	A/Wisconsin/629_D00193/2009	ggtgatgccccattccttgatcggctccgccgagatca aaagtccttaaaaggaagaggcaacaccc	-	*
CY057602	Human H1N1pdm2009 IAVs	Human	H1N1		2009	USA	A/Wisconsin/629_D01080/2009	ggtgatgccccattccttgatcggctccgccgagatca aaagtccttaaaaggaagaggcaacaccc	-	*
CY057418	Human H1N1pdm2009 IAVs	Human	H1N1	pdm	2009	USA	A/Wisconsin/629_D01802/2009	ggtgatgccccattccttgatcggctccgccgagatca aaagtccttaaaaggaagaggcaacaccc	-	*
CY057666	Human H1N1pdm2009 IAVs	Human	H1N1	pdm	2009	USA	A/Wisconsin/629_D00099/2009	ggtgatgccccattccttgatcggctccgccgagatca aaagtccttaaaaggaagaggcaacaccc	-	*
GQ168624	Human H1N1pdm2009 IAVs	Human	H1N1	pdm	2009	USA	A/Alabama/02/2009	ggtgatgccccattccttgatcggctccgccgagatca aaagtccttaaaaggaagaggcaacaccc	-	*
GQ117065	Human H1N1pdm2009 IAVs	Human	H1N1	pdm	2009	USA	A/Arizona/01/2009	ggtgatgccccattccttgatcggctccgccgagatca aaagtccttaaaaggaagaggcaacaccc	-	*
GQ457489	Human H1N1pdm2009 IAVs	Human	H1N1	pdm	2009	USA	A/Arizona/02/2009	ggtgatgccccattccttgatcggctccgccgagatca aaagtccttaaaaggaagaggcaacaccc	-	*
GQ323516	Human H1N1pdm2009 IAVs	Human	H1N1	pdm	2009	USA	A/Arizona/09/2009	ggtgatgccccattccttgatcggctccgccgagatca aaagtccttaaaaggaagaggcaacaccc	-	*
GQ323487	Human H1N1pdm2009 IAVs	Human	H1N1	pdm	2009	USA	A/Arizona/09/2009	ggtgatgccccattccttgatcggctccgccgagatca aaagtccttaaaaggaagaggcaacaccc	-	*

GQ323485	Human H1N1pdm2009 IAVs	Human	H1N1	pdm	2009	USA	A/Arizona/09/2009	ggtgatgccccattccttgatcggctccgccgagatca aaagtccttaaaaggaagaggcaacaccc	-	*
GQ323491	Human H1N1pdm2009 IAVs	Human	H1N1	pdm	2009	USA	A/Arkansas/03/2009	ggtgatgccccattccttgatcggctccgccgagatca aaagtccttaaaaggaagaggcaacaccc	-	*
GQ183621	Human H1N1pdm2009 IAVs	Human	H1N1	pdm	2009	China	A/Beijing/01/2009	ggtgatgccccattccttgatcggctccgccgagatca aaagtccttaaaaggaagaggcaacaccc	-	*
GQ225377	Human H1N1pdm2009 IAVs	Human	H1N1	pdm	2009	China	A/Beijing/01/2009	ggtgatgccccattccttgatcggctccgccgagatca aaagtccttaaaaggaagaggcaacaccc	-	*
GQ183629	Human H1N1pdm2009 IAVs	Human	H1N1	pdm	2009	China	A/Beijing/02/2009	ggtgatgccccattccttgatcggctccgccgagatca aaagtccttaaaaggaagaggcaacaccc	-	*
HQ698631	Human H1N1pdm2009 IAVs	Human	H1N1	pdm	2009	China	A/Beijing/16/2009	ggtgatgccccattccttgatcggctccgccgagatca aaagtccttaaaaggaagaggcaacaccc	-	*
GQ225385	Human H1N1pdm2009 IAVs	Human	H1N1	pdm	2009	China	A/Beijing/3/2009	ggtgatgccccattccttgatcggctccgccgagatca aaagtccttaaaaggaagaggcaacaccc	-	*
GQ232097	Human H1N1pdm2009 IAVs	Human	H1N1	pdm	2009	China	A/Beijing/4/2009	ggtgatgccccattccttgatcggctccgccgagatca aaagtccttaaaaggaagaggcaacaccc	-	*
GQ223411	Human H1N1pdm2009 IAVs	Human	H1N1	pdm	2009	China	A/Beijing/501/2009	ggtgatgccccattccttgatcggctccgccgagatca aaagtccttaaaaggaagaggcaacaccc	-	*
GQ290110	Human H1N1pdm2009 IAVs	Human	H1N1	pdm	2009	China	A/Beijing/502/2009	ggtgatgccccattccttgatcggctccgccgagatca aaagtccttaaaaggaagaggcaacaccc	-	*
KJ023093	Human H1N1pdm2009 IAVs	Human	H1N1	pdm	2009	India	A/Bihar/003/2009	ggtgatgccccattccttgatcggctccgccgagatca aaagtccttaaaaggaagaggcaacaccc	-	*
FJ969514	Human H1N1pdm2009 IAVs	Human	H1N1	pdm	2009	USA	A/California/04/2009	ggtgatgccccattccttgatcggctccgccgagatca aaagtccttaaaaggaagaggcaacaccc	-	*
FJ966086	Human H1N1pdm2009 IAVs	Human	H1N1	pdm	2009	USA	A/California/04/2009	ggtgatgccccattccttgatcggctccgccgagatca aaagtccttaaaaggaagaggcaacaccc	-	*
FJ969538	Human H1N1pdm2009 IAVs	Human	H1N1	pdm	2009	USA	A/California/07/2009	ggtgatgccccattccttgatcggctccgccgagatca aaagtccttaaaaggaagaggcaacaccc	-	*
FJ969528	Human H1N1pdm2009 IAVs	Human	H1N1	pdm	2009	USA	A/California/07/2009	ggtgatgccccattccttgatcggctccgccgagatca aaagtccttaaaaggaagaggcaacaccc	-	*
FJ969533	Human H1N1pdm2009 IAVs	Human	H1N1	pdm	2009	USA	A/California/08/2009	ggtgatgccccattccttgatcggctccgccgagatca aaagtccttaaaaggaagaggcaacaccc	-	*
FJ969519	Human H1N1pdm2009 IAVs	Human	H1N1	pdm	2009	USA	A/California/08/2009	ggtgatgccccattccttgatcggctccgccgagatca aaagtccttaaaaggaagaggcaacaccc	-	*
CY054831	Human H1N1pdm2009 IAVs	Human	H1N1	pdm	2009	USA	A/California/VRDL29/2009	ggtgatgccccattccttgatcggctccgccgagatca aaagtccttaaaaggaagaggcaacaccc	-	*
CY054727	Human H1N1pdm2009 IAVs	Human	H1N1	pdm	2009	USA	A/California/VRDL6/2009	ggtgatgccccattccttgatcggctccgccgagatca aaagtccttaaaaggaagaggcaacaccc	-	*
FJ998219	Human H1N1pdm2009 IAVs	Human	H1N1	pdm	2009	Canada	A/Canada_NS/RV1535/2009	ggtgatgccccattccttgatcggctccgccgagatca aaagtccttaaaaggaagaggcaacaccc	-	*
CY065774	Human H1N1pdm2009 IAVs	Human	H1N1	pdm	2009	Canada	A/Canada/GFA0402/2009	ggtgatgccccattccttgatcggctccgccgagatca aaagtccttaaaaggaagaggcaacaccc	-	*
CY069769	Human H1N1pdm2009 IAVs	Human	H1N1	pdm	2009	United_Kingdom	A/England/196/2009	ggtgatgccccattccttgatcggctccgccgagatca aaagtccttaaaaggaagaggcaacaccc	-	*
CY065202	Human H1N1pdm2009 IAVs	Human	H1N1		2009	United_Kingdom	A/England/197/2009	ggtgatgccccattccttgatcggctccgccgagatca aaagtccttaaaaggaagaggcaacaccc	-	*
CY065338	Human H1N1pdm2009 IAVs	Human	H1N1	pdm	2009	United_Kingdom	A/England/201/2009	ggtgatgccccattccttgatcggctccgccgagatca aaagtccttaaaaggaagaggcaacaccc	-	*
CY065210	Human H1N1pdm2009 IAVs	Human	H1N1	pdm	2009	United_Kingdom	A/England/204/2009	ggtgatgccccattccttgatcggctccgccgagatca aaagtccttaaaaggaagaggcaacaccc	-	*

CY065218	Human H1N1pdm2009 IAVs	Human	H1N1	pdm	2009	United_Kingdom	A/England/205/2009	ggtgatgccccattccttgatcggctccgccgagatca<br>aaagtccttaaaaggaagaggcaacaccc	-	*
CY069777	Human H1N1pdm2009 IAVs	Human	H1N1	pdm	2009	United_Kingdom	A/England/209/2009	ggtgatgccccattccttgatcggctccgccgagatca<br>aaagtccttaaaaggaagaggcaacaccc	-	*
CY065258	Human H1N1pdm2009 IAVs	Human	H1N1	pdm	2009	United_Kingdom	A/England/211/2009	ggtgatgccccattccttgatcggctccgccgagatca<br>aaagtccttaaaaggaagaggcaacaccc	-	*
CY065282	Human H1N1pdm2009 IAVs	Human	H1N1		2009	United_Kingdom	A/England/215/2009	ggtgatgccccattccttgatcggctccgccgagatca<br>aaagtccttaaaaggaagaggcaacaccc	-	*
CY065290	Human H1N1pdm2009 IAVs	Human	H1N1	pdm	2009	United_Kingdom	A/England/216/2009	ggtgatgccccattccttgatcggctccgccgagatca<br>aaagtccttaaaaggaagaggcaacaccc	-	*
CY065306	Human H1N1pdm2009 IAVs	Human	H1N1	pdm	2009	United_Kingdom	A/England/218/2009	ggtgatgccccattccttgatcggctccgccgagatca<br>aaagtccttaaaaggaagaggcaacaccc	-	*
CY065322	Human H1N1pdm2009 IAVs	Human	H1N1		2009	United_Kingdom	A/England/219/2009	ggtgatgccccattccttgatcggctccgccgagatca<br>aaagtccttaaaaggaagaggcaacaccc	-	*
CY065330	Human H1N1pdm2009 IAVs	Human	H1N1	pdm	2009	United_Kingdom	A/England/220/2009	ggtgatgccccattccttgatcggctccgccgagatca<br>aaagtccttaaaaggaagaggcaacaccc	-	*
CY069785	Human H1N1pdm2009 IAVs	Human	H1N1	pdm	2009	United_Kingdom	A/England/221/2009	ggtgatgccccattccttgatcggctccgccgagatca<br>aaagtccttaaaaggaagaggcaacaccc	-	*
CY069793	Human H1N1pdm2009 IAVs	Human	H1N1	pdm	2009	United_Kingdom	A/England/222/2009	ggtgatgccccattccttgatcggctccgccgagatca<br>aaagtccttaaaaggaagaggcaacaccc	-	*
CY065314	Human H1N1pdm2009 IAVs	Human	H1N1	pdm	2009	United_Kingdom	A/England/246/2009	ggtgatgccccattccttgatcggctccgccgagatca<br>aaagtccttaaaaggaagaggcaacaccc	-	*
CY065354	Human H1N1pdm2009 IAVs	Human	H1N1	pdm	2009	United_Kingdom	A/England/247/2009	ggtgatgccccattccttgatcggctccgccgagatca<br>aaagtccttaaaaggaagaggcaacaccc	-	*
KJ867566	Human H1N1pdm2009 IAVs	Human	H1N1	pdm	2009	Belgium	A/Belgium/145_MA/2009	ggtgatgccccattccttgatcggctccgccgagatca<br>aaagtccttaaaaggaagaggcaacaccc	-	*
CY069817	Human H1N1pdm2009 IAVs	Human	H1N1	pdm	2009	United_Kingdom	A/England/249/2009	ggtgatgccccattccttgatcggctccgccgagatca<br>aaagtccttaaaaggaagaggcaacaccc	-	*
CY069825	Human H1N1pdm2009 IAVs	Human	H1N1	pdm	2009	United_Kingdom	A/England/250/2009	ggtgatgccccattccttgatcggctccgccgagatca<br>aaagtccttaaaaggaagaggcaacaccc	-	*
CY065394	Human H1N1pdm2009 IAVs	Human	H1N1	pdm	2009	United_Kingdom	A/England/252/2009	ggtgatgccccattccttgatcggctccgccgagatca<br>aaagtccttaaaaggaagaggcaacaccc	-	*
CY065410	Human H1N1pdm2009 IAVs	Human	H1N1	pdm	2009	United_Kingdom	A/England/253/2009	ggtgatgccccattccttgatcggctccgccgagatca<br>aaagtccttaaaaggaagaggcaacaccc	-	*
CY065418	Human H1N1pdm2009 IAVs	Human	H1N1	pdm	2009	United_Kingdom	A/England/254/2009	ggtgatgccccattccttgatcggctccgccgagatca<br>aaagtccttaaaaggaagaggcaacaccc	-	*
CY065442	Human H1N1pdm2009 IAVs	Human	H1N1		2009	United_Kingdom	A/England/257/2009	ggtgatgccccattccttgatcggctccgccgagatca<br>aaagtccttaaaaggaagaggcaacaccc	-	*
CY065522	Human H1N1pdm2009 IAVs	Human	H1N1		2009	United_Kingdom	A/England/266/2009	ggtgatgccccattccttgatcggctccgccgagatca<br>aaagtccttaaaaggaagaggcaacaccc	-	*
CY069897	Human H1N1pdm2009 IAVs	Human	H1N1	pdm	2009	United_Kingdom	A/England/391/2009	ggtgatgccccattccttgatcggctccgccgagatca<br>aaagtccttaaaaggaagaggcaacaccc	-	*
CY065666	Human H1N1pdm2009 IAVs	Human	H1N1	pdm	2009	United_Kingdom	A/England/414/2009	ggtgatgccccattccttgatcggctccgccgagatca<br>aaagtccttaaaaggaagaggcaacaccc	-	*
CY065250	Human H1N1pdm2009 IAVs	Human	H1N1	pdm	2009	United_Kingdom	A/England/XFL00071/2009	ggtgatgccccattccttgatcggctccgccgagatca<br>aaagtccttaaaaggaagaggcaacaccc	-	*
CY065266	Human H1N1pdm2009 IAVs	Human	H1N1	pdm	2009	United_Kingdom	A/England/XFL00137/2009	ggtgatgccccattccttgatcggctccgccgagatca<br>aaagtccttaaaaggaagaggcaacaccc	-	*
CY065362	Human H1N1pdm2009 IAVs	Human	H1N1		2009	United_Kingdom	A/England/XFL00200/2009	ggtgatgccccattccttgatcggctccgccgagatca<br>aaagtccttaaaaggaagaggcaacaccc	-	*

CY069801	Human H1N1pdm2009 IAVs	Human	H1N1	pdm	2009	United_Kingdom	A/England/XFL00228/2009	ggtgatgccccattccttgatcggctccgccgagatca aaagtccttaaaaggaagaggcaacaccc	-	*
CY069809	Human H1N1pdm2009 IAVs	Human	H1N1	pdm	2009	United_Kingdom	A/England/XFL00230/2009	ggtgatgccccattccttgatcggctccgccgagatca aaagtccttaaaaggaagaggcaacaccc	-	*
CY065370	Human H1N1pdm2009 IAVs	Human	H1N1	pdm	2009	United_Kingdom	A/England/XFL00275/2009	ggtgatgccccattccttgatcggctccgccgagatca aaagtccttaaaaggaagaggcaacaccc	-	*
CY065378	Human H1N1pdm2009 IAVs	Human	H1N1	pdm	2009	United_Kingdom	A/England/XFL00300/2009	ggtgatgccccattccttgatcggctccgccgagatca aaagtccttaaaaggaagaggcaacaccc	-	*
CY069833	Human H1N1pdm2009 IAVs	Human	H1N1	pdm	2009	United_Kingdom	A/England/XFL00303/2009	ggtgatgccccattccttgatcggctccgccgagatca aaagtccttaaaaggaagaggcaacaccc	-	*
CY065386	Human H1N1pdm2009 IAVs	Human	H1N1	pdm	2009	United_Kingdom	A/England/XFL00304/2009	ggtgatgccccattccttgatcggctccgccgagatca aaagtccttaaaaggaagaggcaacaccc	-	*
CY065426	Human H1N1pdm2009 IAVs	Human	H1N1	pdm	2009	United_Kingdom	A/England/XFL00354/2009	ggtgatgccccattccttgatcggctccgccgagatca aaagtccttaaaaggaagaggcaacaccc	-	*
CY069841	Human H1N1pdm2009 IAVs	Human	H1N1	pdm	2009	United_Kingdom	A/England/XFL00364/2009	ggtgatgccccattccttgatcggctccgccgagatca aaagtccttaaaaggaagaggcaacaccc	-	*
CY069849	Human H1N1pdm2009 IAVs	Human	H1N1	pdm	2009	United_Kingdom	A/England/XFL00376/2009	ggtgatgccccattccttgatcggctccgccgagatca aaagtccttaaaaggaagaggcaacaccc	-	*
CY065466	Human H1N1pdm2009 IAVs	Human	H1N1	pdm	2009	United_Kingdom	A/England/XFL00419/2009	ggtgatgccccattccttgatcggctccgccgagatca aaagtccttaaaaggaagaggcaacaccc	-	*
CY069865	Human H1N1pdm2009 IAVs	Human	H1N1	pdm	2009	United_Kingdom	A/England/XFL00421/2009	ggtgatgccccattccttgatcggctccgccgagatca aaagtccttaaaaggaagaggcaacaccc	-	*
GQ457456	Human H1N1pdm2009 IAVs	Human	H1N1	pdm	2009	USA	A/Florida/04/2009	ggtgatgccccattccttgatcggctccgccgagatca aaagtccttaaaaggaagaggcaacaccc	-	*
GQ300857	Human H1N1pdm2009 IAVs	Human	H1N1	pdm	2009	Japan	A/Fukuoka_C/1/2009	ggtgatgccccattccttgatcggctccgccgagatca aaagtccttaaaaggaagaggcaacaccc	-	*
GQ334334	Human H1N1pdm2009 IAVs	Human	H1N1	pdm	2009	Japan	A/Fukuoka_C/2/2009	ggtgatgccccattccttgatcggctccgccgagatca aaagtccttaaaaggaagaggcaacaccc	-	*
GQ334342	Human H1N1pdm2009 IAVs	Human	H1N1		2009	Japan	A/Fukuoka_C/3/2009	ggtgatgccccattccttgatcggctccgccgagatca aaagtccttaaaaggaagaggcaacaccc	-	*
GU931807	Human H1N1pdm2009 IAVs	Human	H1N1	pdm	2009	Hong_Kong	A/Hong_Kong/415742/2009	ggtgatgccccattccttgatcggctccgccgagatca aaagtccttaaaaggaagaggcaacaccc	-	*
GU931808	Human H1N1pdm2009 IAVs	Human	H1N1	pdm	2009	Hong_Kong	A/Hong_Kong/415742M/2009	ggtgatgccccattccttgatcggctccgccgagatca aaagtccttaaaaggaagaggcaacaccc	-	*
HM100232	Human H1N1pdm2009 IAVs	Human	H1N1		2009	Hong_Kong	A/Hong_Kong/415742Md/2009	ggtgatgccccattccttgatcggctccgccgagatca aaagtccttaaaaggaagaggcaacaccc	-	*
GQ232027	Human H1N1pdm2009 IAVs	Human	H1N1	pdm	2009	USA	A/Illinois/01/2009	ggtgatgccccattccttgatcggctccgccgagatca aaagtccttaaaaggaagaggcaacaccc	-	*
GQ323527	Human H1N1pdm2009 IAVs	Human	H1N1	pdm	2009	USA	A/Illinois/04/2009	ggtgatgccccattccttgatcggctccgccgagatca aaagtccttaaaaggaagaggcaacaccc	-	*
KC781782	Human H1N1pdm2009 IAVs	Human	H1N1		2009	USA	A/California/07/2009	ggtgatgccccattccttgatcggctccgccgagatca aaagtccttaaaaggaagaggcaacaccc	-	*
GQ168871	Human H1N1pdm2009 IAVs	Human	H1N1	pdm	2009	USA	A/Indiana/09/2009	ggtgatgccccattccttgatcggctccgccgagatca aaagtccttaaaaggaagaggcaacaccc	-	*
GQ168640	Human H1N1pdm2009 IAVs	Human	H1N1	pdm	2009	USA	A/Iowa/04/2009	ggtgatgccccattccttgatcggctccgccgagatca aaagtccttaaaaggaagaggcaacaccc	-	*
GQ365440	Human H1N1pdm2009 IAVs	Human	H1N1	pdm	2009	Japan	A/Kagoshima/1/2009	ggtgatgccccattccttgatcggctccgccgagatca aaagtccttaaaaggaagaggcaacaccc	-	*
GU136007	Human H1N1pdm2009 IAVs	Human	H1N1	pdm	2009	Japan	A/Kanagawa/140/2009	ggtgatgccccattccttgatcggctccgccgagatca aaagtccttaaaaggaagaggcaacaccc	-	*

JX875038	Human H1N1pdm2009 IAVs	Human	H1N1	pdm	2009	USA	A/Kentucky/96/2009	ggtgatgccccattccttgatcggctccgccgagatcaaaagtccttaaaaggaagaggcaacaccc	-	*
CY083459	Human H1N1pdm2009 IAVs	Human	H1N1	pdm	2009	Peru	A/Lima/WRAIR1687P/2009	ggtgatgccccattccttgatcggctccgccgagatcaaaagtccttaaaaggaagaggcaacaccc	-	*
CY083587	Human H1N1pdm2009 IAVs	Human	H1N1	pdm	2009	Peru	A/Lima/WRAIR8689F/2009	ggtgatgccccattccttgatcggctccgccgagatcaaaagtccttaaaaggaagaggcaacaccc	-	*
GQ166749	Human H1N1pdm2009 IAVs	Human	H1N1	pdm	2009	Portugal	A/Lisboa/26/2009	ggtgatgccccattccttgatcggctccgccgagatcaaaagtccttaaaaggaagaggcaacaccc	-	*
CY058600	Human H1N1pdm2009 IAVs	Human	H1N1	pdm	2009	Nicaragua	A/Managua/3435.01/2009	ggtgatgccccattccttgatcggctccgccgagatcaaaagtccttaaaaggaagaggcaacaccc	-	*
CY058576	Human H1N1pdm2009 IAVs	Human	H1N1	pdm	2009	Nicaragua	A/Managua/4757.02/2009	ggtgatgccccattccttgatcggctccgccgagatcaaaagtccttaaaaggaagaggcaacaccc	-	*
CY058552	Human H1N1pdm2009 IAVs	Human	H1N1		2009	Nicaragua	A/Managua/4935.03/2009	ggtgatgccccattccttgatcggctccgccgagatcaaaagtccttaaaaggaagaggcaacaccc	-	*
CY058608	Human H1N1pdm2009 IAVs	Human	H1N1	pdm	2009	Nicaragua	A/Managua/5747.02/2009	ggtgatgccccattccttgatcggctccgccgagatcaaaagtccttaaaaggaagaggcaacaccc	-	*
CY058584	Human H1N1pdm2009 IAVs	Human	H1N1	pdm	2009	Nicaragua	A/Managua/989.01/2009	ggtgatgccccattccttgatcggctccgccgagatcaaaagtccttaaaaggaagaggcaacaccc	-	*
CY083507	Human H1N1pdm2009 IAVs	Human	H1N1	pdm	2009	Nicaragua	A/Managua/WRAIR1693P/2009	ggtgatgccccattccttgatcggctccgccgagatcaaaagtccttaaaaggaagaggcaacaccc	-	*
CY083611	Human H1N1pdm2009 IAVs	Human	H1N1	pdm	2009	Nicaragua	A/Managua/WRAIR8997F/2009	ggtgatgccccattccttgatcggctccgccgagatcaaaagtccttaaaaggaagaggcaacaccc	-	*
GQ160564	Human H1N1pdm2009 IAVs	Human	H1N1	pdm	2009	USA	A/Maryland/05/2009	ggtgatgccccattccttgatcggctccgccgagatcaaaagtccttaaaaggaagaggcaacaccc	-	*
KC781550	Human H1N1pdm2009 IAVs	Human	H1N1	pdm	2009	USA	A/Massachusetts/16/2009	ggtgatgccccattccttgatcggctccgccgagatcaaaagtccttaaaaggaagaggcaacaccc	-	*
CY092105	Human H1N1pdm2009 IAVs	Human	H1N1	pdm	2009	Mexico	A/Mexico_City/016/2009	ggtgatgccccattccttgatcggctccgccgagatcaaaagtccttaaaaggaagaggcaacaccc	-	*
GQ149687	Human H1N1pdm2009 IAVs	Human	H1N1	pdm	2009	Mexico	A/Mexico/4108/2009	ggtgatgccccattccttgatcggctccgccgagatcaaaagtccttaaaaggaagaggcaacaccc	-	*
GQ149658	Human H1N1pdm2009 IAVs	Human	H1N1	pdm	2009	Mexico	A/Mexico/4108/2009	ggtgatgccccattccttgatcggctccgccgagatcaaaagtccttaaaaggaagaggcaacaccc	-	*
GQ379814	Human H1N1pdm2009 IAVs	Human	H1N1	pdm	2009	Mexico	A/Mexico/4108/2009	ggtgatgccccattccttgatcggctccgccgagatcaaaagtccttaaaaggaagaggcaacaccc	-	*
GQ149694	Human H1N1pdm2009 IAVs	Human	H1N1	pdm	2009	Mexico	A/Mexico/4115/2009	ggtgatgccccattccttgatcggctccgccgagatcaaaagtccttaaaaggaagaggcaacaccc	-	*
GQ303351	Human H1N1pdm2009 IAVs	Human	H1N1	pdm	2009	Mexico	A/Mexico/4176/2009	ggtgatgccccattccttgatcggctccgccgagatcaaaagtccttaaaaggaagaggcaacaccc	-	*
GQ303336	Human H1N1pdm2009 IAVs	Human	H1N1	pdm	2009	Mexico	A/Mexico/4269/2009	ggtgatgccccattccttgatcggctccgccgagatcaaaagtccttaaaaggaagaggcaacaccc	-	*
GQ379818	Human H1N1pdm2009 IAVs	Human	H1N1	pdm	2009	Mexico	A/Mexico/4482/2009	ggtgatgccccattccttgatcggctccgccgagatcaaaagtccttaaaaggaagaggcaacaccc	-	*
GQ379816	Human H1N1pdm2009 IAVs	Human	H1N1	pdm	2009	Mexico	A/Mexico/4486/2009	ggtgatgccccattccttgatcggctccgccgagatcaaaagtccttaaaaggaagaggcaacaccc	-	*
GQ149621	Human H1N1pdm2009 IAVs	Human	H1N1	pdm	2009	Mexico	A/Mexico/4486/2009	ggtgatgccccattccttgatcggctccgccgagatcaaaagtccttaaaaggaagaggcaacaccc	-	*
GQ149643	Human H1N1pdm2009 IAVs	Human	H1N1	pdm	2009	Mexico	A/Mexico/4486/2009	ggtgatgccccattccttgatcggctccgccgagatcaaaagtccttaaaaggaagaggcaacaccc	-	*
GQ149680	Human H1N1pdm2009 IAVs	Human	H1N1	pdm	2009	Mexico	A/Mexico/4603/2009	ggtgatgccccattccttgatcggctccgccgagatcaaaagtccttaaaaggaagaggcaacaccc	-	*

GQ149626	Human H1N1pdm2009 IAVs	Human	H1N1	pdm	2009	Mexico	A/Mexico/4603/2009	ggtgatgccccattccttgatcggctccgccgagatca aaagtccttaaaaggaagaggcaacaccc	-	*
GQ149635	Human H1N1pdm2009 IAVs	Human	H1N1	pdm	2009	Mexico	A/Mexico/4604/2009	ggtgatgccccattccttgatcggctccgccgagatca aaagtccttaaaaggaagaggcaacaccc	-	*
CY097880	Human H1N1pdm2009 IAVs	Human	H1N1	pdm	2009	Mexico	A/Mexico/47N/2009	ggtgatgccccattccttgatcggctccgccgagatca aaagtccttaaaaggaagaggcaacaccc	-	*
CY040892	Human H1N1pdm2009 IAVs	Human	H1N1		2009	Mexico	A/Mexico/47N/2009	ggtgatgccccattccttgatcggctccgccgagatca aaagtccttaaaaggaagaggcaacaccc	-	*
CY044167	Human H1N1pdm2009 IAVs	Human	H1N1	pdm	2009	Mexico	A/Mexico/48N/2009	ggtgatgccccattccttgatcggctccgccgagatca aaagtccttaaaaggaagaggcaacaccc	-	*
GQ117110	Human H1N1pdm2009 IAVs	Human	H1N1	pdm	2009	USA	A/Michigan/02/2009	ggtgatgccccattccttgatcggctccgccgagatca aaagtccttaaaaggaagaggcaacaccc	-	*
KC780152	Human H1N1pdm2009 IAVs	Human	H1N1		2009	USA	A/Mississippi/03/2009	ggtgatgccccattccttgatcggctccgccgagatca aaagtccttaaaaggaagaggcaacaccc	-	*
GQ231984	Human H1N1pdm2009 IAVs	Human	H1N1	pdm	2009	USA	A/Missouri/01/2009	ggtgatgccccattccttgatcggctccgccgagatca aaagtccttaaaaggaagaggcaacaccc	-	*
FJ998220	Human H1N1pdm2009 IAVs	Human	H1N1	pdm	2009	Mexico	A/Mexico/InDRE4487/2009	ggtgatgccccattccttgatcggctccgccgagatca aaagtccttaaaaggaagaggcaacaccc	-	*
GQ504755	Human H1N1pdm2009 IAVs	Human	H1N1	pdm	2009	China	A/Nanjing/1/2009	ggtgatgccccattccttgatcggctccgccgagatca aaagtccttaaaaggaagaggcaacaccc	-	*
GQ455036	Human H1N1pdm2009 IAVs	Human	H1N1	pdm	2009	China	A/Nanjing/2/2009	ggtgatgccccattccttgatcggctccgccgagatca aaagtccttaaaaggaagaggcaacaccc	-	*
GQ169304	Human H1N1pdm2009 IAVs	Human	H1N1	pdm	2009	Japan	A/Narita/1/2009	ggtgatgccccattccttgatcggctccgccgagatca aaagtccttaaaaggaagaggcaacaccc	-	*
CY148039	Human H1N1pdm2009 IAVs	Human	H1N1	pdm	2009	Netherlands	A/Netherlands/602/2009	ggtgatgccccattccttgatcggctccgccgagatca aaagtccttaaaaggaagaggcaacaccc	-	*
CY046945	Human H1N1pdm2009 IAVs	Human	H1N1	pdm	2009	Netherlands	A/Netherlands/602/2009	ggtgatgccccattccttgatcggctccgccgagatca aaagtccttaaaaggaagaggcaacaccc	-	*
GQ457493	Human H1N1pdm2009 IAVs	Human	H1N1	pdm	2009	USA	A/Nevada/04/2009	ggtgatgccccattccttgatcggctccgccgagatca aaagtccttaaaaggaagaggcaacaccc	-	*
GQ160537	Human H1N1pdm2009 IAVs	Human	H1N1	pdm	2009	USA	A/New_Mexico/04/2009	ggtgatgccccattccttgatcggctccgccgagatca aaagtccttaaaaggaagaggcaacaccc	-	*
KC780372	Human H1N1pdm2009 IAVs	Human	H1N1		2009	USA	A/New_York/101/2009	ggtgatgccccattccttgatcggctccgccgagatca aaagtccttaaaaggaagaggcaacaccc	-	*
CY040738	Human H1N1pdm2009 IAVs	Human	H1N1	pdm	2009	USA	A/New_York/3240/2009	ggtgatgccccattccttgatcggctccgccgagatca aaagtccttaaaaggaagaggcaacaccc	-	*
CY039990	Human H1N1pdm2009 IAVs	Human	H1N1	pdm	2009	Thailand	A/Nonthaburi/102/2009	ggtgatgccccattccttgatcggctccgccgagatca aaagtccttaaaaggaagaggcaacaccc	-	*
GQ166233	Human H1N1pdm2009 IAVs	Human	H1N1	pdm	2009	Thailand	A/Nonthaburi/102/2009	ggtgatgccccattccttgatcggctccgccgagatca aaagtccttaaaaggaagaggcaacaccc	-	*
HM241721	Human H1N1pdm2009 IAVs	Human	H1N1	pdm	2009	India	A/Nsk/NIV10348/2009	ggtgatgccccattccttgatcggctccgccgagatca aaagtccttaaaaggaagaggcaacaccc	-	*
KC781845	Human H1N1pdm2009 IAVs	Human	H1N1	pdm	2009	USA	A/Oregon/13/2009	ggtgatgccccattccttgatcggctccgccgagatca aaagtccttaaaaggaagaggcaacaccc	-	*
GQ249340	Human H1N1pdm2009 IAVs	Human	H1N1	pdm	2009	France	A/Paris/2629/2009	ggtgatgccccattccttgatcggctccgccgagatca aaagtccttaaaaggaagaggcaacaccc	-	*
GQ329078	Human H1N1pdm2009 IAVs	Human	H1N1	pdm	2009	France	A/Paris/2722/2009	ggtgatgccccattccttgatcggctccgccgagatca aaagtccttaaaaggaagaggcaacaccc	-	*
GQ221817	Human H1N1pdm2009 IAVs	Human	H1N1	pdm	2009	USA	A/Pennsylvania/06/2009	ggtgatgccccattccttgatcggctccgccgagatca aaagtccttaaaaggaagaggcaacaccc	-	*

FN401578	Human H1N1pdm2009 IAVs	Human	H1N1	pdm	2009	Germany	A/Regensburg/D6/2009	ggtgatgccccattccttgatcggctccgccgagatca aaagtccttaaaaggaagaggcaacaccc	-	*
GQ232043	Human H1N1pdm2009 IAVs	Human	H1N1	pdm	2009	USA	A/Rhode_Island/02/2009	ggtgatgccccattccttgatcggctccgccgagatca aaagtccttaaaaggaagaggcaacaccc	-	*
KF612176	Human H1N1pdm2009 IAVs	Human	H1N1	pdm	2009	Bolivia	A/Santa_Cruz/21641/2009	ggtgatgccccattccttgatcggctccgccgagatca aaagtccttaaaaggaagaggcaacaccc	-	*
KF612178	Human H1N1pdm2009 IAVs	Human	H1N1	pdm	2009	Bolivia	A/Santa_Cruz/87212/2009	ggtgatgccccattccttgatcggctccgccgagatca aaagtccttaaaaggaagaggcaacaccc	-	*
KF612174	Human H1N1pdm2009 IAVs	Human	H1N1	pdm	2009	Bolivia	A/Santa_Cruz/94841/2009	ggtgatgccccattccttgatcggctccgccgagatca aaagtccttaaaaggaagaggcaacaccc	-	*
CY043114	Human H1N1pdm2009 IAVs	Human	H1N1	pdm	2009	Dominican_Republic	A/Santo_Domingo/568T/2009	ggtgatgccccattccttgatcggctccgccgagatca aaagtccttaaaaggaagaggcaacaccc	-	*
CY083156	Human H1N1pdm2009 IAVs	Human	H1N1	pdm	2009	Dominican_Republic	A/Santo_Domingo/WRAIR1044N/2009	ggtgatgccccattccttgatcggctccgccgagatca aaagtccttaaaaggaagaggcaacaccc	-	*
CY065298	Human H1N1pdm2009 IAVs	Human	H1N1	pdm	2009	United_Kingdom	A/Scotland/4/2009	ggtgatgccccattccttgatcggctccgccgagatca aaagtccttaaaaggaagaggcaacaccc	-	*
CY107742	Human H1N1pdm2009 IAVs	Human	H1N1		2009	United_Kingdom	A/Scotland/Aberdeen_416064/2009	ggtgatgccccattccttgatcggctccgccgagatca aaagtccttaaaaggaagaggcaacaccc	-	*
CY107476	Human H1N1pdm2009 IAVs	Human	H1N1	pdm	2009	United_Kingdom	A/Scotland/Edinburgh_17343/2009	ggtgatgccccattccttgatcggctccgccgagatca aaagtccttaaaaggaagaggcaacaccc	-	*
CY107483	Human H1N1pdm2009 IAVs	Human	H1N1	pdm	2009	United_Kingdom	A/Scotland/Edinburgh_17645/2009	ggtgatgccccattccttgatcggctccgccgagatca aaagtccttaaaaggaagaggcaacaccc	-	*
CY107766	Human H1N1pdm2009 IAVs	Human	H1N1	pdm	2009	United_Kingdom	A/Scotland/Inverness_418366/2009	ggtgatgccccattccttgatcggctccgccgagatca aaagtccttaaaaggaagaggcaacaccc	-	*
CY107189	Human H1N1pdm2009 IAVs	Human	H1N1	pdm	2009	United_Kingdom	A/Scotland/Paisley_409499/2009	ggtgatgccccattccttgatcggctccgccgagatca aaagtccttaaaaggaagaggcaacaccc	-	*
GQ200294	Human H1N1pdm2009 IAVs	Human	H1N1	pdm	2009	China	A/Shandong/1/2009	ggtgatgccccattccttgatcggctccgccgagatca aaagtccttaaaaggaagaggcaacaccc	-	*
GQ340063	Human H1N1pdm2009 IAVs	Human	H1N1	pdm	2009	China	A/Shanghai/143T/2009	ggtgatgccccattccttgatcggctccgccgagatca aaagtccttaaaaggaagaggcaacaccc	-	*
GQ166230	Human H1N1pdm2009 IAVs	Human	H1N1	pdm	2009	China	A/Sichuan/1/2009	ggtgatgccccattccttgatcggctccgccgagatca aaagtccttaaaaggaagaggcaacaccc	-	*
CY076767	Human H1N1pdm2009 IAVs	Human	H1N1	pdm	2009	Canada	A/Ontario/147265/2009	ggtgatgccccattccttgatcggctccgccgagatca aaagtccttaaaaggaagaggcaacaccc	-	*
CY076783	Human H1N1pdm2009 IAVs	Human	H1N1	pdm	2009	Canada	A/Ontario/152439/2009	ggtgatgccccattccttgatcggctccgccgagatca aaagtccttaaaaggaagaggcaacaccc	-	*
CY060754	Human H1N1pdm2009 IAVs	Human	H1N1	pdm	2009	Canada	A/Ontario/9739/2009	ggtgatgccccattccttgatcggctccgccgagatca aaagtccttaaaaggaagaggcaacaccc	-	*
CY073282	Human H1N1pdm2009 IAVs	Human	H1N1		2009	USA	A/Silver_Spring/WRAIRSP510P18/2009	ggtgatgccccattccttgatcggctccgccgagatca aaagtccttaaaaggaagaggcaacaccc	-	*
GQ117054	Human H1N1pdm2009 IAVs	Human	H1N1	pdm	2009	USA	A/South_Carolina/09/2009	ggtgatgccccattccttgatcggctccgccgagatca aaagtccttaaaaggaagaggcaacaccc	-	*
JF714040	Human H1N1pdm2009 IAVs	Swine	H1N1		2009	Canada	A/swine/MB/10422/2009	ggtgatgccccattccttgatcggctccgccgagatca aaagtccttaaaaggaagaggcaacaccc	-	*
JF714056	Human H1N1pdm2009 IAVs	Swine	H1N1		2009	Canada	A/swine/MB/31/2009	ggtgatgccccattccttgatcggctccgccgagatca aaagtccttaaaaggaagaggcaacaccc	-	*
JF714032	Human H1N1pdm2009 IAVs	Swine	H1N1		2009	Canada	A/swine/MB/46/2009	ggtgatgccccattccttgatcggctccgccgagatca aaagtccttaaaaggaagaggcaacaccc	-	*
JF714076	Human H1N1pdm2009 IAVs	Swine	H1N1		2009	Canada	A/swine/MB/54/2009	ggtgatgccccattccttgatcggctccgccgagatca aaagtccttaaaaggaagaggcaacaccc	-	*

AB620211	Human H1N1pdm2009 IAVs	Swine	H1N1		2009	Thailand	A/swine/Saraburi/NIAH116627_24/2009	ggtgatgccccattccttgatcggctccgccgagatcaaaagtccttaaaaggaagaggcaacaccc	-	*
KC859194	Human H1N1pdm2009 IAVs	Swine	H1N1		2009	Thailand	A/swine/Thailand/UD401/2009	ggtgatgccccattccttgatcggctccgccgagatcaaaagtccttaaaaggaagaggcaacaccc	-	*
KC859202	Human H1N1pdm2009 IAVs	Swine	H1N1		2009	Thailand	A/swine/Thailand/UD402/2009	ggtgatgccccattccttgatcggctccgccgagatcaaaagtccttaaaaggaagaggcaacaccc	-	*
GQ160530	Human H1N1pdm2009 IAVs	Human	H1N1	pdm	2009	USA	A/Tennessee/05/2009	ggtgatgccccattccttgatcggctccgccgagatcaaaagtccttaaaaggaagaggcaacaccc	-	*
GQ168869	Human H1N1pdm2009 IAVs	Human	H1N1	pdm	2009	USA	A/Texas/07/2009	ggtgatgccccattccttgatcggctccgccgagatcaaaagtccttaaaaggaagaggcaacaccc	-	*
GQ160548	Human H1N1pdm2009 IAVs	Human	H1N1	pdm	2009	USA	A/Texas/23/2009	ggtgatgccccattccttgatcggctccgccgagatcaaaagtccttaaaaggaagaggcaacaccc	-	*
GQ866944	Human H1N1pdm2009 IAVs	Human	H1N1	pdm	2009	Thailand	A/Thailand/CU_H106/2009	ggtgatgccccattccttgatcggctccgccgagatcaaaagtccttaaaaggaagaggcaacaccc	-	*
GQ324574	Human H1N1pdm2009 IAVs	Human	H1N1	pdm	2009	Japan	A/Tokushima/1/2009	ggtgatgccccattccttgatcggctccgccgagatcaaaagtccttaaaaggaagaggcaacaccc	-	*
CY045942	Human H1N1pdm2009 IAVs	Human	H1N1		2009	Canada	A/Toronto/C0270/2009	ggtgatgccccattccttgatcggctccgccgagatcaaaagtccttaaaaggaagaggcaacaccc	-	*
CY045950	Human H1N1pdm2009 IAVs	Human	H1N1	pdm	2009	Canada	A/Toronto/C2781/2009	ggtgatgccccattccttgatcggctccgccgagatcaaaagtccttaaaaggaagaggcaacaccc	-	*
GQ232006	Human H1N1pdm2009 IAVs	Human	H1N1	pdm	2009	USA	A/Vermont/03/2009	ggtgatgccccattccttgatcggctccgccgagatcaaaagtccttaaaaggaagaggcaacaccc	-	*
GQ160604	Human H1N1pdm2009 IAVs	Human	H1N1	pdm	2009	USA	A/Wisconsin/08/2009	ggtgatgccccattccttgatcggctccgccgagatcaaaagtccttaaaaggaagaggcaacaccc	-	*
CY051067	Human H1N1pdm2009 IAVs	Human	H1N1	pdm	2009	USA	A/Wisconsin/629_D00101/2009	ggtgatgccccattccttgatcggctccgccgagatcaaaagtccttaaaaggaagaggcaacaccc	-	*
CY051131	Human H1N1pdm2009 IAVs	Human	H1N1	pdm	2009	USA	A/Wisconsin/629_D00119/2009	ggtgatgccccattccttgatcggctccgccgagatcaaaagtccttaaaaggaagaggcaacaccc	-	*
CY046559	Human H1N1pdm2009 IAVs	Human	H1N1	pdm	2009	USA	A/Wisconsin/629_D00228/2009	ggtgatgccccattccttgatcggctccgccgagatcaaaagtccttaaaaggaagaggcaacaccc	-	*
CY046487	Human H1N1pdm2009 IAVs	Human	H1N1	pdm	2009	USA	A/Wisconsin/629_D00349/2009	ggtgatgccccattccttgatcggctccgccgagatcaaaagtccttaaaaggaagaggcaacaccc	-	*
CY046535	Human H1N1pdm2009 IAVs	Human	H1N1	pdm	2009	USA	A/Wisconsin/629_D00714/2009	ggtgatgccccattccttgatcggctccgccgagatcaaaagtccttaaaaggaagaggcaacaccc	-	*
CY046719	Human H1N1pdm2009 IAVs	Human	H1N1	pdm	2009	USA	A/Wisconsin/629_D00752/2009	ggtgatgccccattccttgatcggctccgccgagatcaaaagtccttaaaaggaagaggcaacaccc	-	*
CY046575	Human H1N1pdm2009 IAVs	Human	H1N1	pdm	2009	USA	A/Wisconsin/629_D00905/2009	ggtgatgccccattccttgatcggctccgccgagatcaaaagtccttaaaaggaagaggcaacaccc	-	*
CY051123	Human H1N1pdm2009 IAVs	Human	H1N1	pdm	2009	USA	A/Wisconsin/629_D00916/2009	ggtgatgccccattccttgatcggctccgccgagatcaaaagtccttaaaaggaagaggcaacaccc	-	*
CY046271	Human H1N1pdm2009 IAVs	Human	H1N1	pdm	2009	USA	A/Wisconsin/629_D00933/2009	ggtgatgccccattccttgatcggctccgccgagatcaaaagtccttaaaaggaagaggcaacaccc	-	*
CY046279	Human H1N1pdm2009 IAVs	Human	H1N1	pdm	2009	USA	A/Wisconsin/629_D01092/2009	ggtgatgccccattccttgatcggctccgccgagatcaaaagtccttaaaaggaagaggcaacaccc	-	*
CY089908	Human H1N1pdm2009 IAVs	Human	H1N1	pdm	2009	USA	A/Wisconsin/629_D01115/2009	ggtgatgccccattccttgatcggctccgccgagatcaaaagtccttaaaaggaagaggcaacaccc	-	*
CY046679	Human H1N1pdm2009 IAVs	Human	H1N1	pdm	2009	USA	A/Wisconsin/629_D01305/2009	ggtgatgccccattccttgatcggctccgccgagatcaaaagtccttaaaaggaagaggcaacaccc	-	*
CY046303	Human H1N1pdm2009 IAVs	Human	H1N1	pdm	2009	USA	A/Wisconsin/629_D01486/2009	ggtgatgccccattccttgatcggctccgccgagatcaaaagtccttaaaaggaagaggcaacaccc	-	*

CY046735	Human H1N1pdm2009 IAVs	Human	H1N1	pdm	2009	USA	A/Wisconsin/629_D01521/2009	ggtgatgccccattccttgatcggctccgccgagatca aaagtccttaaaaggaagaggcaacaccc	-	*
CY046247	Human H1N1pdm2009 IAVs	Human	H1N1	pdm	2009	USA	A/Wisconsin/629_D01606/2009	ggtgatgccccattccttgatcggctccgccgagatca aaagtccttaaaaggaagaggcaacaccc	-	*
CY051235	Human H1N1pdm2009 IAVs	Human	H1N1	pdm	2009	USA	A/Wisconsin/629_D01664/2009	ggtgatgccccattccttgatcggctccgccgagatca aaagtccttaaaaggaagaggcaacaccc	-	*
CY046263	Human H1N1pdm2009 IAVs	Human	H1N1	pdm	2009	USA	A/Wisconsin/629_D01773/2009	ggtgatgccccattccttgatcggctccgccgagatca aaagtccttaaaaggaagaggcaacaccc	-	*
CY046463	Human H1N1pdm2009 IAVs	Human	H1N1	pdm	2009	USA	A/Wisconsin/629_D01861/2009	ggtgatgccccattccttgatcggctccgccgagatca aaagtccttaaaaggaagaggcaacaccc	-	*
CY046839	Human H1N1pdm2009 IAVs	Human	H1N1	pdm	2009	USA	A/Wisconsin/629_D01883/2009	ggtgatgccccattccttgatcggctccgccgagatca aaagtccttaaaaggaagaggcaacaccc	-	*
CY046783	Human H1N1pdm2009 IAVs	Human	H1N1	pdm	2009	USA	A/Wisconsin/629_D01894/2009	ggtgatgccccattccttgatcggctccgccgagatca aaagtccttaaaaggaagaggcaacaccc	-	*
CY046583	Human H1N1pdm2009 IAVs	Human	H1N1	pdm	2009	USA	A/Wisconsin/629_D01909/2009	ggtgatgccccattccttgatcggctccgccgagatca aaagtccttaaaaggaagaggcaacaccc	-	*
CY046567	Human H1N1pdm2009 IAVs	Human	H1N1	pdm	2009	USA	A/Wisconsin/629_D01919/2009	ggtgatgccccattccttgatcggctccgccgagatca aaagtccttaaaaggaagaggcaacaccc	-	*
CY046823	Human H1N1pdm2009 IAVs	Human	H1N1	pdm	2009	USA	A/Wisconsin/629_D02013/2009	ggtgatgccccattccttgatcggctccgccgagatca aaagtccttaaaaggaagaggcaacaccc	-	*
CY046495	Human H1N1pdm2009 IAVs	Human	H1N1	pdm	2009	USA	A/Wisconsin/629_D02028/2009	ggtgatgccccattccttgatcggctccgccgagatca aaagtccttaaaaggaagaggcaacaccc	-	*
CY060506	Human H1N1pdm2009 IAVs	Human	H1N1	pdm	2009	Canada	A/Ontario/10016/2009	ggtgatgccccattccttgatcggctccgccgagatca aaagtccttaaaaggaagaggcaacaccc	-	*
CY060514	Human H1N1pdm2009 IAVs	Human	H1N1	pdm	2009	Canada	A/Ontario/10296/2009	ggtgatgccccattccttgatcggctccgccgagatca aaagtccttaaaaggaagaggcaacaccc	-	*
CY076759	Human H1N1pdm2009 IAVs	Human	H1N1	pdm	2009	Canada	A/Ontario/142358/2009	ggtgatgccccattccttgatcggctccgccgagatca aaagtccttaaaaggaagaggcaacaccc	-	*
CY076775	Human H1N1pdm2009 IAVs	Human	H1N1	pdm	2009	Canada	A/Ontario/147723/2009	ggtgatgccccattccttgatcggctccgccgagatca aaagtccttaaaaggaagaggcaacaccc	-	*
CY076791	Human H1N1pdm2009 IAVs	Human	H1N1	pdm	2009	Canada	A/Ontario/152846/2009	ggtgatgccccattccttgatcggctccgccgagatca aaagtccttaaaaggaagaggcaacaccc	-	*
CY076798	Human H1N1pdm2009 IAVs	Human	H1N1	pdm	2009	Canada	A/Ontario/156785/2009	ggtgatgccccattccttgatcggctccgccgagatca aaagtccttaaaaggaagaggcaacaccc	-	*
CY060546	Human H1N1pdm2009 IAVs	Human	H1N1	pdm	2009	Canada	A/Ontario/25389/2009	ggtgatgccccattccttgatcggctccgccgagatca aaagtccttaaaaggaagaggcaacaccc	-	*
CY060746	Human H1N1pdm2009 IAVs	Human	H1N1	pdm	2009	Canada	A/Ontario/9698/2009	ggtgatgccccattccttgatcggctccgccgagatca aaagtccttaaaaggaagaggcaacaccc	-	*
CY122699	Human H1N1pdm2009 IAVs	Human	H1N1	pdm	2009	Singapore	A/Singapore/GP1142/2009	ggtgatgccccattccttgatcggctccgccgagatca aaagtccttaaaaggaagaggcaacaccc	-	*
GQ392033	Human H1N1pdm2009 IAVs	Human	H1N1	pdm	2009	Italy	A/Italy/127/2009	ggtgatgccccattccttgatcggctccgccgagatca aaagtccttaaaaggaagaggcaacaccc	-	*
GQ283489	Human H1N1pdm2009 IAVs	Human	H1N1	pdm	2009	Finland	A/Finland/554/2009	ggtgatgccccattccttgatcggctccgccgagatca aaagtccttaaaaggaagaggcaacaccc	-	*
GQ283494	Human H1N1pdm2009 IAVs	Human	H1N1	pdm	2009	Finland	A/Finland/555/2009	ggtgatgccccattccttgatcggctccgccgagatca aaagtccttaaaaggaagaggcaacaccc	-	*
CY050202	Human H1N1pdm2009 IAVs	Human	H1N1		2009	Mexico	A/Mexico_City/001/2009	ggtgatgccccattccttgatcggctccgccgagatca aaagtccttaaaaggaagaggcaacaccc	-	*
CY050210	Human H1N1pdm2009 IAVs	Human	H1N1	pdm	2009	Mexico	A/Mexico_City/002/2009	ggtgatgccccattccttgatcggctccgccgagatca aaagtccttaaaaggaagaggcaacaccc	-	*

CY050226	Human H1N1pdm2009 IAVs	Human	H1N1	pdm	2009	Mexico	A/Mexico_City/004/2009	ggtgatgccccattccttgatcggctccgccgagatca<br>aaagtccttaaaaggaagaggcaacaccc	-	*
CY050875	Human H1N1pdm2009 IAVs	Human	H1N1	pdm	2009	Mexico	A/Mexico_City/017/2009	ggtgatgccccattccttgatcggctccgccgagatca<br>aaagtccttaaaaggaagaggcaacaccc	-	*
GQ290062	Human H1N1pdm2009 IAVs	Human	H1N1		2009	Russia	A/Moscow/226/2009	ggtgatgccccattccttgatcggctccgccgagatca<br>aaagtccttaaaaggaagaggcaacaccc	-	*
CY039905	Human H1N1pdm2009 IAVs	Human	H1N1	pdm	2009	USA	A/New_York/1682/2009	ggtgatgccccattccttgatcggctccgccgagatca<br>aaagtccttaaaaggaagaggcaacaccc	-	*
CY040035	Human H1N1pdm2009 IAVs	Human	H1N1	pdm	2009	USA	A/New_York/3099/2009	ggtgatgccccattccttgatcggctccgccgagatca<br>aaagtccttaaaaggaagaggcaacaccc	-	*
CY041601	Human H1N1pdm2009 IAVs	Human	H1N1	pdm	2009	USA	A/New_York/3177/2009	ggtgatgccccattccttgatcggctccgccgagatca<br>aaagtccttaaaaggaagaggcaacaccc	-	*
CY041150	Human H1N1pdm2009 IAVs	Human	H1N1	pdm	2009	USA	A/New_York/3181/2009	ggtgatgccccattccttgatcggctccgccgagatca<br>aaagtccttaaaaggaagaggcaacaccc	-	*
CY041158	Human H1N1pdm2009 IAVs	Human	H1N1	pdm	2009	USA	A/New_York/3183/2009	ggtgatgccccattccttgatcggctccgccgagatca<br>aaagtccttaaaaggaagaggcaacaccc	-	*
CY041754	Human H1N1pdm2009 IAVs	Human	H1N1	pdm	2009	USA	A/New_York/3323/2009	ggtgatgccccattccttgatcggctccgccgagatca<br>aaagtccttaaaaggaagaggcaacaccc	-	*
CY043199	Human H1N1pdm2009 IAVs	Human	H1N1	pdm	2009	USA	A/New_York/3324/2009	ggtgatgccccattccttgatcggctccgccgagatca<br>aaagtccttaaaaggaagaggcaacaccc	-	*
CY041786	Human H1N1pdm2009 IAVs	Human	H1N1	pdm	2009	USA	A/New_York/3337/2009	ggtgatgccccattccttgatcggctccgccgagatca<br>aaagtccttaaaaggaagaggcaacaccc	-	*
CY043207	Human H1N1pdm2009 IAVs	Human	H1N1	pdm	2009	USA	A/New_York/3338/2009	ggtgatgccccattccttgatcggctccgccgagatca<br>aaagtccttaaaaggaagaggcaacaccc	-	*
CY041834	Human H1N1pdm2009 IAVs	Human	H1N1	pdm	2009	USA	A/New_York/3348/2009	ggtgatgccccattccttgatcggctccgccgagatca<br>aaagtccttaaaaggaagaggcaacaccc	-	*
CY043287	Human H1N1pdm2009 IAVs	Human	H1N1	pdm	2009	USA	A/New_York/3449/2009	ggtgatgccccattccttgatcggctccgccgagatca<br>aaagtccttaaaaggaagaggcaacaccc	-	*
CY043135	Human H1N1pdm2009 IAVs	Human	H1N1	pdm	2009	USA	A/New_York/3514/2009	ggtgatgccccattccttgatcggctccgccgagatca<br>aaagtccttaaaaggaagaggcaacaccc	-	*
CY122719	Human H1N1pdm2009 IAVs	Human	H1N1	pdm	2009	Singapore	A/Singapore/GP1156/2009	ggtgatgccccattccttgatcggctccgccgagatca<br>aaagtccttaaaaggaagaggcaacaccc	-	*
CY122947	Human H1N1pdm2009 IAVs	Human	H1N1	pdm	2009	Singapore	A/Singapore/GP2697/2009	ggtgatgccccattccttgatcggctccgccgagatca<br>aaagtccttaaaaggaagaggcaacaccc	-	*
CY123544	Human H1N1pdm2009 IAVs	Human	H1N1	pdm	2009	Singapore	A/Singapore/ON1811/2009	ggtgatgccccattccttgatcggctccgccgagatca<br>aaagtccttaaaaggaagaggcaacaccc	-	*
CY123580	Human H1N1pdm2009 IAVs	Human	H1N1	pdm	2009	Singapore	A/Singapore/ON1888/2009	ggtgatgccccattccttgatcggctccgccgagatca<br>aaagtccttaaaaggaagaggcaacaccc	-	*
CY123612	Human H1N1pdm2009 IAVs	Human	H1N1		2009	Singapore	A/Singapore/ON1908/2009	ggtgatgccccattccttgatcggctccgccgagatca<br>aaagtccttaaaaggaagaggcaacaccc	-	*
CY123779	Human H1N1pdm2009 IAVs	Human	H1N1	pdm	2009	Singapore	A/Singapore/ON2136/2009	ggtgatgccccattccttgatcggctccgccgagatca<br>aaagtccttaaaaggaagaggcaacaccc	-	*
CY050154	Human H1N1pdm2009 IAVs	Human	H1N1	pdm	2009	USA	A/Wisconsin/629_D00117/2009	ggtgatgccccattccttgatcggctccgccgagatca<br>aaagtccttaaaaggaagaggcaacaccc	-	*
CY046527	Human H1N1pdm2009 IAVs	Human	H1N1	pdm	2009	USA	A/Wisconsin/629_D00183/2009	ggtgatgccccattccttgatcggctccgccgagatca<br>aaagtccttaaaaggaagaggcaacaccc	-	*
CY051099	Human H1N1pdm2009 IAVs	Human	H1N1	pdm	2009	USA	A/Wisconsin/629_D00196/2009	ggtgatgccccattccttgatcggctccgccgagatca<br>aaagtccttaaaaggaagaggcaacaccc	-	*
CY051075	Human H1N1pdm2009 IAVs	Human	H1N1	pdm	2009	USA	A/Wisconsin/629_D00579/2009	ggtgatgccccattccttgatcggctccgccgagatca<br>aaagtccttaaaaggaagaggcaacaccc	-	*

CY046903	Human H1N1pdm2009 IAVs	Human	H1N1	pdm	2009	USA	A/Wisconsin/629_D00829/2009	ggtgatgccccattccttgatcggctccgccgagatca aaagtccttaaaaggaagaggcaacaccc	-	*
GQ426222	Human H1N1pdm2009 IAVs	Human	H1N1		2009	Russia	A/Tomsk/01/2009	ggtgatgccccattccttgatcggctccgccgagatca aaagtccttaaaaggaagaggcaacaccc	-	*
CY046639	Human H1N1pdm2009 IAVs	Human	H1N1	pdm	2009	USA	A/Wisconsin/629_D00967/2009	ggtgatgccccattccttgatcggctccgccgagatca aaagtccttaaaaggaagaggcaacaccc	-	*
CY046223	Human H1N1pdm2009 IAVs	Human	H1N1	pdm	2009	USA	A/Wisconsin/629_D01251/2009	ggtgatgccccattccttgatcggctccgccgagatca aaagtccttaaaaggaagaggcaacaccc	-	*
CY046231	Human H1N1pdm2009 IAVs	Human	H1N1	pdm	2009	USA	A/Wisconsin/629_D01296/2009	ggtgatgccccattccttgatcggctccgccgagatca aaagtccttaaaaggaagaggcaacaccc	-	*
CY046863	Human H1N1pdm2009 IAVs	Human	H1N1	pdm	2009	USA	A/Wisconsin/629_D01415/2009	ggtgatgccccattccttgatcggctccgccgagatca aaagtccttaaaaggaagaggcaacaccc	-	*
CY046455	Human H1N1pdm2009 IAVs	Human	H1N1	pdm	2009	USA	A/Wisconsin/629_D01558/2009	ggtgatgccccattccttgatcggctccgccgagatca aaagtccttaaaaggaagaggcaacaccc	-	*
CY051107	Human H1N1pdm2009 IAVs	Human	H1N1	pdm	2009	USA	A/Wisconsin/629_D01618/2009	ggtgatgccccattccttgatcggctccgccgagatca aaagtccttaaaaggaagaggcaacaccc	-	*
CY046919	Human H1N1pdm2009 IAVs	Human	H1N1	pdm	2009	USA	A/Wisconsin/629_D01764/2009	ggtgatgccccattccttgatcggctccgccgagatca aaagtccttaaaaggaagaggcaacaccc	-	*
CY046383	Human H1N1pdm2009 IAVs	Human	H1N1	pdm	2009	USA	A/Wisconsin/629_D01980/2009	ggtgatgccccattccttgatcggctccgccgagatca aaagtccttaaaaggaagaggcaacaccc	-	*
CY046751	Human H1N1pdm2009 IAVs	Human	H1N1	pdm	2009	USA	A/Wisconsin/629_D02064/2009	ggtgatgccccattccttgatcggctccgccgagatca aaagtccttaaaaggaagaggcaacaccc	-	*
CY051059	Human H1N1pdm2009 IAVs	Human	H1N1	pdm	2009	USA	A/Wisconsin/629_D02068/2009	ggtgatgccccattccttgatcggctccgccgagatca aaagtccttaaaaggaagaggcaacaccc	-	*
CY046519	Human H1N1pdm2009 IAVs	Human	H1N1	pdm	2009	USA	A/Wisconsin/629_D02141/2009	ggtgatgccccattccttgatcggctccgccgagatca aaagtccttaaaaggaagaggcaacaccc	-	*
CY046831	Human H1N1pdm2009 IAVs	Human	H1N1	pdm	2009	USA	A/Wisconsin/629_D02144/2009	ggtgatgccccattccttgatcggctccgccgagatca aaagtccttaaaaggaagaggcaacaccc	-	*
CY046207	Human H1N1pdm2009 IAVs	Human	H1N1	pdm	2009	USA	A/Wisconsin/629_D02408/2009	ggtgatgccccattccttgatcggctccgccgagatca aaagtccttaaaaggaagaggcaacaccc	-	*
CY046511	Human H1N1pdm2009 IAVs	Human	H1N1	pdm	2009	USA	A/Wisconsin/629_D02425/2009	ggtgatgccccattccttgatcggctccgccgagatca aaagtccttaaaaggaagaggcaacaccc	-	*
CY046255	Human H1N1pdm2009 IAVs	Human	H1N1	pdm	2009	USA	A/Wisconsin/629_D02432/2009	ggtgatgccccattccttgatcggctccgccgagatca aaagtccttaaaaggaagaggcaacaccc	-	*
CY046591	Human H1N1pdm2009 IAVs	Human	H1N1	pdm	2009	USA	A/Wisconsin/629_D02452/2009	ggtgatgccccattccttgatcggctccgccgagatca aaagtccttaaaaggaagaggcaacaccc	-	*
GQ359762	Human H1N1pdm2009 IAVs	Human	H1N1		2009	China	A/Zhejiang/DTID_ZJU01/2009	ggtgatgccccattccttgatcggctccgccgagatca aaagtccttaaaaggaagaggcaacaccc	-	*
CY073493	Human H1N1pdm2009 IAVs	Human	H1N1	pdm	2009	Greece	A/Athens/INS271/2009	ggtgatgccccattccttgatcggctccgccgagatca aaagtccttaaaaggaagaggcaacaccc	-	*
CY066603	Human H1N1pdm2009 IAVs	Human	H1N1	pdm	2009	Greece	A/Athens/INS272/2009	ggtgatgccccattccttgatcggctccgccgagatca aaagtccttaaaaggaagaggcaacaccc	-	*
CY064472	Human H1N1pdm2009 IAVs	Human	H1N1		2009	USA	A/Boston/103/2009	ggtgatgccccattccttgatcggctccgccgagatca aaagtccttaaaaggaagaggcaacaccc	-	*
CY064496	Human H1N1pdm2009 IAVs	Human	H1N1	pdm	2009	USA	A/Boston/109/2009	ggtgatgccccattccttgatcggctccgccgagatca aaagtccttaaaaggaagaggcaacaccc	-	*
CY063490	Human H1N1pdm2009 IAVs	Human	H1N1	pdm	2009	USA	A/Boston/110/2009	ggtgatgccccattccttgatcggctccgccgagatca aaagtccttaaaaggaagaggcaacaccc	-	*
CY064504	Human H1N1pdm2009 IAVs	Human	H1N1	pdm	2009	USA	A/Boston/115/2009	ggtgatgccccattccttgatcggctccgccgagatca aaagtccttaaaaggaagaggcaacaccc	-	*

CY064568	Human H1N1pdm2009 IAVs	Human	H1N1	pdm	2009	USA	A/Boston/124/2009	ggtgatgccccattccttgatcggctccgccgagatca aaagtccttaaaaggaagaggcaacaccc	-	*
CY064584	Human H1N1pdm2009 IAVs	Human	H1N1	pdm	2009	USA	A/Boston/126/2009	ggtgatgccccattccttgatcggctccgccgagatca aaagtccttaaaaggaagaggcaacaccc	-	*
CY064608	Human H1N1pdm2009 IAVs	Human	H1N1	pdm	2009	USA	A/Boston/130/2009	ggtgatgccccattccttgatcggctccgccgagatca aaagtccttaaaaggaagaggcaacaccc	-	*
CY064624	Human H1N1pdm2009 IAVs	Human	H1N1	pdm	2009	USA	A/Boston/132/2009	ggtgatgccccattccttgatcggctccgccgagatca aaagtccttaaaaggaagaggcaacaccc	-	*
CY064632	Human H1N1pdm2009 IAVs	Human	H1N1		2009	USA	A/Boston/133/2009	ggtgatgccccattccttgatcggctccgccgagatca aaagtccttaaaaggaagaggcaacaccc	-	*
CY089729	Human H1N1pdm2009 IAVs	Human	H1N1	pdm	2009	USA	A/Boston/139/2009	ggtgatgccccattccttgatcggctccgccgagatca aaagtccttaaaaggaagaggcaacaccc	-	*
CY064672	Human H1N1pdm2009 IAVs	Human	H1N1		2009	USA	A/Boston/140/2009	ggtgatgccccattccttgatcggctccgccgagatca aaagtccttaaaaggaagaggcaacaccc	-	*
CY064688	Human H1N1pdm2009 IAVs	Human	H1N1	pdm	2009	USA	A/Boston/142/2009	ggtgatgccccattccttgatcggctccgccgagatca aaagtccttaaaaggaagaggcaacaccc	-	*
CY063530	Human H1N1pdm2009 IAVs	Human	H1N1	pdm	2009	USA	A/Boston/147/2009	ggtgatgccccattccttgatcggctccgccgagatca aaagtccttaaaaggaagaggcaacaccc	-	*
CY075656	Human H1N1pdm2009 IAVs	Human	H1N1	pdm	2009	USA	A/Boston/617/2009	ggtgatgccccattccttgatcggctccgccgagatca aaagtccttaaaaggaagaggcaacaccc	-	*
CY053178	Human H1N1pdm2009 IAVs	Human	H1N1	pdm	2009	USA	A/Brownsville/26OS/2009	ggtgatgccccattccttgatcggctccgccgagatca aaagtccttaaaaggaagaggcaacaccc	-	*
CY053186	Human H1N1pdm2009 IAVs	Human	H1N1	pdm	2009	USA	A/Brownsville/27OS/2009	ggtgatgccccattccttgatcggctccgccgagatca aaagtccttaaaaggaagaggcaacaccc	-	*
CY053234	Human H1N1pdm2009 IAVs	Human	H1N1	pdm	2009	USA	A/Brownsville/34H/2009	ggtgatgccccattccttgatcggctccgccgagatca aaagtccttaaaaggaagaggcaacaccc	-	*
CY053226	Human H1N1pdm2009 IAVs	Human	H1N1	pdm	2009	USA	A/Brownsville/34OS/2009	ggtgatgccccattccttgatcggctccgccgagatca aaagtccttaaaaggaagaggcaacaccc	-	*
CY053250	Human H1N1pdm2009 IAVs	Human	H1N1	pdm	2009	USA	A/Brownsville/35H/2009	ggtgatgccccattccttgatcggctccgccgagatca aaagtccttaaaaggaagaggcaacaccc	-	*
CY053242	Human H1N1pdm2009 IAVs	Human	H1N1		2009	USA	A/Brownsville/35OS/2009	ggtgatgccccattccttgatcggctccgccgagatca aaagtccttaaaaggaagaggcaacaccc	-	*
CY053265	Human H1N1pdm2009 IAVs	Human	H1N1	pdm	2009	USA	A/Brownsville/37H/2009	ggtgatgccccattccttgatcggctccgccgagatca aaagtccttaaaaggaagaggcaacaccc	-	*
CY053313	Human H1N1pdm2009 IAVs	Human	H1N1	pdm	2009	USA	A/Brownsville/37OS/2009	ggtgatgccccattccttgatcggctccgccgagatca aaagtccttaaaaggaagaggcaacaccc	-	*
CY053281	Human H1N1pdm2009 IAVs	Human	H1N1	pdm	2009	USA	A/Brownsville/39H/2009	ggtgatgccccattccttgatcggctccgccgagatca aaagtccttaaaaggaagaggcaacaccc	-	*
CY053273	Human H1N1pdm2009 IAVs	Human	H1N1	pdm	2009	USA	A/Brownsville/39OS/2009	ggtgatgccccattccttgatcggctccgccgagatca aaagtccttaaaaggaagaggcaacaccc	-	*
CY053289	Human H1N1pdm2009 IAVs	Human	H1N1	pdm	2009	USA	A/Brownsville/40OS/2009	ggtgatgccccattccttgatcggctccgccgagatca aaagtccttaaaaggaagaggcaacaccc	-	*
CY053305	Human H1N1pdm2009 IAVs	Human	H1N1	pdm	2009	USA	A/Brownsville/43H/2009	ggtgatgccccattccttgatcggctccgccgagatca aaagtccttaaaaggaagaggcaacaccc	-	*
CY053297	Human H1N1pdm2009 IAVs	Human	H1N1	pdm	2009	USA	A/Brownsville/43OS/2009	ggtgatgccccattccttgatcggctccgccgagatca aaagtccttaaaaggaagaggcaacaccc	-	*
CY181397	Human H1N1pdm2009 IAVs	Human	H1N1	pdm	2009	USA	A/California/07_JRO02/2009	ggtgatgccccattccttgatcggctccgccgagatca aaagtccttaaaaggaagaggcaacaccc	-	*
CY181405	Human H1N1pdm2009 IAVs	Human	H1N1	pdm	2009	USA	A/California/07_JRO03/2009	ggtgatgccccattccttgatcggctccgccgagatca aaagtccttaaaaggaagaggcaacaccc	-	*

CY181413	Human H1N1pdm2009 IAVs	Human	H1N1	pdm	2009	USA	A/California/07_JRO08/2009	ggtgatgccccattccttgatcggctccgccgagatca aaagtccttaaaaggaagaggcaacaccc	-	*
CY181421	Human H1N1pdm2009 IAVs	Human	H1N1	pdm	2009	USA	A/California/07_JRO09/2009	ggtgatgccccattccttgatcggctccgccgagatca aaagtccttaaaaggaagaggcaacaccc	-	*
CY181429	Human H1N1pdm2009 IAVs	Human	H1N1	pdm	2009	USA	A/California/07_JRO10/2009	ggtgatgccccattccttgatcggctccgccgagatca aaagtccttaaaaggaagaggcaacaccc	-	*
CY185082	Human H1N1pdm2009 IAVs	Human	H1N1		2009	NA	A/California/7_KSU_RG52/2009	ggtgatgccccattccttgatcggctccgccgagatca aaagtccttaaaaggaagaggcaacaccc	-	*
CY054775	Human H1N1pdm2009 IAVs	Human	H1N1	pdm	2009	USA	A/California/VRDL21/2009	ggtgatgccccattccttgatcggctccgccgagatca aaagtccttaaaaggaagaggcaacaccc	-	*
CY054847	Human H1N1pdm2009 IAVs	Human	H1N1	pdm	2009	USA	A/California/VRDL31/2009	ggtgatgccccattccttgatcggctccgccgagatca aaagtccttaaaaggaagaggcaacaccc	-	*
CY068926	Human H1N1pdm2009 IAVs	Human	H1N1	pdm	2009	Guam	A/Guam/NHRC0004/2009	ggtgatgccccattccttgatcggctccgccgagatca aaagtccttaaaaggaagaggcaacaccc	-	*
CY053067	Human H1N1pdm2009 IAVs	Human	H1N1	pdm	2009	USA	A/Houston/10H/2009	ggtgatgccccattccttgatcggctccgccgagatca aaagtccttaaaaggaagaggcaacaccc	-	*
CY053075	Human H1N1pdm2009 IAVs	Human	H1N1	pdm	2009	USA	A/Houston/13OS/2009	ggtgatgccccattccttgatcggctccgccgagatca aaagtccttaaaaggaagaggcaacaccc	-	*
CY053091	Human H1N1pdm2009 IAVs	Human	H1N1	pdm	2009	USA	A/Houston/14H/2009	ggtgatgccccattccttgatcggctccgccgagatca aaagtccttaaaaggaagaggcaacaccc	-	*
CY053107	Human H1N1pdm2009 IAVs	Human	H1N1	pdm	2009	USA	A/Houston/15H/2009	ggtgatgccccattccttgatcggctccgccgagatca aaagtccttaaaaggaagaggcaacaccc	-	*
CY053099	Human H1N1pdm2009 IAVs	Human	H1N1	pdm	2009	USA	A/Houston/15OS/2009	ggtgatgccccattccttgatcggctccgccgagatca aaagtccttaaaaggaagaggcaacaccc	-	*
CY051907	Human H1N1pdm2009 IAVs	Human	H1N1	pdm	2009	USA	A/Houston/1OS/2009	ggtgatgccccattccttgatcggctccgccgagatca aaagtccttaaaaggaagaggcaacaccc	-	*
CY053139	Human H1N1pdm2009 IAVs	Human	H1N1	pdm	2009	USA	A/Houston/20H/2009	ggtgatgccccattccttgatcggctccgccgagatca aaagtccttaaaaggaagaggcaacaccc	-	*
CY053131	Human H1N1pdm2009 IAVs	Human	H1N1	pdm	2009	USA	A/Houston/20OS/2009	ggtgatgccccattccttgatcggctccgccgagatca aaagtccttaaaaggaagaggcaacaccc	-	*
CY051915	Human H1N1pdm2009 IAVs	Human	H1N1	pdm	2009	USA	A/Houston/2H/2009	ggtgatgccccattccttgatcggctccgccgagatca aaagtccttaaaaggaagaggcaacaccc	-	*
CY052979	Human H1N1pdm2009 IAVs	Human	H1N1	pdm	2009	USA	A/Houston/2OS/2009	ggtgatgccccattccttgatcggctccgccgagatca aaagtccttaaaaggaagaggcaacaccc	-	*
CY052971	Human H1N1pdm2009 IAVs	Human	H1N1	pdm	2009	USA	A/Houston/2OS/2009	ggtgatgccccattccttgatcggctccgccgagatca aaagtccttaaaaggaagaggcaacaccc	-	*
CY052995	Human H1N1pdm2009 IAVs	Human	H1N1	pdm	2009	USA	A/Houston/3H/2009	ggtgatgccccattccttgatcggctccgccgagatca aaagtccttaaaaggaagaggcaacaccc	-	*
CY052987	Human H1N1pdm2009 IAVs	Human	H1N1	pdm	2009	USA	A/Houston/3OS/2009	ggtgatgccccattccttgatcggctccgccgagatca aaagtccttaaaaggaagaggcaacaccc	-	*
CY053011	Human H1N1pdm2009 IAVs	Human	H1N1	pdm	2009	USA	A/Houston/5H/2009	ggtgatgccccattccttgatcggctccgccgagatca aaagtccttaaaaggaagaggcaacaccc	-	*
CY053003	Human H1N1pdm2009 IAVs	Human	H1N1	pdm	2009	USA	A/Houston/5OS/2009	ggtgatgccccattccttgatcggctccgccgagatca aaagtccttaaaaggaagaggcaacaccc	-	*
CY053027	Human H1N1pdm2009 IAVs	Human	H1N1	pdm	2009	USA	A/Houston/6H/2009	ggtgatgccccattccttgatcggctccgccgagatca aaagtccttaaaaggaagaggcaacaccc	-	*
CY053019	Human H1N1pdm2009 IAVs	Human	H1N1	pdm	2009	USA	A/Houston/6OS/2009	ggtgatgccccattccttgatcggctccgccgagatca aaagtccttaaaaggaagaggcaacaccc	-	*
CY053043	Human H1N1pdm2009 IAVs	Human	H1N1	pdm	2009	USA	A/Houston/7H/2009	ggtgatgccccattccttgatcggctccgccgagatca aaagtccttaaaaggaagaggcaacaccc	-	*

CY051923	Human H1N1pdm2009 IAVs	Human	H1N1	pdm	2009	USA	A/Houston/9OS/2009	ggtgatgccccattccttgatcggctccgccgagatca aaagtccttaaaaggaagaggcaacaccc	-	*
CY057218	Human H1N1pdm2009 IAVs	Human	H1N1	pdm	2009	Nicaragua	A/Managua/109.01/2009	ggtgatgccccattccttgatcggctccgccgagatca aaagtccttaaaaggaagaggcaacaccc	-	*
CY073613	Human H1N1pdm2009 IAVs	Human	H1N1	pdm	2009	Nicaragua	A/Managua/1256.04/2009	ggtgatgccccattccttgatcggctccgccgagatca aaagtccttaaaaggaagaggcaacaccc	-	*
CY058088	Human H1N1pdm2009 IAVs	Human	H1N1	pdm	2009	Nicaragua	A/Managua/1455.01/2009	ggtgatgccccattccttgatcggctccgccgagatca aaagtccttaaaaggaagaggcaacaccc	-	*
CY058120	Human H1N1pdm2009 IAVs	Human	H1N1	pdm	2009	Nicaragua	A/Managua/1637.01/2009	ggtgatgccccattccttgatcggctccgccgagatca aaagtccttaaaaggaagaggcaacaccc	-	*
CY058080	Human H1N1pdm2009 IAVs	Human	H1N1	pdm	2009	Nicaragua	A/Managua/171.01/2009	ggtgatgccccattccttgatcggctccgccgagatca aaagtccttaaaaggaagaggcaacaccc	-	*
CY072682	Human H1N1pdm2009 IAVs	Human	H1N1		2009	Nicaragua	A/Managua/209.01/2009	ggtgatgccccattccttgatcggctccgccgagatca aaagtccttaaaaggaagaggcaacaccc	-	*
CY072650	Human H1N1pdm2009 IAVs	Human	H1N1	pdm	2009	Nicaragua	A/Managua/2275.01/2009	ggtgatgccccattccttgatcggctccgccgagatca aaagtccttaaaaggaagaggcaacaccc	-	*
CY058160	Human H1N1pdm2009 IAVs	Human	H1N1	pdm	2009	Nicaragua	A/Managua/2330.02/2009	ggtgatgccccattccttgatcggctccgccgagatca aaagtccttaaaaggaagaggcaacaccc	-	*
CY073889	Human H1N1pdm2009 IAVs	Human	H1N1	pdm	2009	Nicaragua	A/Managua/2729.02/2009	ggtgatgccccattccttgatcggctccgccgagatca aaagtccttaaaaggaagaggcaacaccc	-	*
CY072874	Human H1N1pdm2009 IAVs	Human	H1N1	pdm	2009	Nicaragua	A/Managua/2999.03/2009	ggtgatgccccattccttgatcggctccgccgagatca aaagtccttaaaaggaagaggcaacaccc	-	*
CY056391	Human H1N1pdm2009 IAVs	Human	H1N1	pdm	2009	Nicaragua	A/Managua/3119.01/2009	ggtgatgccccattccttgatcggctccgccgagatca aaagtccttaaaaggaagaggcaacaccc	-	*
CY072906	Human H1N1pdm2009 IAVs	Human	H1N1	pdm	2009	Nicaragua	A/Managua/3434.02/2009	ggtgatgccccattccttgatcggctccgccgagatca aaagtccttaaaaggaagaggcaacaccc	-	*
FJ985767	Human H1N1pdm2009 IAVs	Human	H1N1	pdm	2009	Spain	A/Castilla_La_Mancha/GP9/2009	ggtgatgccccattccttgatcggctccgccgagatca aaagtccttaaaaggaagaggcaacaccc	-	*
GQ166657	Human H1N1pdm2009 IAVs	Human	H1N1	pdm	2009	United_Kingdom	A/England/195/2009	ggtgatgccccattccttgatcggctccgccgagatca aaagtccttaaaaggaagaggcaacaccc	-	*
CY072858	Human H1N1pdm2009 IAVs	Human	H1N1	pdm	2009	Nicaragua	A/Managua/3438.02/2009	ggtgatgccccattccttgatcggctccgccgagatca aaagtccttaaaaggaagaggcaacaccc	-	*
CY073701	Human H1N1pdm2009 IAVs	Human	H1N1	pdm	2009	Nicaragua	A/Managua/3490.03/2009	ggtgatgccccattccttgatcggctccgccgagatca aaagtccttaaaaggaagaggcaacaccc	-	*
CY072978	Human H1N1pdm2009 IAVs	Human	H1N1	pdm	2009	Nicaragua	A/Managua/3507.01/2009	ggtgatgccccattccttgatcggctccgccgagatca aaagtccttaaaaggaagaggcaacaccc	-	*
CY072642	Human H1N1pdm2009 IAVs	Human	H1N1		2009	Nicaragua	A/Managua/3642.04/2009	ggtgatgccccattccttgatcggctccgccgagatca aaagtccttaaaaggaagaggcaacaccc	-	*
CY073541	Human H1N1pdm2009 IAVs	Human	H1N1	pdm	2009	Nicaragua	A/Managua/4012.01/2009	ggtgatgccccattccttgatcggctccgccgagatca aaagtccttaaaaggaagaggcaacaccc	-	*
CY073589	Human H1N1pdm2009 IAVs	Human	H1N1	pdm	2009	Nicaragua	A/Managua/4064.01/2009	ggtgatgccccattccttgatcggctccgccgagatca aaagtccttaaaaggaagaggcaacaccc	-	*
CY073653	Human H1N1pdm2009 IAVs	Human	H1N1	pdm	2009	Nicaragua	A/Managua/4590.03/2009	ggtgatgccccattccttgatcggctccgccgagatca aaagtccttaaaaggaagaggcaacaccc	-	*
CY072882	Human H1N1pdm2009 IAVs	Human	H1N1		2009	Nicaragua	A/Managua/4606.06/2009	ggtgatgccccattccttgatcggctccgccgagatca aaagtccttaaaaggaagaggcaacaccc	-	*
CY058240	Human H1N1pdm2009 IAVs	Human	H1N1	pdm	2009	Nicaragua	A/Managua/473.02/2009	ggtgatgccccattccttgatcggctccgccgagatca aaagtccttaaaaggaagaggcaacaccc	-	*
CY073685	Human H1N1pdm2009 IAVs	Human	H1N1	pdm	2009	Nicaragua	A/Managua/4736.02/2009	ggtgatgccccattccttgatcggctccgccgagatca aaagtccttaaaaggaagaggcaacaccc	-	*

CY073621	Human H1N1pdm2009 IAVs	Human	H1N1	pdm	2009	Nicaragua	A/Managua/4747.01/2009	ggtgatgccccattccttgatcggctccgccgagatca aaagtccttaaaaggaagaggcaacaccc	-	*
CY069294	Human H1N1pdm2009 IAVs	Human	H1N1	pdm	2009	Nicaragua	A/Managua/4951.02/2009	ggtgatgccccattccttgatcggctccgccgagatca aaagtccttaaaaggaagaggcaacaccc	-	*
CY073565	Human H1N1pdm2009 IAVs	Human	H1N1	pdm	2009	Nicaragua	A/Managua/5119.06/2009	ggtgatgccccattccttgatcggctccgccgagatca aaagtccttaaaaggaagaggcaacaccc	-	*
CY072754	Human H1N1pdm2009 IAVs	Human	H1N1	pdm	2009	Nicaragua	A/Managua/5227.04/2009	ggtgatgccccattccttgatcggctccgccgagatca aaagtccttaaaaggaagaggcaacaccc	-	*
CY073573	Human H1N1pdm2009 IAVs	Human	H1N1	pdm	2009	Nicaragua	A/Managua/5230.01/2009	ggtgatgccccattccttgatcggctccgccgagatca aaagtccttaaaaggaagaggcaacaccc	-	*
CY072994	Human H1N1pdm2009 IAVs	Human	H1N1	pdm	2009	Nicaragua	A/Managua/527.04/2009	ggtgatgccccattccttgatcggctccgccgagatca aaagtccttaaaaggaagaggcaacaccc	-	*
CY073525	Human H1N1pdm2009 IAVs	Human	H1N1	pdm	2009	Nicaragua	A/Managua/5399.04/2009	ggtgatgccccattccttgatcggctccgccgagatca aaagtccttaaaaggaagaggcaacaccc	-	*
CY058192	Human H1N1pdm2009 IAVs	Human	H1N1		2009	Nicaragua	A/Managua/5586.01/2009	ggtgatgccccattccttgatcggctccgccgagatca aaagtccttaaaaggaagaggcaacaccc	-	*
CY072938	Human H1N1pdm2009 IAVs	Human	H1N1	pdm	2009	Nicaragua	A/Managua/5590.02/2009	ggtgatgccccattccttgatcggctccgccgagatca aaagtccttaaaaggaagaggcaacaccc	-	*
CY072810	Human H1N1pdm2009 IAVs	Human	H1N1	pdm	2009	Nicaragua	A/Managua/572.02/2009	ggtgatgccccattccttgatcggctccgccgagatca aaagtccttaaaaggaagaggcaacaccc	-	*
CY058216	Human H1N1pdm2009 IAVs	Human	H1N1	pdm	2009	Nicaragua	A/Managua/578.01/2009	ggtgatgccccattccttgatcggctccgccgagatca aaagtccttaaaaggaagaggcaacaccc	-	*
CY058176	Human H1N1pdm2009 IAVs	Human	H1N1	pdm	2009	Nicaragua	A/Managua/792.01/2009	ggtgatgccccattccttgatcggctccgccgagatca aaagtccttaaaaggaagaggcaacaccc	-	*
CY073581	Human H1N1pdm2009 IAVs	Human	H1N1	pdm	2009	Nicaragua	A/Managua/921.02/2009	ggtgatgccccattccttgatcggctccgccgagatca aaagtccttaaaaggaagaggcaacaccc	-	*
CY050883	Human H1N1pdm2009 IAVs	Human	H1N1	pdm	2009	Mexico	A/Mexico_City/005/2009	ggtgatgccccattccttgatcggctccgccgagatca aaagtccttaaaaggaagaggcaacaccc	-	*
CY050170	Human H1N1pdm2009 IAVs	Human	H1N1	pdm	2009	Mexico	A/Mexico_City/008/2009	ggtgatgccccattccttgatcggctccgccgagatca aaagtccttaaaaggaagaggcaacaccc	-	*
CY050859	Human H1N1pdm2009 IAVs	Human	H1N1	pdm	2009	Mexico	A/Mexico_City/009/2009	ggtgatgccccattccttgatcggctccgccgagatca aaagtccttaaaaggaagaggcaacaccc	-	*
CY050250	Human H1N1pdm2009 IAVs	Human	H1N1	pdm	2009	Mexico	A/Mexico_City/011/2009	ggtgatgccccattccttgatcggctccgccgagatca aaagtccttaaaaggaagaggcaacaccc	-	*
CY050867	Human H1N1pdm2009 IAVs	Human	H1N1	pdm	2009	Mexico	A/Mexico_City/013/2009	ggtgatgccccattccttgatcggctccgccgagatca aaagtccttaaaaggaagaggcaacaccc	-	*
CY062639	Human H1N1pdm2009 IAVs	Human	H1N1	pdm	2009	Mexico	A/Mexico_City/014/2009	ggtgatgccccattccttgatcggctccgccgagatca aaagtccttaaaaggaagaggcaacaccc	-	*
CY050891	Human H1N1pdm2009 IAVs	Human	H1N1	pdm	2009	Mexico	A/Mexico_City/018/2009	ggtgatgccccattccttgatcggctccgccgagatca aaagtccttaaaaggaagaggcaacaccc	-	*
CY064712	Human H1N1pdm2009 IAVs	Human	H1N1		2009	Mexico	A/Mexico_City/020/2009	ggtgatgccccattccttgatcggctccgccgagatca aaagtccttaaaaggaagaggcaacaccc	-	*
CY064728	Human H1N1pdm2009 IAVs	Human	H1N1	pdm	2009	Mexico	A/Mexico_City/022/2009	ggtgatgccccattccttgatcggctccgccgagatca aaagtccttaaaaggaagaggcaacaccc	-	*
CY064736	Human H1N1pdm2009 IAVs	Human	H1N1	pdm	2009	Mexico	A/Mexico_City/023/2009	ggtgatgccccattccttgatcggctccgccgagatca aaagtccttaaaaggaagaggcaacaccc	-	*
CY064752	Human H1N1pdm2009 IAVs	Human	H1N1		2009	Mexico	A/Mexico_City/025/2009	ggtgatgccccattccttgatcggctccgccgagatca aaagtccttaaaaggaagaggcaacaccc	-	*
CY064760	Human H1N1pdm2009 IAVs	Human	H1N1	pdm	2009	Mexico	A/Mexico_City/026/2009	ggtgatgccccattccttgatcggctccgccgagatca aaagtccttaaaaggaagaggcaacaccc	-	*

CY062513	Human H1N1pdm2009 IAVs	Human	H1N1	pdm	2009	Mexico	A/Mexico_city/CIA4/2009	ggtgatgccccattccttgatcggctccgccgagatca aaagtccttaaaaggaagaggcaacaccc	-	*
CY147679	Human H1N1pdm2009 IAVs	Human	H1N1	pdm	2009	Mexico	A/Mexico/24015/2009	ggtgatgccccattccttgatcggctccgccgagatca aaagtccttaaaaggaagaggcaacaccc	-	*
CY147703	Human H1N1pdm2009 IAVs	Human	H1N1	pdm	2009	Mexico	A/Mexico/24021/2009	ggtgatgccccattccttgatcggctccgccgagatca aaagtccttaaaaggaagaggcaacaccc	-	*
CY147735	Human H1N1pdm2009 IAVs	Human	H1N1	pdm	2009	Mexico	A/Mexico/24026/2009	ggtgatgccccattccttgatcggctccgccgagatca aaagtccttaaaaggaagaggcaacaccc	-	*
CY147743	Human H1N1pdm2009 IAVs	Human	H1N1	pdm	2009	Mexico	A/Mexico/24027/2009	ggtgatgccccattccttgatcggctccgccgagatca aaagtccttaaaaggaagaggcaacaccc	-	*
CY147751	Human H1N1pdm2009 IAVs	Human	H1N1	pdm	2009	Mexico	A/Mexico/24028/2009	ggtgatgccccattccttgatcggctccgccgagatca aaagtccttaaaaggaagaggcaacaccc	-	*
CY147554	Human H1N1pdm2009 IAVs	Human	H1N1	pdm	2009	Mexico	A/Mexico/24029/2009	ggtgatgccccattccttgatcggctccgccgagatca aaagtccttaaaaggaagaggcaacaccc	-	*
CY147759	Human H1N1pdm2009 IAVs	Human	H1N1	pdm	2009	Mexico	A/Mexico/24030/2009	ggtgatgccccattccttgatcggctccgccgagatca aaagtccttaaaaggaagaggcaacaccc	-	*
CY147767	Human H1N1pdm2009 IAVs	Human	H1N1	pdm	2009	Mexico	A/Mexico/24032/2009	ggtgatgccccattccttgatcggctccgccgagatca aaagtccttaaaaggaagaggcaacaccc	-	*
CY147775	Human H1N1pdm2009 IAVs	Human	H1N1	pdm	2009	Mexico	A/Mexico/24033/2009	ggtgatgccccattccttgatcggctccgccgagatca aaagtccttaaaaggaagaggcaacaccc	-	*
CY147783	Human H1N1pdm2009 IAVs	Human	H1N1	pdm	2009	Mexico	A/Mexico/24036/2009	ggtgatgccccattccttgatcggctccgccgagatca aaagtccttaaaaggaagaggcaacaccc	-	*
CY147823	Human H1N1pdm2009 IAVs	Human	H1N1	pdm	2009	Mexico	A/Mexico/24043/2009	ggtgatgccccattccttgatcggctccgccgagatca aaagtccttaaaaggaagaggcaacaccc	-	*
CY147831	Human H1N1pdm2009 IAVs	Human	H1N1	pdm	2009	Mexico	A/Mexico/24044/2009	ggtgatgccccattccttgatcggctccgccgagatca aaagtccttaaaaggaagaggcaacaccc	-	*
CY147839	Human H1N1pdm2009 IAVs	Human	H1N1	pdm	2009	Mexico	A/Mexico/24045/2009	ggtgatgccccattccttgatcggctccgccgagatca aaagtccttaaaaggaagaggcaacaccc	-	*
CY147855	Human H1N1pdm2009 IAVs	Human	H1N1	pdm	2009	Mexico	A/Mexico/24048/2009	ggtgatgccccattccttgatcggctccgccgagatca aaagtccttaaaaggaagaggcaacaccc	-	*
CY147871	Human H1N1pdm2009 IAVs	Human	H1N1	pdm	2009	Mexico	A/Mexico/24050/2009	ggtgatgccccattccttgatcggctccgccgagatca aaagtccttaaaaggaagaggcaacaccc	-	*
CY147895	Human H1N1pdm2009 IAVs	Human	H1N1	pdm	2009	Mexico	A/Mexico/24053/2009	ggtgatgccccattccttgatcggctccgccgagatca aaagtccttaaaaggaagaggcaacaccc	-	*
CY147903	Human H1N1pdm2009 IAVs	Human	H1N1	pdm	2009	Mexico	A/Mexico/24054/2009	ggtgatgccccattccttgatcggctccgccgagatca aaagtccttaaaaggaagaggcaacaccc	-	*
CY147919	Human H1N1pdm2009 IAVs	Human	H1N1	pdm	2009	Mexico	A/Mexico/24056/2009	ggtgatgccccattccttgatcggctccgccgagatca aaagtccttaaaaggaagaggcaacaccc	-	*
CY147927	Human H1N1pdm2009 IAVs	Human	H1N1	pdm	2009	Mexico	A/Mexico/24057/2009	ggtgatgccccattccttgatcggctccgccgagatca aaagtccttaaaaggaagaggcaacaccc	-	*
CY147935	Human H1N1pdm2009 IAVs	Human	H1N1	pdm	2009	Mexico	A/Mexico/24058/2009	ggtgatgccccattccttgatcggctccgccgagatca aaagtccttaaaaggaagaggcaacaccc	-	*
CY147943	Human H1N1pdm2009 IAVs	Human	H1N1	pdm	2009	Mexico	A/Mexico/24060/2009	ggtgatgccccattccttgatcggctccgccgagatca aaagtccttaaaaggaagaggcaacaccc	-	*
CY148023	Human H1N1pdm2009 IAVs	Human	H1N1	pdm	2009	Netherlands	A/Netherlands/602/2009	ggtgatgccccattccttgatcggctccgccgagatca aaagtccttaaaaggaagaggcaacaccc	-	*
CY148127	Human H1N1pdm2009 IAVs	Human	H1N1	pdm	2009	Netherlands	A/Netherlands/602/2009	ggtgatgccccattccttgatcggctccgccgagatca aaagtccttaaaaggaagaggcaacaccc	-	*
CY148055	Human H1N1pdm2009 IAVs	Human	H1N1	pdm	2009	Netherlands	A/Netherlands/602/2009	ggtgatgccccattccttgatcggctccgccgagatca aaagtccttaaaaggaagaggcaacaccc	-	*

CY148143	Human H1N1pdm2009 IAVs	Human	H1N1	pdm	2009	Netherlands	A/Netherlands/602/2009	ggtgatgccccattccttgatcggctccgccgagatca aaagtccttaaaaggaagaggcaacaccc	-	*
CY148151	Human H1N1pdm2009 IAVs	Human	H1N1	pdm	2009	Netherlands	A/Netherlands/602/2009	ggtgatgccccattccttgatcggctccgccgagatca aaagtccttaaaaggaagaggcaacaccc	-	*
CY148031	Human H1N1pdm2009 IAVs	Human	H1N1	pdm	2009	Netherlands	A/Netherlands/602/2009	ggtgatgccccattccttgatcggctccgccgagatca aaagtccttaaaaggaagaggcaacaccc	-	*
CY148231	Human H1N1pdm2009 IAVs	Human	H1N1	pdm	2009	Netherlands	A/Netherlands/602/2009	ggtgatgccccattccttgatcggctccgccgagatca aaagtccttaaaaggaagaggcaacaccc	-	*
CY148047	Human H1N1pdm2009 IAVs	Human	H1N1	pdm	2009	Netherlands	A/Netherlands/602/2009	ggtgatgccccattccttgatcggctccgccgagatca aaagtccttaaaaggaagaggcaacaccc	-	*
CY148239	Human H1N1pdm2009 IAVs	Human	H1N1	pdm	2009	Netherlands	A/Netherlands/602/2009	ggtgatgccccattccttgatcggctccgccgagatca aaagtccttaaaaggaagaggcaacaccc	-	*
CY148135	Human H1N1pdm2009 IAVs	Human	H1N1	pdm	2009	Netherlands	A/Netherlands/602/2009	ggtgatgccccattccttgatcggctccgccgagatca aaagtccttaaaaggaagaggcaacaccc	-	*
CY148183	Human H1N1pdm2009 IAVs	Human	H1N1	pdm	2009	Netherlands	A/Netherlands/602/2009	ggtgatgccccattccttgatcggctccgccgagatca aaagtccttaaaaggaagaggcaacaccc	-	*
CY148191	Human H1N1pdm2009 IAVs	Human	H1N1	pdm	2009	Netherlands	A/Netherlands/602/2009	ggtgatgccccattccttgatcggctccgccgagatca aaagtccttaaaaggaagaggcaacaccc	-	*
CY148199	Human H1N1pdm2009 IAVs	Human	H1N1	pdm	2009	Netherlands	A/Netherlands/602/2009	ggtgatgccccattccttgatcggctccgccgagatca aaagtccttaaaaggaagaggcaacaccc	-	*
CY148111	Human H1N1pdm2009 IAVs	Human	H1N1	pdm	2009	Netherlands	A/Netherlands/602/2009	ggtgatgccccattccttgatcggctccgccgagatca aaagtccttaaaaggaagaggcaacaccc	-	*
CY148015	Human H1N1pdm2009 IAVs	Human	H1N1	pdm	2009	Netherlands	A/Netherlands/602/2009	ggtgatgccccattccttgatcggctccgccgagatca aaagtccttaaaaggaagaggcaacaccc	-	*
CY039897	Human H1N1pdm2009 IAVs	Human	H1N1	pdm	2009	USA	A/New_York/1669/2009	ggtgatgccccattccttgatcggctccgccgagatca aaagtccttaaaaggaagaggcaacaccc	-	*
CY041818	Human H1N1pdm2009 IAVs	Human	H1N1	pdm	2009	USA	A/New_York/3351/2009	ggtgatgccccattccttgatcggctccgccgagatca aaagtccttaaaaggaagaggcaacaccc	-	*
CY041826	Human H1N1pdm2009 IAVs	Human	H1N1	pdm	2009	USA	A/New_York/3352/2009	ggtgatgccccattccttgatcggctccgccgagatca aaagtccttaaaaggaagaggcaacaccc	-	*
CY043167	Human H1N1pdm2009 IAVs	Human	H1N1	pdm	2009	USA	A/New_York/3454/2009	ggtgatgccccattccttgatcggctccgccgagatca aaagtccttaaaaggaagaggcaacaccc	-	*
CY043223	Human H1N1pdm2009 IAVs	Human	H1N1	pdm	2009	USA	A/New_York/3455/2009	ggtgatgccccattccttgatcggctccgccgagatca aaagtccttaaaaggaagaggcaacaccc	-	*
CY043303	Human H1N1pdm2009 IAVs	Human	H1N1	pdm	2009	USA	A/New_York/3498/2009	ggtgatgccccattccttgatcggctccgccgagatca aaagtccttaaaaggaagaggcaacaccc	-	*
CY043191	Human H1N1pdm2009 IAVs	Human	H1N1	pdm	2009	USA	A/New_York/3499/2009	ggtgatgccccattccttgatcggctccgccgagatca aaagtccttaaaaggaagaggcaacaccc	-	*
CY045089	Human H1N1pdm2009 IAVs	Human	H1N1	pdm	2009	USA	A/New_York/3574/2009	ggtgatgccccattccttgatcggctccgccgagatca aaagtccttaaaaggaagaggcaacaccc	-	*
CY045147	Human H1N1pdm2009 IAVs	Human	H1N1	pdm	2009	USA	A/New_York/3577/2009	ggtgatgccccattccttgatcggctccgccgagatca aaagtccttaaaaggaagaggcaacaccc	-	*
CY044124	Human H1N1pdm2009 IAVs	Human	H1N1	pdm	2009	USA	A/New_York/4197/2009	ggtgatgccccattccttgatcggctccgccgagatca aaagtccttaaaaggaagaggcaacaccc	-	*
CY051555	Human H1N1pdm2009 IAVs	Human	H1N1	pdm	2009	USA	A/New_York/4434/2009	ggtgatgccccattccttgatcggctccgccgagatca aaagtccttaaaaggaagaggcaacaccc	-	*
CY051595	Human H1N1pdm2009 IAVs	Human	H1N1	pdm	2009	USA	A/New_York/4565/2009	ggtgatgccccattccttgatcggctccgccgagatca aaagtccttaaaaggaagaggcaacaccc	-	*
CY051651	Human H1N1pdm2009 IAVs	Human	H1N1	pdm	2009	USA	A/New_York/4691/2009	ggtgatgccccattccttgatcggctccgccgagatca aaagtccttaaaaggaagaggcaacaccc	-	*

CY051667	Human H1N1pdm2009 IAVs	Human	H1N1	pdm	2009	USA	A/New_York/4735/2009	ggtgatgccccattccttgatcggctccgccgagatca aaagtccttaaaaggaagaggcaacaccc	-	*
CY052302	Human H1N1pdm2009 IAVs	Human	H1N1		2009	USA	A/Texas/42103399/2009	ggtgatgccccattccttgatcggctccgccgagatca aaagtccttaaaaggaagaggcaacaccc	-	*
CY051883	Human H1N1pdm2009 IAVs	Human	H1N1	pdm	2009	USA	A/Texas/42113095/2009	ggtgatgccccattccttgatcggctccgccgagatca aaagtccttaaaaggaagaggcaacaccc	-	*
CY052278	Human H1N1pdm2009 IAVs	Human	H1N1	pdm	2009	USA	A/Texas/42151049/2009	ggtgatgccccattccttgatcggctccgccgagatca aaagtccttaaaaggaagaggcaacaccc	-	*
CY052206	Human H1N1pdm2009 IAVs	Human	H1N1	pdm	2009	USA	A/Texas/42303371/2009	ggtgatgccccattccttgatcggctccgccgagatca aaagtccttaaaaggaagaggcaacaccc	-	*
CY052907	Human H1N1pdm2009 IAVs	Human	H1N1	pdm	2009	USA	A/Texas/45032708/2009	ggtgatgccccattccttgatcggctccgccgagatca aaagtccttaaaaggaagaggcaacaccc	-	*
CY052939	Human H1N1pdm2009 IAVs	Human	H1N1	pdm	2009	USA	A/Texas/45032753/2009	ggtgatgccccattccttgatcggctccgccgagatca aaagtccttaaaaggaagaggcaacaccc	-	*
CY052539	Human H1N1pdm2009 IAVs	Human	H1N1	pdm	2009	USA	A/Texas/45113882/2009	ggtgatgccccattccttgatcggctccgccgagatca aaagtccttaaaaggaagaggcaacaccc	-	*
CY052435	Human H1N1pdm2009 IAVs	Human	H1N1	pdm	2009	USA	A/Texas/45120922/2009	ggtgatgccccattccttgatcggctccgccgagatca aaagtccttaaaaggaagaggcaacaccc	-	*
CY052483	Human H1N1pdm2009 IAVs	Human	H1N1	pdm	2009	USA	A/Texas/45122282/2009	ggtgatgccccattccttgatcggctccgccgagatca aaagtccttaaaaggaagaggcaacaccc	-	*
CY052451	Human H1N1pdm2009 IAVs	Human	H1N1	pdm	2009	USA	A/Texas/45130742/2009	ggtgatgccccattccttgatcggctccgccgagatca aaagtccttaaaaggaagaggcaacaccc	-	*
CY052571	Human H1N1pdm2009 IAVs	Human	H1N1	pdm	2009	USA	A/Texas/45132788/2009	ggtgatgccccattccttgatcggctccgccgagatca aaagtccttaaaaggaagaggcaacaccc	-	*
CY060767	Human H1N1pdm2009 IAVs	Human	H1N1	pdm	2009	USA	A/Texas/46201823/2009	ggtgatgccccattccttgatcggctccgccgagatca aaagtccttaaaaggaagaggcaacaccc	-	*
CY046479	Human H1N1pdm2009 IAVs	Human	H1N1	pdm	2009	USA	A/Wisconsin/629_D00015/2009	ggtgatgccccattccttgatcggctccgccgagatca aaagtccttaaaaggaagaggcaacaccc	-	*
CY051027	Human H1N1pdm2009 IAVs	Human	H1N1	pdm	2009	USA	A/Wisconsin/629_D00017/2009	ggtgatgccccattccttgatcggctccgccgagatca aaagtccttaaaaggaagaggcaacaccc	-	*
CY046671	Human H1N1pdm2009 IAVs	Human	H1N1	pdm	2009	USA	A/Wisconsin/629_D00064/2009	ggtgatgccccattccttgatcggctccgccgagatca aaagtccttaaaaggaagaggcaacaccc	-	*
CY046743	Human H1N1pdm2009 IAVs	Human	H1N1	pdm	2009	USA	A/Wisconsin/629_D00084/2009	ggtgatgccccattccttgatcggctccgccgagatca aaagtccttaaaaggaagaggcaacaccc	-	*
CY089916	Human H1N1pdm2009 IAVs	Human	H1N1	pdm	2009	USA	A/Wisconsin/629_D00112/2009	ggtgatgccccattccttgatcggctccgccgagatca aaagtccttaaaaggaagaggcaacaccc	-	*
CY046759	Human H1N1pdm2009 IAVs	Human	H1N1	pdm	2009	USA	A/Wisconsin/629_D00160/2009	ggtgatgccccattccttgatcggctccgccgagatca aaagtccttaaaaggaagaggcaacaccc	-	*
CY046551	Human H1N1pdm2009 IAVs	Human	H1N1	pdm	2009	USA	A/Wisconsin/629_D00179/2009	ggtgatgccccattccttgatcggctccgccgagatca aaagtccttaaaaggaagaggcaacaccc	-	*
CY089705	Human H1N1pdm2009 IAVs	Human	H1N1	pdm	2009	USA	A/New_York/3750/2009	ggtgatgccccattccttgatcggctccgccgagatca aaagtccttaaaaggaagaggcaacaccc	-	*
KC833453	Human H1N1pdm2009 IAVs	Swine	H1N1		2009	Thailand	A/swine/Thailand/UD400/2009	ggtgatgccccattccttgatcggctccgccgagatca aaagtccttaaaaggaagaggcaacaccc	-	*
GU433029	Human H1N1pdm2009 IAVs	Human	H1N1	pdm	2009	Russia	A/Tomsk/03/2009	ggtgatgccccattccttgatcggctccgccgagatca aaagtccttaaaaggaagaggcaacaccc	-	*
FJ985757	Human H1N1pdm2009 IAVs	Human	H1N1	pdm	2009	Spain	A/Valencia/GP4/2009	ggtgatgccccattccttgatcggctccgccgagatca aaagtccttaaaaggaagaggcaacaccc	-	*
GU211223	Human H1N1pdm2009 IAVs	Human	H1N1	pdm	2009	Russia	A/Vladivostok/01/2009	ggtgatgccccattccttgatcggctccgccgagatca aaagtccttaaaaggaagaggcaacaccc	-	*

GQ496145	Human H1N1pdm2009 IAVs	Human	H1N1	pdm	2009	Russia	A/Vladivostok/IIV17/2009	ggtgatgccccattccttgatcggctccgccgagatca aaagtccttaaaaggaagaggcaacaccc	-	*
GQ496146	Human H1N1pdm2009 IAVs	Human	H1N1	pdm	2009	Russia	A/Vladivostok/IIV18/2009	ggtgatgccccattccttgatcggctccgccgagatca aaagtccttaaaaggaagaggcaacaccc	-	*
CY046807	Human H1N1pdm2009 IAVs	Human	H1N1	pdm	2009	USA	A/Wisconsin/629_D00223/2009	ggtgatgccccattccttgatcggctccgccgagatca aaagtccttaaaaggaagaggcaacaccc	-	*
CY046855	Human H1N1pdm2009 IAVs	Human	H1N1	pdm	2009	USA	A/Wisconsin/629_D00244/2009	ggtgatgccccattccttgatcggctccgccgagatca aaagtccttaaaaggaagaggcaacaccc	-	*
CY050979	Human H1N1pdm2009 IAVs	Human	H1N1	pdm	2009	USA	A/Wisconsin/629_D00367/2009	ggtgatgccccattccttgatcggctccgccgagatca aaagtccttaaaaggaagaggcaacaccc	-	*
CY046879	Human H1N1pdm2009 IAVs	Human	H1N1	pdm	2009	USA	A/Wisconsin/629_D00396/2009	ggtgatgccccattccttgatcggctccgccgagatca aaagtccttaaaaggaagaggcaacaccc	-	*
CY050931	Human H1N1pdm2009 IAVs	Human	H1N1	pdm	2009	USA	A/Wisconsin/629_D00426/2009	ggtgatgccccattccttgatcggctccgccgagatca aaagtccttaaaaggaagaggcaacaccc	-	*
CY046415	Human H1N1pdm2009 IAVs	Human	H1N1	pdm	2009	USA	A/Wisconsin/629_D00447/2009	ggtgatgccccattccttgatcggctccgccgagatca aaagtccttaaaaggaagaggcaacaccc	-	*
CY050408	Human H1N1pdm2009 IAVs	Human	H1N1	pdm	2009	USA	A/Wisconsin/629_D00451/2009	ggtgatgccccattccttgatcggctccgccgagatca aaagtccttaaaaggaagaggcaacaccc	-	*
CY051019	Human H1N1pdm2009 IAVs	Human	H1N1	pdm	2009	USA	A/Wisconsin/629_D00453/2009	ggtgatgccccattccttgatcggctccgccgagatca aaagtccttaaaaggaagaggcaacaccc	-	*
CY051195	Human H1N1pdm2009 IAVs	Human	H1N1	pdm	2009	USA	A/Wisconsin/629_D00496/2009	ggtgatgccccattccttgatcggctccgccgagatca aaagtccttaaaaggaagaggcaacaccc	-	*
CY051163	Human H1N1pdm2009 IAVs	Human	H1N1	pdm	2009	USA	A/Wisconsin/629_D00541/2009	ggtgatgccccattccttgatcggctccgccgagatca aaagtccttaaaaggaagaggcaacaccc	-	*
CY051035	Human H1N1pdm2009 IAVs	Human	H1N1	pdm	2009	USA	A/Wisconsin/629_D00658/2009	ggtgatgccccattccttgatcggctccgccgagatca aaagtccttaaaaggaagaggcaacaccc	-	*
CY051003	Human H1N1pdm2009 IAVs	Human	H1N1	pdm	2009	USA	A/Wisconsin/629_D00665/2009	ggtgatgccccattccttgatcggctccgccgagatca aaagtccttaaaaggaagaggcaacaccc	-	*
CY046431	Human H1N1pdm2009 IAVs	Human	H1N1	pdm	2009	USA	A/Wisconsin/629_D00677/2009	ggtgatgccccattccttgatcggctccgccgagatca aaagtccttaaaaggaagaggcaacaccc	-	*
CY046815	Human H1N1pdm2009 IAVs	Human	H1N1	pdm	2009	USA	A/Wisconsin/629_D00698/2009	ggtgatgccccattccttgatcggctccgccgagatca aaagtccttaaaaggaagaggcaacaccc	-	*
CY046775	Human H1N1pdm2009 IAVs	Human	H1N1	pdm	2009	USA	A/Wisconsin/629_D00722/2009	ggtgatgccccattccttgatcggctccgccgagatca aaagtccttaaaaggaagaggcaacaccc	-	*
CY089940	Human H1N1pdm2009 IAVs	Human	H1N1	pdm	2009	USA	A/Wisconsin/629_D00744/2009	ggtgatgccccattccttgatcggctccgccgagatca aaagtccttaaaaggaagaggcaacaccc	-	*
CY046391	Human H1N1pdm2009 IAVs	Human	H1N1	pdm	2009	USA	A/Wisconsin/629_D00750/2009	ggtgatgccccattccttgatcggctccgccgagatca aaagtccttaaaaggaagaggcaacaccc	-	*
CY050416	Human H1N1pdm2009 IAVs	Human	H1N1	pdm	2009	USA	A/Wisconsin/629_D00752/2009	ggtgatgccccattccttgatcggctccgccgagatca aaagtccttaaaaggaagaggcaacaccc	-	*
CY046423	Human H1N1pdm2009 IAVs	Human	H1N1	pdm	2009	USA	A/Wisconsin/629_D00767/2009	ggtgatgccccattccttgatcggctccgccgagatca aaagtccttaaaaggaagaggcaacaccc	-	*
CY046367	Human H1N1pdm2009 IAVs	Human	H1N1	pdm	2009	USA	A/Wisconsin/629_D00790/2009	ggtgatgccccattccttgatcggctccgccgagatca aaagtccttaaaaggaagaggcaacaccc	-	*
CY046407	Human H1N1pdm2009 IAVs	Human	H1N1	pdm	2009	USA	A/Wisconsin/629_D00807/2009	ggtgatgccccattccttgatcggctccgccgagatca aaagtccttaaaaggaagaggcaacaccc	-	*
CY089892	Human H1N1pdm2009 IAVs	Human	H1N1		2009	USA	A/Wisconsin/629_D00881/2009	ggtgatgccccattccttgatcggctccgccgagatca aaagtccttaaaaggaagaggcaacaccc	-	*
CY050963	Human H1N1pdm2009 IAVs	Human	H1N1	pdm	2009	USA	A/Wisconsin/629_D00941/2009	ggtgatgccccattccttgatcggctccgccgagatca aaagtccttaaaaggaagaggcaacaccc	-	*

CY050899	Human H1N1pdm2009 IAVs	Human	H1N1	pdm	2009	USA	A/Wisconsin/629_D00949/2009	ggtgatgccccattccttgatcggctccgccgagatca aaagtccttaaaaggaagaggcaacaccc	-	*
CY050400	Human H1N1pdm2009 IAVs	Human	H1N1	pdm	2009	USA	A/Wisconsin/629_D00989/2009	ggtgatgccccattccttgatcggctccgccgagatca aaagtccttaaaaggaagaggcaacaccc	-	*
CY046663	Human H1N1pdm2009 IAVs	Human	H1N1	pdm	2009	USA	A/Wisconsin/629_D00997/2009	ggtgatgccccattccttgatcggctccgccgagatca aaagtccttaaaaggaagaggcaacaccc	-	*
CY046351	Human H1N1pdm2009 IAVs	Human	H1N1	pdm	2009	USA	A/Wisconsin/629_D01017/2009	ggtgatgccccattccttgatcggctccgccgagatca aaagtccttaaaaggaagaggcaacaccc	-	*
CY089956	Human H1N1pdm2009 IAVs	Human	H1N1	pdm	2009	USA	A/Wisconsin/629_D01039/2009	ggtgatgccccattccttgatcggctccgccgagatca aaagtccttaaaaggaagaggcaacaccc	-	*
GQ229379	Human H1N1pdm2009 IAVs	Human	H1N1	pdm	2009	Thailand	A/Thailand/104/2009	ggtgatgccccattccttgatcggctccgccgagatca aaagtccttaaaaggaagaggcaacaccc	-	*
CY046911	Human H1N1pdm2009 IAVs	Human	H1N1	pdm	2009	USA	A/Wisconsin/629_D01083/2009	ggtgatgccccattccttgatcggctccgccgagatca aaagtccttaaaaggaagaggcaacaccc	-	*
CY050376	Human H1N1pdm2009 IAVs	Human	H1N1	pdm	2009	USA	A/Wisconsin/629_D01154/2009	ggtgatgccccattccttgatcggctccgccgagatca aaagtccttaaaaggaagaggcaacaccc	-	*
CY051243	Human H1N1pdm2009 IAVs	Human	H1N1	pdm	2009	USA	A/Wisconsin/629_D01199/2009	ggtgatgccccattccttgatcggctccgccgagatca aaagtccttaaaaggaagaggcaacaccc	-	*
CY050915	Human H1N1pdm2009 IAVs	Human	H1N1	pdm	2009	USA	A/Wisconsin/629_D01262/2009	ggtgatgccccattccttgatcggctccgccgagatca aaagtccttaaaaggaagaggcaacaccc	-	*
CY046543	Human H1N1pdm2009 IAVs	Human	H1N1	pdm	2009	USA	A/Wisconsin/629_D01313/2009	ggtgatgccccattccttgatcggctccgccgagatca aaagtccttaaaaggaagaggcaacaccc	-	*
CY051083	Human H1N1pdm2009 IAVs	Human	H1N1	pdm	2009	USA	A/Wisconsin/629_D01326/2009	ggtgatgccccattccttgatcggctccgccgagatca aaagtccttaaaaggaagaggcaacaccc	-	*
CY051211	Human H1N1pdm2009 IAVs	Human	H1N1	pdm	2009	USA	A/Wisconsin/629_D01414/2009	ggtgatgccccattccttgatcggctccgccgagatca aaagtccttaaaaggaagaggcaacaccc	-	*
CY089900	Human H1N1pdm2009 IAVs	Human	H1N1	pdm	2009	USA	A/Wisconsin/629_D01420/2009	ggtgatgccccattccttgatcggctccgccgagatca aaagtccttaaaaggaagaggcaacaccc	-	*
CY046335	Human H1N1pdm2009 IAVs	Human	H1N1	pdm	2009	USA	A/Wisconsin/629_D01505/2009	ggtgatgccccattccttgatcggctccgccgagatca aaagtccttaaaaggaagaggcaacaccc	-	*
CY050907	Human H1N1pdm2009 IAVs	Human	H1N1	pdm	2009	USA	A/Wisconsin/629_D01779/2009	ggtgatgccccattccttgatcggctccgccgagatca aaagtccttaaaaggaagaggcaacaccc	-	*
CY046191	Human H1N1pdm2009 IAVs	Human	H1N1	pdm	2009	USA	A/Wisconsin/629_D01793/2009	ggtgatgccccattccttgatcggctccgccgagatca aaagtccttaaaaggaagaggcaacaccc	-	*
CY046327	Human H1N1pdm2009 IAVs	Human	H1N1	pdm	2009	USA	A/Wisconsin/629_D01810/2009	ggtgatgccccattccttgatcggctccgccgagatca aaagtccttaaaaggaagaggcaacaccc	-	*
CY089988	Human H1N1pdm2009 IAVs	Human	H1N1	pdm	2009	USA	A/Wisconsin/629_D01822/2009	ggtgatgccccattccttgatcggctccgccgagatca aaagtccttaaaaggaagaggcaacaccc	-	*
CY089884	Human H1N1pdm2009 IAVs	Human	H1N1	pdm	2009	USA	A/Wisconsin/629_D01829/2009	ggtgatgccccattccttgatcggctccgccgagatca aaagtccttaaaaggaagaggcaacaccc	-	*
CY046655	Human H1N1pdm2009 IAVs	Human	H1N1	pdm	2009	USA	A/Wisconsin/629_D01837/2009	ggtgatgccccattccttgatcggctccgccgagatca aaagtccttaaaaggaagaggcaacaccc	-	*
CY050939	Human H1N1pdm2009 IAVs	Human	H1N1	pdm	2009	USA	A/Wisconsin/629_D01847/2009	ggtgatgccccattccttgatcggctccgccgagatca aaagtccttaaaaggaagaggcaacaccc	-	*
CY089948	Human H1N1pdm2009 IAVs	Human	H1N1	pdm	2009	USA	A/Wisconsin/629_D01875/2009	ggtgatgccccattccttgatcggctccgccgagatca aaagtccttaaaaggaagaggcaacaccc	-	*
CY046895	Human H1N1pdm2009 IAVs	Human	H1N1	pdm	2009	USA	A/Wisconsin/629_D01880/2009	ggtgatgccccattccttgatcggctccgccgagatca aaagtccttaaaaggaagaggcaacaccc	-	*
CY050440	Human H1N1pdm2009 IAVs	Human	H1N1	pdm	2009	USA	A/Wisconsin/629_D01886/2009	ggtgatgccccattccttgatcggctccgccgagatca aaagtccttaaaaggaagaggcaacaccc	-	*

CY046471	Human H1N1pdm2009 IAVs	Human	H1N1	pdm	2009	USA	A/Wisconsin/629_D01962/2009	ggtgatgccccattccttgatcggctccgccgagatca aaagtccttaaaaggaagaggcaacaccc	-	*
CY052118	Human H1N1pdm2009 IAVs	Human	H1N1	pdm	2009	USA	A/Wisconsin/629_D01973/2009	ggtgatgccccattccttgatcggctccgccgagatca aaagtccttaaaaggaagaggcaacaccc	-	*
CY051203	Human H1N1pdm2009 IAVs	Human	H1N1	pdm	2009	USA	A/Wisconsin/629_D02024/2009	ggtgatgccccattccttgatcggctccgccgagatca aaagtccttaaaaggaagaggcaacaccc	-	*
CY051171	Human H1N1pdm2009 IAVs	Human	H1N1	pdm	2009	USA	A/Wisconsin/629_D02063/2009	ggtgatgccccattccttgatcggctccgccgagatca aaagtccttaaaaggaagaggcaacaccc	-	*
CY050424	Human H1N1pdm2009 IAVs	Human	H1N1	pdm	2009	USA	A/Wisconsin/629_D02206/2009	ggtgatgccccattccttgatcggctccgccgagatca aaagtccttaaaaggaagaggcaacaccc	-	*
CY050923	Human H1N1pdm2009 IAVs	Human	H1N1	pdm	2009	USA	A/Wisconsin/629_D02213/2009	ggtgatgccccattccttgatcggctccgccgagatca aaagtccttaaaaggaagaggcaacaccc	-	*
CY051187	Human H1N1pdm2009 IAVs	Human	H1N1	pdm	2009	USA	A/Wisconsin/629_D02220/2009	ggtgatgccccattccttgatcggctccgccgagatca aaagtccttaaaaggaagaggcaacaccc	-	*
CY046647	Human H1N1pdm2009 IAVs	Human	H1N1	pdm	2009	USA	A/Wisconsin/629_D02227/2009	ggtgatgccccattccttgatcggctccgccgagatca aaagtccttaaaaggaagaggcaacaccc	-	*
CY046871	Human H1N1pdm2009 IAVs	Human	H1N1	pdm	2009	USA	A/Wisconsin/629_D02272/2009	ggtgatgccccattccttgatcggctccgccgagatca aaagtccttaaaaggaagaggcaacaccc	-	*
CY089876	Human H1N1pdm2009 IAVs	Human	H1N1	pdm	2009	USA	A/Wisconsin/629_D02275/2009	ggtgatgccccattccttgatcggctccgccgagatca aaagtccttaaaaggaagaggcaacaccc	-	*
CY046791	Human H1N1pdm2009 IAVs	Human	H1N1	pdm	2009	USA	A/Wisconsin/629_D02276/2009	ggtgatgccccattccttgatcggctccgccgagatca aaagtccttaaaaggaagaggcaacaccc	-	*
CY089972	Human H1N1pdm2009 IAVs	Human	H1N1		2009	USA	A/Wisconsin/629_D02294/2009	ggtgatgccccattccttgatcggctccgccgagatca aaagtccttaaaaggaagaggcaacaccc	-	*
CY046199	Human H1N1pdm2009 IAVs	Human	H1N1	pdm	2009	USA	A/Wisconsin/629_D02312/2009	ggtgatgccccattccttgatcggctccgccgagatca aaagtccttaaaaggaagaggcaacaccc	-	*
CY050432	Human H1N1pdm2009 IAVs	Human	H1N1		2009	USA	A/Wisconsin/629_D02324/2009	ggtgatgccccattccttgatcggctccgccgagatca aaagtccttaaaaggaagaggcaacaccc	-	*
CY046847	Human H1N1pdm2009 IAVs	Human	H1N1	pdm	2009	USA	A/Wisconsin/629_D02361/2009	ggtgatgccccattccttgatcggctccgccgagatca aaagtccttaaaaggaagaggcaacaccc	-	*
CY089964	Human H1N1pdm2009 IAVs	Human	H1N1	pdm	2009	USA	A/Wisconsin/629_D02404/2009	ggtgatgccccattccttgatcggctccgccgagatca aaagtccttaaaaggaagaggcaacaccc	-	*
CY046727	Human H1N1pdm2009 IAVs	Human	H1N1	pdm	2009	USA	A/Wisconsin/629_D02427/2009	ggtgatgccccattccttgatcggctccgccgagatca aaagtccttaaaaggaagaggcaacaccc	-	*
CY051259	Human H1N1pdm2009 IAVs	Human	H1N1	pdm	2009	USA	A/Wisconsin/629_S0086/2009	ggtgatgccccattccttgatcggctccgccgagatca aaagtccttaaaaggaagaggcaacaccc	-	*
CY051267	Human H1N1pdm2009 IAVs	Human	H1N1	pdm	2009	USA	A/Wisconsin/629_S0143/2009	ggtgatgccccattccttgatcggctccgccgagatca aaagtccttaaaaggaagaggcaacaccc	-	*
CY051275	Human H1N1pdm2009 IAVs	Human	H1N1	pdm	2009	USA	A/Wisconsin/629_S0146/2009	ggtgatgccccattccttgatcggctccgccgagatca aaagtccttaaaaggaagaggcaacaccc	-	*
CY051283	Human H1N1pdm2009 IAVs	Human	H1N1	pdm	2009	USA	A/Wisconsin/629_S0148/2009	ggtgatgccccattccttgatcggctccgccgagatca aaagtccttaaaaggaagaggcaacaccc	-	*
CY051291	Human H1N1pdm2009 IAVs	Human	H1N1	pdm	2009	USA	A/Wisconsin/629_S0173/2009	ggtgatgccccattccttgatcggctccgccgagatca aaagtccttaaaaggaagaggcaacaccc	-	*
CY051299	Human H1N1pdm2009 IAVs	Human	H1N1	pdm	2009	USA	A/Wisconsin/629_S0186/2009	ggtgatgccccattccttgatcggctccgccgagatca aaagtccttaaaaggaagaggcaacaccc	-	*
CY051499	Human H1N1pdm2009 IAVs	Human	H1N1	pdm	2009	USA	A/Wisconsin/629_S0189/2009	ggtgatgccccattccttgatcggctccgccgagatca aaagtccttaaaaggaagaggcaacaccc	-	*
CY051307	Human H1N1pdm2009 IAVs	Human	H1N1	pdm	2009	USA	A/Wisconsin/629_S0197/2009	ggtgatgccccattccttgatcggctccgccgagatca aaagtccttaaaaggaagaggcaacaccc	-	*

CY051323	Human H1N1pdm2009 IAVs	Human	H1N1	pdm	2009	USA	A/Wisconsin/629_S0202/2009	ggtgatgccccattccttgatcggctccgccgagatca aaagtccttaaaaggaagaggcaacaccc	-	*
KF897774	Human H1N1pdm2009 IAVs	Human	H1N1	pdm	2009	France	A/StEtienne/1691/2009	ggtgatgccccattccttgatcggctccgccgagatca aaagtccttaaaaggaagaggcaacaccc	-	*
CY051331	Human H1N1pdm2009 IAVs	Human	H1N1	pdm	2009	USA	A/Wisconsin/629_S0203/2009	ggtgatgccccattccttgatcggctccgccgagatca aaagtccttaaaaggaagaggcaacaccc	-	*
CY051507	Human H1N1pdm2009 IAVs	Human	H1N1	pdm	2009	USA	A/Wisconsin/629_S0206/2009	ggtgatgccccattccttgatcggctccgccgagatca aaagtccttaaaaggaagaggcaacaccc	-	*
CY051515	Human H1N1pdm2009 IAVs	Human	H1N1	pdm	2009	USA	A/Wisconsin/629_S0208/2009	ggtgatgccccattccttgatcggctccgccgagatca aaagtccttaaaaggaagaggcaacaccc	-	*
CY051339	Human H1N1pdm2009 IAVs	Human	H1N1	pdm	2009	USA	A/Wisconsin/629_S0220/2009	ggtgatgccccattccttgatcggctccgccgagatca aaagtccttaaaaggaagaggcaacaccc	-	*
CY051347	Human H1N1pdm2009 IAVs	Human	H1N1	pdm	2009	USA	A/Wisconsin/629_S0222/2009	ggtgatgccccattccttgatcggctccgccgagatca aaagtccttaaaaggaagaggcaacaccc	-	*
CY051355	Human H1N1pdm2009 IAVs	Human	H1N1	pdm	2009	USA	A/Wisconsin/629_S0226/2009	ggtgatgccccattccttgatcggctccgccgagatca aaagtccttaaaaggaagaggcaacaccc	-	*
CY051523	Human H1N1pdm2009 IAVs	Human	H1N1	pdm	2009	USA	A/Wisconsin/629_S0229/2009	ggtgatgccccattccttgatcggctccgccgagatca aaagtccttaaaaggaagaggcaacaccc	-	*
CY051363	Human H1N1pdm2009 IAVs	Human	H1N1	pdm	2009	USA	A/Wisconsin/629_S0245/2009	ggtgatgccccattccttgatcggctccgccgagatca aaagtccttaaaaggaagaggcaacaccc	-	*
CY051379	Human H1N1pdm2009 IAVs	Human	H1N1	pdm	2009	USA	A/Wisconsin/629_S0247/2009	ggtgatgccccattccttgatcggctccgccgagatca aaagtccttaaaaggaagaggcaacaccc	-	*
CY051371	Human H1N1pdm2009 IAVs	Human	H1N1	pdm	2009	USA	A/Wisconsin/629_S0248/2009	ggtgatgccccattccttgatcggctccgccgagatca aaagtccttaaaaggaagaggcaacaccc	-	*
CY051387	Human H1N1pdm2009 IAVs	Human	H1N1	pdm	2009	USA	A/Wisconsin/629_S0254/2009	ggtgatgccccattccttgatcggctccgccgagatca aaagtccttaaaaggaagaggcaacaccc	-	*
CY051395	Human H1N1pdm2009 IAVs	Human	H1N1	pdm	2009	USA	A/Wisconsin/629_S0255/2009	ggtgatgccccattccttgatcggctccgccgagatca aaagtccttaaaaggaagaggcaacaccc	-	*
CY051419	Human H1N1pdm2009 IAVs	Human	H1N1	pdm	2009	USA	A/Wisconsin/629_S0280/2009	ggtgatgccccattccttgatcggctccgccgagatca aaagtccttaaaaggaagaggcaacaccc	-	*
CY051435	Human H1N1pdm2009 IAVs	Human	H1N1	pdm	2009	USA	A/Wisconsin/629_S0293/2009	ggtgatgccccattccttgatcggctccgccgagatca aaagtccttaaaaggaagaggcaacaccc	-	*
CY051443	Human H1N1pdm2009 IAVs	Human	H1N1	pdm	2009	USA	A/Wisconsin/629_S0319/2009	ggtgatgccccattccttgatcggctccgccgagatca aaagtccttaaaaggaagaggcaacaccc	-	*
CY051459	Human H1N1pdm2009 IAVs	Human	H1N1	pdm	2009	USA	A/Wisconsin/629_S0339/2009	ggtgatgccccattccttgatcggctccgccgagatca aaagtccttaaaaggaagaggcaacaccc	-	*
CY051451	Human H1N1pdm2009 IAVs	Human	H1N1	pdm	2009	USA	A/Wisconsin/629_S0345/2009	ggtgatgccccattccttgatcggctccgccgagatca aaagtccttaaaaggaagaggcaacaccc	-	*
CY051483	Human H1N1pdm2009 IAVs	Human	H1N1	pdm	2009	USA	A/Wisconsin/629_S0410/2009	ggtgatgccccattccttgatcggctccgccgagatca aaagtccttaaaaggaagaggcaacaccc	-	*
CY051475	Human H1N1pdm2009 IAVs	Human	H1N1	pdm	2009	USA	A/Wisconsin/629_S0435/2009	ggtgatgccccattccttgatcggctccgccgagatca aaagtccttaaaaggaagaggcaacaccc	-	*
CY051491	Human H1N1pdm2009 IAVs	Human	H1N1	pdm	2009	USA	A/Wisconsin/629_S0464/2009	ggtgatgccccattccttgatcggctccgccgagatca aaagtccttaaaaggaagaggcaacaccc	-	*
CY089980	Human H1N1pdm2009 IAVs	Human	H1N1	pdm	2009	USA	A/Wisconsin/629_D00466/2009	ggtgatgccccattccttgatcggctccgccgagatca aaagtccttaaaaggaagaggcaacaccc	-	*
CY089924	Human H1N1pdm2009 IAVs	Human	H1N1	pdm	2009	USA	A/Wisconsin/629_D01717/2009	ggtgatgccccattccttgatcggctccgccgagatca aaagtccttaaaaggaagaggcaacaccc	-	*
GQ396582	Human H1N1pdm2009 IAVs	Human	H1N1		2009	Spain	A/Andalucia/GP230/2009	ggtgatgccccattccttgatcggctccgccgagatca aaagtccttaaaaggaagaggcaacaccc	-	*

GQ396551	Human H1N1pdm2009 IAVs	Human	H1N1	pdm	2009	Spain	A/Aragon/GP221/2009	ggtgatgccccattccttgatcggctccgccgagatca aaagtccttaaaaggaagaggcaacaccc	-	*
CY121684	Human H1N1pdm2009 IAVs	Human	H1N1	pdm	2009	USA	A/California/07/2009	ggtgatgccccattccttgatcggctccgccgagatca aaagtccttaaaaggaagaggcaacaccc	-	*
GQ402282	Human H1N1pdm2009 IAVs	Human	H1N1		2009	Canada	A/Canada_MB/RV1977/2009	ggtgatgccccattccttgatcggctccgccgagatca aaagtccttaaaaggaagaggcaacaccc	-	*
GQ402283	Human H1N1pdm2009 IAVs	Human	H1N1	pdm	2009	Canada	A/Canada_MB/RV1982/2009	ggtgatgccccattccttgatcggctccgccgagatca aaagtccttaaaaggaagaggcaacaccc	-	*
GQ402286	Human H1N1pdm2009 IAVs	Human	H1N1	pdm	2009	Canada	A/Canada_MB/RV2020/2009	ggtgatgccccattccttgatcggctccgccgagatca aaagtccttaaaaggaagaggcaacaccc	-	*
GQ402267	Human H1N1pdm2009 IAVs	Human	H1N1	pdm	2009	Canada	A/Canada_MB/RV2023/2009	ggtgatgccccattccttgatcggctccgccgagatca aaagtccttaaaaggaagaggcaacaccc	-	*
GQ465722	Human H1N1pdm2009 IAVs	Human	H1N1		2009	Canada	A/Canada_NS/RV1551/2009	ggtgatgccccattccttgatcggctccgccgagatca aaagtccttaaaaggaagaggcaacaccc	-	*
GQ465723	Human H1N1pdm2009 IAVs	Human	H1N1	pdm	2009	Canada	A/Canada_NS/RV1554/2009	ggtgatgccccattccttgatcggctccgccgagatca aaagtccttaaaaggaagaggcaacaccc	-	*
GQ465724	Human H1N1pdm2009 IAVs	Human	H1N1	pdm	2009	Canada	A/Canada_NS/RV1565/2009	ggtgatgccccattccttgatcggctccgccgagatca aaagtccttaaaaggaagaggcaacaccc	-	*
GQ465728	Human H1N1pdm2009 IAVs	Human	H1N1	pdm	2009	Canada	A/Canada_PQ/RV1758/2009	ggtgatgccccattccttgatcggctccgccgagatca aaagtccttaaaaggaagaggcaacaccc	-	*
GQ402274	Human H1N1pdm2009 IAVs	Human	H1N1	pdm	2009	Canada	A/Canada_QC/RV1595/2009	ggtgatgccccattccttgatcggctccgccgagatca aaagtccttaaaaggaagaggcaacaccc	-	*
GQ402279	Human H1N1pdm2009 IAVs	Human	H1N1	pdm	2009	Canada	A/Canada_QC/RV1954/2009	ggtgatgccccattccttgatcggctccgccgagatca aaagtccttaaaaggaagaggcaacaccc	-	*
GQ465729	Human H1N1pdm2009 IAVs	Human	H1N1	pdm	2009	Canada	A/Canada_SK/RV1788/2009	ggtgatgccccattccttgatcggctccgccgagatca aaagtccttaaaaggaagaggcaacaccc	-	*
GQ402277	Human H1N1pdm2009 IAVs	Human	H1N1	pdm	2009	Canada	A/Canada_SK/RV1793/2009	ggtgatgccccattccttgatcggctccgccgagatca aaagtccttaaaaggaagaggcaacaccc	-	*
GQ465730	Human H1N1pdm2009 IAVs	Human	H1N1	pdm	2009	Canada	A/Canada_SK/RV1794/2009	ggtgatgccccattccttgatcggctccgccgagatca aaagtccttaaaaggaagaggcaacaccc	-	*
GQ465731	Human H1N1pdm2009 IAVs	Human	H1N1	pdm	2009	Canada	A/Canada_SK/RV1797/2009	ggtgatgccccattccttgatcggctccgccgagatca aaagtccttaaaaggaagaggcaacaccc	-	*
GQ402278	Human H1N1pdm2009 IAVs	Human	H1N1	pdm	2009	Canada	A/Canada_SK/RV1798/2009	ggtgatgccccattccttgatcggctccgccgagatca aaagtccttaaaaggaagaggcaacaccc	-	*
GQ396545	Human H1N1pdm2009 IAVs	Human	H1N1	pdm	2009	Spain	A/CastillaLaMancha/GP248/2009	ggtgatgccccattccttgatcggctccgccgagatca aaagtccttaaaaggaagaggcaacaccc	-	*
GQ464411	Human H1N1pdm2009 IAVs	Human	H1N1	pdm	2009	Spain	A/Catalonia/63/2009	ggtgatgccccattccttgatcggctccgccgagatca aaagtccttaaaaggaagaggcaacaccc	-	*
HM567556	Human H1N1pdm2009 IAVs	Human	H1N1	pdm	2009	United_Kingdom	A/England/213/2009	ggtgatgccccattccttgatcggctccgccgagatca aaagtccttaaaaggaagaggcaacaccc	-	*
HM567564	Human H1N1pdm2009 IAVs	Human	H1N1	pdm	2009	United_Kingdom	A/England/217/2009	ggtgatgccccattccttgatcggctccgccgagatca aaagtccttaaaaggaagaggcaacaccc	-	*
HM567572	Human H1N1pdm2009 IAVs	Human	H1N1		2009	United_Kingdom	A/England/221/2009	ggtgatgccccattccttgatcggctccgccgagatca aaagtccttaaaaggaagaggcaacaccc	-	*
GQ396585	Human H1N1pdm2009 IAVs	Human	H1N1	pdm	2009	Spain	A/Extremadura/GP360/2009	ggtgatgccccattccttgatcggctccgccgagatca aaagtccttaaaaggaagaggcaacaccc	-	*
GQ396524	Human H1N1pdm2009 IAVs	Human	H1N1	pdm	2009	Spain	A/Galicia/GP224/2009	ggtgatgccccattccttgatcggctccgccgagatca aaagtccttaaaaggaagaggcaacaccc	-	*
CY120113	Human H1N1pdm2009 IAVs	Human	H1N1	pdm	2009	Hong_Kong	A/Hong_Kong/H090_665_V10/2009	ggtgatgccccattccttgatcggctccgccgagatca aaagtccttaaaaggaagaggcaacaccc	-	*

CY111631	Human H1N1pdm2009 IAVs	Human	H1N1	pdm	2009	Hong_Kong	A/Hong_Kong/H090_665_V10/2009	ggtgatgccccattccttgatcggctccgccgagatcaaaagtccttaaaaggaagaggcaacaccc	-	*
CY120137	Human H1N1pdm2009 IAVs	Human	H1N1	pdm	2009	Hong_Kong	A/Hong_Kong/H090_681_V10/2009	ggtgatgccccattccttgatcggctccgccgagatcaaaagtccttaaaaggaagaggcaacaccc	-	*
CY111663	Human H1N1pdm2009 IAVs	Human	H1N1	pdm	2009	Hong_Kong	A/Hong_Kong/H090_681_V10/2009	ggtgatgccccattccttgatcggctccgccgagatcaaaagtccttaaaaggaagaggcaacaccc	-	*
CY111671	Human H1N1pdm2009 IAVs	Human	H1N1	pdm	2009	Hong_Kong	A/Hong_Kong/H090_681_V22/2009	ggtgatgccccattccttgatcggctccgccgagatcaaaagtccttaaaaggaagaggcaacaccc	-	*
CY120145	Human H1N1pdm2009 IAVs	Human	H1N1	pdm	2009	Hong_Kong	A/Hong_Kong/H090_681_V32/2009	ggtgatgccccattccttgatcggctccgccgagatcaaaagtccttaaaaggaagaggcaacaccc	-	*
CY111679	Human H1N1pdm2009 IAVs	Human	H1N1	pdm	2009	Hong_Kong	A/Hong_Kong/H090_681_V32/2009	ggtgatgccccattccttgatcggctccgccgagatcaaaagtccttaaaaggaagaggcaacaccc	-	*
CY053059	Human H1N1pdm2009 IAVs	Human	H1N1	pdm	2009	USA	A/Houston/10OS/2009	ggtgatgccccattccttgatcggctccgccgagatcaaaagtccttaaaaggaagaggcaacaccc	-	*
CY053035	Human H1N1pdm2009 IAVs	Human	H1N1	pdm	2009	USA	A/Houston/7OS/2009	ggtgatgccccattccttgatcggctccgccgagatcaaaagtccttaaaaggaagaggcaacaccc	-	*
GQ396540	Human H1N1pdm2009 IAVs	Human	H1N1	pdm	2009	Spain	A/Madrid/GP193/2009	ggtgatgccccattccttgatcggctccgccgagatcaaaagtccttaaaaggaagaggcaacaccc	-	*
CY050234	Human H1N1pdm2009 IAVs	Human	H1N1	pdm	2009	Mexico	A/Mexico_City/006/2009	ggtgatgccccattccttgatcggctccgccgagatcaaaagtccttaaaaggaagaggcaacaccc	-	*
GQ402269	Human H1N1pdm2009 IAVs	Human	H1N1	pdm	2009	Mexico	A/Mexico/InDRE13494/2009	ggtgatgccccattccttgatcggctccgccgagatcaaaagtccttaaaaggaagaggcaacaccc	-	*
GQ402270	Human H1N1pdm2009 IAVs	Human	H1N1	pdm	2009	Mexico	A/Mexico/InDRE13495/2009	ggtgatgccccattccttgatcggctccgccgagatcaaaagtccttaaaaggaagaggcaacaccc	-	*
GQ402273	Human H1N1pdm2009 IAVs	Human	H1N1	pdm	2009	Mexico	A/Mexico/InDRE13555/2009	ggtgatgccccattccttgatcggctccgccgagatcaaaagtccttaaaaggaagaggcaacaccc	-	*
GQ132167	Human H1N1pdm2009 IAVs	Human	H1N1	pdm	2009	Mexico	A/Mexico/InDRE4114/2009	ggtgatgccccattccttgatcggctccgccgagatcaaaagtccttaaaaggaagaggcaacaccc	-	*
GQ402268	Human H1N1pdm2009 IAVs	Human	H1N1	pdm	2009	Mexico	A/Mexico/InDRE4115/2009	ggtgatgccccattccttgatcggctccgccgagatcaaaagtccttaaaaggaagaggcaacaccc	-	*
GQ396516	Human H1N1pdm2009 IAVs	Human	H1N1	pdm	2009	Spain	A/Murcia/GP78/2009	ggtgatgccccattccttgatcggctccgccgagatcaaaagtccttaaaaggaagaggcaacaccc	-	*
GQ214148	Human H1N1pdm2009 IAVs	Human	H1N1	pdm	2009	France	A/Paris/2580/2009	ggtgatgccccattccttgatcggctccgccgagatcaaaagtccttaaaaggaagaggcaacaccc	-	*
GQ214159	Human H1N1pdm2009 IAVs	Human	H1N1	pdm	2009	France	A/Paris/2592/2009	ggtgatgccccattccttgatcggctccgccgagatcaaaagtccttaaaaggaagaggcaacaccc	-	*
GQ866947	Human H1N1pdm2009 IAVs	Human	H1N1	pdm	2009	Thailand	A/Thailand/CU_B938/2009	ggtgatgccccattccttgatcggctccgccgagatcaaaagtccttaaaaggaagaggcaacaccc	-	*
GQ396573	Human H1N1pdm2009 IAVs	Human	H1N1	pdm	2009	Spain	A/Valencia/GP153/2009	ggtgatgccccattccttgatcggctccgccgagatcaaaagtccttaaaaggaagaggcaacaccc	-	*
GQ396577	Human H1N1pdm2009 IAVs	Human	H1N1	pdm	2009	Spain	A/Valencia/GP154/2009	ggtgatgccccattccttgatcggctccgccgagatcaaaagtccttaaaaggaagaggcaacaccc	-	*
GQ396568	Human H1N1pdm2009 IAVs	Human	H1N1	pdm	2009	Spain	A/Valencia/GP278/2009	ggtgatgccccattccttgatcggctccgccgagatcaaaagtccttaaaaggaagaggcaacaccc	-	*
FJ985752	Human H1N1pdm2009 IAVs	Human	H1N1		2009	Spain	A/Castilla_La_Mancha/GP13/2009	ggtgatgccccattccttgatcggctccgccgagatcaaaagtccttaaaaggaagaggcaacaccc	-	*
FJ985762	Human H1N1pdm2009 IAVs	Human	H1N1		2009	Spain	A/Pais_Vasco/GP20/2009	ggtgatgccccattccttgatcggctccgccgagatcaaaagtccttaaaaggaagaggcaacaccc	-	*
GQ385304	Human H1N1pdm2009 IAVs	Human	H1N1	pdm	2009	Canada	A/Toronto/0462/2009	ggtgatgccccattccttgatcggctccgccgagatcaaaagtccttaaaaggaagaggcaacaccc	-	*

GQ373265	Human H1N1pdm2009 IAVs	Human	H1N1	pdm	2009	Canada	A/Toronto/3141/2009	ggtgatgccccattccttgatcggctccgccgagatca aaagtccttaaaaggaagaggcaacaccc	-	*
CY045982	Human H1N1pdm2009 IAVs	Human	H1N1		2009	Canada	A/Toronto/T9842/2009	ggtgatgccccattccttgatcggctccgccgagatca aaagtccttaaaaggaagaggcaacaccc	-	*
CY045974	Human H1N1pdm2009 IAVs	Human	H1N1	pdm	2009	Canada	A/Toronto/T5362/2009	ggtgatgccccattccttgatcggctccgccgagatca aaagtccttaaaaggaagaggcaacaccc	-	*
CY083340	Human H1N1pdm2009 IAVs	Human	H1N1	pdm	2009	USA	A/San_Diego/WRAIR1654P/2009	ggtgatgccccattccttgatcggctccgccgagatca aaagtccttaaaaggaagaggcaacaccc	-	*
HM567580	Human H1N1pdm2009 IAVs	Human	H1N1	pdm	2009	United_Kingdom	A/England/249/2009	ggtgatgccccattccttgatcggctccgccgagatca aaagtccttaaaaggaagaggcaacaccc	-	*
HM567604	Human H1N1pdm2009 IAVs	Human	H1N1	pdm	2009	United_Kingdom	A/England/313/2009	ggtgatgccccattccttgatcggctccgccgagatca aaagtccttaaaaggaagaggcaacaccc	-	*
HM567780	Human H1N1pdm2009 IAVs	Human	H1N1	pdm	2009	United_Kingdom	A/England/391/2009	ggtgatgccccattccttgatcggctccgccgagatca aaagtccttaaaaggaagaggcaacaccc	-	*
HM567804	Human H1N1pdm2009 IAVs	Human	H1N1	pdm	2009	United_Kingdom	A/England/401/2009	ggtgatgccccattccttgatcggctccgccgagatca aaagtccttaaaaggaagaggcaacaccc	-	*
CY069913	Human H1N1pdm2009 IAVs	Human	H1N1	pdm	2009	United_Kingdom	A/England/412/2009	ggtgatgccccattccttgatcggctccgccgagatca aaagtccttaaaaggaagaggcaacaccc	-	*
HM567900	Human H1N1pdm2009 IAVs	Human	H1N1	pdm	2009	United_Kingdom	A/England/432/2009	ggtgatgccccattccttgatcggctccgccgagatca aaagtccttaaaaggaagaggcaacaccc	-	*
HM567916	Human H1N1pdm2009 IAVs	Human	H1N1	pdm	2009	United_Kingdom	A/England/436/2009	ggtgatgccccattccttgatcggctccgccgagatca aaagtccttaaaaggaagaggcaacaccc	-	*
HM567924	Human H1N1pdm2009 IAVs	Human	H1N1	pdm	2009	United_Kingdom	A/England/443/2009	ggtgatgccccattccttgatcggctccgccgagatca aaagtccttaaaaggaagaggcaacaccc	-	*
CY049919	Human H1N1pdm2009 IAVs	Human	H1N1	pdm	2009	Dominican_Republic	A/Santo_Domingo/0574T/2009	ggtgatgccccattccttgatcggctccgccgagatca aaagtccttaaaaggaagaggcaacaccc	-	*
CY044183	Human H1N1pdm2009 IAVs	Human	H1N1	pdm	2009	USA	A/Silver_Spring/SP509/2009	ggtgatgccccattccttgatcggctccgccgagatca aaagtccttaaaaggaagaggcaacaccc	-	*
CY071758	Human H1N1pdm2009 IAVs	Human	H1N1	pdm	2009	Mexico	A/Mexico_City/WR1706T/2009	ggtgatgccccattccttgatcggctccgccgagatca aaagtccttaaaaggaagaggcaacaccc	-	*
CY041964	Human H1N1pdm2009 IAVs	Human	H1N1	pdm	2009	Dominican_Republic	A/Santo_Domingo/572N/2009	ggtgatgccccattccttgatcggctccgccgagatca aaagtccttaaaaggaagaggcaacaccc	-	*
CY073290	Human H1N1pdm2009 IAVs	Human	H1N1	pdm	2009	USA	A/Silver_Spring/WRAIRSP510P2/2009	ggtgatgccccattccttgatcggctccgccgagatca aaagtccttaaaaggaagaggcaacaccc	-	*
CY073298	Human H1N1pdm2009 IAVs	Human	H1N1	pdm	2009	USA	A/Silver_Spring/WRAIRSP510P20/2009	ggtgatgccccattccttgatcggctccgccgagatca aaagtccttaaaaggaagaggcaacaccc	-	*
CY043106	Human H1N1pdm2009 IAVs	Human	H1N1	pdm	2009	Dominican_Republic	A/Santo_Domingo/565T/2009	ggtgatgccccattccttgatcggctccgccgagatca aaagtccttaaaaggaagaggcaacaccc	-	*
CY049943	Human H1N1pdm2009 IAVs	Human	H1N1	pdm	2009	Dominican_Republic	A/Santo_Domingo/WR1056T/2009	ggtgatgccccattccttgatcggctccgccgagatca aaagtccttaaaaggaagaggcaacaccc	-	*
CY049959	Human H1N1pdm2009 IAVs	Human	H1N1	pdm	2009	Dominican_Republic	A/Santo_Domingo/WR1058N/2009	ggtgatgccccattccttgatcggctccgccgagatca aaagtccttaaaaggaagaggcaacaccc	-	*
CY073266	Human H1N1pdm2009 IAVs	Human	H1N1	pdm	2009	USA	A/Silver_Spring/WRAIRSP510P10/2009	ggtgatgccccattccttgatcggctccgccgagatca aaagtccttaaaaggaagaggcaacaccc	-	*
CY073274	Human H1N1pdm2009 IAVs	Human	H1N1	pdm	2009	USA	A/Silver_Spring/WRAIRSP510P12/2009	ggtgatgccccattccttgatcggctccgccgagatca aaagtccttaaaaggaagaggcaacaccc	-	*
CY073306	Human H1N1pdm2009 IAVs	Human	H1N1	pdm	2009	USA	A/Silver_Spring/WRAIRSP510P3/2009	ggtgatgccccattccttgatcggctccgccgagatca aaagtccttaaaaggaagaggcaacaccc	-	*
CY044224	Human H1N1pdm2009 IAVs	Human	H1N1	pdm	2009	Taiwan	A/Taiwan/T1773/2009	ggtgatgccccattccttgatcggctccgccgagatca aaagtccttaaaaggaagaggcaacaccc	-	*

CY044191	Human H1N1pdm2009 IAVs	Human	H1N1	pdm	2009	USA	A/Silver_Spring/SP510/2009	ggtgatgccccattccttgatcggctccgccgagatca aaagtccttaaaaggaagaggcaacaccc	-	*
CY041987	Human H1N1pdm2009 IAVs	Human	H1N1	pdm	2009	Dominican_Republic	A/Santo_Domingo/0573N/2009	ggtgatgccccattccttgatcggctccgccgagatca aaagtccttaaaaggaagaggcaacaccc	-	*
CY040876	Human H1N1pdm2009 IAVs	Human	H1N1	pdm	2009	Norway	A/Norway/1168/2009	ggtgatgccccattccttgatcggctccgccgagatca aaagtccttaaaaggaagaggcaacaccc	-	*
CY062525	Human H1N1pdm2009 IAVs	Human	H1N1	pdm	2009	Mexico	A/Mexico_city/CIA6/2009	ggtgatgccccattccttgatcggctccgccgagatca aaagtccttaaaaggaagaggcaacaccc	-	*
CY051227	Human H1N1pdm2009 IAVs	Human	H1N1	pdm	2009	USA	A/Wisconsin/629_D00022/2009	ggtgatgccccattccttgatcggctccgccgagatca aaagtccttaaaaggaagaggcaacaccc	-	*
GQ433900	Human H1N1pdm2009 IAVs	Human	H1N1	pdm	2009	China	A/Jiangsu/1/2009	ggtgatgccccattccttgatcggctccgccgagatca aaagtccttaaaaggaagaggcaacaccc	-	*
CY107613	Human H1N1pdm2009 IAVs	Human	H1N1	pdm	2009	United_Kingdom	A/Scotland/EastKilbride_413652/2009	ggtgatgccccattccttgatcggctccgccgagatca aaagtccttaaaaggaagaggcaacaccc	-	*
GQ117072	Human H1N1pdm2009 IAVs	Human	H1N1		2009	USA	A/Minnesota/02/2009	ggtgatgccccattccttgatcggctccgccgagatca aaagtccttaaaaggaagaggcaacaccc	-	*
GQ200219	Human H1N1pdm2009 IAVs	Human	H1N1	pdm	2009	USA	A/Minnesota/03/2009	ggtgatgccccattccttgatcggctccgccgagatca aaagtccttaaaaggaagaggcaacaccc	-	*
GQ396593	Human H1N1pdm2009 IAVs	Human	H1N1	pdm	2009	Spain	A/Andalucia/GP251/2009	ggtgatgccccattccttgatcggctccgccgagatca aaagtccttaaaaggaagaggcaacaccc	-	*
GQ166221	Human H1N1pdm2009 IAVs	Human	H1N1	pdm	2009	Germany	A/Hamburg/4/2009	ggtgatgccccattccttgatcggctccgccgagatca aaagtccttaaaaggaagaggcaacaccc	-	*
GQ117061	Human H1N1pdm2009 IAVs	Human	H1N1	pdm	2009	USA	A/Kansas/03/2009	ggtgatgccccattccttgatcggctccgccgagatca aaagtccttaaaaggaagaggcaacaccc	-	*
CY050178	Human H1N1pdm2009 IAVs	Human	H1N1	pdm	2009	Mexico	A/Mexico_City/015/2009	ggtgatgccccattccttgatcggctccgccgagatca aaagtccttaaaaggaagaggcaacaccc	-	*
HQ011407	Human H1N1pdm2009 IAVs	Human	H1N1	pdm	2009	China	A/Guangdong/01/2009	ggtgatgccccattccttgatcggctccgccgagatca aaagtccttaaaaggaagaggcaacaccc	-	*
GQ221698	Human H1N1pdm2009 IAVs	Human	H1N1	pdm	2009	China	A/Guangdong/1/2009	ggtgatgccccattccttgatcggctccgccgagatca aaagtccttaaaaggaagaggcaacaccc	-	*
GQ323504	Human H1N1pdm2009 IAVs	Human	H1N1	pdm	2009	USA	A/Washington/14/2009	ggtgatgccccattccttgatcggctccgccgagatca aaagtccttaaaaggaagaggcaacaccc	-	*
GQ894874	Human H1N1pdm2009 IAVs	Human	H1N1	pdm	2009	USA	A/Washington/29/2009	ggtgatgccccattccttgatcggctccgccgagatca aaagtccttaaaaggaagaggcaacaccc	-	*
HM780474	Human H1N1pdm2009 IAVs	Human	H1N1	pdm	2009	China	A/Guangdong/06/2009	ggtgatgccccattccttgatcggctccgccgagatca aaagtccttaaaaggaagaggcaacaccc	-	*
GQ160577	Human H1N1pdm2009 IAVs	Human	H1N1	pdm	2009	USA	A/Washington/09/2009	ggtgatgccccattccttgatcggctccgccgagatca aaagtccttaaaaggaagaggcaacaccc	-	*
KC782031	Human H1N1pdm2009 IAVs	Human	H1N1	pdm	2009	USA	A/Washington/51/2009	ggtgatgccccattccttgatcggctccgccgagatca aaagtccttaaaaggaagaggcaacaccc	-	*
CY064795	Human H1N1pdm2009 IAVs	Human	H1N1	pdm	2009	China	A/Guangdong/SB1/2009	ggtgatgccccattccttgatcggctccgccgagatca aaagtccttaaaaggaagaggcaacaccc	-	*
GQ223447	Human H1N1pdm2009 IAVs	Human	H1N1	pdm	2009	China	A/Guangdong/1/2009	ggtgatgccccattccttgatcggctccgccgagatca aaagtccttaaaaggaagaggcaacaccc	-	*
CY051547	Human H1N1pdm2009 IAVs	Human	H1N1	pdm	2009	USA	A/New_York/4403/2009	ggtgatgccccattccttgatcggctccgccgagatca aaagtccttaaaaggaagaggcaacaccc	-	*
KC781803	Human H1N1pdm2009 IAVs	Human	H1N1	pdm	2009	USA	A/Oregon/11/2009	ggtgatgccccattccttgatcggctccgccgagatca aaagtccttaaaaggaagaggcaacaccc	-	*
CY051139	Human H1N1pdm2009 IAVs	Human	H1N1	pdm	2009	USA	A/Wisconsin/629_D01152/2009	ggtgatgccccattccttgatcggctccgccgagatca aaagtccttaaaaggaagaggcaacaccc	-	*

CY083595	Human H1N1pdm2009 IAVs	Human	H1N1	pdm	2009	Peru	A/Lima/WRAIR8893F/2009	ggtgatgccccattccttgatcggctccgccgagatca aaagtccttaaaaggaagaggcaacaccc	-	*
CY058200	Human H1N1pdm2009 IAVs	Human	H1N1	pdm	2009	Nicaragua	A/Managua/1127.02/2009	ggtgatgccccattccttgatcggctccgccgagatca aaagtccttaaaaggaagaggcaacaccc	-	*
CY072570	Human H1N1pdm2009 IAVs	Human	H1N1	pdm	2009	Nicaragua	A/Managua/4197.01/2009	ggtgatgccccattccttgatcggctccgccgagatca aaagtccttaaaaggaagaggcaacaccc	-	*
CY058536	Human H1N1pdm2009 IAVs	Human	H1N1	pdm	2009	Nicaragua	A/Managua/4514.03/2009	ggtgatgccccattccttgatcggctccgccgagatca aaagtccttaaaaggaagaggcaacaccc	-	*
CY058144	Human H1N1pdm2009 IAVs	Human	H1N1	pdm	2009	Nicaragua	A/Managua/5665.01/2009	ggtgatgccccattccttgatcggctccgccgagatca aaagtccttaaaaggaagaggcaacaccc	-	*
CY057226	Human H1N1pdm2009 IAVs	Human	H1N1	pdm	2009	Nicaragua	A/Managua/611.01/2009	ggtgatgccccattccttgatcggctccgccgagatca aaagtccttaaaaggaagaggcaacaccc	-	*
CY058096	Human H1N1pdm2009 IAVs	Human	H1N1	pdm	2009	Nicaragua	A/Managua/655.02/2009	ggtgatgccccattccttgatcggctccgccgagatca aaagtccttaaaaggaagaggcaacaccc	-	*
CY083475	Human H1N1pdm2009 IAVs	Human	H1N1	pdm	2009	Nicaragua	A/Managua/WRAIR1689P/2009	ggtgatgccccattccttgatcggctccgccgagatca aaagtccttaaaaggaagaggcaacaccc	-	*
CY083603	Human H1N1pdm2009 IAVs	Human	H1N1	pdm	2009	Nicaragua	A/Managua/WRAIR8964F/2009	ggtgatgccccattccttgatcggctccgccgagatca aaagtccttaaaaggaagaggcaacaccc	-	*
CY072946	Human H1N1pdm2009 IAVs	Human	H1N1	pdm	2009	Nicaragua	A/Managua/1244.01/2009	ggtgatgccccattccttgatcggctccgccgagatca aaagtccttaaaaggaagaggcaacaccc	-	*
CY053083	Human H1N1pdm2009 IAVs	Human	H1N1	pdm	2009	USA	A/Houston/14OS/2009H1N1_	ggtgatgccccattccttgatcggctccgccgagatca aaagtccttaaaaggaagaggcaacaccc	-	*
CY052963	Human H1N1pdm2009 IAVs	Human	H1N1	pdm	2009	USA	A/Houston/1H/2009	ggtgatgccccattccttgatcggctccgccgagatca aaagtccttaaaaggaagaggcaacaccc	-	*
CY061934	Human H1N1pdm2009 IAVs	Human	H1N1	pdm	2009	USA	A/New_York/6939/2009	ggtgatgccccattccttgatcggctccgccgagatca aaagtccttaaaaggaagaggcaacaccc	-	*
KC780701	Human H1N1pdm2009 IAVs	Human	H1N1	pdm	2009	USA	A/California/41/2009	ggtgatgccccattccttgatcggctccgccgagatca aaagtccttaaaaggaagaggcaacaccc	-	*
CY056144	Human H1N1pdm2009 IAVs	Human	H1N1	pdm	2009	USA	A/San_Diego/INS35/2009	ggtgatgccccattccttgatcggctccgccgagatca aaagtccttaaaaggaagaggcaacaccc	-	*
CY063167	Human H1N1pdm2009 IAVs	Human	H1N1	pdm	2009	USA	A/California/VRDL101/2009	ggtgatgccccattccttgatcggctccgccgagatca aaagtccttaaaaggaagaggcaacaccc	-	*
CY066235	Human H1N1pdm2009 IAVs	Human	H1N1	pdm	2009	USA	A/California/VRDL107/2009	ggtgatgccccattccttgatcggctccgccgagatca aaagtccttaaaaggaagaggcaacaccc	-	*
CY062238	Human H1N1pdm2009 IAVs	Human	H1N1	pdm	2009	USA	A/California/VRDL84/2009	ggtgatgccccattccttgatcggctccgccgagatca aaagtccttaaaaggaagaggcaacaccc	-	*
CY063071	Human H1N1pdm2009 IAVs	Human	H1N1	pdm	2009	USA	A/California/VRDL87/2009	ggtgatgccccattccttgatcggctccgccgagatca aaagtccttaaaaggaagaggcaacaccc	-	*
CY062687	Human H1N1pdm2009 IAVs	Human	H1N1	pdm	2009	USA	A/District_of_Columbia/INS114/2009	ggtgatgccccattccttgatcggctccgccgagatca aaagtccttaaaaggaagaggcaacaccc	-	*
CY066779	Human H1N1pdm2009 IAVs	Human	H1N1	pdm	2009	USA	A/Pensacola/INS213/2009	ggtgatgccccattccttgatcggctccgccgagatca aaagtccttaaaaggaagaggcaacaccc	-	*
CY050971	Human H1N1pdm2009 IAVs	Human	H1N1	pdm	2009	USA	A/Wisconsin/629_D01725/2009	ggtgatgccccattccttgatcggctccgccgagatca aaagtccttaaaaggaagaggcaacaccc	-	*
CY066299	Human H1N1pdm2009 IAVs	Human	H1N1	pdm	2009	USA	A/California/VRDL115/2009	ggtgatgccccattccttgatcggctccgccgagatca aaagtccttaaaaggaagaggcaacaccc	-	*
CY057314	Human H1N1pdm2009 IAVs	Human	H1N1	pdm	2009	USA	A/New_York/5447/2009	ggtgatgccccattccttgatcggctccgccgagatca aaagtccttaaaaggaagaggcaacaccc	-	*
GQ402280	Human H1N1pdm2009 IAVs	Human	H1N1	pdm	2009	Canada	A/Canada_MB/RV1964/2009	ggtgatgccccattccttgatcggctccgccgagatca aaagtccttaaaaggaagaggcaacaccc	-	*

CY069262	Human H1N1pdm2009 IAVs	Human	H1N1		2009	Nicaragua	A/Managua/5364.01/2009	ggtgatgccccattccttgatcggctccgccgagatca aaagtccttaaaaggaagaggcaacaccc	-	*
GQ866955	Human H1N1pdm2009 IAVs	Human	H1N1	pdm	2009	Thailand	A/Thailand/CU_B5/2009	ggtgatgccccattccttgatcggctccgccgagatca aaagtccttaaaaggaagaggcaacaccc	-	*
CY112087	Human H1N1pdm2009 IAVs	Human	H1N1	pdm	2009	Hong_Kong	A/Hong_Kong/H090_793_V10/2009	ggtgatgccccattccttgatcggctccgccgagatca aaagtccttaaaaggaagaggcaacaccc	-	*
CY120481	Human H1N1pdm2009 IAVs	Human	H1N1	pdm	2009	Hong_Kong	A/Hong_Kong/H090_793_V10/2009	ggtgatgccccattccttgatcggctccgccgagatca aaagtccttaaaaggaagaggcaacaccc	-	*
CY120489	Human H1N1pdm2009 IAVs	Human	H1N1	pdm	2009	Hong_Kong	A/Hong_Kong/H090_793_V20/2009	ggtgatgccccattccttgatcggctccgccgagatca aaagtccttaaaaggaagaggcaacaccc	-	*
CY112095	Human H1N1pdm2009 IAVs	Human	H1N1	pdm	2009	Hong_Kong	A/Hong_Kong/H090_793_V20/2009	ggtgatgccccattccttgatcggctccgccgagatca aaagtccttaaaaggaagaggcaacaccc	-	*
CY123302	Human H1N1pdm2009 IAVs	Human	H1N1		2009	Singapore	A/Singapore/GP4581/2009	ggtgatgccccattccttgatcggctccgccgagatca aaagtccttaaaaggaagaggcaacaccc	-	*
CY123771	Human H1N1pdm2009 IAVs	Human	H1N1	pdm	2009	Singapore	A/Singapore/ON2135/2009	ggtgatgccccattccttgatcggctccgccgagatca aaagtccttaaaaggaagaggcaacaccc	-	*
CY047314	Human H1N1pdm2009 IAVs	Human	H1N1	pdm	2009	USA	A/New_York/3074/2009	ggtgatgccccattccttgatcggctccgccgagatca aaagtccttaaaaggaagaggcaacaccc	-	*
KC780848	Human H1N1pdm2009 IAVs	Human	H1N1	pdm	2009	USA	A/Kansas/21/2009	ggtgatgccccattccttgatcggctccgccgagatca aaagtccttaaaaggaagaggcaacaccc	-	*
CY044969	Human H1N1pdm2009 IAVs	Human	H1N1	pdm	2009	USA	A/New_York/3653/2009	ggtgatgccccattccttgatcggctccgccgagatca aaagtccttaaaaggaagaggcaacaccc	-	*
CY046447	Human H1N1pdm2009 IAVs	Human	H1N1	pdm	2009	USA	A/Wisconsin/629_D01226/2009	ggtgatgccccattccttgatcggctccgccgagatca aaagtccttaaaaggaagaggcaacaccc	-	*
CY147799	Human H1N1pdm2009 IAVs	Human	H1N1	pdm	2009	Mexico	A/Mexico/24039/2009	ggtgatgccccattccttgatcggctccgccgagatca aaagtccttaaaaggaagaggcaacaccc	-	*
CY040884	Human H1N1pdm2009 IAVs	Human	H1N1	pdm	2009	Norway	A/Norway/1177/2009	ggtgatgccccattccttgatcggctccgccgagatca aaagtccttaaaaggaagaggcaacaccc	-	*
GQ221793	Human H1N1pdm2009 IAVs	Human	H1N1	pdm	2009	USA	A/Oklahoma/01/2009	ggtgatgccccattccttgatcggctccgccgagatca aaagtccttaaaaggaagaggcaacaccc	-	*
FJ981620	Human H1N1pdm2009 IAVs	Human	H1N1	pdm	2009	USA	A/Texas/04/2009	ggtgatgccccattccttgatcggctccgccgagatca aaagtccttaaaaggaagaggcaacaccc	-	*
FJ966966	Human H1N1pdm2009 IAVs	Human	H1N1	pdm	2009	USA	A/Texas/05/2009	ggtgatgccccattccttgatcggctccgccgagatca aaagtccttaaaaggaagaggcaacaccc	-	*
FJ981611	Human H1N1pdm2009 IAVs	Human	H1N1	pdm	2009	USA	A/Texas/05/2009	ggtgatgccccattccttgatcggctccgccgagatca aaagtccttaaaaggaagaggcaacaccc	-	*
FJ984382	Human H1N1pdm2009 IAVs	Human	H1N1		2009	USA	A/Texas/06/2009	ggtgatgccccattccttgatcggctccgccgagatca aaagtccttaaaaggaagaggcaacaccc	-	*
GQ168853	Human H1N1pdm2009 IAVs	Human	H1N1	pdm	2009	USA	A/Texas/08/2009	ggtgatgccccattccttgatcggctccgccgagatca aaagtccttaaaaggaagaggcaacaccc	-	*
GQ894918	Human H1N1pdm2009 IAVs	Human	H1N1	pdm	2009	USA	A/Rhode_Island/07/2009	ggtgatgccccattccttgatcggctccgccgagatca aaagtccttaaaaggaagaggcaacaccc	-	*
KC800982	Human H1N1pdm2009 IAVs	Human	H1N1		2009	France	A/Lyon/969/2009	ggtgatgccccattccttgatcggctccgccgagatca aaagtccttaaaaggaagaggcaacaccc	-	*
CY044239	Human H1N1pdm2009 IAVs	Human	H1N1	pdm	2009	USA	A/San_Antonio/PR921/2009	ggtgatgccccattccttgatcggctccgccgagatca aaagtccttaaaaggaagaggcaacaccc	-	*
CY147791	Human H1N1pdm2009 IAVs	Human	H1N1	pdm	2009	Mexico	A/Mexico/24038/2009	ggtgatgccccattccttgatcggctccgccgagatca aaagtccttaaaaggaagaggcaacaccc	-	*
CY056423	Human H1N1pdm2009 IAVs	Human	H1N1	pdm	2009	USA	A/New_York/4947/2009	ggtgatgccccattccttgatcggctccgccgagatca aaagtccttaaaaggaagaggcaacaccc	-	*

CY056463	Human H1N1pdm2009 IAVs	Human	H1N1	pdm	2009	USA	A/New_York/4987/2009	ggtgatgccccattccttgatcggctccgccgagatca<br>aaagtccttaaaaggaagaggcaacaccc	-	*
CY083355	Human H1N1pdm2009 IAVs	Human	H1N1	pdm	2009	USA	A/San_Diego/WRAIR1657P/2009	ggtgatgccccattccttgatcggctccgccgagatca<br>aaagtccttaaaaggaagaggcaacaccc	-	*
CY052875	Human H1N1pdm2009 IAVs	Human	H1N1	pdm	2009	USA	A/Texas/44151841/2009	ggtgatgccccattccttgatcggctccgccgagatca<br>aaagtccttaaaaggaagaggcaacaccc	-	*
CY052627	Human H1N1pdm2009 IAVs	Human	H1N1	pdm	2009	USA	A/Texas/44302167/2009	ggtgatgccccattccttgatcggctccgccgagatca<br>aaagtccttaaaaggaagaggcaacaccc	-	*
CY052499	Human H1N1pdm2009 IAVs	Human	H1N1	pdm	2009	USA	A/Texas/45113371/2009	ggtgatgccccattccttgatcggctccgccgagatca<br>aaagtccttaaaaggaagaggcaacaccc	-	*
CY056503	Human H1N1pdm2009 IAVs	Human	H1N1	pdm	2009	USA	A/New_York/5079/2009	ggtgatgccccattccttgatcggctccgccgagatca<br>aaagtccttaaaaggaagaggcaacaccc	-	*
CY057274	Human H1N1pdm2009 IAVs	Human	H1N1	pdm	2009	USA	A/New_York/5217/2009	ggtgatgccccattccttgatcggctccgccgagatca<br>aaagtccttaaaaggaagaggcaacaccc	-	*
CY147887	Human H1N1pdm2009 IAVs	Human	H1N1	pdm	2009	Mexico	A/Mexico/24052/2009	ggtgatgccccattccttgatcggctccgccgagatca<br>aaagtccttaaaaggaagaggcaacaccc	-	*
CY055451	Human H1N1pdm2009 IAVs	Human	H1N1	pdm	2009	USA	A/California/VRDL11/2009	ggtgatgccccattccttgatcggctccgccgagatca<br>aaagtccttaaaaggaagaggcaacaccc	-	*
CY147879	Human H1N1pdm2009 IAVs	Human	H1N1	pdm	2009	Mexico	A/Mexico/24051/2009	ggtgatgccccattccttgatcggctccgccgagatca<br>aaagtccttaaaaggaagaggcaacaccc	-	*
CY052411	Human H1N1pdm2009 IAVs	Human	H1N1	pdm	2009	USA	A/Texas/43132503/2009	ggtgatgccccattccttgatcggctccgccgagatca<br>aaagtccttaaaaggaagaggcaacaccc	-	*
CY050955	Human H1N1pdm2009 IAVs	Human	H1N1	pdm	2009	USA	A/Wisconsin/629_D00724/2009	ggtgatgccccattccttgatcggctccgccgagatca<br>aaagtccttaaaaggaagaggcaacaccc	-	*
CY051115	Human H1N1pdm2009 IAVs	Human	H1N1	pdm	2009	USA	A/Wisconsin/629_D02263/2009	ggtgatgccccattccttgatcggctccgccgagatca<br>aaagtccttaaaaggaagaggcaacaccc	-	*
CY051251	Human H1N1pdm2009 IAVs	Human	H1N1	pdm	2009	USA	A/Wisconsin/629_S0035/2009	ggtgatgccccattccttgatcggctccgccgagatca<br>aaagtccttaaaaggaagaggcaacaccc	-	*
CY051403	Human H1N1pdm2009 IAVs	Human	H1N1	pdm	2009	USA	A/Wisconsin/629_S0252/2009	ggtgatgccccattccttgatcggctccgccgagatca<br>aaagtccttaaaaggaagaggcaacaccc	-	*
KF612173	Human H1N1pdm2009 IAVs	Human	H1N1	pdm	2009	Bolivia	A/Santa_Cruz/46631/2009	ggtgatgccccattccttgatcggctccgccgagatca<br>aaagtccttaaaaggaagaggcaacaccc	-	*
CY044208	Human H1N1pdm2009 IAVs	Human	H1N1	pdm	2009	Taiwan	A/Taiwan/T1338/2009	ggtgatgccccattccttgatcggctccgccgagatca<br>aaagtccttaaaaggaagaggcaacaccc	-	*
CY052867	Human H1N1pdm2009 IAVs	Human	H1N1	pdm	2009	USA	A/Texas/45042604/2009	ggtgatgccccattccttgatcggctccgccgagatca<br>aaagtccttaaaaggaagaggcaacaccc	-	*
CY052475	Human H1N1pdm2009 IAVs	Human	H1N1	pdm	2009	USA	A/Texas/45122033/2009	ggtgatgccccattccttgatcggctccgccgagatca<br>aaagtccttaaaaggaagaggcaacaccc	-	*
CY052459	Human H1N1pdm2009 IAVs	Human	H1N1	pdm	2009	USA	A/Texas/45122369/2009	ggtgatgccccattccttgatcggctccgccgagatca<br>aaagtccttaaaaggaagaggcaacaccc	-	*
CY128247	Human H1N1pdm2009 IAVs	Human	H1N1	pdm	2009	Viet_Nam	A/Viet_Nam/13032048/2009	ggtgatgccccattccttgatcggctccgccgagatca<br>aaagtccttaaaaggaagaggcaacaccc	-	*
CY057922	Human H1N1pdm2009 IAVs	Human	H1N1		2009	USA	A/Wisconsin/629_D01330/2009	ggtgatgccccattccttgatcggctccgccgagatca<br>aaagtccttaaaaggaagaggcaacaccc	-	*
CY147863	Human H1N1pdm2009 IAVs	Human	H1N1	pdm	2009	Mexico	A/Mexico/24049/2009	ggtgatgccccattccttgatcggctccgccgagatca<br>aaagtccttaaaaggaagaggcaacaccc	-	*
CY147951	Human H1N1pdm2009 IAVs	Human	H1N1	pdm	2009	Mexico	A/Mexico/24061/2009	ggtgatgccccattccttgatcggctccgccgagatca<br>aaagtccttaaaaggaagaggcaacaccc	-	*
CY052214	Human H1N1pdm2009 IAVs	Human	H1N1	pdm	2009	USA	A/Texas/42163291/2009	ggtgatgccccattccttgatcggctccgccgagatca<br>aaagtccttaaaaggaagaggcaacaccc	-	*

CY050995	Human H1N1pdm2009 IAVs	Human	H1N1	pdm	2009	USA	A/Wisconsin/629_D01903/2009	ggtgatgccccattccttgatcggctccgccgagatca aaagtccttaaaaggaagaggcaacaccc	-	*
CY051859	Human H1N1pdm2009 IAVs	Human	H1N1	pdm	2009	USA	A/Texas/42122969/2009	ggtgatgccccattccttgatcggctccgccgagatca aaagtccttaaaaggaagaggcaacaccc	-	*
CY066283	Human H1N1pdm2009 IAVs	Human	H1N1	pdm	2009	USA	A/California/VRDL113/2009	ggtgatgccccattccttgatcggctccgccgagatca aaagtccttaaaaggaagaggcaacaccc	-	*
CY069078	Human H1N1pdm2009 IAVs	Human	H1N1	pdm	2009	Guam	A/Guam/NHRC0024/2009	ggtgatgccccattccttgatcggctccgccgagatca aaagtccttaaaaggaagaggcaacaccc	-	*
CY046311	Human H1N1pdm2009 IAVs	Human	H1N1	pdm	2009	USA	A/Wisconsin/629_D01642/2009	ggtgatgccccattccttgatcggctccgccgagatca aaagtccttaaaaggaagaggcaacaccc	-	*
CY050332	Human H1N1pdm2009 IAVs	Human	H1N1		2009	USA	A/Wisconsin/629_D01851/2009	ggtgatgccccattccttgatcggctccgccgagatca aaagtccttaaaaggaagaggcaacaccc	-	*
CY041625	Human H1N1pdm2009 IAVs	Human	H1N1	pdm	2009	USA	A/New_York/3100/2009	ggtgatgccccattccttgatcggctccgccgagatca aaagtccttaaaaggaagaggcaacaccc	-	*
CY072762	Human H1N1pdm2009 IAVs	Human	H1N1		2009	Nicaragua	A/Managua/435.01/2009	ggtgatgccccattccttgatcggctccgccgagatca aaagtccttaaaaggaagaggcaacaccc	-	*
CY147615	Human H1N1pdm2009 IAVs	Human	H1N1	pdm	2009	Mexico	A/Mexico/24007/2009	ggtgatgccccattccttgatcggctccgccgagatca aaagtccttaaaaggaagaggcaacaccc	-	*
HM567732	Human H1N1pdm2009 IAVs	Human	H1N1		2009	United_Kingdom	A/England/365/2009	ggtgatgccccattccttgatcggctccgccgagatca aaagtccttaaaaggaagaggcaacaccc	-	*
CY054911	Human H1N1pdm2009 IAVs	Human	H1N1	pdm	2009	USA	A/California/VRDL40/2009	ggtgatgccccattccttgatcggctccgccgagatca aaagtccttaaaaggaagaggcaacaccc	-	*
GQ402271	Human H1N1pdm2009 IAVs	Human	H1N1	pdm	2009	Mexico	A/Mexico/InDRE13547/2009	ggtgatgccccattccttgatcggctccgccgagatca aaagtccttaaaaggaagaggcaacaccc	-	*
GQ323534	Human H1N1pdm2009 IAVs	Human	H1N1	pdm	2009	USA	A/Missouri/04/2009	ggtgatgccccattccttgatcggctccgccgagatca aaagtccttaaaaggaagaggcaacaccc	-	*
CY063522	Human H1N1pdm2009 IAVs	Human	H1N1		2009	USA	A/Boston/146/2009	ggtgatgccccattccttgatcggctccgccgagatca aaagtccttaaaaggaagaggcaacaccc	-	*
CY050218	Human H1N1pdm2009 IAVs	Human	H1N1	pdm	2009	Mexico	A/Mexico_City/003/2009	ggtgatgccccattccttgatcggctccgccgagatca aaagtccttaaaaggaagaggcaacaccc	-	*
CY043127	Human H1N1pdm2009 IAVs	Human	H1N1	pdm	2009	USA	A/New_York/3389/2009	ggtgatgccccattccttgatcggctccgccgagatca aaagtccttaaaaggaagaggcaacaccc	-	*
CY066443	Human H1N1pdm2009 IAVs	Human	H1N1	pdm	2009	USA	A/California/VRDL133/2009	ggtgatgccccattccttgatcggctccgccgagatca aaagtccttaaaaggaagaggcaacaccc	-	*
CY060895	Human H1N1pdm2009 IAVs	Human	H1N1	pdm	2009	USA	A/Texas/JMS364/2009	ggtgatgccccattccttgatcggctccgccgagatca aaagtccttaaaaggaagaggcaacaccc	-	*
CY128471	Human H1N1pdm2009 IAVs	Human	H1N1	pdm	2009	Viet_Nam	A/Viet_Nam/001_1020/2009	ggtgatgccccattccttgatcggctccgccgagatca aaagtccttaaaaggaagaggcaacaccc	-	*
CY089996	Human H1N1pdm2009 IAVs	Human	H1N1	pdm	2009	USA	A/Wisconsin/629_D01183/2009	ggtgatgccccattccttgatcggctccgccgagatca aaagtccttaaaaggaagaggcaacaccc	-	*
CY083829	Human H1N1pdm2009 IAVs	Human	H1N1	pdm	2009	Poland	A/Wroclaw/INS435/2009	ggtgatgccccattccttgatcggctccgccgagatca aaagtccttaaaaggaagaggcaacaccc	-	*
CY057210	Human H1N1pdm2009 IAVs	Human	H1N1	pdm	2009	Nicaragua	A/Managua/167.01/2009	ggtgatgccccattccttgatcggctccgccgagatca aaagtccttaaaaggaagaggcaacaccc	-	*
CY058104	Human H1N1pdm2009 IAVs	Human	H1N1	pdm	2009	Nicaragua	A/Managua/168.02/2009	ggtgatgccccattccttgatcggctccgccgagatca aaagtccttaaaaggaagaggcaacaccc	-	*
CY072914	Human H1N1pdm2009 IAVs	Human	H1N1	pdm	2009	Nicaragua	A/Managua/2652.01/2009	ggtgatgccccattccttgatcggctccgccgagatca aaagtccttaaaaggaagaggcaacaccc	-	*
CY072970	Human H1N1pdm2009 IAVs	Human	H1N1	pdm	2009	Nicaragua	A/Managua/2750.01/2009	ggtgatgccccattccttgatcggctccgccgagatca aaagtccttaaaaggaagaggcaacaccc	-	*

CY090093	Human H1N1pdm2009 IAVs	Human	H1N1	pdm	2009	Nicaragua	A/Managua/3755.03/2009	ggtgatgccccattccttgatcggctccgccgagatca aaagtccttaaaaggaagaggcaacaccc	-	*
CY058168	Human H1N1pdm2009 IAVs	Human	H1N1	pdm	2009	Nicaragua	A/Managua/4016.02/2009	ggtgatgccccattccttgatcggctccgccgagatca aaagtccttaaaaggaagaggcaacaccc	-	*
CY054695	Human H1N1pdm2009 IAVs	Human	H1N1	pdm	2009	Nicaragua	A/Managua/4467.05/2009	ggtgatgccccattccttgatcggctccgccgagatca aaagtccttaaaaggaagaggcaacaccc	-	*
CY058528	Human H1N1pdm2009 IAVs	Human	H1N1	pdm	2009	Nicaragua	A/Managua/4905.02/2009	ggtgatgccccattccttgatcggctccgccgagatca aaagtccttaaaaggaagaggcaacaccc	-	*
CY058136	Human H1N1pdm2009 IAVs	Human	H1N1	pdm	2009	Nicaragua	A/Managua/516.01/2009	ggtgatgccccattccttgatcggctccgccgagatca aaagtccttaaaaggaagaggcaacaccc	-	*
CY051011	Human H1N1pdm2009 IAVs	Human	H1N1	pdm	2009	USA	A/Wisconsin/629_D01619/2009	ggtgatgccccattccttgatcggctccgccgagatca aaagtccttaaaaggaagaggcaacaccc	-	*
CY046375	Human H1N1pdm2009 IAVs	Human	H1N1	pdm	2009	USA	A/Wisconsin/629_D01705/2009	ggtgatgccccattccttgatcggctccgccgagatca aaagtccttaaaaggaagaggcaacaccc	-	*
GQ402272	Human H1N1pdm2009 IAVs	Human	H1N1		2009	Mexico	A/Mexico/InDRE13551/2009	ggtgatgccccattccttgatcggctccgccgagatca aaagtccttaaaaggaagaggcaacaccc	-	*
CY064528	Human H1N1pdm2009 IAVs	Human	H1N1	pdm	2009	USA	A/Boston/118/2009	ggtgatgccccattccttgatcggctccgccgagatca aaagtccttaaaaggaagaggcaacaccc	-	*
CY051411	Human H1N1pdm2009 IAVs	Human	H1N1	pdm	2009	USA	A/Wisconsin/629_S0269/2009	ggtgatgccccattccttgatcggctccgccgagatca aaagtccttaaaaggaagaggcaacaccc	-	*
CY050242	Human H1N1pdm2009 IAVs	Human	H1N1		2009	Mexico	A/Mexico_City/010/2009	ggtgatgccccattccttgatcggctccgccgagatca aaagtccttaaaaggaagaggcaacaccc	-	*
CY063554	Human H1N1pdm2009 IAVs	Human	H1N1	pdm	2009	USA	A/Boston/151/2009	ggtgatgccccattccttgatcggctccgccgagatca aaagtccttaaaaggaagaggcaacaccc	-	*
GQ132170	Human H1N1pdm2009 IAVs	Human	H1N1	pdm	2009	Canada	A/Canada_NS/RV1536/2009	ggtgatgccccattccttgatcggctccgccgagatca aaagtccttaaaaggaagaggcaacaccc	-	*
GQ866945	Human H1N1pdm2009 IAVs	Human	H1N1	pdm	2009	Thailand	A/Thailand/CU_H276/2009	ggtgatgccccattccttgatcggctccgccgagatca aaagtccttaaaaggaagaggcaacaccc	-	*
CY069270	Human H1N1pdm2009 IAVs	Human	H1N1	pdm	2009	Nicaragua	A/Managua/1264.03/2009	ggtgatgccccattccttgatcggctccgccgagatca aaagtccttaaaaggaagaggcaacaccc	-	*
CY064720	Human H1N1pdm2009 IAVs	Human	H1N1	pdm	2009	Mexico	A/Mexico_City/021/2009	ggtgatgccccattccttgatcggctccgccgagatca aaagtccttaaaaggaagaggcaacaccc	-	*
CY046399	Human H1N1pdm2009 IAVs	Human	H1N1	pdm	2009	USA	A/Wisconsin/629_D01189/2009	ggtgatgccccattccttgatcggctccgccgagatca aaagtccttaaaaggaagaggcaacaccc	-	*
CY046319	Human H1N1pdm2009 IAVs	Human	H1N1	pdm	2009	USA	A/Wisconsin/629_D01308/2009	ggtgatgccccattccttgatcggctccgccgagatca aaagtccttaaaaggaagaggcaacaccc	-	*
CY046439	Human H1N1pdm2009 IAVs	Human	H1N1	pdm	2009	USA	A/Wisconsin/629_D02262/2009	ggtgatgccccattccttgatcggctccgccgagatca aaagtccttaaaaggaagaggcaacaccc	-	*
CY064616	Human H1N1pdm2009 IAVs	Human	H1N1	pdm	2009	USA	A/Boston/131/2009	ggtgatgccccattccttgatcggctccgccgagatca aaagtccttaaaaggaagaggcaacaccc	-	*
CY064600	Human H1N1pdm2009 IAVs	Human	H1N1	pdm	2009	USA	A/Boston/129/2009	ggtgatgccccattccttgatcggctccgccgagatca aaagtccttaaaaggaagaggcaacaccc	-	*
CY053194	Human H1N1pdm2009 IAVs	Human	H1N1	pdm	2009	USA	A/Brownsville/29OS/2009	ggtgatgccccattccttgatcggctccgccgagatca aaagtccttaaaaggaagaggcaacaccc	-	*
CY053202	Human H1N1pdm2009 IAVs	Human	H1N1		2009	USA	A/Brownsville/30OS/2009	ggtgatgccccattccttgatcggctccgccgagatca aaagtccttaaaaggaagaggcaacaccc	-	*
CY147807	Human H1N1pdm2009 IAVs	Human	H1N1	pdm	2009	Mexico	A/Mexico/24040/2009	ggtgatgccccattccttgatcggctccgccgagatca aaagtccttaaaaggaagaggcaacaccc	-	*
CY046615	Human H1N1pdm2009 IAVs	Human	H1N1	pdm	2009	USA	A/Wisconsin/629_D01055/2009	ggtgatgccccattccttgatcggctccgccgagatca aaagtccttaaaaggaagaggcaacaccc	-	*

CY117608	Human H1N1pdm2009 IAVs	Human	H1N1	pdm	2009	Mexico	A/Mexico/UASLP_017/2009	ggtgatgccccattccttgatcggctccgccgagatca aaagtccttaaaaggaagaggcaacaccc	-	*
CY054839	Human H1N1pdm2009 IAVs	Human	H1N1	pdm	2009	USA	A/California/VRDL30/2009	ggtgatgccccattccttgatcggctccgccgagatca aaagtccttaaaaggaagaggcaacaccc	-	*
AB704450	Human H1N1pdm2009 IAVs	Human	H1N1	pdm	2009	Japan	A/Gunma/287/2009	ggtgatgccccattccttgatcggctccgccgagatca aaagtccttaaaaggaagaggcaacaccc	-	*
CY124119	Human H1N1pdm2009 IAVs	Human	H1N1	pdm	2009	Singapore	A/Singapore/ON811/2009	ggtgatgccccattccttgatcggctccgccgagatca aaagtccttaaaaggaagaggcaacaccc	-	*
CY054751	Human H1N1pdm2009 IAVs	Human	H1N1	pdm	2009	USA	A/California/VRDL9/2009	ggtgatgccccattccttgatcggctccgccgagatca aaagtccttaaaaggaagaggcaacaccc	-	*
CY061223	Human H1N1pdm2009 IAVs	Human	H1N1	pdm	2009	USA	A/New_York/6772/2009	ggtgatgccccattccttgatcggctccgccgagatca aaagtccttaaaaggaagaggcaacaccc	-	*
CY061598	Human H1N1pdm2009 IAVs	Human	H1N1	pdm	2009	USA	A/New_York/6806/2009	ggtgatgccccattccttgatcggctccgccgagatca aaagtccttaaaaggaagaggcaacaccc	-	*
GQ402281	Human H1N1pdm2009 IAVs	Human	H1N1	pdm	2009	Canada	A/Canada_MB/RV1975/2009	ggtgatgccccattccttgatcggctccgccgagatca aaagtccttaaaaggaagaggcaacaccc	-	*
GQ402285	Human H1N1pdm2009 IAVs	Human	H1N1	pdm	2009	Canada	A/Canada_MB/RV2018/2009	ggtgatgccccattccttgatcggctccgccgagatca aaagtccttaaaaggaagaggcaacaccc	-	*
CY088684	Human H1N1pdm2009 IAVs	Human	H1N1	pdm	2009	India	A/Mum/NIV398/2009	ggtgatgccccattccttgatcggctccgccgagatca aaagtccttaaaaggaagaggcaacaccc	-	*
CY088698	Human H1N1pdm2009 IAVs	Human	H1N1	pdm	2009	India	A/Pune/NIV759/2009	ggtgatgccccattccttgatcggctccgccgagatca aaagtccttaaaaggaagaggcaacaccc	-	*
CY045966	Human H1N1pdm2009 IAVs	Human	H1N1	pdm	2009	Canada	A/Toronto/T5294/2009	ggtgatgccccattccttgatcggctccgccgagatca aaagtccttaaaaggaagaggcaacaccc	-	*
GQ411901	Human H1N1pdm2009 IAVs	Human	H1N1	pdm	2009	Canada	A/Toronto/T5308/2009	ggtgatgccccattccttgatcggctccgccgagatca aaagtccttaaaaggaagaggcaacaccc	-	*
GQ402276	Human H1N1pdm2009 IAVs	Human	H1N1	pdm	2009	Canada	A/Canada_SK/RV1767/2009	ggtgatgccccattccttgatcggctccgccgagatca aaagtccttaaaaggaagaggcaacaccc	-	*
CY046799	Human H1N1pdm2009 IAVs	Human	H1N1	pdm	2009	USA	A/Wisconsin/629_D01026/2009	ggtgatgccccattccttgatcggctccgccgagatca aaagtccttaaaaggaagaggcaacaccc	-	*
GQ223425	Human H1N1pdm2009 IAVs	Human	H1N1	pdm	2009	Japan	A/Amagasaki/1/2009	ggtgatgccccattccttgatcggctccgccgagatca aaagtccttaaaaggaagaggcaacaccc	-	*
GQ223426	Human H1N1pdm2009 IAVs	Human	H1N1	pdm	2009	Japan	A/Amagasaki/2/2009	ggtgatgccccattccttgatcggctccgccgagatca aaagtccttaaaaggaagaggcaacaccc	-	*
GQ267835	Human H1N1pdm2009 IAVs	Human	H1N1	pdm	2009	Japan	A/Himeji/1/2009	ggtgatgccccattccttgatcggctccgccgagatca aaagtccttaaaaggaagaggcaacaccc	-	*
GQ223428	Human H1N1pdm2009 IAVs	Human	H1N1	pdm	2009	Japan	A/Hyogo/2/2009	ggtgatgccccattccttgatcggctccgccgagatca aaagtccttaaaaggaagaggcaacaccc	-	*
GQ131026	Human H1N1pdm2009 IAVs	Human	H1N1	pdm	2009	South_Korea	A/Korea/01/2009	ggtgatgccccattccttgatcggctccgccgagatca aaagtccttaaaaggaagaggcaacaccc	-	*
GQ223432	Human H1N1pdm2009 IAVs	Human	H1N1		2009	Japan	A/Osaka_C/1/2009	ggtgatgccccattccttgatcggctccgccgagatca aaagtccttaaaaggaagaggcaacaccc	-	*
GQ223433	Human H1N1pdm2009 IAVs	Human	H1N1	pdm	2009	Japan	A/Osaka_C/2/2009	ggtgatgccccattccttgatcggctccgccgagatca aaagtccttaaaaggaagaggcaacaccc	-	*
GQ223430	Human H1N1pdm2009 IAVs	Human	H1N1	pdm	2009	Japan	A/Osaka/1/2009	ggtgatgccccattccttgatcggctccgccgagatca aaagtccttaaaaggaagaggcaacaccc	-	*
GQ375891	Human H1N1pdm2009 IAVs	Human	H1N1	pdm	2009	Japan	A/Osaka/180/2009	ggtgatgccccattccttgatcggctccgccgagatca aaagtccttaaaaggaagaggcaacaccc	-	*
GQ223431	Human H1N1pdm2009 IAVs	Human	H1N1	pdm	2009	Japan	A/Osaka/2/2009	ggtgatgccccattccttgatcggctccgccgagatca aaagtccttaaaaggaagaggcaacaccc	-	*

GQ267842	Human H1N1pdm2009 IAVs	Human	H1N1		2009	Japan	A/Sakai/1/2009	ggtgatgccccattccttgatcggctccgccgagatca aaagtccttaaaaggaagaggcaacaccc	-	*
GQ267848	Human H1N1pdm2009 IAVs	Human	H1N1	pdm	2009	Japan	A/Sakai/2/2009	ggtgatgccccattccttgatcggctccgccgagatca aaagtccttaaaaggaagaggcaacaccc	-	*
GQ300863	Human H1N1pdm2009 IAVs	Human	H1N1	pdm	2009	Japan	A/Shiga/2/2009	ggtgatgccccattccttgatcggctccgccgagatca aaagtccttaaaaggaagaggcaacaccc	-	*
GQ223427	Human H1N1pdm2009 IAVs	Human	H1N1	pdm	2009	Japan	A/Hyogo/1/2009	ggtgatgccccattccttgatcggctccgccgagatca aaagtccttaaaaggaagaggcaacaccc	-	*
GQ223429	Human H1N1pdm2009 IAVs	Human	H1N1	pdm	2009	Japan	A/Kobe/1/2009	ggtgatgccccattccttgatcggctccgccgagatca aaagtccttaaaaggaagaggcaacaccc	-	*
GQ232030	Human H1N1pdm2009 IAVs	Human	H1N1	pdm	2009	USA	A/Arizona/07/2009	ggtgatgccccattccttgatcggctccgccgagatca aaagtccttaaaaggaagaggcaacaccc	-	*
KC782195	Human H1N1pdm2009 IAVs	Human	H1N1	pdm	2009	USA	A/Utah/20/2009	ggtgatgccccattccttgatcggctccgccgagatca aaagtccttaaaaggaagaggcaacaccc	-	*
CY065434	Human H1N1pdm2009 IAVs	Human	H1N1	pdm	2009	United_Kingdom	A/England/256/2009	ggtgatgccccattccttgatcggctccgccgagatca aaagtccttaaaaggaagaggcaacaccc	-	*
CY065402	Human H1N1pdm2009 IAVs	Human	H1N1		2009	United_Kingdom	A/England/XFL00311/2009	ggtgatgccccattccttgatcggctccgccgagatca aaagtccttaaaaggaagaggcaacaccc	-	*
GQ160559	Human H1N1pdm2009 IAVs	Human	H1N1	pdm	2009	USA	A/New_Jersey/02/2009	ggtgatgccccattccttgatcggctccgccgagatca aaagtccttaaaaggaagaggcaacaccc	-	*
GQ160551	Human H1N1pdm2009 IAVs	Human	H1N1	pdm	2009	USA	A/New_Jersey/04/2009	ggtgatgccccattccttgatcggctccgccgagatca aaagtccttaaaaggaagaggcaacaccc	-	*
CY147847	Human H1N1pdm2009 IAVs	Human	H1N1	pdm	2009	Mexico	A/Mexico/24047/2009	ggtgatgccccattccttgatcggctccgccgagatca aaagtccttaaaaggaagaggcaacaccc	-	*
CY051179	Human H1N1pdm2009 IAVs	Human	H1N1	pdm	2009	USA	A/Wisconsin/629_D00428/2009	ggtgatgccccattccttgatcggctccgccgagatca aaagtccttaaaaggaagaggcaacaccc	-	*
CY060554	Human H1N1pdm2009 IAVs	Human	H1N1	pdm	2009	Canada	A/Ontario/25913/2009	ggtgatgccccattccttgatcggctccgccgagatca aaagtccttaaaaggaagaggcaacaccc	-	*
CY055490	Human H1N1pdm2009 IAVs	Human	H1N1	pdm	2009	USA	A/California/VRDL73/2009	ggtgatgccccattccttgatcggctccgccgagatca aaagtccttaaaaggaagaggcaacaccc	-	*
KJ023101	Human H1N1pdm2009 IAVs	Human	H1N1	pdm	2009	India	A/Madhya_Pradesh/011/2009	ggtgatgccccattccttgatcggctccgccgagatca aaagtccttaaaaggaagaggcaacaccc	-	*
KJ023102	Human H1N1pdm2009 IAVs	Human	H1N1		2009	India	A/Tamil_Naidu/012/2009	ggtgatgccccattccttgatcggctccgccgagatca aaagtccttaaaaggaagaggcaacaccc	-	*
CY062887	Human H1N1pdm2009 IAVs	Human	H1N1	pdm	2009	Greece	A/Athens/INS162/2009	ggtgatgccccattccttgatcggctccgccgagatca aaagtccttaaaaggaagaggcaacaccc	-	*
CY067099	Human H1N1pdm2009 IAVs	Human	H1N1	pdm	2009	Greece	A/Athens/INS262/2009	ggtgatgccccattccttgatcggctccgccgagatca aaagtccttaaaaggaagaggcaacaccc	-	*
CY067147	Human H1N1pdm2009 IAVs	Human	H1N1	pdm	2009	Greece	A/Athens/INS269/2009	ggtgatgccccattccttgatcggctccgccgagatca aaagtccttaaaaggaagaggcaacaccc	-	*
JX625407	Human H1N1pdm2009 IAVs	Human	H1N1	pdm	2009	United_Kingdom	A/England/685/2009	ggtgatgccccattccttgatcggctccgccgagatca aaagtccttaaaaggaagaggcaacaccc	-	*
HM138495	Human H1N1pdm2009 IAVs	Human	H1N1	pdm	2009	Germany	A/Germany_BY/74/2009	ggtgatgccccattccttgatcggctccgccgagatca aaagtccttaaaaggaagaggcaacaccc	-	*
CY067647	Human H1N1pdm2009 IAVs	Human	H1N1	pdm	2009	Spain	A/Madrid/INS224/2009	ggtgatgccccattccttgatcggctccgccgagatca aaagtccttaaaaggaagaggcaacaccc	-	*
CY065852	Human H1N1pdm2009 IAVs	Human	H1N1		2009	Netherlands	A/Netherlands/1324/2009	ggtgatgccccattccttgatcggctccgccgagatca aaagtccttaaaaggaagaggcaacaccc	-	*
JQ431309	Human H1N1pdm2009 IAVs	Human	H1N1	pdm	2009	France	A/Reunion/0116_3_M2E/2009	ggtgatgccccattccttgatcggctccgccgagatca aaagtccttaaaaggaagaggcaacaccc	-	*

JQ431310	Human H1N1pdm2009 IAVs	Human	H1N1	pdm	2009	France	A/Reunion/0148_4_M1E/2009	ggtgatgccccattccttgatcggctccgccgagatca aaagtccttaaaaggaagaggcaacaccc	-	*
JQ431311	Human H1N1pdm2009 IAVs	Human	H1N1	pdm	2009	France	A/Reunion/0148_6_M1E/2009	ggtgatgccccattccttgatcggctccgccgagatca aaagtccttaaaaggaagaggcaacaccc	-	*
JQ431313	Human H1N1pdm2009 IAVs	Human	H1N1	pdm	2009	France	A/Reunion/0215_4_M1E/2009	ggtgatgccccattccttgatcggctccgccgagatca aaagtccttaaaaggaagaggcaacaccc	-	*
JQ431314	Human H1N1pdm2009 IAVs	Human	H1N1	pdm	2009	France	A/Reunion/0215_4_M2E/2009	ggtgatgccccattccttgatcggctccgccgagatca aaagtccttaaaaggaagaggcaacaccc	-	*
JQ431316	Human H1N1pdm2009 IAVs	Human	H1N1	pdm	2009	France	A/Reunion/1632_5_M1E/2009	ggtgatgccccattccttgatcggctccgccgagatca aaagtccttaaaaggaagaggcaacaccc	-	*
JQ431317	Human H1N1pdm2009 IAVs	Human	H1N1	pdm	2009	France	A/Reunion/1658_4_M1E/2009	ggtgatgccccattccttgatcggctccgccgagatca aaagtccttaaaaggaagaggcaacaccc	-	*
JQ431318	Human H1N1pdm2009 IAVs	Human	H1N1	pdm	2009	France	A/Reunion/1698_3_M1E/2009	ggtgatgccccattccttgatcggctccgccgagatca aaagtccttaaaaggaagaggcaacaccc	-	*
JQ431320	Human H1N1pdm2009 IAVs	Human	H1N1	pdm	2009	France	A/Reunion/1722_7_M1E/2009	ggtgatgccccattccttgatcggctccgccgagatca aaagtccttaaaaggaagaggcaacaccc	-	*
JQ431321	Human H1N1pdm2009 IAVs	Human	H1N1	pdm	2009	France	A/Reunion/1722_8_M1E/2009	ggtgatgccccattccttgatcggctccgccgagatca aaagtccttaaaaggaagaggcaacaccc	-	*
JQ431322	Human H1N1pdm2009 IAVs	Human	H1N1		2009	France	A/Reunion/2133_5_M1E/2009	ggtgatgccccattccttgatcggctccgccgagatca aaagtccttaaaaggaagaggcaacaccc	-	*
JQ431323	Human H1N1pdm2009 IAVs	Human	H1N1	pdm	2009	France	A/Reunion/2133_6_M1E/2009	ggtgatgccccattccttgatcggctccgccgagatca aaagtccttaaaaggaagaggcaacaccc	-	*
JQ431324	Human H1N1pdm2009 IAVs	Human	H1N1	pdm	2009	France	A/Reunion/2154_2_M5E/2009	ggtgatgccccattccttgatcggctccgccgagatca aaagtccttaaaaggaagaggcaacaccc	-	*
JQ431325	Human H1N1pdm2009 IAVs	Human	H1N1	pdm	2009	France	A/Reunion/2154_4_M4E/2009	ggtgatgccccattccttgatcggctccgccgagatca aaagtccttaaaaggaagaggcaacaccc	-	*
JQ431326	Human H1N1pdm2009 IAVs	Human	H1N1	pdm	2009	France	A/Reunion/2224_3_M3E/2009	ggtgatgccccattccttgatcggctccgccgagatca aaagtccttaaaaggaagaggcaacaccc	-	*
JQ431327	Human H1N1pdm2009 IAVs	Human	H1N1	pdm	2009	France	A/Reunion/2245_3_M1E/2009	ggtgatgccccattccttgatcggctccgccgagatca aaagtccttaaaaggaagaggcaacaccc	-	*
JQ431328	Human H1N1pdm2009 IAVs	Human	H1N1	pdm	2009	France	A/Reunion/2336_4_M1E/2009	ggtgatgccccattccttgatcggctccgccgagatca aaagtccttaaaaggaagaggcaacaccc	-	*
JQ431330	Human H1N1pdm2009 IAVs	Human	H1N1	pdm	2009	France	A/Reunion/2433_3_M1E/2009	ggtgatgccccattccttgatcggctccgccgagatca aaagtccttaaaaggaagaggcaacaccc	-	*
JQ431331	Human H1N1pdm2009 IAVs	Human	H1N1	pdm	2009	France	A/Reunion/2692_2_M2E/2009	ggtgatgccccattccttgatcggctccgccgagatca aaagtccttaaaaggaagaggcaacaccc	-	*
JQ431332	Human H1N1pdm2009 IAVs	Human	H1N1		2009	France	A/Reunion/2923_3_M1E/2009	ggtgatgccccattccttgatcggctccgccgagatca aaagtccttaaaaggaagaggcaacaccc	-	*
CY062943	Human H1N1pdm2009 IAVs	Human	H1N1	pdm	2009	Spain	A/Terrassa/INS172/2009	ggtgatgccccattccttgatcggctccgccgagatca aaagtccttaaaaggaagaggcaacaccc	-	*
CY067187	Human H1N1pdm2009 IAVs	Human	H1N1	pdm	2009	Austria	A/Vienna/INS294/2009	ggtgatgccccattccttgatcggctccgccgagatca aaagtccttaaaaggaagaggcaacaccc	-	*
CY065162	Human H1N1pdm2009 IAVs	Human	H1N1		2009	United_Kingdom	A/England/541/2009	ggtgatgccccattccttgatcggctccgccgagatca aaagtccttaaaaggaagaggcaacaccc	-	*
CY123334	Human H1N1pdm2009 IAVs	Human	H1N1	pdm	2009	Singapore	A/Singapore/GP669/2009	ggtgatgccccattccttgatcggctccgccgagatca aaagtccttaaaaggaagaggcaacaccc	-	*
CY064784	Human H1N1pdm2009 IAVs	Human	H1N1	pdm	2009	Spain	A/Barcelona/INS190/2009	ggtgatgccccattccttgatcggctccgccgagatca aaagtccttaaaaggaagaggcaacaccc	-	*
CY072506	Human H1N1pdm2009 IAVs	Human	H1N1	pdm	2009	Greece	A/Athens/INS347/2009	ggtgatgccccattccttgatcggctccgccgagatca aaagtccttaaaaggaagaggcaacaccc	-	*

CY072514	Human H1N1pdm2009 IAVs	Human	H1N1	pdm	2009	Greece	A/Athens/INS348/2009	ggtgatgccccattccttgatcggctccgccgagatca aaagtccttaaaaggaagaggcaacaccc	-	*
CY069182	Human H1N1pdm2009 IAVs	Human	H1N1		2009	Greece	A/Athens/INS355/2009	ggtgatgccccattccttgatcggctccgccgagatca aaagtccttaaaaggaagaggcaacaccc	-	*
CY062751	Human H1N1pdm2009 IAVs	Human	H1N1	pdm	2009	Spain	A/Madrid/INS129/2009	ggtgatgccccattccttgatcggctccgccgagatca aaagtccttaaaaggaagaggcaacaccc	-	*
CY083741	Human H1N1pdm2009 IAVs	Human	H1N1	pdm	2009	Spain	A/Madrid/INS130/2009	ggtgatgccccattccttgatcggctccgccgagatca aaagtccttaaaaggaagaggcaacaccc	-	*
CY065127	Human H1N1pdm2009 IAVs	Human	H1N1	pdm	2009	Netherlands	A/Netherlands/1064b/2009	ggtgatgccccattccttgatcggctccgccgagatca aaagtccttaaaaggaagaggcaacaccc	-	*
JQ431335	Human H1N1pdm2009 IAVs	Human	H1N1	pdm	2009	France	A/Reunion/2969_5_M1E/2009	ggtgatgccccattccttgatcggctccgccgagatca aaagtccttaaaaggaagaggcaacaccc	-	*
JQ431308	Human H1N1pdm2009 IAVs	Human	H1N1	pdm	2009	France	A/Reunion/0116_3_M1E/2009	ggtgatgccccattccttgatcggctccgccgagatca aaagtccttaaaaggaagaggcaacaccc	-	*
JQ431319	Human H1N1pdm2009 IAVs	Human	H1N1	pdm	2009	France	A/Reunion/1709_4_M2E/2009	ggtgatgccccattccttgatcggctccgccgagatca aaagtccttaaaaggaagaggcaacaccc	-	*
JQ431312	Human H1N1pdm2009 IAVs	Human	H1N1		2009	France	A/Reunion/0159_3_M1E/2009	ggtgatgccccattccttgatcggctccgccgagatca aaagtccttaaaaggaagaggcaacaccc	-	*
JQ431315	Human H1N1pdm2009 IAVs	Human	H1N1	pdm	2009	France	A/Reunion/1572_3_M1E/2009	ggtgatgccccattccttgatcggctccgccgagatca aaagtccttaaaaggaagaggcaacaccc	-	*
CY062046	Human H1N1pdm2009 IAVs	Human	H1N1	pdm	2009	USA	A/New_York/0352/2009	ggtgatgccccattccttgatcggctccgccgagatca aaagtccttaaaaggaagaggcaacaccc	-	*
CY056599	Human H1N1pdm2009 IAVs	Human	H1N1	pdm	2009	USA	A/New_York/5916/2009	ggtgatgccccattccttgatcggctccgccgagatca aaagtccttaaaaggaagaggcaacaccc	-	*
JF714064	Human H1N1pdm2009 IAVs	Swine	H1N1		2009	Canada	A/swine/MB/32_2/2009	ggtgatgccccattccttgatcggctccgccgagatca aaagtccttaaaaggaagaggcaacaccc	-	*
JF714086	Human H1N1pdm2009 IAVs	Swine	H1N1		2009	Canada	A/swine/MB/36/2009	ggtgatgccccattccttgatcggctccgccgagatca aaagtccttaaaaggaagaggcaacaccc	-	*
CY123192	Human H1N1pdm2009 IAVs	Human	H1N1		2009	Singapore	A/Singapore/GP3839/2009	ggtgatgccccattccttgatcggctccgccgagatca aaagtccttaaaaggaagaggcaacaccc	-	*
CY060967	Human H1N1pdm2009 IAVs	Human	H1N1	pdm	2009	USA	A/Texas/JMS381/2009	ggtgatgccccattccttgatcggctccgccgagatca aaagtccttaaaaggaagaggcaacaccc	-	*
CY060975	Human H1N1pdm2009 IAVs	Human	H1N1	pdm	2009	USA	A/Texas/JMS382/2009	ggtgatgccccattccttgatcggctccgccgagatca aaagtccttaaaaggaagaggcaacaccc	-	*
CY071558	Human H1N1pdm2009 IAVs	Human	H1N1	pdm	2009	Jordan	A/Amman/WR1324N/2009	ggtgatgccccattccttgatcggctccgccgagatca aaagtccttaaaaggaagaggcaacaccc	-	*
CY053218	Human H1N1pdm2009 IAVs	Human	H1N1	pdm	2009	USA	A/Brownsville/31H/2009	ggtgatgccccattccttgatcggctccgccgagatca aaagtccttaaaaggaagaggcaacaccc	-	*
CY053210	Human H1N1pdm2009 IAVs	Human	H1N1	pdm	2009	USA	A/Brownsville/31OS/2009	ggtgatgccccattccttgatcggctccgccgagatca aaagtccttaaaaggaagaggcaacaccc	-	*
KC781719	Human H1N1pdm2009 IAVs	Human	H1N1	pdm	2009	USA	A/California/06/2009	ggtgatgccccattccttgatcggctccgccgagatca aaagtccttaaaaggaagaggcaacaccc	-	*
FJ971074	Human H1N1pdm2009 IAVs	Human	H1N1	pdm	2009	USA	A/California/06/2009	ggtgatgccccattccttgatcggctccgccgagatca aaagtccttaaaaggaagaggcaacaccc	-	*
GQ323522	Human H1N1pdm2009 IAVs	Human	H1N1		2009	USA	A/Nevada/05/2009	ggtgatgccccattccttgatcggctccgccgagatca aaagtccttaaaaggaagaggcaacaccc	-	*
GQ329103	Human H1N1pdm2009 IAVs	Human	H1N1	pdm	2009	France	A/Paris/2650/2009	ggtgatgccccattccttgatcggctccgccgagatca aaagtccttaaaaggaagaggcaacaccc	-	*
CY045486	Human H1N1pdm2009 IAVs	Human	H1N1	pdm	2009	Germany	A/Sachsen_Anhalt/101/2009	ggtgatgccccattccttgatcggctccgccgagatca aaagtccttaaaaggaagaggcaacaccc	-	*

CY083300	Human H1N1pdm2009 IAVs	Human	H1N1	pdm	2009	USA	A/San_Diego/WRAIR1647P/2009	ggtgatgccccattccttgatcggctccgccgagatca aaagtccttaaaaggaagaggcaacaccc	-	*
GU136020	Human H1N1pdm2009 IAVs	Human	H1N1	pdm	2009	Japan	A/Shizuoka_C/97/2009	ggtgatgccccattccttgatcggctccgccgagatca aaagtccttaaaaggaagaggcaacaccc	-	*
CY069881	Human H1N1pdm2009 IAVs	Human	H1N1	pdm	2009	United_Kingdom	A/England/350/2009	ggtgatgccccattccttgatcggctccgccgagatca aaagtccttaaaaggaagaggcaacaccc	-	*
GQ465727	Human H1N1pdm2009 IAVs	Human	H1N1	pdm	2009	Canada	A/Canada_AB/RV1644/2009	ggtgatgccccattccttgatcggctccgccgagatca aaagtccttaaaaggaagaggcaacaccc	-	*
HM567692	Human H1N1pdm2009 IAVs	Human	H1N1		2009	United_Kingdom	A/England/350/2009	ggtgatgccccattccttgatcggctccgccgagatca aaagtccttaaaaggaagaggcaacaccc	-	*
CY045494	Human H1N1pdm2009 IAVs	Human	H1N1	pdm	2009	Germany	A/Sachsen_Anhalt/97/2009	ggtgatgccccattccttgatcggctccgccgagatca aaagtccttaaaaggaagaggcaacaccc	-	*
CY062508	Human H1N1pdm2009 IAVs	Human	H1N1	pdm	2009	Mexico	A/Mexico_city/CIA3/2009	ggtgatgccccattccttgatcggctccgccgagatca aaagtccttaaaaggaagaggcaacaccc	-	*
CY054299	Human H1N1pdm2009 IAVs	Human	H1N1	pdm	2009	Mexico	A/Mexico_City/MCIG01/2009	ggtgatgccccattccttgatcggctccgccgagatca aaagtccttaaaaggaagaggcaacaccc	-	*
CY049871	Human H1N1pdm2009 IAVs	Human	H1N1	pdm	2009	USA	A/Cherry_Point/WR0101/2009	ggtgatgccccattccttgatcggctccgccgagatca aaagtccttaaaaggaagaggcaacaccc	-	*
CY049879	Human H1N1pdm2009 IAVs	Human	H1N1	pdm	2009	USA	A/Cherry_Point/WR0102/2009	ggtgatgccccattccttgatcggctccgccgagatca aaagtccttaaaaggaagaggcaacaccc	-	*
CY049824	Human H1N1pdm2009 IAVs	Human	H1N1	pdm	2009	USA	A/Craven/WR0019/2009	ggtgatgccccattccttgatcggctccgccgagatca aaagtccttaaaaggaagaggcaacaccc	-	*
CY071742	Human H1N1pdm2009 IAVs	Human	H1N1		2009	Mexico	A/Mexico_City/WR1701T/2009	ggtgatgccccattccttgatcggctccgccgagatca aaagtccttaaaaggaagaggcaacaccc	-	*
GQ377090	Human H1N1pdm2009 IAVs	Human	H1N1	pdm	2009	USA	A/Colorado/03/2009	ggtgatgccccattccttgatcggctccgccgagatca aaagtccttaaaaggaagaggcaacaccc	-	*
GQ117106	Human H1N1pdm2009 IAVs	Human	H1N1	pdm	2009	USA	A/Nebraska/02/2009	ggtgatgccccattccttgatcggctccgccgagatca aaagtccttaaaaggaagaggcaacaccc	-	*
CY066419	Human H1N1pdm2009 IAVs	Human	H1N1	pdm	2009	USA	A/California/VRDL130/2009	ggtgatgccccattccttgatcggctccgccgagatca aaagtccttaaaaggaagaggcaacaccc	-	*
CY063087	Human H1N1pdm2009 IAVs	Human	H1N1	pdm	2009	USA	A/California/VRDL89/2009	ggtgatgccccattccttgatcggctccgccgagatca aaagtccttaaaaggaagaggcaacaccc	-	*
CY051755	Human H1N1pdm2009 IAVs	Human	H1N1	pdm	2009	USA	A/New_York/4820/2009	ggtgatgccccattccttgatcggctccgccgagatca aaagtccttaaaaggaagaggcaacaccc	-	*
AB704514	Human H1N1pdm2009 IAVs	Human	H1N1	pdm	2009	Japan	A/Yamaguchi/217/2009	ggtgatgccccattccttgatcggctccgccgagatca aaagtccttaaaaggaagaggcaacaccc	-	*
CY090061	Human H1N1pdm2009 IAVs	Human	H1N1	pdm	2009	Nicaragua	A/Managua/4606.07/2009	ggtgatgccccattccttgatcggctccgccgagatca aaagtccttaaaaggaagaggcaacaccc	-	*
CY072562	Human H1N1pdm2009 IAVs	Human	H1N1		2009	Nicaragua	A/Managua/451.01/2009	ggtgatgccccattccttgatcggctccgccgagatca aaagtccttaaaaggaagaggcaacaccc	-	*
CY083882	Human H1N1pdm2009 IAVs	Human	H1N1		2009	USA	A/San_Diego/INS50/2009	ggtgatgccccattccttgatcggctccgccgagatca aaagtccttaaaaggaagaggcaacaccc	-	*
CY060999	Human H1N1pdm2009 IAVs	Human	H1N1	pdm	2009	USA	A/Texas/JMS385/2009	ggtgatgccccattccttgatcggctccgccgagatca aaagtccttaaaaggaagaggcaacaccc	-	*
CY063231	Human H1N1pdm2009 IAVs	Human	H1N1	pdm	2009	USA	A/Wisconsin/629_D00675/2009	ggtgatgccccattccttgatcggctccgccgagatca aaagtccttaaaaggaagaggcaacaccc	-	*
CY057522	Human H1N1pdm2009 IAVs	Human	H1N1		2009	USA	A/Wisconsin/629_D00891/2009	ggtgatgccccattccttgatcggctccgccgagatca aaagtccttaaaaggaagaggcaacaccc	-	*
CY057482	Human H1N1pdm2009 IAVs	Human	H1N1		2009	USA	A/Wisconsin/629_D01237/2009	ggtgatgccccattccttgatcggctccgccgagatca aaagtccttaaaaggaagaggcaacaccc	-	*

HQ165789	Human H1N1pdm2009 IAVs	Human	H1N1	pdm	2009	Kenya	A/Mombasa/27/2009	ggtgatgccccattccttgatcggctccgccgagatca aaagtccttaaaaggaagaggcaacaccc	-	*
HM855255	Human H1N1pdm2009 IAVs	Human	H1N1	pdm	2009	Kenya	A/Mombasa/512/2009	ggtgatgccccattccttgatcggctccgccgagatca aaagtccttaaaaggaagaggcaacaccc	-	*
HQ165792	Human H1N1pdm2009 IAVs	Human	H1N1		2009	Kenya	A/Mombasa/91/2009	ggtgatgccccattccttgatcggctccgccgagatca aaagtccttaaaaggaagaggcaacaccc	-	*
CY089231	Human H1N1pdm2009 IAVs	Human	H1N1	pdm	2009	USA	A/Boston/632/2009	ggtgatgccccattccttgatcggctccgccgagatca aaagtccttaaaaggaagaggcaacaccc	-	*
CY060783	Human H1N1pdm2009 IAVs	Human	H1N1	pdm	2009	USA	A/Texas/46221665/2009	ggtgatgccccattccttgatcggctccgccgagatca aaagtccttaaaaggaagaggcaacaccc	-	*
HM855248	Human H1N1pdm2009 IAVs	Human	H1N1	pdm	2009	Kenya	A/Malindi/238/2009	ggtgatgccccattccttgatcggctccgccgagatca aaagtccttaaaaggaagaggcaacaccc	-	*
CY074075	Human H1N1pdm2009 IAVs	Human	H1N1	pdm	2009	Argentina	A/Argentina/HNRG83/2009	ggtgatgccccattccttgatcggctccgccgagatca aaagtccttaaaaggaagaggcaacaccc	-	*
CY074081	Human H1N1pdm2009 IAVs	Human	H1N1	pdm	2009	Argentina	A/Argentina/HNRG84/2009	ggtgatgccccattccttgatcggctccgccgagatca aaagtccttaaaaggaagaggcaacaccc	-	*
CY119398	Human H1N1pdm2009 IAVs	Human	H1N1	pdm	2009	Malaysia	A/Malaysia/2082543/2009	ggtgatgccccattccttgatcggctccgccgagatca aaagtccttaaaaggaagaggcaacaccc	-	*
CY063758	Human H1N1pdm2009 IAVs	Human	H1N1	pdm	2009	Singapore	A/Singapore/GP3084/2009	ggtgatgccccattccttgatcggctccgccgagatca aaagtccttaaaaggaagaggcaacaccc	-	*
CY123144	Human H1N1pdm2009 IAVs	Human	H1N1	pdm	2009	Singapore	A/Singapore/GP3491/2009	ggtgatgccccattccttgatcggctccgccgagatca aaagtccttaaaaggaagaggcaacaccc	-	*
CY063792	Human H1N1pdm2009 IAVs	Human	H1N1		2009	Singapore	A/Singapore/ON1868/2009	ggtgatgccccattccttgatcggctccgccgagatca aaagtccttaaaaggaagaggcaacaccc	-	*
CY062903	Human H1N1pdm2009 IAVs	Human	H1N1	pdm	2009	Greece	A/Athens/INS164/2009	ggtgatgccccattccttgatcggctccgccgagatca aaagtccttaaaaggaagaggcaacaccc	-	*
CY062927	Human H1N1pdm2009 IAVs	Human	H1N1	pdm	2009	Greece	A/Athens/INS167/2009	ggtgatgccccattccttgatcggctccgccgagatca aaagtccttaaaaggaagaggcaacaccc	-	*
CY067075	Human H1N1pdm2009 IAVs	Human	H1N1	pdm	2009	Greece	A/Athens/INS259/2009	ggtgatgccccattccttgatcggctccgccgagatca aaagtccttaaaaggaagaggcaacaccc	-	*
CY066619	Human H1N1pdm2009 IAVs	Human	H1N1	pdm	2009	Greece	A/Athens/INS274/2009	ggtgatgccccattccttgatcggctccgccgagatca aaagtccttaaaaggaagaggcaacaccc	-	*
KC780919	Human H1N1pdm2009 IAVs	Human	H1N1	pdm	2009	USA	A/Kansas/19/2009	ggtgatgccccattccttgatcggctccgccgagatca aaagtccttaaaaggaagaggcaacaccc	-	*
CY072378	Human H1N1pdm2009 IAVs	Human	H1N1	pdm	2009	Greece	A/Athens/INS329/2009	ggtgatgccccattccttgatcggctccgccgagatca aaagtccttaaaaggaagaggcaacaccc	-	*
CY055641	Human H1N1pdm2009 IAVs	Human	H1N1	pdm	2009	Australia	A/Australia/25/2009	ggtgatgccccattccttgatcggctccgccgagatca aaagtccttaaaaggaagaggcaacaccc	-	*
CY128415	Human H1N1pdm2009 IAVs	Human	H1N1	pdm	2009	Viet_Nam	A/Viet_Nam/11032021/2009	ggtgatgccccattccttgatcggctccgccgagatca aaagtccttaaaaggaagaggcaacaccc	-	*
CY069038	Human H1N1pdm2009 IAVs	Human	H1N1	pdm	2009	Guam	A/Guam/NHRC0019/2009	ggtgatgccccattccttgatcggctccgccgagatca aaagtccttaaaaggaagaggcaacaccc	-	*
HM780492	Human H1N1pdm2009 IAVs	Human	H1N1		2009	China	A/Guangdong/0872/2009	ggtgatgccccattccttgatcggctccgccgagatca aaagtccttaaaaggaagaggcaacaccc	-	*
AB704458	Human H1N1pdm2009 IAVs	Human	H1N1	pdm	2009	Japan	A/Gunma/293/2009	ggtgatgccccattccttgatcggctccgccgagatca aaagtccttaaaaggaagaggcaacaccc	-	*
HQ728114	Human H1N1pdm2009 IAVs	Swine	H1N1		2009	Taiwan	A/swine/Taiwan/CH_1204/2009	ggtgatgccccattccttgatcggctccgccgagatca aaagtccttaaaaggaagaggcaacaccc	-	*
JN187329	Human H1N1pdm2009 IAVs	Human	H1N1	pdm	2009	Taiwan	A/Taiwan/6078/2009	ggtgatgccccattccttgatcggctccgccgagatca aaagtccttaaaaggaagaggcaacaccc	-	*

JN187336	Human H1N1pdm2009 IAVs	Human	H1N1	pdm	2009	Taiwan	A/Taiwan/6200/2009	ggtgatgccccattccttgatcggctccgccgagatca aaagtccttaaaaggaagaggcaacaccc	-	*
JN187338	Human H1N1pdm2009 IAVs	Human	H1N1	pdm	2009	Taiwan	A/Taiwan/6341/2009	ggtgatgccccattccttgatcggctccgccgagatca aaagtccttaaaaggaagaggcaacaccc	-	*
JN187337	Human H1N1pdm2009 IAVs	Human	H1N1	pdm	2009	Taiwan	A/Taiwan/7873/2009	ggtgatgccccattccttgatcggctccgccgagatca aaagtccttaaaaggaagaggcaacaccc	-	*
CY051891	Human H1N1pdm2009 IAVs	Human	H1N1	pdm	2009	USA	A/Texas/42114261/2009	ggtgatgccccattccttgatcggctccgccgagatca aaagtccttaaaaggaagaggcaacaccc	-	*
CY052318	Human H1N1pdm2009 IAVs	Human	H1N1	pdm	2009	USA	A/Texas/42132413/2009	ggtgatgccccattccttgatcggctccgccgagatca aaagtccttaaaaggaagaggcaacaccc	-	*
CY052395	Human H1N1pdm2009 IAVs	Human	H1N1	pdm	2009	USA	A/Texas/42142537/2009	ggtgatgccccattccttgatcggctccgccgagatca aaagtccttaaaaggaagaggcaacaccc	-	*
CY051819	Human H1N1pdm2009 IAVs	Human	H1N1	pdm	2009	USA	A/Texas/42152486/2009	ggtgatgccccattccttgatcggctccgccgagatca aaagtccttaaaaggaagaggcaacaccc	-	*
CY051875	Human H1N1pdm2009 IAVs	Human	H1N1	pdm	2009	USA	A/Texas/42173957/2009	ggtgatgccccattccttgatcggctccgccgagatca aaagtccttaaaaggaagaggcaacaccc	-	*
CY052238	Human H1N1pdm2009 IAVs	Human	H1N1	pdm	2009	USA	A/Texas/42191647/2009	ggtgatgccccattccttgatcggctccgccgagatca aaagtccttaaaaggaagaggcaacaccc	-	*
CY052419	Human H1N1pdm2009 IAVs	Human	H1N1	pdm	2009	USA	A/Texas/42192947/2009	ggtgatgccccattccttgatcggctccgccgagatca aaagtccttaaaaggaagaggcaacaccc	-	*
CY051867	Human H1N1pdm2009 IAVs	Human	H1N1	pdm	2009	USA	A/Texas/42201824/2009	ggtgatgccccattccttgatcggctccgccgagatca aaagtccttaaaaggaagaggcaacaccc	-	*
CY052326	Human H1N1pdm2009 IAVs	Human	H1N1	pdm	2009	USA	A/Texas/42202026/2009	ggtgatgccccattccttgatcggctccgccgagatca aaagtccttaaaaggaagaggcaacaccc	-	*
CY052403	Human H1N1pdm2009 IAVs	Human	H1N1	pdm	2009	USA	A/Texas/42291877/2009	ggtgatgccccattccttgatcggctccgccgagatca aaagtccttaaaaggaagaggcaacaccc	-	*
CY051835	Human H1N1pdm2009 IAVs	Human	H1N1	pdm	2009	USA	A/Texas/43011033/2009	ggtgatgccccattccttgatcggctccgccgagatca aaagtccttaaaaggaagaggcaacaccc	-	*
CY052254	Human H1N1pdm2009 IAVs	Human	H1N1	pdm	2009	USA	A/Texas/43143450/2009	ggtgatgccccattccttgatcggctccgccgagatca aaagtccttaaaaggaagaggcaacaccc	-	*
CY052603	Human H1N1pdm2009 IAVs	Human	H1N1	pdm	2009	USA	A/Texas/45033567/2009	ggtgatgccccattccttgatcggctccgccgagatca aaagtccttaaaaggaagaggcaacaccc	-	*
AB704538	Human H1N1pdm2009 IAVs	Human	H1N1	pdm	2009	Japan	A/Yamaguchi/273/2009	ggtgatgccccattccttgatcggctccgccgagatca aaagtccttaaaaggaagaggcaacaccc	-	*
KF411267	Human H1N1pdm2009 IAVs	Human	H1N1	pdm	2009	China	A/Qingdao/1530/2009	ggtgatgccccattccttgatcggctccgccgagatca aaagtccttaaaaggaagaggcaacaccc	-	*
GU136003	Human H1N1pdm2009 IAVs	Human	H1N1	pdm	2009	Japan	A/Hiroshima/216/2009	ggtgatgccccattccttgatcggctccgccgagatca aaagtccttaaaaggaagaggcaacaccc	-	*
CY123290	Human H1N1pdm2009 IAVs	Human	H1N1	pdm	2009	Singapore	A/Singapore/GP4539/2009	ggtgatgccccattccttgatcggctccgccgagatca aaagtccttaaaaggaagaggcaacaccc	-	*
CY123891	Human H1N1pdm2009 IAVs	Human	H1N1	pdm	2009	Singapore	A/Singapore/ON2400/2009	ggtgatgccccattccttgatcggctccgccgagatca aaagtccttaaaaggaagaggcaacaccc	-	*
CY095996	Human H1N1pdm2009 IAVs	Human	H1N1	pdm	2009	China	A/Zhejiang/91/2009	ggtgatgccccattccttgatcggctccgccgagatca aaagtccttaaaaggaagaggcaacaccc	-	*
CY052294	Human H1N1pdm2009 IAVs	Human	H1N1	pdm	2009	USA	A/Texas/42091791/2009	ggtgatgccccattccttgatcggctccgccgagatca aaagtccttaaaaggaagaggcaacaccc	-	*
CY052198	Human H1N1pdm2009 IAVs	Human	H1N1	pdm	2009	USA	A/Texas/42121926/2009	ggtgatgccccattccttgatcggctccgccgagatca aaagtccttaaaaggaagaggcaacaccc	-	*
CY052387	Human H1N1pdm2009 IAVs	Human	H1N1	pdm	2009	USA	A/Texas/42163295/2009	ggtgatgccccattccttgatcggctccgccgagatca aaagtccttaaaaggaagaggcaacaccc	-	*

CY052246	Human H1N1pdm2009 IAVs	Human	H1N1	pdm	2009	USA	A/Texas/42201798/2009	ggtgatgccccattccttgatcggctccgccgagatca aaagtccttaaaaggaagaggcaacaccc	-	*
CY052334	Human H1N1pdm2009 IAVs	Human	H1N1	pdm	2009	USA	A/Texas/42211898/2009	ggtgatgccccattccttgatcggctccgccgagatca aaagtccttaaaaggaagaggcaacaccc	-	*
CY052190	Human H1N1pdm2009 IAVs	Human	H1N1	pdm	2009	USA	A/Texas/42281289/2009	ggtgatgccccattccttgatcggctccgccgagatca aaagtccttaaaaggaagaggcaacaccc	-	*
CY052427	Human H1N1pdm2009 IAVs	Human	H1N1	pdm	2009	USA	A/Texas/43122467/2009	ggtgatgccccattccttgatcggctccgccgagatca aaagtccttaaaaggaagaggcaacaccc	-	*
CY052675	Human H1N1pdm2009 IAVs	Human	H1N1	pdm	2009	USA	A/Texas/44313703/2009	ggtgatgccccattccttgatcggctccgccgagatca aaagtccttaaaaggaagaggcaacaccc	-	*
CY071486	Human H1N1pdm2009 IAVs	Human	H1N1	pdm	2009	USA	A/California/WR1314P/2009	ggtgatgccccattccttgatcggctccgccgagatca aaagtccttaaaaggaagaggcaacaccc	-	*
CY071494	Human H1N1pdm2009 IAVs	Human	H1N1	pdm	2009	USA	A/California/WR1315P/2009	ggtgatgccccattccttgatcggctccgccgagatca aaagtccttaaaaggaagaggcaacaccc	-	*
CY071638	Human H1N1pdm2009 IAVs	Human	H1N1	pdm	2009	Taiwan	A/Taipei/WR1485T/2009	ggtgatgccccattccttgatcggctccgccgagatca aaagtccttaaaaggaagaggcaacaccc	-	*
CY060847	Human H1N1pdm2009 IAVs	Human	H1N1	pdm	2009	USA	A/Texas/JMS356/2009	ggtgatgccccattccttgatcggctccgccgagatca aaagtccttaaaaggaagaggcaacaccc	-	*
CY060943	Human H1N1pdm2009 IAVs	Human	H1N1	pdm	2009	USA	A/Texas/JMS373/2009	ggtgatgccccattccttgatcggctccgccgagatca aaagtccttaaaaggaagaggcaacaccc	-	*
CY069214	Human H1N1pdm2009 IAVs	Human	H1N1	pdm	2009	Germany	A/Berlin/INS361/2009	ggtgatgccccattccttgatcggctccgccgagatca aaagtccttaaaaggaagaggcaacaccc	-	*
CY062246	Human H1N1pdm2009 IAVs	Human	H1N1	pdm	2009	USA	A/California/VRDL85/2009	ggtgatgccccattccttgatcggctccgccgagatca aaagtccttaaaaggaagaggcaacaccc	-	*
CY063618	Human H1N1pdm2009 IAVs	Human	H1N1	pdm	2009	Germany	A/Cologne/INS136/2009	ggtgatgccccattccttgatcggctccgccgagatca aaagtccttaaaaggaagaggcaacaccc	-	*
JN187340	Human H1N1pdm2009 IAVs	Human	H1N1	pdm	2009	Taiwan	A/Taiwan/1017/2009	ggtgatgccccattccttgatcggctccgccgagatca aaagtccttaaaaggaagaggcaacaccc	-	*
CY053478	Human H1N1pdm2009 IAVs	Human	H1N1	pdm	2009	Taiwan	A/Taiwan/143/2009	ggtgatgccccattccttgatcggctccgccgagatca aaagtccttaaaaggaagaggcaacaccc	-	*
CY053486	Human H1N1pdm2009 IAVs	Human	H1N1	pdm	2009	Taiwan	A/Taiwan/156/2009	ggtgatgccccattccttgatcggctccgccgagatca aaagtccttaaaaggaagaggcaacaccc	-	*
CY053494	Human H1N1pdm2009 IAVs	Human	H1N1	pdm	2009	Taiwan	A/Taiwan/167/2009	ggtgatgccccattccttgatcggctccgccgagatca aaagtccttaaaaggaagaggcaacaccc	-	*
CY053502	Human H1N1pdm2009 IAVs	Human	H1N1		2009	Taiwan	A/Taiwan/177/2009	ggtgatgccccattccttgatcggctccgccgagatca aaagtccttaaaaggaagaggcaacaccc	-	*
CY053510	Human H1N1pdm2009 IAVs	Human	H1N1	pdm	2009	Taiwan	A/Taiwan/206/2009	ggtgatgccccattccttgatcggctccgccgagatca aaagtccttaaaaggaagaggcaacaccc	-	*
CY065924	Human H1N1pdm2009 IAVs	Human	H1N1	pdm	2009	Netherlands	A/Netherlands/2290/2009	ggtgatgccccattccttgatcggctccgccgagatca aaagtccttaaaaggaagaggcaacaccc	-	*
CY123262	Human H1N1pdm2009 IAVs	Human	H1N1		2009	Singapore	A/Singapore/GP4381/2009	ggtgatgccccattccttgatcggctccgccgagatca aaagtccttaaaaggaagaggcaacaccc	-	*
CY064464	Human H1N1pdm2009 IAVs	Human	H1N1	pdm	2009	USA	A/Boston/96/2009	ggtgatgccccattccttgatcggctccgccgagatca aaagtccttaaaaggaagaggcaacaccc	-	*
CY082988	Human H1N1pdm2009 IAVs	Human	H1N1	pdm	2009	Cambodia	A/Cambodia/NHRCC00009/2009	ggtgatgccccattccttgatcggctccgccgagatca aaagtccttaaaaggaagaggcaacaccc	-	*
CY081554	Human H1N1pdm2009 IAVs	Human	H1N1	pdm	2009	Cambodia	A/Cambodia/NHRCC00010/2009	ggtgatgccccattccttgatcggctccgccgagatca aaagtccttaaaaggaagaggcaacaccc	-	*
CY128367	Human H1N1pdm2009 IAVs	Human	H1N1	pdm	2009	Viet_Nam	A/Viet_Nam/15032004/2009	ggtgatgccccattccttgatcggctccgccgagatca aaagtccttaaaaggaagaggcaacaccc	-	*

CY075279	Human H1N1pdm2009 IAVs	Human	H1N1	pdm	2009	Chile	A/Chile/3019/2009	ggtgatgccccattccttgatcggctccgccgagatcaaaagtccttaaaaggaagaggcaacaccc	-	*
CY147911	Human H1N1pdm2009 IAVs	Human	H1N1	pdm	2009	Mexico	A/Mexico/24055/2009	ggtgatgccccattccttgatcggctccgccgagatcaaaagtccttaaaaggaagaggcaacaccc	-	*
CY095858	Human H1N1pdm2009 IAVs	Human	H1N1	pdm	2009	China	A/Hubei/73/2009	ggtgatgccccattccttgatcggctccgccgagatcaaaagtccttaaaaggaagaggcaacaccc	-	*
CY107400	Human H1N1pdm2009 IAVs	Human	H1N1	pdm	2009	United_Kingdom	A/Scotland/Dundee_09V500795/2009	ggtgatgccccattccttgatcggctccgccgagatcaaaagtccttaaaaggaagaggcaacaccc	-	*
KF411272	Human H1N1pdm2009 IAVs	Human	H1N1		2009	China	A/Qingdao/1626/2009	ggtgatgccccattccttgatcggctccgccgagatcaaaagtccttaaaaggaagaggcaacaccc	-	*
CY075504	Human H1N1pdm2009 IAVs	Human	H1N1	pdm	2009	USA	A/Boston/591/2009	ggtgatgccccattccttgatcggctccgccgagatcaaaagtccttaaaaggaagaggcaacaccc	-	*
CY056479	Human H1N1pdm2009 IAVs	Human	H1N1	pdm	2009	USA	A/New_York/4990/2009	ggtgatgccccattccttgatcggctccgccgagatcaaaagtccttaaaaggaagaggcaacaccc	-	*
CY056511	Human H1N1pdm2009 IAVs	Human	H1N1	pdm	2009	USA	A/New_York/5083/2009	ggtgatgccccattccttgatcggctccgccgagatcaaaagtccttaaaaggaagaggcaacaccc	-	*
CY056815	Human H1N1pdm2009 IAVs	Human	H1N1	pdm	2009	USA	A/New_York/6809/2009	ggtgatgccccattccttgatcggctccgccgagatcaaaagtccttaaaaggaagaggcaacaccc	-	*
GQ502910	Human H1N1pdm2009 IAVs	Human	H1N1	pdm	2009	Canada	A/Toronto/R8557/2009	ggtgatgccccattccttgatcggctccgccgagatcaaaagtccttaaaaggaagaggcaacaccc	-	*
CY066707	Human H1N1pdm2009 IAVs	Human	H1N1	pdm	2009	Austria	A/Vienna/INS291/2009	ggtgatgccccattccttgatcggctccgccgagatcaaaagtccttaaaaggaagaggcaacaccc	-	*
CY060522	Human H1N1pdm2009 IAVs	Human	H1N1		2009	Canada	A/Ontario/222656/2009	ggtgatgccccattccttgatcggctccgccgagatcaaaagtccttaaaaggaagaggcaacaccc	-	*
KC782179	Human H1N1pdm2009 IAVs	Human	H1N1	pdm	2009	USA	A/Tennessee/18/2009	ggtgatgccccattccttgatcggctccgccgagatcaaaagtccttaaaaggaagaggcaacaccc	-	*
JQ173160	Human H1N1pdm2009 IAVs	Human	H1N1	pdm	2009	Finland	A/Helsinki/P15/2009	ggtgatgccccattccttgatcggctccgccgagatcaaaagtccttaaaaggaagaggcaacaccc	-	*
CY083427	Human H1N1pdm2009 IAVs	Human	H1N1	pdm	2009	USA	A/San_Diego/WRAIR1667P/2009	ggtgatgccccattccttgatcggctccgccgagatcaaaagtccttaaaaggaagaggcaacaccc	-	*
CY080348	Human H1N1pdm2009 IAVs	Human	H1N1	pdm	2009	Cambodia	A/Cambodia/NHRCC00011/2009	ggtgatgccccattccttgatcggctccgccgagatcaaaagtccttaaaaggaagaggcaacaccc	-	*
CY056975	Human H1N1pdm2009 IAVs	Human	H1N1	pdm	2009	Denmark	A/Copenhagen/INS96/2009	ggtgatgccccattccttgatcggctccgccgagatcaaaagtccttaaaaggaagaggcaacaccc	-	*
CY063055	Human H1N1pdm2009 IAVs	Human	H1N1	pdm	2009	Belgium	A/Tessenderlo/INS191/2009	ggtgatgccccattccttgatcggctccgccgagatcaaaagtccttaaaaggaagaggcaacaccc	-	*
GQ329085	Human H1N1pdm2009 IAVs	Human	H1N1	pdm	2009	France	A/Paris/2670/2009	ggtgatgccccattccttgatcggctccgccgagatcaaaagtccttaaaaggaagaggcaacaccc	-	*
CY071534	Human H1N1pdm2009 IAVs	Human	H1N1	pdm	2009	USA	A/California/WR1320P/2009	ggtgatgccccattccttgatcggctccgccgagatcaaaagtccttaaaaggaagaggcaacaccc	-	*
CY049863	Human H1N1pdm2009 IAVs	Human	H1N1	pdm	2009	USA	A/Cherry_Point/WR0100/2009	ggtgatgccccattccttgatcggctccgccgagatcaaaagtccttaaaaggaagaggcaacaccc	-	*
CY049927	Human H1N1pdm2009 IAVs	Human	H1N1	pdm	2009	USA	A/New_Bern/WR0670/2009	ggtgatgccccattccttgatcggctccgccgagatcaaaagtccttaaaaggaagaggcaacaccc	-	*
CY049935	Human H1N1pdm2009 IAVs	Human	H1N1	pdm	2009	USA	A/Craven/WR1001/2009	ggtgatgccccattccttgatcggctccgccgagatcaaaagtccttaaaaggaagaggcaacaccc	-	*
CY062919	Human H1N1pdm2009 IAVs	Human	H1N1	pdm	2009	Greece	A/Athens/INS166/2009	ggtgatgccccattccttgatcggctccgccgagatcaaaagtccttaaaaggaagaggcaacaccc	-	*
CY072410	Human H1N1pdm2009 IAVs	Human	H1N1	pdm	2009	Greece	A/Athens/INS334/2009	ggtgatgccccattccttgatcggctccgccgagatcaaaagtccttaaaaggaagaggcaacaccc	-	*

CY107436	Human H1N1pdm2009 IAVs	Human	H1N1	pdm	2009	United_Kingdom	A/Scotland/Dundee_09V502390/2009	ggtgatgccccattccttgatcggctccgccgagatca<br>aaagtccttaaaaggaagaggcaacaccc	-	*
CY058010	Human H1N1pdm2009 IAVs	Human	H1N1	pdm	2009	USA	A/Wisconsin/629_D01473/2009	ggtgatgccccattccttgatcggctccgccgagatca<br>aaagtccttaaaaggaagaggcaacaccc	-	*
CY095990	Human H1N1pdm2009 IAVs	Human	H1N1	pdm	2009	China	A/Zhejiang/90/2009	ggtgatgccccattccttgatcggctccgccgagatca<br>aaagtccttaaaaggaagaggcaacaccc	-	*
CY128087	Human H1N1pdm2009 IAVs	Human	H1N1	pdm	2009	Viet_Nam	A/Viet_Nam/811/2009	ggtgatgccccattccttgatcggctccgccgagatca<br>aaagtccttaaaaggaagaggcaacaccc	-	*
CY128025	Human H1N1pdm2009 IAVs	Human	H1	pdm	2009	Viet_Nam	A/Viet_Nam/814/2009	ggtgatgccccattccttgatcggctccgccgagatca<br>aaagtccttaaaaggaagaggcaacaccc	-	*
CY128079	Human H1N1pdm2009 IAVs	Human	H1N1	pdm	2009	Viet_Nam	A/Viet_Nam/817/2009	ggtgatgccccattccttgatcggctccgccgagatca<br>aaagtccttaaaaggaagaggcaacaccc	-	*
CY128095	Human H1N1pdm2009 IAVs	Human	H1N1	pdm	2009	Viet_Nam	A/Viet_Nam/818/2009	ggtgatgccccattccttgatcggctccgccgagatca<br>aaagtccttaaaaggaagaggcaacaccc	-	*
CY128111	Human H1N1pdm2009 IAVs	Human	H1N1	pdm	2009	Viet_Nam	A/Viet_Nam/823/2009	ggtgatgccccattccttgatcggctccgccgagatca<br>aaagtccttaaaaggaagaggcaacaccc	-	*
CY128035	Human H1N1pdm2009 IAVs	Human	H1	pdm	2009	Viet_Nam	A/Viet_Nam/831/2009	ggtgatgccccattccttgatcggctccgccgagatca<br>aaagtccttaaaaggaagaggcaacaccc	-	*
CY128029	Human H1N1pdm2009 IAVs	Human	N1	pdm	2009	Viet_Nam	A/Viet_Nam/834/2009	ggtgatgccccattccttgatcggctccgccgagatca<br>aaagtccttaaaaggaagaggcaacaccc	-	*
CY128103	Human H1N1pdm2009 IAVs	Human	H1N1	pdm	2009	Viet_Nam	A/Viet_Nam/835/2009	ggtgatgccccattccttgatcggctccgccgagatca<br>aaagtccttaaaaggaagaggcaacaccc	-	*
CY128127	Human H1N1pdm2009 IAVs	Human	H1N1	pdm	2009	Viet_Nam	A/Viet_Nam/841/2009	ggtgatgccccattccttgatcggctccgccgagatca<br>aaagtccttaaaaggaagaggcaacaccc	-	*
CY128119	Human H1N1pdm2009 IAVs	Human	H1N1	pdm	2009	Viet_Nam	A/Viet_Nam/850/2009	ggtgatgccccattccttgatcggctccgccgagatca<br>aaagtccttaaaaggaagaggcaacaccc	-	*
HQ533872	Human H1N1pdm2009 IAVs	Human	H1N1		2009	China	A/Beijing/7/2009	ggtgatgccccattccttgatcggctccgccgagatca<br>aaagtccttaaaaggaagaggcaacaccc	-	*
GQ160590	Human H1N1pdm2009 IAVs	Human	H1N1	pdm	2009	USA	A/Virginia/04/2009	ggtgatgccccattccttgatcggctccgccgagatca<br>aaagtccttaaaaggaagaggcaacaccc	-	*
CY095889	Human H1N1pdm2009 IAVs	Human	H1N1	pdm	2009	China	A/Zhejiang/77/2009	ggtgatgccccattccttgatcggctccgccgagatca<br>aaagtccttaaaaggaagaggcaacaccc	-	*
CY095953	Human H1N1pdm2009 IAVs	Human	H1N1	pdm	2009	China	A/Zhejiang/85/2009	ggtgatgccccattccttgatcggctccgccgagatca<br>aaagtccttaaaaggaagaggcaacaccc	-	*
CY053162	Human H1N1pdm2009 IAVs	Human	H1N1		2009	USA	A/Houston/23H/2009	ggtgatgccccattccttgatcggctccgccgagatca<br>aaagtccttaaaaggaagaggcaacaccc	-	*
CY095975	Human H1N1pdm2009 IAVs	Human	H1N1	pdm	2009	China	A/Zhejiang/88/2009	ggtgatgccccattccttgatcggctccgccgagatca<br>aaagtccttaaaaggaagaggcaacaccc	-	*
CY084458	Human H1N1pdm2009 IAVs	Human	H1N1	pdm	2009	USA	A/New_York/6418/2009	ggtgatgccccattccttgatcggctccgccgagatca<br>aaagtccttaaaaggaagaggcaacaccc	-	*
CY128431	Human H1N1pdm2009 IAVs	Human	H1N1	pdm	2009	Viet_Nam	A/Viet_Nam/12032005/2009	ggtgatgccccattccttgatcggctccgccgagatca<br>aaagtccttaaaaggaagaggcaacaccc	-	*
CY075544	Human H1N1pdm2009 IAVs	Human	H1N1	pdm	2009	USA	A/Boston/634/2009	ggtgatgccccattccttgatcggctccgccgagatca<br>aaagtccttaaaaggaagaggcaacaccc	-	*
CY119502	Human H1N1pdm2009 IAVs	Human	H1N1		2009	Malaysia	A/Malaysia/2180812/2009	ggtgatgccccattccttgatcggctccgccgagatca<br>aaagtccttaaaaggaagaggcaacaccc	-	*
CY119510	Human H1N1pdm2009 IAVs	Human	H1N1	pdm	2009	Malaysia	A/Malaysia/2190140/2009	ggtgatgccccattccttgatcggctccgccgagatca<br>aaagtccttaaaaggaagaggcaacaccc	-	*
HM855257	Human H1N1pdm2009 IAVs	Human	H1N1	pdm	2009	Kenya	A/Kisii/143/2009	ggtgatgccccattccttgatcggctccgccgagatca<br>aaagtccttaaaaggaagaggcaacaccc	-	*

CY062831	Human H1N1pdm2009 IAVs	Human	H1N1	pdm	2009	Poland	A/Warsaw/INS146/2009	ggtgatgccccattccttgatcggctccgccgagatca aaagtccttaaaaggaagaggcaacaccc	-	*
CY058712	Human H1N1pdm2009 IAVs	Human	H1N1		2009	USA	A/Texas/46193311/2009	ggtgatgccccattccttgatcggctccgccgagatca aaagtccttaaaaggaagaggcaacaccc	-	*
HM855258	Human H1N1pdm2009 IAVs	Human	H1N1	pdm	2009	Kenya	A/Kikuyu/184/2009	ggtgatgccccattccttgatcggctccgccgagatca aaagtccttaaaaggaagaggcaacaccc	-	*
CY051707	Human H1N1pdm2009 IAVs	Human	H1N1	pdm	2009	USA	A/New_York/4780/2009	ggtgatgccccattccttgatcggctccgccgagatca aaagtccttaaaaggaagaggcaacaccc	-	*
CY052166	Human H1N1pdm2009 IAVs	Human	H1N1	pdm	2009	USA	A/New_York/4788/2009	ggtgatgccccattccttgatcggctccgccgagatca aaagtccttaaaaggaagaggcaacaccc	-	*
CY051723	Human H1N1pdm2009 IAVs	Human	H1N1	pdm	2009	USA	A/New_York/4789/2009	ggtgatgccccattccttgatcggctccgccgagatca aaagtccttaaaaggaagaggcaacaccc	-	*
HM855250	Human H1N1pdm2009 IAVs	Human	H1N1	pdm	2009	Kenya	A/Kikuyu/185/2009	ggtgatgccccattccttgatcggctccgccgagatca aaagtccttaaaaggaagaggcaacaccc	-	*
CY100492	Human H1N1pdm2009 IAVs	Human	H1N1		2009	Mexico	A/Mexico_City/INER14/2009	ggtgatgccccattccttgatcggctccgccgagatca aaagtccttaaaaggaagaggcaacaccc	-	*
CY057930	Human H1N1pdm2009 IAVs	Human	H1N1	pdm	2009	USA	A/Wisconsin/629_D01190/2009	ggtgatgccccattccttgatcggctccgccgagatca aaagtccttaaaaggaagaggcaacaccc	-	*
CY057722	Human H1N1pdm2009 IAVs	Human	H1N1		2009	USA	A/Wisconsin/629_D02317/2009	ggtgatgccccattccttgatcggctccgccgagatca aaagtccttaaaaggaagaggcaacaccc	-	*
CY058336	Human H1N1pdm2009 IAVs	Human	H1N1	pdm	2009	USA	A/Wisconsin/629_D01347/2009	ggtgatgccccattccttgatcggctccgccgagatca aaagtccttaaaaggaagaggcaacaccc	-	*
CY046287	Human H1N1pdm2009 IAVs	Human	H1N1	pdm	2009	USA	A/Wisconsin/629_D02473/2009	ggtgatgccccattccttgatcggctccgccgagatca aaagtccttaaaaggaagaggcaacaccc	-	*
CY061071	Human H1N1pdm2009 IAVs	Human	H1N1	pdm	2009	USA	A/Texas/JMS394/2009	ggtgatgccccattccttgatcggctccgccgagatca aaagtccttaaaaggaagaggcaacaccc	-	*
CY061079	Human H1N1pdm2009 IAVs	Human	H1N1	pdm	2009	USA	A/Texas/JMS395/2009	ggtgatgccccattccttgatcggctccgccgagatca aaagtccttaaaaggaagaggcaacaccc	-	*
CY089343	Human H1N1pdm2009 IAVs	Human	H1N1	pdm	2009	USA	A/Boston/685/2009	ggtgatgccccattccttgatcggctccgccgagatca aaagtccttaaaaggaagaggcaacaccc	-	*
HQ165793	Human H1N1pdm2009 IAVs	Human	H1N1	pdm	2009	Kenya	A/Kisii/205/2009	ggtgatgccccattccttgatcggctccgccgagatca aaagtccttaaaaggaagaggcaacaccc	-	*
CY056224	Human H1N1pdm2009 IAVs	Human	H1N1	pdm	2009	USA	A/San_Diego/INS68/2009	ggtgatgccccattccttgatcggctccgccgagatca aaagtccttaaaaggaagaggcaacaccc	-	*
CY083890	Human H1N1pdm2009 IAVs	Human	H1N1	pdm	2009	USA	A/San_Diego/INS72/2009	ggtgatgccccattccttgatcggctccgccgagatca aaagtccttaaaaggaagaggcaacaccc	-	*
CY049703	Human H1N1pdm2009 IAVs	Human	H1N1	pdm	2009	Singapore	A/Singapore/GP2715/2009	ggtgatgccccattccttgatcggctccgccgagatca aaagtccttaaaaggaagaggcaacaccc	-	*
CY066883	Human H1N1pdm2009 IAVs	Human	H1N1	pdm	2009	USA	A/District_of_Columbia/INS229/2009	ggtgatgccccattccttgatcggctccgccgagatca aaagtccttaaaaggaagaggcaacaccc	-	*
CY055308	Human H1N1pdm2009 IAVs	Human	H1N1	pdm	2009	Mongolia	A/Dundgobi/9746/2009	ggtgatgccccattccttgatcggctccgccgagatca aaagtccttaaaaggaagaggcaacaccc	-	*
GQ323578	Human H1N1pdm2009 IAVs	Human	H1N1	pdm	2009	USA	A/West_Virginia/01/2009	ggtgatgccccattccttgatcggctccgccgagatca aaagtccttaaaaggaagaggcaacaccc	-	*
CY065892	Human H1N1pdm2009 IAVs	Human	H1N1		2009	Netherlands	A/Netherlands/2815/2009	ggtgatgccccattccttgatcggctccgccgagatca aaagtccttaaaaggaagaggcaacaccc	-	*
CY056751	Human H1N1pdm2009 IAVs	Human	H1N1	pdm	2009	USA	A/New_York/6607/2009	ggtgatgccccattccttgatcggctccgccgagatca aaagtccttaaaaggaagaggcaacaccc	-	*
CY095939	Human H1N1pdm2009 IAVs	Human	H1N1	pdm	2009	China	A/Zhejiang/83/2009	ggtgatgccccattccttgatcggctccgccgagatca aaagtccttaaaaggaagaggcaacaccc	-	*

CY095959	Human H1N1pdm2009 IAVs	Human	H1N1	pdm	2009	China	A/Zhejiang/86/2009	ggtgatgccccattccttgatcggctccgccgagatca aaagtccttaaaaggaagaggcaacaccc	-	*
CY057786	Human H1N1pdm2009 IAVs	Human	H1N1	pdm	2009	USA	A/Wisconsin/629_D01014/2009	ggtgatgccccattccttgatcggctccgccgagatca aaagtccttaaaaggaagaggcaacaccc	-	*
CY089247	Human H1N1pdm2009 IAVs	Human	H1N1	pdm	2009	USA	A/Boston/635/2009	ggtgatgccccattccttgatcggctccgccgagatca aaagtccttaaaaggaagaggcaacaccc	-	*
CY052310	Human H1N1pdm2009 IAVs	Human	H1N1	pdm	2009	USA	A/Texas/42254309/2009	ggtgatgccccattccttgatcggctccgccgagatca aaagtccttaaaaggaagaggcaacaccc	-	*
KF411262	Human H1N1pdm2009 IAVs	Human	H1N1		2009	China	A/Qingdao/1170/2009	ggtgatgccccattccttgatcggctccgccgagatca aaagtccttaaaaggaagaggcaacaccc	-	*
CY057954	Human H1N1pdm2009 IAVs	Human	H1N1	pdm	2009	USA	A/Wisconsin/629_D00050/2009	ggtgatgccccattccttgatcggctccgccgagatca aaagtccttaaaaggaagaggcaacaccc	-	*
CY066947	Human H1N1pdm2009 IAVs	Human	H1N1	pdm	2009	Denmark	A/Aarhus/INS237/2009	ggtgatgccccattccttgatcggctccgccgagatca aaagtccttaaaaggaagaggcaacaccc	-	*
CY060562	Human H1N1pdm2009 IAVs	Human	H1N1		2009	Canada	A/Ontario/26184/2009	ggtgatgccccattccttgatcggctccgccgagatca aaagtccttaaaaggaagaggcaacaccc	-	*
KC780844	Human H1N1pdm2009 IAVs	Human	H1N1	pdm	2009	USA	A/Oregon/42/2009	ggtgatgccccattccttgatcggctccgccgagatca aaagtccttaaaaggaagaggcaacaccc	-	*
CY083499	Human H1N1pdm2009 IAVs	Human	H1N1	pdm	2009	USA	A/Chicago/WRAIR1691P/2009	ggtgatgccccattccttgatcggctccgccgagatca aaagtccttaaaaggaagaggcaacaccc	-	*
CY075576	Human H1N1pdm2009 IAVs	Human	H1N1	pdm	2009	USA	A/Boston/658/2009	ggtgatgccccattccttgatcggctccgccgagatca aaagtccttaaaaggaagaggcaacaccc	-	*
CY057266	Human H1N1pdm2009 IAVs	Human	H1N1	pdm	2009	USA	A/New_York/5196/2009	ggtgatgccccattccttgatcggctccgccgagatca aaagtccttaaaaggaagaggcaacaccc	-	*
CY061926	Human H1N1pdm2009 IAVs	Human	H1N1	pdm	2009	USA	A/New_York/6937/2009	ggtgatgccccattccttgatcggctccgccgagatca aaagtccttaaaaggaagaggcaacaccc	-	*
CY063594	Human H1N1pdm2009 IAVs	Human	H1N1	pdm	2009	Greece	A/Athens/INS122/2009	ggtgatgccccattccttgatcggctccgccgagatca aaagtccttaaaaggaagaggcaacaccc	-	*
CY062727	Human H1N1pdm2009 IAVs	Human	H1N1	pdm	2009	Greece	A/Athens/INS125/2009	ggtgatgccccattccttgatcggctccgccgagatca aaagtccttaaaaggaagaggcaacaccc	-	*
CY066627	Human H1N1pdm2009 IAVs	Human	H1N1	pdm	2009	Greece	A/Athens/INS275/2009	ggtgatgccccattccttgatcggctccgccgagatca aaagtccttaaaaggaagaggcaacaccc	-	*
CY066259	Human H1N1pdm2009 IAVs	Human	H1N1	pdm	2009	USA	A/California/VRDL110/2009	ggtgatgccccattccttgatcggctccgccgagatca aaagtccttaaaaggaagaggcaacaccc	-	*
CY060927	Human H1N1pdm2009 IAVs	Human	H1N1	pdm	2009	USA	A/Texas/JMS371/2009	ggtgatgccccattccttgatcggctccgccgagatca aaagtccttaaaaggaagaggcaacaccc	-	*
CY067091	Human H1N1pdm2009 IAVs	Human	H1N1	pdm	2009	Greece	A/Athens/INS261/2009	ggtgatgccccattccttgatcggctccgccgagatca aaagtccttaaaaggaagaggcaacaccc	-	*
CY058360	Human H1N1pdm2009 IAVs	Human	H1N1	pdm	2009	USA	A/Wisconsin/629_D02489/2009	ggtgatgccccattccttgatcggctccgccgagatca aaagtccttaaaaggaagaggcaacaccc	-	*
CY066403	Human H1N1pdm2009 IAVs	Human	H1N1	pdm	2009	USA	A/California/VRDL128/2009	ggtgatgccccattccttgatcggctccgccgagatca aaagtccttaaaaggaagaggcaacaccc	-	*
CY071830	Human H1N1pdm2009 IAVs	Human	H1N1	pdm	2009	Ethiopia	A/Addis_Ababa/WR2848T/2009	ggtgatgccccattccttgatcggctccgccgagatca aaagtccttaaaaggaagaggcaacaccc	-	*
CY073068	Human H1N1pdm2009 IAVs	Human	H1N1	pdm	2009	Turkey	A/Ankara/WRAIR1425T/2009	ggtgatgccccattccttgatcggctccgccgagatca aaagtccttaaaaggaagaggcaacaccc	-	*
CY083635	Human H1N1pdm2009 IAVs	Human	H1N1	pdm	2009	Peru	A/Arequipa/WRAIR9939F/2009	ggtgatgccccattccttgatcggctccgccgagatca aaagtccttaaaaggaagaggcaacaccc	-	*
CY107229	Human H1N1pdm2009 IAVs	Human	H1N1	pdm	2009	United_Kingdom	A/Scotland/Aberdeen_11/2009	ggtgatgccccattccttgatcggctccgccgagatca aaagtccttaaaaggaagaggcaacaccc	-	*

CY107337	Human H1N1pdm2009 IAVs	Human	H1N1	pdm	2009	United_Kingdom	A/Scotland/Dundee_09V500199/2009	ggtgatgccccattccttgatcggctccgccgagatcaaaagtccttaaaaggaagaggcaacaccc	-	*
CY107415	Human H1N1pdm2009 IAVs	Human	H1N1	pdm	2009	United_Kingdom	A/Scotland/Perth_09V501016/2009	ggtgatgccccattccttgatcggctccgccgagatcaaaagtccttaaaaggaagaggcaacaccc	-	*
CY073053	Human H1N1pdm2009 IAVs	Human	H1N1	pdm	2009	Dominican_Republic	A/Santo_Domingo/WRAIR1414N/2009	ggtgatgccccattccttgatcggctccgccgagatcaaaagtccttaaaaggaagaggcaacaccc	-	*
CY072234	Human H1N1pdm2009 IAVs	Human	H1N1	pdm	2009	Germany	A/Frankfurt/INS303/2009	ggtgatgccccattccttgatcggctccgccgagatcaaaagtccttaaaaggaagaggcaacaccc	-	*
CY083164	Human H1N1pdm2009 IAVs	Human	H1N1	pdm	2009	Dominican_Republic	A/Santo_Domingo/WRAIR1404T/2009	ggtgatgccccattccttgatcggctccgccgagatcaaaagtccttaaaaggaagaggcaacaccc	-	*
CY107285	Human H1N1pdm2009 IAVs	Human	H1N1	pdm	2009	United_Kingdom	A/Scotland/Aberdeen_3/2009	ggtgatgccccattccttgatcggctccgccgagatcaaaagtccttaaaaggaagaggcaacaccc	-	*
CY123234	Human H1N1pdm2009 IAVs	Human	H1N1	pdm	2009	Singapore	A/Singapore/GP4148/2009	ggtgatgccccattccttgatcggctccgccgagatcaaaagtccttaaaaggaagaggcaacaccc	-	*
KC781707	Human H1N1pdm2009 IAVs	Human	H1N1	pdm	2009	USA	A/Oregon/10/2009	ggtgatgccccattccttgatcggctccgccgagatcaaaagtccttaaaaggaagaggcaacaccc	-	*
HM567700	Human H1N1pdm2009 IAVs	Human	H1N1	pdm	2009	United_Kingdom	A/England/351/2009	ggtgatgccccattccttgatcggctccgccgagatcaaaagtccttaaaaggaagaggcaacaccc	-	*
HM567852	Human H1N1pdm2009 IAVs	Human	H1N1		2009	Ireland	A/Ireland/5/2009	ggtgatgccccattccttgatcggctccgccgagatcaaaagtccttaaaaggaagaggcaacaccc	-	*
CY045009	Human H1N1pdm2009 IAVs	Human	H1N1	pdm	2009	USA	A/New_York/3709/2009	ggtgatgccccattccttgatcggctccgccgagatcaaaagtccttaaaaggaagaggcaacaccc	-	*
CY061279	Human H1N1pdm2009 IAVs	Human	H1N1	pdm	2009	USA	A/Pensacola/INS107/2009	ggtgatgccccattccttgatcggctccgccgagatcaaaagtccttaaaaggaagaggcaacaccc	-	*
CY062304	Human H1N1pdm2009 IAVs	Swine	H1N1		2009	Thailand	A/swine/Thailand/CU_RA29/2009	ggtgatgccccattccttgatcggctccgccgagatcaaaagtccttaaaaggaagaggcaacaccc	-	*
CY062328	Human H1N1pdm2009 IAVs	Swine	H1N1		2009	Thailand	A/swine/Thailand/CU_RA9/2009	ggtgatgccccattccttgatcggctccgccgagatcaaaagtccttaaaaggaagaggcaacaccc	-	*
CY052523	Human H1N1pdm2009 IAVs	Human	H1N1	pdm	2009	USA	A/Texas/45104048/2009	ggtgatgccccattccttgatcggctccgccgagatcaaaagtccttaaaaggaagaggcaacaccc	-	*
CY055419	Human H1N1pdm2009 IAVs	Human	H1N1	pdm	2009	USA	A/Wisconsin/629_D00809/2009	ggtgatgccccattccttgatcggctccgccgagatcaaaagtccttaaaaggaagaggcaacaccc	-	*
CY087220	Human H1N1pdm2009 IAVs	Human	H1N1	pdm	2009	USA	A/Houston/21OS/2009	ggtgatgccccattccttgatcggctccgccgagatcaaaagtccttaaaaggaagaggcaacaccc	-	*
CY052371	Human H1N1pdm2009 IAVs	Human	H1N1	pdm	2009	USA	A/Texas/43272683/2009	ggtgatgccccattccttgatcggctccgccgagatcaaaagtccttaaaaggaagaggcaacaccc	-	*
HM567612	Human H1N1pdm2009 IAVs	Human	H1N1		2009	United_Kingdom	A/England/328/2009	ggtgatgccccattccttgatcggctccgccgagatcaaaagtccttaaaaggaagaggcaacaccc	-	*
HM567636	Human H1N1pdm2009 IAVs	Human	H1N1	pdm	2009	United_Kingdom	A/England/342/2009	ggtgatgccccattccttgatcggctccgccgagatcaaaagtccttaaaaggaagaggcaacaccc	-	*
HM567652	Human H1N1pdm2009 IAVs	Human	H1N1		2009	United_Kingdom	A/England/345/2009	ggtgatgccccattccttgatcggctccgccgagatcaaaagtccttaaaaggaagaggcaacaccc	-	*
HM567684	Human H1N1pdm2009 IAVs	Human	H1N1	pdm	2009	United_Kingdom	A/England/349/2009	ggtgatgccccattccttgatcggctccgccgagatcaaaagtccttaaaaggaagaggcaacaccc	-	*
HM567708	Human H1N1pdm2009 IAVs	Human	H1N1	pdm	2009	United_Kingdom	A/England/360/2009	ggtgatgccccattccttgatcggctccgccgagatcaaaagtccttaaaaggaagaggcaacaccc	-	*
HM567724	Human H1N1pdm2009 IAVs	Human	H1N1	pdm	2009	United_Kingdom	A/England/364/2009	ggtgatgccccattccttgatcggctccgccgagatcaaaagtccttaaaaggaagaggcaacaccc	-	*
HM567748	Human H1N1pdm2009 IAVs	Human	H1N1	pdm	2009	United_Kingdom	A/England/374/2009	ggtgatgccccattccttgatcggctccgccgagatcaaaagtccttaaaaggaagaggcaacaccc	-	*

HM567756	Human H1N1pdm2009 IAVs	Human	H1N1	pdm	2009	United_Kingdom	A/England/377/2009	ggtgatgccccattccttgatcggctccgccgagatca aaagtccttaaaaggaagaggcaacaccc	-	*
HM567796	Human H1N1pdm2009 IAVs	Human	H1N1	pdm	2009	United_Kingdom	A/England/399/2009	ggtgatgccccattccttgatcggctccgccgagatca aaagtccttaaaaggaagaggcaacaccc	-	*
HM567548	Human H1N1pdm2009 IAVs	Human	H1N1	pdm	2009	United_Kingdom	A/England/456/2009	ggtgatgccccattccttgatcggctccgccgagatca aaagtccttaaaaggaagaggcaacaccc	-	*
CY065538	Human H1N1pdm2009 IAVs	Human	H1N1	pdm	2009	United_Kingdom	A/England/359/2009	ggtgatgccccattccttgatcggctccgccgagatca aaagtccttaaaaggaagaggcaacaccc	-	*
CY065546	Human H1N1pdm2009 IAVs	Human	H1N1	pdm	2009	United_Kingdom	A/England/361/2009	ggtgatgccccattccttgatcggctccgccgagatca aaagtccttaaaaggaagaggcaacaccc	-	*
CY065570	Human H1N1pdm2009 IAVs	Human	H1N1	pdm	2009	United_Kingdom	A/England/384/2009	ggtgatgccccattccttgatcggctccgccgagatca aaagtccttaaaaggaagaggcaacaccc	-	*
CY065642	Human H1N1pdm2009 IAVs	Human	H1N1		2009	United_Kingdom	A/England/400/2009	ggtgatgccccattccttgatcggctccgccgagatca aaagtccttaaaaggaagaggcaacaccc	-	*
CY065722	Human H1N1pdm2009 IAVs	Human	H1N1		2009	United_Kingdom	A/England/433/2009	ggtgatgccccattccttgatcggctccgccgagatca aaagtccttaaaaggaagaggcaacaccc	-	*
CY065738	Human H1N1pdm2009 IAVs	Human	H1N1	pdm	2009	United_Kingdom	A/England/445/2009	ggtgatgccccattccttgatcggctccgccgagatca aaagtccttaaaaggaagaggcaacaccc	-	*
CY065586	Human H1N1pdm2009 IAVs	Human	H1N1	pdm	2009	United_Kingdom	A/England/386/2009	ggtgatgccccattccttgatcggctccgccgagatca aaagtccttaaaaggaagaggcaacaccc	-	*
CY065562	Human H1N1pdm2009 IAVs	Human	H1N1		2009	United_Kingdom	A/England/382/2009	ggtgatgccccattccttgatcggctccgccgagatca aaagtccttaaaaggaagaggcaacaccc	-	*
HM567676	Human H1N1pdm2009 IAVs	Human	H1N1	pdm	2009	United_Kingdom	A/England/348/2009	ggtgatgccccattccttgatcggctccgccgagatca aaagtccttaaaaggaagaggcaacaccc	-	*
CY045041	Human H1N1pdm2009 IAVs	Human	H1N1	pdm	2009	USA	A/New_York/3967/2009	ggtgatgccccattccttgatcggctccgccgagatca aaagtccttaaaaggaagaggcaacaccc	-	*
CY052126	Human H1N1pdm2009 IAVs	Human	H1N1	pdm	2009	USA	A/New_York/3969/2009	ggtgatgccccattccttgatcggctccgccgagatca aaagtccttaaaaggaagaggcaacaccc	-	*
CY050258	Human H1N1pdm2009 IAVs	Human	H1N1	pdm	2009	Mexico	A/Mexico_City/012/2009	ggtgatgccccattccttgatcggctccgccgagatca aaagtccttaaaaggaagaggcaacaccc	-	*
CY089207	Human H1N1pdm2009 IAVs	Human	H1N1	pdm	2009	USA	A/Boston/594/2009	ggtgatgccccattccttgatcggctccgccgagatca aaagtccttaaaaggaagaggcaacaccc	-	*
CY069150	Human H1N1pdm2009 IAVs	Human	H1N1	pdm	2009	Germany	A/Frankfurt/INS301/2009	ggtgatgccccattccttgatcggctccgccgagatca aaagtccttaaaaggaagaggcaacaccc	-	*
CY065087	Human H1N1pdm2009 IAVs	Human	H1N1	pdm	2009	USA	A/New_York/7424/2009	ggtgatgccccattccttgatcggctccgccgagatca aaagtccttaaaaggaagaggcaacaccc	-	*
KC780746	Human H1N1pdm2009 IAVs	Human	H1N1	pdm	2009	USA	A/Illinois/15/2009	ggtgatgccccattccttgatcggctccgccgagatca aaagtccttaaaaggaagaggcaacaccc	-	*
CY123528	Human H1N1pdm2009 IAVs	Human	H1N1	pdm	2009	Singapore	A/Singapore/ON1802/2009	ggtgatgccccattccttgatcggctccgccgagatca aaagtccttaaaaggaagaggcaacaccc	-	*
KC780026	Human H1N1pdm2009 IAVs	Human	H1N1	pdm	2009	USA	A/Wisconsin/67/2009	ggtgatgccccattccttgatcggctccgccgagatca aaagtccttaaaaggaagaggcaacaccc	-	*
KC780514	Human H1N1pdm2009 IAVs	Human	H1N1	pdm	2009	USA	A/North_Carolina/46/2009	ggtgatgccccattccttgatcggctccgccgagatca aaagtccttaaaaggaagaggcaacaccc	-	*
KC782325	Human H1N1pdm2009 IAVs	Human	H1N1	pdm	2009	USA	A/North_Carolina/46/2009	ggtgatgccccattccttgatcggctccgccgagatca aaagtccttaaaaggaagaggcaacaccc	-	*
JX875053	Human H1N1pdm2009 IAVs	Human	H1N1	pdm	2009	USA	A/Kentucky/110/2009	ggtgatgccccattccttgatcggctccgccgagatca aaagtccttaaaaggaagaggcaacaccc	-	*
CY095781	Human H1N1pdm2009 IAVs	Human	H1N1	pdm	2009	China	A/Zhejiang/4/2009	ggtgatgccccattccttgatcggctccgccgagatca aaagtccttaaaaggaagaggcaacaccc	-	*

CY095725	Human H1N1pdm2009 IAVs	Human	H1N1	pdm	2009	China	A/Zhejiang/X1/2009	ggtgatgccccattccttgatcggctccgccgagatcaaaagtccttaaaaggaagaggcaacaccc	-	*
CY095821	Human H1N1pdm2009 IAVs	Human	H1N1	pdm	2009	China	A/Zhejiang/5/2009	ggtgatgccccattccttgatcggctccgccgagatcaaaagtccttaaaaggaagaggcaacaccc	-	*
CY095909	Human H1N1pdm2009 IAVs	Human	H1N1	pdm	2009	China	A/Zhejiang/8/2009	ggtgatgccccattccttgatcggctccgccgagatcaaaagtccttaaaaggaagaggcaacaccc	-	*
CY096003	Human H1N1pdm2009 IAVs	Human	H1N1	pdm	2009	China	A/Zhejiang/92/2009	ggtgatgccccattccttgatcggctccgccgagatcaaaagtccttaaaaggaagaggcaacaccc	-	*
CY096041	Human H1N1pdm2009 IAVs	Human	H1N1	pdm	2009	China	A/Zhejiang/98/2009	ggtgatgccccattccttgatcggctccgccgagatcaaaagtccttaaaaggaagaggcaacaccc	-	*
KC781087	Human H1N1pdm2009 IAVs	Human	H1N1	pdm	2009	USA	A/Iowa/14/2009	ggtgatgccccattccttgatcggctccgccgagatcaaaagtccttaaaaggaagaggcaacaccc	-	*
KJ023092	Human H1N1pdm2009 IAVs	Human	H1N1		2009	India	A/Kerala/002/2009	ggtgatgccccattccttgatcggctccgccgagatcaaaagtccttaaaaggaagaggcaacaccc	-	*
KC780590	Human H1N1pdm2009 IAVs	Human	H1N1	pdm	2009	USA	A/Texas/75/2009	ggtgatgccccattccttgatcggctccgccgagatcaaaagtccttaaaaggaagaggcaacaccc	-	*
JX625463	Human H1N1pdm2009 IAVs	Human	H1N1	pdm	2009	United_Kingdom	A/England/688/2009	ggtgatgccccattccttgatcggctccgccgagatcaaaagtccttaaaaggaagaggcaacaccc	-	*
CY096049	Human H1N1pdm2009 IAVs	Human	H1N1	pdm	2009	China	A/Hubei/99/2009	ggtgatgccccattccttgatcggctccgccgagatcaaaagtccttaaaaggaagaggcaacaccc	-	*
CY095946	Human H1N1pdm2009 IAVs	Human	H1N1	pdm	2009	China	A/Zhejiang/84/2009	ggtgatgccccattccttgatcggctccgccgagatcaaaagtccttaaaaggaagaggcaacaccc	-	*
CY095967	Human H1N1pdm2009 IAVs	Human	H1N1	pdm	2009	China	A/Zhejiang/87/2009	ggtgatgccccattccttgatcggctccgccgagatcaaaagtccttaaaaggaagaggcaacaccc	-	*
CY058296	Human H1N1pdm2009 IAVs	Human	H1N1	pdm	2009	USA	A/Wisconsin/629_D02100/2009	ggtgatgccccattccttgatcggctccgccgagatcaaaagtccttaaaaggaagaggcaacaccc	-	*
CY089319	Human H1N1pdm2009 IAVs	Human	H1N1	pdm	2009	USA	A/Boston/678/2009	ggtgatgccccattccttgatcggctccgccgagatcaaaagtccttaaaaggaagaggcaacaccc	-	*
JX625509	Human H1N1pdm2009 IAVs	Human	H1N1	pdm	2009	United_Kingdom	A/England/94200009/2009	ggtgatgccccattccttgatcggctccgccgagatcaaaagtccttaaaaggaagaggcaacaccc	-	*
JX625618	Human H1N1pdm2009 IAVs	Human	H1N1	pdm	2009	United_Kingdom	A/Northern_Ireland/94940023/2009	ggtgatgccccattccttgatcggctccgccgagatcaaaagtccttaaaaggaagaggcaacaccc	-	*
HM568140	Human H1N1pdm2009 IAVs	Human	H1N1	pdm	2009	United_Kingdom	A/England/H093780041/2009	ggtgatgccccattccttgatcggctccgccgagatcaaaagtccttaaaaggaagaggcaacaccc	-	*
CY075560	Human H1N1pdm2009 IAVs	Human	H1N1	pdm	2009	USA	A/Boston/653/2009	ggtgatgccccattccttgatcggctccgccgagatcaaaagtccttaaaaggaagaggcaacaccc	-	*
CY083643	Human H1N1pdm2009 IAVs	Human	H1N1	pdm	2009	USA	A/San_Diego/INS13/2009	ggtgatgccccattccttgatcggctccgccgagatcaaaagtccttaaaaggaagaggcaacaccc	-	*
CY066555	Human H1N1pdm2009 IAVs	Human	H1N1	pdm	2009	USA	A/San_Diego/INS198/2009	ggtgatgccccattccttgatcggctccgccgagatcaaaagtccttaaaaggaagaggcaacaccc	-	*
CY056887	Human H1N1pdm2009 IAVs	Human	H1N1	pdm	2009	USA	A/San_Diego/INS54/2009	ggtgatgccccattccttgatcggctccgccgagatcaaaagtccttaaaaggaagaggcaacaccc	-	*
CY056863	Human H1N1pdm2009 IAVs	Human	H1N1	pdm	2009	USA	A/San_Diego/INS15/2009	ggtgatgccccattccttgatcggctccgccgagatcaaaagtccttaaaaggaagaggcaacaccc	-	*
CY122615	Human H1N1pdm2009 IAVs	Human	H1N1	pdm	2009	Singapore	A/Singapore/GP1099/2009	ggtgatgccccattccttgatcggctccgccgagatcaaaagtccttaaaaggaagaggcaacaccc	-	*
CY123386	Human H1N1pdm2009 IAVs	Human	H1N1	pdm	2009	Singapore	A/Singapore/ON1063/2009	ggtgatgccccattccttgatcggctccgccgagatcaaaagtccttaaaaggaagaggcaacaccc	-	*
CY056807	Human H1N1pdm2009 IAVs	Human	H1N1	pdm	2009	USA	A/New_York/6808/2009	ggtgatgccccattccttgatcggctccgccgagatcaaaagtccttaaaaggaagaggcaacaccc	-	*

CY061966	Human H1N1pdm2009 IAVs	Human	H1N1	pdm	2009	USA	A/New_York/6949/2009	ggtgatgccccattccttgatcggctccgccgagatca aaagtccttaaaaggaagaggcaacaccc	-	*
CY061974	Human H1N1pdm2009 IAVs	Human	H1N1	pdm	2009	USA	A/New_York/6976/2009	ggtgatgccccattccttgatcggctccgccgagatca aaagtccttaaaaggaagaggcaacaccc	-	*
CY052611	Human H1N1pdm2009 IAVs	Human	H1N1	pdm	2009	USA	A/Texas/45034157/2009	ggtgatgccccattccttgatcggctccgccgagatca aaagtccttaaaaggaagaggcaacaccc	-	*
CY087196	Human H1N1pdm2009 IAVs	Human	H1N1	pdm	2009	USA	A/Texas/45034240/2009	ggtgatgccccattccttgatcggctccgccgagatca aaagtccttaaaaggaagaggcaacaccc	-	*
CY083784	Human H1N1pdm2009 IAVs	Human	unknow	pdm	2009	Germany	A/Cologne/INS285/2009	ggtgatgccccattccttgatcggctccgccgagatca aaagtccttaaaaggaagaggcaacaccc	-	*
CY069246	Human H1N1pdm2009 IAVs	Human	H1N1	pdm	2009	Austria	A/Vienna/INS366/2009	ggtgatgccccattccttgatcggctccgccgagatca aaagtccttaaaaggaagaggcaacaccc	-	*
CY061753	Human H1N1pdm2009 IAVs	Swine	H1N1		2009	Hong_Kong	A/swine/Hong_Kong/NS1809/2009	ggtgatgccccattccttgatcggctccgccgagatca aaagtccttaaaaggaagaggcaacaccc	-	*
CY061761	Human H1N1pdm2009 IAVs	Swine	H1N1		2009	Hong_Kong	A/swine/Hong_Kong/NS1810/2009	ggtgatgccccattccttgatcggctccgccgagatca aaagtccttaaaaggaagaggcaacaccc	-	*
JF714104	Human H1N1pdm2009 IAVs	Swine	H1N1		2009	Canada	A/swine/QC/3974_5/2009	ggtgatgccccattccttgatcggctccgccgagatca aaagtccttaaaaggaagaggcaacaccc	-	*
GU324343	Human H1N1pdm2009 IAVs	Swine	H1N1		2009	Taiwan	A/swine/Taiwan/TD_4119B2/2009	ggtgatgccccattccttgatcggctccgccgagatca aaagtccttaaaaggaagaggcaacaccc	-	*
JN187333	Human H1N1pdm2009 IAVs	Human	H1N1	pdm	2009	Taiwan	A/Taiwan/4909/2009	ggtgatgccccattccttgatcggctccgccgagatca aaagtccttaaaaggaagaggcaacaccc	-	*
CY056759	Human H1N1pdm2009 IAVs	Human	H1N1	pdm	2009	USA	A/New_York/6663/2009	ggtgatgccccattccttgatcggctccgccgagatca aaagtccttaaaaggaagaggcaacaccc	-	*
CY128351	Human H1N1pdm2009 IAVs	Human	H1N1	pdm	2009	Viet_Nam	A/Viet_Nam/13032100/2009	ggtgatgccccattccttgatcggctccgccgagatca aaagtccttaaaaggaagaggcaacaccc	-	*
CY128391	Human H1N1pdm2009 IAVs	Human	H1N1	pdm	2009	Viet_Nam	A/Viet_Nam/11032010/2009	ggtgatgccccattccttgatcggctccgccgagatca aaagtccttaaaaggaagaggcaacaccc	-	*
CY128399	Human H1N1pdm2009 IAVs	Human	H1N1	pdm	2009	Viet_Nam	A/Viet_Nam/11032016/2009	ggtgatgccccattccttgatcggctccgccgagatca aaagtccttaaaaggaagaggcaacaccc	-	*
CY128423	Human H1N1pdm2009 IAVs	Human	H1N1	pdm	2009	Viet_Nam	A/Viet_Nam/12032001/2009	ggtgatgccccattccttgatcggctccgccgagatca aaagtccttaaaaggaagaggcaacaccc	-	*
CY061055	Human H1N1pdm2009 IAVs	Human	H1N1	pdm	2009	USA	A/Texas/JMS392/2009	ggtgatgccccattccttgatcggctccgccgagatca aaagtccttaaaaggaagaggcaacaccc	-	*
CY060863	Human H1N1pdm2009 IAVs	Human	H1N1	pdm	2009	USA	A/Texas/JMS359/2009	ggtgatgccccattccttgatcggctccgccgagatca aaagtccttaaaaggaagaggcaacaccc	-	*
CY087204	Human H1N1pdm2009 IAVs	Human	H1N1	pdm	2009	USA	A/Texas/JMS360/2009	ggtgatgccccattccttgatcggctccgccgagatca aaagtccttaaaaggaagaggcaacaccc	-	*
KC781070	Human H1N1pdm2009 IAVs	Human	H1N1	pdm	2009	USA	A/Oklahoma/05/2009	ggtgatgccccattccttgatcggctccgccgagatca aaagtccttaaaaggaagaggcaacaccc	-	*
CY096027	Human H1N1pdm2009 IAVs	Human	H1N1	pdm	2009	China	A/Zhejiang/96/2009	ggtgatgccccattccttgatcggctccgccgagatca aaagtccttaaaaggaagaggcaacaccc	-	*
GU367336	Human H1N1pdm2009 IAVs	Human	H1N1	pdm	2009	Kazakhstan	A/Almati/01/2009	ggtgatgccccattccttgatcggctccgccgagatca aaagtccttaaaaggaagaggcaacaccc	-	*
JX625479	Human H1N1pdm2009 IAVs	Human	H1N1	pdm	2009	United_Kingdom	A/England/646/2009	ggtgatgccccattccttgatcggctccgccgagatca aaagtccttaaaaggaagaggcaacaccc	-	*
HM568068	Human H1N1pdm2009 IAVs	Human	H1N1	pdm	2009	United_Kingdom	A/England/H094240645/2009	ggtgatgccccattccttgatcggctccgccgagatca aaagtccttaaaaggaagaggcaacaccc	-	*
HM568116	Human H1N1pdm2009 IAVs	Human	H1N1	pdm	2009	United_Kingdom	A/England/798/2009	ggtgatgccccattccttgatcggctccgccgagatca aaagtccttaaaaggaagaggcaacaccc	-	*

HM567956	Human H1N1pdm2009 IAVs	Human	H1N1	pdm	2009	United_Kingdom	A/England/857/2009	ggtgatgccccattccttgatcggctccgccgagatca aaagtccttaaaaggaagaggcaacaccc	-	*
CY107517	Human H1N1pdm2009 IAVs	Human	H1N1	pdm	2009	United_Kingdom	A/Scotland/Edinburgh_20342/2009	ggtgatgccccattccttgatcggctccgccgagatca aaagtccttaaaaggaagaggcaacaccc	-	*
CY045081	Human H1N1pdm2009 IAVs	Human	H1N1	pdm	2009	USA	A/New_York/4072/2009	ggtgatgccccattccttgatcggctccgccgagatca aaagtccttaaaaggaagaggcaacaccc	-	*
CY060871	Human H1N1pdm2009 IAVs	Human	H1N1	pdm	2009	USA	A/Texas/JMS361/2009	ggtgatgccccattccttgatcggctccgccgagatca aaagtccttaaaaggaagaggcaacaccc	-	*
CY083571	Human H1N1pdm2009 IAVs	Human	H1N1	pdm	2009	Peru	A/Lima/WRAIR8511F/2009	ggtgatgccccattccttgatcggctccgccgagatca aaagtccttaaaaggaagaggcaacaccc	-	*
KC780675	Human H1N1pdm2009 IAVs	Human	H1N1	pdm	2009	USA	A/West_Virginia/13/2009	ggtgatgccccattccttgatcggctccgccgagatca aaagtccttaaaaggaagaggcaacaccc	-	*
GU592901	Human H1N1pdm2009 IAVs	Human	H1N1	pdm	2009	Kyrgyzstan	A/Bishkek/03/2009	ggtgatgccccattccttgatcggctccgccgagatca aaagtccttaaaaggaagaggcaacaccc	-	*
CY056535	Human H1N1pdm2009 IAVs	Human	H1N1	pdm	2009	USA	A/New_York/5141/2009	ggtgatgccccattccttgatcggctccgccgagatca aaagtccttaaaaggaagaggcaacaccc	-	*
CY053756	Human H1N1pdm2009 IAVs	Human	H1N1	pdm	2009	Russia	A/Russia/200/2009	ggtgatgccccattccttgatcggctccgccgagatca aaagtccttaaaaggaagaggcaacaccc	-	*
CY083780	Human H1N1pdm2009 IAVs	Human	H1N1	pdm	2009	Denmark	A/Aalborg/INS282/2009	ggtgatgccccattccttgatcggctccgccgagatca aaagtccttaaaaggaagaggcaacaccc	-	*
CY062695	Human H1N1pdm2009 IAVs	Human	H1N1	pdm	2009	Denmark	A/Aarhus/INS116/2009	ggtgatgccccattccttgatcggctccgccgagatca aaagtccttaaaaggaagaggcaacaccc	-	*
CY067059	Human H1N1pdm2009 IAVs	Human	H1N1	pdm	2009	Denmark	A/Aarhus/INS254/2009	ggtgatgccccattccttgatcggctccgccgagatca aaagtccttaaaaggaagaggcaacaccc	-	*
CY056951	Human H1N1pdm2009 IAVs	Human	H1N1	pdm	2009	Denmark	A/Aarhus/INS82/2009	ggtgatgccccattccttgatcggctccgccgagatca aaagtccttaaaaggaagaggcaacaccc	-	*
CY073092	Human H1N1pdm2009 IAVs	Human	H1N1		2009	Turkey	A/Ankara/WRAIR1435T/2009	ggtgatgccccattccttgatcggctccgccgagatca aaagtccttaaaaggaagaggcaacaccc	-	*
HM189324	Human H1N1pdm2009 IAVs	Human	H1N1	pdm	2009	Russia	A/Arkhangelsk/CRIE_GNY/2009	ggtgatgccccattccttgatcggctccgccgagatca aaagtccttaaaaggaagaggcaacaccc	-	*
CY062663	Human H1N1pdm2009 IAVs	Human	H1N1	pdm	2009	Greece	A/Athens/INS87/2009	ggtgatgccccattccttgatcggctccgccgagatca aaagtccttaaaaggaagaggcaacaccc	-	*
CY066995	Human H1N1pdm2009 IAVs	Human	H1N1	pdm	2009	Belgium	A/Brussels/INS245/2009	ggtgatgccccattccttgatcggctccgccgagatca aaagtccttaaaaggaagaggcaacaccc	-	*
CY066667	Human H1N1pdm2009 IAVs	Human	H1N1	pdm	2009	Germany	A/Cologne/INS286/2009	ggtgatgccccattccttgatcggctccgccgagatca aaagtccttaaaaggaagaggcaacaccc	-	*
CY073745	Human H1N1pdm2009 IAVs	Human	H1N1	pdm	2009	Denmark	A/Copenhagen/INS433/2009	ggtgatgccccattccttgatcggctccgccgagatca aaagtccttaaaaggaagaggcaacaccc	-	*
CY056983	Human H1N1pdm2009 IAVs	Human	H1N1	pdm	2009	Denmark	A/Copenhagen/INS98/2009	ggtgatgccccattccttgatcggctccgccgagatca aaagtccttaaaaggaagaggcaacaccc	-	*
JX625564	Human H1N1pdm2009 IAVs	Human	H1N1	pdm	2009	United_Kingdom	A/England/94560024/2009	ggtgatgccccattccttgatcggctccgccgagatca aaagtccttaaaaggaagaggcaacaccc	-	*
CY062791	Human H1N1pdm2009 IAVs	Human	H1N1	pdm	2009	Denmark	A/Hvidovre/INS139/2009	ggtgatgccccattccttgatcggctccgccgagatca aaagtccttaaaaggaagaggcaacaccc	-	*
HM189356	Human H1N1pdm2009 IAVs	Human	H1N1	pdm	2009	Russia	A/Lipetsk/CRIE_BVV/2009	ggtgatgccccattccttgatcggctccgccgagatca aaagtccttaaaaggaagaggcaacaccc	-	*
CY067011	Human H1N1pdm2009 IAVs	Human	H1N1	pdm	2009	Germany	A/Munich/INS247/2009	ggtgatgccccattccttgatcggctccgccgagatca aaagtccttaaaaggaagaggcaacaccc	-	*
CY065868	Human H1N1pdm2009 IAVs	Human	H1N1	pdm	2009	Netherlands	A/Netherlands/1493b/2009	ggtgatgccccattccttgatcggctccgccgagatca aaagtccttaaaaggaagaggcaacaccc	-	*

CY057258	Human H1N1pdm2009 IAVs	Human	H1N1	pdm	2009	USA	A/New_York/5186/2009	ggtgatgccccattccttgatcggctccgccgagatca aaagtccttaaaaggaagaggcaacaccc	-	*
JX625595	Human H1N1pdm2009 IAVs	Human	H1N1	pdm	2009	United_Kingdom	A/Northern_Ireland/94620068/2009	ggtgatgccccattccttgatcggctccgccgagatca aaagtccttaaaaggaagaggcaacaccc	-	*
GQ527171	Human H1N1pdm2009 IAVs	Human	H1N1	pdm	2009	Russia	A/Omsk/01/2009	ggtgatgccccattccttgatcggctccgccgagatca aaagtccttaaaaggaagaggcaacaccc	-	*
CY083709	Human H1N1pdm2009 IAVs	Human	H1N1	pdm	2009	Norway	A/Oslo/INS111/2009	ggtgatgccccattccttgatcggctccgccgagatca aaagtccttaaaaggaagaggcaacaccc	-	*
CY071227	Human H1N1pdm2009 IAVs	Human	H1N1	pdm	2009	Norway	A/Oslo/INS404/2009	ggtgatgccccattccttgatcggctccgccgagatca aaagtccttaaaaggaagaggcaacaccc	-	*
CY054650	Human H1N1pdm2009 IAVs	Human	H1N1	pdm	2009	Russia	A/Russia/19/2009	ggtgatgccccattccttgatcggctccgccgagatca aaagtccttaaaaggaagaggcaacaccc	-	*
CY107278	Human H1N1pdm2009 IAVs	Human	H1N1	pdm	2009	United_Kingdom	A/Scotland/Aberdeen_2/2009	ggtgatgccccattccttgatcggctccgccgagatca aaagtccttaaaaggaagaggcaacaccc	-	*
CY107222	Human H1N1pdm2009 IAVs	Human	H1N1		2009	United_Kingdom	A/Scotland/Inverness_10/2009	ggtgatgccccattccttgatcggctccgccgagatca aaagtccttaaaaggaagaggcaacaccc	-	*
CY062999	Human H1N1pdm2009 IAVs	Human	H1N1	pdm	2009	Estonia	A/Tallinn/INS181/2009	ggtgatgccccattccttgatcggctccgccgagatca aaagtccttaaaaggaagaggcaacaccc	-	*
JX625532	Human H1N1pdm2009 IAVs	Human	H1N1		2009	United_Kingdom	A/Northern_Ireland/94480397/2009	ggtgatgccccattccttgatcggctccgccgagatca aaagtccttaaaaggaagaggcaacaccc	-	*
HM173603	Human H1N1pdm2009 IAVs	Human	H1N1	pdm	2009	Russia	A/Blagovechensk/01/2009	ggtgatgccccattccttgatcggctccgccgagatca aaagtccttaaaaggaagaggcaacaccc	-	*
HM568100	Human H1N1pdm2009 IAVs	Human	H1N1	pdm	2009	United_Kingdom	A/England/766/2009	ggtgatgccccattccttgatcggctccgccgagatca aaagtccttaaaaggaagaggcaacaccc	-	*
HM567988	Human H1N1pdm2009 IAVs	Human	H1N1	pdm	2009	United_Kingdom	A/England/869/2009	ggtgatgccccattccttgatcggctccgccgagatca aaagtccttaaaaggaagaggcaacaccc	-	*
HM568124	Human H1N1pdm2009 IAVs	Human	H1N1	pdm	2009	United_Kingdom	A/England/941/2009	ggtgatgccccattccttgatcggctccgccgagatca aaagtccttaaaaggaagaggcaacaccc	-	*
HM568156	Human H1N1pdm2009 IAVs	Human	H1N1	pdm	2009	United_Kingdom	A/England/H093900113/2009	ggtgatgccccattccttgatcggctccgccgagatca aaagtccttaaaaggaagaggcaacaccc	-	*
CY053788	Human H1N1pdm2009 IAVs	Human	H1N1	pdm	2009	Norway	A/Norway/2924/2009	ggtgatgccccattccttgatcggctccgccgagatca aaagtccttaaaaggaagaggcaacaccc	-	*
CY052002	Human H1N1pdm2009 IAVs	Human	H1N1		2009	Norway	A/Norway/3364_2/2009	ggtgatgccccattccttgatcggctccgccgagatca aaagtccttaaaaggaagaggcaacaccc	-	*
GU211239	Human H1N1pdm2009 IAVs	Human	H1N1	pdm	2009	Russia	A/Omsk/02/2009	ggtgatgccccattccttgatcggctccgccgagatca aaagtccttaaaaggaagaggcaacaccc	-	*
GU562454	Human H1N1pdm2009 IAVs	Human	H1N1	pdm	2009	Russia	A/Perm/01/2009	ggtgatgccccattccttgatcggctccgccgagatca aaagtccttaaaaggaagaggcaacaccc	-	*
GU451260	Human H1N1pdm2009 IAVs	Human	H1N1	pdm	2009	Russia	A/Tver/IIV2969/2009	ggtgatgccccattccttgatcggctccgccgagatca aaagtccttaaaaggaagaggcaacaccc	-	*
CY065812	Human H1N1pdm2009 IAVs	Human	H1N1		2009	Netherlands	A/Netherlands/1715b/2009	ggtgatgccccattccttgatcggctccgccgagatca aaagtccttaaaaggaagaggcaacaccc	-	*
HM855262	Human H1N1pdm2009 IAVs	Human	H1N1		2009	Kenya	A/Eldoret/119/2009	ggtgatgccccattccttgatcggctccgccgagatca aaagtccttaaaaggaagaggcaacaccc	-	*
CY071430	Human H1N1pdm2009 IAVs	Human	H1N1	pdm	2009	Turkmenistan	A/Ashgabat/WR0865N/2009	ggtgatgccccattccttgatcggctccgccgagatca aaagtccttaaaaggaagaggcaacaccc	-	*
JF327343	Human H1N1pdm2009 IAVs	Human	H1N1	pdm	2009	Finland	A/Finland/649/2009	ggtgatgccccattccttgatcggctccgccgagatca aaagtccttaaaaggaagaggcaacaccc	-	*
JF327345	Human H1N1pdm2009 IAVs	Human	H1N1	pdm	2009	Finland	A/Finland/688/2009	ggtgatgccccattccttgatcggctccgccgagatca aaagtccttaaaaggaagaggcaacaccc	-	*

CY083922	Human H1N1pdm2009 IAVs	Human	H1N1		2009	Poland	A/Warsaw/INS147/2009	ggtgatgccccattccttgatcggctccgccgagatca aaagtccttaaaaggaagaggcaacaccc	-	*
CY053693	Human H1N1pdm2009 IAVs	Human	H1N1	pdm	2009	Russia	A/Russia/191/2009	ggtgatgccccattccttgatcggctccgccgagatca aaagtccttaaaaggaagaggcaacaccc	-	*
CY053685	Human H1N1pdm2009 IAVs	Human	H1N1	pdm	2009	Russia	A/Russia/14/2009	ggtgatgccccattccttgatcggctccgccgagatca aaagtccttaaaaggaagaggcaacaccc	-	*
CY054634	Human H1N1pdm2009 IAVs	Human	H1N1	pdm	2009	Russia	A/Russia/4/2009	ggtgatgccccattccttgatcggctccgccgagatca aaagtccttaaaaggaagaggcaacaccc	-	*
CY107731	Human H1N1pdm2009 IAVs	Human	H1N1	pdm	2009	United_Kingdom	A/Scotland/Glasgow_444809/2009	ggtgatgccccattccttgatcggctccgccgagatca aaagtccttaaaaggaagaggcaacaccc	-	*
CY054642	Human H1N1pdm2009 IAVs	Human	H1N1		2009	Russia	A/Russia/12/2009	ggtgatgccccattccttgatcggctccgccgagatca aaagtccttaaaaggaagaggcaacaccc	-	*
CY067155	Human H1N1pdm2009 IAVs	Human	H1N1	pdm	2009	Greece	A/Athens/INS270/2009	ggtgatgccccattccttgatcggctccgccgagatca aaagtccttaaaaggaagaggcaacaccc	-	*
CY072402	Human H1N1pdm2009 IAVs	Human	H1N1		2009	Greece	A/Athens/INS333/2009	ggtgatgccccattccttgatcggctccgccgagatca aaagtccttaaaaggaagaggcaacaccc	-	*
CY072442	Human H1N1pdm2009 IAVs	Human	H1N1		2009	Greece	A/Athens/INS338/2009	ggtgatgccccattccttgatcggctccgccgagatca aaagtccttaaaaggaagaggcaacaccc	-	*
CY066651	Human H1N1pdm2009 IAVs	Human	H1N1	pdm	2009	Germany	A/Bonn/INS279/2009	ggtgatgccccattccttgatcggctccgccgagatca aaagtccttaaaaggaagaggcaacaccc	-	*
CY065119	Human H1N1pdm2009 IAVs	Human	H1N1	pdm	2009	Netherlands	A/Netherlands/2464b/2009	ggtgatgccccattccttgatcggctccgccgagatca aaagtccttaaaaggaagaggcaacaccc	-	*
CY056959	Human H1N1pdm2009 IAVs	Human	H1N1	pdm	2009	Greece	A/Athens/INS85/2009	ggtgatgccccattccttgatcggctccgccgagatca aaagtccttaaaaggaagaggcaacaccc	-	*
JQ253797	Human H1N1pdm2009 IAVs	Swine	H1N1		2009	Norway	A/swine/Norway/02_11342/2009	ggtgatgccccattccttgatcggctccgccgagatca aaagtccttaaaaggaagaggcaacaccc	-	*
HM568132	Human H1N1pdm2009 IAVs	Human	H1N1		2009	United_Kingdom	A/England/938/2009	ggtgatgccccattccttgatcggctccgccgagatca aaagtccttaaaaggaagaggcaacaccc	-	*
CY116342	Human H1N1pdm2009 IAVs	Swine	H1N1		2009	United_Kingdom	A/swine/England/MD0040352R/2009	ggtgatgccccattccttgatcggctccgccgagatca aaagtccttaaaaggaagaggcaacaccc	-	*
CY052379	Human H1N1pdm2009 IAVs	Human	H1N1	pdm	2009	USA	A/Texas/43200999/2009	ggtgatgccccattccttgatcggctccgccgagatca aaagtccttaaaaggaagaggcaacaccc	-	*
JQ173168	Human H1N1pdm2009 IAVs	Human	H1N1	pdm	2009	Finland	A/Helsinki/P18/2009	ggtgatgccccattccttgatcggctccgccgagatca aaagtccttaaaaggaagaggcaacaccc	-	*
CY115866	Human H1N1pdm2009 IAVs	Human	H1N1	pdm	2009	Netherlands	A/Netherlands/947b/2009	ggtgatgccccattccttgatcggctccgccgagatca aaagtccttaaaaggaagaggcaacaccc	-	*
CY063586	Human H1N1pdm2009 IAVs	Human	H1N1	pdm	2009	Norway	A/Oslo/INS110/2009	ggtgatgccccattccttgatcggctccgccgagatca aaagtccttaaaaggaagaggcaacaccc	-	*
CY072274	Human H1N1pdm2009 IAVs	Human	H1N1	pdm	2009	Poland	A/Warsaw/INS311/2009	ggtgatgccccattccttgatcggctccgccgagatca aaagtccttaaaaggaagaggcaacaccc	-	*
CY072554	Human H1N1pdm2009 IAVs	Human	H1N1	pdm	2009	Poland	A/Wroclaw/INS437/2009	ggtgatgccccattccttgatcggctccgccgagatca aaagtccttaaaaggaagaggcaacaccc	-	*
CY053631	Human H1N1pdm2009 IAVs	Human	H1N1	pdm	2009	Russia	A/Russia/165/2009	ggtgatgccccattccttgatcggctccgccgagatca aaagtccttaaaaggaagaggcaacaccc	-	*
GU367331	Human H1N1pdm2009 IAVs	Human	H1N1	pdm	2009	Russia	A/Salekhard/01/2009	ggtgatgccccattccttgatcggctccgccgagatca aaagtccttaaaaggaagaggcaacaccc	-	*
CY072306	Human H1N1pdm2009 IAVs	Human	H1N1	pdm	2009	Poland	A/Warsaw/INS315/2009	ggtgatgccccattccttgatcggctccgccgagatca aaagtccttaaaaggaagaggcaacaccc	-	*
CY055435	Human H1N1pdm2009 IAVs	Human	H1N1	pdm	2009	USA	A/New_York/4977/2009	ggtgatgccccattccttgatcggctccgccgagatca aaagtccttaaaaggaagaggcaacaccc	-	*

CY056567	Human H1N1pdm2009 IAVs	Human	H1N1	pdm	2009	USA	A/New_York/5227/2009	ggtgatgccccattccttgatcggctccgccgagatca aaagtccttaaaaggaagaggcaacaccc	-	*
CY065828	Human H1N1pdm2009 IAVs	Human	H1N1	pdm	2009	Netherlands	A/Netherlands/2243/2009	ggtgatgccccattccttgatcggctccgccgagatca aaagtccttaaaaggaagaggcaacaccc	-	*
CY065844	Human H1N1pdm2009 IAVs	Human	H1N1	pdm	2009	Netherlands	A/Netherlands/2636/2009	ggtgatgccccattccttgatcggctccgccgagatca aaagtccttaaaaggaagaggcaacaccc	-	*
CY067655	Human H1N1pdm2009 IAVs	Human	H1N1	pdm	2009	Belgium	A/Brussels/INS244/2009	ggtgatgccccattccttgatcggctccgccgagatca aaagtccttaaaaggaagaggcaacaccc	-	*
CY073753	Human H1N1pdm2009 IAVs	Human	H1N1	pdm	2009	Denmark	A/Copenhagen/INS434/2009	ggtgatgccccattccttgatcggctccgccgagatca aaagtccttaaaaggaagaggcaacaccc	-	*
CY062799	Human H1N1pdm2009 IAVs	Human	H1N1	pdm	2009	Denmark	A/Hvidovre/INS141/2009	ggtgatgccccattccttgatcggctccgccgagatca aaagtccttaaaaggaagaggcaacaccc	-	*
CY067003	Human H1N1pdm2009 IAVs	Human	H1N1	pdm	2009	Germany	A/Munich/INS246/2009	ggtgatgccccattccttgatcggctccgccgagatca aaagtccttaaaaggaagaggcaacaccc	-	*
CY065836	Human H1N1pdm2009 IAVs	Human	H1N1	pdm	2009	Netherlands	A/Netherlands/2457b/2009	ggtgatgccccattccttgatcggctccgccgagatca aaagtccttaaaaggaagaggcaacaccc	-	*
HM567964	Human H1N1pdm2009 IAVs	Human	H1N1	pdm	2009	United_Kingdom	A/England/865/2009	ggtgatgccccattccttgatcggctccgccgagatca aaagtccttaaaaggaagaggcaacaccc	-	*
GU592893	Human H1N1pdm2009 IAVs	Human	H1N1	pdm	2009	Russia	A/Barnaul/04/2009	ggtgatgccccattccttgatcggctccgccgagatca aaagtccttaaaaggaagaggcaacaccc	-	*
GU592909	Human H1N1pdm2009 IAVs	Human	H1N1	pdm	2009	Russia	A/Magadan/02/2009	ggtgatgccccattccttgatcggctccgccgagatca aaagtccttaaaaggaagaggcaacaccc	-	*
GU371268	Human H1N1pdm2009 IAVs	Human	H1N1	pdm	2009	Russia	A/Bryansk/IIV2971/2009	ggtgatgccccattccttgatcggctccgccgagatca aaagtccttaaaaggaagaggcaacaccc	-	*
JX625524	Human H1N1pdm2009 IAVs	Human	H1N1	pdm	2009	United_Kingdom	A/England/94400003/2009	ggtgatgccccattccttgatcggctccgccgagatca aaagtccttaaaaggaagaggcaacaccc	-	*
JX625572	Human H1N1pdm2009 IAVs	Human	H1N1		2009	United_Kingdom	A/Northern_Ireland/1/2009	ggtgatgccccattccttgatcggctccgccgagatca aaagtccttaaaaggaagaggcaacaccc	-	*
HM855260	Human H1N1pdm2009 IAVs	Human	H1N1	pdm	2009	Kenya	A/Garissa/79/2009	ggtgatgccccattccttgatcggctccgccgagatca aaagtccttaaaaggaagaggcaacaccc	-	*
GU727824	Human H1N1pdm2009 IAVs	Human	H1N1	pdm	2009	Russia	A/IIV_Anadyr/177/2009	ggtgatgccccattccttgatcggctccgccgagatca aaagtccttaaaaggaagaggcaacaccc	-	*
CY056991	Human H1N1pdm2009 IAVs	Human	H1N1	pdm	2009	Poland	A/Warsaw/INS100/2009	ggtgatgccccattccttgatcggctccgccgagatca aaagtccttaaaaggaagaggcaacaccc	-	*
CY072290	Human H1N1pdm2009 IAVs	Human	H1N1	pdm	2009	Poland	A/Warsaw/INS313/2009	ggtgatgccccattccttgatcggctccgccgagatca aaagtccttaaaaggaagaggcaacaccc	-	*
CY053420	Human H1N1pdm2009 IAVs	Human	H1N1	pdm	2009	Russia	A/Russia/149/2009	ggtgatgccccattccttgatcggctccgccgagatca aaagtccttaaaaggaagaggcaacaccc	-	*
CY071390	Human H1N1pdm2009 IAVs	Human	H1N1	pdm	2009	Jordan	A/Amman/WR0060N/2009	ggtgatgccccattccttgatcggctccgccgagatca aaagtccttaaaaggaagaggcaacaccc	-	*
CY053359	Human H1N1pdm2009 IAVs	Human	H1N1	pdm	2009	China	A/Beijing/739/2009	ggtgatgccccattccttgatcggctccgccgagatca aaagtccttaaaaggaagaggcaacaccc	-	*
HM104480	Human H1N1pdm2009 IAVs	Human	H1N1	pdm	2009	Russia	A/Yaroslavl/IIV_198/2009	ggtgatgccccattccttgatcggctccgccgagatca aaagtccttaaaaggaagaggcaacaccc	-	*
GU480947	Human H1N1pdm2009 IAVs	Human	H1N1	pdm	2009	Russia	A/Kurgan/01/2009	ggtgatgccccattccttgatcggctccgccgagatca aaagtccttaaaaggaagaggcaacaccc	-	*
CY071235	Human H1N1pdm2009 IAVs	Human	H1N1	pdm	2009	Poland	A/Warsaw/INS405/2009	ggtgatgccccattccttgatcggctccgccgagatca aaagtccttaaaaggaagaggcaacaccc	-	*
CY071243	Human H1N1pdm2009 IAVs	Human	H1N1	pdm	2009	Poland	A/Warsaw/INS406/2009	ggtgatgccccattccttgatcggctccgccgagatca aaagtccttaaaaggaagaggcaacaccc	-	*

CY065900	Human H1N1pdm2009 IAVs	Human	H1N1	pdm	2009	Netherlands	A/Netherlands/2822/2009	ggtgatgccccattccttgatcggctccgccgagatcaaaagtccttaaaaggaagaggcaacaccc	-	*
CY054674	Human H1N1pdm2009 IAVs	Human	H1N1	pdm	2009	Russia	A/Russia/171/2009	ggtgatgccccattccttgatcggctccgccgagatcaaaagtccttaaaaggaagaggcaacaccc	-	*
CY053740	Human H1N1pdm2009 IAVs	Human	H1N1	pdm	2009	Russia	A/Russia/178/2009	ggtgatgccccattccttgatcggctccgccgagatcaaaagtccttaaaaggaagaggcaacaccc	-	*
CY053748	Human H1N1pdm2009 IAVs	Human	H1N1	pdm	2009	Russia	A/Russia/190/2009	ggtgatgccccattccttgatcggctccgccgagatcaaaagtccttaaaaggaagaggcaacaccc	-	*
JX403982	Human H1N1pdm2009 IAVs	Human	H1N1		2009	China	A/Liaoning/1/2009	ggtgatgccccattccttgatcggctccgccgagatcaaaagtccttaaaaggaagaggcaacaccc	-	*
KC683499	Human H1N1pdm2009 IAVs	Human	H1N1	pdm	2009	China	A/Liaoning/14/2009	ggtgatgccccattccttgatcggctccgccgagatcaaaagtccttaaaaggaagaggcaacaccc	-	*
CY096009	Human H1N1pdm2009 IAVs	Human	H1N1	pdm	2009	China	A/Zhejiang/93/2009	ggtgatgccccattccttgatcggctccgccgagatcaaaagtccttaaaaggaagaggcaacaccc	-	*
CY061950	Human H1N1pdm2009 IAVs	Human	H1N1	pdm	2009	USA	A/New_York/6943/2009	ggtgatgccccattccttgatcggctccgccgagatcaaaagtccttaaaaggaagaggcaacaccc	-	*
CY065788	Human H1N1pdm2009 IAVs	Human	H1N1	pdm	2009	Netherlands	A/Netherlands/2629/2009	ggtgatgccccattccttgatcggctccgccgagatcaaaagtccttaaaaggaagaggcaacaccc	-	*
CY075640	Human H1N1pdm2009 IAVs	Human	H1N1	pdm	2009	USA	A/Boston/703/2009	ggtgatgccccattccttgatcggctccgccgagatcaaaagtccttaaaaggaagaggcaacaccc	-	*
CY123316	Human H1N1pdm2009 IAVs	Human	H1N1	pdm	2009	Singapore	A/Singapore/GP4895/2009	ggtgatgccccattccttgatcggctccgccgagatcaaaagtccttaaaaggaagaggcaacaccc	-	*
CY123845	Human H1N1pdm2009 IAVs	Human	H1N1	pdm	2009	Singapore	A/Singapore/ON2291/2009	ggtgatgccccattccttgatcggctccgccgagatcaaaagtccttaaaaggaagaggcaacaccc	-	*
HQ541700	Human H1N1pdm2009 IAVs	Swine	H1N1		2009	China	A/swine/Heilongjiang/105/2009	ggtgatgccccattccttgatcggctccgccgagatcaaaagtccttaaaaggaagaggcaacaccc	-	*
CY056112	Human H1N1pdm2009 IAVs	Human	H1N1		2009	USA	A/District_of_Columbia/INS29/2009	ggtgatgccccattccttgatcggctccgccgagatcaaaagtccttaaaaggaagaggcaacaccc	-	*
CY065594	Human H1N1pdm2009 IAVs	Human	H1N1	pdm	2009	United_Kingdom	A/England/388/2009	ggtgatgccccattccttgatcggctccgccgagatcaaaagtccttaaaaggaagaggcaacaccc	-	*
GQ251039	Human H1N1pdm2009 IAVs	Human	H1N1	pdm	2009	Italy	A/Italy/05/2009	ggtgatgccccattccttgatcggctccgccgagatcaaaagtccttaaaaggaagaggcaacaccc	-	*
CY117606	Human H1N1pdm2009 IAVs	Human	H1N1	pdm	2009	Mexico	A/Mexico/UASLP_016/2009	ggtgatgccccattccttgatcggctccgccgagatcaaaagtccttaaaaggaagaggcaacaccc	-	*
GU367321	Human H1N1pdm2009 IAVs	Human	H1N1	pdm	2009	Russia	A/Tomsk/07/2009	ggtgatgccccattccttgatcggctccgccgagatcaaaagtccttaaaaggaagaggcaacaccc	-	*
CY073084	Human H1N1pdm2009 IAVs	Human	H1N1	pdm	2009	Turkey	A/Ankara/WRAIR1428T/2009	ggtgatgccccattccttgatcggctccgccgagatcaaaagtccttaaaaggaagaggcaacaccc	-	*
CY070995	Human H1N1pdm2009 IAVs	Human	H1N1	pdm	2009	Guam	A/Guam/NHRC0029/2009	ggtgatgccccattccttgatcggctccgccgagatcaaaagtccttaaaaggaagaggcaacaccc	-	*
CY084450	Human H1N1pdm2009 IAVs	Human	H1N1	pdm	2009	USA	A/New_York/6902/2009	ggtgatgccccattccttgatcggctccgccgagatcaaaagtccttaaaaggaagaggcaacaccc	-	*
CY106572	Human H1N1pdm2009 IAVs	Human	H1N1		2009	Mexico	A/Merida/2189_CIR/2009	ggtgatgccccattccttgatcggctccgccgagatcaaaagtccttaaaaggaagaggcaacaccc	-	*
GQ225361	Human H1N1pdm2009 IAVs	Human	H1N1	pdm	2009	China	A/Shanghai/1/2009	ggtgatgccccattccttgatcggctccgccgagatcaaaagtccttaaaaggaagaggcaacaccc	-	*
CY062863	Human H1N1pdm2009 IAVs	Human	H1N1	pdm	2009	Greece	A/Athens/INS159/2009	ggtgatgccccattccttgatcggctccgccgagatcaaaagtccttaaaaggaagaggcaacaccc	-	*
CY067115	Human H1N1pdm2009 IAVs	Human	H1N1	pdm	2009	Greece	A/Athens/INS264/2009	ggtgatgccccattccttgatcggctccgccgagatcaaaagtccttaaaaggaagaggcaacaccc	-	*

CY100468	Human H1N1pdm2009 IAVs	Human	H1N1	pdm	2009	Mexico	A/Mexico_City/INER2/2009	ggtgatgccccattccttgatcggctccgccgagatca aaagtccttaaaaggaagaggcaacaccc	-	*
KC261334	Human H1N1pdm2009 IAVs	Human	H1N1	pdm	2009	Mexico	A/Mexico_City/INERPHAC1/2009	ggtgatgccccattccttgatcggctccgccgagatca aaagtccttaaaaggaagaggcaacaccc	-	*
CY092442	Human H1N1pdm2009 IAVs	Human	H1N1		2009	Australia	A/Sydney/DD3_02/2009	ggtgatgccccattccttgatcggctccgccgagatca aaagtccttaaaaggaagaggcaacaccc	-	*
CY058034	Human H1N1pdm2009 IAVs	Human	H1N1	pdm	2009	USA	A/Wisconsin/629_D00636/2009	ggtgatgccccattccttgatcggctccgccgagatca aaagtccttaaaaggaagaggcaacaccc	-	*
CY055816	Human H1N1pdm2009 IAVs	Human	H1N1	pdm	2009	Australia	A/Australia/57/2009	ggtgatgccccattccttgatcggctccgccgagatca aaagtccttaaaaggaagaggcaacaccc	-	*
HM855242	Human H1N1pdm2009 IAVs	Human	H1N1		2009	Kenya	A/Garissa/78/2009	ggtgatgccccattccttgatcggctccgccgagatca aaagtccttaaaaggaagaggcaacaccc	-	*
CY061007	Human H1N1pdm2009 IAVs	Human	H1N1	pdm	2009	USA	A/Texas/JMS386/2009	ggtgatgccccattccttgatcggctccgccgagatca aaagtccttaaaaggaagaggcaacaccc	-	*
HM855253	Human H1N1pdm2009 IAVs	Human	H1N1	pdm	2009	Kenya	A/Karatina/471/2009	ggtgatgccccattccttgatcggctccgccgagatca aaagtccttaaaaggaagaggcaacaccc	-	*
HM855256	Human H1N1pdm2009 IAVs	Human	H1N1	pdm	2009	Kenya	A/Kitale/532/2009	ggtgatgccccattccttgatcggctccgccgagatca aaagtccttaaaaggaagaggcaacaccc	-	*
HM855252	Human H1N1pdm2009 IAVs	Human	H1N1		2009	Kenya	A/Meru/467/2009	ggtgatgccccattccttgatcggctccgccgagatca aaagtccttaaaaggaagaggcaacaccc	-	*
HM855254	Human H1N1pdm2009 IAVs	Human	H1N1	pdm	2009	Kenya	A/Nyeri/478/2009	ggtgatgccccattccttgatcggctccgccgagatca aaagtccttaaaaggaagaggcaacaccc	-	*
CY062711	Human H1N1pdm2009 IAVs	Human	H1N1	pdm	2009	Greece	A/Athens/INS123/2009	ggtgatgccccattccttgatcggctccgccgagatca aaagtccttaaaaggaagaggcaacaccc	-	*
CY062735	Human H1N1pdm2009 IAVs	Human	H1N1	pdm	2009	Greece	A/Athens/INS126/2009	ggtgatgccccattccttgatcggctccgccgagatca aaagtccttaaaaggaagaggcaacaccc	-	*
CY067131	Human H1N1pdm2009 IAVs	Human	H1N1	pdm	2009	Greece	A/Athens/INS267/2009	ggtgatgccccattccttgatcggctccgccgagatca aaagtccttaaaaggaagaggcaacaccc	-	*
CY067163	Human H1N1pdm2009 IAVs	Human	H1N1	pdm	2009	Greece	A/Athens/INS276/2009	ggtgatgccccattccttgatcggctccgccgagatca aaagtccttaaaaggaagaggcaacaccc	-	*
CY073881	Human H1N1pdm2009 IAVs	Human	H1N1	pdm	2009	Greece	A/Athens/INS323/2009	ggtgatgccccattccttgatcggctccgccgagatca aaagtccttaaaaggaagaggcaacaccc	-	*
CY083906	Human H1N1pdm2009 IAVs	Human	H1N1	pdm	2009	Greece	A/Athens/INS86/2009	ggtgatgccccattccttgatcggctccgccgagatca aaagtccttaaaaggaagaggcaacaccc	-	*
CY063650	Human H1N1pdm2009 IAVs	Human	H1N1	pdm	2009	Greece	A/Athens/INS154/2009	ggtgatgccccattccttgatcggctccgccgagatca aaagtccttaaaaggaagaggcaacaccc	-	*
CY123252	Human H1N1pdm2009 IAVs	Human	H1N1		2009	Singapore	A/Singapore/GP4309/2009	ggtgatgccccattccttgatcggctccgccgagatca aaagtccttaaaaggaagaggcaacaccc	-	*
GQ150332	Human H1N1pdm2009 IAVs	Swine	H1N1		2009	Canada	A/swine/Alberta/OTH_33_8/2009	ggtgatgccccattccttgatcggctccgccgagatca aaagtccttaaaaggaagaggcaacaccc	-	*
CY052859	Human H1N1pdm2009 IAVs	Human	H1N1	pdm	2009	USA	A/Texas/45023717/2009	ggtgatgccccattccttgatcggctccgccgagatca aaagtccttaaaaggaagaggcaacaccc	-	*
CY051315	Human H1N1pdm2009 IAVs	Human	H1N1	pdm	2009	USA	A/Wisconsin/629_S0201/2009	ggtgatgccccattccttgatcggctccgccgagatca aaagtccttaaaaggaagaggcaacaccc	-	*
HM567996	Human H1N1pdm2009 IAVs	Human	H1N1	pdm	2009	United_Kingdom	A/England/859/2009	ggtgatgccccattccttgatcggctccgccgagatca aaagtccttaaaaggaagaggcaacaccc	-	*
CY058480	Human H1N1pdm2009 IAVs	Human	H1N1	pdm	2009	USA	A/Wisconsin/629_D02424/2009	ggtgatgccccattccttgatcggctccgccgagatca aaagtccttaaaaggaagaggcaacaccc	-	*
JX625587	Human H1N1pdm2009 IAVs	Human	H1N1	pdm	2009	United_Kingdom	A/England/94620043/2009	ggtgatgccccattccttgatcggctccgccgagatca aaagtccttaaaaggaagaggcaacaccc	-	*

CY057354	Human H1N1pdm2009 IAVs	Human	H1N1	pdm	2009	Germany	A/Hamburg/INS92/2009	ggtgatgccccattccttgatcggctccgccgagatca<br>aaagtccttaaaaggaagaggcaacaccc	-	*
CY073162	Human H1N1pdm2009 IAVs	Human	H1N1		2009	Nigeria	A/Lagos/WRAIR1982N/2009	ggtgatgccccattccttgatcggctccgccgagatca<br>aaagtccttaaaaggaagaggcaacaccc	-	*
CY073178	Human H1N1pdm2009 IAVs	Human	H1N1	pdm	2009	Nigeria	A/Lagos/WRAIR1984T/2009	ggtgatgccccattccttgatcggctccgccgagatca<br>aaagtccttaaaaggaagaggcaacaccc	-	*
KJ023105	Human H1N1pdm2009 IAVs	Human	H1N1	pdm	2009	India	A/Haryana/015/2009	ggtgatgccccattccttgatcggctccgccgagatca<br>aaagtccttaaaaggaagaggcaacaccc	-	*
CY047763	Human H1N1pdm2009 IAVs	Human	H1N1	pdm	2009	Argentina	A/Argentina/13777/2009	ggtgatgccccattccttgatcggctccgccgagatca<br>aaagtccttaaaaggaagaggcaacaccc	-	*
CY047783	Human H1N1pdm2009 IAVs	Human	H1N1	pdm	2009	Argentina	A/Argentina/7649/2009	ggtgatgccccattccttgatcggctccgccgagatca<br>aaagtccttaaaaggaagaggcaacaccc	-	*
CY047791	Human H1N1pdm2009 IAVs	Human	H1N1	pdm	2009	Argentina	A/Argentina/7785/2009	ggtgatgccccattccttgatcggctccgccgagatca<br>aaagtccttaaaaggaagaggcaacaccc	-	*
CY047799	Human H1N1pdm2009 IAVs	Human	H1N1	pdm	2009	Argentina	A/Argentina/7937/2009	ggtgatgccccattccttgatcggctccgccgagatca<br>aaagtccttaaaaggaagaggcaacaccc	-	*
CY047887	Human H1N1pdm2009 IAVs	Human	H1N1	pdm	2009	Argentina	A/Argentina/9180/2009	ggtgatgccccattccttgatcggctccgccgagatca<br>aaagtccttaaaaggaagaggcaacaccc	-	*
CY047895	Human H1N1pdm2009 IAVs	Human	H1N1	pdm	2009	Argentina	A/Argentina/9333/2009	ggtgatgccccattccttgatcggctccgccgagatca<br>aaagtccttaaaaggaagaggcaacaccc	-	*
CY047903	Human H1N1pdm2009 IAVs	Human	H1N1	pdm	2009	Argentina	A/Argentina/9384/2009	ggtgatgccccattccttgatcggctccgccgagatca<br>aaagtccttaaaaggaagaggcaacaccc	-	*
CY047951	Human H1N1pdm2009 IAVs	Human	H1N1	pdm	2009	Argentina	A/Argentina/9597/2009	ggtgatgccccattccttgatcggctccgccgagatca<br>aaagtccttaaaaggaagaggcaacaccc	-	*
CY047967	Human H1N1pdm2009 IAVs	Human	H1N1	pdm	2009	Argentina	A/Argentina/9711/2009	ggtgatgccccattccttgatcggctccgccgagatca<br>aaagtccttaaaaggaagaggcaacaccc	-	*
CY073992	Human H1N1pdm2009 IAVs	Human	H1N1		2009	Argentina	A/Argentina/HNRG106/2009	ggtgatgccccattccttgatcggctccgccgagatca<br>aaagtccttaaaaggaagaggcaacaccc	-	*
CY053900	Human H1N1pdm2009 IAVs	Human	H1N1	pdm	2009	Argentina	A/Argentina/HNRG13/2009	ggtgatgccccattccttgatcggctccgccgagatca<br>aaagtccttaaaaggaagaggcaacaccc	-	*
CY074009	Human H1N1pdm2009 IAVs	Human	H1N1	pdm	2009	Argentina	A/Argentina/HNRG15/2009	ggtgatgccccattccttgatcggctccgccgagatca<br>aaagtccttaaaaggaagaggcaacaccc	-	*
CY074019	Human H1N1pdm2009 IAVs	Human	H1N1	pdm	2009	Argentina	A/Argentina/HNRG23/2009	ggtgatgccccattccttgatcggctccgccgagatca<br>aaagtccttaaaaggaagaggcaacaccc	-	*
CY053932	Human H1N1pdm2009 IAVs	Human	H1N1		2009	Argentina	A/Argentina/HNRG3/2009	ggtgatgccccattccttgatcggctccgccgagatca<br>aaagtccttaaaaggaagaggcaacaccc	-	*
CY074062	Human H1N1pdm2009 IAVs	Human	H1N1		2009	Argentina	A/Argentina/HNRG8/2009	ggtgatgccccattccttgatcggctccgccgagatca<br>aaagtccttaaaaggaagaggcaacaccc	-	*
CY074068	Human H1N1pdm2009 IAVs	Human	H1N1	pdm	2009	Argentina	A/Argentina/HNRG82/2009	ggtgatgccccattccttgatcggctccgccgagatca<br>aaagtccttaaaaggaagaggcaacaccc	-	*
CY075351	Human H1N1pdm2009 IAVs	Human	H1N1	pdm	2009	Chile	A/Chile/3369/2009	ggtgatgccccattccttgatcggctccgccgagatca<br>aaagtccttaaaaggaagaggcaacaccc	-	*
CY075359	Human H1N1pdm2009 IAVs	Human	H1N1	pdm	2009	Chile	A/Chile/3375/2009	ggtgatgccccattccttgatcggctccgccgagatca<br>aaagtccttaaaaggaagaggcaacaccc	-	*
CY075399	Human H1N1pdm2009 IAVs	Human	H1N1	pdm	2009	Chile	A/Chile/3766/2009	ggtgatgccccattccttgatcggctccgccgagatca<br>aaagtccttaaaaggaagaggcaacaccc	-	*
CY047927	Human H1N1pdm2009 IAVs	Human	H1N1	pdm	2009	Argentina	A/Argentina/9579/2009	ggtgatgccccattccttgatcggctccgccgagatca<br>aaagtccttaaaaggaagaggcaacaccc	-	*
CY074052	Human H1N1pdm2009 IAVs	Human	H1N1		2009	Argentina	A/Argentina/HNRG5/2009	ggtgatgccccattccttgatcggctccgccgagatca<br>aaagtccttaaaaggaagaggcaacaccc	-	*

KC780826	Human H1N1pdm2009 IAVs	Human	H1N1	pdm	2009	USA	A/Kansas/20/2009	ggtgatgccccattccttgatcggctccgccgagatca<br>aaagtccttaaaaggaagaggcaacaccc	-	*
KF411264	Human H1N1pdm2009 IAVs	Human	H1N1	pdm	2009	China	A/Qingdao/FF86/2009	ggtgatgccccattccttgatcggctccgccgagatca<br>aaagtccttaaaaggaagaggcaacaccc	-	*
KF411263	Human H1N1pdm2009 IAVs	Human	H1N1	pdm	2009	China	A/Qingdao/FF85/2009	ggtgatgccccattccttgatcggctccgccgagatca<br>aaagtccttaaaaggaagaggcaacaccc	-	*
CY052222	Human H1N1pdm2009 IAVs	Human	H1N1		2009	USA	A/Texas/44301765/2009	ggtgatgccccattccttgatcggctccgccgagatca<br>aaagtccttaaaaggaagaggcaacaccc	-	*
KJ023091	Human H1N1pdm2009 IAVs	Human	H1N1	pdm	2009	India	A/Karnataka/001/2009	ggtgatgccccattccttgatcggctccgccgagatca<br>aaagtccttaaaaggaagaggcaacaccc	-	*
KJ023099	Human H1N1pdm2009 IAVs	Human	H1N1	pdm	2009	India	A/Rajasthan/009/2009	ggtgatgccccattccttgatcggctccgccgagatca<br>aaagtccttaaaaggaagaggcaacaccc	-	*
CY071123	Human H1N1pdm2009 IAVs	Human	H1N1	pdm	2009	Germany	A/Wurzburg/INS382/2009	ggtgatgccccattccttgatcggctccgccgagatca<br>aaagtccttaaaaggaagaggcaacaccc	-	*
CY066939	Human H1N1pdm2009 IAVs	Human	H1N1	pdm	2009	Denmark	A/Aarhus/INS236/2009	ggtgatgccccattccttgatcggctccgccgagatca<br>aaagtccttaaaaggaagaggcaacaccc	-	*
CY060586	Human H1N1pdm2009 IAVs	Human	H1N1	pdm	2009	Canada	A/Ontario/304434/2009	ggtgatgccccattccttgatcggctccgccgagatca<br>aaagtccttaaaaggaagaggcaacaccc	-	*
CY058248	Human H1N1pdm2009 IAVs	Human	H1N1	pdm	2009	Nicaragua	A/Managua/3275.01/2009	ggtgatgccccattccttgatcggctccgccgagatca<br>aaagtccttaaaaggaagaggcaacaccc	-	*
CY083419	Human H1N1pdm2009 IAVs	Human	H1N1	pdm	2009	USA	A/San_Diego/WRAIR1666P/2009	ggtgatgccccattccttgatcggctccgccgagatca<br>aaagtccttaaaaggaagaggcaacaccc	-	*
CY057962	Human H1N1pdm2009 IAVs	Human	H1N1		2009	USA	A/Wisconsin/629_D00970/2009	ggtgatgccccattccttgatcggctccgccgagatca<br>aaagtccttaaaaggaagaggcaacaccc	-	*
CY088597	Human H1N1pdm2009 IAVs	Human	H1N1	pdm	2009	USA	A/Boston/702/2009	ggtgatgccccattccttgatcggctccgccgagatca<br>aaagtccttaaaaggaagaggcaacaccc	-	*
CY071614	Human H1N1pdm2009 IAVs	Human	H1N1	pdm	2009	USA	A/Ft_Carson/WR1448P/2009	ggtgatgccccattccttgatcggctccgccgagatca<br>aaagtccttaaaaggaagaggcaacaccc	-	*
CY057082	Human H1N1pdm2009 IAVs	Swine	H1N1		2009	Italy	A/swine/Italy/85429/2009	ggtgatgccccattccttgatcggctccgccgagatca<br>aaagtccttaaaaggaagaggcaacaccc	-	*
CY061551	Human H1N1pdm2009 IAVs	Swine	H1N1		2009	Italy	A/swine/Italy/85437/2009	ggtgatgccccattccttgatcggctccgccgagatca<br>aaagtccttaaaaggaagaggcaacaccc	-	*
CY071606	Human H1N1pdm2009 IAVs	Human	H1N1	pdm	2009	USA	A/Ft_Carson/WR1446P/2009	ggtgatgccccattccttgatcggctccgccgagatca<br>aaagtccttaaaaggaagaggcaacaccc	-	*
CY100484	Human H1N1pdm2009 IAVs	Human	H1N1	pdm	2009	Mexico	A/Mexico_City/INER13/2009	ggtgatgccccattccttgatcggctccgccgagatca<br>aaagtccttaaaaggaagaggcaacaccc	-	*
CY054682	Human H1N1pdm2009 IAVs	Human	H1N1		2009	Russia	A/Russia/180/2009	ggtgatgccccattccttgatcggctccgccgagatca<br>aaagtccttaaaaggaagaggcaacaccc	-	*
CY095874	Human H1N1pdm2009 IAVs	Human	H1N1	pdm	2009	China	A/Hubei/75/2009	ggtgatgccccattccttgatcggctccgccgagatca<br>aaagtccttaaaaggaagaggcaacaccc	-	*
CY063311	Human H1N1pdm2009 IAVs	Human	H1N1	pdm	2009	USA	A/Wisconsin/629_D01572/2009	ggtgatgccccattccttgatcggctccgccgagatca<br>aaagtccttaaaaggaagaggcaacaccc	-	*
CY062871	Human H1N1pdm2009 IAVs	Human	H1N1	pdm	2009	Greece	A/Athens/INS160/2009	ggtgatgccccattccttgatcggctccgccgagatca<br>aaagtccttaaaaggaagaggcaacaccc	-	*
CY056671	Human H1N1pdm2009 IAVs	Human	H1N1	pdm	2009	USA	A/New_York/6213/2009	ggtgatgccccattccttgatcggctccgccgagatca<br>aaagtccttaaaaggaagaggcaacaccc	-	*
CY083858	Human H1N1pdm2009 IAVs	Human	H1N1	pdm	2009	USA	A/Pensacola/INS39/2009	ggtgatgccccattccttgatcggctccgccgagatca<br>aaagtccttaaaaggaagaggcaacaccc	-	*
HM855259	Human H1N1pdm2009 IAVs	Human	H1N1	pdm	2009	Kenya	A/Nairobi/37/2009	ggtgatgccccattccttgatcggctccgccgagatca<br>aaagtccttaaaaggaagaggcaacaccc	-	*

CY087028	Human H1N1pdm2009 IAVs	Human	H1N1	pdm	2009	USA	A/New_York/4557/2009	ggtgatgccccattccttgatcggctccgccgagatca aaagtccttaaaaggaagaggcaacaccc	-	*
KJ690522	Human H1N1pdm2009 IAVs	Human	H1N1		2009	Uganda	A/Uganda/MUWRP_092/2009	ggtgatgccccattccttgatcggctccgccgagatca aaagtccttaaaaggaagaggcaacaccc	-	*
CY055840	Human H1N1pdm2009 IAVs	Human	H1N1	pdm	2009	Australia	A/Australia/60/2009	ggtgatgccccattccttgatcggctccgccgagatca aaagtccttaaaaggaagaggcaacactc	-	*
CY043151	Human H1N1pdm2009 IAVs	Human	H1N1	pdm	2009	USA	A/New_York/3551/2009	ggtgatgccccattccttgatcggctccgccgagatca aaagtccttaaaaggaagaggcaacactc	-	*
CY128255	Human H1N1pdm2009 IAVs	Human	H1N1	pdm	2009	Viet_Nam	A/Viet_Nam/13032051/2009	ggtgatgccccattccttgatcggctccgccgagatca aaagtccttaaaaggaagaggcaacactc	-	*
CY088656	Human H1N1pdm2009 IAVs	Human	H1N1	pdm	2009	India	A/Rtng/NIV11650/2009	ggtgatgccccattccttgatcggctccgccgagatca aaagtccttaaaaggaagaggcaacactc	-	*
CY064552	Human H1N1pdm2009 IAVs	Human	H1N1		2009	USA	A/Boston/122/2009	ggtgatgccccattccttgatcggctccgccgagatca aaagtccttaaaaggaagaggcaacactc	-	*
CY122999	Human H1N1pdm2009 IAVs	Human	H1N1	pdm	2009	Singapore	A/Singapore/GP3021/2009	ggtgatgccccattccttgatcggctccgccgagatca aaagtccttaaaaggaagaggcaacactc	-	*
GQ365432	Human H1N1pdm2009 IAVs	Human	H1N1		2009	Japan	A/Iwate/2/2009	ggtgatgccccattccttgatcggctccgccgagatca aaagtccttaaaaggaagaggcaacactc	-	*
GU136005	Human H1N1pdm2009 IAVs	Human	H1N1	pdm	2009	Japan	A/Iwate/3/2009	ggtgatgccccattccttgatcggctccgccgagatca aaagtccttaaaaggaagaggcaacactc	-	*
GU198205	Human H1N1pdm2009 IAVs	Human	H1N1	pdm	2009	China	A/Nanjing/3/2009	ggtgatgccccattccttgatcggctccgccgagatca aaagtccttaaaaggaagaggcaagaccc	-	*
CY057370	Human H1N1pdm2009 IAVs	Human	H1N1	pdm	2009	USA	A/Wisconsin/629_D00643/2009	ggtgatgccccattccttgatcggctccgccgagatca aaagtccttaaaaggaagaggcagcaccc	-	*
CY057530	Human H1N1pdm2009 IAVs	Human	H1N1	pdm	2009	USA	A/Wisconsin/629_D01391/2009	ggtgatgccccattccttgatcggctccgccgagatca aaagtccttaaaaggaagaggcagcaccc	-	*
CY057442	Human H1N1pdm2009 IAVs	Human	H1N1		2009	USA	A/Wisconsin/629_D01522/2009	ggtgatgccccattccttgatcggctccgccgagatca aaagtccttaaaaggaagaggcagcaccc	-	*
CY057306	Human H1N1pdm2009 IAVs	Human	H1N1	pdm	2009	USA	A/New_York/5297/2009	ggtgatgccccattccttgatcggctccgccgagatca aaagtccttaaaaggaagaggtaacaccc	-	*
KC781998	Human H1N1pdm2009 IAVs	Human	H1N1	pdm	2009	USA	A/North_Carolina/45/2009	ggtgatgccccattccttgatcggctccgccgagatca aaagtccttaaaaggaagaggtaacaccc	-	*
HM101146	Human H1N1pdm2009 IAVs	Human	H1N1	pdm	2009	Russia	A/Tver/IIV_183/2009	ggtgatgccccattccttgatcggctccgccgagatca aaagtccttaaaaggaagaggtaacaccc	-	*
HM855246	Human H1N1pdm2009 IAVs	Human	H1N1	pdm	2009	Kenya	A/Embu/169/2009	ggtgatgccccattccttgatcggctccgccgagatca aaagtccttaaaaggaagaggtaacaccc	-	*
CY066395	Human H1N1pdm2009 IAVs	Human	H1N1	pdm	2009	USA	A/California/VRDL127/2009	ggtgatgccccattccttgatcggctccgccgagatca aaagtccttaaaaggaaggggcaacaccc	-	*
CY065554	Human H1N1pdm2009 IAVs	Human	H1N1	pdm	2009	United_Kingdom	A/England/381/2009	ggtgatgccccattccttgatcggctccgccgagatca aaagtccttaaaaggaaggggcaacaccc	-	*
CY069889	Human H1N1pdm2009 IAVs	Human	H1N1	pdm	2009	United_Kingdom	A/England/383/2009	ggtgatgccccattccttgatcggctccgccgagatca aaagtccttaaaaggaaggggcaacaccc	-	*
CY065618	Human H1N1pdm2009 IAVs	Human	H1N1	pdm	2009	United_Kingdom	A/England/394/2009	ggtgatgccccattccttgatcggctccgccgagatca aaagtccttaaaaggaaggggcaacaccc	-	*
CY065626	Human H1N1pdm2009 IAVs	Human	H1N1	pdm	2009	United_Kingdom	A/England/395/2009	ggtgatgccccattccttgatcggctccgccgagatca aaagtccttaaaaggaaggggcaacaccc	-	*
CY065690	Human H1N1pdm2009 IAVs	Human	H1N1	pdm	2009	United_Kingdom	A/England/423/2009	ggtgatgccccattccttgatcggctccgccgagatca aaagtccttaaaaggaaggggcaacaccc	-	*
CY065698	Human H1N1pdm2009 IAVs	Human	H1N1	pdm	2009	United_Kingdom	A/England/428/2009	ggtgatgccccattccttgatcggctccgccgagatca aaagtccttaaaaggaaggggcaacaccc	-	*

HM567620	Human H1N1pdm2009 IAVs	Human	H1N1	pdm	2009	United_Kingdom	A/England/337/2009	ggtgatgccccattccttgatcggctccgccgagatca aaagtccttaaaaggaaggggcaacaccc	-	*
HM567668	Human H1N1pdm2009 IAVs	Human	H1N1	pdm	2009	United_Kingdom	A/England/347/2009	ggtgatgccccattccttgatcggctccgccgagatca aaagtccttaaaaggaaggggcaacaccc	-	*
HM567772	Human H1N1pdm2009 IAVs	Human	H1N1		2009	United_Kingdom	A/England/383/2009	ggtgatgccccattccttgatcggctccgccgagatca aaagtccttaaaaggaaggggcaacaccc	-	*
CY066835	Human H1N1pdm2009 IAVs	Human	H1N1	pdm	2009	Spain	A/Madrid/INS222/2009	ggtgatgccccattccttgatcggctccgccgagatca aaagtccttaaaagggagaggcaacaccc	-	*
CY056551	Human H1N1pdm2009 IAVs	Human	H1N1	pdm	2009	USA	A/New_York/5173/2009	ggtgatgccccattccttgatcggctccgccgagatca aaagtccttaaaagggagaggcaacaccc	-	*
CY058272	Human H1N1pdm2009 IAVs	Human	H1N1		2009	USA	A/New_York/5620/2009	ggtgatgccccattccttgatcggctccgccgagatca aaagtccttaaaagggagaggcaacaccc	-	*
CY071766	Human H1N1pdm2009 IAVs	Human	H1N1	pdm	2009	Mexico	A/Mexico_City/WR1708T/2009	ggtgatgccccattccttgatcggctccgccgagatca aaagtccttaaaagggagaggcaacaccc	-	*
CY062911	Human H1N1pdm2009 IAVs	Human	H1N1	pdm	2009	Greece	A/Athens/INS165/2009	ggtgatgccccattccttgatcggctccgccgagatca aaagtccttaaaagggagaggcaacaccc	-	*
CY071107	Human H1N1pdm2009 IAVs	Human	H1N1	pdm	2009	Belgium	A/Tessenderlo/INS380/2009	ggtgatgccccattccttgatcggctccgccgagatca aaagtccttaaagggaagaggcaacaccc	-	*
CY052579	Human H1N1pdm2009 IAVs	Human	H1N1	pdm	2009	USA	A/Texas/45131774/2009	ggtgatgccccattccttgatcggctccgccgagatca aaagtccttaaagggaagaggcaacaccc	-	*
CY052931	Human H1N1pdm2009 IAVs	Human	H1N1	pdm	2009	USA	A/Texas/45121004/2009	ggtgatgccccattccttgatcggctccgccgagatca aaagtccttaaagggaagaggcaacaccc	-	*
CY052587	Human H1N1pdm2009 IAVs	Human	H1N1	pdm	2009	USA	A/Texas/45132647/2009	ggtgatgccccattccttgatcggctccgccgagatca aaagtccttaaagggaagaggcaacaccc	-	*
CY083749	Human H1N1pdm2009 IAVs	Human	H1N1	pdm	2009	Spain	A/Barcelona/INS189/2009	ggtgatgccccattccttgatcggctccgccgagatca aaagtccttgaaaggaagaggcaacaccc	-	*
CY071099	Human H1N1pdm2009 IAVs	Human	H1N1	pdm	2009	Spain	A/Barcelona/INS379/2009	ggtgatgccccattccttgatcggctccgccgagatca aaagtccttgaaaggaagaggcaacaccc	-	*
CY054743	Human H1N1pdm2009 IAVs	Human	H1N1	pdm	2009	USA	A/California/VRDL8/2009	ggtgatgccccattccttgatcggctccgccgagatca aaagtccttgaaaggaagaggcaacaccc	-	*
CY055920	Human H1N1pdm2009 IAVs	Human	H1N1	pdm	2009	Australia	A/Australia/79/2009	ggtgatgccccattccttgatcggctccgccgagatca aaagtccttgaaaggaagaggcaacaccc	-	*
HM157261	Human H1N1pdm2009 IAVs	Human	H1N1	pdm	2009	Russia	A/Yaroslavl/IIV_196/2009	ggtgatgccccattccttgatcggctccgccgagatca aaagtccttgaaaggaagaggcaacaccc	-	*
JN043446	Human H1N1pdm2009 IAVs	Swine	H1N1		2009	South_Korea	A/swine/Korea/VD01/2009	ggtgatgccccattccttgatcggctccgccgagatca aaagtccttgaaaggaagaggcaacaccc	-	*
CY075528	Human H1N1pdm2009 IAVs	Human	H1N1	pdm	2009	USA	A/Boston/606/2009	ggtgatgccccattccttgatcggctccgccgagatca aaagtctttaaaaggaagaggcaacaccc	-	*
CY058392	Human H1N1pdm2009 IAVs	Human	H1N1		2009	USA	A/Wisconsin/629_D00147/2009	ggtgatgccccattccttgatcggctccgccgagatca aaagtctttaaaaggaagaggcaacaccc	-	*
CY057994	Human H1N1pdm2009 IAVs	Human	H1N1	pdm	2009	USA	A/Wisconsin/629_D00935/2009	ggtgatgccccattccttgatcggctccgccgagatca aaagtctttaaaaggaagaggcaacaccc	-	*
CY058400	Human H1N1pdm2009 IAVs	Human	H1N1	pdm	2009	USA	A/Wisconsin/629_D01591/2009	ggtgatgccccattccttgatcggctccgccgagatca aaagtctttaaaaggaagaggcaacaccc	-	*
CY072394	Human H1N1pdm2009 IAVs	Human	H1N1	pdm	2009	Greece	A/Athens/INS332/2009	ggtgatgccccattccttgatcggctccgccgagatca aaagtctttaaaaggaagaggcaacaccc	-	*
CY072458	Human H1N1pdm2009 IAVs	Human	H1N1	pdm	2009	Greece	A/Athens/INS340/2009	ggtgatgccccattccttgatcggctccgccgagatca aaagtctttaaaaggaagaggcaacaccc	-	*
CY072466	Human H1N1pdm2009 IAVs	Human	H1N1	pdm	2009	Greece	A/Athens/INS341/2009	ggtgatgccccattccttgatcggctccgccgagatca aaagtctttaaaaggaagaggcaacaccc	-	*

CY072522	Human H1N1pdm2009 IAVs	Human	H1N1		2009	Greece	A/Athens/INS349/2009	ggtgatgccccattccttgatcggctccgccgagatcaaaagtctttaaaaggaagaggcaacaccc	-	*
HM568052	Human H1N1pdm2009 IAVs	Human	H1N1		2009	United_Kingdom	A/England/950/2009	ggtgatgccccattccttgatcggctccgccgagatcaaaagtctttaaaaggaagaggcaacaccc	-	*
CY063079	Human H1N1pdm2009 IAVs	Human	H1N1	pdm	2009	USA	A/California/VRDL88/2009	ggtgatgccccattccttgatcggctccgccgagatcaaaagtctttaaaaggaagaggcaacaccc	-	*
CY083765	Human H1N1pdm2009 IAVs	Human	H1N1	pdm	2009	Denmark	A/Aarhus/INS239/2009	ggtgatgccccattccttgatcggctccgccgagatcagaagtccttaaaaggaagaggcaacaccc	-	*
CY067107	Human H1N1pdm2009 IAVs	Human	H1N1	pdm	2009	Greece	A/Athens/INS263/2009	ggtgatgccccattccttgatcggctccgccgagatcagaagtccttaaaaggaagaggcaacaccc	-	*
CY067139	Human H1N1pdm2009 IAVs	Human	H1N1	pdm	2009	Greece	A/Athens/INS268/2009	ggtgatgccccattccttgatcggctccgccgagatcagaagtccttaaaaggaagaggcaacaccc	-	*
CY072434	Human H1N1pdm2009 IAVs	Human	H1N1	pdm	2009	Greece	A/Athens/INS337/2009	ggtgatgccccattccttgatcggctccgccgagatcagaagtccttaaaaggaagaggcaacaccc	-	*
CY073769	Human H1N1pdm2009 IAVs	Human	H1N1	pdm	2009	Spain	A/Barcelona/INS378/2009	ggtgatgccccattccttgatcggctccgccgagatcagaagtccttaaaaggaagaggcaacaccc	-	*
CY063047	Human H1N1pdm2009 IAVs	Human	H1N1	pdm	2009	Germany	A/Bochum/INS187/2009	ggtgatgccccattccttgatcggctccgccgagatcagaagtccttaaaaggaagaggcaacaccc	-	*
CY067179	Human H1N1pdm2009 IAVs	Human	H1N1	pdm	2009	Germany	A/Bonn/INS284/2009	ggtgatgccccattccttgatcggctccgccgagatcagaagtccttaaaaggaagaggcaacaccc	-	*
CY061271	Human H1N1pdm2009 IAVs	Human	H1N1	pdm	2009	Belgium	A/Brussels/INS106/2009	ggtgatgccccattccttgatcggctccgccgagatcagaagtccttaaaaggaagaggcaacaccc	-	*
CY066747	Human H1N1pdm2009 IAVs	Human	H1N1	pdm	2009	Belgium	A/Brussels/INS209/2009	ggtgatgccccattccttgatcggctccgccgagatcagaagtccttaaaaggaagaggcaacaccc	-	*
CY056943	Human H1N1pdm2009 IAVs	Human	H1N1	pdm	2009	Belgium	A/Brussels/INS71/2009	ggtgatgccccattccttgatcggctccgccgagatcagaagtccttaaaaggaagaggcaacaccc	-	*
CY056040	Human H1N1pdm2009 IAVs	Human	H1N1	pdm	2009	USA	A/District_of_Columbia/INS17/2009	ggtgatgccccattccttgatcggctccgccgagatcagaagtccttaaaaggaagaggcaacaccc	-	*
CY066699	Human H1N1pdm2009 IAVs	Human	H1N1	pdm	2009	Denmark	A/Hvidovre/INS290/2009	ggtgatgccccattccttgatcggctccgccgagatcagaagtccttaaaaggaagaggcaacaccc	-	*
CY088733	Human H1N1pdm2009 IAVs	Human	H1N1	pdm	2009	Netherlands	A/Netherlands/2223b/2009	ggtgatgccccattccttgatcggctccgccgagatcagaagtccttaaaaggaagaggcaacaccc	-	*
CY107264	Human H1N1pdm2009 IAVs	Human	H1N1	pdm	2009	United_Kingdom	A/Scotland/Aberdeen_16/2009	ggtgatgccccattccttgatcggctccgccgagatcagaagtccttaaaaggaagaggcaacaccc	-	*
CY107531	Human H1N1pdm2009 IAVs	Human	H1N1	pdm	2009	United_Kingdom	A/Scotland/Edinburgh_21027/2009	ggtgatgccccattccttgatcggctccgccgagatcagaagtccttaaaaggaagaggcaacaccc	-	*
CY063063	Human H1N1pdm2009 IAVs	Human	H1N1	pdm	2009	Belgium	A/Tessenderlo/INS192/2009	ggtgatgccccattccttgatcggctccgccgagatcagaagtccttaaaaggaagaggcaacaccc	-	*
CY072546	Human H1N1pdm2009 IAVs	Human	H1N1	pdm	2009	Poland	A/Wroclaw/INS436/2009	ggtgatgccccattccttgatcggctccgccgagatcagaagtccttaaaaggaagaggcaacaccc	-	*
CY083686	Human H1N1pdm2009 IAVs	Human	N1	pdm	2009	Belgium	A/Brussels/INS79/2009	ggtgatgccccattccttgatcggctccgccgagatcagaagtccttaaaaggaagaggcaacaccc	-	*
CY083733	Human H1N1pdm2009 IAVs	Human	H1N1	pdm	2009	Germany	A/Bochum/INS120/2009	ggtgatgccccattccttgatcggctccgccgagatcagaagtccttaaaaggaagaggcaacaccc	-	*
CY066731	Human H1N1pdm2009 IAVs	Human	H1N1	pdm	2009	Belgium	A/Brussels/INS206/2009	ggtgatgccccattccttgatcggctccgccgagatcagaagtccttaaaaggaagaggcaacaccc	-	*
CY066739	Human H1N1pdm2009 IAVs	Human	H1N1	pdm	2009	Belgium	A/Brussels/INS208/2009	ggtgatgccccattccttgatcggctccgccgagatcagaagtccttaaaaggaagaggcaacaccc	-	*
CY055039	Human H1N1pdm2009 IAVs	Human	H1N1	pdm	2009	USA	A/California/VRDL69/2009	ggtgatgccccattccttgatcggctccgccgagatcagaagtccttaaaaggaagaggcaacaccc	-	*

CY066683	Human H1N1pdm2009 IAVs	Human	H1N1	pdm	2009	Denmark	A/Hvidovre/INS288/2009	ggtgatgccccattccttgatcggctccgccgagatcagaagtccttaaaaggaagaggcaacaccc	-	*
CY072298	Human H1N1pdm2009 IAVs	Human	H1N1	pdm	2009	Poland	A/Warsaw/INS314/2009	ggtgatgccccattccttgatcggctccgccgagatcagaagtccttaaaaggaagaggcaacaccc	-	*
CY107498	Human H1N1pdm2009 IAVs	Human	H1N1	pdm	2009	United_Kingdom	A/Scotland/Edinburgh_20203/2009	ggtgatgccccattccttgatcggctccgccgagatcagaagtccttaaaaggaagaggcaacaccc	-	*
CY062935	Human H1N1pdm2009 IAVs	Human	H1N1	pdm	2009	Germany	A/Berlin/INS171/2009	ggtgatgccccattccttgatcggctccgccgagatcagaagtccttaaaaggaagaggcaacaccc	-	*
CY128279	Human H1N1pdm2009 IAVs	Human	H1N1	pdm	2009	Viet_Nam	A/Viet_Nam/13032061/2009	ggtgatgccccattccttgatcggctccgccgagatcagaagtccttaaaaggaagaggcaacaccc	-	*
CY083702	Human H1N1pdm2009 IAVs	Human	mixed		2009	Poland	A/Warsaw/INS99/2009	ggtgatgccccattccttgatcggctccgccgagatcagaagtccttaaaaggaagaggcaacaccc	-	*
CY083467	Human H1N1pdm2009 IAVs	Human	H1N1	pdm	2009	Peru	A/Lima/WRAIR1688P/2009	ggtgatgccccattccttgatcggctccgccgagatcagaagtccttaaaaggaagaggcaacaccc	-	*
CY083292	Human H1N1pdm2009 IAVs	Human	H1N1		2009	USA	A/San_Diego/WRAIR1646P/2009	ggtgatgccccattccttgatcggctccgccgagatcagaagtccttaaaaggaagaggcaacaccc	-	*
CY051635	Human H1N1pdm2009 IAVs	Human	H1N1	pdm	2009	USA	A/New_York/4620/2009	ggtgatgccccattccttgatcggctccgccgagatcagaagtccttaaaaggaagaggcaacaccc	-	*
CY058058	Human H1N1pdm2009 IAVs	Human	H1N1	pdm	2009	USA	A/Texas/46181235/2009	ggtgatgccccattccttgatcggctccgccgagatcagaagtccttaaaaggaagaggcaacaccc	-	*
CY107215	Human H1N1pdm2009 IAVs	Human	H1N1	pdm	2009	United_Kingdom	A/Scotland/Aberdeen_1/2009	ggtgatgccccattccttgatcggctccgccgagatcagaagtccttaaaaggaagaggcagcaccc	-	*
CY107299	Human H1N1pdm2009 IAVs	Human	H1N1	pdm	2009	United_Kingdom	A/Scotland/Aberdeen_5/2009	ggtgatgccccattccttgatcggctccgccgagatcagaagtccttaaaaggaagaggcagcaccc	-	*
CY065242	Human H1N1pdm2009 IAVs	Human	H1N1		2009	United_Kingdom	A/England/551/2009	ggtgatgccccattccttgatcggctccgccgaggtcaaaagtccttaaaaggaagaggcaacaccc	-	*
CY054887	Human H1N1pdm2009 IAVs	Human	H1N1	pdm	2009	USA	A/California/VRDL36/2009	ggtgatgccccattccttgatcggctccgccgggatcaaaagtccttaaaaggaagaggcaacaccc	-	*
CY122643	Human H1N1pdm2009 IAVs	Human	H1N1	pdm	2009	Singapore	A/Singapore/GP1120/2009	ggtgatgccccattccttgatcggctccgccgggatcaaaagtccttaaaaggaagaggcaacaccc	-	*
CY095882	Human H1N1pdm2009 IAVs	Human	H1N1		2009	China	A/Hubei/76/2009	ggtgatgccccattccttgatcggctccgccgggatcaaaagtccttaaaaggaagaggcaacaccc	-	*
CY090004	Human H1N1pdm2009 IAVs	Human	H1N1	pdm	2009	USA	A/Wisconsin/629_S0235/2009	ggtgatgccccattccttgatcggctccgcgcagatcaagcgtccttaaaaggaagaggcaacaccc	-	-
GQ303354	Human H1N1pdm2009 IAVs	Human	H1N1	pdm	2009	Mexico	A/Mexico/6882/2009	ggtgatgccccattccttgatcggcttcgccgagatcaaaagtccttaaaaggaagaggcaacaccc	-	*
CY123326	Human H1N1pdm2009 IAVs	Human	H1N1	pdm	2009	Singapore	A/Singapore/GP4915/2009	ggtgatgccccattccttgatcggcttcgccgagatcaaaagtccttaaaaggaagaggcaacaccc	-	*
CY051563	Human H1N1pdm2009 IAVs	Human	H1N1	pdm	2009	USA	A/New_York/4459/2009	ggtgatgccccattccttgatcggcttcgccgagatcaaaagtccttaaaaggaagaggcaacaccc	-	*
CY089628	Human H1N1pdm2009 IAVs	Human	H1N1	pdm	2009	China	A/Nanchang/8011/2009	ggtgatgccccattccttgatcggcttcgccgagatcaaaagtccttaaaaggaagaggcaacaccc	-	*
CY147711	Human H1N1pdm2009 IAVs	Human	H1N1	pdm	2009	Mexico	A/Mexico/24022/2009	ggtgatgccccattccttgatcggttccgccgagatcaaaagtccttaaaaggaagaggcaacaccc	-	*
GQ402284	Human H1N1pdm2009 IAVs	Human	H1N1	pdm	2009	Canada	A/Canada_MB/RV2013/2009	ggtgatgccccattctttgatcggctccgccgagatcaaaagtccttaaaaggaagaggcaacaccc	-	-
CY066195	Human H1N1pdm2009 IAVs	Human	H1N1	pdm	2009	USA	A/California/VRDL94/2009	ggtgatgccccatttcttgatcggctccgccgagatcaaaagtccttaaaaggaagaggcaacaccc	-	-
KF411268	Human H1N1pdm2009 IAVs	Human	H1N1	pdm	2009	China	A/Qingdao/1568/2009	ggtgatgccccatttcttgatcggctccgccgagatcaaaagtccttaaaaggaagaggcaacaccc	-	-

CY148223	Human H1N1pdm2009 IAVs	Human	H1N1	pdm	2009	Netherlands	A/Netherlands/602/2009	ggtgatgccccatttcttgatcggctccgccgagatcaa aagtccttaaaaggaagaggcaacaccc	-	-
CY148119	Human H1N1pdm2009 IAVs	Human	H1N1	pdm	2009	Netherlands	A/Netherlands/602/2009	ggtgatgccccatttcttgatcggctccgccgagatcaa aagtccttaaaaggaagaggcaacaccc	-	-
HM855249	Human H1N1pdm2009 IAVs	Human	H1N1	pdm	2009	Kenya	A/Kakamega/215/2009	ggtgatgccccgttccttgatcggctccgccgagatca aaagtccttaaaaggaagaggcaacaccc	-	*
CY100505	Human H1N1pdm2009 IAVs	Human	H1N1	pdm	2009	Mexico	A/Mexico_City/INER16/2009	ggtgatgcccnnnnnnnnnatcggctccgccgagat caaaagtccttaaaaggaagaggcaacacn	-	-
JQ173107	Human H1N1pdm2009 IAVs	Human	H1N1	pdm	2009	China	A/Nanjing/NJU_108/2009	ggtgatgtcccattccttgatcggctccgccgagatcaa aagtccttaaaaggaagaggcaacaccc	-	*
CY123095	Human H1N1pdm2009 IAVs	Human	H1N1	pdm	2009	Singapore	A/Singapore/GP3183/2009	ggtgatgtcccattccttgatcggctccgccgagatcaa aagtccttaaaaggaagaggcaacaccc	-	*
HM855264	Human H1N1pdm2009 IAVs	Human	H1N1	pdm	2009	Kenya	A/Nairobi/3/2009	ggtgttgccccattccttgatcgtctccgccgagatcaa aagtccttaaggggggggggcccccccc	-	-
CY046623	Human H1N1pdm2009 IAVs	Human	H1N1	pdm	2009	USA	A/Wisconsin/629_D00575/2009	ggygatgccccattccttgatcggctccgccgagatca aaagtccttaaaaggaagaggcaacaccc	-	*
GQ249335	Human H1N1pdm2009 IAVs	Human	H1N1	pdm	2009	France	A/Paris/2591/2009	gttgatgccccattccttgatcggctccgccgagatcaa aagtccttaaaaggaagaggcaacaccc	-	*
JX309450	Human H1N1pdm2009 IAVs	Human	H1N1	pdm	2010	Singapore	A/Singapore/GP2726/2010	agtgatgccccattccttgatcggctccgccgagatca aaagtccttaaaaggaagaggcaacaccc	-	*
JX309606	Human H1N1pdm2009 IAVs	Human	H1N1	pdm	2010	Singapore	A/Singapore/KK415/2010	agtgatgccccattccttgatcggctccgccgagatca aaagtccttaaaaggaagaggcaacaccc	-	*
CY093137	Human H1N1pdm2009 IAVs	Human	H1N1	pdm	2010	Australia	A/Sydney/DD3_11/2010	ggcgatgccccattccttgatcggctccgccgagatca aaagtccttaaaaggaagaggcaacaccc	-	*
CY062070	Human H1N1pdm2009 IAVs	Human	H1N1	pdm	2010	USA	A/New_York/1680/2010	ggtgatgccccattcattgatcggctccgccgagatca aaagtccttaaaaggaagaggcaacaccc	-	-
CY062102	Human H1N1pdm2009 IAVs	Human	H1N1		2010	USA	A/New_York/2175/2010	ggtgatgccccattcattgatcggctccgccgagatca aaagtccttaaaaggaagaggcaacaccc	-	-
CY062118	Human H1N1pdm2009 IAVs	Human	H1N1	pdm	2010	USA	A/New_York/2598/2010	ggtgatgccccattcattgatcggctccgccgagatca aaagtccttaaaaggaagaggcaacaccc	-	-
CY062158	Human H1N1pdm2009 IAVs	Human	H1N1	pdm	2010	USA	A/New_York/2971/2010	ggtgatgccccattcattgatcggctccgccgagatca aaagtccttaaaaggaagaggcaacaccc	-	-
CY062174	Human H1N1pdm2009 IAVs	Human	H1N1	pdm	2010	USA	A/New_York/3236/2010	ggtgatgccccattcattgatcggctccgccgagatca aaagtccttaaaaggaagaggcaacaccc	-	-
CY072538	Human H1N1pdm2009 IAVs	Human	H1N1	pdm	2010	USA	A/New_York/INS432/2010	ggtgatgccccattcattgatcggctccgccgagatca aaagtccttaaaaggaagaggcaacaccc	-	-
CY167344	Human H1N1pdm2009 IAVs	Human	H1N1	pdm	2010	USA	A/Tennessee/F1057c56/2010	ggtgatgccccattcattgatcggctccgccgagatca aaagtccttaaaaggaagaggcaacaccc	-	-
KC782019	Human H1N1pdm2009 IAVs	Human	H1N1	pdm	2010	USA	A/Vermont/01/2010	ggtgatgccccattcattgatcggctccgccgagatca aaagtccttaaaaggaagaggcaacaccc	-	-
KJ023126	Human H1N1pdm2009 IAVs	Human	H1N1	pdm	2010	India	A/Delhi/036/2010	ggtgatgccccattccttgatcggctacgccgagatca aaagtccttaaaaggaagaggcaacaccc	-	*
KJ023127	Human H1N1pdm2009 IAVs	Human	H1N1	pdm	2010	India	A/Delhi/037/2010	ggtgatgccccattccttgatcggctacgccgagatca aaagtccttaaaaggaagaggcaacaccc	-	*
CY096478	Human H1N1pdm2009 IAVs	Human	H1N1	pdm	2010	Thailand	A/Bangkok/INS507/2010	ggtgatgccccattccttgatcggctccgcagagatca aaagtccttaaaaggaagaggcaacaccc	+	+
CY096502	Human H1N1pdm2009 IAVs	Human	H1N1		2010	Thailand	A/Bangkok/INS512/2010	ggtgatgccccattccttgatcggctccgcagagatca aaagtccttaaaaggaagaggcaacaccc	+	+
JX309964	Human H1N1pdm2009 IAVs	Human	H1N1	pdm	2010	Singapore	A/Singapore/GP4565/2010	ggtgatgccccattccttgatcggctccgcagagatca aaagtccttaaaaggaagaggcaacaccc	+	+

JX309970	Human H1N1pdm2009 IAVs	Human	H1N1	pdm	2010	Singapore	A/Singapore/GP4588/2010	ggtgatgccccattccttgatcggctccgcagagatca aaagtccttaaaaggaagaggcaacaccc	+	+
JX309986	Human H1N1pdm2009 IAVs	Human	H1N1	pdm	2010	Singapore	A/Singapore/KK734/2010	ggtgatgccccattccttgatcggctccgcagagatca aaagtccttaaaaggaagaggcaacaccc	+	+
KC780355	Human H1N1pdm2009 IAVs	Human	H1N1	pdm	2010	USA	A/North_Carolina/05/2010	ggtgatgccccattccttgatcggctccgccgagacca aaagtccttaaaaggaagaggcaacaccc	-	-
JN375267	Human H1N1pdm2009 IAVs	Swine	H1N1		2010	China	A/swine/Guangdong/1437/2010	ggtgatgccccattccttgatcggctccgccgagacca aaagtccttaaaaggaagaggcaacaccc	-	-
HE603933	Human H1N1pdm2009 IAVs	Swine	H1N1		2010	France	A/swine/La_Reunion/0164/2010	ggtgatgccccattccttgatcggctccgccgagacca aaagtccttaaaaggaagaggcaacaccc	-	-
HE603926	Human H1N1pdm2009 IAVs	Swine	H1N1		2010	France	A/swine/La_Reunion/0167/2010	ggtgatgccccattccttgatcggctccgccgagacca aaagtccttaaaaggaagaggcaacaccc	-	-
CY096302	Human H1N1pdm2009 IAVs	Human	H1N1		2010	Thailand	A/Bangkok/INS478/2010	ggtgatgccccattccttgatcggctccgccgagatca aaaatccttaaaaggaagaggcaacaccc	-	*
JX625658	Human H1N1pdm2009 IAVs	Human	H1N1	pdm	2010	United_Kingdom	A/Northern_Ireland/04380108/2010	ggtgatgccccattccttgatcggctccgccgagatca aaaatccttaaaaggaagaggcaacaccc	-	*
CY080303	Human H1N1pdm2009 IAVs	Human	H1N1	pdm	2010	Thailand	A/Thailand/CU_B2357/2010	ggtgatgccccattccttgatcggctccgccgagatca aaagtcattaaaaggaagaggcaacaccc	-	*
KC222528	Human H1N1pdm2009 IAVs	Swine	H1N2		2010	Germany	A/swine/Papenburg/IDT12653/2010	ggtgatgccccattccttgatcggctccgccgagatca aaagtcattaaaaggaagaggcaacaccc	-	*
CY129786	Human H1N1pdm2009 IAVs	Human	H1N1	pdm	2010	Thailand	A/Bangkok/INS583/2010	ggtgatgccccattccttgatcggctccgccgagatca aaagtccctaaaaggaagaggcaacaccc	-	*
HE577052	Human H1N1pdm2009 IAVs	Swine	H1N1		2010	France	A/swine/Sarthe/0255/2010	ggtgatgccccattccttgatcggctccgccgagatca aaagtccctaaaaggaagaggcaacaccc	-	*
CY062991	Human H1N1pdm2009 IAVs	Human	H1N1	pdm	2010	Austria	A/Vienna/INS179/2010	ggtgatgccccattccttgatcggctccgccgagatca aaagtccttaaaaggaagaggcaacaccc	-	*
CY167776	Human H1N1pdm2009 IAVs	Human	H1N1	pdm	2010	USA	A/Tennessee/F1035/2010	ggtgatgccccattccttgatcggctccgccgagatca aaagtccttaaaaggaagaggcaacaccc	-	*
CY167416	Human H1N1pdm2009 IAVs	Human	H1N1	pdm	2010	USA	A/Tennessee/F1074/2010	ggtgatgccccattccttgatcggctccgccgagatca aaagtccttaaaaggaagaggcaacaccc	-	*
CY167368	Human H1N1pdm2009 IAVs	Human	H1N1	pdm	2010	USA	A/Tennessee/F1066c65/2010	ggtgatgccccattccttgatcggctccgccgagatca aaagtccttaaaaggaagaggcaacaccc	-	*
CY167376	Human H1N1pdm2009 IAVs	Human	H1N1	pdm	2010	USA	A/Tennessee/F1067/2010	ggtgatgccccattccttgatcggctccgccgagatca aaagtccttaaaaggaagaggcaacaccc	-	*
HQ541708	Human H1N1pdm2009 IAVs	Swine	H1N1		2010	China	A/swine/Yunnan/74/2010	ggtgatgccccattccttgatcggctccgccgagatca aaagtccttaaaaggaagaggcaacaccc	-	*
KC859090	Human H1N1pdm2009 IAVs	Swine	H1N1		2010	Thailand	A/swine/Thailand/NR145/2010	ggtgatgccccattccttgatcggctccgccgagatca aaagtccttaaaaggaagaggcaacaccc	-	*
KC859242	Human H1N1pdm2009 IAVs	Swine	H1N1		2010	Thailand	A/swine/Thailand/SB038/2010	ggtgatgccccattccttgatcggctccgccgagatca aaagtccttaaaaggaagaggcaacaccc	-	*
JX309081	Human H1N1pdm2009 IAVs	Human	H1N1	pdm	2010	Singapore	A/Singapore/EN008/2010	ggtgatgccccattccttgatcggctccgccgagatca aaagtccttaaaaggaagaggcaacaccc	-	*
JX309710	Human H1N1pdm2009 IAVs	Human	H1N1	pdm	2010	Singapore	A/Singapore/TT428/2010	ggtgatgccccattccttgatcggctccgccgagatca aaagtccttaaaaggaagaggcaacaccc	-	*
JX625642	Human H1N1pdm2009 IAVs	Human	H1N1		2010	United_Kingdom	A/England/03220137/2010	ggtgatgccccattccttgatcggctccgccgagatca aaagtccttaaaaggaagaggcaacaccc	-	*
JX625634	Human H1N1pdm2009 IAVs	Human	H1N1	pdm	2010	United_Kingdom	A/England/67/2010	ggtgatgccccattccttgatcggctccgccgagatca aaagtccttaaaaggaagaggcaacaccc	-	*
CY071339	Human H1N1pdm2009 IAVs	Human	H1N1	pdm	2010	Thailand	A/Bangkok/INS425/2010	ggtgatgccccattccttgatcggctccgccgagatca aaagtccttaaaaggaagaggcaacaccc	-	*

CY071347	Human H1N1pdm2009 IAVs	Human	H1N1	pdm	2010	Thailand	A/Bangkok/INS426/2010	ggtgatgccccattccttgatcggctccgccgagatca<br>aaagtccttaaaaggaagaggcaacaccc	-	*
CY071355	Human H1N1pdm2009 IAVs	Human	H1N1	pdm	2010	Thailand	A/Bangkok/INS427/2010	ggtgatgccccattccttgatcggctccgccgagatca<br>aaagtccttaaaaggaagaggcaacaccc	-	*
CY096494	Human H1N1pdm2009 IAVs	Human	H1N1	pdm	2010	Thailand	A/Bangkok/INS511/2010	ggtgatgccccattccttgatcggctccgccgagatca<br>aaagtccttaaaaggaagaggcaacaccc	-	*
CY063183	Human H1N1pdm2009 IAVs	Human	H1N1	pdm	2010	USA	A/California/VRDL10/2010	ggtgatgccccattccttgatcggctccgccgagatca<br>aaagtccttaaaaggaagaggcaacaccc	-	*
CY063199	Human H1N1pdm2009 IAVs	Human	H1N1	pdm	2010	USA	A/California/VRDL12/2010	ggtgatgccccattccttgatcggctccgccgagatca<br>aaagtccttaaaaggaagaggcaacaccc	-	*
CY063215	Human H1N1pdm2009 IAVs	Human	H1N1	pdm	2010	USA	A/California/VRDL14/2010	ggtgatgccccattccttgatcggctccgccgagatca<br>aaagtccttaaaaggaagaggcaacaccc	-	*
CY066467	Human H1N1pdm2009 IAVs	Human	H1N1	pdm	2010	USA	A/California/VRDL3/2010	ggtgatgccccattccttgatcggctccgccgagatca<br>aaagtccttaaaaggaagaggcaacaccc	-	*
CY066483	Human H1N1pdm2009 IAVs	Human	H1N1	pdm	2010	USA	A/California/VRDL5/2010	ggtgatgccccattccttgatcggctccgccgagatca<br>aaagtccttaaaaggaagaggcaacaccc	-	*
CY066507	Human H1N1pdm2009 IAVs	Human	H1N1	pdm	2010	USA	A/California/VRDL9/2010	ggtgatgccccattccttgatcggctccgccgagatca<br>aaagtccttaaaaggaagaggcaacaccc	-	*
CY093060	Human H1N1pdm2009 IAVs	Human	H1N1	pdm	2010	Thailand	A/Khon_Kaen/INS440/2010	ggtgatgccccattccttgatcggctccgccgagatca<br>aaagtccttaaaaggaagaggcaacaccc	-	*
CY098198	Human H1N1pdm2009 IAVs	Human	H1N1	pdm	2010	Thailand	A/Khon_Kaen/INS442/2010	ggtgatgccccattccttgatcggctccgccgagatca<br>aaagtccttaaaaggaagaggcaacaccc	-	*
CY098206	Human H1N1pdm2009 IAVs	Human	H1N1	pdm	2010	Thailand	A/Khon_Kaen/INS444/2010	ggtgatgccccattccttgatcggctccgccgagatca<br>aaagtccttaaaaggaagaggcaacaccc	-	*
CY096086	Human H1N1pdm2009 IAVs	Human	H1N1	pdm	2010	Thailand	A/Khon_Kaen/INS445/2010	ggtgatgccccattccttgatcggctccgccgagatca<br>aaagtccttaaaaggaagaggcaacaccc	-	*
CY096102	Human H1N1pdm2009 IAVs	Human	H1N1		2010	Thailand	A/Khon_Kaen/INS447/2010	ggtgatgccccattccttgatcggctccgccgagatca<br>aaagtccttaaaaggaagaggcaacaccc	-	*
CY096110	Human H1N1pdm2009 IAVs	Human	H1N1	pdm	2010	Thailand	A/Khon_Kaen/INS449/2010	ggtgatgccccattccttgatcggctccgccgagatca<br>aaagtccttaaaaggaagaggcaacaccc	-	*
CY096118	Human H1N1pdm2009 IAVs	Human	H1N1	pdm	2010	Thailand	A/Khon_Kaen/INS450/2010	ggtgatgccccattccttgatcggctccgccgagatca<br>aaagtccttaaaaggaagaggcaacaccc	-	*
CY096126	Human H1N1pdm2009 IAVs	Human	H1N1	pdm	2010	Thailand	A/Khon_Kaen/INS451/2010	ggtgatgccccattccttgatcggctccgccgagatca<br>aaagtccttaaaaggaagaggcaacaccc	-	*
CY096142	Human H1N1pdm2009 IAVs	Human	H1N1		2010	Thailand	A/Khon_Kaen/INS453/2010	ggtgatgccccattccttgatcggctccgccgagatca<br>aaagtccttaaaaggaagaggcaacaccc	-	*
CY096150	Human H1N1pdm2009 IAVs	Human	H1N1	pdm	2010	Thailand	A/Khon_Kaen/INS454/2010	ggtgatgccccattccttgatcggctccgccgagatca<br>aaagtccttaaaaggaagaggcaacaccc	-	*
CY093076	Human H1N1pdm2009 IAVs	Human	H1N1	pdm	2010	Thailand	A/Khon_Kaen/INS456/2010	ggtgatgccccattccttgatcggctccgccgagatca<br>aaagtccttaaaaggaagaggcaacaccc	-	*
CY096174	Human H1N1pdm2009 IAVs	Human	H1N1	pdm	2010	Thailand	A/Khon_Kaen/INS458/2010	ggtgatgccccattccttgatcggctccgccgagatca<br>aaagtccttaaaaggaagaggcaacaccc	-	*
CY096182	Human H1N1pdm2009 IAVs	Human	H1N1		2010	Thailand	A/Khon_Kaen/INS459/2010	ggtgatgccccattccttgatcggctccgccgagatca<br>aaagtccttaaaaggaagaggcaacaccc	-	*
CY096190	Human H1N1pdm2009 IAVs	Human	H1N1	pdm	2010	Thailand	A/Khon_Kaen/INS460/2010	ggtgatgccccattccttgatcggctccgccgagatca<br>aaagtccttaaaaggaagaggcaacaccc	-	*
CY098102	Human H1N1pdm2009 IAVs	Human	H1N1		2010	Thailand	A/Khon_Kaen/INS461/2010	ggtgatgccccattccttgatcggctccgccgagatca<br>aaagtccttaaaaggaagaggcaacaccc	-	*
CY096198	Human H1N1pdm2009 IAVs	Human	H1N1	pdm	2010	Thailand	A/Khon_Kaen/INS462/2010	ggtgatgccccattccttgatcggctccgccgagatca<br>aaagtccttaaaaggaagaggcaacaccc	-	*

KF411274	Human H1N1pdm2009 IAVs	Human	H1N1	pdm	2010	China	A/Qingdao/FF894/2010	ggtgatgccccattccttgatcggctccgccgagatca aaagtccttaaaaggaagaggcaacaccc	-	*
CY062142	Human H1N1pdm2009 IAVs	Human	H1N1		2010	USA	A/New_York/2960/2010	ggtgatgccccattccttgatcggctccgccgagatca aaagtccttaaaaggaagaggcaacaccc	-	*
CY064999	Human H1N1pdm2009 IAVs	Human	H1N1	pdm	2010	USA	A/New_York/3681/2010	ggtgatgccccattccttgatcggctccgccgagatca aaagtccttaaaaggaagaggcaacaccc	-	*
CY065007	Human H1N1pdm2009 IAVs	Human	H1N1	pdm	2010	USA	A/New_York/3682/2010	ggtgatgccccattccttgatcggctccgccgagatca aaagtccttaaaaggaagaggcaacaccc	-	*
CY065015	Human H1N1pdm2009 IAVs	Human	H1N1	pdm	2010	USA	A/New_York/3683/2010	ggtgatgccccattccttgatcggctccgccgagatca aaagtccttaaaaggaagaggcaacaccc	-	*
CY065023	Human H1N1pdm2009 IAVs	Human	H1N1	pdm	2010	USA	A/New_York/3834/2010	ggtgatgccccattccttgatcggctccgccgagatca aaagtccttaaaaggaagaggcaacaccc	-	*
KM027515	Human H1N1pdm2009 IAVs	Swine	H1N1		2010	China	A/swine/Guangdong/NS2801/2010	ggtgatgccccattccttgatcggctccgccgagatca aaagtccttaaaaggaagaggcaacaccc	-	*
HQ695944	Human H1N1pdm2009 IAVs	Swine	H1N1		2010	China	A/swine/Jangsu/46/2010	ggtgatgccccattccttgatcggctccgccgagatca aaagtccttaaaaggaagaggcaacaccc	-	*
HQ695960	Human H1N1pdm2009 IAVs	Swine	H1N1		2010	China	A/swine/Jangsu/49/2010	ggtgatgccccattccttgatcggctccgccgagatca aaagtccttaaaaggaagaggcaacaccc	-	*
JQ612495	Human H1N1pdm2009 IAVs	Swine	H1N1		2010	Hungary	A/swine/Hungary/7380/2010	ggtgatgccccattccttgatcggctccgccgagatca aaagtccttaaaaggaagaggcaacaccc	-	*
CY121844	Human H1N1pdm2009 IAVs	Human	H1N1	pdm	2010	USA	A/South_Carolina/10/2010	ggtgatgccccattccttgatcggctccgccgagatca aaagtccttaaaaggaagaggcaacaccc	-	*
CY061582	Human H1N1pdm2009 IAVs	Human	H1N1		2010	USA	A/Texas/JMS401/2010	ggtgatgccccattccttgatcggctccgccgagatca aaagtccttaaaaggaagaggcaacaccc	-	*
CY061111	Human H1N1pdm2009 IAVs	Human	H1N1	pdm	2010	USA	A/Texas/JMS402/2010	ggtgatgccccattccttgatcggctccgccgagatca aaagtccttaaaaggaagaggcaacaccc	-	*
CY061590	Human H1N1pdm2009 IAVs	Human	H1N1	pdm	2010	USA	A/Texas/JMS403/2010	ggtgatgccccattccttgatcggctccgccgagatca aaagtccttaaaaggaagaggcaacaccc	-	*
CY061127	Human H1N1pdm2009 IAVs	Human	H1N1	pdm	2010	USA	A/Texas/JMS405/2010	ggtgatgccccattccttgatcggctccgccgagatca aaagtccttaaaaggaagaggcaacaccc	-	*
CY061135	Human H1N1pdm2009 IAVs	Human	H1N1	pdm	2010	USA	A/Texas/JMS406/2010	ggtgatgccccattccttgatcggctccgccgagatca aaagtccttaaaaggaagaggcaacaccc	-	*
CY061159	Human H1N1pdm2009 IAVs	Human	H1N1	pdm	2010	USA	A/Texas/JMS409/2010	ggtgatgccccattccttgatcggctccgccgagatca aaagtccttaaaaggaagaggcaacaccc	-	*
CY061199	Human H1N1pdm2009 IAVs	Human	H1N1	pdm	2010	USA	A/Texas/JMS414/2010	ggtgatgccccattccttgatcggctccgccgagatca aaagtccttaaaaggaagaggcaacaccc	-	*
CY074986	Human H1N1pdm2009 IAVs	Human	H1N1	pdm	2010	Thailand	A/Thailand/CU_H1222/2010	ggtgatgccccattccttgatcggctccgccgagatca aaagtccttaaaaggaagaggcaacaccc	-	*
CY158405	Human H1N1pdm2009 IAVs	Swine	H1N2		2010	USA	A/swine/North_Carolina/SG1357/2010	ggtgatgccccattccttgatcggctccgccgagatca aaagtccttaaaaggaagaggcaacaccc	-	*
CY159995	Human H1N1pdm2009 IAVs	Swine	H1N1		2010	USA	A/swine/Oklahoma/02989/2010	ggtgatgccccattccttgatcggctccgccgagatca aaagtccttaaaaggaagaggcaacaccc	-	*
CY167392	Human H1N1pdm2009 IAVs	Human	H1N1		2010	USA	A/Tennessee/F1071/2010	ggtgatgccccattccttgatcggctccgccgagatca aaagtccttaaaaggaagaggcaacaccc	-	*
CY167408	Human H1N1pdm2009 IAVs	Human	H1N1	pdm	2010	USA	A/Tennessee/F1072c71/2010	ggtgatgccccattccttgatcggctccgccgagatca aaagtccttaaaaggaagaggcaacaccc	-	*
CY167328	Human H1N1pdm2009 IAVs	Human	H1N1	pdm	2010	USA	A/Tennessee/F1042A/2010	ggtgatgccccattccttgatcggctccgccgagatca aaagtccttaaaaggaagaggcaacaccc	-	*
CY167400	Human H1N1pdm2009 IAVs	Human	H1N1	pdm	2010	USA	A/Tennessee/F1071B/2010	ggtgatgccccattccttgatcggctccgccgagatca aaagtccttaaaaggaagaggcaacaccc	-	*

KC781171	Human H1N1pdm2009 IAVs	Human	H1N1	pdm	2010	USA	A/Alabama/03/2010	ggtgatgccccattccttgatcggctccgccgagatca aaagtccttaaaaggaagaggcaacaccc	-	*
KF918707	Human H1N1pdm2009 IAVs	Human	H1N1	pdm	2010	China	A/Beijing/132/2010	ggtgatgccccattccttgatcggctccgccgagatca aaagtccttaaaaggaagaggcaacaccc	-	*
KC780735	Human H1N1pdm2009 IAVs	Human	H1N1	pdm	2010	USA	A/California/05/2010	ggtgatgccccattccttgatcggctccgccgagatca aaagtccttaaaaggaagaggcaacaccc	-	*
KC781252	Human H1N1pdm2009 IAVs	Human	H1N1		2010	USA	A/Georgia/06/2010	ggtgatgccccattccttgatcggctccgccgagatca aaagtccttaaaaggaagaggcaacaccc	-	*
KC781393	Human H1N1pdm2009 IAVs	Human	H1N1	pdm	2010	USA	A/Nebraska/02/2010	ggtgatgccccattccttgatcggctccgccgagatca aaagtccttaaaaggaagaggcaacaccc	-	*
JX308961	Human H1N1pdm2009 IAVs	Human	H1N1	pdm	2010	Singapore	A/Singapore/GP33/2010	ggtgatgccccattccttgatcggctccgccgagatca aaagtccttaaaaggaagaggcaacaccc	-	*
JX309037	Human H1N1pdm2009 IAVs	Human	H1N1	pdm	2010	Singapore	A/Singapore/GP413/2010	ggtgatgccccattccttgatcggctccgccgagatca aaagtccttaaaaggaagaggcaacaccc	-	*
JX309053	Human H1N1pdm2009 IAVs	Human	H1N1	pdm	2010	Singapore	A/Singapore/GP449/2010	ggtgatgccccattccttgatcggctccgccgagatca aaagtccttaaaaggaagaggcaacaccc	-	*
JX309097	Human H1N1pdm2009 IAVs	Human	H1N1	pdm	2010	Singapore	A/Singapore/GP95/2010	ggtgatgccccattccttgatcggctccgccgagatca aaagtccttaaaaggaagaggcaacaccc	-	*
CY061809	Human H1N1pdm2009 IAVs	Swine	H1N1		2010	Hong_Kong	A/swine/Hong_Kong/189/2010	ggtgatgccccattccttgatcggctccgccgagatca aaagtccttaaaaggaagaggcaacaccc	-	*
JN375252	Human H1N1pdm2009 IAVs	Swine	H1N1		2010	China	A/swine/Guangdong/50/2010	ggtgatgccccattccttgatcggctccgccgagatca aaagtccttaaaaggaagaggcaacaccc	-	*
CY071291	Human H1N1pdm2009 IAVs	Human	H1N1	pdm	2010	Greece	A/Athens/INS414/2010	ggtgatgccccattccttgatcggctccgccgagatca aaagtccttaaaaggaagaggcaacaccc	-	*
CY066451	Human H1N1pdm2009 IAVs	Human	H1N1	pdm	2010	USA	A/California/VRDL1/2010	ggtgatgccccattccttgatcggctccgccgagatca aaagtccttaaaaggaagaggcaacaccc	-	*
CY073971	Human H1N1pdm2009 IAVs	Human	H1N1	pdm	2010	Argentina	A/Argentina/HNRG102/2010	ggtgatgccccattccttgatcggctccgccgagatca aaagtccttaaaaggaagaggcaacaccc	-	*
KJ690436	Human H1N1pdm2009 IAVs	Human	H1N1	pdm	2010	Uganda	A/Uganda/MUWRP_224/2010	ggtgatgccccattccttgatcggctccgccgagatca aaagtccttaaaaggaagaggcaacaccc	-	*
KJ690499	Human H1N1pdm2009 IAVs	Human	H1N1	pdm	2010	Uganda	A/Uganda/MUWRP_233/2010	ggtgatgccccattccttgatcggctccgccgagatca aaagtccttaaaaggaagaggcaacaccc	-	*
CY096246	Human H1N1pdm2009 IAVs	Human	H1N1	pdm	2010	Australia	A/Melbourne/INS471/2010	ggtgatgccccattccttgatcggctccgccgagatca aaagtccttaaaaggaagaggcaacaccc	-	*
JX309706	Human H1N1pdm2009 IAVs	Human	H1N1	pdm	2010	Singapore	A/Singapore/TT421/2010	ggtgatgccccattccttgatcggctccgccgagatca aaagtccttaaaaggaagaggcaacaccc	-	*
KC781116	Human H1N1pdm2009 IAVs	Human	H1N1	pdm	2010	USA	A/Maryland/05/2010	ggtgatgccccattccttgatcggctccgccgagatca aaagtccttaaaaggaagaggcaacaccc	-	*
JX309021	Human H1N1pdm2009 IAVs	Human	H1N1	pdm	2010	Singapore	A/Singapore/GP256/2010	ggtgatgccccattccttgatcggctccgccgagatca aaagtccttaaaaggaagaggcaacaccc	-	*
JX309061	Human H1N1pdm2009 IAVs	Human	H1N1	pdm	2010	Singapore	A/Singapore/GP437/2010	ggtgatgccccattccttgatcggctccgccgagatca aaagtccttaaaaggaagaggcaacaccc	-	*
JX309077	Human H1N1pdm2009 IAVs	Human	H1N1	pdm	2010	Singapore	A/Singapore/GP512/2010	ggtgatgccccattccttgatcggctccgccgagatca aaagtccttaaaaggaagaggcaacaccc	-	*
CY096334	Human H1N1pdm2009 IAVs	Human	H1N1	pdm	2010	Thailand	A/Bangkok/INS482/2010	ggtgatgccccattccttgatcggctccgccgagatca aaagtccttaaaaggaagaggcaacaccc	-	*
HQ695947	Human H1N1pdm2009 IAVs	Swine	H1N1		2010	China	A/swine/Jangsu/48/2010	ggtgatgccccattccttgatcggctccgccgagatca aaagtccttaaaaggaagaggcaacaccc	-	*
CY061175	Human H1N1pdm2009 IAVs	Human	H1N1	pdm	2010	USA	A/Texas/JMS411/2010	ggtgatgccccattccttgatcggctccgccgagatca aaagtccttaaaaggaagaggcaacaccc	-	*

JX309300	Human H1N1pdm2009 IAVs	Human	H1N1	pdm	2010	Singapore	A/Singapore/KK136/2010	ggtgatgccccattccttgatcggctccgccgagatca aaagtccttaaaaggaagaggcaacaccc	-	*
JX309308	Human H1N1pdm2009 IAVs	Human	H1N1	pdm	2010	Singapore	A/Singapore/KK140/2010	ggtgatgccccattccttgatcggctccgccgagatca aaagtccttaaaaggaagaggcaacaccc	-	*
CY063007	Human H1N1pdm2009 IAVs	Human	H1N1	pdm	2010	Estonia	A/Tallinn/INS182/2010	ggtgatgccccattccttgatcggctccgccgagatca aaagtccttaaaaggaagaggcaacaccc	-	*
CY063015	Human H1N1pdm2009 IAVs	Human	H1N1	pdm	2010	Estonia	A/Tallinn/INS183/2010	ggtgatgccccattccttgatcggctccgccgagatca aaagtccttaaaaggaagaggcaacaccc	-	*
CY072530	Human H1N1pdm2009 IAVs	Human	H1N1	pdm	2010	Estonia	A/Tallinn/INS374/2010	ggtgatgccccattccttgatcggctccgccgagatca aaagtccttaaaaggaagaggcaacaccc	-	*
KC780226	Human H1N1pdm2009 IAVs	Human	H1N1	pdm	2010	USA	A/Colorado/02/2010	ggtgatgccccattccttgatcggctccgccgagatca aaagtccttaaaaggaagaggcaacaccc	-	*
CY071299	Human H1N1pdm2009 IAVs	Human	H1N1	pdm	2010	Greece	A/Athens/INS417/2010	ggtgatgccccattccttgatcggctccgccgagatca aaagtccttaaaaggaagaggcaacaccc	-	*
CY083821	Human H1N1pdm2009 IAVs	Human	H1N1	pdm	2010	USA	A/Los_Angeles/INS423/2010	ggtgatgccccattccttgatcggctccgccgagatca aaagtccttaaaaggaagaggcaacaccc	-	*
CY075868	Human H1N1pdm2009 IAVs	Swine	H1N2		2010	Argentina	A/swine/Argentina/CIP051_StaFeN 2/2010	ggtgatgccccattccttgatcggctccgccgagatca aaagtccttaaaaggaagaggcaacaccc	-	*
CY107457	Human H1N1pdm2009 IAVs	Human	H1N1	pdm	2010	United_Kingdom	A/Scotland/Dundee_10V070791/2 010	ggtgatgccccattccttgatcggctccgccgagatca aaagtccttaaaaggaagaggcaacaccc	-	*
JX309434	Human H1N1pdm2009 IAVs	Human	H1N1	pdm	2010	Singapore	A/Singapore/SS09/2010	ggtgatgccccattccttgatcggctccgccgagatca aaagtccttaaaaggaagaggcaacaccc	-	*
CY065055	Human H1N1pdm2009 IAVs	Human	H1N1	pdm	2010	USA	A/New_York/4662/2010	ggtgatgccccattccttgatcggctccgccgagatca aaagtccttaaaaggaagaggcaacaccc	-	*
JF275937	Human H1N1pdm2009 IAVs	Swine	H1N1		2010	China	A/swine/Nanchang/5/2010	ggtgatgccccattccttgatcggctccgccgagatca aaagtccttaaaaggaagaggcaacaccc	-	*
JF275945	Human H1N1pdm2009 IAVs	Swine	H1N1		2010	China	A/swine/Nanchang/6/2010	ggtgatgccccattccttgatcggctccgccgagatca aaagtccttaaaaggaagaggcaacaccc	-	*
KJ023115	Human H1N1pdm2009 IAVs	Human	H1N1	pdm	2010	India	A/Chattisgarh/025/2010	ggtgatgccccattccttgatcggctccgccgagatca aaagtccttaaaaggaagaggcaacaccc	-	*
KJ023116	Human H1N1pdm2009 IAVs	Human	H1N1	pdm	2010	India	A/Chattisgarh/026/2010	ggtgatgccccattccttgatcggctccgccgagatca aaagtccttaaaaggaagaggcaacaccc	-	*
JX625786	Human H1N1pdm2009 IAVs	Human	H1N1	pdm	2010	United_Kingdom	A/England/04960232/2010	ggtgatgccccattccttgatcggctccgccgagatca aaagtccttaaaaggaagaggcaacaccc	-	*
JX625674	Human H1N1pdm2009 IAVs	Human	H1N1	pdm	2010	United_Kingdom	A/England/139/2010	ggtgatgccccattccttgatcggctccgccgagatca aaagtccttaaaaggaagaggcaacaccc	-	*
JX625810	Human H1N1pdm2009 IAVs	Human	H1N1	pdm	2010	United_Kingdom	A/England/213/2010	ggtgatgccccattccttgatcggctccgccgagatca aaagtccttaaaaggaagaggcaacaccc	-	*
KJ023117	Human H1N1pdm2009 IAVs	Human	H1N1	pdm	2010	India	A/Goa/027/2010	ggtgatgccccattccttgatcggctccgccgagatca aaagtccttaaaaggaagaggcaacaccc	-	*
KJ023124	Human H1N1pdm2009 IAVs	Human	H1N1	pdm	2010	India	A/Goa/034/2010	ggtgatgccccattccttgatcggctccgccgagatca aaagtccttaaaaggaagaggcaacaccc	-	*
KJ023125	Human H1N1pdm2009 IAVs	Human	H1N1	pdm	2010	India	A/Goa/035/2010	ggtgatgccccattccttgatcggctccgccgagatca aaagtccttaaaaggaagaggcaacaccc	-	*
KJ023118	Human H1N1pdm2009 IAVs	Human	H1N1	pdm	2010	India	A/Haryana/028/2010	ggtgatgccccattccttgatcggctccgccgagatca aaagtccttaaaaggaagaggcaacaccc	-	*
KJ023119	Human H1N1pdm2009 IAVs	Human	H1N1	pdm	2010	India	A/Haryana/029/2010	ggtgatgccccattccttgatcggctccgccgagatca aaagtccttaaaaggaagaggcaacaccc	-	*
KF897805	Human H1N1pdm2009 IAVs	Human	H1N1	pdm	2010	France	A/Limoges/1159/2010	ggtgatgccccattccttgatcggctccgccgagatca aaagtccttaaaaggaagaggcaacaccc	-	*

KJ023120	Human H1N1pdm2009 IAVs	Human	H1N1	pdm	2010	India	A/Punjab/030/2010	ggtgatgccccattccttgatcggctccgccgagatca aaagtccttaaaaggaagaggcaacaccc	-	*
KJ023121	Human H1N1pdm2009 IAVs	Human	H1N1	pdm	2010	India	A/Punjab/031/2010	ggtgatgccccattccttgatcggctccgccgagatca aaagtccttaaaaggaagaggcaacaccc	-	*
KJ023122	Human H1N1pdm2009 IAVs	Human	H1N1		2010	India	A/Uttar_Pradesh/032/2010	ggtgatgccccattccttgatcggctccgccgagatca aaagtccttaaaaggaagaggcaacaccc	-	*
KJ023123	Human H1N1pdm2009 IAVs	Human	H1N1	pdm	2010	India	A/Uttrakhand/033/2010	ggtgatgccccattccttgatcggctccgccgagatca aaagtccttaaaaggaagaggcaacaccc	-	*
JX309804	Human H1N1pdm2009 IAVs	Human	H1N1	pdm	2010	Singapore	A/Singapore/GP4138/2010	ggtgatgccccattccttgatcggctccgccgagatca aaagtccttaaaaggaagaggcaacaccc	-	*
JX309796	Human H1N1pdm2009 IAVs	Human	H1N1	pdm	2010	Singapore	A/Singapore/GP4099/2010	ggtgatgccccattccttgatcggctccgccgagatca aaagtccttaaaaggaagaggcaacaccc	-	*
KC882134	Human H1N1pdm2009 IAVs	Human	H1N1	pdm	2010	USA	A/Pennsylvania/17/2010	ggtgatgccccattccttgatcggctccgccgagatca aaagtccttaaaaggaagaggcaacaccc	-	*
CY065071	Human H1N1pdm2009 IAVs	Human	H1N1	pdm	2010	USA	A/New_York/7420/2010	ggtgatgccccattccttgatcggctccgccgagatca aaagtccttaaaaggaagaggcaacaccc	-	*
AB704490	Human H1N1pdm2009 IAVs	Human	H1N1	pdm	2010	Japan	A/Tochigi/10/2010	ggtgatgccccattccttgatcggctccgccgagatca aaagtccttaaaaggaagaggcaacaccc	-	*
CY158997	Human H1N1pdm2009 IAVs	Swine	H1N1		2010	USA	A/swine/Minnesota/02976/2010	ggtgatgccccattccttgatcggctccgccgagatca aaagtccttaaaaggaagaggcaacaccc	-	*
CY066499	Human H1N1pdm2009 IAVs	Human	H1N1	pdm	2010	USA	A/California/VRDL8/2010	ggtgatgccccattccttgatcggctccgccgagatca aaagtccttaaaaggaagaggcaacaccc	-	*
JQ695883	Human H1N1pdm2009 IAVs	Swine	H1N1		2010	China	A/swine/Shandong/361/2010	ggtgatgccccattccttgatcggctccgccgagatca aaagtccttaaaaggaagaggcaacaccc	-	*
JQ695875	Human H1N1pdm2009 IAVs	Swine	H1N1		2010	China	A/swine/Shandong/327/2010	ggtgatgccccattccttgatcggctccgccgagatca aaagtccttaaaaggaagaggcaacaccc	-	*
JQ695867	Human H1N1pdm2009 IAVs	Swine	H1N1		2010	China	A/swine/Shandong/94/2010	ggtgatgccccattccttgatcggctccgccgagatca aaagtccttaaaaggaagaggcaacaccc	-	*
CY115947	Human H1N1pdm2009 IAVs	Swine	H1N1		2010	United_Kingdom	A/swine/England/10/2010	ggtgatgccccattccttgatcggctccgccgagatca aaagtccttaaaaggaagaggcaacaccc	-	*
JQ023774	Human H1N1pdm2009 IAVs	Swine	H1N1		2010	USA	A/swine/Minnesota/0432/2010	ggtgatgccccattccttgatcggctccgccgagatca aaagtccttaaaaggaagaggcaacaccc	-	*
CY063191	Human H1N1pdm2009 IAVs	Human	H1N1	pdm	2010	USA	A/California/VRDL11/2010	ggtgatgccccattccttgatcggctccgccgagatca aaagtccttaaaaggaagaggcaacaccc	-	*
CY063207	Human H1N1pdm2009 IAVs	Human	H1N1	pdm	2010	USA	A/California/VRDL13/2010	ggtgatgccccattccttgatcggctccgccgagatca aaagtccttaaaaggaagaggcaacaccc	-	*
CY167384	Human H1N1pdm2009 IAVs	Human	H1N1	pdm	2010	USA	A/Tennessee/F1068/2010	ggtgatgccccattccttgatcggctccgccgagatca aaagtccttaaaaggaagaggcaacaccc	-	*
CY167456	Human H1N1pdm2009 IAVs	Human	H1N1	pdm	2010	USA	A/Tennessee/F1089/2010	ggtgatgccccattccttgatcggctccgccgagatca aaagtccttaaaaggaagaggcaacaccc	-	*
KC781451	Human H1N1pdm2009 IAVs	Human	H1N1	pdm	2010	USA	A/Louisiana/01/2010	ggtgatgccccattccttgatcggctccgccgagatca aaagtccttaaaaggaagaggcaacaccc	-	*
JX309029	Human H1N1pdm2009 IAVs	Human	H1N1	pdm	2010	Singapore	A/Singapore/GP368/2010	ggtgatgccccattccttgatcggctccgccgagatca aaagtccttaaaaggaagaggcaacaccc	-	*
CY062182	Human H1N1pdm2009 IAVs	Human	H1N1		2010	USA	A/New_York/3250/2010	ggtgatgccccattccttgatcggctccgccgagatca aaagtccttaaaaggaagaggcaacaccc	-	*
CY062190	Human H1N1pdm2009 IAVs	Human	H1N1	pdm	2010	USA	A/New_York/3251/2010	ggtgatgccccattccttgatcggctccgccgagatca aaagtccttaaaaggaagaggcaacaccc	-	*
CY065063	Human H1N1pdm2009 IAVs	Human	H1N1	pdm	2010	USA	A/New_York/6530/2010	ggtgatgccccattccttgatcggctccgccgagatca aaagtccttaaaaggaagaggcaacaccc	-	*

CY098094	Human H1N1pdm2009 IAVs	Human	H1N1	pdm	2010	Chile	A/Chile/89/2010	ggtgatgccccattccttgatcggctccgccgagatca aaagtccttaaaaggaagaggcaacaccc	-	*
JX308989	Human H1N1pdm2009 IAVs	Human	H1N1	pdm	2010	Singapore	A/Singapore/TT01/2010	ggtgatgccccattccttgatcggctccgccgagatca aaagtccttaaaaggaagaggcaacaccc	-	*
CY071211	Human H1N1pdm2009 IAVs	Human	H1N1	pdm	2010	Germany	A/Frankfurt/INS402/2010	ggtgatgccccattccttgatcggctccgccgagatca aaagtccttaaaaggaagaggcaacaccc	-	*
CY071219	Human H1N1pdm2009 IAVs	Human	H1N1	pdm	2010	Germany	A/Frankfurt/INS403/2010	ggtgatgccccattccttgatcggctccgccgagatca aaagtccttaaaaggaagaggcaacaccc	-	*
CY096070	Human H1N1pdm2009 IAVs	Human	H1N1	pdm	2010	Thailand	A/Khon_Kaen/INS441/2010	ggtgatgccccattccttgatcggctccgccgagatca aaagtccttaaaaggaagaggcaacaccc	-	*
CY093068	Human H1N1pdm2009 IAVs	Human	H1N1	pdm	2010	Thailand	A/Khon_Kaen/INS448/2010	ggtgatgccccattccttgatcggctccgccgagatca aaagtccttaaaaggaagaggcaacaccc	-	*
CY096134	Human H1N1pdm2009 IAVs	Human	H1N1	pdm	2010	Thailand	A/Khon_Kaen/INS452/2010	ggtgatgccccattccttgatcggctccgccgagatca aaagtccttaaaaggaagaggcaacaccc	-	*
CY071379	Human H1N1pdm2009 IAVs	Human	H1N1	pdm	2010	Germany	A/Berlin/INS430/2010	ggtgatgccccattccttgatcggctccgccgagatca aaagtccttaaaaggaagaggcaacaccc	-	*
HQ695975	Human H1N1pdm2009 IAVs	Swine	H1N1		2010	China	A/swine/Jangsu/295/2010	ggtgatgccccattccttgatcggctccgccgagatca aaagtccttaaaaggaagaggcaacaccc	-	*
CY062110	Human H1N1pdm2009 IAVs	Human	H1N1	pdm	2010	USA	A/New_York/2372/2010	ggtgatgccccattccttgatcggctccgccgagatca aaagtccttaaaaggaagaggcaacaccc	-	*
JX309089	Human H1N1pdm2009 IAVs	Human	H1N1	pdm	2010	Singapore	A/Singapore/TT31/2010	ggtgatgccccattccttgatcggctccgccgagatca aaagtccttaaaaggaagaggcaacaccc	-	*
KC781390	Human H1N1pdm2009 IAVs	Human	H1N1	pdm	2010	USA	A/Alaska/01/2010	ggtgatgccccattccttgatcggctccgccgagatca aaagtccttaaaaggaagaggcaacaccc	-	*
CY167360	Human H1N1pdm2009 IAVs	Human	H1N1	pdm	2010	USA	A/Tennessee/F1063A/2010	ggtgatgccccattccttgatcggctccgccgagatca aaagtccttaaaaggaagaggcaacaccc	-	*
CY061207	Human H1N1pdm2009 IAVs	Human	H1N1	pdm	2010	USA	A/Texas/JMS415/2010	ggtgatgccccattccttgatcggctccgccgagatca aaagtccttaaaaggaagaggcaacaccc	-	*
CY062078	Human H1N1pdm2009 IAVs	Human	H1N1	pdm	2010	USA	A/New_York/1796/2010	ggtgatgccccattccttgatcggctccgccgagatca aaagtccttaaaaggaagaggcaacaccc	-	*
CY062166	Human H1N1pdm2009 IAVs	Human	H1N1	pdm	2010	USA	A/New_York/3230/2010	ggtgatgccccattccttgatcggctccgccgagatca aaagtccttaaaaggaagaggcaacaccc	-	*
CY065031	Human H1N1pdm2009 IAVs	Human	H1N1	pdm	2010	USA	A/New_York/3835/2010	ggtgatgccccattccttgatcggctccgccgagatca aaagtccttaaaaggaagaggcaacaccc	-	*
CY065039	Human H1N1pdm2009 IAVs	Human	H1N1	pdm	2010	USA	A/New_York/3866/2010	ggtgatgccccattccttgatcggctccgccgagatca aaagtccttaaaaggaagaggcaacaccc	-	*
CY061151	Human H1N1pdm2009 IAVs	Human	H1N1	pdm	2010	USA	A/Texas/JMS408/2010	ggtgatgccccattccttgatcggctccgccgagatca aaagtccttaaaaggaagaggcaacaccc	-	*
KC780690	Human H1N1pdm2009 IAVs	Human	H1N1	pdm	2010	USA	A/Indiana/03/2010	ggtgatgccccattccttgatcggctccgccgagatca aaagtccttaaaaggaagaggcaacaccc	-	*
KC780533	Human H1N1pdm2009 IAVs	Human	H1N1	pdm	2010	USA	A/Florida/02/2010	ggtgatgccccattccttgatcggctccgccgagatca aaagtccttaaaaggaagaggcaacaccc	-	*
JX309133	Human H1N1pdm2009 IAVs	Human	H1N1	pdm	2010	Singapore	A/Singapore/KK25/2010	ggtgatgccccattccttgatcggctccgccgagatca aaagtccttaaaaggaagaggcaacaccc	-	*
KM029627	Human H1N1pdm2009 IAVs	Swine	H1N1		2010	China	A/swine/Hong_Kong/3065/2010	ggtgatgccccattccttgatcggctccgccgagatca aaagtccttaaaaggaagaggcaacaccc	-	*
JX309121	Human H1N1pdm2009 IAVs	Human	H1N1	pdm	2010	Singapore	A/Singapore/GP311/2010	ggtgatgccccattccttgatcggctccgccgagatca aaagtccttaaaaggaagaggcaacaccc	-	*
CY096598	Human H1N1pdm2009 IAVs	Human	H1N1	pdm	2010	USA	A/District_of_Columbia/INS527/2010	ggtgatgccccattccttgatcggctccgccgagatca aaagtccttaaaaggaagaggcaacaccc	-	*

CY065047	Human H1N1pdm2009 IAVs	Human	H1N1	pdm	2010	USA	A/New_York/4294/2010	ggtgatgccccattccttgatcggctccgccgagatca aaagtccttaaaaggaagaggcaacaccc	-	*
CY093105	Human H1N1pdm2009 IAVs	Human	H1N1	pdm	2010	Australia	A/Sydney/DD3_27/2010	ggtgatgccccattccttgatcggctccgccgagatca aaagtccttaaaaggaagaggcaacaccc	-	*
CY160038	Human H1N1pdm2009 IAVs	Swine	H1N1		2010	USA	A/swine/Indiana/SG1367/2010	ggtgatgccccattccttgatcggctccgccgagatca aaagtccttaaaaggaagaggcaacaccc	-	*
HM189607	Human H1N1pdm2009 IAVs	Swine	H1N1		2010	South_Korea	A/swine/Korea/SCJ33/2010	ggtgatgccccattccttgatcggctccgccgagatca aaagtccttaaaaggaagaggcaacaccc	-	*
HM189608	Human H1N1pdm2009 IAVs	Swine	H1N1		2010	South_Korea	A/swine/Korea/SCJ41/2010	ggtgatgccccattccttgatcggctccgccgagatca aaagtccttaaaaggaagaggcaacaccc	-	*
HM189609	Human H1N1pdm2009 IAVs	Swine	H1N1		2010	South_Korea	A/swine/Korea/SCJ42/2010	ggtgatgccccattccttgatcggctccgccgagatca aaagtccttaaaaggaagaggcaacaccc	-	*
HM189606	Human H1N1pdm2009 IAVs	Swine	H1N1		2010	South_Korea	A/swine/Korea/SCJ28/2010	ggtgatgccccattccttgatcggctccgccgagatca aaagtccttaaaaggaagaggcaacaccc	-	*
CY064991	Human H1N1pdm2009 IAVs	Human	H1N1	pdm	2010	USA	A/California/VRDL6/2010	ggtgatgccccattccttgatcggctccgccgagatca aaagtccttaaaaggaagaggcaacaccc	-	*
JX309093	Human H1N1pdm2009 IAVs	Human	H1N1	pdm	2010	Singapore	A/Singapore/GP75/2010	ggtgatgccccattccttgatcggctccgccgagatca aaagtccttaaaaggaagaggcaacaccc	-	*
CY096158	Human H1N1pdm2009 IAVs	Human	H1N1	pdm	2010	Thailand	A/Khon_Kaen/INS455/2010	ggtgatgccccattccttgatcggctccgccgagatca aaagtccttaaaaggaagaggcaacaccc	-	*
CY061183	Human H1N1pdm2009 IAVs	Human	H1N1	pdm	2010	USA	A/Texas/JMS412/2010	ggtgatgccccattccttgatcggctccgccgagatca aaagtccttaaaaggaagaggcaacaccc	-	*
CY092642	Human H1N1pdm2009 IAVs	Human	H1N1		2010	Australia	A/Sydney/DD3_37/2010	ggtgatgccccattccttgatcggctccgccgagatca aaagtccttaaaaggaagaggcaacaccc	-	*
CY071179	Human H1N1pdm2009 IAVs	Human	H1N1	pdm	2010	Greece	A/Athens/INS397/2010	ggtgatgccccattccttgatcggctccgccgagatca aaagtccttaaaaggaagaggcaacaccc	-	*
CY065876	Human H1N1pdm2009 IAVs	Human	H1N1	pdm	2010	Netherlands	A/Netherlands/2631b/2010	ggtgatgccccattccttgatcggctccgccgagatca aaagtccttaaaaggaagaggcaacaccc	-	*
CY090865	Human H1N1pdm2009 IAVs	Human	H1N1	pdm	2010	Netherlands	A/Netherlands/2631c/2010	ggtgatgccccattccttgatcggctccgccgagatca aaagtccttaaaaggaagaggcaacaccc	-	*
CY071147	Human H1N1pdm2009 IAVs	Human	H1N1	pdm	2010	Greece	A/Athens/INS390/2010	ggtgatgccccattccttgatcggctccgccgagatca aaagtccttaaaaggaagaggcaacaccc	-	*
CY071155	Human H1N1pdm2009 IAVs	Human	H1N1	pdm	2010	Greece	A/Athens/INS391/2010	ggtgatgccccattccttgatcggctccgccgagatca aaagtccttaaaaggaagaggcaacaccc	-	*
CY083814	Human H1N1pdm2009 IAVs	Human	unknow	pdm	2010	Greece	A/Athens/INS387/2010	ggtgatgccccattccttgatcggctccgccgagatca aaagtccttaaaaggaagaggcaacaccc	-	*
CY071267	Human H1N1pdm2009 IAVs	Human	H1N1	pdm	2010	Greece	A/Athens/INS411/2010	ggtgatgccccattccttgatcggctccgccgagatca aaagtccttaaaaggaagaggcaacaccc	-	*
CY062094	Human H1N1pdm2009 IAVs	Human	H1N1	pdm	2010	USA	A/New_York/1999/2010	ggtgatgccccattccttgatcggctccgccgagatca aaagtccttaaaaggaagaggcaacaccc	-	*
CY071371	Human H1N1pdm2009 IAVs	Human	H1N1	pdm	2010	USA	A/Newark/INS429/2010	ggtgatgccccattccttgatcggctccgccgagatca aaagtccttaaaaggaagaggcaacaccc	-	*
CY159970	Human H1N1pdm2009 IAVs	Swine	H1N1		2010	USA	A/swine/Arkansas/SG1347/2010	ggtgatgccccattccttgatcggctccgccgagatca aaagtccttaaaaggaagaggcaacaccc	-	*
JX309426	Human H1N1pdm2009 IAVs	Human	H1N1	pdm	2010	Singapore	A/Singapore/SS08/2010	ggtgatgccccattccttgatcggctccgccgagatca aaagtccttaaaaggaagaggcaacaccc	-	*
KC473858	Human H1N1pdm2009 IAVs	Human	H1N1	pdm	2010	China	A/Guangzhou/GIRD74/2010	ggtgatgccccattccttgatcggctccgccgagatca aaagtccttaaaaggaagaggcaacaccc	-	*
CY096094	Human H1N1pdm2009 IAVs	Human	H1N1	pdm	2010	Thailand	A/Khon_Kaen/INS446/2010	ggtgatgccccattccttgatcggctccgccgagatca aaagtccttaaaaggaagaggcaacaccc	-	*

JX309914	Human H1N1pdm2009 IAVs	Human	H1N1	pdm	2010	Singapore	A/Singapore/GP4406/2010	ggtgatgccccattccttgatcggctccgccgagatca aaagtccttaaaaggaagaggcaacaccc	-	*
JX309618	Human H1N1pdm2009 IAVs	Human	H1N1	pdm	2010	Singapore	A/Singapore/KK349/2010	ggtgatgccccattccttgatcggctccgccgagatca aaagtccttaaaaggaagaggcaacaccc	-	*
CY088842	Human H1N1pdm2009 IAVs	Human	H1N1		2010	Thailand	A/Thailand/CU_H2543/2010	ggtgatgccccattccttgatcggctccgccgagatca aaagtccttaaaaggaagaggcaacaccc	-	*
JX875031	Human H1N1pdm2009 IAVs	Human	H1N1	pdm	2010	USA	A/Kentucky/180/2010	ggtgatgccccattccttgatcggctccgccgagatca aaagtccttaaaaggaagaggcaacaccc	-	*
KC780500	Human H1N1pdm2009 IAVs	Human	H1N1	pdm	2010	USA	A/Utah/02/2010	ggtgatgccccattccttgatcggctccgccgagatca aaagtccttaaaaggaagaggcaacaccc	-	*
KC780886	Human H1N1pdm2009 IAVs	Human	H1N1	pdm	2010	USA	A/Nevada/01/2010	ggtgatgccccattccttgatcggctccgccgagatca aaagtccttaaaaggaagaggcaacaccc	-	*
CY066459	Human H1N1pdm2009 IAVs	Human	H1N1	pdm	2010	USA	A/California/VRDL2/2010	ggtgatgccccattccttgatcggctccgccgagatca aaagtccttaaaaggaagaggcaacaccc	-	*
GU562462	Human H1N1pdm2009 IAVs	Human	H1N1		2010	Russia	A/Karasuk/01/2010	ggtgatgccccattccttgatcggctccgccgagatca aaagtccttaaaaggaagaggcaacaccc	-	*
CY096166	Human H1N1pdm2009 IAVs	Human	H1N1	pdm	2010	Thailand	A/Khon_Kaen/INS457/2010	ggtgatgccccattccttgatcggctccgccgagatca aaagtccttaaaaggaagaggcaacaccc	-	*
KF897789	Human H1N1pdm2009 IAVs	Human	H1N1	pdm	2010	France	A/Lyon/52.16/2010	ggtgatgccccattccttgatcggctccgccgagatca aaagtccttaaaaggaagaggcaacaccc	-	*
CY061191	Human H1N1pdm2009 IAVs	Human	H1N1	pdm	2010	USA	A/Texas/JMS413/2010	ggtgatgccccattccttgatcggctccgccgagatca aaagtccttaaaaggaagaggcaacaccc	-	*
CY061143	Human H1N1pdm2009 IAVs	Human	H1N1	pdm	2010	USA	A/Texas/JMS407/2010	ggtgatgccccattccttgatcggctccgccgagatca aaagtccttaaaaggaagaggcaacaccc	-	*
CY075063	Human H1N1pdm2009 IAVs	Human	H1N1	pdm	2010	Greece	A/Athens/INS392/2010	ggtgatgccccattccttgatcggctccgccgagatca aaagtccttaaaaggaagaggcaacaccc	-	*
CY096606	Human H1N1pdm2009 IAVs	Human	H1N1	pdm	2010	USA	A/Cambridge/INS528/2010	ggtgatgccccattccttgatcggctccgccgagatca aaagtccttaaaaggaagaggcaacaccc	-	*
CY061119	Human H1N1pdm2009 IAVs	Human	H1N1	pdm	2010	USA	A/Texas/JMS404/2010	ggtgatgccccattccttgatcggctccgccgagatca aaagtccttaaaaggaagaggcaacaccc	-	*
KC780237	Human H1N1pdm2009 IAVs	Human	H1N1	pdm	2010	USA	A/Georgia/01/2010	ggtgatgccccattccttgatcggctccgccgagatca aaagtccttaaaaggaagaggcaacaccc	-	*
KC781265	Human H1N1pdm2009 IAVs	Human	H1N1	pdm	2010	USA	A/Georgia/04/2010	ggtgatgccccattccttgatcggctccgccgagatca aaagtccttaaaaggaagaggcaacaccc	-	*
KC780608	Human H1N1pdm2009 IAVs	Human	H1N1	pdm	2010	USA	A/California/02/2010	ggtgatgccccattccttgatcggctccgccgagatca aaagtccttaaaaggaagaggcaacaccc	-	*
CY066491	Human H1N1pdm2009 IAVs	Human	H1N1	pdm	2010	USA	A/California/VRDL7/2010	ggtgatgccccattccttgatcggctccgccgagatca aaagtccttaaaaggaagaggcaacaccc	-	*
CY083539	Human H1N1pdm2009 IAVs	Human	H1N1	pdm	2010	Mexico	A/Mexico_City/WRAIR1752N/2010	ggtgatgccccattccttgatcggctccgccgagatca aaagtccttaaaaggaagaggcaacaccc	-	*
CY116131	Human H1N1pdm2009 IAVs	Swine	H1N1		2010	United_Kingdom	A/swine/England/373/2010	ggtgatgccccattccttgatcggctccgccgagatca aaagtccttaaaaggaagaggcaacaccc	-	*
KC781401	Human H1N1pdm2009 IAVs	Human	H1N1	pdm	2010	USA	A/Utah/03/2010	ggtgatgccccattccttgatcggctccgccgagatca aaagtccttaaaaggaagaggcaacaccc	-	*
CY093209	Human H1N1pdm2009 IAVs	Human	H1N1	pdm	2010	Chile	A/Chile/115/2010	ggtgatgccccattccttgatcggctccgccgagatca aaagtccttaaaaggaagaggcaacaccc	-	*
CY092956	Human H1N1pdm2009 IAVs	Human	H1N1	pdm	2010	Chile	A/Chile/15/2010	ggtgatgccccattccttgatcggctccgccgagatca aaagtccttaaaaggaagaggcaacaccc	-	*
CY093012	Human H1N1pdm2009 IAVs	Human	H1N1		2010	Chile	A/Chile/54/2010	ggtgatgccccattccttgatcggctccgccgagatca aaagtccttaaaaggaagaggcaacaccc	-	*

CY093169	Human H1N1pdm2009 IAVs	Human	H1N1	pdm	2010	Chile	A/Chile/64/2010	ggtgatgccccattccttgatcggctccgccgagatca aaagtccttaaaaggaagaggcaacaccc	-	*
CY093020	Human H1N1pdm2009 IAVs	Human	H1N1	pdm	2010	Chile	A/Chile/52/2010	ggtgatgccccattccttgatcggctccgccgagatca aaagtccttaaaaggaagaggcaacaccc	-	*
CY093044	Human H1N1pdm2009 IAVs	Human	H1N1	pdm	2010	Chile	A/Chile/72/2010	ggtgatgccccattccttgatcggctccgccgagatca aaagtccttaaaaggaagaggcaacaccc	-	*
CY093185	Human H1N1pdm2009 IAVs	Human	H1N1	pdm	2010	Chile	A/Chile/94/2010	ggtgatgccccattccttgatcggctccgccgagatca aaagtccttaaaaggaagaggcaacaccc	-	*
CY062150	Human H1N1pdm2009 IAVs	Human	H1N1	pdm	2010	USA	A/New_York/2963/2010	ggtgatgccccattccttgatcggctccgccgagatca aaagtccttaaaaggaagaggcaacaccc	-	*
CY088834	Human H1N1pdm2009 IAVs	Human	H1N1	pdm	2010	Thailand	A/Thailand/CU_H2417/2010	ggtgatgccccattccttgatcggctccgccgagatca aaagtccttaaaaggaagaggcaacaccc	-	*
CY071139	Human H1N1pdm2009 IAVs	Human	H1N1	pdm	2010	Greece	A/Athens/INS389/2010	ggtgatgccccattccttgatcggctccgccgagatca aaagtccttaaaaggaagaggcaacaccc	-	*
CY086884	Human H1N1pdm2009 IAVs	Swine	H1N2		2010	USA	A/swine/Indiana/240218/2010	ggtgatgccccattccttgatcggctccgccgagatca aaagtccttaaaaggaagaggcaacaccc	-	*
CY100512	Human H1N1pdm2009 IAVs	Human	H1N1		2010	Mexico	A/Mexico_City/INER17/2010	ggtgatgccccattccttgatcggctccgccgagatca aaagtccttaaaaggaagaggcaacaccc	-	*
CY100518	Human H1N1pdm2009 IAVs	Human	H1N1	pdm	2010	Mexico	A/Mexico_City/INER18/2010	ggtgatgccccattccttgatcggctccgccgagatca aaagtccttaaaaggaagaggcaacaccc	-	*
KC780658	Human H1N1pdm2009 IAVs	Human	H1N1	pdm	2010	USA	A/South_Carolina/01/2010	ggtgatgccccattccttgatcggctccgccgagatca aaagtccttaaaaggaagaggcaacaccc	-	*
CY167424	Human H1N1pdm2009 IAVs	Human	H1N1	pdm	2010	USA	A/Tennessee/F1076/2010	ggtgatgccccattccttgatcggctccgccgagatca aaagtccttaaaaggaagaggcaacaccc	-	*
CY167432	Human H1N1pdm2009 IAVs	Human	H1N1		2010	USA	A/Tennessee/F1076A/2010	ggtgatgccccattccttgatcggctccgccgagatca aaagtccttaaaaggaagaggcaacaccc	-	*
CY167440	Human H1N1pdm2009 IAVs	Human	H1N1	pdm	2010	USA	A/Tennessee/F1078/2010	ggtgatgccccattccttgatcggctccgccgagatca aaagtccttaaaaggaagaggcaacaccc	-	*
CY167784	Human H1N1pdm2009 IAVs	Human	H1N1	pdm	2010	USA	A/Tennessee/F1080/2010	ggtgatgccccattccttgatcggctccgccgagatca aaagtccttaaaaggaagaggcaacaccc	-	*
CY167448	Human H1N1pdm2009 IAVs	Human	H1N1	pdm	2010	USA	A/Tennessee/F1083c80/2010	ggtgatgccccattccttgatcggctccgccgagatca aaagtccttaaaaggaagaggcaacaccc	-	*
CY167464	Human H1N1pdm2009 IAVs	Human	H1N1	pdm	2010	USA	A/Tennessee/F1093A/2010	ggtgatgccccattccttgatcggctccgccgagatca aaagtccttaaaaggaagaggcaacaccc	-	*
CY061167	Human H1N1pdm2009 IAVs	Human	H1N1	pdm	2010	USA	A/Texas/JMS410/2010	ggtgatgccccattccttgatcggctccgccgagatca aaagtccttaaaaggaagaggcaacaccc	-	*
KC859082	Human H1N1pdm2009 IAVs	Swine	H1N1		2010	Thailand	A/swine/Thailand/CB144/2010	ggtgatgccccattccttgatcggctccgccgagatca aaagtccttaaaaggaagaggcaacaccc	-	*
CY090841	Human H1N1pdm2009 IAVs	Human	H1N1	pdm	2010	Nicaragua	A/Managua/0305_10/2010	ggtgatgccccattccttgatcggctccgccgagatca aaagtccttaaaaggaagaggcaacaccc	-	*
JX309140	Human H1N1pdm2009 IAVs	Human	H1N1	pdm	2010	Singapore	A/Singapore/GP521/2010	ggtgatgccccattccttgatcggctccgccgagatca aaagtccttaaaaggaagaggcaacaccc	-	*
JX309249	Human H1N1pdm2009 IAVs	Human	H1N1	pdm	2010	Singapore	A/Singapore/TT123/2010	ggtgatgccccattccttgatcggctccgccgagatca aaagtccttaaaaggaagaggcaacaccc	-	*
JX309340	Human H1N1pdm2009 IAVs	Human	H1N1	pdm	2010	Singapore	A/Singapore/TT144/2010	ggtgatgccccattccttgatcggctccgccgagatca aaagtccttaaaaggaagaggcaacaccc	-	*
JX309238	Human H1N1pdm2009 IAVs	Human	H1N1	pdm	2010	Singapore	A/Singapore/KK100/2010	ggtgatgccccattccttgatcggctccgccgagatca aaagtccttaaaaggaagaggcaacaccc	-	*
JX309069	Human H1N1pdm2009 IAVs	Human	H1N1	pdm	2010	Singapore	A/Singapore/GP489/2010	ggtgatgccccattccttgatcggctccgccgagatca aaagtccttaaaaggaagaggcaacaccc	-	*

KC859074	Human H1N1pdm2009 IAVs	Swine	H1N1		2010	Thailand	A/swine/Thailand/CB143/2010	ggtgatgccccattccttgatcggctccgccgagatca aaagtccttaaaaggaagaggcaacaccc	-	*
KC780417	Human H1N1pdm2009 IAVs	Human	H1N1	pdm	2010	USA	A/Florida/01/2010	ggtgatgccccattccttgatcggctccgccgagatca aaagtccttaaaaggaagaggcaacaccc	-	*
KC782011	Human H1N1pdm2009 IAVs	Human	H1N1	pdm	2010	USA	A/Iowa/01/2010	ggtgatgccccattccttgatcggctccgccgagatca aaagtccttaaaaggaagaggcaacaccc	-	*
JQ689112	Human H1N1pdm2009 IAVs	Swine	H3N2		2010	USA	A/swine/Missouri/A01047968/2010	ggtgatgccccattccttgatcggctccgccgagatca aaagtccttaaaaggaagaggcaacaccc	-	*
JQ689120	Human H1N1pdm2009 IAVs	Swine	H3N2		2010	USA	A/swine/Missouri/A01047969/2010	ggtgatgccccattccttgatcggctccgccgagatca aaagtccttaaaaggaagaggcaacaccc	-	*
JN043447	Human H1N1pdm2009 IAVs	Swine	H1N2		2010	South_Korea	A/swine/Korea/VDS1/2010	ggtgatgccccattccttgatcggctccgccgagatca aaagtccttaaaaggaagaggcaacaccc	-	*
KM029635	Human H1N1pdm2009 IAVs	Swine	H1N1		2010	China	A/swine/Hong_Kong/NS3141/2010	ggtgatgccccattccttgatcggctccgccgagatca aaagtccttaaaaggaagaggcaacaccc	-	*
KM029643	Human H1N1pdm2009 IAVs	Swine	H1N1		2010	China	A/swine/Hong_Kong/NS3160/2010	ggtgatgccccattccttgatcggctccgccgagatca aaagtccttaaaaggaagaggcaacaccc	-	*
KM029651	Human H1N1pdm2009 IAVs	Swine	H1N1		2010	China	A/swine/Hong_Kong/NS3226/2010	ggtgatgccccattccttgatcggctccgccgagatca aaagtccttaaaaggaagaggcaacaccc	-	*
CY071171	Human H1N1pdm2009 IAVs	Human	H1N1	pdm	2010	Greece	A/Athens/INS396/2010	ggtgatgccccattccttgatcggctccgccgagatca aaagtccttaaaaggaagaggcaacaccc	-	*
CY129698	Human H1N1pdm2009 IAVs	Human	H1N1	pdm	2010	Greece	A/Athens/INS555/2010	ggtgatgccccattccttgatcggctccgccgagatca aaagtccttaaaaggaagaggcaacaccc	-	*
CY075071	Human H1N1pdm2009 IAVs	Human	H1N1	pdm	2010	Greece	A/Athens/INS393/2010	ggtgatgccccattccttgatcggctccgccgagatca aaagtccttaaaaggaagaggcaacaccc	-	*
CY096214	Human H1N1pdm2009 IAVs	Human	H1N1	pdm	2010	Australia	A/North_Fitzroy/INS465/2010	ggtgatgccccattccttgatcggctccgccgagatca aaagtccttaaaaggaagaggcaacaccc	-	*
CY092546	Human H1N1pdm2009 IAVs	Human	H1N1	pdm	2010	Australia	A/Sydney/DD3_16/2010	ggtgatgccccattccttgatcggctccgccgagatca aaagtccttaaaaggaagaggcaacaccc	-	*
CY093113	Human H1N1pdm2009 IAVs	Human	H1N1	pdm	2010	Australia	A/Sydney/DD3_34/2010	ggtgatgccccattccttgatcggctccgccgagatca aaagtccttaaaaggaagaggcaacaccc	-	*
CY092658	Human H1N1pdm2009 IAVs	Human	H1N1	pdm	2010	Australia	A/Sydney/DD3_39/2010	ggtgatgccccattccttgatcggctccgccgagatca aaagtccttaaaaggaagaggcaacaccc	-	*
CY092690	Human H1N1pdm2009 IAVs	Human	H1N1	pdm	2010	Australia	A/Sydney/DD3_45/2010	ggtgatgccccattccttgatcggctccgccgagatca aaagtccttaaaaggaagaggcaacaccc	-	*
CY092730	Human H1N1pdm2009 IAVs	Human	H1N1	pdm	2010	Australia	A/Sydney/DD3_53/2010	ggtgatgccccattccttgatcggctccgccgagatca aaagtccttaaaaggaagaggcaacaccc	-	*
JX309570	Human H1N1pdm2009 IAVs	Human	H1N1	pdm	2010	Singapore	A/Singapore/TT350/2010	ggtgatgccccattccttgatcggctccgccgagatca aaagtccttaaaaggaagaggcaacaccc	-	*
CY129858	Human H1N1pdm2009 IAVs	Human	H1N1	pdm	2010	Australia	A/Cairns_North/INS597/2010	ggtgatgccccattccttgatcggctccgccgagatca aaagtccttaaaaggaagaggcaacaccc	-	*
CY093193	Human H1N1pdm2009 IAVs	Human	H1N1	pdm	2010	Chile	A/Chile/106/2010	ggtgatgccccattccttgatcggctccgccgagatca aaagtccttaaaaggaagaggcaacaccc	-	*
CY092972	Human H1N1pdm2009 IAVs	Human	H1N1		2010	Chile	A/Chile/23/2010	ggtgatgccccattccttgatcggctccgccgagatca aaagtccttaaaaggaagaggcaacaccc	-	*
CY092980	Human H1N1pdm2009 IAVs	Human	H1N1	pdm	2010	Chile	A/Chile/28/2010	ggtgatgccccattccttgatcggctccgccgagatca aaagtccttaaaaggaagaggcaacaccc	-	*
CY092988	Human H1N1pdm2009 IAVs	Human	H1N1	pdm	2010	Chile	A/Chile/29/2010	ggtgatgccccattccttgatcggctccgccgagatca aaagtccttaaaaggaagaggcaacaccc	-	*
CY092996	Human H1N1pdm2009 IAVs	Human	H1N1	pdm	2010	Chile	A/Chile/34/2010	ggtgatgccccattccttgatcggctccgccgagatca aaagtccttaaaaggaagaggcaacaccc	-	*

CY093004	Human H1N1pdm2009 IAVs	Human	H1N1	pdm	2010	Chile	A/Chile/37/2010	ggtgatgccccattccttgatcggctccgccgagatca aaagtccttaaaaggaagaggcaacaccc	-	*
CY093028	Human H1N1pdm2009 IAVs	Human	H1N1	pdm	2010	Chile	A/Chile/58/2010	ggtgatgccccattccttgatcggctccgccgagatca aaagtccttaaaaggaagaggcaacaccc	-	*
CY093161	Human H1N1pdm2009 IAVs	Human	H1N1	pdm	2010	Chile	A/Chile/61/2010	ggtgatgccccattccttgatcggctccgccgagatca aaagtccttaaaaggaagaggcaacaccc	-	*
CY098174	Human H1N1pdm2009 IAVs	Human	H1N1	pdm	2010	Chile	A/Chile/70/2010	ggtgatgccccattccttgatcggctccgccgagatca aaagtccttaaaaggaagaggcaacaccc	-	*
CY093052	Human H1N1pdm2009 IAVs	Human	H1N1		2010	Chile	A/Chile/78/2010	ggtgatgccccattccttgatcggctccgccgagatca aaagtccttaaaaggaagaggcaacaccc	-	*
CY093177	Human H1N1pdm2009 IAVs	Human	H1N1	pdm	2010	Chile	A/Chile/87/2010	ggtgatgccccattccttgatcggctccgccgagatca aaagtccttaaaaggaagaggcaacaccc	-	*
CY096222	Human H1N1pdm2009 IAVs	Human	H1N1		2010	Australia	A/Melbourne/INS467/2010	ggtgatgccccattccttgatcggctccgccgagatca aaagtccttaaaaggaagaggcaacaccc	-	*
CY062126	Human H1N1pdm2009 IAVs	Human	H1N1	pdm	2010	USA	A/New_York/2648/2010	ggtgatgccccattccttgatcggctccgccgagatca aaagtccttaaaaggaagaggcaacaccc	-	*
CY096238	Human H1N1pdm2009 IAVs	Human	H1N1	pdm	2010	Australia	A/North_Fitzroy/INS469/2010	ggtgatgccccattccttgatcggctccgccgagatca aaagtccttaaaaggaagaggcaacaccc	-	*
CY096262	Human H1N1pdm2009 IAVs	Human	H1N1		2010	Australia	A/North_Fitzroy/INS473/2010	ggtgatgccccattccttgatcggctccgccgagatca aaagtccttaaaaggaagaggcaacaccc	-	*
CY096581	Human H1N1pdm2009 IAVs	Human	H1N1	pdm	2010	Australia	A/North_Fitzroy/INS529/2010	ggtgatgccccattccttgatcggctccgccgagatca aaagtccttaaaaggaagaggcaacaccc	-	*
CY092434	Human H1N1pdm2009 IAVs	Human	H1N1	pdm	2010	Australia	A/Sydney/DD3_01/2010	ggtgatgccccattccttgatcggctccgccgagatca aaagtccttaaaaggaagaggcaacaccc	-	*
CY092498	Human H1N1pdm2009 IAVs	Human	H1N1	pdm	2010	Australia	A/Sydney/DD3_09/2010	ggtgatgccccattccttgatcggctccgccgagatca aaagtccttaaaaggaagaggcaacaccc	-	*
CY093145	Human H1N1pdm2009 IAVs	Human	H1N1	pdm	2010	Australia	A/Sydney/DD3_20/2010	ggtgatgccccattccttgatcggctccgccgagatca aaagtccttaaaaggaagaggcaacaccc	-	*
CY092578	Human H1N1pdm2009 IAVs	Human	H1N1	pdm	2010	Australia	A/Sydney/DD3_22/2010	ggtgatgccccattccttgatcggctccgccgagatca aaagtccttaaaaggaagaggcaacaccc	-	*
CY092828	Human H1N1pdm2009 IAVs	Human	H1N1	pdm	2010	Australia	A/Sydney/DD3_40/2010	ggtgatgccccattccttgatcggctccgccgagatca aaagtccttaaaaggaagaggcaacaccc	-	*
CY092698	Human H1N1pdm2009 IAVs	Human	H1N1	pdm	2010	Australia	A/Sydney/DD3_47/2010	ggtgatgccccattccttgatcggctccgccgagatca aaagtccttaaaaggaagaggcaacaccc	-	*
CY092836	Human H1N1pdm2009 IAVs	Human	H1N1	pdm	2010	Australia	A/Sydney/DD3_49/2010	ggtgatgccccattccttgatcggctccgccgagatca aaagtccttaaaaggaagaggcaacaccc	-	*
JN187343	Human H1N1pdm2009 IAVs	Human	H1N1	pdm	2010	Taiwan	A/Taiwan/5270/2010	ggtgatgccccattccttgatcggctccgccgagatca aaagtccttaaaaggaagaggcaacaccc	-	*
AB704482	Human H1N1pdm2009 IAVs	Human	H1N1		2010	Japan	A/Tochigi/2/2010	ggtgatgccccattccttgatcggctccgccgagatca aaagtccttaaaaggaagaggcaacaccc	-	*
JX625650	Human H1N1pdm2009 IAVs	Human	H1N1	pdm	2010	United_Kingdom	A/England/03380426/2010	ggtgatgccccattccttgatcggctccgccgagatca aaagtccttaaaaggaagaggcaacaccc	-	*
JX309442	Human H1N1pdm2009 IAVs	Human	H1N1		2010	Singapore	A/Singapore/CC02/2010	ggtgatgccccattccttgatcggctccgccgagatca aaagtccttaaaaggaagaggcaacaccc	-	*
JX309566	Human H1N1pdm2009 IAVs	Human	H1N1	pdm	2010	Singapore	A/Singapore/EN107/2010	ggtgatgccccattccttgatcggctccgccgagatca aaagtccttaaaaggaagaggcaacaccc	-	*
JX309227	Human H1N1pdm2009 IAVs	Human	H1N1	pdm	2010	Singapore	A/Singapore/GP1035/2010	ggtgatgccccattccttgatcggctccgccgagatca aaagtccttaaaaggaagaggcaacaccc	-	*
JX308977	Human H1N1pdm2009 IAVs	Human	H1N1	pdm	2010	Singapore	A/Singapore/GP132/2010	ggtgatgccccattccttgatcggctccgccgagatca aaagtccttaaaaggaagaggcaacaccc	-	*

JX308981	Human H1N1pdm2009 IAVs	Human	H1N1	pdm	2010	Singapore	A/Singapore/GP155/2010	ggtgatgccccattccttgatcggctccgccgagatca<br>aaagtccttaaaaggaagaggcaacaccc	-	*
JX308985	Human H1N1pdm2009 IAVs	Human	H1N1	pdm	2010	Singapore	A/Singapore/GP174/2010	ggtgatgccccattccttgatcggctccgccgagatca<br>aaagtccttaaaaggaagaggcaacaccc	-	*
JX309399	Human H1N1pdm2009 IAVs	Human	H1N1	pdm	2010	Singapore	A/Singapore/GP185/2010	ggtgatgccccattccttgatcggctccgccgagatca<br>aaagtccttaaaaggaagaggcaacaccc	-	*
JX309109	Human H1N1pdm2009 IAVs	Human	H1N1	pdm	2010	Singapore	A/Singapore/GP205/2010	ggtgatgccccattccttgatcggctccgccgagatca<br>aaagtccttaaaaggaagaggcaacaccc	-	*
JX309005	Human H1N1pdm2009 IAVs	Human	H1N1	pdm	2010	Singapore	A/Singapore/GP220/2010	ggtgatgccccattccttgatcggctccgccgagatca<br>aaagtccttaaaaggaagaggcaacaccc	-	*
JX309013	Human H1N1pdm2009 IAVs	Human	H1N1	pdm	2010	Singapore	A/Singapore/GP241/2010	ggtgatgccccattccttgatcggctccgccgagatca<br>aaagtccttaaaaggaagaggcaacaccc	-	*
JX309113	Human H1N1pdm2009 IAVs	Human	H1N1	pdm	2010	Singapore	A/Singapore/GP266/2010	ggtgatgccccattccttgatcggctccgccgagatca<br>aaagtccttaaaaggaagaggcaacaccc	-	*
JX309117	Human H1N1pdm2009 IAVs	Human	H1N1	pdm	2010	Singapore	A/Singapore/GP293/2010	ggtgatgccccattccttgatcggctccgccgagatca<br>aaagtccttaaaaggaagaggcaacaccc	-	*
JX308997	Human H1N1pdm2009 IAVs	Human	H1N1	pdm	2010	Singapore	A/Singapore/GP329/2010	ggtgatgccccattccttgatcggctccgccgagatca<br>aaagtccttaaaaggaagaggcaacaccc	-	*
CY063834	Human H1N1pdm2009 IAVs	Human	H1N1	pdm	2010	Singapore	A/Singapore/GP562/2010	ggtgatgccccattccttgatcggctccgccgagatca<br>aaagtccttaaaaggaagaggcaacaccc	-	*
JX309160	Human H1N1pdm2009 IAVs	Human	H1N1	pdm	2010	Singapore	A/Singapore/GP567/2010	ggtgatgccccattccttgatcggctccgccgagatca<br>aaagtccttaaaaggaagaggcaacaccc	-	*
JX308969	Human H1N1pdm2009 IAVs	Human	H1N1	pdm	2010	Singapore	A/Singapore/GP64/2010	ggtgatgccccattccttgatcggctccgccgagatca<br>aaagtccttaaaaggaagaggcaacaccc	-	*
JX309152	Human H1N1pdm2009 IAVs	Human	H1N1		2010	Singapore	A/Singapore/GP737/2010	ggtgatgccccattccttgatcggctccgccgagatca<br>aaagtccttaaaaggaagaggcaacaccc	-	*
JX309125	Human H1N1pdm2009 IAVs	Human	H1N1	pdm	2010	Singapore	A/Singapore/KK04/2010	ggtgatgccccattccttgatcggctccgccgagatca<br>aaagtccttaaaaggaagaggcaacaccc	-	*
CY063852	Human H1N1pdm2009 IAVs	Human	H1N1		2010	Singapore	A/Singapore/KK066/2010	ggtgatgccccattccttgatcggctccgccgagatca<br>aaagtccttaaaaggaagaggcaacaccc	-	*
JX309129	Human H1N1pdm2009 IAVs	Human	H1N1	pdm	2010	Singapore	A/Singapore/KK12/2010	ggtgatgccccattccttgatcggctccgccgagatca<br>aaagtccttaaaaggaagaggcaacaccc	-	*
JX309614	Human H1N1pdm2009 IAVs	Human	H1N1	pdm	2010	Singapore	A/Singapore/KK420/2010	ggtgatgccccattccttgatcggctccgccgagatca<br>aaagtccttaaaaggaagaggcaacaccc	-	*
JX309146	Human H1N1pdm2009 IAVs	Human	H1N1	pdm	2010	Singapore	A/Singapore/KK81/2010	ggtgatgccccattccttgatcggctccgccgagatca<br>aaagtccttaaaaggaagaggcaacaccc	-	*
JX309722	Human H1N1pdm2009 IAVs	Human	H1N1		2010	Singapore	A/Singapore/SS40/2010	ggtgatgccccattccttgatcggctccgccgagatca<br>aaagtccttaaaaggaagaggcaacaccc	-	*
JX309085	Human H1N1pdm2009 IAVs	Human	H1N1	pdm	2010	Singapore	A/Singapore/TT10/2010	ggtgatgccccattccttgatcggctccgccgagatca<br>aaagtccttaaaaggaagaggcaacaccc	-	*
JX309171	Human H1N1pdm2009 IAVs	Human	H1N1	pdm	2010	Singapore	A/Singapore/TT106/2010	ggtgatgccccattccttgatcggctccgccgagatca<br>aaagtccttaaaaggaagaggcaacaccc	-	*
JX309480	Human H1N1pdm2009 IAVs	Human	H1N1	pdm	2010	Singapore	A/Singapore/TT255/2010	ggtgatgccccattccttgatcggctccgccgagatca<br>aaagtccttaaaaggaagaggcaacaccc	-	*
JX309484	Human H1N1pdm2009 IAVs	Human	H1N1	pdm	2010	Singapore	A/Singapore/TT289/2010	ggtgatgccccattccttgatcggctccgccgagatca<br>aaagtccttaaaaggaagaggcaacaccc	-	*
JX309578	Human H1N1pdm2009 IAVs	Human	H1N1	pdm	2010	Singapore	A/Singapore/TT335/2010	ggtgatgccccattccttgatcggctccgccgagatca<br>aaagtccttaaaaggaagaggcaacaccc	-	*
JX309574	Human H1N1pdm2009 IAVs	Human	H1N1	pdm	2010	Singapore	A/Singapore/TT393/2010	ggtgatgccccattccttgatcggctccgccgagatca<br>aaagtccttaaaaggaagaggcaacaccc	-	*

JX309702	Human H1N1pdm2009 IAVs	Human	H1N1		2010	Singapore	A/Singapore/TT418/2010	ggtgatgccccattccttgatcggctccgccgagatca aaagtccttaaaaggaagaggcaacaccc	-	*
JN375258	Human H1N1pdm2009 IAVs	Swine	H1N1		2010	China	A/swine/Guangdong/1361/2010	ggtgatgccccattccttgatcggctccgccgagatca aaagtccttaaaaggaagaggcaacaccc	-	*
JN375259	Human H1N1pdm2009 IAVs	Swine	H1N1		2010	China	A/swine/Guangdong/1367/2010	ggtgatgccccattccttgatcggctccgccgagatca aaagtccttaaaaggaagaggcaacaccc	-	*
JN375260	Human H1N1pdm2009 IAVs	Swine	H1N1		2010	China	A/swine/Guangdong/1397/2010	ggtgatgccccattccttgatcggctccgccgagatca aaagtccttaaaaggaagaggcaacaccc	-	*
JN375261	Human H1N1pdm2009 IAVs	Swine	H1N1		2010	China	A/swine/Guangdong/1408/2010	ggtgatgccccattccttgatcggctccgccgagatca aaagtccttaaaaggaagaggcaacaccc	-	*
JN375262	Human H1N1pdm2009 IAVs	Swine	H1N1		2010	China	A/swine/Guangdong/1409/2010	ggtgatgccccattccttgatcggctccgccgagatca aaagtccttaaaaggaagaggcaacaccc	-	*
JN375263	Human H1N1pdm2009 IAVs	Swine	H1N1		2010	China	A/swine/Guangdong/1425/2010	ggtgatgccccattccttgatcggctccgccgagatca aaagtccttaaaaggaagaggcaacaccc	-	*
JN375264	Human H1N1pdm2009 IAVs	Swine	H1N1		2010	China	A/swine/Guangdong/1430/2010	ggtgatgccccattccttgatcggctccgccgagatca aaagtccttaaaaggaagaggcaacaccc	-	*
JN375265	Human H1N1pdm2009 IAVs	Swine	H1N1		2010	China	A/swine/Guangdong/1434/2010	ggtgatgccccattccttgatcggctccgccgagatca aaagtccttaaaaggaagaggcaacaccc	-	*
JN375266	Human H1N1pdm2009 IAVs	Swine	H1N1		2010	China	A/swine/Guangdong/1436/2010	ggtgatgccccattccttgatcggctccgccgagatca aaagtccttaaaaggaagaggcaacaccc	-	*
JX309500	Human H1N1pdm2009 IAVs	Human	H1N1	pdm	2010	Singapore	A/Singapore/GP2257/2010	ggtgatgccccattccttgatcggctccgccgagatca aaagtccttaaaaggaagaggcaacaccc	-	*
JX309662	Human H1N1pdm2009 IAVs	Human	H1N1		2010	Singapore	A/Singapore/GP3610/2010	ggtgatgccccattccttgatcggctccgccgagatca aaagtccttaaaaggaagaggcaacaccc	-	*
JX309726	Human H1N1pdm2009 IAVs	Human	H1N1	pdm	2010	Singapore	A/Singapore/SS41/2010	ggtgatgccccattccttgatcggctccgccgagatca aaagtccttaaaaggaagaggcaacaccc	-	*
CY092650	Human H1N1pdm2009 IAVs	Human	H1N1	pdm	2010	Australia	A/Sydney/DD3_38/2010	ggtgatgccccattccttgatcggctccgccgagatca aaagtccttaaaaggaagaggcaacaccc	-	*
CY063844	Human H1N1pdm2009 IAVs	Human	H1N1	pdm	2010	Singapore	A/Singapore/GP999/2010	ggtgatgccccattccttgatcggctccgccgagatca aaagtccttaaaaggaagaggcaacaccc	-	*
CY080335	Human H1N1pdm2009 IAVs	Human	H1N1	pdm	2010	Thailand	A/Thailand/H1818/2010	ggtgatgccccattccttgatcggctccgccgagatca aaagtccttaaaaggaagaggcaacaccc	-	*
CY093201	Human H1N1pdm2009 IAVs	Human	H1N1	pdm	2010	Chile	A/Chile/109/2010	ggtgatgccccattccttgatcggctccgccgagatca aaagtccttaaaaggaagaggcaacaccc	-	*
CY080327	Human H1N1pdm2009 IAVs	Human	H1N1	pdm	2010	Thailand	A/Thailand/CU_H1786/2010	ggtgatgccccattccttgatcggctccgccgagatca aaagtccttaaaaggaagaggcaacaccc	-	*
JX309682	Human H1N1pdm2009 IAVs	Human	H1N1		2010	Singapore	A/Singapore/KK467/2010	ggtgatgccccattccttgatcggctccgccgagatca aaagtccttaaaaggaagaggcaacaccc	-	*
JX309686	Human H1N1pdm2009 IAVs	Human	H1N1	pdm	2010	Singapore	A/Singapore/KK495/2010	ggtgatgccccattccttgatcggctccgccgagatca aaagtccttaaaaggaagaggcaacaccc	-	*
JX309045	Human H1N1pdm2009 IAVs	Human	H1N1	pdm	2010	Singapore	A/Singapore/GP398/2010	ggtgatgccccattccttgatcggctccgccgagatca aaagtccttaaaaggaagaggcaacaccc	-	*
JX309492	Human H1N1pdm2009 IAVs	Human	H1N1		2010	Singapore	A/Singapore/KK315/2010	ggtgatgccccattccttgatcggctccgccgagatca aaagtccttaaaaggaagaggcaacaccc	-	*
JX309626	Human H1N1pdm2009 IAVs	Human	H1N1	pdm	2010	Singapore	A/Singapore/GP3395/2010	ggtgatgccccattccttgatcggctccgccgagatca aaagtccttaaaaggaagaggcaacaccc	-	*
JX309634	Human H1N1pdm2009 IAVs	Human	H1N1	pdm	2010	Singapore	A/Singapore/GP3442/2010	ggtgatgccccattccttgatcggctccgccgagatca aaagtccttaaaaggaagaggcaacaccc	-	*
JX309718	Human H1N1pdm2009 IAVs	Human	H1N1	pdm	2010	Singapore	A/Singapore/SS39/2010	ggtgatgccccattccttgatcggctccgccgagatca aaagtccttaaaaggaagaggcaacaccc	-	*

JX309730	Human H1N1pdm2009 IAVs	Human	H1N1	pdm	2010	Singapore	A/Singapore/TT405/2010	ggtgatgccccattccttgatcggctccgccgagatca aaagtccttaaaaggaagaggcaacaccc	-	*
JX309714	Human H1N1pdm2009 IAVs	Human	H1N1	pdm	2010	Singapore	A/Singapore/SS38/2010	ggtgatgccccattccttgatcggctccgccgagatca aaagtccttaaaaggaagaggcaacaccc	-	*
CY098118	Human H1N1pdm2009 IAVs	Human	H1N1	pdm	2010	Thailand	A/Bangkok/INS486/2010	ggtgatgccccattccttgatcggctccgccgagatca aaagtccttaaaaggaagaggcaacaccc	-	*
JX309734	Human H1N1pdm2009 IAVs	Human	H1N1	pdm	2010	Singapore	A/Singapore/TT414/2010	ggtgatgccccattccttgatcggctccgccgagatca aaagtccttaaaaggaagaggcaacaccc	-	*
CY096206	Human H1N1pdm2009 IAVs	Human	H1N1	pdm	2010	Australia	A/North_Fitzroy/INS464/2010	ggtgatgccccattccttgatcggctccgccgagatca aaagtccttaaaaggaagaggcaacaccc	-	*
CY092490	Human H1N1pdm2009 IAVs	Human	H1N1	pdm	2010	Australia	A/Sydney/DD3_08/2010	ggtgatgccccattccttgatcggctccgccgagatca aaagtccttaaaaggaagaggcaacaccc	-	*
JX309413	Human H1N1pdm2009 IAVs	Human	H1N1	pdm	2010	Singapore	A/Singapore/SS13/2010	ggtgatgccccattccttgatcggctccgccgagatca aaagtccttaaaaggaagaggcaacaccc	-	*
CY092450	Human H1N1pdm2009 IAVs	Human	H1N1	pdm	2010	Australia	A/Sydney/DD3_03/2010	ggtgatgccccattccttgatcggctccgccgagatca aaagtccttaaaaggaagaggcaacaccc	-	*
CY092389	Human H1N1pdm2009 IAVs	Human	H1N1	pdm	2010	Australia	A/Sydney/DD3_29/2010	ggtgatgccccattccttgatcggctccgccgagatca aaagtccttaaaaggaagaggcaacaccc	-	*
CY092674	Human H1N1pdm2009 IAVs	Human	H1N1	pdm	2010	Australia	A/Sydney/DD3_42/2010	ggtgatgccccattccttgatcggctccgccgagatca aaagtccttaaaaggaagaggcaacaccc	-	*
JX309105	Human H1N1pdm2009 IAVs	Human	H1N1	pdm	2010	Singapore	A/Singapore/GP189/2010	ggtgatgccccattccttgatcggctccgccgagatca aaagtccttaaaaggaagaggcaacaccc	-	*
CY092530	Human H1N1pdm2009 IAVs	Human	H1N1	pdm	2010	Australia	A/Sydney/DD3_14/2010	ggtgatgccccattccttgatcggctccgccgagatca aaagtccttaaaaggaagaggcaacaccc	-	*
CY092570	Human H1N1pdm2009 IAVs	Human	H1N1	pdm	2010	Australia	A/Sydney/DD3_19/2010	ggtgatgccccattccttgatcggctccgccgagatca aaagtccttaaaaggaagaggcaacaccc	-	*
CY092820	Human H1N1pdm2009 IAVs	Human	H1N1	pdm	2010	Australia	A/Sydney/DD3_24/2010	ggtgatgccccattccttgatcggctccgccgagatca aaagtccttaaaaggaagaggcaacaccc	-	*
CY093036	Human H1N1pdm2009 IAVs	Human	H1N1	pdm	2010	Chile	A/Chile/71/2010	ggtgatgccccattccttgatcggctccgccgagatca aaagtccttaaaaggaagaggcaacaccc	-	*
CY096254	Human H1N1pdm2009 IAVs	Human	H1N1	pdm	2010	Australia	A/Melbourne/INS472/2010	ggtgatgccccattccttgatcggctccgccgagatca aaagtccttaaaaggaagaggcaacaccc	-	*
CY096230	Human H1N1pdm2009 IAVs	Human	H1N1	pdm	2010	Australia	A/Melbourne/INS468/2010	ggtgatgccccattccttgatcggctccgccgagatca aaagtccttaaaaggaagaggcaacaccc	-	*
CY096278	Human H1N1pdm2009 IAVs	Human	H1N1	pdm	2010	Australia	A/Melbourne/INS475/2010	ggtgatgccccattccttgatcggctccgccgagatca aaagtccttaaaaggaagaggcaacaccc	-	*
CY098549	Human H1N1pdm2009 IAVs	Human	H1N1	pdm	2010	Australia	A/North_Fitzroy/INS463/2010	ggtgatgccccattccttgatcggctccgccgagatca aaagtccttaaaaggaagaggcaacaccc	-	*
CY092706	Human H1N1pdm2009 IAVs	Human	H1N1	pdm	2010	Australia	A/Sydney/DD3_50/2010	ggtgatgccccattccttgatcggctccgccgagatca aaagtccttaaaaggaagaggcaacaccc	-	*
CY096422	Human H1N1pdm2009 IAVs	Human	H1N1		2010	Thailand	A/Bangkok/INS499/2010	ggtgatgccccattccttgatcggctccgccgagatca aaagtccttaaaaggaagaggcaacaccc	-	*
CY092618	Human H1N1pdm2009 IAVs	Human	H1N1	pdm	2010	Australia	A/Sydney/DD3_31/2010	ggtgatgccccattccttgatcggctccgccgagatca aaagtccttaaaaggaagaggcaacaccc	-	*
CY092666	Human H1N1pdm2009 IAVs	Human	H1N1	pdm	2010	Australia	A/Sydney/DD3_41/2010	ggtgatgccccattccttgatcggctccgccgagatca aaagtccttaaaaggaagaggcaacaccc	-	*
JF327347	Human H1N1pdm2009 IAVs	Human	H1N1	pdm	2010	Finland	A/Finland/4/2010	ggtgatgccccattccttgatcggctccgccgagatca aaagtccttaaaaggaagaggcaacaccc	-	*
CY092506	Human H1N1pdm2009 IAVs	Human	H1N1	pdm	2010	Australia	A/Sydney/DD3_10/2010	ggtgatgccccattccttgatcggctccgccgagatca aaagtccttaaaaggaagaggcaacaccc	-	*

JX309546	Human H1N1pdm2009 IAVs	Human	H1N1	pdm	2010	Singapore	A/Singapore/EN103/2010	ggtgatgccccattccttgatcggctccgccgagatcaaaagtccttaaaaggaagaggcaacaccc	-	*
JX309456	Human H1N1pdm2009 IAVs	Human	H1N1	pdm	2010	Singapore	A/Singapore/GP2362/2010	ggtgatgccccattccttgatcggctccgccgagatcaaaagtccttaaaaggaagaggcaacaccc	-	*
JX309646	Human H1N1pdm2009 IAVs	Human	H1N1	pdm	2010	Singapore	A/Singapore/GP3532/2010	ggtgatgccccattccttgatcggctccgccgagatcaaaagtccttaaaaggaagaggcaacaccc	-	*
JX309764	Human H1N1pdm2009 IAVs	Human	H1N1	pdm	2010	Singapore	A/Singapore/GP3828/2010	ggtgatgccccattccttgatcggctccgccgagatcaaaagtccttaaaaggaagaggcaacaccc	-	*
JX309598	Human H1N1pdm2009 IAVs	Human	H1N1	pdm	2010	Singapore	A/Singapore/KK439/2010	ggtgatgccccattccttgatcggctccgccgagatcaaaagtccttaaaaggaagaggcaacaccc	-	*
JX309740	Human H1N1pdm2009 IAVs	Human	H1N1	pdm	2010	Singapore	A/Singapore/TT437/2010	ggtgatgccccattccttgatcggctccgccgagatcaaaagtccttaaaaggaagaggcaacaccc	-	*
JX309690	Human H1N1pdm2009 IAVs	Human	H1N1	pdm	2010	Singapore	A/Singapore/KK499/2010	ggtgatgccccattccttgatcggctccgccgagatcaaaagtccttaaaaggaagaggcaacaccc	-	*
CY092458	Human H1N1pdm2009 IAVs	Human	H1N1	pdm	2010	Australia	A/Sydney/DD3_04/2010	ggtgatgccccattccttgatcggctccgccgagatcaaaagtccttaaaaggaagaggcaacaccc	-	*
CY092474	Human H1N1pdm2009 IAVs	Human	H1N1	pdm	2010	Australia	A/Sydney/DD3_06/2010	ggtgatgccccattccttgatcggctccgccgagatcaaaagtccttaaaaggaagaggcaacaccc	-	*
CY092482	Human H1N1pdm2009 IAVs	Human	H1N1		2010	Australia	A/Sydney/DD3_07/2010	ggtgatgccccattccttgatcggctccgccgagatcaaaagtccttaaaaggaagaggcaacaccc	-	*
CY092514	Human H1N1pdm2009 IAVs	Human	H1N1	pdm	2010	Australia	A/Sydney/DD3_12/2010	ggtgatgccccattccttgatcggctccgccgagatcaaaagtccttaaaaggaagaggcaacaccc	-	*
CY092522	Human H1N1pdm2009 IAVs	Human	H1N1		2010	Australia	A/Sydney/DD3_13/2010	ggtgatgccccattccttgatcggctccgccgagatcaaaagtccttaaaaggaagaggcaacaccc	-	*
CY092610	Human H1N1pdm2009 IAVs	Human	H1N1	pdm	2010	Australia	A/Sydney/DD3_30/2010	ggtgatgccccattccttgatcggctccgccgagatcaaaagtccttaaaaggaagaggcaacaccc	-	*
CY092738	Human H1N1pdm2009 IAVs	Human	H1N1	pdm	2010	Australia	A/Sydney/DD3_54/2010	ggtgatgccccattccttgatcggctccgccgagatcaaaagtccttaaaaggaagaggcaacaccc	-	*
CY092746	Human H1N1pdm2009 IAVs	Human	H1N1	pdm	2010	Australia	A/Sydney/DD3_56/2010	ggtgatgccccattccttgatcggctccgccgagatcaaaagtccttaaaaggaagaggcaacaccc	-	*
CY092466	Human H1N1pdm2009 IAVs	Human	H1N1	pdm	2010	Australia	A/Sydney/DD3_05/2010	ggtgatgccccattccttgatcggctccgccgagatcaaaagtccttaaaaggaagaggcaacaccc	-	*
CY092562	Human H1N1pdm2009 IAVs	Human	H1N1		2010	Australia	A/Sydney/DD3_18/2010	ggtgatgccccattccttgatcggctccgccgagatcaaaagtccttaaaaggaagaggcaacaccc	-	*
CY096573	Human H1N1pdm2009 IAVs	Human	H1N1	pdm	2010	Australia	A/Westmead/INS524/2010	ggtgatgccccattccttgatcggctccgccgagatcaaaagtccttaaaaggaagaggcaacaccc	-	*
JX309654	Human H1N1pdm2009 IAVs	Human	H1N1	pdm	2010	Singapore	A/Singapore/GP3563/2010	ggtgatgccccattccttgatcggctccgccgagatcaaaagtccttaaaaggaagaggcaacaccc	-	*
JX309602	Human H1N1pdm2009 IAVs	Human	H1N1		2010	Singapore	A/Singapore/KK441/2010	ggtgatgccccattccttgatcggctccgccgagatcaaaagtccttaaaaggaagaggcaacaccc	-	*
JX309586	Human H1N1pdm2009 IAVs	Human	H1N1	pdm	2010	Singapore	A/Singapore/KK444/2010	ggtgatgccccattccttgatcggctccgccgagatcaaaagtccttaaaaggaagaggcaacaccc	-	*
JX309772	Human H1N1pdm2009 IAVs	Human	H1N1		2010	Singapore	A/Singapore/GP3848/2010	ggtgatgccccattccttgatcggctccgccgagatcaaaagtccttaaaaggaagaggcaacaccc	-	*
CY096270	Human H1N1pdm2009 IAVs	Human	H1N1	pdm	2010	Australia	A/North_Fitzroy/INS474/2010	ggtgatgccccattccttgatcggctccgccgagatcaaaagtccttaaaaggaagaggcaacaccc	-	*
CY071283	Human H1N1pdm2009 IAVs	Human	H1N1	pdm	2010	Greece	A/Athens/INS413/2010	ggtgatgccccattccttgatcggctccgccgagatcaaaagtccttaaaaggaagaggcaacaccc	-	*
CY071323	Human H1N1pdm2009 IAVs	Human	H1N1	pdm	2010	Greece	A/Athens/INS421/2010	ggtgatgccccattccttgatcggctccgccgagatcaaaagtccttaaaaggaagaggcaacaccc	-	*

CY071187	Human H1N1pdm2009 IAVs	Human	H1N1	pdm	2010	Greece	A/Athens/INS398/2010	ggtgatgccccattccttgatcggctccgccgagatcaaaagtccttaaaaggaagaggcaacaccc	-	*
CY086916	Human H1N1pdm2009 IAVs	Swine	H1N1		2010	USA	A/swine/Minnesota/226128/2010	ggtgatgccccattccttgatcggctccgccgagatcaaaagtccttaaaaggaagaggcaacaccc	-	*
KC839533	Human H1N1pdm2009 IAVs	Swine	H1N2		2010	USA	A/swine/Minnesota/A01134332/2010	ggtgatgccccattccttgatcggctccgccgagatcaaaagtccttaaaaggaagaggcaacaccc	-	*
CY158445	Human H1N1pdm2009 IAVs	Swine	H1N1		2010	USA	A/swine/Illinois/02957/2010	ggtgatgccccattccttgatcggctccgccgagatcaaaagtccttaaaaggaagaggcaacaccc	-	*
KC782135	Human H1N1pdm2009 IAVs	Human	H1N1	pdm	2010	USA	A/Tennessee/01/2010	ggtgatgccccattccttgatcggctccgccgagatcaaaagtccttaaaaggaagaggcaacaccc	-	*
CY080343	Human H1N1pdm2009 IAVs	Human	H1N1	pdm	2010	Thailand	A/Thailand/CU_H1821/2010	ggtgatgccccattccttgatcggctccgccgagatcaaaagtccttaaaaggaagaggcaacaccc	-	*
CY096286	Human H1N1pdm2009 IAVs	Human	H1N1	pdm	2010	Australia	A/North_Fitzroy/INS476/2010	ggtgatgccccattccttgatcggctccgccgagatcaaaagtccttaaaaggaagaggcaacaccc	-	*
CY092948	Human H1N1pdm2009 IAVs	Human	H1N1	pdm	2010	Chile	A/Chile/6/2010	ggtgatgccccattccttgatcggctccgccgagatcaaaagtccttaaaaggaagaggcaacaccc	-	*
CY098142	Human H1N1pdm2009 IAVs	Human	H1N1		2010	Thailand	A/Bangkok/INS490/2010	ggtgatgccccattccttgatcggctccgccgagatcaaaagtccttaaaaggaagaggcaacaccc	-	*
CY096366	Human H1N1pdm2009 IAVs	Human	H1N1	pdm	2010	Thailand	A/Bangkok/INS491/2010	ggtgatgccccattccttgatcggctccgccgagatcaaaagtccttaaaaggaagaggcaacaccc	-	*
CY096382	Human H1N1pdm2009 IAVs	Human	H1N1		2010	Thailand	A/Bangkok/INS493/2010	ggtgatgccccattccttgatcggctccgccgagatcaaaagtccttaaaaggaagaggcaacaccc	-	*
CY096390	Human H1N1pdm2009 IAVs	Human	H1N1	pdm	2010	Thailand	A/Bangkok/INS494/2010	ggtgatgccccattccttgatcggctccgccgagatcaaaagtccttaaaaggaagaggcaacaccc	-	*
CY096406	Human H1N1pdm2009 IAVs	Human	H1N1	pdm	2010	Thailand	A/Bangkok/INS497/2010	ggtgatgccccattccttgatcggctccgccgagatcaaaagtccttaaaaggaagaggcaacaccc	-	*
CY096446	Human H1N1pdm2009 IAVs	Human	H1N1	pdm	2010	Thailand	A/Bangkok/INS503/2010	ggtgatgccccattccttgatcggctccgccgagatcaaaagtccttaaaaggaagaggcaacaccc	-	*
CY096470	Human H1N1pdm2009 IAVs	Human	H1N1	pdm	2010	Thailand	A/Bangkok/INS506/2010	ggtgatgccccattccttgatcggctccgccgagatcaaaagtccttaaaaggaagaggcaacaccc	-	*
CY129770	Human H1N1pdm2009 IAVs	Human	H1N1	pdm	2010	Thailand	A/Bangkok/INS581/2010	ggtgatgccccattccttgatcggctccgccgagatcaaaagtccttaaaaggaagaggcaacaccc	-	*
JX625850	Human H1N1pdm2009 IAVs	Human	H1N1	pdm	2010	United_Kingdom	A/England/287/2010	ggtgatgccccattccttgatcggctccgccgagatcaaaagtccttaaaaggaagaggcaacaccc	-	*
CY129802	Human H1N1pdm2009 IAVs	Human	H1N1		2010	Thailand	A/Khon_Kaen/INS585/2010	ggtgatgccccattccttgatcggctccgccgagatcaaaagtccttaaaaggaagaggcaacaccc	-	*
JX309780	Human H1N1pdm2009 IAVs	Human	H1N1	pdm	2010	Singapore	A/Singapore/GP4183/2010	ggtgatgccccattccttgatcggctccgccgagatcaaaagtccttaaaaggaagaggcaacaccc	-	*
CY098557	Human H1N1pdm2009 IAVs	Human	H1N1	pdm	2010	Thailand	A/Bangkok/INS516/2010	ggtgatgccccattccttgatcggctccgccgagatcaaaagtccttaaaaggaagaggcaacaccc	-	*
JX625962	Human H1N1pdm2009 IAVs	Human	H1N1		2010	United_Kingdom	A/Northern_Ireland/3/2010	ggtgatgccccattccttgatcggctccgccgagatcaaaagtccttaaaaggaagaggcaacaccc	-	*
JX309788	Human H1N1pdm2009 IAVs	Human	H1N1	pdm	2010	Singapore	A/Singapore/GP3956/2010	ggtgatgccccattccttgatcggctccgccgagatcaaaagtccttaaaaggaagaggcaacaccc	-	*
JX309862	Human H1N1pdm2009 IAVs	Human	H1N1		2010	Singapore	A/Singapore/GP4266/2010	ggtgatgccccattccttgatcggctccgccgagatcaaaagtccttaaaaggaagaggcaacaccc	-	*
CY096430	Human H1N1pdm2009 IAVs	Human	H1N1	pdm	2010	Thailand	A/Bangkok/INS500/2010	ggtgatgccccattccttgatcggctccgccgagatcaaaagtccttaaaaggaagaggcaacaccc	-	*
CY129794	Human H1N1pdm2009 IAVs	Human	H1N1	pdm	2010	Thailand	A/Bangkok/INS584/2010	ggtgatgccccattccttgatcggctccgccgagatcaaaagtccttaaaaggaagaggcaacaccc	-	*

CY176526	Human H1N1pdm2009 IAVs	Human	H1N1	pdm	2010	Thailand	A/Khon_Kaen/INS3_647/2010	ggtgatgccccattccttgatcggctccgccgagatca aaagtccttaaaaggaagaggcaacaccc	-	*
KJ690444	Human H1N1pdm2009 IAVs	Human	H1N1	pdm	2010	Uganda	A/Uganda/MUWRP_236/2010	ggtgatgccccattccttgatcggctccgccgagatca aaagtccttaaaaggaagaggcaacaccc	-	*
KJ023129	Human H1N1pdm2009 IAVs	Human	H1N1	pdm	2010	India	A/Delhi/039/2010	ggtgatgccccattccttgatcggctccgccgagatca aaagtccttaaaaggaagaggcaacaccc	-	*
JX625682	Human H1N1pdm2009 IAVs	Human	H1N1		2010	United_Kingdom	A/England/118/2010	ggtgatgccccattccttgatcggctccgccgagatca aaagtccttaaaaggaagaggcaacaccc	-	*
CY081097	Human H1N1pdm2009 IAVs	Human	H1N1	pdm	2010	Canada	A/Ontario/778873/2010	ggtgatgccccattccttgatcggctccgccgagatca aaagtccttaaaaggaagaggcaacaccc	-	*
JX309756	Human H1N1pdm2009 IAVs	Human	H1N1	pdm	2010	Singapore	A/Singapore/GP3760/2010	ggtgatgccccattccttgatcggctccgccgagatca aaagtccttaaaaggaagaggcaacaccc	-	*
CY129515	Human H1N1pdm2009 IAVs	Human	H1	pdm	2010	Australia	A/Darlinghurst/INS589/2010	ggtgatgccccattccttgatcggctccgccgagatca aaagtccttaaaaggaagaggcaacaccc	-	*
CY092844	Human H1N1pdm2009 IAVs	Human	H1N1	pdm	2010	Australia	A/Sydney/DD3_55/2010	ggtgatgccccattccttgatcggctccgccgagatca aaagtccttaaaaggaagaggcaacaccc	-	*
CY081105	Human H1N1pdm2009 IAVs	Human	H1N1	pdm	2010	Canada	A/Ontario/804635/2010	ggtgatgccccattccttgatcggctccgccgagatca aaagtccttaaaaggaagaggcaacaccc	-	*
JX625770	Human H1N1pdm2009 IAVs	Human	H1N1	pdm	2010	United_Kingdom	A/England/183/2010	ggtgatgccccattccttgatcggctccgccgagatca aaagtccttaaaaggaagaggcaacaccc	-	*
CY081073	Human H1N1pdm2009 IAVs	Human	H1N1	pdm	2010	Canada	A/Ontario/3620/2010	ggtgatgccccattccttgatcggctccgccgagatca aaagtccttaaaaggaagaggcaacaccc	-	*
CY081089	Human H1N1pdm2009 IAVs	Human	H1N1	pdm	2010	Canada	A/Ontario/741328/2010	ggtgatgccccattccttgatcggctccgccgagatca aaagtccttaaaaggaagaggcaacaccc	-	*
JX625914	Human H1N1pdm2009 IAVs	Human	H1N1	pdm	2010	United_Kingdom	A/England/05120538/2010	ggtgatgccccattccttgatcggctccgccgagatca aaagtccttaaaaggaagaggcaacaccc	-	*
JN584190	Human H1N1pdm2009 IAVs	Swine	H3N2		2010	Canada	A/swine/Quebec/1267568/2010	ggtgatgccccattccttgatcggctccgccgagatca aaagtccttaaaaggaagaggcaacaccc	-	*
CY065111	Human H1N1pdm2009 IAVs	Human	H1N1	pdm	2010	USA	A/New_York/7480/2010	ggtgatgccccattccttgatcggctccgccgagatca aaagtccttaaaaggaagaggcaacaccc	-	*
CY096438	Human H1N1pdm2009 IAVs	Human	H1N1	pdm	2010	Thailand	A/Bangkok/INS501/2010	ggtgatgccccattccttgatcggctccgccgagatca aaagtccttaaaaggaagaggcaacaccc	-	*
CY096078	Human H1N1pdm2009 IAVs	Human	H1N1	pdm	2010	Thailand	A/Khon_Kaen/INS443/2010	ggtgatgccccattccttgatcggctccgccgagatca aaagtccttaaaaggaagaggcaacaccc	-	*
CY083020	Human H1N1pdm2009 IAVs	Human	H1N1	pdm	2010	Senegal	A/Dakar/WRAIR0018N/2010	ggtgatgccccattccttgatcggctccgccgagatca aaagtccttaaaaggaagaggcaacaccc	-	*
CY083028	Human H1N1pdm2009 IAVs	Human	H1N1	pdm	2010	Senegal	A/Dakar/WRAIR0018T/2010	ggtgatgccccattccttgatcggctccgccgagatca aaagtccttaaaaggaagaggcaacaccc	-	*
CY083036	Human H1N1pdm2009 IAVs	Human	H1N1	pdm	2010	Senegal	A/Dakar/WRAIR0019T/2010	ggtgatgccccattccttgatcggctccgccgagatca aaagtccttaaaaggaagaggcaacaccc	-	*
CY073777	Human H1N1pdm2009 IAVs	Human	H1N1	pdm	2010	Austria	A/Vienna/INS369/2010	ggtgatgccccattccttgatcggctccgccgagatca aaagtccttaaaaggaagaggcaacaccc	-	*
CY129506	Human H1N1pdm2009 IAVs	Human	N1	pdm	2010	Germany	A/Wurzburg/INS579/2010	ggtgatgccccattccttgatcggctccgccgagatca aaagtccttaaaaggaagaggcaacaccc	-	*
CY073737	Human H1N1pdm2009 IAVs	Human	H1N1	pdm	2010	Estonia	A/Tallinn/INS431/2010	ggtgatgccccattccttgatcggctccgccgagatca aaagtccttaaaaggaagaggcaacaccc	-	*
CY083044	Human H1N1pdm2009 IAVs	Human	H1N1	pdm	2010	Senegal	A/Dakar/WRAIR0020T/2010	ggtgatgccccattccttgatcggctccgccgagatca aaagtccttaaaaggaagaggcaacaccc	-	*
CY097793	Human H1N1pdm2009 IAVs	Human	H1N1	pdm	2010	Senegal	A/Dakar/WRAIR0020T/2010	ggtgatgccccattccttgatcggctccgccgagatca aaagtccttaaaaggaagaggcaacaccc	-	*

CY062272	Human H1N1pdm2009 IAVs	Swine	H1N1		2010	Thailand	A/swine/Thailand/CU_RA114/2010	ggtgatgccccattccttgatcggctccgccgagatca aaagtccttaaaaggaagaggcaacaccc	-	*
CY062280	Human H1N1pdm2009 IAVs	Swine	H1N1		2010	Thailand	A/swine/Thailand/CU_RA15/2010	ggtgatgccccattccttgatcggctccgccgagatca aaagtccttaaaaggaagaggcaacaccc	-	*
KF411275	Human H1N1pdm2009 IAVs	Human	H1N1	pdm	2010	China	A/Qingdao/F966/2010	ggtgatgccccattccttgatcggctccgccgagatca aaagtccttaaaaggaagaggcaacaccc	-	*
CY100526	Human H1N1pdm2009 IAVs	Human	H1N1	pdm	2010	Mexico	A/Mexico_City/INER9/2010	ggtgatgccccattccttgatcggctccgccgagatca aaagtccttaaaaggaagaggcaacaccc	-	*
JX309836	Human H1N1pdm2009 IAVs	Human	H1N1	pdm	2010	Singapore	A/Singapore/EN193/2010	ggtgatgccccattccttgatcggctccgccgagatca aaagtccttaaaaggaagaggcaacaccc	-	*
KC781351	Human H1N1pdm2009 IAVs	Human	H1N1	pdm	2010	USA	A/Iowa/04/2010	ggtgatgccccattccttgatcggctccgccgagatca aaagtccttaaaaggaagaggcaacaccc	-	*
CY080311	Human H1N1pdm2009 IAVs	Human	H1N1	pdm	2010	Thailand	A/Thailand/C602/2010	ggtgatgccccattccttgatcggctccgccgagatca aaagtccttaaaaggaagaggcaacaccc	-	*
CY080319	Human H1N1pdm2009 IAVs	Human	H1N1	pdm	2010	Thailand	A/Thailand/H1255/2010	ggtgatgccccattccttgatcggctccgccgagatca aaagtccttaaaaggaagaggcaacaccc	-	*
JQ220554	Human H1N1pdm2009 IAVs	Swine	H3N2		2010	China	A/swine/Guangxi/NS2783/2010	ggtgatgccccattccttgatcggctccgccgagatca aaagtccttaaaaggaagaggcaacaccc	-	*
KC780492	Human H1N1pdm2009 IAVs	Human	H1N1		2010	USA	A/Wisconsin/01/2010	ggtgatgccccattccttgatcggctccgccgagatca aaagtccttaaaaggaagaggcaacaccc	-	*
KC780518	Human H1N1pdm2009 IAVs	Human	H1N1	pdm	2010	USA	A/Wisconsin/02/2010	ggtgatgccccattccttgatcggctccgccgagatca aaagtccttaaaaggaagaggcaacaccc	-	*
JF420891	Human H1N1pdm2009 IAVs	Swine	H3N2		2010	Canada	A/swine/Quebec/1259260/2010	ggtgatgccccattccttgatcggctccgccgagatca aaagtccttaaaaggaagaggcaacaccc	-	*
CY089814	Human H1N1pdm2009 IAVs	Swine	H1N1		2010	Thailand	A/swine/Thailand/CU_RP1/2010	ggtgatgccccattccttgatcggctccgccgagatca aaagtccttaaaaggaagaggcaacaccc	-	*
CY089821	Human H1N1pdm2009 IAVs	Swine	H1N1		2010	Thailand	A/swine/Thailand/CU_RP3/2010	ggtgatgccccattccttgatcggctccgccgagatca aaagtccttaaaaggaagaggcaacaccc	-	*
CY062134	Human H1N1pdm2009 IAVs	Human	H1N1	pdm	2010	USA	A/New_York/2876/2010	ggtgatgccccattccttgatcggctccgccgagatca aaagtccttaaaaggaagaggcaacaccc	-	*
HQ393497	Human H1N1pdm2009 IAVs	Swine	H1N1		2010	Taiwan	A/swine/Taiwan/TD_2542/2010	ggtgatgccccattccttgatcggctccgccgagatca aaagtccttaaaaggaagaggcaacaccc	-	*
CY158789	Human H1N1pdm2009 IAVs	Swine	H1N1		2010	Mexico	A/swine/Mexico/SG1443/2010	ggtgatgccccattccttgatcggctccgccgagatca aaagtccttaaaaggaagaggcaacaccc	-	*
JN375253	Human H1N1pdm2009 IAVs	Swine	H1N1		2010	China	A/swine/Guangdong/275/2010	ggtgatgccccattccttgatcggctccgccgagatca aaagtccttaaaaggaagaggcaacaccc	-	*
JN375254	Human H1N1pdm2009 IAVs	Swine	H1N1		2010	China	A/swine/Guangdong/278/2010	ggtgatgccccattccttgatcggctccgccgagatca aaagtccttaaaaggaagaggcaacaccc	-	*
JN375255	Human H1N1pdm2009 IAVs	Swine	H1N1		2010	China	A/swine/Guangdong/286/2010	ggtgatgccccattccttgatcggctccgccgagatca aaagtccttaaaaggaagaggcaacaccc	-	*
JN375256	Human H1N1pdm2009 IAVs	Swine	H1N1		2010	China	A/swine/Guangdong/294/2010	ggtgatgccccattccttgatcggctccgccgagatca aaagtccttaaaaggaagaggcaacaccc	-	*
JN375257	Human H1N1pdm2009 IAVs	Swine	H1N1		2010	China	A/swine/Guangdong/297/2010	ggtgatgccccattccttgatcggctccgccgagatca aaagtccttaaaaggaagaggcaacaccc	-	*
KC780142	Human H1N1pdm2009 IAVs	Human	H1N1		2010	USA	A/California/06/2010	ggtgatgccccattccttgatcggctccgccgagatca aaagtccttaaaaggaagaggcaacaccc	-	*
JF275929	Human H1N1pdm2009 IAVs	Swine	H1N1		2010	China	A/swine/Nanchang/F9/2010	ggtgatgccccattccttgatcggctccgccgagatca aaagtccttaaaaggaagaggcaacaccc	-	*
CY062983	Human H1N1pdm2009 IAVs	Human	H1N1	pdm	2010	Germany	A/Bonn/INS178/2010	ggtgatgccccattccttgatcggctccgccgagatca aaagtccttaaaaggaagaggcaacaccc	-	*

JQ612487	Human H1N1pdm2009 IAVs	Swine	H1N1		2010	Hungary	A/swine/Hungary/4431/2010	ggtgatgccccattccttgatcggctccgccgagatca aaagtccttaaaaggaagaggcaacaccc	-	*
CY115987	Human H1N1pdm2009 IAVs	Swine	H1N1		2010	United_Kingdom	A/swine/England/1458/2010	ggtgatgccccattccttgatcggctccgccgagatca aaagtccttaaaaggaagaggcaacaccc	-	*
CY115995	Human H1N1pdm2009 IAVs	Swine	H1N1		2010	United_Kingdom	A/swine/England/1464/2010	ggtgatgccccattccttgatcggctccgccgagatca aaagtccttaaaaggaagaggcaacaccc	-	*
CY075079	Human H1N1pdm2009 IAVs	Human	H1N1	pdm	2010	Greece	A/Athens/INS416/2010	ggtgatgccccattccttgatcggctccgccgagatca aaagtccttaaaaggaagaggcaacaccc	-	*
JQ612511	Human H1N1pdm2009 IAVs	Human	H1N1	pdm	2010	Hungary	A/Hungary/2/2010	ggtgatgccccattccttgatcggctccgccgagatca aaagtccttaaaaggaagaggcaacaccc	-	*
HM569744	Human H1N1pdm2009 IAVs	Human	H1N1	pdm	2010	Russia	A/Orenburg/IIV_13/2010	ggtgatgccccattccttgatcggctccgccgagatca aaagtccttaaaaggaagaggcaacaccc	-	*
HM124387	Human H1N1pdm2009 IAVs	Human	H1N1	pdm	2010	Russia	A/Orenburg/IIV_13/2010	ggtgatgccccattccttgatcggctccgccgagatca aaagtccttaaaaggaagaggcaacaccc	-	*
CY071075	Human H1N1pdm2009 IAVs	Human	H1N1	pdm	2010	Estonia	A/Tallinn/INS373/2010	ggtgatgccccattccttgatcggctccgccgagatca aaagtccttaaaaggaagaggcaacaccc	-	*
JX308953	Human H1N1pdm2009 IAVs	Human	H1N1	pdm	2010	Singapore	A/Singapore/GP10/2010	ggtgatgccccattccttgatcggctccgccgagatca aaagtccttaaaaggaagaggcaacaccc	-	*
JF290395	Human H1N1pdm2009 IAVs	Swine	H1N2		2010	United_Kingdom	A/swine/England/1382/2010	ggtgatgccccattccttgatcggctccgccgagatca aaagtccttaaaaggaagaggcaacaccc	-	*
CY099187	Human H1N1pdm2009 IAVs	Swine	H1N1		2010	USA	A/swine/Minnesota/02979/2010	ggtgatgccccattccttgatcggctccgccgagatca aaagtccttaaaaggaagaggcaacaccc	-	*
JX309642	Human H1N1pdm2009 IAVs	Human	H1N1		2010	Singapore	A/Singapore/GP3481/2010	ggtgatgccccattccttgatcggctccgccgagatca aaagtccttaaaaggaagaggcaacaccc	-	*
CY086932	Human H1N1pdm2009 IAVs	Swine	H1N2		2010	USA	A/swine/Minnesota/239106/2010	ggtgatgccccattccttgatcggctccgccgagatca aaagtccttaaaaggaagaggcaacaccc	-	*
JX309363	Human H1N1pdm2009 IAVs	Human	H1N1	pdm	2010	Singapore	A/Singapore/GP1112/2010	ggtgatgccccattccttgatcggctccgccgagatca aaagtccttaaaaggaagaggcaacaccc	-	*
JX309374	Human H1N1pdm2009 IAVs	Human	H1N1	pdm	2010	Singapore	A/Singapore/GP1212/2010	ggtgatgccccattccttgatcggctccgccgagatca aaagtccttaaaaggaagaggcaacaccc	-	*
JX309610	Human H1N1pdm2009 IAVs	Human	H1N1	pdm	2010	Singapore	A/Singapore/KK376/2010	ggtgatgccccattccttgatcggctccgccgagatca aaagtccttaaaaggaagaggcaacaccc	-	*
JX309536	Human H1N1pdm2009 IAVs	Human	H1N1	pdm	2010	Singapore	A/Singapore/SS34/2010	ggtgatgccccattccttgatcggctccgccgagatca aaagtccttaaaaggaagaggcaacaccc	-	*
JX309290	Human H1N1pdm2009 IAVs	Human	H1N1	pdm	2010	Singapore	A/Singapore/TT150/2010	ggtgatgccccattccttgatcggctccgccgagatca aaagtccttaaaaggaagaggcaacaccc	-	*
JX309101	Human H1N1pdm2009 IAVs	Human	H1N1	pdm	2010	Singapore	A/Singapore/GP166/2010	ggtgatgccccattccttgatcggctccgccgagatca aaagtccttaaaaggaagaggcaacaccc	-	*
JX309137	Human H1N1pdm2009 IAVs	Human	H1N1	pdm	2010	Singapore	A/Singapore/TT37/2010	ggtgatgccccattccttgatcggctccgccgagatca aaagtccttaaaaggaagaggcaacaccc	-	*
KC780543	Human H1N1pdm2009 IAVs	Human	H1N1	pdm	2010	USA	A/New_Jersey/01/2010	ggtgatgccccattccttgatcggctccgccgagatca aaagtccttaaaaggaagaggcaacaccc	-	*
CY071163	Human H1N1pdm2009 IAVs	Human	H1N1	pdm	2010	Greece	A/Athens/INS394/2010	ggtgatgccccattccttgatcggctccgccgagatca aaagtccttaaaaggaagaggcaacaccc	-	*
CY071307	Human H1N1pdm2009 IAVs	Human	H1N1	pdm	2010	Greece	A/Athens/INS419/2010	ggtgatgccccattccttgatcggctccgccgagatca aaagtccttaaaaggaagaggcaacaccc	-	*
KJ690396	Human H1N1pdm2009 IAVs	Human	H1N1	pdm	2010	Uganda	A/Uganda/MUWRP_176/2010	ggtgatgccccattccttgatcggctccgccgagatca aaagtccttaaaaggaagaggcaacaccc	-	*
CY096590	Human H1N1pdm2009 IAVs	Human	H1N1	pdm	2010	USA	A/District_of_Columbia/INS525/2010	ggtgatgccccattccttgatcggctccgccgagatca aaagtccttaaaaggaagaggcaacaccc	-	*

KM029659	Human H1N1pdm2009 IAVs	Swine	H1N1		2010	China	A/swine/Hong_Kong/NS3815/2010	ggtgatgccccattccttgatcggctccgccgagatca aaagtccttaaaaggaagaggcaacaccc	-	*
KM029667	Human H1N1pdm2009 IAVs	Swine	H1N1		2010	China	A/swine/Hong_Kong/NS3817/2010	ggtgatgccccattccttgatcggctccgccgagatca aaagtccttaaaaggaagaggcaacaccc	-	*
CY160011	Human H1N1pdm2009 IAVs	Swine	H1N1		2010	USA	A/swine/North_Carolina/SG1362/2010	ggtgatgccccattccttgatcggctccgccgagatca aaagtccttaaaaggaagaggcaacaccc	-	*
CY071331	Human H1N1pdm2009 IAVs	Human	H1N1	pdm	2010	Thailand	A/Bangkok/INS424/2010	ggtgatgccccattccttgatcggctccgccgagatca aaagtccttaaaaggaagaggcaacaccc	-	*
KC781187	Human H1N1pdm2009 IAVs	Human	H1N1	pdm	2010	USA	A/Wyoming/01/2010	ggtgatgccccattccttgatcggctccgccgagatca aaagtccttaaaaggaagaggcaacaccc	-	*
CY092964	Human H1N1pdm2009 IAVs	Human	H1N1	pdm	2010	Chile	A/Chile/19/2010	ggtgatgccccattccttgatcggctccgccgagatca aaagtccttaaaaggaagaggcaacaccc	-	*
KF612175	Human H1N1pdm2009 IAVs	Human	H1N1	pdm	2010	Bolivia	A/Santa_Cruz/005/2010	ggtgatgccccattccttgatcggctccgccgagatca aaagtccttaaaaggaagaggcaacaccc	-	*
KF612177	Human H1N1pdm2009 IAVs	Human	H1N1	pdm	2010	Bolivia	A/Santa_Cruz/995/2010	ggtgatgccccattccttgatcggctccgccgagatca aaagtccttaaaaggaagaggcaacaccc	-	*
KC781297	Human H1N1pdm2009 IAVs	Human	H1N1	pdm	2010	USA	A/Washington/01/2010	ggtgatgccccattccttgatcggctccgccgagatca aaagtccttaaaaggaagaggcaacaccc	-	*
KC781896	Human H1N1pdm2009 IAVs	Human	H1N1	pdm	2010	USA	A/Alabama/01/2010	ggtgatgccccattccttgatcggctccgccgagatca aaagtccttaaaaggaagaggcaacaccc	-	*
CY071363	Human H1N1pdm2009 IAVs	Human	H1N1	pdm	2010	Thailand	A/Bangkok/INS428/2010	ggtgatgccccattccttgatcggctccgccgagatca aaagtccttaaaaggaagaggcaacaccc	-	*
CY096326	Human H1N1pdm2009 IAVs	Human	H1N1	pdm	2010	Thailand	A/Bangkok/INS481/2010	ggtgatgccccattccttgatcggctccgccgagatca aaagtccttaaaaggaagaggcaacaccc	-	*
CY071275	Human H1N1pdm2009 IAVs	Human	H1N1	pdm	2010	Greece	A/Athens/INS412/2010	ggtgatgccccattccttgatcggctccgccgagatca aaagtccttaaaaggaagaggcaacaccc	-	*
CY076743	Human H1N1pdm2009 IAVs	Swine	H1N1		2010	Thailand	A/swine/Thailand/CU_SA43/2010	ggtgatgccccattccttgatcggctccgccgagatca aaagtccttaaaaggaagaggcaacaccc	-	*
KC780576	Human H1N1pdm2009 IAVs	Human	H1N1	pdm	2010	USA	A/Virginia/01/2010	ggtgatgccccattccttgatcggctccgccgagatca aaagtccttaaaaggaagaggcaacaccc	-	*
JN584206	Human H1N1pdm2009 IAVs	Swine	H3N2		2010	Canada	A/swine/Quebec/1257777/2010	ggtgatgccccattccttgatcggctccgccgagatca aaagtccttaaaaggaagaggcaacaccc	-	*
JX625858	Human H1N1pdm2009 IAVs	Human	H1N1	pdm	2010	United_Kingdom	A/England/05060351/2010	ggtgatgccccattccttgatcggctccgccgagatca aaagtccttaaaaggaagaggcaacaccc	-	*
JX625866	Human H1N1pdm2009 IAVs	Human	H1N1	pdm	2010	United_Kingdom	A/England/05060444/2010	ggtgatgccccattccttgatcggctccgccgagatca aaagtccttaaaaggaagaggcaacaccc	-	*
JX625874	Human H1N1pdm2009 IAVs	Human	H1N1	pdm	2010	United_Kingdom	A/England/05060454/2010	ggtgatgccccattccttgatcggctccgccgagatca aaagtccttaaaaggaagaggcaacaccc	-	*
JX625906	Human H1N1pdm2009 IAVs	Human	H1N1	pdm	2010	United_Kingdom	A/England/05100690/2010	ggtgatgccccattccttgatcggctccgccgagatca aaagtccttaaaaggaagaggcaacaccc	-	*
JX625930	Human H1N1pdm2009 IAVs	Human	H1N1	pdm	2010	United_Kingdom	A/England/05140206/2010	ggtgatgccccattccttgatcggctccgccgagatca aaagtccttaaaaggaagaggcaacaccc	-	*
JX625938	Human H1N1pdm2009 IAVs	Human	H1N1	pdm	2010	United_Kingdom	A/England/05140214/2010	ggtgatgccccattccttgatcggctccgccgagatca aaagtccttaaaaggaagaggcaacaccc	-	*
JX625946	Human H1N1pdm2009 IAVs	Human	H1N1	pdm	2010	United_Kingdom	A/England/05160856/2010	ggtgatgccccattccttgatcggctccgccgagatca aaagtccttaaaaggaagaggcaacaccc	-	*
JX625698	Human H1N1pdm2009 IAVs	Human	H1N1	pdm	2010	United_Kingdom	A/England/143/2010	ggtgatgccccattccttgatcggctccgccgagatca aaagtccttaaaaggaagaggcaacaccc	-	*
JX625922	Human H1N1pdm2009 IAVs	Human	H1N1		2010	United_Kingdom	A/England/371/2010	ggtgatgccccattccttgatcggctccgccgagatca aaagtccttaaaaggaagaggcaacaccc	-	*

CY092586	Human H1N1pdm2009 IAVs	Human	H1N1	pdm	2010	Australia	A/Sydney/DD3_25/2010	ggtgatgccccattccttgatcggctccgccgagatca aaagtccttaaaaggaagaggcaacaccc	-	*
CY092413	Human H1N1pdm2009 IAVs	Human	H1N1	pdm	2010	Australia	A/Sydney/DD3_48/2010	ggtgatgccccattccttgatcggctccgccgagatca aaagtccttaaaaggaagaggcaacaccc	-	*
CY167480	Human H1N1pdm2009 IAVs	Human	H1N1	pdm	2010	USA	A/Tennessee/F2008c07A/2010	ggtgatgccccattccttgatcggctccgccgagatca aaagtccttaaaaggaagaggcaacaccc	-	*
JX625666	Human H1N1pdm2009 IAVs	Human	H1N1	pdm	2010	United_Kingdom	A/England/04640543/2010	ggtgatgccccattccttgatcggctccgccgagatca aaagtccttaaaaggaagaggcaacaccc	-	*
JX625762	Human H1N1pdm2009 IAVs	Human	H1N1		2010	United_Kingdom	A/England/04920303/2010	ggtgatgccccattccttgatcggctccgccgagatca aaagtccttaaaaggaagaggcaacaccc	-	*
JX625778	Human H1N1pdm2009 IAVs	Human	H1N1	pdm	2010	United_Kingdom	A/England/04940476/2010	ggtgatgccccattccttgatcggctccgccgagatca aaagtccttaaaaggaagaggcaacaccc	-	*
KC882093	Human H1N1pdm2009 IAVs	Human	H1N1	pdm	2010	USA	A/Kentucky/09/2010	ggtgatgccccattccttgatcggctccgccgagatca aaagtccttaaaaggaagaggcaacaccc	-	*
KC882062	Human H1N1pdm2009 IAVs	Human	H1N1		2010	USA	A/Kentucky/09/2010	ggtgatgccccattccttgatcggctccgccgagatca aaagtccttaaaaggaagaggcaacaccc	-	*
JX309881	Human H1N1pdm2009 IAVs	Human	H1N1	pdm	2010	Singapore	A/Singapore/GP4344/2010	ggtgatgccccattccttgatcggctccgccgagatca aaagtccttaaaaggaagaggcaacaccc	-	*
JX309820	Human H1N1pdm2009 IAVs	Human	H1N1	pdm	2010	Singapore	A/Singapore/TT454/2010	ggtgatgccccattccttgatcggctccgccgagatca aaagtccttaaaaggaagaggcaacaccc	-	*
JX625898	Human H1N1pdm2009 IAVs	Human	H1N1	pdm	2010	United_Kingdom	A/England/05080815/2010	ggtgatgccccattccttgatcggctccgccgagatca aaagtccttaaaaggaagaggcaacaccc	-	*
JX625690	Human H1N1pdm2009 IAVs	Human	H1N1	pdm	2010	United_Kingdom	A/England/142/2010	ggtgatgccccattccttgatcggctccgccgagatca aaagtccttaaaaggaagaggcaacaccc	-	*
JX625738	Human H1N1pdm2009 IAVs	Human	H1N1	pdm	2010	United_Kingdom	A/England/04880374/2010	ggtgatgccccattccttgatcggctccgccgagatca aaagtccttaaaaggaagaggcaacaccc	-	*
JX625882	Human H1N1pdm2009 IAVs	Human	H1N1		2010	United_Kingdom	A/England/282/2010	ggtgatgccccattccttgatcggctccgccgagatca aaagtccttaaaaggaagaggcaacaccc	-	*
JX309926	Human H1N1pdm2009 IAVs	Human	H1N1	pdm	2010	Singapore	A/Singapore/GP4511/2010	ggtgatgccccattccttgatcggctccgccgagatca aaagtccttaaaaggaagaggcaacaccc	-	*
JX309889	Human H1N1pdm2009 IAVs	Human	H1N1	pdm	2010	Singapore	A/Singapore/GP4369/2010	ggtgatgccccattccttgatcggctccgccgagatca aaagtccttaaaaggaagaggcaacaccc	-	*
KJ023110	Human H1N1pdm2009 IAVs	Human	H1N1	pdm	2010	India	A/Assam/020/2010	ggtgatgccccattccttgatcggctccgccgagatca aaagtccttaaaaggaagaggcaacaccc	-	*
KJ023109	Human H1N1pdm2009 IAVs	Human	H1N1	pdm	2010	India	A/Rajasthan/019/2010	ggtgatgccccattccttgatcggctccgccgagatca aaagtccttaaaaggaagaggcaacaccc	-	*
KJ023113	Human H1N1pdm2009 IAVs	Human	H1N1	pdm	2010	India	A/Kerala/023/2010	ggtgatgccccattccttgatcggctccgccgagatca aaagtccttaaaaggaagaggcaacaccc	-	*
KF897781	Human H1N1pdm2009 IAVs	Human	H1N1	pdm	2010	France	A/La_Reunion/803/2010	ggtgatgccccattccttgatcggctccgccgagatca aaagtccttaaaaggaagaggcaacaccc	-	*
KJ023114	Human H1N1pdm2009 IAVs	Human	H1N1	pdm	2010	India	A/Madhya_Pradesh/024/2010	ggtgatgccccattccttgatcggctccgccgagatca aaagtccttaaaaggaagaggcaacaccc	-	*
JX309594	Human H1N1pdm2009 IAVs	Human	H1N1	pdm	2010	Singapore	A/Singapore/GP2892/2010	ggtgatgccccattccttgatcggctccgccgagatca aaagtccttaaaaggaagaggcaacaccc	-	*
JX309670	Human H1N1pdm2009 IAVs	Human	H1N1	pdm	2010	Singapore	A/Singapore/GP3667/2010	ggtgatgccccattccttgatcggctccgccgagatca aaagtccttaaaaggaagaggcaacaccc	-	*
CY092594	Human H1N1pdm2009 IAVs	Human	H1N1	pdm	2010	Australia	A/Sydney/DD3_26/2010	ggtgatgccccattccttgatcggctccgccgagatca aaagtccttaaaaggaagaggcaacaccc	-	*
CY092405	Human H1N1pdm2009 IAVs	Human	H1N1	pdm	2010	Australia	A/Sydney/DD3_46/2010	ggtgatgccccattccttgatcggctccgccgagatca aaagtccttaaaaggaagaggcaacaccc	-	*

CY092722	Human H1N1pdm2009 IAVs	Human	H1N1		2010	Australia	A/Sydney/DD3_52/2010	ggtgatgccccattccttgatcggctccgccgagatcaaaagtccttaaaaggaagaggcaacaccc	-	*
JN714488	Human H1N1pdm2009 IAVs	Human	H1N1	pdm	2010	Russia	A/Nizhnii_Novgorod/CRIE_NGV/2010	ggtgatgccccattccttgatcggctccgccgagatcaaaagtccttaaaaggaagaggcaacaccc	-	*
CY096342	Human H1N1pdm2009 IAVs	Human	H1N1		2010	Thailand	A/Bangkok/INS483/2010	ggtgatgccccattccttgatcggctccgccgagatcaaaagtccttaaaaggaagaggcaacaccc	-	*
CY096462	Human H1N1pdm2009 IAVs	Human	H1N1		2010	Thailand	A/Bangkok/INS505/2010	ggtgatgccccattccttgatcggctccgccgagatcaaaagtccttaaaaggaagaggcaacaccc	-	*
CY096533	Human H1N1pdm2009 IAVs	Human	H1N1	pdm	2010	Thailand	A/Bangkok/INS518/2010	ggtgatgccccattccttgatcggctccgccgagatcaaaagtccttaaaaggaagaggcaacaccc	-	*
CY096541	Human H1N1pdm2009 IAVs	Human	H1N1	pdm	2010	Thailand	A/Bangkok/INS519/2010	ggtgatgccccattccttgatcggctccgccgagatcaaaagtccttaaaaggaagaggcaacaccc	-	*
CY093153	Human H1N1pdm2009 IAVs	Human	H1N1	pdm	2010	Australia	A/Sydney/DD3_23/2010	ggtgatgccccattccttgatcggctccgccgagatcaaaagtccttaaaaggaagaggcaacaccc	-	*
CY092602	Human H1N1pdm2009 IAVs	Human	H1N1		2010	Australia	A/Sydney/DD3_28/2010	ggtgatgccccattccttgatcggctccgccgagatcaaaagtccttaaaaggaagaggcaacaccc	-	*
CY092626	Human H1N1pdm2009 IAVs	Human	H1N1	pdm	2010	Australia	A/Sydney/DD3_35/2010	ggtgatgccccattccttgatcggctccgccgagatcaaaagtccttaaaaggaagaggcaacaccc	-	*
CY092634	Human H1N1pdm2009 IAVs	Human	H1N1	pdm	2010	Australia	A/Sydney/DD3_36/2010	ggtgatgccccattccttgatcggctccgccgagatcaaaagtccttaaaaggaagaggcaacaccc	-	*
CY092682	Human H1N1pdm2009 IAVs	Human	H1N1		2010	Australia	A/Sydney/DD3_44/2010	ggtgatgccccattccttgatcggctccgccgagatcaaaagtccttaaaaggaagaggcaacaccc	-	*
CY088826	Human H1N1pdm2009 IAVs	Human	H1N1	pdm	2010	Thailand	A/Thailand/CU_H2389/2010	ggtgatgccccattccttgatcggctccgccgagatcaaaagtccttaaaaggaagaggcaacaccc	-	*
KC859178	Human H1N1pdm2009 IAVs	Swine	H1N1		2010	Thailand	A/swine/Thailand/CS210/2010	ggtgatgccccattccttgatcggctccgccgagatcaaaagtccttaaaaggaagaggcaacaccc	-	*
KC859098	Human H1N1pdm2009 IAVs	Swine	H1N1		2010	Thailand	A/swine/Thailand/CB228/2010	ggtgatgccccattccttgatcggctccgccgagatcaaaagtccttaaaaggaagaggcaacaccc	-	*
KC859106	Human H1N1pdm2009 IAVs	Swine	H1N1		2010	Thailand	A/swine/Thailand/RY227/2010	ggtgatgccccattccttgatcggctccgccgagatcaaaagtccttaaaaggaagaggcaacaccc	-	*
JX625722	Human H1N1pdm2009 IAVs	Human	H1N1		2010	United_Kingdom	A/England/04860492/2010	ggtgatgccccattccttgatcggctccgccgagatcaaaagtccttaaaaggaagaggcaacaccc	-	*
JX625818	Human H1N1pdm2009 IAVs	Human	H1N1	pdm	2010	United_Kingdom	A/England/05020366/2010	ggtgatgccccattccttgatcggctccgccgagatcaaaagtccttaaaaggaagaggcaacaccc	-	*
JX625834	Human H1N1pdm2009 IAVs	Human	H1N1	pdm	2010	United_Kingdom	A/England/05040482/2010	ggtgatgccccattccttgatcggctccgccgagatcaaaagtccttaaaaggaagaggcaacaccc	-	*
JX625890	Human H1N1pdm2009 IAVs	Human	H1N1	pdm	2010	United_Kingdom	A/England/05080771/2010	ggtgatgccccattccttgatcggctccgccgagatcaaaagtccttaaaaggaagaggcaacaccc	-	*
JX625978	Human H1N1pdm2009 IAVs	Human	H1N1	pdm	2010	United_Kingdom	A/England/05260592/2010	ggtgatgccccattccttgatcggctccgccgagatcaaaagtccttaaaaggaagaggcaacaccc	-	*
JX625754	Human H1N1pdm2009 IAVs	Human	H1N1	pdm	2010	United_Kingdom	A/England/178/2010	ggtgatgccccattccttgatcggctccgccgagatcaaaagtccttaaaaggaagaggcaacaccc	-	*
JX625706	Human H1N1pdm2009 IAVs	Human	H1N1	pdm	2010	United_Kingdom	A/England/209/2010	ggtgatgccccattccttgatcggctccgccgagatcaaaagtccttaaaaggaagaggcaacaccc	-	*
JX625802	Human H1N1pdm2009 IAVs	Human	H1N1		2010	United_Kingdom	A/England/272/2010	ggtgatgccccattccttgatcggctccgccgagatcaaaagtccttaaaaggaagaggcaacaccc	-	*
JX625954	Human H1N1pdm2009 IAVs	Human	H1N1	pdm	2010	United_Kingdom	A/Northern_Ireland/05180459/2010	ggtgatgccccattccttgatcggctccgccgagatcaaaagtccttaaaaggaagaggcaacaccc	-	*
JX625714	Human H1N1pdm2009 IAVs	Human	H1N1	pdm	2010	United_Kingdom	A/England/181/2010	ggtgatgccccattccttgatcggctccgccgagatcaaaagtccttaaaaggaagaggcaacaccc	-	*

JX625730	Human H1N1pdm2009 IAVs	Human	H1N1	pdm	2010	United_Kingdom	A/England/04860493/2010	ggtgatgccccattccttgatcggctccgccgagatca aaagtccttaaaaggaagaggcaacaccc	-	*
JX625970	Human H1N1pdm2009 IAVs	Human	H1N1	pdm	2010	United_Kingdom	A/England/05180496/2010	ggtgatgccccattccttgatcggctccgccgagatca aaagtccttaaaaggaagaggcaacaccc	-	*
JX625842	Human H1N1pdm2009 IAVs	Human	H1N1		2010	United_Kingdom	A/England/05040499/2010	ggtgatgccccattccttgatcggctccgccgagatca aaagtccttaaaaggaagaggcaacaccc	-	*
CY089441	Human H1N1pdm2009 IAVs	Human	H1N1	pdm	2010	Thailand	A/Thailand/CU_B4339/2010	ggtgatgccccattccttgatcggctccgccgagatca aaagtccttaaaaggaagaggcaacaccc	-	*
KC882130	Human H1N1pdm2009 IAVs	Human	H1N1	pdm	2010	USA	A/New_York/14/2010	ggtgatgccccattccttgatcggctccgccgagatca aaagtccttaaaaggaagaggcaacaccc	-	*
KJ023111	Human H1N1pdm2009 IAVs	Human	H1N1	pdm	2010	India	A/Jammu_and_Kashmir/021/2010	ggtgatgccccattccttgatcggctccgccgagatca aaagtccttaaaaggaagaggcaacaccc	-	*
KJ023112	Human H1N1pdm2009 IAVs	Human	H1N1		2010	India	A/Jammu_and_Kashmir/022/2010	ggtgatgccccattccttgatcggctccgccgagatca aaagtccttaaaaggaagaggcaacaccc	-	*
JF764083	Human H1N1pdm2009 IAVs	Human	H1N1	pdm	2010	India	A/India/GWL_DSC/2010	ggtgatgccccattccttgatcggctccgccgagatca aaagtccttaaaaggaagaggcaacaccc	-	*
CY081065	Human H1N1pdm2009 IAVs	Human	H1N1	pdm	2010	Canada	A/Ontario/130741/2010	ggtgatgccccattccttgatcggctccgccgagatca aaagtccttaaaaggaagaggcaacaccc	-	*
JX309812	Human H1N1pdm2009 IAVs	Human	H1N1		2010	Singapore	A/Singapore/TT450/2010	ggtgatgccccattccttgatcggctccgccgagatca aaagtccttaaaaggaagaggcaacaccc	-	*
JX309942	Human H1N1pdm2009 IAVs	Human	H1N1		2010	Singapore	A/Singapore/GP4610/2010	ggtgatgccccattccttgatcggctccgccgagatca aaagtccttaaaaggaagaggcaacaccc	-	*
CY092554	Human H1N1pdm2009 IAVs	Human	H1N1	pdm	2010	Australia	A/Sydney/DD3_17/2010	ggtgatgccccattccttgatcggctccgccgagatca aaagtccttaaaaggaagaggcaacaccc	-	*
CY096294	Human H1N1pdm2009 IAVs	Human	H1N1	pdm	2010	Thailand	A/Bangkok/INS477/2010	ggtgatgccccattccttgatcggctccgccgagatca aaagtccttaaaaggaagaggcaacaccc	-	*
CY096318	Human H1N1pdm2009 IAVs	Human	H1N1	pdm	2010	Thailand	A/Bangkok/INS480/2010	ggtgatgccccattccttgatcggctccgccgagatca aaagtccttaaaaggaagaggcaacaccc	-	*
CY098110	Human H1N1pdm2009 IAVs	Human	H1N1	pdm	2010	Thailand	A/Bangkok/INS484/2010	ggtgatgccccattccttgatcggctccgccgagatca aaagtccttaaaaggaagaggcaacaccc	-	*
CY096350	Human H1N1pdm2009 IAVs	Human	H1N1	pdm	2010	Thailand	A/Bangkok/INS485/2010	ggtgatgccccattccttgatcggctccgccgagatca aaagtccttaaaaggaagaggcaacaccc	-	*
CY098134	Human H1N1pdm2009 IAVs	Human	H1N1	pdm	2010	Thailand	A/Bangkok/INS488/2010	ggtgatgccccattccttgatcggctccgccgagatca aaagtccttaaaaggaagaggcaacaccc	-	*
CY096358	Human H1N1pdm2009 IAVs	Human	H1N1	pdm	2010	Thailand	A/Bangkok/INS489/2010	ggtgatgccccattccttgatcggctccgccgagatca aaagtccttaaaaggaagaggcaacaccc	-	*
CY096398	Human H1N1pdm2009 IAVs	Human	H1N1	pdm	2010	Thailand	A/Bangkok/INS495/2010	ggtgatgccccattccttgatcggctccgccgagatca aaagtccttaaaaggaagaggcaacaccc	-	*
CY096414	Human H1N1pdm2009 IAVs	Human	H1N1	pdm	2010	Thailand	A/Bangkok/INS498/2010	ggtgatgccccattccttgatcggctccgccgagatca aaagtccttaaaaggaagaggcaacaccc	-	*
CY098150	Human H1N1pdm2009 IAVs	Human	H1N1	pdm	2010	Thailand	A/Bangkok/INS502/2010	ggtgatgccccattccttgatcggctccgccgagatca aaagtccttaaaaggaagaggcaacaccc	-	*
CY096454	Human H1N1pdm2009 IAVs	Human	H1N1	pdm	2010	Thailand	A/Bangkok/INS504/2010	ggtgatgccccattccttgatcggctccgccgagatca aaagtccttaaaaggaagaggcaacaccc	-	*
CY098158	Human H1N1pdm2009 IAVs	Human	H1N1	pdm	2010	Thailand	A/Bangkok/INS508/2010	ggtgatgccccattccttgatcggctccgccgagatca aaagtccttaaaaggaagaggcaacaccc	-	*
CY096486	Human H1N1pdm2009 IAVs	Human	H1N1	pdm	2010	Thailand	A/Bangkok/INS510/2010	ggtgatgccccattccttgatcggctccgccgagatca aaagtccttaaaaggaagaggcaacaccc	-	*
CY096510	Human H1N1pdm2009 IAVs	Human	H1N1	pdm	2010	Thailand	A/Bangkok/INS513/2010	ggtgatgccccattccttgatcggctccgccgagatca aaagtccttaaaaggaagaggcaacaccc	-	*

CY096517	Human H1N1pdm2009 IAVs	Human	H1N1	pdm	2010	Thailand	A/Bangkok/INS514/2010	ggtgatgccccattccttgatcggctccgccgagatcaaaagtccttaaaaggaagaggcaacaccc	-	*
CY096525	Human H1N1pdm2009 IAVs	Human	H1N1	pdm	2010	Thailand	A/Bangkok/INS517/2010	ggtgatgccccattccttgatcggctccgccgagatcaaaagtccttaaaaggaagaggcaacaccc	-	*
CY098567	Human H1N1pdm2009 IAVs	Human	H1N1	pdm	2010	Thailand	A/Bangkok/INS520/2010	ggtgatgccccattccttgatcggctccgccgagatcaaaagtccttaaaaggaagaggcaacaccc	-	*
CY176470	Human H1N1pdm2009 IAVs	Human	H1N1	pdm	2010	Thailand	A/Khon_Kaen/INS3_640/2010	ggtgatgccccattccttgatcggctccgccgagatcaaaagtccttaaaaggaagaggcaacaccc	-	*
CY129810	Human H1N1pdm2009 IAVs	Human	H1N1	pdm	2010	Thailand	A/Khon_Kaen/INS586/2010	ggtgatgccccattccttgatcggctccgccgagatcaaaagtccttaaaaggaagaggcaacaccc	-	*
CY092852	Human H1N1pdm2009 IAVs	Human	H1N1		2010	Australia	A/Sydney/DD3_57/2010	ggtgatgccccattccttgatcggctccgccgagatcaaaagtccttaaaaggaagaggcaacaccc	-	*
CY167472	Human H1N1pdm2009 IAVs	Human	H1N1		2010	USA	A/Tennessee/F2005A/2010	ggtgatgccccattccttgatcggctccgccgagatcaaaagtccttaaaaggaagaggcaacaccc	-	*
CY088805	Human H1N1pdm2009 IAVs	Human	H1N1	pdm	2010	Thailand	A/Thailand/CU_H2176/2010	ggtgatgccccattccttgatcggctccgccgagatcaaaagtccttaaaaggaagaggcaacaccc	-	*
CY088819	Human H1N1pdm2009 IAVs	Human	H1N1	pdm	2010	Thailand	A/Thailand/CU_H2358/2010	ggtgatgccccattccttgatcggctccgccgagatcaaaagtccttaaaaggaagaggcaacaccc	-	*
CY081081	Human H1N1pdm2009 IAVs	Human	H1N1	pdm	2010	Canada	A/Ontario/720545/2010	ggtgatgccccattccttgatcggctccgccgagatcaaaagtccttaaaaggaagaggcaacaccc	-	*
JX309936	Human H1N1pdm2009 IAVs	Human	H1N1	pdm	2010	Singapore	A/Singapore/GP4523/2010	ggtgatgccccattccttgatcggctccgccgagatcaaaagtccttaaaaggaagaggcaacaccc	-	*
JX309906	Human H1N1pdm2009 IAVs	Human	H1N1	pdm	2010	Singapore	A/Singapore/TT474/2010	ggtgatgccccattccttgatcggctccgccgagatcaaaagtccttaaaaggaagaggcaacaccc	-	*
HQ891279	Human H1N1pdm2009 IAVs	Human	H1N1	pdm	2010	Russia	A/Vladivistok/7/2010	ggtgatgccccattccttgatcggctccgccgagatcaaaagtccttaaaaggaagaggcaacaccc	-	*
KC610060	Human H1N1pdm2009 IAVs	Swine	H1N1		2010	Thailand	A/swine/Thailand/CU_SPN47/2010	ggtgatgccccattccttgatcggctccgccgagatcaaaagtccttaaaaggaagaggcaacaccc	-	*
JX625794	Human H1N1pdm2009 IAVs	Human	H1N1	pdm	2010	United_Kingdom	A/England/05000088/2010	ggtgatgccccattccttgatcggctccgccgagatcaaaagtccttaaaaggaagaggcaacaccc	-	*
CY096310	Human H1N1pdm2009 IAVs	Human	H1N1	pdm	2010	Thailand	A/Bangkok/INS479/2010	ggtgatgccccattccttgatcggctccgccgagatcaaaagtccttaaaaggaagaggcaacaccc	-	*
CY092538	Human H1N1pdm2009 IAVs	Human	H1N1	pdm	2010	Australia	A/Sydney/DD3_15/2010	ggtgatgccccattccttgatcggctccgccgagatcaaaagtccttaaaaggaagaggcaacaccc	-	*
CY092714	Human H1N1pdm2009 IAVs	Human	H1N1	pdm	2010	Australia	A/Sydney/DD3_51/2010	ggtgatgccccattccttgatcggctccgccgagatcaaaagtccttaaaaggaagaggcaacaccc	-	*
CY081593	Human H1N1pdm2009 IAVs	Human	H1N1	pdm	2010	China	A/Beijing/3907/2010	ggtgatgccccattccttgatcggctccgccgagatcaaaagtccttaaaaggaagaggcaacaccc	-	*
CY098126	Human H1N1pdm2009 IAVs	Human	H1N1	pdm	2010	Thailand	A/Bangkok/INS487/2010	ggtgatgccccattccttgatcggctccgccgagatcaaaagtccttaaaaggaagaggcaacaccc	-	*
CY089433	Human H1N1pdm2009 IAVs	Human	H1N1	pdm	2010	Thailand	A/Thailand/CU_B4148/2010	ggtgatgccccattccttgatcggctccgccgagatcaaaagtccttaaaaggaagaggcaacaccc	-	*
CY089451	Human H1N1pdm2009 IAVs	Human	H1N1	pdm	2010	Thailand	A/Thailand/CU_H2548/2010	ggtgatgccccattccttgatcggctccgccgagatcaaaagtccttaaaaggaagaggcaacaccc	-	*
CY089459	Human H1N1pdm2009 IAVs	Human	H1N1	pdm	2010	Thailand	A/Thailand/CU_H2698/2010	ggtgatgccccattccttgatcggctccgccgagatcaaaagtccttaaaaggaagaggcaacaccc	-	*
HQ834750	Human H1N1pdm2009 IAVs	Human	H1N1	pdm	2010	Russia	A/Moscow/IIV_33/2010	ggtgatgccccattccttgatcggctccgccgagatcaaaagtccttaaaaggaagaggcaacaccc	-	*
JX309920	Human H1N1pdm2009 IAVs	Human	H1N1	pdm	2010	Singapore	A/Singapore/GP4444/2010	ggtgatgccccattccttgatcggctccgccgagatcaaaagtccttaaaaggaagaggcaacaccc	-	*

JX309956	Human H1N1pdm2009 IAVs	Human	H1N1	pdm	2010	Singapore	A/Singapore/GP4682/2010	ggtgatgccccattccttgatcggctccgccgagatcaaaagtccttaaaaggaagaggcaacaccc	-	*
JN187344	Human H1N1pdm2009 IAVs	Human	H1N1	pdm	2010	Taiwan	A/Taiwan/842/2010	ggtgatgccccattccttgatcggctccgccgagatcaaaagtccttaaaaggaagaggcaacaccc	-	*
JX309879	Human H1N1pdm2009 IAVs	Human	H1N1	pdm	2010	Singapore	A/Singapore/GP4307/2010	ggtgatgccccattccttgatcggctccgccgagatcaaaagtccttaaaaggaagaggcaacaccc	-	*
KC882420	Human H1N1pdm2009 IAVs	Human	H1N1	pdm	2010	USA	A/Colorado/11/2010	ggtgatgccccattccttgatcggctccgccgagatcaaaagtccttaaaaggaagaggcaacaccc	-	*
CY098166	Human H1N1pdm2009 IAVs	Human	H1N1	pdm	2010	Thailand	A/Bangkok/INS509/2010	ggtgatgccccattccttgatcggctccgccgagatcaaaagtccttaaaaggaagaggcaacaccc	-	*
CY089792	Human H1N1pdm2009 IAVs	Swine	H1N1		2010	Thailand	A/swine/Thailand/CU_DP83/2010	ggtgatgccccattccttgatcggctccgccgagatcaaaagtccttaaaaggaagaggcaacaccc	-	*
CY092397	Human H1N1pdm2009 IAVs	Human	H1N1	pdm	2010	Australia	A/Sydney/DD3_32/2010	ggtgatgccccattccttgatcggctccgccgagatcaaaagtccttaaaaggaagaggcaacaccc	-	*
JX625826	Human H1N1pdm2009 IAVs	Human	H1N1	pdm	2010	United_Kingdom	A/England/280/2010	ggtgatgccccattccttgatcggctccgccgagatcaaaagtccttaaaaggaagaggcaacaccc	-	*
CY159461	Human H1N1pdm2009 IAVs	Swine	H1N1		2010	USA	A/swine/Arkansas/SG1499/2010	ggtgatgccccattccttgatcggctccgccgagatcaaaagtccttaaaaggaagaggcaacaccc	-	*
CY062086	Human H1N1pdm2009 IAVs	Human	H1N1	pdm	2010	USA	A/New_York/1998/2010	ggtgatgccccattccttgatcggctccgccgagatcaaaagtccttaaaaggaagaggcaacaccc	-	*
JX309698	Human H1N1pdm2009 IAVs	Human	H1N1	pdm	2010	Singapore	A/Singapore/SS37/2010	ggtgatgccccattccttgatcggctccgccgagatcaaaagtccttaaaaggaagaggcaacaccc	-	*
CY067666	Human H1N1pdm2009 IAVs	Swine	H1N2		2010	Italy	A/swine/Italy/116114/2010	ggtgatgccccattccttgatcggctccgccgagatcaaaagtccttaaaaggaagaggcaacaccc	-	*
JN187341	Human H1N1pdm2009 IAVs	Human	H1N1	pdm	2010	Taiwan	A/Taiwan/1164/2010	ggtgatgccccattccttgatcggctccgccgagatcaaaagtccttaaaaggaagaggcaacaccc	-	*
JN409395	Human H1N1pdm2009 IAVs	Swine	H3N2		2010	USA	A/swine/Kansas/10_91088/2010	ggtgatgccccattccttgatcggctccgccgagatcaaaagtccttaaaaggaagaggcaacaccc	-	*
CY160272	Human H1N1pdm2009 IAVs	Swine	H3N2		2010	USA	A/swine/Oklahoma/SG1498/2010	ggtgatgccccattccttgatcggctccgccgagatcaaaagtccttaaaaggaagaggcaacaccc	-	*
CY089383	Human H1N1pdm2009 IAVs	Human	H1N1	pdm	2010	Nicaragua	A/Managua/3246.01/2010	ggtgatgccccattccttgatcggctccgccgagatcaaaagtccttaaaaggaagaggcaacaccc	-	*
CY089375	Human H1N1pdm2009 IAVs	Human	H1N1	pdm	2010	Nicaragua	A/Managua/5708.01/2010	ggtgatgccccattccttgatcggctccgccgagatcaaaagtccttaaaaggaagaggcaacaccc	-	*
CY100534	Human H1N1pdm2009 IAVs	Human	H1N1	pdm	2010	Mexico	A/Mexico_City/INER10/2010	ggtgatgccccattccttgatcggctccgccgagatcaaaagtccttaaaaggaagaggcaacaccc	-	*
KC261336	Human H1N1pdm2009 IAVs	Human	H1N1	pdm	2010	Mexico	A/Mexico_City/INERPHAC3/2010	ggtgatgccccattccttgatcggctccgccgagatcaaaagtccttaaaaggaagaggcaacaccc	-	*
CY100542	Human H1N1pdm2009 IAVs	Human	H1N1		2010	Mexico	A/Mexico_City/INER11/2010	ggtgatgccccattccttgatcggctccgccgagatcaaaagtccttaaaaggaagaggcaacaccc	-	*
KC261337	Human H1N1pdm2009 IAVs	Human	H1N1	pdm	2010	Mexico	A/Mexico_City/INERPHAC4/2010	ggtgatgccccattccttgatcggctccgccgagatcaaaagtccttaaaaggaagaggcaacaccc	-	*
CY120028	Human H1N1pdm2009 IAVs	Human	H1N1	pdm	2010	Mexico	A/Mexico/InDRECTRLA/2010	ggtgatgccccattccttgatcggctccgccgagatcaaaagtccttaaaaggaagaggcaacaccc	-	*
CY159085	Human H1N1pdm2009 IAVs	Swine	H1N1		2010	Mexico	A/swine/Mexico/3146/2010	ggtgatgccccattccttgatcggctccgccgagatcaaaagtccttaaaaggaagaggcaacaccc	-	*
CY066475	Human H1N1pdm2009 IAVs	Human	H1N1	pdm	2010	USA	A/California/VRDL4/2010	ggtgatgccccattccttgatcggctccgccgagatcaaaagtccttaaaaggaagaggcaacaccc	-	*
CY088812	Human H1N1pdm2009 IAVs	Human	H1N1		2010	Thailand	A/Thailand/CU_H2283/2010	ggtgatgccccattccttgatcggctccgccgagatcaaaagtccttaaaaggaagaggcaacaccc	-	*

JF764084	Human H1N1pdm2009 IAVs	Human	H1N1	pdm	2010	India	A/India/Blore/2010	ggtgatgccccattccttgatcggctccgccgagatca aaagtccttaaaaggaagaggcaacaccc	-	*
KJ690452	Human H1N1pdm2009 IAVs	Human	H1N1		2010	Uganda	A/Uganda/MUWRP_212/2010	ggtgatgccccattccttgatcggctccgccgagatca aaagtccttaaaaggaagaggcaacaccc	-	*
KJ690491	Human H1N1pdm2009 IAVs	Human	H1N1	pdm	2010	Uganda	A/Uganda/MUWRP_222/2010	ggtgatgccccattccttgatcggctccgccgagatca aaagtccttaaaaggaagaggcaacaccc	-	*
KJ690420	Human H1N1pdm2009 IAVs	Human	H1N1	pdm	2010	Uganda	A/Uganda/MUWRP_220/2010	ggtgatgccccattccttgatcggctccgccgagatca aaagtccttaaaaggaagaggcaacaccc	-	*
CY086892	Human H1N1pdm2009 IAVs	Swine	H1N2		2010	USA	A/swine/North_Carolina/226124/2010	ggtgatgccccattccttgatcggctccgccgagatca aaagtccttaaaaggaagaggcaacaccc	-	*
CY086900	Human H1N1pdm2009 IAVs	Swine	H1N2		2010	USA	A/swine/North_Carolina/226125/2010	ggtgatgccccattccttgatcggctccgccgagatca aaagtccttaaaaggaagaggcaacaccc	-	*
CY086908	Human H1N1pdm2009 IAVs	Swine	H1N2		2010	USA	A/swine/North_Carolina/226126/2010	ggtgatgccccattccttgatcggctccgccgagatca aaagtccttaaaaggaagaggcaacaccc	-	*
HQ695936	Human H1N1pdm2009 IAVs	Swine	H1N1		2010	China	A/swine/Jangsu/38/2010	ggtgatgccccattccttgatcggctccgccgagatca aaagtccttaaaaggaagaggcaacaccc	-	*
CY088649	Human H1N1pdm2009 IAVs	Human	H1N1	pdm	2010	India	A/Vadu/NIV1043725/2010	ggtgatgccccattccttgatcggctccgccgagatca aaagtccttaaaaggaagaggcaacaccc	-	*
HE589470	Human H1N1pdm2009 IAVs	Swine	H1N1		2010	Cuba	A/swine/Pinar_del_Rio/3/2010	ggtgatgccccattccttgatcggctccgccgagatca aaagtccttaaaaggaagaggcaacaccc	-	*
KC780450	Human H1N1pdm2009 IAVs	Human	H1N1	pdm	2010	USA	A/New_York/03/2010	ggtgatgccccattccttgatcggctccgccgagatca aaagtccttaaaaggaagaggcaacaccc	-	*
HQ606471	Human H1N1pdm2009 IAVs	Human	H1N1	pdm	2010	Iran	A/Shiraz/14/2010	ggtgatgccccattccttgatcggctccgccgagatca aaagtccttaaaaggaagaggcaacaccc	-	*
KC859186	Human H1N1pdm2009 IAVs	Swine	H1N1		2010	Thailand	A/swine/Thailand/CS211/2010	ggtgatgccccattccttgatcggctccgccgagatca aaagtccttaaaaggaagaggcaacaccc	-	*
HE584752	Human H1N1pdm2009 IAVs	Swine	H1N1		2010	Cuba	A/swine/Holguin/121/2010	ggtgatgccccattccttgatcggctccgccgagatca aaagtccttaaaaggaagaggcaacaccc	-	*
HE584760	Human H1N1pdm2009 IAVs	Swine	H1N1		2010	Cuba	A/swine/La_Habana/130/2010	ggtgatgccccattccttgatcggctccgccgagatca aaagtccttaaaaggaagaggcaacaccc	-	*
HE582007	Human H1N1pdm2009 IAVs	Swine	H1N1		2010	Cuba	A/swine/Villa_Clara/84/2010	ggtgatgccccattccttgatcggctccgccgagatca aaagtccttaaaaggaagaggcaacaccc	-	*
CY073002	Human H1N1pdm2009 IAVs	Human	H1N1		2010	Nicaragua	A/Managua/07193_09/2010	ggtgatgccccattccttgatcggctccgccgagatca aaagtccttaaaaggaagaggcaacactc	-	*
JX293384	Human H1N1pdm2009 IAVs	Swine	H1N1		2010	Thailand	A/swine/Thailand/CU_PL63/2010	ggtgatgccccattccttgatcggctccgccgagatca aaagtccttaaaaggaagaggcaacactc	-	*
JX293379	Human H1N1pdm2009 IAVs	Swine	H1N1		2010	Thailand	A/swine/Thailand/CU_PL65/2010	ggtgatgccccattccttgatcggctccgccgagatca aaagtccttaaaaggaagaggcaacactc	-	*
CY071315	Human H1N1pdm2009 IAVs	Human	H1N1	pdm	2010	Greece	A/Athens/INS420/2010	ggtgatgccccattccttgatcggctccgccgagatca aaagtccttaaaaggaaggggcaacaccc	-	*
JX309828	Human H1N1pdm2009 IAVs	Human	H1N1	pdm	2010	Singapore	A/Singapore/TT459/2010	ggtgatgccccattccttgatcggctccgccgagatca aaagtccttaaaaggaaggggcaacaccc	-	*
CY097841	Human H1N1pdm2009 IAVs	Human	H1N1	pdm	2010	USA	A/District_of_Columbia/WRAIR0309/2010	ggtgatgccccattccttgatcggctccgccgagatca aaagtccttaaaaggaaggggcaacaccc	-	*
JF275921	Human H1N1pdm2009 IAVs	Swine	H1N1		2010	China	A/swine/Nanchang/3/2010	ggtgatgccccattccttgatcggctccgccgagatca aaagtccttaaagggaagaggcaacaccc	-	*
KC859138	Human H1N1pdm2009 IAVs	Swine	H1N1		2010	Thailand	A/swine/Thailand/LB158/2010	ggtgatgccccattccttgatcggctccgccgagatca aaagtccttaaagggaagaggcaacaccc	-	*
KC859170	Human H1N1pdm2009 IAVs	Swine	H1N1		2010	Thailand	A/swine/Thailand/LB171/2010	ggtgatgccccattccttgatcggctccgccgagatca aaagtccttaaagggaagaggcaacaccc	-	*

KC859154	Human H1N1pdm2009 IAVs	Swine	H1N1		2010	Thailand	A/swine/Thailand/LB159/2010	ggtgatgccccattccttgatcggctccgccgagatca aaagtccttaaagggaagaggcaacaccc	-	*
KC859114	Human H1N1pdm2009 IAVs	Swine	H1N1		2010	Thailand	A/swine/Thailand/LB164/2010	ggtgatgccccattccttgatcggctccgccgagatca aaagtccttaaagggaagaggcaacaccc	-	*
KC859162	Human H1N1pdm2009 IAVs	Swine	H1N1		2010	Thailand	A/swine/Thailand/LB160/2010	ggtgatgccccattccttgatcggctccgccgagatca aaagtccttaaagggaagaggcaacaccc	-	*
CY071059	Human H1N1pdm2009 IAVs	Human	H1N1	pdm	2010	Austria	A/Vienna/INS368/2010	ggtgatgccccattccttgatcggctccgccgagatca gaagtccttaaaaggaagaggcaacaccc	-	*
JX625746	Human H1N1pdm2009 IAVs	Human	H1N1	pdm	2010	United_Kingdom	A/England/182/2010	ggtgatgccccattccttgatcggcttcgccgagatcaa aagtccttaaaaggaagaggcaacaccc	-	*
KF897797	Human H1N1pdm2009 IAVs	Human	H1N1	pdm	2010	France	A/StEtienne/1139/2010	ggtgatgccccattccttgatcggcttcgccgagatcaa aagtccttaaaaggaagaggcaacaccc	-	*
HQ695985	Human H1N1pdm2009 IAVs	Swine	H1N1		2010	China	A/swine/Jangsu/434/2010	ggtgatgccccattccttgatcggcttcgccgagatcaa aagtccttaaaaggaagaggcaacaccc	-	*
HQ695993	Human H1N1pdm2009 IAVs	Swine	H1N1		2010	China	A/swine/Jangsu/504/2010	ggtgatgccccattccttgatcggcttcgccgagatcaa aagtccttaaaaggaagaggcaacaccc	-	*
CY096374	Human H1N1pdm2009 IAVs	Human	H1N1	pdm	2010	Thailand	A/Bangkok/INS492/2010	ggtgatgccccattccttgatcggcttcgccgagatcaa aagtccttaaaaggaagaggcaacaccc	-	*
CY129762	Human H1N1pdm2009 IAVs	Human	H1N1		2010	Thailand	A/Bangkok/INS580/2010	ggtgatgccccattccttgatcggcttcgccgagatcaa aagtccttaaaaggaagaggcaacaccc	-	*
CY129778	Human H1N1pdm2009 IAVs	Human	H1N1	pdm	2010	Thailand	A/Bangkok/INS582/2010	ggtgatgccccattccttgatcggcttcgccgagatcaa aagtccttaaaaggaagaggcaacaccc	-	*
CY129818	Human H1N1pdm2009 IAVs	Human	H1N1	pdm	2010	Thailand	A/Bangkok/INS587/2010	ggtgatgccccattccttgatcggcttcgccgagatcaa aagtccttaaaaggaagaggcaacaccc	-	*
CY096549	Human H1N1pdm2009 IAVs	Human	H1N1	pdm	2010	Thailand	A/Khon_Kaen/INS521/2010	ggtgatgccccattccttgatcggcttcgccgagatcaa aagtccttaaaaggaagaggcaacaccc	-	*
CY096557	Human H1N1pdm2009 IAVs	Human	H1N1	pdm	2010	Thailand	A/Khon_Kaen/INS522/2010	ggtgatgccccattccttgatcggcttcgccgagatcaa aagtccttaaaaggaagaggcaacaccc	-	*
CY096565	Human H1N1pdm2009 IAVs	Human	H1N1	pdm	2010	Thailand	A/Khon_Kaen/INS523/2010	ggtgatgccccattccttgatcggcttcgccgagatcaa aagtccttaaaaggaagaggcaacaccc	-	*
CY129826	Human H1N1pdm2009 IAVs	Human	H1N1	pdm	2010	Thailand	A/Khon_Kaen/INS588/2010	ggtgatgccccattccttgatcggcttcgccgagatcaa aagtccttaaaaggaagaggcaacaccc	-	*
CY081159	Human H1N1pdm2009 IAVs	Human	H1N1	pdm	2010	Thailand	A/Thailand/CU_C1157/2010	ggtgatgccccattccttgatcggcttcgccgagatcaa aagtccttaaaaggaagaggcaacaccc	-	*
HQ695969	Human H1N1pdm2009 IAVs	Swine	H1N1		2010	China	A/swine/Jangsu/285/2010	ggtgatgccccattccttgatcggcttcgccgagatcaa aagtccttaaaaggaagaggcaacaccc	-	*
JN584182	Human H1N1pdm2009 IAVs	Swine	H3N2		2010	Canada	A/swine/Quebec/1262080/2010	ggtgatgccccatttctagatcggctccgccgagatca aaagtccttaaaaggaagaggcaacaccc	-	-
JF682724	Human H1N1pdm2009 IAVs	Swine	H3N2		2010	Canada	A/swine/Quebec/1265553/2010	ggtgatgccccatttctagatcggctccgccgagatca aaagtccttaaaaggaagaggcaacaccc	-	-
CY167336	Human H1N1pdm2009 IAVs	Human	H1N1	pdm	2010	USA	A/Tennessee/F1052A/2010	ggtgatgccccccttccttgatcggctccgccgagatca aaagtccttaaaaggaagaggcaacaccc	-	-
KJ023128	Human H1N1pdm2009 IAVs	Human	H1N1	pdm	2010	India	A/Delhi/038/2010	ggtgatgccccgttccttgatcggctccgccgagatca aaagtccttaaaaggaagaggcaacaccc	-	*
CY167352	Human H1N1pdm2009 IAVs	Human	H1N1		2010	USA	A/Tennessee/F1060/2010	ggtgatgctccattccttgatcggctccgccgagatcaa aagtccttaaaaggaagaggcaacaccc	-	-
KF411276	Human H1N1pdm2009 IAVs	Human	H1N1	pdm	2010	China	A/Qingdao/F970/2010	ggtgatgctccattccttgatcggctccgccgagatcaa aagtccttaaaaggaagaggcaacaccc	-	-
JN187342	Human H1N1pdm2009 IAVs	Human	H1N1		2010	Taiwan	A/Taiwan/3994/2010	ggtgattccccattccttgatcggctccgccgagatcaa aagtccttaaaaggaagaggcaacaccc	-	*

CY129577	Human H1N1pdm2009 IAVs	Human	H1N1	pdm	2011	Hong_Kong	A/Kowloon/INS628/2011	agtgatgccccattccttgatcggctccgccgagatca aaagtccttaaaaggaagaggcaacaccc	-	*
CY176401	Human H1N1pdm2009 IAVs	Human	H1N1	pdm	2011	Poland	A/Warsaw/INS3_658/2011	agtgatgccccattccttgatcggctccgccgagatca aaagtccttaaaaggaagaggcaacaccc	-	*
KC881957	Human H1N1pdm2009 IAVs	Human	H1N1	pdm	2011	USA	A/New_Mexico/04/2011	ggcgatgccccattccttgatcggctccgccgagatca aaagtcattaaaaggaaggggcaacaccc	-	-
KC881941	Human H1N1pdm2009 IAVs	Human	H1N1	pdm	2011	USA	A/New_Mexico/05/2011	ggcgatgccccattccttgatcggctccgccgagatca aaagtcattaaaaggaaggggcaacaccc	-	-
KC881933	Human H1N1pdm2009 IAVs	Human	H1N1	pdm	2011	USA	A/New_Mexico/06/2011	ggcgatgccccattccttgatcggctccgccgagatca aaagtcattaaaaggaaggggcaacaccc	-	-
KF411282	Human H1N1pdm2009 IAVs	Human	H1N1		2011	China	A/Qingdao/F166/2011	ggcgatgccccattccttgatcggctccgccgagatca aaagtccttaaaaggaaggggcaacaccc	-	*
CY124821	Human H1N1pdm2009 IAVs	Human	H1N1	pdm	2011	Singapore	A/Singapore/TT58/2011	ggcgatgccccattccttgatcggctccgccgagatca aaagtccttaaaaggaaggggcaacaccc	-	*
KM028523	Human H1N1pdm2009 IAVs	Swine	H1N1		2011	China	A/swine/Hong_Kong/829/2011	ggcgatgccccattccttgatcggctccgccgagatca aaagtccttaaaaggaaggggcaacaccc	-	*
KM028531	Human H1N1pdm2009 IAVs	Swine	H1N1		2011	China	A/swine/Hong_Kong/830/2011	ggcgatgccccattccttgatcggctccgccgagatca aaagtccttaaaaggaaggggcaacaccc	-	*
KF612179	Human H1N1pdm2009 IAVs	Human	H1N1	pdm	2011	Bolivia	A/Santa_Cruz/5873/2011	ggtgacgccccattccttgatcggctccgccgagacca aaagtccttaaaaggaagaggcaacaccc	-	-
CY111290	Human H1N1pdm2009 IAVs	Human	H1N1	pdm	2011	USA	A/Boston/DOA40/2011	ggtgatgcaccattccttgatcggctccgccgagatca aaagtccttaaaaggaagaggcaacaccc	-	*
KC222608	Human H1N1pdm2009 IAVs	Swine	H1N2		2011	Germany	A/swine/Altenberge/IDT14252/2011	ggtgatgccccattcattgatcggctccgccgagatca aaagtcattaaaaggaagaggcaacaccc	-	-
JF956830	Human H1N1pdm2009 IAVs	Swine	H1N1		2011	USA	A/swine/OH/9838/2011	ggtgatgccccattcctcgatcggctccgccgagatca aaagtccttaaaaggaagaggcaacaccc	-	-
CY160644	Human H1N1pdm2009 IAVs	Human	H1N1	pdm	2011	Peru	A/Peru/PER045/2011	ggtgatgccccattccttgaccggctccgcagagatca aaagtccttaaaaggaagaggcaacaccc	+	+
JQ693726	Human H1N1pdm2009 IAVs	Human	H1N1	pdm	2011	Taiwan	A/Taiwan/549/2011	ggtgatgccccattccttgaccggctccgccgagatca aaagtccttaaaaggaagaggcaacaccc	-	*
KM028227	Human H1N1pdm2009 IAVs	Swine	H1N2		2011	China	A/swine/Guangxi/3202/2011	ggtgatgccccattccttgataggctccgccgagatca aaagtctttaaaaggaagaggcaacactc	-	-
KM028235	Human H1N1pdm2009 IAVs	Swine	H1N2		2011	China	A/swine/Guangxi/3205/2011	ggtgatgccccattccttgataggctccgccgagatca aaagtctttaaaaggaagaggcaacactc	-	-
KM028243	Human H1N1pdm2009 IAVs	Swine	H1N2		2011	China	A/swine/Guangxi/3212/2011	ggtgatgccccattccttgataggctccgccgagatca aaagtctttaaaaggaagaggcaacactc	-	-
KM028259	Human H1N1pdm2009 IAVs	Swine	H1N2		2011	China	A/swine/Guangxi/3217/2011	ggtgatgccccattccttgataggctccgccgagatca aaagtctttaaaaggaagaggcaacactc	-	-
KM028267	Human H1N1pdm2009 IAVs	Swine	H1N2		2011	China	A/swine/Guangxi/3234/2011	ggtgatgccccattccttgataggctccgccgagatca aaagtctttaaaaggaagaggcaacactc	-	-
KM028283	Human H1N1pdm2009 IAVs	Swine	H1N2		2011	China	A/swine/Guangxi/NS3256/2011	ggtgatgccccattccttgataggctccgccgagatca aaagtctttaaaaggaagaggcaacactc	-	-
KM028291	Human H1N1pdm2009 IAVs	Swine	H1N2		2011	China	A/swine/Guangxi/NS3257/2011	ggtgatgccccattccttgataggctccgccgagatca aaagtctttaaaaggaagaggcaacactc	-	-
KM028299	Human H1N1pdm2009 IAVs	Swine	H1N2		2011	China	A/swine/Guangxi/NS3267/2011	ggtgatgccccattccttgataggctccgccgagatca aaagtctttaaaaggaagaggcaacactc	-	-
KM028315	Human H1N1pdm2009 IAVs	Swine	H1N2		2011	China	A/swine/Guangxi/NS3272/2011	ggtgatgccccattccttgataggctccgccgagatca aaagtctttaaaaggaagaggcaacactc	-	-
KM028251	Human H1N1pdm2009 IAVs	Swine	H1N2		2011	China	A/swine/Guangxi/3215/2011	ggtgatgccccattccttgataggctccgccgagatca aaagtctttaaaaggaagaggcaacactc	-	-

KJ023137	Human H1N1pdm2009 IAVs	Human	H1N1	pdm	2011	India	A/Goa/047/2011	ggtgatgccccattccttgatcggctacgccgagatca aaagtccttaaaaggaagaggcaacaccc	-	*
KJ023140	Human H1N1pdm2009 IAVs	Human	H1N1	pdm	2011	India	A/Delhi/050/2011	ggtgatgccccattccttgatcggctacgccgagatca aaagtccttaaaaggaagaggcaacaccc	-	*
KJ023145	Human H1N1pdm2009 IAVs	Human	H1N1	pdm	2011	India	A/Delhi/055/2011	ggtgatgccccattccttgatcggctacgccgagatca aaagtccttaaaaggaagaggcaacaccc	-	*
KJ023146	Human H1N1pdm2009 IAVs	Human	H1N1	pdm	2011	India	A/Delhi/056/2011	ggtgatgccccattccttgatcggctacgccgagatca aaagtccttaaaaggaagaggcaacaccc	-	*
CY129914	Human H1N1pdm2009 IAVs	Human	H1N1	pdm	2011	Denmark	A/Aarhus/INS609/2011	ggtgatgccccattccttgatcggctccgcagagatca aaagtccttaaaaggaagaggcaacaccc	+	+
CY091608	Human H1N1pdm2009 IAVs	Human	H1N1	pdm	2011	Russia	A/Voronezh/RII1/2011	ggtgatgccccattccttgatcggctccgcagagatca aaagtccttaaaaggaagaggcaacaccc	+	+
CY176566	Human H1N1pdm2009 IAVs	Human	H1N1	pdm	2011	USA	A/Springfield/INS3_652/2011	ggtgatgccccattccttgatcggctccgcagagatca aaagtccttaaaaggaagaggcaacaccc	+	+
CY129674	Human H1N1pdm2009 IAVs	Human	H1N1	pdm	2011	Greece	A/Athens/INS552/2011	ggtgatgccccattccttgatcggctccgcagagatca aaagtccttaaaaggaagaggcaacaccc	+	+
CY117575	Human H1N1pdm2009 IAVs	Human	Mixed	pdm	2011	USA	A/Boston/DOA37/2011_mixed_	ggtgatgccccattccttgatcggctccgcagagatca aaagtccttaaaaggaagaggcaacaccc	+	+
CY134589	Human H1N1pdm2009 IAVs	Human	H1N1	pdm	2011	USA	A/Boston/DOA53/2011	ggtgatgccccattccttgatcggctccgcagagatca aaagtccttaaaaggaagaggcaacaccc	+	+
CY111557	Human H1N1pdm2009 IAVs	Human	H1N1	pdm	2011	USA	A/Boston/DOA58/2011	ggtgatgccccattccttgatcggctccgcagagatca aaagtccttaaaaggaagaggcaacaccc	+	+
CY120751	Human H1N1pdm2009 IAVs	Human	H1N1	pdm	2011	Brazil	A/Brazil/AVS08/2011	ggtgatgccccattccttgatcggctccgcagagatca aaagtccttaaaaggaagaggcaacaccc	+	+
CY120759	Human H1N1pdm2009 IAVs	Human	H1N1	pdm	2011	Brazil	A/Brazil/AVS11/2011	ggtgatgccccattccttgatcggctccgcagagatca aaagtccttaaaaggaagaggcaacaccc	+	+
CY134557	Human H1N1pdm2009 IAVs	Human	H1N1	pdm	2011	USA	A/Boston/DOA46/2011	ggtgatgccccattccttgatcggctccgcagagatca aaagtccttaaaaggaagaggcaacaccc	+	+
CY176758	Human H1N1pdm2009 IAVs	Human	H1N1	pdm	2011	Germany	A/Cologne/INS3_686/2011	ggtgatgccccattccttgatcggctccgcagagatca aaagtccttaaaaggaagaggcaacaccc	+	+
CY129618	Human H1N1pdm2009 IAVs	Human	H1N1	pdm	2011	Germany	A/Cologne/INS538/2011	ggtgatgccccattccttgatcggctccgcagagatca aaagtccttaaaaggaagaggcaacaccc	+	+
CY129906	Human H1N1pdm2009 IAVs	Human	H1N1	pdm	2011	Germany	A/Cologne/INS607/2011	ggtgatgccccattccttgatcggctccgcagagatca aaagtccttaaaaggaagaggcaacaccc	+	+
KC882270	Human H1N1pdm2009 IAVs	Human	H1N1	pdm	2011	USA	A/California/20/2011	ggtgatgccccattccttgatcggctccgcagagatca aaagtccttaaaaggaagaggcaacaccc	+	+
JN187351	Human H1N1pdm2009 IAVs	Human	H1N1	pdm	2011	Taiwan	A/Taiwan/1102/2011	ggtgatgccccattccttgatcggctccgcagagatca aaagtccttaaaaggaagaggcaacaccc	+	+
JQ693729	Human H1N1pdm2009 IAVs	Human	H1N1	pdm	2011	Taiwan	A/Taiwan/1240/2011	ggtgatgccccattccttgatcggctccgcagagatca aaagtccttaaaaggaagaggcaacaccc	+	+
JN187347	Human H1N1pdm2009 IAVs	Human	H1N1	pdm	2011	Taiwan	A/Taiwan/2649/2011	ggtgatgccccattccttgatcggctccgcagagatca aaagtccttaaaaggaagaggcaacaccc	+	+
CY124572	Human H1N1pdm2009 IAVs	Human	H1N1		2011	Singapore	A/Singapore/GP496/2011	ggtgatgccccattccttgatcggctccgcagagatca aaagtccttaaaaggaagaggcaacaccc	+	+
CY124827	Human H1N1pdm2009 IAVs	Human	H1N1	pdm	2011	Singapore	A/Singapore/TT67/2011	ggtgatgccccattccttgatcggctccgcagagatca aaagtccttaaaaggaagaggcaacaccc	+	+
CY129479	Human H1N1pdm2009 IAVs	Human	H1N1	pdm	2011	Greece	A/Athens/INS568/2011	ggtgatgccccattccttgatcggctccgcagagatca aaagtccttaaaaggaagaggcaacaccc	+	+
CY129730	Human H1N1pdm2009 IAVs	Human	H1N1	pdm	2011	Greece	A/Athens/INS571/2011	ggtgatgccccattccttgatcggctccgcagagatca aaagtccttaaaaggaagaggcaacaccc	+	+

CY092884	Human H1N1pdm2009 IAVs	Human	H1N1	pdm	2011	USA	A/California/NHRC0001/2011	ggtgatgccccattccttgatcggctccgcagagatcaaaagtccttaaaaggaagaggcaacaccc	+	+
CY129532	Human H1N1pdm2009 IAVs	Human	H1		2011	United_Kingdom	A/Headington/INS596/2011	ggtgatgccccattccttgatcggctccgcagagatcaaaagtccttaaaaggaagaggcaacaccc	+	+
CY124522	Human H1N1pdm2009 IAVs	Human	H1N1		2011	Singapore	A/Singapore/EN34/2011	ggtgatgccccattccttgatcggctccgcagagatcaaaagtccttaaaaggaagaggcaacaccc	+	+
CY124578	Human H1N1pdm2009 IAVs	Human	H1N1	pdm	2011	Singapore	A/Singapore/GP582/2011	ggtgatgccccattccttgatcggctccgcagagatcaaaagtccttaaaaggaagaggcaacaccc	+	+
CY124618	Human H1N1pdm2009 IAVs	Human	H1N1	pdm	2011	Singapore	A/Singapore/GP767/2011	ggtgatgccccattccttgatcggctccgcagagatcaaaagtccttaaaaggaagaggcaacaccc	+	+
CY124670	Human H1N1pdm2009 IAVs	Human	H1N1	pdm	2011	Singapore	A/Singapore/GP887/2011	ggtgatgccccattccttgatcggctccgcagagatcaaaagtccttaaaaggaagaggcaacaccc	+	+
CY124556	Human H1N1pdm2009 IAVs	Human	H1N1	pdm	2011	Singapore	A/Singapore/GP46/2011	ggtgatgccccattccttgatcggctccgcagagatcaaaagtccttaaaaggaagaggcaacaccc	+	+
CY124654	Human H1N1pdm2009 IAVs	Human	H1N1	pdm	2011	Singapore	A/Singapore/GP868/2011	ggtgatgccccattccttgatcggctccgcagagatcaaaagtccttaaaaggaagaggcaacaccc	+	+
CY097912	Human H1N1pdm2009 IAVs	Human	H1N1		2011	Russia	A/Moscow/WRAIR1933P/2011	ggtgatgccccattccttgatcggctccgcagagatcaaaagtccttaaaaggaagaggcaacaccc	+	+
CY124610	Human H1N1pdm2009 IAVs	Human	H1N1	pdm	2011	Singapore	A/Singapore/GP730/2011	ggtgatgccccattccttgatcggctccgcagagatcaaaagtccttaaaaggaagaggcaacaccc	+	+
CY124690	Human H1N1pdm2009 IAVs	Human	H1N1	pdm	2011	Singapore	A/Singapore/GP917/2011	ggtgatgccccattccttgatcggctccgcagagatcaaaagtccttaaaaggaagaggcaacaccc	+	+
[truncated: 664,730 more chars]
