# Supplementary material for: The influenza A virus NS genome segment displays lineage-specific patterns in predicted RNA secondary structure
Source: BMC Res Notes. 2016 May 20;9:279. doi: 10.1186/s13104-016-2083-6 (PMC4875733; doi:10.1186/s13104-016-2083-6)
Supplement: Supplementary file 3 — 10.1186/s13104-016-2083-6 Scheme of human and swine NS gene phylogenetic tree. [file 13104_2016_2083_MOESM3_ESM.pdf]

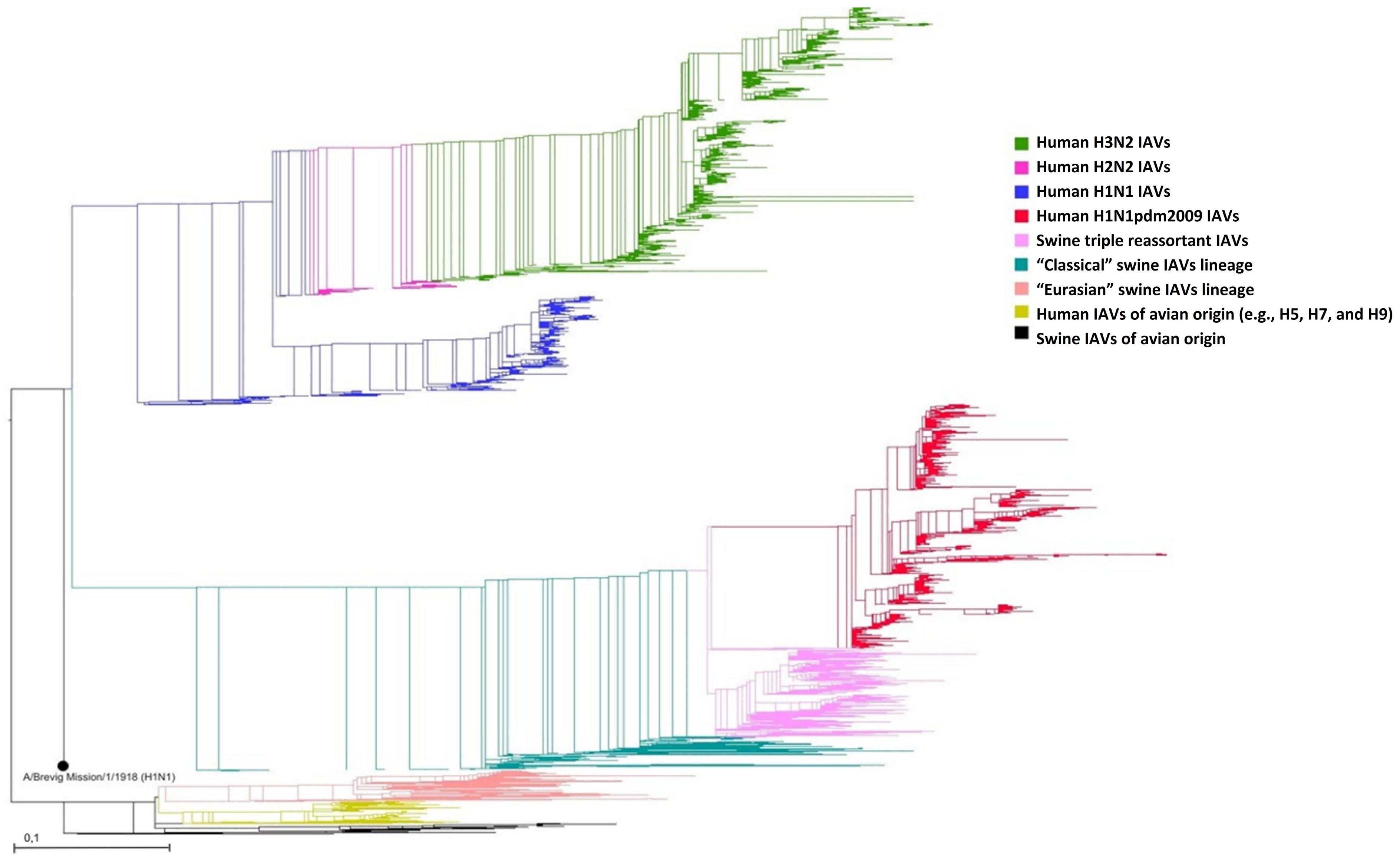

The RAxML phylogenetic tree of human and swine influenza A NS genes. The tree is midpoint rooted and the bootstrap support value is 500. The scale bars indicate the numbers of nucleotide substitutions per site.
